# Multiomics Reveals IL-17 Drives Epithelial Keratinization and Proliferation via EHF in Odontogenic Keratocysts

**DOI:** 10.3390/ijms27094115

**Published:** 2026-05-04

**Authors:** Jing-Rui Yi, Nian-Nian Zhong, Xuan-Hao Liu, Zheng-Rui Zhu, Yi-Jia-Ning Zhang, Jing Wang, Qi-Wen Man, Bing Liu

**Affiliations:** State Key Laboratory of Oral & Maxillofacial Reconstruction and Regeneration, Key Laboratory of Oral Biomedicine Ministry of Education, Hubei Key Laboratory of Stomatology, School & Hospital of Stomatology, Wuhan University, Wuhan 430070, China

**Keywords:** odontogenic keratocyst, epithelium, single-cell RNA sequencing, keratin 13, transcription factor

## Abstract

This study aims to investigate epithelial cell (EpC) heterogeneity in odontogenic keratocysts (OKCs) and the molecular mechanisms driving their characteristic keratinization and rapid proliferation. We integrated single-cell RNA sequencing (scRNA-seq) and spatial transcriptomics to profile OKC EpCs, validating findings via immunostaining and comparative analysis with normal oral mucosa. The regulatory roles of interleukin-17 (IL-17) and the transcription factor EHF were functionally assessed in vitro using human oral keratinocytes. Multi-omics mapping identified a distinct epithelial subpopulation (EpC2) characterized by robust keratinization, elevated keratin 13 (KRT13), and upregulated IL-17 signaling. Clinical OKC tissues exhibited significant upregulation and spatial co-localization of IL-17 and KRT13. In vitro, IL-17 robustly promoted cellular proliferation and KRT13 expression. Regulon analysis pinpointed EHF as the core transcription factor driving EpC2. EHF knockdown suppressed proliferation and downregulated KRT13, while overexpression amplified these processes. Crucially, IL-17 stimulation failed to rescue KRT13 expression in EHF-depleted cells, suggesting EHF as a critical downstream mediator. We present a comprehensive single-cell and spatial transcriptomic atlas of the OKC epithelium. The IL-17/EHF signaling axis appears to be a fundamental driver of OKC pathogenesis, promoting pathological hyperkeratinization and cellular proliferation. Targeting this axis presents a promising therapeutic strategy to manage OKC growth and prevent recurrence.

## 1. Introduction

Odontogenic keratocysts (OKCs) are developmental odontogenic cysts (classified as such by the World Health Organization), yet they exhibit benign, tumor-like aggressive behavior, including local invasion and a high recurrence rate [1]. Predominantly found in the posterior mandible [2,3], OKCs frequently exhibit aggressive growth, sometimes necessitating extensive interventions like osteotomy [4,5]. They present up to 21.1% recurrence rate [6], a 2.76% malignant transformation risk, and a 0.55% concurrent squamous cell carcinoma incidence [7].

Histologically, OKCs feature a fibrous wall lined by a 5- to 8-cell-layer stratified squamous epithelium. A diagnostic hallmark is parakeratinization, frequently forming a corrugated surface [8,9]. Previous studies have linked this keratinization to elevated keratin 13 (KRT13) expression [10,11]. Given that KRT13 has been shown to promote cellular stemness and aggressive behavior in other neoplasms [12], we hypothesize that this abnormal keratinization is a key driver of the cyst’s rapid proliferation and recurrence. Moreover, the absence of rete ridges renders the epithelial lining fragile and prone to detachment from the underlying connective tissue [7].

Current management of OKCs primarily involves surgical enucleation with adjunctive therapies, such as chemical cauterization [13]. However, adverse effects of traditional agents like Carnoy’s solution have spurred the search for safer pharmacological alternatives, including 5-Fluorouracil [14], and targeted therapies addressing underlying genetic drivers like *PTCH1* mutations [15,16,17].

Despite clinical advances, OKC’s cellular drivers remain unclear. Single-cell RNA sequencing (scRNA-seq) and spatial transcriptomics have emerged as powerful tools for resolving cellular heterogeneity and spatial architecture [18,19,20]. While our previous scRNA-seq study elucidated OKC fibroblast heterogeneity [21], the critical epithelial cells (EpCs) remain underexplored at the single-cell level.

To bridge this gap, this study employs a multi-omics approach to comprehensively investigate EpC heterogeneity in OKCs. Integrating omics profiling with immunohistochemistry (IHC), immunofluorescence (IF), and in vitro functional assays, we aim to uncover the specific gene expression variations and cellular interactions governing epithelial keratinization and proliferation, ultimately clarifying EpC’s role in OKC progression.

## 2. Results

### 2.1. Single-Cell and Spatial Omics Mapping of OKC Epithelial Heterogeneity

**Figure 1:**
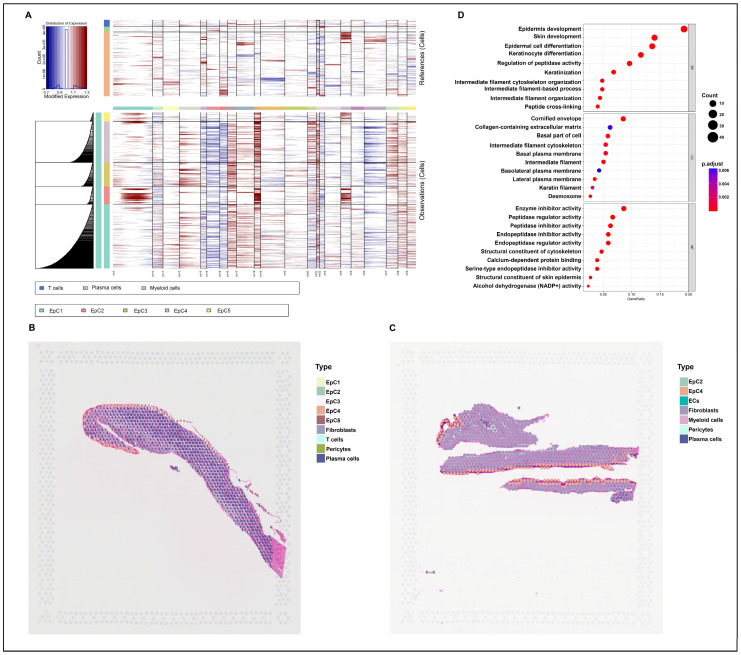
EpC2 exhibits extensive CNVs and pronounced keratinization signatures in OKC. (**A**) InferCNV analysis demonstrating widespread genomic alterations in the EpC2 subpopulation relative to the stable profiles of other epithelial clusters. (**B**,**C**) Spatial transcriptomic mapping resolving the precise anatomical localization of EpC1–EpC5 within the OKC epithelium, clearly delineating tissue morphology and epithelial boundaries. (**D**) GO enrichment of global EpC population, highlighting highly active keratinization and cornification processes. CNV, chromosome copy number variation; EC, endothelial cell; EpC, epithelial cell; GO, Gene Ontology; OKC, odontogenic keratocyst.

In our previous study, Uniform Manifold Approximation and Projection (UMAP) analysis revealed five distinct EpC subgroups (EpC1–EpC5) [21]. Building upon this, inferCNV analysis identified extensive chromosomal copy number variations (CNVs) specifically localized within the EpC2 subgroup (Figure 1A). Spatial transcriptomics further contextualized these subpopulations, demonstrating that EpC1-EpC5 are predominantly and widely distributed across the epithelial layers of the OKC (Figure 1B,C; Supplementary Figure S1). Gene Ontology (GO) enrichment analysis of the global EpC population highlighted active biological processes related to epithelial development, keratinization, and cornification (Figure 1D; Supplementary Table S2).

### 2.2. EpC2 Features Pronounced Keratinization and IL-17 Pathway Activation

**Figure 2:**
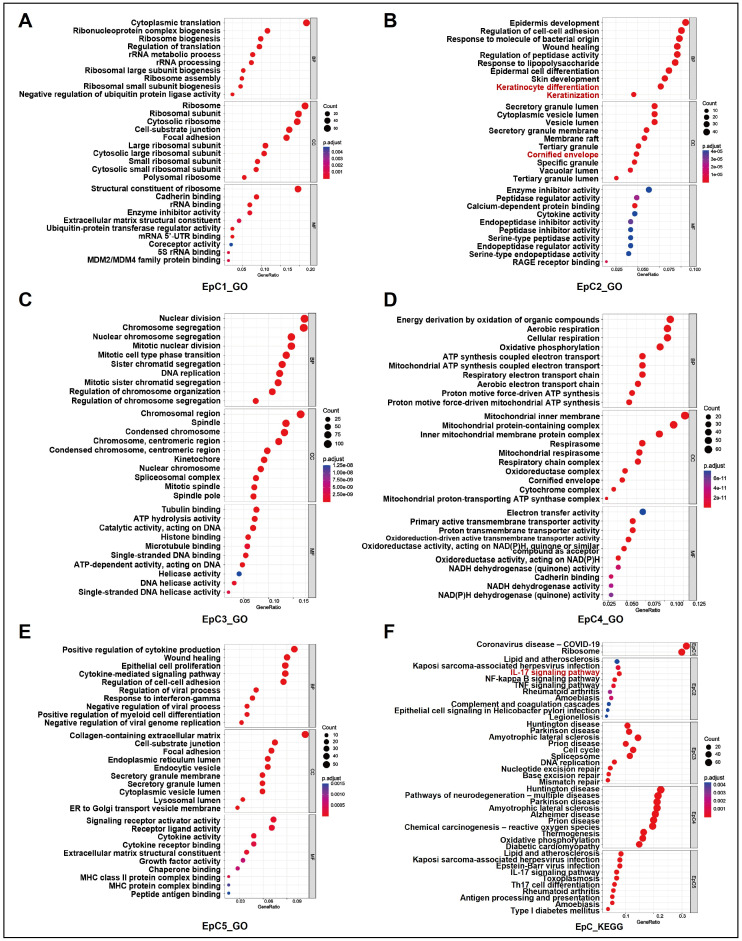
Single-cell transcriptomic profiling reveals distinct functional specializations across epithelial subpopulations. (**A**–**E**) GO enrichment analyses elucidating the distinct biological functions of clusters EpC1–EpC5. (**F**) KEGG pathway enrichment analysis demonstrating significant activation of the IL-17 signaling pathway in EpC2. GO, Gene Ontology; KEGG, Kyoto Encyclopedia of Genes and Genomes; EpC, epithelial cell.

GO enrichment analysis of differentially expressed genes (DEGs) revealed distinct functional roles for EpC1-EpC5. EpC1 was associated with active metabolic processes and microenvironmental interactions (Figure 2A; Supplementary Table S3). EpC2 was significantly enriched in pathways driving keratinocyte differentiation, epidermis development, and cornified envelope formation, while molecularly exhibiting robust peptidase regulation and enzyme inhibitor activity (Figure 2B; Supplementary Table S4). EpC3 demonstrated profound enrichment in cell division-related terms (Figure 2C; Supplementary Table S5), EpC4 in active energy metabolism (Figure 2D; Supplementary Table S6), and EpC5 in cytokine production and wound healing (Figure 2E; Supplementary Table S7). Kyoto Encyclopedia of Genes and Genomes (KEGG) pathway analysis further underscored these divergent functional profiles (Figure 2F; Supplementary Tables S8–S12). Notably, the EpC2 subgroup was uniquely enriched in multiple inflammatory and signal transduction pathways, most prominently the interleukin-17 (IL-17) signaling pathway.

**Figure 3:**
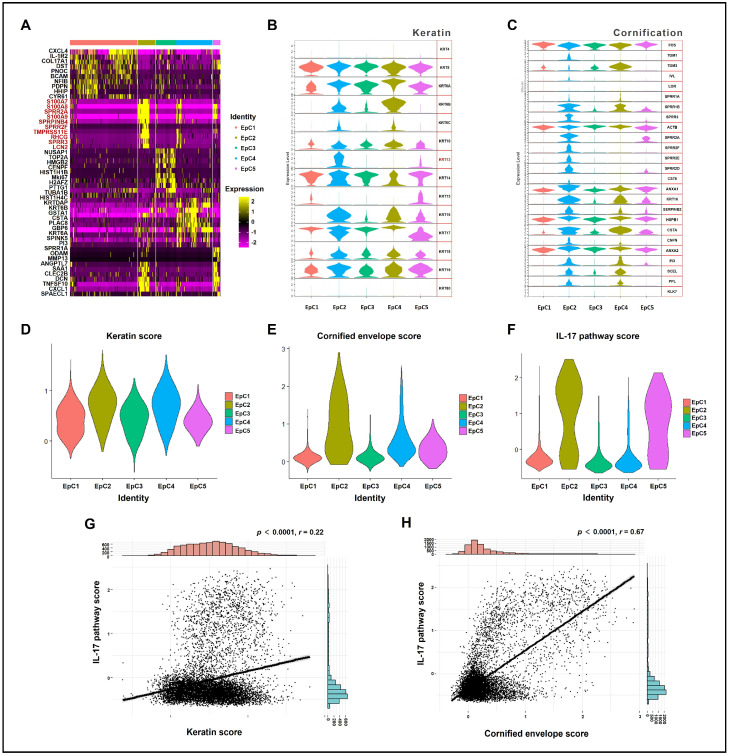
Transcriptomic profiling identifies EpC2 as a specialized subpopulation driven by keratinization and IL-17 pathway activation. (**A**) Heatmap detailing the top 10 DEGs per epithelial cluster, emphasizing the dominance of cornification and keratinization-related genes in EpC2. (**B**) Violin plots illustrating the cluster-specific expression of keratin genes, with KRT13 demonstrating highly specific enrichment in EpC2. (**C**) Expression profiles of cornification-related genes across all epithelial clusters, peaking prominently in EpC2. (**D**–**F**) Overall module scores for (**D**) keratin genes, (**E**) cornification-related genes, and (**F**) the IL-17 signaling pathway within the EpC populations. (**G**,**H**) Pearson correlation analyses revealing a significant positive association between single-cell IL-17 pathway scores and both (**G**) keratin scores (*p* < 0.0001, *r* = 0.22) and (**H**) cornified envelope scores (*p* < 0.0001, *r* = 0.67). Marginal histograms on the top and right show the frequency distributions of the Keratin and IL-17 pathway scores, respectively. DEG, differentially expressed gene; EpC, epithelial cell.

An analysis of the top 10 significantly upregulated DEGs in the EpC2 subgroup identified structural proteins responsible for cornified envelope assembly (e.g., *SPRR2A*/*2F*/*3*), markers of epithelial antimicrobial and inflammatory stress responses (e.g., *S100A7*/*8*/*9*, *LCN2*), and key components of the peptidase regulatory network (e.g., *TMPRSS11E*, *SERPINB4*) (Figure 3A; Supplementary Table S13). This transcriptional profile aligned perfectly with the GO enrichment results. Furthermore, expression profiling and module scoring of the keratin gene family revealed that the majority of *KRT* genes were highly enriched in EpC2, with *KRT13* demonstrating a remarkably specific expression pattern within this subpopulation (Figure 3B,D). Similarly, analysis of cornification-related genes confirmed that core structural proteins (e.g., *IVL*, *SPRR* family, *CNFN*, *PPL*, *SCEL*) and critical cross-linking enzymes (*TGM1*) were almost exclusively expressed in the EpC2 subgroup (Figure 3C,E). Violin plots of the IL-17 pathway module scores demonstrated that EpC2 exhibited significantly elevated IL-17 signaling activation (Figure 3F).

To evaluate the relationship between IL-17 signaling activity and epithelial keratinization at single-cell resolution, we treated individual cells as independent samples and performed Pearson correlation analyses. Scatter plots revealed significant positive correlations between the single-cell IL-17 pathway score and both the keratin score (*p* < 0.0001, *r* = 0.22) and the cornified envelope score (*p* < 0.0001, *r* = 0.67) (Figure 3G,H; Supplementary Table S14).

### 2.3. IL-17 and KRT13 Are Significantly Upregulated and Spatially Co-Localized in OKC Tissues

**Figure 4:**
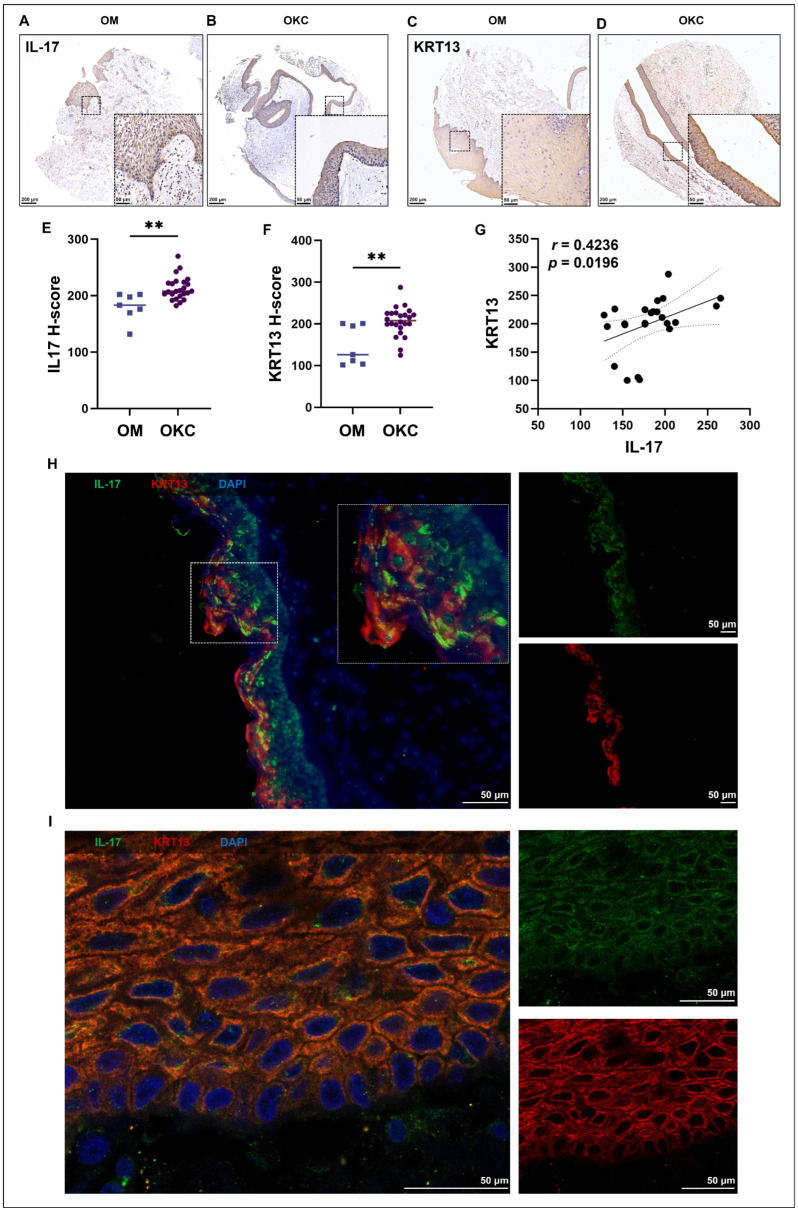
IL-17 and KRT13 are significantly upregulated and spatially co-localized in OKC tissues. (**A**–**F**) Representative immunohistochemical staining and corresponding H-score quantification demonstrating marked upregulation of IL-17 and KRT13 in OKC tissues (*n* = 24) compared to OM (*n* = 7). **, *p* < 0.01. (**G**) Tissue microarray correlation analysis revealing a moderate but statistically significant positive correlation between IL-17 and KRT13 expression intensities in OKC samples (*n* = 24). (**H**,**I**) Dual immunofluorescence co-localization analysis illustrating the spatial overlap of IL-17 and KRT13 within the epithelial cells. OKC, odontogenic keratocyst; OM, oral mucosa.

Using tissue microarrays, IHC and subsequent H-score analysis confirmed that both IL-17 and KRT13 were significantly upregulated in OKC tissues compared to oral mucosa (OM) (Figure 4A–F). Correlation analysis of their expression levels within the OKC epithelium revealed a moderate, statistically significant positive correlation (*r* = 0.4236, *p* = 0.0196) (Figure 4G). Furthermore, dual IF staining demonstrated strong spatial co-localization of IL-17 and KRT13 within the OKC epithelium (Figure 4H,I).

### 2.4. IL-17 Stimulates Epithelial Proliferation and KRT13 Expression in Vitro

**Figure 5:**
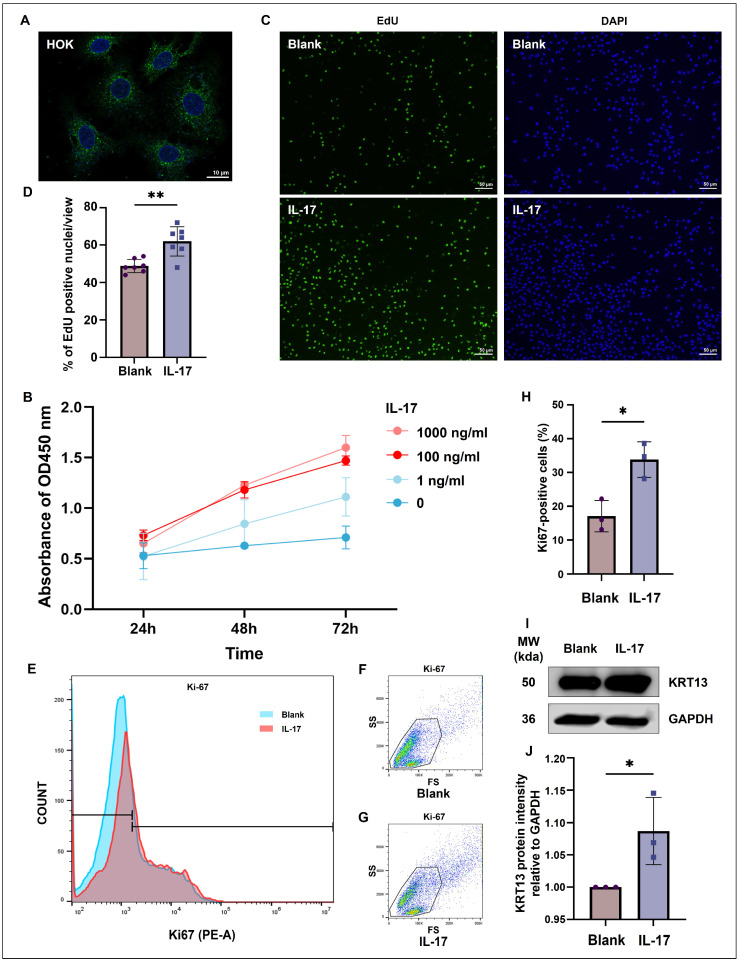
IL-17 stimulation promotes proliferation and upregulates KRT13 expression in HOKs. (**A**) Baseline immunofluorescence confirming endogenous KRT13 expression in untreated HOKs. (**B**) CCK-8 assay demonstrating that exogenous IL-17 enhances HOK proliferation in a time- and dose-dependent manner (*n* = 3). (**C**,**D**) EdU incorporation assay imaging (**C**) and quantification (**D**) showing a significant increase in actively proliferating HOKs following IL-17 stimulation. (**E**) Representative flow cytometry overlay histogram of Ki67 expression, with the Blank group shown in blue and the IL-17 group shown in red. (**F**,**G**) Representative forward scatter (FS) and side scatter (SS) plots showing the cell populations for the Blank and IL-17 groups. (**H**) Flow cytometry quantification revealing a markedly increased proportion of Ki67-positive cells after IL-17 treatment (*n* = 3). (**I**,**J**) Western blot and densitometric analysis confirming the direct upregulation of KRT13 protein levels upon IL-17 stimulation. *, *p* < 0.05; **, *p* < 0.01. CCK-8, Cell Counting Kit-8; HOKs, human oral keratinocytes; OD, optical density.

To model OKC epithelial behavior in vitro, we utilized the human oral keratinocyte (HOK) cell line. Baseline IF confirmed KRT13 expression in untreated HOKs (Figure 5A). Cell Counting Kit-8 (CCK-8) assays demonstrated exogenous IL-17 significantly promoted HOK proliferation time- and dose-dependently, with the proliferative effect plateauing near saturation at a concentration of 100 ng/mL (Figure 5B).

To minimize potential non-specific or off-target effects associated with supraphysiological dosing, we sought to determine the minimal concentration required for a robust effect. Based on our optimization data and corroborating previous literature [22], we established 50 ng/mL as the optimal effective concentration for subsequent IL-17 stimulation assays. Following stimulation at this dosage, 5-ethynyl-2′-deoxyuridine (EdU) incorporation assays revealed a significant increase in the number of actively proliferating HOK nuclei (Figure 5C,D). Flow cytometry further corroborated this finding, showing a marked increase in the proportion of Ki-67-positive cells (Figure 5E–H). Finally, Western blot analysis confirmed that IL-17 stimulation directly upregulated KRT13 protein expression in HOK cells (Figure 5I,J).

### 2.5. EHF Is the Core Transcriptional Integrator of the IL-17 Axis

**Figure 6:**
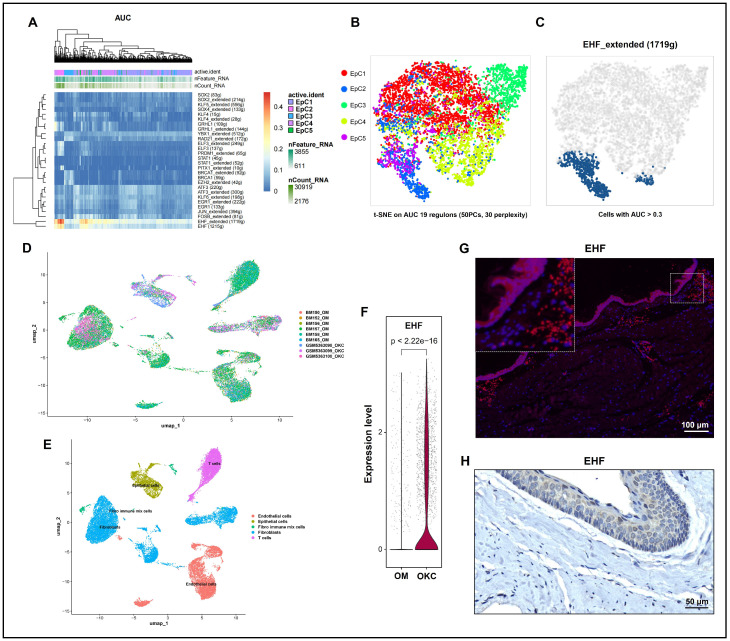
Transcriptomic profiling maps EHF as the central transcriptional driver of EpC2 with robust expression in the OKC epithelium. (**A**–**C**) pySCENIC regulon activity analysis and t-SNE visualization highlighting the specific and robust activation of the EHF regulon within the EpC2 subpopulation. g = gene number. (**D**) UMAP visualization demonstrating the spatial distribution and successful integration of cells across all utilized datasets. (**E**) Unsupervised graph-based clustering partitioning the cells into five distinct major clusters, robustly annotated via canonical lineage markers as epithelial cells, T cells, macrophages/monocytes, fibroblasts, and endothelial cells. (**F**) Differential expression analysis revealing significant EHF upregulation in OKC EpCs compared to OM tissues. (**G**,**H**) Immunofluorescence and immunohistochemistry validation confirming the specific localization of EHF protein within the OKC epithelial layers. AUC, area under the curve; EpCs, epithelial cells; GEO, Gene Expression Omnibus; OKC, odontogenic keratocyst; OM, oral mucosa; SCENIC, single-cell regulatory network inference and clustering; scRNA-seq, single-cell RNA sequencing; t-SNE, t-distributed stochastic neighbor embedding.

To investigate the underlying transcriptional regulatory mechanisms driving the heterogeneity of the five EpC subgroups, we performed regulon analysis on the single-cell transcriptomic data using the pySCENIC algorithm. Heatmap visualization based on regulon area under the curve (AUC) scores revealed that the transcription factor ETS homologous factor (EHF) and its extended target gene networks—EHF_extended (1719 g) and EHF (1215 g)—were highly and specifically activated within the EpC2 subgroup (Figure 6A). Subsequent t-distributed stochastic neighbor embedding (t-SNE) dimensionality reduction, computed solely on the AUC scores of 19 key regulons, successfully segregated the five EpC subgroups into distinct spatial clusters (Figure 6B). This indicates that divergent transcriptional regulatory networks are primary drivers of epithelial heterogeneity. To further validate the core regulatory role of EHF in EpC2, cells exhibiting high EHF_extended regulon activity (AUC > 0.3) were highlighted in the t-SNE space. These high-activity cells mapped perfectly onto the spatial coordinates corresponding to the EpC2 subpopulation (Figure 6C).

We further investigated EHF expression by integrating our OKC scRNA-seq data with a publicly available scRNA-seq dataset of OM. UMAP visualization demonstrated successful integration across datasets (Figure 6D). Following unsupervised clustering, cells were partitioned into five distinct populations and robustly annotated based on canonical lineage markers (Figure 6E). Differential expression analysis of the scRNA-seq datasets revealed that EHF was significantly upregulated in OKC EpCs compared to OM (Figure 6F). Consistent with the transcriptomic profiles, IF and IHC confirmed robust EHF protein localization in situ within the epithelial layers of OKC tissues (Figure 6G,H).

**Figure 7:**
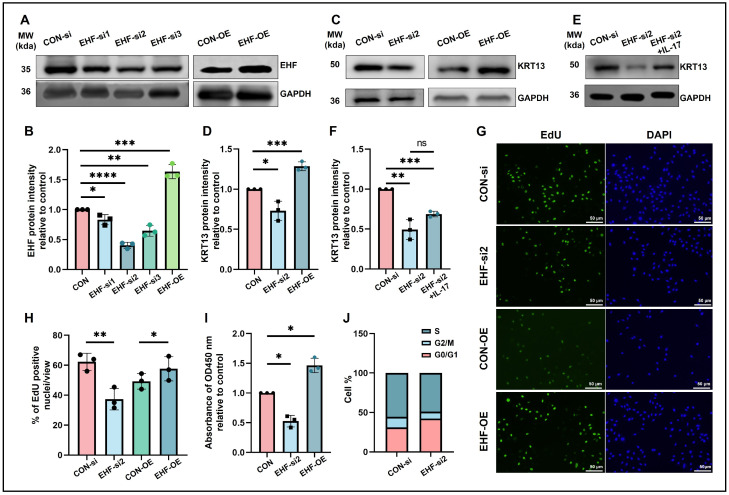
The transcription factor EHF is a critical downstream mediator of IL-17–induced proliferation and KRT13 upregulation. (**A**,**B**) Western blot verification and quantification of EHF knockdown (EHF-si2) and overexpression (EHF-OE) in engineered HOK cell lines (*n* = 3). (**C**,**D**) Western blot analysis showing that KRT13 expression tightly mirrors EHF levels: downregulated in EHF-si2 cells and upregulated in EHF-OE cells (*n* = 3). (**E**,**F**) Rescue experiments demonstrating that exogenous IL-17 stimulation fails to significantly rescue KRT13 expression in EHF-depleted (EHF-si2) cells, confirming EHF dependency (*n* = 3). (**G**,**H**) EdU assays revealing significantly blunted proliferation in EHF-si2 cells, contrasted by amplified proliferation in EHF-OE cells (*n* = 3). (**I**) CCK-8 assays corroborating the marked suppression of proliferative capacity following EHF knockdown and its enhancement upon EHF overexpression. (**J**) Flow cytometry cell cycle analysis demonstrating that EHF silencing induces a pronounced G0/G1 cell cycle arrest, impeding the G1-to-S phase transition. *, *p* < 0.05; **, *p* < 0.01; ***, *p* < 0.001; ****, *p* < 0.0001. CCK-8, Cell Counting Kit-8; DAPI, 4′,6-diamidino-2-phenylindole; EdU, 5-ethynyl-2′-deoxyuridine; HOK, human oral keratinocyte; OE, overexpression.

To functionally characterize EHF, we engineered HOK cell lines with EHF knockdown (EHF-si1/2/3 vs. CON-si) and overexpression (EHF-OE vs. CON-OE). Western blot analysis confirmed highly efficient knockdown (specifically via EHF-si2, *p* < 0.0001) and successful overexpression (*p* < 0.001) (Figure 7A,B). Strikingly, KRT13 expression directly mirrored EHF levels, decreasing significantly in EHF-knockdown cells and increasing in EHF-OE cells (Figure 7C,D). Notably, when EHF-knockdown cells were stimulated with exogenous IL-17, the expected KRT13 upregulation was largely abrogated; quantitative analysis indicated that any compensatory increase was not statistically significant (Figure 7E,F). This strongly suggests EHF as a key downstream mediator of IL-17-driven KRT13 expression.

Furthermore, EdU incorporation assays revealed that EHF knockdown significantly blunted HOK cell proliferation, whereas EHF overexpression robustly amplified it (Figure 7G,H). CCK-8 assays corroborated these highly significant differences in proliferative capacity (Figure 7I). Finally, flow cytometry demonstrated that silencing EHF induced a pronounced G0/G1 cell cycle arrest, effectively impeding the G1-to-S phase transition (Figure 7J).

## 3. Discussion

The aggressive clinical behavior and high recurrence of OKCs correlate with their distinct epithelial lining. By integrating scRNA-seq, spatial transcriptomics, and functional assays, we comprehensively decoded this epithelial heterogeneity, identifying a specialized EpC2 subpopulation driven by hyperactive IL-17 signaling and robust cornification. Crucially, our data suggest that the IL-17/EHF signaling axis may serve as an important driver of OKC pathogenesis, potentially contributing to the upregulation of KRT13 and the stimulation of cellular proliferation.

Molecularly, EpC2 harbored the highest CNV frequency (Figure 1A) and dominated keratinization processes (Figure 1D), evidenced by the upregulation of keratin and cornified envelope genes (Figure 3A–E). Stacked violin plots revealed that KRT13 expression is highly specific to EpC2 and largely absent in other clusters, highlighting it as a crucial identifier for this subset (Figure 3B). Previous studies link strong epithelial KRT13 expression in OKCs to active keratinization [23,24,25,26]. Recent spatial multi-omics of the healthy human tooth-associated epithelium (TAE) reveals a specialized, non-keratinized barrier that specifically lacks cornification markers (e.g., CNFN) to maintain permeability for immune surveillance [27]. Interestingly, this physiological “dynamic epithelium” handles constant environmental exposure by maintaining a non-keratinized, highly permeable state characterized by KRT19 expression rather than KRT13. In stark contrast, our data shows that the OKC EpC2 subpopulation responds to inflammatory cues by undergoing a pathological phenotypic shift, aberrantly overexpressing KRT13 alongside CNFN and IVL to drive rigid hyperkeratinization. This suggests that while normal mucosal barriers utilize inflammation to facilitate immune transmigration, the OKC epithelium misinterprets these signals, converting them into a hyperproliferative and hyperkeratotic drive. Corroborating this, our IHC analysis confirmed significantly higher KRT13 levels in OKC tissues versus OM (Figure 4C,D,F), cementing KRT13 as a defining marker for this pathological epithelial subpopulation.

Exploring the regulatory mechanisms behind EpC2 keratinization, KEGG enrichment analysis revealed pronounced activation of the IL-17 signaling pathway (Figure 2F and Figure 3F). Subsequent IHC confirmed markedly elevated IL-17 expression in OKC compared to OM (Figure 4A,B,E), suggesting IL-17 drives EpC2 keratinization. At single-cell resolution, correlation analysis showed the IL-17 pathway gene set positively correlates with keratin and cornified envelope gene sets (Figure 3G,H; Supplementary Table S14). Additionally, IHC and IF co-localization assays confirmed a strong positive correlation between epithelial IL-17 and KRT13 expression (Figure 4G–I).

The oral mucosa is inherently primed for inflammatory responsiveness; under homeostatic conditions, healthy oral epithelia continuously express immune mediators and acute-phase proteins to orchestrate local protection without inducing tissue damage [27]. However, our findings suggest that in OKCs, this physiological inflammatory capacity is hijacked. In vitro, we used HOKs, which retain epithelial traits and endogenously express KRT13 (Figure 5A). Exogenous IL-17 significantly upregulated KRT13 in HOKs (Figure 5I,J), indicating IL-17 directly drives OKC epithelial keratinization. IL-17 induces autocrine cytokine production in keratinocytes, fueling hyperproliferation and specific keratin expression in inflammatory diseases like psoriasis [28,29,30,31]. Aligning with this, our assays demonstrated that IL-17 markedly promoted HOK proliferation (Figure 5B–H). These functional outcomes mirror the transcriptomic signatures of EpC2, implying IL-17 synergistically amplifies keratinization and proliferation to fuel OKC pathological remodeling, turning a physiological mucosal defense mechanism into a driver of disease progression.

Mechanistically, pySCENIC identified the transcription factor EHF as a core regulator within EpC2 (Figure 6A–C). Corroborating this, a comparative analysis incorporating an external OM scRNA-seq dataset reveals that EHF was significantly upregulated in OKC EpCs (Figure 6D–F), with robust expression validated in situ via IF and IHC (Figure 6G,H). Although differential expression analysis of the scRNA-seq datasets revealed that EHF was significantly upregulated in OKC epithelial cells compared to OM (Figure 6F), quantitative IHC H-score analysis did not detect a statistically significant difference in EHF protein expression between OKC and OM tissues. This seemingly discordant result can be explained as follows. First, differences in sample size and sequencing depth between the OKC scRNA-seq dataset (our study) and the public OM dataset (GSE164241) may contribute to unbalanced statistical power. More importantly, our pySCENIC regulon analysis clearly demonstrated that EHF hyperactivation is not uniformly distributed across all OKC epithelial cells but is highly restricted to the pathogenic EpC2 subpopulation. In conventional IHC or whole-epithelium H-score quantification, the concentrated EHF signal within EpC2 is likely masked or diluted by the surrounding, non-pathogenic epithelial layers, which maintain a basal level of EHF essential for general epithelial homeostasis. Thus, single-cell resolution is crucial to unmask this localized transcriptional driver. Hypothesizing that EHF mediates IL-17-induced epithelial alterations, we developed EHF knockdown and overexpression models (Figure 7A,B). Depleting or overexpressing EHF profoundly downregulated or upregulated KRT13, respectively (Figure 7C,D). Crucially, exogenous IL-17 supplementation failed to significantly rescue KRT13 expression in EHF-depleted cells (Figure 7E,F). Rather than suggesting alternative pathways, this lack of significant rescue highlights that EHF is an indispensable bottleneck; IL-17 cannot efficiently drive KRT13 expression without it. Furthermore, EHF knockdown significantly impaired HOK proliferation and induced G0/G1 cell cycle arrest (Figure 7G–J). Together, these findings help to elucidate a functional IL-17/EHF axis, underscoring EHF as a critical downstream integrator that likely mediates IL-17-driven pathological epithelial differentiation and proliferation in OKCs [32].

Despite providing novel insights into OKC pathogenesis, this study has limitations. First, the scRNA-seq datasets were relatively limited in scale, and current spatial transcriptomics resolution prevented granular epithelial stratification. Second, technical bottlenecks hindered the isolation of primary OKC epithelial cells or patient-derived organoids or three-dimensional (3D) models, necessitating the use of normal OM-derived HOK cells as an in vitro proxy. Developing primary OKC cultures or organoid models remains a priority for future research to better replicate the in vivo environment. Finally, the potential cellular sources of IL-17 are highly diverse; however, the specific source of IL-17 in OKC remains to be conclusively determined [33]. A recent spatial transcriptomic atlas of the human oral mucosa identified highly organized subepithelial immune hubs, termed T-B-APC niches, located immediately adjacent to the basal epithelium [27]. Given this spatial architecture, it is highly plausible that T cells residing within analogous subepithelial inflammatory zones in the OKC capsular wall serve as the primary source of IL-17. Furthermore, recent maps of the oral mucosal barrier highlight that the stromal compartment plays an active, orchestrating role in establishing this immune zonation. Subepithelial stromal fibroblasts secrete specific chemokines (e.g., CXCL13, CCL19) to recruit and retain these immune cell aggregates [27]. This aligns perfectly with our previous single-cell profiling, which identified a highly active, CXCL-enriched fibroblast subpopulation within OKCs [21]. It is therefore highly probable that pathogenic stromal-epithelial cross-talk exists in OKCs, where cyst-wall fibroblasts actively orchestrate the recruitment of IL-17-producing cells to the subepithelial zone, thereby creating a localized inflammatory niche that fuels EpC2-driven hyperkeratinization. These recruited immune cells may secrete cytokines that diffuse to the EpC2 layer, activating the EHF transcriptional network. Further studies are required to clarify the cell type(s) responsible for IL-17 production within the OKC microenvironment.

## 4. Materials and Methods

### 4.1. Sample Collection and scRNA-Seq Processing

Following written informed consent and Institutional Review Board approval (IRB-ID: 2020A95), fresh OKC tissues (*n* = 3) were collected from the Department of Oral and Maxillofacial Surgery, School & Hospital of Stomatology, Wuhan University. Tissues were washed with Hanks’ balanced salt solution (Servicebio, Wuhan, China), minced (2–3 mm), and dissociated into single-cell suspensions (1 × 10^5^ cells/mL) in phosphate-buffered saline (PBS; Servicebio, Wuhan, China). For scRNA-seq, the dissociated cells were processed using the Singleron GEXSCOPE^®^ platform (Singleron Biotechnologies, Nanjing, China). In this workflow, single-cell suspensions were loaded onto GEXSCOPE^®^ chips, where individual cells were captured in microwells. Cells were then lysed, and the released mRNAs were captured by barcoded beads for subsequent reverse transcription and library preparation.

Raw sequencing reads were evaluated (FastQC), filtered (fastp), and trimmed (Cutadapt). Reads were mapped to the GRCh38 reference genome (Ensembl v92) via STAR 2.5.2b. FeatureCounts generated gene and unique molecular identifier (UMI) counts to construct expression matrices. Subsequent cell clustering identified EpCs, fibroblasts, myeloid cells, endothelial cells, pericytes, T cells, and plasma cells based on established protocols [21].

### 4.2. CNV, Enrichment, and Correlation Analyses

CNVs within EpC subgroups were inferred using the R package inferCNV (v1.14.2) (https://github.com/broadinstitute/inferCNV, accessed on 1 September 2023). T cells, myeloid cells, and plasma cells were selected as normal reference cells. Parameters were set with a cutoff of 1, and denoise processing was applied. The CNV scores were calculated as the sum of squared CNV regions. To identify DEGs among clusters, the FindAllMarkers function in the Seurat R package (v4.3.0.1) was utilized with a significance threshold of *p*-value < 0.05. We utilize the GO (http://www.geneontology.org) and KEGG (http://www.genome.jp/kegg) databases (accessed on 1 September 2023) for the functional enrichment of the screened DEGs [34,35,36]. The R package enrichplot (v1.18.4) was used to visualize the enrichment results. Furthermore, to evaluate specific biological activities, IL-17 pathway, keratin-related, and cornification-related gene module scores were calculated using the AddModuleScore function in Seurat. Pearson correlation coefficients between these modules were calculated and plotted using ggpubr 1.18.4.

### 4.3. DEG Identification and Gene Set Extraction

The DoHeatmap function in the Seurat package was used to plot the expression profiles of the identified DEGs. Keratin-associated genes were extracted from all GO-enriched functional pathways across the five EpC subgroup. Simultaneously, cornification-related genes were integrated from both the Cornified Envelope pathway and relevant published literature [37]. The expression patterns of these two specific gene sets were visualized using the VlnPlot function in Seurat.

### 4.4. pySCENIC Analysis

Gene regulatory networks were inferred utilizing pySCENIC, scanpy, and cistarget resources, incorporating appropriate gene and transcription factor (TF) annotation databases (https://resources.aertslab.org/cistarget/databases/; https://resources.aertslab.org/cistarget/motif2tf/; https://resources.aertslab.org/cistarget/tf_lists/) (accessed on 10 October 2023). Seurat expression matrices were converted to .loom format and processed sequentially through the GRN, ctx, and AUCell modules to generate AUC matrices. Nineteen representative regulons were clustered and visualized using seaborn.clustermap. Dimensionality reduction was performed via t-SNE (PC = 50, perplexity = 30) in scanpy. Cells exhibiting high EHF-regulon activity (AUC > 0.3) were mapped using matplotlib.pyplot.scatter.

### 4.5. Comparative scRNA-Seq Analysis of EHF Expression

To compare EHF expression between OM and OKC, single-cell transcriptomic data from Gene Expression Omnibus (GEO) datasets GSE164241 (OM samples: BM150, BM152, BM156, BM157, BM158, BM165, BM168, BM169) and GSE176351 (OKC samples from this study) were integrated. Utilizing the first 15 principal components, a shared nearest neighbor graph (FindNeighbors, dims = 1:15) and UMAP embeddings (RunUMAP, dims = 1:15) were generated. Unsupervised clustering (FindClusters, resolution = 0.1) partitioned the cells into five major clusters, which were manually annotated via canonical markers as epithelial cells, T cells, macrophages/monocytes, fibroblasts, and endothelial cells. The epithelial cluster was subsequently isolated to evaluate differential EHF expression between OM and OKC using the FindMarkers function (Wilcoxon rank-sum test).

### 4.6. Spatial Transcriptomic

Two OKC tissues from two distinct patients were formalin-fixed paraffin-embedded and sectioned at 10 μm for subsequent spatial transcriptomics studies. Sections were processed using the 10× Genomics Visium platform (10× Genomics, Pleasanton, CA, USA). Following hematoxylin and eosin (H&E) staining (Servicebio, Wuhan, China) and imaging, RNA probes were hybridized, ligated, and digested with RNase (Servicebio, Wuhan, China). Sequencing libraries were prepared and sequenced on an Illumina NovaSeq 6000 (PE150) (Illumina, San Diego, CA, USA). Raw reads were quality-filtered (Q > 20) and aligned using Space Ranger (10x Genomics, Pleasanton, CA, USA) to generate a gene-spot matrix. Spatial data analysis was conducted in Seurat v4.3.0.1, employing the same quality control, principal component analysis (PCA) (*n* = 10), and clustering pipeline as the scRNA-seq data. To integrate spatial and single-cell modalities, Cell2location was used to deconvolute spatial cell-type compositions, with results visualized via ggplot2.

### 4.7. IHC, Scoring and IF

A tissue microarray comprising 24 OKC and 7 OM samples (Servicebio, Wuhan, China) was deparaffinized, rehydrated, antigen retrieved, and blocked as detailed in Supplementary Table S15. Sections were incubated overnight at 4 °C with primary antibodies against KRT13, IL-17, and EHF (1:200; Proteintech, Wuhan, China). Slides were scanned using the Aperio ScanScope CS (Leica Biosystems, Vista, CA, USA). To ensure reproducibility and mitigate potential bias arising from intra-tumoral heterogeneity, the H-score ((1+) × 1 + (2+) × 2 + (3+) × 3) was independently quantified by three blinded observers. The average scores from the independent evaluations were used for the final statistical analysis.

For IF, slides were incubated with primary antibodies against IL-17 and KRT13 (1:200; Proteintech, Wuhan, China), followed by fluorescent secondary antibodies (Dylight 488, Goat Anti-Rabbit IgG; Dylight 594, Goat Anti-Mouse IgG; Abbkine, Wuhan, China). Nuclei were counterstained with 4′,6-diamidino-2-phenylindole (DAPI) (AntGene, Wuhan, China). Images were acquired using Olympus IX83 (Olympus Corporation, Tokyo, Japan) and ZEISS LSM 980 microscopes (Carl Zeiss AG, Oberkochen, Germany). Cultured cells at ~70–80% confluence were similarly fixed and stained for KRT13.

### 4.8. Cell Culture

HOKs (Catalog No. 2610, ScienCell, Carlsbad, CA, USA) were cultured in Dulbecco’s Modified Eagle Medium (DMEM) (HyClone, Logan, UT, USA) supplemented with 10% fetal bovine serum (FBS; Gibco, Grand Island, NY, USA) at 37 °C in a 5% CO_2_ humidified incubator.

### 4.9. Construction of Knockdown and Overexpression Cell Lines

EHF knockdown was achieved by transfecting cells with small interfering RNAs (siRNAs) designed via siDirect 2 (EHF-si1, si2, si3) [38,39] or a non-targeting control (CON-si) (Sangon Biotech, Shanghai, China) (Supplementary Table S1). Transfections were performed using Lipofectamine™ 3000 Transfection Reagent (Thermo Fisher Scientific, Waltham, MA, USA) at a final siRNA concentration of 100 nM. Knockdown efficiency was evaluated at 7 days post-transfection via Western blot to select the most effective sequence for downstream assays.

Stable EHF-overexpressing HOK cell lines (EHF-OE) and empty vector-transduced control cells (CON-OE) were established using a GV358 lentiviral vector (pGC-EGFP-IRES-puromycin). Cells were transduced at a multiplicity of infection (MOI) of 50 in the presence of HitransG P transduction reagent (GeneChem, Shanghai, China). Transduction was confirmed via enhanced green fluorescent protein (EGFP) expression, insert integrity was verified by Sanger sequencing (Supplementary Table S1), and final EHF overexpression was validated by Western blot.

### 4.10. Western Blot Analysis

HOK cells were seeded at an initial density of 6 × 10^5^ cells/well in six-well plates and cultured to 70–80% confluence. Cells were harvested and lysed on ice in radioimmunoprecipitation assay (RIPA) buffer (Servicebio, Wuhan, China) supplemented with protease and phosphatase inhibitors (Servicebio, Wuhan, China) to prevent protein degradation. Protein concentrations were determined using a bicinchoninic acid (BCA) assay (Beyotime, Shanghai, China). Equal amounts of protein were separated via sodium dodecyl sulfate-polyacrylamide gel electrophoresis (SDS-PAGE), transferred to polyvinylidene fluoride (PVDF) membranes (Immobilon-P, Merck Millipore, Burlington, MA, USA), blocked in 5% non-fat milk, and incubated overnight with primary antibodies against EHF (1:1000; CUSABIO, Wuhan, China) or KRT13 (1:1000; Proteintech, Wuhan, China). GAPDH (1:1000; Proteintech, Wuhan, China) served as the loading control. Following incubation with horseradish peroxidase (HRP)-conjugated secondary antibodies, signals were visualized using enhanced chemiluminescence and quantified with ImageStudio software, version 6.1 (LI-COR Biosciences, Lincoln, NE, USA).

### 4.11. Cell Proliferation and Cell Cycle Analysis

To evaluate cell proliferation and cell cycle dynamics, cells were initially seeded at a density of 6 × 10^5^ cells per well. Proliferation was comprehensively assessed using a CCK-8 assay, EdU incorporation, and Ki-67 flow cytometry. For the CCK-8 assay (Biosharp, Hefei, China), treated cells were incubated with 10 μL of reagent for 2 h at 37 °C, after which the absorbance of 100 μL of supernatant was measured at 450 nm. In parallel, the EdU incorporation assay (Beyotime, Shanghai, China) was performed by incubating cells with 50 μM EdU for 2 h. These cells were subsequently fixed, permeabilized, and stained via a Click-iT reaction, utilizing DAPI as a nuclear counterstain prior to quantification under a fluorescence microscope. For Ki-67 expression analysis, cells were fixed with 4% paraformaldehyde, permeabilized using 0.1% Triton X-100, and sequentially stained with a primary anti-Ki-67 antibody (ImmunoWay, San Jose, CA, USA) and a DyLight 488-conjugated secondary antibody (Abbkine, Wuhan, China). Data acquisition was performed using a CytoFLEX flow cytometer (Beckman Coulter, Brea, CA, USA), and the results were analyzed using FlowJo 10 software (Tree Star, Ashland, OR, USA).

Finally, to determine cell cycle distribution, cells were fixed in 70% cold ethanol at −20 °C, treated with RNase A (Servicebio, Wuhan, China), and stained with propidium iodide (PI; Servicebio, Wuhan, China). The stained cells were then run on the CytoFLEX flow cytometer and analyzed using FlowJo 10 software to quantify the proportions of cells in the G0/G1, S, and G2/M phases.

### 4.12. Statistical Analysis

For all in vitro assays (including CCK-8, EdU incorporation, flow cytometry, and Western blotting), experiments were performed in at least three independent biological replicates (*n* ≥ 3). The “*n*” values specified in the figure legends represent these independent biological replicates rather than technical replicates. Statistical analyses were performed using GraphPad Prism 9.3.1 (GraphPad Software, San Diego, CA, USA), with bioinformatics visualizations generated in R 4.2.3 (R Foundation for Statistical Computing, Vienna, Austria) and Python 3.8 (Python Software Foundation, Wilmington, DE, USA). Data distribution normality and variance homogeneity were verified using Shapiro–Wilk and Levene tests, respectively. Group comparisons were conducted using Student’s *t*-tests or one-way analysis of variance (ANOVA), as appropriate. Pearson correlation was used to assess variable associations. Statistical significance was defined as *p* < 0.05. Fluorescence intensity quantification was performed using ImageJ 1.8.0 (National Institutes of Health, Bethesda, MD, USA).

## 5. Conclusions

In conclusion, we present a comprehensive single-cell and spatial transcriptomic atlas of the OKC epithelium. We identified EpC2, a highly active subpopulation likely responsible for OKC’s characteristic hyperkeratinization and rapid proliferation. Crucially, we propose a novel pathogenic mechanism where elevated IL-17 signaling promotes robust KRT13 expression and cellular proliferation—a process that appears to be mediated by the transcription factor EHF. By highlighting the IL-17/EHF regulatory axis, our findings provide novel insights into the molecular drivers of OKC’s aggressive clinical behavior and highlight both the IL-17 pathway and EHF as promising therapeutic targets to manage OKC growth and prevent recurrence.

## Data Availability

The original sequence data and processed gene barcode matrices generated in this study have been deposited in the NCBI Gene Expression Omnibus (GEO) database under accession numbers GSE176351 (scRNA-seq data of OKC) and GSE304124 (spatial transcriptomics of OKC). The publicly available scRNA-seq dataset of OM analyzed in this study can be accessed in GEO under the accession number GSE164241.

## Supplementary Materials

The following supporting information can be downloaded at: https://www.mdpi.com/article/10.3390/ijms27094115/s1.

## Abbreviations

The following abbreviations are used in this manuscript:

ANOVA	analysis of variance
AUC	area under the curve
BCA	bicinchoninic acid
CCK-8	Cell Counting Kit-8
CNVs	chromosomal copy number variations
DAPI	4′,6-diamidino-2-phenylindole
DEGs	differentially expressed genes
DMEM	Dulbecco’s Modified Eagle Medium
EdU	5-ethynyl-2′-deoxyuridine
EGFP	enhanced green fluorescent protein
EHF	ETS homologous factor
EpCs	epithelial cells
FBS	fetal bovine serum
GEO	Gene Expression Omnibus
GO	Gene Ontology
H&E	hematoxylin and eosin
HOKs	human oral keratinocytes
HRP	horseradish peroxidase
IF	immunofluorescence
IHC	immunohistochemistry
IL-17	interleukin-17
KEGG	Kyoto Encyclopedia of Genes and Genomes
KRT13	keratin 13
MOI	multiplicity of infection
OKCs	odontogenic keratocysts
OM	oral mucosa
PBS	phosphate-buffered saline
PCA	principal component analysis
PI	propidium iodide
PVDF	polyvinylidene fluoride
RIPA	radio immunoprecipitation assay
scRNA-seq	single-cell RNA sequencing
SDS-PAGE	sodium dodecyl sulfate-polyacrylamide gel electrophoresis
siRNAs	small interfering RNAs
TF	transcription factor
t-SNE	t-distributed stochastic neighbor embedding
UMAP	Uniform Manifold Approximation and Projection
UMI	unique molecular identifier

## References

[1] Stoelinga P.J.W. (2022). The odontogenic keratocyst revisited. Int. J. Oral Maxillofac. Surg..

[2] Mustakim K.R., Sodnom-Ish B., Yoon H.J., Kim S.M. (2022). Odontogenic Keratocyst in the Masseter Muscle. J. Craniofac. Surg..

[3] Krishan V., Mathew P., Chitran P. (2022). Peripheral Odontogenic Keratocyst of Buccal Mucosa. J. Coll. Physicians Surg. Pak..

[4] Borghesi A., Nardi C., Giannitto C., Tironi A., Maroldi R., Di Bartolomeo F., Preda L. (2018). Odontogenic keratocyst: Imaging features of a benign lesion with an aggressive behaviour. Insights Imaging.

[5] Fidele N.B., Bing L., Sun Y., Wu T., Zheng Y., Zhao Y. (2019). Management of mandibular odontogenic keratocyst through radical resection: Report of 35 cases. Oncol. Lett..

[6] Valdivia A., Ramos-Ibarra M.L., Franco-Barrera M.J., Arias-Ruiz L.F., García-Cruz J.M., Torres-Bugarín O. (2022). What is Currently Known about Odontogenic Keratocysts?. Oral Health Prev. Dent..

[7] Li J., Jiang E.H., Jiang S.C., Liu B., Xiong X.P., Sun Y.F., Deng W.W. (2023). A retrospective study of the malignant change of odontogenic keratocyst. J. Stomatol. Oral Maxillofac. Surg..

[8] Chen P., Liu B., Wei B., Yu S. (2022). The clinicopathological features and treatments of odontogenic keratocysts. Am. J. Cancer Res..

[9] Cserni D., Zombori T., Stájer A., Rimovszki A., Cserni G., Baráth Z. (2020). Immunohistochemical Characterization of Reactive Epithelial Changes in Odontogenic Keratocysts. Pathol. Oncol. Res..

[10] Kureel K., Urs A.B., Augustine J. (2019). Cytokeratin and fibronectin expression in orthokeratinized odontogenic cyst: A comparative immunohistochemical study. J. Oral Maxillofac. Pathol..

[11] Tsuji K., Wato M., Hayashi T., Yasuda N., Matsushita T., Ito T., Gamoh S., Yoshida H., Tanaka A., Morita S. (2014). The expression of cytokeratin in keratocystic odontogenic tumor, orthokeratinized odontogenic cyst, dentigerous cyst, radicular cyst and dermoid cyst. Med. Mol. Morphol..

[12] Yin L., Li Q., Mrdenovic S., Chu G.C., Wu B.J., Bu H., Duan P., Kim J., You S., Lewis M.S. (2022). KRT13 promotes stemness and drives metastasis in breast cancer through a plakoglobin/c-Myc signaling pathway. Breast Cancer Res..

[13] Mohanty S., Dabas J., Verma A., Gupta S., Urs A.B., Hemavathy S. (2021). Surgical management of the odontogenic keratocyst: A 20-year experience. Int. J. Oral Maxillofac. Surg..

[14] Singh A.K., Khanal N., Chaulagain R., Bhujel N., Singh R.P. (2022). How effective is 5-Fluorouracil as an adjuvant in the management of odontogenic keratocyst? A systematic review and meta-analysis. Br. J. Oral Maxillofac. Surg..

[15] Ally M.S., Tang J.Y., Joseph T., Thompson B., Lindgren J., Raphael M.A., Ulerio G., Chanana A.M., Mackay-Wiggan J.M., Bickers D.R. (2014). The use of vismodegib to shrink keratocystic odontogenic tumors in patients with basal cell nevus syndrome. JAMA Dermatol..

[16] Al-Moraissi E.A., Kaur A., Gomez R.S., Ellis E. (2023). Effectiveness of different treatments for odontogenic keratocyst: A network meta-analysis. Int. J. Oral Maxillofac. Surg..

[17] Liu X.H., Zhong N.N., Yi J.R., Lin H., Liu B., Man Q.W. (2025). Trends in Research of Odontogenic Keratocyst and Ameloblastoma. J. Dent. Res..

[18] Sklavenitis-Pistofidis R., Getz G., Ghobrial I. (2021). Single-cell RNA sequencing: One step closer to the clinic. Nat. Med..

[19] Su M., Pan T., Chen Q.Z., Zhou W.W., Gong Y., Xu G., Yan H.-Y., Li S., Shi Q.-Z., Zhang Y. (2022). Data analysis guidelines for single-cell RNA-seq in biomedical studies and clinical applications. Mil. Med. Res..

[20] Robles-Remacho A., Sanchez-Martin R.M., Diaz-Mochon J.J. (2023). Spatial Transcriptomics: Emerging Technologies in Tissue Gene Expression Profiling. Anal. Chem..

[21] Man Q.W., Li R.F., Li S.R., Wang J., Bu L.L., Zhao Y., Liu B. (2021). Single-Cell RNA Sequencing Reveals CXCLs Enriched Fibroblasts Within Odontogenic Keratocysts. J. Inflamm. Res..

[22] Zhu L., Wu Y., Wei H., Xing X., Zhan N., Xiong H., Peng B. (2011). IL-17R activation of human periodontal ligament fibroblasts induces IL-23 p19 production: Differential involvement of NF-κB versus JNK/AP-1 pathways. Mol. Immunol..

[23] Naganuma K., Hatta M., Ikebe T., Yamazaki J. (2014). Epigenetic alterations of the keratin 13 gene in oral squamous cell carcinoma. BMC Cancer.

[24] Dos Santos J.N., Oliveira G.Q., Gurgel C.A., de Souza R.O., Sales C.B., Neto A.A., Ramos E.A.G. (2009). Altered expression of cytokeratins in primary, recurrent and syndrome keratocystic odontogenic tumors. J. Mol. Histol..

[25] Aragaki T., Michi Y., Katsube K., Uzawa N., Okada N., Akashi T., Amagasa T., Yamaguchi A., Sakamoto K. (2010). Comprehensive keratin profiling reveals different histopathogenesis of keratocystic odontogenic tumor and orthokeratinized odontogenic cyst. Hum. Pathol..

[26] Dmello C., Srivastava S.S., Tiwari R., Chaudhari P.R., Sawant S., Vaidya M.M. (2019). Multifaceted role of keratins in epithelial cell differentiation and transformation. J. Biosci..

[27] Theofilou V.I., Fraser D., Kanasi E., Brenchley L., Greenwell-Wild T., Adade E.E., Valm A.M., Douagi I., Belkaid Y., Tran D.T. (2026). Distinct spatial organization governs oral mucosal immunity. Nat. Immunol..

[28] Borowczyk J., Buerger C., Tadjrischi N., Drukala J., Wolnicki M., Wnuk D., Modarressi A., Boehncke W.-H., Brembilla N.C. (2020). IL-17E (IL-25) and IL-17A Differentially Affect the Functions of Human Keratinocytes. J. Investig. Dermatol..

[29] Eyerich K., Dimartino V., Cavani A. (2017). IL-17 and IL-22 in immunity: Driving protection and pathology. Eur. J. Immunol..

[30] Furue M., Furue K., Tsuji G., Nakahara T. (2020). Interleukin-17A and Keratinocytes in Psoriasis. Int. J. Mol. Sci..

[31] Yang L., Zhang S., Wang G. (2019). Keratin 17 in disease pathogenesis: From cancer to dermatoses. J. Pathol..

[32] Singh H., Shetty D., Kumar A., Chavan R., Shori D., Mali J. (2013). A molecular insight into the role of inflammation in the behavior and pathogenesis of odontogenic cysts. Ann. Med. Health Sci. Res..

[33] Kuen D.S., Kim B.S., Chung Y. (2020). IL-17-Producing Cells in Tumor Immunity: Friends or Foes?. Immune Netw..

[34] Aleksander S.A., Balhoff J., Carbon S., Cherry J.M., Drabkin H.J., Ebert D., Feuermann M., Gaudet P., Harris N.L., Hill D.P. (2023). The Gene Ontology knowledgebase in 2023. Genetics.

[35] Ashburner M., Ball C.A., Blake J.A., Botstein D., Butler H., Cherry J.M., Davis A.P., Dolinski K., Dwight S.S., Eppig J.T. (2000). Gene ontology: Tool for the unification of biology. Nat. Genet..

[36] Thomas P.D., Ebert D., Muruganujan T., Mushayahama T., Albou L.P., Mi H. (2022). PANTHER: Making genome-scale phylogenetics accessible to all. Protein Sci..

[37] Roy R.R., Ochiai T., Shimada K., Hasegawa H. (2023). Comprehensive cornified envelope protein profile of odontogenic keratocysts clarifies the characteristics of non-keratinized oral epithelium. J. Oral Pathol. Med..

[38] Naito Y., Yamada T., Ui-Tei K., Morishita S., Saigo K. (2004). siDirect: Highly effective, target-specific siRNA design software for mammalian RNA interference. Nucleic Acids Res..

[39] Naito Y., Yoshimura J., Morishita S., Ui-Tei K. (2009). siDirect 2.0: Updated software for designing functional siRNA with reduced seed-dependent off-target effect. BMC Bioinform..

