# Supplementary material for: Multiomics Reveals IL-17 Drives Epithelial Keratinization and Proliferation via EHF in Odontogenic Keratocysts
Source: Int J Mol Sci. 2026 May 4;27(9):4115. doi: 10.3390/ijms27094115 (PMC13163638; doi:10.3390/ijms27094115)
Supplement: Supplementary file 1 [file ijms-27-04115-s001.zip › ijms-4235677-supplementary/Supplementary Figure S1.pdf]

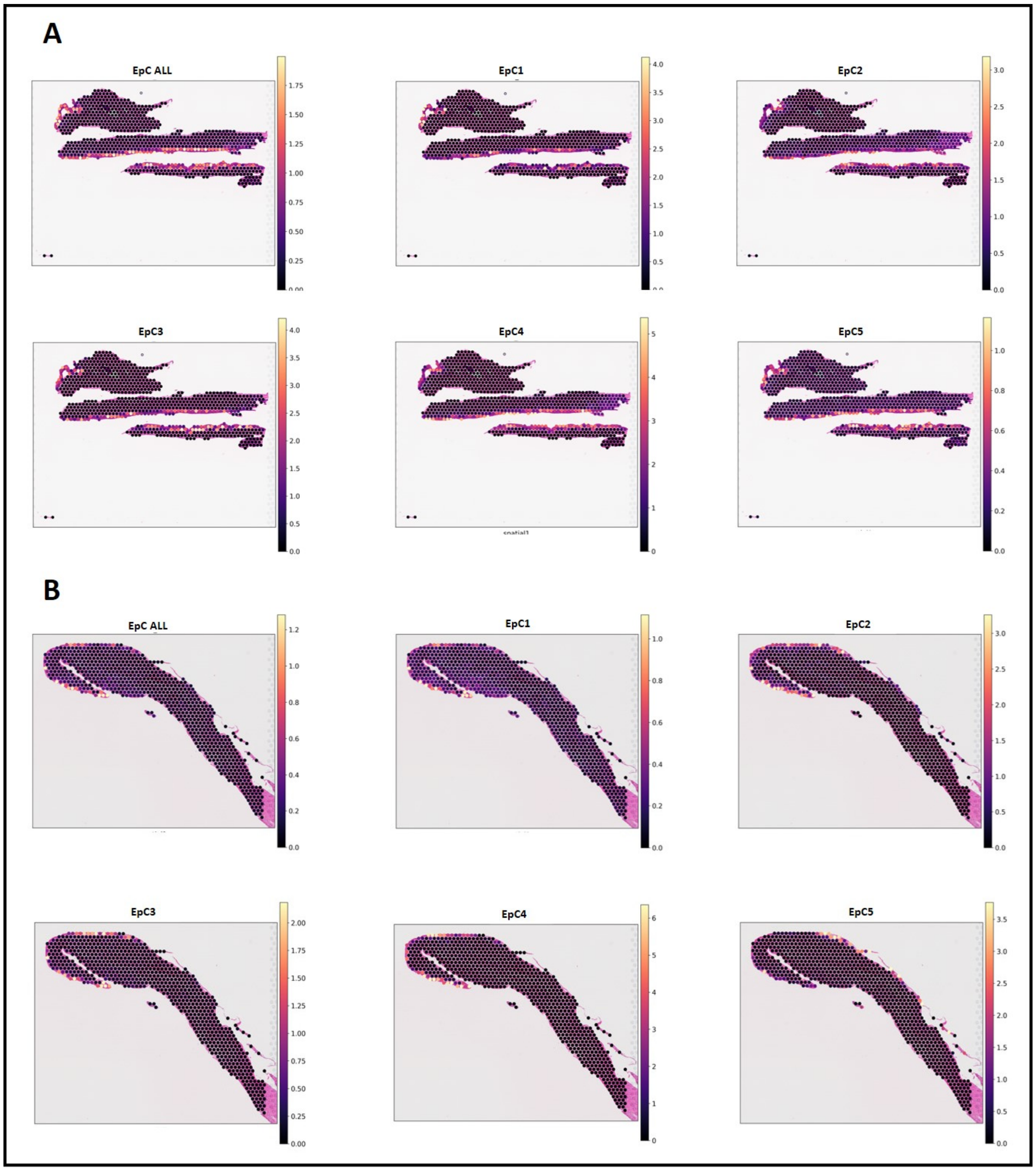

Supplementary Figure S1. Spatial distribution of epithelial cell subpopulations across independent OKC tissue samples. (**A, B**) Spatial transcriptomic maps illustrating the deconvoluted abundance and localization of the overall epithelial cell population (EpC ALL) and the five specific epithelial subpopulations (EpC1–EpC5) in two distinct odontogenic keratocyst (OKC) samples: Sample 1 (**A**) and Sample 2 (**B**). The color gradient within each hexagonal spot indicates the localized abundance of the designated cell type, estimated via Cell2location integration with single-cell RNA sequencing data. Darker colors (purple) represent lower abundance, while lighter colors (yellow) represent higher abundance within the spatial context.
