# Supplementary material for: Multiomics Reveals IL-17 Drives Epithelial Keratinization and Proliferation via EHF in Odontogenic Keratocysts
Source: Int J Mol Sci. 2026 May 4;27(9):4115. doi: 10.3390/ijms27094115 (PMC13163638; doi:10.3390/ijms27094115)
Supplement: Supplementary file 1 [file ijms-27-04115-s001.zip › ijms-4235677-supplementary/Supplementary Table S1.pdf]

Supplementary Table S1. Molecular Constructs and Reagents for EHF Modulation.

Category	Item / ID	
A. siRNA Sequences (designed via siDirect 2.0)		
Name	Sense Strand (5'→3')	Antisense Strand (5'→3')
EHF-si1	AUUUACCUAAAGAUUCUGGGU	ACCCAGAAUCUUUAGGUAAAU
EHF-si2	AAAUAGUUCUCGUCUUUCCAG	CUGGAAAGACGAGAACUAUUU
EHF-si3	UAAAUAGUUCUCGUCUUUCCA	UGGAAAGACGAGAACUAUUUA
B. Lentiviral Overexpression System		
Vector Backbone	GV358 (pGC-EGFP-IRES-puromycin)	
Insert Gene	Human EHF (full-length CDS)	
Cloning Strategy	AgeI / NheI digestion, In-fusion recombination	
Viral Titer	3×10^8 TU/mL	
Multiplicity of Infection (MOI)	50	
