# Supplementary material for: Multiomics Reveals IL-17 Drives Epithelial Keratinization and Proliferation via EHF in Odontogenic Keratocysts
Source: Int J Mol Sci. 2026 May 4;27(9):4115. doi: 10.3390/ijms27094115 (PMC13163638; doi:10.3390/ijms27094115)
Supplement: Supplementary file 1 [file ijms-27-04115-s001.zip › ijms-4235677-supplementary/Supplementary Table S10.pdf]

Supplementary Table S10. EpC3 KEGG enrichment.

ID	Description	GeneRatio	BgRatio	pvalue	p.adjust	qvalue	geneID	Count
hsa04110	Cell cycle	44/346	157/8577	1.04308782918963e-25	2.513841668347e-23	2.14107501781029e-23	PTTG1/CCNB1/TTK/CDC20/CDK1/RAD21/CCNB2/MAD2L1/PCNA/ESCO2/BUB1B/NDC80/KNL1/BUB3/MCM4/BUB1/SGO1/CCNA2/MCM7/PRKDC/SMC1A/MCM3/ORC6/PLK1/FBXO5/ANAPC11/SMC3/CDC6/AURKB/DBF4/MCM6/YWHAQ/TRIP13/CDCA5/CHEK1/CCNE2/CDC45/CDK4/MCM5/CDC25B/MCM2/YWHAE/RBX1/YWHAH	44
hsa03030	DNA replication	21/346	36/8577	9.27440443939195e-21	1.11756573494673e-18	9.51846771411279e-19	PCNA/RPA3/MCM4/MCM7/MCM3/FEN1/RFC2/POLD3/RFC4/POLE3/LIG1/MCM6/RPA2/RFC3/POLD2/RFC5/RNASEH2B/RFC1/POLA1/MCM5/MCM2	21
hsa03040	Spliceosome	40/346	216/8577	1.99780711081266e-16	1.60490504568617e-14	1.36692065476656e-14	SNRPG/LSM3/LSM4/HNRNPA3/RBMX/SNRPB/SNRPD1/LSM5/SRSF10/SRSF3/SNRPD2/HNRNPM/SNRPA1/SNRPC/HNRNPU/LSM2/SNRPF/SRSF2/MAGOHB/HNRNPA1/FUS/SRSF7/SNRPD3/SNRPE/PCBP1/DDX46/CWC15/LSM6/PPIH/SNRPA/TCERG1/HNRNPK/TRA2B/SNRNP40/RBM8A/LSM8/WBP11/LSM7/SRSF1/MAGOH	40
hsa05014	Amyotrophic lateral sclerosis	50/346	364/8577	9.4228214082105e-15	5.67724989844682e-13	4.83539519631854e-13	TUBA1B/TUBB/HNRNPA2B1/TUBB4B/TUBA1C/HNRNPA3/PFN1/PSMC3/UQCRC1/SRSF3/NDUFA6/TPR/CYC1/PSMA4/NDUFB6/HNRNPA1/FUS/COX7B/RAB8A/ACTB/SRSF7/DCTN3/ATP5MC1/VDAC1/EIF2S1/PSMA7/UQCRFS1/CYCS/NUP107/SIGMAR1/UQCRQ/TUBB6/TOMM40/NUP37/NDUFV1/BAX/ATP5F1B/NDUFB2/NDUFA2/PSMA3/ATP5MC3/ATP5PF/COX8A/NDC1/UQCR10/PSMD1/PSMB3/NUP93/NDUFA4/COX7A2	50
hsa03430	Mismatch repair	13/346	23/8577	4.76738834983604e-13	2.29788118462097e-11	1.95713837519585e-11	PCNA/RPA3/RFC2/POLD3/RFC4/LIG1/RPA2/RFC3/POLD2/RFC5/MSH2/RFC1/MSH6	13
hsa05012	Parkinson disease	39/346	266/8577	1.3448919620283e-12	5.40198271414701e-11	4.60094618588629e-11	TUBA1B/TUBB/CALM2/TUBB4B/TUBA1C/SLC25A5/PSMC3/VDAC3/UQCRC1/NDUFA6/GNAS/CYC1/PSMA4/NDUFB6/COX7B/PARK7/ATP5MC1/VDAC1/EIF2S1/PSMA7/CALM3/UQCRFS1/CYCS/UQCRQ/TUBB6/NDUFV1/BAX/ATP5F1B/NDUFB2/NDUFA2/PSMA3/ATP5MC3/ATP5PF/COX8A/UQCR10/PSMD1/PSMB3/NDUFA4/COX7A2	39
hsa05020	Prion disease	35/346	272/8577	8.23263223038847e-10	2.68506848891181e-08	2.28691135766675e-08	TUBA1B/TUBB/TUBB4B/TUBA1C/SLC25A5/PSMC3/VDAC3/UQCRC1/NDUFA6/CYC1/PSMA4/NDUFB6/COX7B/ATP5MC1/VDAC1/EIF2S1/PSMA7/UQCRFS1/CYCS/UQCRQ/TUBB6/NDUFV	35

							1/BAX/ATP5F1B/NDUFB2/NDUFA2/PSMA3/ATP5MC3/ATP5PF/COX8A/UQCR10/PSMD1/PSMB3/NDUFA4/COX7A2	
hsa03410	Base excision repair	14/346	44/8577	8.91309041962425e-10	2.68506848891181e-08	2.28691135766675e-08	HMGB1/PCNA/FEN1/PARP1/RFC2/POLD3/RFC4/POLE3/LIG1/RFC3/POLD2/RFC5/RFC1/PARP2	14
hsa05016	Huntington disease	37/346	306/8577	1.54424154180142e-09	4.13513568415714e-08	3.52195439358219e-08	TUBA1B/TUBB/TUBB4B/TUBA1C/SLC25A5/PSMC3/VDAC3/UQCRC1/NDUFA6/CYC1/PSMA4/NDUFB6/COX7B/DCTN3/ATP5MC1/VDAC1/PSMA7/UQCRFS1/CYCS/POLR2H/UQCRQ/TUBB6/POLR2E/NDUFV1/BAX/ATP5F1B/NDUFB2/NDUFA2/PSMA3/ATP5MC3/ATP5PF/COX8A/UQCR10/PSMD1/PSMB3/NDUFA4/COX7A2	37
hsa03420	Nucleotide excision repair	16/346	63/8577	2.26557540766187e-09	5.46003673246511e-08	4.65039162625332e-08	PCNA/RPA3/RFC2/POLD3/RFC4/POLE3/LIG1/RPA2/RFC3/POLD2/RFC5/RFC1/POLR2H/POLR2E/RAD23A/RBX1	16
hsa04114	Oocyte meiosis	22/346	131/8577	1.02283325997774e-08	2.24093468776942e-07	1.90863622675273e-07	PTTG1/CCNB1/CDC20/CDK1/CCNB2/MAD2L1/CALM2/AURKA/BUB1/SGO1/SMC1A/PLK1/FBXO5/ANAPC11/SMC3/YWHAQ/PPP1CA/CCNE2/CALM3/YWHAE/RBX1/YWHAH	22
hsa00190	Oxidative phosphorylation	22/346	134/8577	1.57466352909686e-08	3.16244925426953e-07	2.69350340503411e-07	UQCRC1/NDUFA6/CYC1/NDUFB6/COX7B/COX17/ATP5ME/ATP5MC1/UQCRFS1/CYCS/UQCRQ/NDUFV1/ATP5F1B/NDUFB2/NDUFA2/ATP5MC3/ATP5PF/COX8A/UQCR10/ATP5MF/NDUFA4/COX7A2	22
hsa04714	Thermogenesis	28/346	232/8577	1.75457544612791e-07	3.21856281294009e-06	2.74129612807739e-06	UQCRC1/NDUFA6/GNAS/CYC1/RHEB/NDUFB6/COX7B/COX17/COX20/ATP5ME/ACTB/ATP5MC1/UQCRFS1/UQCRQ/NDUFV1/ATP5F1B/NDUFB2/NDUFA2/NDUFAF8/ATP5MC3/ATP5PF/COA6/COX8A/UQCR10/ACTL6A/ATP5MF/NDUFA4/COX7A2	28
hsa03460	Fanconi anemia pathway	13/346	55/8577	1.8697045386374e-07	3.21856281294009e-06	2.74129612807739e-06	UBE2T/CENPX/RPA3/FANCI/BRCA2/USP1/BRCA1/RPA2/FANCD2/RAD51C/BRIP1/BLM/CENPS	13
hsa05010	Alzheimer disease	38/346	384/8577	2.21628860656426e-07	3.56083702787992e-06	3.03281598793004e-06	TUBA1B/TUBB/CALM2/TUBB4B/GAPDH/TUBA1C/SLC25A5/PSMC3/VDAC3/UQCRC1/NDUFA6/CYC1/PSMA4/NDUFB6/COX7B/ATP5MC1/VDAC1/EIF2S1/PSMA7/CALM3/UQCRFS1/CYCS/UQCRQ/TUBB6/NDUFV1/ATP5F1B/NDUFB2/NDUFA2/PSMA3/ATP5MC3/ATP5PF/NAE1/COX8A/UQCR10/PSMD1/PSMB3/NDUFA4/COX7A2	38

hsa03013	Nucleocytoplasmic transport	18/346	108/8577	2.59747916447554e-07	3.91245299149129e-06	3.3322923491627e-06	KPNA2/RAN/CSE1L/XPO1/KPNB1/TPR/MAGOHB/SRRM1/NUP107/NUP37/RBM8A/SUMO2/IPO5/NDC1/RANGAP1/RNPS1/NUP93/MAGOH	18
hsa03440	Homologous recombination	11/346	41/8577	4.18138078337973e-07	5.86452486643142e-06	4.99489997359305e-06	RPA3/BRCA2/BRCA1/POLD3/BARD1/RPA2/POLD2/TOPBP1/RAD51C/BRIP1/BLM	11
hsa05022	Pathways of neurodegeneration - multiple diseases	43/346	476/8577	4.38014305376621e-07	5.86452486643142e-06	4.99489997359305e-06	TUBA1B/TUBB/CALM2/TUBB4B/TUBA1C/SLC25A5/PSMC3/VDAC3/UQCRC1/NDUFA6/CYC1/PSMA4/NDUFB6/FUS/COX7B/RAB8A/PARK7/DCTN3/ATP5MC1/VDAC1/EIF2S1/PSMA7/CALM3/UQCRFS1/CYCS/SIGMAR1/UQCRQ/TUBB6/TOMM40/NDUFV1/BAX/ATP5F1B/NDUFB2/NDUFA2/PSMA3/ATP5MC3/ATP5PF/COX8A/UQCR10/PSMD1/PSMB3/NDUFA4/COX7A2	43
hsa05415	Diabetic cardiomyopathy	25/346	203/8577	5.58524744597341e-07	7.08444544462943e-06	6.03392383360009e-06	GAPDH/SLC25A5/PARP1/VDAC3/UQCRC1/NDUFA6/CYC1/PPP1CA/NDUFB6/COX7B/ATP5MC1/VDAC1/UQCRFS1/UQCRQ/MPC2/NDUFV1/ATP5F1B/NDUFB2/NDUFA2/ATP5MC3/ATP5PF/COX8A/UQCR10/NDUFA4/COX7A2	25
hsa05208	Chemical carcinogenesis - reactive oxygen species	23/346	223/8577	3.14328708835171e-05	0.000361150262498476	0.000307596860393985	AKR1C2/SLC25A5/AKR1C3/VDAC3/UQCRC1/NDUFA6/CYC1/NDUFB6/COX7B/ATP5MC1/VDAC1/UQCRFS1/UQCRQ/NDUFV1/ATP5F1B/NDUFB2/NDUFA2/ATP5MC3/ATP5PF/COX8A/UQCR10/NDUFA4/COX7A2	23
hsa04814	Motor proteins	21/346	193/8577	3.1469524948e-05	0.000361150262498476	0.000307596860393985	TUBA1B/TUBB/KIF11/CENPE/KIF20B/KIF23/TUBB4B/TUBA1C/KIF2C/KIF15/KIFC1/KIF20A/KIF18A/KIF22/TPM3/KIF4A/KIF14/TUBG1/ACTB/DCTN3/TUBB6	21
hsa03018	RNA degradation	12/346	79/8577	6.85716544574792e-05	0.00075117130564784	0.00063978337	LSM3/LSM4/EXOSC8/LSM5/HSPD1/ENO1/BTG3/LSM2/EXOSC9/LSM6/LSM8/LSM7	12

						8909495		
hsa03450	Non-homologous end-joining	5/346	13/8577	0.00010219404538489	0.00107081586685907	0.000912029237988723	PRKDC/FEN1/XRCC5/XRCC6/XRCC4	5
hsa04932	Non-alcoholic fatty liver disease	17/346	155/8577	0.00016185750308358	0.00161567354408255	0.00137609233935836	UQCRC1/NDUFA6/CYC1/NDUFB6/COX7B/EIF2S1/UQCRFS1/CYCS/UQCRQ/NDUFV1/BAX/NDUFB2/NDUFA2/COX8A/UQCR10/NDUFA4/COX7A2	17
hsa04115	p53 signaling pathway	11/346	74/8577	0.000167600990050057	0.00161567354408255	0.00137609233935836	CCNB1/CDK1/CCNB2/RRM2/SIVA1/GTSE1/CHEK1/CCNE2/CYCS/BAX/CDK4	11
hsa03082	ATP-dependent chromatin remodeling	14/346	117/8577	0.000245871945054489	0.00227904379838969	0.00194109430306175	RBBP7/POLE3/BRD8/RUVBL2/UCHL5/ACTB/MORF4L2/CDK2AP2/RBBP4/BAZ1B/YEATS4/BCL7C/ACTL6A/MORF4L1	14
hsa04218	Cellular senescence	16/346	156/8577	0.00053699808709083	0.00479320514773666	0.00408244159776654	CCNB1/CDK1/CCNB2/CALM2/CCNA2/SLC25A5/VDAC3/RHEB/PPP1CA/CHEK1/VDAC1/CCNE2/CALM3/EIF4EBP1/CDK4/RBBP4	16
hsa01232	Nucleotide metabolism	11/346	85/8577	0.000575843200801247	0.00495636469261073	0.00422140692316703	TK1/DUT/RRM1/RRM2/DTYMK/TYMS/HPRT1/DCTPP1/NME1/IMPDH2/GMPS	11
hsa05166	Human T-cell leukemia	19/346	222/8577	0.00156824019045245	0.0130326167551394	0.0111000666837832	PTTG1/CDC20/CCNB2/MAD2L1/RANBP1/BUB1B/RAN/BUB3/CCNA2/SLC25A5/FDPS/XPO1/ANAPC11/VDAC3/CHEK1/VDAC1/CCNE2/BAX/CDK4	19

	virus 1 infection							
hsa00240	Pyrimidine metabolism	8/346	58/8577	0.00212426076113985	0.0169614762229248	0.0144463326642076	TK1/DUT/RRM1/RRM2/DTYMK/TYMS/DCTPP1/NME1	8
hsa01200	Carbon metabolism	12/346	115/8577	0.00224971209965096	0.0169614762229248	0.0144463326642076	GAPDH/ACAT2/PHGDH/ENO1/TPI1/PKM/MDH1/GOT1/IDH2/SUCLG1/TKT/PGAM1	12
hsa03050	Proteasome	7/346	46/8577	0.00225214622047134	0.0169614762229248	0.0144463326642076	PSME2/PSMC3/PSMA4/PSMA7/PSMA3/PSMD1/PSMB3	7
hsa00620	Pyruvate metabolism	7/346	47/8577	0.00255662225657643	0.0185333574338678	0.0157851264450938	ACAT2/PKM/LDHB/MDH1/GLO1/ACYP1/LDHA	7
hsa04914	Progesterone-mediated oocyte maturation	11/346	102/8577	0.00261466453423861	0.0185333574338678	0.0157851264450938	CCNB1/CDK1/CCNB2/MAD2L1/AURKA/BUB1/CCNA2/KIF22/PLK1/ANAPC11/CDC25B	11
hsa01230	Biosynthesis of amino acids	9/346	75/8577	0.00305277683099692	0.0210205490362931	0.0179035032193805	GAPDH/PHGDH/ENO1/TPI1/PKM/GOT1/IDH2/TKT/PGAM1	9
hsa00270	Cysteine and methionine metabolism	7/346	52/8577	0.00457920977936581	0.0306552654674211	0.0261095294437524	DNMT1/PHGDH/LDHB/MDH1/GOT1/LDHA/ADI1	7
hsa00100	Steroid biosynthesis	4/346	20/8577	0.00754923391864711	0.047878036168262	0.0407784103638833	LBR/EBP/FDFT1/SQLE	4

hsa00670	One carbon pool by folate	4/346	20/8577	0.00754923391864711	0.047878036168262	0.0407784103638833	DHFR/TYMS/MTHFD1/MTHFD2	4
hsa04260	Cardiac muscle contraction	9/346	87/8577	0.00818107861494687	0.0505548704154409	0.0430583084997203	TPM3/UQCRC1/CYC1/COX7B/UQCRFS1/UQCRQ/COX8A/UQCR10/COX7A2	9
hsa04210	Apoptosis	12/346	136/8577	0.00871767554677809	0.052523995169338	0.044735440305835	TUBA1B/BIRC5/LMNB1/TUBA1C/LMNA/PARP1/ACTB/LMNB2/EIF2S1/CYCS/BAX/PARP2	12
hsa00983	Drug metabolism - other enzymes	8/346	80/8577	0.0149706538759187	0.0879982337584489	0.0749493583004916	TK1/DUT/RRM1/RRM2/HPRT1/NME1/IMPDH2/GMPS	8
hsa00010	Glycolysis / Gluconeogenesis	7/346	67/8577	0.0178333587419291	0.102329510876308	0.0871555126485259	GAPDH/ENO1/TPI1/PKM/LDHB/LDHA/PGAM1	7
hsa01524	Platinum drug resistance	7/346	73/8577	0.0272653609020966	0.152812836683844	0.130152885579164	TOP2A/BIRC5/BRCA1/MSH2/CYCS/BAX/MSH6	7
hsa05017	Spinocerebellar ataxia	11/346	143/8577	0.0296016279464882	0.162136189434174	0.138093718889 12	SLC25A5/PSMC3/VDAC3/NOP56/PSMA4/VDAC1/PSMA7/CYCS/PSMA3/PSMD1/PSMB3	11
hsa05203	Viral carcinogenesis	14/346	204/8577	0.0363277634278492	0.194555355246926	0.165705587565628	CDC20/CDK1/RANBP1/CCNA2/VDAC3/YWHAQ/PKM/CHEK1/CCNE2/BAX/CDK4/HNRNPK/YWHAE/YWHAH	14
hsa03015	mRNA surveillance pathway	8/346	97/8577	0.0416729507958154	0.218330024821554	0.185954814763936	PPP1CA/MAGOHB/FUS/SRRM1/NUDT21/RBM8A/RNPS1/MAGOH	8

hsa03083	Polycomb repressive complex	7/346	84/8577	0.0523476605435736	0.268420982787261	0.22861800237395	CBX3/EZH2/RBBP7/AURKB/PHF19/SUZ12/RBBP4	7
hsa04540	Gap junction	7/346	88/8577	0.0641876802298318	0.322275644487281	0.274486790456518	TUBA1B/CDK1/TUBB/TUBB4B/TUBA1C/GNAS/TUBB6	7
hsa04922	Glucagon signaling pathway	8/346	107/8577	0.0669309022845851	0.329190764297653	0.280376497217918	CALM2/GNAS/PKM/LDHB/CALM3/LDHA/PRMT1/PGAM1	8
hsa00480	Glutathione metabolism	5/346	57/8577	0.078898786991803	0.380292153300491	0.323900283440033	RRM1/RRM2/GGCT/IDH2/TXNDC12	5
hsa00790	Folate biosynthesis	3/346	27/8577	0.0933067554667259	0.440920158185901	0.375538025098277	DHFR/GGH/AKR1C3	3
hsa00020	Citrate cycle (TCA cycle)	3/346	30/8577	0.118791757769287	0.540166294762231	0.460067383614916	MDH1/IDH2/SUCLG1	3
hsa01523	Antifolate resistance	3/346	30/8577	0.118791757769287	0.540166294762231	0.460067383614916	DHFR/GGH/TYMS	3
hsa03008	Ribosome biogenesis in eukaryotes	10/346	164/8577	0.126258265571416	0.563485963013172	0.479929079657429	RAN/XPO1/NOP58/DKC1/NOP56/NHP2/FBL/TCOF1/RPP30/POP7	10
hsa05132	Salmonella infection	14/346	249/8577	0.131045791457488	0.569469544151587	0.485025381566104	TUBA1B/TUBB/TUBB4B/GAPDH/TUBA1C/CSE1L/HSP90B1/PFN1/ACTB/ARPC5/DCTN3/CYCS/TUBB6/BAX	14

hsa00360	Phenylalanine metabolism	2/346	16/8577	0.134385017177491	0.569469544151587	0.485025381566104	MIF/GOT1	2
hsa00640	Propanoate metabolism	3/346	32/8577	0.137050761663038	0.569469544151587	0.485025381566104	LDHB/SUCLG1/LDHA	3
hsa04215	Apoptosis - multiple species	3/346	32/8577	0.137050761663038	0.569469544151587	0.485025381566104	BIRC5/CYCS/BAX	3
hsa00230	Purine metabolism	8/346	128/8577	0.145192946003902	0.59307627096509	0.5051315695051	RRM1/RRM2/PAICS/HPRT1/NME1/IMPDH2/NUDT5/GMPS	8
hsa05230	Central carbon metabolism in cancer	5/346	70/8577	0.151400686166884	0.604105939813246	0.514525696717986	PKM/LDHB/IDH2/LDHA/PGAM1	5
hsa05164	Influenza A	10/346	171/8577	0.152906482691319	0.604105939813246	0.514525696717986	KPNA2/SLC25A5/FDPS/XPO1/ACTB/VDAC1/EIF2S1/CYCS/BAX/CDK4	10
hsa05222	Small cell lung cancer	6/346	92/8577	0.166638209502015	0.647738846612673	0.551688469488845	CKS1B/CKS2/CCNE2/CYCS/BAX/CDK4	6
hsa05130	Pathogenic Escherichia coli infection	11/346	198/8577	0.176329249740897	0.66765326162549	0.568649862489498	TUBA1B/TUBB/TUBB4B/GAPDH/TUBA1C/NCL/ACTB/ARPC5/CYCS/TUBB6/BAX	11
hsa01210	2-Oxocarbox	2/346	19/8577	0.177302110971084	0.66765326162549	0.568649862489498	GOT1/IDH2	2

	ylic acid metabolism							
hsa05216	Thyroid cancer	3/346	37/8577	0.1863080435837	0.690772900056489	0.588341190264317	TPM3/TPR/BAX	3
hsa04978	Mineral absorption	4/346	60/8577	0.2225249088275 74	0.812553076173415	0.692063113578 58	MT2A/MT1X/MT1E/FTH1	4
hsa04216	Ferroptosis	3/346	41/8577	0.228500238171334	0.821918767153605	0.700040006966381	VDAC3/PCBP1/FTH1	3
hsa03010	Ribosome	9/346	167/8577	0.232376770832033	0.823570614272351	0.701446908858303	MRPL27/RPS27L/MRPL13/MRPL22/MRPL18/MRPL11/RPL35/MRPL4/MRPS16	9
hsa00900	Terpenoid backbone biosynthesis	2/346	23/8577	0.237051686677203	0.827963137524724	0.705188083936 76	FDPS/ACAT2	2
hsa00310	Lysine degradation	4/346	63/8577	0.248824877981676	0.84460275483921	0.719360284750583	EZH2/ACAT2/NSD2/HADH	4
hsa03250	Viral life cycle - HIV-1	4/346	63/8577	0.248824877981676	0.84460275483921	0.719360284750583	RAN/XPO1/PSIP1/PPIA	4
hsa05210	Colorectal cancer	5/346	86/8577	0.266110024733261	0.890729388343276	0.758647000336051	BIRC5/MSH2/CYCS/BAX/MSH6	5

hsa04066	HIF-1 signaling pathway	6/346	109/8577	0.276749713718043	0.913653164466415	0.778171509373012	GAPDH/ENO1/LDHB/EIF4EBP1/LDHA/RBX1	6
hsa00062	Fatty acid elongation	2/346	27/8577	0.297672315412655	0.956520373525998	0.814682126392529	HACD3/HADH	2
hsa00650	Butanoate metabolism	2/346	27/8577	0.297672315412655	0.956520373525998	0.814682126392529	ACAT2/HADH	2
hsa05031	Amphetamine addiction	4/346	69/8577	0.303120484087724	0.961211008751861	0.818677207716152	CALM2/GNAS/PPP1CA/CALM3	4
hsa04217	Necroptosis	8/346	159/8577	0.312063986493712	0.976719749934866	0.831886225102856	HMGB1/SLC25A5/PARP1/VDAC3/VDAC1/BAX/FTH1/PPIA	8
hsa04744	Phototransduction	2/346	29/8577	0.327777390811272	0.999999822595679	0.851714197013135	CALM2/CALM3	2
hsa05161	Hepatitis B	8/346	162/8577	0.330222574234114	0.999999822595679	0.851714197013135	BIRC5/PCNA/CCNA2/VDAC3/YWHAQ/CCNE2/CYCS/BAX	8
hsa00630	Glyoxylate and dicarboxylate metabolism	2/346	30/8577	0.342704936028557	0.999999822595679	0.851714197013135	ACAT2/MDH1	2
hsa04120	Ubiquitin mediated proteolysis	7/346	142/8577	0.348799451245969	0.999999822595679	0.851714197013135	UBE2C/CDC20/UBE2S/BRCA1/ANAPC11/SAE1/RBX1	7

hsa05170	Human immunodeficiency virus 1 infection	10/346	212/8577	0.351756965902604	0.999999822595679	0.851714197013135	CCNB1/CDK1/CCNB2/CALM2/CHEK1/CALM3/CYCS/CFL1/BAX/RBX1	10
hsa05214	Glioma	4/346	75/8577	0.358559812113463	0.999999822595679	0.851714197013135	CALM2/CALM3/BAX/CDK4	4
hsa00130	Ubiquinone and other terpenoid-quinone biosynthesis	1/346	11/8577	0.364417482449972	0.999999822595679	0.851714197013135	VKORC1	1
hsa04971	Gastric acid secretion	4/346	76/8577	0.367823582814871	0.999999822595679	0.851714197013135	CALM2/GNAS/ACTB/CALM3	4
hsa03020	RNA polymerase	2/346	34/8577	0.401197806048843	0.999999822595679	0.851714197013135	POLR2H/POLR2E	2
hsa01212	Fatty acid metabolism	3/346	57/8577	0.405655124077673	0.999999822595679	0.851714197013135	ACAT2/HACD3/HADH	3
hsa01240	Biosynthesis of cofactors	7/346	153/8577	0.422058612898102	0.999999822595679	0.851714197013135	DHFR/GGH/MTHFD1/VKORC1/MTHFD2/NME1/MMAB	7
hsa00350	Tyrosine metabolism	2/346	36/8577	0.429528012535517	0.999999822595679	0.851714197013135	MIF/GOT1	2

hsa05160	Hepatitis C	7/346	158/8577	0.455225946862178	0.999999822595679	0.851714197013135	YWHAQ/EIF2S1/CYCS/BAX/CDK4/YWHAE/YWHAH	7
hsa00260	Glycine, serine and threonine metabolism	2/346	40/8577	0.483956638343396	0.999999822595679	0.851714197013135	PHGDH/PGAM1	2
hsa00380	Tryptophan metabolism	2/346	42/8577	0.509943662427417	0.999999822595679	0.851714197013135	ACAT2/HADH	2
hsa04720	Long-term potentiation	3/346	67/8577	0.511256967329069	0.999999822595679	0.851714197013135	CALM2/PPP1CA/CALM3	3
hsa00071	Fatty acid degradation	2/346	43/8577	0.522612606335894	0.999999822595679	0.851714197013135	ACAT2/HADH	2
hsa04924	Renin secretion	3/346	69/8577	0.531202830710306	0.999999822595679	0.851714197013135	CALM2/GNAS/CALM3	3
hsa04530	Tight junction	7/346	170/8577	0.532862103996814	0.999999822595679	0.851714197013135	TUBA1B/PCNA/TUBA1C/RAB8A/ACTB/ARPC5/CDK4	7
hsa05414	Dilated cardiomyopathy	4/346	96/8577	0.545856564913892	0.999999822595679	0.851714197013135	TPM3/LMNA/GNAS/ACTB	4
hsa04723	Retrograde endocannabinoid signaling	6/346	148/8577	0.554119058785922	0.999999822595679	0.851714197013135	NDUFA6/NDUFB6/NDUFV1/NDUFB2/NDUFA2/NDUFA4	6

hsa04750	Inflammatory mediator regulation of TRP channels	4/346	98/8577	0.562310412692263	0.999999822595679	0.851714197013135	CALM2/GNAS/PPP1CA/CALM3	4
hsa05169	Epstein-Barr virus infection	8/346	202/8577	0.572699862673503	0.999999822595679	0.851714197013135	CCNA2/PSMC3/CCNE2/VIM/CYCS/BAX/CDK4/PSMD1	8
hsa04145	Phagosome	6/346	152/8577	0.580474073378168	0.999999822595679	0.851714197013135	TUBA1B/TUBB/TUBB4B/TUBA1C/ACTB/TUBB6	6
hsa00280	Valine, leucine and isoleucine degradation	2/346	48/8577	0.582591672176712	0.999999822595679	0.851714197013135	ACAT2/HADH	2
hsa04918	Thyroid hormone synthesis	3/346	75/8577	0.588182489965855	0.999999822595679	0.851714197013135	HSP90B1/TTF2/GNAS	3
hsa00220	Arginine biosynthesis	1/346	22/8577	0.596274953889001	0.999999822595679	0.851714197013135	GOT1	1
hsa05133	Pertussis	3/346	76/8577	0.59723833546521	0.999999822595679	0.851714197013135	CALM2/CALM3/CFL1	3
hsa05212	Pancreatic cancer	3/346	76/8577	0.59723833546521	0.999999822595679	0.851714197013135	BRCA2/BAX/CDK4	3

hsa 05110	Vibrio cholerae infection	2/346	50/8577	0.604982875307654	0.999999822595679	0.851714197013135	GNAS/ACTB	2
hsa 04390	Hippo signaling pathway	6/346	157/8577	0.612280373712415	0.999999822595679	0.851714197013135	BIRC5/YWHAQ/PPP1CA/ACTB/YWHAE/YWHAH	6
hsa 03060	Protein export	1/346	23/8577	0.612603284216 81	0.999999822595679	0.851714197013135	SRP9	1
hsa 04964	Proximal tubule bicarbonate reclamation	1/346	23/8577	0.61260328421681	0.999999822595679	0.851714197013135	MDH1	1
hsa 04068	FoxO signaling pathway	5/346	131/8577	0.613924991493655	0.999999822595679	0.851714197013135	CCNB1/CCNB2/PLK1/PLK4/PRMT1	5
hsa 04913	Ovarian steroidogenesis	2/346	51/8577	0.615833415183019	0.999999822595679	0.851714197013135	AKR1C3/GNAS	2
hsa 04979	Cholesterol metabolism	2/346	51/8577	0.615833415183019	0.999999822595679	0.851714197013135	VDAC3/VDAC1	2
hsa 05207	Chemical carcinogenesis - receptor activation	8/346	212/8577	0.627710318291631	0.999999822595679	0.851714197013135	BIRC5/KPNA2/HSP90B1/PTGES3/CDC6/KPNB1/GNAS/EIF4EBP1	8

hsa 049 10	Insulin signaling pathway	5/346	137/ 8577	0.65337 348307 1056	0.99999 982259 5679	0.85171 419701 3135	CALM2/RHEB/PPP1CA/CALM3/EIF4EBP1	5
hsa 051 62	Measles	5/346	138/ 8577	0.65969 378314 6622	0.99999 982259 5679	0.85171 419701 3135	EIF2S1/CCNE2/CYCS/BAX/CDK4	5
hsa 054 18	Fluid shear stress and atheroscler osis	5/346	139/ 8577	0.66593 973700 8355	0.99999 982259 5679	0.85171 419701 3135	CALM2/HSP90B1/ACTB/CALM3/SUMO2	5
hsa 051 34	Legionellos is	2/346	56/8 577	0.66666 371785 803	0.99999 982259 5679	0.85171 419701 3135	HSPD1/CYCS	2
hsa 010 40	Biosynthesi s of unsaturated fatty acids	1/346	27/8 577	0.67159 066210 5251	0.99999 982259 5679	0.85171 419701 3135	HACD3	1
hsa 040 22	cGMP- PKG signaling pathway	6/346	167/ 8577	0.67167 422858 2411	0.99999 982259 5679	0.85171 419701 3135	CALM2/SLC25A5/VDAC3/PPP1CA/VDAC1/CALM3	6
hsa 041 41	Protein processing in endoplasmi c reticulum	6/346	170/ 8577	0.68832 031789 3539	0.99999 982259 5679	0.85171 419701 3135	HSP90B1/EIF2S1/PDIA6/BAX/RAD23A/RBX1	6
hsa 051 63	Human cytomegalo	8/346	225/ 8577	0.69309 724782 2618	0.99999 982259 5679	0.85171 419701 3135	CALM2/GNAS/RHEB/CALM3/CYCS/EIF4EBP1/BAX/CDK4	8

	virus infection							
hsa04211	Longevity regulating pathway	3/346	89/8577	0.702807717534323	0.999999822595679	0.851714197013135	RHEB/EIF4EBP1/BAX	3
hsa05416	Viral myocarditis	2/346	60/8577	0.703314530877446	0.999999822595679	0.851714197013135	ACTB/CYCS	2
hsa00030	Pentose phosphate pathway	1/346	30/8577	0.709873312092682	0.999999822595679	0.851714197013135	TKT	1
hsa05410	Hypertrophic cardiomyopathy	3/346	90/8577	0.709984084066461	0.999999822595679	0.851714197013135	TPM3/LMNA/ACTB	3
hsa00590	Arachidonic acid metabolism	2/346	61/8577	0.711937912715317	0.999999822595679	0.851714197013135	AKR1C3/PTGES3	2
hsa04722	Neurotrophin signaling pathway	4/346	119/8577	0.713359337711064	0.999999822595679	0.851714197013135	CALM2/CALM3/BAX/YWHAE	4
hsa00140	Steroid hormone biosynthesis	2/346	62/8577	0.720350874315293	0.999999822595679	0.851714197013135	AKR1C2/AKR1C3	2
hsa04152	AMPK signaling pathway	4/346	121/8577	0.725540139395491	0.999999822595679	0.851714197013135	CCNA2/RHEB/RAB8A/EIF4EBP1	4

hsa04912	GnRH signaling pathway	3/346	93/8577	0.730715030494088	0.999999822595679	0.851714197013135	CALM2/GNAS/CALM3	3
hsa04970	Salivary secretion	3/346	93/8577	0.730715030494088	0.999999822595679	0.851714197013135	CALM2/GNAS/CALM3	3
hsa04261	Adrenergic signaling in cardiomyocytes	5/346	154/8577	0.750507284933092	0.999999822595679	0.851714197013135	CALM2/TPM3/GNAS/PPP1CA/CALM3	5
hsa04921	Oxytocin signaling pathway	5/346	154/8577	0.750507284933092	0.999999822595679	0.851714197013135	CALM2/GNAS/PPP1CA/ACTB/CALM3	5
hsa00051	Fructose and mannose metabolism	1/346	34/8577	0.754083739805051	0.999999822595679	0.851714197013135	TPI1	1
hsa04710	Circadian rhythm	1/346	34/8577	0.754083739805051	0.999999822595679	0.851714197013135	RBX1	1
hsa04713	Circadian entrainment	3/346	97/8577	0.756526576667716	0.999999822595679	0.851714197013135	CALM2/GNAS/CALM3	3
hsa05221	Acute myeloid leukemia	2/346	67/8577	0.759364028735954	0.999999822595679	0.851714197013135	CCNA2/EIF4EBP1	2
hsa01522	Endocrine resistance	3/346	98/8577	0.762659718687993	0.999999822595679	0.851714197013135	GNAS/BAX/CDK4	3

hsa04925	Aldosterone synthesis and secretion	3/346	98/8577	0.762659718687993	0.999999822595679	0.851714197013135	CALM2/GNAS/CALM3	3
hsa05417	Lipid and atherosclerosis	7/346	215/8577	0.770785218653188	0.999999822595679	0.851714197013135	CALM2/HSPD1/HSP90B1/EIF2S1/CALM3/CYCS/BAX	7
hsa00040	Pentose and glucuronate interconversions	1/346	36/8577	0.773601181035304	0.999999822595679	0.851714197013135	DCXR	1
hsa04916	Melanogenesis	3/346	101/8577	0.780311407946141	0.999999822595679	0.851714197013135	CALM2/GNAS/CALM3	3
hsa00250	Alanine, aspartate and glutamate metabolism	1/346	37/8577	0.782772705606406	0.999999822595679	0.851714197013135	GOT1	1
hsa04728	Dopaminergic synapse	4/346	132/8577	0.785693479159887	0.999999822595679	0.851714197013135	CALM2/GNAS/PPP1CA/CALM3	4
hsa05131	Shigellosis	8/346	247/8577	0.785992953191399	0.999999822595679	0.851714197013135	CBX3/PFN1/ACTB/ARPC5/VDAC1/CYCS/BAX/RBX1	8
hsa04613	Neutrophil extracellula	6/346	191/8577	0.788970676306514	0.999999822595679	0.851714197013135	HMGB1/SLC25A5/VDAC3/HAT1/ACTB/VDAC1	6

	r trap formation							
hsa 05218	Melanoma	2/346	72/8577	0.793587213294798	0.999999822595679	0.851714197013135	BAX/CDK4	2
hsa 05223	Non-small cell lung cancer	2/346	72/8577	0.793587213294798	0.999999822595679	0.851714197013135	BAX/CDK4	2
hsa 04270	Vascular smooth muscle contraction	4/346	134/8577	0.795422437144678	0.999999822595679	0.851714197013135	CALM2/GNAS/PPP1CA/CALM3	4
hsa 05202	Transcriptional misregulation in cancer	6/346	193/8577	0.797115874375294	0.999999822595679	0.851714197013135	CCNA2/NSD2/TAF15/EWSR1/FUS/BAX	6
hsa 04915	Estrogen signaling pathway	4/346	137/8577	0.809348139906672	0.999999822595679	0.851714197013135	CALM2/HSP90B1/GNAS/CALM3	4
hsa 05206	MicroRNAs in cancer	10/346	310/8577	0.809384370775139	0.999999822595679	0.851714197013135	STMN1/KIF23/DNMT1/EZH2/BRCA1/CDCA5/CCNE2/VIM/HNRNPK/CDC25B	10
hsa 04623	Cytosolic DNA-sensing pathway	2/346	75/8577	0.811998764487717	0.999999822595679	0.851714197013135	POLR2H/POLR2E	2

hsa05219	Bladder cancer	1/346	41/8577	0.815899969302017	0.999999822595679	0.851714197013135	CDK4	1
hsa05220	Chronic myeloid leukemia	2/346	76/8577	0.817803100407245	0.999999822595679	0.851714197013135	BAX/CDK4	2
hsa04810	Regulation of actin cytoskeleton	7/346	229/8577	0.823257180655032	0.999999822595679	0.851714197013135	DIAPH3/PFN1/PPP1CA/ACTB/ARPC5/ITGAE/CFL1	7
hsa05100	Bacterial invasion of epithelial cells	2/346	77/8577	0.823446818519254	0.999999822595679	0.851714197013135	ACTB/ARPC5	2
hsa05412	Arrhythmogenic right ventricular cardiomyopathy	2/346	77/8577	0.823446818519254	0.999999822595679	0.851714197013135	LMNA/ACTB	2
hsa04140	Autophagy - animal	4/346	141/8577	0.826706340175333	0.999999822595679	0.851714197013135	HMGB1/RHEB/RAB8A/EIF2S1	4
hsa00860	Porphyrin metabolism	1/346	43/8577	0.830523025676709	0.999999822595679	0.851714197013135	MMAB	1
hsa04940	Type I diabetes mellitus	1/346	43/8577	0.830523025676709	0.999999822595679	0.851714197013135	HSPD1	1

hsa04975	Fat digestion and absorption	1/346	43/8577	0.830523025676709	0.999999822595679	0.851714197013135	ACAT2	1
hsa01521	EGFR tyrosine kinase inhibitor resistance	2/346	79/8577	0.834266253153779	0.999999822595679	0.851714197013135	EIF4EBP1/BAX	2
hsa04962	Vasopressin-regulated water reabsorption	1/346	44/8577	0.83739425055553	0.999999822595679	0.851714197013135	GNAS	1
hsa03022	Basal transcription factors	1/346	45/8577	0.843987663107714	0.999999822595679	0.851714197013135	TAF15	1
hsa05224	Breast cancer	4/346	147/8577	0.850276781395684	0.999999822595679	0.851714197013135	BRCA2/BRCA1/BAX/CDK4	4
hsa04930	Type II diabetes mellitus	1/346	46/8577	0.850314464392844	0.999999822595679	0.851714197013135	PKM	1
hsa04146	Peroxisome	2/346	83/8577	0.854128870920335	0.999999822595679	0.851714197013135	PRDX1/IDH2	2
hsa05152	Tuberculosis	5/346	180/8577	0.857723589425 56	0.999999822595679	0.851714197013135	CALM2/HSPD1/CALM3/CYCS/BAX	5

hsa05030	Cocaine addiction	1/346	49/8577	0.867800563559519	0.999999822595679	0.851714197013135	GNAS	1
hsa00330	Arginine and proline metabolism	1/346	50/8577	0.873164189850374	0.999999822595679	0.851714197013135	GOT1	1
hsa00565	Ether lipid metabolism	1/346	50/8577	0.873164189850374	0.999999822595679	0.851714197013135	PAFAH1B3	1
hsa05034	Alcoholism	5/346	188/8577	0.881789740445715	0.999999822595679	0.851714197013135	CALM2/GNAS/PPP1CA/HAT1/CALM3	5
hsa04611	Platelet activation	3/346	124/8577	0.882473247233412	0.999999822595679	0.851714197013135	GNAS/PPP1CA/ACTB	3
hsa04961	Endocrine and other factor-regulated calcium reabsorption	1/346	53/8577	0.887987674741928	0.999999822595679	0.851714197013135	GNAS	1
hsa05167	Kaposi sarcoma-associated herpesvirus infection	5/346	194/8577	0.897485499902384	0.999999822595679	0.851714197013135	CALM2/CALM3/CYCS/BAX/CDK4	5

hsa 04657	IL-17 signaling pathway	2/346	94/8577	0.89806723652241	0.999999822595679	0.851714197013135	HSP90B1/SRSF1	2
hsa 04070	Phosphatidylinositol signaling system	2/346	97/8577	0.907715659028044	0.999999822595679	0.851714197013135	CALM2/CALM3	2
hsa 04666	Fc gamma R-mediated phagocytosis	2/346	97/8577	0.907715659028044	0.999999822595679	0.851714197013135	ARPC5/CFL1	2
hsa 05215	Prostate cancer	2/346	97/8577	0.907715659028044	0.999999822595679	0.851714197013135	HSP90B1/CCNE2	2
hsa 04923	Regulation of lipolysis in adipocytes	1/346	58/8577	0.908953642657209	0.999999822595679	0.851714197013135	GNAS	1
hsa 05213	Endometrial cancer	1/346	58/8577	0.908953642657209	0.999999822595679	0.851714197013135	BAX	1
hsa 05231	Choline metabolism in cancer	2/346	98/8577	0.910737657785128	0.999999822595679	0.851714197013135	RHEB/EIF4EBP1	2
hsa 05225	Hepatocellular carcinoma	4/346	168/8577	0.912531228436076	0.999999822595679	0.851714197013135	ACTB/BAX/CDK4/ACTL6A	4

hsa04730	Long-term depression	1/346	60/8577	0.91619958337932	0.999999822595679	0.851714197013135	GNAS	1
hsa04933	AGE-RAGE signaling pathway in diabetic complications	2/346	100/8577	0.916506046997291	0.999999822595679	0.851714197013135	BAX/CDK4	2
hsa05322	Systemic lupus erythematosus	3/346	137/8577	0.919272231204251	0.999999822595679	0.851714197013135	SNRPB/SNRPD1/SNRPD3	3
hsa04972	Pancreatic secretion	2/346	102/8577	0.921923591754464	0.999999822595679	0.851714197013135	GNAS/RAB8A	2
hsa05146	Amoebiasis	2/346	102/8577	0.921923591754464	0.999999822595679	0.851714197013135	GNAS/PRDX1	2
hsa05165	Human papillomavirus infection	9/346	331/8577	0.92367557013228	0.999999822595679	0.851714197013135	CCNA2/GNAS/TUBG1/RHEB/PKM/CCNE2/EIF4EBP1/BAX/CDK4	9
hsa05217	Basal cell carcinoma	1/346	63/8577	0.926004486468596	0.999999822595679	0.851714197013135	BAX	1

hsa04625	C-type lectin receptor signaling pathway	2/346	104/8577	0.927009514129339	0.999999822595679	0.851714197013135	CALM2/CALM3	2
hsa04015	Rap1 signaling pathway	5/346	210/8577	0.930831640925303	0.999999822595679	0.851714197013135	CALM2/PFN1/GNAS/ACTB/CALM3	5
hsa04927	Cortisol synthesis and secretion	1/346	65/8577	0.931896819574875	0.999999822595679	0.851714197013135	GNAS	1
hsa04350	TGF-beta signaling pathway	2/346	108/8577	0.936259211281012	0.999999822595679	0.851714197013135	ID3/RBX1	2
hsa05204	Chemical carcinogenesis - DNA adducts	1/346	69/8577	0.942314659302535	0.999999822595679	0.851714197013135	AKR1C2	1
hsa05211	Renal cell carcinoma	1/346	69/8577	0.942314659302535	0.999999822595679	0.851714197013135	RBX1	1
hsa04020	Calcium signaling pathway	6/346	253/8577	0.946440794254	0.999999822595679	0.851714197013135	CALM2/SLC25A5/VDAC3/GNAS/VDAC1/CALM3	6
hsa00562	Inositol phosphate metabolism	1/346	73/8577	0.951142761997985	0.999999822595679	0.851714197013135	TPI1	1

hsa04934	Cushing syndrome	3/346	155/8577	0.953020917192801	0.999999822595679	0.851714197013135	GNAS/CCNE2/CDK4	3
hsa04151	PI3K-Akt signaling pathway	9/346	359/8577	0.957615396668657	0.999999822595679	0.851714197013135	BRCA1/HSP90B1/YWHAQ/RHEB/CCNE2/EIF4EBP1/CDK4/YWHAE/YWHAH	9
hsa04919	Thyroid hormone signaling pathway	2/346	121/8577	0.959236288713685	0.999999822595679	0.851714197013135	RHEB/ACTB	2
hsa04612	Antigen processing and presentation	1/346	78/8577	0.960307405121706	0.999999822595679	0.851714197013135	PSME2	1
hsa04012	ErbB signaling pathway	1/346	85/8577	0.970330432245686	0.999999822595679	0.851714197013135	EIF4EBP1	1
hsa04911	Insulin secretion	1/346	86/8577	0.971539295934216	0.999999822595679	0.851714197013135	GNAS	1
hsa04512	ECM-receptor interaction	1/346	89/8577	0.974879061328691	0.999999822595679	0.851714197013135	HMMR	1
hsa04976	Bile secretion	1/346	89/8577	0.974879061328691	0.999999822595679	0.851714197013135	GNAS	1

hsa05135	Yersinia infection	2/346	137/8577	0.976769347950794	0.999999822595679	0.851714197013135	ACTB/ARPC5	2
hsa05032	Morphine addiction	1/346	91/8577	0.976885465963111	0.999999822595679	0.851714197013135	GNAS	1
hsa04371	Apelin signaling pathway	2/346	139/8577	0.978364130321241	0.999999822595679	0.851714197013135	CALM2/CALM3	2
hsa04520	Adherens junction	1/346	93/8577	0.978732045513166	0.999999822595679	0.851714197013135	ACTB	1
hsa04550	Signaling pathways regulating pluripotency of stem cells	2/346	143/8577	0.981242324469188	0.999999822595679	0.851714197013135	RIF1/ID3	2
hsa04621	NOD-like receptor signaling pathway	3/346	186/8577	0.982363084316402	0.999999822595679	0.851714197013135	VDAC3/VDAC1/YWHAE	3
hsa04024	cAMP signaling pathway	4/346	225/8577	0.982677641684644	0.999999822595679	0.851714197013135	CALM2/GNAS/PPP1CA/CALM3	4
hsa04072	Phospholipase D signaling pathway	2/346	148/8577	0.984322177430549	0.999999822595679	0.851714197013135	GNAS/RHEB	2

hsa 05226	Gastric cancer	2/346	149/8577	0.984876382508325	0.999999822595679	0.851714197013135	CCNE2/BAX	2
hsa 05142	Chagas disease	1/346	102/8577	0.985381576181746	0.999999822595679	0.851714197013135	GNAS	1
hsa 04064	NF-kappa B signaling pathway	1/346	104/8577	0.986550900681068	0.999999822595679	0.851714197013135	PARP1	1
hsa 04928	Parathyroid hormone synthesis, secretion and action	1/346	106/8577	0.987626939826741	0.999999822595679	0.851714197013135	GNAS	1
hsa 04150	mTOR signaling pathway	2/346	156/8577	0.988256816935913	0.999999822595679	0.851714197013135	RHEB/EIF4EBP1	2
hsa 04931	Insulin resistance	1/346	108/8577	0.988617115810405	0.999999822595679	0.851714197013135	PPP1CA	1
hsa 05145	Toxoplasmosis	1/346	111/8577	0.989956184548992	0.999999822595679	0.851714197013135	CYCS	1
hsa 04670	Leukocyte transendothelial migration	1/346	115/8577	0.991500435661955	0.999999822595679	0.851714197013135	ACTB	1

hsa04724	Glutamatergic synapse	1/346	115/8577	0.991500435661955	0.999999822595679	0.851714197013135	GNAS	1
hsa04726	Serotonergic synapse	1/346	115/8577	0.991500435661955	0.999999822595679	0.851714197013135	GNAS	1
hsa04144	Endocytosis	4/346	250/8577	0.991955891276053	0.999999822595679	0.851714197013135	VPS29/RAB8A/ARPC5/SNX5	4
hsa04935	Growth hormone synthesis, secretion and action	1/346	120/8577	0.993102052817329	0.999999822595679	0.851714197013135	GNAS	1
hsa04310	Wnt signaling pathway	2/346	171/8577	0.993210593365757	0.999999822595679	0.851714197013135	CACYBP/RBX1	2
hsa04071	Sphingolipid signaling pathway	1/346	121/8577	0.993384267518192	0.999999822595679	0.851714197013135	BAX	1
hsa04660	T cell receptor signaling pathway	1/346	121/8577	0.993384267518192	0.999999822595679	0.851714197013135	CDK4	1
hsa04926	Relaxin signaling pathway	1/346	129/8577	0.995264524596963	0.999999822595679	0.851714197013135	GNAS	1

hsa04936	Alcoholic liver disease	1/346	142/8577	0.997251533116153	0.999999822595679	0.851714197013135	TRA2B	1
hsa04510	Focal adhesion	2/346	203/8577	0.997937697550384	0.999999822595679	0.851714197013135	PPP1CA/ACTB	2
hsa05205	Proteoglycans in cancer	2/346	205/8577	0.998087422321287	0.999999822595679	0.851714197013135	PPP1CA/ACTB	2
hsa05171	Coronavirus disease - COVID-19	2/346	232/8577	0.999314801647019	0.999999822595679	0.851714197013135	RPS27L/RPL35	2
hsa04014	Ras signaling pathway	2/346	236/8577	0.999412247560605	0.999999822595679	0.851714197013135	CALM2/CALM3	2
hsa04360	Axon guidance	1/346	182/8577	0.999487425329659	0.999999822595679	0.851714197013135	CFL1	1
hsa04010	MAPK signaling pathway	2/346	301/8577	0.999953333353802	0.999999822595679	0.851714197013135	STMN1/CDC25B	2
hsa04080	Neuroactive ligand-receptor interaction	3/346	367/8577	0.999971405496546	0.999999822595679	0.851714197013135	NTS/NMU/TMEM97	3
hsa04740	Olfactory transduction	2/346	439/8577	0.999999822595679	0.999999822595679	0.851714197013135	CALM2/CALM3	2

2
