# Supplementary material for: Multiomics Reveals IL-17 Drives Epithelial Keratinization and Proliferation via EHF in Odontogenic Keratocysts
Source: Int J Mol Sci. 2026 May 4;27(9):4115. doi: 10.3390/ijms27094115 (PMC13163638; doi:10.3390/ijms27094115)
Supplement: Supplementary file 1 [file ijms-27-04115-s001.zip › ijms-4235677-supplementary/Supplementary Table S11.pdf]

Supplementary Table S11. EpC4 KEGG enrichment.

ID	Description	GeneRatio	BgRatio	pvalue	p.adjust	qvalue	geneID	Count
hsa00190	Oxidative phosphorylation	51/316	134/8577	3.06713114450156e-39	8.8333376961645e-37	7.68397065675128e-37	COX6C/CYCS/ATP5F1E/COX5A/COX7B/COX6A1/UQCRQ/COX5B/UQCR11/ATP5PD/NDUFB2/UQCR10/ATP5MF/ATP5ME/COX8A/ATP5PB/NDUFC2/ATP5MC1/COX7A2/NDUFA4/NDUFB9/COX6B1/NDUFS6/UQCRB/ATP5MC3/NDUFAB1/ATP5PF/NDUFC1/NDUFB8/ATP6V0E1/NDUFB3/ATP5F1B/SDHB/ATP5F1C/UQCRC2/NDUFA12/CYC1/NDUFA3/SDHC/COX7C/ATP5PO/NDUFB1/NDUFA13/COX4I1/NDUFA1/ATP6V0B/COX17/ATP5MG/NDUFS8/NDUFS5/ATP6V0D1	51
hsa05012	Parkinson disease	67/316	266/8577	1.49290589749432e-38	1.7187010350055e-36	1.49506888278987e-36	CALML3/TXN/CALML5/TUBA4A/COX6C/NFE2L2/CYCS/SLC25A5/ATP5F1E/PSMA7/COX5A/COX7B/COX6A1/UQCRQ/COX5B/UQCR11/ATP5PD/NDUFB2/UQCR10/COX8A/ATP5PB/NDUFC2/ATP5MC1/COX7A2/NDUFA4/NDUFB9/COX6B1/NDUFS6/PSMB6/TUBA1A/UQCRB/VDAC1/ATP5MC3/CALM1/NDUFAB1/ATP5PF/NDUFC1/PSMD8/NDUFB8/NDUFB3/ATP5F1B/SDHB/ATP5F1C/UQCRC2/PSMA3/NDUFA12/VDAC2/CYC1/NDUFA3/SDHC/COX7C/PSMD11/PSMD7/ATP5PO/NDUFB1/NDUFA13/PSMB1/PSMB3/COX4I1/NDUFA1/PSMD1/PSMA1/SEM1/TUBA1C/NDUFS8/NDUFS5/PSMB5	67
hsa05208	Chemical carcinogenesis - reactive oxygen species	62/316	223/8577	2.19519037635504e-38	1.7187010350055e-36	1.49506888278987e-36	GSTA1/GSTM3/AKR1C2/AKR1C3/NQO1/COX6C/MGST2/NFE2L2/SLC25A5/ATP5F1E/COX5A/COX7B/COX6A1/UQCRQ/COX5B/UQCR11/ATP5PD/GSTM4/NDUFB2/UQCR10/COX8A/ATP5PB/NDUFC2/ATP5MC1/COX7A2/NDUFA4/NDUFB9/GSTO1/COX6B1/NDUFS6/AKR1C1/HIF1A/UQCRB/VDAC1/ATP5MC3/MAPK13/EPHX1/NDUFAB1/ATP5PF/NDUFC1/NDUFB8/GSTA4/NDUFB3/ATP5F1B/SDHB/ATP5F1C/UQCRC2/NDUFA12/VDAC2/CYC1/NDUFA3/SDHC/COX7C/ATP5PO/NDUFB1/NDUFA13/COX4I1/NDUFA1/PLD1/NDUFS8/NDUFS5/CBR1	62
hsa05016	Huntington disease	71/316	306/8577	2.38708477084097e-38	1.7187010350055e-36	1.49506888278987e-36	GPX2/POLR2J3/TUBA4A/COX6C/CYCS/SLC25A5/ATP5F1E/PSMA7/COX5A/COX7B/COX6A1/UQCRQ/COX5B/UQCR11/ATP5PD/NDUFB2/UQCR10/CLTB/COX8A/ATP5PB/NDUFC2/ATP5MC1/COX7A2/NDUFA4/NDUFB9/COX6B1/NDUFS6/PSMB6/TUBA1A/UQCRB/VDAC1/ATP5MC3/GPX3/NDUFAB1/ATP5PF/AP2S1/NDUFC1/PSMD8/NDUFB8/NDUFB3/ATP5F1B/SDHB/ATP5F1C/POLR2L/UQCRC2/PSMA3/NDUFA12/VDAC2/CYC1/NDUFA3/SDHC/CO	71

							X7C/PSMD11/PSMD7/ATP5PO/NDUFB1/NDUFA13/PSMB1/PSMB3/COX4I1/NDUFA1/PSMD1/HDAC1/PSMA1/POLR2E/SEM1/TUBA1C/POLR2I/NDUFS8/NDUFS5/PSMB5	
hsa05020	Prion disease	63/316	272/8577	7.31326682432697e-34	4.21244169081234e-32	3.66433158776804e-32	TUBA4A/COX6C/CYCS/SLC25A5/ATP5F1E/PSMA7/COX5A/COX7B/COX6A1/UQCRQ/COX5B/UQCR11/ATP5PD/NDUFB2/UQCR10/COX8A/ATP5PB/NDUFC2/ATP5MC1/COX7A2/NDUFA4/NDUFB9/COX6B1/NDUFS6/PSMB6/TUBA1A/UQCRB/VDAC1/ATP5MC3/MAPK13/NDUFAB1/ATP5PF/NDUFC1/PSMD8/NDUFB8/NDUFB3/ATP5F1B/SDHB/ATP5F1C/UQCRC2/PSMA3/NDUFA12/VDAC2/CYC1/NDUFA3/SDHC/COX7C/PSMD11/PSMD7/ATP5PO/NDUFB1/NDUFA13/PSMB1/PSMB3/COX4I1/NDUFA1/PSMD1/PSMA1/SEM1/TUBA1C/NDUFS8/NDUFS5/PSMB5	63
hsa05014	Amyotrophic lateral sclerosis	67/316	364/8577	1.4481865467381e-29	6.95129542434287e-28	6.0468140021696e-28	GPX2/TUBA4A/COX6C/CYCS/ATP5F1E/PSMA7/COX5A/COX7B/COX6A1/UQCRQ/COX5B/UQCR11/ATP5PD/NDUFB2/UQCR10/COX8A/ATP5PB/NDUFC2/ATP5MC1/COX7A2/NDUFA4/NDUFB9/COX6B1/NDUFS6/PSMB6/TUBA1A/UQCRB/PFN1/VDAC1/ATP5MC3/MAPK13/ANXA7/GPX3/NDUFAB1/ATP5PF/NDUFC1/PSMD8/NDUFB8/ACTB/NDUFB3/ATP5F1B/SDHB/ATP5F1C/CASP1/UQCRC2/PSMA3/NDUFA12/CYC1/NDUFA3/SDHC/COX7C/PSMD11/PSMD7/ATP5PO/NDUFB1/NDUFA13/PSMB1/PSMB3/COX4I1/NDUFA1/PSMD1/PSMA1/SEM1/TUBA1C/NDUFS8/NDUFS5/PSMB5	67
hsa04714	Thermogenesis	52/316	232/8577	3.64453774363105e-27	1.49946695737963e-25	1.30436087666796e-25	MGLL/COA3/COX6C/ATP5F1E/COX5A/COX7B/COX6A1/UQCRQ/COX5B/UQCR11/ATP5PD/NDUFB2/UQCR10/ATP5MF/ATP5ME/COX8A/ATP5PB/NDUFC2/ATP5MC1/COX7A2/NDUFA4/NDUFB9/COX6B1/NDUFS6/UQCRB/ATP5MC3/MAPK13/ACSL1/NDUFAB1/ATP5PF/NDUFC1/NDUFB8/ACTB/NDUFB3/ATP5F1B/SDHB/ATP5F1C/UQCRC2/NDUFA12/CYC1/NDUFA3/SDHC/COX7C/ATP5PO/NDUFB1/NDUFA13/COX4I1/NDUFA1/COX17/ATP5MG/NDUFS8/NDUFS5	52
hsa05010	Alzheimer disease	65/316	384/8577	1.80173785232362e-26	6.48625626836503e-25	5.64228432701344e-25	CALML3/CALML5/TUBA4A/COX6C/CYCS/SLC25A5/ATP5F1E/PSMA7/COX5A/COX7B/COX6A1/UQCRQ/COX5B/UQCR11/ATP5PD/NDUFB2/UQCR10/COX8A/ATP5PB/NDUFC2/ATP5MC1/COX7A2/NDUFA4/NDUFB9/COX6B1/NDUFS6/PSMB6/TUBA1A/UQCRB/VDAC1/ATP5MC3/CALM1/NDUFAB1/ATP5PF/NDUFC1/PSMD8/NDUFB8/NDUFB3/ATP5F1B/SDHB/ATP5F1C/UQCRC2/PSMA3/NDUFA12/VDAC2/CYC1/NDUFA3/SDHC/COX7C/PSMD11/PSMD7/ATP5PO/NDUFB1/NDUFA13/PSMB1/PSMB3/COX4I1/NDUFA1/PSMD1/PSMA1/SEM1/TUBA1C/NDUFS8/NDUFS5/PSMB5	65

hsa05415	Diabetic cardiomyopathy	47/316	203/8577	3.11840616682601e-25	9.97889973384323e-24	8.68047564566772e-24	COX6C/SLC25A5/ATP5F1E/COX5A/COX7B/COX6A1/UQCRQ/COX5B/UQCR11/ATP5PD/NDUFB2/UQCR10/COX8A/ATP5PB/NDUFC2/ATP5MC1/COX7A2/NDUFA4/NDUFB9/COX6B1/NDUFS6/UQCRB/VDAC1/ATP5MC3/MAPK13/NDUFAB1/ATP5PF/NDUFC1/NDUFB8/NDUFB3/ATP5F1B/SDHB/ATP5F1C/UQCRC2/NDUFA12/VDAC2/CYC1/NDUFA3/SDHC/COX7C/ATP5PO/NDUFB1/NDUFA13/COX4I1/NDUFA1/NDUFS8/NDUFS5	47
hsa05022	Pathways of neurodegeneration - multiple diseases	68/316	476/8577	2.89540222838934e-23	8.33875841776129e-22	7.25374453007012e-22	CALML3/GPX2/CALML5/TUBA4A/COX6C/CYCS/SLC25A5/ATP5F1E/PSMA7/COX5A/COX7B/COX6A1/UQCRQ/COX5B/UQCR11/ATP5PD/NDUFB2/UQCR10/COX8A/ATP5PB/NDUFC2/ATP5MC1/COX7A2/NDUFA4/NDUFB9/COX6B1/NDUFS6/PSMB6/TUBA1A/UQCRB/VDAC1/ATP5MC3/MAPK13/CALM1/GPX3/NDUFAB1/ATP5PF/NDUFC1/PSMD8/NDUFB8/NDUFB3/ATP5F1B/SDHB/ATP5F1C/UQCRC2/PSMA3/NDUFA12/VDAC2/CYC1/NDUFA3/SDHC/COX7C/PSMD11/PSMD7/ATP5PO/NDUFB1/NDUFA13/PSMB1/PSMB3/COX4I1/NDUFA1/PSMD1/PSMA1/SEM1/TUBA1C/NDUFS8/NDUFS5/PSMB5	68
hsa04932	Non-alcoholic fatty liver disease	37/316	155/8577	1.74327238511298e-20	4.56420406284125e-19	3.9703237096353e-19	COX6C/CYCS/COX5A/COX7B/COX6A1/UQCRQ/COX5B/UQCR11/NDUFB2/UQCR10/COX8A/NDUFC2/COX7A2/NDUFA4/NDUFB9/COX6B1/NDUFS6/UQCRB/MAPK13/NDUFAB1/NDUFC1/CDC42/NDUFB8/NDUFB3/SDHB/UQCRC2/NDUFA12/CYC1/NDUFA3/SDHC/COX7C/NDUFB1/NDUFA13/COX4I1/NDUFA1/NDUFS8/NDUFS5	37
hsa03050	Proteasome	13/316	46/8577	6.12780702215986e-09	1.47067368531837e-07	1.27931409760881e-07	PSMA7/POMP/PSMB6/PSMD8/PSMA3/PSMD11/PSMD7/PSMB1/PSMB3/PSMD1/PSMA1/SEM1/PSMB5	13
hsa00480	Glutathione metabolism	14/316	57/8577	1.17077622486004e-08	2.59371963661302e-07	2.2562327248315e-07	GSTA1/GSTM3/GPX2/GSTP1/PRDX6/ODC1/MGST2/PGD/GSTM4/IDH1/GSTO1/GGCT/GPX3/GSTA4	14
hsa04260	Cardiac muscle contraction	17/316	87/8577	1.31618120367187e-08	2.70757276183927e-07	2.35527162762334e-07	COX6C/ATP1B1/COX5A/COX7B/COX6A1/UQCRQ/COX5B/UQCR11/UQCR10/COX8A/COX7A2/COX6B1/UQCRB/UQCRC2/CYC1/COX7C/COX4I1	17
hsa00980	Metabolism of xenobiotics by cytochrome P450	14/316	78/8577	7.73067454816048e-07	1.48428951324681e-05	1.29115827541207e-05	GSTA1/GSTM3/ADH7/ALDH3A1/GSTP1/MGST2/GSTM4/GSTO1/AKR1C1/EPHX1/GSTA4/UGT1A7/ALDH3B2/CBR1	14

hsa05132	Salmonella infection	26/316	249/8577	1.42821609610704e-06	2.57078897299268e-05	2.23628572943077e-05	DYNLT3/TXN/TUBA4A/DYNLL1/DYNLT1/ANXA2/CYCS/S100A10/ARPC2/RALA/PAK1/ARL8B/ARPC3/TUBA1A/PFN1/MAPK13/PYCARD/ACTR3/CDC42/ACTB/BRK1/CASP1/TUBA1C/ARF1/MYL12B/ABI1	26
hsa04723	Retrograde endocannabinoid signaling	19/316	148/8577	1.88689979974046e-06	3.19663024897207e-05	2.78069444172278e-05	MGLL/NDUFB2/NDUFC2/NDUFA4/NDUFB9/NDUFS6/MAPK13/NDUFAB1/NDUFC1/NDUFB8/GNG5/NDUFB3/NDUFA12/NDUFA3/NDUFB1/NDUFA13/NDUFA1/NDUFS8/NDUFS5	19
hsa00100	Steroid biosynthesis	7/316	20/8577	4.41933379489793e-06	7.07093407183669e-05	6.15088563266495e-05	FDFT1/MSMO1/SQLE/TM7SF2/DHCR24/DHCR7/SC5D	7
hsa05204	Chemical carcinogenesis - DNA adducts	12/316	69/8577	6.77589158109195e-06	0.000102708251334446	8.93441659999935e-05	GSTA1/GSTM3/AKR1C2/GSTP1/MGST2/GSTM4/GSTO1/CYP2C18/EPHX1/GSTA4/UGT1A7/CBR1	12
hsa05418	Fluid shear stress and atherosclerosis	17/316	139/8577	1.27651476625198e-05	0.000183818126340285	0.000159900270719984	GSTA1/GSTM3/CALML3/TXN/GSTP1/CALML5/NQO1/MGST2/NFE2L2/GSTM4/SDC1/GSTO1/MAPK13/CALM1/ACTB/GSTA4/SDC4	17
hsa01200	Carbon metabolism	15/316	115/8577	1.91162406660766e-05	0.000262165586277621	0.000228053397419861	TALDO1/PGD/ME1/IDH1/TPI1/SUCLG1/HK1/ENO1/SDHB/MDH2/SDHC/PCCB/PGK1/GPI/PGAM1	15
hsa05130	Pathogenic Escherichia coli infection	20/316	198/8577	3.91392473510825e-05	0.000512368328959625	0.000445700520074528	CLDN4/CLDN7/TUBA4A/CYCS/SLC9A3R1/ARPC2/PAK1/ARPC3/TUBA1A/MAPK13/PYCARD/TJP1/ACTR3/CDC42/ACTB/BRK1/CASP1/TUBA1C/ARF1/ABI1	20
hsa00982	Drug metabolism - cytochrome P450	11/316	72/8577	5.77521102337241e-05	0.000723156858578806	0.000629061887214019	GSTA1/GSTM3/ADH7/ALDH3A1/GSTP1/MGST2/GSTM4/GSTO1/GSTA4/UGT1A7/ALDH3B2	11

hsa05017	Spinocerebellar ataxia	16/316	143/8577	6.92212387868047e-05	0.000830654865441656	0.000722572580318399	CYCS/SLC25A5/PSMA7/PSMB6/VDAC1/PSMD8/PSMA3/VDAC2/PSMD11/PSMD7/PSMB1/PSMB3/PSMD1/PSMA1/SEM1/PSMB5	16
hsa00010	Glycolysis / Gluconeogenesis	10/316	67/8577	0.000153212731727981	0.00176501066950634	0.00153535284847408	ADH7/ALDH3A1/TPI1/LDHA/HK1/ENO1/ALDH3B2/PGK1/GPI/PGAM1	10
hsa05110	Vibrio cholerae infection	8/316	50/8577	0.000431761754404756	0.00478259174109884	0.00416029544730089	TJP1/SEC61B/ATP6V0E1/ACTB/SEC61G/ATP6V0B/ARF1/ATP6V0D1	8
hsa04530	Tight junction	16/316	170/8577	0.000517078433015507	0.00551550328549874	0.00479784277028033	CLDN4/CLDN7/TUBA4A/SLC9A3R1/ARPC2/ARPC3/TUBA1A/TJP1/ACTR3/CDC42/ACTB/CCND1/TUBA1C/MYL12B/DLG3/YBX3	16
hsa00983	Drug metabolism - other enzymes	10/316	80/8577	0.000666368243672598	0.00685407336348958	0.00596224218022851	GSTA1/GSTM3/GSTP1/MGST2/GSTM4/GSTO1/GSTA4/UGT1A7/CES2/CMPK1	10
hsa05120	Epithelial cell signaling in Helicobacter pylori infection	9/316	70/8577	0.00100223444516262	0.0099532248347184	0.0086581414863413	HBEGF/PAK1/MAPK13/PTPRZ1/TJP1/CDC42/ATP6V0E1/ATP6V0B/ATP6V0D1	9
hsa00020	Citrate cycle (TCA cycle)	5/316	30/8577	0.00438901073842426	0.0421345030888729	0.0366520896752623	IDH1/SUCLG1/SDHB/MDH2/SDHC	5

hsa00920	Sulfur metabolism	3/316	10/8577	0.0049014226664568	0.0450924712153557	0.0392251759841179	TST/SQOR/ETHE1	3
hsa04144	Endocytosis	18/316	250/8577	0.00501027457948397	0.0450924712153557	0.0392251759841179	SMAP1/RAB11A/CLTB/ARPC2/RAB10/ARPC3/AP2S1/ACTR3/CDC42/CAPZB/VPS29/VPS25/SNX1/PLD1/CHMP4B/ARF1/CHMP2A/CAPZA1	18
hsa01524	Platinum drug resistance	8/316	73/8577	0.00520104154633054	0.0453909080407029	0.0394847811172781	GSTA1/GSTM3/GSTP1/MGST2/CYCS/GSTM4/GSTO1/GSTA4	8
hsa03060	Protein export	4/316	23/8577	0.00919369614902255	0.0778760144387792	0.0677430242559556	SEC61B/OXA1L/SEC61G/SEC11C	4
hsa01250	Biosynthesis of nucleotide sugars	5/316	37/8577	0.0108870466713621	0.0895848411814939	0.0779283340686972	NAGK/HK1/UGDH/GPI/CMAS	5
hsa00790	Folate biosynthesis	4/316	27/8577	0.016239506493729	0.126404807302539	0.109957398165 22	AKR1C3/AKR1B10/PTS/CBR1	4
hsa01040	Biosynthesis of unsaturated fatty acids	4/316	27/8577	0.016239506493729	0.126404807302539	0.10995739816522	ELOVL6/SCD/TECR/ELOVL1	4
hsa01212	Fatty acid metabolism	6/316	57/8577	0.0178529334903196	0.135306443295054	0.1177007803516 92	ELOVL6/ACSL1/SCD/TECR/ACADVL/ELOVL1	6
hsa05131	Shigellosis	16/316	247/8577	0.0201525419643117	0.148818771428764	0.129454925438764	CYCS/ARPC2/HK1/ARPC3/PFN1/VDAC1/MAPK13/PYCARD/CD44/UBE2V2/ACTR3/CDC42/ACTB/CASP1/ARF1/MYL12B	16

hsa05133	Pertussis	7/316	76/8577	0.0214695875525788	0.1545810303785670	0.1344674167766770	CALML3/CALML5/MAPK13/CALM1/PYCARD/CASP1/CFL1	7
hsa05100	Bacterial invasion of epithelial cells	7/316	77/8577	0.0228990566399878	0.1595688696609580	0.1388062535793420	CDH1/CLTB/ARPC2/ARPC3/ACTR3/CDC42/ACTB	7
hsa00030	Pentose phosphate pathway	4/316	30/8577	0.0232704601588897	0.1595688696609580	0.1388062535793420	TALDO1/PGD/GPI/DERA	4
hsa04666	Fc gamma R-mediated phagocytosis	8/316	97/8577	0.0263019638968178	0.176161990750780	0.153240328211570	ARPC2/PAK1/ARPC3/ACTR3/CDC42/CFL1/SCIN/PLD1	8
hsa00620	Pyruvate metabolism	5/316	47/8577	0.0284755276761263	0.1863852720619180	0.1621333872468430	ADH7/ME1/LDHA/MDH2/GLO1	5
hsa00520	Amino sugar and nucleotide sugar metabolism	5/316	49/8577	0.0333721895794841	0.2135820133086980	0.1857913712261340	NAGK/HK1/UGDH/GPI/CMAS	5
hsa03020	RNA polymerase	4/316	34/8577	0.0351365417609347	0.2199853049380260	0.1913614860206510	POLR2J3/POLR2L/POLR2E/POLR2I	4
hsa00830	Retinol metabolism	6/316	68/8577	0.0386082649055860	0.2365783041023140	0.2057954545919250	ADH7/ALDH1A1/RDH11/RDH12/CYP2C18/UGT1A7	6
hsa00040	Pentose and glucuronate interconversions	4/316	36/8577	0.0421670313388445	0.2530021880330670	0.2200823126895830	AKR1B10/UGDH/UGT1A7/DCXR	4

hsa04961	Endocrine and other factor-regulated calcium reabsorption	5/316	53/8577	0.0446543258674943	0.262458078568813	0.228307831502978	RAB11A/ATP1B1/CLTB/ATP2B1/AP2S1	5
hsa05225	Hepatocellular carcinoma	11/316	168/8577	0.0459466898045404	0.2646529332741 53	0.2302170983890 65	GSTA1/GSTM3/GSTP1/NQO1/MGST2/NFE2L2/GSTM4/GSTO1/ACTB/GSTA4/CCND1	11
hsa05164	Influenza A	11/316	171/8577	0.0510430343771208	0.288243017659035	0.250737712729716	TMPRSS11D/RAB11A/CYCS/SLC25A5/TMPRSS4/VDAC1/PYCARD/ACTB/CASP1/NLRX1/PRSS3	11
hsa04115	p53 signaling pathway	6/316	74/8577	0.0545768997011082	0.294593963473 92	0.256262292787986	PERP/SFN/SERPINB5/CYCS/CCND1/CD82	6
hsa04520	Adherens junction	7/316	93/8577	0.0552363681513601	0.294593963473 92	0.256262292787986	CDH1/NECTIN4/TJP1/CDC42/ACTB/CTNND1/MYL12B	7
hsa04912	GnRH signaling pathway	7/316	93/8577	0.0552363681513601	0.294593963473 92	0.256262292787986	CALML3/CALML5/HBEGF/MAPK13/CALM1/CDC42/PLD1	7
hsa01230	Biosynthesis of amino acids	6/316	75/8577	0.0575746032505639	0.301481558839317	0.262253695189172	TALDO1/IDH1/TPI1/ENO1/PGK1/PGAM1	6
hsa04915	Estrogen signaling pathway	9/316	137/8577	0.0659494055576912	0.339168371439555	0.295036814337 04	KRT16/CALML3/KRT17/CALML5/HBEGF/CALM1/KRT19/KRT10/KRT18	9
hsa00062	Fatty acid elongation	3/316	27/8577	0.0755385375913246	0.381668400461429	0.332006868822442	ELOVL6/TECR/ELOVL1	3

hsa00140	Steroid hormone biosynthesis	5/316	62/8577	0.0774577185704758	0.384617637039604	0.334572359705503	AKR1C2/AKR1C3/SULT2B1/AKR1C1/UGT1A7	5
hsa04625	C-type lectin receptor signaling pathway	7/316	104/8577	0.0887250447393138	0.429630448461139	0.373728240985932	CALML3/CALML5/PAK1/MAPK13/CALM1/PYCARD/CASP1	7
hsa04744	Phototransduction	3/316	29/8577	0.089506343429404	0.429630448461139	0.373728240985932	CALML3/CALML5/CALM1	3
hsa04540	Gap junction	6/316	88/8577	0.105321563122112	0.494630434380963	0.430270626398644	TUBA4A/GJA1/TUBA1A/TJP1/GUCY1A1/TUBA1C	6
hsa04066	HIF-1 signaling pathway	7/316	109/8577	0.107050481293 76	0.494630434380963	0.430270626398644	LDHA/HK1/ENO1/HIF1A/ELOB/PGK1/EIF4E2	7
hsa04145	Phagosome	9/316	152/8577	0.1083986296670 91	0.494630434380963	0.430270626398644	TUBA4A/TUBA1A/SEC61B/ATP6V0E1/ACTB/SEC61G/ATP6V0B/TUBA1C/ATP6V0D1	9
hsa04924	Renin secretion	5/316	69/8577	0.1099178743068 81	0.494630434380963	0.430270626398644	CALML3/CLCA2/CALML5/CALM1/GUCY1A1	5
hsa00640	Propanoate metabolism	3/316	32/8577	0.112285287440459	0.497366462208686	0.432650650605509	SUCLG1/LDHA/PCCB	3
hsa05230	Central carbon metabolism in cancer	5/316	70/8577	0.115023080786011	0.497366462208686	0.432650650605509	IDH1/LDHA/HK1/HIF1A/PGAM1	5

hsa00360	Phenylalanine metabolism	2/316	16/8577	0.115706781138826	0.497366462208686	0.432650650605509	ALDH3A1/ALDH3B2	2
hsa04218	Cellular senescence	9/316	156/8577	0.121831002003073	0.509723142419846	0.443399517163463	CALML3/CALML5/SLC25A5/VDAC1/MAPK13/CALM1/CDKN2B/CCND1/VDAC2	9
hsa04390	Hippo signaling pathway	9/316	157/8577	0.125324359228597	0.509723142419846	0.443399517163463	CDH1/YWHAZ/YWHAB/SOX2/ACTB/CCND1/FRMD6/DLG3/YWHAQ	9
hsa05169	Epstein-Barr virus infection	11/316	202/8577	0.126191668522243	0.509723142419846	0.443399517163463	CYCS/MAPK13/CD44/PSMD8/HES1/CCND1/PSMD11/PSMD7/PSMD1/HDAC1/SEM1	11
hsa04970	Salivary secretion	6/316	93/8577	0.127874444090326	0.509723142419846	0.443399517163463	CALML3/CALML5/ATP1B1/ATP2B1/CALM1/GUCY1A1	6
hsa00051	Fructose and mannose metabolism	3/316	34/8577	0.128568062193183	0.509723142419846	0.443399517163463	AKR1B10/TPI1/HK1	3
hsa00600	Sphingolipid metabolism	4/316	53/8577	0.130333137128646	0.509723142419846	0.443399517163463	DEGS2/SPTLC2/ASAH1/CERS3	4
hsa04670	Leukocyte transendothelial migration	7/316	115/8577	0.131503340721781	0.509723142419846	0.443399517163463	CLDN4/CLDN7/MAPK13/CDC42/ACTB/CTNND1/MYL12B	7
hsa05135	Yersinia infection	8/316	137/8577	0.132740401671835	0.509723142419846	0.443399517163463	ARPC2/ARPC3/MAPK13/PYCARD/ACTR3/CDC42/ACTB/CASP1	8

hsa04810	Regulation of actin cytoskeleton	12/316	229/8577	0.139011255556943	0.517135728312597	0.449847599921046	ARPC2/PAK1/ARPC3/PFN1/ACTR3/CDC42/ACTB/BRK1/CFL1/TMSB4X/SCIN/MYL12B	12
hsa03320	PPAR signaling pathway	5/316	75/8577	0.142176764977051	0.517135728312597	0.449847599921046	DBI/ME1/HMGCS1/ACSL1/SCD	5
hsa05150	Staphylococcus aureus infection	6/316	96/8577	0.142439571123653	0.517135728312597	0.449847599921046	KRT16/KRT17/DSG1/KRT19/KRT10/KRT18	6
hsa00350	Tyrosine metabolism	3/316	36/8577	0.145628101740613	0.517135728312597	0.449847599921046	ADH7/ALDH3A1/ALDH3B2	3
hsa04971	Gastric acid secretion	5/316	76/8577	0.147916725969673	0.517135728312597	0.449847599921046	CALML3/CALML5/ATP1B1/CALM1/ACTB	5
hsa05212	Pancreatic cancer	5/316	76/8577	0.147916725969673	0.517135728312597	0.449847599921046	RALA/CDC42/RALB/CCND1/PLD1	5
hsa05134	Legionellosis	4/316	56/8577	0.150536144041258	0.517135728312597	0.449847599921046	CYCS/PYCARD/CASP1/ARF1	4
hsa04015	Rap1 signaling pathway	11/316	210/8577	0.152287390543103	0.517135728312597	0.449847599921046	CALML3/CALML5/CDH1/RALA/PFN1/MAPK13/CALM1/CDC42/ACTB/RALB/CTNND1	11
hsa05412	Arrhythmogenic right ventricular cardiomyopathy	5/316	77/8577	0.153753917215552	0.517135728312597	0.449847599921046	DSC2/JUP/DSP/GJA1/ACTB	5

hsa04960	Aldosterone-regulated sodium reabsorption	3/316	37/8577	0.154422474426678	0.517135728312597	0.449847599921046	SFN/SGK1/ATP1B1	3
hsa05216	Thyroid cancer	3/316	37/8577	0.154422474426678	0.517135728312597	0.449847599921046	CDH1/CCND1/NCOA4	3
hsa04071	Sphingolipid signaling pathway	7/316	121/8577	0.158482533058018	0.522607678121874	0.454607556260987	DEGS2/SPTSSB/SPTLC2/ASAH1/MAPK13/CERS3/PLD1	7
hsa04721	Synaptic vesicle cycle	5/316	78/8577	0.159685679426128	0.522607678121874	0.454607556260987	CLTB/AP2S1/ATP6V0E1/ATP6V0B/ATP6V0D1	5
hsa04022	cGMP-PKG signaling pathway	9/316	167/8577	0.163113621802173	0.527828349202538	0.459148929496 36	CALML3/CALML5/SLC25A5/ATP1B1/ATP2B1/VDAC1/CALM1/VDAC2/GUCY1A1	9
hsa04814	Motor proteins	10/316	193/8577	0.174842730543502	0.559496737739205	0.486696723618168	DYNLT3/TUBA4A/DYNLL1/DYNLT1/TUBA1A/ACTB/CAPZB/TUBA1C/MYL12B/CAPZA1	10
hsa04978	Mineral absorption	4/316	60/8577	0.179171388349062	0.567047910379 45	0.493265360637095	ATP1B1/ATP2B1/ATOX1/HMOX2	4
hsa00590	Arachidonic acid metabolism	4/316	61/8577	0.186600468599797	0.584140597355887	0.508133999161919	AKR1C3/PTGR1/HPGD/CBR1	4
hsa04216	Ferroptosis	3/316	41/8577	0.191110798853 68	0.585530958189999	0.509343450472294	ACSL1/VDAC2/NCOA4	3

hsa05219	Bladder cancer	3/316	41/8577	0.191110798853680	0.585530958189999	0.509343450472294	CDH1/HBEGF/CCND1	3
hsa00340	Histidine metabolism	2/316	22/8577	0.193548558584669	0.586757735498787	0.510410603248214	ALDH3A1/ALDH3B2	2
hsa04922	Glucagon signaling pathway	6/316	107/8577	0.201723286096663	0.595926682777285	0.518386514989013	CALML3/CALML5/LDHA/CALM1/PGAM1/PPP4C	6
hsa03420	Nucleotide excision repair	4/316	63/8577	0.201747548770815	0.595926682777285	0.518386514989013	POLR2J3/POLR2L/POLR2E/POLR2I	4
hsa01240	Biosynthesis of cofactors	8/316	153/8577	0.202780607333937	0.595926682777285	0.518386514989013	NQO1/RDH11/RDH12/PTS/UGDH/UGT1A7/CMPK1/PANK3	8
hsa00900	Terpenoid backbone biosynthesis	2/316	23/8577	0.207136810010845	0.599420783008387	0.521425973523377	HMGCS1/HMGCR	2
hsa04114	Oocyte meiosis	7/316	131/8577	0.208425263201220	0.599420783008387	0.521425973523377	CALML3/CALML5/YWHAZ/MAPK13/CALM1/YWHAB/YWHAQ	7
hsa00071	Fatty acid degradation	3/316	43/8577	0.210213538485580	0.599420783008387	0.521425973523377	ADH7/ACSL1/ACADVL	3
hsa05205	Proteoglycans in cancer	10/316	205/8577	0.224369361302455	0.633513490736344	0.551082641795504	HBEGF/SDC1/PAK1/HIF1A/MAPK13/CD44/CDC42/ACTB/CCND1/SDC4	10

hsa04514	Cell adhesion molecules	8/316	158/8577	0.227265116120359	0.635459742161781	0.552775652903889	CLDN4/CLDN7/CDH1/SDC1/VTCN1/LRRC4/ALCAM/SDC4	8
hsa05031	Amphetamine addiction	4/316	69/8577	0.24913292791085	0.683336030841188	0.594422424489045	CALML3/CALML5/CALM1/HDAC1	4
hsa05211	Renal cell carcinoma	4/316	69/8577	0.24913292791085	0.683336030841188	0.594422424489045	PAK1/HIF1A/CDC42/ELOB	4
hsa05207	Chemical carcinogenesis - receptor activation	10/316	212/8577	0.255547079427106	0.694316593160 44	0.6039742294305	GSTA1/GSTM3/MGST2/GSTM4/GSTO1/EPHX1/GSTA4/UGT1A7/CCND1/KLF4	10
hsa00650	Butanoate metabolism	2/316	27/8577	0.262205844273195	0.69921558472852	0.60823577911326	HMGCS1/BDH1	2
hsa04966	Collecting duct acid secretion	2/316	27/8577	0.262205844273195	0.69921558472852	0.60823577911326	ATP6V0E1/ATP6V0D1	2
hsa04722	Neurotrophin signaling pathway	6/316	119/8577	0.27466956601203	0.72573243129784	0.631302334243004	CALML3/CALML5/MAPK13/CALM1/CDC42/NTRK2	6
hsa04713	Circadian entrainment	5/316	97/8577	0.286470191017499	0.734858785728161	0.639241195187508	CALML3/CALML5/CALM1/GNG5/GUCY1A1	5
hsa04979	Cholesterol metabolism	3/316	51/8577	0.289825632628342	0.734858785728161	0.639241195187508	TSPO/VDAC1/VDAC2	3

hsa04392	Hippo signaling pathway - multiple species	2/316	29/8577	0.289854655600797	0.734858785728161	0.639241195187508	PAK1/FRMD6	2
hsa04750	Inflammatory mediator regulation of TRP channels	5/316	98/8577	0.293669844762308	0.734858785728161	0.639241195187508	CALML3/CALML5/MAPK13/CALM1/F2RL1	5
hsa04925	Aldosterone synthesis and secretion	5/316	98/8577	0.293669844762308	0.734858785728161	0.639241195187508	CALML3/CALML5/ATP1B1/ATP2B1/CALM1	5
hsa04623	Cytosolic DNA-sensing pathway	4/316	75/8577	0.298590702025998	0.734858785728161	0.639241195187508	PYCARD/POLR2L/CASP1/POLR2E	4
hsa05214	Glioma	4/316	75/8577	0.298590702025998	0.734858785728161	0.639241195187508	CALML3/CALML5/CALM1/CCND1	4
hsa00270	Cysteine and methionine metabolism	3/316	52/8577	0.299986376763723	0.734858785728161	0.639241195187508	TST/LDHA/MDH2	3
hsa00053	Ascorbate and aldarate metabolism	2/316	30/8577	0.303639567714066	0.734858785728161	0.639241195187508	UGDH/UGT1A7	2
hsa00630	Glyoxylate and dicarboxylate metabolism	2/316	30/8577	0.303639567714066	0.734858785728161	0.639241195187508	MDH2/PCCB	2

hsa00410	beta-Alanine metabolism	2/316	31/8577	0.317375927892296	0.761702226941511	0.662591849459356	ALDH3A1/ALDH3B2	2
hsa04972	Pancreatic secretion	5/316	102/8577	0.322748974245674	0.761899217891428	0.662763208545906	CLCA2/RAB11A/ATP1B1/ATP2B1/PRSS3	5
hsa05146	Amoebiasis	5/316	102/8577	0.322748974245674	0.761899217891428	0.662763208545906	SERPINB13/SERPINB3/HSPB1/PRDX1/SERPINB6	5
hsa00052	Galactose metabolism	2/316	32/8577	0.331048637255752	0.775138272598835	0.674279637713899	AKR1B10/HK1	2
hsa05203	Viral carcinogenesis	9/316	204/8577	0.338053153668363	0.779788652906068	0.678324924677062	YWHAZ/YWHAB/CDC42/CDKN2B/CCND1/HDAC1/SCIN/YWHAQ/ATP6V0D1	9
hsa00130	Ubiquinone and other terpenoid-quinone biosynthesis	1/316	11/8577	0.338449936157147	0.779788652906068	0.678324924677062	NQO1	1
hsa04360	Axon guidance	8/316	182/8577	0.356089968786363	0.803835038086653	0.69924246734146	EPHB6/ABLIM1/PAK1/CDC42/LRRC4/CFL1/EPHA4/MYL12B	8
hsa04110	Cell cycle	7/316	157/8577	0.358037982215039	0.803835038086653	0.69924246734146	SFN/YWHAZ/YWHAB/CDKN2B/CCND1/HDAC1/YWHAQ	7
hsa04142	Lysosome	6/316	132/8577	0.359337996736643	0.803835038086653	0.69924246734146	LGMN/CLTB/ASAH1/CTSC/ATP6V0B/ATP6V0D1	6

hsa05160	Hepatitis C	7/316	158/8577	0.364100300510819	0.803835038086653	0.699242467341 46	CLDN4/CLDN7/YWHAZ/CYCS/YWHAB/CCND1/YWHAQ	7
hsa04146	Peroxisome	4/316	83/8577	0.365989832861047	0.803835038086653	0.699242467341 46	PRDX1/IDH1/ACSL1/ECH1	4
hsa04217	Necroptosis	7/316	159/8577	0.370170634387394	0.803835038086653	0.699242467341 46	SLC25A5/VDAC1/PYCARD/CASP1/VDAC2/CHMP4B/CHMP2A	7
hsa04014	Ras signaling pathway	10/316	236/8577	0.371038259655761	0.803835038086653	0.699242467341 46	CALML3/CALML5/RALA/PAK1/CALM1/CDC42/GNG5/RALB/NTRK2/PLD1	10
hsa04370	VEGF signaling pathway	3/316	59/8577	0.371215486338628	0.803835038086653	0.699242467341 46	HSPB1/MAPK13/CDC42	3
hsa04621	NOD-like receptor signaling pathway	8/316	186/8577	0.378610194423601	0.805303445224659	0.70051981	TXN/CARD18/VDAC1/MAPK13/PYCARD/CASP1/VDAC2/NLRX1	8
hsa05170	Human immunodeficiency virus 1 infection	9/316	212/8577	0.380115443787422	0.805303445224659	0.70051981	CALML3/CALML5/CYCS/PAK1/MAPK13/CALM1/GNG5/ELOB/CFL1	9
hsa05416	Viral myocarditis	3/316	60/8577	0.381323070443342	0.805303445224659	0.70051981	CYCS/ACTB/CCND1	3
hsa00500	Starch and sucrose metabolism	2/316	36/8577	0.384840640312855	0.805303445224659	0.70051981	HK1/GPI	2

hsa04210	Apoptosis	6/316	136/8577	0.385874567503483	0.805303445224659	0.70051981	TUBA4A/CYCS/TUBA1A/CTSC/ACTB/TUBA1C	6
hsa05210	Colorectal cancer	4/316	86/8577	0.391291928110027	0.807457754512125	0.702393806922097	CYCS/RALA/RALB/CCND1	4
hsa04910	Insulin signaling pathway	6/316	137/8577	0.392514186221172	0.807457754512125	0.702393806922097	CALML3/CALML5/HK1/CALM1/SOCS2/EIF4E2	6
hsa05417	Lipid and atherosclerosis	9/316	215/8577	0.396010453361111	0.808872415375885	0.703624396416157	CALML3/CALML5/NFE2L2/CYCS/MAPK13/CALM1/PYCARD/CDC42/CASP1	9
hsa04371	Apelin signaling pathway	6/316	139/8577	0.405786745600081	0.818008047229055	0.711571327633462	CALML3/CALML5/CDH1/CALM1/GNG5/CCND1	6
hsa04613	Neutrophil extracellular trap formation	8/316	191/8577	0.406874239008941	0.818008047229055	0.711571327633462	SLC25A5/VDAC1/MAPK13/ACTB/CLCN3/CASP1/VDAC2/HDAC1	8
hsa00533	Glycosaminoglycan biosynthesis - keratan sulfate	1/316	14/8577	0.409004023614527	0.818008047229055	0.711571327633462	B4GALT4	1
hsa04976	Bile secretion	4/316	89/8577	0.416448474457757	0.827152832026 07	0.719526220841391	ATP1B1/EPHX1/UGT1A7/HMGCR	4
hsa05167	Kaposi sarcoma-associated	8/316	194/8577	0.423836555932303	0.836061151428105	0.727275416812 46	CALML3/CALML5/CYCS/HIF1A/MAPK13/CALM1/GNG5/CCND1	8

	herpesvirus infection							
hsa 034 40	Homologous recombination	2/316	41/85 77	0.44927 838006 7308	0.87719 448795 296	0.76305 660867 2531	SEM1/SSBP1	2
hsa 047 20	Long-term potentiation	3/316	67/85 77	0.45078 050075 3604	0.87719 448795 296	0.76305 660867 2531	CALML3/CALML5/CALM1	3
hsa 049 19	Thyroid hormone signaling pathway	5/316	121/8 577	0.46211 446167 221	0.89321 452994 3601	0.77699 217151 5267	ATP1B1/HIF1A/ACTB/CCND1/HDAC1	5
hsa 008 60	Porphyrin metabolism	2/316	43/85 77	0.47398 013321 2869	0.91004 185576 8708	0.79162 997687 4826	UGT1A7/HMOX2	2
hsa 049 17	Prolactin signaling pathway	3/316	70/85 77	0.47959 413164 3043	0.91286 456870 1028	0.79408 540698 4081	MAPK13/CCND1/SOCS2	3
hsa 046 11	Platelet activation	5/316	124/8 577	0.48359 598534 508	0.91286 456870 1028	0.79408 540698 4081	MAPK13/ACTB/GUCY1A1/MYL12B/VAMP8	5
hsa 049 62	Vasopressin-regulated water reabsorption	2/316	44/85 77	0.48607 830304 7024	0.91286 456870 1028	0.79408 540698 4081	DYNLL1/RAB11A	2
hsa 015 22	Endocrine resistance	4/316	98/85 77	0.49017 365119 9311	0.91286 456870 1028	0.79408 540698 4081	HBEGF/MAPK13/CCND1/BIK	4

hsa00061	Fatty acid biosynthesis	1/316	18/8577	0.491544367108391	0.912864568701028	0.794085406984081	ACSL1	1
hsa05152	Tuberculosis	7/316	180/8577	0.496948922819664	0.912864568701028	0.794085406984081	CALML3/CALML5/CYCS/MAPK13/CALM1/ATP6V0B/ATP6V0D1	7
hsa04261	Adrenergic signaling in cardiomyocytes	6/316	154/8577	0.503728017866 17	0.912864568701028	0.794085406984081	CALML3/CALML5/ATP1B1/ATP2B1/MAPK13/CALM1	6
hsa04921	Oxytocin signaling pathway	6/316	154/8577	0.503728017866 17	0.912864568701028	0.794085406984081	CALML3/CALML5/CALM1/ACTB/CCND1/GUCY1A1	6
hsa04933	AGE-RAGE signaling pathway in diabetic complications	4/316	100/8577	0.506059034049489	0.912864568701028	0.794085406984081	MAPK13/F3/CDC42/CCND1	4
hsa04930	Type II diabetes mellitus	2/316	46/8577	0.509748449531535	0.912864568701028	0.794085406984081	HK1/SOCS2	2
hsa01210	2-Oxocarboxylic acid metabolism	1/316	19/8577	0.510316651253005	0.912864568701028	0.794085406984081	IDH1	1
hsa04973	Carbohydrate digestion and absorption	2/316	47/8577	0.521313696809906	0.926779905439833	0.806190122422077	ATP1B1/HK1	2

hsa 04918	Thyroid hormone synthesis	3/316	75/8577	0.52596559454124	0.929313443115811	0.808394003879982	GPX2/ATP1B1/GPX3	3
hsa 00280	Valine, leucine and isoleucine degradation	2/316	48/8577	0.532695376220267	0.935465050923395	0.813745183186287	HMGCS1/PCCB	2
hsa 04728	Dopaminergic synapse	5/316	132/8577	0.539289553365944	0.941305402238739	0.818825605748611	CALML3/CALML5/MAPK13/CALM1/GNG5	5
hsa 00770	Pantothenate and CoA biosynthesis	1/316	21/8577	0.545813682678412	0.946953859104714	0.823739102583779	PANK3	1
hsa 03430	Mismatch repair	1/316	23/8577	0.578745094323812	0.992134447412249	0.86304093	SSBP1	1
hsa 04964	Proximal tubule bicarbonate reclamation	1/316	23/8577	0.578745094323812	0.992134447412249	0.86304093	ATP1B1	1
hsa 04020	Calcium signaling pathway	9/316	253/8577	0.591483246188456	0.999992176891026	0.869876235745849	CALML3/CALML5/SLC25A5/C4orf3/ATP2B1/VDAC1/CALM1/NTRK2/VDAC2	9
hsa 04936	Alcoholic liver disease	5/316	142/8577	0.604734148580378	0.999992176891026	0.869876235745849	ADH7/MAPK13/SCD/CCND1/ACADVL	5
hsa 01232	Nucleotide metabolism	3/316	85/8577	0.611383275345839	0.999992176891026	0.869876235745849	APRT/GUK1/CMPK1	3

hsa 049 50	Maturity onset diabetes of the young	1/316	26/85 77	0.62373 246104 7301	0.99999 217689 1026	0.86987 623574 5849	HES1	1
hsa 045 10	Focal adhesion	7/316	203/8 577	0.62521 557758 7075	0.99999 217689 1026	0.86987 623574 5849	EMP2/PAK1/CDC42/ACTB/CCND1/EMP1/MYL12B	7
hsa 052 13	Endometrial cancer	2/316	58/85 77	0.63611 319118 7239	0.99999 217689 1026	0.86987 623574 5849	CDH1/CCND1	2
hsa 045 12	ECM-receptor interaction	3/316	89/85 77	0.64253 647764 3267	0.99999 217689 1026	0.86987 623574 5849	SDC1/CD44/SDC4	3
hsa 006 01	Glycosphingol ipid biosynthesis - lacto and neolacto series	1/316	28/85 77	0.65102 991611 9515	0.99999 217689 1026	0.86987 623574 5849	B4GALT4	1
hsa 041 52	AMPK signaling pathway	4/316	121/8 577	0.65700 471660 5466	0.99999 217689 1026	0.86987 623574 5849	RAB10/SCD/HMGCR/CCND1	4
hsa 052 22	Small cell lung cancer	3/316	92/85 77	0.66472 458271 8947	0.99999 217689 1026	0.86987 623574 5849	CYCS/CDKN2B/CCND1	3
hsa 053 23	Rheumatoid arthritis	3/316	93/85 77	0.67189 515578 6256	0.99999 217689 1026	0.86987 623574 5849	ATP6V0E1/ATP6V0B/ATP6V0D1	3
hsa 043 30	Notch signaling pathway	2/316	62/85 77	0.67216 711853 3963	0.99999 217689 1026	0.86987 623574 5849	HES1/HDAC1	2

hsa00561	Glycerolipid metabolism	2/316	63/8577	0.680716552916708	0.999992176891026	0.869876235745849	AKR1B10/MGLL	2
hsa03250	Viral life cycle - HIV-1	2/316	63/8577	0.680716552916708	0.999992176891026	0.869876235745849	ELL2/CHMP4B	2
hsa05034	Alcoholism	6/316	188/8577	0.697287589695913	0.999992176891026	0.869876235745849	CALML3/CALML5/CALM1/GNG5/NTRK2/HDAC1	6
hsa04070	Phosphatidylinositol signaling system	3/316	97/8577	0.699451422232891	0.999992176891026	0.869876235745849	CALML3/CALML5/CALM1	3
hsa04215	Apoptosis - multiple species	1/316	32/8577	0.699843544030904	0.999992176891026	0.869876235745849	CYCS	1
hsa04130	SNARE interactions in vesicular transport	1/316	33/8577	0.710943537019346	0.999992176891026	0.869876235745849	VAMP8	1
hsa04640	Hematopoietic cell lineage	3/316	99/8577	0.712557604862363	0.999992176891026	0.869876235745849	CD24/CD9/CD44	3
hsa05221	Acute myeloid leukemia	2/316	67/8577	0.713107831180326	0.999992176891026	0.869876235745849	JUP/CCND1	2
hsa04068	FoxO signaling pathway	4/316	131/8577	0.716995211100073	0.999992176891026	0.869876235745849	SGK1/MAPK13/CDKN2B/CCND1	4

hsa05161	Hepatitis B	5/316	162/8577	0.718228684425703	0.999992176891026	0.869876235745849	YWHAZ/CYCS/MAPK13/YWHAB/YWHAQ	5
hsa04916	Melanogenesis	3/316	101/8577	0.725220424112566	0.999992176891026	0.869876235745849	CALML3/CALML5/CALM1	3
hsa04024	cAMP signaling pathway	7/316	225/8577	0.729002411177864	0.999992176891026	0.869876235745849	CALML3/CALML5/ATP1B1/ATP2B1/PAK1/CALM1/PLD1	7
hsa05163	Human cytomegalovirus infection	7/316	225/8577	0.729002411177864	0.999992176891026	0.869876235745849	CALML3/CALML5/CYCS/MAPK13/CALM1/GNG5/CCND1	7
hsa04270	Vascular smooth muscle contraction	4/316	134/8577	0.733412951133838	0.999992176891026	0.869876235745849	CALML3/CALML5/CALM1/GUCY1A1	4
hsa00512	Mucin type O-glycan biosynthesis	1/316	36/8577	0.741847725089968	0.999992176891026	0.869876235745849	GALNT7	1
hsa03030	DNA replication	1/316	36/8577	0.741847725089968	0.999992176891026	0.869876235745849	SSBP1	1
hsa03010	Ribosome	5/316	167/8577	0.742668276540799	0.999992176891026	0.869876235745849	MRPL14/MRPL33/RPS26/MRPL36/RPL22L1	5
hsa04622	RIG-I-like receptor signaling pathway	2/316	71/8577	0.742706160878464	0.999992176891026	0.869876235745849	MAPK13/NLRX1	2

hsa05218	Melanoma	2/316	72/8577	0.749686162691106	0.999992176891026	0.869876235745849	CDH1/CCND1	2
hsa05143	African trypanosomiasis	1/316	37/8577	0.751398845435544	0.999992176891026	0.869876235745849	F2RL1	1
hsa04928	Parathyroid hormone synthesis, secretion and action	3/316	106/8577	0.754971462598355	0.999992176891026	0.869876235745849	SLC9A3R1/HBEGF/PLD1	3
hsa04141	Protein processing in endoplasmic reticulum	5/316	170/8577	0.756568939015531	0.999992176891026	0.869876235745849	NFE2L2/SEC61B/SSR4/SEC61G/RPN2	5
hsa05220	Chronic myeloid leukemia	2/316	76/8577	0.776001108081517	0.999992176891026	0.869876235745849	CCND1/HDAC1	2
hsa00260	Glycine, serine and threonine metabolism	1/316	40/8577	0.777989778091071	0.999992176891026	0.869876235745849	PGAM1	1
hsa00513	Various types of N-glycan biosynthesis	1/316	42/8577	0.794122090932812	0.999992176891026	0.869876235745849	RPN2	1
hsa04072	Phospholipase D signaling pathway	4/316	148/8577	0.800639701087408	0.999992176891026	0.869876235745849	RALA/RALB/PLD1/ARF1	4

hsa05226	Gastric cancer	4/316	149/8577	0.8048710532916	0.999992176891026	0.869876235745849	JUP/CDH1/CDKN2B/CCND1	4
hsa04012	ErbB signaling pathway	2/316	85/8577	0.826525903009853	0.999992176891026	0.869876235745849	HBEGF/PAK1	2
hsa04660	T cell receptor signaling pathway	3/316	121/8577	0.828899040120539	0.999992176891026	0.869876235745849	PAK1/MAPK13/CDC42	3
hsa00514	Other types of O-glycan biosynthesis	1/316	47/8577	0.829522348779355	0.999992176891026	0.869876235745849	GALNT7	1
hsa04610	Complement and coagulation cascades	2/316	86/8577	0.831458939170039	0.999992176891026	0.869876235745849	SERPINB2/F3	2
hsa05166	Human T-cell leukemia virus 1 infection	6/316	222/8577	0.833226120945048	0.999992176891026	0.869876235745849	TSPO/SLC25A5/VDAC1/CDKN2B/CCND1/VDAC2	6
hsa05235	PD-L1 expression and PD-1 checkpoint pathway in cancer	2/316	89/8577	0.845509179822388	0.999992176891026	0.869876235745849	HIF1A/MAPK13	2
hsa00330	Arginine and proline metabolism	1/316	50/8577	0.847777595423599	0.999992176891026	0.869876235745849	ODC1	1

hsa00565	Ether lipid metabolism	1/316	50/8577	0.847777595423599	0.999992176891026	0.869876235745849	PLD1	1
hsa05144	Malaria	1/316	50/8577	0.847777595423599	0.999992176891026	0.869876235745849	SDC1	1
hsa04913	Ovarian steroidogenesis	1/316	51/8577	0.853418768150952	0.999992176891026	0.869876235745849	AKR1C3	1
hsa00230	Purine metabolism	3/316	128/8577	0.856317615242859	0.999992176891026	0.869876235745849	APRT/GUK1/GUCY1A1	3
hsa00510	N-Glycan biosynthesis	1/316	53/8577	0.864083537088032	0.999992176891026	0.869876235745849	RPN2	1
hsa05165	Human papillomavirus infection	9/316	331/8577	0.867107449165651	0.999992176891026	0.869876235745849	SLC9A3R1/CDC42/ATP6V0E1/HES1/CCND1/HDAC1/ATP6V0B/DLG3/ATP6V0D1	9
hsa03460	Fanconi anemia pathway	1/316	55/8577	0.873974649013867	0.999992176891026	0.869876235745849	HES1	1
hsa05215	Prostate cancer	2/316	97/8577	0.877930352675206	0.999992176891026	0.869876235745849	GSTP1/CCND1	2
hsa04340	Hedgehog signaling pathway	1/316	56/8577	0.878647731730555	0.999992176891026	0.869876235745849	CCND1	1

hsa05231	Choline metabolism in cancer	2/316	98/8577	0.881510980981987	0.999992176891026	0.869876235745849	HIF1A/PLD1	2
hsa00240	Pyrimidine metabolism	1/316	58/8577	0.887482007406155	0.999992176891026	0.869876235745849	CMPK1	1
hsa04923	Regulation of lipolysis in adipocytes	1/316	58/8577	0.887482007406155	0.999992176891026	0.869876235745849	MGLL	1
hsa04730	Long-term depression	1/316	60/8577	0.895675046948846	0.999992176891026	0.869876235745849	GUCY1A1	1
hsa04974	Protein digestion and absorption	2/316	103/8577	0.898003048833321	0.999992176891026	0.869876235745849	ATP1B1/PRSS3	2
hsa04213	Longevity regulating pathway - multiple species	1/316	61/8577	0.899545739113242	0.999992176891026	0.869876235745849	HDAC1	1
hsa04620	Toll-like receptor signaling pathway	2/316	104/8577	0.901034597150939	0.999992176891026	0.869876235745849	MAPK13/TOLLIP	2
hsa04550	Signaling pathways regulating pluripotency of stem cells	3/316	143/8577	0.902464153242593	0.999992176891026	0.869876235745849	MAPK13/SOX2/KLF4	3

hsa00310	Lysine degradation	1/316	63/8577	0.906862881750599	0.999992176891026	0.869876235745849	BBOX1	1
hsa04350	TGF-beta signaling pathway	2/316	108/8577	0.912341065266012	0.999992176891026	0.869876235745849	CDKN2B/HDAC1	2
hsa04659	Th17 cell differentiation	2/316	108/8577	0.912341065266012	0.999992176891026	0.869876235745849	HIF1A/MAPK13	2
hsa00970	Aminoacyl-tRNA biosynthesis	1/316	66/8577	0.916854317811685	0.999992176891026	0.869876235745849	FARSB	1
hsa05145	Toxoplasmosis	2/316	111/8577	0.920015553962369	0.999992176891026	0.869876235745849	CYCS/MAPK13	2
hsa04664	Fc epsilon RI signaling pathway	1/316	68/8577	0.922914185096478	0.999992176891026	0.869876235745849	MAPK13	1
hsa04920	Adipocytokine signaling pathway	1/316	69/8577	0.925776932482718	0.999992176891026	0.869876235745849	ACSL1	1
hsa04724	Glutamatergic synapse	2/316	115/8577	0.929270240240007	0.999992176891026	0.869876235745849	GNG5/PLD1	2
hsa04726	Serotonergic synapse	2/316	115/8577	0.929270240240007	0.999992176891026	0.869876235745849	CYP2C18/GNG5	2

hsa05202	Transcriptional misregulation in cancer	4/316	193/8577	0.929750920863947	0.999992176891026	0.869876235745849	JUP/NUPR1/HPGD/HDAC1	4
hsa03082	ATP-dependent chromatin remodeling	2/316	117/8577	0.933509946387751	0.999992176891026	0.869876235745849	ACTB/HDAC1	2
hsa04137	Mitophagy - animal	1/316	72/8577	0.933744737652387	0.999992176891026	0.869876235745849	HIF1A	1
hsa05223	Non-small cell lung cancer	1/316	72/8577	0.933744737652387	0.999992176891026	0.869876235745849	CCND1	1
hsa05171	Coronavirus disease - COVID-19	5/316	232/8577	0.933930610370487	0.999992176891026	0.869876235745849	HBEGF/MAPK13/CASP1/RPS26/RPL22L1	5
hsa00562	Inositol phosphate metabolism	1/316	73/8577	0.936206426412157	0.999992176891026	0.869876235745849	TPI1	1
hsa04935	Growth hormone synthesis, secretion and action	2/316	120/8577	0.939421923448162	0.999992176891026	0.869876235745849	MAPK13/SOCS2	2
hsa03008	Ribosome biogenesis in eukaryotes	3/316	164/8577	0.944746040713779	0.999992176891026	0.869876235745849	EIF6/NOP10/REXO2	3

hsa05140	Leishmaniasis	1/316	77/8577	0.945174395080546	0.999992176891026	0.869876235745849	MAPK13	1
hsa04612	Antigen processing and presentation	1/316	78/8577	0.947212617569316	0.999992176891026	0.869876235745849	LGMN	1
hsa01521	EGFR tyrosine kinase inhibitor resistance	1/316	79/8577	0.949175297043148	0.999992176891026	0.869876235745849	EIF4E2	1
hsa03018	RNA degradation	1/316	79/8577	0.949175297043148	0.999992176891026	0.869876235745849	ENO1	1
hsa04926	Relaxin signaling pathway	2/316	129/8577	0.954310517648017	0.999992176891026	0.869876235745849	MAPK13/GNG5	2
hsa03083	Polycomb repressive complex	1/316	84/8577	0.957949724413126	0.999992176891026	0.869876235745849	HDAC1	1
hsa04151	PI3K-Akt signaling pathway	8/316	359/8577	0.958103542233488	0.999992176891026	0.869876235745849	YWHAZ/SGK1/YWHAB/GNG5/NTRK2/CCND1/EIF4E2/YWHAQ	8
hsa04650	Natural killer cell mediated cytotoxicity	2/316	132/8577	0.958445877961789	0.999992176891026	0.869876235745849	RAET1L/PAK1	2
hsa04742	Taste transduction	1/316	86/8577	0.961020827298434	0.999992176891026	0.869876235745849	SCN9A	1

hsa04911	Insulin secretion	1/316	86/8577	0.961020827298434	0.999992176891026	0.869876235745849	ATP1B1	1
hsa04211	Longevity regulating pathway	1/316	89/8577	0.965213282824707	0.999992176891026	0.869876235745849	EIF4E2	1
hsa04727	GABAergic synapse	1/316	89/8577	0.965213282824707	0.999992176891026	0.869876235745849	GNG5	1
hsa05162	Measles	2/316	138/8577	0.965668453436952	0.999992176891026	0.869876235745849	CYCS/CCND1	2
hsa05410	Hypertrophic cardiomyopathy	1/316	90/8577	0.966508358534814	0.999992176891026	0.869876235745849	ACTB	1
hsa05032	Morphine addiction	1/316	91/8577	0.967755366747728	0.999992176891026	0.869876235745849	GNG5	1
hsa04140	Autophagy - animal	2/316	141/8577	0.968812114678412	0.999992176891026	0.869876235745849	HIF1A/VAMP8	2
hsa04658	Th1 and Th2 cell differentiation	1/316	92/8577	0.968956086062802	0.999992176891026	0.869876235745849	MAPK13	1
hsa04657	IL-17 signaling pathway	1/316	94/8577	0.971225446759225	0.999992176891026	0.869876235745849	MAPK13	1

hsa 054 14	Dilated cardiomyopath y	1/316	96/85 77	0.97332 940003 1904	0.99999 217689 1026	0.86987 623574 5849	ACTB	1
hsa 052 24	Breast cancer	2/316	147/8 577	0.97428 996646 947	0.99999 217689 1026	0.86987 623574 5849	HES1/CCND1	2
hsa 030 15	mRNA surveillance pathway	1/316	97/85 77	0.97432 314010 8536	0.99999 217689 1026	0.86987 623574 5849	PYM1	1
hsa 040 62	Chemokine signaling pathway	3/316	192/8 577	0.97507 846752 9997	0.99999 217689 1026	0.86987 623574 5849	PAK1/CDC42/GNG5	3
hsa 005 64	Glycerophosph olipid metabolism	1/316	99/85 77	0.97620 124619 2753	0.99999 217689 1026	0.86987 623574 5849	PLD1	1
hsa 049 14	Progesterone-mediated oocyte maturation	1/316	102/8 577	0.97876 472937 9749	0.99999 217689 1026	0.86987 623574 5849	MAPK13	1
hsa 051 42	Chagas disease	1/316	102/8 577	0.97876 472937 9749	0.99999 217689 1026	0.86987 623574 5849	MAPK13	1
hsa 049 34	Cushing syndrome	2/316	155/8 577	0.98016 914882 7593	0.99999 217689 1026	0.86987 623574 5849	CDKN2B/CCND1	2
hsa 041 50	mTOR signaling pathway	2/316	156/8 577	0.98080 553133 2713	0.99999 217689 1026	0.86987 623574 5849	SGK1/EIF4E2	2

hsa03013	Nucleocytoplasmic transport	1/316	108/8577	0.983095147963177	0.999992176891026	0.869876235745849	PYM1	1
hsa04725	Cholinergic synapse	1/316	113/8577	0.986022868912 75	0.999992176891026	0.869876235745849	GNG5	1
hsa04630	JAK-STAT signaling pathway	2/316	166/8577	0.98617336086 32	0.999992176891026	0.869876235745849	CCND1/SOCS2	2
hsa04668	TNF signaling pathway	1/316	114/8577	0.986544699421206	0.999992176891026	0.869876235745849	MAPK13	1
hsa04010	MAPK signaling pathway	5/316	301/8577	0.988126809305252	0.999992176891026	0.869876235745849	HSPB1/PAK1/MAPK13/CDC42/NTRK2	5
hsa04310	Wnt signaling pathway	2/316	171/8577	0.988279051591811	0.999992176891026	0.869876235745849	CTNNBIP1/CCND1	2
hsa04380	Osteoclast differentiation	1/316	128/8577	0.992105232705648	0.999992176891026	0.869876235745849	MAPK13	1
hsa04120	Ubiquitin mediated proteolysis	1/316	142/8577	0.995371994528234	0.999992176891026	0.869876235745849	ELOB	1
hsa05206	MicroRNAs in cancer	4/316	310/8577	0.997202417003729	0.999992176891026	0.869876235745849	SERPINB5/CD44/CCND1/HDAC1	4

hsa03040	Spliceosome	2/316	216/8577	0.997430808426411	0.999992176891026	0.869876235745849	PRPF4/SF3B6	2
hsa04080	Neuroactive ligand-receptor interaction	4/316	367/8577	0.999509954043236	0.999992176891026	0.869876235745849	NTS/TSPO/F2RL1/PRSS3	4
hsa04060	Cytokine-cytokine receptor interaction	1/316	297/8577	0.999988227183661	0.999992176891026	0.869876235745849	CXCL17	1
hsa04740	Olfactory transduction	3/316	439/8577	0.999992176891026	0.999992176891026	0.869876235745849	CALML3/CALML5/CALM1	3

2
