# Supplementary material for: Multiomics Reveals IL-17 Drives Epithelial Keratinization and Proliferation via EHF in Odontogenic Keratocysts
Source: Int J Mol Sci. 2026 May 4;27(9):4115. doi: 10.3390/ijms27094115 (PMC13163638; doi:10.3390/ijms27094115)
Supplement: Supplementary file 1 [file ijms-27-04115-s001.zip › ijms-4235677-supplementary/Supplementary Table S12.pdf]

Supplementary Table S12. EpC5 KEGG enrichment.

ID	Description	GeneRatio	BgRatio	pvalue	p.adjust	qvalue	geneID	Count
hsa04657	IL-17 signaling pathway	18/262	94/8577	3.37014689718496e-10	9.30160543623049e-08	6.70481856387323e-08	CXCL1/MMP13/S100A9/S100A8/CXCL8/CSF3/CXCL3/CXCL2/CXCL6/CCL20/FOSB/NFKBIA/MMP1/TNFAIP3/IL1B/JUN/FOS/HSP90AA1	18
hsa05145	Toxoplasmosis	18/262	111/8577	5.58658443089631e-09	7.70948651463691e-07	5.55718135494422e-07	LAMB3/LAMA3/BIRC3/LAMC2/ITGA6/NFKBIA/HSPA6/STAT1/HLA-DRA/HLA-DRB1/IFNGR1/LAMB1/JAK1/TGFB3/TGFB2/HSPA1A/BIRC2/STAT3	18
hsa04612	Antigen processing and presentation	14/262	78/8577	7.75785534754475e-08	7.13722691974117e-06	5.14468301995073e-06	B2M/CD74/HLA-B/HLA-C/CTSB/TAPBP/HSPA6/HLA-DRA/HLA-DRB1/HLA-A/HSPA5/HSP90AA1/HSPA1A/HLA-E	14
hsa05323	Rheumatoid arthritis	15/262	93/8577	1.16613207418436e-07	8.0463113118721e-06	5.79997268475906e-06	CXCL1/CXCL8/CXCL3/CXCL2/CXCL6/CCL20/MMP1/HLA-DRA/HLA-DRB1/IL1B/JUN/FOS/TGFB3/TGFB2/IL1A	15
hsa04659	Th17 cell differentiation	16/262	108/8577	1.49160847916448e-07	8.23367880498792e-06	5.93503163288603e-06	NFKBIA/STAT1/HLA-DRA/HLA-DRB1/IFNGR1/HIF1A/TGFBR2/IL1B/RUNX1/JUN/JAK1/FOS/HSP90AA1/IL6ST/PPP3CA/STAT3	16
hsa05167	Kaposi sarcoma-associated herpesvirus infection	21/262	194/8577	4.26048113918312e-07	1.85058888090852e-05	1.33394850683337e-05	CLEC2B/CXCL1/CXCL8/CXCL3/HLA-B/CXCL2/HLA-C/NFKBIA/STAT1/IFNGR1/HLA-A/HIF1A/ZFP36/JUN/JAK1/FOS/PIK3R1/IL6ST/PPP3CA/HLA-E/STAT3	21
hsa04940	Type I diabetes mellitus	10/262	43/8577	4.69352252404334e-07	1.85058888090852e-05	1.33394850683337e-05	GAD2/HLA-B/HLA-C/CPE/HLA-DRA/HLA-DRB1/HLA-A/IL1B/IL1A/HLA-E	10
hsa05417	Lipid and atherosclerosis	22/262	215/8577	5.96884236691193e-07	2.05925061658462e-05	1.48435685177152e-05	CXCL1/TNFSF10/CXCL8/SOD2/CXCL3/CXCL2/NFKBIA/MMP1/HSPA6/HSPA5/IL1B/XBP1/JUN/FOS/VAV3/PIK3R1/HSP90AA1/HSPA1A/PPP3CA/ATF6/STAT3/ABCA1	22
hsa05169	Epstein-Barr virus infection	21/262	202/8577	8.36872354668856e-07	2.56640855431782e-05	1.84992836295221e-05	B2M/HLA-B/HLA-C/VIM/NFKBIA/TAPBP/STAT1/HLA-DRA/HLA-DRB1/GADD45B/HLA-	21

							A/TNFAIP3/GADD45A/JUN/JAK1/CD58/ISG15/PIK3R1/OAS2/HLA-E/STAT3	
hsa05146	Amoebiasis	14/262	102/8577	2.33810901661105e-06	6.4531808858465e-05	4.65160635936304e-05	CXCL1/CXCL8/LAMB3/CXCL3/LAMA3/LAMC2/CXCL2/LAMB1/IL1B/RAB7B/COL1A1/TGFB3/TGFB2/PIK3R1	14
hsa05140	Leishmaniasis	12/262	77/8577	3.22032764296803e-06	7.27248539577366e-05	5.24218054844097e-05	NFKBIA/STAT1/HLA-DRA/HLA-DRB1/IFNGR1/IL1B/JUN/JAK1/FOS/TGFB3/TGFB2/IL1A	12
hsa04210	Apoptosis	16/262	136/8577	3.55690387483911e-06	7.27248539577366e-05	5.24218054844097e-05	TNFSF10/BIRC3/CTSB/NFKBIA/GADD45B/GADD45A/CTSD/PTPN13/PARP4/JUN/FOS/CAPN2/PIK3R1/MCL1/CTSV/BIRC2	16
hsa05321	Inflammatory bowel disease	11/262	65/8577	3.63109075101028e-06	7.27248539577366e-05	5.24218054844097e-05	STAT1/HLA-DRA/HLA-DRB1/IFNGR1/MAF/IL1B/JUN/TGFB3/TGFB2/IL1A/STAT3	11
hsa04621	NOD-like receptor signaling pathway	19/262	186/8577	3.68894186742142e-06	7.27248539577366e-05	5.24218054844097e-05	CXCL1/CXCL8/CXCL3/IFI16/BIRC3/TXNIP/CXCL2/CTSB/NFKBIA/STAT1/TNFAIP3/IL1B/JUN/JAK1/HSP90AA1/OAS2/GBP3/BIRC2/GBP1	19
hsa05162	Measles	16/262	138/8577	4.31256307751561e-06	7.93511606262871e-05	5.71982050281017e-05	NFKBIA/HSPA6/STAT1/TNFAIP3/IL1B/CD46/MX1/JUN/MSN/JAK1/FOS/PIK3R1/IL1A/OAS2/HSPA1A/STAT3	16
hsa04218	Cellular senescence	17/262	156/8577	5.06746622324935e-06	8.74137923510514e-05	6.30099418548769e-05	CXCL8/HLA-B/HLA-C/SERPINE1/GADD45B/HLA-A/TGFBR2/GADD45A/IGFBP3/TGFB3/CAPN2/TGFB2/PIK3R1/IL1A/ZFP36L1/PPP3CA/HLA-E	17
hsa04933	AGE-RAGE signaling pathway in diabetic complications	13/262	100/8577	9.93425436237054e-06	0.000160897117062423	0.000115978471109069	CXCL8/SERPINE1/EGR1/STAT1/TGFBR2/IL1B/JUN/COL1A1/TGFB3/TGFB2/PIK3R1/IL1A/STAT3	13

hsa04610	Complement and coagulation cascades	12/262	86/8577	1.04932902432015e-05	0.000160897117062423	0.000115978471109069	CLU/CFH/PLAUR/C1R/SERPINE1/CD59/CD46/SERPINE2/C1S/PROS1/SERPING1/PLAU	12
hsa05166	Human T-cell leukemia virus 1 infection	20/262	222/8577	1.37988088980199e-05	0.000200445855571237	0.000144486143031899	MMP7/B2M/HLA-B/HLA-C/ETS2/NFKBIA/EGR1/HLA-DRA/HLA-DRB1/HLA-A/TGFBR2/ZFP36/JUN/JAK1/FOS/TGFB3/TGFB2/PIK3R1/PPP3CA/HLA-E	20
hsa05222	Small cell lung cancer	12/262	92/8577	2.1158711253037e-05	0.000291990215291911	0.000210473496148631	LAMB3/LAMA3/BIRC3/LAMC2/ITGA6/NFKBIA/GADD45B/LAMB1/ITGAV/GADD45A/PIK3R1/BIRC2	12
hsa05332	Graft-versus-host disease	8/262	42/8577	3.26331940217818e-05	0.000421967844396645	0.000304164464496437	HLA-B/HLA-C/HLA-DRA/HLA-DRB1/HLA-A/IL1B/IL1A/HLA-E	8
hsa04380	Osteoclast differentiation	14/262	128/8577	3.36351180316166e-05	0.000421967844396645	0.000304164464496437	FOSB/NFKBIA/STAT1/IFNGR1/TGFBR2/IL1B/JUN/FOSL2/JAK1/FOS/TGFB2/PIK3R1/IL1A/PPP3CA	14
hsa04060	Cytokine-cytokine receptor interaction	23/262	297/8577	3.66030123915057e-05	0.000439236148698068	0.000316611869198836	CXCL1/TNFSF10/CXCL8/CSF3/CXCL3/TNFRSF21/CXCL2/CXCL6/CCL20/IL1RN/INHBA/BMP2/IFNGR1/IL20RB/TGFBR2/IL1B/TGFB3/IL13RA1/TGFB2/IL1A/IL6ST/CXCL16/CXCL17	23
hsa04668	TNF signaling pathway	13/262	114/8577	4.13645467716759e-05	0.000475692287874272	0.000342890321923103	CXCL1/CXCL3/BIRC3/CXCL2/CXCL6/CCL20/NFKBIA/TNFAIP3/IL1B/JUN/FOS/PIK3R1/BIRC2	13

hsa05205	Proteoglycans in cancer	18/262	205/8577	5.27206577127605e-05	0.000582036061148875	0.000419545444535231	DCN/LUM/PLAUR/CAV1/WNT5A/HIF1A/ITGAV/HBEGF/MSN/COL1A1/CD63/TGFB2/VAV3/PIK3R1/RDX/STAT3/WNT2B/PLAU	18
hsa05164	Influenza A	16/262	171/8577	6.46474679073183e-05	0.000686257736246917	0.000494670908278670	TNFSF10/CXCL8/NFKBIA/STAT1/HLA-DRA/HLA-DRB1/RSAD2/IFNGR1/IL1B/MX1/JAK1/DNAJB1/PIK3R1/IL1A/OAS2/DNAJC3	16
hsa04064	NF-kappa B signaling pathway	12/262	104/8577	7.28148376836164e-05	0.000744329451876967	0.000536530382931910	CXCL1/CXCL8/CXCL3/BIRC3/CXCL2/NFKBIA/GADD45B/TNFAIP3/IL1B/GADD45A/BIRC2/PLAU	12
hsa05171	Coronavirus disease - COVID-19	19/262	232/8577	8.44194996651593e-05	0.000832135068127999	0.000599822760778764	CXCL8/CSF3/NFKBIA/C1R/MMP1/STAT1/HBEGF/IL1B/RPS4Y1/MX1/JUN/JAK1/C1S/FOS/ISG15/PIK3R1/OAS2/IL6ST/STAT3	19
hsa05212	Pancreatic cancer	10/262	76/8577	9.61612958063372e-05	0.000915190263536175	0.000659690922228593	STAT1/GADD45B/TGFBR2/GADD45A/JAK1/PLD1/TGFB3/TGFB2/PIK3R1/STAT3	10
hsa04510	Focal adhesion	17/262	203/8577	0.000152570550906077	0.00140364906833591	0.00101178365337714	LAMB3/LAMA3/BIRC3/LAMC2/CAV1/ITGA6/COL6A6/LAMB1/ITGAV/MYL9/PDGFD/JUN/COL1A1/CAPN2/VAV3/PIK3R1/BIRC2	17
hsa04141	Protein processing in endoplasmic reticulum	15/262	170/8577	0.000210802408783553	0.00187682144594389	0.0013528575640099	CRYAB/HSPA6/HSPA5/XBP1/HSPH1/LMAN1/CAPN2/TUSC3/DNAJB1/HSP90AA1/HSPA1A/DNAJC3/SSR1/ATF6/SSR3	15

hsa05142	Chagas disease	11/262	102/8577	0.000266206243925568	0.0022695526190696	0.00163594754006161	CXCL8/NFKBIA/SERPINE1/IFNGR1/TGFBR2/IL1B/JUN/FOS/TGFB3/TGFB2/PIK3R1	11
hsa05134	Legionellosis	8/262	56/8577	0.000271359552280061	0.0022695526190696	0.00163594754006161	CXCL1/CXCL8/CXCL3/CXCL2/NFKBIA/HSPA6/IL1B/HSPA1A	8
hsa04514	Cell adhesion molecules	14/262	158/8577	0.000327282828734375	0.00265676649207904	0.00191506051488535	CLDN1/HLA-B/HLA-C/ITGA6/HLA-DRA/NRCAM/HLA-DRB1/HLA-A/ITGAV/CDH3/CDH2/CD58/HLA-E/MPZL1	14
hsa05235	PD-L1 expression and PD-1 checkpoint pathway in cancer	10/262	89/8577	0.000361643306039841	0.00285181578477131	0.0020556566869633	NFKBIA/STAT1/IFNGR1/HIF1A/JUN/JAK1/FOS/PIK3R1/PPP3CA/STAT3	10
hsa04350	TGF-beta signaling pathway	11/262	108/8577	0.000438670305333298	0.00336313900755528	0.00242423063473665	DCN/ID2/PITX2/INHBA/BMP2/TFRC/ID1/TGFBR2/TGFB3/SKIL/TGFB2	11
hsa04658	Th1 and Th2 cell differentiation	10/262	92/8577	0.000473288364280404	0.00348740862201537	0.00251380713028568	NFKBIA/STAT1/HLA-DRA/HLA-DRB1/IFNGR1/MAF/JUN/JAK1/FOS/PPP3CA	10
hsa05133	Pertussis	9/262	76/8577	0.000480150462451391	0.00348740862201537	0.00251380713028568	CXCL8/CXCL6/C1R/IL1B/JUN/C1S/FOS/SERPING1/IL1A	9

hsa05202	Transcriptional misregulation in cancer	15/262	193/8577	0.000816873359184897	0.00578094992653927	0.00416704628572053	CXCL8/BIRC3/TSPAN7/ID2/NFKBIZ/GADD45B/MAF/TGFBR2/RUNX1/GADD45A/RUNX2/PBX1/IGFBP3/BIRC2/PLAU	15
hsa05330	Allograft rejection	6/262	38/8577	0.000930679330910863	0.00642168738328496	0.00462890509321456	HLA-B/HLA-C/HLA-DRA/HLA-DRB1/HLA-A/HLA-E	6
hsa04915	Estrogen signaling pathway	12/262	137/8577	0.000959937593884853	0.00646201892468828	0.00465797702809338	KRT15/HSPA6/KRT14/HBEGF/FKBP5/CTSD/KRT19/JUN/FOS/PIK3R1/HSP90AA1/HSPA1A	12
hsa04390	Hippo signaling pathway	13/262	157/8577	0.00100909840257342	0.00663121807405387	0.00477993980166355	BIRC3/ID2/DLG2/SERPINE1/BMP2/WNT5A/ID1/TGFBR2/AREG/TGFB3/TGFB2/BIRC2/WNT2B	13
hsa04217	Necroptosis	13/262	159/8577	0.00113442270676946	0.00721969598420123	0.00520412868426404	TNFSF10/BIRC3/STAT1/IFNGR1/TNFAIP3/IL1B/JAK1/CAPN2/HSP90AA1/IL1A/BIRC2/STAT3/PYGL	13
hsa05165	Human papillomavirus infection	21/262	331/8577	0.00118773585375034	0.00721969598420123	0.00520412868426404	LAMB3/LAMA3/LAMC2/HLA-B/HLA-C/DLG2/ITGA6/STAT1/WNT5A/COL6A6/LAMB1/HLA-A/ITGAV/MX1/PKM/JAK1/COL1A1/ISG15/PIK3R1/HLA-E/WNT2B	21
hsa05210	Colorectal cancer	9/262	86/8577	0.00118897544740234	0.00721969598420123	0.00520412868426404	GADD45B/TGFBR2/GADD45A/AREG/JUN/FOS/TGFB3/TGFB2/PIK3R1	9
hsa05152	Tuberculosis	14/262	180/8577	0.00120328266403354	0.00721969598420123	0.00520412868426404	CD74/CLEC7A/STAT1/HLA-DRA/HLA-DRB1/IFNGR1/IL1B/CTSD/JAK1/TGFB3/TGFB2/IL1A/PPP3CA/LAMP2	14

hsa04216	Ferroptosis	6/262	41/8577	0.00140416120978725	0.00824571263619747	0.00594370590481053	GCLC/PRNP/SLC7A11/SAT1/TFRC/SLC39A14	6
hsa04512	ECM-receptor interaction	9/262	89/8577	0.0015180434112916	0.00872874961492669	0.00629189045469544	LAMB3/LAMA3/LAMC2/ITGA6/CD47/COL6A6/LAMB1/ITGAV/COL1A1	9
hsa05220	Chronic myeloid leukemia	8/262	76/8577	0.00213046796004262	0.0120001868769747	0.00865002028889483	NFKBIA/GADD45B/TGFBR2/RUNX1/GADD45A/TGFB3/TGFB2/PIK3R1	8
hsa04068	FoxO signaling pathway	11/262	131/8577	0.0021768880119147	0.0120067632827122	0.00865476071865983	TNFSF10/SOD2/SGK1/GADD45B/TGFBR2/GADD45A/TGFB3/TGFB2/PIK3R1/STAT3/FBXO32	11
hsa05416	Viral myocarditis	7/262	60/8577	0.00221864104137073	0.0120067632827122	0.00865476071865983	HLA-B/HLA-C/CAV1/HLA-DRA/HLA-DRB1/HLA-A/HLA-E	7
hsa04142	Lysosome	11/262	132/8577	0.00231209204842467	0.0122129933761761	0.00880341627802168	NCOA7/CTSB/NPC2/CTSD/LAPTM4B/LAPTM4A/CD63/GM2A/FUCA1/CTSV/LAMP2	11
hsa04145	Phagosome	12/262	152/8577	0.00234524872803382	0.0122129933761761	0.00880341627802168	HLA-B/HLA-C/C1R/CLEC7A/HLA-DRA/TFRC/HLA-DRB1/HLA-A/ITGAV/RAB7B/HLA-E/LAMP2	12
hsa04640	Hematopoietic cell lineage	9/262	99/8577	0.00317481074875156	0.0162268104936191	0.011696671179611	CSF3/ITGA6/HLA-DRA/TFRC/HLA-DRB1/MME/CD59/IL1B/IL1A	9
hsa04061	Viral protein interaction with cytokine and cytokine receptor	9/262	100/8577	0.0033980474394714	0.0170520198780747	0.0122915017427769	CXCL1/TNFSF10/CXCL8/CXCL3/CXCL2/CXCL6/CCL20/IL20RB/IL6ST	9

hsa05418	Fluid shear stress and atherosclerosis	11/262	139/8577	0.00346101575830038	0.0170578633801947	0.0122957138781724	ASS1/CAV1/DUSP1/ITGAV/IL1B/NPPC/JUN/FOS/PIK3R1/HSP90AA1/IL1A	11
hsa04974	Protein digestion and absorption	9/262	103/8577	0.00414257940181594	0.0200588055245824	0.0144588643941498	COL17A1/ATP1A1/COL6A6/COL14A1/MME/COL1A1/SLC38A2/COL12A1/ATP1B1	9
hsa04928	Parathyroid hormone synthesis, secretion and action	9/262	106/8577	0.00500896473088673	0.0238357632021507	0.017181385374548	MMP13/CYP24A1/PTHLH/EGR1/HBEGF/NR4A2/RUNX2/PLD1/FOS	9
hsa05320	Autoimmune thyroid disease	6/262	53/8577	0.0052860851988645	0.0244784758849104	0.0176446679719606	HLA-B/HLA-C/HLA-DRA/HLA-DRB1/HLA-A/HLA-E	6
hsa05120	Epithelial cell signaling in Helicobacter pylori infection	7/262	70/8577	0.00532140780106747	0.0244784758849104	0.0176446679719606	CXCL1/CXCL8/CXCL3/CXCL2/NFKBIA/HBEGF/JUN	7
hsa05150	Staphylococcus aureus infection	8/262	96/8577	0.00889621253790648	0.0401034726738553	0.0289075375109025	CFH/KRT15/C1R/KRT14/HLA-DRA/HLA-DRB1/KRT19/C1S	8
hsa05163	Human cytomegalovirus infection	14/262	225/8577	0.00900875110789502	0.0401034726738553	0.0289075375109025	CXCL8/B2M/HLA-B/HLA-C/NFKBIA/TAPBP/HLA-A/ITGAV/IL1B/JAK1/PIK3R1/PPP3CA/HLA-E/STAT3	14
hsa04978	Mineral absorption	6/262	60/8577	0.00966010500824049	0.0423204600361012	0.0305055947628647	MT2A/ATP1A1/HEPHL1/MT1E/SLC5A1/ATP1B1	6
hsa05161	Hepatitis B	11/262	162/8577	0.0107214227036306	0.0462361354094072	0.0333281067596413	CXCL8/NFKBIA/STAT1/TGFBR2/JUN/JAK1/FOS/TGFB3/TGFB2/PIK3R1/STAT3	11

hsa04550	Signaling pathways regulating pluripotency of stem cells	10/262	143/8577	0.0120987504787753	0.0513731558791075	0.0370309933682354	ID2/INHBA/WNT5A/ID1/JAK1/SKIL/PIK3R1/IL6ST/STAT3/WNT2B	10
hsa05170	Human immunodeficiency virus 1 infection	13/262	212/8577	0.0130340425732679	0.0545059962154838	0.0392892192399941	B2M/HLA-B/HLA-C/NFKBIA/TAPBP/BST2/APOBEC3A/HLA-A/JUN/FOS/PIK3R1/PPP3CA/HLA-E	13
hsa04062	Chemokine signaling pathway	12/262	192/8577	0.0145811403821406	0.060065593215982	0.0432967090687284	CXCL1/CXCL8/CXCL3/CXCL2/CXCL6/CCL20/NFKBIA/STAT1/VAV3/PIK3R1/STAT3/CXCL16	12
hsa05144	Malaria	5/262	50/8577	0.0176974793905872	0.0718309457617951	0.0517774551829874	CXCL8/CSF3/IL1B/TGFB3/TGFB2	5
hsa04066	HIF-1 signaling pathway	8/262	109/8577	0.018206140616408	0.0727507273402753	0.0524404556342945	SERPINE1/TFRC/IFNGR1/HIF1A/PFKFB3/TIMP1/PIK3R1/STAT3	8
hsa05211	Renal cell carcinoma	6/262	69/8577	0.0184512714268814	0.0727507273402753	0.0524404556342945	EPAS1/HIF1A/JUN/TGFB3/TGFB2/PIK3R1	6
hsa04650	Natural killer cell mediated cytotoxicity	9/262	132/8577	0.0195919540339636	0.0761602720193514	0.0548981365814546	TNFSF10/HLA-B/HLA-C/IFNGR1/HLA-A/VAV3/PIK3R1/PPP3CA/HLA-E	9
hsa05203	Viral carcinogenesis	12/262	204/8577	0.0223972779309538	0.0858562320686564	0.0618872153355304	HLA-B/HLA-C/NFKBIA/HLA-A/JUN/PKM/SP100/JAK1/PIK3R1/IL6ST/HLA-E/STAT3	12
hsa05160	Hepatitis C	10/262	158/8577	0.0228124774464148	0.0862499147289107	0.0621709911661484	CLDN1/NFKBIA/STAT1/RSAD2/IFIT1/MX1/JAK1/PIK3R1/OAS2/STAT3	10

hsa 04960	Aldosterone-regulated sodium reabsorption	4/262	37/8577	0.0254900369156982	0.095070948496388	0.0685294022342385	ATP1A1/SGK1/PIK3R1/ATP1B1	4
hsa 04630	JAK-STAT signaling pathway	10/262	166/8577	0.0307519992497139	0.113167357238947	0.0815737243255569	CSF3/STAT1/IFNGR1/IL20RB/JAK1/IL13RA1/PIK3R1/IL6ST/MCL1/STAT3	10
hsa 04964	Proximal tubule bicarbonate reclamation	3/262	23/8577	0.0317766296053295	0.115399339093039	0.0831825899640898	ATP1A1/CA2/ATP1B1	3
hsa 05219	Bladder cancer	4/262	41/8577	0.0355797919916296	0.127532760905062	0.091928649164976	CXCL8/TYMP/MMP1/HBEGF	4
hsa 04213	Longevity regulating pathway - multiple species	5/262	61/8577	0.0380140018778996	0.134208442125024	0.0967406390603718	SOD2/CRYAB/HSPA6/PIK3R1/HSPA1A	5
hsa 00380	Tryptophan metabolism	4/262	42/8577	0.0384147352459307	0.134208442125024	0.0967406390603718	KYNU/CYP1B1/TDO2/MAOA	4
hsa 04620	Toll-like receptor signaling pathway	7/262	104/8577	0.0396405768732358	0.135071595271767	0.0973628203904038	CXCL8/NFKBIA/STAT1/IL1B/JUN/FOS/PIK3R1	7
hsa 04625	C-type lectin receptor signaling pathway	7/262	104/8577	0.0396405768732358	0.135071595271767	0.0973628203904038	NFKBIA/CLEC7A/STAT1/IL1B/JUN/PIK3R1/PPP3CA	7
hsa 05130	Pathogenic Escherichia coli infection	11/262	198/8577	0.0401566938571407	0.135161554933791	0.0974276654557072	TNFSF10/CXCL8/CLDN1/NFKBIA/MYH10/IL1B/MYO1B/JUN/FOS/MYO6/TMED10	11

hsa04010	MAPK signaling pathway	15/262	301/8577	0.0424846542593384	0.139819674107382	0.100785348612873	DUSP1/HSPA6/GADD45B/TGFBR2/IL1B/GADD45A/PDGFD/AREG/JUN/FOS/TGFB3/TGFB2/IL1A/HSPA1A/PPP3CA	15
hsa05217	Basal cell carcinoma	5/262	63/8577	0.0427903513522379	0.139819674107382	0.100785348612873	BMP2/WNT5A/GADD45B/GADD45A/WNT2B	5
hsa04662	B cell receptor signaling pathway	6/262	84/8577	0.0430604068808968	0.139819674107382	0.100785348612873	NFKBIA/JUN/FOS/VAV3/PIK3R1/PPP3CA	6
hsa04926	Relaxin signaling pathway	8/262	129/8577	0.0437052457706582	0.140263346891188	0.1011051585147 42	MMP13/NFKBIA/MMP1/TGFBR2/JUN/COL1A1/FOS/PIK3R1	8
hsa04151	PI3K-Akt signaling pathway	17/262	359/8577	0.0481437713435571	0.152731964262319	0.110092834651328	CSF3/LAMB3/LAMA3/LAMC2/LPAR6/ITGA6/SGK1/COL6A6/LAMB1/ITGAV/PDGFD/AREG/JAK1/COL1A1/PIK3R1/HSP90AA1/MCL1	17
hsa00514	Other types of O-glycan biosynthesis	4/262	47/8577	0.0544744076414587	0.168931870888119	0.121770112882 74	GALNT1/GALNT11/GALNT5/B4GALT1	4
hsa04973	Carbohydrate digestion and absorption	4/262	47/8577	0.0544744076414587	0.168931870888119	0.121770112882 74	ATP1A1/PIK3R1/SLC5A1/ATP1B1	4
hsa04924	Renin secretion	5/262	69/8577	0.059201105958165	0.179555002686303	0.1294275190988 22	CTSB/CLCA4/KCNMA1/ADRB2/PPP3CA	5
hsa05031	Amphetamine addiction	5/262	69/8577	0.059201105958165	0.179555002686303	0.1294275190988 22	FOSB/JUN/FOS/PPP3CA/MAOA	5

hsa04137	Mitophagy - animal	5/262	72/8577	0.068581083075697	0.205743249227091	0.148304630449734	HIF1A/OPTN/CITED2/RAB7B/JUN	5
hsa04115	p53 signaling pathway	5/262	74/8577	0.075265901635530	0.223369772595766	0.161010247980930	SERPINE1/GADD45B/SHISA5/GADD45A/IGFBP3	5
hsa04530	Tight junction	9/262	170/8577	0.0762146796370448	0.223392047741776	0.161026304436291	CLDN1/DLG2/MYH10/MYL9/RUNX1/CGNL1/JUN/MSN/RDX	9
hsa04919	Thyroid hormone signaling pathway	7/262	121/8577	0.076892190345901	0.223392047741776	0.161026304436291	ATP1A1/STAT1/HIF1A/ITGAV/DIO2/PIK3R1/ATP1B1	7
hsa03320	PPAR signaling pathway	5/262	75/8577	0.0787364808082569	0.223420880923803	0.161047088080087	FABP4/FABP5/MMP1/SLC27A6/CYP27A1	5
hsa05231	Choline metabolism in cancer	6/262	98/8577	0.0789818803487568	0.223420880923803	0.161047088080087	HIF1A/PDGFD/JUN/PLD1/FOS/PIK3R1	6
hsa05132	Salmonella infection	12/262	249/8577	0.0793306026468576	0.223420880923803	0.161047088080087	TNFSF10/CXCL8/BIRC3/NFKBIA/MYL9/IL1B/RAB7B/JUN/FOS/HSP90AA1/MYO6/BIRC2	12
hsa04144	Endocytosis	12/262	250/8577	0.0811752293484746	0.226306700001808	0.163127255150045	HLA-B/HLA-C/CAV1/HSPA6/TFRC/HLA-A/TGFBR2/RAB31/DAB2/PLD1/HSPA1A/HLA-E	12
hsa00430	Taurine and hypotaurine metabolism	2/262	16/8577	0.0842188749954183	0.232444094987355	0.167551235517201	GAD2/FMO2	2

hsa05226	Gastric cancer	8/262	149/8577	0.0858467141157487	0.232519482540842	0.1676055766598 75	WNT5A/GADD45B/TGFBR2/GADD45A/TGFB3/TGFB2/PIK3R1/WNT2B	8
hsa05412	Arrhythmogenic right ventricular cardiomyopathy	5/262	77/8577	0.0859311131129199	0.232519482540842	0.167605576659875	ITGA6/GJA1/ITGAV/CDH2/DSG2	5
hsa00512	Mucin type O-glycan biosynthesis	3/262	36/8577	0.0962764505818035	0.257983498646386	0.185960645477372	GALNT1/GALNT11/GALNT5	3
hsa04931	Insulin resistance	6/262	108/8577	0.11250244830714	0.298564189738179	0.215212173381068	NFKBIA/PTPN1/SLC27A6/PIK3R1/STAT3/PYGL	6
hsa05135	Yersinia infection	7/262	137/8577	0.125910901919817	0.330965799332091	0.238568024690 18	CXCL8/NFKBIA/IL1B/JUN/FOS/VAV3/PIK3R1	7
hsa00770	Pantothenate and CoA biosynthesis	2/262	21/8577	0.133643983903128	0.347978675068521	0.250831310404083	DPYD/VNN1	2
hsa04140	Autophagy - animal	7/262	141/8577	0.140184927749703	0.361598505223533	0.260648808113073	CTSB/HIF1A/RAB7B/CTSD/PIK3R1/VMP1/LAMP2	7
hsa05225	Hepatocellular carcinoma	8/262	168/8577	0.142227801938611	0.363471049398672	0.261998582518494	WNT5A/GADD45B/TGFBR2/GADD45A/TGFB3/TGFB2/PIK3R1/WNT2B	8
hsa04024	cAMP signaling pathway	10/262	225/8577	0.150808155918017	0.381862853517 18	0.2752558326268	NFKBIA/ATP1A1/MYL9/JUN/PLD1/ADRB2/FOS/VAV3/PIK3R1/ATP1B1	10

hsa04970	Salivary secretion	5/262	93/8577	0.154827828976086	0.388477098158179	0.280023537573973	ATP1A1/KCNMA1/CST3/ADRB2/ATP1B1	5
hsa05224	Breast cancer	7/262	147/8577	0.162989078994941	0.400294307730541	0.288541663467095	WNT5A/GADD45B/GADD45A/JUN/FOS/PIK3R1/WNT2B	7
hsa04810	Regulation of actin cytoskeleton	10/262	229/8577	0.163037000112008	0.400294307730541	0.288541663467095	ITGA6/IQGAP2/MYH10/ITGAV/MYL9/PDGFD/MSN/VAV3/PIK3R1/RDX	10
hsa04660	T cell receptor signaling pathway	6/262	121/8577	0.165086336571104	0.400294307730541	0.288541663467095	NFKBIA/JUN/FOS/VAV3/PIK3R1/PPP3CA	6
hsa04917	Prolactin signaling pathway	4/262	70/8577	0.165338953193049	0.400294307730541	0.288541663467095	STAT1/FOS/PIK3R1/STAT3	4
hsa05215	Prostate cancer	5/262	97/8577	0.174846909647993	0.418364351074441	0.30156698075 16	NFKBIA/PDGFD/PIK3R1/HSP90AA1/PLAU	5
hsa05218	Melanoma	4/262	72/8577	0.177350105346774	0.418364351074441	0.30156698075 16	GADD45B/GADD45A/PDGFD/PIK3R1	4
hsa05223	Non-small cell lung cancer	4/262	72/8577	0.177350105346774	0.418364351074441	0.30156698075 16	GADD45B/GADD45A/PIK3R1/STAT3	4
hsa05030	Cocaine addiction	3/262	49/8577	0.187962552532198	0.439641224566836	0.316903857525294	FOSB/JUN/MAOA	3

hsa00330	Arginine and proline metabolism	3/262	50/8577	0.195772690686163	0.450291534646745	0.324580854493649	SAT1/P4HA2/MAOA	3
hsa04932	Non-alcoholic fatty liver disease	7/262	155/8577	0.195778928107 28	0.450291534646745	0.324580854493649	CXCL8/IL1B/XBP1/JUN/FOS/PIK3R1/IL1A	7
hsa04972	Pancreatic secretion	5/262	102/8577	0.201164877982756	0.456995730767798	0.329413398608367	ATP1A1/CLCA4/KCNMA1/CA2/ATP1B1	5
hsa04971	Gastric acid secretion	4/262	76/8577	0.202206846358476	0.456995730767798	0.329413398608367	ATP1A1/CA2/KCNJ15/ATP1B1	4
hsa04979	Cholesterol metabolism	3/262	51/8577	0.203661140885649	0.456995730767798	0.329413398608367	NPC2/CYP27A1/ABCA1	3
hsa01521	EGFR tyrosine kinase inhibitor resistance	4/262	79/8577	0.221488987405286	0.492991617127895	0.355360090149398	PDGFD/JAK1/PIK3R1/STAT3	4
hsa05208	Chemical carcinogenesis - reactive oxygen species	9/262	223/8577	0.242310258930821	0.531011546907272	0.38276576035 65	SOD2/NFKBIA/CYP1B1/HIF1A/PTPN1/JUN/PLD1/FOS/PIK3R1	9
hsa00410	beta-Alanine metabolism	2/262	31/8577	0.244342269772549	0.531011546907272	0.38276576035 65	GAD2/DPYD	2
hsa05310	Asthma	2/262	31/8577	0.244342269772549	0.531011546907272	0.38276576035 65	HLA-DRA/HLA-DRB1	2

hsa04022	cGMP-PKG signaling pathway	7/262	167/8577	0.249283524879099	0.537517600520557	0.387455478636099	ATP1A1/MYL9/KCNMA1/NPPC/ADRB2/PPP3CA/ATP1B1	7
hsa00052	Galactose metabolism	2/262	32/8577	0.255766752762305	0.542374992464772	0.390956802348749	AKR1B1/B4GALT1	2
hsa04215	Apoptosis - multiple species	2/262	32/8577	0.255766752762305	0.542374992464772	0.390956802348749	BIRC3/BIRC2	2
hsa04923	Regulation of lipolysis in adipocytes	3/262	58/8577	0.26060156758865	0.542374992464772	0.390956802348749	FABP4/ADRB2/PIK3R1	3
hsa05213	Endometrial cancer	3/262	58/8577	0.26060156758865	0.542374992464772	0.390956802348749	GADD45B/GADD45A/PIK3R1	3
hsa04012	ErbB signaling pathway	4/262	85/8577	0.261361862310923	0.542374992464772	0.390956802348749	HBEGF/AREG/JUN/PIK3R1	4
hsa04936	Alcoholic liver disease	6/262	142/8577	0.266881680584473	0.549696595830706	0.396234388299021	CXCL1/CXCL8/CXCL3/CXCL2/NFKBIA/IL1B	6
hsa04670	Leukocyte transendothelial migration	5/262	115/8577	0.274858058661678	0.561334885986016	0.404623544818295	CLDN1/MYL9/MSN/VAV3/PIK3R1	5
hsa00051	Fructose and mannose metabolism	2/262	34/8577	0.278633620942334	0.561334885986016	0.404623544818295	PFKFB3/AKR1B1	2

hsa 047 10	Circadian rhythm	2/262	34/8577	0.27863 362094 2334	0.56133 488598 6016	0.40462 354481 8295	BHLHE40/BHLHE41	2
hsa 049 76	Bile secretion	4/262	89/8577	0.28866 891663 1547	0.57733 783326 3094	0.41615 885006 3786	ATP1A1/CA2/SLC5A1/ATP1B1	4
hsa 054 10	Hypertrophic cardiomyopathy	4/262	90/8577	0.29556 122335 0805	0.58686 976722 8937	0.42302 969491 3307	ITGA6/ITGAV/TGFB3/TGFB2	4
hsa 032 50	Viral life cycle - HIV-1	3/262	63/8577	0.30243 363179 6438	0.59622 630268 4406	0.42977 410834 2306	BST2/APOBEC3A/MX1	3
hsa 049 35	Growth hormone synthesis, secretion and action	5/262	120/8577	0.30462 236880 1701	0.59628 208361 184	0.42981 431656 2311	STAT1/IGFBP3/FOS/PIK3R1/STAT3	5
hsa 002 50	Alanine, aspartate and glutamate metabolism	2/262	37/8577	0.31282 041779 9792	0.59957 246744 9601	0.43218 610353 9186	GAD2/ASS1	2
hsa 051 43	African trypanosomiasis	2/262	37/8577	0.31282 041779 9792	0.59957 246744 9601	0.43218 610353 9186	APOL1/IL1B	2
hsa 052 16	Thyroid cancer	2/262	37/8577	0.31282 041779 9792	0.59957 246744 9601	0.43218 610353 9186	GADD45B/GADD45A	2
hsa 049 12	GnRH signaling pathway	4/262	93/8577	0.31635 333763 653	0.60216 221508 7463	0.43405 285526 8995	EGR1/HBEGF/JUN/PLD1	4

hsa 05020	Prion disease	10/262	272/8577	0.319416363258429	0.60382819355703	0.435253732197859	CAV1/PRNP/HSPA6/EGR1/HSPA5/IL1B/PIK3R1/IL1A/HSPA1A/PPP3CA	10
hsa 05221	Acute myeloid leukemia	3/262	67/8577	0.336136507308946	0.628953806113582	0.453364871683703	RUNX1/PIK3R1/STAT3	3
hsa 05414	Dilated cardiomyopathy	4/262	96/8577	0.337265084437718	0.628953806113582	0.453364871683703	ITGA6/ITGAV/TGFB3/TGFB2	4
hsa 00830	Retinol metabolism	3/262	68/8577	0.344559596135417	0.638244621029363	0.460061912183637	SDR16C5/RDH10/DHRS3	3
hsa 01522	Endocrine resistance	4/262	98/8577	0.351240168509253	0.644329997019256	0.464448396020745	HBEGF/JUN/FOS/PIK3R1	4
hsa 00533	Glycosaminoglycan biosynthesis - keratan sulfate	1/262	14/8577	0.352513875180825	0.644329997019256	0.464448396020745	B4GALT1	1
hsa 05230	Central carbon metabolism in cancer	3/262	70/8577	0.361375335053018	0.656181529438374	0.472991262638645	HIF1A/PKM/PIK3R1	3
hsa 00513	Various types of N-glycan biosynthesis	2/262	42/8577	0.368903647058963	0.662687982572219	0.477681268902172	TUSC3/B4GALT1	2
hsa 04622	RIG-I-like receptor signaling pathway	3/262	71/8577	0.369760685927977	0.662687982572219	0.477681268902172	CXCL8/NFKBIA/ISG15	3

hsa01524	Platinum drug resistance	3/262	73/8577	0.386469024082519	0.68816419772113	0.496045131080449	BIRC3/PIK3R1/BIRC2	3
hsa00360	Phenylalanine metabolism	1/262	16/8577	0.391531913421548	0.688889367713141	0.49656785086874	MAOA	1
hsa05010	Alzheimer disease	13/262	384/8577	0.391868227286098	0.688889367713141	0.49656785086874	WNT5A/APP/MME/IL1B/XBP1/SLC39A14/CAPN2/PIK3R1/IL1A/SLC39A6/PPP3CA/ATF6/WNT2B	13
hsa04918	Thyroid hormone synthesis	3/262	75/8577	0.403072525757067	0.698396159731634	0.503420572804267	ATP1A1/HSPA5/ATP1B1	3
hsa05214	Glioma	3/262	75/8577	0.403072525757067	0.698396159731634	0.503420572804267	GADD45B/GADD45A/PIK3R1	3
hsa00120	Primary bile acid biosynthesis	1/262	17/8577	0.410153410756386	0.698396159731634	0.503420572804267	CYP27A1	1
hsa00910	Nitrogen metabolism	1/262	17/8577	0.410153410756386	0.698396159731634	0.503420572804267	CA2	1
hsa03050	Proteasome	2/262	46/8577	0.412458601580639	0.698396159731634	0.503420572804267	PSMB9/PSMB8	2
hsa04930	Type II diabetes mellitus	2/262	46/8577	0.412458601580639	0.698396159731634	0.503420572804267	PKM/PIK3R1	2

hsa05012	Parkinson disease	9/262	266/8577	0.425423403846917	0.715956460132616	0.516078455244334	DUSP1/LRRK2/UBE2L6/HSPA5/XBP1/SLC39A14/SLC39A6/ATF6/MAOA	9
hsa00511	Other glycan degradation	1/262	18/8577	0.428207126455198	0.716273738797785	0.516307157256985	FUCA1	1
hsa04672	Intestinal immune network for IgA production	2/262	49/8577	0.444135083982239	0.738441464934326	0.532286181817649	HLA-DRA/HLA-DRB1	2
hsa00100	Steroid biosynthesis	1/262	20/8577	0.462679681833664	0.764668216683181	0.551191048638907	CYP24A1	1
hsa04724	Glutamatergic synapse	4/262	115/8577	0.468567631272928	0.765234711427978	0.551599391532753	SLC1A3/PLD1/SLC38A2/PPP3CA	4
hsa04726	Serotonergic synapse	4/262	115/8577	0.468567631272928	0.765234711427978	0.551599391532753	DUSP1/APP/CYP4X1/MAOA	4
hsa05207	Chemical carcinogenesis - receptor activation	7/262	212/8577	0.471734456272753	0.765395049342477	0.551714966917346	CYP1B1/JUN/ADRB2/FOS/PIK3R1/HSP90AA1/STAT3	7
hsa04072	Phospholipase D signaling pathway	5/262	148/8577	0.4742121501361	0.765395049342477	0.551714966917346	CXCL8/LPAR6/PDGFD/PLD1/PIK3R1	5
hsa00532	Glycosaminoglycan biosynthesis - chondroitin sulfate / dermatan sulfate	1/262	21/8577	0.479131466730191	0.768838865218213	0.554197351358666	DSE	1

hsa00510	N-Glycan biosynthesis	2/262	53/8577	0.484852774691803	0.769076815028378	0.554368871244712	TUSC3/B4GALT1	2
hsa04961	Endocrine and other factor-regulated calcium reabsorption	2/262	53/8577	0.484852774691803	0.769076815028378	0.554368871244712	ATP1A1/ATP1B1	2
hsa04911	Insulin secretion	3/262	86/8577	0.491521818490267	0.771991322227119	0.556469717394834	ATP1A1/KCNMA1/ATP1B1	3
hsa00220	Arginine biosynthesis	1/262	22/8577	0.495081391428261	0.771991322227119	0.556469717394834	ASS1	1
hsa00340	Histidine metabolism	1/262	22/8577	0.495081391428261	0.771991322227119	0.556469717394834	MAOA	1
hsa04260	Cardiac muscle contraction	3/262	87/8577	0.499256255200555	0.774127676603107	0.558009652471347	ATP1A1/ASPH/ATP1B1	3
hsa01240	Biosynthesis of cofactors	5/262	153/8577	0.503543426257689	0.774232634495361	0.558085308617937	GCLC/KYNU/SDR16C5/TDO2/DHRS3	5
hsa00515	Mannose type O-glycan biosynthesis	1/262	23/8577	0.510544708254187	0.774232634495361	0.558085308617937	B4GALT1	1
hsa03060	Protein export	1/262	23/8577	0.510544708254187	0.774232634495361	0.558085308617937	HSPA5	1

hsa 046 14	Renin-angiotensin system	1/262	23/8577	0.51054 470825 4187	0.77423 263449 5361	0.55808 530861 7937	MME	1
hsa 041 50	mTOR signaling pathway	5/262	156/8577	0.52083 719013 6173	0.78552 494250 0458	0.56622 507296 9438	SGK1/WNT5A/PIK3R1/MIOS/WNT2B	5
hsa 005 34	Glycosaminoglycan biosynthesis - heparan sulfate / heparin	1/262	24/8577	0.52553 620772 0799	0.78830 431158 1199	0.56822 850834 8004	HS6ST2	1
hsa 002 40	Pyrimidine metabolism	2/262	58/8577	0.53307 231779 6491	0.79528 626871 2604	0.57326 126920 9314	TYMP/DPYD	2
hsa 043 70	VEGF signaling pathway	2/262	59/8577	0.54234 091214 9945	0.80344 043016 7386	0.57913 898284 3768	PIK3R1/PPP3CA	2
hsa 045 20	Adherens junction	3/262	93/8577	0.54436 000159 8918	0.80344 043016 7386	0.57913 898284 3768	MYL9/TGFBR2/PTPN1	3
hsa 006 50	Butanoate metabolism	1/262	27/8577	0.56782 106951 4224	0.82483 481676 8031	0.59456 056586 254	GAD2	1
hsa 007 90	Folate biosynthesis	1/262	27/8577	0.56782 106951 4224	0.82483 481676 8031	0.59456 056586 254	AKR1B1	1
hsa 049 66	Collecting duct acid secretion	1/262	27/8577	0.56782 106951 4224	0.82483 481676 8031	0.59456 056586 254	CA2	1

hsa04666	Fc gamma R-mediated phagocytosis	3/262	97/8577	0.573091507088548	0.828132230138426	0.596937419893831	PLD1/VAV3/PIK3R1	3
hsa04925	Aldosterone synthesis and secretion	3/262	98/8577	0.580097338833158	0.830952266558163	0.598970169258173	ATP1A1/NR4A2/ATP1B1	3
hsa00601	Glycosphingolipid biosynthesis - lacto and neolacto series	1/262	28/8577	0.581064447267122	0.830952266558163	0.598970169258173	B4GALT1	1
hsa04270	Vascular smooth muscle contraction	4/262	134/8577	0.589396322826699	0.836692520638282	0.603107881009288	MYH10/MYL9/KCNMA1/NPPC	4
hsa05415	Diabetic cardiomyopathy	6/262	203/8577	0.591141454798786	0.836692520638282	0.603107881009288	TGFBR2/CTSD/COL1A1/TGFB3/TGFB2/PIK3R1	6
hsa05022	Pathways of neurodegeneration - multiple diseases	14/262	476/8577	0.598416805902856	0.841834057969029	0.606814023478 82	PRNP/LRRK2/WNT5A/UBE2L6/APP/OPTN/HSPA5/IL1B/XBP1/CAPN2/IL1A/PPP3CA/ATF6/WNT2B	14
hsa04310	Wnt signaling pathway	5/262	171/8577	0.603028978276337	0.841834057969029	0.606814023478 82	MMP7/WNT5A/JUN/PPP3CA/WNT2B	5
hsa01523	Antifolate resistance	1/262	30/8577	0.606350544264398	0.841834057969029	0.606814023478 82	IL1B	1
hsa05322	Systemic lupus erythematosus	4/262	137/8577	0.606974556289264	0.841834057969029	0.606814023478 82	C1R/HLA-DRA/HLA-DRB1/C1S	4

hsa05206	MicroRNAs in cancer	9/262	310/8577	0.610576623659481	0.842595740650083	0.607363062482326	VIM/CYP24A1/CYP1B1/TGFB2/PIK3R1/RDX/MCL1/STAT3/PLAU	9
hsa04664	Fc epsilon RI signaling pathway	2/262	68/8577	0.619929807770078	0.851246900221599	0.613599024187194	VAV3/PIK3R1	2
hsa04920	Adipocytokine signaling pathway	2/262	69/8577	0.627898644544395	0.857920920268579	0.618409816669571	NFKBIA/STAT3	2
hsa05131	Shigellosis	7/262	247/8577	0.634277653630603	0.859436964673019	0.619502617556067	CXCL8/NFKBIA/MYL9/IL1B/JUN/CAPN2/PIK3R1	7
hsa04120	Ubiquitin mediated proteolysis	4/262	142/8577	0.635236017367014	0.859436964673019	0.619502617556067	BIRC3/UBE2L6/UBA6/BIRC2	4
hsa04922	Glucagon signaling pathway	3/262	107/8577	0.639814566671867	0.861408880007002	0.620924021057679	PKM/PPP3CA/PYGL	3
hsa00982	Drug metabolism - cytochrome P450	2/262	72/8577	0.651027630946146	0.872250612335613	0.628738999738485	FMO2/MAOA	2
hsa04360	Axon guidance	5/262	182/8577	0.657941617842824	0.876747137479315	0.631980202073 19	WNT5A/MYL9/PIK3R1/PPP3CA/SEMA6D	5
hsa01230	Biosynthesis of amino acids	2/262	75/8577	0.673002979852982	0.876747137479315	0.631980202073 19	ASS1/PKM	2

hsa04623	Cytosolic DNA-sensing pathway	2/262	75/8577	0.673002979852982	0.876747137479315	0.631980202073190	NFKBIA/IL1B	2
hsa00040	Pentose and glucuronate interconversions	1/262	36/8577	0.673443453426140	0.876747137479315	0.631980202073190	AKR1B1	1
hsa00350	Tyrosine metabolism	1/262	36/8577	0.673443453426140	0.876747137479315	0.631980202073190	MAOA	1
hsa00500	Starch and sucrose metabolism	1/262	36/8577	0.673443453426140	0.876747137479315	0.631980202073190	PYGL	1
hsa00760	Nicotinate and nicotinamide metabolism	1/262	37/8577	0.683460759971317	0.881472755850857	0.635386540258627	NNMT	1
hsa01250	Biosynthesis of nucleotide sugars	1/262	37/8577	0.683460759971317	0.881472755850857	0.635386540258627	UAP1	1
hsa05100	Bacterial invasion of epithelial cells	2/262	77/8577	0.687022721561622	0.881945447213989	0.635727267442578	CAV1/PIK3R1	2
hsa04921	Oxytocin signaling pathway	4/262	154/8577	0.697543053897879	0.891305013313956	0.642473865432257	MYL9/JUN/FOS/PPP3CA	4
hsa00983	Drug metabolism - other enzymes	2/262	80/8577	0.707125912741478	0.891814546062987	0.642841148763938	TYMP/DPYD	2

hsa04722	Neurotrophin signaling pathway	3/262	119/8577	0.709805831210008	0.891814546062987	0.642841148763938	NFKBIA/JUN/PIK3R1	3
hsa00260	Glycine, serine and threonine metabolism	1/262	40/8577	0.711712786847917	0.891814546062987	0.642841148763938	MAOA	1
hsa04110	Cell cycle	4/262	157/8577	0.711875087702952	0.891814546062987	0.642841148763938	GADD45B/GADD45A/TGFB3/TGFB2	4
hsa04071	Sphingolipid signaling pathway	3/262	121/8577	0.720398533660938	0.891814546062987	0.642841148763938	CTSD/PLD1/PIK3R1	3
hsa04152	AMPK signaling pathway	3/262	121/8577	0.720398533660938	0.891814546062987	0.642841148763938	PFKFB3/PIK3R1/RAB2A	3
hsa03440	Homologous recombination	1/262	41/8577	0.720560303521906	0.891814546062987	0.642841148763938	RBBP8	1
hsa00860	Porphyrin metabolism	1/262	43/8577	0.737452002661705	0.904607789931691	0.652062823406139	HEPHL1	1
hsa04975	Fat digestion and absorption	1/262	43/8577	0.737452002661705	0.904607789931691	0.652062823406139	ABCA1	1
hsa03410	Base excision repair	1/262	44/8577	0.745512416922618	0.910448792347976	0.656273156955635	PARP4	1

hsa02010	ABC transporters	1/262	45/8577	0.753326286226061	0.9132737212251	0.658309432919694	ABCA1	1
hsa04540	Gap junction	2/262	88/8577	0.755521738665 81	0.9132737212251	0.658309432919694	GJA1/PDGFD	2
hsa04211	Longevity regulating pathway	2/262	89/8577	0.761061434354 25	0.9132737212251	0.658309432919694	SOD2/PIK3R1	2
hsa04727	GABAergic synapse	2/262	89/8577	0.761061434354 25	0.9132737212251	0.658309432919694	GAD2/SLC38A2	2
hsa00620	Pyruvate metabolism	1/262	47/8577	0.768244214226498	0.917902178036854	0.661645734740524	PKM	1
hsa04728	Dopaminergic synapse	3/262	132/8577	0.773357315302082	0.920028530273167	0.663178460036722	FOS/PPP3CA/MAOA	3
hsa00520	Amino sugar and nucleotide sugar metabolism	1/262	49/8577	0.782263195417255	0.926629364528594	0.667936498458826	UAP1	1
hsa00565	Ether lipid metabolism	1/262	50/8577	0.788952576608704	0.930257070067155	0.670551434945432	PLD1	1
hsa04910	Insulin signaling pathway	3/262	137/8577	0.794581655610449	0.930257070067155	0.670551434945432	PTPN1/PIK3R1/PYGL	3

hsa 049 13	Ovarian steroidogenesis	1/262	51/8577	0.79543 720484 0031	0.93025 707006 7155	0.67055 143494 5432	CYP1B1	1
hsa 002 70	Cysteine and methionine metabolism	1/262	52/8577	0.80172 332404 3867	0.93365 247863 3365	0.67299 892624 6018	GCLC	1
hsa 047 50	Inflammatory mediator regulation of TRP channels	2/262	98/8577	0.80620 382201 3747	0.93492 544065 4597	0.67391 650756 5671	IL1B/PIK3R1	2
hsa 005 64	Glycerophospholipid metabolism	2/262	99/8577	0.81072 285614 7224	0.93623 225228 7171	0.67485 848849 0753	PLD1/GPD2	2
hsa 049 16	Melanogenesis	2/262	101/8577	0.81948 074817 4919	0.94240 286040 1157	0.67930 640967 1315	WNT5A/WNT2B	2
hsa 049 14	Progesterone-mediated oocyte maturation	2/262	102/8577	0.82372 258766 4188	0.94335 034935 8157	0.67998 938226 0457	PIK3R1/HSP90AA1	2
hsa 004 80	Glutathione metabolism	1/262	57/8577	0.83038 186013 5845	0.94704 708015 4931	0.68265 407379 5888	GCLC	1
hsa 048 14	Motor proteins	4/262	193/8577	0.84647 958006 4236	0.95434 206934 2669	0.68791 247561 3137	MYH10/MYL9/MYO1B/MYO6	4
hsa 042 61	Adrenergic signaling in cardiomyocytes	3/262	154/8577	0.85471 571340 0653	0.95434 206934 2669	0.68791 247561 3137	ATP1A1/ADRB2/ATP1B1	3

hsa00140	Steroid hormone biosynthesis	1/262	62/8577	0.854911651607425	0.954342069342669	0.687912475613137	CYP1B1	1
hsa04934	Cushing syndrome	3/262	155/8577	0.857722418706724	0.954342069342669	0.687912475613137	WNT5A/PBX1/WNT2B	3
hsa00310	Lysine degradation	1/262	63/8577	0.859375908481042	0.954342069342669	0.687912475613137	KMT2E	1
hsa00561	Glycerolipid metabolism	1/262	63/8577	0.859375908481042	0.954342069342669	0.687912475613137	AKR1B1	1
hsa04929	GnRH secretion	1/262	64/8577	0.863703311814137	0.954342069342669	0.687912475613137	PIK3R1	1
hsa04725	Cholinergic synapse	2/262	113/8577	0.864802365085339	0.954342069342669	0.687912475613137	FOS/PIK3R1	2
hsa04927	Cortisol synthesis and secretion	1/262	65/8577	0.8678980413225	0.954342069342669	0.687912475613137	PBX1	1
hsa00010	Glycolysis / Gluconeogenesis	1/262	67/8577	0.875905565689202	0.955533344388822	0.688771175016681	PKM	1
hsa04720	Long-term potentiation	1/262	67/8577	0.875905565689202	0.955533344388822	0.688771175016681	PPP3CA	1

hsa05204	Chemical carcinogenesis - DNA adducts	1/262	69/8577	0.88342944431368	0.959946955238487	0.691952610755431	CYP1B1	1
hsa04015	Rap1 signaling pathway	4/262	210/8577	0.888922752212529	0.962128155335914	0.693524871695224	ID1/PDGFD/VAV3/PIK3R1	4
hsa04611	Platelet activation	2/262	124/8577	0.896953651905408	0.967028155960518	0.697056908758726	COL1A1/PIK3R1	2
hsa00980	Metabolism of xenobiotics by cytochrome P450	1/262	78/8577	0.912042458244552	0.974795703461213	0.702655941854192	CYP1B1	1
hsa04721	Synaptic vesicle cycle	1/262	78/8577	0.912042458244552	0.974795703461213	0.702655941854192	SLC1A3	1
hsa03018	RNA degradation	1/262	79/8577	0.914753939117587	0.974795703461213	0.702655941854192	MPHOSPH6	1
hsa04146	Peroxisome	1/262	83/8577	0.924792178747878	0.978894282666934	0.705610295286234	SOD2	1
hsa01232	Nucleotide metabolism	1/262	85/8577	0.92936050429028	0.978894282666934	0.705610295286234	TYMP	1
hsa04371	Apelin signaling pathway	2/262	139/8577	0.929457333173592	0.978894282666934	0.705610295286234	SERPINE1/EGR1	2

hsa04014	Ras signaling pathway	4/262	236/8577	0.93410151672066	0.978894282666934	0.705610295286234	ETS2/PDGFD/PLD1/PIK3R1	4
hsa05017	Spinocerebellar ataxia	2/262	143/8577	0.936333661681415	0.978894282666934	0.705610295286234	XBP1/PIK3R1	2
hsa04070	Phosphatidylinositol signaling system	1/262	97/8577	0.951513790366335	0.986040814885689	0.710761685787167	PIK3R1	1
hsa04713	Circadian entrainment	1/262	97/8577	0.951513790366335	0.986040814885689	0.710761685787167	FOS	1
hsa04020	Calcium signaling pathway	4/262	253/8577	0.95388731005246	0.986040814885689	0.710761685787167	PDGFD/ASPH/ADRB2/PPP3CA	4
hsa01200	Carbon metabolism	1/262	115/8577	0.972455607635618	0.999303913707405	0.720322043061401	PKM	1
hsa05034	Alcoholism	2/262	188/8577	0.980618545516461	0.999303913707405	0.720322043061401	FOSB/MAOA	2
hsa04613	Neutrophil extracellular trap formation	2/262	191/8577	0.982131515217319	0.999303913707405	0.720322043061401	CLEC7A/PIK3R1	2
hsa04114	Oocyte meiosis	1/262	131/8577	0.983354782500525	0.999303913707405	0.720322043061401	PPP3CA	1

hsa05014	Amyotrophic lateral sclerosis	5/262	364/8577	0.988404528892292	0.999303913707405	0.720322043061401	OPTN/HSPA5/XBP1/PPP3CA/ATF6	5
hsa03040	Spliceosome	2/262	216/8577	0.990995193984740	0.999303913707405	0.720322043061401	HSPA6/HSPA1A	2
hsa03010	Ribosome	1/262	167/8577	0.994659609567178	0.999303913707405	0.720322043061401	RPS4Y1	1
hsa04080	Neuroactive ligand-receptor interaction	3/262	367/8577	0.999244332574 81	0.999303913707405	0.720322043061401	LPAR6/NR3C1/ADRB2	3
hsa05016	Huntington disease	2/262	306/8577	0.999303913707405	0.999303913707405	0.720322043061401	SOD2/SLC1A3	2
