# Supplementary material for: Multiomics Reveals IL-17 Drives Epithelial Keratinization and Proliferation via EHF in Odontogenic Keratocysts
Source: Int J Mol Sci. 2026 May 4;27(9):4115. doi: 10.3390/ijms27094115 (PMC13163638; doi:10.3390/ijms27094115)
Supplement: Supplementary file 1 [file ijms-27-04115-s001.zip › ijms-4235677-supplementary/Supplementary Table S13.pdf]

Supplementary Table S13. EpC1-5 DEGs.

	p_val	avg_log2FC	pct.1	pct.2	p_val_adj	cluster	gene
CXCL14	0	1.8560001811096	0.833	0.403	0	EpC1	CXCL14
IL1R2	0	1.16218704257581	0.699	0.307	0	EpC1	IL1R2
COL17A1	0	1.04331611320739	0.726	0.233	0	EpC1	COL17A1
DST	0	1.00158553623066	0.772	0.406	0	EpC1	DST
KLK11	0	-0.931685911	0.195	0.611	0	EpC1	KLK11
EMP2	0	-0.937752898	0.509	0.879	0	EpC1	EMP2
SULT2B1	0	-0.955371294	0.076	0.462	0	EpC1	SULT2B1
ANXA2	0	-0.957243035	0.988	0.997	0	EpC1	ANXA2
HSPB1	0	-0.965338133	0.978	0.991	0	EpC1	HSPB1
CLDN7	0	-1.008227005	0.114	0.603	0	EpC1	CLDN7
PERP	0	-1.035984865	0.997	0.995	0	EpC1	PERP
GSTP1	0	-1.048960391	0.999	0.998	0	EpC1	GSTP1
S100A16	0	-1.063775776	0.526	0.866	0	EpC1	S100A16
CLCA2	0	-1.1552423	0.309	0.791	0	EpC1	CLCA2
EHF	0	-1.156949637	0.133	0.557	0	EpC1	EHF
TXN	0	-1.171681421	0.994	0.989	0	EpC1	TXN
S100A11	0	-1.188734664	0.997	0.997	0	EpC1	S100A11
TMPRSS11D	0	-1.193213326	0.195	0.699	0	EpC1	TMPRSS11D
GJB2	0	-1.203914537	0.112	0.514	0	EpC1	GJB2
AKR1B10	0	-1.2049588	0.156	0.619	0	EpC1	AKR1B10
SFN	0	-1.247688031	0.721	0.933	0	EpC1	SFN
GBP6	0	-1.436695822	0.229	0.639	0	EpC1	GBP6
CALML3	0	-1.439564975	0.206	0.728	0	EpC1	CALML3
CLDN4	0	-1.453619142	0.236	0.721	0	EpC1	CLDN4
PLAC8	0	-1.549190107	0.145	0.582	0	EpC1	PLAC8
LYPD3	0	-1.772474681	0.292	0.795	0	EpC1	LYPD3
TACSTD2	0	-1.828181431	0.436	0.833	0	EpC1	TACSTD2

ANXA1	0	-2.064544708	0.98	0.989	0	EpC1	ANXA1
KRT6A	0	-2.0669602	0.871	0.945	0	EpC1	KRT6A
KRT16	0	-2.074371126	0.133	0.577	0	EpC1	KRT16
CSTB	0	-2.251866801	0.916	0.962	0	EpC1	CSTB
KRT6B	0	-2.32075677	0.141	0.596	0	EpC1	KRT6B
SLPI	0	-2.588456313	0.079	0.466	0	EpC1	SLPI
CSTA	0	-3.084905031	0.433	0.786	0	EpC1	CSTA
MAL2	1.64484604250587e-303	-0.880321848	0.151	0.554	3.29725837680726e-299	EpC1	MAL2
MPZL2	1.03569031003785e-295	-0.809528932	0.322	0.721	2.07614479550188e-291	EpC1	MPZL2
SERPINB3	1.77087786956181e-295	-2.983626061	0.245	0.596	3.54990177732361e-291	EpC1	SERPINB3
S100A10	7.64798176192401e-288	-0.769888041	0.968	0.99	1.53311442399529e-283	EpC1	S100A10
SCEL	6.14671246171315e-285	-0.996861931	0.086	0.451	1.23216998007502e-280	EpC1	SCEL
SPINK5	3.06150735248141e-276	-1.478842886	0.053	0.393	6.13709763878423e-272	EpC1	SPINK5
PNOC	2.06971714902487e-275	0.83537099297969	0.367	0.052	4.14895499693526e-271	EpC1	PNOC
ANKRD22	2.34289340755729e-257	-0.619808335	0.025	0.331	4.69656412478935e-253	EpC1	ANKRD22
ZNF750	2.7678722265791e-253	-0.545718505	0.088	0.438	5.54847666540047e-249	EpC1	ZNF750
SERPINB13	7.24829901755633e-250	-0.836094698	0.215	0.588	1.45299402105934e-245	EpC1	SERPINB13
GLTP	1.21978284103062e-248	-0.959511564	0.251	0.616	2.44517668312998e-244	EpC1	GLTP
DSC2	1.34139189776727e-240	-1.119487416	0.588	0.822	2.68895419826428e-236	EpC1	DSC2
CXCL17	1.21985232413085e-236	-0.704243706	0.012	0.286	2.44531596895269e-232	EpC1	CXCL17
DYNLT3	1.56925353264588e-236	-1.005588953	0.404	0.726	3.14572563154193e-232	EpC1	DYNLT3
CTSC	7.11342964887755e-236	-0.977999828	0.165	0.512	1.42595810741399e-231	EpC1	CTSC
SDCBP2	7.46215539751839e-235	-0.528860401	0.03	0.319	1.49586367098654e-230	EpC1	SDCBP2
SERPINB2	9.95292564781585e-234	-0.870193069	0.043	0.343	1.99516347536117e-229	EpC1	SERPINB2
SPRR1B	1.44601032775552e-227	-3.880308464	0.274	0.563	2.89867230301872e-223	EpC1	SPRR1B
NMU	3.57900546711505e-226	-0.505811811	0.043	0.337	7.17447435937883e-222	EpC1	NMU
CD82	7.01923111757791e-221	-0.540844176	0.174	0.525	1.40707506982967e-216	EpC1	CD82
CD24	2.59157521362561e-220	-1.256672814	0.514	0.737	5.1950716732339e-216	EpC1	CD24
TMEM40	4.94218473410793e-218	-0.495342687	0.04	0.322	9.90710351799275e-214	EpC1	TMEM40

TMPRSS11A	3.50413969262365e-217	-1.293191707	0.074	0.367	7.02439842783337e-213	EpC1	TMPRSS11A
LY6D	2.66234705703566e-215	-1.448460913	0.364	0.686	5.33694091053369e-211	EpC1	LY6D
TMSB10	2.66383106215508e-214	-0.554981104	0.996	0.998	5.33991574719608e-210	EpC1	TMSB10
TM4SF1	7.09668492324564e-212	-0.764985861	0.671	0.862	1.42260145971382e-207	EpC1	TM4SF1
SERPINB1	1.39000869404981e-210	-1.538837257	0.159	0.471	2.78641142809225e-206	EpC1	SERPINB1
HBEGF	2.29166599570062e-209	-0.872029223	0.151	0.468	4.59387365498146e-205	EpC1	HBEGF
CLDN1	1.12448296166588e-206	-0.77081992	0.036	0.302	2.25413854495542e-202	EpC1	CLDN1
HIF1A	1.23275132943383e-206	-0.620579253	0.292	0.641	2.47117331498306e-202	EpC1	HIF1A
SERPINB5	7.32473805380432e-204	-0.733173451	0.645	0.869	1.46831699026561e-199	EpC1	SERPINB5
GAS5	8.61167521452244e-204	0.688290115677529	0.803	0.619	1.72629641350317e-199	EpC1	GAS5
ITM2B	3.61315197179055e-203	-0.5965308	0.828	0.946	7.24292444265134e-199	EpC1	ITM2B
SLC9A3R1	2.39661300169075e-202	-0.552511399	0.297	0.647	4.80425042318928e-198	EpC1	SLC9A3R1
DMKN	4.21849454740784e-199	-1.232987164	0.265	0.58	8.45639416973376e-195	EpC1	DMKN
S100A14	1.32533605436069e-198	-0.862262199	0.573	0.82	2.65676865457144e-194	EpC1	S100A14
RPL34	1.75493485990268e-198	0.465027612991072	0.999	0.992	3.51794242016091e-194	EpC1	RPL34
LDHA	8.82168470324863e-198	-0.613464565	0.394	0.739	1.76839491561322e-193	EpC1	LDHA
TMPRSS11F	2.40635592942884e-197	-0.625115878	0.038	0.296	4.82378109613305e-193	EpC1	TMPRSS11F
SERPINB4	4.50345933546108e-197	-2.68699195	0.062	0.334	9.02763458386529e-193	EpC1	SERPINB4
H2AFJ	4.81468234808e-197	-0.497231446	0.103	0.402	9.65151223496116e-193	EpC1	H2AFJ
RPL3	9.81893091745149e-197	0.474814440917394	0.998	0.978	1.96830289171233e-192	EpC1	RPL3
SBSN	6.60092923808607e-196	-1.478776322	0.015	0.251	1.32322227506673e-191	EpC1	SBSN
KRTDAP	1.05426369440051e-195	-2.414875107	0.063	0.332	2.11337700179525e-191	EpC1	KRTDAP
PTTG1	2.17825168036879e-194	-0.966099462	0.158	0.463	4.36652331846728e-190	EpC1	PTTG1
TALDO1	2.0068080713893e-193	-0.628287093	0.57	0.843	4.02284745990698e-189	EpC1	TALDO1
RHCG	1.67989350785063e-189	-2.130332237	0.021	0.257	3.36751452583738e-185	EpC1	RHCG
ATP1B1	2.64216256000602e-189	-0.629053456	0.225	0.557	5.29647906778807e-185	EpC1	ATP1B1
DSG3	6.80466200660495e-189	-0.788841356	0.624	0.866	1.36406254584403e-184	EpC1	DSG3
TUBA4A	3.59563427419506e-187	-0.644238633	0.254	0.578	7.20780846605142e-183	EpC1	TUBA4A
AP2S1	4.24000471214159e-187	-0.519241703	0.35	0.702	8.49951344595904e-183	EpC1	AP2S1

AKR1C2	5.29794613481596e-187	-1.009659871	0.296	0.597	1.06202628218521e-182	EpC1	AKR1C2
KLK13	5.50864726766257e-187	-1.334196002	0.02	0.253	1.10426343127564e-182	EpC1	KLK13
SERPINB11	3.5766553991879e-185	-0.595807828	0.012	0.236	7.16976341321207e-181	EpC1	SERPINB11
TMEM45B	6.94411549553754e-184	-0.494229359	0.008	0.228	1.39201739223546e-179	EpC1	TMEM45B
LGALS7B	2.02878336938318e-183	-0.974923005	0.143	0.451	4.06689914226552e-179	EpC1	LGALS7B
FAM3B	5.95107530924648e-183	-0.461061825	0.027	0.264	1.19295255649155e-178	EpC1	FAM3B
LGMN	7.82917673379324e-183	-0.47111291	0.088	0.365	1.56943676805619e-178	EpC1	LGMN
PHLDA2	8.98304481375724e-183	-0.56810293	0.161	0.465	1.80074116336578e-178	EpC1	PHLDA2
NUCB2	3.99995919098295e-182	-0.616734315	0.181	0.49	8.01831819424443e-178	EpC1	NUCB2
BHLHE40	4.89978550648571e-182	-0.65514247	0.157	0.456	9.82211002630126e-178	EpC1	BHLHE40
DHCR24	6.67265215657904e-182	-0.42598486	0.118	0.415	1.33759985130784e-177	EpC1	DHCR24
EEF1A1	8.13378280297002e-182	0.426130342882416	0.999	0.99	1.63049810068337e-177	EpC1	EEF1A1
LGALS3	1.59734983864079e-181	-0.857619927	0.472	0.761	3.20204748653932e-177	EpC1	LGALS3
AQP3	1.11011582509583e-180	-0.908162878	0.042	0.285	2.22533818298709e-176	EpC1	AQP3
SOSTDC1	2.93465047616512e-180	0.640879007573183	0.669	0.362	5.88280034452059e-176	EpC1	SOSTDC1
KRT6C	4.88056973740639e-179	-0.807972232	0.027	0.26	9.78359009560485e-175	EpC1	KRT6C
NQO1	6.97671123851581e-179	-0.636962274	0.245	0.564	1.39855153487288e-174	EpC1	NQO1
HOPX	4.51670446854637e-178	-1.019222904	0.234	0.544	9.05418577764805e-174	EpC1	HOPX
TMEM154	1.23967105438241e-177	-0.600480003	0.142	0.432	2.48504459561497e-173	EpC1	TMEM154
PDZK1IP1	9.63609323025267e-177	-1.251892142	0.022	0.245	1.93165124893645e-172	EpC1	PDZK1IP1
GRHL1	1.90606808889688e-175	-0.433108553	0.073	0.336	3.82090409100268e-171	EpC1	GRHL1
YWHAZ	8.78307062481690e-174	-0.5042009	0.931	0.976	1.76065433745079e-169	EpC1	YWHAZ
BCAM	3.62221469148804e-173	0.593809446294358	0.462	0.186	7.26109157055692e-169	EpC1	BCAM
RPS27	6.42491835656556e-173	0.415135128641614	1	0.999	1.28793913375713e-168	EpC1	RPS27
TAGLN2	8.14099405908934e-173	-0.589255411	0.511	0.811	1.63194366908505e-168	EpC1	TAGLN2
ANXA3	3.00418542333987e-172	-0.452108664	0.074	0.337	6.02219009962711e-168	EpC1	ANXA3
DSG1	2.67059411611289e-171	-0.575687108	0.03	0.256	5.3534729651599e-167	EpC1	DSG1
SDR16C5	1.48579166708754e-170	-0.685888047	0.034	0.262	2.97841797584367e-166	EpC1	SDR16C5
MGLL	5.48717470175392e-170	-0.623078531	0.177	0.464	1.09995904071359e-165	EpC1	MGLL

TMPRSS4	1.16593388132367e-168	-0.481253091	0.173	0.465	2.33723105850142e-164	EpC1	TMPRSS4
CMPK1	1.69299302817896e-168	-0.463490748	0.156	0.444	3.39377382428754e-164	EpC1	CMPK1
PGD	3.5447401005121e-168	-0.481448513	0.211	0.518	7.10578600548655e-164	EpC1	PGD
LAD1	1.26971247176235e-167	-0.450842889	0.162	0.458	2.5452656208948e-163	EpC1	LAD1
NECTIN4	1.47422293139822e-166	-0.353175185	0.039	0.269	2.95522728828087e-162	EpC1	NECTIN4
SLITRK6	3.35531010590142e-166	0.473537495738642	0.271	0.051	6.72605463828998e-162	EpC1	SLITRK6
PI3	4.63529078850284e-165	-3.754206349	0.149	0.409	9.29190391463278e-161	EpC1	PI3
IL1RN	8.70967633395136e-165	-1.761932033	0.213	0.492	1.74594171790389e-160	EpC1	IL1RN
SQOR	2.87359438158636e-164	-0.404786106	0.06	0.305	5.76040729732802e-160	EpC1	SQOR
PDPN	4.13026124149424e-164	0.789462898267871	0.535	0.27	8.27952168469936e-160	EpC1	PDPN
NFIB	1.35155163019506e-161	0.674433789597153	0.541	0.279	2.70932039788903e-157	EpC1	NFIB
DYNLT1	1.84894452094731e-161	-0.51609272	0.428	0.749	3.70639418669098e-157	EpC1	DYNLT1
CTNNBIP1	4.0951399969014e-160	-0.495262475	0.102	0.36	8.20911763778854e-156	EpC1	CTNNBIP1
MGST2	1.29899714137307e-159	-0.477859215	0.243	0.55	2.60396966959645e-155	EpC1	MGST2
UQCR10	4.08497924597837e-159	-0.501526567	0.747	0.929	8.18874939648824e-155	EpC1	UQCR10
PSMA7	1.37643473899124e-158	-0.51584766	0.972	0.986	2.75920107778183e-154	EpC1	PSMA7
CLTB	1.38834648885345e-158	-0.46877627	0.218	0.517	2.78307937155562e-154	EpC1	CLTB
TMPRSS11E	5.03856130165597e-158	-1.672931737	0.017	0.216	1.01002999852995e-153	EpC1	TMPRSS11E
SPRR2A	5.11067102898721e-158	-2.524984214	0.028	0.236	1.02448511447078e-153	EpC1	SPRR2A
RAB10	8.34673504016092e-158	-0.548210199	0.362	0.671	1.67318650615066e-153	EpC1	RAB10
KLK10	1.20927967518872e-156	-0.89420219	0.153	0.424	2.4241220368833e-152	EpC1	KLK10
RHOV	1.14175214664546e-155	-0.368228235	0.073	0.319	2.28875635316549e-151	EpC1	RHOV
SDC1	1.15299708907305e-154	-0.46818349	0.21	0.5	2.31129796475584e-150	EpC1	SDC1
PPIC	4.12172229008255e-154	-0.385181744	0.089	0.34	8.26240450269947e-150	EpC1	PPIC
ARPC3	5.48657623211604e-154	-0.50032034	0.604	0.856	1.09983907148998e-149	EpC1	ARPC3
NAA20	5.56490365542276e-154	-0.45921724	0.33	0.645	1.11554058676605e-149	EpC1	NAA20
DBI	6.87710822476868e-154	-1.094491372	0.826	0.865	1.37858511473713e-149	EpC1	DBI
COX17	1.194412136686e-153	-0.455654875	0.257	0.567	2.39431856920075e-149	EpC1	COX17
C12orf75	2.10733723531394e-153	-0.537324834	0.209	0.505	4.22436822191033e-149	EpC1	C12orf75

ENO1	1.51884074664888e-152	-0.532935215	0.658	0.887	3.04466816073234e-148	EpC1	ENO1
SDC4	2.32613432073823e-151	-0.471974055	0.166	0.44	4.66296885935185e-147	EpC1	SDC4
ATP1B3	6.73159909059994e-151	0.555296873256263	0.915	0.778	1.34941635370166e-146	EpC1	ATP1B3
CENPW	2.79532780485878e-150	-0.57465901	0.06	0.285	5.60351411761991e-146	EpC1	CENPW
EPCAM	5.61659028968727e-150	0.713221038760012	0.722	0.496	1.12590168947071e-145	EpC1	EPCAM
FDFT1	4.36001666995999e-149	-0.525608119	0.208	0.486	8.74008941660179e-145	EpC1	FDFT1
CERS3	4.70322266633721e-149	-0.447578283	0.137	0.402	9.42808015693956e-145	EpC1	CERS3
VAMP8	5.70737651670208e-149	-0.522077322	0.335	0.637	1.1441006965381e-144	EpC1	VAMP8
HEBP2	8.02658533190365e-149	-0.441723652	0.203	0.491	1.60900929563341e-144	EpC1	HEBP2
SUSD4	4.01488069343889e-148	-0.330011258	0.031	0.239	8.0482298380676e-144	EpC1	SUSD4
FABP5	2.40690738042889e-146	-1.729148176	0.399	0.643	4.82488653480776e-142	EpC1	FABP5
DUSP22	5.30031874112641e-146	-0.358741526	0.061	0.287	1.0625018948462e-141	EpC1	DUSP22
GSTA1	7.06538235368595e-146	-1.802974501	0.223	0.472	1.41632654661988e-141	EpC1	GSTA1
DEGS2	1.42560999469623e-145	-0.336059442	0.014	0.201	2.85777779536806e-141	EpC1	DEGS2
CDH1	1.50478863178606e-145	-0.468025216	0.357	0.67	3.01649929127834e-141	EpC1	CDH1
C5orf46	1.80542713132594e-145	-0.818072317	0.029	0.228	3.61915922745597e-141	EpC1	C5orf46
RALA	3.64823260181261e-145	-0.444463771	0.275	0.575	7.31324707359355e-141	EpC1	RALA
POF1B	4.36779924830752e-145	-0.493020454	0.091	0.329	8.75569037315726e-141	EpC1	POF1B
PFN1	1.92621836757396e-144	-0.475590816	0.952	0.972	3.86129733963876e-140	EpC1	PFN1
MSMO1	2.41761291986819e-144	-0.469564618	0.11	0.356	4.84634685916777e-140	EpC1	MSMO1
DYNLL1	2.79291534219536e-144	-0.575906943	0.906	0.934	5.59867809496483e-140	EpC1	DYNLL1
HIGD1A	8.9487846400803e-144	-0.494688568	0.286	0.568	1.7938733689505e-139	EpC1	HIGD1A
JUP	1.28144702366032e-143	-0.5326288	0.378	0.675	2.56878870362948e-139	EpC1	JUP
DENND2C	2.17969891570916e-143	-0.437224017	0.117	0.37	4.36942444643057e-139	EpC1	DENND2C
SPRR2D	1.83985783177443e-142	-1.446107534	0.015	0.198	3.68817900957503e-138	EpC1	SPRR2D
COX6C	2.04289878813232e-142	-0.48143876	0.947	0.974	4.09519491069006e-138	EpC1	COX6C
SQLE	2.59845246151099e-142	-0.474250412	0.158	0.419	5.20885780434494e-138	EpC1	SQLE
CIB1	4.11373762387043e-142	-0.679413799	0.177	0.437	8.24639844081067e-138	EpC1	CIB1
PTGR1	4.6284972116101e-142	-0.509237594	0.194	0.46	9.27828551039362e-138	EpC1	PTGR1

KCNK7	3.67005388985539e-141	-0.374594886	0.037	0.24	7.35699002760411e-137	EpC1	KCNK7
STMP1	4.54746242212107e-141	-0.411888499	0.284	0.585	9.11584317138389e-137	EpC1	STMP1
ADH7	8.71788800417683e-141	-0.738413698	0.312	0.574	1.74758782931729e-136	EpC1	ADH7
PTS	4.03498859346327e-140	-0.389807512	0.178	0.451	8.08853813445647e-136	EpC1	PTS
GM2A	5.64824911507485e-140	-0.409015782	0.112	0.359	1.1322480176079e-135	EpC1	GM2A
RAB25	6.9485790890705e-140	-0.48816287	0.218	0.491	1.39291216419507e-135	EpC1	RAB25
TIMM8B	4.61205790935035e-139	-0.492336786	0.379	0.671	9.24533128508371e-135	EpC1	TIMM8B
S100A9	8.45877594418074e-139	-3.966816751	0.098	0.311	1.69564622577047e-134	EpC1	S100A9
PRSS3	1.3273149044726e-138	-0.521563238	0.008	0.179	2.66073545750578e-134	EpC1	PRSS3
CDKN2B	5.10318184772828e-138	-0.322137158	0.027	0.219	1.02298383319561e-133	EpC1	CDKN2B
RPL13A	2.35617206403159e-137	0.366991360505994	1	0.992	4.72318251955773e-133	EpC1	RPL13A
DECR1	2.97367488096899e-137	-0.493240529	0.345	0.642	5.96102866639043e-133	EpC1	DECR1
ACTB	2.63201252698566e-136	-0.476248498	0.986	0.992	5.27613231159544e-132	EpC1	ACTB
CAPN2	4.5345224435405e-136	-0.426758682	0.141	0.392	9.08990369032128e-132	EpC1	CAPN2
SAT1	9.06603812346851e-136	-0.839917704	0.969	0.973	1.8173780022305e-131	EpC1	SAT1
LDHB	4.92155648816342e-135	0.551954928387699	0.718	0.49	9.86575213617239e-131	EpC1	LDHB
ARPC2	1.17227087441747e-133	-0.431044265	0.912	0.965	2.34993419485726e-129	EpC1	ARPC2
KRT13	3.37515315769804e-133	-1.046445641	0.081	0.297	6.76583201992149e-129	EpC1	KRT13
COA3	4.49064765425478e-133	-0.478547994	0.314	0.597	9.00195228771913e-129	EpC1	COA3
EPHB6	6.39594424601483e-133	-0.471690359	0.153	0.4	1.28213098355613e-128	EpC1	EPHB6
RAB11A	1.11250222548145e-132	-0.496582507	0.447	0.731	2.23012196120012e-128	EpC1	RAB11A
ATP5F1E	3.80922275953774e-132	-0.444572621	0.869	0.955	7.63596794376936e-128	EpC1	ATP5F1E
SPRR1A	1.62286588952713e-131	-1.989900514	0.025	0.205	3.25319696214608e-127	EpC1	SPRR1A
PTCH1	2.66480375437806e-131	0.543987405078333	0.347	0.126	5.34186560602625e-127	EpC1	PTCH1
HMGB2	3.691884777162e-131	-0.886229477	0.141	0.37	7.40075222429895e-127	EpC1	HMGB2
UQCRQ	1.97634619716246e-130	-0.452926798	0.766	0.92	3.96178358683187e-126	EpC1	UQCRQ
IL36G	2.40376908548333e-130	-1.290161608	0.013	0.182	4.81859550875988e-126	EpC1	IL36G
HHIP	2.51046218142381e-130	0.590421957011505	0.249	0.06	5.03247248888217e-126	EpC1	HHIP
DIRAS3	3.05569251587723e-130	-0.59731829	0.096	0.318	6.12544121732749e-126	EpC1	DIRAS3

ALDH1A1	1.26514872187235e-129	-0.447728059	0.058	0.263	2.5361171278653e-125	EpC1	ALDH1A1
PLAUR	2.1102116659673e-129	-0.872276021	0.082	0.292	4.23013030559806e-125	EpC1	PLAUR
HMGCS1	5.56176305914667e-129	-0.432928348	0.142	0.385	1.11491102283654e-124	EpC1	HMGCS1
F3	1.00390310616273e-128	-0.467499815	0.078	0.291	2.0124241666138e-124	EpC1	F3
EIF5A	2.76256334824672e-128	-0.437463769	0.398	0.694	5.53783448789537e-124	EpC1	EIF5A
CALML5	6.87871036611906e-128	-0.540651022	0.014	0.181	1.37890627999223e-123	EpC1	CALML5
NUSAP1	2.28410229946262e-127	-0.891376324	0.028	0.206	4.57871146950276e-123	EpC1	NUSAP1
RPL5	1.14269553491309e-126	0.395765825354871	0.996	0.958	2.29064746928678e-122	EpC1	RPL5
FAM84A	1.1686892999434e-126	-0.364841962	0.132	0.372	2.34275457066653e-122	EpC1	FAM84A
GPCPD1	1.65203024173286e-126	-0.640540023	0.083	0.295	3.31165982257769e-122	EpC1	GPCPD1
PLAT	1.86655211172357e-126	-0.927627394	0.061	0.263	3.74169036316107e-122	EpC1	PLAT
ELF3	3.4002451181925e-126	-0.528097009	0.165	0.407	6.81613136392868e-122	EpC1	ELF3
TYMP	3.98346829848256e-126	-0.412283599	0.056	0.255	7.98526055113813e-122	EpC1	TYMP
NAGK	2.10865839924568e-125	-0.347156064	0.103	0.329	4.22701662712788e-121	EpC1	NAGK
TMPRSS11BNL	2.53913446389916e-125	-0.35004387	0.022	0.194	5.08994894633225e-121	EpC1	TMPRSS11BNL
TGFBI	2.94419587918063e-125	0.48567415610927	0.456	0.221	5.90193505940549e-121	EpC1	TGFBI
LY6G6C	4.1967980357517e-125	-0.63201091	0.053	0.249	8.41290134246786e-121	EpC1	LY6G6C
CAPG	2.21190420026812e-124	-0.349437961	0.21	0.478	4.43398315985748e-120	EpC1	CAPG
PYGL	2.62004063007255e-124	-0.271148574	0.031	0.212	5.25213344704344e-120	EpC1	PYGL
ARL8B	3.77417601593615e-124	-0.356003378	0.173	0.427	7.56571324154561e-120	EpC1	ARL8B
SOX4	4.63664344390541e-124	0.573151580222376	0.857	0.69	9.29461544765278e-120	EpC1	SOX4
TECR	5.81434220746649e-124	-0.385360673	0.318	0.599	1.16554303890873e-119	EpC1	TECR
GJB6	1.9102882121445e-123	-0.414681519	0.103	0.327	3.82936375006487e-119	EpC1	GJB6
S100A2	6.39302038976105e-123	-1.015662192	0.989	0.968	1.2815448673315e-118	EpC1	S100A2
ACTR3	9.08463126812371e-123	-0.391645683	0.337	0.626	1.82110518400808e-118	EpC1	ACTR3
RPL11	3.24641349076944e-122	0.392697585031934	0.999	0.98	6.50776048359643e-118	EpC1	RPL11
CDKN1A	3.36309093553084e-122	-0.423564159	0.277	0.554	6.74165208936512e-118	EpC1	CDKN1A
CYSRT1	3.67830137234851e-122	-0.470611123	0.009	0.164	7.37352293100983e-118	EpC1	CYSRT1
SGK1	5.31682580104986e-122	-0.618087156	0.531	0.774	1.06581090007846e-117	EpC1	SGK1

FAM114A1	1.15961085932372e-121	-0.328051407	0.112	0.339	2.32455592860033e-117	EpC1	FAM114A1
RDH11	1.51895671196222e-121	-0.360314859	0.152	0.391	3.04490062479946e-117	EpC1	RDH11
ENSA	9.91971883233629e-121	-0.4326478	0.419	0.699	1.98850683713013e-116	EpC1	ENSA
MBOAT2	1.8705013892959e-120	-0.433962447	0.035	0.213	3.74960708498256e-116	EpC1	MBOAT2
ELL2	2.24530234344885e-120	-0.318649195	0.07	0.275	4.50093307767757e-116	EpC1	ELL2
IDH2	5.17549941736735e-120	-0.327233659	0.094	0.309	1.03748061320546e-115	EpC1	IDH2
CEACAM1	6.14684138839617e-120	-0.570501515	0.004	0.151	1.2321958247179e-115	EpC1	CEACAM1
CLCA4	8.80168727147723e-120	-1.443560915	0.016	0.174	1.76438623044033e-115	EpC1	CLCA4
APOBEC3A	2.2372493643708e-119	-0.892017532	0.012	0.168	4.4847900758177e-115	EpC1	APOBEC3A
TMEM147	4.90598864760189e-119	-0.385930771	0.275	0.546	9.83454484298275e-115	EpC1	TMEM147
GSTM3	1.06122383233389e-118	-1.095973221	0.306	0.514	2.12732929429652e-114	EpC1	GSTM3
PRDX6	2.51021946433255e-118	-0.584035149	0.408	0.655	5.03198593820103e-114	EpC1	PRDX6
CTSD	1.456869219515e-117	-0.507163674	0.209	0.453	2.92044003743978e-113	EpC1	CTSD
S100A7	2.48878874295006e-117	-4.448508798	0.066	0.252	4.98902591411769e-113	EpC1	S100A7
B4GALT4	5.70134772806601e-117	-0.330436051	0.066	0.262	1.14289216556811e-112	EpC1	B4GALT4
CLINT1	8.68114933091416e-117	-0.405984517	0.312	0.59	1.74022319487505e-112	EpC1	CLINT1
GRN	1.23132086662166e-116	-0.595613899	0.219	0.458	2.46830580922978e-112	EpC1	GRN
PLEKHA5	2.35266925593609e-116	-0.344739887	0.097	0.311	4.71616079044948e-112	EpC1	PLEKHA5
EP400P1	2.62639064064142e-116	-0.292603272	0.029	0.198	5.26486267822979e-112	EpC1	EP400P1
ECM1	3.54854044680922e-116	-0.769558279	0.013	0.165	7.11340417967377e-112	EpC1	ECM1
PLEKHN1	9.67481761984556e-116	-0.28179235	0.031	0.202	1.93941394007424e-111	EpC1	PLEKHN1
CLIC3	1.44339237055058e-115	-0.484032864	0.015	0.169	2.89342434600569e-111	EpC1	CLIC3
CYCS	3.24543318170771e-115	-0.494901094	0.571	0.788	6.50579535605127e-111	EpC1	CYCS
CXCL8	8.11043578315789e-115	-1.958853554	0.117	0.323	1.62581795709183e-110	EpC1	CXCL8
CD58	1.58585522036601e-114	-0.323559279	0.103	0.318	3.17900537474571e-110	EpC1	CD58
PTBP3	3.74153710965182e-114	-0.339716698	0.159	0.395	7.50028529000803e-110	EpC1	PTBP3
CLEC2B	3.08153023188029e-113	-1.492551959	0.038	0.206	6.17723550282723e-109	EpC1	CLEC2B
CYR61	8.89681921090178e-113	0.654440604278639	0.457	0.241	1.78345637901737e-108	EpC1	CYR61
GPX3	1.00501029794158e-112	-0.46556097	0.011	0.157	2.01464364325369e-108	EpC1	GPX3

NOP10	1.48616473862539e-112	-0.386740711	0.429	0.719	2.97916583504845e-108	EpC1	NOP10
PGAM1	3.98813980419743e-112	-0.293431376	0.105	0.317	7.99462505149417e-108	EpC1	PGAM1
DERA	4.81158123447646e-112	-0.289480921	0.092	0.301	9.64529574263151e-108	EpC1	DERA
TMEM54	7.73570801166898e-112	-0.314691158	0.107	0.32	1.55070002801916e-107	EpC1	TMEM54
AKR1C3	1.06035767452533e-111	-0.886276105	0.511	0.664	2.12559299435347e-107	EpC1	AKR1C3
CTNND1	1.069232758128e-111	-0.365063145	0.214	0.463	2.14338398694339e-107	EpC1	CTNND1
PGM2	2.01791246780273e-111	-0.285273289	0.072	0.267	4.04510733295736e-107	EpC1	PGM2
C6orf132	3.43836613943699e-111	-0.270612726	0.045	0.222	6.8925487631154e-107	EpC1	C6orf132
TPD52L1	3.84316159495085e-111	-0.325721416	0.093	0.298	7.70400173323847e-107	EpC1	TPD52L1
PKP1	5.30577897370121e-111	-0.456704317	0.425	0.679	1.06359645306814e-106	EpC1	PKP1
PPP4C	7.89275245726736e-111	-0.305550072	0.147	0.374	1.58218115758382e-106	EpC1	PPP4C
MANF	8.12354981617073e-111	-0.353508137	0.196	0.436	1.62844679614958e-106	EpC1	MANF
RPL13	8.20124339337943e-111	0.33108754192396	0.999	0.99	1.64402125063684e-106	EpC1	RPL13
AK2	9.64891672781067e-111	-0.363060252	0.294	0.563	1.93422184725693e-106	EpC1	AK2
PELI1	9.83067195347495e-111	-0.304090342	0.057	0.242	1.97065649979359e-106	EpC1	PELI1
GLRX3	1.06140021187377e-110	-0.376384936	0.244	0.499	2.12768286472215e-106	EpC1	GLRX3
PSMD1	1.27531111968678e-110	-0.3278923	0.168	0.403	2.55648867052411e-106	EpC1	PSMD1
TGM1	1.64072860689277e-110	-0.383025724	0.013	0.16	3.28900456537725e-106	EpC1	TGM1
GGCT	3.17522175164649e-110	-0.386067348	0.239	0.488	6.36504952335054e-106	EpC1	GGCT
PPL	4.05540294761209e-110	-0.385515281	0.114	0.326	8.1294607487832e-106	EpC1	PPL
PDLIM5	7.39974499915809e-110	-0.34758438	0.22	0.469	1.48335288253123e-105	EpC1	PDLIM5
CAPZB	2.1208437856342e-109	-0.353644848	0.424	0.709	4.25144345268232e-105	EpC1	CAPZB
PRDM1	2.23929042406679e-109	-0.518951042	0.073	0.26	4.48888158408429e-105	EpC1	PRDM1
COPS9	2.9436983117717e-109	-0.457971556	0.462	0.721	5.90093763577755e-105	EpC1	COPS9
MAPK13	4.42124520553679e-109	-0.302716751	0.113	0.326	8.86282813901906e-105	EpC1	MAPK13
POLR2J3	7.82625605308163e-109	-0.74784345	0.271	0.49	1.56885128840074e-104	EpC1	POLR2J3
KLK12	1.58233192559029e-108	-0.414981828	0.003	0.136	3.17194257803829e-104	EpC1	KLK12
TOP2A	1.60758429232692e-108	-0.847551074	0.019	0.169	3.22256347239854e-104	EpC1	TOP2A
RER1	2.07835510364799e-108	-0.309197275	0.17	0.403	4.16627064077277e-104	EpC1	RER1

TJP1	3.09808634903388e-108	-0.324026902	0.112	0.323	6.21042389527332e-104	EpC1	TJP1
TPX2	6.7889652389669e-108	-0.508073592	0.018	0.166	1.3609159718033e-103	EpC1	TPX2
LOXL4	7.81513228149675e-108	0.339566187043737	0.186	0.035	1.56662141714884e-103	EpC1	LOXL4
TST	1.1800860188592e-107	-0.332201883	0.064	0.248	2.36560043340514e-103	EpC1	TST
PLD1	1.52042484707753e-107	-0.323550576	0.129	0.346	3.04784364845161e-103	EpC1	PLD1
PIR	5.48903228131194e-107	-0.343437016	0.157	0.383	1.10033141111179e-102	EpC1	PIR
SLC25A39	6.24812820173445e-107	-0.309452324	0.142	0.364	1.25249977931969e-102	EpC1	SLC25A39
MT-RNR2	8.62888935350127e-107	0.292762307156748	1	1	1.72974715980287e-102	EpC1	MT-RNR2
ZCCHC6	3.06277666246041e-106	-0.326376052	0.143	0.363	6.13964209756814e-102	EpC1	ZCCHC6
ACADVL	3.3411181801302e-106	-0.320446285	0.206	0.448	6.697605503889e-102	EpC1	ACADVL
ASAH1	5.24373657648384e-106	-0.34890198	0.187	0.416	1.05115943412195e-101	EpC1	ASAH1
BGN	6.02768417548423e-106	0.392037134007316	0.32	0.122	1.20830956981757e-101	EpC1	BGN
WFDC5	6.74252359083444e-106	-0.29155482	0.01	0.15	1.35160627901867e-101	EpC1	WFDC5
TMEM79	1.260298414765e-105	-0.270387931	0.043	0.212	2.52639420223793e-101	EpC1	TMEM79
GPNMB	1.34585798583537e-105	-0.685916947	0.129	0.342	2.69790691840557e-101	EpC1	GPNMB
FAM135A	1.6617494528245e-105	-0.302758273	0.084	0.278	3.331142953132e-101	EpC1	FAM135A
CMC2	1.68148163849957e-105	-0.340201063	0.16	0.384	3.37069809253624e-101	EpC1	CMC2
XBP1	1.82115432600048e-105	-0.322188664	0.113	0.319	3.65068596190056e-101	EpC1	XBP1
ATP6V0B	9.12654232088513e-105	-0.365637252	0.35	0.619	1.82950667364463e-100	EpC1	ATP6V0B
NDUFB1	1.1313520205429e-104	-0.375355315	0.429	0.71	2.26790826038029e-100	EpC1	NDUFB1
TMEM254	1.30211612426272e-104	-0.26396693	0.056	0.233	2.61022198269704e-100	EpC1	TMEM254
COX7A2	2.96113492948056e-104	-0.370768246	0.916	0.963	5.93589107963673e-100	EpC1	COX7A2
SPRR3	4.61330002643739e-104	-4.077468478	0.047	0.21	9.24782123299639e-100	EpC1	SPRR3
FDCSP	5.97895313518275e-104	-3.975631969	0.112	0.299	1.19854094547873e-99	EpC1	FDCSP
TRIM16	7.18495788414051e-104	-0.269230955	0.064	0.246	1.44029665745481e-99	EpC1	TRIM16
MUC15	7.47212366766481e-104	-0.446325982	0.136	0.343	1.49786191042009e-99	EpC1	MUC15
NCCRP1	8.24498575000914e-104	-0.35262244	0.003	0.129	1.65278984344683e-99	EpC1	NCCRP1
LGALS7	8.28914468199551e-104	-0.777975966	0.103	0.297	1.66164194295282e-99	EpC1	LGALS7
NDUFB3	1.26979289186122e-103	-0.344259775	0.432	0.724	2.54542683102501e-99	EpC1	NDUFB3

FMO2	1.32322580339142e-103	-0.825130631	0.061	0.234	2.65253844547845e-99	EpC1	FMO2
NDRG2	1.40324337796601e-103	-0.288303546	0.047	0.216	2.81294167547067e-99	EpC1	NDRG2
MKI67	1.71893674878559e-103	-0.609179575	0.031	0.185	3.4457806066156e-99	EpC1	MKI67
SPRR2E	2.03327080333651e-103	-1.552915124	0.009	0.143	4.07589465236838e-99	EpC1	SPRR2E
ZFAS1	2.47498422261281e-103	0.515292577860397	0.765	0.661	4.96135337264963e-99	EpC1	ZFAS1
ELOVL6	5.73552869903112e-103	-0.28234226	0.055	0.229	1.14974408300778e-98	EpC1	ELOVL6
CLIP1	1.11427779136787e-102	-0.32397909	0.13	0.34	2.23368126057603e-98	EpC1	CLIP1
ARPC5L	1.48259078377921e-102	-0.293225072	0.151	0.374	2.97200148516381e-98	EpC1	ARPC5L
SRD5A3	3.39397279750291e-102	-0.281009404	0.044	0.209	6.80355786987433e-98	EpC1	SRD5A3
TRIM29	8.84358438470258e-102	-0.388594962	0.41	0.672	1.77278492575748e-97	EpC1	TRIM29
RHEB	9.84058242030856e-102	-0.305942898	0.135	0.346	1.97264315197505e-97	EpC1	RHEB
KRT42P	1.64216075025113e-101	0.568520226205237	0.79	0.613	3.29187543995342e-97	EpC1	KRT42P
MTCH2	4.30959338074192e-101	-0.339203476	0.261	0.512	8.63901089103526e-97	EpC1	MTCH2
DSP	8.68290909221122e-101	-0.532829765	0.929	0.94	1.74057595662466e-96	EpC1	DSP
GADD45A	1.15796533688051e-100	-0.419271605	0.288	0.542	2.32125731431068e-96	EpC1	GADD45A
SPDL1	1.24125857387704e-100	-0.367096576	0.096	0.289	2.48822693719392e-96	EpC1	SPDL1
MXD1	1.30548236125603e-100	-0.520316718	0.072	0.247	2.61696994137384e-96	EpC1	MXD1
SYNGR2	1.46565707989322e-100	-0.339694151	0.277	0.53	2.93805618235396e-96	EpC1	SYNGR2
CD59	1.60157801893826e-100	-0.458399351	0.254	0.485	3.21052329676364e-96	EpC1	CD59
SOD2	1.66729369472335e-100	-0.789460846	0.202	0.408	3.34225694044244e-96	EpC1	SOD2
REXO2	5.92099630869728e-100	-0.342727419	0.229	0.466	1.18692292004146e-95	EpC1	REXO2
RPS3	6.39687461990194e-100	0.337054285177166	0.999	0.98	1.28231748630554e-95	EpC1	RPS3
EIF6	1.04391168907256e-99	-0.395464557	0.346	0.597	2.09262537191486e-95	EpC1	EIF6
SPCS3	1.06124569778594e-99	-0.315382235	0.207	0.442	2.12737312578169e-95	EpC1	SPCS3
TTK	1.12040356642159e-99	-0.405567551	0.012	0.144	2.24596098924872e-95	EpC1	TTK
DBNL	1.38191296041321e-99	-0.270430743	0.091	0.282	2.77018272044433e-95	EpC1	DBNL
ME1	1.63032053131383e-99	-0.323616911	0.11	0.307	3.2681405370717e-95	EpC1	ME1
CARHSP1	1.72553955244828e-99	-0.314686377	0.073	0.253	3.45901658683782e-95	EpC1	CARHSP1
TNIP3	2.4003812468469e-99	-0.446145917	0.02	0.162	4.81180424742929e-95	EpC1	TNIP3

SEC11C	2.55360877128004e-99	-0.291567453	0.112	0.314	5.11896414290798e-95	EpC1	SEC11C
PLS3	3.06150782991432e-99	-0.373858464	0.24	0.475	6.13709859584624e-95	EpC1	PLS3
CREG1	3.11801724517803e-99	-0.329528996	0.161	0.373	6.25037736968387e-95	EpC1	CREG1
SCD	4.01757025923114e-99	-0.270630689	0.037	0.194	8.05362134165474e-95	EpC1	SCD
ERG28	4.10673098132045e-99	-0.308403284	0.134	0.342	8.23235292515497e-95	EpC1	ERG28
TMBIM1	4.43059667594127e-99	-0.362867426	0.336	0.593	8.88157409659186e-95	EpC1	TMBIM1
ABRACL	6.39606473112292e-99	-0.321622675	0.226	0.468	1.2821551360009e-94	EpC1	ABRACL
RDH10	6.92492708844599e-99	-0.449199708	0.029	0.176	1.38817088414988e-94	EpC1	RDH10
THBD	1.70237908789206e-98	-0.251246578	0.017	0.157	3.41258911958843e-94	EpC1	THBD
RRM1	1.9199596650749e-98	-0.391342865	0.182	0.4	3.84875114460914e-94	EpC1	RRM1
BIRC5	5.88440774083736e-98	-0.391652448	0.016	0.151	1.17958837572826e-93	EpC1	BIRC5
HNRNPA1	1.31945143314692e-97	0.371271926978581	0.96	0.859	2.64497234288631e-93	EpC1	HNRNPA1
RPN2	1.5091759126393e-97	-0.329744086	0.356	0.622	3.02529403447673e-93	EpC1	RPN2
TNFSF10	1.64481050642158e-97	-1.043965188	0.347	0.561	3.29718714117269e-93	EpC1	TNFSF10
TMEM165	2.25647615279493e-97	-0.281136216	0.133	0.339	4.52333209589272e-93	EpC1	TMEM165
TMSB4X	2.52026697746813e-97	-0.462696355	0.996	0.996	5.0521271830326e-93	EpC1	TMSB4X
TUBB2A	3.19326502165298e-97	-0.325517256	0.134	0.338	6.40121906240556e-93	EpC1	TUBB2A
CLDND1	3.77530849921552e-97	-0.310354463	0.194	0.419	7.56798341752744e-93	EpC1	CLDND1
COX5A	4.37122967494925e-97	-0.390826936	0.539	0.791	8.76256700640326e-93	EpC1	COX5A
RAC1	7.01211215426138e-97	-0.447364716	0.419	0.659	1.40564800244324e-92	EpC1	RAC1
APPL2	1.11057915868539e-96	-0.282495345	0.087	0.272	2.22626698150073e-92	EpC1	APPL2
SPINT1	1.20399938540678e-96	-0.331384954	0.117	0.311	2.41353716798643e-92	EpC1	SPINT1
MYL6	1.42627775967681e-96	-0.331771571	0.979	0.987	2.85911639704813e-92	EpC1	MYL6
ADIPOR1	1.96563944974655e-96	-0.318759962	0.149	0.357	3.94032084096193e-92	EpC1	ADIPOR1
PTPN13	4.07804054946861e-96	-0.377500625	0.205	0.427	8.17484008546478e-92	EpC1	PTPN13
ZNF706	5.86093908282404e-96	-0.376853795	0.405	0.668	1.17488384854291e-91	EpC1	ZNF706
CENPF	9.12412344779627e-96	-0.71222568	0.035	0.185	1.82902178634524e-91	EpC1	CENPF
DLG1	1.8470679556883e-95	-0.443662095	0.11	0.3	3.70263242397276e-91	EpC1	DLG1
UBE2C	4.75958596638963e-95	-0.574590029	0.007	0.129	9.54106602822465e-91	EpC1	UBE2C

SPRR2F	5.98510446371694e-95	-1.732237525	0.009	0.134	1.1997740407967e-90	EpC1	SPRR2F
ROMO1	8.03924607901805e-95	-0.34203841	0.43	0.697	1.61154726899996e-90	EpC1	ROMO1
RAET1L	8.4454808270856e-95	-0.262218572	0.036	0.188	1.69298108659758e-90	EpC1	RAET1L
RPS3A	9.00849947056547e-95	0.34684009704598	0.998	0.965	1.80584380386955e-90	EpC1	RPS3A
GARS	9.36647287162712e-95	-0.285979962	0.111	0.305	1.87760315184637e-90	EpC1	GARS
TMEM141	1.05521316181392e-94	-0.296193138	0.149	0.358	2.11528030417218e-90	EpC1	TMEM141
ZDHHC12	1.78431477578282e-94	-0.274181342	0.104	0.295	3.57683739953424e-90	EpC1	ZDHHC12
SERF2	2.52929723461348e-94	-0.320702618	0.959	0.987	5.07022923650618e-90	EpC1	SERF2
SAR1B	2.53331457179953e-94	-0.275360839	0.135	0.338	5.07828239062933e-90	EpC1	SAR1B
WFDC21P	3.20750072662612e-94	-0.569014988	0.009	0.133	6.42975595659473e-90	EpC1	WFDC21P
PNPLA8	5.52883266099559e-94	-0.31094332	0.141	0.345	1.10830979522318e-89	EpC1	PNPLA8
PPP1CB	5.97993552724931e-94	-0.301860436	0.243	0.479	1.1987378757924e-89	EpC1	PPP1CB
MINOS1	6.91742244716431e-94	-0.332676803	0.296	0.537	1.38666650375856e-89	EpC1	MINOS1
RPL23A	6.95723492717087e-94	0.41689212991514	0.986	0.899	1.39464731350067e-89	EpC1	RPL23A
MRPL13	8.37155595835582e-94	-0.313686185	0.304	0.561	1.67816210741201e-89	EpC1	MRPL13
TMEM45A	8.80808847337135e-94	-0.445907947	0.03	0.172	1.76566941537202e-89	EpC1	TMEM45A
B3GNT5	1.83483058342406e-93	-0.271539491	0.073	0.246	3.67810138753188e-89	EpC1	B3GNT5
SMC4	4.20706744322933e-93	-0.462502344	0.08	0.251	8.43348739669752e-89	EpC1	SMC4
IL13RA1	5.0312213533191e-93	-0.26278375	0.049	0.208	1.00855863248635e-88	EpC1	IL13RA1
SAA1	8.49676625715369e-93	-1.031641932	0.025	0.163	1.70326176390903e-88	EpC1	SAA1
PPDPF	8.57973777721391e-93	-0.477297612	0.669	0.846	1.7198942348203e-88	EpC1	PPDPF
BTG3	1.53979137784877e-92	-0.314298821	0.177	0.393	3.08666579603564e-88	EpC1	BTG3
H2AFY	8.56766890209079e-92	-0.313861284	0.267	0.507	1.71747490811312e-87	EpC1	H2AFY
POMP	1.94655238430093e-91	-0.351223285	0.513	0.763	3.90205890956964e-87	EpC1	POMP
ATOX1	3.79954837305332e-91	-0.341320691	0.307	0.551	7.61657466862269e-87	EpC1	ATOX1
CFH	4.1412781488018e-91	-0.526819325	0.037	0.181	8.30160617708809e-87	EpC1	CFH
RPL23	4.17643614496179e-91	0.345831495153704	0.996	0.971	8.3720838961904e-87	EpC1	RPL23
DAPL1	4.61585753235292e-91	-0.444960124	0.174	0.383	9.25294800935465e-87	EpC1	DAPL1
DSTN	4.86855977192997e-91	-0.386751686	0.713	0.875	9.75951491881082e-87	EpC1	DSTN

SNRPG	6.7893850571066e-91	-0.403926626	0.513	0.761	1.36100012854759e-86	EpC1	SNRPG
MIEN1	1.1577953138081e-90	-0.313455889	0.301	0.552	2.32091648605972e-86	EpC1	MIEN1
SH3GLB1	1.19494347852696e-90	-0.321246667	0.268	0.508	2.39538369705515e-86	EpC1	SH3GLB1
TP63	1.42227090913949e-90	0.459175451830288	0.656	0.513	2.85108426446102e-86	EpC1	TP63
ANXA8L1	1.67313498801582e-90	-0.256631424	0.079	0.254	3.35396639697652e-86	EpC1	ANXA8L1
GALNT1	2.37216058739518e-90	-0.381421774	0.083	0.254	4.75523311349237e-86	EpC1	GALNT1
HMGN2	2.58418554466082e-90	-0.58372344	0.371	0.574	5.18025834282707e-86	EpC1	HMGN2
VPS29	3.17296186086556e-90	-0.339199119	0.4	0.656	6.36051934629111e-86	EpC1	VPS29
PBK	3.2683908203364e-90	-0.282825335	0.007	0.124	6.55181623844635e-86	EpC1	PBK
HS3ST1	4.70428416136859e-90	-0.42162762	0.019	0.148	9.43020802987948e-86	EpC1	HS3ST1
UBE2V2	8.45527008035832e-90	-0.31866759	0.298	0.541	1.69494344030863e-85	EpC1	UBE2V2
NDUFA4	8.69768809913456e-90	-0.363184306	0.965	0.969	1.74353855635251e-85	EpC1	NDUFA4
ELOB	1.11005973786858e-89	-0.336840898	0.758	0.913	2.22522575053136e-85	EpC1	ELOB
TXNDC17	1.43641030378931e-89	-0.462496173	0.499	0.747	2.87942809497604e-85	EpC1	TXNDC17
CHMP4B	3.00154607722598e-89	-0.265230229	0.145	0.345	6.0168992664072e-85	EpC1	CHMP4B
RPL31	3.7822010419502e-89	0.27900574431097	1	0.998	7.58180020869338e-85	EpC1	RPL31
CXCL1	4.62258160811099e-89	-2.273671573	0.085	0.248	9.26642709161928e-85	EpC1	CXCL1
MAP3K8	5.05790063295178e-89	-0.260932974	0.054	0.211	1.01390676088151e-84	EpC1	MAP3K8
MTPN	5.28836729887291e-89	-0.355845274	0.257	0.48	1.06010610873206e-84	EpC1	MTPN
S100A12	9.08991008583735e-89	-0.698823688	0.009	0.126	1.82216337580696e-84	EpC1	S100A12
CTAGE5	9.84925787972236e-89	-0.279134145	0.108	0.293	1.97438223456914e-84	EpC1	CTAGE5
PSMA5	1.2171115780405e-88	-0.29112779	0.226	0.451	2.43982186934e-84	EpC1	PSMA5
UBL5	1.32826059619726e-88	-0.365360751	0.649	0.859	2.66263119113704e-84	EpC1	UBL5
BCAP29	4.05993537994364e-88	-0.270669784	0.128	0.32	8.13854646263503e-84	EpC1	BCAP29
ANLN	6.84426446873213e-88	-0.31474581	0.013	0.135	1.37200125540204e-83	EpC1	ANLN
HM13	1.04461476141682e-87	-0.302064679	0.169	0.371	2.09403475073616e-83	EpC1	HM13
COX7B	1.31886038822864e-87	-0.386725761	0.768	0.882	2.64378753424313e-83	EpC1	COX7B
PCLAF	1.52787624211642e-87	-0.392040162	0.027	0.161	3.06278071494658e-83	EpC1	PCLAF
SUB1	1.84890500090028e-87	-0.3647501	0.613	0.836	3.7063149648047e-83	EpC1	SUB1

CEP55	2.56763062900865e-87	-0.281763939	0.006	0.119	5.14707235891074e-83	EpC1	CEP55
TPM3	2.6847993670026e-87	-0.362128782	0.469	0.727	5.3819488110934e-83	EpC1	TPM3
IL1A	5.25345206871431e-87	-0.54652943	0.015	0.138	1.05310700169447e-82	EpC1	IL1A
KIF11	1.20582201788914e-86	-0.28980946	0.018	0.143	2.41719081706057e-82	EpC1	KIF11
IVL	1.23773249021187e-86	-0.302370149	0.004	0.112	2.48115854987872e-82	EpC1	IVL
HDAC1	1.32454014158627e-86	-0.301329076	0.242	0.467	2.65517316782383e-82	EpC1	HDAC1
SEC61G	1.37380751341558e-86	-0.317032225	0.405	0.66	2.75393454139288e-82	EpC1	SEC61G
ARF4	4.8095641896466e-86	-0.318515529	0.382	0.633	9.64125237456558e-82	EpC1	ARF4
MZT2A	4.99578541865147e-86	-0.273922999	0.136	0.328	1.00145514502287e-81	EpC1	MZT2A
EFHD2	1.55878325489592e-85	-0.265672457	0.071	0.234	3.12473691276436e-81	EpC1	EFHD2
FKBP1A	2.0616626264588e-85	-0.472710109	0.538	0.735	4.13280890099931e-81	EpC1	FKBP1A
BZW1	2.6576877074155e-85	-0.309921725	0.344	0.593	5.32760077828511e-81	EpC1	BZW1
IDI1	3.24832616806984e-85	-0.307098793	0.176	0.379	6.5115946365128e-81	EpC1	IDI1
EMC3	7.20365746446324e-85	-0.27475615	0.183	0.389	1.4440451753263e-80	EpC1	EMC3
MRPS15	1.05035619423412e-84	-0.286497711	0.231	0.453	2.10554402696172e-80	EpC1	MRPS15
CDK1	1.07554223937614e-84	-0.389644878	0.012	0.128	2.15603197305341e-80	EpC1	CDK1
RPL30	1.68331276537559e-84	0.473939339680255	0.82	0.688	3.37436876947191e-80	EpC1	RPL30
MT1X	1.88584460916279e-84	-0.621584162	0.676	0.785	3.78036410352773e-80	EpC1	MT1X
PSMD8	1.95431001772029e-84	-0.316501918	0.383	0.631	3.91760986152209e-80	EpC1	PSMD8
INSIG1	2.89782454824234e-84	-0.294213057	0.07	0.229	5.8089790894066e-80	EpC1	INSIG1
GPR87	3.4068180386293e-84	-0.251079813	0.114	0.298	6.8293074402363e-80	EpC1	GPR87
PDZD11	3.51732266907133e-84	-0.278908217	0.18	0.386	7.05082502242039e-80	EpC1	PDZD11
RNF181	4.25813972230534e-84	-0.272258152	0.249	0.475	8.53586688733328e-80	EpC1	RNF181
VEGFA	5.50692137745165e-84	-0.279745012	0.051	0.199	1.10391745932396e-79	EpC1	VEGFA
PRPF4	1.02195594528267e-83	-0.297766547	0.175	0.376	2.04861288791363e-79	EpC1	PRPF4
HK1	1.25493045162837e-83	-0.303732035	0.268	0.493	2.51563358333423e-79	EpC1	HK1
SRA1	1.39652412037763e-83	-0.261593174	0.133	0.323	2.79947225170901e-79	EpC1	SRA1
VPS25	2.41650870858955e-83	-0.250090953	0.125	0.312	4.84413335723861e-79	EpC1	VPS25
ATP6V1D	4.01294809871463e-83	-0.284023906	0.232	0.452	8.04435575868335e-79	EpC1	ATP6V1D

ASPM	5.39659527978292e-83	-0.307273696	0.007	0.116	1.08180148978528e-78	EpC1	ASPM
RPL12	5.52982162100668e-83	0.339951459472405	0.996	0.941	1.108508042147e-78	EpC1	RPL12
TMEM167A	5.87424983470433e-83	-0.295510073	0.27	0.497	1.17755212186483e-78	EpC1	TMEM167A
CRNDE	6.00987946685435e-83	-0.30006863	0.166	0.363	1.20474043792562e-78	EpC1	CRNDE
DLGAP5	6.30660979030689e-83	-0.325450586	0.005	0.111	1.26422299856492e-78	EpC1	DLGAP5
MRPL14	6.97925320102242e-83	-0.303294262	0.274	0.501	1.39906109667695e-78	EpC1	MRPL14
DCXR	7.45903978774646e-83	-0.297989373	0.201	0.407	1.49523911585165e-78	EpC1	DCXR
SERPINB6	8.10150837597153e-83	-0.276316302	0.102	0.276	1.62402836904725e-78	EpC1	SERPINB6
TK1	9.52463296749591e-83	-0.299285725	0.014	0.131	1.90930792466423e-78	EpC1	TK1
SNHG8	1.14743280059183e-82	0.518527903039559	0.675	0.562	2.30014379206638e-78	EpC1	SNHG8
HMMR	1.23768167900022e-82	-0.289900695	0.005	0.112	2.48105669372384e-78	EpC1	HMMR
ACSL1	1.37223332401724e-82	-0.259571082	0.095	0.266	2.75077892132496e-78	EpC1	ACSL1
CDKN3	1.8679538753206e-82	-0.405713265	0.017	0.136	3.74450033846768e-78	EpC1	CDKN3
NDUFA3	2.65627779370554e-82	-0.290788567	0.355	0.603	5.32477446526212e-78	EpC1	NDUFA3
SPTSSA	3.21393339223042e-82	-0.336919656	0.308	0.534	6.4426508780651e-78	EpC1	SPTSSA
UCHL3	3.28884666618978e-82	-0.263885577	0.12	0.3	6.59282202704404e-78	EpC1	UCHL3
CASP1	3.99874295234456e-82	-0.257760134	0.1	0.272	8.01588012226991e-78	EpC1	CASP1
AURKAIP1	4.35867411664185e-82	-0.283787067	0.295	0.531	8.73739813422025e-78	EpC1	AURKAIP1
GPRC5A	5.23382740851119e-82	-0.36036464	0.019	0.138	1.04917304231015e-77	EpC1	GPRC5A
S100A8	6.23675992678824e-82	-4.295836622	0.162	0.31	1.25022089492397e-77	EpC1	S100A8
CENPU	8.17685287154233e-82	-0.275878861	0.028	0.157	1.63913192662938e-77	EpC1	CENPU
GLO1	9.2030268925301e-82	-0.286817744	0.317	0.555	1.84483877087658e-77	EpC1	GLO1
ETHE1	1.15614698986813e-81	-0.336958388	0.32	0.548	2.31761225588965e-77	EpC1	ETHE1
CRYAB	1.36104792815263e-81	-0.768206807	0.34	0.556	2.72835667677477e-77	EpC1	CRYAB
CCNB2	2.12537375816078e-81	-0.269528746	0.006	0.111	4.2605242356091e-77	EpC1	CCNB2
MORF4L2	2.7217117298208e-81	-0.293630441	0.38	0.631	5.45594333359878e-77	EpC1	MORF4L2
PSMB6	3.53160589228987e-81	-0.342320897	0.492	0.739	7.07945717168426e-77	EpC1	PSMB6
FAM96B	6.69398098938713e-81	-0.360092806	0.345	0.572	1.34187542913254e-76	EpC1	FAM96B
TMEM94	7.20096992158731e-81	0.40515258964196	0.253	0.097	1.44350643048139e-76	EpC1	TMEM94

MAD2L1	2.38198961634239e-80	-0.261632543	0.024	0.147	4.77493638491995e-76	EpC1	MAD2L1
MX1	4.34993471966154e-80	-0.293912071	0.049	0.193	8.71987913903352e-76	EpC1	MX1
ATP5ME	5.1066296787878e-80	-0.348374222	0.489	0.715	1.0236749854098e-75	EpC1	ATP5ME
H2AFV	1.20903997212415e-79	-0.406003347	0.246	0.451	2.42364152812008e-75	EpC1	H2AFV
YBX3	1.45216226333812e-79	-0.293912687	0.338	0.579	2.91100447308759e-75	EpC1	YBX3
PSMB3	2.30171975643707e-79	-0.297796717	0.445	0.705	4.61402742375376e-75	EpC1	PSMB3
HMGB3	6.3538331010082e-79	-0.404085192	0.247	0.448	1.2736893834281e-74	EpC1	HMGB3
CSF3	7.35575034917081e-79	-0.550998491	0.009	0.115	1.47453371499478e-74	EpC1	CSF3
ACSL3	7.73242262786919e-79	-0.2609693	0.165	0.357	1.55004143998266e-74	EpC1	ACSL3
GNG5	9.22428084074702e-79	-0.325855866	0.529	0.781	1.84909933733615e-74	EpC1	GNG5
BSG	1.04965001346372e-78	-0.288192335	0.349	0.593	2.10412841698937e-74	EpC1	BSG
NDUFB8	1.35110024575037e-78	-0.289423942	0.306	0.535	2.7084155526312e-74	EpC1	NDUFB8
FAM162A	1.96104397159377e-78	-0.311460082	0.344	0.575	3.93110874545688e-74	EpC1	FAM162A
ETFA	2.62442448437082e-78	-0.269239313	0.236	0.449	5.26092132136975e-74	EpC1	ETFA
SERINC2	3.25233853585684e-78	-0.365355793	0.175	0.361	6.51963782897863e-74	EpC1	SERINC2
PRSS23	3.38864239377465e-78	-0.280290077	0.121	0.298	6.79287254256066e-74	EpC1	PRSS23
GNAI3	4.66178223242887e-78	-0.262975886	0.239	0.453	9.34500866312692e-74	EpC1	GNAI3
KYNU	4.84599533683708e-78	-0.313297667	0.006	0.108	9.71428225222361e-74	EpC1	KYNU
ERO1A	5.91793452428838e-78	-0.283913361	0.041	0.175	1.18630915473885e-73	EpC1	ERO1A
F2RL1	6.18436066943165e-78	-0.256327367	0.105	0.275	1.23971693979427e-73	EpC1	F2RL1
NDUFS6	7.22974721432359e-78	-0.311883189	0.406	0.646	1.44927512658331e-73	EpC1	NDUFS6
GJB5	8.78663141232102e-78	-0.260323864	0.152	0.339	1.76136813291387e-73	EpC1	GJB5
HMGCR	1.00117178179764e-77	-0.262826835	0.109	0.278	2.00694895379155e-73	EpC1	HMGCR
GGH	1.32927675139641e-77	-0.256470043	0.086	0.246	2.66466817584925e-73	EpC1	GGH
TMEM50A	1.4872856761169e-77	-0.277657163	0.365	0.605	2.98141286634393e-73	EpC1	TMEM50A
CST3	1.60711349147077e-77	-0.320844933	0.378	0.616	3.2216197050023e-73	EpC1	CST3
NAMPT	2.49728270056543e-77	-0.274303258	0.142	0.322	5.00605290155346e-73	EpC1	NAMPT
LCN2	5.08714967516377e-77	-1.957350376	0.085	0.231	1.01977002388333e-72	EpC1	LCN2
TIMM17A	8.86479707816537e-77	-0.27067589	0.199	0.399	1.77703722228903e-72	EpC1	TIMM17A

HSPA4	9.66134548469678e-77	-0.258312282	0.212	0.42	1.93671331586232e-72	EpC1	HSPA4
CDC20	1.25378523257711e-76	-0.388048494	0.024	0.143	2.51333787722407e-72	EpC1	CDC20
SEC61B	1.68980558850426e-76	-0.287887579	0.502	0.755	3.38738428271564e-72	EpC1	SEC61B
TMTC3	1.91474282732942e-76	-0.271552153	0.196	0.39	3.83829347166456e-72	EpC1	TMTC3
COPE	2.31015400184231e-76	-0.266867154	0.287	0.511	4.6309347120931e-72	EpC1	COPE
HEPHL1	2.96012641043896e-76	-0.391488513	0.004	0.1	5.93386940236593e-72	EpC1	HEPHL1
ELOC	2.98662992384038e-76	-0.309441768	0.437	0.68	5.98699834533042e-72	EpC1	ELOC
KRT18	3.7259992454255e-76	-0.353302994	0.319	0.548	7.46913808737996e-72	EpC1	KRT18
HLA-C	3.87706666664649e-76	-0.311522921	0.169	0.355	7.77196783995956e-72	EpC1	HLA-C
ZNF770	5.64731322801178e-76	-0.264838942	0.21	0.411	1.13206040968724e-71	EpC1	ZNF770
SELENOS	5.68044194858688e-76	-0.268223448	0.265	0.485	1.13870139301373e-71	EpC1	SELENOS
MRPS10	8.10161189575838e-76	-0.260234331	0.184	0.378	1.62404912062373e-71	EpC1	MRPS10
ATP5MF	1.1819990452216e-75	-0.353635208	0.721	0.871	2.36943528605123e-71	EpC1	ATP5MF
NDUFS8	1.65003283066883e-75	-0.276077261	0.319	0.549	3.30765581235873e-71	EpC1	NDUFS8
KDELR2	1.89618266813174e-75	-0.262904663	0.25	0.46	3.80108777653688e-71	EpC1	KDELR2
ATP5MD	1.98637049198543e-75	-0.349905761	0.624	0.822	3.98187828823398e-71	EpC1	ATP5MD
VDAC3	3.39613012693557e-75	-0.27590428	0.222	0.426	6.80788245245504e-71	EpC1	VDAC3
CDH13	3.42502066712106e-75	0.368719517961952	0.322	0.158	6.86579642931087e-71	EpC1	CDH13
TUBB4B	3.45653427050666e-75	-0.342926534	0.262	0.47	6.92896859865764e-71	EpC1	TUBB4B
FAM136A	4.46216783163415e-75	-0.283795982	0.193	0.384	8.94486163529381e-71	EpC1	FAM136A
PRSS22	6.09452263000547e-75	-1.205101258	0.054	0.188	1.2217080064109e-70	EpC1	PRSS22
RPL9	1.36144838314121e-74	0.297016630619775	0.997	0.982	2.72915942884488e-70	EpC1	RPL9
GSN	2.15676500575974e-74	0.481127688142884	0.626	0.5	4.32345113054598e-70	EpC1	GSN
MDM2	2.51734585061493e-74	-0.29375127	0.151	0.329	5.04627149214269e-70	EpC1	MDM2
EMC2	3.28176693021319e-74	-0.252686603	0.195	0.391	6.57862998830536e-70	EpC1	EMC2
PTP4A1	3.64428885930984e-74	-0.257294704	0.203	0.402	7.3053414473725e-70	EpC1	PTP4A1
SUCLG1	8.3477079704779e-74	-0.368283569	0.631	0.824	1.673381539762e-69	EpC1	SUCLG1
LSM4	1.28702035572405e-73	-0.276250355	0.205	0.398	2.57996100508443e-69	EpC1	LSM4
CNIH4	1.55941423734892e-73	-0.268305606	0.317	0.543	3.12600178018964e-69	EpC1	CNIH4

KRT15	1.68848384358719e-73	0.378100366538236	0.278	0.117	3.38473471285487e-69	EpC1	KRT15
NCOA7	1.80485680570792e-73	-0.776032258	0.171	0.339	3.6180159527221e-69	EpC1	NCOA7
ADM	1.83526597147385e-73	-0.372526869	0.151	0.328	3.67897416641649e-69	EpC1	ADM
FOS	3.06443884658282e-73	0.445005451462366	0.975	0.918	6.14297411185992e-69	EpC1	FOS
MZT2B	4.62088270260903e-73	-0.256320183	0.223	0.427	9.26302146565007e-69	EpC1	MZT2B
S100A13	5.00413010811755e-73	-0.334996158	0.189	0.375	1.00312792147324e-68	EpC1	S100A13
NCOA4	6.46109373949539e-73	-0.259079233	0.237	0.444	1.29519085101925e-68	EpC1	NCOA4
ABI1	8.25140589062833e-73	-0.258430309	0.24	0.444	1.65407682483536e-68	EpC1	ABI1
UBE2T	1.36672944185897e-72	-0.279906959	0.039	0.163	2.7397458391505e-68	EpC1	UBE2T
CARD18	1.53178604390184e-72	-0.251908665	0.032	0.154	3.07061830360563e-68	EpC1	CARD18
P4HB	2.76373279958915e-72	-0.26253681	0.177	0.366	5.54017877005642e-68	EpC1	P4HB
EIF3I	3.80105621667396e-72	-0.304587632	0.482	0.723	7.61959729194463e-68	EpC1	EIF3I
WDR61	5.37040164858621e-72	-0.262437857	0.203	0.394	1.07655071447559e-67	EpC1	WDR61
MRPL52	1.70606708653419e-71	-0.265578802	0.306	0.528	3.41998208166644e-67	EpC1	MRPL52
RPS15	2.53689891490653e-71	0.279182658819614	0.999	0.987	5.08546756482164e-67	EpC1	RPS15
SMAP1	3.79542234885057e-71	-0.33713011	0.228	0.417	7.60830364050585e-67	EpC1	SMAP1
CHMP2A	2.50573242559736e-70	-0.260533478	0.291	0.502	5.02299122035248e-66	EpC1	CHMP2A
NDUFC2	3.14651554108951e-70	-0.286809699	0.331	0.546	6.30750505366802e-66	EpC1	NDUFC2
CNFN	3.9381305286013e-70	-1.30777222	0.115	0.269	7.89437645763416e-66	EpC1	CNFN
SRI	4.4848903494098e-70	-0.329270204	0.344	0.552	8.99041119442689e-66	EpC1	SRI
RACK1	5.1394000213758e-70	0.306988698054949	0.992	0.956	1.03024412828499e-65	EpC1	RACK1
SREK1IP1	5.411793387278e-70	-0.261718067	0.195	0.385	1.08484810241375e-65	EpC1	SREK1IP1
PFDN5	5.95614557523572e-70	0.376059953887267	0.869	0.834	1.19396894201175e-65	EpC1	PFDN5
LTA4H	7.67163195250033e-70	-0.296892255	0.172	0.351	1.53785534119822e-65	EpC1	LTA4H
FTH1	1.70920311878779e-69	0.598087403015944	0.738	0.599	3.42626857192201e-65	EpC1	FTH1
PSMD11	2.15053681471173e-69	-0.26194334	0.263	0.468	4.31096609877113e-65	EpC1	PSMD11
RPL7	2.34019834281823e-69	0.255912813136738	0.998	0.992	4.69116159801343e-65	EpC1	RPL7
IGFBP5	2.353477789941e-69	0.282530206966067	0.365	0.188	4.71778157771574e-65	EpC1	IGFBP5
GHITM	2.35486073232191e-69	-0.280249133	0.405	0.638	4.7205538240125e-65	EpC1	GHITM

PSMA3	2.41659136677727e-69	-0.288527035	0.331	0.548	4.84429905384172e-65	EpC1	PSMA3
HMGA1	2.56440182011736e-69	-0.278255041	0.255	0.456	5.14059988860726e-65	EpC1	HMGA1
ACAT2	3.66737574765621e-69	-0.285633698	0.066	0.204	7.35162142375164e-65	EpC1	ACAT2
NET1	4.00938796049404e-69	-0.304441142	0.189	0.366	8.03721910560636e-65	EpC1	NET1
HHIP-AS1	6.27254797550815e-69	0.344296913239438	0.186	0.061	1.25739496717036e-64	EpC1	HHIP-AS1
CXCL6	6.8159926753761e-69	-0.496559029	0.009	0.104	1.36633389170589e-64	EpC1	CXCL6
VAMP5	8.04859100046881e-69	-0.310791235	0.036	0.155	1.61342055195398e-64	EpC1	VAMP5
CALM1	1.64152793790984e-68	-0.306829774	0.953	0.971	3.29060690433407e-64	EpC1	CALM1
SLIRP	2.58370715613582e-68	-0.318528902	0.483	0.711	5.17929936518987e-64	EpC1	SLIRP
CD55	2.70847563063579e-68	-0.379776672	0.171	0.34	5.42941024917251e-64	EpC1	CD55
ARL5B	3.90788936893428e-68	-0.287704538	0.118	0.277	7.83375502896565e-64	EpC1	ARL5B
C4orf3	7.93781311345893e-68	-0.337550127	0.514	0.743	1.59121401672398e-63	EpC1	C4orf3
BLOC1S2	1.55251473871923e-67	-0.256015777	0.258	0.458	3.11217104523657e-63	EpC1	BLOC1S2
TPI1	2.10609342885002e-67	-0.378933627	0.898	0.914	4.22187488747276e-63	EpC1	TPI1
CAP1	2.40509803669427e-67	-0.309873775	0.197	0.378	4.82125952435733e-63	EpC1	CAP1
SCPEP1	2.46423737696612e-67	-0.306602373	0.193	0.369	4.93981024586629e-63	EpC1	SCPEP1
SDHB	2.57117595488792e-67	-0.268301259	0.303	0.513	5.15417931916832e-63	EpC1	SDHB
PLS1	3.0264159786774e-67	-0.311349902	0.083	0.225	6.06675347085671e-63	EpC1	PLS1
ATP5PD	3.84330960395914e-67	-0.314768438	0.798	0.9	7.70429843209649e-63	EpC1	ATP5PD
VDAC1	5.66412906516563e-67	-0.314952921	0.509	0.728	1.1354313124031e-62	EpC1	VDAC1
RPL10	8.50360276921653e-67	0.285560115014464	0.996	0.979	1.70463221111715e-62	EpC1	RPL10
ECT2	1.60514473692219e-66	-0.250625258	0.033	0.147	3.21767313963423e-62	EpC1	ECT2
CCT5	2.69405792289168e-66	-0.308949242	0.306	0.507	5.40050851222866e-62	EpC1	CCT5
EMP1	3.43024710516793e-66	-0.600497276	0.622	0.781	6.87627334701963e-62	EpC1	EMP1
ARF1	4.3857718183158e-66	-0.259241311	0.52	0.768	8.79171818699586e-62	EpC1	ARF1
GSTM4	7.93097013254463e-66	-0.26911081	0.11	0.262	1.5898422727699e-61	EpC1	GSTM4
ANAPC11	1.69820068787293e-65	-0.297683861	0.476	0.702	3.40421309891008e-61	EpC1	ANAPC11
ODC1	1.92930232518869e-65	-0.510925559	0.404	0.578	3.86747944107324e-61	EpC1	ODC1
PDCD5	3.27551346423914e-65	-0.32993842	0.469	0.675	6.56609429041378e-61	EpC1	PDCD5

MRPL36	3.81370031907512e-65	-0.261379075	0.259	0.459	7.64494365961798e-61	EpC1	MRPL36
CENPX	5.32512502207293e-65	-0.318610877	0.232	0.417	1.06747456192474e-60	EpC1	CENPX
NFKBIZ	5.42236895625237e-65	-0.49013956	0.281	0.465	1.08696808097035e-60	EpC1	NFKBIZ
SPINT2	1.07024957523376e-64	-0.279239814	0.703	0.87	2.1454222985136e-60	EpC1	SPINT2
CFL1	1.15172560032316e-64	-0.278025362	0.815	0.926	2.30874913840781e-60	EpC1	CFL1
CCL20	1.41281036508492e-64	-0.861174832	0.028	0.134	2.83211965784923e-60	EpC1	CCL20
BAG3	1.53293313067143e-64	-0.251912167	0.207	0.388	3.07291775374395e-60	EpC1	BAG3
CCT6A	1.57122385294392e-64	-0.274742457	0.392	0.61	3.14967533561137e-60	EpC1	CCT6A
CTNNAL1	1.84932789871657e-64	0.261201489988136	0.251	0.106	3.70716270576724e-60	EpC1	CTNNAL1
UGCG	1.85764912953032e-64	-0.25976442	0.065	0.195	3.72384344505648e-60	EpC1	UGCG
TGM3	6.68455383071973e-64	-0.847388523	0.393	0.541	1.33998566090608e-59	EpC1	TGM3
CA2	1.43732973838328e-63	-0.365239078	0.052	0.175	2.88127119356311e-59	EpC1	CA2
COX6A1	3.36842796736788e-63	-0.323895033	0.891	0.927	6.75235070338566e-59	EpC1	COX6A1
SAMD9	4.07688629275174e-63	-0.301276433	0.057	0.181	8.17252626245014e-59	EpC1	SAMD9
HIST1H4C	6.48206857338472e-63	-0.443129587	0.727	0.594	1.2993954662207e-58	EpC1	HIST1H4C
BTF3	8.10254189044034e-63	0.295321572550968	0.986	0.964	1.62423554735767e-58	EpC1	BTF3
LSM3	1.39659796812142e-62	-0.289343586	0.352	0.557	2.79962028689621e-58	EpC1	LSM3
ARPC5	1.45131432195512e-62	-0.278463376	0.472	0.699	2.90930468979124e-58	EpC1	ARPC5
ATP5MC1	3.31318229917428e-62	-0.313148499	0.474	0.673	6.64160523692476e-58	EpC1	ATP5MC1
IL1B	3.49371823246998e-62	-0.65978878	0.026	0.129	7.00350756880933e-58	EpC1	IL1B
ACTN4	1.93633777081204e-61	-0.262436412	0.296	0.497	3.88158269536982e-57	EpC1	ACTN4
EZR	2.45655809228177e-61	-0.292252262	0.583	0.803	4.92441635178804e-57	EpC1	EZR
PSME2	2.96859075343739e-61	-0.274132184	0.281	0.475	5.9508370243406e-57	EpC1	PSME2
ZFP36L2	5.25141610429819e-61	0.38140741967107	0.393	0.24	1.05269887226762e-56	EpC1	ZFP36L2
GJA1	6.53350900691833e-61	-0.333667792	0.349	0.537	1.30970721552685e-56	EpC1	GJA1
PTPRZ1	9.54059726803288e-61	-0.267896696	0.236	0.418	1.91250812834987e-56	EpC1	PTPRZ1
PGK1	1.34257993415864e-60	-0.259793365	0.421	0.644	2.69133573601441e-56	EpC1	PGK1
UQCR11	1.46169716140309e-60	-0.287674256	0.531	0.755	2.93011812974864e-56	EpC1	UQCR11
COX5B	1.98207762661249e-60	-0.304936538	0.894	0.938	3.97327281030739e-56	EpC1	COX5B

TSTD1	2.13725923541193e-60	-0.279927291	0.396	0.611	4.28434986330675e-56	EpC1	TSTD1
ILF2	4.80714473409181e-60	-0.283368063	0.341	0.535	9.63640233396045e-56	EpC1	ILF2
CD9	1.13375919802072e-59	-0.367112237	0.988	0.983	2.27273368835234e-55	EpC1	CD9
ZFAND5	1.23474607309101e-59	-0.275383377	0.229	0.403	2.47517197811823e-55	EpC1	ZFAND5
IFI16	2.47895996246177e-59	-0.337813276	0.256	0.44	4.96932314075086e-55	EpC1	IFI16
CXCL2	2.60593049429319e-59	-0.445409494	0.045	0.158	5.22384826886012e-55	EpC1	CXCL2
KIF20B	4.10748732375269e-59	-0.265667122	0.042	0.152	8.23386908919465e-55	EpC1	KIF20B
ITGB1	4.42026535915643e-59	0.405528065000241	0.553	0.433	8.86086393896498e-55	EpC1	ITGB1
CIRBP	4.91734441744072e-59	0.412816139508542	0.625	0.525	9.85730861920168e-55	EpC1	CIRBP
YWHAB	7.22691874162486e-59	-0.276812906	0.576	0.797	1.44870813094612e-54	EpC1	YWHAB
GPX2	8.36769428418711e-59	-0.667764745	0.679	0.695	1.67738799620815e-54	EpC1	GPX2
PITX2	1.07331776628103e-58	-0.307270387	0.141	0.287	2.15157279428694e-54	EpC1	PITX2
AKR1B1	1.92684931289723e-58	-0.324794932	0.156	0.308	3.86256213263378e-54	EpC1	AKR1B1
RPL4	2.58355204689952e-58	0.288551927101814	0.97	0.905	5.17898843321478e-54	EpC1	RPL4
CCNB1	3.06847683031145e-58	-0.545926601	0.047	0.156	6.15106865404234e-54	EpC1	CCNB1
PSMD7	5.45283886646158e-58	-0.252734582	0.158	0.316	1.09307607917089e-53	EpC1	PSMD7
TFRC	6.06093725921904e-58	-0.463704698	0.178	0.333	1.21497548298305e-53	EpC1	TFRC
FGFBP1	7.12600226230654e-58	-0.260784279	0.135	0.286	1.42847841350197e-53	EpC1	FGFBP1
PA2G4	4.47285971371163e-57	-0.296293397	0.378	0.571	8.96629458210633e-53	EpC1	PA2G4
SSR4	5.2763717984644e-57	-0.254531228	0.533	0.77	1.05770149072017e-52	EpC1	SSR4
SLC25A5	7.65160668755523e-57	-0.463230762	0.924	0.875	1.53384107658732e-52	EpC1	SLC25A5
RPS11	8.59251286413384e-57	0.27383904624228	0.994	0.965	1.72245512874427e-52	EpC1	RPS11
CD47	1.22811908464573e-56	-0.279122419	0.117	0.257	2.46188751708082e-52	EpC1	CD47
TCIM	1.63931743256196e-56	-0.266515022	0.029	0.127	3.28617572531371e-52	EpC1	TCIM
VPS4B	2.7778753772361e-56	-0.253604852	0.374	0.574	5.56852898120748e-52	EpC1	VPS4B
TIPARP	4.29028869807609e-56	-0.293162558	0.152	0.3	8.60031272416334e-52	EpC1	TIPARP
PHLDA1	4.68897405663321e-56	-0.368719642	0.114	0.252	9.39951739392693e-52	EpC1	PHLDA1
APRT	9.80587201905118e-56	-0.317693849	0.491	0.685	1.965685104939e-51	EpC1	APRT
CHCHD2	2.05109026951386e-55	-0.269840758	0.759	0.889	4.11161555426749e-51	EpC1	CHCHD2

RPS6	2.11291567582001e-55	0.250481947358947	0.999	0.982	4.23555076374879e-51	EpC1	RPS6
EIF2S2	7.97574368794911e-55	-0.250120178	0.476	0.699	1.59881757968628e-50	EpC1	EIF2S2
EIF4A2	8.98403244183447e-55	0.328857743099451	0.758	0.704	1.80093914329014e-50	EpC1	EIF4A2
TMEM14A	1.50372847201976e-54	-0.3733315	0.363	0.533	3.01437409501081e-50	EpC1	TMEM14A
FAM46A	1.90059821169383e-54	-0.275486436	0.062	0.177	3.80993917516146e-50	EpC1	FAM46A
C9orf16	2.34957965076736e-54	-0.266566793	0.386	0.583	4.70996736792824e-50	EpC1	C9orf16
CD14	8.58312187308882e-54	-0.494891499	0.017	0.101	1.72057261067939e-49	EpC1	CD14
CLIC1	1.14117424665497e-53	-0.290165404	0.631	0.818	2.28759789484455e-49	EpC1	CLIC1
C1QBP	1.95271280197567e-53	-0.296008949	0.347	0.523	3.91440808284043e-49	EpC1	C1QBP
NDUFAB1	2.0362825811929e-53	-0.254621221	0.469	0.681	4.08193206225928e-49	EpC1	NDUFAB1
RPL22L1	5.86101075447988e-53	-0.253886419	0.297	0.478	1.17489821584304e-48	EpC1	RPL22L1
DKK3	7.17703824194948e-53	0.320728099938486	0.374	0.23	1.43870908598119e-48	EpC1	DKK3
RAB31	9.18681988063589e-53	-0.308977216	0.064	0.177	1.84158991327227e-48	EpC1	RAB31
PLK2	3.39144615779013e-52	-0.295926065	0.318	0.5	6.7984929679061e-48	EpC1	PLK2
PLP2	4.25766037768569e-52	-0.28078073	0.788	0.892	8.53490599310874e-48	EpC1	PLP2
MT-CO2	6.80945304833355e-51	-0.279861913	1	1	1.36502295806894e-46	EpC1	MT-CO2
CKAP2	8.33478576066318e-51	-0.443930219	0.162	0.298	1.67079115358254e-46	EpC1	CKAP2
SLC25A6	1.14693134888997e-50	0.291921387049252	0.906	0.83	2.29913858198483e-46	EpC1	SLC25A6
COX6B1	1.93715847089103e-50	-0.252146468	0.92	0.954	3.88322787074817e-46	EpC1	COX6B1
FXYD3	2.93784469609363e-50	-0.332228648	0.918	0.941	5.8892034777893e-46	EpC1	FXYD3
TNFAIP3	3.1898658970346e-50	-0.385337926	0.266	0.424	6.39440517719556e-46	EpC1	TNFAIP3
AKR1C1	3.29593304722155e-50	-0.284039289	0.141	0.281	6.60702738646033e-46	EpC1	AKR1C1
CD46	1.74453260359674e-49	-0.328159872	0.437	0.622	3.49709005717002e-45	EpC1	CD46
COX8A	1.88761969372663e-49	-0.290737878	0.764	0.874	3.78392243804439e-45	EpC1	COX8A
NDUFB2	4.29563651873114e-49	-0.295596366	0.659	0.8	8.61103296544845e-45	EpC1	NDUFB2
RPS25	5.8236274795282e-49	0.261806133307032	0.974	0.916	1.16740436454622e-44	EpC1	RPS25
RAMP1	7.22009875768646e-49	0.271325654366288	0.256	0.129	1.44734099696583e-44	EpC1	RAMP1
RAN	8.25638638825584e-49	-0.327974493	0.68	0.809	1.65507521538977e-44	EpC1	RAN
HMGN1	8.86943408621326e-49	-0.264425945	0.525	0.725	1.77796675692231e-44	EpC1	HMGN1

IGFBP7	1.16661444697323e-48	0.25263411975746	0.252	0.126	2.33859532040253e-44	EpC1	IGFBP7
KLF6	1.18997687810384e-48	-0.364284167	0.518	0.697	2.38542764984696e-44	EpC1	KLF6
KRT17	3.02255859012773e-48	-0.526933125	0.997	0.963	6.05902094977004e-44	EpC1	KRT17
RPL37	9.28675611660699e-48	0.257182103676156	0.999	0.993	1.86162313113504e-43	EpC1	RPL37
H2AFZ	5.33805425817124e-47	-0.752582022	0.722	0.818	1.07006635659301e-42	EpC1	H2AFZ
KPNA2	6.32125360414368e-47	-0.363530688	0.147	0.28	1.26715849748664e-42	EpC1	KPNA2
HLA-B	6.68822077065252e-47	-0.266406609	0.16	0.296	1.340720735685e-42	EpC1	HLA-B
S100A4	1.62590636084145e-46	-0.616873128	0.179	0.317	3.25929189094277e-42	EpC1	S100A4
RPL26	1.69206773792207e-46	0.279283054970578	0.931	0.849	3.39191898743858e-42	EpC1	RPL26
BUB3	1.76532518942377e-46	-0.258071787	0.284	0.448	3.5387708747189e-42	EpC1	BUB3
HPGD	2.24534164527381e-46	-0.26573652	0.136	0.267	4.50101186211588e-42	EpC1	HPGD
RND3	2.88815245419124e-46	-0.297530922	0.363	0.534	5.78959040967177e-42	EpC1	RND3
JPT1	3.68849030935714e-46	-0.29041909	0.502	0.693	7.39394767413732e-42	EpC1	JPT1
PRDX1	9.67217876309918e-46	-0.480472389	0.636	0.791	1.93888495485086e-41	EpC1	PRDX1
HSPH1	1.4649879825614e-45	-0.274702408	0.291	0.452	2.93671490984258e-41	EpC1	HSPH1
TNFAIP8	2.30235095165009e-45	-0.277162447	0.197	0.342	4.61529271767778e-41	EpC1	TNFAIP8
ATP5PF	6.21681402807261e-45	-0.253395003	0.828	0.916	1.24622254006744e-40	EpC1	ATP5PF
MT-ND6	1.28662292961928e-44	-0.418168426	0.838	0.771	2.57916432471481e-40	EpC1	MT-ND6
NDUFB9	2.35672458053316e-44	-0.258789633	0.602	0.782	4.72429009413676e-40	EpC1	NDUFB9
ANP32E	2.82360597514528e-44	-0.268229988	0.182	0.317	5.66020053777622e-40	EpC1	ANP32E
VSNL1	5.9815017239135e-44	-0.445515101	0.724	0.782	1.1990518355757e-39	EpC1	VSNL1
JUN	8.24481668869918e-44	0.340466006835894	0.92	0.864	1.65275595341664e-39	EpC1	JUN
TSPO	1.39701616561331e-43	-0.333276865	0.791	0.858	2.80045860558843e-39	EpC1	TSPO
CXCL3	3.57261756022459e-43	-0.627966116	0.046	0.136	7.16166916122622e-39	EpC1	CXCL3
RANBP1	6.2809873698948e-43	-0.290512131	0.369	0.536	1.25908672816911e-38	EpC1	RANBP1
CALM2	7.38860474965219e-43	-0.369473193	0.812	0.911	1.48111970811528e-38	EpC1	CALM2
EEF2	3.10936419050426e-41	0.329007465868311	0.714	0.693	6.23303145628484e-37	EpC1	EEF2
PLSCR1	4.86547241009363e-41	-0.250091009	0.154	0.277	9.75332599327368e-37	EpC1	PLSCR1
ABLIM1	9.08672611695154e-41	-0.271648771	0.288	0.43	1.82152511740411e-36	EpC1	ABLIM1

SDC2	3.49086610170359e-40	0.269726275466901	0.268	0.151	6.99779018747501e-36	EpC1	SDC2
CYB5A	9.15660133742771e-40	-0.266909214	0.405	0.574	1.83553230410076e-35	EpC1	CYB5A
PDLIM1	1.35107191266686e-39	0.348417461767873	0.607	0.555	2.70835875613198e-35	EpC1	PDLIM1
RPL9P9	1.48504575548605e-38	0.341494171699487	0.626	0.538	2.97692272144733e-34	EpC1	RPL9P9
RPS15A	2.23191864225639e-38	0.252576760087018	0.942	0.871	4.47410411026716e-34	EpC1	RPS15A
TMPO	2.80900580208435e-38	-0.313335831	0.248	0.377	5.63093303085828e-34	EpC1	TMPO
ARL6IP1	4.03252096538909e-38	-0.469459338	0.349	0.501	8.08359152721897e-34	EpC1	ARL6IP1
DUSP1	4.95876744711352e-38	-0.318593378	0.534	0.683	9.94034522448377e-34	EpC1	DUSP1
SMC2	8.84719555323635e-38	-0.283257171	0.161	0.276	1.77350882060176e-33	EpC1	SMC2
TPM4	1.87176019262021e-37	-0.2576126	0.721	0.857	3.75213048212647e-33	EpC1	TPM4
TUBA1C	2.10956917221782e-37	-0.407747741	0.128	0.231	4.22884236262783e-33	EpC1	TUBA1C
JUNB	3.07437480189724e-36	0.380752031659893	0.751	0.74	6.1628917278832e-32	EpC1	JUNB
HIST1H1B	6.87999566530649e-35	-0.673701402	0.039	0.111	1.37916393106734e-30	EpC1	HIST1H1B
NFKBIA	2.5895372397373e-34	-0.510200159	0.701	0.749	5.19098635077739e-30	EpC1	NFKBIA
FDPS	7.46679724961038e-34	-0.291964487	0.143	0.251	1.4967941766569e-29	EpC1	FDPS
UBB	1.1265255288004e-33	0.284454807871272	0.893	0.864	2.25823307503328e-29	EpC1	UBB
CKS2	1.43560440872503e-33	-0.318873834	0.165	0.275	2.87781259773019e-29	EpC1	CKS2
TXNIP	3.50988118703738e-32	0.308165091754007	0.53	0.442	7.03590782753512e-28	EpC1	TXNIP
HSPA6	1.00951119413628e-30	-0.291808932	0.07	0.152	2.02366613976558e-26	EpC1	HSPA6
EIF3E	4.1053173689018e-30	0.283602736943843	0.684	0.662	8.22951919770054e-26	EpC1	EIF3E
FAM210B	5.73950853475804e-30	0.41725015319067	0.396	0.312	1.1505418808776e-25	EpC1	FAM210B
LRRC75A-AS1	9.68464185351276e-30	0.376802773610955	0.476	0.374	1.94138330595517e-25	EpC1	LRRC75A-AS1
IFI27	6.11676936340296e-29	-0.464937326	0.054	0.126	1.22616758658776e-24	EpC1	IFI27
NR4A1	1.02564002323695e-28	0.373396222617133	0.288	0.194	2.0559979905808e-24	EpC1	NR4A1
LUM	3.74870542001431e-28	-0.356981917	0.11	0.198	7.51465488496069e-24	EpC1	LUM
PCNA	1.41790956708564e-27	-0.251084687	0.207	0.312	2.84234151817988e-23	EpC1	PCNA
CKS1B	3.28269774371068e-27	-0.332341372	0.201	0.298	6.58049589704244e-23	EpC1	CKS1B
SLC7A8	3.30022978136519e-27	0.271501439946361	0.354	0.26	6.61564061972467e-23	EpC1	SLC7A8
MT1F	4.08278264996162e-27	0.252370956976017	0.249	0.155	8.18434610011307e-23	EpC1	MT1F

HSPA5	4.45642117138841e-27	-0.266857591	0.679	0.803	8.93334188016521e-23	EpC1	HSPA5
SRSF11	3.15103586279321e-26	0.263572431643565	0.782	0.776	6.31656649055527e-22	EpC1	SRSF11
HES1	3.36481849942194e-26	-0.273625959	0.46	0.598	6.74511516394122e-22	EpC1	HES1
PCNT	6.89403567014025e-26	0.262129152055761	0.225	0.141	1.38197839043631e-21	EpC1	PCNT
CAST	6.04691355082459e-25	0.250868058649489	0.837	0.849	1.2121642903983e-20	EpC1	CAST
ALDH3A1	2.80467389652207e-24	-0.423957291	0.596	0.654	5.62224929296813e-20	EpC1	ALDH3A1
ASS1	4.81100024401406e-24	-0.283850429	0.116	0.198	9.64413108915058e-20	EpC1	ASS1
DCN	1.55559795559315e-23	-1.025049915	0.168	0.248	3.11835166178202e-19	EpC1	DCN
RAD21	2.14146864199739e-23	-0.252383259	0.403	0.525	4.29278803974796e-19	EpC1	RAD21
HSP90AA1	8.71848248282615e-23	-0.2658672	0.916	0.93	1.74770699850733e-18	EpC1	HSP90AA1
PTMA	7.29921343552736e-22	-0.287124277	1	0.996	1.46320032528581e-17	EpC1	PTMA
S100A6	8.4015015554854e-22	-0.427101072	0.998	0.994	1.6841650018126e-17	EpC1	S100A6
IGKC	9.86773400251972e-22	-0.575534553	0.044	0.102	1.9780859581451e-17	EpC1	IGKC
ITGA2	2.56767019424196e-21	0.264394233890617	0.602	0.572	5.14715167137743e-17	EpC1	ITGA2
ST13	2.59503247562807e-21	0.266064070679575	0.56	0.534	5.20200210064403e-17	EpC1	ST13
SON	3.88575341507668e-21	0.254006835810504	0.749	0.767	7.78938129586271e-17	EpC1	SON
VCAN	1.40580561703462e-19	0.314775509517394	0.353	0.278	2.8180779399076e-15	EpC1	VCAN
EEF1D	2.2111097921355e-19	0.29911276948436	0.332	0.252	4.43239068931482e-15	EpC1	EEF1D
MT2A	3.72456579190019e-19	-0.444528149	0.442	0.532	7.46626458644312e-15	EpC1	MT2A
AKAP9	7.33848803695384e-18	0.295066707542987	0.519	0.479	1.47107331188777e-13	EpC1	AKAP9
MAGED2	9.91949573991152e-18	0.260317409912371	0.409	0.346	1.98846211602266e-13	EpC1	MAGED2
EDN1	1.42721292548878e-17	0.255883505909376	0.199	0.131	2.8609910304348e-13	EpC1	EDN1
GLUL	3.76878448305954e-17	-0.81726263	0.749	0.825	7.55490537474115e-13	EpC1	GLUL
NTS	3.77045550973656e-17	-1.143849113	0.331	0.371	7.5582551148179e-13	EpC1	NTS
ARID5B	1.16648075175036e-15	0.25181069045778	0.482	0.437	2.33832731495878e-11	EpC1	ARID5B
XPA	2.01616284052894e-15	0.281712440251285	0.537	0.512	4.04160003012431e-11	EpC1	XPA
UGP2	2.3050639264629e-14	0.25164931335499	0.536	0.517	4.62073114698753e-10	EpC1	UGP2
SLC3A2	9.81500653587775e-14	0.257927484687356	0.471	0.444	1.96751621018205e-09	EpC1	SLC3A2
B2M	6.96329998103592e-13	-0.552492779	0.988	0.969	1.39586311419846e-08	EpC1	B2M

KRT19	1.75443761174929e-12	-0.427630807	0.701	0.707	3.51694563651263e-08	EpC1	KRT19
LAMC2	2.10107220013193e-11	-0.256156201	0.123	0.174	4.21180933238447e-07	EpC1	LAMC2
CD74	2.33235111678039e-11	-0.292017382	0.217	0.281	4.67543104869797e-07	EpC1	CD74
SPARCL1	1.04228370912724e-10	-0.578304287	0.072	0.112	2.08936192331647e-06	EpC1	SPARCL1
ATF3	1.27793947488059e-10	0.270639918546053	0.588	0.564	2.56175747134564e-06	EpC1	ATF3
SELENOP	2.13405767512099e-10	0.273004508365933	0.342	0.306	4.27793201554753e-06	EpC1	SELENOP
CRABP2	3.20569830591109e-06	-0.375363695	0.236	0.269	0.0642614282402938	EpC1	CRABP2
HIST1H1D	0.000139427562607705	-0.275919356	0.105	0.129	1	EpC1	HIST1H1D
TUBA1B	0.00138386913202947	-0.829714009	0.274	0.284	1	EpC1	TUBA1B
KRTDAP.1	0	3.08070583276062	0.568	0.096	0	EpC4	KRTDAP
KRT6B.1	0	2.68672792461564	0.855	0.242	0	EpC4	KRT6B
GSTA1.1	0	2.53213152458617	0.732	0.24	0	EpC4	GSTA1
CSTA.1	0	2.21297047630744	0.947	0.524	0	EpC4	CSTA
KRT6A.1	0	2.09700449859959	0.988	0.887	0	EpC4	KRT6A
GBP6.1	0	1.96657863019799	0.932	0.301	0	EpC4	GBP6
PLAC8.1	0	1.92218207830141	0.876	0.227	0	EpC4	PLAC8
SPINK5.1	0	1.80267437028192	0.576	0.132	0	EpC4	SPINK5
GSTM3.1	0	1.766805417	0.792	0.301	0	EpC4	GSTM3
DBI.1	0	1.73105695392954	0.987	0.803	0	EpC4	DBI
DMKN.1	0	1.64222913551282	0.76	0.334	0	EpC4	DMKN
TGM3.1	0	1.58288961130679	0.813	0.366	0	EpC4	TGM3
DYNLT3.1	0	1.52488431494047	0.952	0.462	0	EpC4	DYNLT3
TXN.1	0	1.52349042253952	1	0.988	0	EpC4	TXN
DSC2.1	0	1.49330135206368	0.971	0.634	0	EpC4	DSC2
GPX2.1	0	1.4189225327373	0.962	0.6	0	EpC4	GPX2
PERP.1	0	1.39153988376197	1	0.994	0	EpC4	PERP
CALML3.1	0	1.36920786053578	0.902	0.361	0	EpC4	CALML3
POLR2J3.1	0	1.34445478388799	0.769	0.27	0	EpC4	POLR2J3
KRT17.1	0	1.32190834522158	0.998	0.972	0	EpC4	KRT17

CSTB.1	0	1.29063626579632	0.994	0.925	0	EpC4	CSTB
LYPD3.1	0	1.27781823548004	0.95	0.445	0	EpC4	LYPD3
C5orf46.1	0	1.2654617332657	0.407	0.052	0	EpC4	C5orf46
AKR1C3.1	0	1.23983491723727	0.907	0.495	0	EpC4	AKR1C3
AKR1C2.1	0	1.22660609805364	0.864	0.332	0	EpC4	AKR1C2
ALDH3A1.1	0	1.20734767587725	0.9	0.54	0	EpC4	ALDH3A1
ADH7.1	0	1.19656999161515	0.864	0.324	0	EpC4	ADH7
SFN.1	0	1.17694438842726	0.989	0.789	0	EpC4	SFN
MAL2.1	0	1.15940741730162	0.779	0.242	0	EpC4	MAL2
GSTP1.1	0	1.13479580194026	1	0.998	0	EpC4	GSTP1
KLK11.1	0	1.1237624592119	0.819	0.296	0	EpC4	KLK11
S100A11.1	0	1.12233654396532	1	0.997	0	EpC4	S100A11
LY6G6C.1	0	1.08853773685138	0.457	0.066	0	EpC4	LY6G6C
SERPINB13.1	0	1.05076182538428	0.794	0.299	0	EpC4	SERPINB13
S100A16.1	0	1.01333284711529	0.964	0.632	0	EpC4	S100A16
SULT2B1.1	0	1.01008924469602	0.678	0.162	0	EpC4	SULT2B1
FKBP1A.1	0	0.969868730134086	0.922	0.557	0	EpC4	FKBP1A
SERPINB5.1	0	0.964656718660925	0.967	0.704	0	EpC4	SERPINB5
MGLL.1	0	0.939275433595621	0.683	0.223	0	EpC4	MGLL
ANKRD22.1	0	0.924855480155311	0.578	0.069	0	EpC4	ANKRD22
TMEM154.1	0	0.92261005858127	0.637	0.193	0	EpC4	TMEM154
TMPRSS11D.1	0	0.909198031503836	0.838	0.353	0	EpC4	TMPRSS11D
DSG1.1	0	0.882829161619934	0.464	0.054	0	EpC4	DSG1
CALML5.1	0	0.881955808123053	0.34	0.031	0	EpC4	CALML5
POF1B.1	0	0.86320645107937	0.576	0.108	0	EpC4	POF1B
MUC15.1	0	0.860708986180604	0.595	0.139	0	EpC4	MUC15
EPHB6.1	0	0.821966064495104	0.643	0.175	0	EpC4	EPHB6
CD9.1	0	0.812591035299917	0.996	0.982	0	EpC4	CD9
SERPINB11.1	0	0.775206939068817	0.4	0.049	0	EpC4	SERPINB11

YWHAZ.1	0	0.712536854544809	0.997	0.942	0	EpC4	YWHAZ
TMEM40.1	0	0.652665163341816	0.502	0.096	0	EpC4	TMEM40
KCNK7.1	0	0.604344302548618	0.42	0.061	0	EpC4	KCNK7
DEGS2.1	0	0.603570358685187	0.393	0.028	0	EpC4	DEGS2
SUSD4.1	0	0.571552106880321	0.436	0.052	0	EpC4	SUSD4
COL17A1.1	0	-1.51277561	0.052	0.585	0	EpC4	COL17A1
SCEL.1	2.04641234821556e-305	0.950297661702331	0.617	0.18	4.10223819323292e-301	EpC4	SCEL
HSPB1.1	1.25976140638929e-304	0.79446638675792	0.999	0.981	2.52531771524796e-300	EpC4	HSPB1
CTNNBIP1.1	2.76697398709276e-302	0.770776810276815	0.546	0.146	5.54667605452615e-298	EpC4	CTNNBIP1
COX6C.1	1.54628041106762e-298	0.730705124323335	0.998	0.95	3.09967371202615e-294	EpC4	COX6C
CLCA2.1	5.28282561805676e-297	0.89776641871258	0.908	0.466	1.05899522339566e-292	EpC4	CLCA2
NQO1.1	5.15281138180347e-296	0.866285056964699	0.761	0.31	1.03293256959632e-291	EpC4	NQO1
DYNLL1.1	6.66298425821654e-293	0.820760888276264	0.987	0.9	1.33566182440209e-288	EpC4	DYNLL1
CLDN7.1	7.67567387980675e-293	0.778264905816031	0.734	0.269	1.53866558594606e-288	EpC4	CLDN7
RDH12	1.26044389582966e-288	0.438064089961667	0.268	0.015	2.52668583358014e-284	EpC4	RDH12
AKR1B10.1	1.32608028141743e-287	1.06167932682088	0.746	0.302	2.65826053212937e-283	EpC4	AKR1B10
TUBA4A.1	1.37202983986099e-280	0.861314731159216	0.754	0.329	2.75037101698534e-276	EpC4	TUBA4A
PRDX6.1	2.34576681567745e-280	0.968578078975857	0.827	0.453	4.70232415870702e-276	EpC4	PRDX6
ITM2B.1	2.73401378551303e-278	0.725699147731073	0.985	0.864	5.48060403443942e-274	EpC4	ITM2B
TSPO.1	9.32107864200762e-275	0.805360866308373	0.967	0.783	1.86850342457685e-270	EpC4	TSPO
TALDO1.1	6.06884327815649e-274	0.797329113736085	0.944	0.648	1.21656032353925e-269	EpC4	TALDO1
SMAP1.1	1.75635070319106e-273	0.718470626117756	0.654	0.228	3.52078061961679e-269	EpC4	SMAP1
SDCBP2.1	2.51620831581248e-270	0.622849536249004	0.458	0.102	5.04399118987769e-266	EpC4	SDCBP2
WFDC5.1	4.85066942199792e-268	0.519345142391111	0.282	0.024	9.72365192333703e-264	EpC4	WFDC5
EP400P1.1	1.15418635733046e-264	0.486932338885265	0.347	0.05	2.31368197190463e-260	EpC4	EP400P1
DIRAS3.1	5.41400960244532e-264	0.90524392690334	0.493	0.129	1.08529236490619e-259	EpC4	DIRAS3
GLTP.1	1.14292560267787e-263	0.844661257945997	0.776	0.347	2.29110866312806e-259	EpC4	GLTP
TST.1	2.91058574761708e-261	0.578688107895752	0.416	0.084	5.8345601896732e-257	EpC4	TST
CLDN4.1	5.19229320778011e-260	0.991058059367079	0.827	0.397	1.0408470964316e-255	EpC4	CLDN4

JUP.1	6.10318701401107e-257	0.806451197151251	0.834	0.447	1.22344486882866e-252	EpC4	JUP
B4GALT4.1	1.86463730703378e-255	0.523106412913398	0.43	0.091	3.73785194567992e-251	EpC4	B4GALT4
ANXA2.1	4.08829645861012e-251	0.661318589581269	0.999	0.991	8.19539908092986e-247	EpC4	ANXA2
DSP.1	9.28556156723948e-251	0.842586625102667	0.991	0.918	1.86138367176883e-246	EpC4	DSP
RAB25.1	2.35186841313435e-250	0.745349686268377	0.669	0.271	4.71455542096912e-246	EpC4	RAB25
CXCL14.1	3.93025447442728e-248	-2.25039429	0.32	0.686	7.87858811943693e-244	EpC4	CXCL14
FAM3B.1	2.9801105644297e-247	0.593640886586544	0.398	0.08	5.97392963745578e-243	EpC4	FAM3B
SPTSSB	1.68999096153593e-246	0.433490020549192	0.231	0.013	3.38775588149493e-242	EpC4	SPTSSB
PLEKHN1.1	1.22923017172626e-243	0.446966487929897	0.344	0.055	2.46411480224246e-239	EpC4	PLEKHN1
KRT16.1	1.19550078592414e-242	1.54327625951238	0.683	0.278	2.39650087546354e-238	EpC4	KRT16
TACSTD2.1	5.23441484599086e-240	0.412506719339419	0.947	0.559	1.04929080002733e-235	EpC4	TACSTD2
TIMM8B.1	8.59162834372597e-240	0.719201912998989	0.835	0.444	1.72227781778331e-235	EpC4	TIMM8B
ODC1.1	6.43261177692443e-238	0.944913667918289	0.79	0.407	1.28948135680227e-233	EpC4	ODC1
COA3.1	1.84046594845784e-236	0.713865993498788	0.771	0.373	3.68939804027859e-232	EpC4	COA3
ME1.1	6.07312291195429e-236	0.563892517610647	0.481	0.134	1.21741821893036e-231	EpC4	ME1
CDH1.1	3.38464153006773e-235	0.702957293063312	0.829	0.432	6.78485241117376e-231	EpC4	CDH1
GPNMB.1	2.33707348451145e-234	1.22423501416001	0.513	0.16	4.68489750705165e-230	EpC4	GPNMB
NUCB2.1	4.11621114368958e-234	0.803168141200394	0.64	0.258	8.25135685864014e-230	EpC4	NUCB2
ABLIM1.1	4.69760646171183e-233	0.669209451243743	0.669	0.269	9.41682191314753e-229	EpC4	ABLIM1
KLK10.1	5.8778587059997e-233	1.24152875435515	0.578	0.213	1.1782755562047e-228	EpC4	KLK10
PPDPF.1	7.12591012638039e-233	0.794219000576185	0.937	0.711	1.42845994393421e-228	EpC4	PPDPF
NAGK.1	3.79730028509418e-232	0.536648490394067	0.493	0.141	7.61206815149979e-228	EpC4	NAGK
DSTN.1	2.77420098957216e-231	0.663512670276471	0.957	0.752	5.56116330369635e-227	EpC4	DSTN
TMEM79.1	2.81953650142705e-231	0.446888503101814	0.355	0.065	5.65204287076066e-227	EpC4	TMEM79
KRT6C.1	5.33057593247932e-229	0.91954422707803	0.386	0.08	1.0685672514248e-224	EpC4	KRT6C
PSMA7.1	1.12112613133376e-227	0.629057439648343	0.995	0.975	2.24740944287166e-223	EpC4	PSMA7
CAPNS2	5.71058421071626e-227	0.78196265021314	0.795	0.41	1.14474371088018e-222	EpC4	CAPNS2
MGST2.1	4.60890583359453e-226	0.651293167231194	0.71	0.315	9.2390126340236e-222	EpC4	MGST2
DST.1	4.74175027684368e-226	-1.240977263	0.299	0.659	9.50531260496084e-222	EpC4	DST

CYP2C18	1.06468576413462e-224	0.376970435700732	0.283	0.036	2.13426908278426e-220	EpC4	CYP2C18
LAD1.1	4.70302916012796e-222	0.573897344412654	0.614	0.231	9.4276922543925e-218	EpC4	LAD1
COPS9.1	8.09492185783871e-221	0.720372927878973	0.862	0.521	1.62270803562235e-216	EpC4	COPS9
PKP1.1	1.48729291232971e-220	0.69980829845294	0.84	0.476	2.98142737205613e-216	EpC4	PKP1
LAMB3	1.9249039408031e-218	-0.955075726	0.069	0.468	3.85866243973389e-214	EpC4	LAMB3
FETUB	2.68979140559642e-216	0.303884957704162	0.195	0.009	5.39195585165858e-212	EpC4	FETUB
TM4SF1.1	8.79057500785738e-215	0.744370604068241	0.95	0.72	1.76215866607509e-210	EpC4	TM4SF1
TM7SF2	8.89028077290056e-215	0.429398241580845	0.322	0.056	1.78214568373565e-210	EpC4	TM7SF2
EMP2.1	1.23714465954026e-214	0.690574400427481	0.939	0.639	2.4799801845144e-210	EpC4	EMP2
DECR1.1	2.93872415814615e-212	0.717854514317801	0.785	0.419	5.89096644741977e-208	EpC4	DECR1
HIGD1A.1	4.39823004027805e-212	0.673777814670342	0.72	0.351	8.81669193874138e-208	EpC4	HIGD1A
LGALS7.1	4.70522788444908e-212	1.17624255495033	0.451	0.132	9.43209981716662e-208	EpC4	LGALS7
RAET1L.1	1.16301155784726e-211	0.438413183540349	0.319	0.055	2.33137296886062e-207	EpC4	RAET1L
ALDH1A1.1	2.668872767997e-210	0.608830672661767	0.399	0.098	5.35002235072678e-206	EpC4	ALDH1A1
DSG3.1	4.07073411585621e-210	0.946822656847883	0.947	0.695	8.16019360864536e-206	EpC4	DSG3
NFE2L2	3.32068445878448e-209	0.721257781996445	0.962	0.884	6.65664406607937e-205	EpC4	NFE2L2
COX6A1.1	3.91099895234998e-209	0.587761076568844	0.986	0.887	7.83998849988078e-205	EpC4	COX6A1
EIF6.1	6.2757347358059e-208	0.66370198659922	0.765	0.393	1.25803378513965e-203	EpC4	EIF6
SYTL5	1.21885677847693e-207	0.27724703242083	0.191	0.009	2.44332029813486e-203	EpC4	SYTL5
CARD18.1	2.09006524374136e-207	0.479764411326756	0.28	0.04	4.18974478760392e-203	EpC4	CARD18
DUSP22.1	2.12827220357614e-205	0.480799213091866	0.418	0.11	4.26633445928873e-201	EpC4	DUSP22
ENSA.1	1.24348724211686e-203	0.640045932055533	0.836	0.487	2.49269452554746e-199	EpC4	ENSA
RAB11A.1	2.46749224257244e-203	0.705054676952312	0.855	0.521	4.9463349494607e-199	EpC4	RAB11A
PIR.1	5.32083697685673e-201	0.500556789249003	0.553	0.194	1.0666149803807e-196	EpC4	PIR
LGMN.1	2.03032429303837e-200	0.535685247519741	0.492	0.159	4.06998807782472e-196	EpC4	LGMN
FAM129A	3.78106481371873e-198	0.387264086437321	0.266	0.037	7.57952252558058e-194	EpC4	FAM129A
PGD.1	5.03458470691878e-197	0.586010416222794	0.656	0.291	1.00923285034894e-192	EpC4	PGD
CTTNBP2	7.67191765421437e-197	0.38220714040448	0.262	0.036	1.53791261296381e-192	EpC4	CTTNBP2
PLEKHA5.1	2.83266607758536e-195	0.511280662684292	0.455	0.137	5.67836241912761e-191	EpC4	PLEKHA5

FDFT1.1	2.79739682288998e-192	0.638825330990677	0.632	0.273	5.60766167116525e-188	EpC4	FDFT1
MPZL2.1	1.13662269201265e-190	0.595053443845072	0.823	0.45	2.27847384840855e-186	EpC4	MPZL2
ELOVL6.1	1.37974497826357e-189	0.42053957768214	0.359	0.084	2.76583678342715e-185	EpC4	ELOVL6
SRI.1	1.9018025416339e-186	0.629690338236023	0.729	0.372	3.81235337495932e-182	EpC4	SRI
LRRC4	2.02920295863124e-185	0.308507615199234	0.211	0.02	4.06774025087218e-181	EpC4	LRRC4
SERF2.1	2.69224978325383e-185	0.497974182200464	0.995	0.967	5.39688391551063e-181	EpC4	SERF2
SERPINB3.1	1.46679464367136e-184	0.847557230327541	0.721	0.346	2.9403365427036e-180	EpC4	SERPINB3
GSTM4.1	2.05152570274217e-184	0.498855160233005	0.418	0.121	4.11248842371695e-180	EpC4	GSTM4
GRHL1.1	3.11966832885483e-184	0.50257053943727	0.45	0.142	6.25368713202239e-180	EpC4	GRHL1
COX7B.1	3.88717018209346e-184	0.569493905625118	0.973	0.785	7.79222134702456e-180	EpC4	COX7B
COX5B.1	6.44772155362749e-184	0.561745550155213	0.985	0.897	1.29251026264017e-179	EpC4	COX5B
ZNF750.1	7.58385995343336e-184	0.479842797228544	0.543	0.196	1.52026056626525e-179	EpC4	ZNF750
TMPRSS4.1	9.87770006188579e-184	0.508818172817983	0.604	0.246	1.98008375440563e-179	EpC4	TMPRSS4
HOPX.1	9.56222306560589e-182	1.1802174341659	0.674	0.317	1.91684323573136e-177	EpC4	HOPX
CD200	1.39417317167025e-181	0.504820025577677	0.351	0.085	2.79475953993018e-177	EpC4	CD200
GJB6.1	2.46376086760474e-181	0.51930220631656	0.465	0.149	4.93885503520047e-177	EpC4	GJB6
CYCS.1	9.19356705536216e-181	0.630763416241705	0.912	0.619	1.8429424519179e-176	EpC4	CYCS
NPR3	1.1885895420351e-180	0.418268612980424	0.371	0.093	2.38264659596356e-176	EpC4	NPR3
ATP5F1E.1	9.28469317513864e-180	0.576874419701318	0.977	0.897	1.86120959388829e-175	EpC4	ATP5F1E
VTCN1	9.71671520885345e-180	0.300023013880367	0.185	0.014	1.94781273076676e-175	EpC4	VTCN1
CYB5A.1	2.18273997843393e-177	0.665512726703364	0.775	0.409	4.37552056076866e-173	EpC4	CYB5A
DYNLT1.1	2.28320896838326e-176	0.618790652682693	0.852	0.525	4.57692069802108e-172	EpC4	DYNLT1
NUPR1	8.41074735140395e-176	0.623613575207904	0.811	0.458	1.68601841406244e-171	EpC4	NUPR1
PI3.1	4.65421898161493e-175	3.24777418585265	0.517	0.219	9.3298473705453e-171	EpC4	PI3
SPRR1A.1	1.17524031037955e-174	2.24484604712373	0.304	0.066	2.35588672618684e-170	EpC4	SPRR1A
S100A10.1	4.99881622135518e-174	0.595302083305756	0.996	0.975	1.00206269973286e-169	EpC4	S100A10
APRT.1	9.95951942984296e-173	0.645824943258531	0.83	0.523	1.99648526490632e-168	EpC4	APRT
SPRR1B.1	8.25950113097426e-172	1.7103425086939	0.657	0.36	1.6556995967151e-167	EpC4	SPRR1B
ACSL1.1	8.8464038490575e-172	0.408735485723881	0.406	0.119	1.77335011558207e-167	EpC4	ACSL1

FAM96B.1	1.51763247871064e-171	0.610164511240572	0.733	0.385	3.04224606682335e-167	EpC4	FAM96B
SLC25A5.1	6.23385301176816e-171	0.668803935832939	0.981	0.871	1.24963817473905e-166	EpC4	SLC25A5
CREG1.1	3.21184391645685e-169	0.5005909680016	0.514	0.201	6.43846231492941e-165	EpC4	CREG1
UGT1A7	4.99126001502876e-168	0.332316067274319	0.287	0.057	1.00054798261266e-163	EpC4	UGT1A7
EPHX1	7.57092489691173e-168	0.402256199771242	0.397	0.117	1.51766760483493e-163	EpC4	EPHX1
NECTIN4.1	1.80190502671815e-167	0.378827906833719	0.369	0.1	3.61209881655921e-163	EpC4	NECTIN4
GLRX3.1	2.29605950572688e-167	0.524240250389925	0.647	0.299	4.6026808851801e-163	EpC4	GLRX3
SOX21-AS1	2.51430513622169e-167	0.353244119757261	0.27	0.05	5.04017607606999e-163	EpC4	SOX21-AS1
COX5A.1	5.14801917174453e-166	0.573829031275453	0.913	0.602	1.03197192316791e-161	EpC4	COX5A
GJA1.1	9.47381376400978e-166	0.547954294051836	0.722	0.366	1.8991207071334e-161	EpC4	GJA1
ATP5MD.1	1.23992540592939e-165	0.574616896790989	0.931	0.668	2.48555446872606e-161	EpC4	ATP5MD
MAPK13.1	1.47142233239472e-165	0.413648920392376	0.459	0.156	2.94961320751846e-161	EpC4	MAPK13
NDUFA4.1	3.37309835887255e-165	0.511298543099599	0.991	0.96	6.76171297019591e-161	EpC4	NDUFA4
ATP5PD.1	1.18391256890792e-164	0.535260841409978	0.969	0.817	2.37327113563281e-160	EpC4	ATP5PD
UQCRQ.1	1.02617788882889e-162	0.540489498958448	0.968	0.812	2.0570761959464e-158	EpC4	UQCRQ
C4orf3.1	1.38247037511808e-162	0.617249976034989	0.868	0.566	2.7713001139617e-158	EpC4	C4orf3
SLC9A9	3.12398417838795e-162	0.416510312190788	0.395	0.12	6.26233868399648e-158	EpC4	SLC9A9
ARPC2.1	6.9040053035876e-162	0.509142562036135	0.987	0.926	1.38397690315717e-157	EpC4	ARPC2
SPTLC2	2.00019690854299e-161	0.498762997124588	0.57	0.244	4.00959472286528e-157	EpC4	SPTLC2
C9orf16.1	4.38618908293312e-160	0.579158068977615	0.746	0.414	8.79255463564773e-156	EpC4	C9orf16
HEBP2.1	4.78006200887636e-160	0.533048412570327	0.607	0.282	9.58211230299355e-156	EpC4	HEBP2
ZNF706.1	7.75069716457037e-160	0.552312906957649	0.808	0.467	1.55370475360978e-155	EpC4	ZNF706
TMEM14A.1	1.87250283459397e-159	0.656623463707991	0.712	0.374	3.75361918222708e-155	EpC4	TMEM14A
VSNL1.1	3.57244884045378e-159	0.720716463039949	0.919	0.704	7.16133094557364e-155	EpC4	VSNL1
AADAC	1.37886977132178e-158	0.361512160267767	0.251	0.045	2.76408234359164e-154	EpC4	AADAC
ALDH3B2	2.31667790806951e-155	0.287584636742444	0.232	0.038	4.64401253451615e-151	EpC4	ALDH3B2
COX7A2.1	6.00697202744713e-155	0.484015988900393	0.989	0.927	1.20415761262205e-150	EpC4	COX7A2
RDH11.1	1.56032652182242e-154	0.471081930234497	0.514	0.209	3.12783054564523e-150	EpC4	RDH11
SCD.1	4.32867382313008e-154	0.377862669900486	0.296	0.067	8.67725954584656e-150	EpC4	SCD

LIPH	4.50790698320644e-154	0.30844820149601	0.238	0.04	9.03655033853562e-150	EpC4	LIPH
PRSS23.1	7.90375642046723e-154	0.439877863341861	0.435	0.149	1.58438701204686e-149	EpC4	PRSS23
TRIM29.1	1.09654744393846e-153	0.563409856171777	0.791	0.478	2.19813900611904e-149	EpC4	TRIM29
MSMO1.1	1.2692073568774e-151	0.554772175631622	0.46	0.176	2.54425306759644e-147	EpC4	MSMO1
KRT14	5.47416428971411e-151	-0.651573478	0.548	0.895	1.09735097351609e-146	EpC4	KRT14
PFN1.1	1.52522799829717e-150	0.469086868169997	0.985	0.956	3.05747204538651e-146	EpC4	PFN1
HK1.1	1.98470245215977e-150	0.471115853696998	0.642	0.311	3.97853453559948e-146	EpC4	HK1
SGK1.1	5.03351859400689e-149	0.697398073682851	0.871	0.598	1.00901913735462e-144	EpC4	SGK1
SQOR.1	6.18498294892166e-149	0.434106966255286	0.398	0.129	1.23984168194084e-144	EpC4	SQOR
IDH1	1.26069997104361e-148	0.484180399377808	0.61	0.285	2.52719916195402e-144	EpC4	IDH1
TPI1.1	3.01315614021067e-147	0.521699236165029	0.974	0.886	6.04017279866631e-143	EpC4	TPI1
UQCR10.1	4.90715980653459e-147	0.506799249337261	0.969	0.807	9.83689254817924e-143	EpC4	UQCR10
COX6B1.1	5.09564143639792e-147	0.472998015383276	0.985	0.924	1.02147228234033e-142	EpC4	COX6B1
UBL5.1	3.38432361800979e-146	0.548786431413534	0.927	0.712	6.78421512466242e-142	EpC4	UBL5
SLC9A3R1.1	5.77700142039271e-146	0.5064471754159	0.738	0.408	1.15805770473192e-141	EpC4	SLC9A3R1
NDUFC2.1	8.90547999163504e-146	0.497448083173433	0.699	0.369	1.78519251912316e-141	EpC4	NDUFC2
BNIPL	1.22159585035713e-144	0.266152080889921	0.208	0.031	2.4488110416259e-140	EpC4	BNIPL
TMSB10.1	2.72327029822956e-144	0.448265415450987	0.997	0.997	5.45906763983097e-140	EpC4	TMSB10
ANXA3.1	5.06955283005473e-144	0.425492874684823	0.43	0.15	1.01624256031277e-139	EpC4	ANXA3
TSTD1.1	1.006017421448e-143	0.534861595597434	0.759	0.435	2.01666252303466e-139	EpC4	TSTD1
KANK1	1.64086318888937e-143	0.310415796294124	0.25	0.05	3.28927434844763e-139	EpC4	KANK1
ATP5ME.1	5.13071633853229e-143	0.510099759483681	0.851	0.536	1.02850339722218e-138	EpC4	ATP5ME
FAM84A.1	3.65380982158244e-142	0.439761726504055	0.483	0.194	7.32442716834416e-138	EpC4	FAM84A
UTRN	2.72690978106427e-141	0.549541405367787	0.617	0.301	5.46636334712144e-137	EpC4	UTRN
PHLDA2.1	3.35941983280319e-141	0.522870201314612	0.561	0.253	6.73429299683727e-137	EpC4	PHLDA2
NDUFB2.1	4.31784672650001e-141	0.528805982667703	0.919	0.677	8.65555554794192e-137	EpC4	NDUFB2
NOP10.1	5.65916211436461e-141	0.507068924837827	0.831	0.51	1.13443563744553e-136	EpC4	NOP10
SDC1.1	2.43354828152118e-139	0.484187738559013	0.605	0.293	4.87829088513735e-135	EpC4	SDC1
SBSN.1	1.05161244195058e-138	0.729655550098966	0.322	0.087	2.10806230113413e-134	EpC4	SBSN

APPL2.1	3.33398164065938e-138	0.364989255389664	0.383	0.126	6.68329959686579e-134	EpC4	APPL2
ZCCHC6.1	3.47229397407191e-138	0.421363036123425	0.481	0.194	6.96056050042454e-134	EpC4	ZCCHC6
ATP5PB	4.9430689189106e-138	0.496287175723169	0.917	0.67	9.90887595484819e-134	EpC4	ATP5PB
SAMD12	1.08299511536234e-137	0.462517327516792	0.562	0.258	2.17097200825535e-133	EpC4	SAMD12
FAM162A.1	2.14222907914249e-137	0.49836923306908	0.711	0.394	4.29431241204904e-133	EpC4	FAM162A
FAM135A.1	8.0624980780856e-137	0.392652539083568	0.383	0.128	1.61620836473304e-132	EpC4	FAM135A
GRHL3	1.58946336458034e-136	0.308193563047739	0.294	0.076	3.18623826063775e-132	EpC4	GRHL3
UQCR11.1	4.8928976197609e-135	0.530309805380953	0.865	0.586	9.8083025685727e-131	EpC4	UQCR11
EIF5A.1	1.27031534535585e-134	0.497885840281577	0.802	0.482	2.54647414130033e-130	EpC4	EIF5A
ATP1B1.1	1.3262800352582e-134	0.564515200294161	0.652	0.328	2.65866095867859e-130	EpC4	ATP1B1
ATP5MF.1	5.24028949009247e-134	0.49513965783749	0.947	0.757	1.05046843118394e-129	EpC4	ATP5MF
PDCD5.1	1.02588857253947e-133	0.503415814749581	0.82	0.506	2.05649623251262e-129	EpC4	PDCD5
ARL8B.1	1.71978494874145e-133	0.43425673475976	0.535	0.241	3.44748090824711e-129	EpC4	ARL8B
PRPF4.1	2.39976780891091e-132	0.424646303470856	0.505	0.215	4.8105745497428e-128	EpC4	PRPF4
UNC5B-AS1	2.45618713109117e-132	0.371313837021694	0.261	0.061	4.92367272298535e-128	EpC4	UNC5B-AS1
OGFOD1	1.234235287684e-130	0.379911273985266	0.427	0.161	2.47414805769135e-126	EpC4	OGFOD1
PYCARD	2.32906717281836e-130	0.457857990862793	0.562	0.265	4.66884805463168e-126	EpC4	PYCARD
SQLE.1	2.72441352411169e-130	0.483687195263611	0.518	0.231	5.4613593504343e-126	EpC4	SQLE
ATP2B1	1.57058704872196e-129	0.509106647885862	0.583	0.285	3.14839879786804e-125	EpC4	ATP2B1
TMEM254.1	2.43277494492081e-129	0.330495597390252	0.326	0.098	4.87674065458827e-125	EpC4	TMEM254
NEBL	4.86368801498463e-129	0.291879474828724	0.257	0.059	9.74974899483819e-125	EpC4	NEBL
PLS3.1	6.55612499574051e-128	0.514946678493594	0.589	0.298	1.31424081664614e-123	EpC4	PLS3
B2M.1	1.63763417264598e-127	-0.814918937	0.947	0.987	3.28280146248614e-123	EpC4	B2M
DHCR7	3.16253699365472e-127	0.343713007059723	0.367	0.122	6.33962165748025e-123	EpC4	DHCR7
SUB1.1	6.95887322695521e-127	0.474140136923184	0.924	0.675	1.39497572707544e-122	EpC4	SUB1
H2AFJ.1	9.12584211092701e-127	0.444504459300767	0.473	0.2	1.82936630955643e-122	EpC4	H2AFJ
PTS.1	9.63666574573353e-127	0.43677413705075	0.544	0.258	1.93176601538974e-122	EpC4	PTS
CLTB.1	1.21395172288405e-126	0.471891689873539	0.609	0.309	2.43348762369336e-122	EpC4	CLTB
SCIN	1.35558905320704e-126	0.252092250099436	0.166	0.021	2.71741381605882e-122	EpC4	SCIN

COX8A.1	2.44967858489475e-126	0.495424034666222	0.945	0.786	4.91062569128001e-122	EpC4	COX8A
A2ML1	4.07377433668861e-126	0.311995075966604	0.191	0.031	8.16628803532598e-122	EpC4	A2ML1
HTATIP2	1.51746029406272e-125	0.370121234673012	0.424	0.164	3.04190090547813e-121	EpC4	HTATIP2
TMBIM1.1	1.82566322146386e-125	0.481320049026476	0.702	0.404	3.65972449374645e-121	EpC4	TMBIM1
CD24.1	2.52122440554407e-125	0.670271856978649	0.81	0.581	5.05404644335363e-121	EpC4	CD24
RNH1	4.44860939568742e-125	0.428847510504694	0.591	0.294	8.917682394595e-121	EpC4	RNH1
CDKN2B.1	7.91490462720255e-125	0.332066815549371	0.293	0.081	1.58662178156902e-120	EpC4	CDKN2B
GGCT.1	1.19695123505838e-124	0.442957333529386	0.602	0.303	2.39940844579804e-120	EpC4	GGCT
RTCB	2.03980690179175e-124	0.392668506409132	0.483	0.207	4.08899691533174e-120	EpC4	RTCB
ANXA1.1	4.90328216182396e-124	0.279315550716229	0.995	0.982	9.82911942159232e-120	EpC4	ANXA1
ATP5MC1.1	6.17907545304856e-124	0.479954482286039	0.814	0.509	1.23865746531812e-119	EpC4	ATP5MC1
GALNT7	2.0200239469516e-123	0.32549817375512	0.314	0.094	4.04934000405918e-119	EpC4	GALNT7
CALM1.1	4.4091756813132e-122	0.471039926206631	0.988	0.954	8.83863357076044e-118	EpC4	CALM1
CASP1.1	4.42287401839355e-122	0.339511464349937	0.38	0.135	8.86609325727172e-118	EpC4	CASP1
ANXA7	1.90196236948901e-121	0.443167110631812	0.578	0.294	3.81267376587767e-117	EpC4	ANXA7
NDUFS6.1	1.44541037108759e-120	0.458913893085647	0.77	0.463	2.89746962988218e-116	EpC4	NDUFS6
ATP5MC3	1.609287203372e-120	0.451489205380713	0.978	0.884	3.22597712787951e-116	EpC4	ATP5MC3
PTPRZ1.1	2.18488513119122e-120	0.435596584395772	0.555	0.266	4.37982073398591e-116	EpC4	PTPRZ1
TMEM9B	1.66252850304139e-119	0.431220821310549	0.605	0.315	3.33270463719677e-115	EpC4	TMEM9B
MIEN1.1	2.71879744039047e-119	0.452892091069227	0.669	0.365	5.45010134900673e-115	EpC4	MIEN1
SOX21	5.20517177039808e-119	0.255358756483235	0.211	0.042	1.043428733094e-114	EpC4	SOX21
TAGLN2.1	1.80257262675225e-118	0.508639057514219	0.873	0.611	3.61343708758757e-114	EpC4	TAGLN2
POMP.1	5.86140920910333e-118	0.463881075542807	0.849	0.586	1.17497809005685e-113	EpC4	POMP
PDPN.1	7.60403480226651e-118	-0.822917406	0.18	0.457	1.52430481646234e-113	EpC4	PDPN
LMO4	8.50035691131493e-118	0.515980382000453	0.794	0.502	1.70398154644219e-113	EpC4	LMO4
IGFBP5.1	8.79530625619777e-118	-0.757614931	0.067	0.332	1.76310709211741e-113	EpC4	IGFBP5
STAP2	9.12059576475576e-118	0.36096992728141	0.44	0.181	1.82831462700294e-113	EpC4	STAP2
S100A14.1	1.22024320030835e-117	0.607252654149865	0.877	0.654	2.44609951933811e-113	EpC4	S100A14
PPL.1	4.09184120155024e-117	0.401823816675358	0.425	0.168	8.20250487262761e-113	EpC4	PPL

IL1R2.1	5.08972781985643e-117	-0.9883248	0.281	0.55	1.02028683876842e-112	EpC4	IL1R2
HMGCS1.1	1.53477620669305e-116	0.456604702700713	0.477	0.211	3.07661238393688e-112	EpC4	HMGCS1
TINCR	2.42936128853488e-116	0.331827941145098	0.395	0.147	4.86989763899701e-112	EpC4	TINCR
FRMD4A	6.17482952829938e-116	0.269586341901751	0.238	0.057	1.23780632724289e-111	EpC4	FRMD4A
ASAH1.1	1.86147925482626e-115	0.430073806233386	0.514	0.248	3.73152131422472e-111	EpC4	ASAH1
ERG28.1	9.01685005893041e-115	0.407231990937362	0.438	0.187	1.80751776281319e-110	EpC4	ERG28
MT-CO2.1	9.07427320208244e-115	0.423050046238227	1	1	1.81902880608945e-110	EpC4	MT-CO2
EMC2.1	1.99593558492391e-114	0.370232317320773	0.511	0.235	4.00105247353846e-110	EpC4	EMC2
SUCLG1.1	3.30443943343684e-114	0.483012073868444	0.908	0.682	6.62407928826748e-110	EpC4	SUCLG1
RIT1	4.73613083238338e-114	0.3277875945859	0.362	0.131	9.49404786659572e-110	EpC4	RIT1
TCIM.1	6.82733035857442e-114	0.431592126250931	0.206	0.043	1.36860664367983e-109	EpC4	TCIM
RALA.1	1.07269398256697e-113	0.463546347717945	0.654	0.371	2.15032235745376e-109	EpC4	RALA
RAB10.1	2.10657657920652e-113	0.444844404938536	0.757	0.459	4.2228434106774e-109	EpC4	RAB10
ZDHHC3	5.56533372314978e-113	0.354529397761668	0.468	0.204	1.11562679814261e-108	EpC4	ZDHHC3
CD44	7.5419447794473e-113	0.43033247214335	0.743	0.425	1.51185825048801e-108	EpC4	CD44
NDRG2.1	4.70384116037451e-112	0.325063839280798	0.294	0.09	9.42931999008675e-108	EpC4	NDRG2
NDUFB9.1	4.98784276159597e-112	0.457953435566066	0.896	0.638	9.99862959989528e-108	EpC4	NDUFB9
CES2	6.85292660047659e-112	0.313199953036046	0.274	0.078	1.37373766633154e-107	EpC4	CES2
VDAC1.1	2.87012815512696e-111	0.432599517970427	0.85	0.558	5.75345889976749e-107	EpC4	VDAC1
LGALS7B.1	5.28674157555046e-111	1.12256351760261	0.519	0.245	1.05978021623484e-106	EpC4	LGALS7B
MTCH2.1	2.24586252814669e-110	0.406866713094408	0.617	0.328	4.50205602392285e-106	EpC4	MTCH2
GJB5.1	2.92120509193567e-110	0.361867836628839	0.447	0.192	5.85584772729425e-106	EpC4	GJB5
TUBA1A	5.50499311158891e-110	0.546248352687971	0.633	0.351	1.10353091914911e-105	EpC4	TUBA1A
UPK1B	1.45248888671939e-109	0.911855475642296	0.135	0.015	2.91165922231768e-105	EpC4	UPK1B
UCHL3.1	2.06289694217845e-109	0.349512734984345	0.4	0.161	4.13528321029092e-105	EpC4	UCHL3
TMEM134	4.28362930433269e-109	0.431008370292452	0.624	0.344	8.5869633034653e-105	EpC4	TMEM134
TMEM141.1	4.4698242126563e-109	0.363963130168039	0.457	0.201	8.96020961669083e-105	EpC4	TMEM141
UQCRB	1.16797575947159e-108	0.436871463009078	0.944	0.774	2.34132420743674e-104	EpC4	UQCRB
TJP1.1	2.02889382396963e-108	0.376415248039708	0.41	0.17	4.06712055952953e-104	EpC4	TJP1

UGDH	4.63714936073508e-108	0.388204934514048	0.465	0.211	9.29562960852955e-104	EpC4	UGDH
SNX31	4.95307929192832e-108	0.302928802121483	0.294	0.092	9.92894274859951e-104	EpC4	SNX31
NLRX1	4.95602968961459e-108	0.301528640282187	0.272	0.08	9.9348571158014e-104	EpC4	NLRX1
DLG3	5.375587093339e-108	0.260786673275344	0.247	0.066	1.07759018873074e-103	EpC4	DLG3
SLPI.1	1.08830981432562e-107	0.29257040741586	0.508	0.222	2.18162585379714e-103	EpC4	SLPI
STMP1.1	1.70338947912806e-107	0.406560921542777	0.669	0.379	3.41461454986011e-103	EpC4	STMP1
IFITM3	4.66384501044317e-107	-0.863476855	0.536	0.718	9.34914370793438e-103	EpC4	IFITM3
KCNK1	6.18507408492349e-107	0.300541444379661	0.252	0.07	1.23985995106376e-102	EpC4	KCNK1
ATOX1.1	1.14333581622028e-106	0.431596297765644	0.659	0.371	2.29193097719517e-102	EpC4	ATOX1
PAK1	2.42517557056781e-106	0.45863238662652	0.544	0.276	4.86150694876024e-102	EpC4	PAK1
ANXA8L1.1	2.98132692963986e-106	0.3192573043614	0.34	0.122	5.97636796315607e-102	EpC4	ANXA8L1
MT-CO3	3.92428499301406e-106	0.281310901392174	1	1	7.86662169699598e-102	EpC4	MT-CO3
PNOC.1	1.07463047992762e-105	-0.650005274	0.023	0.25	2.1542042600629e-101	EpC4	PNOC
SLIRP.1	1.20807760452106e-105	0.431552081841206	0.833	0.535	2.42171236602291e-101	EpC4	SLIRP
ACTB.1	1.63119894816282e-105	0.433940588822388	0.997	0.987	3.26990141148719e-101	EpC4	ACTB
PELI1.1	2.24054720548341e-105	0.316629593923191	0.317	0.107	4.49140092811205e-101	EpC4	PELI1
GSTA4	5.0396553792941e-105	0.405987217402289	0.559	0.285	1.01024931733329e-100	EpC4	GSTA4
PDLIM5.1	5.70559651919901e-105	0.400602527139922	0.559	0.291	1.14374387823863e-100	EpC4	PDLIM5
NDUFB8.1	9.02909276716221e-105	0.386386102620041	0.651	0.361	1.80997193610534e-100	EpC4	NDUFB8
PSMB6.1	1.46534201629795e-104	0.456431455426669	0.826	0.564	2.93742460587088e-100	EpC4	PSMB6
SMAGP	1.71571819423135e-104	0.270572958319446	0.243	0.066	3.43932869215616e-100	EpC4	SMAGP
BDH1	8.32182997744402e-104	0.292392727977746	0.29	0.093	1.66819403727843e-99	EpC4	BDH1
C6orf132.1	1.38130096096417e-103	0.29237515494812	0.292	0.094	2.76895590634877e-99	EpC4	C6orf132
RPS18	3.11313310712609e-103	0.337798497588575	0.999	0.993	6.24058662654495e-99	EpC4	RPS18
TMEM147.1	4.90918824950947e-103	0.405869426770206	0.637	0.355	9.84095876496668e-99	EpC4	TMEM147
COX4I1	6.15879270520323e-103	0.364694925802052	0.994	0.968	1.23459158568504e-98	EpC4	COX4I1
BCAM.1	7.32146737254204e-103	-0.558994253	0.12	0.372	1.46766134949978e-98	EpC4	BCAM
CLCN3	1.17392645801861e-102	0.374740637918946	0.466	0.215	2.35325297774411e-98	EpC4	CLCN3
OST4	4.68489359462429e-102	0.437180522511208	0.797	0.525	9.39133769978385e-98	EpC4	OST4

FARSB	5.20292115729773e-102	0.352326646205849	0.442	0.198	1.0429775751919e-97	EpC4	FARSB
GARS.1	9.30570496173506e-102	0.341677866198214	0.392	0.161	1.86542161662941e-97	EpC4	GARS
SDHB.1	1.19134471511509e-101	0.381744224601754	0.634	0.349	2.3881696159197e-97	EpC4	SDHB
FAM114A1.1	3.37691122888435e-101	0.335211170860092	0.415	0.179	6.76935624942157e-97	EpC4	FAM114A1
TMTC3.1	4.39922353505911e-101	0.364409983080504	0.494	0.241	8.81868349837948e-97	EpC4	TMTC3
PYM1	5.93852548777225e-101	0.315071537936045	0.407	0.17	1.19043681927882e-96	EpC4	PYM1
OCIAD2	1.86039548895835e-100	0.416073180158666	0.593	0.325	3.72934879716592e-96	EpC4	OCIAD2
ENO1.1	2.0416761525586e-100	0.441992322434954	0.936	0.735	4.09274401541898e-96	EpC4	ENO1
KRT15.1	1.05736891613185e-99	-0.656177348	0.025	0.243	2.11960172927791e-95	EpC4	KRT15
HMGCR.1	4.40701892424924e-99	0.33860826012963	0.367	0.148	8.83431013555003e-95	EpC4	HMGCR
OSTF1	1.01942981573523e-98	0.370897094401813	0.568	0.301	2.04354900862285e-94	EpC4	OSTF1
HMGA1.1	1.06687615982203e-98	0.365352547156803	0.572	0.299	2.13865994997925e-94	EpC4	HMGA1
TRIM16.1	1.08863761135415e-98	0.290931340945973	0.32	0.114	2.18228295572053e-94	EpC4	TRIM16
GUCY1A1	1.54152042254309e-98	0.309055223429384	0.363	0.142	3.09013183902987e-94	EpC4	GUCY1A1
GHITM.1	2.14837062463326e-98	0.395751513899514	0.748	0.464	4.30662375413983e-94	EpC4	GHITM
ARPC3.1	3.46767367165131e-98	0.411887878804491	0.917	0.685	6.95129864219222e-94	EpC4	ARPC3
TXNL1	4.04018490919531e-98	0.418413887136717	0.645	0.377	8.09895466897291e-94	EpC4	TXNL1
JAKMIP2	4.08417153188723e-98	0.298902403791353	0.272	0.085	8.18713025282114e-94	EpC4	JAKMIP2
F2RL1.1	5.92916753904427e-98	0.30617259895879	0.367	0.144	1.18856092487681e-93	EpC4	F2RL1
HPGD.1	8.54715871036774e-98	0.432371406312498	0.375	0.155	1.71336343508032e-93	EpC4	HPGD
LDHA.1	9.74689920482436e-98	0.465248729577975	0.786	0.518	1.95386341459909e-93	EpC4	LDHA
PPFIBP2	9.88395229560135e-98	0.291303202873672	0.329	0.121	1.98133707717625e-93	EpC4	PPFIBP2
GPI	1.03679706097885e-97	0.33422329750023	0.52	0.258	2.07836338843821e-93	EpC4	GPI
MRPL14.1	1.64840280212705e-97	0.385347540536963	0.603	0.333	3.30438825714388e-93	EpC4	MRPL14
NTRK2	1.86274393324326e-97	0.374881413973881	0.536	0.267	3.73405648857944e-93	EpC4	NTRK2
ZDHHC2	5.02534109436689e-97	0.253114060283096	0.255	0.076	1.00737987577679e-92	EpC4	ZDHHC2
PANK3	9.66709327390459e-97	0.281129868559839	0.309	0.109	1.93786551768691e-92	EpC4	PANK3
EPHA4	1.25006629724177e-96	0.263066440968462	0.251	0.075	2.50588289945085e-92	EpC4	EPHA4
VPS25.1	1.47427406931056e-96	0.31272476043932	0.403	0.171	2.95532979933996e-92	EpC4	VPS25

NAA20.1	1.52543410584142e-96	0.407256099293687	0.709	0.437	3.05788520856972e-92	EpC4	NAA20
ALCAM	1.74416490515808e-96	0.389035485911294	0.647	0.359	3.49635296887988e-92	EpC4	ALCAM
HMOX2	2.88646478583413e-96	0.357783067618571	0.454	0.214	5.78620730968309e-92	EpC4	HMOX2
SYNGR2.1	4.07850775263564e-96	0.365128823056216	0.626	0.348	8.17577664093341e-92	EpC4	SYNGR2
OXA1L	4.72282232507326e-96	0.337613823067165	0.441	0.204	9.46736963284186e-92	EpC4	OXA1L
DHCR24.1	1.10389410498036e-95	0.350129647137366	0.466	0.221	2.21286612284363e-91	EpC4	DHCR24
NDUFAB1.1	2.63878079678156e-95	0.410277687417961	0.792	0.519	5.28969998522832e-91	EpC4	NDUFAB1
MINOS1.1	3.71407861787965e-95	0.371507277041013	0.634	0.362	7.44524199740154e-91	EpC4	MINOS1
UQCRC2	3.76349060539226e-95	0.362991505792606	0.719	0.43	7.54429326756932e-91	EpC4	UQCRC2
GIPC1	5.46231488179835e-95	0.270712301998196	0.291	0.099	1.0949756412053e-90	EpC4	GIPC1
SOCS2	6.98888651362454e-95	0.268845484674167	0.308	0.109	1.40099219052117e-90	EpC4	SOCS2
PPP1R14B	1.49958514361995e-94	0.331983428715461	0.436	0.199	3.00606837890055e-90	EpC4	PPP1R14B
ATP5PF.1	2.44917427933605e-94	0.388149950843724	0.966	0.848	4.90961476035705e-90	EpC4	ATP5PF
DBNL.1	2.84823001292718e-94	0.302048982281079	0.357	0.144	5.70956188391383e-90	EpC4	DBNL
TOLLIP	4.9539854374579e-94	0.273438894996513	0.317	0.116	9.93075920792811e-90	EpC4	TOLLIP
BLOC1S2.1	7.46204346671146e-94	0.357241151287583	0.567	0.303	1.49584123333698e-89	EpC4	BLOC1S2
ATP6V0D1	1.49942230973406e-93	0.281763320041114	0.353	0.14	3.0057419620929e-89	EpC4	ATP6V0D1
PSMD8.1	2.34139137535023e-93	0.385420799901466	0.73	0.451	4.69355315102706e-89	EpC4	PSMD8
CLINT1.1	9.0158281473407e-93	0.397346820991901	0.672	0.398	1.80731291041592e-88	EpC4	CLINT1
EIF3I.1	1.71801989665483e-92	0.405638769685316	0.812	0.551	3.44394268483427e-88	EpC4	EIF3I
PGAM1.1	3.16164281826459e-92	0.294273328172735	0.389	0.167	6.3378291934932e-88	EpC4	PGAM1
EIF3K	3.68876193214974e-92	0.402680683169903	0.91	0.707	7.39449216918736e-88	EpC4	EIF3K
GID8	4.15725809006349e-92	0.339500731322246	0.42	0.193	8.33363956734127e-88	EpC4	GID8
ROMO1.1	4.37167935146783e-92	0.408460699089422	0.772	0.513	8.76346842795242e-88	EpC4	ROMO1
HIST1H4C.1	4.4966370276855e-92	-1.233940455	0.568	0.682	9.01395858569836e-88	EpC4	HIST1H4C
UBE2V2.1	1.09026060152191e-91	0.396751666544858	0.629	0.367	2.18553640181081e-87	EpC4	UBE2V2
NSG1	1.3550657782867e-91	0.331109619709974	0.392	0.17	2.71636485915352e-87	EpC4	NSG1
CHMP4B.1	3.2553921681954e-91	0.298982965976054	0.432	0.198	6.52575914036451e-87	EpC4	CHMP4B
ACTR3.1	4.65770546004907e-91	0.38167783156726	0.703	0.429	9.33683636521436e-87	EpC4	ACTR3

MT-CO1	6.97027377884516e-91	0.312830321341131	1	1	1.3972610817073e-86	EpC4	MT-CO1
RHOV.1	8.61835734506125e-91	0.300276024405457	0.372	0.155	1.72763591339098e-86	EpC4	RHOV
SARS	1.90442447771957e-90	0.354532619145282	0.548	0.294	3.81760930803665e-86	EpC4	SARS
MRPL33	4.11452936988915e-90	0.378520116268312	0.669	0.404	8.2479855748798e-86	EpC4	MRPL33
GJB3	1.1560177675486e-89	0.267355102561841	0.319	0.121	2.31735321682793e-85	EpC4	GJB3
PCCB	1.24615765838099e-89	0.30909783184405	0.38	0.162	2.49804764199052e-85	EpC4	PCCB
SERPINB6.1	2.21468400910243e-89	0.33123010552249	0.355	0.147	4.43955556464672e-85	EpC4	SERPINB6
CERS3.1	3.14439174934153e-89	0.345834627055845	0.465	0.224	6.30324770073004e-85	EpC4	CERS3
ELOVL1	3.88496122091892e-89	0.363156277032357	0.52	0.273	7.78779326345408e-85	EpC4	ELOVL1
ELL2.1	1.19776386884439e-88	0.297068953143718	0.336	0.133	2.40103745148546e-84	EpC4	ELL2
PSMD11.1	1.62532054803649e-88	0.34376491903112	0.571	0.312	3.25811757059395e-84	EpC4	PSMD11
MARVELD2	2.55563864979725e-88	0.269882009463519	0.325	0.125	5.12303323738356e-84	EpC4	MARVELD2
G6PD	3.02098327167292e-88	0.259523332954997	0.28	0.098	6.05586306639554e-84	EpC4	G6PD
YWHAB.1	3.8030623739596e-88	0.404188207921613	0.874	0.64	7.62361883483942e-84	EpC4	YWHAB
RAB18	3.81358882763354e-88	0.363534670421699	0.723	0.453	7.6447201638742e-84	EpC4	RAB18
GSTO1	1.24773724100776e-87	0.484034843823368	0.909	0.781	2.50121407332416e-83	EpC4	GSTO1
SELENOW	1.62780208332906e-87	0.457550583532911	0.937	0.819	3.26309205624143e-83	EpC4	SELENOW
A4GALT	2.90511311356586e-87	0.25075093296717	0.288	0.102	5.82358974745411e-83	EpC4	A4GALT
SOX2	1.07931676666741e-86	0.434976348171771	0.783	0.511	2.16359839046149e-82	EpC4	SOX2
SC5D	2.24728649145252e-86	0.298289937889673	0.362	0.155	4.50491050076572e-82	EpC4	SC5D
PSMA3.1	2.59181366383999e-86	0.355907771579282	0.654	0.385	5.19554967053364e-82	EpC4	PSMA3
AP2S1.1	5.58651203381128e-86	0.373539742342029	0.746	0.478	1.11987220229781e-81	EpC4	AP2S1
SERPINB2.1	1.05581933936573e-85	0.414937785811876	0.371	0.155	2.11649544769254e-81	EpC4	SERPINB2
TIMM17A.1	1.08926950725241e-85	0.337012735	0.489	0.251	2.18354965423818e-81	EpC4	TIMM17A
REEP5	1.31244529297955e-85	0.374069804674668	0.65	0.39	2.63092783430681e-81	EpC4	REEP5
COX7C	1.37371186668833e-85	0.369256383961533	0.985	0.955	2.75374280796343e-81	EpC4	COX7C
TRAPPC1	1.90844445818885e-85	0.333609686180645	0.525	0.281	3.82566776088538e-81	EpC4	TRAPPC1
BGN.1	2.32803257348948e-85	-0.409902518	0.053	0.262	4.666774096817e-81	EpC4	BGN
SCAMP2	2.37458475065746e-85	0.283432690708658	0.378	0.166	4.76009259116794e-81	EpC4	SCAMP2

SMDT1	3.68566957569956e-85	0.354667375344829	0.66	0.395	7.38829323144734e-81	EpC4	SMDT1
SEC61B.1	5.85266594126812e-85	0.369168400965793	0.828	0.581	1.17322541458661e-80	EpC4	SEC61B
NDUFC1	7.04155061571822e-85	0.393466457404916	0.801	0.551	1.41154923642687e-80	EpC4	NDUFC1
SDHC	1.00085906739108e-84	0.339001342412477	0.6	0.348	2.00632208649216e-80	EpC4	SDHC
CTNNAL1.1	1.30196068961555e-84	-0.39610896	0.025	0.218	2.60991039840333e-80	EpC4	CTNNAL1
HIF1A.1	3.57148672299371e-84	0.383208557784594	0.688	0.417	7.15940228491319e-80	EpC4	HIF1A
FAHD1	3.70311654439253e-84	0.288585132156289	0.368	0.159	7.42326742488927e-80	EpC4	FAHD1
LDHB.1	4.34423274686202e-84	-0.587988519	0.448	0.64	8.70844896435961e-80	EpC4	LDHB
TMSB4X.1	5.64108826296837e-84	0.371718630625112	0.999	0.994	1.13081255319464e-79	EpC4	TMSB4X
PLD1.1	6.72009040680872e-84	0.312915620940544	0.415	0.194	1.34710932294888e-79	EpC4	PLD1
LINC01133	1.03438939407842e-83	0.352096775756329	0.468	0.233	2.0735369793696e-79	EpC4	LINC01133
MT-RNR2.1	2.04016611452622e-83	-0.341326654	1	1	4.08971699317926e-79	EpC4	MT-RNR2
NACA	2.15594510156149e-83	0.318066186446643	0.994	0.961	4.32180755059017e-79	EpC4	NACA
ACADVL.1	5.53896053735976e-83	0.331076747308095	0.521	0.28	1.11034002931914e-78	EpC4	ACADVL
NDUFB3.1	5.86766710279787e-83	0.361326187758615	0.79	0.528	1.17623254742686e-78	EpC4	NDUFB3
GSDMC	6.78784743454972e-83	0.265024549449952	0.305	0.117	1.36069189672984e-78	EpC4	GSDMC
RPL36	7.33313345657154e-83	0.331535525518128	0.998	0.989	1.46999993270433e-78	EpC4	RPL36
PPIC.1	1.05271908533899e-82	0.280493685455628	0.39	0.174	2.11028067847053e-78	EpC4	PPIC
FGFBP1.1	1.87525906301339e-82	0.330294537126395	0.378	0.167	3.75914431771664e-78	EpC4	FGFBP1
AKR1C1.1	2.56223831570653e-82	0.41906244168606	0.373	0.168	5.13626292766531e-78	EpC4	AKR1C1
COMMD8	5.03548966527183e-82	0.286831246490007	0.392	0.178	1.00941425830039e-77	EpC4	COMMD8
F3.1	6.30373372421327e-82	0.378345183394453	0.344	0.147	1.26364646235579e-77	EpC4	F3
ELOB.1	7.75008908717381e-82	0.351300979788833	0.954	0.807	1.55358285841486e-77	EpC4	ELOB
TBL1X	1.15740350601391e-81	0.265014999458099	0.274	0.099	2.32013106815549e-77	EpC4	TBL1X
RNF130	2.82482089313457e-81	0.282627599351914	0.382	0.172	5.66263596237755e-77	EpC4	RNF130
CYB5R2	3.27569970238785e-81	0.261061647172008	0.295	0.112	6.56646762340668e-77	EpC4	CYB5R2
CMAS	7.09684585070737e-81	0.308695232873822	0.44	0.216	1.4226337192328e-76	EpC4	CMAS
PPP4C.1	1.04452776678526e-80	0.295714032295763	0.439	0.218	2.09386036129773e-76	EpC4	PPP4C
POLR2L	1.50801161054888e-80	0.360005466868946	0.846	0.599	3.02296007450629e-76	EpC4	POLR2L

CDC42	2.92608694997972e-80	0.363397409861459	0.942	0.784	5.86563389992935e-76	EpC4	CDC42
HBEGF.1	3.98832583067068e-80	0.471912528008194	0.503	0.268	7.99499796016245e-76	EpC4	HBEGF
CTNND1.1	4.14957289877846e-80	0.343002560985736	0.531	0.293	8.3182338328913e-76	EpC4	CTNND1
EIF4E2	4.24358789486254e-80	0.292576078685357	0.437	0.214	8.50669629404145e-76	EpC4	EIF4E2
NFIB.1	5.020225160028e-80	-0.599822172	0.234	0.45	1.00635433557921e-75	EpC4	NFIB
CAPG.1	5.87536020892048e-80	0.321011665539618	0.54	0.298	1.1777747074802e-75	EpC4	CAPG
ATP5MPL	9.94349303532901e-80	0.345630724103091	0.947	0.796	1.99327261386205e-75	EpC4	ATP5MPL
RPL27	1.71515196823228e-79	0.315609519790145	0.994	0.979	3.43819363551842e-75	EpC4	RPL27
NUCKS1	1.86846395299756e-79	-0.620086898	0.779	0.847	3.7455228401789e-75	EpC4	NUCKS1
NCOA4.1	1.99570937510645e-79	0.34051189826462	0.528	0.293	4.00059901333838e-75	EpC4	NCOA4
CDC42SE1	3.02392575416169e-79	0.275204589002385	0.347	0.151	6.06176156679252e-75	EpC4	CDC42SE1
TPD52	3.96868641732309e-79	0.29882208631739	0.497	0.262	7.95562879216586e-75	EpC4	TPD52
CADM1	4.1416916589745e-79	0.324901691492071	0.454	0.229	8.30243509958028e-75	EpC4	CADM1
FRMD6	4.43241661596143e-79	0.296712209107414	0.379	0.173	8.88522234835627e-75	EpC4	FRMD6
RPS26	4.6849974933621e-79	0.353968588714513	0.997	0.976	9.39154597519366e-75	EpC4	RPS26
SH3BGRL3	9.56830336786711e-79	0.396888934105772	0.854	0.621	1.91806209312264e-74	EpC4	SH3BGRL3
COPE.1	1.85262231070349e-78	0.32501362880026	0.599	0.349	3.71376668403621e-74	EpC4	COPE
MANF.1	1.86960051868103e-78	0.338819338851481	0.502	0.272	3.74780119974799e-74	EpC4	MANF
DERA.1	3.19883727047265e-78	0.271903192438847	0.359	0.158	6.41238919238948e-74	EpC4	DERA
PDZD11.1	4.19711547388455e-78	0.317592183605682	0.464	0.238	8.41353767894896e-74	EpC4	PDZD11
C1QBP.1	4.38445966405245e-78	0.385769532758665	0.629	0.384	8.78908784255954e-74	EpC4	C1QBP
SNX1	9.15009840867787e-78	0.31553823178031	0.479	0.253	1.83422872700357e-73	EpC4	SNX1
TCF4	1.0731901381732e-77	0.36472857318387	0.501	0.278	2.151316950982e-73	EpC4	TCF4
PSMD7.1	1.65633540618329e-77	0.348738865857391	0.401	0.194	3.32028995523502e-73	EpC4	PSMD7
MT-ND5	1.73210815508423e-77	0.279344020503306	1	1	3.47218400768184e-73	EpC4	MT-ND5
POLR2I	2.08510179378932e-77	0.314876072527165	0.498	0.268	4.17979505583008e-73	EpC4	POLR2I
MYL12B	8.3706301459045e-77	0.307054495089559	0.989	0.962	1.67797651904802e-72	EpC4	MYL12B
PSMD1.1	9.20743860855451e-77	0.300171137528164	0.466	0.242	1.84572314347084e-72	EpC4	PSMD1
ATP5F1B	9.55116758722626e-77	0.362816087023432	0.936	0.781	1.91462705453538e-72	EpC4	ATP5F1B

PTGR1.1	1.00834584756838e-76	0.452551901178349	0.498	0.289	2.02133008603557e-72	EpC4	PTGR1
MT-ATP6	1.45573528472611e-76	0.279350500402018	1	1	2.91816695176197e-72	EpC4	MT-ATP6
VDAC2	1.50801424145242e-76	0.342028720124105	0.83	0.585	3.02296534841552e-72	EpC4	VDAC2
MRPL36.1	2.80473769187271e-76	0.332606684399143	0.55	0.311	5.62237717712804e-72	EpC4	MRPL36
SDC4.1	3.31221394600441e-76	0.305048325616497	0.491	0.26	6.63966407616044e-72	EpC4	SDC4
PSMA1	3.49761328882432e-76	0.323923412578332	0.653	0.395	7.01131559877723e-72	EpC4	PSMA1
MDH2	3.63751472123358e-76	0.35575787528652	0.822	0.559	7.29176201018483e-72	EpC4	MDH2
LGALSL	6.01510177609732e-76	0.259879144239343	0.271	0.102	1.20578730203647e-71	EpC4	LGALSL
TECR.1	7.51067833656254e-76	0.327424592755886	0.659	0.412	1.50559057934733e-71	EpC4	TECR
PDLIM4	8.78153177047844e-76	0.319660427557208	0.469	0.248	1.76034585871011e-71	EpC4	PDLIM4
BRK1	1.19291514967769e-75	0.351882835273701	0.784	0.544	2.39131770904389e-71	EpC4	BRK1
PON2	1.26909378949228e-75	0.363581048071025	0.432	0.221	2.54402541041622e-71	EpC4	PON2
CAPZA1	1.31194956460117e-75	0.29708359646712	0.483	0.257	2.6299340971995e-71	EpC4	CAPZA1
DDAH2	2.98494560010718e-75	0.308496148740932	0.376	0.177	5.98362194997486e-71	EpC4	DDAH2
RER1.1	6.64521642903497e-75	0.285319442331645	0.466	0.244	1.33210008536435e-70	EpC4	RER1
ATP8B1	1.06797961306707e-74	0.307256062824109	0.503	0.273	2.14087193235424e-70	EpC4	ATP8B1
SEC11C.1	4.65599494447725e-74	0.288687102588357	0.374	0.174	9.33340746569909e-70	EpC4	SEC11C
RAB6A	1.3893644324685e-73	0.29130676560108	0.423	0.214	2.78511994132635e-69	EpC4	RAB6A
MRPS7	1.45706992327201e-73	0.302296728013356	0.497	0.267	2.92084236819107e-69	EpC4	MRPS7
ITGB1.1	1.52813657070501e-73	-0.513971797	0.338	0.535	3.06330256963526e-69	EpC4	ITGB1
NIPSNAP3A	2.20315372352351e-73	0.271744798634966	0.348	0.156	4.41644195417523e-69	EpC4	NIPSNAP3A
GUK1	2.32221326314248e-73	0.338152486435412	0.699	0.453	4.65510870729541e-69	EpC4	GUK1
CLIC1.1	2.4421889338637e-73	0.371537506541882	0.876	0.688	4.89561193682318e-69	EpC4	CLIC1
ECH1	1.26903745717946e-72	0.328976092247175	0.637	0.388	2.54391248666196e-68	EpC4	ECH1
ATP5F1C	1.39346330805022e-72	0.351001079843182	0.903	0.676	2.79333654731746e-68	EpC4	ATP5F1C
RALB	1.73896720846592e-72	0.378931349120226	0.603	0.369	3.48593366609078e-68	EpC4	RALB
C19orf70	1.85379052079513e-72	0.345885996561039	0.64	0.396	3.71610847798592e-68	EpC4	C19orf70
POLR2E	4.0071342676083e-72	0.330141479076573	0.604	0.365	8.03270135284759e-68	EpC4	POLR2E
CAPZB.1	4.04080755334598e-72	0.321878414214506	0.773	0.518	8.10020282143735e-68	EpC4	CAPZB

CTTNBP2NL	8.37349270683215e-72	0.307649185924044	0.408	0.205	1.67855034801157e-67	EpC4	CTTNBP2NL
TSTA3	1.14756814595046e-71	0.254338761262262	0.31	0.132	2.30041510537229e-67	EpC4	TSTA3
BIK	2.22246148543772e-71	0.276359473967353	0.261	0.1	4.45514629370845e-67	EpC4	BIK
NDUFA3.1	2.69532709937515e-71	0.328897817075454	0.676	0.431	5.40305270340744e-67	EpC4	NDUFA3
TMEM256	4.37814848361335e-71	0.27837838322318	0.415	0.209	8.77643645025132e-67	EpC4	TMEM256
PMVK	6.24574813421429e-71	0.258853207765741	0.383	0.184	1.2520226709846e-66	EpC4	PMVK
PRDX1.1	7.30849747147169e-71	0.657117497686136	0.861	0.677	1.46506140313122e-66	EpC4	PRDX1
WDR61.1	8.05233615495507e-71	0.299144703492823	0.471	0.255	1.61417130562229e-66	EpC4	WDR61
ATP5MG	9.50943708651903e-71	0.314374584040792	0.985	0.928	1.9062617583636e-66	EpC4	ATP5MG
ZDHHC5	9.82218645528658e-71	0.255776306085976	0.344	0.156	1.96895549682675e-66	EpC4	ZDHHC5
DHRS7	1.03594237351875e-70	0.290159548445442	0.426	0.219	2.07665008195569e-66	EpC4	DHRS7
CYC1	2.08371909317141e-70	0.348819602621378	0.683	0.439	4.1770232941714e-66	EpC4	CYC1
FAM136A.1	2.18879793961143e-70	0.321565855221388	0.457	0.247	4.38766434974508e-66	EpC4	FAM136A
SF3B6	3.32037313135162e-70	0.337139467247892	0.839	0.617	6.65601997910745e-66	EpC4	SF3B6
STRBP	3.43614287553053e-70	0.262848193581386	0.382	0.182	6.8880920082885e-66	EpC4	STRBP
PGK1.1	3.82621129452536e-70	0.323498262011264	0.739	0.481	7.67002316100553e-66	EpC4	PGK1
MRPL20	7.15171391473872e-70	0.297096924651736	0.548	0.315	1.43363257134852e-65	EpC4	MRPL20
VPS29.1	1.29402261787155e-69	0.327113005271557	0.724	0.481	2.59399773978532e-65	EpC4	VPS29
LGALS3.1	1.64800504227634e-69	0.315586545872924	0.811	0.572	3.30359090774716e-65	EpC4	LGALS3
NDUFA13	2.83139735691146e-69	0.324125087205481	0.754	0.511	5.67581914166472e-65	EpC4	NDUFA13
GNG5.1	3.62592225836814e-69	0.354649795778956	0.834	0.614	7.26852375912478e-65	EpC4	GNG5
PLEKHA1	4.28462581829343e-69	0.287074059685684	0.473	0.258	8.58896091535101e-65	EpC4	PLEKHA1
MT1E	5.67893754806497e-69	-0.602406863	0.366	0.553	1.1383998208851e-64	EpC4	MT1E
ARF6	7.61382013936039e-69	0.257585508108759	0.376	0.181	1.52626638513618e-64	EpC4	ARF6
MPST	1.19333487100389e-68	0.250288581706802	0.385	0.187	2.3921590824144e-64	EpC4	MPST
EIF3J	1.3400776349151e-68	0.296387884314713	0.561	0.33	2.68631962695081e-64	EpC4	EIF3J
C8orf59	1.63567677342888e-68	0.307221599818077	0.587	0.348	3.27887766001554e-64	EpC4	C8orf59
ABI1.1	2.3290697505846e-68	0.295240802459881	0.52	0.298	4.66885322202189e-64	EpC4	ABI1
MRPL41	2.44318398308457e-68	0.31386539642486	0.615	0.383	4.89760661249134e-64	EpC4	MRPL41

AC106795.1	5.48507578359589e-68	0.256072582919741	0.373	0.18	1.09953829157963e-63	EpC4	AC106795.1
DCXR.1	1.51799490845719e-67	0.291981246700641	0.477	0.261	3.04297259349329e-63	EpC4	DCXR
LEPROTL1	2.18228751192939e-67	0.256054234638091	0.385	0.189	4.37461354641367e-63	EpC4	LEPROTL1
TIMM13	4.25944057530571e-67	0.326753774921651	0.683	0.443	8.53847457725783e-63	EpC4	TIMM13
HDAC1.1	7.09433062537317e-67	0.327259525546126	0.529	0.313	1.42212951716231e-62	EpC4	HDAC1
PSMC4	1.17314929399546e-66	0.283320630263905	0.468	0.257	2.35169507474331e-62	EpC4	PSMC4
CYR61.1	1.42969822752481e-66	-0.637999767	0.19	0.386	2.86597306689624e-62	EpC4	CYR61
INSIG1.1	1.58145815394266e-66	0.275939918407287	0.282	0.117	3.17019101539346e-62	EpC4	INSIG1
GTF3C6	1.66850660513444e-66	0.296687386009921	0.512	0.294	3.34468834065251e-62	EpC4	GTF3C6
LRRC8A	1.94220764691395e-66	0.324717414057586	0.443	0.242	3.89334944900371e-62	EpC4	LRRC8A
STMN1	2.04850222122914e-66	-0.814218502	0.548	0.654	4.10642755267593e-62	EpC4	STMN1
ATP6V0E1	2.78412521932187e-66	0.356116874095877	0.887	0.706	5.58105741465262e-62	EpC4	ATP6V0E1
NDUFA12	4.99350109368595e-66	0.328810059772218	0.807	0.569	1.00099722924029e-61	EpC4	NDUFA12
TMEM45B.1	1.20558951349154e-65	0.310895075741417	0.244	0.092	2.41672473874515e-61	EpC4	TMEM45B
NTS.1	1.25795282563922e-65	1.42607858883882	0.471	0.315	2.52169223427639e-61	EpC4	NTS
ATP5PO	1.33574916920643e-65	0.330687956191771	0.921	0.776	2.6776427845912e-61	EpC4	ATP5PO
MMAB	1.44506148746122e-65	0.252515688227188	0.302	0.132	2.89677025776475e-61	EpC4	MMAB
TOMM22	1.53692044198217e-65	0.277808707909447	0.489	0.274	3.08091071799746e-61	EpC4	TOMM22
PSMB1	1.89842038515801e-65	0.31749366823587	0.826	0.592	3.80557350408775e-61	EpC4	PSMB1
CTSC.1	2.37143935260311e-65	0.364334893740633	0.529	0.299	4.7537873262282e-61	EpC4	CTSC
EMC7	6.44690521625576e-65	0.273393686413635	0.452	0.246	1.29234661965063e-60	EpC4	EMC7
PSMD6	8.81599918598961e-65	0.271349108375339	0.468	0.258	1.76725519682348e-60	EpC4	PSMD6
SSNA1	1.10526502438981e-64	0.263755530093161	0.403	0.208	2.21561426789182e-60	EpC4	SSNA1
GLO1.1	1.19379645184998e-64	0.298099299853212	0.624	0.39	2.39308436737848e-60	EpC4	GLO1
CHCHD5	1.48727436442819e-64	0.256565193118712	0.337	0.158	2.98139019093275e-60	EpC4	CHCHD5
RPL35	1.52815114556779e-64	0.293114778110589	0.992	0.961	3.06333178640519e-60	EpC4	RPL35
ZNF770.1	2.11094629108228e-64	0.283699629925235	0.477	0.27	4.23160293510354e-60	EpC4	ZNF770
MPC1	2.42725746271813e-64	0.261636488789766	0.405	0.208	4.86568030976477e-60	EpC4	MPC1
MRPL52.1	3.18469370799405e-64	0.301505929990867	0.604	0.371	6.38403700704488e-60	EpC4	MRPL52

ERGIC2	3.46638733398866e-64	0.265477027633567	0.409	0.214	6.94872004971367e-60	EpC4	ERGIC2
PSMB3.1	4.28977871067974e-64	0.311202120238957	0.767	0.53	8.5992904034286e-60	EpC4	PSMB3
PRR13	4.77966507715898e-64	0.278180785344785	0.517	0.302	9.58131661367289e-60	EpC4	PRR13
TMEM159	5.0481513854777e-64	0.274468589369161	0.454	0.252	1.01195242673286e-59	EpC4	TMEM159
ABRACL.1	5.07434556340398e-64	0.298743085306021	0.523	0.306	1.01720331163996e-59	EpC4	ABRACL
ORMDL2	5.44748268328261e-64	0.254885960695587	0.32	0.147	1.09200237869083e-59	EpC4	ORMDL2
KLK7	5.77935503180117e-64	0.45270118708178	0.131	0.031	1.15852950967486e-59	EpC4	KLK7
TMPRSS11BNL.1	7.31404697898309e-64	0.317759607047278	0.222	0.082	1.46617385740695e-59	EpC4	TMPRSS11BNL
NDUFA9	2.1550162507267e-63	0.268149041411373	0.443	0.24	4.31994557620674e-59	EpC4	NDUFA9
ATP6V0B.1	2.73595455871141e-63	0.298872826081052	0.681	0.439	5.48449450839288e-59	EpC4	ATP6V0B
DAAM1	2.82631560093004e-63	0.278576090290416	0.369	0.184	5.66563225362436e-59	EpC4	DAAM1
MTIF3	3.12821606618123e-63	0.26772792720898	0.442	0.237	6.27082192626689e-59	EpC4	MTIF3
CD82.1	3.31066077259337e-63	0.297916165808432	0.534	0.313	6.63655058474067e-59	EpC4	CD82
SH3YL1	3.38620624157952e-63	0.268013178479971	0.39	0.2	6.78798903187032e-59	EpC4	SH3YL1
VDAC3.1	3.83044106804294e-63	0.25579422779559	0.501	0.28	7.67850216499888e-59	EpC4	VDAC3
NDUFV3	4.1963815103411e-63	0.282956474706665	0.482	0.275	8.41206637562976e-59	EpC4	NDUFV3
ARPC1A	8.36050305623692e-63	0.280802906614418	0.497	0.287	1.67594644265325e-58	EpC4	ARPC1A
SMIM37	8.75796243792699e-63	0.289451155420746	0.476	0.268	1.75562115030685e-58	EpC4	SMIM37
SSR4.1	9.13034147790657e-63	0.340287534949217	0.822	0.612	1.83026825266115e-58	EpC4	SSR4
CAPNS1	1.23842090073503e-62	0.286922546877784	0.498	0.29	2.48253853761344e-58	EpC4	CAPNS1
UQCRH	1.60851933328319e-62	0.288469086764976	0.975	0.909	3.22443785549949e-58	EpC4	UQCRH
ETHE1.1	1.91707192841106e-62	0.287768243642406	0.621	0.388	3.84296238769281e-58	EpC4	ETHE1
PSMC5	4.15666494301773e-62	0.285296073174158	0.571	0.353	8.33245054477334e-58	EpC4	PSMC5
PDHB	5.35407779182623e-62	0.250388205428963	0.439	0.236	1.07327843414949e-57	EpC4	PDHB
EMP1.1	5.75232628476805e-62	0.296581033275069	0.865	0.659	1.1531113270446e-57	EpC4	EMP1
NDUFB1.1	6.29476212902404e-62	0.309387584771396	0.759	0.527	1.26184801638416e-57	EpC4	NDUFB1
LAMTOR1	9.37921798192765e-62	0.28476154117399	0.56	0.345	1.88015803665722e-57	EpC4	LAMTOR1
ATG3	1.1615365000453e-61	0.261653200351671	0.409	0.216	2.32841606799081e-57	EpC4	ATG3
SLC25A39.1	1.49632188755998e-61	0.254476498696192	0.41	0.216	2.99952685580273e-57	EpC4	SLC25A39

TIMM23	3.27918689350709e-61	0.250919727686156	0.392	0.202	6.57345804672431e-57	EpC4	TIMM23
TXNL4A	4.0102044128443e-61	0.272008809383152	0.511	0.296	8.03885576598769e-57	EpC4	TXNL4A
SEC61G.1	4.20413181535992e-61	0.302760592596296	0.716	0.49	8.42760263707049e-57	EpC4	SEC61G
YWHAQ	5.75118615826054e-61	0.279011230737387	0.769	0.515	1.15288277728491e-56	EpC4	YWHAQ
NDUFA10	8.69200086656717e-61	0.287979179564313	0.7	0.46	1.74239849371205e-56	EpC4	NDUFA10
CPNE3	9.56615670550865e-61	0.263710168535246	0.564	0.339	1.91763177318626e-56	EpC4	CPNE3
TBCA	1.14122974130792e-60	0.303823346623881	0.724	0.494	2.28770913942586e-56	EpC4	TBCA
FHL2	1.98287733222932e-60	-0.294081137	0.044	0.203	3.97487590018689e-56	EpC4	FHL2
NDUFA1	3.60324157837918e-60	0.312282217022157	0.871	0.677	7.22305806801891e-56	EpC4	NDUFA1
ENTPD3	4.34768764650963e-60	0.282114766871158	0.423	0.229	8.7153746561932e-56	EpC4	ENTPD3
PPA2	5.49210758790726e-60	0.279919190335142	0.621	0.393	1.10094788707189e-55	EpC4	PPA2
MME	6.12070730123956e-60	-0.414706377	0.021	0.164	1.22695698560648e-55	EpC4	MME
SPINT2.1	6.49598815682065e-60	0.286287948935964	0.922	0.754	1.30218578591627e-55	EpC4	SPINT2
MRPS33	6.74273003150364e-60	0.294251716173257	0.592	0.37	1.35164766211522e-55	EpC4	MRPS33
C19orf33	7.75125277703391e-60	0.314339967735049	0.55	0.343	1.55381613168422e-55	EpC4	C19orf33
SEM1	8.06611838297959e-60	0.293297488931022	0.947	0.807	1.61693409105209e-55	EpC4	SEM1
TMEM167A.1	9.67024360681888e-60	0.287543328316351	0.558	0.342	1.93849703342291e-55	EpC4	TMEM167A
AURKAIP1.1	1.19882610436668e-59	0.271452903224222	0.597	0.369	2.40316680881345e-55	EpC4	AURKAIP1
IVL.1	1.40440035972953e-59	0.372515273700901	0.141	0.038	2.81526096111381e-55	EpC4	IVL
CSNK1A1	1.45373718176811e-59	0.267988805609991	0.577	0.357	2.91416155457236e-55	EpC4	CSNK1A1
FIS1	1.8093773226894e-59	0.281247972481415	0.527	0.316	3.62707778106317e-55	EpC4	FIS1
COX14	2.06512912647835e-59	0.273655945927772	0.59	0.366	4.13975784693851e-55	EpC4	COX14
HSD17B10	2.37088143177576e-59	0.262069452086134	0.465	0.263	4.7526689181377e-55	EpC4	HSD17B10
ATP6V1F	3.07310896787233e-59	0.287710480890255	0.666	0.436	6.16035423699687e-55	EpC4	ATP6V1F
PLP2.1	3.54462268405407e-59	0.300272758512611	0.938	0.815	7.10555063245478e-55	EpC4	PLP2
NDUFS8.1	3.89012200834896e-59	0.297104140184949	0.611	0.392	7.79813857793632e-55	EpC4	NDUFS8
LAMA3	4.03542289209883e-59	-0.427303063	0.086	0.257	8.08940872950132e-55	EpC4	LAMA3
SERPINE2	4.63551088286331e-59	-0.328391608	0.036	0.187	9.2923451157878e-55	EpC4	SERPINE2
RPS2	4.74806884612018e-59	0.264512680279704	0.998	0.99	9.51797880893251e-55	EpC4	RPS2

SERBP1	4.90820146923534e-59	0.306018697897348	0.851	0.628	9.83898066522916e-55	EpC4	SERBP1
HHIP.1	8.18595120322207e-59	-0.463592399	0.033	0.181	1.6409557781979e-54	EpC4	HHIP
ID2	1.0210762849137e-58	-0.669582643	0.401	0.55	2.046849520738e-54	EpC4	ID2
PPCS	1.43427707489012e-58	0.264355428550128	0.499	0.289	2.87515182432473e-54	EpC4	PPCS
SYNGR1	1.70489105665764e-58	0.256080071429176	0.345	0.173	3.4176246121759e-54	EpC4	SYNGR1
CFL1.1	1.91812977624824e-58	0.28894466094505	0.959	0.849	3.84508294946723e-54	EpC4	CFL1
TSHZ2	2.07813518018387e-58	0.264295697534362	0.421	0.228	4.16582978219659e-54	EpC4	TSHZ2
EPRS	2.8411482348677e-58	0.263923920667193	0.513	0.302	5.6953657516158e-54	EpC4	EPRS
CRABP2.1	3.24633336719627e-58	-0.563071397	0.119	0.297	6.50759986788164e-54	EpC4	CRABP2
SNX6	3.79513812653787e-58	0.291011329932836	0.657	0.433	7.60773388845781e-54	EpC4	SNX6
ANK3	4.13870051204186e-58	0.265218844450612	0.407	0.219	8.29643904643912e-54	EpC4	ANK3
ACTN4.1	4.63009228013595e-58	0.315015479619231	0.564	0.355	9.28148298476053e-54	EpC4	ACTN4
TRAPPC2L	6.93084618876022e-58	0.263812543899209	0.401	0.214	1.38935742699887e-53	EpC4	TRAPPC2L
UQCRFS1	7.35785772964484e-58	0.283047119029445	0.652	0.421	1.4749561604846e-53	EpC4	UQCRFS1
DMAC1	9.9967520227375e-58	0.273512336170643	0.436	0.242	2.00394891047796e-53	EpC4	DMAC1
MRPL3	1.62485458590615e-57	0.26326873440249	0.497	0.29	3.25718350290747e-53	EpC4	MRPL3
UBE2N	1.70215068010442e-57	0.268826723043464	0.622	0.397	3.41213125333732e-53	EpC4	UBE2N
GTF2H5	2.02967673678914e-57	0.273569144357834	0.526	0.315	4.06868998656751e-53	EpC4	GTF2H5
NEDD8	3.58199408658932e-57	0.279622508888184	0.789	0.563	7.18046534597695e-53	EpC4	NEDD8
TIMM17B	4.81535442262248e-57	0.254664290483878	0.385	0.203	9.65285947558903e-53	EpC4	TIMM17B
NIT2	7.86585723064097e-57	0.250677228970761	0.385	0.205	1.57678974045429e-52	EpC4	NIT2
CCT6A.1	1.70893804332343e-56	0.289912855599225	0.679	0.458	3.42573720164614e-52	EpC4	CCT6A
RPLP2	1.99198515567392e-56	0.25474917303797	0.999	0.998	3.99313344306394e-52	EpC4	RPLP2
CHCHD2.1	2.03676462891829e-56	0.277317197815105	0.933	0.798	4.0828983751296e-52	EpC4	CHCHD2
ILF2.1	3.22191262474016e-56	0.252187331782754	0.622	0.391	6.45864604755412e-52	EpC4	ILF2
RAMP1.1	3.73337000650755e-56	-0.355874121	0.066	0.226	7.48391351504504e-52	EpC4	RAMP1
NDUFS3	4.6228459271623e-56	0.260292067044263	0.57	0.35	9.26695694558955e-52	EpC4	NDUFS3
CLDND1.1	4.77510533181212e-56	0.272033483073747	0.463	0.271	9.57217614815058e-52	EpC4	CLDND1
REXO2.1	6.63933642452739e-56	0.293427107193709	0.51	0.31	1.33092137966076e-51	EpC4	REXO2

CCND1	9.16496669687585e-56	0.380047201432533	0.69	0.482	1.83720922405573e-51	EpC4	CCND1
ARF1.1	9.24009038733039e-56	0.276096031878311	0.829	0.6	1.85226851904425e-51	EpC4	ARF1
NME1	1.11139726883849e-55	0.298080218685807	0.644	0.42	2.22790696511363e-51	EpC4	NME1
CHCHD10	1.26277442345863e-55	0.261250203333294	0.523	0.315	2.53135760926517e-51	EpC4	CHCHD10
H3F3AP4	2.18762905077792e-55	0.254803443093907	0.549	0.338	4.38532119518942e-51	EpC4	H3F3AP4
SSBP1	2.31547611436098e-55	0.283274891576549	0.787	0.551	4.64160341884802e-51	EpC4	SSBP1
KRT10	7.24491628218018e-55	0.369439805629733	0.662	0.447	1.45231591792584e-50	EpC4	KRT10
APP	7.77580767080142e-55	-0.385610706	0.188	0.367	1.55873840568885e-50	EpC4	APP
PSMD14	1.25289760671567e-54	0.255717682331939	0.488	0.29	2.51155854242224e-50	EpC4	PSMD14
ZFP36L2.1	2.28302253731572e-54	-0.409670943	0.177	0.352	4.57654697830309e-50	EpC4	ZFP36L2
TUBB2A.1	2.34454560628537e-54	0.283186442548712	0.377	0.203	4.69987612235966e-50	EpC4	TUBB2A
SELENOS.1	3.2046471575862e-54	0.273861892376068	0.54	0.336	6.4240356920973e-50	EpC4	SELENOS
DAPL1.1	3.36083849877793e-54	0.352523815244832	0.431	0.242	6.73713685465025e-50	EpC4	DAPL1
MRPS36	3.45769375328566e-54	0.270177937686757	0.559	0.351	6.93129289783642e-50	EpC4	MRPS36
KIF5B	5.64127715890635e-54	0.277196949546791	0.691	0.471	1.13085041927437e-49	EpC4	KIF5B
GNAI3.1	6.34139860231369e-54	0.262097700885063	0.507	0.308	1.2711967638198e-49	EpC4	GNAI3
LOXL4.1	9.07208291380827e-54	-0.278859263	0.009	0.133	1.81858974090201e-49	EpC4	LOXL4
CMPK1.1	3.66466747251069e-53	0.266809232123504	0.457	0.268	7.34619241539493e-49	EpC4	CMPK1
PSMB5	4.22916578769978e-53	0.294957981655114	0.715	0.501	8.47778573802298e-49	EpC4	PSMB5
RPL22L1.1	1.4661533553642e-52	0.294635405877106	0.548	0.348	2.93905101616308e-48	EpC4	RPL22L1
SDHD	2.87781989080412e-52	0.261515363545201	0.727	0.495	5.76887775310593e-48	EpC4	SDHD
CHMP2A.1	3.30492524470956e-52	0.279855288767556	0.555	0.359	6.62505314554478e-48	EpC4	CHMP2A
MRPL13.1	3.42442620013916e-52	0.256536694457109	0.612	0.391	6.86460476079896e-48	EpC4	MRPL13
RPL38	5.09596367662438e-52	0.271061152658558	0.971	0.916	1.02153687861612e-47	EpC4	RPL38
PTCH1.1	5.80571468982684e-52	-0.425699963	0.105	0.265	1.16381356672269e-47	EpC4	PTCH1
IGFBP7.1	5.82075567556886e-52	-0.389653443	0.068	0.219	1.16682868272453e-47	EpC4	IGFBP7
SLITRK6.1	5.95053055852235e-52	-0.325453428	0.044	0.185	1.19284335576139e-47	EpC4	SLITRK6
DYNLRB1	8.85949401421706e-52	0.256418048901669	0.555	0.35	1.77597417008995e-47	EpC4	DYNLRB1
SPCS1	9.53104673683839e-52	0.264631052767071	0.736	0.512	1.91059362886662e-47	EpC4	SPCS1

GPX3.1	1.92741176886894e-51	0.365661703374269	0.177	0.063	3.86368963187468e-47	EpC4	GPX3
SAA1.1	5.00667111895606e-51	-0.872607912	0.011	0.129	1.00363729250593e-46	EpC4	SAA1
COX17.1	5.41358196762584e-51	0.285052920825037	0.585	0.376	1.08520664123028e-46	EpC4	COX17
MYL6.1	9.06770558361473e-51	0.256603836581051	0.992	0.981	1.81771226129141e-46	EpC4	MYL6
NDUFS5	1.20138809911593e-50	0.271463161509387	0.944	0.828	2.40830258348778e-46	EpC4	NDUFS5
RPN2.1	2.21447590075403e-50	0.280104089852566	0.658	0.452	4.43913839065152e-46	EpC4	RPN2
EIF5B	2.23444687806831e-50	0.260395411729713	0.717	0.487	4.47917221177574e-46	EpC4	EIF5B
BUD23	4.32683113985884e-50	0.255890379142234	0.479	0.29	8.67356570296103e-46	EpC4	BUD23
TMOD3	5.29145862470997e-50	0.28992562274179	0.653	0.447	1.06072579590936e-45	EpC4	TMOD3
PA2G4.1	2.19635011915366e-49	0.257131184256935	0.65	0.43	4.40280344885542e-45	EpC4	PA2G4
SNU13	2.59930586448219e-49	0.257120431913299	0.72	0.497	5.21056853594099e-45	EpC4	SNU13
ASS1.1	2.97089779929545e-49	-0.417648724	0.055	0.195	5.95546172846766e-45	EpC4	ASS1
CDKN1A.1	4.90052514972653e-49	0.259372849205382	0.591	0.377	9.82359271514181e-45	EpC4	CDKN1A
AL450405.1	5.79560876321257e-49	0.392595259578578	0.518	0.339	1.16178773267359e-44	EpC4	AL450405.1
YBX3.1	6.44300830989514e-49	0.282714657799712	0.625	0.42	1.29156544580158e-44	EpC4	YBX3
MRPS15.1	7.77042919976933e-49	0.259665911866497	0.495	0.308	1.55766023738576e-44	EpC4	MRPS15
CFH.1	1.30695385801931e-48	-0.447161303	0.023	0.145	2.6199197037855e-44	EpC4	CFH
NDUFS4	3.49348595304251e-48	0.280135048979444	0.695	0.486	7.00304194146901e-44	EpC4	NDUFS4
C3orf14	9.52435388018597e-48	0.250536338090253	0.338	0.18	1.90925197882208e-43	EpC4	C3orf14
KLF4	1.80280558523238e-47	0.336710623866757	0.599	0.412	3.61390407615683e-43	EpC4	KLF4
CTSV	3.01286909300412e-47	-0.270395773	0.068	0.211	6.03959738383605e-43	EpC4	CTSV
DCN.1	3.08296983106495e-47	-1.046613165	0.102	0.247	6.1801213233528e-43	EpC4	DCN
BLOC1S1	3.14949914682488e-47	0.253675281136102	0.451	0.27	6.31348598972515e-43	EpC4	BLOC1S1
LY6E	4.02575727927152e-47	-0.430400094	0.428	0.557	8.0700330420277e-43	EpC4	LY6E
TKT	4.43516799662923e-47	0.258893058744742	0.785	0.555	8.89073776604294e-43	EpC4	TKT
MT2A.1	1.91929490852965e-46	-0.784672755	0.387	0.525	3.84741857363853e-42	EpC4	MT2A
PPA1	6.92742887868035e-46	0.260067024355503	0.645	0.441	1.38867239302026e-41	EpC4	PPA1
TGFBI.1	2.43310803857027e-45	-0.413210185	0.205	0.366	4.87740837411797e-41	EpC4	TGFBI
CAV1	5.25119167774955e-45	-0.414194437	0.158	0.312	1.05265388372168e-40	EpC4	CAV1

CLIC3.1	6.7564261978679e-45	0.294698214548869	0.184	0.072	1.3543931956246e-40	EpC4	CLIC3
HSBP1	2.1480493509043e-44	0.262481914418318	0.881	0.678	4.30597972882275e-40	EpC4	HSBP1
RPL37A	7.79431326937673e-44	0.261438158253642	0.998	0.986	1.56244803797926e-39	EpC4	RPL37A
KRT18.1	1.64669614725797e-43	0.307161357717977	0.588	0.398	3.30096709679332e-39	EpC4	KRT18
TOP2A.1	1.68963853872196e-43	-0.660800264	0.019	0.128	3.38704941472204e-39	EpC4	TOP2A
CLEC2B.1	2.65422510405337e-43	-1.224128771	0.04	0.159	5.32065964358538e-39	EpC4	CLEC2B
NUSAP1.1	4.57149076178306e-43	-0.684499785	0.036	0.154	9.16401038107032e-39	EpC4	NUSAP1
PHLDB2	2.18390840077659e-42	0.2561091958228	0.321	0.178	4.37786278019675e-38	EpC4	PHLDB2
NDUFA6	5.19707662182796e-42	0.251879518582597	0.806	0.587	1.04180597961163e-37	EpC4	NDUFA6
GADD45A.1	2.04831243307986e-41	0.274940089455622	0.573	0.38	4.10604710335188e-37	EpC4	GADD45A
SOX4.1	9.79273025540879e-41	-0.396453223	0.678	0.794	1.96305070699925e-36	EpC4	SOX4
HNRNPA1.1	1.32187220229749e-40	-0.329104067	0.906	0.905	2.64982501672554e-36	EpC4	HNRNPA1
EMP3	3.98869854889879e-40	-0.257932696	0.055	0.177	7.99574511112252e-36	EpC4	EMP3
PDLIM1.1	4.03350813612296e-40	-0.405389732	0.512	0.6	8.08557040967209e-36	EpC4	PDLIM1
CBR1	1.10136600367391e-39	0.294555392952421	0.9	0.777	2.20779829096472e-35	EpC4	CBR1
PPP1CA	2.95248750443655e-39	0.251475207237754	0.431	0.268	5.91855645139351e-35	EpC4	PPP1CA
LAMC2.1	4.17825378850218e-39	-0.370970883	0.059	0.18	8.37572754443147e-35	EpC4	LAMC2
NCOA7.1	9.80993366219931e-39	-0.738977687	0.16	0.296	1.96649930192447e-34	EpC4	NCOA7
CAP1.1	1.21893247486366e-38	0.290852521518246	0.409	0.26	2.44347203911169e-34	EpC4	CAP1
TTK.1	1.76234129418379e-38	-0.301511783	0.013	0.107	3.53278935832082e-34	EpC4	TTK
MKI67.1	3.67613771272987e-38	-0.474132228	0.035	0.141	7.3691856589383e-34	EpC4	MKI67
TUBB	7.13387785654456e-38	-0.559054549	0.569	0.617	1.43005715512292e-33	EpC4	TUBB
RBBP8	3.22310145544391e-36	-0.370993549	0.209	0.344	6.46102917758286e-32	EpC4	RBBP8
LPAR6	1.99065624701801e-35	-0.332773798	0.185	0.322	3.9904695127723e-31	EpC4	LPAR6
CDC20.1	1.38449340408904e-34	-0.300349387	0.02	0.111	2.77535547783689e-30	EpC4	CDC20
RAB31.1	2.80832959121072e-34	-0.262009546	0.045	0.151	5.62957749854102e-30	EpC4	RAB31
CXCL1.1	5.38011281240921e-34	-1.873377485	0.087	0.202	1.07849741437555e-29	EpC4	CXCL1
ITGA6	7.26735332963846e-34	-0.337697855	0.209	0.344	1.45681364845933e-29	EpC4	ITGA6
CENPF.1	4.22954412109963e-33	-0.564314402	0.042	0.141	8.47854414515633e-29	EpC4	CENPF

CDH13.1	7.0209586682928e-33	-0.289008176	0.137	0.263	1.40742137464598e-28	EpC4	CDH13
TNC	1.01269306593062e-31	-0.27485843	0.079	0.191	2.03004451996451e-27	EpC4	TNC
MGST3	1.21538196387174e-31	0.250416901638744	0.754	0.59	2.4363546847773e-27	EpC4	MGST3
HMGB1	4.54431851936908e-31	-0.451397447	0.91	0.931	9.10954090392726e-27	EpC4	HMGB1
FDPS.1	4.76714803244142e-31	0.318327712141883	0.293	0.173	9.55622494583207e-27	EpC4	FDPS
ETS2	6.825706077567e-31	-0.37015516	0.184	0.304	1.36828104030908e-26	EpC4	ETS2
TPX2.1	9.5770453684391e-31	-0.353995167	0.032	0.121	1.9198145145573e-26	EpC4	TPX2
MT1F.1	1.10864207477244e-30	-0.308846698	0.109	0.226	2.22238390308883e-26	EpC4	MT1F
FTH1.1	1.13271850070992e-30	-0.499862349	0.577	0.689	2.27064750652311e-26	EpC4	FTH1
TUBA1C.1	2.15954115867938e-30	0.325533313554079	0.268	0.158	4.32901620668869e-26	EpC4	TUBA1C
S100A2.1	3.28567374737643e-30	0.379472318546725	0.954	0.985	6.5864615939908e-26	EpC4	S100A2
CTGF	2.2442241619336e-29	-0.449863511	0.094	0.202	4.49877175501209e-25	EpC4	CTGF
TMEM94.1	6.92187709305573e-29	-0.292898617	0.088	0.193	1.38755948207395e-24	EpC4	TMEM94
SOSTDC1.1	7.60255351079068e-29	-0.477415387	0.414	0.528	1.5240078767731e-24	EpC4	SOSTDC1
DSG2	1.5209885451039e-28	-0.281418564	0.237	0.365	3.04897363751528e-24	EpC4	DSG2
GAS5.1	2.19974513559812e-28	-0.364242688	0.688	0.707	4.40960909882e-24	EpC4	GAS5
HIST1H1C	5.15792438713488e-28	-0.528907133	0.337	0.442	1.03395752264506e-23	EpC4	HIST1H1C
CLCA4.1	2.73035670389113e-27	-0.879931387	0.038	0.123	5.47327304862016e-23	EpC4	CLCA4
DEK	2.76547651682151e-27	-0.445869774	0.453	0.519	5.54367422562039e-23	EpC4	DEK
PCLAF.1	3.32394472413326e-27	-0.285333529	0.037	0.12	6.66317959399754e-23	EpC4	PCLAF
PHLDA1.1	3.56566830998178e-27	-0.354515051	0.109	0.215	7.14773869418948e-23	EpC4	PHLDA1
CCNB1.1	3.68937368390115e-27	-0.429250197	0.042	0.128	7.39571848674825e-23	EpC4	CCNB1
PLSCR1.1	8.79594811264221e-26	-0.26659385	0.141	0.247	1.76323575866026e-21	EpC4	PLSCR1
SDC2.1	8.90228208659661e-26	-0.275932845	0.124	0.23	1.78455146707916e-21	EpC4	SDC2
TXNIP.1	9.33888952485346e-26	-0.427059064	0.406	0.506	1.87207379415212e-21	EpC4	TXNIP
EDN1.1	1.39540560543394e-25	-0.319854612	0.087	0.186	2.79723007665288e-21	EpC4	EDN1
HIST1H1D.1	1.95993625693233e-25	-0.374567889	0.053	0.139	3.92888822064655e-21	EpC4	HIST1H1D
CDKN3.1	4.30947410986102e-25	-0.272650615	0.026	0.1	8.63877180062739e-21	EpC4	CDKN3
SMC2.1	1.05024660965796e-24	-0.285234923	0.145	0.249	2.10532435372035e-20	EpC4	SMC2

JUNB.1	3.88698388127471e-24	-0.352830557	0.729	0.75	7.79184788840329e-20	EpC4	JUNB
TIMP1	3.89929970565287e-24	-0.326779234	0.301	0.407	7.81653618995173e-20	EpC4	TIMP1
BIRC5.1	5.43663330402385e-24	-0.250807173	0.033	0.108	1.08982751212462e-19	EpC4	BIRC5
FXYD5	5.54979629319225e-24	-0.258442223	0.106	0.204	1.11251216493332e-19	EpC4	FXYD5
FDCSP.1	1.45275555592654e-23	-4.783642525	0.147	0.236	2.91219378741035e-19	EpC4	FDCSP
PRDX3	5.70618107603382e-23	-0.291369884	0.509	0.571	1.14386105850174e-18	EpC4	PRDX3
CKS1B.1	7.55090518841974e-23	-0.377199218	0.181	0.277	1.51365445407062e-18	EpC4	CKS1B
ID3	2.20158777341676e-22	-0.411233457	0.459	0.545	4.41330285059124e-18	EpC4	ID3
MT-ND6.1	3.41781046299344e-22	0.351913078395702	0.726	0.825	6.85134285411665e-18	EpC4	MT-ND6
FMO2.1	1.91531994687908e-21	-0.52099357	0.091	0.176	3.8394503655138e-17	EpC4	FMO2
CRYAB.1	3.06369097358956e-21	0.25433854583732	0.56	0.426	6.14147492565762e-17	EpC4	CRYAB
EPCAM.1	5.79939997744138e-21	-0.409018653	0.57	0.607	1.1625477194779e-16	EpC4	EPCAM
TP63.1	9.41957213138585e-21	-0.308350047	0.548	0.588	1.88824742945761e-16	EpC4	TP63
SMC4.1	1.63875454945006e-20	-0.316081316	0.108	0.194	3.28504736982759e-16	EpC4	SMC4
S100A8.1	2.78010815535618e-20	-1.0971161	0.164	0.268	5.57300480822701e-16	EpC4	S100A8
FOS.1	3.21519308331553e-20	-0.313751379	0.926	0.949	6.44517605481431e-16	EpC4	FOS
CKS2.1	3.53118051482762e-20	-0.333832579	0.155	0.247	7.07860446002345e-16	EpC4	CKS2
PPIB	4.37390214201029e-20	0.271776337724029	0.302	0.209	8.76792423387383e-16	EpC4	PPIB
CXCL3.1	2.80512316472112e-19	-0.524335779	0.044	0.112	5.62314989599996e-15	EpC4	CXCL3
FOSB	3.13088520789732e-19	-0.353557324	0.497	0.556	6.27617248775096e-15	EpC4	FOSB
HES1.1	3.79691370088188e-19	0.374991716110767	0.614	0.51	7.61129320478781e-15	EpC4	HES1
JUN.1	4.04401121259125e-19	-0.311211158	0.863	0.898	8.10662487676043e-15	EpC4	JUN
MXD1.1	4.22795536953063e-19	-0.308928697	0.105	0.187	8.4753593337611e-15	EpC4	MXD1
ATP1B3.1	6.19934010826294e-19	-0.290661943	0.879	0.827	1.24271971810239e-14	EpC4	ATP1B3
IL36G.1	1.0017085317724e-18	-0.840671602	0.054	0.122	2.00802492279095e-14	EpC4	IL36G
CENPW.1	1.01507147830403e-18	-0.294991839	0.119	0.204	2.03481228540827e-14	EpC4	CENPW
RDH10.1	1.9083303185557e-18	-0.276316774	0.057	0.126	3.82543895657675e-14	EpC4	RDH10
LUM.1	2.84050769408023e-18	-0.345769846	0.099	0.177	5.69408172355323e-14	EpC4	LUM
IL1RN.1	7.16850020206651e-18	-0.710720927	0.49	0.326	1.43699755050625e-13	EpC4	IL1RN

HS3ST1.1	1.33880067008452e-17	-0.254759333	0.042	0.104	2.68375982325143e-13	EpC4	HS3ST1
KRT8	1.58026936853779e-16	0.269723855361925	0.552	0.458	3.16780797617085e-12	EpC4	KRT8
BEX3	1.73237581538618e-16	-0.288721576	0.492	0.541	3.47272055952314e-12	EpC4	BEX3
PLAUR.1	4.21608399801399e-16	-0.494723092	0.139	0.215	8.45156198241885e-12	EpC4	PLAUR
NAP1L1	1.71104729572056e-15	-0.291336395	0.52	0.555	3.42996540900144e-11	EpC4	NAP1L1
PPP1R15A	2.0468260499433e-15	-0.318619828	0.603	0.635	4.10306749971634e-11	EpC4	PPP1R15A
FTL	2.27682396412277e-15	0.357137988071276	0.931	0.959	4.56412131848051e-11	EpC4	FTL
ID1	4.24511070279765e-15	-0.302716231	0.437	0.497	8.50974891482818e-11	EpC4	ID1
CTSB	2.19397763518497e-14	-0.428571954	0.777	0.745	4.39804756749179e-10	EpC4	CTSB
LGALS1	3.37716807546695e-14	-0.515524132	0.26	0.331	6.76987112408104e-10	EpC4	LGALS1
SNHG8.1	2.22620610396581e-13	-0.308186826	0.604	0.616	4.46265275600987e-09	EpC4	SNHG8
SERPINB4.1	3.2246068335202e-13	-0.454727586	0.286	0.187	6.4640468584746e-09	EpC4	SERPINB4
SELENOP.1	3.35544131119035e-13	-0.277303023	0.272	0.338	6.72631765241217e-09	EpC4	SELENOP
IFI16.1	3.42197818209953e-13	-0.338934662	0.314	0.371	6.85969746383673e-09	EpC4	IFI16
SPARC	1.15160803336845e-12	-0.31213955	0.227	0.298	2.3085134636904e-08	EpC4	SPARC
PDZK1IP1.1	3.60401637126739e-12	-0.446842144	0.098	0.159	7.22461121784261e-08	EpC4	PDZK1IP1
CBX3	6.2144927741969e-12	-0.272927407	0.661	0.631	1.24575722151551e-07	EpC4	CBX3
CKAP2.1	1.04645470934819e-11	-0.345770284	0.189	0.251	2.09772311035938e-07	EpC4	CKAP2
SPRR2A.1	1.58336606318035e-10	-1.161479278	0.1	0.155	3.17401561025133e-06	EpC4	SPRR2A
EGR1	1.88545633640171e-10	-0.293199281	0.644	0.647	3.77958577195087e-06	EpC4	EGR1
H2AFZ.1	2.87457920554165e-10	-0.497991229	0.795	0.768	5.76238147542879e-06	EpC4	H2AFZ
S100A9.1	9.39102907006612e-10	-1.18828304	0.171	0.228	1.88252568738546e-05	EpC4	S100A9
GSN.1	1.25652459442419e-09	-0.255589274	0.554	0.558	2.51882920198274e-05	EpC4	GSN
SOD2.1	1.32168724247929e-09	-0.514026103	0.282	0.325	2.64945424627399e-05	EpC4	SOD2
NR4A1.1	1.54360867144617e-09	-0.299401131	0.194	0.25	3.09431794278099e-05	EpC4	NR4A1
CCNL1	2.12654848973918e-09	-0.281063475	0.503	0.516	4.26287910253115e-05	EpC4	CCNL1
S100A7.1	4.33459738795635e-09	-0.703944844	0.124	0.182	8.6891339238973e-05	EpC4	S100A7
HMGB2.1	6.36919820658406e-09	-0.542500361	0.235	0.276	0.000127676947249184	EpC4	HMGB2
APOBEC3A.1	1.21034292346068e-08	-0.465143959	0.065	0.107	0.000242625342436928	EpC4	APOBEC3A

SPRR2D.1	7.55835504700732e-08	-0.636452115	0.082	0.125	0.00151514785272309	EpC4	SPRR2D
HIST1H1E	7.76973256094146e-08	-0.342542329	0.317	0.355	0.00155752058916633	EpC4	HIST1H1E
GLUL.1	4.48823753560921e-07	-0.593148252	0.811	0.784	0.00899712096388222	EpC4	GLUL
GADD45B	6.05811106865024e-07	-0.334469243	0.584	0.592	0.0121440894482163	EpC4	GADD45B
PRSS22.1	6.64260314210209e-07	-0.828529112	0.098	0.137	0.0133157622586578	EpC4	PRSS22
TNFSF10.1	6.91118602111583e-07	-0.760055242	0.473	0.461	0.0138541634979288	EpC4	TNFSF10
CXCL8.1	8.17247444339434e-07	-0.978085138	0.198	0.24	0.0163825422692283	EpC4	CXCL8
SPARCL1.1	1.78444729645549e-06	-0.462293854	0.068	0.103	0.0357710305047467	EpC4	SPARCL1
ARL6IP1.1	2.13975779507714e-06	-0.412349436	0.427	0.433	0.0428935847601164	EpC4	ARL6IP1
ECM1.1	4.60376844960064e-06	-0.360591084	0.071	0.104	0.0922871423406944	EpC4	ECM1
SPRR3.1	4.77429978557786e-05	0.893653426288933	0.161	0.128	0.957056135016937	EpC4	SPRR3
KRT19.1	5.68136384912237e-05	0.371556295538992	0.703	0.705	1	EpC4	KRT19
IFI27.1	7.96534310847466e-05	-0.29155794	0.072	0.1	1	EpC4	IFI27
TFRC.1	0.00011898665614809	-0.296327431	0.241	0.27	1	EpC4	TFRC
KPNA2.1	0.000223849489260227	-0.265638153	0.199	0.227	1	EpC4	KPNA2
TMPRSS11E.1	0.00120231426029513	-0.800254118	0.109	0.131	1	EpC4	TMPRSS11E
LCN2.1	0.00208255602659231	-0.52098751	0.143	0.172	1	EpC4	LCN2
NUSAP1.2	0	2.13585257179013	0.707	0.033	0	EpC3	NUSAP1
TOP2A.2	0	2.08258454287614	0.628	0.018	0	EpC3	TOP2A
HMGB2.2	0	2.03957020638272	0.845	0.175	0	EpC3	HMGB2
H2AFZ.2	0	1.96479137797041	0.982	0.742	0	EpC3	H2AFZ
CENPF.2	0	1.87764032020208	0.64	0.034	0	EpC3	CENPF
HIST1H1B.1	0	1.80872641085245	0.375	0.032	0	EpC3	HIST1H1B
PTTG1.1	0	1.77219833828167	0.745	0.259	0	EpC3	PTTG1
STMN1.1	0	1.76590675781711	0.959	0.577	0	EpC3	STMN1
MKI67.2	0	1.6577205921017	0.652	0.031	0	EpC3	MKI67
UBE2C.1	0	1.55732808683145	0.486	0.009	0	EpC3	UBE2C
CCNB1.2	0	1.54516313948885	0.514	0.043	0	EpC3	CCNB1
HMGB1.1	0	1.52748217155049	0.999	0.914	0	EpC3	HMGB1

TPX2.2	0	1.39995243711586	0.576	0.024	0	EpC3	TPX2
CENPW.2	0	1.25849856535261	0.677	0.106	0	EpC3	CENPW
CKS1B.2	0	1.25472661483688	0.704	0.183	0	EpC3	CKS1B
SMC4.2	0	1.23208412800452	0.659	0.097	0	EpC3	SMC4
TTK.2	0	1.20608093359913	0.545	0.011	0	EpC3	TTK
CDKN3.2	0	1.18427308462286	0.484	0.019	0	EpC3	CDKN3
PCLAF.2	0	1.1743310234929	0.554	0.029	0	EpC3	PCLAF
CDK1.1	0	1.16554360230737	0.473	0.013	0	EpC3	CDK1
CDC20.2	0	1.16393712187103	0.498	0.025	0	EpC3	CDC20
BIRC5.2	0	1.14775407675995	0.537	0.019	0	EpC3	BIRC5
PTMA.1	0	1.1460732845024	1	0.998	0	EpC3	PTMA
DLGAP5.1	0	1.02526953477194	0.438	0.004	0	EpC3	DLGAP5
SMC2.2	0	1.01010505265252	0.664	0.155	0	EpC3	SMC2
ANLN.1	0	0.99728537298508	0.508	0.013	0	EpC3	ANLN
ASPM.1	0	0.971264298229827	0.435	0.008	0	EpC3	ASPM
TK1.1	0	0.920223878061328	0.462	0.018	0	EpC3	TK1
HMMR.1	0	0.920015309527106	0.429	0.006	0	EpC3	HMMR
KIF11.1	0	0.896403408482184	0.492	0.023	0	EpC3	KIF11
CEP55.1	0	0.895323159279959	0.455	0.007	0	EpC3	CEP55
PBK.1	0	0.894727822656809	0.472	0.007	0	EpC3	PBK
UBE2T.1	0	0.880900228329916	0.518	0.042	0	EpC3	UBE2T
KIF20B.1	0	0.877693587809005	0.479	0.042	0	EpC3	KIF20B
CENPE	0	0.849482946918668	0.362	0.011	0	EpC3	CENPE
CCNB2.1	0	0.84688421697284	0.409	0.009	0	EpC3	CCNB2
MAD2L1.1	0	0.838036418972191	0.51	0.025	0	EpC3	MAD2L1
CENPU.1	0	0.793979925963646	0.457	0.042	0	EpC3	CENPU
ECT2.1	0	0.783605691812614	0.447	0.04	0	EpC3	ECT2
CENPK	0	0.773008519552598	0.46	0.025	0	EpC3	CENPK
ESCO2	0	0.742920858593399	0.344	0.003	0	EpC3	ESCO2

BUB1B	0	0.732338338553314	0.418	0.006	0	EpC3	BUB1B
ZWINT	0	0.702310333561101	0.461	0.028	0	EpC3	ZWINT
LMNB1	0	0.697117168980274	0.461	0.033	0	EpC3	LMNB1
KIF23	0	0.697076020250478	0.364	0.009	0	EpC3	KIF23
CENPM	0	0.689785349766937	0.398	0.02	0	EpC3	CENPM
RRM2	0	0.688940417184583	0.354	0.005	0	EpC3	RRM2
DEPDC1	0	0.685865409330725	0.359	0.003	0	EpC3	DEPDC1
CKAP2L	0	0.682634518981959	0.387	0.008	0	EpC3	CKAP2L
NUF2	0	0.678534818093187	0.364	0.003	0	EpC3	NUF2
NDC80	0	0.677701002085652	0.366	0.003	0	EpC3	NDC80
ATAD2	0	0.668321574575698	0.393	0.042	0	EpC3	ATAD2
SGO2	0	0.650730017534751	0.347	0.014	0	EpC3	SGO2
CDCA8	0	0.650611041342536	0.355	0.008	0	EpC3	CDCA8
SPC25	0	0.643215963877445	0.344	0.003	0	EpC3	SPC25
DHFR	0	0.619970045036105	0.352	0.027	0	EpC3	DHFR
KNL1	0	0.618194596813521	0.348	0.01	0	EpC3	KNL1
BUB1	0	0.598019144432787	0.327	0.007	0	EpC3	BUB1
CDCA3	0	0.586677917970725	0.297	0.007	0	EpC3	CDCA3
CCNA2	0	0.577618086607309	0.328	0.003	0	EpC3	CCNA2
FANCI	0	0.57286410297599	0.371	0.035	0	EpC3	FANCI
RACGAP1	0	0.55920934568914	0.353	0.027	0	EpC3	RACGAP1
SGO1	0	0.556173765145463	0.32	0.004	0	EpC3	SGO1
KIF2C	0	0.549834985139536	0.302	0.004	0	EpC3	KIF2C
KIFC1	0	0.528964533334271	0.339	0.009	0	EpC3	KIFC1
NCAPG	0	0.528668807143399	0.311	0.002	0	EpC3	NCAPG
RAD51AP1	0	0.525178482067389	0.296	0.01	0	EpC3	RAD51AP1
KIF15	0	0.516314665853447	0.299	0.005	0	EpC3	KIF15
ARHGAP11A	0	0.508013081164029	0.304	0.009	0	EpC3	ARHGAP11A
KIF20A	0	0.50102843455439	0.274	0.003	0	EpC3	KIF20A

SKA3	0	0.463355116417378	0.289	0.006	0	EpC3	SKA3
GTSE1	0	0.461479785101988	0.263	0.001	0	EpC3	GTSE1
DIAPH3	0	0.45178537340682	0.269	0.004	0	EpC3	DIAPH3
MELK	0	0.432129987877685	0.289	0.013	0	EpC3	MELK
TACC3	0	0.424330462382819	0.273	0.005	0	EpC3	TACC3
CENPA	0	0.423881775158784	0.225	0.001	0	EpC3	CENPA
KIF4A	0	0.422496620591068	0.243	0.004	0	EpC3	KIF4A
PIMREG	0	0.399870593023768	0.233	0.003	0	EpC3	PIMREG
HJURP	0	0.383549664351775	0.224	0.001	0	EpC3	HJURP
SPAG5	3.71069357760848e-307	0.373855804288451	0.239	0.006	7.43845634567397e-303	EpC3	SPAG5
PLK1	9.66998872169959e-306	0.414972986631552	0.211	0.001	1.9384459391519e-301	EpC3	PLK1
AURKA	2.93965102678644e-304	0.620502588376876	0.31	0.021	5.8928244482961e-300	EpC3	AURKA
ASF1B	3.41984905983737e-302	0.409655484851485	0.25	0.008	6.85542942534998e-298	EpC3	ASF1B
KIF18A	1.00829256208001e-297	0.462645075241829	0.251	0.009	2.02122326994559e-293	EpC3	KIF18A
TUBB.1	1.6038071568694e-291	1.36650662114657	0.916	0.557	3.2149918266604e-287	EpC3	TUBB
CLSPN	2.99077313150554e-291	0.497313524517789	0.274	0.014	5.995303819416e-287	EpC3	CLSPN
NEK2	1.88731441368256e-289	0.370862983539003	0.203	0.002	3.78331047366806e-285	EpC3	NEK2
CKAP2.2	1.50998612110857e-287	1.30291639931605	0.627	0.174	3.02691817837425e-283	EpC3	CKAP2
CENPN	8.08846677073854e-287	0.518344975383779	0.339	0.031	1.62141404886225e-282	EpC3	CENPN
TMPO.1	4.20216743660173e-285	1.08117961978449	0.731	0.253	8.42366484341183e-281	EpC3	TMPO
AURKB	7.53963479442158e-285	0.366266004786595	0.201	0.002	1.51139519088975e-280	EpC3	AURKB
TYMS	1.53859732214253e-284	0.496991243666513	0.33	0.029	3.08427219196691e-280	EpC3	TYMS
BRCA2	1.80709407296537e-284	0.432030755383297	0.269	0.014	3.62250077866638e-280	EpC3	BRCA2
NCAPH	7.60933246869758e-283	0.31741243349953	0.204	0.003	1.52536678667512e-278	EpC3	NCAPH
HMGN2.1	1.1163093849062e-281	1.26921131845603	0.873	0.42	2.23775379298297e-277	EpC3	HMGN2
NCAPG2	2.00179773996764e-277	0.47598425718626	0.309	0.025	4.01280374953912e-273	EpC3	NCAPG2
MND1	9.68382214845878e-272	0.380870954243851	0.218	0.006	1.94121898788005e-267	EpC3	MND1
DEK.1	4.1338300404571e-268	1.13078023671435	0.891	0.442	8.28667569910031e-264	EpC3	DEK
KIF14	1.14767110198729e-266	0.380103752826918	0.203	0.004	2.30062149104372e-262	EpC3	KIF14

CDCA2	1.66820590255882e-265	0.353824923549516	0.222	0.007	3.34408555226941e-261	EpC3	CDCA2
ORC6	6.59499360482428e-263	0.409681418665983	0.266	0.017	1.32203241802308e-258	EpC3	ORC6
SKA1	4.91478606714954e-257	0.307421346395232	0.186	0.002	9.85218015020797e-253	EpC3	SKA1
NCAPD2	2.82839293204479e-253	0.482099567304347	0.309	0.03	5.66979647157699e-249	EpC3	NCAPD2
HELLS	3.29022574316375e-250	0.715519651468626	0.426	0.072	6.59558652474605e-246	EpC3	HELLS
DTYMK	7.08100328763488e-246	0.658117020364812	0.484	0.099	1.41945791903929e-241	EpC3	DTYMK
DEPDC1B	2.31552508453493e-243	0.271007524207614	0.171	0.001	4.64170158445872e-239	EpC3	DEPDC1B
PRC1	1.12777566162415e-242	0.283935628241149	0.196	0.005	2.26073909129176e-238	EpC3	PRC1
TROAP	8.59144456984914e-240	0.332698269870905	0.192	0.005	1.72224097847196e-235	EpC3	TROAP
GINS2	3.67887518984546e-239	0.549525370873252	0.302	0.031	7.37467320556421e-235	EpC3	GINS2
FAM111B	2.81811888027969e-237	0.559997894257317	0.299	0.031	5.64920110740867e-233	EpC3	FAM111B
CDCA5	1.91113579239771e-236	0.268886537290917	0.175	0.003	3.83106280944044e-232	EpC3	CDCA5
PSRC1	1.02926729967746e-233	0.307264516909367	0.181	0.004	2.06326922893344e-229	EpC3	PSRC1
HIST1H3B	4.28947062536411e-229	0.567750545614852	0.223	0.013	8.59867281560489e-225	EpC3	HIST1H3B
H2AFV.1	7.68088111515908e-229	1.02737318586239	0.733	0.299	1.53970942834479e-224	EpC3	H2AFV
ATAD5	2.37610432447876e-222	0.390123748820879	0.274	0.026	4.76313872885012e-218	EpC3	ATAD5
PRR11	3.26397310430199e-222	0.319963407505756	0.201	0.009	6.54296048488376e-218	EpC3	PRR11
BRCA1	1.3167445452314e-217	0.384311652410738	0.229	0.016	2.63954611537086e-213	EpC3	BRCA1
CDC6	5.19197304948742e-217	0.34709611943966	0.21	0.011	1.04078291750025e-212	EpC3	CDC6
ANP32E.1	1.35058396832193e-215	0.834725674359381	0.613	0.2	2.70738062289814e-211	EpC3	ANP32E
KNSTRN	1.80083474068589e-213	0.431005426438453	0.256	0.023	3.60995332117894e-209	EpC3	KNSTRN
CKS2.2	3.46650623907644e-213	1.05037272855447	0.564	0.172	6.94895840685262e-209	EpC3	CKS2
FEN1	4.19905341144185e-212	0.401367267949333	0.272	0.028	8.41742246857633e-208	EpC3	FEN1
VRK1	2.54675369892006e-209	0.5430975009666	0.401	0.076	5.10522246485515e-205	EpC3	VRK1
RRM1.1	2.93314002913424e-208	0.842394395931319	0.664	0.244	5.8797725024025e-204	EpC3	RRM1
PARPBP	2.37055266294438e-207	0.353235678990799	0.255	0.024	4.7520098681383e-203	EpC3	PARPBP
TRIP13	1.15645828095102e-206	0.285854102239897	0.202	0.011	2.31823626999441e-202	EpC3	TRIP13
MT2A.2	5.22068432248888e-206	1.26209728167277	0.851	0.435	1.04653837928612e-201	EpC3	MT2A
NUCKS1.1	4.20344686989202e-201	1.01396677406875	0.972	0.808	8.42622959538555e-197	EpC3	NUCKS1

HNRNPA2B1	1.3410994667359e-200	0.822484383006133	0.99	0.886	2.68836799101878e-196	EpC3	HNRNPA2B1
KPNA2.2	2.16869449528184e-197	1.09985320261521	0.543	0.169	4.34736498524199e-193	EpC3	KPNA2
RAD21.1	9.92873287792342e-196	1.01485356677751	0.791	0.419	1.99031379270853e-191	EpC3	RAD21
HMGB3.1	2.30868143346454e-190	0.931758273498735	0.702	0.303	4.62798280152301e-186	EpC3	HMGB3
KIF22	5.55627466490724e-190	0.482053678841198	0.369	0.07	1.1138108193273e-185	EpC3	KIF22
GMNN	1.9154096042176e-189	0.54204147402834	0.381	0.075	3.83963009261459e-185	EpC3	GMNN
HIST1H2AJ	2.04280078016963e-189	0.412816577540261	0.2	0.014	4.09499844392805e-185	EpC3	HIST1H2AJ
PCNA.1	4.25724893204961e-189	0.894616832284039	0.594	0.212	8.53408120918664e-185	EpC3	PCNA
FANCD2	7.8712817981842e-188	0.277868133084542	0.182	0.01	1.577877149264e-183	EpC3	FANCD2
HIST1H1A	6.54953322796602e-183	0.517062828683938	0.184	0.011	1.31291943087807e-178	EpC3	HIST1H1A
CENPH	7.58828702605965e-179	0.367682130394993	0.266	0.034	1.52114801724392e-174	EpC3	CENPH
RANBP1.1	1.25853460531545e-176	0.893173678985985	0.784	0.409	2.52285846981535e-172	EpC3	RANBP1
CIP2A	2.66183467860696e-174	0.290262739771873	0.181	0.012	5.33591379673551e-170	EpC3	CIP2A
CBX3.1	5.83401185193646e-173	0.81939696314801	0.898	0.597	1.16948601583918e-168	EpC3	CBX3
SPDL1.1	8.84484220319545e-173	0.705345078236255	0.506	0.154	1.77303706805256e-168	EpC3	SPDL1
RPA3	1.50005583658126e-172	0.713692849882658	0.553	0.187	3.0070119300108e-168	EpC3	RPA3
SKA2	7.85437943790912e-172	0.589564505770929	0.458	0.123	1.57448890212326e-167	EpC3	SKA2
FBXO5	3.90516428647389e-171	0.387301805303146	0.254	0.032	7.82829232866556e-167	EpC3	FBXO5
NUDT1	1.53036942071698e-170	0.527409440934963	0.434	0.11	3.06777854076925e-166	EpC3	NUDT1
PHF19	4.73920289629526e-166	0.306279005584932	0.21	0.02	9.50020612591349e-162	EpC3	PHF19
DNAJC9	1.4510363321139e-163	0.484140173010312	0.392	0.091	2.90874743135552e-159	EpC3	DNAJC9
MCM7	5.04802940013471e-163	0.530761681122209	0.399	0.096	1.011927973551e-158	EpC3	MCM7
MIS18BP1	7.36040726043245e-160	0.633433332920666	0.503	0.161	1.47546723942629e-155	EpC3	MIS18BP1
CENPJ	3.04684801654749e-159	0.357024992632866	0.238	0.031	6.1077115339711e-155	EpC3	CENPJ
TMEM106C	1.86360629647327e-158	0.6233372755936	0.439	0.124	3.73578518191032e-154	EpC3	TMEM106C
NCAPD3	6.60208035879296e-158	0.416121407811807	0.305	0.056	1.32345302872364e-153	EpC3	NCAPD3
GGH.1	1.1026322762182e-156	0.539816172735742	0.452	0.129	2.21033666090701e-152	EpC3	GGH
CCDC18	8.40110901538671e-156	0.342921224885253	0.209	0.023	1.68408631322442e-151	EpC3	CCDC18
RAN.1	1.11628268110302e-155	0.794176967051964	0.925	0.723	2.23770026253911e-151	EpC3	RAN

TUBA1B.1	7.44102735777288e-154	2.19894010305509	0.552	0.236	1.49162834413915e-149	EpC3	TUBA1B
ITGB3BP	1.56809676827312e-152	0.459998054566507	0.345	0.075	3.14340678168029e-148	EpC3	ITGB3BP
DNMT1	1.83872643765191e-152	0.579693295794585	0.458	0.136	3.68591101691702e-148	EpC3	DNMT1
RFC3	2.18799564281779e-150	0.302697009877684	0.217	0.026	4.38605606559254e-146	EpC3	RFC3
MCM4	6.47021684173352e-149	0.634338438478074	0.495	0.164	1.2970196680939e-144	EpC3	MCM4
DUT	2.8876242829341e-147	0.908924848800817	0.727	0.384	5.78853163756971e-143	EpC3	DUT
CCDC34	1.74281871783946e-146	0.63413679269741	0.509	0.171	3.49365440178098e-142	EpC3	CCDC34
DDX39A	2.30097096959458e-145	0.603165483137348	0.506	0.175	4.6125264056493e-141	EpC3	DDX39A
HMGB1P5	1.62298988647912e-144	0.551746978895378	0.527	0.187	3.25344552643605e-140	EpC3	HMGB1P5
ARL6IP1.2	1.2210590309314e-143	1.35151194457585	0.695	0.391	2.44773493340508e-139	EpC3	ARL6IP1
MIS18A	3.05555670956322e-143	0.346989621375655	0.264	0.045	6.12516897999044e-139	EpC3	MIS18A
TMEM97	1.08136559677576e-141	0.432402246456844	0.352	0.084	2.16770547529669e-137	EpC3	TMEM97
CKAP5	1.99516467561471e-141	0.588886714337712	0.48	0.162	3.99950710873724e-137	EpC3	CKAP5
MZT1	1.63508648937561e-140	0.462317334470159	0.364	0.092	3.27769437660236e-136	EpC3	MZT1
HIST1H1D.2	6.77445860776292e-140	1.11943454130353	0.338	0.084	1.35800797251216e-135	EpC3	HIST1H1D
ZWILCH	1.95218472445288e-134	0.315154964142504	0.255	0.045	3.91334949863824e-130	EpC3	ZWILCH
GPSM2	8.69646634356718e-132	0.297237506512029	0.208	0.029	1.74329364323148e-127	EpC3	GPSM2
CALM2.1	1.54367796863551e-131	0.944317177186386	0.948	0.853	3.09445685592674e-127	EpC3	CALM2
CENPX.1	2.26202694193645e-131	0.74296687830794	0.621	0.288	4.5344592078058e-127	EpC3	CENPX
SNRPG.1	3.63505501391429e-131	0.737286781996229	0.875	0.613	7.28683128089259e-127	EpC3	SNRPG
TMSB15A	2.65554892643823e-129	0.251472275867604	0.139	0.01	5.32331337793807e-125	EpC3	TMSB15A
H2AFX	4.41936165800885e-128	0.357310537810622	0.281	0.058	8.85905237964454e-124	EpC3	H2AFX
RFC4	6.95436523099092e-128	0.333596706850194	0.241	0.042	1.39407205420444e-123	EpC3	RFC4
WDR76	1.01239101685609e-122	0.263431569758385	0.185	0.024	2.02943903238973e-118	EpC3	WDR76
NSD2	1.3603163376221e-122	0.337190902994946	0.278	0.059	2.72689013039725e-118	EpC3	NSD2
ANP32B	1.17104238833318e-121	0.670160216030243	0.747	0.428	2.3474715716527e-117	EpC3	ANP32B
LSM4.1	1.83402274797945e-121	0.56474190585436	0.594	0.266	3.67648200059961e-117	EpC3	LSM4
SIVA1	2.11889626633766e-121	0.631612876333083	0.593	0.271	4.24753945550048e-117	EpC3	SIVA1
CSE1L	1.71752433784485e-120	0.513773862622898	0.483	0.179	3.44294928764378e-116	EpC3	CSE1L

MASTL	4.08574139612592e-119	0.281589913095771	0.194	0.028	8.19027720267401e-115	EpC3	MASTL
TTF2	1.67677397629173e-116	0.350921529310841	0.269	0.058	3.3612611128744e-112	EpC3	TTF2
EXOSC8	6.97340451422635e-115	0.567230870603671	0.574	0.255	1.39788866892181e-110	EpC3	EXOSC8
CEP152	8.2114392806166e-115	0.322578398916731	0.232	0.043	1.6460651181924e-110	EpC3	CEP152
MCM3	9.39612664918396e-115	0.444034069666954	0.336	0.092	1.88354754809542e-110	EpC3	MCM3
EZH2	4.30008036351929e-113	0.474046816480794	0.399	0.13	8.61994109671076e-109	EpC3	EZH2
BUB3.1	9.41113338044833e-113	0.69342675501335	0.641	0.332	1.88655579744467e-108	EpC3	BUB3
LSM3.1	9.87465955127726e-113	0.634227230609986	0.733	0.422	1.97947425364904e-108	EpC3	LSM3
HIST1H2AH	3.05933308443362e-110	0.338809575292339	0.186	0.028	6.13273910105564e-106	EpC3	HIST1H2AH
UBE2S	5.00141482519524e-109	0.547402671330335	0.44	0.165	1.00258361585864e-104	EpC3	UBE2S
NRM	7.28381879395884e-109	0.331049380000303	0.298	0.075	1.46011431543699e-104	EpC3	NRM
MCM6	7.05832074838991e-108	0.303700562977346	0.216	0.039	1.41491097722224e-103	EpC3	MCM6
DBF4	8.99135578368139e-108	0.35602290501239	0.266	0.061	1.80240718039677e-103	EpC3	DBF4
LIG1	5.01345083597492e-107	0.316913652450092	0.26	0.058	1.00499635457953e-102	EpC3	LIG1
PHGDH	1.50669453563771e-106	0.433999990571577	0.411	0.142	3.02031986613935e-102	EpC3	PHGDH
HNRNPA3	2.4569826023101e-106	0.619392656428984	0.885	0.657	4.92526732459082e-102	EpC3	HNRNPA3
PTN	4.77725147299991e-106	0.781576966849278	0.756	0.458	9.57647830277563e-102	EpC3	PTN
SMC1A	5.56707030654102e-105	0.495690428509442	0.474	0.188	1.11597491364921e-100	EpC3	SMC1A
TUBB4B.1	1.26613057060512e-104	0.684018698250631	0.635	0.335	2.53808534183503e-100	EpC3	TUBB4B
PTMAP2	4.08812798594487e-104	0.530806697135841	0.646	0.335	8.19506136062508e-100	EpC3	PTMAP2
LBR	9.33380787455177e-103	0.514848543724445	0.47	0.188	1.87105512653265e-98	EpC3	LBR
MT-CO3.1	1.5146650366466e-102	0.377255769858495	1	1	3.03629753246177e-98	EpC3	MT-CO3
CHEK1	2.72979591470893e-102	0.257678009837806	0.186	0.03	5.47214889062553e-98	EpC3	CHEK1
PABPC1	7.00586299599469e-101	-0.463432808	0.998	1	1.4043952961771e-96	EpC3	PABPC1
PAICS	1.38689153948727e-96	0.513251174276237	0.534	0.246	2.78016278005619e-92	EpC3	PAICS
SNRPB	2.35085361854804e-94	0.557939084895402	0.656	0.365	4.71252116374141e-90	EpC3	SNRPB
MT-ATP6.1	5.42883297124697e-94	0.409192111881179	1	1	1.08826385741617e-89	EpC3	MT-ATP6
HIST1H4C.2	8.10438760072704e-94	2.16669920468824	0.789	0.633	1.62460553844174e-89	EpC3	HIST1H4C
ACAT2.1	1.22878971455161e-93	0.389684173648578	0.343	0.11	2.46323186179016e-89	EpC3	ACAT2

PSIP1	1.26196749329876e-93	0.383971501718187	0.324	0.1	2.5297400370667e-89	EpC3	PSIP1
NASP	1.30583778072071e-93	0.498328785181061	0.509	0.225	2.61768241523273e-89	EpC3	NASP
CDCA4	1.36586083200469e-93	0.292442646918825	0.243	0.058	2.7380046238366e-89	EpC3	CDCA4
RFC5	3.48453837216176e-93	0.279814254052884	0.236	0.054	6.98510562083547e-89	EpC3	RFC5
SNRPD1	8.12812619954974e-93	0.586330710122275	0.783	0.511	1.62936417796174e-88	EpC3	SNRPD1
USP1	1.5864634933139e-92	0.472624513950293	0.423	0.165	3.18022471869705e-88	EpC3	USP1
MT-ND4	2.30101897039643e-92	0.336971583645551	1	1	4.61262262805669e-88	EpC3	MT-ND4
WDR34	3.44163137200307e-92	0.306624687761038	0.281	0.077	6.89909424831736e-88	EpC3	WDR34
MT-CO2.2	1.13110830008817e-90	0.418021391859843	1	1	2.26741969835675e-86	EpC3	MT-CO2
PRKDC	1.68025077055113e-90	0.572923263362319	0.631	0.339	3.36823069464681e-86	EpC3	PRKDC
MRPL51	3.80740867903874e-90	0.626967164592386	0.802	0.567	7.63233143800105e-86	EpC3	MRPL51
NMU.1	5.16403539722313e-90	0.511960320852499	0.424	0.169	1.03518253572735e-85	EpC3	NMU
LSM5	5.24379695834115e-90	0.54660887806831	0.627	0.338	1.05117153826907e-85	EpC3	LSM5
CBX5	1.58118736016688e-89	0.462107067674023	0.465	0.196	3.16964818219052e-85	EpC3	CBX5
CACYBP	4.90262853507164e-89	0.513884343836623	0.674	0.379	9.8278091614046e-85	EpC3	CACYBP
PFN1.2	9.44514710606219e-89	0.511139957620206	0.981	0.96	1.89337418888123e-84	EpC3	PFN1
PA2G4.2	1.65750631277276e-88	0.563377912899201	0.728	0.445	3.32263715458427e-84	EpC3	PA2G4
HP1BP3	3.0639614800563e-88	0.65541126084775	0.808	0.579	6.14201718292086e-84	EpC3	HP1BP3
RFC2	6.02088861645584e-88	0.363749684094189	0.287	0.084	1.20694733205474e-83	EpC3	RFC2
XPO1	1.17247801135947e-87	0.460740866996949	0.548	0.266	2.35034942157119e-83	EpC3	XPO1
RBMX	3.55689306921768e-87	0.639625604207962	0.782	0.537	7.13014784655376e-83	EpC3	RBMX
HNRNPD	5.71820713115134e-87	0.552841527207202	0.818	0.564	1.1462718015106e-82	EpC3	HNRNPD
TCOF1	2.45414710960783e-86	0.303701551668462	0.271	0.076	4.91958329591985e-82	EpC3	TCOF1
HIST1H2BH	2.54528625464432e-86	0.284934763009875	0.189	0.038	5.10228082606e-82	EpC3	HIST1H2BH
FAM111A	1.59117139299899e-84	0.414277306094262	0.32	0.106	3.18966217440578e-80	EpC3	FAM111A
SASS6	8.41419411700437e-84	0.251465174078683	0.185	0.037	1.6867093526947e-79	EpC3	SASS6
MTHFD1	3.84714161194972e-83	0.31804490620018	0.304	0.096	7.71198007531441e-79	EpC3	MTHFD1
SRSF10	3.9468048496368e-83	0.514636781940902	0.656	0.373	7.91176500158192e-79	EpC3	SRSF10
POLD3	1.48335887356587e-82	0.347847392061343	0.273	0.081	2.97400197950144e-78	EpC3	POLD3

HMGN1.1	1.49613084614666e-82	0.538989920932233	0.84	0.602	2.99914389418559e-78	EpC3	HMGN1
NCL	1.76284754245143e-82	0.509819831212937	0.964	0.855	3.53380418359815e-78	EpC3	NCL
CCT5.1	2.25051233679891e-81	0.537668920464001	0.656	0.379	4.51137703034709e-77	EpC3	CCT5
CEP295	6.13318278900181e-80	0.341688110720731	0.279	0.085	1.2294578218833e-75	EpC3	CEP295
SNRPD2	6.35329855888456e-80	0.462634363722943	0.973	0.877	1.273582229114e-75	EpC3	SNRPD2
ILF2.2	2.4509660921594e-79	0.524276125949364	0.677	0.411	4.91320662834273e-75	EpC3	ILF2
MGME1	2.93655918596246e-79	0.318846657959225	0.289	0.091	5.88662654418035e-75	EpC3	MGME1
HSPD1	7.08292668689666e-78	0.532517099769348	0.867	0.634	1.41984348365531e-73	EpC3	HSPD1
MIR1244-2	1.40840814562215e-76	0.379792268039054	0.466	0.211	2.82329496871415e-72	EpC3	MIR1244-2
RBBP7	5.21016514675028e-76	0.450061302912064	0.564	0.299	1.04442970531756e-71	EpC3	RBBP7
CEP78	2.10424735157443e-75	0.26748005143833	0.225	0.06	4.21817424096609e-71	EpC3	CEP78
TPM3.1	1.57871364145998e-74	0.542006467879172	0.806	0.579	3.16468936567067e-70	EpC3	TPM3
TUBG1	2.03256620902141e-74	0.346998659022211	0.321	0.116	4.07448222260431e-70	EpC3	TUBG1
HNRNPR	2.39494452723223e-74	0.491046849406773	0.738	0.479	4.80090579928974e-70	EpC3	HNRNPR
NOP58	1.29992103027782e-73	0.489205963383947	0.716	0.429	2.60582169729491e-69	EpC3	NOP58
SSRP1	2.44245637125939e-73	0.430374361152225	0.522	0.263	4.89614804182656e-69	EpC3	SSRP1
BARD1	4.08606642956005e-73	0.318376273570179	0.253	0.076	8.19092876469608e-69	EpC3	BARD1
MZT2B.1	1.85756964273043e-72	0.425496497980282	0.561	0.299	3.72368410581742e-68	EpC3	MZT2B
LYAR	4.02826073712878e-72	0.328337228981049	0.319	0.115	8.07505147364836e-68	EpC3	LYAR
SLC25A5.2	8.94115218154373e-72	0.546978123503845	0.958	0.888	1.79234336631226e-67	EpC3	SLC25A5
YBX1	2.70558900199766e-71	0.481069664914647	0.894	0.696	5.42362371340451e-67	EpC3	YBX1
BANF1	2.06778076820904e-70	0.511440313339503	0.752	0.51	4.14507332795185e-66	EpC3	BANF1
RPA2	3.13511250440006e-70	0.331290666770033	0.358	0.143	6.28464652632037e-66	EpC3	RPA2
TPRKB	1.6855107786476e-69	0.467132661566888	0.571	0.319	3.37877490687698e-65	EpC3	TPRKB
KPNB1	1.94908622484867e-69	0.392203519669659	0.484	0.238	3.90713824633165e-65	EpC3	KPNB1
H2AFY.1	5.90195034958385e-69	0.471466638223495	0.613	0.364	1.18310496707758e-64	EpC3	H2AFY
SLBP	1.82617035683674e-68	0.376474079307738	0.367	0.152	3.66074109731493e-64	EpC3	SLBP
C19orf48	3.93372472764079e-68	0.338843566524933	0.367	0.15	7.88554458902873e-64	EpC3	C19orf48
CMSS1	7.38221016025668e-68	0.290215712056218	0.281	0.096	1.47983784872505e-63	EpC3	CMSS1

TMA7	7.59849686842454e-68	0.484645625981765	0.859	0.636	1.52319468224438e-63	EpC3	TMA7
RAD51C	1.15431379274109e-67	0.277156402006869	0.26	0.084	2.31393742892879e-63	EpC3	RAD51C
NAA38	1.23427981526277e-67	0.462468750672388	0.65	0.386	2.47423731767575e-63	EpC3	NAA38
PARP1	3.98883697895952e-67	0.435107087691091	0.521	0.277	7.99602260802226e-63	EpC3	PARP1
GAPDH	6.12726826442291e-67	0.83494745343176	0.798	0.56	1.22827219628622e-62	EpC3	GAPDH
DKC1	9.69889423769505e-67	0.358217190315066	0.439	0.203	1.94424033888835e-62	EpC3	DKC1
SRSF3	1.24717581016049e-66	0.462226353136328	0.859	0.651	2.50008862904771e-62	EpC3	SRSF3
TUBA1C.2	2.1519939276246e-66	0.642345822089373	0.365	0.156	4.31388702731628e-62	EpC3	TUBA1C
SMC3	1.23006520167072e-65	0.457613695139751	0.587	0.338	2.46578870326913e-61	EpC3	SMC3
SFPQ	8.36465117332105e-65	0.482929414846377	0.634	0.394	1.67677797420394e-60	EpC3	SFPQ
HNRNPA1.2	1.74275978210968e-64	0.40777845793103	0.982	0.893	3.49353625921706e-60	EpC3	HNRNPA1
CRNDE.1	3.68192347696201e-64	0.410786961284043	0.478	0.241	7.38078380191804e-60	EpC3	CRNDE
PSME2.1	3.71463406779238e-64	0.460482711856221	0.604	0.352	7.44635545229661e-60	EpC3	PSME2
SYNE2	4.23078997807171e-64	0.55856181945005	0.662	0.415	8.48104159004255e-60	EpC3	SYNE2
HSP90B1	4.58443298810511e-64	0.51134124309603	0.917	0.817	9.18995436795549e-60	EpC3	HSP90B1
POLE3	1.08647614709579e-63	0.357447357483624	0.431	0.204	2.17795008446822e-59	EpC3	POLE3
TEX30	2.35224157291663e-63	0.251262077711642	0.206	0.059	4.71530345706869e-59	EpC3	TEX30
ERH	5.54002503715119e-63	0.443843702834074	0.807	0.587	1.11055341894733e-58	EpC3	ERH
SUPT16H	7.93283484896823e-63	0.469060979030423	0.57	0.331	1.59021607382417e-58	EpC3	SUPT16H
TPI1.2	7.96178177892368e-63	0.47350272875231	0.966	0.898	1.59601877540304e-58	EpC3	TPI1
MTHFD2	9.32924705919094e-63	0.256545500764223	0.253	0.084	1.87014086548541e-58	EpC3	MTHFD2
UQCRC1	9.44786156050972e-63	0.449929080777325	0.729	0.483	1.89391832841978e-58	EpC3	UQCRC1
PSMC3	1.89001044519996e-62	0.393895387486215	0.525	0.286	3.78871493844783e-58	EpC3	PSMC3
FDPS.2	3.31079351459412e-62	0.426904734655423	0.388	0.173	6.63681667935538e-58	EpC3	FDPS
PTGES3	3.49300146831945e-62	0.470880855110009	0.764	0.537	7.00207074339317e-58	EpC3	PTGES3
HSPB11	8.8317417949795e-62	0.449564115344279	0.541	0.307	1.77041096022159e-57	EpC3	HSPB11
VDAC3.2	3.44881238690733e-61	0.396387424903639	0.54	0.301	6.91348931079443e-57	EpC3	VDAC3
MTATP6P1	1.97117912327044e-60	0.54660572977168	0.997	0.976	3.95142567050793e-56	EpC3	MTATP6P1
NPM1	2.35978921906935e-60	0.416476707550205	0.998	0.938	4.73043346854641e-56	EpC3	NPM1

SAE1	4.52758245205339e-60	0.338097369596706	0.401	0.187	9.07599178338623e-56	EpC3	SAE1
CDCA7	7.24690835527164e-60	0.28384373154297	0.244	0.082	1.45271524889775e-55	EpC3	CDCA7
MT1X.1	1.12825746601222e-59	0.5759466553412	0.865	0.715	2.2617049163681e-55	EpC3	MT1X
HADH	4.88612969836221e-59	0.289817190750524	0.318	0.128	9.79473559333688e-55	EpC3	HADH
BRD8	5.19076904312014e-59	0.352190986562423	0.378	0.171	1.04054156238386e-54	EpC3	BRD8
HIST1H1E.1	8.37116371310653e-59	0.956118045988132	0.518	0.318	1.67808347792933e-54	EpC3	HIST1H1E
EXOSC9	2.43835231626973e-58	0.276549989432895	0.263	0.094	4.8879210531943e-54	EpC3	EXOSC9
MT-ND4L	3.23342638321026e-58	0.408769598015563	0.999	0.975	6.4817265277833e-54	EpC3	MT-ND4L
TOPBP1	9.31671034755117e-58	0.269221169049952	0.28	0.104	1.86762775627011e-53	EpC3	TOPBP1
MT-CYB	1.15289843481344e-57	0.274695088635295	1	1	2.31110020242702e-53	EpC3	MT-CYB
PPIH	1.64491082603406e-57	0.264174018404228	0.278	0.104	3.29738824186787e-53	EpC3	PPIH
BOLA3	1.77142788480575e-57	0.388756525653179	0.518	0.284	3.55100433788161e-53	EpC3	BOLA3
FAM96A	1.96401275171332e-57	0.385727146377299	0.502	0.277	3.93705996208452e-53	EpC3	FAM96A
NDUFA6.1	6.71481376871932e-57	0.445396321559058	0.811	0.613	1.34605156807747e-52	EpC3	NDUFA6
CMC2.1	1.14625947463065e-56	0.404495695492786	0.471	0.253	2.29779174284461e-52	EpC3	CMC2
SNRPA1	1.31913073662416e-56	0.384745573375707	0.481	0.259	2.64432947463679e-52	EpC3	SNRPA1
NUP107	2.90105406688449e-56	0.252357395884752	0.245	0.086	5.81545298247664e-52	EpC3	NUP107
NUDCD2	3.32885366567235e-56	0.409959823730701	0.486	0.272	6.67302005820679e-52	EpC3	NUDCD2
MZT2A.1	5.09632919702137e-56	0.345877688999172	0.425	0.212	1.0216101508349e-51	EpC3	MZT2A
ANAPC11.1	6.27827413985437e-56	0.479411190530877	0.767	0.573	1.25854283407521e-51	EpC3	ANAPC11
FXYD3.1	1.43233736296399e-55	-0.517348764	0.85	0.943	2.87126347779761e-51	EpC3	FXYD3
RUVBL2	1.73006436218597e-55	0.313087552304012	0.383	0.177	3.46808702043799e-51	EpC3	RUVBL2
HNRNPF	1.73167282757108e-55	0.415769080646462	0.663	0.441	3.47131135014899e-51	EpC3	HNRNPF
HPRT1	2.0633163686798e-54	0.308460804705097	0.368	0.169	4.13612399265553e-50	EpC3	HPRT1
ANXA5	3.33089239889641e-54	0.396902427030528	0.586	0.349	6.67710690282773e-50	EpC3	ANXA5
LSM2	5.15525293595569e-54	0.363629984191072	0.466	0.251	1.03342200354168e-49	EpC3	LSM2
ENO1.2	1.15051465285557e-53	0.443824997622075	0.895	0.766	2.30632167311427e-49	EpC3	ENO1
SRSF2	1.20451564120793e-53	0.36217055004404	0.632	0.388	2.41457205436541e-49	EpC3	SRSF2
PRDX2	2.28646653108519e-53	0.567396803226379	0.653	0.433	4.58345080821336e-49	EpC3	PRDX2

LMNA	5.85614346963098e-53	0.484340568946898	0.765	0.58	1.17392251992223e-48	EpC3	LMNA
EBP	1.31782801938197e-52	0.32073258234595	0.432	0.221	2.6417180476531e-48	EpC3	EBP
PAFAH1B3	2.00255983751811e-52	0.393893496916046	0.481	0.265	4.01433145028881e-48	EpC3	PAFAH1B3
CSTB.2	4.20504088182847e-52	-1.513827728	0.886	0.95	8.42942495171336e-48	EpC3	CSTB
RHEB.1	7.18367285589865e-52	0.34187042101868	0.428	0.222	1.44003906069344e-47	EpC3	RHEB
NOP56	7.30613084955189e-52	0.374233087812867	0.474	0.262	1.46458699010117e-47	EpC3	NOP56
HNRNPM	7.5725980166799e-52	0.410880567393581	0.694	0.472	1.51800299842365e-47	EpC3	HNRNPM
VKORC1	9.92851027343595e-52	0.3306537729596	0.33	0.149	1.99026916941297e-47	EpC3	VKORC1
CDK5RAP2	1.09150839651305e-51	0.272367710723294	0.255	0.097	2.18803773165007e-47	EpC3	CDK5RAP2
MAGOHB	3.14008394555538e-51	0.321107388289178	0.359	0.169	6.29461227726031e-47	EpC3	MAGOHB
POLD2	6.19210070037565e-51	0.344060924564331	0.486	0.271	1.2412685063973e-46	EpC3	POLD2
RECQL	1.3217570153993e-50	0.28409595319403	0.283	0.116	2.64959411306944e-46	EpC3	RECQL
LGALS1.1	1.7668430001672e-50	0.940149889404394	0.492	0.286	3.54181347813516e-46	EpC3	LGALS1
FBL	2.71445516430516e-50	0.368687910158446	0.578	0.357	5.44139682236613e-46	EpC3	FBL
SNRPF	5.97235495466258e-50	0.386652951180063	0.696	0.47	1.19721827421166e-45	EpC3	SNRPF
CD24.2	7.22689573823751e-50	-0.905117307	0.494	0.659	1.44870351968709e-45	EpC3	CD24
NAP1L1.1	1.95379673766499e-49	0.40672738384209	0.741	0.516	3.91658094032324e-45	EpC3	NAP1L1
HSPE1	3.3023187735265e-49	0.443520964297799	0.848	0.681	6.61982821341122e-45	EpC3	HSPE1
SNRPC	3.90833124473453e-49	0.399181840738348	0.693	0.476	7.83464081319485e-45	EpC3	SNRPC
CEP57	4.56114465418025e-49	0.318815558509087	0.4	0.201	9.14327057376973e-45	EpC3	CEP57
PCM1	6.45051463990609e-49	0.367363124729353	0.522	0.306	1.29307016471558e-44	EpC3	PCM1
HNRNPAB	1.33598140278315e-48	0.332364241027473	0.421	0.221	2.6781083200191e-44	EpC3	HNRNPAB
MSH2	4.2396178409447e-48	0.250491613052001	0.233	0.087	8.49873792395775e-44	EpC3	MSH2
XRCC5	7.12441040536186e-48	0.410689952605691	0.848	0.693	1.42815930985884e-43	EpC3	XRCC5
UCHL5	7.2946288032831e-48	0.288061489319107	0.384	0.19	1.46228128990613e-43	EpC3	UCHL5
COMMD4	9.99372987714919e-48	0.28986278760636	0.346	0.164	2.00334309117333e-43	EpC3	COMMD4
TAF15	1.00588798191042e-47	0.35022483324097	0.49	0.284	2.01640304853762e-43	EpC3	TAF15
LGALS3.2	2.17477989451247e-47	-0.739833484	0.518	0.648	4.3595637765397e-43	EpC3	LGALS3
NUP37	3.28301382044795e-47	0.251696540288275	0.284	0.121	6.58112950446997e-43	EpC3	NUP37

MRTO4	3.32677905566752e-47	0.296484152706574	0.354	0.17	6.66886129499112e-43	EpC3	MRTO4
CALM3	5.36527521634535e-47	0.260603124410526	0.29	0.125	1.07552306986859e-42	EpC3	CALM3
PHPT1	5.40891329699023e-47	0.422422792326624	0.66	0.449	1.08427075951466e-42	EpC3	PHPT1
HINT2	1.03008264153402e-46	0.320108047232035	0.461	0.252	2.06490366321909e-42	EpC3	HINT2
ACTB.2	3.95161252728133e-46	0.346919917891379	0.996	0.988	7.92140247218814e-42	EpC3	ACTB
SERPINB3.2	5.391097529398e-46	-2.293000217	0.257	0.465	1.08069941074312e-41	EpC3	SERPINB3
MT-ND2	6.6883994346335e-46	0.25603716540579	1	1	1.34075655066663e-41	EpC3	MT-ND2
HAT1	8.09409871983372e-46	0.319610360536797	0.36	0.18	1.62254302937787e-41	EpC3	HAT1
SNRPA	8.4766660270331e-46	0.258425484594915	0.31	0.14	1.69923247177905e-41	EpC3	SNRPA
XRCC6	8.66151305960106e-46	0.341235868956391	0.658	0.438	1.73628690792763e-41	EpC3	XRCC6
CYC1.1	1.37952515398151e-45	0.386977653480984	0.691	0.467	2.76539612367134e-41	EpC3	CYC1
RNASEH2B	1.67013859721609e-45	0.292189079953426	0.35	0.169	3.34795983197938e-41	EpC3	RNASEH2B
FAM83D	1.75283268923354e-45	0.263444463759695	0.291	0.126	3.51372840883756e-41	EpC3	FAM83D
MT-CO1.1	3.85108871090039e-45	0.252237699593242	1	1	7.71989242987092e-41	EpC3	MT-CO1
FUS	4.6441838858623e-45	0.345683023139713	0.61	0.392	9.30973101759956e-41	EpC3	FUS
SRP9	4.68524449339557e-45	0.390016235496875	0.814	0.654	9.39204111146076e-41	EpC3	SRP9
EWSR1	1.39188452572731e-44	0.383306771609595	0.633	0.427	2.79017172027297e-40	EpC3	EWSR1
LDHB.2	1.81341667425785e-44	0.385215010399871	0.794	0.562	3.63517506521728e-40	EpC3	LDHB
NAP1L4	2.7378618999422e-44	0.353992835409602	0.536	0.331	5.48831796462413e-40	EpC3	NAP1L4
TIMM10	3.24432764849215e-44	0.319906552655253	0.459	0.26	6.50357920416737e-40	EpC3	TIMM10
C12orf57	5.14608566256781e-44	0.364564584831505	0.636	0.419	1.03158433191834e-39	EpC3	C12orf57
DCXR.2	8.11411637289204e-44	0.333444221474566	0.486	0.286	1.62655576810994e-39	EpC3	DCXR
SRSF7	1.11585549922175e-43	0.348083561829991	0.845	0.658	2.23684393373991e-39	EpC3	SRSF7
TPR	1.55883686536571e-43	0.398685063872715	0.615	0.407	3.12484438031209e-39	EpC3	TPR
COX20	2.30571017515982e-43	0.272736960620352	0.248	0.101	4.62202661712538e-39	EpC3	COX20
MT-ND5.1	2.73016203028698e-43	0.257838493190936	1	1	5.47288280591329e-39	EpC3	MT-ND5
HNRNPH3	2.95916350685967e-43	0.328817045624652	0.561	0.353	5.9319391658509e-39	EpC3	HNRNPH3
YWHAQ.1	3.44524439815108e-43	0.382597647118077	0.74	0.551	6.90633692053365e-39	EpC3	YWHAQ
RAB8A	3.47056874128759e-43	0.306338350108536	0.427	0.235	6.9571020987851e-39	EpC3	RAB8A

LSM6	4.64830942475074e-43	0.297694434309705	0.414	0.223	9.31800107285534e-39	EpC3	LSM6
UBC	6.95414558343311e-43	-0.364311975	0.907	0.966	1.394028023655e-38	EpC3	UBC
NTS.2	7.47633146918883e-43	0.701815198215156	0.527	0.326	1.49870540631359e-38	EpC3	NTS
FUBP1	8.02410363470586e-43	0.3120323493029	0.457	0.263	1.60851181461314e-38	EpC3	FUBP1
HIST1H1C.1	1.16368314304668e-42	1.05374206787776	0.546	0.396	2.33271922855137e-38	EpC3	HIST1H1C
CNTRL	1.44923370890229e-42	0.262029197315439	0.272	0.119	2.90513389286553e-38	EpC3	CNTRL
SYNCRIP	1.85460177504836e-42	0.35821322	0.722	0.515	3.71773471826193e-38	EpC3	SYNCRIP
BTG3.1	2.90122426055001e-42	0.34023131174262	0.462	0.268	5.81579415269854e-38	EpC3	BTG3
MIF	3.14602376946469e-42	0.291192862562351	0.277	0.122	6.30651924826892e-38	EpC3	MIF
IMPDH2	3.33889066344577e-42	0.294306059983917	0.559	0.343	6.6931402239434e-38	EpC3	IMPDH2
NFKBIA.1	4.0837663377524e-42	-0.631169594	0.605	0.746	8.18631800065846e-38	EpC3	NFKBIA
SRRM1	4.7021266715487e-42	0.352126908760897	0.774	0.57	9.42588312578653e-38	EpC3	SRRM1
SNX5	4.72189074623868e-42	0.254990492710749	0.372	0.19	9.46550218991006e-38	EpC3	SNX5
PLP2.2	5.36921968825045e-42	0.392750061766754	0.908	0.835	1.07631377870668e-37	EpC3	PLP2
NHP2	9.43480761869206e-42	0.360782478108595	0.399	0.212	1.89130153524301e-37	EpC3	NHP2
HAUS1	9.57939258401381e-42	0.319164188317741	0.357	0.184	1.92028503739141e-37	EpC3	HAUS1
HACD3	1.02921447863825e-41	0.339188584413792	0.497	0.299	2.06316334387824e-37	EpC3	HACD3
GJA1.2	1.51825228370168e-41	-0.437796945	0.271	0.481	3.04348852790839e-37	EpC3	GJA1
CASP8AP2	3.23226382263594e-41	0.261048105784335	0.26	0.112	6.47939605885601e-37	EpC3	CASP8AP2
ILF3	5.07994153587435e-41	0.323164865599947	0.586	0.378	1.01832508028137e-36	EpC3	ILF3
TCP1	9.25428307524501e-41	0.348176766007488	0.549	0.355	1.85511358526361e-36	EpC3	TCP1
NUDC	1.22812453577533e-40	0.324684269461146	0.507	0.316	2.46189844441522e-36	EpC3	NUDC
TPGS2	1.8272994841996e-40	0.354092914371912	0.573	0.373	3.66300454602652e-36	EpC3	TPGS2
ETFB	1.98866642520671e-40	0.323312016563917	0.504	0.306	3.98648071596938e-36	EpC3	ETFB
ITM2B.2	2.1249261507973e-40	-0.405804391	0.871	0.896	4.25962696188828e-36	EpC3	ITM2B
COX7B.2	2.31690685590543e-40	0.344594567775578	0.93	0.814	4.64447148334802e-36	EpC3	COX7B
PERP.2	2.67083069498074e-40	-0.582697411	0.984	0.998	5.35394721115839e-36	EpC3	PERP
PSMA4	3.94725747655982e-40	0.375461603108965	0.786	0.613	7.91267233751182e-36	EpC3	PSMA4
PNRC1	4.42476625083146e-40	-0.502562167	0.329	0.52	8.86988642641674e-36	EpC3	PNRC1

SIGMAR1	9.37544767020592e-40	0.251020521404525	0.308	0.148	1.87940223996948e-35	EpC3	SIGMAR1
EIF2S2.1	1.02091258477951e-39	0.371003045653231	0.759	0.573	2.04652136744901e-35	EpC3	EIF2S2
PPP1CA.1	1.04702885043159e-39	0.339651485938355	0.476	0.281	2.09887403357517e-35	EpC3	PPP1CA
C1QBP.2	1.61983053095524e-39	0.341924161684381	0.625	0.415	3.24711228235287e-35	EpC3	C1QBP
PDCD5.2	1.99754834879549e-39	0.364979726661999	0.762	0.553	4.00428541999543e-35	EpC3	PDCD5
TCERG1	2.23214982234655e-39	0.262661540085925	0.383	0.204	4.4745675338759e-35	EpC3	TCERG1
NDUFB6	3.89822055818192e-39	0.362294655179019	0.731	0.548	7.81437293093147e-35	EpC3	NDUFB6
HMGN3	6.95563449105042e-39	0.392110503699842	0.796	0.605	1.39432649007597e-34	EpC3	HMGN3
ATP5ME.2	8.77655813799385e-39	0.324108844189092	0.792	0.584	1.75934884434225e-34	EpC3	ATP5ME
HNRNPU	1.16123047338968e-38	0.378519005292763	0.638	0.449	2.32780260695695e-34	EpC3	HNRNPU
PKM	4.45636554880303e-38	0.341697347804798	0.855	0.688	8.93323037913056e-34	EpC3	PKM
FGD5-AS1	5.80410781281878e-38	0.281246555007012	0.435	0.254	1.16349145215765e-33	EpC3	FGD5-AS1
GJB2.1	1.244465763797e-37	-0.771512782	0.177	0.357	2.49465607010747e-33	EpC3	GJB2
TAGLN2.2	1.64420228778608e-37	0.379953641200269	0.813	0.653	3.29596790609597e-33	EpC3	TAGLN2
RPS27.1	1.86066252809238e-37	-0.295712215	1	0.999	3.72988410381399e-33	EpC3	RPS27
PARK7	1.91794592409465e-37	0.349561756286121	0.752	0.564	3.84471439944014e-33	EpC3	PARK7
SULT2B1.2	2.13431573652039e-37	-0.563440572	0.132	0.311	4.27844932542877e-33	EpC3	SULT2B1
NAA50	3.09055446750876e-37	0.300281479552561	0.497	0.308	6.19532548556807e-33	EpC3	NAA50
LYRM2	3.51038268421267e-37	0.289334250820358	0.485	0.293	7.03691312877271e-33	EpC3	LYRM2
RBBP4	6.24905814248836e-37	0.26667535377141	0.451	0.267	1.25268619524322e-32	EpC3	RBBP4
EIF5A.2	6.53739502529504e-37	0.331063249418993	0.734	0.532	1.31048620677064e-32	EpC3	EIF5A
PHIP	9.46883607817905e-37	0.282461114409054	0.382	0.21	1.89812288023177e-32	EpC3	PHIP
DCTPP1	1.86297977490478e-36	0.292314299769177	0.354	0.191	3.73452925677413e-32	EpC3	DCTPP1
SNRPE	2.16615249513903e-36	0.338946110466266	0.78	0.592	4.34226929175571e-32	EpC3	SNRPE
LUC7L3	4.53575699833076e-36	0.330598632481392	0.524	0.336	9.09237847885385e-32	EpC3	LUC7L3
AURKAIP1.2	9.94406400650175e-36	0.306533750960867	0.59	0.398	1.99338707074334e-31	EpC3	AURKAIP1
RPP30	1.22916793283112e-35	0.252467121071032	0.346	0.184	2.46399003815327e-31	EpC3	RPP30
RHCG.1	1.38557043505419e-35	-1.646769865	0.025	0.17	2.77751449410962e-31	EpC3	RHCG
POLR2H	1.7506331041329e-35	0.283665258352828	0.39	0.222	3.50931912054481e-31	EpC3	POLR2H

SLPI.2	2.45690596471309e-35	-1.826465504	0.148	0.313	4.92511369686386e-31	EpC3	SLPI
CCT6A.2	2.87335878809341e-35	0.327732539989335	0.674	0.486	5.75993502661206e-31	EpC3	CCT6A
MRPL27	3.43772406153646e-35	0.30094721475875	0.514	0.325	6.891261653756e-31	EpC3	MRPL27
YPEL5	7.18685677094133e-35	-0.378570695	0.217	0.404	1.4406773083029e-30	EpC3	YPEL5
NOLC1	8.24855849090862e-35	0.292237400205862	0.488	0.303	1.65350603508754e-30	EpC3	NOLC1
EIF4EBP1	1.10128686209678e-34	0.254511325711873	0.308	0.157	2.20763964375921e-30	EpC3	EIF4EBP1
RPL34.1	1.15427234042828e-34	-0.287764181	0.999	0.994	2.31385433362253e-30	EpC3	RPL34
GGCT.2	1.34188448262094e-34	0.33662767722796	0.529	0.351	2.68994163386195e-30	EpC3	GGCT
DDT	1.92414458503533e-34	0.258228495259186	0.261	0.121	3.85714023516182e-30	EpC3	DDT
MT1E.1	2.01487788442332e-34	0.385728438965423	0.687	0.48	4.03902420711499e-30	EpC3	MT1E
JPT1.1	2.15630507974535e-34	0.500560734117799	0.714	0.59	4.32252916285752e-30	EpC3	JPT1
MT-ATP8	2.2510405798887e-34	0.297840151369797	0.962	0.888	4.5124359464449e-30	EpC3	MT-ATP8
G3BP1	3.04516977904086e-34	0.318749690174021	0.618	0.433	6.10434733906531e-30	EpC3	G3BP1
EEF1E1	3.74371784685463e-34	0.279300212311516	0.447	0.269	7.5046567958048e-30	EpC3	EEF1E1
LYPD3.2	4.14777799807561e-34	-0.850508356	0.471	0.582	8.31463577494236e-30	EpC3	LYPD3
PSMA7.2	7.08704601549441e-34	0.311193326966159	0.986	0.978	1.42066924426601e-29	EpC3	PSMA7
SET	8.38903272504787e-34	0.312807159273255	0.745	0.562	1.6816655000631e-29	EpC3	SET
NAE1	1.09610396630993e-33	0.257770567653669	0.412	0.24	2.19725001086489e-29	EpC3	NAE1
RPL35.1	1.1966342199004e-33	0.278361456402909	0.988	0.966	2.39877295721234e-29	EpC3	RPL35
PHB2	1.20661980008985e-33	0.289056009423452	0.53	0.344	2.41879005126011e-29	EpC3	PHB2
TACSTD2.2	1.41305909943438e-33	-0.992589054	0.573	0.666	2.83261827072615e-29	EpC3	TACSTD2
MDH1	3.22698219250542e-33	0.34318491579417	0.78	0.647	6.46880850309635e-29	EpC3	MDH1
CBX1	3.69756742255876e-33	0.302346523331313	0.638	0.448	7.4121436552613e-29	EpC3	CBX1
WBP11	4.54325633006072e-33	0.257724684258665	0.426	0.254	9.10741163923973e-29	EpC3	WBP11
BAX	4.70787730928547e-33	0.257853053394638	0.378	0.216	9.43741085419366e-29	EpC3	BAX
RPL18	7.74643069896375e-33	-0.274481443	0.998	0.997	1.55284949791427e-28	EpC3	RPL18
RAD23A	8.5508490530217e-33	0.272478563621338	0.442	0.268	1.71410320116873e-28	EpC3	RAD23A
HNRNPDL	8.60893485262442e-33	0.295963379374064	0.828	0.678	1.72574708055709e-28	EpC3	HNRNPDL
ID3.1	8.73860124938439e-33	0.349281585046543	0.693	0.497	1.75174000645159e-28	EpC3	ID3

SNRPD3	9.04262713274228e-33	0.318755182315861	0.774	0.603	1.81268503502952e-28	EpC3	SNRPD3
NDUFV1	1.12909610543296e-32	0.261566525668161	0.434	0.26	2.26338605295092e-28	EpC3	NDUFV1
PPIA	1.38541304884161e-32	0.276831201796802	0.991	0.972	2.77719899770789e-28	EpC3	PPIA
RPL3.1	1.51643200089539e-32	-0.274457643	0.999	0.985	3.0398395889949e-28	EpC3	RPL3
SRSF1	1.761311580387e-32	0.256040249829442	0.447	0.273	3.53072519404379e-28	EpC3	SRSF1
CSTA.2	1.79444137816417e-32	-1.894386014	0.529	0.642	3.59713718666789e-28	EpC3	CSTA
ARL2	2.27777594827747e-32	0.252018178899631	0.407	0.24	4.56602966591701e-28	EpC3	ARL2
SOSTDC1.2	2.60805101788829e-32	-0.833169574	0.384	0.519	5.22809907045887e-28	EpC3	SOSTDC1
MRPL11	2.6967055828175e-32	0.268964079445101	0.368	0.211	5.40581601131596e-28	EpC3	MRPL11
RIF1	3.16983281784081e-32	0.251854633287758	0.29	0.148	6.3542468666437e-28	EpC3	RIF1
MPC2	3.26819785275407e-32	0.271047858201772	0.39	0.228	6.5514294156308e-28	EpC3	MPC2
VPS29.2	3.48300972314467e-32	0.328675945113585	0.684	0.518	6.9820412910158e-28	EpC3	VPS29
NUDT21	5.45078420071304e-32	0.264806652919149	0.443	0.276	1.09266420087494e-27	EpC3	NUDT21
RPS27L	6.13433252007214e-32	0.318492850277519	0.631	0.452	1.22968829697366e-27	EpC3	RPS27L
KHDRBS1	6.60415417129145e-32	0.297516210698637	0.467	0.298	1.32386874517708e-27	EpC3	KHDRBS1
HNRNPH1	6.90856764970563e-32	0.27971852376087	0.568	0.387	1.38489147105999e-27	EpC3	HNRNPH1
NDUFAF8	6.97062377063013e-32	0.264266999378514	0.444	0.27	1.39733124106052e-27	EpC3	NDUFAF8
NME1.1	7.81018536620814e-32	0.306070201535772	0.632	0.449	1.56562975851008e-27	EpC3	NME1
ITGAE	1.36017848035819e-31	0.272565942219245	0.403	0.24	2.72661378172603e-27	EpC3	ITGAE
MDK	1.71510755732505e-31	0.253601674027973	0.349	0.193	3.4381046094138e-27	EpC3	MDK
FOS.2	1.7312756847337e-31	-0.472282592	0.876	0.954	3.47051523761717e-27	EpC3	FOS
TMPRSS11F.1	1.82187160611523e-31	-0.403185523	0.054	0.199	3.65212382161858e-27	EpC3	TMPRSS11F
PHB	2.05266302264192e-31	0.250985513914977	0.331	0.177	4.114768295188e-27	EpC3	PHB
TRA2B	2.44506547667643e-31	0.282435124591944	0.692	0.501	4.90137825454557e-27	EpC3	TRA2B
LSM8	2.46680427917517e-31	0.262456614648688	0.454	0.282	4.94495585803455e-27	EpC3	LSM8
CLIC1.2	2.59624882255884e-31	0.327209623199429	0.852	0.715	5.20444038970145e-27	EpC3	CLIC1
HSPB1.2	4.58967823092766e-31	-0.457312468	0.97	0.988	9.20046898171759e-27	EpC3	HSPB1
RFC1	4.67497202406308e-31	0.285925624298484	0.39	0.23	9.37144891943684e-27	EpC3	RFC1
GNAS	6.80517769006486e-31	0.397030069365703	0.708	0.541	1.3641659197504e-26	EpC3	GNAS

CFL1.2	7.09183043700669e-31	0.278561965008811	0.926	0.868	1.42162832940236e-26	EpC3	CFL1
PRDX4	7.56235551826665e-31	0.285371911060525	0.408	0.251	1.51594978719173e-26	EpC3	PRDX4
EIF2S1	9.06250661144537e-31	0.281407524438822	0.489	0.317	1.81667007533034e-26	EpC3	EIF2S1
ADIRF	1.07527158512252e-30	0.358939881026604	0.395	0.228	2.1554894195366e-26	EpC3	ADIRF
SEPT7	1.50773813323746e-30	0.302858308525053	0.815	0.661	3.02241186188781e-26	EpC3	SEPT7
ANXA1.2	3.39321191054624e-30	-1.276305615	0.976	0.986	6.802032595881e-26	EpC3	ANXA1
DDX46	3.97159896925972e-30	0.289008335910942	0.411	0.253	7.96146729377804e-26	EpC3	DDX46
DDX21	4.03448291822633e-30	0.351785879434746	0.596	0.425	8.0875244578765e-26	EpC3	DDX21
TKT.1	4.30816643697538e-30	0.299294774133444	0.775	0.585	8.63615043956084e-26	EpC3	TKT
EIF5B.1	4.77002899576766e-30	0.331831591598767	0.685	0.52	9.56200012491585e-26	EpC3	EIF5B
CD9.2	4.98283113880169e-30	-0.348956384	0.973	0.987	9.98858330084186e-26	EpC3	CD9
CYCS.2	5.29681498178338e-30	0.299690903430168	0.831	0.668	1.0617995312483e-25	EpC3	CYCS
CCT4	6.488783481393e-30	0.299090602802257	0.561	0.393	1.30074153668004e-25	EpC3	CCT4
VDAC1.2	6.96501670649646e-30	0.318251541657093	0.764	0.608	1.39620724898428e-25	EpC3	VDAC1
PRELID1	7.96474539743215e-30	0.260448998558033	0.254	0.126	1.59661286236925e-25	EpC3	PRELID1
ATP5MC1.2	8.53619271738292e-30	0.313389970533813	0.733	0.559	1.71116519212658e-25	EpC3	ATP5MC1
YWHAE	8.59436483118748e-30	0.269409424421902	0.894	0.775	1.72282637405984e-25	EpC3	YWHAE
ARL3	1.76861691503869e-29	0.252641783965438	0.395	0.236	3.54536946788655e-25	EpC3	ARL3
SUMO2	1.98241477121844e-29	0.292346483415841	0.913	0.867	3.97394865038448e-25	EpC3	SUMO2
COX17.2	2.00373042486889e-29	0.306638483775188	0.571	0.404	4.01667800969218e-25	EpC3	COX17
CXCL17.1	2.19037456098201e-29	-0.414631994	0.046	0.18	4.39082484494454e-25	EpC3	CXCL17
HSPA1A	2.8657068112737e-29	-0.445108693	0.662	0.79	5.74459587387925e-25	EpC3	HSPA1A
PRMT1	2.96731248234839e-29	0.26788298625013	0.626	0.44	5.94827460211559e-25	EpC3	PRMT1
HNRNPK	3.92258417838575e-29	0.296146899460143	0.874	0.756	7.86321224399208e-25	EpC3	HNRNPK
MORF4L1	4.0408398800843e-29	0.278342259093886	0.857	0.706	8.10026762361699e-25	EpC3	MORF4L1
PDIA6	5.15298434630412e-29	0.280893732889507	0.592	0.415	1.03296724206012e-24	EpC3	PDIA6
SPRR1B.2	5.21875251705187e-29	-2.788031651	0.311	0.451	1.04615112956822e-24	EpC3	SPRR1B
CCT2	6.6629110276747e-29	0.295775715654498	0.649	0.473	1.33564714460767e-24	EpC3	CCT2
CCT8	7.28603990315238e-29	0.281945532522143	0.587	0.424	1.46055955898593e-24	EpC3	CCT8

CDK2AP2	9.58860977676548e-29	0.262036549305817	0.366	0.219	1.92213271585041e-24	EpC3	CDK2AP2
NFKBIZ.1	1.14575400604145e-28	-0.541710624	0.237	0.405	2.2967784805107e-24	EpC3	NFKBIZ
FDFT1.2	1.14712640040546e-28	0.290522108401572	0.507	0.337	2.29952958225279e-24	EpC3	FDFT1
RBM8A	1.95017369361401e-28	0.302670589727577	0.828	0.69	3.90931818621865e-24	EpC3	RBM8A
TMPRSS11E.2	2.60530390297875e-28	-1.245245712	0.023	0.142	5.22259220391121e-24	EpC3	TMPRSS11E
CHCHD2.2	2.88593799146146e-28	0.318001141596437	0.9	0.819	5.78515129768364e-24	EpC3	CHCHD2
MRPL22	3.52985834302389e-28	0.25120968561239	0.422	0.265	7.07595403442569e-24	EpC3	MRPL22
ZFP36	3.67492103638937e-28	-0.430854296	0.477	0.615	7.36674670954612e-24	EpC3	ZFP36
UQCRQ.2	3.8715126823264e-28	0.28084081778586	0.92	0.839	7.76083432299151e-24	EpC3	UQCRQ
MORF4L2.1	4.75708152619487e-28	0.290033964675181	0.662	0.495	9.53604562741024e-24	EpC3	MORF4L2
KRT5	5.62486058956134e-28	0.283806239048629	0.977	0.922	1.12755955378347e-23	EpC3	KRT5
ARPC5.1	5.65958063688523e-28	0.30272087323708	0.737	0.574	1.13451953447001e-23	EpC3	ARPC5
HBEGF.2	7.22220071196673e-28	-0.53482905	0.193	0.345	1.44776235472085e-23	EpC3	HBEGF
ENY2	7.57584011456513e-28	0.261895054117975	0.616	0.432	1.51865290936573e-23	EpC3	ENY2
SLIRP.2	8.57737158286748e-28	0.309193344278029	0.75	0.585	1.71941990750161e-23	EpC3	SLIRP
CNIH4.1	1.02952206018794e-27	0.288735688135942	0.578	0.419	2.06377992185274e-23	EpC3	CNIH4
DNAJB1	1.13604863409922e-27	-0.45315704	0.737	0.834	2.2773230919153e-23	EpC3	DNAJB1
LSM7	1.18330007872178e-27	0.268786128223329	0.649	0.469	2.37204333780567e-23	EpC3	LSM7
AKR1C2.2	1.24364322005697e-27	0.536868294792741	0.587	0.441	2.49300719892621e-23	EpC3	AKR1C2
PRSS3.1	1.61289475378063e-27	-0.350122194	0.008	0.116	3.23320882342865e-23	EpC3	PRSS3
PRDX3.1	1.80972870811194e-27	0.316860512016529	0.681	0.536	3.6277821682812e-23	EpC3	PRDX3
EGR1.1	2.75339251231628e-27	-0.430124238	0.528	0.665	5.51945063018921e-23	EpC3	EGR1
AKR1C3.2	3.83624907667249e-27	0.468267273296936	0.73	0.574	7.69014489909767e-23	EpC3	AKR1C3
MAGOH	4.61128405188575e-27	0.255694903538986	0.5	0.338	9.24378001041017e-23	EpC3	MAGOH
SDR16C5.1	6.57750467787651e-27	-0.446143374	0.05	0.176	1.31852658772713e-22	EpC3	SDR16C5
COA6	8.11963794032287e-27	0.260527347270709	0.457	0.297	1.62766262151712e-22	EpC3	COA6
PDZK1IP1.2	8.3573557967225e-27	-0.838566285	0.04	0.161	1.67531554301099e-22	EpC3	PDZK1IP1
CWC15	1.1021593575488e-26	0.301258257574344	0.64	0.476	2.20938864814233e-22	EpC3	CWC15
HIST2H2AC	1.12392982692777e-26	0.26414753749667	0.219	0.108	2.2530297310594e-22	EpC3	HIST2H2AC

ATP5PF.2	1.13723396452688e-26	0.266876789540855	0.939	0.867	2.27969920529058e-22	EpC3	ATP5PF
SDCBP2.2	1.36192605044116e-26	-0.287413553	0.072	0.206	2.73011696071434e-22	EpC3	SDCBP2
IL1RN.2	1.49339014089956e-26	-1.313276019	0.245	0.385	2.99364987644726e-22	EpC3	IL1RN
GLUL.2	3.54491296537244e-26	-0.879104933	0.752	0.796	7.10613253038558e-22	EpC3	GLUL
ATP5F1B.1	3.69443993707111e-26	0.286626363463948	0.881	0.808	7.40587429785275e-22	EpC3	ATP5F1B
ATP5IF1	5.5907687670536e-26	0.271973255086436	0.786	0.634	1.12072550704356e-21	EpC3	ATP5IF1
ATP5MC3.1	5.6483956372418e-26	0.27517307067343	0.954	0.899	1.13227738944149e-21	EpC3	ATP5MC3
SERPINB1.1	6.44749472629859e-26	-1.070911019	0.21	0.349	1.29246479283381e-21	EpC3	SERPINB1
CLIC3.2	8.35637951285959e-26	-0.337865009	0.01	0.113	1.67511983714783e-21	EpC3	CLIC3
PTMS	9.48250083991592e-26	0.278618740151639	0.359	0.223	1.90086211836955e-21	EpC3	PTMS
FOSB.1	1.23564557037951e-25	-0.426507443	0.425	0.56	2.47697511038277e-21	EpC3	FOSB
HMGA1.2	1.23720164628072e-25	0.270577541857253	0.501	0.344	2.48009442013433e-21	EpC3	HMGA1
SERPINB4.2	1.32931279395286e-25	-1.977266108	0.094	0.229	2.66474042675791e-21	EpC3	SERPINB4
MRPL18	1.42368365298988e-25	0.25046669312425	0.558	0.391	2.8539162507835e-21	EpC3	MRPL18
CLCA4.2	1.92182330630778e-25	-1.03996922	0.013	0.117	3.85248699982457e-21	EpC3	CLCA4
KRT6C.2	4.8341037193943e-25	-0.505156579	0.05	0.171	9.69044431589781e-21	EpC3	KRT6C
UQCRFS1.1	5.31350010940999e-25	0.295379854385352	0.601	0.458	1.06514423193233e-20	EpC3	UQCRFS1
SERPINB11.2	6.00458621911838e-25	-0.362446681	0.036	0.15	1.20367935348447e-20	EpC3	SERPINB11
NDUFB2.2	6.02975252122306e-25	0.276647396131556	0.849	0.718	1.20872419040437e-20	EpC3	NDUFB2
DCTN3	6.50044359483823e-25	0.287218494478355	0.446	0.3	1.30307892302127e-20	EpC3	DCTN3
MLF2	7.51050078807222e-25	0.256762256088731	0.524	0.372	1.50555498797696e-20	EpC3	MLF2
TNFSF10.2	8.87613843882341e-25	-0.857126012	0.367	0.479	1.77931071144654e-20	EpC3	TNFSF10
BTF3.1	9.63955525946708e-25	-0.273346644	0.964	0.975	1.93234524731277e-20	EpC3	BTF3
KRT16.2	1.18747597513272e-24	-1.267188899	0.268	0.393	2.38041433975105e-20	EpC3	KRT16
EEF1D.1	2.15374373549929e-24	0.303798564079731	0.424	0.267	4.31739469218188e-20	EpC3	EEF1D
POLR2E.1	2.57978856069698e-24	0.264887967538701	0.561	0.401	5.17144414877317e-20	EpC3	POLR2E
SERPINB13.2	4.34400640349952e-24	-0.477491082	0.305	0.437	8.70799523645513e-20	EpC3	SERPINB13
GLO1.2	5.01589828089845e-24	0.252272722264978	0.58	0.426	1.0054869693889e-19	EpC3	GLO1
MINOS1.2	1.24453060052218e-23	0.250038788281409	0.562	0.406	2.49478604180675e-19	EpC3	MINOS1

NDUFA2	1.86494240802384e-23	0.276942969793907	0.589	0.436	3.73846355112459e-19	EpC3	NDUFA2
TMEM45B.2	2.90936257823734e-23	-0.27448438	0.035	0.143	5.83210822433456e-19	EpC3	TMEM45B
MUC15.2	5.0269234274436e-23	-0.346828896	0.135	0.267	1.00769707026534e-18	EpC3	MUC15
DNAJC8	5.87090958246461e-23	0.261539333695626	0.57	0.422	1.17688253490085e-18	EpC3	DNAJC8
SPRR2A.2	1.18162345260399e-22	-1.868859368	0.048	0.156	2.36868237308997e-18	EpC3	SPRR2A
IL36G.2	1.23284156125625e-22	-0.883897506	0.021	0.119	2.47135419369427e-18	EpC3	IL36G
CLDN1.1	1.30156631083183e-22	-0.401455817	0.077	0.198	2.60911982669349e-18	EpC3	CLDN1
PI3.2	1.668251260913e-22	-2.788220585	0.181	0.309	3.3441764776262e-18	EpC3	PI3
SERBP1.1	1.71951055417892e-22	0.253645543442232	0.811	0.662	3.44693085690707e-18	EpC3	SERBP1
ATF3.1	1.89208292982797e-22	-0.524324575	0.469	0.591	3.79286944113315e-18	EpC3	ATF3
TM4SF1.2	2.11947720349274e-22	-0.369619997	0.708	0.786	4.24870400212155e-18	EpC3	TM4SF1
MRPL13.2	2.23345582910404e-22	0.25351805165201	0.575	0.424	4.47718555502195e-18	EpC3	MRPL13
S100A8.2	2.24524422571633e-22	-3.015706873	0.135	0.26	4.50081657487095e-18	EpC3	S100A8
SH3BGRL3.1	2.70287618908746e-22	-0.315132663	0.6	0.69	5.41818560864472e-18	EpC3	SH3BGRL3
TMPRSS11A.1	6.69574157200578e-22	-0.706652521	0.126	0.251	1.34222835552428e-17	EpC3	TMPRSS11A
SUCLG1.2	6.76886596756926e-22	0.297598187736067	0.82	0.724	1.35688687185893e-17	EpC3	SUCLG1
PRSS22.2	7.30278365160043e-22	-0.909443701	0.038	0.142	1.46391601079982e-17	EpC3	PRSS22
ATP6V1G1	7.59583981376175e-22	-0.280983667	0.709	0.77	1.52266204906668e-17	EpC3	ATP6V1G1
JUN.2	1.21121074748942e-21	-0.335864243	0.821	0.9	2.4279930644173e-17	EpC3	JUN
DUSP1.1	1.36837140366216e-21	-0.377088409	0.514	0.631	2.74303731578117e-17	EpC3	DUSP1
COX8A.2	1.42620589835491e-21	0.25673246251529	0.894	0.814	2.85897234384225e-17	EpC3	COX8A
ECM1.2	1.47799428401085e-21	-0.519233318	0.017	0.109	2.96278734172815e-17	EpC3	ECM1
NDUFA4.2	1.49023575589422e-21	0.250490090801886	0.955	0.969	2.98732659626556e-17	EpC3	NDUFA4
SPINK5.2	1.72725996352981e-21	-0.886088506	0.133	0.256	3.46246532289186e-17	EpC3	SPINK5
PLK2.1	2.32334656692079e-21	-0.343009595	0.302	0.436	4.65738052804941e-17	EpC3	PLK2
JUNB.2	3.89837523965103e-21	-0.368176854	0.67	0.756	7.81468300540446e-17	EpC3	JUNB
PLAC8.2	4.97734927613463e-21	-0.767748952	0.292	0.399	9.97759435893948e-17	EpC3	PLAC8
EHF.1	5.14650476409268e-21	-0.569398124	0.258	0.382	1.03166834501002e-16	EpC3	EHF
ATP6V0E1.1	6.72028879765002e-21	-0.286548662	0.689	0.76	1.34714909237692e-16	EpC3	ATP6V0E1

RPL37.1	8.63355016290861e-21	-0.26802369	0.998	0.995	1.73068146565666e-16	EpC3	RPL37
CYSRT1.1	1.1714949945076e-20	-0.291120255	0.017	0.106	2.34837886598994e-16	EpC3	CYSRT1
LCN2.2	2.45474239908833e-20	-1.525143987	0.072	0.179	4.92077661321247e-16	EpC3	LCN2
KLK13.1	3.07353326273565e-20	-0.905689814	0.058	0.162	6.16120477847988e-16	EpC3	KLK13
UGP2.1	6.20555088838548e-20	0.267856051414884	0.65	0.506	1.24396473108575e-15	EpC3	UGP2
PSMA3.2	6.3215315686682e-20	0.258176978055659	0.57	0.431	1.26721421825523e-15	EpC3	PSMA3
TIPARP.1	8.71616343627217e-20	-0.278919137	0.127	0.25	1.74724212243512e-15	EpC3	TIPARP
RBX1	3.33854506049863e-19	0.253212984081803	0.724	0.589	6.69244742827556e-15	EpC3	RBX1
GJB6.2	3.48110365757682e-19	-0.272100101	0.121	0.242	6.97822039197849e-15	EpC3	GJB6
GADD45B.1	4.10187623709318e-19	-0.454105494	0.506	0.603	8.22262110487699e-15	EpC3	GADD45B
S100A9.2	4.66201908465232e-19	-2.592899234	0.121	0.229	9.34548345709403e-15	EpC3	S100A9
SPRR3.2	8.30229813801473e-19	-3.397846003	0.053	0.149	1.66427868474643e-14	EpC3	SPRR3
C19orf33.1	1.50915110625742e-18	-0.275424333	0.286	0.41	3.02524430760362e-14	EpC3	C19orf33
SAT1.1	2.45995801301847e-18	-0.533095278	0.963	0.972	4.93123183289683e-14	EpC3	SAT1
SBSN.2	3.85115467793591e-18	-0.984739845	0.062	0.157	7.72002466739033e-14	EpC3	SBSN
VSNL1.2	4.76174714183511e-18	0.317197883267208	0.823	0.745	9.54539832052266e-14	EpC3	VSNL1
FDCSP.2	6.39047481355366e-18	-4.423115105	0.121	0.229	1.28103458112497e-13	EpC3	FDCSP
SOX4.2	7.1039043596161e-18	-0.360710227	0.703	0.776	1.42404866792864e-13	EpC3	SOX4
PPP1R15A.1	8.52250746138114e-18	-0.334309769	0.537	0.641	1.70842184570846e-13	EpC3	PPP1R15A
FMO2.2	1.24472824626821e-17	-0.552438191	0.071	0.169	2.49518224246925e-13	EpC3	FMO2
SPRR1A.2	1.66835191735094e-17	-1.450263385	0.046	0.136	3.3443782535217e-13	EpC3	SPRR1A
SPRR2D.2	1.90889756479406e-17	-0.961497493	0.039	0.127	3.82657605838618e-13	EpC3	SPRR2D
PRDX1.2	2.28421535770022e-17	0.307212926319471	0.797	0.709	4.57893810604585e-13	EpC3	PRDX1
AQP3.1	2.59752057160652e-17	-0.556998775	0.089	0.188	5.20698973784242e-13	EpC3	AQP3
GPCPD1.1	2.64785852124931e-17	-0.418537666	0.109	0.213	5.30789719169636e-13	EpC3	GPCPD1
CERS3.2	6.83003148569378e-17	-0.252028608	0.181	0.298	1.36914811162217e-12	EpC3	CERS3
TMPRSS11D.2	9.01122718342568e-17	-0.476850998	0.398	0.482	1.80639060118951e-12	EpC3	TMPRSS11D
MPZL2.2	9.35470229430604e-17	-0.361877863	0.478	0.551	1.87524362191659e-12	EpC3	MPZL2
KLF4.1	1.24872424163645e-16	-0.312136726	0.365	0.472	2.50319261478443e-12	EpC3	KLF4

APOBEC3A.2	1.58377051885279e-16	-0.485253728	0.028	0.108	3.1748263820923e-12	EpC3	APOBEC3A
TMEM45A.1	2.73909525874608e-16	-0.270014089	0.036	0.119	5.4907903556824e-12	EpC3	TMEM45A
KRT19.2	3.75093643444548e-16	-0.429165072	0.644	0.714	7.51912717648942e-12	EpC3	KRT19
SGK1.2	4.4681222720491e-16	-0.388604272	0.616	0.672	8.95679790654962e-12	EpC3	SGK1
GPX3.2	4.82090017731502e-16	-0.282612982	0.025	0.101	9.6639764954457e-12	EpC3	GPX3
TSPO.2	7.83624643008682e-16	-0.278329905	0.789	0.833	1.5708539593752e-11	EpC3	TSPO
CFH.2	1.67171803839486e-15	-0.340486618	0.045	0.127	3.35112597976635e-11	EpC3	CFH
S100A6.1	2.34641651520935e-15	-0.387055139	0.994	0.996	4.70362654638866e-11	EpC3	S100A6
TNFAIP3.1	2.38388037462199e-15	-0.368983285	0.254	0.368	4.77872659896724e-11	EpC3	TNFAIP3
GSTO1.1	6.83732048878047e-15	-0.289597711	0.804	0.813	1.37060926518093e-10	EpC3	GSTO1
BHLHE40.1	6.83854632793494e-15	-0.321652536	0.229	0.335	1.37085499689784e-10	EpC3	BHLHE40
KLF6.1	7.6680192911229e-15	-0.305655074	0.541	0.627	1.5371311470985e-10	EpC3	KLF6
PHLDA1.2	1.85485906378023e-14	-0.313603429	0.109	0.202	3.71825047925384e-10	EpC3	PHLDA1
MME.1	2.11483618908328e-14	-0.295826414	0.059	0.141	4.23940062463634e-10	EpC3	MME
ERRFI1	2.54306057024598e-14	-0.272921266	0.178	0.281	5.09781921911509e-10	EpC3	ERRFI1
PNOC.2	3.05660722431941e-14	-0.338471523	0.113	0.208	6.12727484187068e-10	EpC3	PNOC
S100A7.2	3.52398002267753e-14	-3.350098788	0.093	0.18	7.06417035345938e-10	EpC3	S100A7
MXD1.2	4.78858883330801e-14	-0.290460777	0.091	0.179	9.59920517524923e-10	EpC3	MXD1
C9orf16.2	4.90539099209957e-14	-0.267797269	0.412	0.507	9.83334678276279e-10	EpC3	C9orf16
TNIP3.1	4.97089551152774e-14	-0.260068393	0.035	0.108	9.96465714240851e-10	EpC3	TNIP3
CD55.1	6.91415235271798e-14	-0.267102674	0.174	0.278	1.38601098062585e-09	EpC3	CD55
KRT6B.2	7.81258081666041e-14	-1.057556137	0.317	0.401	1.56610995050775e-09	EpC3	KRT6B
BNIP3L	7.89590457967179e-14	-0.25606331	0.274	0.377	1.58281303204101e-09	EpC3	BNIP3L
CXCL1.2	8.43223955943867e-14	-1.842558756	0.102	0.186	1.69032674208508e-09	EpC3	CXCL1
DSC2.2	8.91010098963992e-14	-0.541555932	0.691	0.72	1.78611884438322e-09	EpC3	DSC2
FTH1.2	1.12663474557425e-13	0.386474494386098	0.732	0.651	2.25845201097814e-09	EpC3	FTH1
MAP1LC3B	1.19327577525951e-13	-0.284265122	0.433	0.521	2.39204061908522e-09	EpC3	MAP1LC3B
BTG1	2.14222930200997e-13	-0.26742683	0.515	0.59	4.29431285880918e-09	EpC3	BTG1
KRT17.2	2.18291233910651e-13	-0.345465343	0.992	0.976	4.37586607497291e-09	EpC3	KRT17

KRT15.2	2.21360789850285e-13	-0.380700662	0.114	0.202	4.43739839333882e-09	EpC3	KRT15
PRDM1.1	3.61722539994813e-13	-0.297357816	0.101	0.187	7.25109003673602e-09	EpC3	PRDM1
F3.2	7.09576188604455e-13	-0.25024749	0.118	0.207	1.42241642767649e-08	EpC3	F3
RAB11A.2	8.68595212149376e-13	-0.27433054	0.553	0.61	1.74118596227464e-08	EpC3	RAB11A
CDKN1A.2	1.16339573694164e-12	-0.25561417	0.343	0.442	2.33214309427321e-08	EpC3	CDKN1A
KLK11.2	1.50327565292731e-12	-0.362211877	0.355	0.434	3.01346637385808e-08	EpC3	KLK11
SAA1.2	1.54699429984447e-12	-0.623515574	0.041	0.11	3.10110477346823e-08	EpC3	SAA1
LY6D.1	2.34222951103855e-12	-0.95370659	0.483	0.549	4.69523327782787e-08	EpC3	LY6D
CAPNS2.1	3.39095158052364e-12	-0.338841108	0.45	0.511	6.79750153831769e-08	EpC3	CAPNS2
GRN.1	3.59102625610561e-12	-0.371225767	0.277	0.362	7.1985712329893e-08	EpC3	GRN
FABP5.1	6.42042443436005e-12	-0.291676719	0.662	0.512	1.28703828211182e-07	EpC3	FABP5
MIR205HG	7.03665584192253e-12	-0.272549264	0.497	0.563	1.41056803007179e-07	EpC3	MIR205HG
CTSD.1	7.45715118824634e-12	-0.293271335	0.266	0.354	1.49486052719586e-07	EpC3	CTSD
RND3.1	1.19700694516634e-11	-0.275811314	0.384	0.468	2.39952012228045e-07	EpC3	RND3
TXNIP.2	1.41275506316349e-11	-0.379310263	0.421	0.491	2.83200879961752e-07	EpC3	TXNIP
VAMP8.1	1.90480572627591e-11	-0.278882052	0.442	0.509	3.81837355889269e-07	EpC3	VAMP8
C4orf3.2	2.02909879024273e-11	-0.252552948	0.603	0.645	4.06753143492057e-07	EpC3	C4orf3
VIM	2.3331981212667e-11	0.272478563621338	0.176	0.107	4.67712895389122e-07	EpC3	VIM
TSC22D1	2.58438976807548e-11	-0.286638386	0.554	0.623	5.1806677290841e-07	EpC3	TSC22D1
RAB10.2	3.57648222643947e-11	-0.291848845	0.485	0.538	7.16941627112057e-07	EpC3	RAB10
ABLIM1.2	3.83481664246743e-11	-0.251167304	0.292	0.377	7.68727344149021e-07	EpC3	ABLIM1
CXCL8.2	4.03992867796801e-11	-1.402397881	0.163	0.24	8.09844102785467e-07	EpC3	CXCL8
CALML5.2	4.3170475487073e-11	-0.303408192	0.05	0.114	8.65395351613865e-07	EpC3	CALML5
ID2.1	6.6246485729497e-11	-0.41504005	0.462	0.522	1.3279770529335e-06	EpC3	ID2
CLCA2.2	8.07518137614027e-11	-0.450828671	0.572	0.573	1.61875085866108e-06	EpC3	CLCA2
BTG2	9.21514888352308e-11	-0.373524277	0.499	0.553	1.84726874519104e-06	EpC3	BTG2
RDH10.2	1.09134750182562e-10	-0.256784188	0.054	0.118	2.18771520215964e-06	EpC3	RDH10
DMKN.2	1.66062227908997e-10	-0.650199374	0.384	0.445	3.32888342066375e-06	EpC3	DMKN
EMP1.2	1.69000811937118e-10	-0.394197329	0.684	0.713	3.38779027609146e-06	EpC3	EMP1

CCND2	2.06015082895063e-10	-0.291037808	0.263	0.345	4.12977835171444e-06	EpC3	CCND2
SOD2.2	2.36286199052135e-10	-0.459211686	0.25	0.325	4.7365931461991e-06	EpC3	SOD2
S100A11.2	4.67550858768459e-10	-0.346505302	0.996	0.998	9.37252451487254e-06	EpC3	S100A11
CLEC2B.2	7.54216906760132e-10	-0.997752348	0.076	0.139	1.51190321129136e-05	EpC3	CLEC2B
HOPX.2	1.33651669301831e-09	-0.590757667	0.343	0.413	2.67918136282451e-05	EpC3	HOPX
CLDN4.2	2.20168380755085e-09	-0.424985153	0.458	0.508	4.41349536061643e-05	EpC3	CLDN4
LY6G6C.2	5.69658969345972e-09	-0.320655003	0.102	0.17	0.000114193836995094	EpC3	LY6G6C
SERPINB5.2	8.39895965827776e-09	-0.296073426	0.769	0.767	0.000168365545309836	EpC3	SERPINB5
TMEM154.2	2.26681017547407e-08	-0.2575921	0.239	0.31	0.000454404767775532	EpC3	TMEM154
GLTP.2	3.52732925676806e-08	-0.43782938	0.415	0.456	0.000707088422811726	EpC3	GLTP
DAPL1.2	1.32613094293967e-07	-0.299726927	0.23	0.297	0.00265836208821687	EpC3	DAPL1
CXCL3.2	1.52844011145311e-07	-0.413854973	0.052	0.102	0.00306391104741891	EpC3	CXCL3
CD46.1	2.63749817685419e-07	-0.270575083	0.511	0.543	0.00528712884532192	EpC3	CD46
LGALS7B.2	3.67034758273523e-07	-0.532008411	0.254	0.321	0.00735757876435104	EpC3	LGALS7B
KRTDAP.2	4.41073333915273e-07	-1.517569643	0.16	0.218	0.00884175605166557	EpC3	KRTDAP
CTGF.1	7.06557850444998e-07	-0.280548317	0.126	0.184	0.0141636586700204	EpC3	CTGF
LAMB3.1	1.23807583495895e-06	-0.302580636	0.333	0.378	0.0248184681875871	EpC3	LAMB3
CALML3.2	1.58033132011283e-06	-0.421430469	0.483	0.493	0.0316793216429817	EpC3	CALML3
S100A16.2	8.30189665658072e-06	-0.25070958	0.725	0.71	0.166419820377817	EpC3	S100A16
PLAUR.2	1.44278005138108e-05	-0.370286368	0.156	0.203	0.289219689099852	EpC3	PLAUR
PLAT.1	1.57639885174325e-05	-0.525584677	0.132	0.178	0.316004913820452	EpC3	PLAT
GPX2.2	1.7611357195339e-05	-0.377502971	0.738	0.679	0.353037266337765	EpC3	GPX2
DCN.2	3.70003173224227e-05	-0.609496575	0.173	0.218	0.741708361045285	EpC3	DCN
DYNLT3.2	0.000295748105486489	-0.338554951	0.598	0.578	1	EpC3	DYNLT3
S100A14.2	0.000469585942792899	-0.303799228	0.732	0.704	1	EpC3	S100A14
GBP6.2	0.000579741548400496	-0.515697679	0.461	0.453	1	EpC3	GBP6
POLR2J3.2	0.000738996679444015	-0.351240689	0.374	0.393	1	EpC3	POLR2J3
CIB1.1	0.00120688091826921	-0.308246855	0.296	0.323	1	EpC3	CIB1
KLK10.2	0.00200713893145762	-0.408582254	0.28	0.305	1	EpC3	KLK10

GSTA1.2	0.00580169001391973	-0.944380971	0.361	0.359	1	EpC3	GSTA1
S100A7.3	0	4.44306747011806	0.603	0.109	0	EpC2	S100A7
S100A8.3	0	3.95743253286182	0.669	0.186	0	EpC2	S100A8
S100A9.3	0	3.84499372245055	0.673	0.153	0	EpC2	S100A9
SPRR2A.3	0	3.79320386984228	0.623	0.077	0	EpC2	SPRR2A
SERPINB4.3	0	3.50128532498908	0.654	0.151	0	EpC2	SERPINB4
SPRR2F.1	0	3.39698810436476	0.483	0.023	0	EpC2	SPRR2F
TMPRSS11E.3	0	3.00362651811413	0.593	0.063	0	EpC2	TMPRSS11E
RHCG.2	0	2.99725736258068	0.573	0.093	0	EpC2	RHCG
CXCL1.3	0	2.93865364412502	0.606	0.116	0	EpC2	CXCL1
SPRR2E.1	0	2.9117065392058	0.457	0.032	0	EpC2	SPRR2E
CLCA4.3	0	2.78917479037946	0.559	0.041	0	EpC2	CLCA4
SPRR2D.3	0	2.78083001032377	0.566	0.054	0	EpC2	SPRR2D
PRSS22.3	0	2.71251952349286	0.535	0.073	0	EpC2	PRSS22
IL36G.3	0	2.67583366622039	0.577	0.042	0	EpC2	IL36G
PDZK1IP1.3	0	2.13788905038307	0.638	0.078	0	EpC2	PDZK1IP1
CLEC2B.3	0	1.98417242184141	0.52	0.077	0	EpC2	CLEC2B
CRNN	0	1.91595048429524	0.293	0.005	0	EpC2	CRNN
APOBEC3A.3	0	1.89923878903473	0.479	0.046	0	EpC2	APOBEC3A
ECM1.3	0	1.83200770195643	0.496	0.042	0	EpC2	ECM1
AQP3.2	0	1.82285130468371	0.612	0.116	0	EpC2	AQP3
FMO2.3	0	1.80953638335203	0.615	0.094	0	EpC2	FMO2
S100A12.1	0	1.76951708047903	0.409	0.027	0	EpC2	S100A12
PLAUR.3	0	1.69434939536559	0.661	0.134	0	EpC2	PLAUR
SDR16C5.2	0	1.48108957771516	0.605	0.099	0	EpC2	SDR16C5
IL1A.1	0	1.45806343668885	0.443	0.034	0	EpC2	IL1A
CEACAM1.1	0	1.4236743457205	0.415	0.04	0	EpC2	CEACAM1
CLDN1.2	0	1.3004247283471	0.636	0.12	0	EpC2	CLDN1
MXD1.3	0	1.25524199019153	0.608	0.108	0	EpC2	MXD1

HS3ST1.2	0	1.22142474697557	0.482	0.036	0	EpC2	HS3ST1
CXCL6.1	0	1.22016577632587	0.338	0.024	0	EpC2	CXCL6
RDH10.3	0	1.19689798115192	0.501	0.057	0	EpC2	RDH10
CYSRT1.2	0	1.13861401697546	0.443	0.047	0	EpC2	CYSRT1
HEPHL1.1	0	1.10078575416663	0.339	0.018	0	EpC2	HEPHL1
GPRC5A.1	0	1.05834994114895	0.437	0.037	0	EpC2	GPRC5A
TNIP3.2	0	1.00758527254975	0.45	0.05	0	EpC2	TNIP3
FAM83A	0	0.77005997116863	0.337	0.011	0	EpC2	FAM83A
SPRR2C	0	0.689854207256474	0.293	0.006	0	EpC2	SPRR2C
LRG1	0	0.644937556140381	0.345	0.023	0	EpC2	LRG1
PRDM1.2	1.02563471156826e-307	1.17740283139133	0.589	0.12	2.05598734280974e-303	EpC2	PRDM1
FDCSP.3	1.03682364928121e-306	2.92349106083375	0.646	0.156	2.07841668734911e-302	EpC2	FDCSP
VMO1	5.54399685918473e-305	1.23139692583342	0.284	0.014	1.11134961039217e-300	EpC2	VMO1
TMPRSS11F.2	1.08957386255352e-302	1.11872300516673	0.595	0.123	2.18415976487478e-298	EpC2	TMPRSS11F
SAA1.3	4.29231147278797e-297	1.49182529552506	0.441	0.054	8.60436757835076e-293	EpC2	SAA1
GJB2.2	1.06206856037999e-296	1.80231382599649	0.767	0.274	2.12902263613772e-292	EpC2	GJB2
TMPRSS11A.2	6.03915819189062e-295	1.73475365859372	0.666	0.176	1.21060965114639e-290	EpC2	TMPRSS11A
PLAT.2	5.17396999265446e-293	2.01156787678848	0.564	0.119	1.03717402472751e-288	EpC2	PLAT
SLPI.3	1.0987478143479e-292	2.86721318817335	0.72	0.233	2.2025498686418e-288	EpC2	SLPI
ELF3.1	6.21920724880577e-290	1.15569493592624	0.753	0.236	1.2467022850956e-285	EpC2	ELF3
IL36A	1.49536954327122e-289	0.789658725464071	0.211	0.003	2.9976177864415e-285	EpC2	IL36A
VNN1	4.64922437049847e-289	0.559902501695766	0.299	0.019	9.31983517310124e-285	EpC2	VNN1
SLC5A1	9.880389499914e-289	0.654832825948859	0.331	0.026	1.98062287915276e-284	EpC2	SLC5A1
LCN2.3	2.12814653754759e-287	3.1588201291265	0.544	0.114	4.26608254916789e-283	EpC2	LCN2
C15orf48	2.61297575120857e-286	1.92675118951402	0.292	0.018	5.23797119087271e-282	EpC2	C15orf48
SPRR3.3	1.60529162183944e-283	3.23615201476805	0.499	0.087	3.21796758513934e-279	EpC2	SPRR3
CFH.3	1.29270499402732e-281	0.93838651629759	0.466	0.068	2.59135643102716e-277	EpC2	CFH
PRSS3.2	1.91405669835363e-281	0.989596626851552	0.43	0.057	3.83691805751969e-277	EpC2	PRSS3
CXCL17.2	1.41148701792549e-279	1.15666432903386	0.546	0.11	2.82946687613343e-275	EpC2	CXCL17

NCCRP1.1	2.86328800699341e-278	0.848524479291337	0.352	0.034	5.73974713881899e-274	EpC2	NCCRP1
MBOAT2.1	1.44507497894866e-273	0.893258352520466	0.491	0.084	2.89679730280049e-269	EpC2	MBOAT2
CXCL8.3	5.77055452476978e-273	2.88295580824608	0.637	0.175	1.15676536003535e-268	EpC2	CXCL8
GPR157	7.82811270816171e-270	0.619827131680573	0.346	0.035	1.5692234734781e-265	EpC2	GPR157
TACSTD2.3	1.09199805112048e-268	2.03303739016992	0.954	0.613	2.18901929327611e-264	EpC2	TACSTD2
SOD2.3	7.63311521457055e-268	1.43879732133924	0.746	0.256	1.53013427591281e-263	EpC2	SOD2
SERPINB1.2	8.29559397268514e-267	2.34950729386338	0.723	0.277	1.66293476776446e-262	EpC2	SERPINB1
ANXA1.3	2.75172154250428e-264	2.56848165483929	0.997	0.983	5.51610100410407e-260	EpC2	ANXA1
LY6D.2	3.24564257077789e-264	2.39510403074061	0.898	0.492	6.50621509738137e-260	EpC2	LY6D
CST6	2.63131433334293e-263	0.551948241488107	0.257	0.014	5.27473271261924e-259	EpC2	CST6
RAB31.2	7.68116596168413e-263	0.91755573899962	0.468	0.08	1.5397665286792e-258	EpC2	RAB31
TMPRSS2	2.44079232995893e-262	0.502730122607009	0.296	0.022	4.89281230463566e-258	EpC2	TMPRSS2
KYNU.1	7.52062618174832e-261	0.729062170818093	0.316	0.027	1.50758472439327e-256	EpC2	KYNU
VAMP5.1	9.09262165108314e-259	0.889683649832789	0.411	0.059	1.82270693617613e-254	EpC2	VAMP5
PHLDA1.3	2.02869993324441e-258	1.10168353378217	0.58	0.137	4.06673188618174e-254	EpC2	PHLDA1
PRSS27	1.70185499885098e-256	0.873277476019277	0.267	0.017	3.41153853069668e-252	EpC2	PRSS27
WFDC21P.1	1.72308251683737e-255	1.22083997767876	0.353	0.04	3.4540912132522e-251	EpC2	WFDC21P
NCOA7.2	4.41160816886072e-255	1.42420245016479	0.688	0.205	8.84350973529819e-251	EpC2	NCOA7
KRT13.1	1.10426823623036e-254	1.94464009663097	0.579	0.148	2.21361610634738e-250	EpC2	KRT13
EHF.2	1.3408093921844e-254	1.52824855883731	0.774	0.31	2.68778650757285e-250	EpC2	EHF
IL1RN.3	6.73064191082935e-254	2.92778582261942	0.736	0.316	1.34922447744485e-249	EpC2	IL1RN
ODAPH	6.24927074254342e-248	2.45332355153876	0.262	0.017	1.25272881305025e-243	EpC2	ODAPH
CD14.1	8.94558163903205e-248	1.42115367427793	0.31	0.03	1.79323129536037e-243	EpC2	CD14
STEAP4	2.86197514786269e-244	0.375365511808216	0.202	0.006	5.73711538140555e-240	EpC2	STEAP4
DHRS9	3.12986936611932e-243	0.761136875796755	0.22	0.009	6.27413613132279e-239	EpC2	DHRS9
SLC6A14	1.87743464014839e-242	0.438334270444402	0.177	0.002	3.76350547964146e-238	EpC2	SLC6A14
CIB1.2	6.6286569460164e-239	1.4559010896429	0.704	0.268	1.32878057139845e-234	EpC2	CIB1
IL1B.1	3.57270329983666e-235	1.64440368539629	0.352	0.046	7.16184103485258e-231	EpC2	IL1B
FUT3	9.62362858476946e-229	0.327815208584341	0.203	0.008	1.92915258610289e-224	EpC2	FUT3

DUOX2	2.3194272309512e-226	0.396381135264102	0.189	0.006	4.64952382716478e-222	EpC2	DUOX2
TMEM45B.3	2.01212073218948e-225	0.736606605823967	0.453	0.085	4.03349721974702e-221	EpC2	TMEM45B
MME.2	1.11932310364955e-222	1.01048431116206	0.445	0.087	2.24379509357589e-218	EpC2	MME
LYN	1.52060759078985e-222	0.468450180310658	0.294	0.03	3.04820997649733e-218	EpC2	LYN
CD24.3	7.97532637701135e-222	1.48702902134296	0.921	0.598	1.5987339255357e-217	EpC2	CD24
CLDN4.3	1.56480739348769e-221	1.16932050976215	0.909	0.446	3.13681290098542e-217	EpC2	CLDN4
CSF3.1	5.71885076151434e-221	1.08277838908966	0.309	0.034	1.14640082365316e-216	EpC2	CSF3
SAA2	9.82633854258256e-219	0.758623945693105	0.268	0.023	1.9697878242461e-214	EpC2	SAA2
TMEM45A.2	1.07998840325377e-218	0.803594228211436	0.405	0.068	2.1649447531625e-214	EpC2	TMEM45A
ADGRF1	3.03737192847974e-216	0.384585580699753	0.175	0.005	6.08871576783049e-212	EpC2	ADGRF1
DOC2B	1.38800607530417e-214	0.439293415026342	0.267	0.024	2.78239697855474e-210	EpC2	DOC2B
CRCT1	1.96188197501856e-213	0.899079587296522	0.174	0.005	3.93278860712221e-209	EpC2	CRCT1
TNFSF10.3	2.73459517582516e-212	1.61484963997679	0.816	0.416	5.48176948945911e-208	EpC2	TNFSF10
CTSL	5.81687876436088e-211	0.796949391708228	0.372	0.06	1.16605151710378e-206	EpC2	CTSL
GPCPD1.2	1.17460928181957e-208	1.42630161212477	0.539	0.153	2.3546217663355e-204	EpC2	GPCPD1
SLC26A9	1.24805751975018e-207	0.267960523041431	0.156	0.003	2.50185610409121e-203	EpC2	SLC26A9
SLC52A3	1.27673498577414e-205	0.303594521907217	0.197	0.01	2.55934295248285e-201	EpC2	SLC52A3
CSTB.3	2.95775080822457e-201	1.88996924313145	0.985	0.935	5.92910727016697e-197	EpC2	CSTB
BHLHE40.2	3.38679586225603e-201	0.961516077640739	0.712	0.268	6.78917098547843e-197	EpC2	BHLHE40
CLIC3.3	8.99972592619848e-199	0.82297390706591	0.372	0.062	1.80408505916575e-194	EpC2	CLIC3
LYPD3.3	3.68097939894211e-198	1.23202430785633	0.914	0.52	7.37889130311936e-194	EpC2	LYPD3
LGALS3.3	5.51129664673211e-197	1.32747167686166	0.905	0.593	1.10479452580392e-192	EpC2	LGALS3
SERPINB3.3	3.54619962401263e-196	2.66707089367222	0.747	0.395	7.10871176629572e-192	EpC2	SERPINB3
PLS1.1	4.8313105184884e-195	0.805804478807628	0.48	0.118	9.68484506536186e-191	EpC2	PLS1
KLK12.1	1.00247834010349e-189	0.88077295626262	0.311	0.044	2.00956808057146e-185	EpC2	KLK12
NFKBIZ.2	1.14089320285345e-188	1.06102363383471	0.783	0.328	2.28703451444004e-184	EpC2	NFKBIZ
SULT1B1	1.84915696260376e-188	0.262606982935556	0.17	0.007	3.7068200472355e-184	EpC2	SULT1B1
KRT16.3	1.1538281335309e-187	1.3085805555597	0.765	0.324	2.31296387647605e-183	EpC2	KRT16
ADGRE2	3.75340986546818e-186	0.302766994734791	0.185	0.01	7.5240854163175e-182	EpC2	ADGRE2

MTATP6P1.1	1.05770004368147e-185	-1.127384609	0.877	0.993	2.12026550756388e-181	EpC2	MTATP6P1
TIPARP.2	2.51186422075284e-185	0.720827803760082	0.597	0.184	5.03528301692115e-181	EpC2	TIPARP
PGLYRP4	3.02495897844062e-183	0.289526990740784	0.198	0.013	6.06383276818207e-179	EpC2	PGLYRP4
MUC1	3.97775255055164e-183	0.294242822406418	0.171	0.008	7.97380276283581e-179	EpC2	MUC1
HBEGF.3	4.22810642494749e-183	1.03657510265368	0.707	0.273	8.47566213944974e-179	EpC2	HBEGF
ERO1A.1	5.83613576898073e-182	0.734536382201189	0.386	0.078	1.16991177624988e-177	EpC2	ERO1A
KLK13.2	4.85541418447519e-181	2.3506116028396	0.439	0.108	9.73316327419896e-177	EpC2	KLK13
CLEC7A	1.59705921024232e-180	0.380676680703364	0.277	0.034	3.20146489285175e-176	EpC2	CLEC7A
TYMP.1	4.80294058572309e-179	0.712967953678088	0.478	0.122	9.62797469814051e-175	EpC2	TYMP
RPL23A.1	4.816624076864e-179	-1.072859973	0.715	0.968	9.65540462448158e-175	EpC2	RPL23A
PLBD1	4.88951137252452e-179	0.491764231530433	0.281	0.037	9.80151449736266e-175	EpC2	PLBD1
HMOX1	5.17648724043198e-179	0.418915168011413	0.217	0.018	1.037678632217e-174	EpC2	HMOX1
CD177	2.58959099787075e-178	0.605214407411332	0.135	0.002	5.19109411433171e-174	EpC2	CD177
PAPPA	4.65413164414447e-178	0.387135506165164	0.248	0.026	9.329672293852e-174	EpC2	PAPPA
QPCT	9.71902616364273e-174	0.355752944651855	0.235	0.024	1.94827598476382e-169	EpC2	QPCT
GALNT1.1	4.71483473644171e-172	0.870271731202653	0.482	0.135	9.45135771267106e-168	EpC2	GALNT1
MAL	1.14444357465245e-171	2.28300767526969	0.178	0.011	2.2941515897483e-167	EpC2	MAL
CYP24A1	2.62012187191908e-171	0.523360990661509	0.26	0.032	5.25229630444898e-167	EpC2	CYP24A1
CD55.2	3.06093095467394e-171	0.916998869514619	0.607	0.217	6.13594219173938e-167	EpC2	CD55
DLG1.1	3.64680090572509e-170	1.05137246268841	0.528	0.171	7.31037709561652e-166	EpC2	DLG1
GRN.2	1.32343967053849e-169	1.1515785160262	0.677	0.306	2.65296716356145e-165	EpC2	GRN
SAT1.2	8.40759846874839e-168	1.29591852319903	0.987	0.969	1.6853871890453e-163	EpC2	SAT1
ST3GAL1	2.92174541765847e-167	0.393716832999561	0.28	0.039	5.85693086423817e-163	EpC2	ST3GAL1
SLC12A8	7.76927817698071e-163	0.411032017093564	0.199	0.017	1.55742950335755e-158	EpC2	SLC12A8
ADAM28	1.36383616270109e-161	0.414725528230836	0.277	0.039	2.7339459717506e-157	EpC2	ADAM28
SPINT1.1	1.5807690202702e-159	0.692592863770251	0.541	0.18	3.16880957803365e-155	EpC2	SPINT1
S100A6.2	2.19764640028596e-159	1.29348549267261	0.991	0.996	4.40540197401323e-155	EpC2	S100A6
ALOX15B	5.46615819336894e-158	0.303171543724711	0.147	0.007	1.09574607144274e-153	EpC2	ALOX15B
EPHA2	4.23847504808162e-157	0.519909620350056	0.373	0.081	8.49644708138442e-153	EpC2	EPHA2

CD59.1	2.10182615239854e-156	0.912922949659262	0.706	0.336	4.2133207050981e-152	EpC2	CD59
CTSD.2	2.24105464541007e-155	0.966869780342124	0.669	0.298	4.49241814218902e-151	EpC2	CTSD
ALDH1A3	3.4688331360383e-155	0.420475245868804	0.177	0.013	6.95362290450237e-151	EpC2	ALDH1A3
PLEK	5.15871124205056e-155	0.411701115299541	0.232	0.027	1.03411525558145e-150	EpC2	PLEK
SBSN.3	1.15831500032734e-154	1.83840818382756	0.417	0.107	2.32195824965619e-150	EpC2	SBSN
CXCL3.3	1.82660197206032e-153	1.37050926316046	0.328	0.064	3.66160631319212e-149	EpC2	CXCL3
TGFA	7.64794491628149e-152	0.487599496782366	0.31	0.057	1.53310703791779e-147	EpC2	TGFA
SPINK7	1.16075571033328e-151	0.535187177038962	0.125	0.004	2.3268508969341e-147	EpC2	SPINK7
TGM1.1	1.20332157308436e-150	0.671670997023819	0.323	0.062	2.41217842540491e-146	EpC2	TGM1
NPM1.1	2.69117427872932e-150	-0.899972028	0.715	0.978	5.39472795914079e-146	EpC2	NPM1
DNASE1L3	1.68157500255014e-149	2.34040679617878	0.242	0.034	3.370885250112e-145	EpC2	DNASE1L3
PTPN22	3.22398071923196e-148	0.253052515625656	0.143	0.007	6.46279174977238e-144	EpC2	PTPN22
BCL2A1	6.95239011731417e-148	0.401495264166052	0.195	0.019	1.3936761229168e-143	EpC2	BCL2A1
MT-ND4L.1	2.50712887176074e-147	-0.800564716	0.882	0.991	5.02579053633158e-143	EpC2	MT-ND4L
GLUL.3	4.62211279660738e-147	2.13530227130766	0.913	0.774	9.26548731207916e-143	EpC2	GLUL
PFKFB3	5.83605452868125e-147	0.600327053506477	0.395	0.097	1.16989549081944e-142	EpC2	PFKFB3
HSPB1.3	6.78706597840976e-147	0.806424429168727	0.998	0.984	1.36053524603202e-142	EpC2	HSPB1
CXCL14.2	7.71482332323161e-147	-2.112036314	0.21	0.65	1.54651348337501e-142	EpC2	CXCL14
CD47.1	2.05676630891723e-146	0.64601914727776	0.491	0.153	4.12299374285547e-142	EpC2	CD47
ARNTL2	4.29109292679271e-146	0.385684089368556	0.31	0.057	8.60192488104867e-142	EpC2	ARNTL2
PLSCR1.2	5.94004744783964e-146	0.718455299732136	0.525	0.181	1.19074191139393e-141	EpC2	PLSCR1
CCL20.1	7.00428577470426e-146	1.4110835360023	0.304	0.056	1.40407912639722e-141	EpC2	CCL20
SERPINB2.2	1.49994987336376e-145	1.00469054752937	0.513	0.166	3.006799516145e-141	EpC2	SERPINB2
VEGFA.1	1.50921466795969e-145	0.580810454852414	0.39	0.097	3.02537172339199e-141	EpC2	VEGFA
SPRR1B.3	4.73631932371223e-145	2.32904538824314	0.718	0.394	9.49442571631353e-141	EpC2	SPRR1B
EFHD2.1	7.86801094345935e-145	0.550045058431125	0.438	0.123	1.57722147372586e-140	EpC2	EFHD2
ATP1B3.2	9.87824017517229e-145	-0.931095234	0.51	0.884	1.98019202551504e-140	EpC2	ATP1B3
TNFAIP3.2	2.00880111935547e-144	0.942237173595641	0.695	0.306	4.02684272385998e-140	EpC2	TNFAIP3
IL19	2.66113099867083e-144	1.26284998250049	0.114	0.003	5.33450319993554e-140	EpC2	IL19

OASL	5.37869803265858e-144	0.28189795209604	0.167	0.013	1.07821380762674e-139	EpC2	OASL
PSCA	1.06630698113261e-143	0.42941433981087	0.107	0.002	2.13751897437843e-139	EpC2	PSCA
RPS3A.1	3.21247356053695e-143	-0.818257415	0.89	0.992	6.43972449945237e-139	EpC2	RPS3A
MT-CYB.1	4.16562132090537e-143	-0.568597007	1	1	8.35040449988691e-139	EpC2	MT-CYB
CRABP2.2	8.90458870438531e-143	1.24699377517985	0.557	0.213	1.78501385168108e-138	EpC2	CRABP2
NET1.1	4.02428668112725e-142	0.719975147941038	0.605	0.243	8.06708508098769e-138	EpC2	NET1
MT-ND2.1	8.75904384631066e-142	-0.56921277	1	1	1.75583792943144e-137	EpC2	MT-ND2
DPYD	1.77261423823426e-141	0.265833125832924	0.202	0.022	3.55338250196439e-137	EpC2	DPYD
CDKN1A.3	2.88371493159342e-141	0.68693989422689	0.786	0.38	5.78069495187216e-137	EpC2	CDKN1A
B2M.2	3.65261208542429e-141	1.0466179358334	0.995	0.975	7.32202618644154e-137	EpC2	B2M
CXCL2.1	1.910708026872e-140	0.915464563226475	0.343	0.075	3.83020531066761e-136	EpC2	CXCL2
PITX2.1	1.96307895753115e-139	0.654580964904193	0.526	0.18	3.93518807826694e-135	EpC2	PITX2
RPL30.1	8.68519903359513e-139	-1.104056607	0.406	0.794	1.74103499827448e-134	EpC2	RPL30
CTSC.2	1.6371485827428e-138	1.22431013063964	0.661	0.313	3.28182804896622e-134	EpC2	CTSC
RPS23	3.46445291472845e-138	-0.805990416	0.692	0.958	6.94484231286464e-134	EpC2	RPS23
CD46.2	1.27587025173492e-137	0.926284345762837	0.824	0.5	2.55760950662783e-133	EpC2	CD46
ASS1.2	2.48461009542816e-137	0.860651635480266	0.432	0.124	4.98064939729528e-133	EpC2	ASS1
FABP5P7	1.37523267995789e-136	0.3254596190216	0.258	0.042	2.75679143024359e-132	EpC2	FABP5P7
TMPRSS11D.3	2.62669889964004e-136	0.927304255617742	0.783	0.428	5.26548061421841e-132	EpC2	TMPRSS11D
FABP5.2	8.47200029200158e-136	2.25477467389833	0.76	0.502	1.69829717853464e-131	EpC2	FABP5
GPX3.3	5.97214626301642e-135	0.643267056261263	0.308	0.062	1.19717643988427e-130	EpC2	GPX3
IL2RG	1.05780084273515e-134	0.271948243602948	0.152	0.011	2.12046756934687e-130	EpC2	IL2RG
MT-ATP6.2	1.9287536540776e-133	-0.577013271	1	1	3.86637957496396e-129	EpC2	MT-ATP6
PPP1R15A.2	2.40511204216188e-133	0.859744102384448	0.909	0.589	4.82128759971771e-129	EpC2	PPP1R15A
SAMD9.1	5.4192860190841e-133	0.696649729349093	0.367	0.093	1.0863500753856e-128	EpC2	SAMD9
LMTK3	1.47532000843928e-132	0.452321388308318	0.304	0.062	2.95742648891738e-128	EpC2	LMTK3
UGCG.1	2.79025668977737e-132	0.586211460815268	0.385	0.103	5.59334856032772e-128	EpC2	UGCG
LNX1	3.65842567172593e-132	0.511489112952588	0.365	0.092	7.33368010154179e-128	EpC2	LNX1
CEACAM6	5.79460701895539e-132	0.580499541058785	0.139	0.009	1.1615869230198e-127	EpC2	CEACAM6

EEF1A1.1	1.18148527315739e-131	-0.677986568	0.973	0.997	2.36840537857131e-127	EpC2	EEF1A1
CCNL1.1	3.7284688314371e-131	0.749612158342624	0.826	0.47	7.47408861949882e-127	EpC2	CCNL1
BCL3	5.47209175067656e-130	0.401042357753267	0.325	0.072	1.09693551234062e-125	EpC2	BCL3
SLC1A1	7.39727017842882e-130	0.268040336024132	0.183	0.02	1.48285677996784e-125	EpC2	SLC1A1
S100A14.3	2.30378984355608e-129	0.987403895801128	0.901	0.682	4.61817712039252e-125	EpC2	S100A14
CAPN2.1	3.02932241841068e-129	0.680708788923547	0.582	0.238	6.07257971994606e-125	EpC2	CAPN2
IL13RA1.1	4.19116559327477e-129	0.481651267816499	0.384	0.102	8.4016105482786e-125	EpC2	IL13RA1
CLCA2.3	1.30040888151892e-128	0.8577933109059	0.878	0.532	2.60679964389282e-124	EpC2	CLCA2
RPS25.1	2.80329285437411e-128	-0.714978955	0.791	0.962	5.61948085587833e-124	EpC2	RPS25
PTMA.2	5.45252373450578e-128	-0.795023964	0.984	1	1.09301290781903e-123	EpC2	PTMA
DENND2C.1	8.33939994826139e-128	0.667069205383505	0.557	0.215	1.67171611362848e-123	EpC2	DENND2C
NUCKS1.2	1.71521513455431e-127	-0.95071407	0.597	0.862	3.43832025872757e-123	EpC2	NUCKS1
OSBPL3	2.47329636550711e-127	0.464502728021519	0.355	0.089	4.95796989429555e-123	EpC2	OSBPL3
NFKBIA.2	1.78133865715389e-126	0.969319048881303	0.925	0.701	3.57087147213069e-122	EpC2	NFKBIA
HNRNPA1.3	3.37127664022918e-126	-0.753106364	0.665	0.937	6.75806115300341e-122	EpC2	HNRNPA1
TXNDC17.1	2.0594502460348e-125	0.931873412765835	0.851	0.606	4.12837396320136e-121	EpC2	TXNDC17
HSPB8	2.43056566024584e-125	0.359709489405109	0.247	0.042	4.87231192252882e-121	EpC2	HSPB8
FAM20C	1.20831517945417e-124	0.289045593934728	0.212	0.03	2.42218860873382e-120	EpC2	FAM20C
KRT80	7.36902777453418e-124	0.3944128231997	0.198	0.026	1.47719530768312e-119	EpC2	KRT80
ICAM1	9.60086252328821e-124	0.27734819743582	0.164	0.016	1.92458890141835e-119	EpC2	ICAM1
SRD5A3.1	1.19032311740657e-123	0.512653752978504	0.374	0.102	2.38612172115321e-119	EpC2	SRD5A3
RPS4X	2.03871147891929e-122	-0.694791432	0.854	0.986	4.08680103064161e-118	EpC2	RPS4X
S100A11.3	2.96324253935474e-122	0.712367821574864	0.997	0.998	5.94011599439052e-118	EpC2	S100A11
HMGB1.2	1.07426496293203e-120	-0.969772007	0.793	0.944	2.15347154469355e-116	EpC2	HMGB1
ZFAND5.1	1.13012383456819e-120	0.652110400186295	0.629	0.283	2.26544623877539e-116	EpC2	ZFAND5
MPZL2.3	1.14081717017957e-120	0.781967597337311	0.823	0.503	2.28688209934197e-116	EpC2	MPZL2
CHI3L2	2.07104728927032e-120	0.369644245357705	0.166	0.017	4.15162139607128e-116	EpC2	CHI3L2
OSGIN1	6.0927809843159e-120	0.456177822083915	0.347	0.089	1.22135887611596e-115	EpC2	OSGIN1
VAMP8.2	1.20718794304688e-119	0.796900262765784	0.771	0.464	2.41992895063179e-115	EpC2	VAMP8

TMEM41A	3.28681796139081e-119	0.570399045889147	0.405	0.124	6.58875528540402e-115	EpC2	TMEM41A
S100A13.1	3.36982316358895e-119	0.737967080121935	0.583	0.251	6.75514751373041e-115	EpC2	S100A13
ANXA2.2	3.57904181484917e-119	0.848041174049895	0.999	0.992	7.17454722204664e-115	EpC2	ANXA2
RPS3.1	1.22990554447958e-118	-0.661200034	0.942	0.995	2.46546865446377e-114	EpC2	RPS3
CPNE8	2.51183091469648e-118	0.362504530925612	0.298	0.065	5.03521625160057e-114	EpC2	CPNE8
RPS18.1	5.63664576038948e-118	-0.65460183	0.972	0.998	1.12992200912768e-113	EpC2	RPS18
RPL3.2	5.87862771298876e-118	-0.663278149	0.927	0.995	1.17842971134573e-113	EpC2	RPL3
DUSP5	1.23026221752251e-117	0.298935479251365	0.18	0.022	2.46618364124562e-113	EpC2	DUSP5
RPL5.1	3.63710128092245e-117	-0.689122919	0.871	0.989	7.29093322773715e-113	EpC2	RPL5
S100A7A	6.53148391474212e-117	0.508374820789996	0.118	0.007	1.3093012655492e-112	EpC2	S100A7A
SNRPD2.1	7.6101948371229e-117	-0.721699019	0.672	0.92	1.52553965704966e-112	EpC2	SNRPD2
DST.2	2.88344588861899e-116	-1.108192439	0.224	0.619	5.78015562832564e-112	EpC2	DST
S100A10.2	5.46442113928853e-116	0.658260152315676	0.992	0.978	1.09539786158178e-111	EpC2	S100A10
RSAD2	9.38205198522972e-115	0.67169812131514	0.185	0.025	1.88072614095915e-110	EpC2	RSAD2
MT-CO3.2	9.55655639146698e-115	-0.508166531	1	1	1.91570729423347e-110	EpC2	MT-CO3
MT-ND4.1	2.32956094926088e-114	-0.496320934	1	1	4.66983787888836e-110	EpC2	MT-ND4
CDA	2.73250882285953e-114	0.353480826984063	0.232	0.04	5.47758718630422e-110	EpC2	CDA
CLDN7.2	3.85235907085424e-114	0.767586721900243	0.689	0.34	7.72243899343441e-110	EpC2	CLDN7
MAP3K8.1	8.86226197629147e-114	0.461559443679996	0.375	0.108	1.77652903576739e-109	EpC2	MAP3K8
RPS6.1	1.64569686897871e-113	-0.636687175	0.939	0.997	3.29896394355472e-109	EpC2	RPS6
STMN1.2	2.13812874012353e-113	-1.140661851	0.271	0.677	4.28609287245163e-109	EpC2	STMN1
SMIM3	4.46285117938694e-112	0.402572614698698	0.323	0.081	8.94623147419906e-108	EpC2	SMIM3
FUCA1	4.96891384599576e-112	0.409808664470526	0.305	0.073	9.9606846956831e-108	EpC2	FUCA1
RPL21	5.93245402439941e-110	-0.619363453	0.97	0.998	1.18921973373111e-105	EpC2	RPL21
FAM46A.1	3.23243630951583e-109	0.555238458379037	0.346	0.095	6.47974182605543e-105	EpC2	FAM46A
KRT42P.1	7.48361769478682e-109	-0.850817901	0.307	0.745	1.50016600309697e-104	EpC2	KRT42P
RPL18A	8.75959487125097e-109	-0.6310318	0.927	0.989	1.75594838789097e-104	EpC2	RPL18A
DUSP1.2	1.17410375610134e-108	0.751502747594262	0.885	0.579	2.35360838948075e-104	EpC2	DUSP1
WNT5A	1.65401374882719e-108	0.504776363331875	0.383	0.117	3.31563596089898e-104	EpC2	WNT5A

ALDH3A1.2	2.29136897818264e-108	-1.134190382	0.285	0.674	4.59327825366492e-104	EpC2	ALDH3A1
NCL.1	8.23405360420023e-108	-0.71833032	0.6	0.907	1.65059838549798e-103	EpC2	NCL
DHRS3	2.00760731155822e-107	0.517675142803022	0.264	0.057	4.0244496167496e-103	EpC2	DHRS3
C2orf54	3.11782304138305e-107	0.259664686715913	0.167	0.021	6.24998806875645e-103	EpC2	C2orf54
MT-ND1	4.22457361132751e-107	-0.451121099	1	1	8.46858026126714e-103	EpC2	MT-ND1
CMPK1.2	4.80833039314871e-106	0.567042876932331	0.602	0.275	9.6387791061059e-102	EpC2	CMPK1
RPL4.1	8.23263436237955e-106	-0.643720569	0.749	0.96	1.6503138842826e-101	EpC2	RPL4
KIF13B	3.08432457514281e-105	0.380045136683009	0.28	0.065	6.18283704333128e-101	EpC2	KIF13B
RPL31.1	3.63981584653972e-105	-0.550459406	0.996	0.999	7.29637484597351e-101	EpC2	RPL31
RPS5	1.2535611749563e-104	-0.638512093	0.855	0.98	2.51288873131739e-100	EpC2	RPS5
FXYD5.1	2.6105299469074e-104	0.725560088716582	0.419	0.148	5.23306833157058e-100	EpC2	FXYD5
RAB11FIP1	2.98317007063783e-104	0.355843817726114	0.281	0.065	5.9800627236006e-100	EpC2	RAB11FIP1
HAS2	1.38691275331334e-103	0.338336451151584	0.179	0.026	2.78020530529192e-99	EpC2	HAS2
RPL13.1	2.06079372455688e-103	-0.575274643	0.968	0.998	4.13106710024672e-99	EpC2	RPL13
CDC42EP1	2.31240440079106e-103	0.310205295409416	0.248	0.051	4.63544586182576e-99	EpC2	CDC42EP1
AREG	5.92247266860571e-103	0.443410289358241	0.236	0.047	1.1872188711487e-98	EpC2	AREG
RPL34.2	1.34409492775258e-102	-0.582410028	0.977	0.997	2.69437269217281e-98	EpC2	RPL34
RPL6	2.23691389871939e-102	-0.581846377	0.929	0.995	4.4841176013729e-98	EpC2	RPL6
SMPDL3A	4.34229426101493e-102	0.436876585513109	0.259	0.058	8.70456307563053e-98	EpC2	SMPDL3A
CSTA.3	6.16222286346751e-102	1.34357402235833	0.83	0.599	1.2352791952107e-97	EpC2	CSTA
ADIPOR1.1	2.04266000836666e-101	0.548324450279914	0.526	0.227	4.09471625277181e-97	EpC2	ADIPOR1
COL17A1.2	1.09471942852169e-100	-1.086229839	0.13	0.5	2.19447456641457e-96	EpC2	COL17A1
DSE	1.34813098389368e-100	0.2998511854331	0.265	0.059	2.70246337031327e-96	EpC2	DSE
SORT1	2.05293531641512e-100	0.386668078876744	0.271	0.064	4.11531413528574e-96	EpC2	SORT1
GAPDH.1	5.60310405377668e-99	-1.278105534	0.281	0.634	1.12319823862007e-94	EpC2	GAPDH
HINT1	8.13275832281772e-99	-0.663088659	0.707	0.932	1.63029273339204e-94	EpC2	HINT1
RBM47	9.14858514068227e-99	0.475452209581889	0.443	0.161	1.83392537730117e-94	EpC2	RBM47
CTSB.1	1.49186545876646e-98	0.701784113535979	0.882	0.735	2.99059349864325e-94	EpC2	CTSB
RPL29	2.67037209562287e-98	-0.565799741	0.921	0.987	5.35302790288561e-94	EpC2	RPL29

RPL15	2.84950073824044e-98	-0.552524773	0.996	0.999	5.71210917987679e-94	EpC2	RPL15
RPS15.1	3.03052750927913e-98	-0.547537238	0.961	0.996	6.07499544510095e-94	EpC2	RPS15
HNRNPA2B1.1	1.87618935037461e-97	-0.663725617	0.746	0.921	3.76100917176093e-93	EpC2	HNRNPA2B1
BTF3.2	3.20635706516234e-97	-0.587286075	0.936	0.979	6.42746337282442e-93	EpC2	BTF3
LTA4H.1	4.965292202706e-97	0.633966082296056	0.528	0.235	9.95342474954444e-93	EpC2	LTA4H
SULT2B1.3	1.29818554310721e-96	0.707529646591466	0.559	0.25	2.60234273971272e-92	EpC2	SULT2B1
RPL7.1	2.99313777612909e-96	-0.522747646	0.979	0.997	6.00004398602838e-92	EpC2	RPL7
CEACAM5	4.32323818492134e-96	0.578375942854563	0.117	0.01	8.66636326549333e-92	EpC2	CEACAM5
LDHB.3	1.95518997194015e-95	-0.809878128	0.274	0.636	3.91937381775123e-91	EpC2	LDHB
TFRC.2	2.11543787533151e-95	1.04848972039965	0.512	0.229	4.24060676488954e-91	EpC2	TFRC
FXYD3.2	2.26691487514069e-95	0.601839715397785	0.976	0.924	4.54425755870702e-91	EpC2	FXYD3
RPL10A	2.62325659942332e-95	-0.571946071	0.846	0.973	5.25858017920399e-91	EpC2	RPL10A
PPIA.1	3.37549759433727e-95	-0.579020341	0.915	0.983	6.76652247760849e-91	EpC2	PPIA
RPL8	5.87625878073274e-95	-0.545120217	0.939	0.993	1.17795483518569e-90	EpC2	RPL8
ADM.1	1.11196760929993e-94	0.632951261130545	0.512	0.212	2.22905026960263e-90	EpC2	ADM
CALML3.3	1.38541291849248e-94	0.671002913971498	0.793	0.451	2.77719873641003e-90	EpC2	CALML3
RPL9P9.1	3.12403908147409e-94	-0.786816175	0.257	0.621	6.26244874272296e-90	EpC2	RPL9P9
NACA.1	4.75446997734552e-94	-0.565262087	0.906	0.977	9.53081051658682e-90	EpC2	NACA
CNFN.1	1.0801019963699e-93	2.3888016424402	0.42	0.169	2.16517246192311e-89	EpC2	CNFN
RND3.2	2.27697912810571e-93	0.614305655476114	0.745	0.418	4.56443236020071e-89	EpC2	RND3
RACK1.1	2.80717659121878e-93	-0.568243441	0.895	0.983	5.62726619475716e-89	EpC2	RACK1
EMP2.2	4.29739474868238e-93	0.709843369773248	0.931	0.682	8.6145575132087e-89	EpC2	EMP2
ZC3H12A	1.25326956725937e-92	0.414268559091487	0.418	0.15	2.51230417452813e-88	EpC2	ZC3H12A
RAC1.1	1.67937176936907e-92	0.692938967627871	0.792	0.518	3.36646864887723e-88	EpC2	RAC1
RPS7	1.77497672845754e-92	-0.542736779	0.916	0.991	3.55811834986599e-88	EpC2	RPS7
ARL5B.1	2.28799924726034e-92	0.550641428738299	0.446	0.172	4.58652329105808e-88	EpC2	ARL5B
IL1R2.2	2.95086912639792e-92	-1.125233915	0.174	0.527	5.91531225077726e-88	EpC2	IL1R2
RPL14	3.59934129403695e-92	-0.546140202	0.95	0.995	7.21523955802647e-88	EpC2	RPL14
RGS10	4.18615336232127e-92	0.394366135736566	0.299	0.083	8.39156303010921e-88	EpC2	RGS10

MX2	4.28856114712687e-92	0.25677089379699	0.146	0.018	8.59684967553053e-88	EpC2	MX2
RND1	5.25689309452323e-92	0.269954218506075	0.166	0.025	1.05379678972813e-87	EpC2	RND1
ETS2.1	5.81203269085659e-92	0.596697748221406	0.538	0.239	1.16508007320911e-87	EpC2	ETS2
FABP4	1.10231771230385e-91	2.02770477494396	0.137	0.016	2.20970608608429e-87	EpC2	FABP4
PTPN13.1	2.27731638600184e-91	0.574779891264333	0.596	0.29	4.56510842737929e-87	EpC2	PTPN13
CNN3	2.52444802270356e-91	0.423856562067656	0.354	0.114	5.06050850631156e-87	EpC2	CNN3
IL4R	5.26616077481662e-91	0.272639252538012	0.235	0.051	1.05565458891974e-86	EpC2	IL4R
CARHSP1.1	1.08609888730433e-90	0.547778698876685	0.39	0.142	2.17719382949026e-86	EpC2	CARHSP1
RPL11.1	1.12655415887369e-90	-0.566640738	0.94	0.995	2.25829046687821e-86	EpC2	RPL11
SERINC2.1	1.35605501551358e-90	0.674821861547514	0.523	0.243	2.71834788409852e-86	EpC2	SERINC2
TNFRSF21	1.73874453824062e-90	0.490036095413897	0.348	0.11	3.48548730135714e-86	EpC2	TNFRSF21
RPL23.1	2.47852859029061e-90	-0.572230577	0.907	0.992	4.96845841209656e-86	EpC2	RPL23
CITED2	4.30982420477747e-90	0.459846683141524	0.375	0.128	8.63947360089691e-86	EpC2	CITED2
THBD.1	4.90811029468749e-90	0.418800144989923	0.27	0.07	9.83879789673054e-86	EpC2	THBD
GCLM	4.32266034011002e-89	0.513555381056653	0.544	0.246	8.66520491778454e-85	EpC2	GCLM
RPS8	5.32792287681728e-89	-0.545174066	0.935	0.994	1.06803541988679e-84	EpC2	RPS8
EMP1.3	7.37044046234687e-89	1.14005361136173	0.879	0.686	1.47747849508205e-84	EpC2	EMP1
SDC4.2	8.98058137185203e-89	0.60929298194001	0.577	0.281	1.80024734180146e-84	EpC2	SDC4
PLAU	9.43845287134809e-89	0.37433823866224	0.294	0.081	1.89203226259044e-84	EpC2	PLAU
STOM	1.45701651346803e-88	0.63248382640726	0.538	0.255	2.92073530289801e-84	EpC2	STOM
MT-CO2.3	1.52668626477799e-88	-0.533840939	1	1	3.06039528637395e-84	EpC2	MT-CO2
MTPN.1	2.03776569573008e-88	0.63048870876384	0.629	0.345	4.08490511366052e-84	EpC2	MTPN
GAS5.2	3.12885820717637e-88	-0.70269817	0.416	0.741	6.27210916210576e-84	EpC2	GAS5
RPL26.1	4.30870770909411e-88	-0.578849634	0.657	0.917	8.63723547365006e-84	EpC2	RPL26
CERS3.3	7.01300513810061e-88	0.537705163715771	0.539	0.247	1.40582700998365e-83	EpC2	CERS3
NFE2L2.1	1.71369053085775e-87	-0.65368099	0.712	0.928	3.43526403815744e-83	EpC2	NFE2L2
IVL.2	2.60095703162307e-87	0.264800818616374	0.213	0.043	5.21387846559161e-83	EpC2	IVL
RAB10.3	3.83087825943349e-87	0.676180952943351	0.773	0.499	7.67937855886038e-83	EpC2	RAB10
S100P	2.97818210480859e-86	0.863122709520113	0.148	0.021	5.97006384729931e-82	EpC2	S100P

RPL13A.1	4.10220226927872e-86	-0.511328529	0.979	0.998	8.22327466899612e-82	EpC2	RPL13A
SOX2.1	4.2928877085354e-86	-0.695686732	0.26	0.619	8.60552270053006e-82	EpC2	SOX2
YBX1.1	5.11201491821063e-86	-0.658278135	0.465	0.758	1.0247545105045e-81	EpC2	YBX1
RPS27.2	5.74018215157792e-86	-0.496006341	0.995	1	1.15067691410531e-81	EpC2	RPS27
S100A2.2	7.91942735001817e-86	1.33559065035015	0.97	0.978	1.58752840658464e-81	EpC2	S100A2
RUNX1	9.50179571978826e-86	0.346357459582329	0.312	0.092	1.90472996998876e-81	EpC2	RUNX1
ZNF750.2	1.01405421227322e-85	0.456251700164256	0.544	0.244	2.03277307392289e-81	EpC2	ZNF750
FBXO32	1.05720038766868e-85	0.333415981115048	0.251	0.062	2.11926389712063e-81	EpC2	FBXO32
FOSL1	4.66199523198685e-85	0.408315153121267	0.401	0.145	9.34543564204085e-81	EpC2	FOSL1
CD82.2	6.45665594099353e-85	0.473278778714959	0.642	0.329	1.29430124993156e-80	EpC2	CD82
DDIT4L	1.02653779919685e-84	0.264433258201426	0.132	0.016	2.05779767227001e-80	EpC2	DDIT4L
PROM2	1.18248626349976e-84	0.25511197666998	0.211	0.045	2.37041196381162e-80	EpC2	PROM2
N4BP1	1.94735506936353e-84	0.398212822537108	0.413	0.154	3.90366797204613e-80	EpC2	N4BP1
SLC25A5.3	2.73361265390246e-84	-0.687376236	0.651	0.931	5.47979992601288e-80	EpC2	SLC25A5
USP6NL	3.72740997838804e-84	0.418807649722077	0.331	0.107	7.47196604267666e-80	EpC2	USP6NL
TMSB4X.2	1.04345429198108e-83	0.656555287374394	0.997	0.995	2.09170847370528e-79	EpC2	TMSB4X
RPS13	1.68598162724618e-83	-0.518598243	0.97	0.996	3.37971876997769e-79	EpC2	RPS13
TP63.2	3.53866703972984e-83	-0.666935756	0.295	0.616	7.09361194784245e-79	EpC2	TP63
FNDC3B	1.49281942122292e-82	0.401826760555308	0.329	0.107	2.99250581178346e-78	EpC2	FNDC3B
B3GNT5.1	1.52205819572361e-82	0.430733703449133	0.379	0.139	3.05111785914756e-78	EpC2	B3GNT5
CA2.1	2.55540298906019e-82	0.655246189247636	0.305	0.094	5.12256083187006e-78	EpC2	CA2
CYB5R1	2.93886935328252e-82	0.336557236233638	0.312	0.096	5.89125750559013e-78	EpC2	CYB5R1
CTAGE5.1	3.46861364680582e-82	0.43824516918753	0.442	0.178	6.95318291638695e-78	EpC2	CTAGE5
YPEL5.1	3.61432379033139e-82	0.48407627634829	0.651	0.342	7.24527347009831e-78	EpC2	YPEL5
RPS4Y1	4.25387818781663e-82	0.319930296627928	0.356	0.111	8.52732421529723e-78	EpC2	RPS4Y1
HSPH1.1	8.84946572344466e-82	0.59528662065288	0.643	0.344	1.77396389892172e-77	EpC2	HSPH1
PHLDA2.2	1.65537645211832e-81	0.532152865832425	0.592	0.292	3.31836763591638e-77	EpC2	PHLDA2
ANXA11	2.01898409064111e-81	0.598458206085843	0.452	0.198	4.04725550809916e-77	EpC2	ANXA11
TM4SF1.3	2.14669761383087e-81	0.612868837531737	0.932	0.754	4.30327003668537e-77	EpC2	TM4SF1

RPSA	2.4073283716506e-81	-0.576118579	0.624	0.881	4.8257304538108e-77	EpC2	RPSA
MT-ATP8.1	3.56776487485028e-81	-0.548752891	0.776	0.914	7.15194146812487e-77	EpC2	MT-ATP8
RAB27B	4.50243703394312e-81	0.406176024646653	0.357	0.125	9.02558527824238e-77	EpC2	RAB27B
KRT17.3	1.66647930076405e-80	-0.76426762	0.941	0.983	3.34062440631161e-76	EpC2	KRT17
MMP12	3.30879099069388e-80	1.63908773648139	0.155	0.025	6.63280241994495e-76	EpC2	MMP12
GPX2.3	5.35235786779699e-80	-0.764292251	0.344	0.734	1.07293365817859e-75	EpC2	GPX2
RPL36.1	8.87880633185924e-80	-0.486265003	0.964	0.995	1.7798455172845e-75	EpC2	RPL36
DCN.3	9.96017584729129e-80	1.5028123494892	0.419	0.184	1.99661685034801e-75	EpC2	DCN
KTN1	1.74989000424927e-79	-0.660458724	0.654	0.837	3.50782950251808e-75	EpC2	KTN1
CDH3	6.41479611739605e-79	0.388269853686004	0.388	0.145	1.28591002969321e-74	EpC2	CDH3
TMPRSS11BNL.2	7.86666453556104e-79	0.345461066419936	0.301	0.091	1.57695157279857e-74	EpC2	TMPRSS11BNL
RPL22	3.11127438029516e-78	-0.533471138	0.797	0.949	6.23686062273967e-74	EpC2	RPL22
PITX1	3.48624874316132e-78	0.466750928349048	0.448	0.19	6.98853423054117e-74	EpC2	PITX1
FTL.1	4.28044255691298e-78	-0.600623714	0.827	0.969	8.58057514958776e-74	EpC2	FTL
RPL12.1	6.3013280677154e-78	-0.524493752	0.827	0.985	1.26316422445423e-73	EpC2	RPL12
RPS2.1	6.60190707744918e-78	-0.510431122	0.958	0.996	1.32341829274546e-73	EpC2	RPS2
KLF6.2	8.00592608106359e-78	0.641113733183693	0.844	0.585	1.60486794221001e-73	EpC2	KLF6
AFDN	9.06347891940398e-78	0.42525167006031	0.316	0.105	1.81686498418372e-73	EpC2	AFDN
F3.3	1.21099696331454e-77	0.551327894356535	0.413	0.165	2.42756451266034e-73	EpC2	F3
COX4I1.1	1.23170979822433e-77	-0.498177838	0.909	0.983	2.46908546152049e-73	EpC2	COX4I1
SKIL	1.26102215481747e-77	0.478362801486221	0.449	0.19	2.5278450115471e-73	EpC2	SKIL
AKR1C3.3	1.32691257695051e-77	-0.956469991	0.305	0.634	2.65992895175499e-73	EpC2	AKR1C3
LY96	1.38663972546354e-77	0.319773297076756	0.22	0.052	2.77965799366422e-73	EpC2	LY96
GM2A.1	1.74136356434138e-77	0.552894332220377	0.477	0.216	3.49073740107872e-73	EpC2	GM2A
KRT4	7.01716585391582e-77	1.50864212481184	0.159	0.028	1.40666106707597e-72	EpC2	KRT4
RPL19	8.27592836971266e-77	-0.476994347	0.96	0.996	1.6589926009926e-72	EpC2	RPL19
AKIRIN2	2.94822661199047e-76	0.340990859376094	0.352	0.125	5.9100150663961e-72	EpC2	AKIRIN2
GSTM3.2	3.26540620631189e-76	-1.042303177	0.139	0.458	6.54583328117281e-72	EpC2	GSTM3
EEF1B2	4.10351750844634e-76	-0.546199367	0.732	0.941	8.22591119743153e-72	EpC2	EEF1B2

TUBB.2	3.65414439439913e-75	-0.81238772	0.368	0.638	7.3250978530125e-71	EpC2	TUBB
EZR.1	6.23604173094647e-75	0.562891240597742	0.89	0.678	1.25007692538553e-70	EpC2	EZR
AGFG1	6.45352653216382e-75	0.256462567959955	0.226	0.057	1.29367392863756e-70	EpC2	AGFG1
GNA15	1.04274175039699e-74	0.279784258728816	0.3	0.094	2.0902801128458e-70	EpC2	GNA15
MDM2.1	4.95751344168172e-74	0.566717667700555	0.466	0.219	9.93783144519518e-70	EpC2	MDM2
EPCAM.2	5.43024050399252e-74	-0.7020759	0.278	0.641	1.08854601143034e-69	EpC2	EPCAM
HLA-E	8.21161170602077e-74	0.438913389816193	0.646	0.35	1.64609968258892e-69	EpC2	HLA-E
LAMC2.2	1.12745866011633e-73	0.651289162592936	0.344	0.125	2.26010363006919e-69	EpC2	LAMC2
IFI27.2	2.67982932745521e-73	1.12581452749446	0.249	0.073	5.37198586981672e-69	EpC2	IFI27
HNRNPK.1	3.79577834708346e-73	-0.553537516	0.61	0.794	7.6090172745635e-69	EpC2	HNRNPK
ARHGAP32	3.93644200295607e-73	0.354041751071728	0.318	0.108	7.89099163912575e-69	EpC2	ARHGAP32
S100A16.3	9.70977618636988e-73	0.591825521521232	0.898	0.687	1.94642173431971e-68	EpC2	S100A16
AGTRAP	1.08128383925494e-72	0.361491631927688	0.341	0.122	2.16754158417045e-68	EpC2	AGTRAP
ITSN2	1.90853287025854e-72	0.420605108761984	0.371	0.143	3.82584499172026e-68	EpC2	ITSN2
ID1.1	5.51690769177295e-72	0.726328272910751	0.704	0.452	1.10591931589281e-67	EpC2	ID1
RPL32	1.19372210903374e-71	-0.451850389	0.961	0.997	2.39293533976903e-67	EpC2	RPL32
ADRB2	1.42880008096049e-71	0.368812318376084	0.328	0.115	2.8641726422934e-67	EpC2	ADRB2
UAP1	1.44348994794803e-71	0.346645275397123	0.322	0.111	2.89361994965662e-67	EpC2	UAP1
RPS24	1.62091120955995e-71	-0.506799103	0.924	0.994	3.24927861068388e-67	EpC2	RPS24
HMGN3.1	7.63158190287184e-71	-0.637090986	0.392	0.663	1.52982690824969e-66	EpC2	HMGN3
GLTP.3	2.85517591370503e-70	0.891557112470476	0.672	0.421	5.7234856366131e-66	EpC2	GLTP
RPL35A	4.78058660731953e-70	-0.468048106	0.959	0.996	9.58316391303273e-66	EpC2	RPL35A
PI3.3	5.61232096535605e-70	0.509565108052637	0.524	0.26	1.12504586071527e-65	EpC2	PI3
RPS9	6.37338040925823e-70	-0.4731255	0.872	0.966	1.2776078368399e-65	EpC2	RPS9
RPS15A.1	1.9323127580402e-69	-0.496151654	0.721	0.928	3.87351415476738e-65	EpC2	RPS15A
TRIB1	3.67070197453604e-69	0.3276511053851	0.27	0.084	7.35828917815495e-65	EpC2	TRIB1
ID2.2	7.25597070045033e-69	0.715740543778245	0.73	0.485	1.45453188661227e-64	EpC2	ID2
TXNRD1	1.42724748440453e-68	0.522521044486687	0.501	0.251	2.86106030723732e-64	EpC2	TXNRD1
CYLD	1.77721010678937e-68	0.275825213306729	0.229	0.063	3.56259538006997e-64	EpC2	CYLD

NFIB.2	6.00091338133091e-68	-0.680908158	0.144	0.432	1.20294309642159e-63	EpC2	NFIB
ODF2L	6.79532622295597e-68	0.421347361156887	0.443	0.2	1.36219109465375e-63	EpC2	ODF2L
NAMPT.1	7.1453644925191e-68	0.430494107443273	0.458	0.211	1.43235976617038e-63	EpC2	NAMPT
EIF3E.1	7.75311918234913e-68	-0.579869711	0.452	0.702	1.55419027129371e-63	EpC2	EIF3E
HLA-C.1	9.69420744771965e-68	0.419275864890466	0.498	0.24	1.94330082496988e-63	EpC2	HLA-C
RPLP1	1.66089309771058e-67	-0.406505286	0.999	1	3.32942630367062e-63	EpC2	RPLP1
LRRC75A-AS1.1	1.67357083353872e-67	-0.817023555	0.162	0.455	3.35484009291171e-63	EpC2	LRRC75A-AS1
RPS11.1	1.73487188166546e-67	-0.472573264	0.914	0.987	3.47772417398658e-63	EpC2	RPS11
RPL7A	3.99517428100212e-67	-0.444307615	0.915	0.989	8.00872636369686e-63	EpC2	RPL7A
PTGR1.2	5.92993937572658e-67	0.431943553419627	0.594	0.305	1.18871564725815e-62	EpC2	PTGR1
ATP5MC2	7.93902482190757e-67	-0.544004872	0.593	0.838	1.59145691579959e-62	EpC2	ATP5MC2
SLC25A6.1	8.61441514580515e-67	-0.498608237	0.671	0.89	1.7268456601281e-62	EpC2	SLC25A6
RPL10.1	1.40202854543917e-66	-0.450744603	0.95	0.991	2.81050642218737e-62	EpC2	RPL10
RHOV.2	5.99328642830962e-66	0.339082606768311	0.421	0.179	1.20141419741895e-61	EpC2	RHOV
PPP4R1	1.89783493662919e-65	0.33790819779491	0.352	0.137	3.80439991396688e-61	EpC2	PPP4R1
RPL9.1	3.0757646616504e-65	-0.442481172	0.953	0.993	6.16567784074439e-61	EpC2	RPL9
RPL37A.1	4.75393021394303e-65	-0.458923684	0.95	0.995	9.5297285068702e-61	EpC2	RPL37A
GALNT5	7.47168060140101e-65	0.346194252796366	0.251	0.077	1.49777309335685e-60	EpC2	GALNT5
GSN.2	3.72440272284025e-64	-0.71283809	0.334	0.587	7.46593769820557e-60	EpC2	GSN
PNRC1.1	1.37349745054765e-63	0.575464087407922	0.713	0.464	2.75331298936783e-59	EpC2	PNRC1
S100A4.1	2.19154890514188e-63	1.45251841105026	0.446	0.229	4.3931789352474e-59	EpC2	S100A4
MT-CO1.2	2.734348592392e-63	-0.383098302	1	1	5.481275188309e-59	EpC2	MT-CO1
SDCBP2.3	4.56482993528209e-63	0.33683892058736	0.391	0.161	9.15065808826648e-59	EpC2	SDCBP2
SRSF11.1	4.92884254550223e-63	-0.551441308	0.629	0.798	9.88035776671376e-59	EpC2	SRSF11
CEBPB	7.54410053495116e-63	0.272601816651333	0.258	0.082	1.51229039323631e-58	EpC2	CEBPB
DEK.2	8.28389734023266e-63	-0.684874146	0.265	0.535	1.66059006082304e-58	EpC2	DEK
MT-ND3	1.11651006343087e-62	-0.386594063	0.999	1	2.23815607315352e-58	EpC2	MT-ND3
ZFP36.1	1.97083317277988e-62	0.51840186819947	0.823	0.566	3.95073217815456e-58	EpC2	ZFP36
CBX3.2	2.15971896921732e-62	-0.607761729	0.437	0.665	4.32937264569305e-58	EpC2	CBX3

RAN.2	2.84817123906723e-62	-0.579439118	0.585	0.772	5.70944406583417e-58	EpC2	RAN
TRIP10	2.95830822683106e-62	0.284321719853845	0.256	0.082	5.93022467150554e-58	EpC2	TRIP10
HIST1H4C.3	5.58091686081295e-62	-1.124151777	0.444	0.682	1.11875059391856e-57	EpC2	HIST1H4C
RPS27A	5.788553137143e-62	-0.441192998	0.967	0.995	1.16037336187169e-57	EpC2	RPS27A
BMP2	9.79611144129465e-62	0.27196609212541	0.182	0.044	1.96372849952192e-57	EpC2	BMP2
FTH1.3	9.82221477622399e-62	-1.107418627	0.474	0.687	1.96896117404186e-57	EpC2	FTH1
RPS28	2.19543713750036e-61	-0.433921071	0.929	0.985	4.40097328583322e-57	EpC2	RPS28
RAI14	4.86125438453282e-61	0.305536090218105	0.282	0.098	9.74487053923449e-57	EpC2	RAI14
LMO7	5.98100594236899e-61	0.527350940861446	0.379	0.168	1.19895245120729e-56	EpC2	LMO7
MAOA	1.61926106621447e-60	0.315831761305782	0.292	0.104	3.24597073333352e-56	EpC2	MAOA
SLC9A3R1.2	1.68840937038148e-60	0.405418800233552	0.737	0.454	3.38458542386672e-56	EpC2	SLC9A3R1
CLIP1.1	3.03871996460639e-60	0.371984014235229	0.454	0.216	6.09141804104996e-56	EpC2	CLIP1
SNAI1	4.58272355959412e-60	0.251814155801761	0.147	0.031	9.18652764756238e-56	EpC2	SNAI1
LRP10	5.7014438964786e-60	0.388145864244851	0.343	0.14	1.1429114434881e-55	EpC2	LRP10
ANXA3.2	9.38888480719901e-60	0.40066011952007	0.419	0.191	1.88209584845111e-55	EpC2	ANXA3
HIF1A.2	9.9870229529375e-60	0.52319124989653	0.695	0.454	2.00199862114585e-55	EpC2	HIF1A
TPI1.3	1.60758166968744e-59	-0.528966264	0.796	0.922	3.22255821505543e-55	EpC2	TPI1
PNPLA8.1	2.5164684341051e-59	0.385075321948251	0.462	0.224	5.04451262300709e-55	EpC2	PNPLA8
HMGN2.2	3.50843898314943e-59	-0.69134533	0.247	0.514	7.03301678562134e-55	EpC2	HMGN2
TMA7.1	5.18707200344769e-59	-0.559125855	0.472	0.693	1.03980045381112e-54	EpC2	TMA7
ARF4.1	8.51985838114104e-59	0.433879204547448	0.748	0.488	1.70789081108353e-54	EpC2	ARF4
PDXK	9.79708445893866e-59	0.26528257211925	0.284	0.101	1.96392355063884e-54	EpC2	PDXK
XPA.1	1.4054507793373e-58	-0.589840227	0.288	0.555	2.81736663225956e-54	EpC2	XPA
SOD1	1.50206337881552e-58	-0.516413242	0.609	0.804	3.01103624917358e-54	EpC2	SOD1
PTBP3.1	1.69388973052518e-58	0.376490316819117	0.504	0.259	3.39557135381078e-54	EpC2	PTBP3
NTS.3	2.65144660998272e-58	-1.420404471	0.116	0.385	5.31508987437137e-54	EpC2	NTS
SKP1	8.63365079697918e-58	-0.461088378	0.851	0.925	1.73070163876245e-53	EpC2	SKP1
TC2N	1.16329482966183e-57	0.291634395204502	0.322	0.126	2.33194081554011e-53	EpC2	TC2N
RPL24	1.25159809886836e-57	-0.408957793	0.968	0.992	2.50895354899151e-53	EpC2	RPL24

SCPEP1.1	1.67672235673848e-57	0.45229817908688	0.49	0.262	3.36115763631796e-53	EpC2	SCPEP1
PLK2.2	3.56339046287265e-57	0.455996696011394	0.652	0.386	7.14317252187451e-53	EpC2	PLK2
XBP1.1	3.78483126471985e-57	0.364654739176428	0.423	0.199	7.58707275325742e-53	EpC2	XBP1
TPM4.1	5.4508654972567e-57	0.550146529131841	0.902	0.781	1.09268049758008e-52	EpC2	TPM4
ZFAS1.1	6.89162241331527e-57	-0.516716409	0.491	0.738	1.38149462897318e-52	EpC2	ZFAS1
ADH7.2	7.14348895358349e-57	-0.654573898	0.211	0.488	1.43198379563535e-52	EpC2	ADH7
FOSB.2	1.62314659523383e-56	0.417407348760049	0.801	0.507	3.25375966480575e-52	EpC2	FOSB
VSNL1.3	2.27048273478461e-56	-0.592915976	0.547	0.784	4.55140969014922e-52	EpC2	VSNL1
RPS20	2.87785492641998e-56	-0.428719096	0.919	0.98	5.76894798550149e-52	EpC2	RPS20
CDKN2B.2	4.12161122923612e-56	0.302439768390285	0.295	0.11	8.26218187012673e-52	EpC2	CDKN2B
HM13.1	5.071382946695e-56	0.434094512733925	0.477	0.253	1.01660942549448e-51	EpC2	HM13
ETHE1.2	7.11043870633208e-56	0.48585317546251	0.656	0.416	1.42535854307133e-51	EpC2	ETHE1
PYGL.1	9.53282932751203e-56	0.273755163159093	0.292	0.108	1.91095096699306e-51	EpC2	PYGL
RPL41	9.92527136898089e-56	-0.44079735	0.848	0.967	1.98961989862591e-51	EpC2	RPL41
DUSP10	1.24602489547739e-55	0.345815239322914	0.343	0.143	2.49778150547397e-51	EpC2	DUSP10
RPL37.2	2.20297462474426e-55	-0.426054029	0.987	0.997	4.41608293276235e-51	EpC2	RPL37
KRT6C.3	2.32316263971482e-55	0.555594056983188	0.326	0.131	4.65701182757232e-51	EpC2	KRT6C
SCEL.2	2.58728399571357e-55	0.69416334761141	0.487	0.258	5.18646949780742e-51	EpC2	SCEL
ASCC2	4.04261464347415e-55	0.31817297194936	0.278	0.103	8.10382531430829e-51	EpC2	ASCC2
CLTB.2	5.64700407500046e-55	0.377644497436543	0.609	0.35	1.13199843687459e-50	EpC2	CLTB
UBB.1	6.01023422516121e-55	-0.561270232	0.786	0.889	1.20481155277582e-50	EpC2	UBB
TPD52L1.1	6.07061722126884e-55	0.333710684715645	0.395	0.18	1.21691592817555e-50	EpC2	TPD52L1
EIF4A2.1	7.72290092396742e-55	-0.481999041	0.505	0.758	1.54813271921851e-50	EpC2	EIF4A2
RPL35.2	1.28506560825247e-54	-0.423064898	0.892	0.979	2.5760425183029e-50	EpC2	RPL35
NDRG1	1.70215255030935e-54	0.297252684509843	0.315	0.125	3.41213500235012e-50	EpC2	NDRG1
SRP14	2.772064889463e-54	-0.440768402	0.829	0.904	5.55688127741753e-50	EpC2	SRP14
ITM2A	2.8232261280909e-54	-0.592725996	0.199	0.468	5.65943909637101e-50	EpC2	ITM2A
MFF	3.57415230695871e-54	0.391955095224663	0.514	0.282	7.16474571452942e-50	EpC2	MFF
NR4A2	3.93054131581869e-54	0.266496329678011	0.236	0.077	7.87916312169014e-50	EpC2	NR4A2

RPS21	5.64526178558195e-54	-0.427556487	0.883	0.976	1.13164917753776e-49	EpC2	RPS21
SH3GLB1.1	7.23035310520958e-54	0.411295897687204	0.617	0.37	1.44939658347031e-49	EpC2	SH3GLB1
HSPA6.1	8.76567052781242e-54	0.572446182827394	0.264	0.095	1.75716631400528e-49	EpC2	HSPA6
CEBPD	9.37466392709263e-54	0.340773260522769	0.442	0.211	1.87924513082499e-49	EpC2	CEBPD
HSP90AB1	9.85092512946698e-54	-0.415270963	0.865	0.983	1.97471645145295e-49	EpC2	HSP90AB1
LUM.2	1.08023507902156e-53	0.512760762740539	0.324	0.136	2.16543923940662e-49	EpC2	LUM
ODAM	1.43099659618976e-53	0.612030880452268	0.155	0.036	2.86857577672199e-49	EpC2	ODAM
PKN2	1.76962589208591e-53	0.379772222537693	0.494	0.261	3.54739206327542e-49	EpC2	PKN2
ARPC3.2	9.82077401854401e-53	0.441669486068927	0.878	0.723	1.96867235975733e-48	EpC2	ARPC3
RBP1	1.01949340069991e-52	0.42832950439282	0.617	0.359	2.04367647104304e-48	EpC2	RBP1
HOPX.3	1.13418702227271e-52	0.494884178361846	0.615	0.375	2.27359130484788e-48	EpC2	HOPX
MYL6.2	2.2447351351375e-52	0.336388258148935	0.989	0.983	4.49979605189663e-48	EpC2	MYL6
UBC.1	2.29532873380617e-52	0.361102919834616	0.99	0.953	4.60121597978785e-48	EpC2	UBC
TKT.2	4.67751271952591e-52	-0.513074326	0.385	0.641	9.37654199756163e-48	EpC2	TKT
GALNT3	4.80564918654875e-52	0.371329936524877	0.447	0.23	9.63340435935562e-48	EpC2	GALNT3
EEF1D.2	5.48561791864873e-52	-0.658031664	0.074	0.317	1.09964696797232e-47	EpC2	EEF1D
RSL1D1	5.61824036176456e-52	-0.524267383	0.435	0.651	1.12623246291932e-47	EpC2	RSL1D1
CIRBP.1	7.26711932773798e-52	-0.533373804	0.364	0.598	1.45676674043836e-47	EpC2	CIRBP
TMSB10.2	1.01312697226691e-51	0.410309566641529	0.997	0.997	2.03091432860625e-47	EpC2	TMSB10
PRDX2.1	1.04903618844304e-51	-0.652523428	0.252	0.491	2.10289794335292e-47	EpC2	PRDX2
SUMO2.1	1.30351173781513e-51	-0.454379956	0.79	0.885	2.61301962962422e-47	EpC2	SUMO2
ATP5F1B.2	1.35288244714277e-51	-0.467565223	0.702	0.834	2.7119881535424e-47	EpC2	ATP5F1B
HLA-B.1	1.8473389766351e-51	0.355344994209976	0.429	0.208	3.70317571256273e-47	EpC2	HLA-B
TGM3.2	2.88961328258003e-51	-0.801465608	0.244	0.505	5.79251878625992e-47	EpC2	TGM3
RIOK3	4.84084200010802e-51	0.346772457780507	0.674	0.405	9.70395187341654e-47	EpC2	RIOK3
LGALS7B.3	7.20977877486182e-51	0.31323383954528	0.521	0.283	1.4452722532088e-46	EpC2	LGALS7B
APLP2	9.54510090010969e-51	0.447656068077266	0.541	0.321	1.91341092643599e-46	EpC2	APLP2
DBI.2	3.273462240203e-50	-0.728395793	0.696	0.868	6.56198240671093e-46	EpC2	DBI
KLK11.3	5.05927640735359e-50	0.349713070041236	0.667	0.39	1.0141825486181e-45	EpC2	KLK11

GRHL1.2	8.31310783108054e-50	0.291902432317609	0.406	0.191	1.66644559581841e-45	EpC2	GRHL1
TUBA1B.2	9.65779602116274e-50	-1.12868507	0.074	0.307	1.93600179040228e-45	EpC2	TUBA1B
CCND2.1	4.24112187685886e-49	-0.521164555	0.122	0.362	8.50175291435127e-45	EpC2	CCND2
MCL1	4.44836013909706e-49	0.314950540601763	0.498	0.266	8.91718273483397e-45	EpC2	MCL1
PEBP1	5.17523433596405e-49	-0.489143801	0.491	0.7	1.03742747498735e-44	EpC2	PEBP1
SOSTDC1.3	9.7931482298543e-49	-0.603042391	0.245	0.535	1.96313449415659e-44	EpC2	SOSTDC1
ATP5MC3.2	1.40025628358362e-48	-0.44938602	0.827	0.918	2.80695374607172e-44	EpC2	ATP5MC3
COX7C.1	2.2678616534833e-48	-0.402001958	0.912	0.969	4.54615547057262e-44	EpC2	COX7C
BAMBI	2.5943889865763e-48	0.256008338130717	0.202	0.064	5.20071216249085e-44	EpC2	BAMBI
MX1.1	2.59488612719221e-48	0.471144318832745	0.273	0.108	5.2017087305695e-44	EpC2	MX1
MORF4L1.1	3.26051530567436e-48	-0.492527393	0.598	0.743	6.53602898175483e-44	EpC2	MORF4L1
H2AFJ.2	3.44032834596847e-48	0.333705943118178	0.462	0.24	6.89648220232839e-44	EpC2	H2AFJ
PCBP2	4.54131994421841e-48	-0.472281588	0.45	0.652	9.10352996018022e-44	EpC2	PCBP2
ACER3	9.59240512823581e-48	0.261110486560322	0.278	0.11	1.92289353200615e-43	EpC2	ACER3
RBMX.1	9.68922935998475e-48	-0.525599787	0.368	0.597	1.94230291750254e-43	EpC2	RBMX
RPL18.1	1.12529217534856e-47	-0.342090345	0.99	0.998	2.25576069470372e-43	EpC2	RPL18
DKK3.1	1.27358098020051e-47	-0.427530505	0.092	0.323	2.55302043290994e-43	EpC2	DKK3
AK2.1	1.75905820271733e-47	0.373605368885536	0.652	0.413	3.52620807316716e-43	EpC2	AK2
RPS14	2.13078101836744e-47	-0.331749764	0.992	0.998	4.27136362941937e-43	EpC2	RPS14
SHISA5	2.751863954382e-47	0.337785477834312	0.444	0.231	5.51638648295416e-43	EpC2	SHISA5
SNRPE.1	4.27675282893633e-47	-0.489698683	0.414	0.645	8.57317872088576e-43	EpC2	SNRPE
KRT6A.2	5.08433313594487e-47	0.251415012092941	0.962	0.905	1.01920542043151e-42	EpC2	KRT6A
SRP9.1	5.2562715424714e-47	-0.483016296	0.532	0.695	1.05367219340382e-42	EpC2	SRP9
ATP5PO.1	9.99087466105263e-47	-0.452108806	0.705	0.825	2.00277073455461e-42	EpC2	ATP5PO
DNAJB6	1.28017123245383e-46	0.339196393545533	0.557	0.328	2.56623125257696e-42	EpC2	DNAJB6
CFLAR	1.64337057222636e-46	0.26660414691445	0.35	0.159	3.29430064908496e-42	EpC2	CFLAR
BCAM.2	2.11187490386421e-46	-0.477993854	0.111	0.338	4.2334644322862e-42	EpC2	BCAM
NDUFB2.3	3.75932345041922e-46	-0.486574729	0.602	0.754	7.53593978871036e-42	EpC2	NDUFB2
GSTO1.2	4.19328883014124e-46	-0.49564833	0.7	0.827	8.40586678890113e-42	EpC2	GSTO1

COMMD6	5.0661860410304e-46	-0.459165059	0.577	0.757	1.01556765378495e-41	EpC2	COMMD6
ATP1B1.2	5.23439496231247e-46	0.455892159283836	0.601	0.38	1.04928681414516e-41	EpC2	ATP1B1
PPIC.2	7.75320860696502e-46	0.317121130821876	0.403	0.203	1.55420819735221e-41	EpC2	PPIC
ZNF185	1.53633202942822e-45	0.250098060641945	0.225	0.08	3.0797311861918e-41	EpC2	ZNF185
UPP1	2.1790737340537e-45	0.322956195739163	0.271	0.11	4.36817120728404e-41	EpC2	UPP1
MDH1.1	3.20471878650212e-45	-0.514196143	0.535	0.682	6.42417927942215e-41	EpC2	MDH1
TNIP1	3.99261119000245e-45	0.271321820195719	0.266	0.106	8.00358839147891e-41	EpC2	TNIP1
IER3	5.34355553898744e-45	0.425883502719286	0.734	0.493	1.07116914334542e-40	EpC2	IER3
SLC7A8.1	5.83750728293472e-45	-0.429310865	0.108	0.329	1.17018670993709e-40	EpC2	SLC7A8
CHP1	6.32940260457366e-45	0.269006577238759	0.34	0.155	1.26879204611284e-40	EpC2	CHP1
SERPINB13.3	9.31671401250874e-45	0.375039240743894	0.639	0.389	1.8676284909475e-40	EpC2	SERPINB13
FKBP1A.2	2.02105137944194e-44	-0.566930728	0.538	0.66	4.05139959522931e-40	EpC2	FKBP1A
PNOC.3	2.03633932404811e-44	-0.57486357	0.026	0.218	4.08204580898685e-40	EpC2	PNOC
RBM8A.1	2.12752573709639e-44	-0.462803322	0.568	0.728	4.26483809258342e-40	EpC2	RBM8A
ALDH3A2	2.59821789736239e-44	-0.432332861	0.349	0.589	5.20838759705265e-40	EpC2	ALDH3A2
PTGS2	7.77571950772013e-44	0.306664080627511	0.167	0.05	1.55872073251758e-39	EpC2	PTGS2
IFI16.2	1.07949253734667e-43	0.446496816863869	0.551	0.331	2.16395074036513e-39	EpC2	IFI16
SQOR.2	1.46821678821434e-43	0.279338910020801	0.363	0.172	2.94318737365447e-39	EpC2	SQOR
UBA6	5.04262229574831e-43	0.28973372140416	0.42	0.217	1.01084406540571e-38	EpC2	UBA6
CYB5A.2	5.58495359992117e-43	-0.598860045	0.318	0.522	1.1195597986402e-38	EpC2	CYB5A
UBE2H	5.69270428688944e-43	0.299599488055759	0.363	0.178	1.14115950134986e-38	EpC2	UBE2H
CAPNS2.2	7.005389533858e-43	-0.532732882	0.304	0.53	1.40430038595717e-38	EpC2	CAPNS2
BZW1.1	9.16834309406035e-43	0.359623331509281	0.686	0.453	1.83788605663534e-38	EpC2	BZW1
CNBP	9.79591696027093e-43	-0.439171735	0.569	0.727	1.96368951385591e-38	EpC2	CNBP
SELENOK	1.38164017740936e-42	0.389982850750848	0.67	0.439	2.7696358996348e-38	EpC2	SELENOK
ISG15	1.95269580265691e-42	0.622570861370324	0.252	0.101	3.91437400600603e-38	EpC2	ISG15
CST3.1	2.26223391522667e-42	0.354763064447356	0.706	0.481	4.53487410646338e-38	EpC2	CST3
AKR1B10.2	2.49645766723804e-42	0.410739218970802	0.624	0.38	5.00439903974537e-38	EpC2	AKR1B10
MPP7	3.49344560518715e-42	0.256059102691162	0.269	0.112	7.00296106015816e-38	EpC2	MPP7

OAS1	4.1601852724233e-42	0.260140544934186	0.18	0.058	8.33950739709974e-38	EpC2	OAS1
RPS19	8.03498058758556e-42	-0.292848069	0.994	0.999	1.6106922085874e-37	EpC2	RPS19
RPL27.1	8.47743435368035e-42	-0.359668444	0.947	0.987	1.69938649053876e-37	EpC2	RPL27
SON.1	8.51596775472853e-42	-0.488485762	0.663	0.772	1.70711089611288e-37	EpC2	SON
HNRNPC	8.92224777769418e-42	-0.445672333	0.639	0.753	1.78855378951657e-37	EpC2	HNRNPC
SRSF3.1	1.15979753472403e-41	-0.446860545	0.537	0.699	2.3249301381078e-37	EpC2	SRSF3
SYNE2.1	1.19988146751685e-41	-0.490444985	0.252	0.475	2.40528238978428e-37	EpC2	SYNE2
AKR1B1.1	1.2214804617142e-41	0.548332703799457	0.398	0.218	2.44857973355228e-37	EpC2	AKR1B1
CTNND1.2	1.35315143929458e-41	0.334747114010333	0.548	0.324	2.71252737520991e-37	EpC2	CTNND1
MYO1B	1.46733947383859e-41	0.289526090303208	0.332	0.155	2.94142870925683e-37	EpC2	MYO1B
HSPA5.1	1.59558505194375e-41	0.551766573488925	0.866	0.731	3.19850979512643e-37	EpC2	HSPA5
PDCD5.3	1.70551734114941e-41	-0.48676619	0.409	0.605	3.41888006206811e-37	EpC2	PDCD5
VPS4B.1	2.04338111320905e-41	0.39648580222346	0.663	0.459	4.09616177953886e-37	EpC2	VPS4B
CBX1.1	3.18738712474727e-41	-0.44553216	0.279	0.5	6.38943623026837e-37	EpC2	CBX1
ADIRF.1	4.7982498748565e-41	-0.59393739	0.07	0.275	9.61857169913734e-37	EpC2	ADIRF
STAT3	6.49724979772377e-41	0.314195413352196	0.454	0.254	1.30243869445171e-36	EpC2	STAT3
HSPD1.1	8.93384005594368e-41	-0.452459194	0.495	0.689	1.79087757761447e-36	EpC2	HSPD1
RPL38.1	1.63194940362116e-40	-0.385848736	0.851	0.939	3.27140577449898e-36	EpC2	RPL38
SMIM14	1.6940588753896e-40	0.273029742494523	0.445	0.241	3.395910421606e-36	EpC2	SMIM14
NDFIP2	4.49277720919572e-40	0.317425185841282	0.458	0.26	9.00622119355375e-36	EpC2	NDFIP2
PICALM	5.41808484368469e-40	0.273723664898535	0.356	0.176	1.08610928776503e-35	EpC2	PICALM
AL365357.1	1.15626460004029e-39	-0.483287668	0.068	0.264	2.31784801724076e-35	EpC2	AL365357.1
RAP2B	1.34806396157649e-39	0.278808816292773	0.449	0.244	2.70232901737622e-35	EpC2	RAP2B
SERBP1.2	2.25825791833562e-39	-0.414763355	0.535	0.702	4.52690382309558e-35	EpC2	SERBP1
TNFAIP8.1	2.59029593182931e-39	0.496048897279764	0.442	0.255	5.19250722494502e-35	EpC2	TNFAIP8
ATP6V1D.1	2.91983440208684e-39	0.334930050211108	0.534	0.328	5.85310004242328e-35	EpC2	ATP6V1D
AKR1C2.3	3.22187388442682e-39	-0.739568306	0.272	0.486	6.458568388722e-35	EpC2	AKR1C2
NDUFA6.2	9.99460913609121e-39	-0.462810769	0.488	0.66	2.00351934742084e-34	EpC2	NDUFA6
TMEM173	1.0379921758169e-38	0.256949742228964	0.266	0.115	2.08075911564256e-34	EpC2	TMEM173

FAM3C	1.19495808131786e-38	0.271538198041433	0.307	0.143	2.39541296980978e-34	EpC2	FAM3C
TMEM165.1	1.21489468366958e-38	0.288412556111778	0.412	0.223	2.43537788288405e-34	EpC2	TMEM165
CCDC186	1.52438553561003e-38	0.2994591194739	0.437	0.243	3.05578324468386e-34	EpC2	CCDC186
ERRFI1.1	2.58434478558435e-38	0.335073545817421	0.439	0.243	5.18057755718239e-34	EpC2	ERRFI1
HNRNPA3.1	3.24414481969873e-38	-0.442307834	0.568	0.704	6.50321270556808e-34	EpC2	HNRNPA3
TMEM54.1	3.68101096965662e-38	0.28335979248225	0.385	0.202	7.37895458977366e-34	EpC2	TMEM54
ELOC.1	3.8201459965314e-38	0.370385765763855	0.761	0.544	7.65786466464685e-34	EpC2	ELOC
GADD45A.2	4.98561496423365e-38	0.358185184685107	0.63	0.399	9.99416375730278e-34	EpC2	GADD45A
STK38L	6.09759634923962e-38	0.269494995548078	0.254	0.109	1.22232416416857e-33	EpC2	STK38L
SSR3	9.93525651947509e-38	0.303782584361396	0.499	0.298	1.99162152189398e-33	EpC2	SSR3
C19orf33.2	1.15998595746661e-37	0.297244626407438	0.591	0.366	2.32530785033758e-33	EpC2	C19orf33
ELL2.2	1.23129484725935e-37	0.256242862099041	0.332	0.162	2.4682536508161e-33	EpC2	ELL2
AC005083.1	1.25553589378929e-37	0.437460430255907	0.111	0.028	2.51684725269001e-33	EpC2	AC005083.1
CTNNAL1.2	1.50546584498839e-37	-0.332746781	0.023	0.192	3.01785683286372e-33	EpC2	CTNNAL1
NPC2	3.15311457430908e-37	0.391629906761224	0.771	0.584	6.32073347565997e-33	EpC2	NPC2
SPRR1A.3	3.31101430018297e-37	0.407263928866477	0.253	0.106	6.63725926614679e-33	EpC2	SPRR1A
PFDN5.1	4.2028278243229e-37	-0.409693691	0.781	0.859	8.42498865663768e-33	EpC2	PFDN5
DUT.1	6.87007475049682e-37	-0.481725167	0.249	0.455	1.37717518448459e-32	EpC2	DUT
TGFBI.2	1.04203123086941e-36	-0.447229658	0.149	0.351	2.08885580540083e-32	EpC2	TGFBI
LGMN.2	1.09549247133118e-36	0.289294951518815	0.403	0.217	2.19602420803049e-32	EpC2	LGMN
B4GALT1	1.28537155144912e-36	0.283822603764837	0.33	0.165	2.5766558120349e-32	EpC2	B4GALT1
TCEAL4	1.7181660186107e-36	-0.417532287	0.295	0.501	3.44423560090702e-32	EpC2	TCEAL4
OPTN	3.08268481230936e-36	0.261602117266088	0.373	0.194	6.17954997475535e-32	EpC2	OPTN
EFNB2	3.28170581169079e-36	0.253270204929371	0.324	0.158	6.57850747011536e-32	EpC2	EFNB2
ERH.1	3.46880639664684e-36	-0.448104147	0.489	0.635	6.95356930271826e-32	EpC2	ERH
HNRNPDL.1	4.97319640284925e-36	-0.386676039	0.556	0.718	9.96926950915161e-32	EpC2	HNRNPDL
ATP5PB.1	5.35258695766025e-36	-0.412352865	0.642	0.741	1.07297958153257e-31	EpC2	ATP5PB
SNHG8.2	5.64416979641818e-36	-0.410018692	0.42	0.639	1.13143027738999e-31	EpC2	SNHG8
AKR1A1	6.46599902938893e-36	0.339277775781672	0.554	0.357	1.2961741654313e-31	EpC2	AKR1A1

UQCRH.1	6.85854711312102e-36	-0.359849586	0.875	0.931	1.37486435429624e-31	EpC2	UQCRH
TPT1	7.16073889298592e-36	0.273005725750341	0.994	0.987	1.43544171848796e-31	EpC2	TPT1
TMEM14A.2	7.63109275034608e-36	-0.421114537	0.268	0.481	1.52972885273438e-31	EpC2	TMEM14A
TUBB2A.2	1.49725436349176e-35	0.271204687678907	0.409	0.223	3.00139609705557e-31	EpC2	TUBB2A
FAM213A	1.66063046447046e-35	-0.416160491	0.703	0.81	3.32889982907748e-31	EpC2	FAM213A
ATP2B1.1	2.06462475100258e-35	-0.412850284	0.178	0.381	4.13874677585976e-31	EpC2	ATP2B1
MCFD2	2.53669148241956e-35	0.291818439087666	0.364	0.194	5.08505174565825e-31	EpC2	MCFD2
SPARC.1	2.74816562696898e-35	-0.39436149	0.108	0.304	5.50897281582201e-31	EpC2	SPARC
KDM5B	3.60336801613015e-35	0.286722795149824	0.406	0.225	7.22331152513449e-31	EpC2	KDM5B
COX7B.3	3.74545422900852e-35	-0.388904581	0.753	0.841	7.50813754747048e-31	EpC2	COX7B
C8orf59.1	3.82115466281135e-35	-0.369144331	0.227	0.43	7.65988663707163e-31	EpC2	C8orf59
ALCAM.1	3.91122222145684e-35	-0.354570617	0.228	0.456	7.84043606513239e-31	EpC2	ALCAM
SPTSSA.1	5.92085201991541e-35	0.34851285713928	0.606	0.409	1.18689399591224e-30	EpC2	SPTSSA
HNRNPD.1	6.02330473868765e-35	-0.431862054	0.463	0.617	1.20743166791733e-30	EpC2	HNRNPD
HP1BP3.1	6.6802925436614e-35	-0.472379265	0.473	0.629	1.33913144330237e-30	EpC2	HP1BP3
CBR1.1	6.77361337907699e-35	-0.352911933	0.595	0.835	1.35783853796977e-30	EpC2	CBR1
COX6A1.2	7.28380950226627e-35	-0.335780201	0.864	0.917	1.4601124528243e-30	EpC2	COX6A1
MAFB	1.43007705663868e-34	0.345271586208396	0.481	0.295	2.8667324677379e-30	EpC2	MAFB
KIF21A	1.48649172246867e-34	-0.425456704	0.304	0.506	2.97982130686069e-30	EpC2	KIF21A
YWHAE.1	1.71675489780164e-34	-0.363406665	0.729	0.8	3.44140686813317e-30	EpC2	YWHAE
APEX1	1.90181908888894e-34	-0.365770663	0.245	0.448	3.81238654558678e-30	EpC2	APEX1
HIGD2A	2.94718263374138e-34	-0.390744275	0.368	0.572	5.90792230759798e-30	EpC2	HIGD2A
ATP5MG.1	6.7800747108952e-34	-0.329578624	0.905	0.947	1.35913377654605e-29	EpC2	ATP5MG
RGCC	1.15192390847353e-33	0.250936512558514	0.205	0.082	2.30914666692603e-29	EpC2	RGCC
HCAR2	1.52960946366914e-33	0.268766826163444	0.325	0.165	3.06625513087115e-29	EpC2	HCAR2
RPL28	2.71442436113546e-33	-0.343372071	0.911	0.952	5.44133507433215e-29	EpC2	RPL28
REXO2.2	2.88004528139418e-33	0.282223869696633	0.538	0.334	5.77333877108278e-29	EpC2	REXO2
IGKC.1	3.0368190735045e-33	0.824465699819419	0.174	0.062	6.08760751474711e-29	EpC2	IGKC
ATP5PF.3	3.36015824546688e-33	-0.357785518	0.847	0.881	6.7357732188629e-29	EpC2	ATP5PF

C12orf57.1	4.48814120994921e-33	-0.396172522	0.277	0.472	8.99692786946418e-29	EpC2	C12orf57
STMP1.2	5.22826588175858e-33	0.279516113571608	0.638	0.423	1.04805817865732e-28	EpC2	STMP1
CYC1.2	5.98732933814375e-33	-0.416403914	0.337	0.52	1.2002200391243e-28	EpC2	CYC1
ATP5MC1.3	6.46887988374386e-33	-0.417901985	0.428	0.604	1.29675166149529e-28	EpC2	ATP5MC1
PPL.2	9.8303621146335e-33	0.3601768299798	0.376	0.21	1.97059438949943e-28	EpC2	PPL
RASSF9	1.0140918877606e-32	-0.352527476	0.134	0.326	2.0328485982049e-28	EpC2	RASSF9
NDRG2.2	1.107458166386e-32	0.258033331118448	0.264	0.123	2.22001064033738e-28	EpC2	NDRG2
SSB	4.32096255930182e-32	-0.384537581	0.28	0.473	8.66180154637642e-28	EpC2	SSB
SET.1	4.34526847951657e-32	-0.387760612	0.436	0.607	8.71052519403892e-28	EpC2	SET
RNF7	5.64206693248629e-32	-0.405636323	0.417	0.586	1.1310087372862e-27	EpC2	RNF7
CDH13.2	5.65178554477347e-32	-0.333266305	0.081	0.253	1.13295693030529e-27	EpC2	CDH13
CLINT1.2	5.76580390544592e-32	0.325001858128836	0.642	0.44	1.15581305088569e-27	EpC2	CLINT1
RANBP1.2	6.07628788929306e-32	-0.44784594	0.304	0.481	1.21805267028769e-27	EpC2	RANBP1
PRMT1.1	6.64706939349886e-32	-0.366041935	0.293	0.488	1.33247153062078e-27	EpC2	PRMT1
GJB6.3	1.09707055487456e-31	0.341840799052118	0.373	0.206	2.19918763430154e-27	EpC2	GJB6
RPLP2.1	1.12866414918862e-31	-0.277635112	0.999	0.998	2.2625201534635e-27	EpC2	RPLP2
ATP5MPL.1	1.7048629426275e-31	-0.369907776	0.788	0.838	3.41756825479109e-27	EpC2	ATP5MPL
NAP1L1.2	3.69280567153169e-31	-0.42616085	0.4	0.567	7.40259824915243e-27	EpC2	NAP1L1
EIF4A3	4.59607834963121e-31	0.312972107166541	0.657	0.462	9.21329865967072e-27	EpC2	EIF4A3
VDAC1.3	4.63100712211778e-31	-0.409123789	0.523	0.643	9.28331687699731e-27	EpC2	VDAC1
HMGN1.2	5.39358605422708e-31	-0.390536098	0.521	0.65	1.08119826043036e-26	EpC2	HMGN1
UBA52	5.69554594793229e-31	-0.305187401	0.937	0.965	1.14172914072251e-26	EpC2	UBA52
IGHG3	1.35357139997856e-30	0.442645444997037	0.106	0.029	2.71336922839701e-26	EpC2	IGHG3
KRT5.1	1.51833255091696e-30	0.396159841396651	0.941	0.928	3.04364943156814e-26	EpC2	KRT5
ANP32B.1	1.74365545611341e-30	-0.396472424	0.315	0.492	3.49533172732493e-26	EpC2	ANP32B
CKMT1B	1.74463910360829e-30	-0.332672693	0.131	0.308	3.49730354709318e-26	EpC2	CKMT1B
SNHG5	1.76704894852396e-30	-0.345480097	0.502	0.676	3.54222632221113e-26	EpC2	SNHG5
ATP5F1A	2.35345809041945e-30	-0.368083105	0.586	0.716	4.71774208805482e-26	EpC2	ATP5F1A
ACSL4	2.47988145520622e-30	-0.294419023	0.059	0.216	4.97117036510639e-26	EpC2	ACSL4

SSR1	3.89151252927136e-30	0.276839344808105	0.541	0.356	7.80092601617737e-26	EpC2	SSR1
ATP5ME.3	5.14473591837194e-30	-0.385653567	0.481	0.63	1.03131376219684e-25	EpC2	ATP5ME
SEM1.1	6.2791593842311e-30	-0.345516066	0.793	0.847	1.25872029016297e-25	EpC2	SEM1
ILF3.1	6.45334941781574e-30	-0.359888897	0.244	0.428	1.29363842429534e-25	EpC2	ILF3
GABARAPL2	9.285043086249e-30	0.284339292147754	0.595	0.404	1.86127973706948e-25	EpC2	GABARAPL2
LAMP2	1.4160555293993e-29	0.268291090382631	0.667	0.468	2.83862491423384e-25	EpC2	LAMP2
CUTA	1.50123637839176e-29	-0.388154096	0.422	0.589	3.00937844412412e-25	EpC2	CUTA
ATP6V0B.2	1.7339821678137e-29	0.302907457636476	0.662	0.475	3.47594065359935e-25	EpC2	ATP6V0B
LGALS1.2	2.09561284701831e-29	-0.683939091	0.159	0.335	4.2008655131329e-25	EpC2	LGALS1
CADM1.1	2.11050186008153e-29	-0.316927395	0.129	0.304	4.23071202871944e-25	EpC2	CADM1
SREK1IP1.1	2.17248270461108e-29	0.262944710768301	0.454	0.278	4.35495882966338e-25	EpC2	SREK1IP1
NTRK2.1	2.81784652726725e-29	-0.336607561	0.169	0.354	5.64865514855993e-25	EpC2	NTRK2
IGHG4	2.82966787921748e-29	0.405738510488933	0.117	0.035	5.67235223067936e-25	EpC2	IGHG4
LAMB3.2	4.78584334293789e-29	0.408151749243786	0.529	0.351	9.59370156525329e-25	EpC2	LAMB3
IDI1.1	5.22442152995561e-29	0.278564676634736	0.443	0.267	1.0472875398949e-24	EpC2	IDI1
POLR2J3.3	6.52490921714173e-29	-0.542341567	0.233	0.412	1.30798330166823e-24	EpC2	POLR2J3
MRPL51.1	7.86534092121772e-29	-0.428002766	0.484	0.614	1.5766862410673e-24	EpC2	MRPL51
ATP5F1C.1	8.58005805346781e-29	-0.353736089	0.661	0.74	1.71995843739816e-24	EpC2	ATP5F1C
BANF1.1	9.27281984348652e-29	-0.375657886	0.398	0.563	1.85882946582531e-24	EpC2	BANF1
EIF1AX	9.89341705297964e-29	-0.35085134	0.334	0.514	1.9832343824403e-24	EpC2	EIF1AX
TACC2	1.09353458458158e-28	0.26510400984716	0.374	0.214	2.19209942825223e-24	EpC2	TACC2
SNRPD1.1	1.09693930077018e-28	-0.40929459	0.424	0.565	2.1989245223239e-24	EpC2	SNRPD1
XRCC5.1	1.12687391101825e-28	-0.393837418	0.675	0.719	2.25893144202718e-24	EpC2	XRCC5
NUPR1.1	1.71951772157984e-28	-0.325308216	0.362	0.568	3.44694522467894e-24	EpC2	NUPR1
EEF2.1	1.72191781312333e-28	-0.389592849	0.606	0.716	3.45175644818703e-24	EpC2	EEF2
PDPN.2	2.00393449509454e-28	-0.548319112	0.24	0.41	4.01708708886651e-24	EpC2	PDPN
SFN.2	2.65112820820985e-28	0.392773914998723	0.906	0.828	5.31445160617747e-24	EpC2	SFN
SDHD.1	2.70164524986831e-28	-0.369188027	0.414	0.57	5.41571806788601e-24	EpC2	SDHD
TUBA1A.1	3.50990322827433e-28	-0.371814855	0.245	0.443	7.03595201139872e-24	EpC2	TUBA1A

HACD3.1	3.52738631619293e-28	-0.330788609	0.171	0.347	7.07099860944034e-24	EpC2	HACD3
PFN1.3	3.66816581552928e-28	-0.34275734	0.951	0.965	7.35320519380999e-24	EpC2	PFN1
NDUFA1.1	4.04970943067914e-28	-0.357864032	0.668	0.731	8.1180475247394e-24	EpC2	NDUFA1
ATP5PD.2	4.85505384411064e-28	-0.342561067	0.818	0.859	9.7324409359042e-24	EpC2	ATP5PD
EID1	4.94211935204354e-28	-0.382369485	0.4	0.557	9.90697245310649e-24	EpC2	EID1
CENPP	4.98957536032656e-28	-0.342971157	0.079	0.235	1.00021027673106e-23	EpC2	CENPP
HSPE1.1	6.40299012156693e-28	-0.36355478	0.592	0.719	1.28354339976931e-23	EpC2	HSPE1
PRKX	2.29784693889178e-27	-0.320361483	0.182	0.361	4.60626397370246e-23	EpC2	PRKX
SLITRK6.2	3.00450258502671e-27	-0.303389332	0.033	0.167	6.02282588194455e-23	EpC2	SLITRK6
FAM210B.1	3.2427926412449e-27	-0.410469618	0.2	0.37	6.50050212863953e-23	EpC2	FAM210B
RHOC	3.78298419326611e-27	-0.332773402	0.234	0.41	7.58337011382125e-23	EpC2	RHOC
ANAPC16	3.89781149811239e-27	-0.367362832	0.383	0.539	7.81355292911611e-23	EpC2	ANAPC16
PPIB.1	5.83298776597419e-27	-0.350544262	0.092	0.251	1.16928072756719e-22	EpC2	PPIB
PA2G4.3	6.00123996421562e-27	-0.377878403	0.345	0.502	1.20300856322666e-22	EpC2	PA2G4
ITM2B.3	6.31557795229594e-27	0.264717549951675	0.965	0.883	1.26602075631724e-22	EpC2	ITM2B
C1QBP.3	9.21594759690309e-27	-0.349829614	0.289	0.464	1.84742885527519e-22	EpC2	C1QBP
PYCR2	1.02146218312778e-26	-0.25654685	0.114	0.275	2.04762309229794e-22	EpC2	PYCR2
ILF2.3	1.65503177060735e-26	-0.343375799	0.3	0.467	3.31767668735949e-22	EpC2	ILF2
LPAR6.1	1.76502074314564e-26	0.280080701226317	0.43	0.27	3.53816058170975e-22	EpC2	LPAR6
ST13.1	1.96079049860704e-26	-0.368993472	0.417	0.563	3.93060063350767e-22	EpC2	ST13
RPS29	3.09959641866858e-26	-0.280963535	0.966	0.987	6.21345098086304e-22	EpC2	RPS29
SEPT7.1	3.48994363646206e-26	-0.393545595	0.622	0.69	6.99594101365185e-22	EpC2	SEPT7
TCF4.1	4.59681511608698e-26	-0.311839465	0.181	0.352	9.21477558170795e-22	EpC2	TCF4
VCAN.1	4.76022182867453e-26	-0.402920898	0.162	0.332	9.54234067776096e-22	EpC2	VCAN
BCLAF1	6.21590559509249e-26	-0.343469782	0.393	0.548	1.24604043559224e-21	EpC2	BCLAF1
SLC3A2.1	6.35393987218606e-26	-0.371839444	0.315	0.475	1.27371078677842e-21	EpC2	SLC3A2
UQCRC1.1	6.68051742017181e-26	-0.361063528	0.374	0.536	1.33917652204764e-21	EpC2	UQCRC1
NHP2.1	7.38018493688059e-26	-0.327085203	0.101	0.256	1.47943187244708e-21	EpC2	NHP2
NDUFB9.2	7.79160004816504e-26	-0.348483832	0.612	0.713	1.56190414565516e-21	EpC2	NDUFB9

SPINT2.2	1.2768658235957e-25	0.259906819966006	0.914	0.778	2.55960522997995e-21	EpC2	SPINT2
TMEM147.2	2.23612069349073e-25	0.255713959112238	0.585	0.402	4.48252754217152e-21	EpC2	TMEM147
STARD7	2.45244949541102e-25	-0.32275858	0.21	0.375	4.91618025850092e-21	EpC2	STARD7
HNRNPM.1	2.45329268244105e-25	-0.36492208	0.377	0.52	4.91787051122132e-21	EpC2	HNRNPM
ITGA2.1	2.8124419382449e-25	-0.38600622	0.464	0.602	5.63782110940572e-21	EpC2	ITGA2
COX8A.3	2.98627035289413e-25	-0.351642068	0.801	0.828	5.98627754941157e-21	EpC2	COX8A
COX5B.2	3.26645635349702e-25	-0.304027386	0.896	0.921	6.54793840622013e-21	EpC2	COX5B
MT-ND6.2	3.73950533927413e-25	0.288522934883753	0.932	0.783	7.49621240310892e-21	EpC2	MT-ND6
EIF3K.1	3.85233479137884e-25	-0.324263203	0.684	0.766	7.72239032279801e-21	EpC2	EIF3K
SYNCRIP.1	4.22715403576757e-25	-0.346011863	0.412	0.561	8.47375298009968e-21	EpC2	SYNCRIP
PMAIP1	4.2322931827413e-25	0.391577834583826	0.674	0.512	8.48405491412321e-21	EpC2	PMAIP1
RPL39	4.67663758578867e-25	-0.285203803	0.96	0.983	9.37478770447196e-21	EpC2	RPL39
KLF5	4.72540552357968e-25	0.273412044824375	0.809	0.629	9.47254791256783e-21	EpC2	KLF5
LUC7L3.1	4.99744081853798e-25	-0.327651443	0.214	0.381	1.00178698648412e-20	EpC2	LUC7L3
MRPS21	5.15258792001881e-25	-0.355192608	0.477	0.61	1.03288777444697e-20	EpC2	MRPS21
PARK7.1	5.89995200536916e-25	-0.348880996	0.481	0.605	1.1827043789963e-20	EpC2	PARK7
PRKDC.1	5.90961987816882e-25	-0.35094898	0.236	0.398	1.18464240077772e-20	EpC2	PRKDC
OCIAD2.1	9.20040207734112e-25	-0.342410644	0.247	0.409	1.8443126004238e-20	EpC2	OCIAD2
EIF3M	1.05079040760816e-24	-0.332425551	0.324	0.48	2.10641445109132e-20	EpC2	EIF3M
PTCH1.2	2.11687930502151e-24	-0.350316137	0.098	0.244	4.24349625484612e-20	EpC2	PTCH1
FBL.1	2.42710089179403e-24	-0.314918138	0.242	0.407	4.86536644769031e-20	EpC2	FBL
CCDC34.1	2.51477530924079e-24	-0.311109913	0.09	0.234	5.04111858490408e-20	EpC2	CCDC34
LMO4.1	3.56000904837874e-24	-0.371553706	0.443	0.59	7.13639413838002e-20	EpC2	LMO4
SNU13.1	3.56678773757071e-24	-0.358841419	0.435	0.567	7.14998269873425e-20	EpC2	SNU13
RBBP8.1	3.96535107820261e-24	-0.334970649	0.171	0.33	7.94894277136494e-20	EpC2	RBBP8
CAV1.1	4.1072558691502e-24	-0.360813508	0.139	0.293	8.2334051152985e-20	EpC2	CAV1
CKS1B.3	5.41370675087096e-24	-0.415478864	0.128	0.271	1.08523165527959e-19	EpC2	CKS1B
HSP90B1.1	6.02768487007833e-24	-0.358117516	0.808	0.834	1.2083097090559e-19	EpC2	HSP90B1
ID3.2	6.34003558982246e-24	-0.48876399	0.384	0.543	1.27092353433581e-19	EpC2	ID3

SAMD12.1	7.45371646391202e-24	-0.283273174	0.188	0.351	1.4941720023558e-19	EpC2	SAMD12
PNKD	7.78975734988472e-24	-0.314945562	0.281	0.444	1.56153475835789e-19	EpC2	PNKD
TMPRSS4.2	8.95355559111769e-24	0.30187097338298	0.464	0.315	1.79482975379545e-19	EpC2	TMPRSS4
RBM39	9.27454891297868e-24	-0.345789413	0.71	0.763	1.85917607509571e-19	EpC2	RBM39
GPX1	1.06252979027771e-23	0.283323469498887	0.786	0.611	2.12994721759069e-19	EpC2	GPX1
PRNP	1.06719093978967e-23	0.467225765758089	0.553	0.415	2.13929095790237e-19	EpC2	PRNP
PIR.2	1.10378853561551e-23	-0.270299782	0.144	0.299	2.21265449849484e-19	EpC2	PIR
YWHAQ.2	1.21353737581321e-23	-0.329582491	0.462	0.592	2.43265702355515e-19	EpC2	YWHAQ
H2AFZ.3	1.27157188258298e-23	-0.709030889	0.744	0.779	2.54899299582583e-19	EpC2	H2AFZ
ZFYVE21	1.27630372216992e-23	-0.287258986	0.212	0.372	2.55847844146182e-19	EpC2	ZFYVE21
CARD16	1.45045556498854e-23	-0.304249331	0.174	0.332	2.90758322557603e-19	EpC2	CARD16
PRDX6.2	2.22080846806281e-23	-0.411855947	0.446	0.556	4.45183265507872e-19	EpC2	PRDX6
NDUFA4.3	2.50263888501851e-23	-0.300201108	0.956	0.969	5.01678990890811e-19	EpC2	NDUFA4
IGFBP7.2	3.56982041185023e-23	-0.298985535	0.065	0.199	7.15606199759497e-19	EpC2	IGFBP7
MT-ND5.2	3.74564502328062e-23	-0.258660264	1	1	7.50852001366833e-19	EpC2	MT-ND5
RAD21.2	3.99282391796731e-23	-0.430056625	0.358	0.485	8.00401482595727e-19	EpC2	RAD21
PHB2.1	5.71301535348655e-23	-0.280656211	0.228	0.388	1.14523105775991e-18	EpC2	PHB2
SRRM1.1	5.86349623373522e-23	-0.362369016	0.492	0.612	1.17539645501456e-18	EpC2	SRRM1
SRSF7.1	5.96675490037344e-23	-0.294195279	0.572	0.699	1.19609568732886e-18	EpC2	SRSF7
SMAP1.2	6.02365180139017e-23	-0.322152468	0.192	0.35	1.20750124010667e-18	EpC2	SMAP1
CRYAB.2	6.05696316873495e-23	0.769918500034485	0.586	0.441	1.21417883680461e-18	EpC2	CRYAB
IRF6	6.94565909286891e-23	0.259632950257433	0.548	0.385	1.3923268217565e-18	EpC2	IRF6
UGP2.2	7.86400084120902e-23	-0.409285663	0.421	0.54	1.57641760862876e-18	EpC2	UGP2
PLP2.3	8.45736430605022e-23	-0.324005005	0.812	0.849	1.69536324879083e-18	EpC2	PLP2
BLCAP	8.56710666139303e-23	-0.355006939	0.322	0.46	1.71736220134285e-18	EpC2	BLCAP
DDX6	8.72840680054683e-23	-0.298601086	0.233	0.384	1.74969642723762e-18	EpC2	DDX6
IMPDH2.1	9.59196513275807e-23	-0.278554503	0.229	0.392	1.92280533051268e-18	EpC2	IMPDH2
CCT2.1	1.03344351446386e-22	-0.343616203	0.365	0.515	2.07164086909425e-18	EpC2	CCT2
SDC1.2	1.1740501562411e-22	0.274803204368054	0.507	0.35	2.3535009432009e-18	EpC2	SDC1

CTGF.2	1.38086543118497e-22	0.319195681519907	0.288	0.161	2.76808284335339e-18	EpC2	CTGF
DDT.1	2.34055982699622e-22	-0.251828873	0.036	0.154	4.69188622919662e-18	EpC2	DDT
UTRN.1	2.35087400003607e-22	-0.334394479	0.241	0.396	4.7125620204723e-18	EpC2	UTRN
PARP1.1	2.80546386960352e-22	-0.282333178	0.178	0.328	5.62383287300721e-18	EpC2	PARP1
HNRNPR.1	3.21166716147534e-22	-0.358240569	0.4	0.53	6.43810799189346e-18	EpC2	HNRNPR
KLK7.1	4.47051193434727e-22	0.3562063960315	0.122	0.046	8.96158822359254e-18	EpC2	KLK7
ESD	4.48867586916662e-22	-0.319057645	0.293	0.449	8.9979996473314e-18	EpC2	ESD
HSP90AA1.1	4.52765908533041e-22	0.301948438576117	0.946	0.92	9.07614540245335e-18	EpC2	HSP90AA1
BGN.2	5.73336330678383e-22	-0.271765632	0.092	0.228	1.14931000847789e-17	EpC2	BGN
PHB.1	6.35090611041458e-22	-0.274807874	0.081	0.214	1.27310263889371e-17	EpC2	PHB
GPX4	6.77312106146615e-22	-0.368352248	0.596	0.672	1.3577398479815e-17	EpC2	GPX4
DEGS1	8.01271744356169e-22	-0.288603723	0.273	0.437	1.60622933873638e-17	EpC2	DEGS1
PAICS.1	8.07514948887276e-22	-0.275218069	0.156	0.303	1.61874446653943e-17	EpC2	PAICS
TUBA1C.3	9.26917566492029e-22	-0.379978722	0.072	0.2	1.85809895378992e-17	EpC2	TUBA1C
EXOSC8.1	9.99952827883824e-22	-0.291351458	0.17	0.316	2.00450543877591e-17	EpC2	EXOSC8
DSC3	1.47801665375072e-21	-0.33876565	0.623	0.72	2.9628321841087e-17	EpC2	DSC3
EEF1E1.1	1.58859253942252e-21	-0.262623067	0.164	0.311	3.18449260452638e-17	EpC2	EEF1E1
SNRPF.1	2.05100910792348e-21	-0.310203044	0.369	0.519	4.11145285774341e-17	EpC2	SNRPF
ABLIM1.3	2.89209043505465e-21	-0.274929004	0.224	0.385	5.79748448611055e-17	EpC2	ABLIM1
TNC.1	2.97162050484486e-21	-0.258767906	0.057	0.179	5.956910464012e-17	EpC2	TNC
CALD1	3.0475850124331e-21	-0.305264845	0.206	0.361	6.1091889159234e-17	EpC2	CALD1
ATP5MF.2	3.58073154783539e-21	-0.303218287	0.77	0.807	7.17793446079083e-17	EpC2	ATP5MF
TFAM	4.43010169733487e-21	-0.309399194	0.195	0.341	8.88058186247748e-17	EpC2	TFAM
ATP5MD.2	4.4429901222072e-21	-0.335583952	0.695	0.737	8.90641799897655e-17	EpC2	ATP5MD
SEPT11	8.75783693458172e-21	-0.270902855	0.132	0.268	1.75559599190625e-16	EpC2	SEPT11
CYR61.2	9.55906350842265e-21	-0.459235295	0.213	0.356	1.9162098708984e-16	EpC2	CYR61
RSL24D1	1.00693438372579e-20	-0.305060888	0.325	0.467	2.01850066561671e-16	EpC2	RSL24D1
EIF4B	1.03366721760987e-20	-0.324492514	0.315	0.453	2.07208930442075e-16	EpC2	EIF4B
TMPO.2	1.59461295912616e-20	-0.365650661	0.199	0.334	3.1965611378643e-16	EpC2	TMPO

FDPS.3	1.83519164542169e-20	-0.312590569	0.09	0.217	3.67882517241231e-16	EpC2	FDPS
MTCO1P12	1.95334507076273e-20	-0.289025413	0.386	0.521	3.91567552885096e-16	EpC2	MTCO1P12
CENPF.3	2.28926394563102e-20	-0.534990246	0.027	0.129	4.58905850541194e-16	EpC2	CENPF
NOP58.1	2.5020376515189e-20	-0.320574614	0.342	0.485	5.01558467623478e-16	EpC2	NOP58
MKI67.3	2.58212881293051e-20	-0.430837789	0.026	0.127	5.1761354184005e-16	EpC2	MKI67
RRM1.2	2.69538997701705e-20	-0.310228973	0.179	0.317	5.40317874792839e-16	EpC2	RRM1
SLIRP.3	3.34271679067593e-20	-0.333413035	0.514	0.62	6.70081007858897e-16	EpC2	SLIRP
TOP2A.3	4.25151430086519e-20	-0.592312518	0.017	0.113	8.52258556751435e-16	EpC2	TOP2A
SPTLC2.1	4.36589754636227e-20	-0.257936372	0.195	0.34	8.75187822143781e-16	EpC2	SPTLC2
UBXN4	5.85335989601949e-20	-0.328880092	0.549	0.636	1.17336452475607e-15	EpC2	UBXN4
PTMAP2.1	5.89645884293392e-20	-0.273019402	0.252	0.394	1.18200413965453e-15	EpC2	PTMAP2
TRMT112	6.48822641449594e-20	-0.312884525	0.547	0.636	1.30062986704986e-15	EpC2	TRMT112
PTN.1	6.51187500090942e-20	-0.36856539	0.361	0.517	1.3053704626823e-15	EpC2	PTN
EPHB6.2	7.68352908569016e-20	-0.301522831	0.166	0.305	1.54024024051745e-15	EpC2	EPHB6
KRT19.3	8.55842682642222e-20	0.465567505048082	0.731	0.701	1.7156222416246e-15	EpC2	KRT19
NDUFB10	8.61576278312476e-20	-0.31791724	0.442	0.561	1.72711580750519e-15	EpC2	NDUFB10
CCT5.2	1.13835007376145e-19	-0.334173243	0.299	0.432	2.28193655786221e-15	EpC2	CCT5
TRA2B.1	1.58424339980216e-19	-0.284387808	0.402	0.544	3.17577431924341e-15	EpC2	TRA2B
THOC2	1.75900934125434e-19	-0.318644353	0.322	0.458	3.52611012547844e-15	EpC2	THOC2
OST4.1	1.77774460914398e-19	-0.319891505	0.508	0.602	3.56366684349003e-15	EpC2	OST4
GADD45B.2	2.24286355411468e-19	0.310414569260804	0.733	0.571	4.49604428057829e-15	EpC2	GADD45B
CASC19	2.27718292478365e-19	-0.267073756	0.049	0.159	4.56484089102131e-15	EpC2	CASC19
LSM7.1	2.5771165701916e-19	-0.30852535	0.375	0.51	5.16608787660609e-15	EpC2	LSM7
NDUFS4.1	2.61991121053899e-19	-0.307395344	0.422	0.552	5.25187401264646e-15	EpC2	NDUFS4
HHIP.2	3.51175261463461e-19	-0.362236538	0.05	0.158	7.03965929129655e-15	EpC2	HHIP
MACF1	3.54543532983736e-19	-0.294665038	0.237	0.375	7.10717966219198e-15	EpC2	MACF1
PCLAF.3	4.24798823966435e-19	-0.27666729	0.018	0.111	8.51551722523116e-15	EpC2	PCLAF
LIMA1	4.44573849480155e-19	-0.353861316	0.387	0.51	8.9119273866792e-15	EpC2	LIMA1
FAM96B.2	4.52270836871979e-19	-0.333013177	0.357	0.485	9.06622119593569e-15	EpC2	FAM96B

RGS2	5.79211744679033e-19	0.27848605690933	0.288	0.172	1.16108786338359e-14	EpC2	RGS2
APRT.2	6.91962128453458e-19	-0.340907691	0.517	0.608	1.3871072826978e-14	EpC2	APRT
F2R	9.71532687679979e-19	-0.256866158	0.185	0.328	1.94753442572329e-14	EpC2	F2R
AL450405.1.1	1.07838679037037e-18	-0.310854169	0.241	0.401	2.16173415997645e-14	EpC2	AL450405.1
BIRC5.3	1.22050771805173e-18	-0.267116203	0.013	0.1	2.4466297716065e-14	EpC2	BIRC5
SIVA1.1	1.92723786796042e-18	-0.290805133	0.199	0.33	3.86334103011345e-14	EpC2	SIVA1
MDH2.1	2.02843994633684e-18	-0.298766493	0.533	0.635	4.06621071642684e-14	EpC2	MDH2
HMGB3.2	2.05507778178769e-18	-0.317407314	0.236	0.373	4.1196089213716e-14	EpC2	HMGB3
RPL7L1	2.2868458486772e-18	-0.269462366	0.25	0.387	4.58421118825832e-14	EpC2	RPL7L1
PSMA7.3	2.41447550169312e-18	-0.294043189	0.976	0.98	4.84005759069404e-14	EpC2	PSMA7
SERPINB11.3	2.48419447613325e-18	0.319871661822486	0.223	0.122	4.97981624685672e-14	EpC2	SERPINB11
DYNC1H1	2.88695923735222e-18	-0.272783395	0.267	0.401	5.78719848719625e-14	EpC2	DYNC1H1
KRT6B.3	3.65882260598517e-18	-0.286350237	0.578	0.365	7.33447579595788e-14	EpC2	KRT6B
COX6C.2	4.09772068002395e-18	-0.26753065	0.952	0.963	8.21429087517601e-14	EpC2	COX6C
ISOC2	4.172833214657e-18	-0.255701127	0.191	0.325	8.36486146210142e-14	EpC2	ISOC2
LARS	5.00251870837384e-18	-0.293308981	0.279	0.409	1.00280490028062e-13	EpC2	LARS
SLC25A3	5.52447467281055e-18	-0.289403999	0.642	0.701	1.1074361929116e-13	EpC2	SLC25A3
UQCRB.1	7.22062386271474e-18	-0.273962636	0.789	0.819	1.4474462595198e-13	EpC2	UQCRB
SSRP1.1	1.06766420096857e-17	-0.251717999	0.183	0.314	2.1402396572616e-13	EpC2	SSRP1
TOMM20	1.49122519290959e-17	-0.265956016	0.473	0.586	2.98931002170656e-13	EpC2	TOMM20
TMEM94.2	1.64514881747935e-17	-0.272712447	0.072	0.181	3.2978653195191e-13	EpC2	TMEM94
NDUFA10.1	2.10099434474709e-17	-0.280703322	0.411	0.533	4.21165326348002e-13	EpC2	NDUFA10
ODC1.2	2.22026790173749e-17	-0.285705077	0.356	0.519	4.45074903582297e-13	EpC2	ODC1
CCT4.1	2.63533274647344e-17	-0.28905673	0.304	0.431	5.28278802358066e-13	EpC2	CCT4
CCNB1.3	2.68543446250471e-17	-0.407582779	0.028	0.118	5.38322192353695e-13	EpC2	CCNB1
EIF3A	3.92748468613847e-17	-0.299045179	0.535	0.62	7.87303580183317e-13	EpC2	EIF3A
CAVIN1	3.99400138171841e-17	-0.265668968	0.223	0.353	8.00637516979272e-13	EpC2	CAVIN1
SRSF10.1	4.06034950837078e-17	-0.294578328	0.301	0.427	8.13937662448007e-13	EpC2	SRSF10
TTC3	4.08662243031597e-17	-0.261809686	0.225	0.359	8.1920433238114e-13	EpC2	TTC3

EIF3D	4.23716373857306e-17	-0.262553091	0.305	0.436	8.49381843034356e-13	EpC2	EIF3D
SF3B5	4.28450485124964e-17	-0.305758286	0.537	0.624	8.58871842481502e-13	EpC2	SF3B5
ALDH1A1.2	4.64858475819322e-17	-0.271487203	0.076	0.183	9.31855300627414e-13	EpC2	ALDH1A1
SUPT16H.1	4.71719170161969e-17	-0.294741561	0.249	0.379	9.45608248506683e-13	EpC2	SUPT16H
TSPO.3	8.08806190775614e-17	-0.299807412	0.797	0.831	1.6213328900288e-12	EpC2	TSPO
NQO1.2	1.05326883836348e-16	-0.386106937	0.318	0.433	2.11138271338343e-12	EpC2	NQO1
ANP32A	1.65746987688286e-16	-0.273666971	0.352	0.469	3.32256411519938e-12	EpC2	ANP32A
NME1.2	2.08292602376839e-16	-0.287467119	0.368	0.488	4.17543350724612e-12	EpC2	NME1
SSBP1.1	2.39535093257508e-16	-0.283084583	0.525	0.619	4.80172047944001e-12	EpC2	SSBP1
MRPL20.1	3.42417033010985e-16	-0.253780026	0.262	0.386	6.86409184373821e-12	EpC2	MRPL20
SEPT10	4.05891466960377e-16	-0.275804039	0.296	0.415	8.13650034668771e-12	EpC2	SEPT10
LGALS7.2	4.67450799134116e-16	-0.394608601	0.108	0.223	9.37051871944248e-12	EpC2	LGALS7
AGR2	5.50044981461954e-16	-0.311883384	0.251	0.382	1.10262016983863e-11	EpC2	AGR2
TPGS2.1	6.47170901160049e-16	-0.255477655	0.289	0.415	1.29731878846543e-11	EpC2	TPGS2
U2SURP	7.05080464089091e-16	-0.297989124	0.327	0.444	1.41340429831299e-11	EpC2	U2SURP
C11orf58	8.36381402069537e-16	-0.257113247	0.701	0.743	1.67661015858859e-11	EpC2	C11orf58
PTGES3.1	9.23516247694829e-16	-0.278783326	0.486	0.579	1.85128067012905e-11	EpC2	PTGES3
SF3B6.1	9.35193219666026e-16	-0.286756752	0.627	0.676	1.87468832814252e-11	EpC2	SF3B6
DYNLL1.2	1.42110567534081e-15	-0.282826653	0.871	0.928	2.84874843678819e-11	EpC2	DYNLL1
PSMA4.1	1.43637578853459e-15	-0.302519347	0.592	0.642	2.87935890569643e-11	EpC2	PSMA4
PCM1.1	1.52590309083935e-15	-0.276546135	0.232	0.35	3.05882533589655e-11	EpC2	PCM1
HNRNPH1.1	1.83565975684417e-15	-0.25232185	0.304	0.426	3.67976354856983e-11	EpC2	HNRNPH1
KRT8.1	2.24891334613328e-15	-0.251939803	0.357	0.498	4.50817169365877e-11	EpC2	KRT8
HSPA9	3.04382999621936e-15	-0.276288975	0.444	0.546	6.10166161042133e-11	EpC2	HSPA9
NDUFAB1.2	3.0520297263477e-15	-0.31538515	0.538	0.591	6.1180987894366e-11	EpC2	NDUFAB1
CWC15.1	3.19865495709547e-15	-0.301959945	0.404	0.511	6.41202372699359e-11	EpC2	CWC15
GPNMB.2	3.65194290403746e-15	-0.593802944	0.15	0.259	7.3206847454335e-11	EpC2	GPNMB
HNRNPU.1	3.87146532247958e-15	-0.268458972	0.378	0.488	7.76073938544256e-11	EpC2	HNRNPU
SETX	4.52302710410959e-15	0.335308418494032	0.313	0.208	9.06686013289808e-11	EpC2	SETX

BEX3.1	5.56482417565435e-15	-0.349490155	0.463	0.538	1.11552465425167e-10	EpC2	BEX3
MAGED2.1	5.88181835003523e-15	-0.270647727	0.27	0.389	1.17906930644806e-10	EpC2	MAGED2
TMEM123	6.48105155109346e-15	-0.260281291	0.317	0.445	1.29919159393219e-10	EpC2	TMEM123
NUSAP1.3	8.0246236325405e-15	-0.586489697	0.049	0.135	1.60861605337907e-10	EpC2	NUSAP1
POLR2E.2	1.17581582380043e-14	-0.258279177	0.323	0.436	2.35704040039033e-10	EpC2	POLR2E
NR4A1.2	1.23952792875364e-14	-0.280635425	0.137	0.25	2.48475768597954e-10	EpC2	NR4A1
TPX2.3	1.69549790244646e-14	-0.317404226	0.03	0.108	3.39879509524418e-10	EpC2	TPX2
EWSR1.1	1.76299514596504e-14	-0.258230779	0.361	0.468	3.53410006960151e-10	EpC2	EWSR1
FOS.3	2.38010532763915e-14	-0.28718669	0.923	0.947	4.77115913978543e-10	EpC2	FOS
GSTA1.3	2.57468368188114e-14	-0.789086022	0.25	0.374	5.16121090869894e-10	EpC2	GSTA1
C19orf70.1	2.63156658239266e-14	-0.269381591	0.358	0.468	5.27523837106433e-10	EpC2	C19orf70
CCT3	4.0058460946556e-14	-0.25732465	0.385	0.49	8.03011908134661e-10	EpC2	CCT3
G3BP1.1	5.24656521564346e-14	-0.26325993	0.365	0.47	1.05172646312789e-09	EpC2	G3BP1
PPP1CA.2	5.94523186191814e-14	-0.270518056	0.213	0.32	1.19178117904011e-09	EpC2	PPP1CA
PSME1	7.01645556818074e-14	-0.255142488	0.463	0.554	1.40651868319751e-09	EpC2	PSME1
UFD1	7.65059729234137e-14	-0.263484983	0.304	0.409	1.53363873322275e-09	EpC2	UFD1
RBBP6	8.02121751625931e-14	0.429448855234041	0.476	0.372	1.60793326330934e-09	EpC2	RBBP6
ZFR	8.03160449611759e-14	-0.256949987	0.347	0.458	1.61001543729173e-09	EpC2	ZFR
BUB3.2	8.22445163585396e-14	-0.277409228	0.28	0.386	1.64867357492328e-09	EpC2	BUB3
TIMM13.1	1.129996375563e-13	-0.270857056	0.414	0.513	2.2651907344536e-09	EpC2	TIMM13
POLR1D	1.54629222129278e-13	-0.265381364	0.532	0.6	3.09969738680351e-09	EpC2	POLR1D
XRCC6.1	1.65485783903304e-13	-0.255966786	0.383	0.479	3.31732802412563e-09	EpC2	XRCC6
PSMA1.1	2.61212990692151e-13	-0.251601167	0.372	0.469	5.23627561141486e-09	EpC2	PSMA1
TPR.1	2.63475419803462e-13	-0.274666234	0.343	0.448	5.28162826538019e-09	EpC2	TPR
SMC3.1	2.96800906666416e-13	-0.280215757	0.284	0.384	5.94967097503497e-09	EpC2	SMC3
CALM2.2	4.20662223347194e-13	-0.306408879	0.871	0.866	8.43259492921785e-09	EpC2	CALM2
SELENOW.1	4.68568830491048e-13	-0.26283709	0.836	0.849	9.39293077602355e-09	EpC2	SELENOW
CCT8.1	4.94568215394291e-13	-0.254265831	0.362	0.457	9.91411444579395e-09	EpC2	CCT8
LSM3.2	5.78255750309918e-13	-0.286556226	0.382	0.475	1.15917147707126e-08	EpC2	LSM3

TXN2	6.17884071719155e-13	-0.262810084	0.358	0.454	1.23861041016822e-08	EpC2	TXN2
IFI6	6.59120318291952e-13	0.26155537013447	0.153	0.084	1.32127259004805e-08	EpC2	IFI6
ATP5IF1.1	9.33433726469951e-13	-0.269176755	0.62	0.66	1.87116124808166e-08	EpC2	ATP5IF1
COPS9.2	1.10965994879243e-12	-0.280053746	0.558	0.61	2.2244243333493e-08	EpC2	COPS9
SUCLG1.3	1.24234169494977e-12	-0.262368997	0.735	0.737	2.4903981616963e-08	EpC2	SUCLG1
SNRPG.2	1.30208760819978e-12	-0.29969058	0.639	0.65	2.61016481939728e-08	EpC2	SNRPG
DIRAS3.2	1.45496465206909e-12	-0.285027785	0.13	0.229	2.91662214153769e-08	EpC2	DIRAS3
ATRX	1.77788061200222e-12	-0.257007043	0.348	0.45	3.56393947481965e-08	EpC2	ATRX
PCNA.2	3.62499508893616e-12	-0.273024637	0.178	0.276	7.26666515528142e-08	EpC2	PCNA
AKAP9.1	3.73477411574487e-12	-0.29950031	0.422	0.507	7.48672819242216e-08	EpC2	AKAP9
SNRPD3.1	4.28707633122308e-12	-0.260303172	0.595	0.631	8.59387321356978e-08	EpC2	SNRPD3
RTN3	7.29410443059281e-12	-0.258725014	0.388	0.47	1.46217617415663e-07	EpC2	RTN3
WASF2	7.87334396566881e-12	-0.25070299	0.436	0.522	1.57829053135797e-07	EpC2	WASF2
DYNLT3.3	7.92554545032486e-12	-0.448931389	0.559	0.583	1.58875484097212e-07	EpC2	DYNLT3
HMGB2.3	1.05145691134332e-11	-0.56214501	0.192	0.276	2.10775052447882e-07	EpC2	HMGB2
C5orf46.2	1.33827164774182e-11	-0.388108656	0.068	0.147	2.68269934506326e-07	EpC2	C5orf46
CKAP2.3	1.91107064943961e-11	-0.351753857	0.156	0.247	3.83093222386663e-07	EpC2	CKAP2
PDLIM1.2	2.08267666052412e-11	-0.284067897	0.529	0.585	4.17493363368666e-07	EpC2	PDLIM1
SOX4.3	2.33163729432763e-11	-0.26272303	0.693	0.776	4.67400012020917e-07	EpC2	SOX4
CENPX.2	3.70961778111394e-11	-0.257169296	0.251	0.345	7.436299804021e-07	EpC2	CENPX
TUBB4B.2	8.13826236150580e-11	-0.269745002	0.3	0.386	1.63139607298745e-06	EpC2	TUBB4B
CLIC1.3	1.24337963420346e-10	-0.251563933	0.756	0.73	2.49247881472425e-06	EpC2	CLIC1
MT1X.2	4.1803750108192e-10	0.329200976195254	0.771	0.731	8.37997974668816e-06	EpC2	MT1X
LY6G6C.3	1.41966457353758e-09	-0.303837786	0.093	0.169	2.84585960411344e-05	EpC2	LY6G6C
ITGB1.2	4.98707688846549e-09	-0.279528481	0.444	0.493	9.99709433061793e-05	EpC2	ITGB1
TSC22D1.1	8.37058560679422e-09	0.256899087876988	0.674	0.606	0.000167796759073797	EpC2	TSC22D1
AHNAK	2.92798588956866e-08	-0.261599419	0.623	0.644	0.000586944051422933	EpC2	AHNAK
PTTG1.2	3.02203872083118e-08	-0.4825195	0.263	0.333	0.000605797881977818	EpC2	PTTG1
ANAPC11.2	4.51220042662179e-08	-0.252451127	0.585	0.601	0.000904515697520605	EpC2	ANAPC11

GBP6.3	6.00706229756221e-08	-0.494380932	0.409	0.46	0.00120417570816932	EpC2	GBP6
HIST1H1D.3	9.67171762774891e-08	-0.301116092	0.068	0.125	0.00193879251565855	EpC2	HIST1H1D
HIST1H1E.2	2.92418822878765e-06	-0.312399366	0.292	0.353	0.0586182772342773	EpC2	HIST1H1E
PRDX1.3	3.23646958822276e-06	-0.333838903	0.736	0.719	0.0648782693655134	EpC2	PRDX1
KRTDAP.3	0.000118215380524456	-0.864596688	0.165	0.216	1	EpC2	KRTDAP
TXN.2	0.000189365353190814	-0.264885801	0.985	0.992	1	EpC2	TXN
ARL6IP1.3	0.00659599423908473	-0.331118207	0.429	0.432	1	EpC2	ARL6IP1
ODAM.1	0	2.99969800551685	0.522	0.026	0	EpC5	ODAM
MMP13	0	1.99035689791339	0.489	0.021	0	EpC5	MMP13
MMP7	1.13748026924586e-282	1.41358850273224	0.397	0.024	2.28019294773025e-278	EpC5	MMP7
ANGPTL7	2.48702120915273e-269	2.5208094090777	0.273	0.009	4.98548271586757e-265	EpC5	ANGPTL7
GAD2	1.19358236708791e-268	0.887407399286485	0.205	0.003	2.39265521306443e-264	EpC5	GAD2
CFH.4	8.53593274771474e-257	1.4900625979895	0.638	0.088	1.7111130786069e-252	EpC5	CFH
SAA1.4	2.53228356479716e-232	2.20019165318025	0.567	0.076	5.07621563399238e-228	EpC5	SAA1
KRT16P6	2.1464966277572e-228	1.1262284618703	0.365	0.027	4.30286714000208e-224	EpC5	KRT16P6
BHLHE41	9.31889495393125e-228	0.50348611772967	0.304	0.016	1.86806568246506e-223	EpC5	BHLHE41
CLEC2B.4	4.68896233032859e-215	2.50882338203529	0.62	0.104	9.3994938873767e-211	EpC5	CLEC2B
MMP10	1.73695204057474e-202	0.999403774694591	0.208	0.007	3.48189406053613e-198	EpC5	MMP10
SLC1A3	2.42695691917869e-200	0.592146090425062	0.385	0.036	4.8650778401856e-196	EpC5	SLC1A3
DCN.4	7.11451910158315e-192	2.30187835819249	0.747	0.183	1.42617649910336e-187	EpC5	DCN
KRT17.4	2.37158622706932e-184	-2.817151804	0.767	0.99	4.75408175078316e-180	EpC5	KRT17
TSPAN7	4.68366711656119e-183	0.786026173299993	0.418	0.049	9.38887910185857e-179	EpC5	TSPAN7
MMP12.1	9.40784081338629e-181	2.00642319807538	0.316	0.026	1.88589576945142e-176	EpC5	MMP12
CLDN1.3	6.57996381891904e-180	1.18982811386153	0.701	0.154	1.31901954714051e-175	EpC5	CLDN1
CSF3.2	3.04114503397868e-177	1.26281954321654	0.413	0.048	6.09627933511366e-173	EpC5	CSF3
ADAM28.1	1.11369526568058e-170	0.763314747855345	0.405	0.049	2.23251352958329e-166	EpC5	ADAM28
TNFSF10.4	4.14745569019377e-167	2.07839351829973	0.909	0.44	8.31398967656243e-163	EpC5	TNFSF10
FDCSP.4	5.60711259588896e-161	3.80720316887712	0.691	0.189	1.1240017909719e-156	EpC5	FDCSP
CYP24A1.1	8.07092210982145e-151	0.612003426043179	0.362	0.043	1.61789704613481e-146	EpC5	CYP24A1

CLEC7A.1	2.73919535566283e-149	0.552453531875813	0.37	0.047	5.49099100996171e-145	EpC5	CLEC7A
SAA2.1	1.74652163763057e-146	0.859550404775079	0.334	0.037	3.50107727479424e-142	EpC5	SAA2
TNFRSF21.1	3.34342948527456e-143	0.864562207348312	0.557	0.116	6.70223874618138e-139	EpC5	TNFRSF21
COL6A6	3.159183781603e-142	0.278283803713824	0.114	0.002	6.33289980860138e-138	EpC5	COL6A6
B2M.3	5.15794907773408e-136	1.57513359182079	0.997	0.976	1.03396247212257e-131	EpC5	B2M
SLC7A11	1.39461084848867e-134	0.563108399936376	0.304	0.034	2.79563690688039e-130	EpC5	SLC7A11
PDZK1IP1.4	1.16873244709267e-133	1.05366269027694	0.582	0.121	2.34284106344197e-129	EpC5	PDZK1IP1
MMP28	1.11342829147668e-131	0.273060619905766	0.185	0.011	2.23197835309416e-127	EpC5	MMP28
BIRC3	1.61660620146019e-129	0.861867108549939	0.332	0.043	3.2406487914471e-125	EpC5	BIRC3
SAMSN1	4.52887225476271e-129	0.449938381989845	0.172	0.009	9.07857732189732e-125	EpC5	SAMSN1
IFI16.3	7.55217218638261e-127	1.15096109904002	0.828	0.332	1.51390843648226e-122	EpC5	IFI16
FAM20A	4.34998674832646e-125	0.321984723705397	0.253	0.024	8.71998343569522e-121	EpC5	FAM20A
PAPPA.1	4.40413329292708e-123	0.528874384523225	0.309	0.039	8.82852559900162e-119	EpC5	PAPPA
CHI3L2.1	1.29547260110401e-119	0.526923494396627	0.243	0.024	2.59690437617311e-115	EpC5	CHI3L2
TMEM45A.3	4.22046783267274e-117	0.881292800373677	0.451	0.09	8.46034981737577e-113	EpC5	TMEM45A
DEPDC7	8.03562564754336e-116	0.541701441375839	0.347	0.053	1.61082151730654e-111	EpC5	DEPDC7
LAMB3.3	4.6283764992708e-115	1.31625974796411	0.797	0.349	9.27804353043825e-111	EpC5	LAMB3
CXCL1.4	1.1224309034607e-114	2.44182468571682	0.572	0.153	2.25002498907731e-110	EpC5	CXCL1
KYNU.2	7.10044472058596e-113	0.720051668607189	0.327	0.048	1.42335514868866e-108	EpC5	KYNU
CYP27A1	9.79002957464635e-111	0.253307724823685	0.172	0.012	1.96250932853361e-106	EpC5	CYP27A1
LAMC2.3	1.27578134233859e-110	1.0760599647347	0.522	0.131	2.55743127885194e-106	EpC5	LAMC2
LAMA3.1	9.46734214016998e-109	1.22959593409484	0.628	0.194	1.89782340541847e-104	EpC5	LAMA3
PITX2.2	1.28153200271941e-108	0.811860882385748	0.643	0.199	2.56895905265133e-104	EpC5	PITX2
KRT42P.2	3.06431897121686e-107	-1.706676569	0.106	0.724	6.14273380970131e-103	EpC5	KRT42P
APOL1	3.36199491385723e-106	0.3822894857259	0.281	0.037	6.7394550043182e-102	EpC5	APOL1
GPX2.4	1.62032092585171e-105	-1.771596647	0.116	0.718	3.24809532796234e-101	EpC5	GPX2
S100A9.4	3.41609003041172e-105	1.64285895016096	0.646	0.192	6.84789407496333e-101	EpC5	S100A9
LUM.3	2.2753663076785e-102	1.20216175334291	0.516	0.139	4.56119930037232e-98	EpC5	LUM
NRCAM	2.92336669989519e-102	0.448162852868674	0.284	0.039	5.86018088660989e-98	EpC5	NRCAM

ASS1.3	2.03008040363876e-100	0.779519703794863	0.539	0.141	4.06949917713425e-96	EpC5	ASS1
NFE2L2.2	3.1983881693124e-100	-1.310264088	0.59	0.919	6.41148892420364e-96	EpC5	NFE2L2
HAS2.1	1.66683277839814e-98	0.392541547250044	0.256	0.033	3.3413329875769e-94	EpC5	HAS2
CPE	2.34736914527272e-96	0.51057468611651	0.203	0.021	4.70553618861369e-92	EpC5	CPE
FAM46A.2	2.56672053617126e-96	0.74180973748618	0.451	0.108	5.1452479868089e-92	EpC5	FAM46A
EPAS1	2.10865253624152e-95	0.541194005248037	0.304	0.049	4.22700487414975e-91	EpC5	EPAS1
TGFBR2	3.24211981629873e-94	0.393989133244423	0.311	0.051	6.49915338375244e-90	EpC5	TGFBR2
TMEM176A	4.7267155952988e-94	0.289620858207745	0.157	0.012	9.47517408233597e-90	EpC5	TMEM176A
NNMT	1.3594017783981e-93	0.379690161458167	0.241	0.031	2.72505680497684e-89	EpC5	NNMT
TDO2	3.20135328965469e-92	0.360784248119303	0.144	0.01	6.4174328044418e-88	EpC5	TDO2
NCOA7.3	1.11449330196737e-90	1.50337937776176	0.638	0.243	2.23411327312379e-86	EpC5	NCOA7
S100A8.4	1.3125078405719e-88	1.54479900158988	0.643	0.222	2.63105321721043e-84	EpC5	S100A8
TMPRSS11A.3	2.63190773204808e-88	1.10850437657677	0.633	0.213	5.27592223966358e-84	EpC5	TMPRSS11A
DBI.3	4.05236183019817e-88	-1.668746622	0.529	0.864	8.12336452481526e-84	EpC5	DBI
GCLC	8.84022239958497e-88	0.677689809401436	0.514	0.149	1.7721109822208e-83	EpC5	GCLC
MMP1	9.03002826847408e-87	0.582240236362264	0.162	0.014	1.81015946669831e-82	EpC5	MMP1
TMEM176B	9.13863964547026e-87	0.345406362866259	0.165	0.015	1.83193170333097e-82	EpC5	TMEM176B
C1S	3.77159336978354e-86	0.329369659040082	0.233	0.031	7.56053606906808e-82	EpC5	C1S
CXCL8.4	7.02988298812799e-86	1.49926350197542	0.613	0.209	1.40921034380014e-81	EpC5	CXCL8
HLA-C.2	1.92584246425441e-85	0.78138504777884	0.663	0.25	3.86054380384439e-81	EpC5	HLA-C
SPARCL1.2	8.14210737936222e-85	2.43248729735144	0.352	0.081	1.63216684526695e-80	EpC5	SPARCL1
IQGAP2	4.3692349764759e-84	0.439085989367396	0.17	0.016	8.7585684338436e-80	EpC5	IQGAP2
BST2	8.34567577332453e-84	0.372924542734063	0.21	0.026	1.67297416552064e-79	EpC5	BST2
OAS2	1.43966025732551e-83	0.286454394799991	0.22	0.029	2.88594295183472e-79	EpC5	OAS2
BMP2.1	1.80468526576612e-83	0.488883578126331	0.286	0.049	3.61767208375476e-79	EpC5	BMP2
DAB2	1.44690903011571e-81	0.253248747511487	0.165	0.016	2.90047384176995e-77	EpC5	DAB2
EHF.3	5.27083282032033e-81	0.861359996192815	0.765	0.344	1.05659114716141e-76	EpC5	EHF
PABPC1.1	2.40924334161354e-80	-0.717782222	1	1	4.82956920259849e-76	EpC5	PABPC1
RPS18.2	5.64265487499094e-80	-0.820304041	0.962	0.996	1.13112659624068e-75	EpC5	RPS18

CYP1B1	1.00059751783941e-77	0.4152174088739	0.258	0.042	2.00579778426088e-73	EpC5	CYP1B1
CXCL2.2	3.23724398329496e-77	0.786092699862805	0.387	0.092	6.48937928891307e-73	EpC5	CXCL2
SOD2.4	1.00801655552891e-76	1.40627825623513	0.643	0.297	2.02066998721325e-72	EpC5	SOD2
C1R	1.53961991288685e-76	0.625605816652894	0.327	0.07	3.08632207737297e-72	EpC5	C1R
TYMP.2	2.0419409188686e-76	0.656016067938476	0.489	0.147	4.09327476596399e-72	EpC5	TYMP
HLA-B.2	3.05048648769817e-76	0.822641152980537	0.582	0.216	6.11500521323975e-72	EpC5	HLA-B
EPCAM.3	2.12903193639909e-75	-1.374463346	0.122	0.623	4.26785741970561e-71	EpC5	EPCAM
CXCL6.2	9.13317531427716e-75	0.741048627155917	0.276	0.05	1.8308363235e-70	EpC5	CXCL6
PHLDA1.4	1.3859170287014e-74	0.56682573010664	0.552	0.17	2.77820927573483e-70	EpC5	PHLDA1
CXCL3.4	1.59249981329875e-73	1.15385958084749	0.352	0.082	3.19232512573868e-69	EpC5	CXCL3
PLEK.1	5.51548379166234e-72	0.372881231183194	0.246	0.041	1.10563388087663e-67	EpC5	PLEK
RPL23A.2	6.63480868906195e-72	-1.011760647	0.803	0.945	1.33001374980936e-67	EpC5	RPL23A
MXD1.4	4.36713548248516e-71	0.543765068296095	0.496	0.15	8.75435978818976e-67	EpC5	MXD1
ETS2.2	6.85389216067705e-71	0.824392667212145	0.63	0.256	1.37393122252932e-66	EpC5	ETS2
LRRK2	8.20011206272197e-71	0.432264231420892	0.243	0.042	1.64379446409325e-66	EpC5	LRRK2
IFITM3.1	8.55416101315387e-71	1.37603514624363	0.899	0.662	1.71476711669682e-66	EpC5	IFITM3
SLC25A5.4	1.99650080038821e-70	-1.114783592	0.676	0.909	4.0021855044582e-66	EpC5	SLC25A5
IFIT1	3.12194547542986e-70	0.335116341760125	0.19	0.025	6.2582519000467e-66	EpC5	IFIT1
IFI44	2.56324832053202e-69	0.290650224584779	0.187	0.025	5.13828758333848e-65	EpC5	IFI44
RSAD2.1	2.83840096657886e-69	0.453181914666387	0.22	0.034	5.68985857760399e-65	EpC5	RSAD2
PLAUR.4	4.44394537552215e-69	0.928770607009084	0.522	0.179	8.90833289977171e-65	EpC5	PLAUR
RPL29.1	1.06026289483586e-68	-0.769663844	0.881	0.985	2.12540299898797e-64	EpC5	RPL29
AKR1C3.4	1.4004757692014e-68	-1.618002162	0.167	0.618	2.80739372694112e-64	EpC5	AKR1C3
INHBA	1.58598549278127e-67	0.46198567926958	0.205	0.031	3.17926651882933e-63	EpC5	INHBA
FMO2.4	5.37995427981939e-67	0.749373690678428	0.463	0.139	1.0784656349326e-62	EpC5	FMO2
TGFB3	2.41532227218602e-66	0.334790357918754	0.192	0.028	4.84175502682409e-62	EpC5	TGFB3
OGN	1.48088796526284e-65	0.376284682427762	0.106	0.008	2.96858801516589e-61	EpC5	OGN
STMN1.3	4.19320991376014e-63	-1.3561369	0.215	0.651	8.40570859312359e-59	EpC5	STMN1
RPL18A.1	5.84454146425078e-63	-0.767413288	0.909	0.986	1.17159678192371e-58	EpC5	RPL18A

ZBED2	4.87327614033639e-62	0.751344826290582	0.425	0.135	9.76896935091832e-58	EpC5	ZBED2
FNDC3B.1	5.16707457623607e-62	0.40707243569803	0.41	0.119	1.03579176955228e-57	EpC5	FNDC3B
DSE.1	1.67212522704031e-61	0.377078396758817	0.306	0.072	3.35194223012501e-57	EpC5	DSE
TGM3.3	6.22841655769908e-61	-1.408711777	0.053	0.496	1.24854838315636e-56	EpC5	TGM3
RPS2.2	7.06699512469338e-61	-0.700106169	0.962	0.993	1.41664984269604e-56	EpC5	RPS2
RAB7B	8.16686537754602e-61	0.324036864784069	0.291	0.066	1.63712983358288e-56	EpC5	RAB7B
BASP1	1.31715281385208e-60	0.402724801197421	0.327	0.082	2.64036453064788e-56	EpC5	BASP1
SOX2.2	1.83971441945168e-59	-0.937673919	0.159	0.599	3.68789152523284e-55	EpC5	SOX2
CLCA4.4	1.99199118182456e-59	0.401895645640393	0.354	0.089	3.99314552308551e-55	EpC5	CLCA4
CPNE8.1	2.11908991990148e-59	0.390303669140354	0.322	0.081	4.2479276534345e-55	EpC5	CPNE8
ALDH3A1.3	5.54699089239162e-59	-1.334644125	0.223	0.649	1.11194979428882e-54	EpC5	ALDH3A1
COX6A1.3	1.77498745507737e-57	-0.794008694	0.752	0.919	3.5581398524481e-53	EpC5	COX6A1
PSMB9	3.06118538763062e-57	0.320597027153778	0.2	0.035	6.13645222804435e-53	EpC5	PSMB9
OMD	4.50796828500185e-57	0.275096271024857	0.137	0.016	9.03667322411471e-53	EpC5	OMD
TIPARP.3	2.59337607769652e-56	0.683256172619001	0.534	0.217	5.19868168535044e-52	EpC5	TIPARP
BHLHE40.3	3.01221514143035e-56	0.764649018799484	0.641	0.304	6.03828647251129e-52	EpC5	BHLHE40
IL20RB	4.95911325168837e-56	0.390178032548986	0.352	0.099	9.94103842433451e-52	EpC5	IL20RB
TXN.3	7.58069661197055e-56	-1.04910273	0.932	0.994	1.51962644283562e-51	EpC5	TXN
DLG2	3.65969236485406e-55	0.459816548725945	0.17	0.026	7.33621931458646e-51	EpC5	DLG2
RPS3A.2	4.14577883895225e-55	-0.724173391	0.919	0.983	8.31062826056368e-51	EpC5	RPS3A
SDR16C5.3	6.33205888943421e-55	0.444411444514558	0.446	0.144	1.26932452497598e-50	EpC5	SDR16C5
ITGA6.1	2.23340886098612e-54	0.678326465456603	0.646	0.294	4.47709140273278e-50	EpC5	ITGA6
CAPNS2.3	2.52641640120212e-54	-0.960442199	0.109	0.524	5.06445431784977e-50	EpC5	CAPNS2
CD74.1	2.59357529132628e-54	1.40583420743583	0.532	0.237	5.19908102899266e-50	EpC5	CD74
DAPL1.3	5.71743490499504e-54	0.862220867299578	0.597	0.272	1.14611700105531e-49	EpC5	DAPL1
NACA.2	5.79531727066989e-54	-0.677952843	0.863	0.974	1.16172930007849e-49	EpC5	NACA
S100A7.4	8.73897612163927e-54	-0.394718965	0.473	0.152	1.75181515334381e-49	EpC5	S100A7
SLC39A14	9.72032543407975e-53	0.342747352563975	0.268	0.063	1.94853643651563e-48	EpC5	SLC39A14
ID2.3	1.24166273376753e-52	1.08669811360725	0.772	0.5	2.4890371161104e-48	EpC5	ID2

LNX1.1	4.55217297568441e-52	0.375453939961058	0.37	0.111	9.12528594705697e-48	EpC5	LNX1
ODAPH.1	5.26518286235925e-52	1.40875081337267	0.203	0.038	1.05545855658854e-47	EpC5	ODAPH
SELENOM	5.47108752332033e-52	0.428097309896261	0.332	0.093	1.09673420492479e-47	EpC5	SELENOM
RPL8.1	1.41542904883293e-51	-0.612648425	0.934	0.99	2.83736907129049e-47	EpC5	RPL8
MTATP6P1.2	1.76707371210336e-51	-0.906664967	0.942	0.981	3.54227596328239e-47	EpC5	MTATP6P1
SRPX	3.02037221876854e-51	0.288495840938291	0.19	0.035	6.05463814974341e-47	EpC5	SRPX
MYH10	3.1363625175136e-51	0.423765345248203	0.342	0.101	6.28715230260777e-47	EpC5	MYH10
HTRA1	8.04558401201527e-51	0.263884630352504	0.18	0.031	1.61281777104858e-46	EpC5	HTRA1
RPS5.1	9.01606886226711e-51	-0.688960699	0.866	0.971	1.80736116413007e-46	EpC5	RPS5
ARNTL2.1	2.06720365868577e-50	0.356740682296245	0.291	0.076	4.14391645420149e-46	EpC5	ARNTL2
LPAR6.2	2.62549298276916e-50	0.710777809011186	0.595	0.273	5.26306323325905e-46	EpC5	LPAR6
DYNLL1.3	2.10407098204902e-49	-0.842473532	0.79	0.928	4.21782069061547e-45	EpC5	DYNLL1
STAT1	1.01939689274909e-48	0.481243162781304	0.395	0.135	2.04348301120483e-44	EpC5	STAT1
RPS6.2	1.23156770441717e-48	-0.591561709	0.962	0.991	2.46880062027466e-44	EpC5	RPS6
AKR1C2.4	5.52135549730896e-48	-1.351636263	0.106	0.48	1.10681092299055e-43	EpC5	AKR1C2
SAT1.3	1.39760533432946e-47	0.584034191182674	0.99	0.97	2.80163965319685e-43	EpC5	SAT1
FAM213A.1	1.68059559167979e-47	-0.778418473	0.524	0.812	3.3689219230813e-43	EpC5	FAM213A
IL1R2.3	5.93208267295848e-47	-1.133932681	0.119	0.504	1.18914529262126e-42	EpC5	IL1R2
RAI14.1	7.30478684124966e-47	0.438180932610652	0.344	0.108	1.46431757019691e-42	EpC5	RAI14
TMEM123.1	7.77674477423634e-47	0.851428568581137	0.684	0.416	1.55892625744342e-42	EpC5	TMEM123
UBE2L6	1.30030070428113e-46	0.394403408546624	0.334	0.103	2.60658279180196e-42	EpC5	UBE2L6
CRYAB.3	1.47560099720592e-46	1.16862586195482	0.742	0.443	2.95798975899899e-42	EpC5	CRYAB
CGNL1	1.65747040078578e-46	0.261175256370818	0.165	0.029	3.32256516541517e-42	EpC5	CGNL1
C12orf75.1	3.46507846642862e-46	1.11100758565074	0.608	0.358	6.94609629380281e-42	EpC5	C12orf75
RACK1.2	9.8587185761896e-46	-0.610259155	0.856	0.979	1.97627872578297e-41	EpC5	RACK1
PPIA.2	1.05754359358862e-45	-0.622481992	0.904	0.978	2.11995188770776e-41	EpC5	PPIA
FXYD3.3	1.51047795878859e-45	0.792915006503701	0.962	0.929	3.02790411618762e-41	EpC5	FXYD3
ACTG1	1.65552478493406e-45	-0.566007968	0.97	0.996	3.31866498387882e-41	EpC5	ACTG1
CD47.2	2.0545597295809e-45	0.50980217681589	0.458	0.179	4.11857043391787e-41	EpC5	CD47

RUNX1.1	2.42945451966059e-45	0.364952737830839	0.339	0.107	4.87008453011162e-41	EpC5	RUNX1
RPS23.1	2.72465212076083e-45	-0.678313623	0.765	0.935	5.46183764127717e-41	EpC5	RPS23
RIN2	3.28148845527579e-45	0.368993208504266	0.339	0.107	6.57807175744586e-41	EpC5	RIN2
GRN.3	4.59915356778638e-45	0.755201697818405	0.623	0.336	9.21946324198457e-41	EpC5	GRN
RGS4	4.60405142530833e-45	0.42286516565809	0.106	0.012	9.22928148717309e-41	EpC5	RGS4
COX7B.4	5.29399725586153e-45	-0.733479949	0.62	0.841	1.06123468991e-40	EpC5	COX7B
RPL36.2	5.39052314463526e-45	-0.590198182	0.949	0.993	1.08058426957358e-40	EpC5	RPL36
COX4I1.2	5.49693278707881e-45	-0.594375951	0.916	0.977	1.10191514649782e-40	EpC5	COX4I1
GALNT5.1	6.29887817303452e-45	0.308943812038448	0.304	0.087	1.2626731185665e-40	EpC5	GALNT5
ITGAV	1.43335978551337e-44	0.386961869418979	0.322	0.098	2.87331302604009e-40	EpC5	ITGAV
PMEPA1	1.64275931140627e-44	0.348032419692662	0.253	0.065	3.29307531564501e-40	EpC5	PMEPA1
TNIP3.3	2.78336726602659e-44	0.384273881200117	0.306	0.087	5.5795380214769e-40	EpC5	TNIP3
RPL31.2	3.81650201445763e-44	-0.546344281	0.995	0.999	7.65055993818177e-40	EpC5	RPL31
RAB31.3	4.61681490240363e-44	0.32247241072913	0.359	0.113	9.25486715335831e-40	EpC5	RAB31
HEPHL1.2	1.04882782529049e-43	0.359438633087684	0.215	0.048	2.10248025857732e-39	EpC5	HEPHL1
RPL6.1	1.44144337400798e-43	-0.543880774	0.947	0.989	2.88951738753641e-39	EpC5	RPL6
PTMA.3	1.93021565371403e-43	-0.631535323	0.997	0.998	3.86931029943514e-39	EpC5	PTMA
RPL7.2	3.21073237119274e-43	-0.536588489	0.975	0.996	6.43623411129296e-39	EpC5	RPL7
XPA.2	3.46782642977639e-43	-0.796284866	0.197	0.541	6.95160486112975e-39	EpC5	XPA
DUSP1.3	3.71670006658276e-43	0.712661901508282	0.863	0.602	7.45049695347181e-39	EpC5	DUSP1
APOBEC3A.4	4.53776320787116e-43	0.463055969026696	0.301	0.086	9.09640012649852e-39	EpC5	APOBEC3A
RPL18.2	1.30596276244945e-42	-0.536025077	0.987	0.997	2.61793295360617e-38	EpC5	RPL18
RPS13.1	2.34428687399676e-42	-0.571442206	0.977	0.994	4.6993574676139e-38	EpC5	RPS13
LAMB1	2.70801914776963e-42	0.305744822448692	0.195	0.043	5.42849518361901e-38	EpC5	LAMB1
RPL3.3	5.11432752757447e-42	-0.557500754	0.959	0.988	1.02521809617758e-37	EpC5	RPL3
AREG.1	8.53264187519993e-42	0.308207750018466	0.238	0.06	1.71045339030258e-37	EpC5	AREG
RPS4X.1	9.16273381031241e-42	-0.580433985	0.886	0.975	1.83676161961523e-37	EpC5	RPS4X
CAST.1	1.49161007510913e-41	-0.643877368	0.61	0.855	2.99008155656377e-37	EpC5	CAST
POLR2J3.4	2.16214101415109e-41	-0.905218349	0.063	0.408	4.33422787696728e-37	EpC5	POLR2J3

SNRPD2.2	2.77418392085404e-41	-0.644829641	0.732	0.899	5.56112908774401e-37	EpC5	SNRPD2
COX7C.2	6.5684314960313e-41	-0.630800059	0.878	0.967	1.31670777769443e-36	EpC5	COX7C
GSN.3	7.49935726567941e-41	-0.739992656	0.213	0.575	1.50332115747809e-36	EpC5	GSN
CALM1.2	7.93315125965403e-41	-0.639458285	0.891	0.966	1.59027950151025e-36	EpC5	CALM1
TKT.3	8.88475422441746e-41	-0.732711134	0.289	0.628	1.78103783182672e-36	EpC5	TKT
RPS8.1	9.91395852320794e-41	-0.549052648	0.927	0.99	1.98735212556226e-36	EpC5	RPS8
RPL5.2	1.38760641120741e-40	-0.573739631	0.901	0.979	2.78159581190637e-36	EpC5	RPL5
TAPBP	1.91017440153058e-40	0.623700251523556	0.577	0.301	3.8291356053082e-36	EpC5	TAPBP
TPI1.4	2.36677090388618e-40	-0.685322851	0.767	0.914	4.74442895393023e-36	EpC5	TPI1
PPIC.3	2.41592524284384e-40	0.449653546918312	0.491	0.212	4.84296374180476e-36	EpC5	PPIC
GSTA1.4	3.19398721581156e-40	-2.181360288	0.046	0.376	6.40266677281586e-36	EpC5	GSTA1
PFKFB3.1	3.23368080110856e-40	0.429937740162031	0.349	0.121	6.48223653390222e-36	EpC5	PFKFB3
PALMD	3.50067447175411e-40	0.292609319441692	0.253	0.069	7.01745204607829e-36	EpC5	PALMD
RDH10.4	3.65905551771833e-40	0.305747906350126	0.316	0.099	7.33494269081816e-36	EpC5	RDH10
PRNP.1	4.80358195341296e-40	0.681783914422509	0.689	0.418	9.62926038381162e-36	EpC5	PRNP
ITM2A.1	6.91305694384292e-40	-0.747444278	0.099	0.454	1.38579139496275e-35	EpC5	ITM2A
MT-CO3.3	9.28670996335436e-40	-0.441639828	1	1	1.86161387925401e-35	EpC5	MT-CO3
GAPDH.2	1.01149401432202e-39	-1.319006094	0.284	0.609	2.02764090110991e-35	EpC5	GAPDH
ADH7.3	1.20559893603945e-39	-0.915785304	0.134	0.472	2.41674362718468e-35	EpC5	ADH7
DDX3Y	1.97235120527671e-39	0.251698283746941	0.22	0.055	3.9537752260977e-35	EpC5	DDX3Y
BTF3.3	2.04492458259773e-39	-0.566076313	0.889	0.978	4.09925581827542e-35	EpC5	BTF3
RPL13.2	2.12788527612491e-39	-0.510103318	0.97	0.995	4.26555882451999e-35	EpC5	RPL13
FABP5.3	3.00480663774741e-39	1.17199522026771	0.716	0.523	6.02343538602846e-35	EpC5	FABP5
DSC3.1	3.93733388826424e-39	-0.644377627	0.415	0.724	7.89277951241449e-35	EpC5	DSC3
OSGIN1.1	6.25472482158931e-39	0.407631371494532	0.322	0.109	1.25382213773579e-34	EpC5	OSGIN1
MME.3	7.56430856152701e-39	0.486196727773621	0.344	0.118	1.5163412942437e-34	EpC5	MME
ACTB.3	9.25424104380041e-39	-0.498069546	0.965	0.991	1.85510515964023e-34	EpC5	ACTB
RPS4Y1.1	1.40935613380564e-38	0.390733408864805	0.367	0.128	2.82519530582679e-34	EpC5	RPS4Y1
EMP1.4	1.62245488779811e-38	-0.818022356	0.408	0.725	3.25237306808009e-34	EpC5	EMP1

WNT5A.1	2.20899181793508e-38	0.369559094423866	0.375	0.137	4.42814499823266e-34	EpC5	WNT5A
RPL4.2	3.51049674590451e-38	-0.571151441	0.787	0.942	7.03714177684019e-34	EpC5	RPL4
TSPO.4	4.04563906822678e-38	-0.691145577	0.658	0.836	8.1098880761674e-34	EpC5	TSPO
SMIM3.1	7.48943295048129e-38	0.301913566283977	0.306	0.099	1.50133172925348e-33	EpC5	SMIM3
HNRNPA1.4	1.0240769779593e-37	-0.61509163	0.762	0.913	2.05286471001721e-33	EpC5	HNRNPA1
RPS7.1	1.71549397547005e-37	-0.506607346	0.942	0.984	3.43887922322727e-33	EpC5	RPS7
COX8A.4	1.97055862628391e-37	-0.673472323	0.653	0.834	3.95018182224872e-33	EpC5	COX8A
PRDM1.3	2.36466018336427e-37	0.521815866973214	0.408	0.163	4.74019780357202e-33	EpC5	PRDM1
RPL9P9.2	2.79481956110799e-37	-0.715549508	0.253	0.595	5.60249529219708e-33	EpC5	RPL9P9
PLAC8.3	4.32081651378307e-37	-1.252315106	0.086	0.4	8.66150878352954e-33	EpC5	PLAC8
RPL15.1	5.70733577482314e-37	-0.499551291	0.997	0.999	1.14409252942105e-32	EpC5	RPL15
CAV1.2	6.34293421849042e-37	0.756591903918219	0.519	0.262	1.27150459343859e-32	EpC5	CAV1
FOSB.3	7.23291267586991e-37	0.696209046542045	0.775	0.529	1.44990967500488e-32	EpC5	FOSB
FBLN1	1.56743646094043e-36	0.271731101893773	0.276	0.085	3.14208312960118e-32	EpC5	FBLN1
RPL10A.1	1.95608551389365e-36	-0.538672483	0.843	0.964	3.9211690211512e-32	EpC5	RPL10A
RND3.3	2.07582950312138e-36	0.708057385054904	0.696	0.444	4.16120782195712e-32	EpC5	RND3
MT-CO2.4	2.15424492648174e-36	-0.510301508	1	1	4.3183993796253e-32	EpC5	MT-CO2
COX6B1.2	2.88515712963045e-36	-0.562729725	0.841	0.944	5.78358598205721e-32	EpC5	COX6B1
IGFBP3	4.6655225514548e-36	0.341774506877489	0.195	0.048	9.35250650664629e-32	EpC5	IGFBP3
RPL37A.2	5.74146278125816e-36	-0.572848745	0.949	0.991	1.15093362913101e-31	EpC5	RPL37A
ODC1.3	7.14661421096719e-36	-0.899716022	0.21	0.515	1.43261028473048e-31	EpC5	ODC1
PLSCR1.3	1.78282526841121e-35	0.400056481059184	0.471	0.208	3.57385153305712e-31	EpC5	PLSCR1
RPL32.1	1.99275523769432e-35	-0.502558532	0.972	0.994	3.99467714948204e-31	EpC5	RPL32
ATP5PD.3	2.10431477955973e-35	-0.592597084	0.699	0.862	4.21830940710544e-31	EpC5	ATP5PD
GALNT1.2	2.36413746889182e-35	0.334623527978185	0.413	0.164	4.73914997014055e-31	EpC5	GALNT1
EIF1AY	3.0033479406157e-35	0.251517120308404	0.213	0.056	6.02051128175823e-31	EpC5	EIF1AY
IFITM2	4.11676667653912e-35	0.432232952879744	0.299	0.102	8.25247047979033e-31	EpC5	IFITM2
RPL19.1	7.95762097480926e-35	-0.46870339	0.97	0.993	1.59518470061026e-30	EpC5	RPL19
KRT15.3	8.90014144805857e-35	0.87833070771879	0.408	0.178	1.78412235467782e-30	EpC5	KRT15

RPL30.2	1.21441663523932e-34	-0.793560611	0.504	0.761	2.43441958700074e-30	EpC5	RPL30
ATP5F1B.3	1.44104887513168e-34	-0.590648899	0.625	0.829	2.88872657508896e-30	EpC5	ATP5F1B
RPL7A.1	5.14064874669421e-34	-0.486541565	0.906	0.985	1.03049444776232e-29	EpC5	RPL7A
COX6C.3	8.93659632180347e-34	-0.599772293	0.909	0.965	1.79143009866872e-29	EpC5	COX6C
YPEL5.2	9.46677463258398e-34	0.585829205199693	0.615	0.366	1.89770964284778e-29	EpC5	YPEL5
ATP5PO.2	1.18025086656228e-33	-0.592208564	0.625	0.821	2.36593088711074e-29	EpC5	ATP5PO
PLS1.2	1.70053849084691e-33	0.369763210870286	0.377	0.15	3.40889945875171e-29	EpC5	PLS1
YBX1.2	1.737924332572e-33	-0.634639065	0.463	0.737	3.48384311707383e-29	EpC5	YBX1
AC092747.4	1.74189732872557e-33	0.318367204390117	0.162	0.037	3.49180738516329e-29	EpC5	AC092747.4
TNS4	1.81395582235301e-33	0.325494872812534	0.344	0.131	3.63625584148885e-29	EpC5	TNS4
NTS.4	1.863559842152e-33	-1.916019993	0.071	0.368	3.73569205957791e-29	EpC5	NTS
DHRS3.1	2.68601835000685e-33	0.265792859530627	0.243	0.073	5.38439238442374e-29	EpC5	DHRS3
COX5B.3	2.72568228707258e-33	-0.592235242	0.8	0.924	5.46390271266569e-29	EpC5	COX5B
AGTRAP.1	4.86416098846715e-33	0.34125078558053	0.352	0.138	9.75069711748124e-29	EpC5	AGTRAP
NDFIP2.1	6.50428005712875e-33	0.413112099904473	0.534	0.27	1.30384798025203e-28	EpC5	NDFIP2
IL1B.2	8.17145007745501e-33	0.374975710813482	0.246	0.074	1.63804888252663e-28	EpC5	IL1B
FKBP1A.3	8.56749469923535e-33	-0.69895044	0.395	0.659	1.71743998740872e-28	EpC5	FKBP1A
DSP.2	9.43074385032236e-33	-0.760328746	0.876	0.938	1.89048691223562e-28	EpC5	DSP
MED21	1.50904323423096e-32	0.628980293129424	0.392	0.175	3.02502806733939e-28	EpC5	MED21
MT-ATP6.3	1.87614318467695e-32	-0.417520769	1	1	3.76091662800341e-28	EpC5	MT-ATP6
RPS19.1	2.1428380427254e-32	-0.432667744	0.992	0.999	4.29553314044734e-28	EpC5	RPS19
IL1A.2	2.91694064043053e-32	0.279756799015794	0.246	0.074	5.84729920780703e-28	EpC5	IL1A
CCND1.1	3.96492723115166e-32	-0.832501416	0.263	0.547	7.94809312756662e-28	EpC5	CCND1
TSC22D1.2	4.65118100125872e-32	0.740765058179796	0.81	0.604	9.32375743512324e-28	EpC5	TSC22D1
IL1RN.4	4.85469664510264e-32	0.607714013807324	0.595	0.354	9.73172489477275e-28	EpC5	IL1RN
GSTM3.3	5.31757315262461e-32	-1.124697443	0.144	0.435	1.06596071417513e-27	EpC5	GSTM3
RPS3.2	1.19401970434387e-31	-0.495427545	0.949	0.99	2.39353189932772e-27	EpC5	RPS3
EEF1B2.1	1.27893167938287e-31	-0.516760492	0.797	0.923	2.56374644449091e-27	EpC5	EEF1B2
ADRB2.1	1.29501872614301e-31	0.32425774934166	0.339	0.13	2.59599453842629e-27	EpC5	ADRB2

SHISA5.1	1.3587866600576e-31	0.445546310617648	0.486	0.244	2.72382373875146e-27	EpC5	SHISA5
SERPINE2.1	1.4104592990597e-31	0.32332726001539	0.359	0.14	2.82740671089506e-27	EpC5	SERPINE2
RPSA.1	1.4821947735223e-31	-0.552929341	0.638	0.862	2.97120764300281e-27	EpC5	RPSA
DSC2.3	1.97443300253094e-31	-1.005739744	0.527	0.726	3.95794839687353e-27	EpC5	DSC2
COL17A1.3	2.33049426109283e-31	0.873016981975864	0.666	0.445	4.67170879578668e-27	EpC5	COL17A1
SPRR2D.4	3.53751434032109e-31	-0.311710757	0.314	0.104	7.09130124660766e-27	EpC5	SPRR2D
SCPEP1.2	4.89756391623114e-31	0.62492365209794	0.504	0.278	9.81765662647695e-27	EpC5	SCPEP1
ATP5MC3.3	5.33219204778389e-31	-0.533545251	0.81	0.912	1.06889121789876e-26	EpC5	ATP5MC3
RPL21.1	1.06757725124733e-30	-0.46312251	0.987	0.995	2.14006535785041e-26	EpC5	RPL21
NPM1.2	1.13441958499704e-30	-0.558111514	0.841	0.952	2.27405750008507e-26	EpC5	NPM1
TNFAIP8.2	1.17953193731944e-30	0.474577058109032	0.516	0.264	2.36448972155054e-26	EpC5	TNFAIP8
GJB2.3	1.62587512829448e-30	0.48489175701846	0.585	0.319	3.25922928217912e-26	EpC5	GJB2
RPL26.2	2.07147835496763e-30	-0.548964298	0.724	0.895	4.15248551036811e-26	EpC5	RPL26
TXNDC17.2	2.07395514265689e-30	0.685026508061822	0.775	0.627	4.15745047896999e-26	EpC5	TXNDC17
CDC42EP3	2.68811490591943e-30	0.27609527157916	0.235	0.074	5.38859514040609e-26	EpC5	CDC42EP3
IFNGR1	3.45540499018761e-30	0.502663856645808	0.6	0.353	6.92670484333008e-26	EpC5	IFNGR1
CDH3.1	4.87818829541035e-30	0.346987882329884	0.38	0.163	9.77881625697958e-26	EpC5	CDH3
HINT1.1	5.43647048706224e-30	-0.53543931	0.77	0.912	1.0897948738365e-25	EpC5	HINT1
OPTN.1	7.74553824073204e-30	0.372120817164454	0.438	0.204	1.55267059573715e-25	EpC5	OPTN
IFI6.1	8.73771085294588e-30	0.287885847329011	0.253	0.084	1.75156151758153e-25	EpC5	IFI6
HLA-A	1.38283504557929e-29	0.47409571844369	0.537	0.293	2.77203113236824e-25	EpC5	HLA-A
TSC22D3	2.47455461001078e-29	0.644636174943741	0.322	0.133	4.9604921712276e-25	EpC5	TSC22D3
SCEL.3	2.85016444918538e-29	-0.802176064	0.038	0.299	5.71343965483701e-25	EpC5	SCEL
CLU	2.9822646068731e-29	1.167955529	0.327	0.139	5.97824763093781e-25	EpC5	CLU
NR4A2.1	3.38950128351433e-29	0.316100152999041	0.256	0.088	6.79459427293283e-25	EpC5	NR4A2
UQCRH.2	3.45647906442142e-29	-0.498741392	0.841	0.929	6.92885793253919e-25	EpC5	UQCRH
IL13RA1.2	3.59381739222145e-29	0.281633183996275	0.324	0.126	7.20416634444712e-25	EpC5	IL13RA1
VSNL1.4	5.06431643827201e-29	-0.720642445	0.567	0.766	1.01519287321601e-24	EpC5	VSNL1
NFKBIA.3	5.10355878962243e-29	0.647307149703005	0.891	0.718	1.02305939496771e-24	EpC5	NFKBIA

SLC5A1.1	5.19381328285718e-29	0.291137813743103	0.195	0.056	1.04115181068155e-24	EpC5	SLC5A1
APLP2.1	5.28182084356463e-29	0.421692167446741	0.597	0.334	1.05879380630097e-24	EpC5	APLP2
FTL.2	7.38224487876884e-29	-0.751498163	0.896	0.955	1.479844808398e-24	EpC5	FTL
PRKX.1	1.03589710582105e-28	-0.512795384	0.084	0.353	2.07655933832888e-24	EpC5	PRKX
RPL35A.1	1.06431015586589e-28	-0.465233837	0.97	0.992	2.13351613844876e-24	EpC5	RPL35A
NDUFB2.4	1.46903066619639e-28	-0.593519266	0.557	0.745	2.94481887345728e-24	EpC5	NDUFB2
GPC3	2.05469616273083e-28	-0.566031625	0.043	0.305	4.11884392781023e-24	EpC5	GPC3
CTSB.2	2.1140278873409e-28	0.725543411526334	0.808	0.75	4.23778030296358e-24	EpC5	CTSB
MT-CYB.2	2.11492209191308e-28	-0.344592354	1	1	4.23957282544896e-24	EpC5	MT-CYB
MCC	2.23690943377612e-28	0.336441653281433	0.352	0.149	4.4841086509476e-24	EpC5	MCC
PKP1.2	2.3582166478042e-28	-0.622127499	0.316	0.577	4.7272810921883e-24	EpC5	PKP1
ATP1A1	2.42077489157828e-28	0.580976980312184	0.843	0.684	4.85268534765783e-24	EpC5	ATP1A1
PRDX2.2	2.94380220036294e-28	-0.703859711	0.203	0.477	5.90114589084755e-24	EpC5	PRDX2
GSTP1.2	3.10744278769276e-28	-0.649455942	0.992	0.999	6.22917981220891e-24	EpC5	GSTP1
RPS24.1	6.17789353810418e-28	-0.477467336	0.939	0.988	1.23842053864836e-23	EpC5	RPS24
MIR205HG.1	6.29016225887258e-28	0.555300489905024	0.775	0.542	1.2609259264136e-23	EpC5	MIR205HG
PPFIBP1	6.90756381630598e-28	0.348662288798717	0.405	0.184	1.3846902426167e-23	EpC5	PPFIBP1
TMEM14A.3	7.7829208917841e-28	-0.601069823	0.19	0.47	1.56016432196704e-23	EpC5	TMEM14A
KLK11.4	8.377651549768e-28	-0.709459699	0.175	0.436	1.67938402966649e-23	EpC5	KLK11
RPL14.1	9.42133542181611e-28	-0.441196439	0.949	0.992	1.88860089865726e-23	EpC5	RPL14
APP.1	1.01308702770621e-27	0.496898604326996	0.547	0.312	2.03083425573988e-23	EpC5	APP
SLC25A6.2	1.48100959523088e-27	-0.497564484	0.671	0.875	2.96883183459982e-23	EpC5	SLC25A6
RPL22.1	1.99671510611919e-27	-0.477739822	0.841	0.936	4.00261510172652e-23	EpC5	RPL22
SPTLC2.2	2.36104065750817e-27	-0.466752659	0.076	0.336	4.73294210204087e-23	EpC5	SPTLC2
TMSB4X.3	2.38638792295237e-27	-0.740841798	0.995	0.996	4.78375323035032e-23	EpC5	TMSB4X
CD9.3	2.52122194232323e-27	-0.517063165	0.959	0.987	5.05404150558115e-23	EpC5	CD9
RPL35.3	2.58134864603461e-27	-0.440589882	0.927	0.971	5.17457149584098e-23	EpC5	RPL35
AGR2.1	3.01018416636938e-27	-0.675649832	0.119	0.379	6.03421517990406e-23	EpC5	AGR2
TMA7.2	3.18070482485171e-27	-0.564897781	0.433	0.679	6.37604089189773e-23	EpC5	TMA7

RPS15.2	3.57479972924356e-27	-0.409543531	0.977	0.993	7.16604353724165e-23	EpC5	RPS15
CXCL14.3	5.38208629358073e-27	-0.867392148	0.359	0.61	1.07889301841119e-22	EpC5	CXCL14
KRT6B.4	5.532613601098e-27	-1.772345306	0.152	0.403	1.10906772247611e-22	EpC5	KRT6B
GALNT11	5.96453131711631e-27	0.31679517332274	0.365	0.161	1.19564994782914e-22	EpC5	GALNT11
RPL27.2	9.49141971417856e-27	-0.444957892	0.952	0.984	1.90264999590423e-22	EpC5	RPL27
KCNJ15	1.07443934416214e-26	0.324103779695102	0.375	0.166	2.15382110930743e-22	EpC5	KCNJ15
RPS9.1	1.22949090502833e-26	-0.442406856	0.853	0.96	2.4646374682198e-22	EpC5	RPS9
LRRC75A-AS1.2	1.60018992873984e-26	-0.771367562	0.172	0.434	3.20774073115187e-22	EpC5	LRRC75A-AS1
VCAN.2	1.65883171942569e-26	-0.650752963	0.068	0.325	3.32529406476073e-22	EpC5	VCAN
GAS5.3	1.67043368155795e-26	-0.560382675	0.471	0.715	3.34855135805107e-22	EpC5	GAS5
MBNL2	1.97746720810691e-26	0.309175445358583	0.334	0.14	3.96403076537112e-22	EpC5	MBNL2
MAOA.1	2.00784264990962e-26	0.251782522466444	0.301	0.117	4.02492137600883e-22	EpC5	MAOA
GSTO1.3	2.23619654454278e-26	-0.559840134	0.641	0.821	4.48267959319045e-22	EpC5	GSTO1
PNRC1.2	3.44749838680285e-26	0.449609531207179	0.732	0.481	6.910855266185e-22	EpC5	PNRC1
ADD3	3.83515498552289e-26	-0.507647957	0.256	0.525	7.68795168397918e-22	EpC5	ADD3
SLPI.4	4.23832488607885e-26	0.258220308644725	0.527	0.278	8.49614606663366e-22	EpC5	SLPI
CTGF.3	4.41932575293351e-26	0.638121849157748	0.37	0.166	8.85898040433051e-22	EpC5	CTGF
TALDO1.2	4.97437990260714e-26	-0.583128517	0.559	0.728	9.97164195276627e-22	EpC5	TALDO1
G0S2	7.35459626914563e-26	0.445584394439292	0.192	0.06	1.47430236811293e-21	EpC5	G0S2
RBBP8.2	8.32394129012807e-26	0.601936553217965	0.524	0.3	1.66861727101907e-21	EpC5	RBBP8
ATP5MC2.1	8.34284674277251e-26	-0.542998841	0.625	0.818	1.67240705805618e-21	EpC5	ATP5MC2
STOM.1	1.08824975117883e-25	0.36788873743652	0.514	0.277	2.18150545121308e-21	EpC5	STOM
N4BP1.1	1.83256087107193e-25	0.357986839265987	0.377	0.175	3.67355152215079e-21	EpC5	N4BP1
RPS25.2	1.90064009917743e-25	-0.416567888	0.843	0.947	3.81002314281108e-21	EpC5	RPS25
MYO1B.1	2.18835501396393e-25	0.364145424241724	0.362	0.166	4.3867764609921e-21	EpC5	MYO1B
RPS21.1	2.32914562620792e-25	-0.499202363	0.894	0.968	4.6690053222964e-21	EpC5	RPS21
RALB.1	2.46330976499601e-25	-0.533445707	0.18	0.439	4.93795075491101e-21	EpC5	RALB
MUC15.3	2.65188113931462e-25	-0.503363656	0.028	0.261	5.31596093187009e-21	EpC5	MUC15
DSTN.2	2.98790901064878e-25	-0.502244063	0.656	0.81	5.98956240274654e-21	EpC5	DSTN

DYNLT3.4	3.29129600302736e-25	-0.844983058	0.382	0.591	6.59773196766864e-21	EpC5	DYNLT3
RPL34.3	3.36126860449517e-25	-0.4047815	0.98	0.996	6.73799904457101e-21	EpC5	RPL34
ATP5ME.4	4.45288131158158e-25	-0.536462237	0.4	0.624	8.92624587719644e-21	EpC5	ATP5ME
HLA-DRB1	4.93668521025679e-25	0.473682271376243	0.271	0.108	9.89607917248077e-21	EpC5	HLA-DRB1
EPHB6.3	5.60402508767576e-25	-0.491562295	0.061	0.3	1.12338286907548e-20	EpC5	EPHB6
COX5A.2	5.71294790695863e-25	-0.535588132	0.461	0.688	1.14521753742893e-20	EpC5	COX5A
CCL20.2	6.82135302119822e-25	0.71320213895139	0.228	0.078	1.36740842662939e-20	EpC5	CCL20
LRRC8D	8.81588021401801e-25	0.266764159767118	0.281	0.112	1.76723134770205e-20	EpC5	LRRC8D
NFKBIZ.3	1.02588469626106e-24	0.630191199896689	0.59	0.371	2.05648846212492e-20	EpC5	NFKBIZ
AMTN	1.04461478894202e-24	0.784883520796416	0.17	0.05	2.09403480591317e-20	EpC5	AMTN
NUPR1.2	1.19569883483398e-24	-0.591258818	0.324	0.555	2.39689788430819e-20	EpC5	NUPR1
DNAJB9	1.43342404739616e-24	0.263806014373939	0.281	0.111	2.87344184541035e-20	EpC5	DNAJB9
CYCS.3	1.57063430876263e-24	-0.60922889	0.537	0.698	3.14849353534557e-20	EpC5	CYCS
TUBB.3	1.71239146629686e-24	-0.681453335	0.377	0.618	3.43265993333868e-20	EpC5	TUBB
SLFN5	2.17326260684219e-24	0.303325552787586	0.352	0.157	4.35652222167585e-20	EpC5	SLFN5
CTAGE5.2	2.27090602720283e-24	0.346691497179098	0.405	0.199	4.5522582221308e-20	EpC5	CTAGE5
ATP1B3.3	3.29477725935014e-24	-0.425763502	0.577	0.854	6.60471049409329e-20	EpC5	ATP1B3
NEDD9	4.58400429498038e-24	0.262148678270301	0.205	0.068	9.18909500971767e-20	EpC5	NEDD9
UAP1.1	4.82284787886074e-24	0.27012257413432	0.306	0.127	9.66788085796423e-20	EpC5	UAP1
HIF1A.3	5.01901586123384e-24	0.451842953215067	0.684	0.472	1.00611191954294e-19	EpC5	HIF1A
GSTA4.1	7.89834001591409e-24	-0.471736571	0.119	0.363	1.58330123959014e-19	EpC5	GSTA4
PDCD5.4	1.21805251831306e-23	-0.545862815	0.367	0.593	2.44170807821036e-19	EpC5	PDCD5
MT-ND6.3	1.34204729719553e-23	0.47489225242528	0.949	0.793	2.69026801195816e-19	EpC5	MT-ND6
ZFAS1.2	1.93698801726785e-23	-0.54701658	0.511	0.719	3.88288617941514e-19	EpC5	ZFAS1
ARL6IP5	2.40122767199254e-23	0.419018240572677	0.673	0.466	4.81350099127624e-19	EpC5	ARL6IP5
MPP7.1	2.64249906828896e-23	0.267654787081801	0.294	0.122	5.29715363229205e-19	EpC5	MPP7
PPDPF.2	4.33330937366351e-23	-0.532460381	0.61	0.774	8.68655197044587e-19	EpC5	PPDPF
GBP6.4	4.57306840785216e-23	-1.097463565	0.258	0.464	9.16717293038045e-19	EpC5	GBP6
TUSC3	4.74407876805746e-23	0.316732903230972	0.385	0.19	9.50998029844798e-19	EpC5	TUSC3

SLC16A1	4.80224116801379e-23	0.395795771053047	0.377	0.188	9.62657264540044e-19	EpC5	SLC16A1
ATP2B1.2	5.23626502418622e-23	-0.452923032	0.122	0.369	1.04966168674837e-18	EpC5	ATP2B1
IGFBP5.2	5.97485827307839e-23	0.949079609068014	0.451	0.258	1.19772008942129e-18	EpC5	IGFBP5
FAU	6.18580658298408e-23	-0.393811293	0.982	0.991	1.24000678762499e-18	EpC5	FAU
HMGA1.3	6.72466209769674e-23	-0.436422825	0.134	0.377	1.34802576410429e-18	EpC5	HMGA1
RPL13A.2	7.15046674581462e-23	-0.387977421	0.987	0.996	1.433382563866e-18	EpC5	RPL13A
RPLP0	8.11172061431277e-23	-0.324851136	0.997	0.999	1.62607551434514e-18	EpC5	RPLP0
CD59.2	8.32915031695475e-23	0.407166614930791	0.597	0.368	1.66966147253675e-18	EpC5	CD59
ATP5MG.2	9.47897236299595e-23	-0.41655522	0.876	0.946	1.90015479988617e-18	EpC5	ATP5MG
KIF21A.1	9.68882538153652e-23	-0.539449295	0.261	0.493	1.94222193598281e-18	EpC5	KIF21A
GEM	9.86632198858557e-23	0.259244241763943	0.134	0.035	1.97780290583186e-18	EpC5	GEM
YWHAE.2	1.33604598095994e-22	-0.441666752	0.63	0.8	2.6782377734323e-18	EpC5	YWHAE
UBA6.1	1.38428515634362e-22	0.329404133138204	0.438	0.23	2.77493802440643e-18	EpC5	UBA6
CKB	1.46002954295928e-22	-0.404867402	0.094	0.327	2.92677522181618e-18	EpC5	CKB
RPL12.2	1.47661447356878e-22	-0.449199035	0.896	0.97	2.96002137371597e-18	EpC5	RPL12
F2R.1	1.51237360180612e-22	-0.433690856	0.091	0.323	3.03170412218054e-18	EpC5	F2R
EEF1A1.2	1.78673452596164e-22	-0.379056322	0.975	0.995	3.58168803074269e-18	EpC5	EEF1A1
FDFT1.3	2.12977918423535e-22	-0.483541866	0.134	0.372	4.26935535271818e-18	EpC5	FDFT1
RPL23.2	2.15690261444595e-22	-0.401210934	0.937	0.985	4.32372698091834e-18	EpC5	RPL23
ID1.2	2.47654938889043e-22	0.485165289880221	0.684	0.472	4.96449090496975e-18	EpC5	ID1
AVPI1	3.2188236057425e-22	0.300406614611964	0.428	0.217	6.45245380007142e-18	EpC5	AVPI1
NPC2.1	3.2635184806487e-22	0.429661411947056	0.767	0.598	6.54204914630839e-18	EpC5	NPC2
CADM1.2	3.73552857781558e-22	-0.415644831	0.071	0.294	7.48824058708912e-18	EpC5	CADM1
RPS15A.2	3.89668293673869e-22	-0.468328191	0.772	0.91	7.81129061498637e-18	EpC5	RPS15A
CYP4F11	5.36455070125056e-22	-0.371562078	0.015	0.217	1.07537783357269e-17	EpC5	CYP4F11
MX1.2	5.49194430407844e-22	0.352251038455624	0.278	0.12	1.10091515519556e-17	EpC5	MX1
RASSF9.1	8.68101328335894e-22	-0.435358517	0.089	0.314	1.74019592278213e-17	EpC5	RASSF9
GPD2	8.83240723361852e-22	0.25379769454251	0.296	0.128	1.77054435405117e-17	EpC5	GPD2
SULT2B1.4	9.53844359650956e-22	-0.644351685	0.081	0.298	1.91207640335631e-17	EpC5	SULT2B1

S100A11.4	1.17565209217762e-21	-0.658508579	0.99	0.998	2.35671218397926e-17	EpC5	S100A11
COL14A1	1.56323430510697e-21	0.270683579914792	0.114	0.028	3.13365948801744e-17	EpC5	COL14A1
ALCAM.2	1.702717247383e-21	-0.477509495	0.197	0.441	3.41326699410397e-17	EpC5	ALCAM
LDHB.4	1.79543327469403e-21	-0.563761432	0.38	0.605	3.59912554245165e-17	EpC5	LDHB
GJA1.3	1.80731154890363e-21	0.488245309995099	0.661	0.441	3.62293673093221e-17	EpC5	GJA1
HSPA6.2	1.83788143668256e-21	0.546940067471561	0.261	0.107	3.68421712797387e-17	EpC5	HSPA6
SRPK2	1.90064380327232e-21	0.266391352467651	0.296	0.13	3.81003056803969e-17	EpC5	SRPK2
CD46.3	2.23786514514303e-21	0.424885027121194	0.706	0.53	4.48602446995371e-17	EpC5	CD46
SEM1.2	2.28028147650178e-21	-0.427729079	0.752	0.846	4.57105224779546e-17	EpC5	SEM1
YWHAZ.2	2.36691310633191e-21	-0.410022548	0.929	0.957	4.74471401295294e-17	EpC5	YWHAZ
SP100	2.60039447029932e-21	0.311792174347478	0.324	0.15	5.21275075516201e-17	EpC5	SP100
RNF145	3.45738321484374e-21	0.295555035471069	0.471	0.257	6.93067039247577e-17	EpC5	RNF145
B4GALT1.1	3.73174395676918e-21	0.259305274500589	0.365	0.175	7.48065393573951e-17	EpC5	B4GALT1
EGR1.2	5.90324109876494e-21	0.543128844812298	0.815	0.637	1.18336371065842e-16	EpC5	EGR1
H2AFZ.4	7.60186851825217e-21	-0.813211541	0.658	0.781	1.52387056316883e-16	EpC5	H2AFZ
RUNX2	8.51236843713168e-21	0.312960137813462	0.294	0.13	1.70638937690742e-16	EpC5	RUNX2
ATP5MD.3	1.04366311070985e-20	-0.46068297	0.575	0.74	2.09212707172897e-16	EpC5	ATP5MD
PTCH1.3	1.21283891227687e-20	-0.452989584	0.035	0.236	2.43125688355022e-16	EpC5	PTCH1
KRT14.1	1.24283165281044e-20	0.617481714701726	0.873	0.807	2.4913803312238e-16	EpC5	KRT14
SKIL.1	1.33454743849461e-20	0.305863401987195	0.408	0.211	2.6752337952063e-16	EpC5	SKIL
CYC1.3	1.37931378688444e-20	-0.476251781	0.278	0.51	2.76497241718855e-16	EpC5	CYC1
ANKLE2	1.43486129775208e-20	0.267069016217897	0.354	0.173	2.87632295747383e-16	EpC5	ANKLE2
CTSD.3	1.83630118817698e-20	0.406603283407731	0.539	0.332	3.68104936181958e-16	EpC5	CTSD
EEF1D.3	1.95768278275139e-20	-0.600725272	0.081	0.299	3.92437090630345e-16	EpC5	EEF1D
LGALS3.4	2.0051482578587e-20	0.34830339129887	0.835	0.619	4.01952019770355e-16	EpC5	LGALS3
XBP1.2	2.03810853612067e-20	0.309490127062685	0.415	0.216	4.08559237150749e-16	EpC5	XBP1
PSMB8	2.19146735421904e-20	0.263554193164122	0.291	0.13	4.39301545826748e-16	EpC5	PSMB8
CCNL1.2	2.93138187536731e-20	0.492805773355943	0.678	0.504	5.87624810736132e-16	EpC5	CCNL1
CHCHD10.1	4.14773060758511e-20	-0.413808945	0.152	0.377	8.31454077596512e-16	EpC5	CHCHD10

LY6E.1	4.21529107806634e-20	0.447565900995662	0.694	0.517	8.44997249509179e-16	EpC5	LY6E
AL365357.1.1	5.10082669828588e-20	-0.486670151	0.046	0.251	1.02251171993839e-15	EpC5	AL365357.1
CLIC1.4	8.28618121853748e-20	-0.449557143	0.595	0.741	1.66104788706802e-15	EpC5	CLIC1
RPL11.2	9.53741118288918e-20	-0.374609736	0.952	0.991	1.91186944572197e-15	EpC5	RPL11
RBM8A.2	1.31774362237784e-19	-0.443319511	0.519	0.719	2.64154886541862e-15	EpC5	RBM8A
LMAN1	1.44345425194326e-19	0.366699440284625	0.666	0.465	2.89354839344546e-15	EpC5	LMAN1
ABLIM1.4	1.5063062909586e-19	-0.368196672	0.142	0.378	3.0195415908556e-15	EpC5	ABLIM1
IL6ST	1.57854921705335e-19	0.266923490562689	0.352	0.175	3.16435976050514e-15	EpC5	IL6ST
COX7A2.2	1.5822335428802e-19	-0.392727836	0.894	0.945	3.17174536005765e-15	EpC5	COX7A2
JUP.2	1.63190796967584e-19	-0.509498996	0.354	0.55	3.27132271601219e-15	EpC5	JUP
HIGD2A.1	1.67894520378835e-19	-0.48404458	0.342	0.558	3.36561355551412e-15	EpC5	HIGD2A
POF1B.2	1.68614568352896e-19	-0.410057434	0.038	0.231	3.38004763720215e-15	EpC5	POF1B
VIM.1	1.70508752797807e-19	0.572263339918728	0.248	0.11	3.41801845858483e-15	EpC5	VIM
LAPTM4B	2.5757976529748e-19	0.347820154764276	0.41	0.224	5.16344397515328e-15	EpC5	LAPTM4B
PRDX1.4	3.32190305700864e-19	-0.630615909	0.572	0.729	6.65908686807952e-15	EpC5	PRDX1
PNKD.1	3.4332293470548e-19	-0.424941719	0.215	0.436	6.88225154910606e-15	EpC5	PNKD
PIR.3	3.56265102987078e-19	-0.367875601	0.086	0.291	7.14169025447896e-15	EpC5	PIR
SAMD12.2	3.99423303542963e-19	-0.380073579	0.124	0.342	8.00683954282223e-15	EpC5	SAMD12
SH3BGRL3.2	4.1270866128454e-19	-0.434503595	0.504	0.687	8.2731578241099e-15	EpC5	SH3BGRL3
TACSTD2.4	6.25978578348667e-19	0.790044297676412	0.696	0.651	1.25483665815774e-14	EpC5	TACSTD2
IGHG3.1	6.2827213516036e-19	1.01467404591193	0.122	0.034	1.25943432214246e-14	EpC5	IGHG3
ZFAND5.2	6.77188302229213e-19	0.345181063424973	0.519	0.313	1.35749167064868e-14	EpC5	ZFAND5
GLTP.4	7.84202811217661e-19	-0.641381983	0.253	0.461	1.57201295536692e-14	EpC5	GLTP
NTRK2.2	8.4466811750333e-19	-0.376512128	0.124	0.343	1.69322170834718e-14	EpC5	NTRK2
RPL38.2	8.80694060013729e-19	-0.406661921	0.866	0.932	1.76543931270352e-14	EpC5	RPL38
ATP5PB.2	9.34883476389215e-19	-0.443949031	0.6	0.736	1.87406741676982e-14	EpC5	ATP5PB
PAK1.1	9.99716057110186e-19	-0.432633631	0.137	0.352	2.00403080808308e-14	EpC5	PAK1
IGKC.2	1.02314473340485e-18	1.31388859828967	0.19	0.07	2.05099593258336e-14	EpC5	IGKC
MYL9	1.13622236795527e-18	0.278266873753419	0.154	0.051	2.27767135880313e-14	EpC5	MYL9

IGHG4.1	1.14432206184884e-18	1.12900367765258	0.134	0.04	2.29390800518218e-14	EpC5	IGHG4
TMEM59	1.19611161401401e-18	0.352258855928312	0.785	0.624	2.39772534145249e-14	EpC5	TMEM59
TFRC.3	1.23137681057578e-18	0.552475294026325	0.425	0.254	2.46841795448022e-14	EpC5	TFRC
VDAC2.1	1.46079752890214e-18	-0.421758107	0.458	0.654	2.92831472643724e-14	EpC5	VDAC2
DEGS1.1	1.68370225508788e-18	-0.45002633	0.215	0.429	3.37514954054916e-14	EpC5	DEGS1
DLX5	2.65869218761954e-18	-0.383924355	0.063	0.259	5.32961435930213e-14	EpC5	DLX5
TUBA1B.3	2.66388785328328e-18	-1.141104063	0.094	0.289	5.34002959069166e-14	EpC5	TUBA1B
FABP4.1	3.72580451104515e-18	1.05170927162147	0.104	0.027	7.4687477228411e-14	EpC5	FABP4
STAT3.1	4.06568928416619e-18	0.266785328272653	0.468	0.267	8.15008073903954e-14	EpC5	STAT3
ADIRF.2	4.07575497761803e-18	-0.605538864	0.066	0.261	8.17025842813309e-14	EpC5	ADIRF
NFIB.3	4.21925487110163e-18	-0.51877	0.2	0.408	8.45791831461032e-14	EpC5	NFIB
RPS16	4.29793594975695e-18	-0.294880126	0.99	0.998	8.61564240488279e-14	EpC5	RPS16
RPL10.2	4.33201568720165e-18	-0.341001916	0.97	0.987	8.68395864656442e-14	EpC5	RPL10
PNPLA8.2	4.89651497382418e-18	0.291594999993518	0.435	0.243	9.81555391652794e-14	EpC5	PNPLA8
RPS11.2	4.93484018562864e-18	-0.349602581	0.924	0.981	9.89238063611117e-14	EpC5	RPS11
UQCRQ.3	5.27502581906831e-18	-0.413876688	0.79	0.853	1.05743167569043e-13	EpC5	UQCRQ
AL450405.1.2	7.12906567382479e-18	-0.621345871	0.195	0.392	1.42909250497492e-13	EpC5	AL450405.1
SMIM14.1	7.20094307369441e-18	0.300603366878864	0.443	0.255	1.44350104855278e-13	EpC5	SMIM14
LINC01133.1	7.21123597422411e-18	-0.389852088	0.096	0.3	1.44556436339297e-13	EpC5	LINC01133
ATP5MC1.4	8.72741811406187e-18	-0.473018489	0.408	0.592	1.74949823514484e-13	EpC5	ATP5MC1
CTSV.1	9.30258414340557e-18	0.254044879605811	0.339	0.168	1.86479601738708e-13	EpC5	CTSV
KLK10.3	1.03740382905591e-17	-0.724667213	0.116	0.311	2.07957971572547e-13	EpC5	KLK10
SDC2.2	1.08222619036247e-17	-0.387088582	0.035	0.213	2.1694306212006e-13	EpC5	SDC2
RPLP2.2	1.2297057488764e-17	-0.321837446	0.99	0.999	2.46506814419764e-13	EpC5	RPLP2
ANKRD22.2	1.25203041200804e-17	-0.388247049	0.028	0.201	2.50982016391132e-13	EpC5	ANKRD22
RPS27.3	1.4096862385523e-17	-0.332599596	1	0.999	2.82585703380194e-13	EpC5	RPS27
ENSA.2	2.26765600055747e-17	-0.417184895	0.382	0.582	4.54574321871751e-13	EpC5	ENSA
TMEM154.3	2.30196598170303e-17	-0.468774923	0.119	0.31	4.61452100692189e-13	EpC5	TMEM154
PHB2.2	2.59315834694795e-17	-0.35559294	0.175	0.379	5.19824522229186e-13	EpC5	PHB2

PPL.3	2.65325860178875e-17	-0.35009975	0.056	0.239	5.31872219314572e-13	EpC5	PPL
HNRNPA2B1.2	2.77963409689076e-17	-0.400956697	0.787	0.906	5.57205451062723e-13	EpC5	HNRNPA2B1
TUBB4B.3	3.4502847316936e-17	-0.4423402	0.19	0.386	6.916440773153e-13	EpC5	TUBB4B
GADD45A.3	4.05196390209285e-17	0.404043636879399	0.61	0.417	8.12256683813533e-13	EpC5	GADD45A
POR	5.3687878333414e-17	-0.317090751	0.063	0.244	1.07622720907162e-12	EpC5	POR
CFL1.3	5.41355522141461e-17	-0.344146683	0.785	0.88	1.08520127968477e-12	EpC5	CFL1
TSTD1.2	5.47033005491769e-17	-0.389795458	0.316	0.524	1.0965823628088e-12	EpC5	TSTD1
SERPINE1	5.98617697054306e-17	0.449615373079774	0.159	0.057	1.19998903551506e-12	EpC5	SERPINE1
RPL37.3	6.19250468541152e-17	-0.388360814	0.98	0.997	1.24134948923759e-12	EpC5	RPL37
MAL2.2	6.20302254020098e-17	-0.573402725	0.192	0.381	1.24345789840869e-12	EpC5	MAL2
HMGN2.3	6.31374567254831e-17	-0.568191326	0.299	0.492	1.26565345751903e-12	EpC5	HMGN2
EEF2.2	6.98226143477275e-17	-0.425262256	0.542	0.711	1.39966412721455e-12	EpC5	EEF2
SSR1.1	7.08066972959543e-17	0.293335319487553	0.57	0.368	1.4193910539947e-12	EpC5	SSR1
ASPH	7.38100562100169e-17	0.345193488839183	0.418	0.246	1.479596386786e-12	EpC5	ASPH
MSN	8.77105105975993e-17	0.296846189856678	0.408	0.227	1.75824489543948e-12	EpC5	MSN
RPS14.1	8.78974489559758e-17	-0.305458659	0.977	0.998	1.76199226177149e-12	EpC5	RPS14
CD58.1	1.01531815518211e-16	0.28255972994766	0.382	0.212	2.03530677387805e-12	EpC5	CD58
JAK1	1.34629274648253e-16	0.291921211637742	0.456	0.266	2.69877843959889e-12	EpC5	JAK1
NDUFB9.3	1.35394982687216e-16	-0.414356456	0.552	0.709	2.71412782294793e-12	EpC5	NDUFB9
NDUFS5.1	1.49345121326243e-16	-0.36458898	0.777	0.86	2.99377230210586e-12	EpC5	NDUFS5
SLC27A5	1.49435771337642e-16	-0.398385169	0.078	0.262	2.99558947223437e-12	EpC5	SLC27A5
HSPH1.2	1.55079892638102e-16	0.376857297023407	0.562	0.37	3.1087315278234e-12	EpC5	HSPH1
LBH	1.86148803094144e-16	-0.369558171	0.157	0.358	3.73153890682521e-12	EpC5	LBH
TMPRSS4.3	1.97322524258482e-16	-0.362151486	0.147	0.343	3.95552732128553e-12	EpC5	TMPRSS4
PERP.3	2.00863589746135e-16	-0.602364193	0.997	0.996	4.02651152005102e-12	EpC5	PERP
PTPN13.2	2.1310181893765e-16	0.340536579342358	0.504	0.317	4.27183906242414e-12	EpC5	PTPN13
TNFAIP3.3	2.15078470160476e-16	0.46155612354077	0.532	0.343	4.31146301283691e-12	EpC5	TNFAIP3
NPR3.1	2.17795385074019e-16	-0.285878179	0.013	0.168	4.36592628919378e-12	EpC5	NPR3
UGP2.3	2.89683978147866e-16	-0.467484591	0.357	0.534	5.80700502595212e-12	EpC5	UGP2

PTHLH	2.98391765598979e-16	0.418570765466412	0.309	0.159	5.98156133319714e-12	EpC5	PTHLH
LGALS3BP	3.08964932623962e-16	0.364503099381094	0.587	0.408	6.19351103937995e-12	EpC5	LGALS3BP
ETV5	3.10963440725681e-16	-0.260555933	0.028	0.192	6.23357313278701e-12	EpC5	ETV5
MAF	3.37534749398052e-16	0.341382806917295	0.415	0.244	6.76622158643334e-12	EpC5	MAF
WNK1	3.38896652620897e-16	0.291629882505001	0.352	0.191	6.79352229843851e-12	EpC5	WNK1
RPS28.1	3.47525571305529e-16	-0.344030055	0.934	0.981	6.96649760239064e-12	EpC5	RPS28
MT-CO1.3	3.56151329979104e-16	-0.293044146	1	1	7.13940956076111e-12	EpC5	MT-CO1
NDUFA4.4	3.59572982019461e-16	-0.374223337	0.937	0.969	7.20799999756212e-12	EpC5	NDUFA4
HSPA5.2	4.18751401197846e-16	0.451115259377293	0.848	0.742	8.39429058841203e-12	EpC5	HSPA5
B4GALT4.2	4.30739927081511e-16	-0.286017757	0.023	0.181	8.63461257827597e-12	EpC5	B4GALT4
PYCARD.1	4.53911177950335e-16	-0.361417834	0.154	0.347	9.09910347319241e-12	EpC5	PYCARD
PSMA7.4	4.56661797844596e-16	-0.334936957	0.967	0.98	9.15424239959278e-12	EpC5	PSMA7
RPL24.1	4.98785480649728e-16	-0.302375121	0.954	0.991	9.99865374510445e-12	EpC5	RPL24
RPL41.1	5.38365632108134e-16	-0.367089643	0.909	0.955	1.07920774612397e-11	EpC5	RPL41
JPT1.2	6.20283105198016e-16	-0.409339347	0.408	0.617	1.24341951267994e-11	EpC5	JPT1
PA2G4.4	7.16198665419425e-16	-0.407216695	0.299	0.493	1.43569184469978e-11	EpC5	PA2G4
CENPP.1	7.43712480566867e-16	-0.336897846	0.053	0.225	1.49084603854434e-11	EpC5	CENPP
C8orf59.2	7.64641209808766e-16	-0.374822344	0.223	0.416	1.53279976918265e-11	EpC5	C8orf59
SPRR2F.2	8.65872466567602e-16	-0.652037397	0.19	0.072	1.73572794648142e-11	EpC5	SPRR2F
CARD16.1	8.66314728671781e-16	-0.358042231	0.132	0.323	1.73661450509545e-11	EpC5	CARD16
MGLL.2	8.84228434704519e-16	-0.470619962	0.159	0.343	1.77252432020868e-11	EpC5	MGLL
CAPN2.2	1.28471641290471e-15	0.270310054392291	0.456	0.269	2.57534252130878e-11	EpC5	CAPN2
LAD1.2	1.29202832481014e-15	-0.353334841	0.147	0.334	2.58999997991441e-11	EpC5	LAD1
SERPINB13.4	1.35602942868425e-15	-0.558282627	0.246	0.428	2.71829659274044e-11	EpC5	SERPINB13
SQLE.2	1.42217165489293e-15	-0.391255564	0.127	0.31	2.85088529939836e-11	EpC5	SQLE
PFN1.4	1.51169072113258e-15	-0.309716473	0.939	0.964	3.03033521958238e-11	EpC5	PFN1
EIF3E.2	2.13717313574973e-15	-0.367500117	0.496	0.682	4.28417726792392e-11	EpC5	EIF3E
LY6G6C.4	2.17720011048406e-15	-0.461463314	0.018	0.168	4.36441534147635e-11	EpC5	LY6G6C
UBA52.1	2.25678938684028e-15	-0.323314329	0.914	0.964	4.52396000486003e-11	EpC5	UBA52

GM2A.2	3.08092387952514e-15	0.251677708835957	0.41	0.238	6.17602000889609e-11	EpC5	GM2A
PSD3	3.16289465077236e-15	-0.381494191	0.119	0.295	6.34033861693827e-11	EpC5	PSD3
PAFAH1B3.1	3.22279672455499e-15	-0.348489277	0.122	0.304	6.46041831404293e-11	EpC5	PAFAH1B3
RAN.3	3.23245831107009e-15	-0.411785941	0.633	0.756	6.47978593037111e-11	EpC5	RAN
CITED2.1	3.48566271679298e-15	0.284894844267066	0.296	0.15	6.98735948208321e-11	EpC5	CITED2
ELOVL6.2	3.77418779183924e-15	-0.252441716	0.013	0.158	7.56573684752093e-11	EpC5	ELOVL6
KRTDAP.4	3.86657360095645e-15	-1.821021493	0.056	0.218	7.7509334404773e-11	EpC5	KRTDAP
MT-ND4L.2	4.44549325611524e-15	-0.343362254	0.959	0.979	8.91143578120862e-11	EpC5	MT-ND4L
TPGS2.2	4.87518159942364e-15	-0.353959074	0.213	0.41	9.77278903420463e-11	EpC5	TPGS2
CD63	6.14928296084505e-15	0.310993939224693	0.83	0.672	1.232685262331e-10	EpC5	CD63
ATP5F1A.1	7.48452945566383e-15	-0.379063833	0.562	0.708	1.50034877468237e-10	EpC5	ATP5F1A
SERINC2.2	7.81624867708669e-15	0.363193357741201	0.435	0.268	1.5668452098088e-10	EpC5	SERINC2
ISG15.1	8.63314349544224e-15	0.272533434775776	0.238	0.113	1.73059994509635e-10	EpC5	ISG15
NHP2.2	9.76970100106477e-15	-0.39251004	0.078	0.246	1.95843426267344e-10	EpC5	NHP2
NT5E	9.79362850223307e-15	-0.28555862	0.018	0.162	1.96323076955764e-10	EpC5	NT5E
DSG1.2	1.20403341384562e-14	-0.388146695	0.018	0.16	2.41360538139493e-10	EpC5	DSG1
SNRPE.2	1.20727390319073e-14	-0.405231389	0.461	0.626	2.42010126633613e-10	EpC5	SNRPE
HHIP.3	1.29593290983556e-14	-0.419674439	0.013	0.152	2.59782711105636e-10	EpC5	HHIP
PINLYP	1.34183907104844e-14	-0.277837195	0.081	0.251	2.6898506018237e-10	EpC5	PINLYP
SUCLG1.4	1.3853778687544e-14	-0.440857903	0.643	0.742	2.77712847570507e-10	EpC5	SUCLG1
ETV4	1.40671176657522e-14	-0.263502422	0.053	0.213	2.81989440727669e-10	EpC5	ETV4
STRBP.1	1.74070252404334e-14	-0.282988187	0.073	0.239	3.48941227969727e-10	EpC5	STRBP
UQCRB.2	1.81472281767407e-14	-0.369227278	0.714	0.821	3.63779336030945e-10	EpC5	UQCRB
PAIP2	1.93067517777878e-14	-0.346853796	0.23	0.423	3.87023146137533e-10	EpC5	PAIP2
ERRFI1.2	2.00246304290479e-14	0.381541209265003	0.43	0.258	4.01413741580695e-10	EpC5	ERRFI1
HBEGF.4	2.28062830250748e-14	0.440261464531684	0.478	0.316	4.5717474952065e-10	EpC5	HBEGF
UTRN.2	2.58560355926476e-14	-0.374545529	0.2	0.387	5.18310089490213e-10	EpC5	UTRN
KCNK7.2	3.08024133233e-14	-0.26351182	0.015	0.155	6.17465177478871e-10	EpC5	KCNK7
NDUFA6.3	3.3892905617381e-14	-0.394729755	0.516	0.646	6.79417186006019e-10	EpC5	NDUFA6

CUTA.1	3.81582144255692e-14	-0.423571206	0.415	0.578	7.6491956637496e-10	EpC5	CUTA
LY6D.3	3.88249237111998e-14	-1.075926158	0.375	0.549	7.78284420714711e-10	EpC5	LY6D
TIMM8B.2	3.90448345574629e-14	-0.434938371	0.397	0.546	7.82692753538901e-10	EpC5	TIMM8B
C5orf46.3	4.04340885041e-14	-0.573746398	0.01	0.145	8.10541738153189e-10	EpC5	C5orf46
ILF2.4	4.30475572904976e-14	-0.384401409	0.284	0.456	8.62931333445315e-10	EpC5	ILF2
LGALS7.3	4.62713239686234e-14	-0.574264675	0.058	0.217	9.27554960275025e-10	EpC5	LGALS7
CKMT1B.1	5.04982851271379e-14	-0.320712954	0.122	0.295	1.01228862365861e-09	EpC5	CKMT1B
VMP1	5.09311537976601e-14	0.292327044324858	0.671	0.489	1.02096590902789e-09	EpC5	VMP1
NQO1.3	5.35006675720003e-14	-0.354578786	0.23	0.429	1.07247438214832e-09	EpC5	NQO1
HLA-DRA	5.4640849001899e-14	0.483091912678618	0.294	0.162	1.09533045909207e-09	EpC5	HLA-DRA
ANXA5.1	5.89207089824856e-14	0.336826841289657	0.547	0.373	1.18112453226291e-09	EpC5	ANXA5
PKM.1	5.90480544724718e-14	0.388870726948885	0.805	0.706	1.18367729995517e-09	EpC5	PKM
DMKN.3	5.99876263323855e-14	-0.867357944	0.286	0.445	1.202511957459e-09	EpC5	DMKN
SOX15	6.2014400956922e-14	-0.315934332	0.147	0.321	1.24314068158246e-09	EpC5	SOX15
DANCR	6.57531991532186e-14	-0.316011578	0.182	0.369	1.31808863022542e-09	EpC5	DANCR
ALDH3A2.1	7.66179697951145e-14	-0.412048845	0.397	0.569	1.53588382251286e-09	EpC5	ALDH3A2
KRT19.4	7.67762335050289e-14	0.395168794344103	0.841	0.697	1.53905637684181e-09	EpC5	KRT19
FDPS.4	7.76286748654924e-14	-0.374016798	0.056	0.21	1.55614441635366e-09	EpC5	FDPS
LAPTM4A	7.90802130448745e-14	0.332787056910598	0.871	0.719	1.58524195069755e-09	EpC5	LAPTM4A
COPS9.3	8.57599797582722e-14	-0.421342467	0.473	0.611	1.71914455423432e-09	EpC5	COPS9
APEX1.1	8.81494774227542e-14	-0.340029282	0.251	0.433	1.76704442441653e-09	EpC5	APEX1
PTBP3.2	8.947314340049e-14	0.286452993086702	0.446	0.28	1.79357863260622e-09	EpC5	PTBP3
TST.2	1.01581928313477e-13	-0.272860863	0.03	0.172	2.03631133497196e-09	EpC5	TST
TRIM29.2	1.21318981387452e-13	-0.392192406	0.41	0.561	2.43196030089287e-09	EpC5	TRIM29
MDH1.2	1.43346220140626e-13	-0.372191806	0.522	0.672	2.873518328939e-09	EpC5	MDH1
MCL1.1	1.45995122442176e-13	0.268975714633653	0.453	0.285	2.92661822447586e-09	EpC5	MCL1
CD200.1	1.67089051319108e-13	-0.272832549	0.02	0.156	3.34946712274284e-09	EpC5	CD200
MT-ATP8.2	1.75947299887637e-13	-0.322017417	0.83	0.901	3.52703957354758e-09	EpC5	MT-ATP8
ATP5PF.4	2.02071854880791e-13	-0.308510355	0.78	0.882	4.05073240294034e-09	EpC5	ATP5PF

CST3.2	2.0703064338293e-13	0.360439345172633	0.668	0.499	4.15013627725422e-09	EpC5	CST3
TIMP1.1	2.182837307223e-13	0.342959776726999	0.539	0.373	4.37571566605923e-09	EpC5	TIMP1
RPL9.2	2.18926559062735e-13	-0.280890345	0.962	0.99	4.38860180297160e-09	EpC5	RPL9
RBM3	2.47955269221486e-13	-0.354539855	0.557	0.683	4.97051132681391e-09	EpC5	RBM3
HNRNPD.2	2.5014901105578e-13	-0.365497074	0.438	0.607	5.01448707562417e-09	EpC5	HNRNPD
DDAH2.1	2.63541167944627e-13	-0.263052354	0.076	0.233	5.282946252618e-09	EpC5	DDAH2
ENTPD3.1	3.95524096283433e-13	-0.317904733	0.122	0.284	7.9286760340977e-09	EpC5	ENTPD3
CBX3.3	4.20955443525583e-13	-0.374250358	0.491	0.646	8.43847282091384e-09	EpC5	CBX3
ATP5F1C.2	4.49509745822878e-13	-0.353394263	0.608	0.737	9.01087236476542e-09	EpC5	ATP5F1C
UFD1.1	5.32366149746725e-13	-0.324563772	0.228	0.405	1.06718118378229e-08	EpC5	UFD1
DST.3	6.10452836023697e-13	0.47515607130848	0.699	0.565	1.2237137550931e-08	EpC5	DST
TBX3	6.58682708601611e-13	-0.314570184	0.167	0.337	1.32039535766279e-08	EpC5	TBX3
HSBP1.1	6.87591095144138e-13	-0.310926591	0.603	0.734	1.37834510932594e-08	EpC5	HSBP1
MDH2.2	7.07869286320829e-13	-0.345952501	0.468	0.631	1.41899477135873e-08	EpC5	MDH2
SDHB.2	7.28569745382164e-13	-0.335164619	0.251	0.427	1.46049091159309e-08	EpC5	SDHB
SMAP1.3	7.72498739210893e-13	-0.308797131	0.162	0.34	1.54855097262216e-08	EpC5	SMAP1
CDH1.2	7.93276704328737e-13	-0.375823342	0.377	0.536	1.59020248149739e-08	EpC5	CDH1
SLC39A6	8.10552392467324e-13	0.298413144546279	0.372	0.228	1.62483332594e-08	EpC5	SLC39A6
ISOC2.1	8.3474921431792e-13	-0.326753953	0.154	0.317	1.6733382750217e-08	EpC5	ISOC2
SLC7A8.2	8.35852768751442e-13	-0.302980852	0.144	0.311	1.67555046023914e-08	EpC5	SLC7A8
TUBA4A.2	8.81223805180392e-13	-0.383703341	0.271	0.44	1.76650123986461e-08	EpC5	TUBA4A
EIF3K.2	9.0264072281171e-13	-0.344676737	0.651	0.762	1.80943359294835e-08	EpC5	EIF3K
SRP9.2	9.28977649223591e-13	-0.315635765	0.544	0.683	1.86222859563361e-08	EpC5	SRP9
SERPINB11.4	1.11097371973544e-12	-0.35926903	0.015	0.14	2.22705791858166e-08	EpC5	SERPINB11
HLA-E.1	1.21049944360176e-12	0.270397401039685	0.542	0.377	2.42656718464408e-08	EpC5	HLA-E
GGCT.3	1.26922325389222e-12	-0.338777106	0.218	0.384	2.54428493475234e-08	EpC5	GGCT
MTPN.2	1.54573849876923e-12	0.29947851299791	0.529	0.371	3.0985873946328e-08	EpC5	MTPN
FAM210B.2	1.74428651165309e-12	-0.438705818	0.2	0.358	3.49659674125978e-08	EpC5	FAM210B
S100A14.4	1.87913326276658e-12	-0.508303189	0.585	0.714	3.76691053854189e-08	EpC5	S100A14

SVIL	1.98891172305354e-12	0.302675119156516	0.504	0.345	3.98697244003313e-08	EpC5	SVIL
RANBP1.3	2.1157983590623e-12	-0.405620743	0.309	0.468	4.24132939057629e-08	EpC5	RANBP1
PPP1CA.3	2.12455566296356e-12	-0.345711429	0.157	0.316	4.25888428197675e-08	EpC5	PPP1CA
LRRC8A.1	2.18661857843317e-12	-0.281870234	0.137	0.299	4.38329560232714e-08	EpC5	LRRC8A
RAB2A	2.23089876546134e-12	0.256876121089399	0.651	0.473	4.47205966524379e-08	EpC5	RAB2A
HMGB1.3	2.47093035802653e-12	-0.501074303	0.886	0.928	4.95322699569999e-08	EpC5	HMGB1
FUS.1	2.51398494726417e-12	-0.319846473	0.261	0.43	5.03953422528576e-08	EpC5	FUS
SKP1.1	2.68314957618456e-12	-0.25576837	0.83	0.921	5.37864164041957e-08	EpC5	SKP1
CHL1	2.8079173754736e-12	0.394499161360089	0.342	0.206	5.62875117087438e-08	EpC5	CHL1
C3orf14.1	3.02607160812275e-12	-0.276503135	0.078	0.226	6.06606314564286e-08	EpC5	C3orf14
CTNNAL1.3	3.15533241243376e-12	0.309012134369571	0.294	0.165	6.32517935396471e-08	EpC5	CTNNAL1
ATP5MPL.2	3.32087664110159e-12	-0.334854627	0.767	0.836	6.65702931475225e-08	EpC5	ATP5MPL
ARRDC3	3.37562067157705e-12	0.269028286068537	0.273	0.148	6.76676919824336e-08	EpC5	ARRDC3
EIF3A.1	3.72046752708565e-12	-0.31356579	0.453	0.619	7.45804920479589e-08	EpC5	EIF3A
TUBA1A.2	3.82581963889892e-12	-0.264954563	0.235	0.429	7.66923804813678e-08	EpC5	TUBA1A
FAM96B.3	3.84372261357517e-12	-0.382687301	0.327	0.477	7.70512635117279e-08	EpC5	FAM96B
CENPX.3	4.0183303141785e-12	-0.348035693	0.18	0.342	8.05514494780223e-08	EpC5	CENPX
ECH1.1	4.34540551189801e-12	-0.332048826	0.291	0.457	8.71079988915075e-08	EpC5	ECH1
HHIP-AS1.1	4.42075438272369e-12	-0.259348238	0.008	0.124	8.86184423560792e-08	EpC5	HHIP-AS1
SLITRK6.3	4.72002623588699e-12	-0.281854971	0.03	0.157	9.46176459245906e-08	EpC5	SLITRK6
RAB25.2	4.76503994658699e-12	-0.365768138	0.218	0.375	9.55199907692829e-08	EpC5	RAB25
APRT.3	5.06162043839381e-12	-0.393708941	0.471	0.604	1.01465243308042e-07	EpC5	APRT
SUB1.2	5.16741281991504e-12	-0.307651383	0.628	0.741	1.03585957388017e-07	EpC5	SUB1
CLTA	5.82539508341791e-12	-0.325366159	0.375	0.529	1.16775869842195e-07	EpC5	CLTA
ATP5F1D	7.21196613767995e-12	-0.330325007	0.385	0.551	1.44571073195932e-07	EpC5	ATP5F1D
CALML5.3	8.31490940021726e-12	-0.359459399	0.003	0.111	1.66680673836755e-07	EpC5	CALML5
DEK.3	8.83850464622383e-12	-0.412085938	0.344	0.512	1.77176664138203e-07	EpC5	DEK
HMGB3.3	9.07212443842332e-12	-0.373712097	0.208	0.365	1.81859806492634e-07	EpC5	HMGB3
SLC25A3.1	1.02732182158653e-11	-0.280344561	0.524	0.703	2.05936932355235e-07	EpC5	SLC25A3

HNRNPF.1	1.03754741262889e-11	-0.311563206	0.316	0.479	2.07986754335588e-07	EpC5	HNRNPF
CENPF.4	1.04660150415373e-11	-0.543288546	0.01	0.123	2.09801737522657e-07	EpC5	CENPF
BAIAP2	1.08069443644368e-11	-0.286467853	0.208	0.369	2.166360067295e-07	EpC5	BAIAP2
DIRAS3.3	1.08080795040368e-11	-0.391007998	0.081	0.225	2.16658761737922e-07	EpC5	DIRAS3
TCF4.2	1.19329176133884e-11	-0.305992608	0.18	0.34	2.39207266477984e-07	EpC5	TCF4
NDUFAB1.3	1.32650439556816e-11	-0.339338527	0.428	0.593	2.65911071135593e-07	EpC5	NDUFAB1
OGFRL1	1.3346918461854e-11	0.351225993591763	0.246	0.134	2.67552327486325e-07	EpC5	OGFRL1
SRSF3.2	1.33596310009114e-11	-0.342286934	0.562	0.686	2.6780716304427e-07	EpC5	SRSF3
PSMB6.2	1.43011370000967e-11	-0.348737784	0.524	0.633	2.86680592303939e-07	EpC5	PSMB6
UBB.2	1.53214060442233e-11	-0.427381404	0.846	0.879	3.071329055625e-07	EpC5	UBB
BLOC1S1.1	1.57334639546599e-11	-0.296040712	0.167	0.322	3.15393018435113e-07	EpC5	BLOC1S1
PCBP2.1	1.5959990493156e-11	-0.312219157	0.481	0.636	3.19933969425805e-07	EpC5	PCBP2
PTPN1	1.61997344024436e-11	0.296681500991798	0.382	0.245	3.24739875831384e-07	EpC5	PTPN1
NDUFC2.2	1.72578153741114e-11	-0.307099979	0.296	0.457	3.45950166989436e-07	EpC5	NDUFC2
SBSN.4	1.82758910226725e-11	-0.993727062	0.03	0.15	3.66358511440492e-07	EpC5	SBSN
GATM	1.84462734931074e-11	-0.273416161	0.096	0.242	3.69773998442831e-07	EpC5	GATM
COA3.2	1.90462992549296e-11	-0.391164074	0.337	0.476	3.81802114864319e-07	EpC5	COA3
LMNA.1	1.97845397536408e-11	-0.357339146	0.466	0.612	3.96600883901484e-07	EpC5	LMNA
PBX1	1.99921401729239e-11	0.289166959366296	0.337	0.205	4.00762441906433e-07	EpC5	PBX1
MGST2.2	2.0318681921703e-11	-0.344479552	0.266	0.418	4.07308297802458e-07	EpC5	MGST2
NREP	2.29103535023132e-11	0.265345268229279	0.192	0.093	4.59260946307371e-07	EpC5	NREP
SERBP1.3	2.47038112567914e-11	-0.323009398	0.562	0.688	4.9521260045364e-07	EpC5	SERBP1
TMEM40.2	2.65792665621491e-11	-0.263149892	0.066	0.201	5.3280797750484e-07	EpC5	TMEM40
HMOX2.1	2.77296498652761e-11	-0.254915216	0.129	0.279	5.55868561199324e-07	EpC5	HMOX2
RPS20.1	3.18903223775695e-11	-0.292675459	0.942	0.975	6.39273402380758e-07	EpC5	RPS20
TMEM94.3	3.20545974134499e-11	-0.306646376	0.048	0.174	6.42566459750017e-07	EpC5	TMEM94
C12orf57.2	3.59708556123205e-11	-0.314493666	0.294	0.457	7.21071771604576e-07	EpC5	C12orf57
MFF.1	3.98853142059347e-11	0.257943655015931	0.446	0.303	7.99541008572167e-07	EpC5	MFF
SERF2.2	4.20946591186945e-11	-0.252838784	0.975	0.974	8.4382953669335e-07	EpC5	SERF2

HNRNPK.2	4.34660129094909e-11	-0.303064015	0.678	0.777	8.71319694783655e-07	EpC5	HNRNPK
PON2.1	4.89593724602418e-11	-0.292951034	0.134	0.279	9.81439580338008e-07	EpC5	PON2
C9orf16.3	5.13625353404755e-11	-0.366951887	0.365	0.501	1.02961338343517e-06	EpC5	C9orf16
HNRNPA3.2	5.36504522629577e-11	-0.321879973	0.575	0.694	1.07547696606325e-06	EpC5	HNRNPA3
IFI27.3	5.84462438352274e-11	0.321663195157786	0.185	0.089	1.17161340392097e-06	EpC5	IFI27
GOLGB1	6.70555648497938e-11	0.294576527514647	0.633	0.48	1.34419585297897e-06	EpC5	GOLGB1
OCIAD2.2	7.20381728467296e-11	-0.294610876	0.243	0.397	1.44407721288554e-06	EpC5	OCIAD2
SLIRP.4	7.36194536376179e-11	-0.340062374	0.476	0.614	1.47577556761969e-06	EpC5	SLIRP
SNRPG.3	8.30645248651957e-11	-0.349677813	0.539	0.654	1.66511146544771e-06	EpC5	SNRPG
CALML3.4	9.84358089425368e-11	-0.666228575	0.397	0.497	1.97324422606209e-06	EpC5	CALML3
LBR.1	1.02578388312758e-10	-0.255672989	0.096	0.234	2.05628637211754e-06	EpC5	LBR
NDUFA12.1	1.11510394031311e-10	-0.276602935	0.478	0.634	2.23533735875167e-06	EpC5	NDUFA12
SF3B5.1	1.12095189013222e-10	-0.32275376	0.494	0.62	2.24706015895905e-06	EpC5	SF3B5
NCL.2	1.21713722317969e-10	-0.285703182	0.767	0.876	2.43987327758601e-06	EpC5	NCL
FOS.4	1.26866446095932e-10	0.378720127587284	0.982	0.942	2.54316477843904e-06	EpC5	FOS
KRT6A.3	1.27419865574009e-10	-1.154520549	0.911	0.911	2.55425862529659e-06	EpC5	KRT6A
LAMP2.1	1.31965794414762e-10	0.272234976919922	0.633	0.484	2.64538631483831e-06	EpC5	LAMP2
PHB.2	1.41617248750611e-10	-0.267632143	0.073	0.204	2.83885936845476e-06	EpC5	PHB
FOXE1	1.45889723055944e-10	-0.289462664	0.157	0.307	2.92450538837945e-06	EpC5	FOXE1
UQCRC1.2	1.56315500155539e-10	-0.323171089	0.372	0.524	3.13350051611793e-06	EpC5	UQCRC1
C1QBP.4	1.60518862430229e-10	-0.312206504	0.291	0.452	3.21776111627637e-06	EpC5	C1QBP
CYB5A.3	1.72193088029689e-10	-0.434566245	0.372	0.504	3.45178264264314e-06	EpC5	CYB5A
RTN3.1	1.90418147032613e-10	-0.296671135	0.319	0.467	3.81712217541576e-06	EpC5	RTN3
ANP32A.1	2.05177300597665e-10	-0.277849001	0.301	0.463	4.11298416778079e-06	EpC5	ANP32A
ZFYVE21.1	2.31941888611115e-10	-0.274551071	0.21	0.361	4.64950709909841e-06	EpC5	ZFYVE21
SNRPF.2	2.38629821778458e-10	-0.31454027	0.357	0.508	4.78357340737097e-06	EpC5	SNRPF
FKBP5	2.45646158836091e-10	0.255241799571003	0.243	0.135	4.92422290002829e-06	EpC5	FKBP5
HDAC2	2.58864712433578e-10	-0.299851735	0.296	0.444	5.1892020254435e-06	EpC5	HDAC2
C19orf70.2	3.05531961926488e-10	-0.331307674	0.324	0.462	6.12469370877838e-06	EpC5	C19orf70

ATP5MF.3	3.11191436062939e-10	-0.305704853	0.724	0.807	6.23814352731767e-06	EpC5	ATP5MF
OST4.2	3.51758844837851e-10	-0.311572436	0.471	0.597	7.05135780361957e-06	EpC5	OST4
UQCRC2.1	3.70919504918539e-10	-0.287624807	0.354	0.507	7.43545239559702e-06	EpC5	UQCRC2
DNAJB4	4.17770200448738e-10	0.287822080393118	0.291	0.173	8.3746214381954e-06	EpC5	DNAJB4
UQCRFS1.2	4.18300251358888e-10	-0.304723017	0.337	0.485	8.38524683874027e-06	EpC5	UQCRFS1
SRSF1.1	4.82380768427787e-10	-0.256998394	0.165	0.304	9.66980488390342e-06	EpC5	SRSF1
IMPDH2.2	4.93515469256727e-10	-0.277338079	0.235	0.38	9.89301109672036e-06	EpC5	IMPDH2
AKR1C1.2	5.04081791611194e-10	-0.316504703	0.094	0.224	1.0104823594638e-05	EpC5	AKR1C1
ELOVL1.1	5.04644258839773e-10	-0.268672455	0.195	0.34	1.01160988127021e-05	EpC5	ELOVL1
PARP4	5.218450715508e-10	0.326266718556942	0.415	0.286	1.04609063043073e-05	EpC5	PARP4
SLC38A2	5.84939172373949e-10	0.309326556627484	0.722	0.592	1.17256906494082e-05	EpC5	SLC38A2
TOP2A.4	6.24157194690703e-10	-0.559727677	0.01	0.106	1.25118551247698e-05	EpC5	TOP2A
NR4A1.3	6.3429299395377e-10	-0.399625905	0.111	0.243	1.27150373567973e-05	EpC5	NR4A1
CNBP.1	7.01176837810053e-10	-0.263770687	0.59	0.714	1.40557908907403e-05	EpC5	CNBP
ERH.2	7.16913295868831e-10	-0.295968094	0.484	0.624	1.43712439289866e-05	EpC5	ERH
DCXR.3	7.22996565752315e-10	-0.267029757	0.18	0.321	1.44931891570709e-05	EpC5	DCXR
TUBA1C.4	7.23059222349007e-10	-0.389669977	0.071	0.19	1.44944451712082e-05	EpC5	TUBA1C
HPGD.2	7.44098108778313e-10	-0.277685569	0.086	0.214	1.49161906885701e-05	EpC5	HPGD
RSRP1	8.57347714827014e-10	-0.283028013	0.281	0.431	1.71863922914223e-05	EpC5	RSRP1
YWHAQ.3	9.38519423653448e-10	-0.271493263	0.428	0.585	1.8813560366557e-05	EpC5	YWHAQ
EIF5A.3	9.8897160242326e-10	-0.324253704	0.43	0.567	1.98249247421767e-05	EpC5	EIF5A
GPI.1	1.00710688275586e-09	-0.260909631	0.19	0.329	2.01884645717239e-05	EpC5	GPI
FBL.2	1.02183680790988e-09	-0.270277469	0.246	0.395	2.04837406513614e-05	EpC5	FBL
SGK1.3	1.03404617574925e-09	0.456815063812577	0.757	0.659	2.07284896390695e-05	EpC5	SGK1
MRPL41.1	1.19871028005916e-09	-0.298451817	0.309	0.446	2.4029346274066e-05	EpC5	MRPL41
RAB11A.3	1.22125801546475e-09	-0.361383587	0.514	0.607	2.44813381780065e-05	EpC5	RAB11A
CBX1.2	1.33969195477931e-09	-0.263111718	0.324	0.482	2.6855464925506e-05	EpC5	CBX1
NME1.3	1.49326550921534e-09	-0.292968404	0.327	0.482	2.99340003977307e-05	EpC5	NME1
NDUFB7	1.72464044362832e-09	-0.286778989	0.453	0.587	3.45721423329732e-05	EpC5	NDUFB7

CCDC34.2	1.8187856594517e-09	-0.270475663	0.096	0.223	3.64593773293688e-05	EpC5	CCDC34
MRPL20.2	1.93829294671468e-09	-0.271661508	0.238	0.378	3.88550204098425e-05	EpC5	MRPL20
RPL15P3	2.09135573448726e-09	-0.26299363	0.23	0.365	4.19233170535315e-05	EpC5	RPL15P3
KLF5.1	2.27081815518899e-09	-0.32548325	0.552	0.656	4.55208207389185e-05	EpC5	KLF5
H3F3AP4.1	2.27184010530763e-09	-0.253273599	0.253	0.397	4.55413067509967e-05	EpC5	H3F3AP4
IDH1.1	2.44942738429458e-09	-0.275720868	0.235	0.37	4.91012213455691e-05	EpC5	IDH1
MKI67.4	2.54148713101045e-09	-0.404180733	0.023	0.12	5.09466510282354e-05	EpC5	MKI67
BUD31	2.5823401048544e-09	-0.270216601	0.18	0.315	5.17655897419114e-05	EpC5	BUD31
ANP32B.2	2.64264692925303e-09	-0.29904999	0.337	0.478	5.29745003438063e-05	EpC5	ANP32B
NDUFS4.2	3.23618759846864e-09	-0.283562841	0.41	0.543	6.48726165989023e-05	EpC5	NDUFS4
HIST1H4C.4	3.81330305398894e-09	-0.796989706	0.542	0.66	7.64414730202624e-05	EpC5	HIST1H4C
MRPL51.2	3.85299322379446e-09	-0.315707559	0.499	0.604	7.72371021641837e-05	EpC5	MRPL51
KMT2E	3.88904427424652e-09	0.251076507474925	0.489	0.36	7.79597815215458e-05	EpC5	KMT2E
MT2A.3	4.26351656229686e-09	0.821786460853152	0.592	0.486	8.54664530078028e-05	EpC5	MT2A
TSPAN13	4.50239428605446e-09	-0.274571955	0.241	0.376	9.02549958582478e-05	EpC5	TSPAN13
PPIB.2	4.505595776511e-09	-0.30036975	0.114	0.238	9.03191729359394e-05	EpC5	PPIB
DYNLT1.2	4.65382973986832e-09	-0.333655068	0.514	0.608	9.32906709654004e-05	EpC5	DYNLT1
POLR2E.3	4.88947842751715e-09	-0.292657786	0.299	0.429	9.80144845580088e-05	EpC5	POLR2E
XRCC5.2	5.43961214077995e-09	-0.304181989	0.633	0.718	0.000109042464974075	EpC5	XRCC5
ANXA2.3	5.6943806216595e-09	-0.371212583	0.995	0.993	0.000114149553941786	EpC5	ANXA2
JUN.3	5.8452594633205e-09	0.376306845215109	0.944	0.887	0.000117174071201723	EpC5	JUN
BCAM.3	6.37112513081859e-09	-0.295512955	0.187	0.318	0.000127715574372389	EpC5	BCAM
TIMM10.1	6.78158089195597e-09	-0.260375731	0.165	0.293	0.000135943570560149	EpC5	TIMM10
NUSAP1.4	6.94722455075989e-09	-0.576955915	0.033	0.13	0.000139264063344533	EpC5	NUSAP1
HNRNPM.2	7.18143142970149e-09	-0.281191325	0.375	0.509	0.000143958974439796	EpC5	HNRNPM
RPL39.1	8.91293443294465e-09	-0.261715062	0.957	0.981	0.000178668683642809	EpC5	RPL39
GJB6.4	8.97524760253433e-09	-0.282651978	0.111	0.232	0.000179917813440403	EpC5	GJB6
HMGB2.4	9.05183710430427e-09	-0.580304304	0.154	0.272	0.000181453126592883	EpC5	HMGB2
GADD45B.3	9.59774098788488e-09	0.454370185286423	0.706	0.584	0.00019239631584314	EpC5	GADD45B

PSMB1.1	1.02734293515007e-08	-0.264289683	0.532	0.655	0.000205941164780184	EpC5	PSMB1
MRPS33.1	1.10498308648139e-08	-0.276351777	0.296	0.43	0.000221504909516059	EpC5	MRPS33
ZNF706.2	1.38786972370299e-08	-0.297572058	0.435	0.555	0.000278212364813501	EpC5	ZNF706
MAGED2.2	2.38376847179059e-08	-0.286203254	0.256	0.381	0.000477850227855141	EpC5	MAGED2
HMGCS1.2	2.77851900264444e-08	-0.252268723	0.157	0.281	0.000556981919270105	EpC5	HMGCS1
NDUFB10.1	2.8480963859934e-08	-0.254837029	0.428	0.553	0.000570929401536237	EpC5	NDUFB10
AKR1B1.2	2.84897092164225e-08	0.33521306274923	0.344	0.234	0.000571104710952406	EpC5	AKR1B1
CHCHD2.3	2.96532906328523e-08	-0.252465593	0.777	0.833	0.000594429864026157	EpC5	CHCHD2
IGFBP7.3	3.10849059098387e-08	0.298842410570052	0.289	0.177	0.000623128023868627	EpC5	IGFBP7
S100A2.3	3.21820769468737e-08	0.310290909404629	0.992	0.977	0.00064512191447703	EpC5	S100A2
RBMS1	3.48208999997437e-08	0.250384930208582	0.37	0.252	0.000698019761394862	EpC5	RBMS1
HMGN3.2	4.34493452780823e-08	-0.259179815	0.519	0.637	0.000870985575444438	EpC5	HMGN3
UBL5.2	5.3912610637208e-08	-0.292752586	0.716	0.766	0.00108073219283347	EpC5	UBL5
CA2.2	5.85326407157847e-08	0.34914506076518	0.203	0.115	0.00117334531578862	EpC5	CA2
RPS10	6.24872684518734e-08	-0.253553043	0.425	0.555	0.00125261978338625	EpC5	RPS10
CD44.1	6.49586510859851e-08	-0.267790742	0.382	0.509	0.00130216111966966	EpC5	CD44
SRSF7.2	6.68411079563251e-08	-0.278615983	0.61	0.688	0.00133989685009249	EpC5	SRSF7
EIF2S2.2	7.33095664869229e-08	-0.278244175	0.504	0.603	0.00146956356979686	EpC5	EIF2S2
CRABP2.3	8.3858191880253e-08	0.386794889886288	0.359	0.249	0.00168102131443155	EpC5	CRABP2
PNOC.4	8.42119724984031e-08	-0.311187256	0.091	0.2	0.00168811320070299	EpC5	PNOC
KRT6C.4	1.04231604467164e-07	-0.453146991	0.063	0.159	0.00208942674314877	EpC5	KRT6C
NDUFS6.2	1.06018118904692e-07	-0.297101484	0.433	0.542	0.00212523921156345	EpC5	NDUFS6
POSTN	1.19241959635017e-07	0.290663644941344	0.154	0.08	0.00239032432284355	EpC5	POSTN
ZFP36.2	1.35077452710568e-07	0.304780458377454	0.734	0.589	0.00270776261703604	EpC5	ZFP36
SSBP1.2	1.84797737582476e-07	-0.268543568	0.511	0.613	0.0037044554475783	EpC5	SSBP1
LSM7.2	1.85599507625139e-07	-0.268593579	0.385	0.5	0.00372052772985353	EpC5	LSM7
EIF3M.1	2.24393505241416e-07	-0.258999795	0.352	0.467	0.00449819220606943	EpC5	EIF3M
TXN2.1	2.3450380246521e-07	-0.252820846	0.327	0.449	0.0047008632242176	EpC5	TXN2
MACF1.1	2.49467471721696e-07	-0.263008156	0.246	0.365	0.00500082493813312	EpC5	MACF1

NDUFA2.1	2.52977050696768e-07	-0.268222172	0.352	0.463	0.00507117795826742	EpC5	NDUFA2
SPRR1A.4	2.65546652619276e-07	-1.428065209	0.043	0.128	0.005323148198406	EpC5	SPRR1A
HMGN1.3	2.72815643174588e-07	-0.252970482	0.537	0.639	0.0054688623830778	EpC5	HMGN1
TPX2.4	2.99610617223986e-07	-0.308271876	0.025	0.103	0.00600599443287202	EpC5	TPX2
DSG3.2	4.64647056366977e-07	-0.405928309	0.701	0.759	0.00931431489193242	EpC5	DSG3
UQCR11.2	4.94230411638757e-07	-0.27198104	0.582	0.657	0.00990734283171053	EpC5	UQCR11
RRM1.3	5.08680284001707e-07	-0.262343419	0.197	0.306	0.0101970049730982	EpC5	RRM1
KLF6.3	5.19387750342789e-07	0.274598446443029	0.719	0.61	0.0104116468433715	EpC5	KLF6
S100A16.4	6.26969904301548e-07	-0.395073356	0.701	0.713	0.0125682387016288	EpC5	S100A16
DUT.2	6.57996305485377e-07	-0.272682408	0.311	0.437	0.0131901939397599	EpC5	DUT
KRT8.2	8.04575769762673e-07	-0.343134155	0.385	0.486	0.0161285258806625	EpC5	KRT8
KLK13.3	8.37901595325512e-07	-0.83688545	0.066	0.152	0.0167965753798952	EpC5	KLK13
EIF6.2	8.49503017852984e-07	-0.269762575	0.387	0.488	0.0170291374958809	EpC5	EIF6
GNG5.2	9.46567701361905e-07	-0.258226719	0.595	0.671	0.0189748961415008	EpC5	GNG5
PTN.2	1.27700089655988e-06	-0.286787028	0.382	0.504	0.0255987599724393	EpC5	PTN
CKS1B.4	1.52670860245096e-06	-0.305672956	0.159	0.259	0.0306044006447319	EpC5	CKS1B
NDUFB11	1.9153524978979e-06	-0.270049251	0.635	0.696	0.0383951561728612	EpC5	NDUFB11
MT1E.2	2.02456722403242e-06	0.402060667910843	0.597	0.503	0.0405844745729539	EpC5	MT1E
SYNGR2.2	2.80979975486627e-06	0.261883412084082	0.516	0.41	0.0563252458860493	EpC5	SYNGR2
FTH1.4	2.91341518782958e-06	-0.772122687	0.641	0.663	0.0584023208552317	EpC5	FTH1
NOP10.2	3.09888422557415e-06	-0.254657878	0.506	0.592	0.0621202331858594	EpC5	NOP10
ZFP36L1	3.15765968295392e-06	0.261923408045085	0.813	0.72	0.0632984460044943	EpC5	ZFP36L1
HOPX.4	3.58517581358157e-06	-0.50565118	0.296	0.409	0.0718684343590561	EpC5	HOPX
PRSS22.4	4.47363738621164e-06	-0.46191857	0.21	0.123	0.0896785350439986	EpC5	PRSS22
CCNB1.4	5.76422191353782e-06	-0.369644485	0.041	0.11	0.115549592478779	EpC5	CCNB1
CWC15.2	6.78812296078786e-06	-0.257104188	0.418	0.502	0.136074712871953	EpC5	CWC15
SMC4.3	8.33913812639477e-06	-0.289457245	0.096	0.177	0.16716636288171	EpC5	SMC4
HSP90AA1.2	1.0086125546961e-05	0.288542726465099	0.937	0.923	0.20218647271438	EpC5	HSP90AA1
TXNIP.3	1.20571928508012e-05	0.772494144045318	0.522	0.48	0.241698487887162	EpC5	TXNIP

PRSS3.3	2.06536484920605e-05	-0.271496336	0.041	0.105	0.414023037671844	EpC5	PRSS3
SERPINB5.3	2.11947468882924e-05	-0.282015426	0.732	0.769	0.42486989612271	EpC5	SERPINB5
DIO2	3.03362012544711e-05	0.292123345360893	0.154	0.093	0.608119490347128	EpC5	DIO2
PRDX6.3	4.36770953860468e-05	-0.28006066	0.456	0.548	0.875551054108694	EpC5	PRDX6
CNFN.2	4.65325983884111e-05	-0.920126951	0.127	0.203	0.932792467294088	EpC5	CNFN
SPARC.2	5.24935196835398e-05	0.69401484967259	0.329	0.278	1	EpC5	SPARC
H2AFV.2	7.53345472246754e-05	-0.261592614	0.281	0.362	1	EpC5	H2AFV
PI3.4	0.000155148534495433	-2.93301079	0.233	0.295	1	EpC5	PI3
SPRR2E.2	0.000507415782985109	-0.662316419	0.134	0.08	1	EpC5	SPRR2E
HSPA1A.1	0.00082273430749067	0.288149871881999	0.82	0.77	1	EpC5	HSPA1A
NUCB2.2	0.00138801381862161	-0.253617649	0.294	0.353	1	EpC5	NUCB2
KPNA2.3	0.00177889071020373	-0.261858113	0.165	0.223	1	EpC5	KPNA2
SOSTDC1.4	0.00207304999988222	0.853231257798857	0.327	0.51	1	EpC5	SOSTDC1
HIST1H1C.2	0.00250022157884782	-0.30519397	0.357	0.419	1	EpC5	HIST1H1C
LGALS1.3	0.00340672125807499	0.447138524421783	0.359	0.312	1	EpC5	LGALS1
CSTA.4	0.00436131418157829	-1.46791518	0.608	0.627	1	EpC5	CSTA
COL1A1	0.00531721057276412	0.253738755462362	0.218	0.17	1	EpC5	COL1A1
KRT16.4	0.00633319532309292	-0.27143257	0.461	0.372	1	EpC5	KRT16
GPNMB.3	0.00649528368392748	-0.485269132	0.203	0.248	1	EpC5	GPNMB
DNAJB1.1	0.0074223584228271	0.314375150625329	0.843	0.82	1	EpC5	DNAJB1
