# Supplementary material for: Multiomics Reveals IL-17 Drives Epithelial Keratinization and Proliferation via EHF in Odontogenic Keratocysts
Source: Int J Mol Sci. 2026 May 4;27(9):4115. doi: 10.3390/ijms27094115 (PMC13163638; doi:10.3390/ijms27094115)
Supplement: Supplementary file 1 [file ijms-27-04115-s001.zip › ijms-4235677-supplementary/Supplementary Table S14.pdf]

Supplementary Table S14. Keratin, Cornified Envelope, and IL-17 Pathway Scores.

	Keratin_score	Cornified Envelope_score	IL-17_pathway_score
A_98763_AAACATCGAGATGTACATTGGCTC	0.553862657	0.080814202	0.261111308
A_98763_AAACATCGCAGATCTGGATAGACA	0.170241606	-0.102656132	0.335267427
A_98763_AACAACCAAACCGAGAACACAGAA	0.225093012	0.023213095	0.240843211
A_98763_AACAACCAAAGGTACAGGAGAACA	0.809509269	0.47955782	0.25542274
A_98763_AACCGAGACGACTGGATGAAGAGA	0.512275637	0.214387387	0.367947573
A_98763_AACTCACCATGCCTAAAGCAGGAA	0.691701788	0.512203069	1.010953146
A_98763_AACTCACCCAAGACTAAATGTTGC	0.414685138	0.197966986	0.068544242
A_98763_AAGAGATCACTATGCAGTCTGTCA	0.532257533	0.152814918	0.325906816
A_98763_AAGAGATCATAGCGACCCATCCTC	0.415776633	0.025557987	0.506231811
A_98763_AAGGTACAACTATGCAACGCTCGA	0.442732678	0.046757025	0.102605491
A_98763_ACACGACCACTATGCACCTAATCC	0.307749239	0.145284903	0.61867454
A_98763_ACAGCAGAACACAGAAACGCTCGA	0.195896636	0.034534923	0.019579204
A_98763_ACAGCAGAATAGCGACATAGCGAC	0.432033595	0.073607246	-0.033675267
A_98763_ACAGCAGAGAATCTGAAAACATCG	0.367842921	0.061406368	-0.13619864
A_98763_ACAGCAGAGACAGTGCAAGGTACA	0.478960892	-0.021673601	0.407459256
A_98763_ACATTGGCCACCTTACACATTGGC	0.397067984	0.118968325	0.422783075
A_98763_ACGCTCGAAGAGTCAACGACACAC	0.288369374	-0.050127802	0.042171886
A_98763_ACTATGCATGGTGGTAAGATCGCA	0.929925068	0.678770973	0.540905651
A_98763_AGAGTCAAACACAGAAAGGCTAAC	0.269948144	0.040383136	-0.073438452
A_98763_AGAGTCAAACGTATCAAACAACCA	0.686688735	0.050407919	0.085447552
A_98763_AGATGTACTAGGATGAGTACGCAA	0.465976257	-0.007633769	0.120071785
A_98763_AGATGTACTCCGTCTACAGATCTG	0.220285231	0.142721673	0.241964642
A_98763_AGCACCTCCTGAGCCAACATTGGC	0.460945626	0.830013226	0.81798379
A_98763_AGCACCTCGCCACATACGAACTTA	0.16746563	0.539941998	0.614243982
A_98763_AGGCTAACTGGAACAAGACTAGTA	0.576742095	0.046748494	0.415266841
A_98763_AGTACAAGAGTACAAGCTGGCATA	0.40414092	0.606108525	0.074518384
A_98763_AGTACAAGCTGGCATATATCAGCA	0.270730154	0.062545317	0.09196475
A_98763_AGTCACTAAAGAGATCAAGAGATC	0.557680058	0.381258463	1.025062799
A_98763_AGTGGTCAGCCAAGACATTGGCTC	0.698096674	0.262234095	0.123766541
A_98763_ATGCCTAAGGTGCGAACCATCCTC	0.879197827	0.14590591	0.602004394

A_98763_ATTGAGGACGACACACGCGAGTAA	0.369807255	0.187861609	0.430222567
A_98763_CAACCACAGACAGTGCAAACATCG	0.372568857	0.116875781	0.271409939
A_98763_CACCTTACGATGAATCGCGAGTAA	0.180439822	0.130451759	0.70461663
A_98763_CAGCGTTACATCAAGTGCTCGGTA	0.216269211	-0.037722512	-0.030128971
A_98763_CATACCAAACAGATTCGAATCTGA	0.259473988	0.269012004	0.176793275
A_98763_CATACCAAAGTCACTACGACTGGA	0.389383594	0.321941532	-0.076232975
A_98763_CATCAAGTAAGACGGACAGCGTTA	0.214818739	0.017221528	0.330149205
A_98763_CATCAAGTTGGTGGTAGACTAGTA	0.552506892	0.182209191	-0.232644535
A_98763_CCAGTTCAGCTCGGTATGAAGAGA	0.546287838	0.175862616	0.062139136
A_98763_CCGAAGTACGACACACCTGAGCCA	0.5142908	0.075594945	0.06450529
A_98763_CCGACAACAACAACCAAAACATCG	0.266320438	0.149108073	0.154234761
A_98763_CCGACAACATCCTGTAAAGGTACA	0.287221581	-0.07414415	0.308264092
A_98763_CCGACAACCCTCTATCCCGTGAGA	0.297486766	0.379795222	0.40844325
A_98763_CCTAATCCACACAGAACCTCCTGA	0.143626779	0.1625618	0.315275485
A_98763_CCTCCTGAAGTGGTCACTAAGGTC	0.268305348	0.113923087	0.040514766
A_98763_CCTCCTGAATAGCGACGTACGCAA	0.433734287	0.113214721	0.22407376
A_98763_CCTCTATCATGCCTAAGGAGAACA	0.392809372	0.187727747	-0.015638375
A_98763_CGAACTTAAAACATCGATGCCTAA	0.122625683	0.235904466	0.614348965
A_98763_CGACTGGATCCGTCTAGGTGCGAA	0.290234717	0.167487842	0.800648331
A_98763_CGCATACAAAGAGATCCTGGCATA	0.299838195	0.251394471	0.605933882
A_98763_CGCTGATCCCATCCTCACGCTCGA	0.280918738	0.090925851	0.457014873
A_98763_CGCTGATCTGGCTTCAATAGCGAC	0.301214737	0.093571721	0.134153339
A_98763_CGGATTGCGGAGAACACGCTGATC	0.083583674	0.089792964	0.786757981
A_98763_CTAAGGTCAGGCTAACCGACTGGA	0.157573138	-0.05178337	0.162480883
A_98763_CTGAGCCACGACTGGAGACAGTGC	0.275233886	0.46459859	0.87613763
A_98763_GAACAGGCACTATGCAATAGCGAC	0.557528978	0.048683363	0.448031411
A_98763_GAACAGGCCAAGACTAACATTGGC	0.33221655	0.05699137	0.094377606
A_98763_GAATCTGAACAGCAGAAGCACCTC	0.316242473	0.260764925	0.497732695
A_98763_GAATCTGAACCACTGTAGCCATGC	0.395393926	0.017706829	0.667946177
A_98763_GAATCTGACAAGGAGCGAATCTGA	0.42228183	0.180613981	0.728305431
A_98763_GACAGTGCCTGGCATAACCACTGT	0.208908374	0.082832014	-0.120512552
A_98763_GACAGTGCGACTAGTATTCACGCA	0.4190663	0.229102153	0.195585207

A_98763_GAGCTGAAATTGAGGAACAGATTC	-0.001816422	0.291751891	0.757773489
A_98763_GAGTTAGCAACGTGATCCGACAAC	0.534359456	0.091909403	0.353901748
A_98763_GAGTTAGCCGCATACAATGCCTAA	0.625442912	-0.000653437	-0.003847335
A_98763_GATGAATCAGCCATGCAGCACCTC	0.016098113	-0.093878928	-0.059960991
A_98763_GCCAAGACACAAGCTAGAATCTGA	0.6897969	0.123316246	-0.114132196
A_98763_GCCAAGACACCACTGTGGAGAACA	0.552674071	0.05329721	-0.069097228
A_98763_GCCAAGACCCATCCTCACCACTGT	0.380157814	0.002883602	-0.106669757
A_98763_GCGAGTAACCTAATCCCCGTGAGA	0.38545511	-0.013996484	0.289348225
A_98763_GCTAACGACAAGGAGCAGATGTAC	0.297568045	0.010529542	-0.091080601
A_98763_GCTCGGTATATCAGCAGTCTGTCA	0.420310356	0.085671039	0.492635135
A_98763_GGAGAACAACACAGAAGGTGCGAA	0.5022402	0.077354516	0.589381318
A_98763_GGAGAACAAGATGTACCAGATCTG	0.254064468	0.140466702	0.244642875
A_98763_GGTGCGAAACATTGGCCTCAATGA	0.365287455	-0.043853863	0.215745568
A_98763_GGTGCGAAATGCCTAAACGTATCA	-0.051124288	0.267813705	0.903435981
A_98763_GTCTGTCAAACGCTTAGAACAGGC	0.333633717	0.052652689	0.017030175
A_98763_GTGTTCTAGATAGACACATCAAGT	0.336204783	0.142031404	0.263028511
A_98763_TATCAGCACAGATCTGACACGACC	0.165437575	0.277761954	0.195419252
A_98763_TGAAGAGAGCGAGTAAAGCCATGC	0.383112172	0.333707585	0.213680571
A_98763_TGGAACAAAGCACCTCGGTGCGAA	0.310976465	0.366687165	1.101015421
A_98763_AAGACGGAAGATGTACAGTCACTA	0.606764874	0.167388213	0.227244635
A_98763_AAGACGGACAAGACTAACACGACC	0.562969857	0.238319601	0.485528588
A_98763_AAGGTACAGAGTTAGCTCCGTCTA	0.486983483	0.228842799	0.379901322
A_98763_ACACAGAAACACAGAACATCAAGT	0.599975856	0.562375315	0.63793959
A_98763_ACAGATTCAACGCTTAGACTAGTA	0.550073394	0.293202756	0.291301621
A_98763_ACCTCCAAAGTGGTCAAGATCGCA	0.228321614	0.939153718	1.080115837
A_98763_AGCACCTCCTCAATGACTCAATGA	0.385371679	0.200126185	0.169688766
A_98763_AGTGGTCAACTATGCAATTGGCTC	0.691326243	0.058990182	0.068071098
A_98763_AGTGGTCACCAGTTCACACTTCGA	0.657823999	0.305283313	0.448447918
A_98763_CAAGGAGCTGGCTTCAAACGTGAT	0.769319543	0.420713199	-0.05322886
A_98763_CACCTTACGAGTTAGCCGAACTTA	0.64360817	0.271368713	0.71959507
A_98763_CAGATCTGAGCAGGAACCATCCTC	0.655766518	0.600764622	0.244273178
A_98763_CCAGTTCAATTGGCTCGGTGCGAA	0.592983777	0.244305586	0.783729571

A_98763_CCGACAACAGCAGGAAACACGACC	0.237328545	0.174708424	1.235159411
A_98763_CCTAATCCCATCAAGTAAGACGGA	0.170894362	-0.032562158	0.284621408
A_98763_CGCATACAAGTACAAGCCGAAGTA	0.511320768	0.392880309	0.281760195
A_98763_CGCATACACGAACTTAAGATGTAC	0.619241597	0.225111792	1.022006524
A_98763_CGGATTGCAAACATCGTTCACGCA	0.407717897	0.205328353	0.95856486
A_98763_CGGATTGCCAAGACTACACTTCGA	0.563380248	0.337954959	0.500542982
A_98763_GAATCTGAACCACTGTAACGCTTA	1.029362953	0.848995113	1.440432506
A_98763_GACAGTGCCATCAAGTACAAGCTA	0.53524075	0.553754882	1.158292601
A_98763_GACTAGTAAATGTTGCCACTTCGA	0.358404486	0.692185769	0.781237014
A_98763_GCCACATAGACTAGTACGAACTTA	0.526110639	0.310639619	0.64768097
A_98763_GCTCGGTAAGATGTACGCCACATA	0.307009602	0.101565515	0.07796346
A_98763_GCTCGGTACTCAATGAACCACTGT	0.993405428	0.429348778	1.00659884
A_98763_GGTGCGAAAAGAGATCCATCAAGT	0.680502365	0.251617308	0.135029473
A_98763_GTACGCAAAGATCGCAAGGCTAAC	0.150435865	0.169194496	0.493352838
A_98763_GTACGCAACCAGTTCATGGCTTCA	0.450807084	0.095499747	0.356982131
A_98763_GTACGCAACCTAATCCCGGATTGC	0.028041157	0.0708424	-0.114677022
A_98763_TATCAGCAGAATCTGAAACCGAGA	0.544000553	0.229644953	0.447866332
A_98763_TGGAACAACAAGACTAACATTGGC	0.732718176	0.262584274	0.970690363
A_98763_TGGCTTCAAAGACGGAACACGACC	0.762149154	0.274066807	0.444481582
A_98763_ACTATGCAATCCTGTAATCATTCC	0.379677	1.196649934	1.730921907
A_98763_ACACGACCAAGAGATCGTCTGTCA	0.233709187	0.170817126	0.453183001
A_98763_AGCCATGCGTACGCAATGGCTTCA	0.203531058	0.018018917	0.220330278
A_98763_CACTTCGAGGTGCGAACGAACTTA	0.37183665	0.23780303	0.018511432
A_98763_CCTCCTGACAATGGAAACAGCAGA	0.384629403	0.297042758	0.577500666
A_98763_CTGAGCCAAACGCTTACCGAAGTA	0.482296017	0.068232194	0.042325451
A_98763_GACAGTGCGACAGTGCGTCTGTCA	0.187309915	0.077152435	0.587067912
A_98763_TGAAGAGAAAGGACACACAGCAGA	0.252769767	0.145846725	0.597593541
A_98763_GACAGTGCTGGAACAACCGAAGTA	0.417522199	-0.026486572	-0.123437938
A_98763_AACAACCAACCTCCAACCGTGAGA	0.283108922	0.277419995	0.82761946
A_98763_AACCGAGAGAATCTGAGTCGTAGA	0.384716638	0.315726724	1.096139196
A_98763_AACCGAGAGATGAATCCAGCGTTA	0.00417161	0.199636623	0.906616548
A_98763_AACTCACCGAACAGGCAGCACCTC	0.191863317	0.140259423	1.145267188

A_98763_AAGACGGAAATGTTGCGAATCTGA	0.266154818	0.232457825	0.911638337
A_98763_AAGAGATCCTGTAGCCACCTCCAA	0.211504915	0.318937605	1.036951314
A_98763_AATGTTGCCACCTTACAGCACCTC	0.165101205	0.249948819	0.850893679
A_98763_ACACGACCATTGAGGAACCACTGT	0.446784627	0.424861843	0.976675794
A_98763_ACAGCAGACTGAGCCACCTAATCC	0.452201161	0.427417346	0.996140574
A_98763_ACAGCAGATGGCTTCAAAGACGGA	0.023340086	0.215182022	0.938850822
A_98763_ACCTCCAAAAGAGATCGTCTGTCA	0.219108869	0.224147611	1.013894222
A_98763_AGCCATGCTGGCTTCACGACTGGA	0.519418884	0.140754268	0.511557016
A_98763_ATAGCGACAAACATCGGTCGTAGA	0.210271397	0.261720854	0.863308353
A_98763_ATCCTGTACCTCTATCCATCAAGT	0.280973222	0.356460153	0.863252457
A_98763_CATCAAGTCATCAAGTACAAGCTA	0.561647763	0.127000126	0.433500501
A_98763_CCGAAGTAACTATGCACGCATACA	0.185690078	0.139172375	0.544460102
A_98763_CCTCCTGACATCAAGTCTCAATGA	0.638097687	0.32777872	0.531001155
A_98763_CCTCCTGAGAGCTGAATGGTGGTA	0.375265094	0.242336368	0.661009981
A_98763_CCTCCTGAGCGAGTAAAACGCTTA	0.098694475	0.110908794	0.608040182
A_98763_CCTCTATCCGCATACAAACGCTTA	-0.024403411	0.296229216	1.0035626
A_98763_CGGATTGCACAGATTCGCTAACGA	0.641824569	0.382557697	0.750602035
A_98763_CGGATTGCAGTCACTAAAGACGGA	0.650364885	0.524518062	1.215820513
A_98763_GAACAGGCCGGATTGCAACGCTTA	0.204140853	0.340392181	1.049787353
A_98763_GAGCTGAAGAATCTGACGCTGATC	0.441653906	0.265083367	0.715284169
A_98763_GAGCTGAAGCGAGTAAACACGACC	0.445710536	0.583799142	1.143698656
A_98763_GAGCTGAAGTACGCAAAGCAGGAA	0.14487598	0.200733107	1.076866904
A_98763_GTACGCAACGACTGGAGTACGCAA	0.405075352	0.492135744	1.129052562
A_98763_GTCTGTCACGAACTTACGCATACA	0.526291549	0.417549945	1.157788882
A_98763_GTGTTCTACAGCGTTAATCCTGTA	0.542643599	0.629776615	2.130201063
A_98763_TATCAGCATGAAGAGAAGCACCTC	0.586569271	0.131558642	0.61656856
A_98763_TGAAGAGAGCGAGTAACTGGCATA	0.386011429	0.235228408	0.90247186
A_98763_AACGTGATGAATCTGAAGGCTAAC	0.890305827	0.590787909	-0.197582912
A_98763_AATCCGTCCAAGACTAATAGCGAC	0.69437016	0.695082612	1.026945275
A_98763_ACAAGCTAACCACTGTGAGTTAGC	0.850502615	0.322756226	0.045742925
A_98763_ACACAGAAGTACGCAAAGATGTAC	0.567822736	0.007574396	-0.315378196
A_98763_ACAGCAGAATTGAGGACACTTCGA	0.703810943	0.09882412	-0.304257879

A_98763_AGAGTCAAAACTCACCGATAGACA	0.324977907	0.404099428	-0.135921386
A_98763_AGATCGCACCGTGAGACTGAGCCA	0.310346742	0.44607087	-0.113260776
A_98763_AGCAGGAAACACAGAAGTCGTAGA	0.599163342	0.317485234	-0.400870797
A_98763_AGCCATGCCGACACACAGATGTAC	0.454023997	0.0364373	-0.187301394
A_98763_ATGCCTAAGATAGACAACACGACC	0.862773141	0.451360164	0.114895059
A_98763_CAGCGTTACAATGGAAGACTAGTA	0.461301795	0.279814137	-0.28205332
A_98763_CCAGTTCATTCACGCAGCCACATA	0.181194281	0.192270507	-0.025426994
A_98763_CCATCCTCAACTCACCAACTCACC	0.65532078	0.243639285	-0.35482093
A_98763_CCGACAACCGACACACAACTCACC	0.435473331	0.591373459	0.457618832
A_98763_CCGTGAGACAGCGTTAGCCACATA	0.687597686	0.362161274	0.250286282
A_98763_CGAACTTACGCATACAAAGGTACA	0.361937411	0.032782298	0.109594186
A_98763_CGCATACAGTGTTCTAAGCACCTC	0.362398495	1.01681718	0.125668363
A_98763_CGGATTGCCATACCAAAACAACCA	0.68142191	0.282295547	0.503826333
A_98763_CGGATTGCCGACACACAGCCATGC	0.830106576	0.388588166	-0.00811107
A_98763_CTAAGGTCAGATGTACTATCAGCA	0.511599553	0.138527346	-0.077501267
A_98763_CTCAATGAAGTCACTACAACCACA	1.047617983	0.715221706	0.466418848
A_98763_CTGAGCCAAAGACGGAGAACAGGC	0.470832835	1.010131104	1.006646655
A_98763_GACAGTGCATGCCTAAACAGCAGA	0.50047719	0.013268063	0.032001567
A_98763_GACTAGTAAAGGTACACCTCTATC	0.572227473	0.238275271	-0.142693188
A_98763_GACTAGTACTGTAGCCGAGTTAGC	0.342050653	1.075937908	1.237540455
A_98763_GAGTTAGCTGAAGAGATGAAGAGA	0.577153665	0.872421051	0.34835647
A_98763_GATGAATCACCACTGTAAACATCG	0.724292741	0.661119296	0.380769339
A_98763_GCCAAGACCATACCAACGAACTTA	1.062632747	0.90018739	0.491276703
A_98763_GCTAACGAAACTCACCGATAGACA	0.527373572	0.364187069	0.055377464
A_98763_GCTCGGTACGACTGGACCGAAGTA	0.528617823	0.496010129	-0.148547701
A_98763_GGAGAACAACCACTGTTCTTCACA	0.858907305	0.475502188	-0.05424129
A_98763_GTCTGTCACGACACACTGGTGGTA	0.910383064	0.598007773	0.293691613
A_98763_TATCAGCAATTGAGGAAGATGTAC	0.342406583	0.406526411	-0.06640046
A_98763_TATCAGCACCAGTTCAACGCTCGA	0.548939713	0.552762846	-0.16777395
A_98763_TCCGTCTACCGACAACCCTAATCC	0.447488023	0.082625779	-0.204250092
A_98763_TCCGTCTAGTCTGTCAGAATCTGA	0.654412698	1.029123757	0.731476794
A_98763_TCTTCACAAGAGTCAAACCACTGT	0.602443317	0.306850212	-0.091520402

A_98763_TGGAACAACATCAAGTAAACATCG	0.194955165	0.337818894	-0.496714998
A_98763_TGGCTTCACAGATCTGGCTAACGA	0.506608157	0.273170361	0.23112561
A_98763_AAACATCGCGCATACAGAATCTGA	0.312980024	0.039078662	0.067696995
A_98763_AACAACCAAAGGTACAAAACATCG	0.477464111	-0.011056072	-0.039434025
A_98763_AACCGAGAAAGACGGACAACCACA	0.45998566	0.090149434	-0.202813032
A_98763_AACCGAGAGCTCGGTAGAATCTGA	0.383589829	0.216806714	-0.147985433
A_98763_AACCGAGATAGGATGAACACAGAA	0.366738067	0.07512018	0.502834951
A_98763_AACGCTTACCGACAACGGTGCGAA	0.220355924	0.050421832	-0.000275605
A_98763_AACGCTTATTCACGCAATTGAGGA	0.447144883	0.283426924	0.088365457
A_98763_AACGTGATAAGGTACAGAATCTGA	0.351851796	0.127269955	0.302712708
A_98763_AACGTGATACAGATTCATTGGCTC	0.576672655	0.286409358	0.59615951
A_98763_AACTCACCAACGCTTACGAACTTA	0.141700181	0.08384346	0.055911323
A_98763_AACTCACCACTATGCAAATGTTGC	-0.057230935	0.151711373	0.189315942
A_98763_AACTCACCCCATCCTCATCCTGTA	0.334273658	0.035061065	0.028337676
A_98763_AACTCACCCTGAGCCAACAGATTC	0.513401956	0.115318921	-0.157290401
A_98763_AAGACGGAACAAGCTAAACGTGAT	0.507907545	0.075960328	-0.072885954
A_98763_AAGACGGAGTGTTCTAGACTAGTA	0.422838041	0.019126286	-0.393855374
A_98763_AAGAGATCAATCCGTCAGATCGCA	0.336966366	-0.166270559	-0.407232684
A_98763_AAGAGATCACACGACCAGTGGTCA	0.374011118	0.041460984	0.149069385
A_98763_AAGAGATCATCCTGTAAACAACCA	0.295496195	-0.006090423	-0.12264313
A_98763_AAGAGATCCGAACTTAAAGAGATC	0.352429875	0.007244846	-0.232006073
A_98763_AAGAGATCGAACAGGCCACTTCGA	0.252398042	0.204165048	0.009219245
A_98763_AAGGACACGACTAGTACGAACTTA	0.155374758	-0.081740294	-0.24235715
A_98763_AAGGACACGCTAACGAGAATCTGA	0.176718299	0.064872763	-0.363653554
A_98763_AAGGTACACCTAATCCGCTCGGTA	0.572694823	-0.021359652	-0.438863964
A_98763_AAGGTACAGCCACATAATTGGCTC	0.573921136	0.10294926	-0.055535
A_98763_AATCCGTCAGTACAAGAAACATCG	0.309400097	0.049909979	-0.094370207
A_98763_AATCCGTCGAGTTAGCATAGCGAC	0.59947517	0.03371234	-0.181772005
A_98763_AATGTTGCCCGACAACTGGTGGTA	0.512023773	-0.022930502	-0.098051723
A_98763_ACAAGCTAAGATCGCAAACAACCA	0.261854919	0.021981737	-0.352732897
A_98763_ACAAGCTACACTTCGAATAGCGAC	0.60138083	0.185697944	0.12696834
A_98763_ACAAGCTACAGCGTTATCTTCACA	0.316983328	0.233619407	0.012429689

A_98763_ACAAGCTAGCGAGTAAAGCAGGAA	0.158225715	0.152165583	0.026143552
A_98763_ACAAGCTATGGCTTCACGCTGATC	0.233530474	0.140277419	-0.042687834
A_98763_ACACAGAATGGTGGTAATCCTGTA	0.302010055	0.33946591	0.573205583
A_98763_ACACGACCACAAGCTAATGCCTAA	0.459362549	-0.081685363	-0.050302967
A_98763_ACACGACCCTAAGGTCCAGATCTG	0.377838756	-0.095789487	-0.100166635
A_98763_ACAGATTCATCCTGTAAACTCACC	0.281434092	0.226386484	0.145050434
A_98763_ACAGATTCCCTCCTGACAACCACA	0.394549307	0.159542092	-0.003269038
A_98763_ACATTGGCAAACATCGCGGATTGC	0.359027419	-0.036185815	0.305157237
A_98763_ACATTGGCCCATCCTCTTCACGCA	0.413340406	0.236332508	0.264936255
A_98763_ACATTGGCGAACAGGCAGATCGCA	0.456602304	0.36466664	0.333775231
A_98763_ACCACTGTAGTCACTAACCACTGT	0.170165615	0.240903845	0.008794316
A_98763_ACCACTGTCCAGTTCAGAGTTAGC	0.456951174	-0.026748511	-0.103189293
A_98763_ACCACTGTCCTAATCCCTGAGCCA	0.240533811	0.169940669	-0.212286737
A_98763_ACCTCCAAAGTGGTCATCTTCACA	0.436587133	-0.025971986	-0.296154092
A_98763_AGATCGCACAGCGTTACCATCCTC	0.136112005	0.234384865	-0.023948412
A_98763_AGATCGCACGAACTTAACACGACC	0.442778203	0.437634752	0.412644713
A_98763_AGATCGCAGAATCTGAGGAGAACA	0.127213379	-0.020159631	-0.083159939
A_98763_AGATCGCAGACTAGTAAGTCACTA	0.429701949	0.10351216	-0.024078747
A_98763_AGATGTACACAGATTCATAGCGAC	0.32647093	0.142397077	0.15239401
A_98763_AGATGTACAGCAGGAATGGTGGTA	0.396818398	0.0434869	0.250978932
A_98763_AGATGTACAGTGGTCAGCTCGGTA	0.45271171	-0.009985399	0.128613142
A_98763_AGATGTACCAAGGAGCTGAAGAGA	0.26577239	0.201118086	0.158430589
A_98763_AGATGTACCTAAGGTCCCATCCTC	0.510339799	0.071968825	0.195661235
A_98763_AGCACCTCCCTCTATCAGCCATGC	0.294789862	0.077270792	0.22954628
A_98763_AGCACCTCGGAGAACAGTCTGTCA	0.235347046	0.059832271	0.000473023
A_98763_AGCACCTCGTCGTAGAAATGTTGC	0.375528637	-0.008214292	-0.104422635
A_98763_AGCAGGAAACACAGAACTGAGCCA	0.319304273	0.332625993	0.160223104
A_98763_AGCAGGAAACAGCAGATGGAACAA	0.343747727	0.127127634	-0.116021048
A_98763_AGCAGGAACCGAAGTAAAGACGGA	0.455476977	0.120808326	0.111636405
A_98763_AGCAGGAACGACACACACGCTCGA	0.156415223	0.013841679	-0.130946809
A_98763_AGCAGGAAGAGCTGAAAACTCACC	0.215492933	0.005269863	-0.430020567
A_98763_AGCAGGAAGTACGCAACTGGCATA	0.486281695	0.12647148	0.660156109

A_98763_AGCAGGAATATCAGCACGAACTTA	0.409049258	0.360852379	0.333549628
A_98763_AGCCATGCAAACATCGCAGCGTTA	0.558981732	0.133468214	-0.197677708
A_98763_AGCCATGCAGCACCTCATCCTGTA	0.499014602	0.206070412	0.323558433
A_98763_AGCCATGCGAACAGGCCATACCAA	0.279639519	0.309930042	-0.214090613
A_98763_AGCCATGCGCCAAGACCTAAGGTC	0.506633962	0.080112494	-0.222878986
A_98763_AGCCATGCGGAGAACAACCTCCAA	0.490555017	0.106045854	-0.153357839
A_98763_AGTACAAGAAGGACACAACGTGAT	0.296696904	0.076199057	0.174236818
A_98763_AGTACAAGAGCACCTCCCGTGAGA	0.225818781	0.07165548	-0.344526714
A_98763_AGTCACTAGGTGCGAACCGTGAGA	0.103465216	0.225884839	-0.193483577
A_98763_AGTCACTATGGCTTCAAACGCTTA	0.324243024	0.118992835	-0.465150864
A_98763_AGTGGTCAACGCTCGAGACTAGTA	0.34137509	0.174177637	0.292026957
A_98763_AGTGGTCACCGACAACCCTAATCC	0.513692396	0.221033264	0.490031185
A_98763_ATAGCGACCTCAATGAGTACGCAA	0.235135932	0.068368238	-0.317135141
A_98763_ATCATTCCCATCAAGTCACTTCGA	0.522019533	0.188348835	0.105222069
A_98763_ATCATTCCGGAGAACAAACGTGAT	0.365786654	-0.020964279	0.034932243
A_98763_ATCCTGTAACGCTCGAACAGATTC	0.338455683	-0.049993136	-0.255390576
A_98763_ATCCTGTAACGCTCGAACTATGCA	0.334915879	0.175861006	0.034895117
A_98763_ATGCCTAAAGCCATGCGATAGACA	0.271155729	0.003779891	-0.271107148
A_98763_ATTGAGGATCTTCACAAGGCTAAC	0.357561504	-0.015298592	-0.020556765
A_98763_ATTGGCTCGTCTGTCACGGATTGC	0.341397348	0.048919897	-0.324122119
A_98763_ATTGGCTCTGAAGAGAACATTGGC	0.280274033	0.097319293	-0.022467355
A_98763_CAAGACTAAACGTGATTTCACGCA	0.247632354	0.225517384	0.785088914
A_98763_CAAGACTAACGTATCAACACGACC	0.023918044	0.033763387	-0.128308839
A_98763_CAAGACTAAGCCATGCAGATGTAC	0.615082556	0.270124906	-0.142142267
A_98763_CAAGACTAGCCACATACCGTGAGA	0.430071158	-0.017800643	0.072711845
A_98763_CAAGGAGCACACGACCTCTTCACA	0.463741328	0.048274299	-0.352131148
A_98763_CAAGGAGCATTGGCTCCGAACTTA	0.39018071	0.046586529	-0.156254908
A_98763_CAAGGAGCGAACAGGCCGCTGATC	0.490461784	0.228488788	-0.232050948
A_98763_CAATGGAAATTGGCTCAGATCGCA	0.287985101	-0.003698676	-0.038697922
A_98763_CACCTTACAGATCGCACACTTCGA	0.259782043	-0.009512036	-0.182110049
A_98763_CACCTTACCTGAGCCACAATGGAA	0.534519221	0.006344319	0.100245204
A_98763_CACCTTACTGGAACAACGCTGATC	0.186072239	0.04222241	-0.20640706

A_98763_CACTTCGACCATCCTCCAGATCTG	0.246318612	0.054782109	0.478960472
A_98763_CAGCGTTAGCCACATAGCCACATA	0.509667144	0.080782199	-0.099813879
A_98763_CATACCAAACAGATTCAGTCACTA	0.527166226	-0.027791737	-0.326523729
A_98763_CATACCAAAGCACCTCAAGGTACA	0.455365805	0.429708819	0.222104101
A_98763_CATACCAAAGGCTAACATCCTGTA	0.368297203	0.122491753	0.317528551
A_98763_CATCAAGTAAGGTACAAGCACCTC	0.426755239	0.015884392	-0.155162583
A_98763_CATCAAGTCCGACAACAACTCACC	0.43782022	0.067892414	0.02672685
A_98763_CATCAAGTCGACTGGAGGAGAACA	0.18065407	0.112698244	0.05846587
A_98763_CATCAAGTCTGTAGCCTCTTCACA	0.591874083	0.246592575	-0.180812686
A_98763_CATCAAGTGAATCTGACTGGCATA	0.362042808	0.059656099	-0.088807003
A_98763_CCAGTTCAAGCCATGCATCATTCC	0.465662257	0.158945577	0.101056575
A_98763_CCATCCTCAGTACAAGTGGAACAA	0.364359892	0.104815286	0.012016849
A_98763_CCATCCTCCGCTGATCCAGCGTTA	0.155397981	0.00635952	-0.162977503
A_98763_CCATCCTCTATCAGCAATGCCTAA	0.31154267	0.094736211	-0.034391825
A_98763_CCGAAGTAACAGATTCGGTGCGAA	0.339936422	0.004831315	0.180635967
A_98763_CCGACAACCCATCCTCCACTTCGA	0.376688315	-0.070963097	-0.200020445
A_98763_CCGACAACGCTCGGTAGCTCGGTA	0.355377472	0.078948422	0.626139005
A_98763_CCGACAACTGGAACAATGGAACAA	0.557110483	0.016267653	0.263038369
A_98763_CCTAATCCAGTGGTCAAGTCACTA	0.294572726	0.395045282	0.106174873
A_98763_CCTAATCCCAAGGAGCATGCCTAA	0.571266658	0.063822614	-0.243627944
A_98763_CCTCCTGAATTGAGGACCGAAGTA	0.563965018	0.160375407	0.366547545
A_98763_CCTCCTGAGATGAATCAAGGTACA	0.411777601	0.107377317	0.127718778
A_98763_CCTCCTGATGAAGAGAGCTCGGTA	0.422906326	-0.052337145	0.27071973
A_98763_CCTCTATCAAGACGGAGCCACATA	0.003003069	0.051344159	-0.276582568
A_98763_CCTCTATCATAGCGACAGTGGTCA	0.379254972	0.197455827	-0.052120222
A_98763_CCTCTATCATTGAGGAGCCACATA	0.341672963	0.027111184	0.079089135
A_98763_CCTCTATCCTGAGCCAACCACTGT	0.398217635	0.092224782	0.204870293
A_98763_CGAACTTAACATTGGCCAATGGAA	0.565992537	0.205234016	-0.096898116
A_98763_CGACACACAAGGACACACAAGCTA	0.225742753	0.024916083	0.0150181
A_98763_CGACACACATCATTCCCCTCCTGA	0.318825622	0.220785603	0.280333639
A_98763_CGACACACATCCTGTAGACTAGTA	0.275940317	-0.079092607	0.008935043
A_98763_CGACTGGAACTATGCAAGTGGTCA	0.232904171	0.033145615	-0.366897671

A_98763_CGACTGGACCGTGAGAAACGTGAT	0.212387867	0.161338502	0.190525017
A_98763_CGCATACAATAGCGACACCTCCAA	0.523410439	0.024356915	-0.128750374
A_98763_CGCATACACGAACTTAATGCCTAA	0.458104894	0.181567826	0.297848783
A_98763_CGCTGATCGGAGAACAACACAGAA	0.436255109	0.019402401	-0.24962819
A_98763_CGGATTGCAAACATCGATCATTCC	0.364216748	-0.038732992	-0.252986164
A_98763_CGGATTGCAAGGACACCGCTGATC	0.427686028	-0.012404266	-0.131075717
A_98763_CGGATTGCACGTATCAGTCGTAGA	0.456394646	0.083431894	0.272257504
A_98763_CGGATTGCCCGTGAGACCATCCTC	0.227782949	-0.077103969	-0.218267946
A_98763_CTAAGGTCAAACATCGAAGAGATC	0.426537549	0.335470524	-0.188456184
A_98763_CTAAGGTCACGCTCGAGGAGAACA	0.316083434	-7.73E-05	-0.08446305
A_98763_CTAAGGTCACGTATCAGTACGCAA	0.437433634	0.175075592	-0.011734753
A_98763_CTAAGGTCCAGCGTTAACACGACC	0.355400828	-0.061313747	0.063113523
A_98763_CTAAGGTCTGGTGGTAACAGCAGA	0.334424898	-0.025877181	-0.306680831
A_98763_CTCAATGAAGAGTCAATCCGTCTA	0.24222711	-0.010296021	0.114864635
A_98763_CTCAATGACCGACAACTCTTCACA	0.601292723	0.154805527	0.118235353
A_98763_CTGGCATAAGCACCTCTATCAGCA	0.440601168	0.049056845	-0.426031051
A_98763_CTGGCATAGAGCTGAATGGTGGTA	0.614336854	0.079039096	0.166416679
A_98763_CTGGCATAGTCTGTCAACGTATCA	0.555989048	0.075025946	0.039337209
A_98763_CTGTAGCCCGCATACAGTACGCAA	0.657191036	0.054359813	-0.259382617
A_98763_CTGTAGCCTGGCTTCACAGCGTTA	0.676112057	0.151933941	-0.143498104
A_98763_GAACAGGCAGCCATGCGTACGCAA	0.313462618	0.093876876	0.015981768
A_98763_GAACAGGCCAAGGAGCGACTAGTA	0.41467693	0.094737076	-0.008021126
A_98763_GAACAGGCCAAGGAGCGGAGAACA	0.637432129	0.025723941	0.097597109
A_98763_GAACAGGCGCTAACGAAACGTGAT	0.234589371	0.064862157	-0.06506087
A_98763_GAACAGGCTCTTCACAGAACAGGC	0.363643997	0.133598577	-0.298694385
A_98763_GAATCTGAACCTCCAAACAGCAGA	0.495532571	0.302902475	0.445063467
A_98763_GAATCTGACAATGGAAAAGGTACA	0.410440393	0.028283672	-0.021546617
A_98763_GAATCTGAGAGCTGAACTAAGGTC	0.416109907	0.144193274	-0.018832178
A_98763_GAATCTGAGGAGAACACAGCGTTA	0.51110138	-0.006517168	-0.549065717
A_98763_GACAGTGCACGTATCAAGTCACTA	0.491425915	0.464047986	0.320560972
A_98763_GACAGTGCCGACTGGATTCACGCA	0.395255754	-0.004533223	0.223418275
A_98763_GACAGTGCGATGAATCGGAGAACA	0.504008758	0.166710628	0.075648661

A_98763_GACTAGTACATCAAGTAGGCTAAC	0.173621614	-0.013330203	0.186109069
A_98763_GAGCTGAAACATTGGCAACAACCA	0.393722932	0.006074187	-0.282040973
A_98763_GAGCTGAAAGTACAAGGCGAGTAA	0.448077148	0.184996532	0.044284439
A_98763_GAGTTAGCAAGGTACAAGATGTAC	0.314769058	0.094889993	0.488195897
A_98763_GAGTTAGCAGATCGCAAGTGGTCA	0.289448347	0.153081042	-0.295899225
A_98763_GAGTTAGCATAGCGACACGCTCGA	0.520852614	0.032125684	-0.14532733
A_98763_GAGTTAGCCATCAAGTTGGTGGTA	0.627575158	0.11866719	0.043627948
A_98763_GATAGACACACTTCGAAGGCTAAC	0.259114736	0.062043703	0.104427534
A_98763_GATGAATCACACAGAAAGCAGGAA	0.480698717	-0.00935954	-0.212093589
A_98763_GATGAATCAGGCTAACGAACAGGC	0.17910103	-0.086207724	-0.229066666
A_98763_GATGAATCCTCAATGAGGTGCGAA	0.258708024	0.096506914	-0.11997481
A_98763_GCCAAGACATTGAGGAAGATGTAC	0.588094322	0.066174028	-0.330104842
A_98763_GCCAAGACCTAAGGTCAAGGTACA	0.397288994	0.17009825	0.25588973
A_98763_GCCAAGACGTCTGTCATCTTCACA	0.044958401	0.10585633	-0.025248031
A_98763_GCCACATAACACGACCAAGAGATC	0.173487488	0.104115735	0.114158619
A_98763_GCCACATACTGTAGCCAATCCGTC	0.351881189	0.076786314	0.207949753
A_98763_GCGAGTAAACACAGAACCGTGAGA	0.330251329	0.208594714	-0.400904641
A_98763_GCGAGTAAAGCACCTCCGCTGATC	0.670410731	0.050687317	-0.225338939
A_98763_GCGAGTAACGACTGGATGGAACAA	0.392106127	0.111158327	0.225773758
A_98763_GCTAACGAAACCGAGAACCTCCAA	0.606373509	0.220167609	-0.064102117
A_98763_GCTAACGACCAGTTCAATCCTGTA	0.337703627	-0.007783717	-0.068746391
A_98763_GCTCGGTAACGCTCGACGCATACA	0.326383039	0.200295914	-0.023918743
A_98763_GCTCGGTACTGAGCCACAATGGAA	0.40197559	0.011314707	-0.263833521
A_98763_GGTGCGAAGCTCGGTAAACGTGAT	0.380383342	0.157410614	0.052183608
A_98763_GTACGCAACGCTGATCAAGGTACA	0.611496753	0.270124754	0.535286863
A_98763_GTACGCAAGACAGTGCCCGTGAGA	0.288323699	0.111958742	-0.136701288
A_98763_GTACGCAAGCTAACGAAGATGTAC	0.293897212	0.0258137	0.008617266
A_98763_GTCTGTCAACCACTGTACGCTCGA	0.565705518	0.007879636	-0.22209681
A_98763_TAGGATGACGACACACACACGACC	0.359469495	-0.062959722	0.381451998
A_98763_TATCAGCAATGCCTAAAGGCTAAC	0.131948551	0.029720457	0.1688848
A_98763_TATCAGCAGTACGCAAGCGAGTAA	0.516203368	-0.002096112	0.152002549
A_98763_TCCGTCTACTGTAGCCAACTCACC	0.479501333	0.087634759	0.380892791

A_98763_TCTTCACAAAGGACACAACGTGAT	0.527442199	0.205425563	-0.181735665
A_98763_TCTTCACAATTGAGGAATTGAGGA	0.273697779	-0.082336881	-0.01463253
A_98763_TCTTCACAATTGGCTCGAATCTGA	0.203805062	0.035939273	0.034192565
A_98763_TCTTCACACCTAATCCGACTAGTA	0.243196398	-0.028042422	-0.063929562
A_98763_TGAAGAGAACGTATCACAACCACA	0.424993613	0.111123426	0.062752501
A_98763_TGAAGAGACCGTGAGAAGTGGTCA	0.405148936	0.104754492	-0.123658307
A_98763_TGGAACAAACCTCCAACCGTGAGA	0.140664482	0.141627224	0.276641211
A_98763_TGGAACAAAGATGTACGGTGCGAA	0.150776108	0.067645149	0.047669584
A_98763_TGGAACAACGGATTGCAACTCACC	0.184261032	0.127140071	-0.07948047
A_98763_TGGCTTCAGTCGTAGACCGTGAGA	0.499578432	0.156868935	-0.038587031
A_98763_TGGTGGTAAACCGAGACCTCTATC	0.448487006	0.029343065	-0.024284997
A_98763_TGGTGGTACTGAGCCAGGAGAACA	0.403182965	0.081995066	0.07472279
A_98763_TTCACGCACAACCACATGGCTTCA	0.47495566	0.110419011	-0.12363312
A_98763_TTCACGCAGAGTTAGCTTCACGCA	0.217707786	-0.055547934	-0.289482484
B_98618_AAACATCGAACCGAGAGCGAGTAA	0.360497056	0.065385379	-0.226687865
B_98618_AAACATCGACATTGGCGGTGCGAA	0.195208821	0.096816101	-0.217590531
B_98618_AAACATCGACCACTGTGGTGCGAA	0.067879947	-0.033479395	-0.315630924
B_98618_AAACATCGAGCACCTCCATACCAA	0.298254474	-0.024314926	-0.280736517
B_98618_AAACATCGAGCCATGCTATCAGCA	0.062360787	0.071074639	-0.272893432
B_98618_AAACATCGCCGACAACACACGACC	0.121238729	0.137930029	-0.281080074
B_98618_AAACATCGCCGTGAGAACCACTGT	0.233088354	0.080803365	-0.363661031
B_98618_AAACATCGCCTCTATCACCACTGT	0.246073637	0.080258349	-0.185679417
B_98618_AAACATCGCGCATACAGCGAGTAA	0.381383009	0.183780214	-0.362983698
B_98618_AAACATCGGCGAGTAAAACTCACC	0.007793961	-0.038530206	-0.503740871
B_98618_AAACATCGTGGCTTCAAGTCACTA	-0.012614492	0.036397643	-0.186151896
B_98618_AACAACCAAATCCGTCAGAGTCAA	0.250320485	0.059228532	-0.225459029
B_98618_AACAACCAACAAGCTAGTACGCAA	0.261846645	-0.078042025	-0.050983717
B_98618_AACAACCAACAGCAGAGATGAATC	0.117873263	0.045478832	-0.249731201
B_98618_AACAACCAACATTGGCCTGGCATA	-0.028899625	0.125873249	-0.167830715
B_98618_AACAACCAACATTGGCGGAGAACA	-0.063240766	0.192307595	-0.162422992
B_98618_AACAACCACTAAGGTCCCATCCTC	0.09454935	0.031194157	-0.342862823
B_98618_AACAACCAGATAGACATGGAACAA	0.447970536	0.304910624	-0.330638448

B_98618_AACAACCAGCGAGTAAAACTCACC	0.115412175	0.073847027	-0.015431686
B_98618_AACCGAGAAAGGACACCGAACTTA	0.205080787	0.242106034	-0.089212664
B_98618_AACCGAGAACAGCAGACGAACTTA	0.212940243	-0.061848669	-0.173209863
B_98618_AACCGAGAACCTCCAAAATCCGTC	0.015172753	0.12825886	-0.385662651
B_98618_AACCGAGACCTCCTGAGTACGCAA	0.094343793	0.022663589	-0.196353663
B_98618_AACCGAGACTCAATGAGTCGTAGA	0.310976063	0.024522763	-0.332588048
B_98618_AACCGAGAGATAGACACCTCTATC	-0.018998901	0.098728889	-0.062802894
B_98618_AACCGAGAGATGAATCATAGCGAC	0.415395654	-0.045342326	-0.309894889
B_98618_AACCGAGAGCTCGGTAACTATGCA	0.124728946	-0.003920937	-0.309328882
B_98618_AACCGAGATGAAGAGAAGCAGGAA	0.029282296	0.151122378	-0.311197407
B_98618_AACGCTTAAACGCTTAGACTAGTA	0.016615591	0.187780714	-0.174070228
B_98618_AACGCTTAACACAGAAACCACTGT	0.325853045	0.001999663	-0.162441928
B_98618_AACGCTTACCGACAACGCCACATA	0.124602513	0.021481538	-0.133382818
B_98618_AACGCTTACTGGCATATGGAACAA	0.04911144	0.000313519	-0.321461176
B_98618_AACGCTTAGCCAAGACAAGGTACA	0.245262859	0.06605129	-0.405269008
B_98618_AACGTGATAGATGTACGAGCTGAA	0.117526058	0.01403572	-0.469075632
B_98618_AACGTGATCAGATCTGATTGAGGA	0.326641915	-0.009094981	-0.345671777
B_98618_AACGTGATCCAGTTCACGAACTTA	0.22680024	0.175822489	-0.141509941
B_98618_AACGTGATCCTCTATCGCCACATA	0.155009401	0.19286903	-0.135361602
B_98618_AACGTGATCGACTGGAAACTCACC	0.303252747	0.109553577	0.095074925
B_98618_AACGTGATCGACTGGATGGTGGTA	0.079531067	-0.000395147	-0.307101143
B_98618_AACGTGATCTGGCATACGACACAC	0.163705232	0.119265454	-0.306906529
B_98618_AACGTGATCTGTAGCCGGAGAACA	0.100429427	-0.026978131	-0.462111498
B_98618_AACGTGATGACAGTGCCAAGGAGC	0.220488244	0.018806332	-0.418428383
B_98618_AACGTGATGCCAAGACAAGGTACA	0.006992267	0.159817176	-0.354439314
B_98618_AACGTGATTGGCTTCAGAACAGGC	-0.054799892	0.170116182	-0.165776985
B_98618_AACGTGATTGGTGGTAACAGCAGA	0.487867006	0.111932838	-0.346572509
B_98618_AACGTGATTTCACGCAGGAGAACA	0.194088287	0.096692146	-0.260198195
B_98618_AACTCACCAAACATCGCATCAAGT	0.410362477	0.220492959	-0.289622936
B_98618_AACTCACCAATGTTGCCCGACAAC	0.174000882	0.02687687	-0.280197819
B_98618_AACTCACCAATGTTGCGGTGCGAA	-0.010318069	-0.013289651	-0.314000013
B_98618_AACTCACCAGCAGGAAGAATCTGA	0.183147729	-0.018321298	-0.201385291

B_98618_AACTCACCAGTCACTAAGTCACTA	0.091738721	0.056351672	-0.318403586
B_98618_AACTCACCCAAGACTAGGAGAACA	0.103663198	0.081062375	-0.483245616
B_98618_AACTCACCGAGTTAGCATAGCGAC	0.225440273	0.085546803	-0.28389382
B_98618_AACTCACCGCTAACGAACGTATCA	0.11587974	0.186024584	-0.145842344
B_98618_AACTCACCGCTCGGTAAGTACAAG	0.095143058	0.084252753	-0.404843456
B_98618_AACTCACCGTGTTCTACGCTGATC	0.21517301	0.021395189	-0.35814327
B_98618_AACTCACCTGGTGGTACACTTCGA	0.074127036	0.049312056	-0.120673014
B_98618_AAGACGGAAAACATCGGATGAATC	0.008265827	-0.002027326	-0.324402748
B_98618_AAGACGGAAAGGACACAAGGACAC	0.151465372	0.054770089	-0.284994462
B_98618_AAGACGGAACGTATCACCGTGAGA	0.282359665	0.009906587	-0.201852677
B_98618_AAGACGGAACTATGCAACATTGGC	-0.008514787	0.094627729	-0.085964538
B_98618_AAGACGGAAGCCATGCCAGATCTG	0.196733604	0.046452536	-0.319976515
B_98618_AAGACGGAAGTACAAGCCTCCTGA	0.115445326	0.031899538	-0.331677463
B_98618_AAGACGGACACCTTACTAGGATGA	0.307395986	0.069099248	-0.228800355
B_98618_AAGACGGACAGCGTTAACCACTGT	0.294808168	-0.012497494	-0.330289305
B_98618_AAGACGGACCTAATCCCCTCTATC	-0.033597237	0.209170428	-0.069998868
B_98618_AAGACGGACGAACTTACACCTTAC	0.190532881	0.094320582	-0.287569786
B_98618_AAGACGGAGAATCTGACGACACAC	0.138525132	-0.015696042	-0.274187677
B_98618_AAGACGGAGCTAACGATTCACGCA	0.014312409	0.109711348	-0.132159275
B_98618_AAGACGGATCTTCACAAACTCACC	-0.048686876	-0.029599133	-0.382494936
B_98618_AAGACGGATGAAGAGAGATAGACA	0.22252009	0.013503974	-0.341017456
B_98618_AAGAGATCAAACATCGACGCTCGA	0.341653494	0.042124991	-0.341046186
B_98618_AAGAGATCAACCGAGAATTGAGGA	0.090876282	0.229511768	-0.29735529
B_98618_AAGAGATCACATTGGCGTCTGTCA	0.198714165	0.17190582	-0.212183078
B_98618_AAGAGATCAGATGTACGAGCTGAA	0.195891665	0.033254307	-0.250608054
B_98618_AAGAGATCATTGGCTCCCTCCTGA	0.287813098	0.11418379	-0.28012051
B_98618_AAGAGATCCACTTCGAACATTGGC	0.085764255	0.010267507	-0.216800462
B_98618_AAGAGATCCCTAATCCACGTATCA	-0.004476303	0.080181641	-0.205797569
B_98618_AAGAGATCCGACACACACAGCAGA	0.15540988	-0.055032836	-0.249287641
B_98618_AAGAGATCCTCAATGAGAATCTGA	0.158536406	0.018201296	-0.292413462
B_98618_AAGAGATCGCGAGTAAAGTGGTCA	0.141472753	-0.062765462	-0.279290109
B_98618_AAGAGATCGCTCGGTATGAAGAGA	0.364458619	0.234332658	-0.259289453

B_98618_AAGAGATCTCCGTCTAGACTAGTA	0.343589295	0.644482652	0.049846571
B_98618_AAGAGATCTTCACGCAATTGAGGA	0.29280086	0.137059858	-0.253845607
B_98618_AAGGACACAAACATCGACGTATCA	-0.00150596	0.010630328	-0.33606007
B_98618_AAGGACACAACAACCATCTTCACA	0.279174471	-0.047298122	-0.130886737
B_98618_AAGGACACAGTGGTCATCCGTCTA	0.204879585	0.066024421	-0.331472326
B_98618_AAGGACACATCCTGTAATTGGCTC	0.196954102	-0.006765514	-0.092685122
B_98618_AAGGACACATGCCTAAGTCTGTCA	0.244341847	0.037988403	-0.261093677
B_98618_AAGGACACCTGTAGCCATAGCGAC	0.012849396	-0.001380419	-0.188203583
B_98618_AAGGACACGGAGAACAAGCACCTC	0.094291207	-0.06951256	-0.303894018
B_98618_AAGGACACGGTGCGAATGGTGGTA	0.194144203	-0.026388836	-0.012204002
B_98618_AAGGACACTATCAGCACGGATTGC	0.327280399	-0.032701455	-0.379564518
B_98618_AAGGACACTGGAACAAACACAGAA	0.210992205	0.165697267	-0.269644848
B_98618_AAGGACACTGGCTTCACCAGTTCA	0.128053344	0.142986993	-0.411458698
B_98618_AAGGTACAAACCGAGACACCTTAC	0.088745367	0.155805831	-0.203072005
B_98618_AAGGTACAAAGAGATCTAGGATGA	0.212840291	0.300436741	-0.108182567
B_98618_AAGGTACAAATGTTGCGAGCTGAA	0.347482534	0.024818018	-0.423525598
B_98618_AAGGTACAACTATGCATCCGTCTA	0.454522389	0.052660251	-0.2441503
B_98618_AAGGTACAATCATTCCCGACACAC	0.108470626	0.127183066	-0.38584225
B_98618_AAGGTACACATCAAGTACAGCAGA	-0.008547196	0.092926444	-0.215760116
B_98618_AAGGTACACCATCCTCATTGGCTC	0.273162295	-0.023199483	-0.120200165
B_98618_AAGGTACACCTAATCCTCCGTCTA	0.188316552	0.110878527	-0.027254218
B_98618_AAGGTACACGACTGGAACCTCCAA	0.068060768	0.158192239	-0.250816566
B_98618_AAGGTACACGGATTGCACGTATCA	0.303944841	0.049659531	-0.197249361
B_98618_AAGGTACACTAAGGTCAAGGACAC	0.37850042	0.066266995	-0.21368427
B_98618_AAGGTACAGAACAGGCCCTCTATC	0.191153879	0.089051332	-0.445177589
B_98618_AAGGTACAGATGAATCACAGCAGA	0.016657552	0.051619605	-0.39543333
B_98618_AAGGTACAGCCAAGACCAGCGTTA	-0.027212472	0.128161564	-0.247510306
B_98618_AAGGTACAGCTCGGTAAGAGTCAA	0.219510109	0.173743948	-0.290516367
B_98618_AAGGTACATGGAACAAAGATCGCA	0.32656283	0.120468731	-0.329048958
B_98618_AATCCGTCATAGCGACAAGACGGA	0.094540911	0.157845432	-0.182738458
B_98618_AATCCGTCATCATTCCCAAGGAGC	0.061358516	0.167474941	-0.049291344
B_98618_AATCCGTCCATACCAACAAGACTA	0.024012522	0.168697262	-0.395979809

B_98618_AATCCGTCCGGATTGCACCTCCAA	0.179890611	0.086735357	-0.239909979
B_98618_AATCCGTCCTGGCATACGCATACA	0.071861378	0.071197711	-0.218262764
B_98618_AATCCGTCGAATCTGAAAGACGGA	0.130618818	0.121835891	-0.246208751
B_98618_AATCCGTCTCCGTCTAGACAGTGC	0.053970393	0.082744928	-0.290203364
B_98618_AATGTTGCAAACATCGCATCAAGT	-0.053508021	-0.042655807	-0.31887809
B_98618_AATGTTGCAGCCATGCGCGAGTAA	0.273501992	0.280167822	-0.243446267
B_98618_AATGTTGCATGCCTAACAACCACA	0.274145281	0.159534694	-0.332934135
B_98618_AATGTTGCATTGGCTCCCATCCTC	0.008349769	0.119673859	-0.314989771
B_98618_AATGTTGCCCAGTTCAATCCTGTA	0.368972209	0.06966005	-0.370576107
B_98618_AATGTTGCCTGAGCCAAGATGTAC	0.202840325	-0.001865093	-0.275347911
B_98618_AATGTTGCGAACAGGCCCTCTATC	0.304302947	0.032872058	-0.363385562
B_98618_AATGTTGCGATAGACAAGCCATGC	0.29184524	-0.040069131	-0.371653962
B_98618_AATGTTGCGCCACATAGTCGTAGA	0.213906705	0.07289919	-0.096534251
B_98618_AATGTTGCTGGAACAATAGGATGA	0.185248321	0.079455048	-0.202728051
B_98618_AATGTTGCTGGCTTCACGCTGATC	-0.021950372	0.080594102	-0.291923612
B_98618_ACAAGCTAAACAACCAGATAGACA	0.104750944	0.141997052	-0.398394126
B_98618_ACAAGCTAAGCCATGCCCAGTTCA	0.127607394	0.01449334	-0.17078597
B_98618_ACAAGCTACAGATCTGAGATCGCA	0.214421226	0.146353483	-0.135401193
B_98618_ACAAGCTACTCAATGACGCTGATC	0.094859424	-0.005575966	-0.3392099
B_98618_ACAAGCTACTGGCATAACACGACC	0.277178142	0.109449161	-0.301703662
B_98618_ACAAGCTAGACTAGTAATCATTCC	0.147115557	-0.035972672	-0.147252101
B_98618_ACAAGCTAGTGTTCTACTGTAGCC	0.208067641	0.20589711	-0.143480833
B_98618_ACAAGCTATCCGTCTACCTCCTGA	0.234687693	0.140153453	-0.170011481
B_98618_ACACAGAAAACGTGATCACCTTAC	0.084204128	0.098349493	-0.328229525
B_98618_ACACAGAAATAGCGACATCATTCC	0.144409956	0.090374469	-0.143317996
B_98618_ACACAGAACGGATTGCCGACACAC	0.066310516	0.162806555	-0.330384177
B_98618_ACACAGAACGGATTGCGTCTGTCA	0.261381327	0.168535841	-0.243639007
B_98618_ACACAGAAGAGCTGAACGAACTTA	0.225987361	0.074651515	-0.199414419
B_98618_ACACAGAATCCGTCTAACCTCCAA	0.342578893	0.144894062	-0.202590165
B_98618_ACACAGAATTCACGCACGGATTGC	0.415457434	0.006291134	-0.26307502
B_98618_ACACGACCAACGCTTAACACGACC	0.208663691	0.069900655	-0.281101067
B_98618_ACACGACCAAGGTACAAACGCTTA	0.279160183	0.075899676	-0.300352462

B_98618_ACACGACCACAGCAGAAGAGTCAA	0.04608813	0.049068841	-0.297703868
B_98618_ACACGACCACTATGCAACCACTGT	0.25260539	0.006946485	-0.341577852
B_98618_ACACGACCAGATGTACTGAAGAGA	0.080091368	-0.052040209	-0.406381535
B_98618_ACACGACCAGTACAAGACACGACC	0.144537293	0.021877281	-0.29315948
B_98618_ACACGACCCCAGTTCAACGCTCGA	0.056242586	-0.124367767	-0.540193643
B_98618_ACACGACCCCGTGAGAGGAGAACA	0.202351911	0.174261293	-0.097720153
B_98618_ACAGATTCAAGACGGAACCACTGT	0.186871253	0.138282415	-0.284160136
B_98618_ACAGATTCAGATGTACATCATTCC	0.17725497	0.25553653	0.11812633
B_98618_ACAGATTCATCCTGTAGTACGCAA	0.239422296	0.114120668	-0.384199108
B_98618_ACAGATTCCCGAAGTAACGTATCA	0.076648175	0.097016739	-0.15942577
B_98618_ACAGATTCCCTCCTGATGGAACAA	0.316116045	0.187766923	-0.25409006
B_98618_ACAGATTCCTGGCATAACAAGCTA	0.061823045	0.15208164	-0.1174862
B_98618_ACAGCAGACAAGGAGCCCTAATCC	0.214019619	-0.070694412	-0.398426444
B_98618_ACAGCAGACAAGGAGCCGACTGGA	0.148715774	-0.062108497	-0.405643022
B_98618_ACAGCAGACACTTCGACGACTGGA	0.0225527	0.103779747	-0.151906817
B_98618_ACAGCAGACGACTGGAAAGAGATC	0.033787734	0.044332649	-0.316223942
B_98618_ACAGCAGACTGAGCCACTGGCATA	0.325375736	-0.109578244	-0.344467088
B_98618_ACAGCAGAGAACAGGCCTCAATGA	0.111209296	0.08690167	-0.172160447
B_98618_ACAGCAGAGAGTTAGCCCATCCTC	0.271387321	-0.042417891	-0.340990485
B_98618_ACAGCAGAGAGTTAGCGACTAGTA	0.453426684	0.222612505	-0.440713389
B_98618_ACAGCAGAGAGTTAGCGTACGCAA	0.25739172	0.124293304	-0.123808907
B_98618_ACAGCAGAGGTGCGAACACTTCGA	-0.16294155	0.044845022	-0.166045221
B_98618_ACAGCAGATATCAGCAAGAGTCAA	0.201666983	0.070229992	-0.237906697
B_98618_ACATTGGCAATGTTGCCTAAGGTC	0.149690039	-0.00174443	-0.311830044
B_98618_ACATTGGCACCACTGTCCAGTTCA	0.263793865	0.096750789	-0.157900282
B_98618_ACATTGGCACCACTGTCTGTAGCC	0.346102214	0.052281962	-0.30085232
B_98618_ACATTGGCACTATGCAAGCCATGC	0.245355901	0.128282376	-0.289337354
B_98618_ACATTGGCAGCCATGCCGACACAC	0.165797737	0.090825642	-0.025409021
B_98618_ACATTGGCCAAGACTAACTATGCA	0.242046589	0.016465674	-0.189968216
B_98618_ACATTGGCCTAAGGTCAACTCACC	0.199086407	0.122965479	-0.156717831
B_98618_ACATTGGCCTAAGGTCTTCACGCA	0.20866528	0.029704681	-0.156656621
B_98618_ACATTGGCGAGTTAGCCGAACTTA	0.013867491	0.009283545	-0.339676298

B_98618_ACATTGGCGCGAGTAAAACGCTTA	0.296815467	0.030458442	-0.369819194
B_98618_ACCACTGTAGAGTCAAAAGAGATC	0.058380275	0.038990621	-0.059390881
B_98618_ACCACTGTCATACCAAAACAACCA	0.124106086	0.112691949	-0.110066555
B_98618_ACCACTGTCGAACTTAAACGTGAT	0.120261166	-0.127147504	-0.307124082
B_98618_ACCACTGTCGAACTTAAGATCGCA	0.411598343	0.105215352	-0.09772096
B_98618_ACCACTGTGCTAACGAAAGGTACA	0.137278984	0.161562496	-0.160708174
B_98618_ACCACTGTGGTGCGAACCTCTATC	0.184377248	0.063703513	-0.356851768
B_98618_ACCACTGTGTCGTAGAAACAACCA	0.124503658	0.044704106	-0.26915232
B_98618_ACCACTGTTGGAACAACCGTGAGA	0.103000596	0.08819364	-0.437467543
B_98618_ACCTCCAAAACAACCAAACTCACC	0.247562729	0.014961726	-0.391814927
B_98618_ACCTCCAAAACAACCAAGCACCTC	0.212438544	0.128444387	-0.051274432
B_98618_ACCTCCAAAACCGAGACCGAAGTA	-0.061027148	-0.067999117	-0.398892723
B_98618_ACCTCCAAAAGGTACAGCGAGTAA	-0.040605201	0.014572188	-0.283066764
B_98618_ACCTCCAAACAGCAGAAAGAGATC	0.114379133	0.04128933	-0.153368704
B_98618_ACCTCCAAAGCACCTCCAAGGAGC	0.021918451	0.209251832	-0.206439308
B_98618_ACCTCCAACAAGGAGCCGACACAC	0.086466293	0.135925266	-0.155270963
B_98618_ACCTCCAACATCAAGTCGGATTGC	0.019152103	0.08037795	-0.175780989
B_98618_ACCTCCAACTGGCATACCGTGAGA	0.242479419	0.005598616	-0.212910099
B_98618_ACCTCCAAGCCAAGACGTCTGTCA	0.072095851	0.128059686	-0.134022033
B_98618_ACCTCCAATAGGATGAAGCAGGAA	0.113074682	0.038705082	-0.287740898
B_98618_ACCTCCAATAGGATGACATCAAGT	0.284120914	0.016981132	-0.474938848
B_98618_ACGCTCGAACCTCCAAAATCCGTC	0.420437606	0.109382919	-0.275409903
B_98618_ACGCTCGAACCTCCAAAGTCACTA	0.11105307	0.092664761	-0.415068496
B_98618_ACGCTCGAATTGAGGAACAAGCTA	0.142683735	-0.051387482	-0.202898775
B_98618_ACGCTCGACCTCCTGATGGTGGTA	0.136717906	-0.019550057	-0.325517268
B_98618_ACGCTCGAGCCAAGACGCCACATA	0.123480629	0.044561476	-0.273036099
B_98618_ACGCTCGAGTCTGTCATCTTCACA	0.23038804	-0.034007	-0.355816208
B_98618_ACGCTCGATCTTCACAACACAGAA	0.201664622	0.171169949	-0.409659782
B_98618_ACGCTCGATGGAACAAGCTCGGTA	0.297372804	0.170564012	-0.093905498
B_98618_ACGCTCGATGGTGGTACCATCCTC	0.146766234	0.197013277	-0.153769067
B_98618_ACGTATCAAACTCACCCGACTGGA	0.091285468	0.304655473	-0.238731051
B_98618_ACGTATCAAATGTTGCAAACATCG	0.319738499	0.13682983	-0.139737736

B_98618_ACGTATCAACAGCAGACGAACTTA	0.343494495	0.026045005	-0.332107025
B_98618_ACGTATCAACCACTGTCTAAGGTC	0.096413068	-0.037822951	-0.43897679
B_98618_ACGTATCAAGCACCTCTTCACGCA	0.434984594	0.098120976	-0.443936986
B_98618_ACGTATCACCGTGAGACGGATTGC	0.218278881	0.206298099	-0.285289551
B_98618_ACGTATCACCTCTATCAGATGTAC	0.036844058	-0.182315061	-0.492261832
B_98618_ACGTATCACGCATACACTGGCATA	0.32684989	-0.058736579	-0.353395927
B_98618_ACGTATCAGTCGTAGACCATCCTC	0.046372784	-0.048816149	-0.374140858
B_98618_ACGTATCATGGAACAAACTATGCA	0.125918626	0.077784849	-0.186496192
B_98618_ACTATGCAAAACATCGAGATGTAC	0.210332854	0.108472891	-0.042928721
B_98618_ACTATGCAAATCCGTCGACTAGTA	0.404651224	0.020278143	-0.434061406
B_98618_ACTATGCAATCCTGTAATAGCGAC	-0.012603514	0.167054024	-0.048278506
B_98618_ACTATGCACAACCACACGAACTTA	0.113226158	0.122183781	-0.359280055
B_98618_ACTATGCACAATGGAAGTCTGTCA	0.001325187	-0.002649424	-0.423708046
B_98618_ACTATGCACCATCCTCATCATTCC	0.363191212	-0.004467766	-0.393467152
B_98618_AGAGTCAAACACGACCAACTCACC	0.269731396	0.053390555	-0.388096362
B_98618_AGAGTCAAATCCTGTATCCGTCTA	0.218176105	0.092088782	-0.256469469
B_98618_AGAGTCAACAAGACTACGAACTTA	0.160313358	0.048245126	-0.322638191
B_98618_AGAGTCAACAATGGAAAGATGTAC	0.025611565	-0.019983605	-0.262327177
B_98618_AGAGTCAACAGATCTGCTAAGGTC	0.297849914	0.087279834	-0.261934532
B_98618_AGAGTCAACCAGTTCACAATGGAA	0.127096296	0.037722602	-0.242935453
B_98618_AGAGTCAACCTCCTGACCATCCTC	-0.007618411	-0.10598139	-0.379259289
B_98618_AGAGTCAACTCAATGAACACGACC	-0.017285193	0.179955779	-0.400905853
B_98618_AGAGTCAACTGTAGCCCCTAATCC	0.184854344	-0.037408226	-0.324966644
B_98618_AGAGTCAAGCGAGTAAACACGACC	0.076184658	0.139339896	-0.222558957
B_98618_AGAGTCAAGCGAGTAAACTATGCA	0.128611946	0.032756334	-0.25043001
B_98618_AGAGTCAAGCTCGGTATGGCTTCA	0.2133913	0.074053939	-0.074313124
B_98618_AGAGTCAAGGAGAACAATTGAGGA	0.239370302	0.112180465	-0.381774653
B_98618_AGAGTCAATGGCTTCAACAGATTC	0.218690969	0.101467972	-0.30014505
B_98618_AGAGTCAATGGCTTCACCAGTTCA	0.047846127	0.001027443	-0.285353428
B_98618_AGATCGCAAAACATCGAGTACAAG	0.220790491	0.015692517	-0.115318149
B_98618_AGATCGCAACGTATCATATCAGCA	0.291555062	-0.030859775	-0.195387147
B_98618_AGATCGCAAGTCACTAGCTAACGA	0.149307381	0.001078451	-0.202246809

B_98618_AGATCGCAAGTGGTCAAATGTTGC	0.09414825	0.10236786	-0.188860917
B_98618_AGATCGCACCGTGAGAACAGCAGA	0.136754473	0.072851084	-0.314538464
B_98618_AGATCGCACGACTGGAACACGACC	0.003893836	0.022962405	-0.335049217
B_98618_AGATCGCACTGTAGCCAGCAGGAA	0.013573869	0.068799241	0.110227461
B_98618_AGATCGCACTGTAGCCGAACAGGC	0.061634223	0.017907795	-0.282755097
B_98618_AGATCGCAGAACAGGCAAGGTACA	0.113873419	-0.057387563	-0.36172698
B_98618_AGATCGCATATCAGCAGATAGACA	0.283090236	-0.011546256	-0.236497953
B_98618_AGATCGCATGGCTTCACGCTGATC	0.421803839	0.008069303	-0.370709006
B_98618_AGATCGCATTCACGCACACCTTAC	0.106337569	-0.010038717	-0.13402519
B_98618_AGATGTACAACCGAGAGCGAGTAA	0.469188095	-0.087202695	-0.272727639
B_98618_AGATGTACAAGGACACAGAGTCAA	0.183549869	0.219654175	-0.248024704
B_98618_AGATGTACACACAGAAACCACTGT	0.412813218	0.062141388	-0.295789509
B_98618_AGATGTACACACAGAACTCAATGA	0.364316384	0.057157899	-0.223380365
B_98618_AGATGTACACAGCAGACGCATACA	0.118087699	0.019157678	-0.323029192
B_98618_AGATGTACCAACCACAGAACAGGC	0.191932937	0.127927718	-0.203252648
B_98618_AGATGTACCATCAAGTTTCACGCA	0.08262559	0.137199157	0.046136886
B_98618_AGATGTACCCTCCTGAAGTGGTCA	0.354233913	-0.139578103	-0.288053712
B_98618_AGATGTACCCTCCTGAGTCGTAGA	0.081097139	0.072624847	-0.383297851
B_98618_AGATGTACCGAACTTAGGAGAACA	-0.038863264	0.080413878	-0.279774811
B_98618_AGCACCTCACAGCAGACCGTGAGA	0.160479823	0.047992941	-0.266782674
B_98618_AGCACCTCAGCACCTCAGTCACTA	0.360425816	0.033321151	-0.347399746
B_98618_AGCACCTCAGTCACTAAAGGACAC	-0.071050553	-0.010007987	-0.318722095
B_98618_AGCACCTCATGCCTAACCGTGAGA	0.187050328	0.099993216	-0.15119884
B_98618_AGCACCTCATTGAGGACGCATACA	-0.059308133	-0.057398647	-0.392067163
B_98618_AGCACCTCATTGGCTCACAGATTC	0.010339205	0.034562466	0.009508323
B_98618_AGCACCTCCCAGTTCATGGCTTCA	0.063342916	0.134507079	-0.375011937
B_98618_AGCACCTCCCTCTATCAGTCACTA	0.445755929	0.282026075	-0.37123118
B_98618_AGCACCTCCTGAGCCAACACGACC	-0.158208262	0.114039214	-0.11953182
B_98618_AGCACCTCCTGTAGCCAGCAGGAA	0.129127275	0.021031642	-0.437162706
B_98618_AGCACCTCCTGTAGCCATGCCTAA	0.053037757	0.266253737	-0.148353624
B_98618_AGCACCTCCTGTAGCCGAGCTGAA	0.050117659	0.068281386	-0.284597947
B_98618_AGCACCTCGCTCGGTAGTGTTCTA	0.011210524	0.095519981	-0.394003908

B_98618_AGCACCTCTCTTCACACCTCTATC	0.210656793	-0.069504083	-0.40483083
B_98618_AGCACCTCTGAAGAGAGTCGTAGA	0.182214784	0.067822183	-0.379649779
B_98618_AGCAGGAAAACGCTTAGCTAACGA	0.125126331	-0.06104512	-0.300030038
B_98618_AGCAGGAAAGATGTACCAAGACTA	0.159778833	0.112077957	-0.297540667
B_98618_AGCAGGAAAGTCACTAAACGCTTA	-0.123088197	-0.021456963	-0.380967253
B_98618_AGCAGGAAAGTCACTATGGCTTCA	0.135772478	0.070136351	-0.309363618
B_98618_AGCAGGAACCGAAGTATGAAGAGA	0.103759462	0.196344293	-0.26754983
B_98618_AGCAGGAACCGTGAGAGAGTTAGC	0.120243955	-0.025590647	-0.16049069
B_98618_AGCAGGAACCTCCTGATGGAACAA	0.261193728	-0.019105571	-0.217589848
B_98618_AGCAGGAACTCAATGAGAATCTGA	0.15922518	-0.011574927	-0.141860648
B_98618_AGCAGGAAGATGAATCAAACATCG	0.050037679	0.083659845	-0.191360243
B_98618_AGCAGGAAGTCGTAGACATACCAA	0.224711698	0.084786331	-0.212812663
B_98618_AGCAGGAAGTCTGTCATCCGTCTA	0.085109285	0.13908955	0.05032487
B_98618_AGCAGGAAGTGTTCTAATCCTGTA	0.182251568	0.017654261	-0.204698918
B_98618_AGCAGGAATCTTCACACCGAAGTA	0.011362284	0.06689864	-0.455125773
B_98618_AGCCATGCAACTCACCTGGCTTCA	0.268997541	-0.038154282	-0.445003936
B_98618_AGCCATGCACACGACCATAGCGAC	-0.015906865	0.036921619	-0.089574321
B_98618_AGCCATGCAGCCATGCCGAACTTA	0.12690293	0.051235629	-0.258098728
B_98618_AGCCATGCATCATTCCGACTAGTA	0.191325209	0.062502717	-0.189863972
B_98618_AGCCATGCCCGAAGTACAAGGAGC	0.323458532	0.130234297	-0.244024459
B_98618_AGCCATGCCCGAAGTACTGAGCCA	0.312627967	0.171996356	-0.220322324
B_98618_AGCCATGCCCTAATCCAACGCTTA	0.133530343	0.110359149	-0.080779884
B_98618_AGCCATGCCCTAATCCAAGGTACA	0.057849136	0.07269605	-0.112441886
B_98618_AGCCATGCCCTCCTGAACACGACC	0.127651299	-0.00651047	-0.125529628
B_98618_AGCCATGCGAATCTGAATAGCGAC	0.009363819	0.00705749	-0.440541255
B_98618_AGCCATGCGAGCTGAAATCCTGTA	0.413180126	0.11863041	-0.243237055
B_98618_AGCCATGCGGTGCGAAGCCACATA	0.220256213	0.066360305	-0.283339399
B_98618_AGCCATGCGTCTGTCAAACCGAGA	0.318546558	0.044139602	-0.415402144
B_98618_AGCCATGCTAGGATGAACCTCCAA	0.43009108	-0.052811485	-0.294370601
B_98618_AGCCATGCTGGCTTCACGACACAC	0.188360385	0.077887766	-0.303813661
B_98618_AGCCATGCTGGTGGTACATACCAA	0.538930174	0.328818965	-0.078347245
B_98618_AGGCTAACACGTATCACATCAAGT	0.101406986	0.123031477	-0.420267346

B_98618_AGGCTAACAGATCGCAAAGACGGA	0.236601537	0.027448312	-0.383475201
B_98618_AGGCTAACAGCCATGCATCATTCC	0.516219231	0.054604239	-0.427050039
B_98618_AGGCTAACCACCTTACACAGCAGA	0.100182354	-0.078938866	-0.415055026
B_98618_AGGCTAACCCATCCTCACAGCAGA	0.305361258	0.115993048	-0.232239184
B_98618_AGGCTAACCCGAAGTAAGCAGGAA	0.205262275	0.046179116	-0.331722078
B_98618_AGGCTAACCCTCCTGAATGCCTAA	0.183124096	-0.112828623	-0.368024162
B_98618_AGGCTAACCTGGCATAGCTCGGTA	0.07240315	-0.020741992	-0.243960349
B_98618_AGGCTAACGGTGCGAAGATGAATC	0.11750052	0.031606155	-0.353970262
B_98618_AGGCTAACGTCGTAGATGAAGAGA	0.272461476	0.017776723	-0.319134742
B_98618_AGGCTAACTCCGTCTAGAACAGGC	0.142498958	0.14989128	-0.374763193
B_98618_AGTACAAGAACGCTTAAACCGAGA	0.077454001	0.039083383	-0.348584767
B_98618_AGTACAAGAATGTTGCCTCAATGA	0.125419251	0.111481375	-0.35902994
B_98618_AGTACAAGACAAGCTATCTTCACA	0.177569755	-0.226464306	-0.310654058
B_98618_AGTACAAGACAGATTCAGAGTCAA	0.180428181	0.11733603	-0.147259022
B_98618_AGTACAAGACAGATTCAGCACCTC	0.19821771	0.113454853	-0.333748027
B_98618_AGTACAAGACCTCCAACCAGTTCA	0.175767519	0.078115179	-0.434887896
B_98618_AGTACAAGACCTCCAACCGTGAGA	0.2585112	0.010684732	-0.406968866
B_98618_AGTACAAGACGCTCGAAAACATCG	-0.004213035	-0.037924768	-0.435720622
B_98618_AGTACAAGACGCTCGACTAAGGTC	0.142213535	0.121764415	-0.262735022
B_98618_AGTACAAGACTATGCACTGGCATA	0.294580152	0.027214956	-0.311700387
B_98618_AGTACAAGAGCAGGAAAAGACGGA	0.284543082	0.280120235	-0.178973564
B_98618_AGTACAAGAGTGGTCACGCATACA	0.029721313	0.188531972	-0.213559123
B_98618_AGTACAAGCAATGGAAAGGCTAAC	0.087986715	-0.036056597	-0.271983868
B_98618_AGTACAAGCATCAAGTAAGGTACA	0.092936115	0.033906031	-0.404496063
B_98618_AGTACAAGCCGAAGTACCGACAAC	0.196718291	0.094554894	-0.394979255
B_98618_AGTACAAGCCTCCTGAAAGGACAC	-0.043236021	0.262039859	-0.203155728
B_98618_AGTACAAGCGCATACACACTTCGA	0.134263242	0.075821634	-0.199378551
B_98618_AGTACAAGCGCTGATCCCATCCTC	0.305521889	0.17357276	-0.396678659
B_98618_AGTACAAGCGGATTGCGACTAGTA	0.252440597	0.279088155	-0.097398266
B_98618_AGTACAAGGAATCTGATCTTCACA	0.147165189	0.031705057	-0.318951501
B_98618_AGTACAAGGAGCTGAATTCACGCA	0.23959028	-0.052587513	-0.249478711
B_98618_AGTACAAGGATAGACACTGAGCCA	0.328527183	0.042926455	-0.340134111

B_98618_AGTACAAGGCCAAGACGTCGTAGA	0.188131257	0.107835436	-0.222783885
B_98618_AGTACAAGGGAGAACACCATCCTC	0.002469642	0.040788481	-0.266269265
B_98618_AGTCACTAAATCCGTCGAGCTGAA	0.340307237	0.068887824	-0.342966726
B_98618_AGTCACTAACAGATTCTGAAGAGA	0.205184278	0.1873057	-0.132841797
B_98618_AGTCACTAAGTGGTCACAACCACA	0.193626249	0.134006252	-0.281000814
B_98618_AGTCACTACAAGACTATGGCTTCA	0.08843716	0.102764191	-0.065339804
B_98618_AGTCACTACACCTTACAGTCACTA	0.141496516	-0.014439649	-0.413421863
B_98618_AGTCACTACGGATTGCACACGACC	0.159715283	0.081125384	-0.315910996
B_98618_AGTCACTAGATGAATCACCTCCAA	0.014522721	0.178697673	-0.09638672
B_98618_AGTCACTATGGTGGTAGCCACATA	0.210713705	0.208078395	-0.281418787
B_98618_AGTGGTCAACACGACCCACCTTAC	0.14516033	0.118661238	-0.191148446
B_98618_AGTGGTCAACAGATTCAGTACAAG	0.128140851	0.097054438	-0.364170039
B_98618_AGTGGTCAAGCCATGCAGCCATGC	0.079930284	-0.088366983	-0.199056015
B_98618_AGTGGTCACAAGGAGCGTGTTCTA	0.363476176	0.064547389	-0.223134073
B_98618_AGTGGTCACACTTCGAATTGAGGA	0.054598738	0.184231441	-0.331103319
B_98618_AGTGGTCACCTCCTGACATCAAGT	0.284552506	-0.062692107	-0.358192428
B_98618_AGTGGTCACTGTAGCCCGCTGATC	0.068496972	-0.036955013	-0.358830972
B_98618_AGTGGTCAGAGTTAGCTGGTGGTA	0.111963241	0.094997573	-0.280416899
B_98618_AGTGGTCAGCTCGGTACCAGTTCA	0.075258418	0.034071538	-0.336530756
B_98618_AGTGGTCAGGAGAACACGACTGGA	0.01192522	0.035397803	-0.227329631
B_98618_ATAGCGACAACAACCATGGTGGTA	0.356746325	-0.049689277	-0.074712949
B_98618_ATAGCGACAACGTGATGATAGACA	0.073341714	0.068174185	-0.155820671
B_98618_ATAGCGACACACAGAACTAAGGTC	0.192552504	0.102623922	-0.232524637
B_98618_ATAGCGACACACGACCTAGGATGA	0.12625531	0.190316621	-0.124073553
B_98618_ATAGCGACAGCACCTCCCTCTATC	0.348674664	-0.067177121	-0.280876458
B_98618_ATAGCGACAGGCTAACACGTATCA	-0.042786844	0.014625251	-0.188103145
B_98618_ATAGCGACAGTACAAGCTGTAGCC	0.125630813	0.079411718	-0.141107187
B_98618_ATAGCGACATCATTCCACAGCAGA	0.155807608	0.025320667	-0.272554151
B_98618_ATAGCGACATGCCTAAAACGTGAT	0.202675411	-0.031056436	-0.193818104
B_98618_ATAGCGACCATCAAGTACACAGAA	0.070813172	0.060252036	-0.163771908
B_98618_ATAGCGACCCTAATCCTAGGATGA	0.257123066	0.097581279	-0.08061224
B_98618_ATAGCGACGAGCTGAAAAGAGATC	0.266028872	0.098070299	-0.017023472

B_98618_ATAGCGACGGAGAACACCGACAAC	0.285720586	0.052861868	-0.236042377
B_98618_ATAGCGACTGGAACAAACAAGCTA	0.030741321	0.027478961	-0.395565979
B_98618_ATAGCGACTTCACGCAAACCGAGA	0.404118693	-0.076698535	-0.398043418
B_98618_ATAGCGACTTCACGCAACTATGCA	0.133706741	0.110185517	-0.343517551
B_98618_ATCATTCCAAACATCGTAGGATGA	0.406143015	0.38090398	-0.336806883
B_98618_ATCATTCCAACGCTTAGGTGCGAA	0.327034452	-0.030665187	-0.447226591
B_98618_ATCATTCCAACGTGATTGGCTTCA	0.094269296	-0.068025204	-0.083416666
B_98618_ATCATTCCAGTACAAGAGGCTAAC	0.364061645	0.015028615	-0.279138331
B_98618_ATCATTCCATTGGCTCCTAAGGTC	0.166143499	0.056270948	-0.097500915
B_98618_ATCATTCCCAATGGAAAGATCGCA	0.192270206	-0.13722215	-0.426464874
B_98618_ATCATTCCCCGAAGTAAGTACAAG	0.105972769	0.114675623	-0.035355398
B_98618_ATCATTCCCTGGCATACGCATACA	0.210459522	0.085673063	-0.335276537
B_98618_ATCATTCCGACAGTGCTGGCTTCA	0.121344126	0.083138729	-0.014017152
B_98618_ATCATTCCGAGCTGAAATTGAGGA	0.149661955	0.024017261	-0.202968946
B_98618_ATCATTCCGCTCGGTACCTCTATC	0.043570538	-0.137587826	-0.415213862
B_98618_ATCATTCCGGTGCGAATATCAGCA	0.192389266	0.026371712	-0.30220591
B_98618_ATCCTGTAAAGAGATCCTCAATGA	-0.009571394	-0.026709354	-0.322604504
B_98618_ATCCTGTAAATGTTGCCCAGTTCA	0.15704743	0.227883344	-0.281276224
B_98618_ATCCTGTAACACAGAAAAGGACAC	0.474507135	0.339943924	-0.19169721
B_98618_ATCCTGTAACCTCCAACGAACTTA	0.060635687	0.176623388	-0.211175034
B_98618_ATCCTGTAATGCCTAAAGTACAAG	0.264694237	0.090139657	-0.197328205
B_98618_ATCCTGTAATGCCTAACAAGGAGC	0.184595302	0.045324043	-0.296254349
B_98618_ATCCTGTACATCAAGTAGATCGCA	0.269292458	0.123210131	-0.085803787
B_98618_ATCCTGTAGAATCTGACAAGACTA	0.133286947	0.038252739	-0.175123115
B_98618_ATCCTGTAGAGCTGAAAACTCACC	0.14339854	0.037277823	-0.390515076
B_98618_ATCCTGTAGCCACATAACAGCAGA	0.173579119	0.157663557	-0.32584437
B_98618_ATGCCTAAAACGCTTAAAGAGATC	0.089839344	-0.006177318	-0.208049285
B_98618_ATGCCTAAAACTCACCATTGAGGA	0.070277276	0.133691602	-0.061751627
B_98618_ATGCCTAAAGATCGCAGCTCGGTA	0.332827764	0.193541996	-0.283317182
B_98618_ATGCCTAAATGCCTAACTCAATGA	0.13723891	0.098682984	-0.163394066
B_98618_ATGCCTAACCGTGAGAAGTCACTA	0.156031967	0.166776581	-0.168956035
B_98618_ATGCCTAACTGAGCCACGCATACA	0.13114	0.064108908	-0.315981355

B_98618_ATGCCTAAGAGCTGAATGGCTTCA	-0.038411796	-0.053130713	-0.288875355
B_98618_ATGCCTAAGATAGACACAAGGAGC	-0.000308254	0.162431934	-0.188514451
B_98618_ATGCCTAAGTACGCAACCGAAGTA	0.179970641	0.153184441	-0.128271065
B_98618_ATGCCTAAGTCTGTCACGACACAC	0.309983795	0.062278251	-0.316266618
B_98618_ATGCCTAATATCAGCACCGAAGTA	0.174389798	0.151667456	-0.128406105
B_98618_ATGCCTAATGGAACAAGATAGACA	0.236535795	0.078353293	-0.127558831
B_98618_ATTGAGGAACAGCAGAATCATTCC	0.193463157	0.129391812	-0.242061154
B_98618_ATTGAGGAACATTGGCACCACTGT	-0.000931082	0.052615439	-0.159474513
B_98618_ATTGAGGACAAGACTAATGCCTAA	0.110412976	-0.057398508	-0.306674342
B_98618_ATTGAGGACAGATCTGTGGCTTCA	0.144996296	-0.07366038	-0.46397297
B_98618_ATTGAGGACCATCCTCTGGAACAA	-0.089097349	0.020077228	-0.254917703
B_98618_ATTGAGGACGACACACCCATCCTC	0.278264992	0.016035994	-0.184449794
B_98618_ATTGAGGACGACTGGACAACCACA	0.031744696	0.123962471	-0.325961826
B_98618_ATTGAGGACTGGCATAACAAGCTA	-0.015911733	-0.062962805	-0.190788106
B_98618_ATTGAGGAGACTAGTAGGAGAACA	0.203165016	-0.01292629	-0.321099111
B_98618_ATTGAGGAGAGCTGAAACATTGGC	0.181322912	0.089647873	-0.260877102
B_98618_ATTGAGGAGAGTTAGCCAAGGAGC	0.020597459	0.105464774	-0.31889319
B_98618_ATTGAGGATAGGATGAAAGGTACA	0.017978857	-0.028655263	-0.037476489
B_98618_ATTGAGGATGGAACAAAGATCGCA	-0.036147306	-0.047799522	-0.28658947
B_98618_ATTGAGGATTCACGCATCCGTCTA	0.184545991	0.06147333	-0.204415218
B_98618_ATTGGCTCAAACATCGGATAGACA	0.159070995	0.069018554	-0.380808198
B_98618_ATTGGCTCAACGCTTACCGACAAC	0.14192138	0.101433848	-0.329085137
B_98618_ATTGGCTCACATTGGCACAGCAGA	0.090633764	-0.06404003	-0.304050089
B_98618_ATTGGCTCACTATGCACTCAATGA	0.207729554	0.026617174	-0.43220227
B_98618_ATTGGCTCAGCCATGCAAGACGGA	0.162160967	0.124155556	-0.227354158
B_98618_ATTGGCTCCCTCCTGAACATTGGC	0.368197398	-0.054631805	-0.367126663
B_98618_ATTGGCTCCCTCCTGACGAACTTA	0.081392653	-0.044024381	-0.396927988
B_98618_ATTGGCTCCGAACTTAGAGCTGAA	0.154529549	-0.077586417	-0.338688516
B_98618_ATTGGCTCCTGTAGCCGTACGCAA	0.140901801	0.052377995	-0.184420986
B_98618_ATTGGCTCGAATCTGAAACGCTTA	0.020390185	-0.00798059	-0.199343855
B_98618_ATTGGCTCGGTGCGAAATGCCTAA	0.221563709	0.070886011	-0.193080656
B_98618_ATTGGCTCGTCGTAGAGATAGACA	0.166909846	0.093278722	-0.363420802

B_98618_ATTGGCTCTCTTCACACTAAGGTC	0.245176314	-0.037961705	-0.248710976
B_98618_CAACCACAAAGACGGAAGCACCTC	0.155576865	0.031954445	-0.381574525
B_98618_CAACCACAACAAGCTACGGATTGC	0.256776515	0.152619189	-0.395331084
B_98618_CAACCACAACGCTCGACCTCTATC	0.150064876	-0.060723954	-0.363667328
B_98618_CAACCACACAAGACTAAGCAGGAA	-0.037616968	0.033248502	-0.308714858
B_98618_CAACCACACCATCCTCACACAGAA	0.239367976	-0.032640753	-0.423973608
B_98618_CAACCACACGCTGATCAACTCACC	0.084497652	0.061156284	-0.419173542
B_98618_CAAGACTAAAGAGATCACAGCAGA	0.129396224	-0.023154465	-0.245821484
B_98618_CAAGACTAAAGAGATCACCTCCAA	0.230721334	0.164466864	-0.195811927
B_98618_CAAGACTACATACCAAACAGATTC	0.406922879	-0.000603487	-0.304059789
B_98618_CAAGACTACCTCCTGAACACAGAA	0.282422268	0.130035521	-0.217005653
B_98618_CAAGACTACGACTGGAGACTAGTA	0.18366411	0.081729139	-0.415563505
B_98618_CAAGACTACGCATACATGGAACAA	-0.034286588	0.041633115	-0.215305508
B_98618_CAAGACTAGAACAGGCGTCGTAGA	0.279905795	0.217797083	-0.235525351
B_98618_CAAGACTAGAGCTGAACGACACAC	0.042515881	0.200180834	-0.452270653
B_98618_CAAGACTATGAAGAGACAAGACTA	0.219082906	0.023874856	-0.122411447
B_98618_CAAGGAGCAAACATCGGGTGCGAA	0.239148032	0.065153809	-0.404701984
B_98618_CAAGGAGCAACAACCACAACCACA	-0.180928867	-0.002663379	-0.417398051
B_98618_CAAGGAGCAAGGTACAAGGCTAAC	0.252336452	0.121254691	-0.147379483
B_98618_CAAGGAGCACACAGAACCTCCTGA	0.100967636	0.005101373	-0.019021918
B_98618_CAAGGAGCATCATTCCATGCCTAA	0.216202332	0.059862355	-0.318640937
B_98618_CAAGGAGCATGCCTAAGCCAAGAC	0.152196064	0.221149615	-0.318626979
B_98618_CAAGGAGCCACCTTACGCTCGGTA	0.200795588	-0.033805617	-0.172495284
B_98618_CAAGGAGCGAACAGGCCTGGCATA	0.286816433	0.068730949	-0.457405464
B_98618_CAAGGAGCGAGTTAGCGGAGAACA	0.101332137	-0.124547654	-0.25863285
B_98618_CAAGGAGCTAGGATGAAATGTTGC	-0.05142633	0.036585692	-0.294359971
B_98618_CAAGGAGCTGGCTTCAAGTGGTCA	0.057872694	0.073883537	-0.162133652
B_98618_CAATGGAAAACTCACCGGTGCGAA	0.490353046	0.002058171	-0.414498776
B_98618_CAATGGAAACACAGAAAACGTGAT	0.222668081	0.094479618	-0.315849554
B_98618_CAATGGAAACTATGCACACTTCGA	0.237920896	0.043155863	-0.207833517
B_98618_CAATGGAAAGTCACTAACACGACC	-0.019529813	0.085599515	-0.436581794
B_98618_CAATGGAACAACCACAATAGCGAC	0.264886403	0.020252097	-0.333890908

B_98618_CAATGGAACCATCCTCTCCGTCTA	0.261770945	0.206817459	-0.322112981
B_98618_CAATGGAACCGACAACCCGTGAGA	0.228833657	0.010368992	-0.43675764
B_98618_CAATGGAACCTCCTGACACCTTAC	0.157143768	0.26255032	0.091921117
B_98618_CAATGGAACGAACTTACAAGACTA	-0.023862382	0.021052944	-0.430381838
B_98618_CAATGGAACGACTGGACGCATACA	0.362029302	-0.014970913	-0.321963408
B_98618_CAATGGAAGCCACATATCCGTCTA	0.398642795	0.190971735	-0.173345059
B_98618_CAATGGAAGCTAACGACCAGTTCA	-0.127753959	0.095328748	-0.216245188
B_98618_CAATGGAATGGTGGTACTGAGCCA	0.090744964	0.206107615	-0.33576591
B_98618_CAATGGAATTCACGCAATGCCTAA	0.484646413	0.026838101	-0.414475928
B_98618_CACCTTACAACGCTTAACACAGAA	0.087394892	0.081021258	-0.424819025
B_98618_CACCTTACAAGAGATCAATCCGTC	0.260257242	0.034290995	-0.461626758
B_98618_CACCTTACACAGATTCGCCACATA	0.208476805	0.086724989	-0.172811702
B_98618_CACCTTACAGCACCTCAATCCGTC	0.097696687	0.082227756	0.091351384
B_98618_CACCTTACATAGCGACCGACTGGA	0.094020792	-0.008225951	-0.438373072
B_98618_CACCTTACATTGAGGAAACAACCA	0.137675426	0.025383747	-0.340433587
B_98618_CACCTTACATTGAGGAACCTCCAA	0.130888664	-0.115918554	-0.391007457
B_98618_CACCTTACCCGACAACCTGTAGCC	0.420039402	0.116905062	-0.235675628
B_98618_CACCTTACCTAAGGTCCACCTTAC	0.095991477	0.07221267	-0.280199269
B_98618_CACCTTACCTCAATGAACAGATTC	0.213676486	-0.068030552	-0.285395797
B_98618_CACCTTACGAGCTGAATCTTCACA	-0.034599268	0.047017293	-0.100911544
B_98618_CACCTTACGCCAAGACCGCTGATC	0.143613718	0.039273676	-0.143983653
B_98618_CACCTTACGCGAGTAAGGAGAACA	0.296353594	0.060191464	-0.26589037
B_98618_CACCTTACGCTCGGTACGCTGATC	0.067147543	0.029046228	-0.303050731
B_98618_CACCTTACTGAAGAGACAAGGAGC	0.078718178	0.117477167	-0.446479009
B_98618_CACCTTACTTCACGCAAGTACAAG	0.23502391	0.047616708	-0.427404541
B_98618_CACTTCGAAACCGAGAACACAGAA	0.346305948	0.295144969	-0.243674136
B_98618_CACTTCGAAACGTGATTGGCTTCA	0.146960812	0.149051451	-0.338750073
B_98618_CACTTCGAACAAGCTAGCCACATA	-0.009366739	-0.036673152	-0.420368907
B_98618_CACTTCGAACACAGAAGTACGCAA	0.141942073	0.094519049	0.061827839
B_98618_CACTTCGAACGCTCGAAGATGTAC	0.205610838	0.044215524	-0.33679299
B_98618_CACTTCGAAGAGTCAACACTTCGA	-0.009800843	0.067307804	-0.27951915
B_98618_CACTTCGAAGATCGCAACAGATTC	0.029755975	0.03778263	-0.254791641

B_98618_CACTTCGAATAGCGACCAGCGTTA	0.121495866	-0.021909583	-0.236005319
B_98618_CACTTCGAATCCTGTACAAGACTA	0.121802457	0.127071206	-0.017992446
B_98618_CACTTCGACAAGACTAAGTCACTA	0.163926906	0.005955648	-0.350468377
B_98618_CACTTCGACACCTTACGACTAGTA	0.122728678	0.079011087	-0.184857634
B_98618_CACTTCGACCGACAACAGATGTAC	0.181538019	0.277160637	-0.318914628
B_98618_CACTTCGACGCTGATCCTGTAGCC	0.064286542	-0.041883899	-0.149425366
B_98618_CACTTCGAGAATCTGACCATCCTC	0.168744045	-0.002804713	-0.28299154
B_98618_CACTTCGAGGTGCGAACGACACAC	0.138779324	0.109625819	-0.324438604
B_98618_CACTTCGAGTCTGTCACTAAGGTC	0.065212816	0.118801673	-0.315737207
B_98618_CACTTCGAGTGTTCTACCATCCTC	0.183427749	-0.032979475	-0.392293122
B_98618_CACTTCGATCCGTCTAGCTAACGA	0.038692378	0.028909018	-0.411430756
B_98618_CACTTCGATGAAGAGAATCATTCC	0.218953212	0.121654493	-0.210868197
B_98618_CAGATCTGACCACTGTACAAGCTA	0.358185537	0.031553463	-0.337047493
B_98618_CAGATCTGACCTCCAATAGGATGA	0.361136676	0.189307892	-0.058695192
B_98618_CAGATCTGAGAGTCAAGAACAGGC	0.054044342	0.056451136	-0.20358985
B_98618_CAGATCTGCAACCACAACTATGCA	0.318180429	0.078712347	-0.217502729
B_98618_CAGATCTGCAAGGAGCAGCAGGAA	0.240042688	0.142657882	-0.065718904
B_98618_CAGATCTGCAAGGAGCGCTAACGA	0.092685872	0.03598335	-0.321622249
B_98618_CAGATCTGCCTCTATCGATGAATC	0.213568456	-0.036547567	-0.304681131
B_98618_CAGATCTGGAGCTGAAGTCGTAGA	-0.027325532	0.102500999	-0.314701218
B_98618_CAGATCTGGATGAATCATTGAGGA	0.41748477	-0.081886112	-0.421578574
B_98618_CAGATCTGGCTCGGTAAGCACCTC	0.127279516	0.06060886	-0.119067636
B_98618_CAGATCTGTCTTCACACAAGGAGC	0.231928393	0.088595432	-0.290469718
B_98618_CAGCGTTAAATCCGTCCAGATCTG	0.077817358	0.10004323	-0.170060748
B_98618_CAGCGTTAACAGCAGACCTCTATC	0.285739692	0.133518047	-0.327179481
B_98618_CAGCGTTAAGAGTCAAAGTACAAG	0.142888786	0.105920951	-0.370356378
B_98618_CAGCGTTAATCATTCCGCTCGGTA	0.196287701	0.15564006	-0.131907599
B_98618_CAGCGTTAATCCTGTACAGCGTTA	0.173609349	-0.118778866	-0.45562013
B_98618_CAGCGTTACAACCACAAATGTTGC	0.251340331	0.086569611	-0.470350083
B_98618_CAGCGTTACAGCGTTAATTGAGGA	0.192160994	0.069529248	-0.180947704
B_98618_CAGCGTTACATCAAGTACCACTGT	0.053317369	-0.067391733	-0.292227296
B_98618_CAGCGTTACATCAAGTCCATCCTC	0.035834457	0.168809319	-0.300056244

B_98618_CAGCGTTACGACTGGAAAGGACAC	0.178889827	-0.015047614	-0.300287437
B_98618_CAGCGTTAGAGCTGAATATCAGCA	0.457095268	0.006479749	-0.413437255
B_98618_CAGCGTTAGCGAGTAACTAAGGTC	0.289680814	0.402484397	-0.285110516
B_98618_CAGCGTTATCTTCACACAACCACA	0.008375438	-0.032112545	-0.335155971
B_98618_CAGCGTTATCTTCACACGCTGATC	0.330345872	0.297131156	-0.26523603
B_98618_CAGCGTTATGGAACAAGGAGAACA	-0.011940239	0.033578974	-0.301218216
B_98618_CATACCAAAACGTGATAGTACAAG	0.267954157	0.062657767	-0.179880052
B_98618_CATACCAAAAGACGGAAACTCACC	0.306048396	0.081949111	-0.266716653
B_98618_CATACCAAACTATGCAGTGTTCTA	0.050430351	0.125314253	-0.179928812
B_98618_CATACCAAAGCACCTCCACCTTAC	0.011339215	-0.052243113	-0.23984195
B_98618_CATACCAAAGTCACTAAATCCGTC	0.10463767	0.142003321	-0.198537233
B_98618_CATACCAACAACCACATCCGTCTA	0.073139425	0.012121223	-0.116746917
B_98618_CATACCAACAAGGAGCATCATTCC	0.152366502	-0.120605232	-0.413228218
B_98618_CATACCAACATCAAGTAAGACGGA	0.020133047	-0.054617379	-0.333423784
B_98618_CATACCAACCTCTATCTCCGTCTA	0.348122226	0.038608162	-0.341631413
B_98618_CATACCAACGAACTTAGCTCGGTA	0.186636247	0.292593762	-0.18997694
B_98618_CATACCAACGCATACAACGCTCGA	0.04420776	-0.026533476	-0.253022093
B_98618_CATACCAAGGTGCGAAACCTCCAA	0.125616394	0.148645898	-0.29976664
B_98618_CATACCAAGTGTTCTAAGATCGCA	0.28154417	0.0777843	-0.291007103
B_98618_CATACCAATGGAACAAAGAGTCAA	0.539076796	-0.003015223	-0.335674672
B_98618_CATACCAATTCACGCAGGTGCGAA	0.528141064	0.057415903	-0.218573046
B_98618_CATCAAGTAAACATCGATGCCTAA	0.283184516	0.03594212	-0.181529457
B_98618_CATCAAGTAATGTTGCGAATCTGA	0.037000511	0.111598834	-0.070057191
B_98618_CATCAAGTACCTCCAAGAGCTGAA	0.352054429	0.178046436	-0.100681565
B_98618_CATCAAGTAGATGTACAAACATCG	0.098247676	-0.056467603	-0.148306773
B_98618_CATCAAGTATCATTCCCATCAAGT	0.151545185	0.145417332	-0.1857784
B_98618_CATCAAGTATCCTGTATAGGATGA	0.340204885	0.080382341	-0.292169227
B_98618_CATCAAGTATGCCTAATGGAACAA	0.278906222	-0.034877187	-0.107844413
B_98618_CATCAAGTATTGAGGAAAGACGGA	0.019365833	0.15639816	-0.202432785
B_98618_CATCAAGTATTGAGGACAAGACTA	0.268359222	0.16823417	-0.374870983
B_98618_CATCAAGTCAAGACTAAGTACAAG	0.166510493	-0.00393368	-0.282132762
B_98618_CATCAAGTCAATGGAACAAGACTA	0.239872398	-0.026607964	-0.211927593

B_98618_CATCAAGTCATACCAACTAAGGTC	0.01729428	0.02357187	-0.323495623
B_98618_CATCAAGTCCATCCTCAATGTTGC	0.2486137	-0.049492022	-0.430338205
B_98618_CATCAAGTCCATCCTCCTCAATGA	0.241244397	0.025825538	-0.336827076
B_98618_CATCAAGTCCGAAGTAAGTACAAG	0.238332424	-0.059003438	-0.336014483
B_98618_CATCAAGTCGACTGGACCTCCTGA	0.172531705	0.081820219	-0.346091652
B_98618_CATCAAGTCTGAGCCACATCAAGT	0.16883251	0.038968087	-0.321016479
B_98618_CATCAAGTGAATCTGAAGAGTCAA	0.174994866	0.079728414	-0.442647481
B_98618_CATCAAGTGATAGACAAGCCATGC	0.387367194	0.185234716	-0.315724022
B_98618_CATCAAGTGGTGCGAAGAACAGGC	0.241505288	0.082850474	-0.23602857
B_98618_CATCAAGTTCCGTCTAAGTGGTCA	0.489619544	0.085469835	-0.348689662
B_98618_CATCAAGTTGAAGAGAAACGTGAT	0.036696807	-0.000453146	-0.230789077
B_98618_CCAGTTCAAAGAGATCGAATCTGA	0.226820194	0.002027807	-0.398476453
B_98618_CCAGTTCACAATGGAACAGCGTTA	0.221735648	0.246334201	-0.380635957
B_98618_CCAGTTCACATACCAATATCAGCA	0.302601181	-0.031919489	-0.248616349
B_98618_CCAGTTCACCTAATCCACCACTGT	0.171218675	0.165883037	-0.365133414
B_98618_CCAGTTCACGCATACAGTCTGTCA	0.041241217	0.168170221	-0.278212445
B_98618_CCAGTTCAGCTCGGTACAGCGTTA	0.090859187	0.025513353	-0.154382142
B_98618_CCATCCTCAATGTTGCAAGGTACA	0.149704766	-0.003205024	-0.315467601
B_98618_CCATCCTCAGATGTACAGAGTCAA	0.203363145	0.012066997	-0.355372045
B_98618_CCATCCTCAGTCACTACCAGTTCA	0.151229648	-0.021379388	-0.21985765
B_98618_CCATCCTCATTGAGGACGAACTTA	0.054798477	0.1373845	-0.284385339
B_98618_CCATCCTCATTGGCTCAGTACAAG	-0.025181974	0.155247782	-0.028440025
B_98618_CCATCCTCCAATGGAAGAACAGGC	0.202615621	0.061227573	-0.25977871
B_98618_CCATCCTCCGACACACCAATGGAA	0.053829674	0.010363966	-0.242985022
B_98618_CCATCCTCCTGAGCCAAGCACCTC	0.010171163	-0.02539291	-0.210615207
B_98618_CCGAAGTAAAGAGATCGAGTTAGC	0.091669775	0.100332373	-0.220712782
B_98618_CCGAAGTAAAGGTACAAATGTTGC	0.309798963	0.041033252	-0.009374292
B_98618_CCGAAGTAAATCCGTCAAACATCG	0.226045845	-0.022721555	-0.210703803
B_98618_CCGAAGTAAATCCGTCAGATGTAC	0.283815807	0.013638708	-0.206944314
B_98618_CCGAAGTAACGTATCACCAGTTCA	0.023669941	0.143490089	-0.23233341
B_98618_CCGAAGTAAGCAGGAATGGAACAA	0.301336418	0.174431224	-0.068301016
B_98618_CCGAAGTACAAGACTACCAGTTCA	0.192907076	-0.017044807	-0.247343796

B_98618_CCGAAGTACGCTGATCAGATGTAC	0.261456276	0.001971947	-0.232545125
B_98618_CCGAAGTAGATGAATCAGCAGGAA	0.485959335	0.1799503	-0.370357844
B_98618_CCGAAGTAGATGAATCAGTACAAG	0.276624804	0.044759228	-0.079598333
B_98618_CCGAAGTAGATGAATCCAAGGAGC	0.065614798	0.103832321	-0.056173157
B_98618_CCGAAGTAGCCAAGACATTGGCTC	-0.056780843	0.123100001	-0.213717737
B_98618_CCGAAGTAGCCACATATCTTCACA	0.045608836	0.142939943	-0.293272433
B_98618_CCGAAGTAGGAGAACAAAACATCG	0.239177053	0.010196986	-0.419039591
B_98618_CCGAAGTAGTACGCAAACAGCAGA	0.206743813	0.152494762	-0.257506126
B_98618_CCGAAGTAGTCTGTCAACTATGCA	0.108399896	0.07370413	-0.127908328
B_98618_CCGAAGTATGGAACAAAACGCTTA	0.247274708	0.124162896	-0.221071019
B_98618_CCGACAACAACTCACCAATGTTGC	0.162554363	0.133161487	-0.202923891
B_98618_CCGACAACACGTATCAACAAGCTA	0.2266654	0.087675752	-0.209538854
B_98618_CCGACAACAGCACCTCCAACCACA	0.145356997	0.137532027	-0.365601736
B_98618_CCGACAACAGGCTAACGCTCGGTA	0.227155554	0.119165967	-0.334344614
B_98618_CCGACAACATCCTGTACCTAATCC	0.116399669	0.131054725	-0.429419311
B_98618_CCGACAACATTGAGGAAACAACCA	0.200246634	0.138341956	-0.027202021
B_98618_CCGACAACATTGAGGAACGCTCGA	0.097042631	0.011719876	-0.138779058
B_98618_CCGACAACATTGGCTCATCCTGTA	0.158494119	0.149820581	-0.160321078
B_98618_CCGACAACCGCTGATCCAGATCTG	-0.057096663	0.026537503	-0.245484381
B_98618_CCGACAACGCGAGTAAATTGAGGA	0.119529181	0.019402157	-0.341403529
B_98618_CCGACAACGCTAACGAATTGGCTC	0.137257454	0.029656752	-0.426983709
B_98618_CCGACAACGGTGCGAAACCACTGT	0.311473255	0.197813149	-0.264351569
B_98618_CCGACAACGGTGCGAACGACACAC	0.168229238	0.188284342	-0.138992536
B_98618_CCGACAACGTCGTAGAAAACATCG	0.179986088	-0.056948132	-0.274467673
B_98618_CCGACAACTATCAGCAAAGACGGA	0.016876469	0.141013617	-0.368270726
B_98618_CCGTGAGAAACAACCAAGTACAAG	0.257895043	0.027231638	-0.330565138
B_98618_CCGTGAGAACTATGCACACTTCGA	-0.004921017	0.052373869	-0.231666217
B_98618_CCGTGAGAAGAGTCAAAGCACCTC	0.109375248	0.1593796	-0.252713843
B_98618_CCGTGAGAAGAGTCAACAAGACTA	0.092303675	-0.039450309	-0.331595322
B_98618_CCGTGAGAATCATTCCAATCCGTC	0.139599763	0.131805724	-0.282326562
B_98618_CCGTGAGAATGCCTAAGTCGTAGA	0.232286986	0.106675715	-0.219998378
B_98618_CCGTGAGAATTGGCTCTCTTCACA	0.182663665	0.082750072	-0.30959638

B_98618_CCGTGAGACAACCACACAGATCTG	0.067783226	0.176097826	-0.346975478
B_98618_CCGTGAGACAGCGTTAAGTCACTA	0.189223675	0.225975916	-0.436511153
B_98618_CCGTGAGACATACCAAAGAGTCAA	0.217914534	0.020685762	-0.249393263
B_98618_CCGTGAGACGGATTGCGTACGCAA	0.118770584	0.001030673	-0.291957574
B_98618_CCGTGAGACTGAGCCATCCGTCTA	-0.051902606	0.01035266	-0.440549097
B_98618_CCGTGAGAGAACAGGCAATGTTGC	0.321820514	0.124444506	-0.093883182
B_98618_CCGTGAGAGAACAGGCCACCTTAC	0.329062743	0.243680934	-0.128761726
B_98618_CCGTGAGAGCTCGGTAACCTCCAA	0.031920153	0.054654797	-0.28967681
B_98618_CCGTGAGATATCAGCAGAATCTGA	0.374457235	0.080261983	-0.131141139
B_98618_CCTAATCCAACTCACCGTACGCAA	0.346116689	0.056014364	-0.444112022
B_98618_CCTAATCCACACGACCCGCTGATC	0.026927157	0.113086347	-0.258356135
B_98618_CCTAATCCACGTATCACGACACAC	0.092327009	-0.061928345	-0.158369632
B_98618_CCTAATCCAGATCGCATGGAACAA	0.21487347	-0.022786546	-0.432093087
B_98618_CCTAATCCATCCTGTAGCCACATA	0.132254567	-0.007242608	-0.319457736
B_98618_CCTAATCCATTGAGGAGATGAATC	0.06368039	0.115574524	-0.28915343
B_98618_CCTAATCCGCTAACGAACGTATCA	-0.001612138	-0.046181387	-0.443824415
B_98618_CCTAATCCGCTCGGTACAAGACTA	0.107769676	0.207549488	-0.179413881
B_98618_CCTAATCCGTCTGTCAAAGACGGA	0.23192683	0.019521109	-0.404353521
B_98618_CCTAATCCTCTTCACACTCAATGA	0.204010865	-0.054830641	-0.355030305
B_98618_CCTCCTGAAACTCACCCAACCACA	0.105434876	0.118111114	-0.29427966
B_98618_CCTCCTGAAAGAGATCACCACTGT	0.31666769	-0.076969483	-0.255181678
B_98618_CCTCCTGAAAGAGATCATCCTGTA	0.102372904	0.0489207	-0.282942052
B_98618_CCTCCTGAAAGAGATCGTGTTCTA	0.261246202	0.100066804	-0.267823352
B_98618_CCTCCTGAAAGGACACAAGACGGA	0.004067291	0.06510575	-0.10767291
B_98618_CCTCCTGACAAGACTATGGCTTCA	0.382154372	0.158352845	-0.315257804
B_98618_CCTCCTGACCGACAACAACCGAGA	0.40490167	0.133928248	-0.352324956
B_98618_CCTCCTGACGCTGATCCTGAGCCA	0.179768557	0.083884316	-0.287939348
B_98618_CCTCCTGAGTGTTCTATTCACGCA	0.191403838	0.188892701	-0.356001312
B_98618_CCTCCTGATGGAACAAAGAGTCAA	0.083741042	0.091066315	-0.402245804
B_98618_CCTCCTGATGGCTTCAACAGATTC	0.043362823	0.174935224	-0.148420068
B_98618_CCTCCTGATGGCTTCACAGCGTTA	0.177739048	0.050292825	-0.064391603
B_98618_CCTCTATCAAGAGATCCGCATACA	0.319128804	0.006350086	-0.24401163

B_98618_CCTCTATCACCTCCAAAGCAGGAA	0.031717525	0.016954001	-0.165931457
B_98618_CCTCTATCAGTGGTCAAAGGACAC	0.128947572	0.003132432	-0.175263653
B_98618_CCTCTATCCAACCACAGCTCGGTA	0.582651559	0.188346046	-0.247961254
B_98618_CCTCTATCCGCTGATCAACGCTTA	0.174391698	0.058209198	-0.340703052
B_98618_CCTCTATCCTGTAGCCTGGTGGTA	0.078067066	0.115149448	-0.359239448
B_98618_CCTCTATCGCCACATACTCAATGA	0.028243296	0.020567696	-0.040589501
B_98618_CCTCTATCGCGAGTAATCTTCACA	0.40262044	0.145985781	-0.126098009
B_98618_CCTCTATCGGAGAACAAGTCACTA	0.18349256	0.075372593	-0.279038195
B_98618_CCTCTATCGGTGCGAACAAGGAGC	0.131737734	0.054386155	-0.131030637
B_98618_CCTCTATCTGAAGAGAAGTCACTA	0.177713632	0.141231636	-0.072783182
B_98618_CGAACTTAACAGCAGAAGCAGGAA	0.136114131	0.15165171	-0.177450826
B_98618_CGAACTTAACATTGGCTTCACGCA	0.184443059	0.149547451	-0.280823103
B_98618_CGAACTTAACCTCCAACAAGACTA	-0.067131002	-0.048563097	-0.351940708
B_98618_CGAACTTAATAGCGACGTACGCAA	0.136369618	0.163453035	-0.416330151
B_98618_CGAACTTACAAGACTAAGAGTCAA	0.097169374	-0.042048933	-0.118261136
B_98618_CGAACTTACGACACACAGCAGGAA	0.183856505	0.076779651	-0.258306647
B_98618_CGAACTTAGATAGACAATTGAGGA	0.13830122	0.191774071	-0.020201044
B_98618_CGAACTTAGATAGACAATTGGCTC	0.243018405	0.137381148	-0.08972395
B_98618_CGAACTTATGGCTTCAGCTCGGTA	-0.107657	0.122296421	-0.196750712
B_98618_CGACACACACCACTGTACCACTGT	-0.032349877	0.123610405	-0.328129761
B_98618_CGACACACAGGCTAACCAATGGAA	0.096437225	0.100607471	-0.19433168
B_98618_CGACACACAGTACAAGCTGAGCCA	0.127581942	0.168811362	-0.221047926
B_98618_CGACACACCAAGGAGCCCATCCTC	0.14178476	-0.016244096	-0.470945764
B_98618_CGACACACCACTTCGACTCAATGA	0.258682778	-0.188739802	-0.4036475
B_98618_CGACACACCAGCGTTACGACACAC	0.164201333	0.063464572	-0.436166475
B_98618_CGACACACCCGAAGTACACCTTAC	0.195991482	0.138254702	-0.161121139
B_98618_CGACACACCCGACAACCTGAGCCA	0.326446528	-0.06441225	-0.29753459
B_98618_CGACACACCGACACACCATACCAA	0.30347846	0.139082996	-0.083352276
B_98618_CGACACACGAATCTGACCGTGAGA	0.302188591	0.210823591	-0.355723531
B_98618_CGACACACGAGTTAGCTCTTCACA	0.054350958	0.020702712	-0.290561331
B_98618_CGACACACGCCACATAATTGAGGA	0.267547085	0.035561165	0.088532487
B_98618_CGACACACGCTCGGTAAGCCATGC	0.380201183	0.391518433	-0.517706143

B_98618_CGACACACGCTCGGTACGCTGATC	0.163752571	0.135746349	-0.330090988
B_98618_CGACTGGAAAACATCGCATACCAA	0.136875984	0.021696872	-0.253410876
B_98618_CGACTGGAAAGGACACATCATTCC	0.205428856	-0.008491548	-0.195938709
B_98618_CGACTGGAAATGTTGCAGTGGTCA	0.060880206	0.05749445	-0.073989233
B_98618_CGACTGGAACAGCAGATATCAGCA	0.060273972	0.300983161	-0.342542141
B_98618_CGACTGGAAGAGTCAAGGTGCGAA	0.407976578	0.198375602	-0.248756536
B_98618_CGACTGGAAGATGTACCGACTGGA	0.117353548	0.104614464	-0.270151888
B_98618_CGACTGGAAGTACAAGCTAAGGTC	0.006303099	0.028824075	-0.40593149
B_98618_CGACTGGAATAGCGACAGCAGGAA	0.207554792	0.155749998	-0.300741575
B_98618_CGACTGGAATTGGCTCATGCCTAA	0.195581517	0.020916509	-0.293624964
B_98618_CGACTGGACAGCGTTAGCTCGGTA	0.254715259	0.102309277	-0.215199688
B_98618_CGACTGGACTCAATGAACGTATCA	0.226853811	0.022732699	-0.349187229
B_98618_CGACTGGACTGTAGCCACGCTCGA	0.189888357	0.267728318	-0.334478105
B_98618_CGACTGGAGACAGTGCACAGATTC	0.036194819	-0.006615163	-0.220851688
B_98618_CGACTGGAGACTAGTAAGTCACTA	0.308512479	0.088653087	-0.259271283
B_98618_CGACTGGAGAGTTAGCCGGATTGC	0.012957387	0.213534667	-0.171876398
B_98618_CGACTGGATAGGATGAAAGAGATC	0.122256039	-0.064020556	-0.462772022
B_98618_CGACTGGATCTTCACAACCACTGT	0.145826992	0.031019217	-0.411247009
B_98618_CGCATACAAACGCTTAACGCTCGA	0.249882545	0.056466071	-0.241570341
B_98618_CGCATACAAAGACGGAACCTCCAA	0.017612785	0.076261603	-0.113892353
B_98618_CGCATACAAATCCGTCCCTCCTGA	0.036974311	0.148802508	-0.333277556
B_98618_CGCATACAACGTATCACCGAAGTA	0.187611746	0.158245901	-0.212069464
B_98618_CGCATACAAGTCACTAACTATGCA	0.165068185	0.191367646	-0.325620968
B_98618_CGCATACACAACCACAGCCACATA	0.255045587	0.000528171	-0.353479253
B_98618_CGCATACACACTTCGAAGCACCTC	0.130283395	-0.012807819	-0.413813185
B_98618_CGCATACAGACTAGTACCATCCTC	0.252037371	-0.125562725	-0.25552679
B_98618_CGCATACAGAGCTGAAACCACTGT	0.102856809	0.005009589	-0.173866264
B_98618_CGCATACAGAGCTGAAAGTCACTA	0.14449187	0.040844395	-0.126685584
B_98618_CGCATACAGCCAAGACAACTCACC	0.45987371	0.070818231	-0.179542218
B_98618_CGCATACAGCCACATAGAACAGGC	0.174987729	0.126482837	-0.173174852
B_98618_CGCATACAGCTAACGAACAAGCTA	0.262768058	0.026772505	-0.17734419
B_98618_CGCATACAGGTGCGAACCTAATCC	0.201165863	0.208383238	-0.274511829

B_98618_CGCATACATCTTCACACAATGGAA	0.118618195	0.105197844	-0.166804358
B_98618_CGCATACATGGAACAACCGACAAC	0.320587792	0.103236506	-0.16034683
B_98618_CGCTGATCAAACATCGGAACAGGC	0.176861688	0.17243771	-0.050125726
B_98618_CGCTGATCAACAACCAGCCAAGAC	0.180757242	-0.038687922	-0.33098076
B_98618_CGCTGATCAACAACCATATCAGCA	0.168936959	0.07995576	-0.202390054
B_98618_CGCTGATCAAGACGGAACACGACC	0.157253331	0.033085117	-0.09336575
B_98618_CGCTGATCAATCCGTCGCCAAGAC	0.244396821	-0.062797087	-0.274953971
B_98618_CGCTGATCAATGTTGCCAACCACA	0.137115993	0.065568386	-0.188405708
B_98618_CGCTGATCACAAGCTACCTCCTGA	0.08141509	0.110986824	-0.355673604
B_98618_CGCTGATCAGATCGCAACAGCAGA	0.041881973	-0.029895443	-0.362195662
B_98618_CGCTGATCAGTGGTCACGGATTGC	0.17929101	0.049128677	-0.257655734
B_98618_CGCTGATCCCATCCTCCAACCACA	0.022054745	0.026674997	-0.422731836
B_98618_CGCTGATCCCTCCTGAATCCTGTA	0.080472367	0.138732301	-0.079524337
B_98618_CGCTGATCCCTCCTGAGATGAATC	0.252222643	-0.042225586	-0.34954919
B_98618_CGCTGATCCGAACTTAAACGTGAT	0.123520878	-0.034323276	-0.22164173
B_98618_CGCTGATCCTGTAGCCAACGCTTA	0.076911653	-0.005557789	-0.426480456
B_98618_CGCTGATCGAATCTGAATTGAGGA	0.149690272	0.076746228	-0.148963187
B_98618_CGCTGATCGACTAGTAAAGAGATC	0.097880221	0.106704712	-0.365927495
B_98618_CGCTGATCGAGTTAGCCCGAAGTA	0.175329886	0.01545991	-0.269809152
B_98618_CGCTGATCGCGAGTAAATCCTGTA	0.164583391	0.083373088	-0.238520384
B_98618_CGCTGATCTCTTCACATCTTCACA	0.113883979	0.259390367	-0.078415357
B_98618_CGGATTGCACAAGCTATATCAGCA	0.113132565	0.067336827	-0.340014368
B_98618_CGGATTGCACACGACCTGGCTTCA	0.367471353	-0.024833069	-0.230893147
B_98618_CGGATTGCCCATCCTCAACAACCA	-0.025071249	0.038472874	-0.403263288
B_98618_CGGATTGCCGCATACAACCTCCAA	0.380788471	0.054932688	-0.359792114
B_98618_CGGATTGCCGCTGATCATGCCTAA	0.107088528	0.016932034	-0.205993214
B_98618_CGGATTGCGAGCTGAAACAGCAGA	0.109153084	0.005264679	-0.33413169
B_98618_CGGATTGCGATAGACAATTGGCTC	0.26322637	-0.014299682	-0.08417819
B_98618_CTAAGGTCAACGCTTAAGATGTAC	0.173894615	0.011060096	-0.278516395
B_98618_CTAAGGTCAACGTGATAACCGAGA	0.1171634	0.139947732	-0.149410966
B_98618_CTAAGGTCACACAGAAAGTCACTA	0.309585604	0.10208937	-0.459611995
B_98618_CTAAGGTCACGTATCACCGTGAGA	0.096826646	-0.025503045	-0.38041909

B_98618_CTAAGGTCATTGAGGAATAGCGAC	0.005035248	0.130570451	0.071348805
B_98618_CTAAGGTCCAACCACAGCTAACGA	0.105814442	0.08105297	-0.137640048
B_98618_CTAAGGTCCCGACAACAGCCATGC	0.228121007	0.08404463	-0.0445097
B_98618_CTAAGGTCCCTAATCCACGTATCA	0.154166512	0.100835373	-0.232234955
B_98618_CTAAGGTCTTCACGCACTAAGGTC	0.08311295	0.044940691	-0.352738815
B_98618_CTCAATGAAACCGAGAAACTCACC	0.170994348	0.079900647	-0.164792948
B_98618_CTCAATGAAAGAGATCTATCAGCA	0.092029127	0.152746327	-0.250441513
B_98618_CTCAATGAAAGGACACCAACCACA	0.171827953	0.067967874	-0.288015705
B_98618_CTCAATGAACGTATCAAACAACCA	-0.037825718	-0.106460648	-0.340891715
B_98618_CTCAATGAAGGCTAACAGATGTAC	0.331636824	0.108930439	-0.349753148
B_98618_CTCAATGAATGCCTAACCTAATCC	0.15402971	0.052131366	-0.199153828
B_98618_CTCAATGACCATCCTCAAGACGGA	0.32857615	0.088498402	-0.288846141
B_98618_CTGAGCCAACAAGCTAATCCTGTA	-0.010962335	0.162810482	-0.094682536
B_98618_CTGAGCCAACACAGAACATCAAGT	0.236879836	-0.088412208	-0.401705975
B_98618_CTGAGCCAACCTCCAAACATTGGC	0.258529731	0.01943353	-0.365150361
B_98618_CTGAGCCAACGTATCACATACCAA	0.247499585	0.035024919	-0.18612283
B_98618_CTGAGCCAAGATCGCATCTTCACA	0.108838413	0.08715581	-0.101190482
B_98618_CTGAGCCACCAGTTCACCTCTATC	0.173839404	-0.055162648	-0.342134302
B_98618_CTGAGCCACCAGTTCATGGAACAA	0.147037899	0.159787432	-0.433683461
B_98618_CTGAGCCACCGTGAGAGCCACATA	0.196814658	0.121274704	-0.284292818
B_98618_CTGAGCCACCTCTATCGACTAGTA	0.431714881	0.107672177	-0.155256421
B_98618_CTGAGCCAGACTAGTAAGGCTAAC	0.157550462	0.349792365	-0.167288557
B_98618_CTGAGCCATCCGTCTAACACGACC	0.309646467	-0.009244357	-0.195495897
B_98618_CTGAGCCATGAAGAGACAACCACA	2.71E-05	0.045101888	-0.28879761
B_98618_CTGGCATAAACGTGATACCTCCAA	0.167207477	0.302356353	-0.294782581
B_98618_CTGGCATAACACAGAATTCACGCA	0.179636594	0.051760419	-0.332274822
B_98618_CTGGCATAACACGACCGTGTTCTA	0.15816312	0.195624695	-0.430259517
B_98618_CTGGCATAAGATGTACTCCGTCTA	0.367315827	0.269837442	-0.186109419
B_98618_CTGGCATACATACCAAGAACAGGC	0.07356326	0.090112385	-0.349578142
B_98618_CTGGCATACGCTGATCGATGAATC	0.09685976	0.079018901	0.095419304
B_98618_CTGGCATACTAAGGTCACCTCCAA	0.214898268	0.253678372	-0.377230712
B_98618_CTGGCATACTCAATGAGCTAACGA	0.135151077	0.201484837	-0.352342826

B_98618_CTGGCATACTGTAGCCGTACGCAA	0.177998995	0.093786703	-0.25712128
B_98618_CTGGCATAGAACAGGCCAACCACA	0.469805387	0.389192304	-0.304348143
B_98618_CTGGCATAGAATCTGAAACGCTTA	0.101956482	-0.011241702	-0.364064529
B_98618_CTGGCATAGACTAGTAAAGGACAC	0.20751104	0.077516349	-0.153287551
B_98618_CTGTAGCCAACAACCAGCGAGTAA	0.319944691	0.052087013	-0.303685357
B_98618_CTGTAGCCAAGACGGAAGGCTAAC	0.04246027	0.027565362	-0.180722617
B_98618_CTGTAGCCACAAGCTAAAGGACAC	-0.061198249	0.198998896	-0.109821856
B_98618_CTGTAGCCACAGATTCGACTAGTA	0.255044305	0.119379653	-0.229829152
B_98618_CTGTAGCCACTATGCAGCGAGTAA	0.192215646	0.106036824	-0.206644689
B_98618_CTGTAGCCCCTCTATCGCCAAGAC	0.201217206	0.10621995	-0.134183186
B_98618_CTGTAGCCGCTAACGAACAGCAGA	0.102012167	0.173823836	-0.203857918
B_98618_CTGTAGCCTCCGTCTAACGTATCA	0.151538388	0.012093878	-0.458309736
B_98618_CTGTAGCCTGGAACAACCTCCTGA	0.036940636	0.022515071	-0.04435502
B_98618_GAACAGGCAAACATCGGGTGCGAA	0.131507225	0.039368663	-0.390239497
B_98618_GAACAGGCAAGGACACCAAGGAGC	0.09897918	0.113228142	-0.034112766
B_98618_GAACAGGCAGTACAAGAAGGACAC	0.316398905	0.017569795	-0.24507548
B_98618_GAACAGGCCAGATCTGACGCTCGA	0.206971493	-0.107019021	-0.470896097
B_98618_GAACAGGCCAGCGTTACCTCTATC	0.013299352	-0.066520289	-0.42392318
B_98618_GAACAGGCCCTCCTGAAAGGACAC	0.073163079	0.187038745	-0.418329488
B_98618_GAACAGGCCCTCCTGAGCGAGTAA	0.284145853	-0.009904954	-0.28411976
B_98618_GAACAGGCCGGATTGCCGACTGGA	0.162522136	-0.03303505	-0.284413141
B_98618_GAACAGGCCTCAATGAAACGCTTA	0.104523191	0.114989332	-0.220806635
B_98618_GAACAGGCCTGGCATACACTTCGA	0.098444471	-0.139678956	-0.39556945
B_98618_GAACAGGCCTGTAGCCACCACTGT	0.144718953	0.092792909	-0.364241317
B_98618_GAACAGGCGATGAATCCTGAGCCA	0.127484153	0.096209101	-0.174964485
B_98618_GAACAGGCGGAGAACAAGTCACTA	0.262994949	0.399757589	-0.254946405
B_98618_GAACAGGCTAGGATGAAGCCATGC	0.189036053	-0.001212716	-0.360045842
B_98618_GAACAGGCTGGAACAAAGTGGTCA	0.038067827	-0.026702701	-0.376752228
B_98618_GAATCTGAACTATGCAGCTCGGTA	0.278416536	0.145641582	-0.303703261
B_98618_GAATCTGAAGAGTCAAAGTACAAG	0.06191177	0.128981826	-0.333094752
B_98618_GAATCTGAAGGCTAACACCACTGT	0.075519752	0.089302643	-0.328689532
B_98618_GAATCTGACAACCACAATAGCGAC	0.224551914	-0.015331075	-0.338994533

B_98618_GAATCTGACACTTCGAGATAGACA	0.076302239	0.11422173	-0.266672242
B_98618_GAATCTGACCATCCTCGCTCGGTA	0.073510551	0.017032108	-0.412025
B_98618_GAATCTGACGACACACCACCTTAC	0.175288005	0.062702222	-0.097653339
B_98618_GAATCTGACTGAGCCAAGGCTAAC	0.169999626	-0.027935076	-0.167092786
B_98618_GAATCTGACTGTAGCCAAGGTACA	0.178205878	-0.000261204	-0.394328251
B_98618_GAATCTGAGAGTTAGCGAGTTAGC	0.055323927	-0.032880738	-0.396930079
B_98618_GAATCTGAGATAGACAAGTACAAG	-0.025313869	-0.026106313	-0.263432238
B_98618_GAATCTGAGCCACATAGGAGAACA	0.159496899	-0.126708563	-0.402441538
B_98618_GAATCTGAGTACGCAACGACACAC	0.10300998	0.1420558	-0.018621751
B_98618_GAATCTGATCCGTCTAACATTGGC	0.009107067	-0.035771042	-0.354356373
B_98618_GACAGTGCACGCTCGAACGTATCA	0.20591198	0.027904778	-0.307176393
B_98618_GACAGTGCAGGCTAACAGTGGTCA	0.28790833	0.049135514	-0.335435554
B_98618_GACAGTGCCCAGTTCAAACTCACC	0.190358591	0.003739172	-0.424607287
B_98618_GACAGTGCCGAACTTAGATGAATC	0.23770031	0.107790746	-0.19744318
B_98618_GACAGTGCGATAGACACAGATCTG	0.109253647	0.001497671	-0.287718173
B_98618_GACAGTGCGCTAACGACACTTCGA	0.200241698	0.040561497	-0.431432877
B_98618_GACAGTGCTCCGTCTAAGTACAAG	0.082429646	0.172545253	-0.365974907
B_98618_GACAGTGCTGAAGAGACAACCACA	0.172595484	0.103250728	-0.147182682
B_98618_GACTAGTAACGCTCGACAAGGAGC	0.086803237	0.095769773	-0.260893141
B_98618_GACTAGTACAAGACTACGCTGATC	0.140534792	0.013004661	-0.389155118
B_98618_GAGCTGAAAACTCACCAGAGTCAA	0.215365749	0.043872763	-0.242496137
B_98618_GAGCTGAAAATCCGTCCCGACAAC	0.183410627	0.009775791	-0.413422138
B_98618_GAGCTGAAACACGACCTGAAGAGA	-0.085351181	0.155475187	-0.132434045
B_98618_GAGCTGAAACAGATTCAACGCTTA	0.121592998	0.000386747	-0.219957422
B_98618_GAGCTGAAACGCTCGAAACTCACC	-0.017179889	-0.009265178	-0.29965271
B_98618_GAGCTGAAACGTATCAATTGAGGA	0.096380309	0.081592663	-0.398564727
B_98618_GAGCTGAAACGTATCACCTAATCC	0.160188359	-0.039318824	-0.320284909
B_98618_GAGCTGAAACGTATCACGCATACA	-0.018536727	0.109887456	-0.038033795
B_98618_GAGCTGAAAGATGTACCTGGCATA	0.220876634	0.012788637	-0.321216584
B_98618_GAGCTGAAAGCAGGAACTGAGCCA	0.096572254	0.115174793	-0.296676827
B_98618_GAGCTGAAAGCCATGCAGATGTAC	0.193505813	0.087713946	-0.267646168
B_98618_GAGCTGAAAGTGGTCACGACACAC	0.086331705	0.060981596	-0.289509215

B_98618_GAGCTGAACAGCGTTAGACTAGTA	0.330075306	0.057847692	-0.433358499
B_98618_GAGCTGAACGGATTGCATTGAGGA	0.25626753	0.016344253	-0.296471266
B_98618_GAGCTGAAGCTAACGACCTCCTGA	0.036455738	0.077699933	-0.301609877
B_98618_GAGCTGAATATCAGCAATCATTCC	0.303272483	0.057157151	-0.337183493
B_98618_GAGTTAGCAACGCTTACCGAAGTA	-0.064978807	0.071241997	-0.087272586
B_98618_GAGTTAGCACACAGAACTGGCATA	-0.014254981	0.029677535	-0.407025505
B_98618_GAGTTAGCACACGACCTCCGTCTA	0.096898896	0.165208195	-0.27024453
B_98618_GAGTTAGCACGTATCAACATTGGC	0.207427464	0.279646265	-0.014342816
B_98618_GAGTTAGCAGAGTCAACACCTTAC	0.136642551	0.053251815	-0.160318651
B_98618_GAGTTAGCAGCAGGAAAACCGAGA	0.177082947	-0.141387821	-0.43954795
B_98618_GAGTTAGCCAGCGTTAAAGACGGA	0.253305634	0.034924573	-0.293046622
B_98618_GAGTTAGCCAGCGTTATGGCTTCA	0.098890711	-0.114874901	-0.427322238
B_98618_GAGTTAGCCATACCAAACCTCCAA	0.091407963	0.02459487	-0.367552673
B_98618_GAGTTAGCCATACCAAAGATGTAC	0.265370229	0.155934553	-0.37143675
B_98618_GAGTTAGCCTGTAGCCGCTCGGTA	0.054301176	-0.144410402	-0.357787686
B_98618_GAGTTAGCGACAGTGCAGATCGCA	0.074437513	-0.019063111	-0.188444291
B_98618_GAGTTAGCGATGAATCTAGGATGA	0.029393511	0.031267985	-0.347190413
B_98618_GAGTTAGCGCTCGGTAAACAACCA	0.447155177	-0.080532703	-0.349530865
B_98618_GAGTTAGCGTCTGTCAATTGAGGA	0.197219752	0.117504757	-0.182305792
B_98618_GAGTTAGCGTCTGTCATTCACGCA	0.589287541	0.058192881	-0.215723677
B_98618_GATAGACAAAGAGATCATTGAGGA	-0.017130871	-0.073291569	-0.395471158
B_98618_GATAGACAAAGGACACAGATCGCA	0.221817384	0.102680277	-0.205463212
B_98618_GATAGACAAGGCTAACGAATCTGA	0.014958428	0.059208295	-0.179256023
B_98618_GATAGACACTCAATGACGACTGGA	0.297717724	-0.0627218	-0.32857799
B_98618_GATAGACACTGAGCCAGATAGACA	0.158396056	0.048852714	-0.158113345
B_98618_GATAGACAGAGCTGAACAATGGAA	0.178471785	0.127508648	-0.224139287
B_98618_GATAGACAGGAGAACATCCGTCTA	0.150223181	0.042531629	-0.256837945
B_98618_GATAGACAGTGTTCTAAAGACGGA	0.327568281	0.035813367	-0.169150837
B_98618_GATGAATCAAGGACACTGGAACAA	0.274585246	0.055990528	-0.169396212
B_98618_GATGAATCACCTCCAAAACTCACC	0.321905616	0.045968748	-0.039894258
B_98618_GATGAATCAGCAGGAAAACGTGAT	0.083973003	0.242535933	-0.193667341
B_98618_GATGAATCCCTAATCCACGCTCGA	0.207647687	-0.018247542	-0.384696129

B_98618_GATGAATCCTAAGGTCATAGCGAC	0.23521258	0.125657979	-0.332908691
B_98618_GATGAATCCTGGCATACCGTGAGA	0.221357591	0.073395822	-0.211647599
B_98618_GCCAAGACAACAACCACCTCCTGA	0.032430303	0.135842683	-0.280245304
B_98618_GCCAAGACAAGGACACAGTCACTA	0.146294191	0.062055494	-0.295443007
B_98618_GCCAAGACAGGCTAACTGAAGAGA	0.034044523	0.098584175	-0.248807059
B_98618_GCCAAGACATCATTCCACACGACC	-0.010049961	-0.038526563	-0.175305475
B_98618_GCCAAGACATCCTGTAAGCAGGAA	0.140215541	-0.010277777	-0.332265965
B_98618_GCCAAGACATTGAGGAAGATGTAC	0.224237537	0.092240095	-0.40160409
B_98618_GCCAAGACCCGAAGTACGCTGATC	0.303282402	0.069807402	-0.172124971
B_98618_GCCAAGACCCGACAACCACCTTAC	0.140692848	0.072553917	-0.210973566
B_98618_GCCAAGACCCGTGAGAACAGATTC	0.344939	0.031699238	-0.379976668
B_98618_GCCAAGACGATGAATCCCTCTATC	0.236528638	0.079764277	-0.062190162
B_98618_GCCAAGACGCGAGTAAAACGCTTA	0.163169693	0.04435126	-0.439342847
B_98618_GCCAAGACTAGGATGACGAACTTA	0.113124828	0.266926588	-0.207701976
B_98618_GCCAAGACTCTTCACAAGCACCTC	-0.015228235	0.247786729	-0.141768065
B_98618_GCCACATAAAGACGGACCATCCTC	0.381023829	-0.010722822	-0.329859675
B_98618_GCCACATAATGCCTAATATCAGCA	0.118005606	0.115311761	-0.277238804
B_98618_GCCACATACCATCCTCGAATCTGA	0.099020454	0.027189199	-0.261287007
B_98618_GCCACATACTGAGCCAAACGCTTA	0.152730342	-0.051242284	-0.407418685
B_98618_GCGAGTAAAAGGACACAGTGGTCA	0.229102807	0.103463862	-0.366681042
B_98618_GCGAGTAAAATCCGTCAACAACCA	0.150573565	0.142651388	-0.175144523
B_98618_GCGAGTAAACACAGAAGTGTTCTA	0.171506426	-0.06380264	-0.322877291
B_98618_GCGAGTAAACACGACCACAGCAGA	0.154697833	-0.069273959	-0.438173519
B_98618_GCGAGTAAACCTCCAAAAGGACAC	0.273100335	0.113266178	-0.277476349
B_98618_GCGAGTAAAGATCGCAAAGGTACA	0.269986005	-0.003402268	-0.279217001
B_98618_GCGAGTAACAAGGAGCGTCTGTCA	0.1778006	0.065325056	-0.257731892
B_98618_GCGAGTAACAGATCTGAAGGACAC	0.357017027	0.116723924	-0.277259486
B_98618_GCGAGTAACATACCAACGACTGGA	0.169285762	0.070757946	-0.323824723
B_98618_GCGAGTAACGACTGGAAGTCACTA	0.207173999	0.006667691	-0.216061605
B_98618_GCGAGTAAGAATCTGAACGCTCGA	0.039102779	0.145083313	-0.280948389
B_98618_GCGAGTAAGCGAGTAAATTGGCTC	0.02835438	0.055768086	-0.416974117
B_98618_GCGAGTAATGGCTTCAACACGACC	0.082789282	0.022126129	-0.307126736

B_98618_GCGAGTAATTCACGCAACGTATCA	-0.067404323	0.047511976	-0.319479009
B_98618_GCTAACGAAACGTGATAGAGTCAA	0.178904269	-0.025216881	-0.263190568
B_98618_GCTAACGAACCACTGTCACCTTAC	0.091822111	0.207741058	-0.403194371
B_98618_GCTAACGAACCTCCAAAGTACAAG	0.33559491	0.08005853	-0.421290572
B_98618_GCTAACGAATAGCGACAGTACAAG	0.051585579	0.097733717	-0.307844588
B_98618_GCTAACGAATCATTCCCCTAATCC	0.131721481	0.051551784	-0.159280848
B_98618_GCTAACGACGACTGGACCTCCTGA	0.38542996	0.1010357	-0.10061935
B_98618_GCTAACGAGATAGACAAAGAGATC	0.227472962	0.125807152	-0.269700404
B_98618_GCTAACGAGATAGACATTCACGCA	0.328770282	0.104501022	-0.265622846
B_98618_GCTAACGAGCCAAGACGACTAGTA	0.136744802	0.169867692	-0.13979877
B_98618_GCTAACGATGAAGAGAGTCTGTCA	-0.141409868	0.198024754	-0.17862481
B_98618_GCTAACGATTCACGCAATCATTCC	0.234361397	0.170759003	-0.095069507
B_98618_GCTCGGTAAACCGAGACTGAGCCA	0.014601302	-0.075447221	-0.347180045
B_98618_GCTCGGTAACACGACCACATTGGC	0.29664527	0.067000796	-0.323222907
B_98618_GCTCGGTAACCACTGTACAAGCTA	0.131937648	0.065894924	-0.235000811
B_98618_GCTCGGTACAAGGAGCACACGACC	0.261268609	0.181888635	-0.219508112
B_98618_GCTCGGTACAAGGAGCAGCACCTC	0.254044605	0.177187032	-0.27480752
B_98618_GCTCGGTACGACACACAACAACCA	-0.012452016	-0.042802669	-0.324493768
B_98618_GCTCGGTAGAATCTGATCCGTCTA	0.080920758	0.057530113	-0.108233053
B_98618_GCTCGGTAGACTAGTACAATGGAA	0.198859203	0.046033513	-0.430067884
B_98618_GCTCGGTAGTCGTAGAAACTCACC	0.266343995	0.09000074	-0.136198367
B_98618_GCTCGGTATGGAACAAAAGAGATC	0.104309341	0.04235903	-0.302832735
B_98618_GGAGAACAACAAGCTAGACAGTGC	0.060596853	0.183982235	-0.17303179
B_98618_GGAGAACAACAAGCTATGGAACAA	0.062962764	0.135947677	-0.30430751
B_98618_GGAGAACAACACGACCGAACAGGC	0.261739513	0.116782498	-0.387866155
B_98618_GGAGAACAACCTCCAACGGATTGC	-0.039952682	0.11388308	-0.118114573
B_98618_GGAGAACAAGAGTCAATCCGTCTA	0.18725183	0.097689906	-0.277648297
B_98618_GGAGAACAAGATCGCAGTCTGTCA	0.259409506	0.126804717	-0.096343809
B_98618_GGAGAACAAGTACAAGCAACCACA	0.137435207	0.013833701	-0.303558571
B_98618_GGAGAACAATCCTGTAACAGCAGA	0.356507234	0.199809737	-0.08632729
B_98618_GGAGAACACATACCAAATTGGCTC	0.220372705	0.079198335	-0.299771743
B_98618_GGAGAACACCGACAACAATGTTGC	0.215348231	0.057910012	-0.352230213

B_98618_GGAGAACACTGTAGCCAGATGTAC	0.134008458	-0.044666632	-0.228322161
B_98618_GGAGAACAGTCTGTCACGAACTTA	0.089159348	-0.030572072	-0.422014771
B_98618_GGAGAACAGTGTTCTACCTCCTGA	0.24988678	0.030917494	-0.166457987
B_98618_GGAGAACATGGCTTCAAGATCGCA	0.129131564	0.021721315	-0.280587666
B_98618_GGTGCGAAAAGGACACAGAGTCAA	0.428913517	0.033226111	-0.448158608
B_98618_GGTGCGAAACAGCAGACTAAGGTC	0.055057112	-0.000907398	-0.298177358
B_98618_GGTGCGAAACCACTGTGGTGCGAA	0.295332013	-0.085520316	-0.432263313
B_98618_GGTGCGAAACGCTCGAACAGCAGA	0.265409733	0.040451053	-0.479990024
B_98618_GGTGCGAAAGCACCTCCCTCCTGA	0.152245031	-0.016715922	-0.196768879
B_98618_GGTGCGAAATGCCTAACAGATCTG	0.233855617	0.03711513	-0.221336679
B_98618_GGTGCGAACAAGACTAGAACAGGC	0.19536304	0.017692426	-0.208898646
B_98618_GGTGCGAACGACACACGAGTTAGC	0.14608327	0.141524246	-0.162159262
B_98618_GGTGCGAACGCATACAACGCTCGA	0.185196223	0.101629655	-0.347428451
B_98618_GGTGCGAACGCTGATCGAACAGGC	0.506975425	0.013937861	-0.350942537
B_98618_GGTGCGAAGAATCTGAACGTATCA	0.081594326	0.058918295	-0.446750354
B_98618_GGTGCGAAGAGTTAGCTCCGTCTA	0.158875528	0.081925384	-0.360868902
B_98618_GGTGCGAATCTTCACATCCGTCTA	-0.066784213	0.154899272	-0.275676256
B_98618_GTACGCAAAACTCACCCACTTCGA	0.073588028	0.059221912	-0.12956758
B_98618_GTACGCAAAAGAGATCAGCACCTC	0.030745244	0.071308629	-0.260303154
B_98618_GTACGCAAAGCAGGAAAACGCTTA	0.168209386	0.199528036	-0.199781226
B_98618_GTACGCAAAGGCTAACCGCTGATC	0.458712823	0.143841016	-0.213715678
B_98618_GTACGCAAAGTGGTCACACTTCGA	0.333475549	-0.175769316	-0.428071713
B_98618_GTACGCAAGCTCGGTAATAGCGAC	0.377498314	0.021142163	-0.236258364
B_98618_GTACGCAATGGCTTCAGTACGCAA	0.163421693	0.485446234	-0.308569052
B_98618_GTCGTAGAATCATTCCACTATGCA	0.015000097	0.218741496	-0.354686153
B_98618_GTCGTAGAGCGAGTAAGAACAGGC	0.246598007	0.149470915	-0.098047441
B_98618_GTCGTAGAGGTGCGAAAGTACAAG	0.165739583	0.113938339	-0.235296073
B_98618_GTCGTAGAGTACGCAACCGAAGTA	0.336225971	0.20221247	-0.220983723
B_98618_GTCTGTCAACGTATCAGCGAGTAA	0.189956295	0.078822199	-0.105283218
B_98618_GTCTGTCACAAGACTAATTGGCTC	0.184342361	0.107519405	-0.239465657
B_98618_GTCTGTCACGCTGATCCATCAAGT	0.061174528	0.120993371	-0.125810118
B_98618_GTCTGTCAGCTCGGTAACCTCCAA	0.154696236	0.103584349	-0.246174947

B_98618_GTCTGTCATGGCTTCAAACTCACC	0.049030581	0.088017668	-0.116525753
B_98618_GTGTTCTAAAACATCGTAGGATGA	0.071529158	0.01708155	-0.145432515
B_98618_GTGTTCTAAACAACCAACGCTCGA	0.213307875	0.041006323	-0.119627968
B_98618_GTGTTCTAACAAGCTAAGATGTAC	0.078695709	0.034495503	-0.244851966
B_98618_GTGTTCTAACATTGGCACAGCAGA	0.205947043	0.312470345	-0.206727789
B_98618_GTGTTCTAACGTATCACAAGACTA	0.288903718	0.100561049	-0.346273127
B_98618_GTGTTCTAAGGCTAACAGTGGTCA	0.118334327	0.059922711	-0.371145423
B_98618_GTGTTCTAGAATCTGACTCAATGA	0.067406105	0.053073534	-0.209358342
B_98618_GTGTTCTAGAGTTAGCAAGGACAC	0.432577924	0.209512961	-0.214766901
B_98618_GTGTTCTATATCAGCAGCTCGGTA	0.145522942	0.069242673	-0.258712711
B_98618_GTGTTCTATGAAGAGAACGTATCA	0.290965754	-0.007970888	-0.435243099
B_98618_TAGGATGAAACCGAGATAGGATGA	0.204483249	0.044121152	-0.372054241
B_98618_TAGGATGAAAGAGATCAGAGTCAA	0.14910279	0.00317834	-0.461728956
B_98618_TAGGATGAACGTATCACATACCAA	0.200384891	-0.029252096	-0.329859753
B_98618_TAGGATGACGACACACACCACTGT	0.109050271	0.102409394	-0.097224903
B_98618_TAGGATGAGCGAGTAATGAAGAGA	0.324193543	0.108136326	-0.420525154
B_98618_TAGGATGAGTACGCAAACATTGGC	0.194397337	0.013689469	-0.252191131
B_98618_TAGGATGAGTCGTAGACTGGCATA	0.281357631	0.01293357	-0.301186661
B_98618_TAGGATGATGGTGGTAAGTCACTA	0.045232288	0.199672911	-0.227929345
B_98618_TAGGATGATGGTGGTAGTCTGTCA	0.223926483	0.155580622	-0.122770665
B_98618_TATCAGCAAAGGACACATCATTCC	-0.025910103	-0.095439226	-0.416057258
B_98618_TATCAGCAACGTATCACCAGTTCA	0.070218278	0.164976331	-0.398014691
B_98618_TATCAGCAAGTGGTCAAGTGGTCA	-0.085767985	0.175046096	-0.122578245
B_98618_TATCAGCACAGATCTGACAAGCTA	0.266954865	0.093993119	-0.141148652
B_98618_TATCAGCACTAAGGTCAACAACCA	0.096690665	0.146144089	-0.112323601
B_98618_TATCAGCAGTCGTAGAAACGCTTA	-0.009348594	0.130955773	-0.274406645
B_98618_TATCAGCATTCACGCACTGAGCCA	0.222583395	0.007286058	-0.423064217
B_98618_TCCGTCTAAACGCTTAATTGAGGA	0.140518207	-0.02185294	-0.353250152
B_98618_TCCGTCTAAGAGTCAAAGTCACTA	0.113036883	0.141526589	-0.216066203
B_98618_TCCGTCTACCGACAACTCTTCACA	0.213281761	0.104333893	-0.174957601
B_98618_TCCGTCTACCTAATCCAGTGGTCA	0.222333052	0.024154763	-0.438269148
B_98618_TCCGTCTACCTAATCCGTACGCAA	0.096207465	0.246187494	-0.217093475

B_98618_TCCGTCTACCTCCTGAGCTAACGA	0.070833869	-0.111674952	-0.322156556
B_98618_TCCGTCTACGCATACACGCTGATC	0.184018406	0.133123197	-0.096083458
B_98618_TCCGTCTAGCCAAGACCCTCTATC	0.340236368	-0.076268153	-0.368967923
B_98618_TCTTCACAAATGTTGCGATGAATC	0.063013303	0.18255112	-0.236336957
B_98618_TCTTCACAACCACTGTAATCCGTC	-0.023249289	0.009807347	-0.282771052
B_98618_TCTTCACAATAGCGACAAGACGGA	0.061281046	0.027339549	-0.347115202
B_98618_TCTTCACACAGATCTGAGATCGCA	0.238900753	0.108654466	-0.365907047
B_98618_TCTTCACACCATCCTCGCTAACGA	0.37328757	0.081493056	-0.154377592
B_98618_TCTTCACACCGACAACGCTAACGA	0.10301088	-0.10947908	-0.326070931
B_98618_TCTTCACACTGTAGCCCCAGTTCA	0.373178974	-0.01542972	-0.348084307
B_98618_TCTTCACAGTCTGTCAAACAACCA	0.142101395	0.107622443	-0.039769867
B_98618_TCTTCACATATCAGCACTGAGCCA	0.5053208	0.121413014	-0.293726965
B_98618_TGAAGAGAAAGAGATCAGTGGTCA	-0.003716552	0.082564536	-0.198147081
B_98618_TGAAGAGAAAGGACACCGGATTGC	0.211621101	0.132906858	-0.271232833
B_98618_TGAAGAGAAGAGTCAAAAGGACAC	0.006447243	0.060385358	-0.259016666
B_98618_TGAAGAGAAGTGGTCAAGATGTAC	0.086609427	-0.13725603	-0.459895072
B_98618_TGAAGAGAATGCCTAAGACTAGTA	-0.026401771	0.039508115	-0.224545068
B_98618_TGAAGAGACGAACTTAGAATCTGA	0.174492323	0.009512032	-0.211140243
B_98618_TGAAGAGAGAGTTAGCGTCGTAGA	0.301024059	0.065685726	-0.286500126
B_98618_TGAAGAGAGGTGCGAAGATAGACA	0.173074499	0.005612714	-0.265281946
B_98618_TGAAGAGAGTGTTCTACAGATCTG	0.258401626	0.116839406	-0.293597916
B_98618_TGAAGAGATCCGTCTAATCATTCC	0.175253427	0.00478804	-0.214176308
B_98618_TGAAGAGATGAAGAGAGTACGCAA	0.063587532	0.015869259	-0.308206074
B_98618_TGAAGAGATTCACGCACTCAATGA	-0.187831886	0.221251363	-0.121401176
B_98618_TGGAACAAAAGAGATCCCATCCTC	0.10040254	0.014235508	-0.444831172
B_98618_TGGAACAACACCTTACCATACCAA	0.16383821	-0.017125605	-0.304935311
B_98618_TGGAACAACGAACTTAACCACTGT	0.19923268	0.122571518	-0.399701575
B_98618_TGGAACAACGACACACGCCACATA	0.384262169	0.220501633	-0.206972014
B_98618_TGGAACAACGCTGATCGCTAACGA	0.073649641	0.090997054	-0.338696078
B_98618_TGGAACAACTCAATGAATTGGCTC	0.11768242	0.151559104	-0.231822986
B_98618_TGGAACAACTGTAGCCCTAAGGTC	0.087210109	0.053119155	0.068843208
B_98618_TGGAACAAGCTCGGTACACCTTAC	0.096326961	0.05244496	-0.2874542

B_98618_TGGAACAATAGGATGACCTCTATC	0.186553098	0.148704367	-0.074311356
B_98618_TGGCTTCAACCACTGTAGATGTAC	-0.008467166	0.068871866	-0.405998573
B_98618_TGGCTTCAACGCTCGAATCCTGTA	0.228691428	0.130983982	-0.380787064
B_98618_TGGCTTCACAAGACTACCTCTATC	0.051846136	0.050503367	-0.183615955
B_98618_TGGCTTCAGAGTTAGCACGTATCA	0.32620902	0.1614881	-0.403863661
B_98618_TGGCTTCAGATGAATCACCACTGT	0.255325176	0.094050141	-0.019601049
B_98618_TGGCTTCAGCGAGTAACCTAATCC	0.210096525	0.097153435	-0.21757123
B_98618_TGGCTTCAGTCTGTCAAGATCGCA	0.308041618	0.050919962	-0.406050892
B_98618_TGGCTTCATCTTCACAACCTCCAA	-0.003705047	0.030736177	-0.415869882
B_98618_TGGCTTCATGGAACAATGGAACAA	0.181631878	0.185649677	-0.153390593
B_98618_TGGTGGTAAACAACCACCGTGAGA	0.134310313	0.141565619	-0.352536021
B_98618_TGGTGGTACAACCACATCCGTCTA	0.120489288	0.0500989	-0.164528447
B_98618_TGGTGGTACAGATCTGAAACATCG	0.160985801	0.023646738	-0.122609633
B_98618_TTCACGCAAACCGAGAGTCTGTCA	0.16746062	0.089907198	-0.216190362
B_98618_TTCACGCAAACGTGATACAGCAGA	0.122060126	0.067892393	-0.284650031
B_98618_TTCACGCACCTCCTGAATCCTGTA	0.307699269	0.160331396	-0.152620133
B_98618_TTCACGCACGACACACCCGACAAC	0.217743504	-0.01918125	-0.440753725
B_98618_TTCACGCAGGTGCGAACACTTCGA	0.313653907	0.131280188	-0.408678434
B_98618_TTCACGCAGTACGCAAACCACTGT	0.326003252	-0.020168189	-0.220601034
B_98618_TTCACGCAGTCTGTCACCAGTTCA	0.238146548	0.044304335	-0.195494971
B_98618_AAACATCGAAGGTACAACAGATTC	0.049778568	0.110578337	-0.266708752
B_98618_AAACATCGACAAGCTACCGACAAC	0.058616505	-0.029300639	-0.250775345
B_98618_AAACATCGACACGACCACAAGCTA	0.151777707	0.038931682	-0.453453821
B_98618_AAACATCGACGCTCGAACGCTCGA	0.03722422	0.20824463	-0.258644317
B_98618_AAACATCGACGTATCAAACCGAGA	0.171366479	0.208850928	-0.131833023
B_98618_AAACATCGCAGCGTTAAGCCATGC	-0.04473042	0.176385554	-0.166433191
B_98618_AAACATCGCGACTGGACGACACAC	0.236162973	0.128216028	-0.432307814
B_98618_AAACATCGCGCATACAATTGGCTC	0.324002683	0.054833324	-0.166878598
B_98618_AAACATCGTAGGATGAGATGAATC	0.13951778	0.082809518	-0.195678451
B_98618_AACAACCAAACGCTTACTGTAGCC	0.194992477	0.127288614	-0.276831703
B_98618_AACAACCACTGGCATAAGCCATGC	0.206792912	0.029638561	-0.401969235
B_98618_AACAACCAGGAGAACAAACAACCA	0.132125908	0.020081022	-0.524146035

B_98618_AACCGAGAAAACATCGTGGCTTCA	0.164373439	0.071603796	-0.360666233
B_98618_AACCGAGAACAGATTCAAGGACAC	0.216709525	-0.021305191	-0.434662681
B_98618_AACCGAGACGAACTTACCGAAGTA	0.018656847	-0.101817469	-0.256653839
B_98618_AACCGAGACTAAGGTCACAGATTC	-0.077186919	-0.006616156	-0.357044295
B_98618_AACCGAGAGATGAATCAAACATCG	0.064168501	0.072154818	-0.333623739
B_98618_AACCGAGAGCGAGTAACCATCCTC	0.019083229	0.098400103	-0.305141212
B_98618_AACGCTTAAGCCATGCACGTATCA	0.115836378	0.195359152	-0.378090377
B_98618_AACGCTTAAGCCATGCATGCCTAA	-0.003568814	-0.0138358	-0.4495188
B_98618_AACGCTTAAGCCATGCTTCACGCA	0.025254544	0.01669835	-0.426450726
B_98618_AACGCTTAAGTCACTAACCTCCAA	-0.053475133	0.084992204	-0.413775543
B_98618_AACGCTTAATAGCGACAGCCATGC	0.264461039	0.029065603	-0.47970951
B_98618_AACGCTTACATACCAACGCTGATC	0.162332489	-0.058634427	-0.416569922
B_98618_AACGCTTACTGAGCCAACGCTCGA	0.079472971	0.185462788	-0.329358363
B_98618_AACGTGATAACGCTTAAAACATCG	0.175769701	0.03032978	-0.337205272
B_98618_AACGTGATAACTCACCGGAGAACA	-0.099351841	0.081681634	-0.396671934
B_98618_AACGTGATACGTATCAATTGAGGA	0.148948399	0.045899641	-0.117938571
B_98618_AACGTGATCAACCACACGAACTTA	0.004691606	0.082456226	-0.42003421
B_98618_AACGTGATCCAGTTCACCGAAGTA	-0.007316159	0.048565699	-0.418567498
B_98618_AACGTGATCGCATACAAGCCATGC	0.246884982	0.043414874	-0.412036824
B_98618_AACGTGATCTGTAGCCCTGGCATA	-0.085410435	-0.093182225	-0.389833719
B_98618_AACGTGATGCTCGGTATCTTCACA	-0.062374527	-0.021057715	-0.179359518
B_98618_AACGTGATTGAAGAGAATGCCTAA	0.087712313	-0.01940992	-0.382576943
B_98618_AACTCACCAAGGTACAAGATCGCA	0.407019167	0.369024587	-0.129166212
B_98618_AACTCACCACGCTCGAACGTATCA	0.454026275	-0.021011099	-0.327780597
B_98618_AAGACGGAAACGCTTAACCTCCAA	0.143787092	0.103572186	-0.367637672
B_98618_AAGACGGAACAGATTCATCCTGTA	-0.034487863	0.131767507	-0.438288963
B_98618_AAGACGGAACGCTCGAGCCACATA	0.117031508	0.007523763	-0.303627133
B_98618_AAGACGGACAACCACAAGTACAAG	0.074135373	-0.038385695	-0.44656376
B_98618_AAGACGGACAACCACACGCATACA	0.157256878	-0.102689752	-0.540586954
B_98618_AAGACGGACCGACAACAAGGTACA	0.370434269	0.044249643	-0.448318581
B_98618_AAGACGGAGACAGTGCATCCTGTA	-0.054437825	0.023205647	-0.387027419
B_98618_AAGACGGAGTACGCAAAGGCTAAC	-0.173144264	0.044047423	-0.262238601

B_98618_AAGAGATCATCCTGTACCTAATCC	0.229464107	0.121773937	-0.139667698
B_98618_AAGAGATCCCTCTATCCGCTGATC	0.04418124	-0.064024895	-0.272219884
B_98618_AAGGACACAACAACCACCGACAAC	0.179064788	0.120719058	-0.332146519
B_98618_AAGGACACAATCCGTCCCTAATCC	0.173620926	0.158897486	-0.205468355
B_98618_AAGGACACACACGACCAACAACCA	-0.058660006	0.079720785	-0.321996412
B_98618_AAGGACACACATTGGCACATTGGC	0.440216978	0.18597157	-0.493432907
B_98618_AAGGACACGACTAGTACCATCCTC	0.325457577	0.325140499	-0.22928527
B_98618_AAGGACACGCGAGTAAAAGGTACA	0.036375596	-0.041519214	-0.409750314
B_98618_AAGGACACTGGTGGTACCGACAAC	0.016311775	0.122379928	-0.294947787
B_98618_AAGGTACAATAGCGACTTCACGCA	0.10190057	-0.013730286	-0.305732053
B_98618_AAGGTACACTGGCATACAGATCTG	0.289824554	0.024599827	-0.25397523
B_98618_AAGGTACAGAACAGGCCTCAATGA	-0.003334944	0.029775367	-0.315944706
B_98618_AAGGTACAGGTGCGAACAATGGAA	-0.009492399	0.185009684	-0.398477496
B_98618_AATCCGTCCGAACTTACGGATTGC	0.110176896	0.153220067	-0.483297127
B_98618_AATCCGTCTATCAGCACCGAAGTA	0.260864476	0.083169421	-0.271273748
B_98618_AATGTTGCAAGGTACAAGTGGTCA	-0.192408543	-0.016364276	-0.373552537
B_98618_AATGTTGCACAGATTCACGTATCA	0.165139598	0.107783028	-0.372709262
B_98618_AATGTTGCAGGCTAACCTAAGGTC	0.26890386	0.01314049	-0.339043038
B_98618_AATGTTGCCGACTGGACCGACAAC	0.046172857	0.215390717	-0.317689548
B_98618_AATGTTGCGACTAGTAACGTATCA	0.271814319	0.042206875	-0.372075006
B_98618_AATGTTGCGCGAGTAAAACGTGAT	0.035505273	0.072486909	-0.422985882
B_98618_AATGTTGCTAGGATGAAACGCTTA	0.185582436	0.124339289	-0.174683619
B_98618_AATGTTGCTCCGTCTAAGCAGGAA	0.217478193	-0.01685474	-0.244238752
B_98618_AATGTTGCTGGAACAACCAGTTCA	0.386619566	0.095137483	-0.347553809
B_98618_ACAAGCTAAGTACAAGAAGGACAC	-0.174560944	-0.049665723	-0.530334775
B_98618_ACAAGCTAGCCACATAGGTGCGAA	0.181389996	-0.016039262	-0.398264462
B_98618_ACAAGCTATGAAGAGACAACCACA	0.063196036	0.013782949	-0.4714807
B_98618_ACAAGCTATGGTGGTAGATGAATC	0.200101461	0.017667232	-0.538209276
B_98618_ACACAGAAACAGCAGATAGGATGA	0.119790758	0.080014427	-0.378546901
B_98618_ACACAGAAAGAGTCAATCTTCACA	0.005209728	0.095975891	-0.368438152
B_98618_ACACAGAACCTCTATCCCTCTATC	0.131678245	0.057904999	-0.422251258
B_98618_ACACAGAAGGAGAACAATCCTGTA	-0.001670218	0.21496267	-0.072878666

B_98618_ACACGACCAACCGAGAAGCACCTC	0.201022606	-0.084693078	-0.391643784
B_98618_ACACGACCAAGGACACCTGAGCCA	0.199163909	0.102901752	-0.151757677
B_98618_ACACGACCACAAGCTAAATGTTGC	0.026172672	0.245133456	-0.193160706
B_98618_ACACGACCAGCAGGAAGGAGAACA	0.28542093	0.171644026	-0.432918612
B_98618_ACACGACCGAGCTGAACGAACTTA	0.098301276	-0.095878095	-0.382238074
B_98618_ACACGACCTGGAACAAACAAGCTA	0.100025824	0.011406344	-0.488516753
B_98618_ACAGATTCAACGTGATTGAAGAGA	0.236137561	0.13924453	-0.34550675
B_98618_ACAGATTCACGCTCGACGACTGGA	0.072050655	0.102027274	-0.336532296
B_98618_ACAGATTCAGCAGGAAACATTGGC	0.144023412	0.121391659	-0.208456845
B_98618_ACAGATTCCAAGGAGCAAGGTACA	0.207865279	0.031098386	-0.447940829
B_98618_ACAGATTCCATACCAAACGCTCGA	0.455228049	0.110974195	-0.26459962
B_98618_ACAGATTCTGAAGAGAAGCAGGAA	0.385683394	0.101638549	-0.441086207
B_98618_ACAGCAGAAAGGTACAAACGCTTA	-0.025711579	0.122891057	-0.419838663
B_98618_ACAGCAGACAAGGAGCACAGCAGA	0.208440878	0.226963978	-0.088931135
B_98618_ACAGCAGACCGACAACCGGATTGC	0.189166218	0.01476943	-0.313954368
B_98618_ACAGCAGACTCAATGAAGTGGTCA	0.157544528	0.122774845	-0.376828354
B_98618_ACAGCAGAGAGTTAGCACGTATCA	0.105099139	-0.006853056	-0.379722132
B_98618_ACAGCAGAGCCAAGACATCCTGTA	0.239520751	0.008249006	-0.426715702
B_98618_ACAGCAGAGTCTGTCAAACAACCA	0.174297324	0.054362852	-0.387178273
B_98618_ACAGCAGATCTTCACACCGACAAC	0.035463272	-0.131810386	-0.563799059
B_98618_ACATTGGCAGTCACTAACAGATTC	0.015037738	0.133119599	-0.09155723
B_98618_ACATTGGCCATCAAGTACCTCCAA	0.299903231	0.054923513	-0.418369328
B_98618_ACATTGGCCCTCCTGATGGCTTCA	0.004327275	0.084992981	-0.396841918
B_98618_ACATTGGCCGACTGGAGATGAATC	0.053668914	0.075638686	-0.27530972
B_98618_ACCACTGTAAGGACACCTGGCATA	0.067028986	0.052804515	-0.068478399
B_98618_ACCACTGTAGATCGCACCATCCTC	-0.08868544	0.142533836	-0.290460808
B_98618_ACCACTGTCAGATCTGTGGCTTCA	0.304605164	0.148451252	-0.105175804
B_98618_ACCACTGTCCGAAGTAGAGTTAGC	0.292623437	0.050033423	-0.332900851
B_98618_ACCACTGTCCGACAACCAACCACA	-0.018511915	0.130221809	-0.30920357
B_98618_ACCTCCAACAAGGAGCAACGCTTA	0.323439523	0.015971691	-0.27340049
B_98618_ACCTCCAATTCACGCAGGTGCGAA	0.088715514	0.164531418	-0.438897041
B_98618_ACGCTCGACCGACAACAGTGGTCA	-0.015934367	0.181404333	-0.090964889

B_98618_ACGCTCGAGACTAGTAACCTCCAA	0.324625411	0.204724419	-0.211861659
B_98618_ACGTATCAAACTCACCACTATGCA	0.225313921	0.02632706	-0.32568274
B_98618_ACGTATCAATCATTCCACCACTGT	0.337900791	0.05875607	-0.155037565
B_98618_ACGTATCAATTGGCTCCCTCTATC	0.210316585	-0.025442197	-0.260836747
B_98618_ACGTATCACAACCACAAGTACAAG	0.205177154	0.207986266	-0.160093553
B_98618_ACGTATCACCGTGAGAAGCCATGC	-0.031373927	0.233217147	-0.378302401
B_98618_ACGTATCACTCAATGAAGATGTAC	0.106766939	0.066441627	-0.413740744
B_98618_ACGTATCAGACTAGTAGTGTTCTA	0.412988463	0.203183503	-0.475768435
B_98618_ACGTATCAGCTAACGATGGAACAA	0.249356917	0.087288873	-0.449230543
B_98618_ACTATGCAAACGCTTATTCACGCA	0.080334165	-0.041077023	-0.422476635
B_98618_ACTATGCAACCACTGTTCTTCACA	0.42810663	0.110148708	-0.257140372
B_98618_ACTATGCACATACCAAGAGCTGAA	0.369626429	0.015487529	-0.258321052
B_98618_ACTATGCACTAAGGTCCAGCGTTA	0.253918378	0.048756757	-0.417947512
B_98618_ACTATGCAGGTGCGAAGATAGACA	0.2827582	0.049506779	-0.307676696
B_98618_AGAGTCAAAACTCACCGTACGCAA	0.284885616	-0.015591712	-0.182574377
B_98618_AGAGTCAAACAAGCTAAGTCACTA	0.183084572	0.255021273	-0.43951073
B_98618_AGAGTCAAAGCCATGCAGTACAAG	0.575365293	-0.012537719	-0.498732165
B_98618_AGAGTCAAAGGCTAACATGCCTAA	0.084791432	-0.008299173	-0.453985032
B_98618_AGAGTCAAAGTACAAGCGCTGATC	0.284565325	0.052743224	-0.373499161
B_98618_AGAGTCAAATCCTGTAACAAGCTA	0.108830031	0.102077674	-0.397067221
B_98618_AGAGTCAAGAGCTGAAGATAGACA	0.082852222	-0.046135538	-0.414505659
B_98618_AGATCGCAACACAGAATCCGTCTA	0.05700459	0.14092659	-0.32029395
B_98618_AGATCGCAACGTATCAAAACATCG	-0.037485846	0.063107245	-0.490770754
B_98618_AGATCGCACTGTAGCCCAAGACTA	0.113854754	0.143727715	-0.429813393
B_98618_AGATCGCATGAAGAGACGCATACA	0.047844426	-0.012607634	-0.443804181
B_98618_AGATGTACAACCGAGAGTCGTAGA	0.261384436	0.193153887	-0.469709556
B_98618_AGATGTACGAATCTGACCGTGAGA	0.022340533	0.11987525	-0.280202379
B_98618_AGATGTACGCCACATAATGCCTAA	0.160529615	0.12072607	-0.334634521
B_98618_AGATGTACTGGAACAATAGGATGA	0.29106589	0.215137968	-0.378459637
B_98618_AGCACCTCAACTCACCAAGAGATC	0.338630495	-0.072739312	-0.197895733
B_98618_AGCACCTCACAGATTCAGCCATGC	-0.080549278	0.010394868	-0.456791431
B_98618_AGCACCTCCTGAGCCAATTGGCTC	0.605108613	0.167528067	-0.223527353

B_98618_AGCACCTCTGGAACAAACAGCAGA	-0.047357991	0.075339162	-0.530446631
B_98618_AGCAGGAAAACGCTTACGGATTGC	0.192284762	-0.012910765	-0.421671176
B_98618_AGCAGGAAACTATGCAAGTGGTCA	0.008627899	0.14746486	-0.382299902
B_98618_AGCAGGAAAGCCATGCCTGAGCCA	0.395270618	-0.034999729	-0.334963613
B_98618_AGCAGGAAATCATTCCGAGCTGAA	0.185114895	0.145141655	-0.357805869
B_98618_AGCAGGAACCGTGAGACCGACAAC	0.14414756	0.171328462	-0.231431329
B_98618_AGCAGGAACGACACACAAGGACAC	0.161287015	0.151215546	-0.43366086
B_98618_AGCCATGCAACGTGATATTGGCTC	0.484502045	0.054074862	-0.257541558
B_98618_AGCCATGCCCGACAACCAACCACA	0.09419715	-0.02302931	-0.421500236
B_98618_AGCCATGCCGGATTGCAAGGTACA	0.225300727	0.111125652	-0.185975773
B_98618_AGCCATGCGCCACATAGACAGTGC	0.132774609	0.146243443	-0.401956272
B_98618_AGGCTAACAGTACAAGCGAACTTA	0.254011413	0.225062661	-0.174004651
B_98618_AGGCTAACCATACCAACCATCCTC	0.059245015	0.114744763	-0.060850855
B_98618_AGTACAAGAAGACGGAAATGTTGC	0.444579529	0.012795079	-0.445097547
B_98618_AGTACAAGACACGACCAAGAGATC	0.072327254	0.070726902	-0.469723653
B_98618_AGTACAAGACAGCAGAGTGTTCTA	-0.019649483	0.044055829	-0.311210096
B_98618_AGTACAAGACCTCCAAGAACAGGC	0.065385514	0.091493984	-0.416928735
B_98618_AGTACAAGAGATCGCAGAACAGGC	0.497118645	0.175126941	-0.197460058
B_98618_AGTACAAGAGATGTACATCCTGTA	0.131459606	0.056047833	-0.278309209
B_98618_AGTACAAGATTGGCTCAGCACCTC	0.449527154	0.139483043	-0.238166711
B_98618_AGTACAAGCAAGACTACAAGACTA	0.165962368	0.123839693	-0.327974911
B_98618_AGTACAAGCAAGACTAGAGTTAGC	0.193575607	0.076202875	-0.432697186
B_98618_AGTACAAGCACTTCGACGACTGGA	0.200746708	0.075594294	-0.477533844
B_98618_AGTACAAGCAGCGTTAATCCTGTA	-0.098936141	0.005610152	-0.164022599
B_98618_AGTACAAGCTGGCATAACAGATTC	0.232136819	0.019241981	-0.37022993
B_98618_AGTACAAGGAATCTGACTCAATGA	0.09907332	0.157633323	-0.268647882
B_98618_AGTCACTAAAACATCGCTCAATGA	0.038173898	0.116886953	-0.383176667
B_98618_AGTCACTAAGTCACTAAGCACCTC	-0.224138907	0.014265666	-0.253399657
B_98618_AGTCACTACAAGACTATGGTGGTA	-0.049688125	0.106377025	-0.271915638
B_98618_AGTCACTACATACCAAAGCACCTC	0.058175991	0.036626689	-0.298378152
B_98618_AGTCACTACCATCCTCCCTCTATC	0.300745144	0.021892298	-0.436278982
B_98618_AGTCACTACCGTGAGACGAACTTA	-0.048667925	-0.015484794	-0.440683875

B_98618_AGTCACTAGAACAGGCGATGAATC	0.184704284	0.251033664	-0.439711068
B_98618_AGTCACTAGCCAAGACCTGGCATA	0.21547827	0.12789871	-0.395476652
B_98618_AGTCACTAGGAGAACAGCTCGGTA	0.572623521	0.160394316	-0.334961261
B_98618_AGTCACTATCTTCACAAAGAGATC	0.203622949	-0.005558038	-0.32779389
B_98618_AGTCACTATGGTGGTACAGATCTG	0.485182374	0.110644938	-0.352606548
B_98618_AGTGGTCAAAGAGATCCCTAATCC	-0.036738369	-0.040522155	-0.329238814
B_98618_AGTGGTCAAAGAGATCGTCTGTCA	0.02245789	-0.065118903	-0.512534594
B_98618_AGTGGTCAAGTGGTCACCAGTTCA	0.193532227	0.222687721	-0.133998285
B_98618_AGTGGTCACAAGGAGCCTGAGCCA	-0.040990696	0.092552787	-0.315750304
B_98618_AGTGGTCACTGAGCCACAGATCTG	0.338079631	0.066005881	-0.432459361
B_98618_AGTGGTCATATCAGCAGACAGTGC	-0.052607796	0.020901479	-0.436490172
B_98618_ATAGCGACAACGCTTACAGATCTG	-0.056177596	-0.090545687	-0.455576286
B_98618_ATAGCGACACAGATTCAACGTGAT	0.009649645	0.032435377	-0.303947994
B_98618_ATAGCGACACCTCCAAGCTAACGA	-0.049583032	0.072043484	-0.422853465
B_98618_ATAGCGACCAAGGAGCGAACAGGC	0.044607229	-0.036372469	-0.369403399
B_98618_ATAGCGACCACCTTACACACGACC	0.46913546	0.139214046	-0.45779622
B_98618_ATAGCGACGATAGACACAACCACA	0.303947258	-0.016107558	-0.172666457
B_98618_ATAGCGACGCTAACGACACCTTAC	0.082142788	0.041209899	-0.43917366
B_98618_ATAGCGACTAGGATGAAAGGTACA	0.256478685	0.130503031	-0.402066093
B_98618_ATCATTCCAAACATCGAAGAGATC	0.379792519	0.148992535	-0.311131907
B_98618_ATCATTCCCTGAGCCAAATCCGTC	0.253366198	0.038699936	-0.398840439
B_98618_ATCATTCCGCCAAGACTGGAACAA	0.388080687	0.193993086	-0.486203531
B_98618_ATCCTGTACATACCAAATCATTCC	0.290256448	-0.023641005	-0.173794123
B_98618_ATCCTGTACGCTGATCAGTACAAG	0.15938941	0.02262921	-0.451401918
B_98618_ATCCTGTAGGAGAACATGGTGGTA	0.319106591	0.264302621	-0.272904233
B_98618_ATCCTGTAGTCGTAGACGACTGGA	0.057825426	0.170956664	-0.249105263
B_98618_ATCCTGTATGGAACAAACCTCCAA	-0.031428726	0.008893671	-0.289558809
B_98618_ATGCCTAAACAAGCTACCGACAAC	0.248493932	0.169817771	-0.010388384
B_98618_ATGCCTAAAGCCATGCATTGGCTC	0.182791709	0.090267941	-0.460000464
B_98618_ATGCCTAAGTACGCAAGGAGAACA	-0.010155151	0.119229511	-0.382012186
B_98618_ATGCCTAATATCAGCACGCATACA	0.00923032	0.121357935	-0.190661534
B_98618_ATTGAGGAACAGATTCCAGATCTG	0.275148051	0.131664697	-0.295189096

B_98618_ATTGAGGAACAGCAGACTGGCATA	-0.041551288	-0.124356035	-0.413021517
B_98618_ATTGAGGAACGTATCAATTGAGGA	0.166639452	0.088240904	-0.2702106
B_98618_ATTGAGGAATCCTGTAGAGTTAGC	0.045643297	0.036857002	-0.31680558
B_98618_ATTGAGGAGGTGCGAAACAGATTC	0.361922291	-0.020320204	-0.431917982
B_98618_ATTGAGGATCCGTCTACGGATTGC	0.248226964	-0.026060922	-0.323445486
B_98618_ATTGGCTCAAGGTACAGCCAAGAC	0.106468125	-0.070188354	-0.376662145
B_98618_ATTGGCTCACAAGCTAATCCTGTA	-0.024365004	0.080937129	-0.389240354
B_98618_ATTGGCTCACAGCAGAGCGAGTAA	0.372082413	0.051012326	-0.489922429
B_98618_ATTGGCTCCTGGCATAGCCAAGAC	0.112038022	0.13370036	-0.214220282
B_98618_ATTGGCTCTAGGATGAACATTGGC	0.345596673	0.16833325	-0.418874533
B_98618_CAACCACAAAGGACACCCTCCTGA	0.157261049	0.096796109	-0.179006654
B_98618_CAACCACAACACAGAACAAGACTA	0.421217249	0.465298429	-0.09400795
B_98618_CAACCACAAGATCGCAGACTAGTA	0.156571628	0.066179981	0.0018407
B_98618_CAACCACAAGCCATGCGCGAGTAA	0.142578279	0.305432372	-0.148326362
B_98618_CAACCACAAGCCATGCTCTTCACA	-0.092824173	0.042659899	-0.202989948
B_98618_CAACCACACAGATCTGCCATCCTC	0.175183019	0.07341433	-0.404005777
B_98618_CAACCACACCTAATCCTGAAGAGA	0.179801446	0.240735904	-0.484697552
B_98618_CAACCACACGCATACACGGATTGC	0.197929128	0.100292836	-0.388214911
B_98618_CAACCACAGAACAGGCCACTTCGA	0.114666665	0.032817053	-0.306159842
B_98618_CAAGACTAAGCCATGCAGCAGGAA	-0.036277294	0.105588423	-0.290172957
B_98618_CAAGACTACCGTGAGATATCAGCA	0.263434143	0.030989735	-0.341511067
B_98618_CAAGACTACTAAGGTCAGTCACTA	0.176120908	0.172876261	-0.158947939
B_98618_CAAGACTATATCAGCAAATGTTGC	0.166992432	0.25687467	-0.169477997
B_98618_CAAGGAGCAACTCACCCACCTTAC	0.206699443	0.035265865	-0.371148476
B_98618_CAAGGAGCACCACTGTCATACCAA	-0.087837526	0.097504518	-0.343376034
B_98618_CAAGGAGCACGTATCAACAAGCTA	-0.051491748	0.032395424	-0.530542319
B_98618_CAAGGAGCATTGAGGACAGCGTTA	0.29290987	0.050212246	-0.351674758
B_98618_CAAGGAGCCACCTTACCCATCCTC	0.19058386	0.048663519	-0.267530947
B_98618_CAATGGAAATTGAGGAGAGTTAGC	0.328739252	-0.002635795	-0.389380762
B_98618_CAATGGAACACCTTACAACTCACC	0.211299737	0.153585123	-0.109422812
B_98618_CAATGGAACTCAATGATTCACGCA	0.084694431	-0.055077143	-0.36719679
B_98618_CACCTTACACACAGAAATTGAGGA	0.162403269	0.147588941	-0.245197107

B_98618_CACCTTACAGCACCTCAGTGGTCA	0.167560206	0.096159343	-0.441936445
B_98618_CACTTCGAAACAACCACTGAGCCA	0.320566619	0.166972958	-0.399474997
B_98618_CACTTCGACAACCACACGCATACA	0.293772651	0.053433985	-0.579382877
B_98618_CACTTCGACGACACACCTGTAGCC	0.122051352	0.107154814	-0.34048474
B_98618_CACTTCGACGGATTGCCCTCTATC	-0.000260749	0.029313664	-0.425968229
B_98618_CACTTCGAGAATCTGAAGCCATGC	0.258929457	0.019924791	-0.372787346
B_98618_CACTTCGAGTGTTCTAATCATTCC	0.105273066	0.029486152	-0.33269895
B_98618_CACTTCGATGGTGGTAATGCCTAA	0.339084419	-0.009895695	-0.275445888
B_98618_CAGATCTGGCCACATAATCCTGTA	0.274850594	0.154373255	-0.294107463
B_98618_CAGATCTGGGAGAACACATACCAA	0.443911083	-0.104618359	-0.392696511
B_98618_CAGCGTTACGCATACACTGTAGCC	0.094867844	0.032622127	-0.308755236
B_98618_CAGCGTTATCTTCACACCTAATCC	0.506186728	0.250705539	-0.334456439
B_98618_CATACCAAAAGACGGACATACCAA	0.149681584	0.15797456	-0.327634544
B_98618_CATACCAAACCACTGTAAGAGATC	0.216452092	0.02358413	-0.320468007
B_98618_CATACCAAAGTGGTCAAGATGTAC	0.060513278	0.176518597	-0.333331338
B_98618_CATACCAACGCATACACCGTGAGA	0.215601516	0.095801173	-0.204571345
B_98618_CATCAAGTAGCACCTCATCCTGTA	-0.070694495	-0.076756478	-0.42116847
B_98618_CATCAAGTAGCAGGAAGTCTGTCA	0.354077529	0.180387648	-0.248257233
B_98618_CATCAAGTATTGGCTCACCTCCAA	0.142437842	0.182655892	-0.303674529
B_98618_CATCAAGTCAGCGTTACAAGACTA	0.351734161	0.09300607	-0.148146129
B_98618_CATCAAGTCATCAAGTAACGTGAT	0.092866083	-0.004253046	-0.474237814
B_98618_CATCAAGTCCGTGAGAAGATGTAC	0.338333211	-0.022489534	-0.252677066
B_98618_CATCAAGTGAACAGGCCAACCACA	0.159058901	0.083552717	-0.508309284
B_98618_CATCAAGTGAGCTGAAAACCGAGA	0.180664867	0.05414644	-0.43026123
B_98618_CATCAAGTTAGGATGACGAACTTA	0.258139098	0.233234089	-0.141964258
B_98618_CATCAAGTTATCAGCAACAAGCTA	-0.163789835	-0.050876837	-0.209008912
B_98618_CATCAAGTTCCGTCTAGCCAAGAC	0.115254203	-0.046358461	-0.412674654
B_98618_CCAGTTCAAACGTGATCCGACAAC	0.145822373	-0.06256279	-0.433465945
B_98618_CCATCCTCATTGAGGACGCTGATC	0.120106681	0.120095666	-0.378880507
B_98618_CCATCCTCTTCACGCACCGACAAC	-0.093535448	0.046158946	-0.362693855
B_98618_CCGAAGTAAACAACCAGTCTGTCA	0.191610614	-0.010245934	-0.442892735
B_98618_CCGAAGTAAACCGAGAAATGTTGC	0.208757264	-0.0390968	-0.40223016

B_98618_CCGAAGTAAAGAGATCCTGTAGCC	0.063438842	0.039981769	-0.497295152
B_98618_CCGAAGTAAGATGTACTATCAGCA	0.339445185	0.066146167	-0.262193697
B_98618_CCGAAGTACAATGGAATCCGTCTA	0.115346449	0.008551667	-0.343917863
B_98618_CCGAAGTACCGAAGTAAGATCGCA	0.019944461	0.108901943	-0.379043771
B_98618_CCGAAGTACCGTGAGAAGGCTAAC	-0.323167832	-0.037646367	-0.402759648
B_98618_CCGAAGTACGACTGGACCGAAGTA	0.268801829	0.099378335	-0.202949359
B_98618_CCGAAGTACTGTAGCCATTGAGGA	0.095534107	-0.089330153	-0.51214124
B_98618_CCGACAACAACCGAGACAAGGAGC	0.142808377	0.189373338	-0.204051865
B_98618_CCGACAACACACGACCCAAGACTA	-0.075445382	0.056170863	-0.379873924
B_98618_CCGACAACCAGCGTTACAAGACTA	0.031641255	0.054470606	-0.378506017
B_98618_CCGACAACCCTCTATCACGCTCGA	0.472259712	0.106757606	-0.355026449
B_98618_CCGACAACCGAACTTAACCTCCAA	0.679345868	0.235788068	-0.185140168
B_98618_CCGACAACGGAGAACAGTACGCAA	0.011675025	-0.0394495	-0.432488809
B_98618_CCGTGAGACTGAGCCAATTGGCTC	0.121556651	0.031072241	-0.221042144
B_98618_CCGTGAGATCCGTCTAAGTACAAG	0.001406258	0.06982736	-0.39643541
B_98618_CCTAATCCACAGATTCCCGACAAC	0.011841698	-0.014192757	-0.207448615
B_98618_CCTAATCCACTATGCAGCCAAGAC	-0.0743607	-0.018690917	-0.402717339
B_98618_CCTAATCCAGCCATGCACACAGAA	0.250815419	0.027980493	-0.28171072
B_98618_CCTAATCCGAATCTGACTGGCATA	0.485082728	0.150433539	-0.427568794
B_98618_CCTAATCCGTGTTCTAATCCTGTA	0.07042142	0.201347554	-0.25387861
B_98618_CCTCCTGAAACGTGATAGCCATGC	0.151801042	0.146788913	-0.148419504
B_98618_CCTCCTGAACTATGCATCCGTCTA	0.230778479	-0.005097576	-0.447266329
B_98618_CCTCCTGACACCTTACAGTACAAG	-0.057392474	-0.146665219	-0.514163421
B_98618_CCTCCTGACCAGTTCAGAACAGGC	0.147927808	0.285674991	-0.399598774
B_98618_CCTCCTGACCGAAGTAATTGAGGA	0.123617147	0.079829506	-0.45213418
B_98618_CCTCCTGAGTGTTCTAAATCCGTC	0.075726206	0.144203763	-0.288543481
B_98618_CCTCTATCAACGTGATACAGCAGA	0.138727932	0.099840933	0.036527308
B_98618_CCTCTATCAAGGACACAAGACGGA	0.528274103	-0.047991432	-0.514504483
B_98618_CCTCTATCAAGGTACACCTCCTGA	-0.029477196	0.099397761	-0.221962099
B_98618_CCTCTATCACAGCAGAACGTATCA	0.152159218	0.095578291	-0.395991475
B_98618_CCTCTATCAGCACCTCAATCCGTC	0.083167046	0.034335005	-0.19333883
B_98618_CCTCTATCATTGGCTCCATACCAA	-0.143067956	0.091679102	-0.417642524

B_98618_CCTCTATCTCCGTCTAACTATGCA	0.326713917	0.061705363	-0.52494118
B_98618_CCTCTATCTGGTGGTACATCAAGT	0.098641812	0.298183411	-0.453463688
B_98618_CGAACTTAACAGCAGACCATCCTC	0.091964749	0.07187433	-0.512642193
B_98618_CGAACTTACAAGACTAGATGAATC	0.145822812	-0.008604312	-0.338677438
B_98618_CGACACACACACGACCCAATGGAA	-0.06177864	0.006367594	-0.375297115
B_98618_CGACACACCAATGGAAGTGTTCTA	0.380934729	0.12419981	-0.388424817
B_98618_CGACACACGAATCTGACTAAGGTC	0.061142588	0.060420283	-0.401506656
B_98618_CGACTGGACCGTGAGACCAGTTCA	0.225005227	0.184969984	-0.38368399
B_98618_CGACTGGACCTCTATCAAGACGGA	0.153580354	0.117652118	-0.149786462
B_98618_CGACTGGACTGTAGCCCAGATCTG	0.027486028	0.219810389	-0.458146729
B_98618_CGCATACAAAGAGATCCAGATCTG	0.353854955	0.149328282	-0.384043565
B_98618_CGCATACAAATCCGTCGCGAGTAA	0.104802872	0.089813184	-0.273927187
B_98618_CGCATACAAGATGTACCTGGCATA	0.02950202	0.165162085	-0.253531487
B_98618_CGCATACAAGCACCTCCAACCACA	0.314310833	0.132910718	-0.459800604
B_98618_CGCATACACAAGACTATGAAGAGA	0.201432554	0.012855803	-0.223284826
B_98618_CGCATACACAAGGAGCTATCAGCA	0.053946828	0.193961132	-0.311504761
B_98618_CGCTGATCAAGGACACACAGATTC	0.068804018	0.122279414	-0.42910295
B_98618_CGCTGATCAGCACCTCCGAACTTA	0.284746337	-0.065536261	-0.373579623
B_98618_CGCTGATCCCATCCTCCGACACAC	0.336579512	0.058837689	-0.305933207
B_98618_CGCTGATCCGACACACCTCAATGA	0.326834885	0.028694015	-0.382107147
B_98618_CGCTGATCGACAGTGCAGATCGCA	0.261633925	0.020063288	-0.281038182
B_98618_CGCTGATCGCGAGTAAGAGCTGAA	-0.003097778	0.064005464	-0.480956503
B_98618_CGCTGATCTGGCTTCAAGGCTAAC	0.033913768	0.011865079	-0.449712981
B_98618_CGGATTGCACACGACCAGATGTAC	0.034854193	0.115828027	-0.358432261
B_98618_CGGATTGCAGCACCTCAAGAGATC	0.306229451	-0.068094862	-0.404828956
B_98618_CGGATTGCCAGCGTTAGAACAGGC	0.398655099	0.03392655	-0.249186108
B_98618_CGGATTGCCCATCCTCAAGACGGA	-0.055333978	0.097660891	-0.401245785
B_98618_CGGATTGCCCTCTATCCAAGGAGC	0.029805822	0.026619614	-0.488183962
B_98618_CTAAGGTCACAAGCTACCTCCTGA	-0.058648085	-0.035046575	-0.242499798
B_98618_CTAAGGTCCATACCAACAGCGTTA	-0.140698385	-0.063902685	-0.530526392
B_98618_CTAAGGTCGAGTTAGCCATACCAA	0.155862408	0.137562567	-0.293546377
B_98618_CTAAGGTCTTCACGCATGGCTTCA	0.111872973	-0.10429044	-0.312956592

B_98618_CTCAATGAAACTCACCAACTCACC	0.226451229	-0.091297572	-0.467548978
B_98618_CTCAATGAACGTATCAACCTCCAA	0.193596345	0.169487026	-0.497217638
B_98618_CTCAATGAAGCCATGCGAACAGGC	0.367995055	-0.015451761	-0.413758937
B_98618_CTCAATGAATGCCTAAGAGTTAGC	0.045655173	0.095339821	-0.345133565
B_98618_CTCAATGACAGCGTTAACATTGGC	0.132237292	0.128861599	-0.542784923
B_98618_CTCAATGATCTTCACACGACTGGA	-0.027168327	0.0847811	-0.453944958
B_98618_CTCAATGATGGTGGTACACTTCGA	0.462226265	0.093578536	-0.381839829
B_98618_CTGAGCCAAATCCGTCAGATCGCA	0.108018772	0.173752133	-0.320674142
B_98618_CTGAGCCAAATGTTGCCCGACAAC	0.241212189	0.023077699	-0.115716154
B_98618_CTGAGCCATGGTGGTACTGGCATA	0.330623954	-0.065750163	-0.323926196
B_98618_CTGGCATAAACTCACCAACGCTTA	0.133157528	0.054422168	-0.416294163
B_98618_CTGGCATAAAGACGGACCATCCTC	0.096852943	0.117265115	-0.264188603
B_98618_CTGGCATAATCATTCCAACTCACC	-0.151824378	-0.104329143	-0.437653751
B_98618_CTGGCATACACTTCGAACACAGAA	0.415652924	-0.049172161	-0.562062483
B_98618_CTGGCATAGCCACATAAACTCACC	0.175991766	0.146135855	-0.450347461
B_98618_CTGGCATAGCCACATAATGCCTAA	0.338054791	0.179180612	-0.023639855
B_98618_CTGTAGCCAGTGGTCACAAGACTA	0.245792231	-0.024159639	-0.295505468
B_98618_CTGTAGCCGCCACATAAGTACAAG	0.330558729	0.133396629	-0.449138588
B_98618_CTGTAGCCGGAGAACAACAGATTC	0.2759019	0.224224056	-0.368184243
B_98618_GAACAGGCAGATGTACCTGAGCCA	0.14326522	0.068108927	-0.329139099
B_98618_GAACAGGCCGAACTTAGCCACATA	-0.019749356	0.104958405	-0.402301972
B_98618_GAACAGGCCTAAGGTCCATCAAGT	0.133657106	0.116062057	-0.270983538
B_98618_GAACAGGCCTCAATGAACTATGCA	0.160734318	0.133303145	-0.434049083
B_98618_GAACAGGCGCTCGGTAGCCAAGAC	0.098366281	-0.055280738	-0.470802204
B_98618_GAACAGGCTGGTGGTAACCACTGT	-0.097710296	0.008240406	-0.435754543
B_98618_GAATCTGAAACCGAGATCTTCACA	0.053978909	0.083460931	-0.087407585
B_98618_GAATCTGAAAGAGATCGGAGAACA	0.128138707	0.141140294	-0.447285975
B_98618_GAATCTGACACTTCGACCGTGAGA	-0.081724385	0.034588017	-0.319304842
B_98618_GAATCTGACCATCCTCCTGAGCCA	0.379055817	-0.033551167	-0.488605369
B_98618_GAATCTGACGACACACCTCAATGA	0.093245202	0.192587929	-0.504503558
B_98618_GACAGTGCACAGCAGAGACAGTGC	-0.084211343	0.097582951	-0.176006699
B_98618_GACAGTGCAGCAGGAAACCACTGT	-0.026713869	-0.015689551	-0.431223011

B_98618_GACAGTGCCAGCGTTACTAAGGTC	-0.026307453	0.060481607	-0.258550937
B_98618_GACAGTGCCGAACTTACGACACAC	0.0542901	0.010313969	-0.488615739
B_98618_GACAGTGCGATAGACAAGTACAAG	0.024362481	0.01745832	-0.497980713
B_98618_GACAGTGCGCCAAGACATAGCGAC	0.244580881	0.060160946	-0.42595645
B_98618_GACAGTGCTATCAGCAACTATGCA	-0.041815934	-0.074380807	-0.432474124
B_98618_GACAGTGCTGGTGGTAAGCACCTC	0.59078842	-0.119371018	-0.465202422
B_98618_GACTAGTAAACGTGATCTCAATGA	0.136302605	0.254389414	-0.280006539
B_98618_GACTAGTAACACGACCACCTCCAA	0.083287222	0.194974344	-0.415170157
B_98618_GACTAGTAACTATGCACATACCAA	0.005060396	0.140165049	-0.451310404
B_98618_GACTAGTACACCTTACCACCTTAC	0.081871544	0.056724085	-0.08185672
B_98618_GACTAGTACTGGCATACACTTCGA	0.06670405	0.129917094	-0.445166302
B_98618_GACTAGTAGATAGACAAGTACAAG	0.529017458	0.052206274	-0.179445303
B_98618_GACTAGTAGTCTGTCAACACGACC	0.256531555	0.02225755	-0.419111838
B_98618_GACTAGTATGGAACAAACATTGGC	-0.081775671	-0.03085626	-0.414698053
B_98618_GAGCTGAAACGTATCAATTGGCTC	0.143503851	0.103955434	-0.366235453
B_98618_GAGCTGAAAGTCACTAGTCGTAGA	0.091411572	-0.11312512	-0.420407297
B_98618_GAGCTGAAATGCCTAAACAGCAGA	0.263505	-0.079972613	-0.451431713
B_98618_GAGCTGAACCGTGAGAGAGTTAGC	0.28771886	0.082566937	-0.210953421
B_98618_GAGCTGAACCTCTATCGGTGCGAA	-0.02252171	0.063328198	-0.260607772
B_98618_GAGCTGAACGCATACAAATGTTGC	0.045131053	0.011916212	-0.368961715
B_98618_GAGCTGAAGACTAGTAACTATGCA	-0.195366584	-0.168392694	-0.498303311
B_98618_GAGCTGAAGCCAAGACTATCAGCA	0.38253562	0.123648268	-0.278410328
B_98618_GAGCTGAAGGAGAACACCTAATCC	0.051566675	0.073918398	-0.463275755
B_98618_GAGCTGAATAGGATGAGACTAGTA	0.275368538	0.114214061	-0.19806223
B_98618_GAGCTGAATGGTGGTAATCATTCC	-0.054608773	-0.078725396	-0.257889047
B_98618_GAGTTAGCAATGTTGCCGAACTTA	0.088621402	0.078164925	-0.181326054
B_98618_GAGTTAGCCAAGACTAACATTGGC	0.068517368	-0.046658504	-0.35406581
B_98618_GAGTTAGCCCGAAGTAGGAGAACA	0.192147931	-0.005230479	-0.41558045
B_98618_GAGTTAGCCCTCTATCCAATGGAA	0.156482412	0.094810226	-0.246319149
B_98618_GAGTTAGCCGAACTTAAACGTGAT	0.503747845	0.057196919	-0.462634081
B_98618_GAGTTAGCTCTTCACAAGGCTAAC	0.160517788	0.007698784	-0.417270192
B_98618_GATAGACAAACGCTTAACGCTCGA	-0.041962293	0.251598395	-0.130732676

B_98618_GATAGACAAAGGACACACAGCAGA	0.306162707	0.165352255	-0.257661565
B_98618_GATAGACAAGCACCTCCAGCGTTA	0.315848128	0.155280517	-0.280291517
B_98618_GATAGACAAGCAGGAACCATCCTC	0.396759037	-0.007039405	-0.15876011
B_98618_GATAGACAGATGAATCAACCGAGA	-0.054814299	-0.043504529	-0.53789554
B_98618_GATGAATCAACGTGATTCTTCACA	0.320839162	0.009803572	-0.524975378
B_98618_GATGAATCATTGGCTCGATAGACA	0.249745273	0.136835391	-0.493287782
B_98618_GATGAATCCAATGGAATGAAGAGA	-0.055808629	0.048937251	-0.330481404
B_98618_GATGAATCCCTCCTGAAGAGTCAA	0.064582415	-0.03292915	-0.380036526
B_98618_GATGAATCCGCATACACCGTGAGA	0.394866035	0.119324532	-0.243796137
B_98618_GATGAATCGACTAGTAAGCCATGC	0.091745226	0.041604111	-0.362503912
B_98618_GATGAATCGATAGACACAAGACTA	-0.023777962	0.044039548	-0.480825178
B_98618_GCCACATAACACAGAACTGAGCCA	0.172897937	0.11976299	-0.181927291
B_98618_GCCACATAAGATGTACGAACAGGC	0.160336754	0.15543643	-0.416505412
B_98618_GCCACATAAGATGTACTTCACGCA	0.084412828	0.145407052	-0.299309952
B_98618_GCCACATACCAGTTCACGGATTGC	0.575218692	-0.046250896	-0.4888213
B_98618_GCGAGTAAAACGCTTATAGGATGA	-0.02400328	0.056746392	-0.486515396
B_98618_GCGAGTAAAACGTGATCGACTGGA	0.134256772	0.030534135	-0.382570756
B_98618_GCGAGTAAAACTCACCAAGAGATC	0.184099524	0.095116459	-0.368560324
B_98618_GCGAGTAAAGAGTCAAGCCACATA	0.145025403	0.005756634	-0.364049817
B_98618_GCGAGTAAAGTACAAGAAGGACAC	0.235647258	0.104752613	-0.283943592
B_98618_GCGAGTAAATTGGCTCAGAGTCAA	0.048887934	-0.025656259	-0.403943566
B_98618_GCGAGTAACAACCACAGCGAGTAA	0.23470387	0.039025712	-0.488170763
B_98618_GCGAGTAAGGAGAACAACAGCAGA	0.220841451	-0.01136978	-0.281652177
B_98618_GCTAACGAAAACATCGCACCTTAC	0.116719202	0.004418779	-0.429399454
B_98618_GCTAACGAAAGACGGAAAACATCG	0.295199343	0.277506506	0.005056556
B_98618_GCTAACGACCAGTTCAAACGCTTA	0.263008594	0.093223736	-0.348679588
B_98618_GCTAACGACGCTGATCGTACGCAA	0.099022447	0.028258294	-0.413912104
B_98618_GCTAACGACTGAGCCACGCATACA	0.224728533	-0.023726509	-0.418046914
B_98618_GCTAACGAGAGTTAGCCTCAATGA	0.321063743	0.044758431	-0.330956651
B_98618_GCTAACGAGCCACATAAGATCGCA	-0.026875766	0.138192496	-0.214099196
B_98618_GCTCGGTAAGTCACTAAATGTTGC	0.231661115	0.092055848	-0.346491625
B_98618_GCTCGGTAATCCTGTACTGTAGCC	0.17351817	0.143927551	-0.412462789

B_98618_GCTCGGTACAGATCTGCAATGGAA	0.139767389	0.069767012	-0.382036109
B_98618_GCTCGGTATATCAGCAGAACAGGC	-0.047715393	-0.010872809	-0.493195169
B_98618_GGAGAACAAATGTTGCATAGCGAC	0.319194075	0.102167792	-0.439867896
B_98618_GGAGAACAATCCTGTACTCAATGA	-0.059487642	0.074017432	-0.393961895
B_98618_GGAGAACACAAGGAGCGTCGTAGA	-0.028607537	0.143219167	-0.18102748
B_98618_GGAGAACACCAGTTCATATCAGCA	0.085759982	0.070747821	-0.403376989
B_98618_GGAGAACACCTAATCCAGCAGGAA	0.238690574	0.210934087	-0.435475946
B_98618_GGAGAACAGACAGTGCAAGACGGA	-0.097326748	0.028600012	0.152130302
B_98618_GGAGAACATGAAGAGAAGTACAAG	0.129490406	0.14661959	-0.191205284
B_98618_GGTGCGAAAAGGACACAGATCGCA	-0.005031906	-0.015669935	-0.512154635
B_98618_GGTGCGAAAAGGTACAATCCTGTA	-0.008832244	-0.038343623	-0.302932318
B_98618_GGTGCGAACCATCCTCCATCAAGT	0.127236261	0.071113206	-0.375362059
B_98618_GGTGCGAACGCATACAAACTCACC	0.024315072	0.022429339	-0.35478857
B_98618_GGTGCGAACTCAATGAACATTGGC	0.287391136	-0.080476796	-0.399442745
B_98618_GGTGCGAAGAATCTGATGGAACAA	0.20445196	0.202818458	-0.226171311
B_98618_GGTGCGAATCTTCACAGCTAACGA	0.10780903	0.156785177	-0.272797819
B_98618_GTACGCAAACGTATCAAGAGTCAA	0.107169218	0.096700696	-0.278232068
B_98618_GTACGCAAAGGCTAACCACCTTAC	0.141531098	0.190006267	-0.278472182
B_98618_GTACGCAAATAGCGACAAGGTACA	0.031169324	0.025307554	-0.332843548
B_98618_GTACGCAACGGATTGCAAGAGATC	0.275045963	0.044002822	-0.427119307
B_98618_GTCGTAGAAGTCACTAAAGACGGA	0.2733457	0.133234348	-0.258446856
B_98618_GTCGTAGACAAGGAGCACTATGCA	0.100034134	-0.013551417	-0.475414457
B_98618_GTCGTAGAGCGAGTAAACGCTCGA	0.351515275	0.052522791	-0.452714443
B_98618_GTCGTAGATATCAGCAGCGAGTAA	0.100063705	0.105727997	-0.025401043
B_98618_GTCGTAGATGGTGGTAGATAGACA	0.095787303	0.064680799	-0.382746158
B_98618_GTCTGTCAAAGGACACACCTCCAA	0.239551534	-0.187644646	-0.500528826
B_98618_GTCTGTCAAAGGTACAAACGCTTA	0.524145544	0.19203632	-0.282925652
B_98618_GTCTGTCAATAGCGACATCATTCC	0.704859938	0.141608523	-0.212884244
B_98618_GTCTGTCACCTCTATCACAGCAGA	0.111376542	0.072567995	-0.253049223
B_98618_GTCTGTCAGAATCTGAACGCTCGA	0.277337395	0.169134669	-0.265849966
B_98618_GTGTTCTAAACGTGATAACCGAGA	0.468436502	0.388013425	0.013870321
B_98618_GTGTTCTACAACCACACCATCCTC	0.006896522	0.147148058	-0.500937219

B_98618_GTGTTCTACACCTTACAACAACCA	0.029021092	0.014826789	-0.445971664
B_98618_GTGTTCTACGGATTGCCCAGTTCA	0.106646308	0.228351273	0.052362439
B_98618_TAGGATGAAATCCGTCATTGAGGA	0.365215155	0.066850895	-0.376290262
B_98618_TAGGATGAAGATGTACGCTAACGA	0.194995634	0.159050534	-0.328957323
B_98618_TAGGATGACGCATACATGAAGAGA	0.12174664	0.213237853	-0.456296497
B_98618_TATCAGCACTGTAGCCACAGATTC	0.214667871	0.071139155	-0.33974511
B_98618_TCCGTCTAAGATGTACATGCCTAA	0.168760295	0.078154104	0.176482067
B_98618_TCCGTCTAAGCACCTCACGCTCGA	0.129806809	0.024546988	-0.389480799
B_98618_TCCGTCTAATGCCTAACTGTAGCC	0.061057903	0.016558192	-0.412460617
B_98618_TCCGTCTACAATGGAACGACTGGA	0.331307639	0.149933219	-0.239950902
B_98618_TCCGTCTACTGTAGCCCGCTGATC	0.237498246	0.057310405	-0.176510228
B_98618_TCCGTCTAGTCTGTCAACATTGGC	0.119119378	0.124528259	-0.42630566
B_98618_TCCGTCTATTCACGCAAAGGACAC	0.736939065	0.199200259	-0.062896664
B_98618_TCTTCACAAGCCATGCGATGAATC	0.120377362	0.036561531	-0.351908528
B_98618_TCTTCACACACCTTACTCCGTCTA	0.458650406	0.147104412	-0.007573648
B_98618_TCTTCACACAGCGTTAAGCACCTC	0.601887295	0.102870924	-0.44273418
B_98618_TGAAGAGAAAGAGATCATGCCTAA	0.176749995	0.045863628	-0.357164364
B_98618_TGAAGAGACCAGTTCACCGTGAGA	-0.005754775	0.120009582	-0.352931717
B_98618_TGAAGAGACCATCCTCCCGAAGTA	0.467700344	-0.034379847	-0.479167514
B_98618_TGGAACAACGGATTGCGTCGTAGA	0.004245314	0.005941198	-0.450664865
B_98618_TGGAACAAGATGAATCACGCTCGA	0.514008787	0.094164116	-0.376593275
B_98618_TGGCTTCAACGCTCGACCTCTATC	0.421507319	0.023971257	-0.411928
B_98618_TGGCTTCACCGAAGTACCGTGAGA	0.339692557	0.09390302	-0.437329534
B_98618_TGGCTTCACCTCTATCGAGCTGAA	0.214115498	0.009843196	-0.325485315
B_98618_TGGCTTCAGTCTGTCAAGTGGTCA	-0.053418713	-0.0795412	-0.271902648
B_98618_TGGTGGTAAACCGAGAAACCGAGA	0.048970976	0.073933523	-0.366770306
B_98618_TGGTGGTAAACTCACCCTCAATGA	0.009289485	0.051894012	-0.2252703
B_98618_TGGTGGTAACGTATCAACTATGCA	0.346115088	0.136675769	-0.298180565
B_98618_TGGTGGTAATGCCTAAAGAGTCAA	-0.017659952	0.053890238	-0.519177098
B_98618_TGGTGGTACAGCGTTAACACGACC	0.171803836	0.099757733	-0.28304864
B_98618_TGGTGGTACGACACACCAAGGAGC	0.050426527	0.060199593	-0.42140688
B_98618_TGGTGGTACGACTGGAGCCACATA	0.442910159	0.057990735	-0.341922062

B_98618_TGGTGGTAGAATCTGAGCCACATA	0.007876339	0.151662604	-0.154071197
B_98618_TTCACGCAAGCAGGAAAGATGTAC	-0.094617376	0.02224385	-0.392200603
B_98618_TTCACGCAAGTGGTCACGCTGATC	-0.015576976	0.081818832	-0.487037486
B_98618_TTCACGCAATAGCGACACCACTGT	0.016845575	-0.012397531	-0.447898845
B_98618_TTCACGCACAATGGAAGTCTGTCA	0.414921179	0.158612985	-0.085909468
B_98618_TTCACGCACCGTGAGACATACCAA	-0.018874955	0.12375465	-0.382760578
B_98618_TTCACGCACGGATTGCCAAGGAGC	0.314276526	0.249417711	-0.293055227
B_98618_TTCACGCAGCGAGTAAGGTGCGAA	0.039053237	0.054205075	-0.412748722
B_98618_TTCACGCATCTTCACAAAGGTACA	0.165788628	-0.060689255	-0.404900243
B_98618_ATAGCGACCACTTCGACGCATACA	0.574543539	0.704584839	-0.132294482
B_98618_ATTGAGGAAGATGTACAGAGTCAA	0.514048028	0.285532332	-0.230882202
B_98618_CGGATTGCAGCACCTCGTACGCAA	0.309549174	0.186792237	-0.432442504
B_98618_CTGGCATAGCCACATACCATCCTC	0.084162228	0.247017729	-0.389326425
B_98618_TGGAACAAATTGGCTCGAGTTAGC	0.589417727	0.200755673	-0.063302522
B_98618_AAACATCGTGGCTTCAAATCCGTC	0.251222772	0.03069304	-0.075878462
B_98618_AACGCTTACATACCAAACAGATTC	0.031112744	0.026838663	0.003136151
B_98618_AACGTGATACCTCCAACCAGTTCA	0.33562111	0.10017406	-0.356357456
B_98618_AACGTGATGTGTTCTACTGGCATA	0.173223307	-0.04335006	-0.296466388
B_98618_AACTCACCCTGAGCCAGATGAATC	0.132235927	-0.034572083	-0.47567168
B_98618_AAGGACACCTGAGCCAAGTGGTCA	0.217843301	0.048193587	-0.352121444
B_98618_AAGGACACGATAGACAGTCGTAGA	0.141571478	0.077160226	-0.279255824
B_98618_AAGGTACAACAAGCTAGTGTTCTA	-0.080394629	0.08818517	0.107630273
B_98618_AAGGTACACACCTTACTCCGTCTA	0.13468602	0.091778968	-0.359518124
B_98618_AATGTTGCTATCAGCACATACCAA	0.341502717	0.097409101	-0.181750675
B_98618_ACCACTGTGGTGCGAAAAGGACAC	0.107318717	0.103883977	-0.156704056
B_98618_ACGTATCAACACAGAACACTTCGA	0.009204421	0.044322092	-0.522264727
B_98618_AGAGTCAAACAAGCTAGTCTGTCA	0.207216977	-0.015673225	-0.337603572
B_98618_AGAGTCAAGAGTTAGCTATCAGCA	-0.038503658	0.124813345	-0.227784858
B_98618_AGATCGCAACGTATCAAAGGTACA	0.052979093	-0.031333215	-0.261119293
B_98618_AGATCGCACATACCAAGCCAAGAC	0.113474309	-0.081917022	-0.381853786
B_98618_AGATGTACAATCCGTCTTCACGCA	0.133933551	0.088555826	-0.264283901
B_98618_AGCACCTCAGTGGTCAACCTCCAA	-0.14453641	-0.004974538	-0.467570986

B_98618_AGCACCTCGACTAGTAACAGCAGA	0.131324197	0.138758419	-0.225512536
B_98618_CAATGGAAAGCCATGCCAAGGAGC	0.376883377	-0.055620701	-0.345606229
B_98618_CACTTCGAAGTCACTACAAGACTA	0.073880569	0.053753054	-0.384453968
B_98618_CCGAAGTAAGCACCTCAGTACAAG	0.045963905	0.046194095	-0.416880419
B_98618_CCTCCTGAAAGGTACAAACGCTTA	0.188219041	-0.095581281	-0.525352832
B_98618_CCTCCTGACAACCACAGCGAGTAA	-0.188385959	-0.109336514	-0.508335759
B_98618_CCTCCTGACGCATACACCATCCTC	0.245562054	0.190650501	-0.295136064
B_98618_CCTCTATCTGGAACAATGGCTTCA	0.307856245	0.077759109	-0.460361327
B_98618_CGAACTTAACAAGCTAACAAGCTA	-0.025600399	0.072390172	-0.079038536
B_98618_CGACTGGAACAGCAGAATGCCTAA	0.135003062	0.134148133	-0.43030978
B_98618_CGACTGGAATCCTGTAATTGGCTC	0.060781193	-0.049221064	-0.527887422
B_98618_CTAAGGTCAAACATCGATCCTGTA	0.023599079	-0.057648949	-0.183603248
B_98618_CTGAGCCAATTGAGGAGATGAATC	0.082095501	0.066280208	-0.379822323
B_98618_CTGGCATAATTGGCTCGCTCGGTA	0.065562407	0.097496592	-0.214629465
B_98618_CTGGCATAGGAGAACATATCAGCA	0.269315119	0.036240389	-0.456018957
B_98618_CTGTAGCCCAACCACACCATCCTC	0.502971338	0.066602621	-0.387278277
B_98618_GAACAGGCTGAAGAGATTCACGCA	-0.002373842	-0.054128703	-0.36885385
B_98618_GAGCTGAAAGCAGGAAAACGTGAT	0.199535575	-0.151905766	-0.498246399
B_98618_GCCAAGACCAGCGTTAGCTAACGA	0.254225464	0.090131237	-0.246544664
B_98618_GCTAACGAGCTAACGAAACCGAGA	0.055781005	-0.060707537	-0.434340002
B_98618_GGTGCGAAGGAGAACAGCTAACGA	0.216605844	0.121410379	-0.308945322
B_98618_GTACGCAAGATGAATCCAACCACA	0.289757982	0.083142637	-0.265744434
B_98618_GTCGTAGAAGTGGTCATGAAGAGA	0.042275536	-0.041498658	-0.196733937
B_98618_GTGTTCTAAGCAGGAACTCAATGA	0.019947426	-0.084164275	-0.444795661
B_98618_TAGGATGACACTTCGATAGGATGA	0.279601549	0.10049841	-0.207585442
B_98618_TCTTCACATGGCTTCAGCCACATA	0.158279082	0.005944229	-0.140214972
B_98618_TGGTGGTACGACTGGAGAGCTGAA	0.193855014	0.1920594	-0.320246332
B_98618_TTCACGCAAGCAGGAACTGAGCCA	0.000163131	0.079241136	-0.43153544
B_98618_TTCACGCACCGTGAGACCGACAAC	0.072335463	-0.071128258	-0.143879414
B_98618_AACTCACCGGAGAACACGCATACA	0.074247762	0.110551337	-0.424151642
B_98618_ACAGCAGAGAACAGGCAGGCTAAC	0.273835594	0.099940993	-0.418157258
B_98618_ACCTCCAAGACTAGTACAAGGAGC	0.150042525	0.055908343	-0.317961025

B_98618_ACGCTCGACACTTCGAAATGTTGC	0.291831462	-0.047284632	-0.46538279
B_98618_ACTATGCATGGTGGTAATTGAGGA	0.184305004	0.014369844	-0.443954383
B_98618_AGCCATGCCGGATTGCGGAGAACA	0.049871519	0.002008415	-0.326937127
B_98618_AGTGGTCACACCTTACGTGTTCTA	0.146944525	0.152360159	-0.473958057
B_98618_CCTAATCCACAAGCTAGAGTTAGC	0.110667133	-0.04586542	-0.383091431
B_98618_CTGGCATAATAGCGACGCTCGGTA	0.319747068	0.279766218	-0.52327693
B_98618_GAGCTGAACCTAATCCATGCCTAA	0.245000955	0.190391901	-0.213559188
B_98618_AACAACCAAGTGGTCAACGCTCGA	0.157788135	0.221477054	-0.341462459
B_98618_AACTCACCACACGACCTCCGTCTA	0.228910531	0.222795527	-0.512244275
B_98618_AAGACGGAGAGTTAGCCAATGGAA	0.212994547	0.226995641	-0.466441322
B_98618_AATCCGTCGCCAAGACCTCAATGA	0.158700476	0.056801534	-0.38760238
B_98618_AATGTTGCCACTTCGAAATGTTGC	0.533970565	0.108530067	-0.42658411
B_98618_AATGTTGCTCTTCACAAGCACCTC	0.134676573	0.175149321	-0.41924162
B_98618_ACATTGGCAATCCGTCCCGACAAC	0.231885333	0.020402644	-0.464009476
B_98618_ACCTCCAAATTGAGGAGCTAACGA	0.422516794	0.074995861	-0.337378136
B_98618_ACGCTCGAATTGAGGAGATAGACA	0.146192666	-0.00743993	-0.493010622
B_98618_ACTATGCATGGAACAAATGCCTAA	0.549833146	0.144402158	-0.500185889
B_98618_AGATCGCAGAATCTGAGAACAGGC	0.706161744	0.251619482	-0.243474612
B_98618_AGATCGCAGATAGACAATGCCTAA	0.222827856	0.045992031	-0.424302262
B_98618_ATCCTGTAACACGACCGCCACATA	0.359373753	0.353204337	-0.230391269
B_98618_CACCTTACAAGGACACCAAGACTA	0.195892977	0.114478551	-0.367989522
B_98618_CACCTTACCGGATTGCAGCCATGC	-0.006139403	0.123103918	-0.373951951
B_98618_CATCAAGTCGAACTTACCTCTATC	-0.000161619	0.068927735	-0.418067685
B_98618_CCGAAGTAAGGCTAACCCATCCTC	0.591194725	0.223040301	-0.318110968
B_98618_CGACACACCACCTTACGCTAACGA	-0.023653346	-0.032722087	-0.384442994
B_98618_CGACACACCAGCGTTAATCCTGTA	0.096665687	-0.034231348	-0.413401381
B_98618_GACAGTGCATTGGCTCATCCTGTA	0.807383062	0.156936825	-0.282690458
B_98618_GATAGACAAGTCACTATGAAGAGA	0.237559399	0.242875157	-0.339790329
B_98618_GCCAAGACCACCTTACGACTAGTA	0.23533439	0.031882564	-0.506989023
B_98618_GTGTTCTAGACTAGTACCGTGAGA	-0.083261821	0.130026182	-0.360704002
B_98618_GTGTTCTAGCCAAGACCAAGACTA	0.558299691	0.312331819	-0.382975972
B_98618_TCCGTCTACCAGTTCAATGCCTAA	0.586686833	0.114302062	-0.419648767

B_98618_TCCGTCTATAGGATGAGAGTTAGC	0.457267431	0.097405066	-0.433586677
B_98618_AGTGGTCACGCTGATCCACTTCGA	0.42957158	0.242976783	-0.14400535
B_98618_CAGATCTGAGAGTCAACCGAAGTA	0.397256791	0.173442146	-0.305728982
B_98618_CAGATCTGGCTAACGACAACCACA	0.193006748	-0.143337279	-0.462285079
B_98618_GCCAAGACCCGACAACCTGAGCCA	0.453794651	0.053016025	-0.23782239
B_98618_GCGAGTAAGCCACATATCCGTCTA	0.316916932	0.197459518	-0.338226607
ab_99547_AAACATCGCAAGACTAAATCCGTC	0.139312342	0.119238555	-0.224736596
ab_99547_AAACATCGCCGTGAGACTAAGGTC	0.324233757	0.000497463	-0.400276504
ab_99547_AAACATCGGAACAGGCAGTACAAG	0.446944226	0.263728783	-0.205036232
ab_99547_AAACATCGGAATCTGAGATGAATC	0.393058715	0.03649761	-0.321130868
ab_99547_AACAACCAAACAACCAAGCAGGAA	0.489996683	0.125899306	-0.2484959
ab_99547_AACAACCAACACGACCCAAGACTA	0.362087161	0.14323085	-0.237239646
ab_99547_AACAACCAAGGCTAACCTGTAGCC	0.444489523	-0.016674797	-0.345763037
ab_99547_AACAACCAAGTCACTACAGCGTTA	0.287486142	0.120697641	-0.385390563
ab_99547_AACAACCAAGTGGTCACCGTGAGA	0.570946788	0.142617205	-0.124249382
ab_99547_AACAACCAATGCCTAAGCGAGTAA	0.400666006	0.106262247	-0.279479738
ab_99547_AACAACCACGACACACGAGCTGAA	0.478434702	0.132676034	-0.296155979
ab_99547_AACAACCACTGAGCCAAGCCATGC	0.457994857	0.219071228	-0.282303318
ab_99547_AACAACCACTGTAGCCCGCTGATC	0.4659829	0.147200204	-0.226648294
ab_99547_AACAACCAGAACAGGCAGGCTAAC	0.514728694	0.232155232	-0.232102627
ab_99547_AACAACCAGATAGACAGATGAATC	0.603652569	0.187254102	-0.253531899
ab_99547_AACAACCAGTCTGTCAAGGCTAAC	0.321783973	0.306411137	-0.19452205
ab_99547_AACAACCAGTCTGTCAGCCACATA	0.356155043	0.222595151	-0.224956847
ab_99547_AACCGAGAAATCCGTCCCATCCTC	0.583548669	0.11325112	-0.361763359
ab_99547_AACCGAGAAGTGGTCACGCTGATC	0.347102828	0.228598078	-0.401690972
ab_99547_AACCGAGACAACCACATGAAGAGA	0.509766303	0.14868632	-0.270339557
ab_99547_AACCGAGACAAGGAGCAAGACGGA	0.527645678	0.109298235	-0.267167049
ab_99547_AACCGAGACGAACTTACGACACAC	0.545909281	0.155101705	-0.257098332
ab_99547_AACCGAGACGACACACAGGCTAAC	0.410662416	0.205309146	-0.277223382
ab_99547_AACCGAGACGACACACGAATCTGA	0.311399779	0.061263627	-0.349090274
ab_99547_AACCGAGAGCCACATACAGATCTG	0.37320754	0.18975542	-0.334410826
ab_99547_AACCGAGAGGTGCGAACCGTGAGA	0.455801142	0.091430839	-0.222915295

ab_99547_AACCGAGATCTTCACAACACAGAA	0.327767749	0.145359415	-0.330077461
ab_99547_AACCGAGATGGTGGTAAAGGACAC	0.280810403	-0.079881498	-0.393301482
ab_99547_AACGCTTAAACTCACCTATCAGCA	0.419585598	0.125211427	-0.415859287
ab_99547_AACGCTTAACAGCAGACACTTCGA	0.554485439	0.17417383	-0.319888384
ab_99547_AACGCTTAACATTGGCGCCAAGAC	0.420443326	0.14828162	-0.185659796
ab_99547_AACGCTTAAGAGTCAAAAGACGGA	0.475558169	0.228259773	-0.367735417
ab_99547_AACGCTTAAGATGTACCCGAAGTA	0.373587116	0.036826426	-0.206628942
ab_99547_AACGCTTAAGTACAAGACAAGCTA	0.473503252	0.030354853	-0.27068663
ab_99547_AACGCTTAAGTGGTCACCGTGAGA	0.652646685	0.007929981	-0.40092216
ab_99547_AACGCTTAATCCTGTAATAGCGAC	0.623314625	0.015915101	-0.370015076
ab_99547_AACGCTTAATTGGCTCCAGCGTTA	0.394670658	0.042740654	-0.361200143
ab_99547_AACGCTTACAGCGTTATGGAACAA	0.400884763	0.206516017	-0.171731515
ab_99547_AACGCTTACATCAAGTACGTATCA	0.405760455	0.051704344	-0.288901921
ab_99547_AACGCTTACCATCCTCCATACCAA	0.365518658	0.081645648	-0.285621608
ab_99547_AACGCTTACGAACTTACCATCCTC	0.610808177	0.034277315	-0.192625337
ab_99547_AACGCTTACTGAGCCAACAAGCTA	0.374654502	0.12014096	-0.234892637
ab_99547_AACGCTTACTGGCATATATCAGCA	0.412889156	0.164207701	-0.189221408
ab_99547_AACGCTTAGATAGACACAAGACTA	0.514660233	-0.033886881	-0.310408211
ab_99547_AACGCTTAGCTAACGACTGAGCCA	0.396762505	0.151707542	-0.304336132
ab_99547_AACGCTTATAGGATGACATACCAA	0.548371113	0.122068861	-0.355211232
ab_99547_AACGTGATAACGCTTACCGTGAGA	0.327976132	0.091379204	-0.38124296
ab_99547_AACGTGATAAGACGGACGCTGATC	0.388557692	0.045452338	-0.200897209
ab_99547_AACGTGATACAGCAGAGTCTGTCA	0.626430949	0.133730269	-0.171638109
ab_99547_AACGTGATACCTCCAACAATGGAA	0.469902533	0.094201034	-0.215261993
ab_99547_AACGTGATAGATCGCAGTGTTCTA	0.416101174	0.01404842	-0.327950589
ab_99547_AACGTGATAGGCTAACTATCAGCA	0.390655657	0.071542387	-0.428026535
ab_99547_AACGTGATCAAGACTAACCACTGT	0.700522353	0.219287415	-0.334039349
ab_99547_AACGTGATCATACCAAACGCTCGA	0.567015591	-0.089794095	-0.329881994
ab_99547_AACGTGATCCAGTTCAATCCTGTA	0.707165932	0.157781508	-0.296906869
ab_99547_AACGTGATCCATCCTCAAGAGATC	0.341368194	0.206421703	-0.269831738
ab_99547_AACGTGATCGCATACACGACTGGA	0.628785163	0.044082	-0.122769321
ab_99547_AACGTGATGAATCTGAAACAACCA	0.762667283	0.093303927	-0.331900779

ab_99547_AACGTGATGACAGTGCCCTCTATC	0.474522161	0.038265744	-0.286816102
ab_99547_AACGTGATGCCACATAACGCTCGA	0.723204418	0.154714944	-0.215291601
ab_99547_AACGTGATGTCTGTCACCGAAGTA	0.669592145	0.063610574	-0.280605978
ab_99547_AACGTGATTCTTCACATGGTGGTA	0.596160045	0.198875645	-0.198938902
ab_99547_AACGTGATTGGAACAAGCCAAGAC	0.315113984	0.207288642	-0.139889126
ab_99547_AACGTGATTGGAACAAGGAGAACA	0.376217535	0.150602023	-0.311991166
ab_99547_AACTCACCAACTCACCCCGTGAGA	0.32513405	0.010390459	-0.405356454
ab_99547_AACTCACCACACAGAAATCCTGTA	0.674232931	0.044255442	-0.175603862
ab_99547_AACTCACCACGTATCAAACGCTTA	0.314168878	0.158261989	-0.325009797
ab_99547_AACTCACCAGCACCTCATCCTGTA	0.469672001	0.108918716	-0.234974747
ab_99547_AACTCACCATAGCGACAGAGTCAA	0.44208771	0.195521037	-0.220193429
ab_99547_AACTCACCCAGCGTTACTGAGCCA	0.631435798	0.117300528	-0.314248301
ab_99547_AACTCACCCATCAAGTGGAGAACA	0.398808268	0.200135746	-0.314999964
ab_99547_AACTCACCCGCATACACAGATCTG	0.577110305	0.152104435	-0.194893854
ab_99547_AACTCACCCTGTAGCCCAAGGAGC	0.426386218	0.179170417	-0.361200362
ab_99547_AACTCACCGAACAGGCACAGCAGA	0.439556187	0.051019355	-0.275449041
ab_99547_AACTCACCGACAGTGCCCTAATCC	0.765441559	0.253590033	-0.293326148
ab_99547_AACTCACCGACAGTGCCTAAGGTC	0.188938839	0.010879398	-0.402080136
ab_99547_AACTCACCGGTGCGAACTGTAGCC	0.590580252	0.11231608	-0.327560318
ab_99547_AACTCACCGGTGCGAATGGCTTCA	0.353112987	0.040961528	-0.275961551
ab_99547_AACTCACCTGGCTTCATTCACGCA	0.61576952	0.106598888	-0.339064908
ab_99547_AACTCACCTGGTGGTACGCATACA	0.459896911	0.130417761	-0.277394415
ab_99547_AAGACGGAAACCGAGATGGCTTCA	0.483830385	-0.001676926	-0.337994407
ab_99547_AAGACGGAAACGCTTAGCTCGGTA	0.472070278	0.113837508	-0.223651844
ab_99547_AAGACGGAAAGGACACTTCACGCA	0.540275883	0.103711215	-0.236892865
ab_99547_AAGACGGAACCTCCAACACTTCGA	0.717264803	0.410274437	-0.236163651
ab_99547_AAGACGGAACGTATCACATACCAA	0.49814544	0.020999314	-0.311118806
ab_99547_AAGACGGAATAGCGACCCGAAGTA	0.617672377	0.119881935	-0.24395881
ab_99547_AAGACGGACCAGTTCACGCTGATC	0.515260499	-0.005934141	-0.290086234
ab_99547_AAGACGGAGACAGTGCAAGGACAC	0.774913136	0.11425267	-0.351868105
ab_99547_AAGACGGAGAGTTAGCACTATGCA	0.670794975	0.281926432	-0.214420737
ab_99547_AAGACGGAGATGAATCACATTGGC	0.394386474	0.017311806	-0.397479636

ab_99547_AAGACGGAGTCTGTCACAATGGAA	0.51107558	0.168482808	-0.379676034
ab_99547_AAGACGGAGTCTGTCACACCTTAC	0.465931501	0.141748387	-0.173374773
ab_99547_AAGACGGATATCAGCAAGCAGGAA	0.542036013	0.090937532	-0.380458561
ab_99547_AAGACGGATCCGTCTACCAGTTCA	0.387355537	0.050195712	-0.103195402
ab_99547_AAGAGATCAAGGTACAATAGCGAC	0.602744457	0.13316291	-0.25571153
ab_99547_AAGAGATCACTATGCACAGCGTTA	0.66429539	0.046085215	-0.208412899
ab_99547_AAGAGATCAGCAGGAAAGTGGTCA	0.425227058	0.04292103	-0.205886703
ab_99547_AAGAGATCATTGAGGACACTTCGA	0.435141253	0.118709205	-0.054807394
ab_99547_AAGAGATCCAAGACTAAACGTGAT	0.502547933	0.131634871	-0.249474641
ab_99547_AAGAGATCCGAACTTATTCACGCA	0.515788441	0.156218887	-0.233116951
ab_99547_AAGAGATCCGGATTGCTGGTGGTA	0.544339244	0.186622101	-0.218237165
ab_99547_AAGAGATCGAATCTGAGGAGAACA	0.304861599	0.064724751	-0.300160063
ab_99547_AAGGACACAATGTTGCCCTAATCC	0.345347086	-0.012244965	-0.351133825
ab_99547_AAGGACACACACGACCCTGTAGCC	0.802264798	0.292842732	-0.310867431
ab_99547_AAGGACACACATTGGCTATCAGCA	0.51415356	0.161781002	-0.233196663
ab_99547_AAGGACACAGCAGGAAACAAGCTA	0.507554373	0.040413368	-0.283844658
ab_99547_AAGGACACAGCAGGAAGTCTGTCA	0.518728752	0.083295328	-0.365127706
ab_99547_AAGGACACAGCAGGAATGAAGAGA	0.200828833	0.164404454	-0.275539673
ab_99547_AAGGACACATCCTGTACCTAATCC	0.472649551	0.039211708	-0.283204153
ab_99547_AAGGACACCACTTCGACAGATCTG	0.343891287	0.093694204	-0.313807761
ab_99547_AAGGACACCACTTCGATGGCTTCA	0.689336818	0.149377362	-0.059938781
ab_99547_AAGGACACCAGCGTTAACAGATTC	0.475583366	0.216861808	-0.284529599
ab_99547_AAGGACACCATCAAGTAGTCACTA	0.589199911	0.254228765	-0.228653991
ab_99547_AAGGACACCCTCTATCCGCTGATC	0.633514828	0.094691749	-0.330285969
ab_99547_AAGGACACCTCAATGAATGCCTAA	0.49260046	0.070823721	-0.208926087
ab_99547_AAGGACACGCTAACGACAGATCTG	0.471195618	0.041960781	-0.340704628
ab_99547_AAGGACACGCTAACGATGAAGAGA	0.478814379	0.168774787	-0.259592071
ab_99547_AAGGACACTCTTCACAAGTACAAG	0.487920544	0.148169406	-0.287960981
ab_99547_AAGGACACTGGAACAAGAGTTAGC	0.434192056	-0.019710057	-0.385451477
ab_99547_AAGGTACAAATCCGTCAGTCACTA	0.462106331	0.138231758	-0.208730879
ab_99547_AAGGTACAAATGTTGCAGCACCTC	0.257397493	0.164183476	-0.231407611
ab_99547_AAGGTACAAGCACCTCACCACTGT	0.545133319	-0.02049776	-0.301239469

ab_99547_AAGGTACAAGTGGTCACGCATACA	0.676780863	0.166103387	-0.350661516
ab_99547_AAGGTACAATCCTGTAACAGCAGA	0.452651168	0.272759886	-0.154844045
ab_99547_AAGGTACACAGCGTTACGACTGGA	0.392980668	0.064922063	-0.139377627
ab_99547_AAGGTACACCTCTATCCGCATACA	0.345599076	0.22484423	-0.172049484
ab_99547_AAGGTACACGCTGATCATTGAGGA	0.598967118	0.194952003	-0.260452459
ab_99547_AAGGTACAGACTAGTAAAGAGATC	0.389151286	0.087288475	-0.321602429
ab_99547_AATCCGTCCACCTTACAGGCTAAC	0.360939165	0.089502703	-0.29495304
ab_99547_AATCCGTCCCTCTATCAACTCACC	0.501312465	0.228643147	-0.199380007
ab_99547_AATCCGTCCGAACTTACAATGGAA	0.54780344	0.174249634	-0.117090301
ab_99547_AATCCGTCGTCTGTCATGGAACAA	0.571294769	0.162448902	-0.177197981
ab_99547_AATGTTGCAACCGAGAAGTCACTA	0.491043275	0.036744697	-0.14484284
ab_99547_AATGTTGCAGCCATGCGACTAGTA	0.40799657	0.172505077	-0.410764448
ab_99547_AATGTTGCATCATTCCATCCTGTA	0.356691008	0.133520802	-0.36823186
ab_99547_AATGTTGCCAATGGAAAGCACCTC	0.408574024	0.17572731	-0.11866271
ab_99547_AATGTTGCCATACCAAGAACAGGC	0.700681498	0.13012437	-0.234450688
ab_99547_AATGTTGCCGACACACGACAGTGC	0.43841107	0.042138278	-0.258478316
ab_99547_AATGTTGCGCGAGTAAGATGAATC	0.403203799	0.018816499	-0.242100252
ab_99547_AATGTTGCGTACGCAAACCTCCAA	0.271404472	0.111974376	-0.180339487
ab_99547_AATGTTGCGTCTGTCAATCCTGTA	0.454489851	0.094406155	-0.138489069
ab_99547_AATGTTGCTAGGATGAGCTCGGTA	0.396839475	0.177486702	-0.25481089
ab_99547_AATGTTGCTAGGATGATATCAGCA	0.278088798	0.076308563	-0.315789667
ab_99547_AATGTTGCTATCAGCACCATCCTC	0.556303792	0.074619364	-0.239310488
ab_99547_AATGTTGCTTCACGCATGAAGAGA	0.554583758	0.274715741	-0.322806402
ab_99547_ACAAGCTAACACAGAAGGAGAACA	0.390848153	0.066384022	-0.39269727
ab_99547_ACAAGCTAAGTACAAGGCCAAGAC	0.623599681	-0.007217977	-0.462753837
ab_99547_ACAAGCTACACCTTACAAGGTACA	0.24067982	0.082452849	-0.335925402
ab_99547_ACAAGCTACACCTTACATTGAGGA	0.45791418	0.199906943	-0.201591377
ab_99547_ACAAGCTACACTTCGAAAGACGGA	0.61805868	0.106261447	-0.209114797
ab_99547_ACAAGCTACAGCGTTACAATGGAA	0.53947135	0.072783828	-0.21763976
ab_99547_ACAAGCTACCGACAACATCATTCC	0.368906846	0.15907082	-0.249017107
ab_99547_ACAAGCTAGATGAATCCTCAATGA	0.412034947	0.149619106	-0.347872653
ab_99547_ACAAGCTAGGAGAACAGCCAAGAC	0.443316734	0.055734486	-0.215799101

ab_99547_ACAAGCTATGAAGAGAACCACTGT	0.152250359	0.204231012	-0.156282974
ab_99547_ACAAGCTATGAAGAGACCAGTTCA	0.596761868	0.281042544	-0.337960996
ab_99547_ACACAGAAAACGCTTACGACTGGA	0.449032916	0.077011742	-0.300545331
ab_99547_ACACAGAAAACTCACCGAACAGGC	0.610380182	0.064794849	-0.209644586
ab_99547_ACACAGAAACCTCCAAGATAGACA	0.367798363	0.093091865	-0.29606513
ab_99547_ACACAGAAAGGCTAACCGAACTTA	0.574353766	0.104325659	-0.312115581
ab_99547_ACACAGAAATAGCGACACTATGCA	0.369834978	0.194560499	-0.400495361
ab_99547_ACACAGAAATTGAGGAAAGGACAC	0.525641329	0.345179744	-0.250803331
ab_99547_ACACAGAACCGTGAGACTAAGGTC	0.431478698	0.101258421	-0.369942194
ab_99547_ACACAGAACGCATACAATCATTCC	0.399773241	0.180988658	-0.258095487
ab_99547_ACACAGAAGACAGTGCCTGAGCCA	0.674847469	0.105175717	-0.19661362
ab_99547_ACACAGAAGACAGTGCGCTCGGTA	0.51948955	0.064249686	-0.420356621
ab_99547_ACACAGAATGGAACAAGAGTTAGC	0.513571892	0.091444979	-0.316214347
ab_99547_ACACGACCAACCGAGACTCAATGA	0.468871461	0.184851378	-0.275130106
ab_99547_ACACGACCAACTCACCCCGTGAGA	0.407437368	0.101058265	-0.183353858
ab_99547_ACACGACCAAGAGATCAAGGTACA	0.405301641	0.113419793	-0.206744689
ab_99547_ACACGACCAATCCGTCCAAGGAGC	0.285699976	0.012189324	-0.21402632
ab_99547_ACACGACCAGTCACTAGACAGTGC	0.474170484	0.170589254	-0.332756636
ab_99547_ACACGACCCACCTTACCACTTCGA	0.651486566	0.163006561	-0.426853896
ab_99547_ACACGACCCCATCCTCGTCTGTCA	0.4086347	0.084333491	-0.305933409
ab_99547_ACACGACCCGCATACAGGTGCGAA	0.291279814	-0.042406661	-0.378499063
ab_99547_ACACGACCGATGAATCACACGACC	0.473017969	0.274425245	-0.344673683
ab_99547_ACACGACCGCTCGGTACAGATCTG	0.603569713	0.23331894	-0.402843511
ab_99547_ACAGATTCAAGACGGAAACAACCA	0.395717197	0.081808795	-0.268366145
ab_99547_ACAGATTCAAGACGGAGCCAAGAC	0.374940891	0.117512952	-0.271414709
ab_99547_ACAGATTCAGTCACTAACACGACC	0.504470091	0.110673566	-0.351397188
ab_99547_ACAGATTCCAGCGTTACCAGTTCA	0.498580019	0.112699508	-0.276232156
ab_99547_ACAGATTCCCTAATCCCGACACAC	0.520698939	0.102626831	-0.413507437
ab_99547_ACAGATTCCGACTGGAAGATGTAC	0.374069303	0.027761786	-0.287067414
ab_99547_ACAGATTCGAACAGGCGATGAATC	0.335496644	0.156838948	-0.261070439
ab_99547_ACAGATTCGCTCGGTAAGGCTAAC	0.502211482	0.029431667	-0.282583396
ab_99547_ACAGATTCGTACGCAAATCATTCC	0.583896298	0.243620396	-0.115717068

ab_99547_ACAGATTCTGGAACAACCATCCTC	0.655445183	0.138060998	-0.248052484
ab_99547_ACAGATTCTGGCTTCACAATGGAA	0.597227056	0.137012232	-0.177732219
ab_99547_ACAGATTCTGGTGGTACCATCCTC	0.468873539	0.123167531	-0.257122801
ab_99547_ACAGATTCTGGTGGTAGAGCTGAA	0.525079125	0.26278936	-0.138990518
ab_99547_ACAGCAGAAACCGAGACAGATCTG	0.335468925	0.018098615	-0.378831247
ab_99547_ACAGCAGAACGCTCGAATGCCTAA	0.418569836	0.134205872	-0.296455104
ab_99547_ACAGCAGAATCATTCCGAATCTGA	0.324560238	0.183507723	-0.299569935
ab_99547_ACAGCAGACAAGGAGCACAGATTC	0.481911913	0.080109967	-0.200826077
ab_99547_ACAGCAGACATACCAAAGTCACTA	0.601551194	0.188394556	-0.225733244
ab_99547_ACAGCAGACCGACAACTCCGTCTA	0.747137002	0.19880431	-0.323505352
ab_99547_ACAGCAGACCGTGAGAAAGGTACA	0.543370491	0.056825985	-0.177003453
ab_99547_ACAGCAGACTGAGCCAGACTAGTA	0.720614252	0.102824777	-0.361036821
ab_99547_ACAGCAGACTGAGCCAGAGCTGAA	0.659732028	0.123343332	-0.337897063
ab_99547_ACAGCAGAGACAGTGCGACTAGTA	0.534134083	0.094241726	-0.01425591
ab_99547_ACAGCAGAGGAGAACATCTTCACA	0.671239885	0.24588481	-0.308911813
ab_99547_ACAGCAGATCTTCACATCCGTCTA	0.660716041	0.096472999	-0.090433958
ab_99547_ACAGCAGATGGTGGTACGGATTGC	0.427332682	0.14911095	-0.378428149
ab_99547_ACATTGGCAGGCTAACGTCTGTCA	0.455050187	0.065199123	-0.360198292
ab_99547_ACATTGGCAGTACAAGAACGTGAT	0.452923078	0.129234586	-0.33320317
ab_99547_ACATTGGCCAAGGAGCTGAAGAGA	0.602474768	0.170189104	-0.131658582
ab_99547_ACATTGGCGCCAAGACACGTATCA	0.667120585	0.009378578	-0.282223744
ab_99547_ACATTGGCTCCGTCTATAGGATGA	0.647614826	0.362748748	-0.183944653
ab_99547_ACCACTGTAAACATCGCATCAAGT	0.642682431	0.003163708	-0.333708025
ab_99547_ACCACTGTAACTCACCGAGTTAGC	0.3831391	0.149059096	-0.266428103
ab_99547_ACCACTGTACACGACCTCCGTCTA	0.33394687	0.125920009	-0.340659519
ab_99547_ACCACTGTAGATGTACAGCCATGC	0.525701621	0.03698062	-0.473768184
ab_99547_ACCACTGTATAGCGACCCATCCTC	0.76599878	0.347425024	-0.29973476
ab_99547_ACCACTGTATTGAGGAAGAGTCAA	0.464861304	0.021965136	-0.365091161
ab_99547_ACCACTGTCATACCAAAGAGTCAA	0.74897835	0.076175935	-0.287100064
ab_99547_ACCACTGTCCAGTTCAGGAGAACA	0.480311354	0.2415669	-0.295729825
ab_99547_ACCACTGTCCGACAACTGGCTTCA	0.579851924	0.019562969	-0.350574543
ab_99547_ACCACTGTCCTAATCCGTCGTAGA	0.454720118	0.165814892	-0.187878556

ab_99547_ACCACTGTGTACGCAAACAGATTC	0.489597639	0.088389512	-0.359446191
ab_99547_ACCACTGTTCTTCACAGAGTTAGC	0.587467444	0.031670119	-0.279807728
ab_99547_ACCTCCAAAACAACCAGCCAAGAC	0.597913694	0.174576437	-0.310519001
ab_99547_ACCTCCAAAGTACAAGAAGACGGA	0.485050311	-0.001220307	-0.251206162
ab_99547_ACCTCCAAATCCTGTAAACTCACC	0.421033045	0.164753709	-0.180753465
ab_99547_ACCTCCAACGACACACCTGGCATA	0.584246532	0.072229023	-0.296957302
ab_99547_ACCTCCAACGCTGATCCGCATACA	0.493146008	0.213339039	-0.419248718
ab_99547_ACCTCCAACGGATTGCTGGCTTCA	0.569051917	0.187019098	-0.290849036
ab_99547_ACCTCCAACTCAATGAAGTACAAG	0.501332024	-0.044912781	-0.340931964
ab_99547_ACCTCCAACTCAATGAATTGAGGA	0.322975641	0.166230592	-0.217726818
ab_99547_ACCTCCAAGAGTTAGCACACGACC	0.701591307	0.317957384	-0.360967432
ab_99547_ACCTCCAATGAAGAGACGGATTGC	0.427504125	0.15013575	-0.286359244
ab_99547_ACGCTCGAAAGAGATCAGTCACTA	0.768387718	0.140133814	-0.306681601
ab_99547_ACGCTCGAAATCCGTCGACTAGTA	0.436432886	0.180975248	-0.189206768
ab_99547_ACGCTCGAACGTATCAGACTAGTA	0.584602983	0.074887985	-0.334142562
ab_99547_ACGCTCGAAGATGTACCGACACAC	0.464609376	0.183112129	-0.337612997
ab_99547_ACGCTCGAAGTACAAGGAATCTGA	0.555007485	0.077694141	-0.264564195
ab_99547_ACGCTCGAAGTGGTCAGTCTGTCA	0.674908651	0.142984001	-0.418962564
ab_99547_ACGCTCGAATCCTGTAATCCTGTA	0.524409974	0.118091961	-0.329417769
ab_99547_ACGCTCGACAAGGAGCGAATCTGA	0.487362502	0.008490004	-0.339565084
ab_99547_ACGCTCGACCAGTTCAACCACTGT	0.496926441	0.133100066	-0.18943404
ab_99547_ACGCTCGACGGATTGCGGTGCGAA	0.762731905	0.078458816	-0.261449156
ab_99547_ACGCTCGAGACTAGTAAGTCACTA	0.732687413	0.278920227	-0.167637968
ab_99547_ACGCTCGAGCCAAGACCAAGACTA	0.449836181	0.149608844	-0.233230412
ab_99547_ACGCTCGAGTCTGTCAACAGATTC	0.41780491	0.125159139	-0.390830362
ab_99547_ACGTATCAAACGCTTAACACAGAA	0.67418464	0.090272068	-0.125290261
ab_99547_ACGTATCAACACAGAAATCCTGTA	0.714433465	0.187128484	-0.297090508
ab_99547_ACGTATCAAGATCGCACGGATTGC	0.348359634	0.195498822	-0.205737967
ab_99547_ACGTATCACAGCGTTAACACAGAA	0.462132054	0.06677761	-0.30324952
ab_99547_ACGTATCACCATCCTCGGAGAACA	0.651872012	0.162538704	-0.218907686
ab_99547_ACGTATCACGACACACCGCATACA	0.590046901	0.217582193	-0.205438979
ab_99547_ACGTATCACGCTGATCCCTAATCC	0.384363023	0.045568852	-0.287966792

ab_99547_ACGTATCAGACTAGTAAGTCACTA	0.620943504	0.162909557	-0.223888735
ab_99547_ACGTATCAGGAGAACAGAATCTGA	0.464163526	0.17578411	-0.259990372
ab_99547_ACGTATCAGTCGTAGAAGATCGCA	0.468391646	0.113803703	-0.398274094
ab_99547_ACGTATCAGTCTGTCATGAAGAGA	0.608434506	0.111004961	-0.098139645
ab_99547_ACGTATCATGAAGAGAGACTAGTA	0.532350047	0.070381311	-0.184438188
ab_99547_ACGTATCATGGAACAAAAGGTACA	0.591124168	0.161195166	-0.231838384
ab_99547_ACTATGCAACAGCAGACCAGTTCA	0.633018404	0.10078756	-0.230962981
ab_99547_ACTATGCAAGCAGGAACGACACAC	0.561598087	0.027232506	-0.35183926
ab_99547_ACTATGCAATCCTGTAACGCTCGA	0.513269281	0.157329004	-0.216720524
ab_99547_ACTATGCACCGACAACAGCAGGAA	0.431497569	0.047999177	-0.327188631
ab_99547_ACTATGCACGACTGGAACATTGGC	0.56114618	0.106949519	-0.295157181
ab_99547_ACTATGCACGCATACATGGCTTCA	0.485559611	0.175149126	-0.244806606
ab_99547_ACTATGCACTGAGCCATAGGATGA	0.520181774	0.22172448	-0.36924818
ab_99547_ACTATGCAGAGTTAGCTAGGATGA	0.287562031	0.132467911	-0.229303052
ab_99547_ACTATGCAGCCAAGACCGACACAC	0.289071132	0.12851268	-0.392621321
ab_99547_ACTATGCAGGAGAACAGTGTTCTA	0.569920851	0.114677485	-0.235412648
ab_99547_AGAGTCAAAACCGAGAACACGACC	0.516955733	-0.015938587	-0.285860961
ab_99547_AGAGTCAAACGTATCACATCAAGT	0.611343507	0.075756134	-0.312574248
ab_99547_AGAGTCAAACTATGCAACAAGCTA	0.470907066	0.078637679	-0.24621061
ab_99547_AGAGTCAAAGAGTCAAAGTACAAG	0.603593529	0.020486	-0.324565881
ab_99547_AGAGTCAAAGATGTACCTGAGCCA	0.500450581	0.238755865	-0.276792646
ab_99547_AGAGTCAACAGATCTGGATAGACA	0.481864873	0.088614612	-0.343220982
ab_99547_AGAGTCAACAGATCTGTAGGATGA	0.458324405	0.025884593	-0.378687739
ab_99547_AGAGTCAACGAACTTAAAGACGGA	0.555710798	0.088834816	-0.39180387
ab_99547_AGAGTCAACTGTAGCCCAGCGTTA	0.672317272	0.087890197	-0.274824295
ab_99547_AGAGTCAAGCTAACGATGGCTTCA	0.426423174	0.104596566	-0.379728099
ab_99547_AGAGTCAAGGTGCGAAATCATTCC	0.430172043	0.057947871	-0.274234486
ab_99547_AGAGTCAATGGTGGTATATCAGCA	0.625190062	0.091305987	-0.369480625
ab_99547_AGATCGCAAACAACCAAGAGTCAA	0.365273046	0.135804849	-0.314030082
ab_99547_AGATCGCAACGCTCGAATTGAGGA	0.702375115	0.057787713	-0.387793313
ab_99547_AGATCGCAAGATCGCAAGTCACTA	0.416335188	0.050821587	-0.330471715
ab_99547_AGATCGCACAGATCTGAGATCGCA	0.508245488	0.14956276	-0.289690128

ab_99547_AGATCGCACAGCGTTACAGCGTTA	0.482696893	0.018592465	-0.162399712
ab_99547_AGATCGCACCGACAACAACCGAGA	0.418784228	0.155792048	-0.132740589
ab_99547_AGATCGCACCTAATCCCTAAGGTC	0.530755111	0.194197341	-0.076791626
ab_99547_AGATCGCACGACACACAACAACCA	0.627030176	0.168621792	-0.358836832
ab_99547_AGATCGCACGACACACCTGAGCCA	0.434007989	0.106454501	-0.267279756
ab_99547_AGATCGCAGACAGTGCGACTAGTA	0.419915702	-0.033137359	-0.334646868
ab_99547_AGATCGCAGAGCTGAAGAACAGGC	0.621084874	0.158721271	-0.208196371
ab_99547_AGATCGCAGCCACATATGGCTTCA	0.457979738	0.038702666	-0.204275604
ab_99547_AGATCGCAGGTGCGAACTGTAGCC	0.450631325	0.098663896	-0.333171107
ab_99547_AGATCGCATAGGATGACTCAATGA	0.551352728	0.143008232	-0.313398092
ab_99547_AGATCGCATCCGTCTACGACACAC	0.481980266	0.192343267	-0.362295951
ab_99547_AGATCGCATGGCTTCACTCAATGA	0.625611936	0.064479497	-0.369442614
ab_99547_AGATCGCATGGCTTCAGCTCGGTA	0.565764286	0.080619186	-0.29288016
ab_99547_AGATGTACAAGGACACCGCTGATC	0.206381374	0.133651719	-0.345928032
ab_99547_AGATGTACACCTCCAACGACTGGA	0.434247697	0.156966792	-0.355388657
ab_99547_AGATGTACACTATGCAAACTCACC	0.621773161	0.24285481	-0.312874352
ab_99547_AGATGTACAGAGTCAAACACAGAA	0.661930003	0.162711159	-0.355693673
ab_99547_AGATGTACAGTCACTAGAACAGGC	0.330804308	0.170611714	-0.292208197
ab_99547_AGATGTACAGTGGTCAGTACGCAA	0.351486053	0.138755132	-0.314114209
ab_99547_AGATGTACATAGCGACAACCGAGA	0.560234309	0.161530176	-0.229637469
ab_99547_AGATGTACCAACCACAGGAGAACA	0.467281444	0.179049622	-0.209833041
ab_99547_AGATGTACCAAGGAGCAGTCACTA	0.610869614	0.214180411	-0.389096672
ab_99547_AGATGTACCCATCCTCAACCGAGA	0.692253636	0.151527269	-0.286537701
ab_99547_AGATGTACCGAACTTACTGGCATA	0.435525632	0.06693971	-0.219845163
ab_99547_AGATGTACCGCTGATCGTACGCAA	0.659310982	-0.084269663	-0.353625876
ab_99547_AGATGTACGAACAGGCCTCAATGA	0.822939629	0.247030721	-0.324415905
ab_99547_AGATGTACGCCAAGACCCGACAAC	0.580535944	0.235843206	-0.312889017
ab_99547_AGATGTACGCGAGTAAAGCACCTC	0.536786423	0.095772944	-0.149636602
ab_99547_AGATGTACGTCTGTCAAACGCTTA	0.49613896	0.130471711	-0.353399829
ab_99547_AGCACCTCAAGGTACAACTATGCA	0.247022799	0.077744569	-0.155100519
ab_99547_AGCACCTCACAGATTCAGATCGCA	0.699624087	0.267064405	-0.244600107
ab_99547_AGCACCTCATCCTGTATGGTGGTA	0.694649924	0.06665155	-0.28181456

ab_99547_AGCACCTCATGCCTAACAATGGAA	0.567884594	0.142197556	-0.303476014
ab_99547_AGCACCTCGAACAGGCGTACGCAA	0.707959795	0.165668565	-0.223753497
ab_99547_AGCACCTCTGGAACAACGAACTTA	0.51461665	0.066688709	-0.139875588
ab_99547_AGCACCTCTTCACGCAAATCCGTC	0.386622292	0.122801015	-0.195326194
ab_99547_AGCAGGAAAACCGAGAGGAGAACA	0.437087976	0.162366031	-0.312662608
ab_99547_AGCAGGAAAACGCTTATCTTCACA	0.533928781	0.068762187	-0.277692753
ab_99547_AGCAGGAAAGATCGCACGCATACA	0.612908835	0.067840469	-0.336211389
ab_99547_AGCAGGAAAGTGGTCAAACCGAGA	0.532973895	0.168164221	-0.277685665
ab_99547_AGCAGGAAAGTGGTCAAACTCACC	0.615627128	0.083799916	-0.191811694
ab_99547_AGCAGGAACTGGCATAAAGACGGA	0.456838637	0.157439639	-0.23338444
ab_99547_AGCAGGAAGAGTTAGCTAGGATGA	0.455622009	0.171988677	-0.307015933
ab_99547_AGCAGGAAGTCGTAGAACATTGGC	0.554208485	0.112040379	-0.30717098
ab_99547_AGCAGGAATAGGATGATAGGATGA	0.598683499	0.010289827	-0.395377222
ab_99547_AGCAGGAATGGTGGTAAACGCTTA	0.580816093	0.169847851	-0.170788763
ab_99547_AGCCATGCAATCCGTCCCTAATCC	0.309698132	0.042549448	-0.277844713
ab_99547_AGCCATGCATCCTGTATCTTCACA	0.484587304	0.212326217	-0.239967474
ab_99547_AGCCATGCATCCTGTATGGTGGTA	0.672769219	0.108676508	-0.169517736
ab_99547_AGCCATGCCAACCACACTAAGGTC	0.560065571	0.125058946	-0.281474856
ab_99547_AGCCATGCCAAGACTATCTTCACA	0.602116286	0.048382373	-0.316467637
ab_99547_AGCCATGCCAGATCTGAACTCACC	0.54320645	0.014538918	-0.213883125
ab_99547_AGCCATGCCCATCCTCGCTAACGA	0.370037828	0.189139305	-0.233061465
ab_99547_AGCCATGCCCGAAGTACATACCAA	0.463280459	0.327666015	-0.19681621
ab_99547_AGCCATGCCGAACTTAAACGTGAT	0.378304637	0.14314087	-0.201397296
ab_99547_AGCCATGCCTCAATGACGACTGGA	0.522241163	0.084147926	-0.398205311
ab_99547_AGCCATGCCTGGCATAAACTCACC	0.589034559	0.168122774	-0.248147452
ab_99547_AGCCATGCGAGTTAGCAGCAGGAA	0.899527516	0.06678202	-0.200066516
ab_99547_AGCCATGCTGGAACAACGAACTTA	0.608694577	0.223253126	-0.290195634
ab_99547_AGGCTAACAATCCGTCCCTAATCC	0.510499826	0.101032147	-0.213841005
ab_99547_AGGCTAACAATGTTGCCAAGGAGC	0.498874542	0.062082918	-0.220610794
ab_99547_AGGCTAACACAAGCTATAGGATGA	0.474534026	0.053941041	-0.194546976
ab_99547_AGGCTAACACACAGAAATGCCTAA	0.330903507	0.166299419	-0.159663421
ab_99547_AGGCTAACACAGCAGAACAGCAGA	0.592041159	0.301291494	-0.24837382

ab_99547_AGGCTAACAGCCATGCGACAGTGC	0.526975531	0.08100255	-0.230033892
ab_99547_AGGCTAACAGGCTAACCAATGGAA	0.835756026	0.072926315	-0.310003454
ab_99547_AGGCTAACATTGGCTCACAGATTC	0.601050733	0.234591539	-0.320936558
ab_99547_AGGCTAACCCGACAACCTGTAGCC	0.557505269	0.280137636	-0.208814087
ab_99547_AGGCTAACCCTCCTGACAGCGTTA	0.74819929	0.004848719	-0.084000684
ab_99547_AGGCTAACCCTCTATCGCCACATA	0.661221364	0.265228644	-0.197738938
ab_99547_AGGCTAACCGACACACCACCTTAC	0.426647493	0.113875965	-0.286929724
ab_99547_AGGCTAACGACAGTGCACTATGCA	0.477989603	0.013130778	-0.377947408
ab_99547_AGGCTAACGTGTTCTACCGTGAGA	0.423877515	0.059377116	-0.166226474
ab_99547_AGTACAAGAACCGAGAAAGACGGA	0.664200777	0.032505484	-0.342760851
ab_99547_AGTACAAGAGCAGGAACCTCCTGA	0.3777097	0.278845928	-0.250854475
ab_99547_AGTACAAGAGCAGGAAGTCTGTCA	0.495542136	0.148907289	-0.323177783
ab_99547_AGTACAAGATTGAGGAACTATGCA	0.458341258	0.205512636	-0.045687276
ab_99547_AGTACAAGCGCATACAAACGCTTA	0.680515586	0.176551775	-0.251380266
ab_99547_AGTACAAGCGGATTGCACACAGAA	0.500131665	0.091392358	-0.319805581
ab_99547_AGTACAAGCTAAGGTCCAGATCTG	0.374131074	0.155533161	-0.225748953
ab_99547_AGTACAAGCTGGCATACGCTGATC	0.512667782	-0.019246122	-0.391326052
ab_99547_AGTACAAGGAACAGGCCGGATTGC	0.507977778	0.020893463	-0.230066034
ab_99547_AGTACAAGGACTAGTACGCATACA	0.385432898	0.089741806	-0.233208842
ab_99547_AGTACAAGGTACGCAAACCACTGT	0.473029502	0.133230272	-0.333468339
ab_99547_AGTACAAGGTCGTAGAAACAACCA	0.504894103	0.138572132	-0.363373384
ab_99547_AGTCACTAACCACTGTAGAGTCAA	0.496111915	0.07754512	-0.316657583
ab_99547_AGTCACTAACGCTCGAGCCACATA	0.462771373	0.115716225	-0.291200411
ab_99547_AGTCACTAAGAGTCAAAAGGACAC	0.390202946	0.19245814	-0.299877031
ab_99547_AGTCACTAAGCACCTCAGATCGCA	0.487794545	0.256805076	-0.149187898
ab_99547_AGTCACTAATGCCTAACACCTTAC	0.536833115	0.050008132	-0.183435603
ab_99547_AGTCACTACCATCCTCTATCAGCA	0.535500445	0.150308172	-0.243917433
ab_99547_AGTCACTAGAATCTGAGAGTTAGC	0.372198679	-0.016855513	-0.317438465
ab_99547_AGTCACTAGACTAGTAATTGGCTC	0.476606534	0.211957848	-0.330365596
ab_99547_AGTCACTAGCGAGTAAACCACTGT	0.425598779	0.078629712	-0.227921731
ab_99547_AGTCACTAGTACGCAAAGGCTAAC	0.59001337	0.027232466	-0.303636181
ab_99547_AGTCACTAGTGTTCTATTCACGCA	0.523931077	0.154762466	-0.236579614

ab_99547_AGTCACTATATCAGCACAAGACTA	0.526087359	0.205438965	-0.257091823
ab_99547_AGTCACTATGGAACAAGACAGTGC	0.759492895	0.245601464	-0.222655882
ab_99547_AGTGGTCAAATGTTGCGAATCTGA	0.393889798	0.104293956	-0.192499429
ab_99547_AGTGGTCAACCTCCAAGTGTTCTA	0.438454475	0.11643171	-0.243947547
ab_99547_AGTGGTCAACTATGCACAACCACA	0.400316061	0.057937187	-0.291083863
ab_99547_AGTGGTCAACTATGCACTCAATGA	0.458926079	0.066281012	-0.175745914
ab_99547_AGTGGTCAATCCTGTACGGATTGC	0.556911282	0.153051045	-0.327759932
ab_99547_AGTGGTCACAAGACTAACAGATTC	0.450027862	0.050641391	-0.246865713
ab_99547_AGTGGTCACCTCTATCAAGGACAC	0.491462547	0.253076493	-0.306230903
ab_99547_AGTGGTCACGAACTTACTAAGGTC	0.621888845	0.129512725	-0.342475499
ab_99547_AGTGGTCACGAACTTATGGAACAA	0.48458371	0.219124761	-0.125013479
ab_99547_AGTGGTCAGCGAGTAAGTACGCAA	0.74407434	0.215890459	-0.206185515
ab_99547_AGTGGTCAGTCGTAGAAGAGTCAA	0.391287734	0.032939838	-0.345175385
ab_99547_AGTGGTCATTCACGCAAAGGTACA	0.286994629	0.134370155	-0.134755263
ab_99547_AGTGGTCATTCACGCAAGAGTCAA	0.481705838	0.225297228	-0.154013486
ab_99547_ATAGCGACAAGGACACAGTACAAG	0.344311561	0.163206492	-0.170442964
ab_99547_ATAGCGACACGCTCGAAGATGTAC	0.336294969	0.095409985	-0.34614
ab_99547_ATAGCGACACGCTCGATGGCTTCA	0.569781776	-0.104654094	-0.268096382
ab_99547_ATAGCGACAGATGTACACAGATTC	0.512948513	0.09748702	-0.367407081
ab_99547_ATAGCGACAGTCACTACGACTGGA	0.419046644	0.298449534	-0.391363588
ab_99547_ATAGCGACATCATTCCACACGACC	0.583408278	0.112818032	-0.316326397
ab_99547_ATAGCGACCAGCGTTATATCAGCA	0.643416862	0.074533586	-0.330426286
ab_99547_ATAGCGACCTCAATGAAAGAGATC	0.334940961	0.065667686	-0.419970805
ab_99547_ATAGCGACCTCAATGAGAGTTAGC	0.608883771	0.217542477	-0.239229562
ab_99547_ATAGCGACGAGTTAGCCATACCAA	0.573444722	0.042308478	-0.413942223
ab_99547_ATAGCGACGGTGCGAAAAGACGGA	0.506821257	0.124861306	-0.295835467
ab_99547_ATAGCGACGTCTGTCAATCCTGTA	0.71026023	0.075049931	-0.253422304
ab_99547_ATAGCGACTGGTGGTAAGCACCTC	0.508428311	0.241981139	-0.333021024
ab_99547_ATAGCGACTTCACGCAAGCACCTC	0.432148205	0.117048015	-0.157402539
ab_99547_ATCATTCCCAACCACAAGTCACTA	0.386517968	0.079328315	-0.311224754
ab_99547_ATCATTCCCAAGGAGCAGGCTAAC	0.486149466	0.13988983	-0.427283205
ab_99547_ATCATTCCCAATGGAACCAGTTCA	0.308326622	0.011663492	-0.386997621

ab_99547_ATCATTCCCGCTGATCAAGGTACA	0.763564614	0.007703332	-0.307405028
ab_99547_ATCATTCCGAGCTGAAATTGGCTC	0.205584707	0.060490156	-0.363689938
ab_99547_ATCATTCCGTCGTAGAATAGCGAC	0.545662681	0.14405398	-0.314344032
ab_99547_ATCATTCCTATCAGCAAAGGACAC	0.480550019	0.160898781	-0.271309688
ab_99547_ATCCTGTAAAGACGGAAACAACCA	0.493517012	0.240331378	-0.179514604
ab_99547_ATCCTGTAACACGACCCGCATACA	0.431263396	0.042623555	-0.223542539
ab_99547_ATCCTGTAAGATGTACAGTACAAG	0.54339084	0.161785058	-0.304746229
ab_99547_ATCCTGTAAGATGTACGTACGCAA	0.623820627	0.136697642	-0.383142999
ab_99547_ATCCTGTACAGCGTTAAGCACCTC	0.719799213	0.255801643	-0.255997425
ab_99547_ATCCTGTAGCTAACGAATTGGCTC	0.530590124	0.210842441	-0.363582688
ab_99547_ATCCTGTATGGCTTCACAATGGAA	0.550171491	0.162399539	-0.262293142
ab_99547_ATCCTGTATGGTGGTACACTTCGA	0.574528308	0.112353013	-0.208088827
ab_99547_ATGCCTAAAAGAGATCATGCCTAA	0.390544931	0.186133633	-0.198964911
ab_99547_ATGCCTAAAAGGTACACAATGGAA	0.401008908	0.019836365	-0.412266349
ab_99547_ATGCCTAAACAAGCTAAAGGACAC	0.440443264	0.112557196	-0.190391748
ab_99547_ATGCCTAAACAAGCTAGTCTGTCA	0.319003091	0.097762124	-0.28239507
ab_99547_ATGCCTAAACGCTCGAAAGAGATC	0.585921694	0.1045084	-0.324328356
ab_99547_ATGCCTAAATTGGCTCCGACACAC	0.330263306	0.185133149	-0.356972981
ab_99547_ATGCCTAAGCCACATATATCAGCA	0.757458774	0.165713259	-0.382149732
ab_99547_ATGCCTAAGTGTTCTACCGACAAC	0.72019723	0.161648586	-0.375976709
ab_99547_ATTGAGGAAACGTGATAACTCACC	0.387464716	0.016137567	-0.209339624
ab_99547_ATTGAGGAAAGGACACGAGCTGAA	0.474109006	0.112788867	-0.209277729
ab_99547_ATTGAGGAACACGACCAGGCTAAC	0.539398616	0.192288094	-0.35835491
ab_99547_ATTGAGGAACGCTCGAAGGCTAAC	0.553645795	0.188903269	-0.225658311
ab_99547_ATTGAGGAAGAGTCAACCTCCTGA	0.844056174	0.112126045	-0.256296773
ab_99547_ATTGAGGAAGATGTACCGACTGGA	0.510201744	0.081339479	-0.292529335
ab_99547_ATTGAGGAAGATGTACGTGTTCTA	0.355774234	0.182315069	-0.336255599
ab_99547_ATTGAGGAAGTACAAGAGTACAAG	0.242230156	0.102432551	-0.220451114
ab_99547_ATTGAGGAATGCCTAATCTTCACA	0.68377012	0.033965238	-0.343711689
ab_99547_ATTGAGGAATTGGCTCAGCACCTC	0.450858969	0.067234935	-0.318447398
ab_99547_ATTGAGGACAAGGAGCACAGATTC	0.46346053	0.182552177	-0.301622026
ab_99547_ATTGAGGACCAGTTCACAAGACTA	0.514693414	0.047439169	-0.301239785

ab_99547_ATTGAGGATAGGATGAAGCCATGC	0.466346177	0.037083356	-0.328305051
ab_99547_ATTGGCTCAAGGACACGCTAACGA	0.636120457	0.215592466	-0.166252726
ab_99547_ATTGGCTCAGCACCTCATCCTGTA	0.285910547	-0.002969927	-0.212878196
ab_99547_ATTGGCTCAGGCTAACCTAAGGTC	0.50856203	0.116101775	-0.272188503
ab_99547_ATTGGCTCAGGCTAACGAGCTGAA	0.39191991	0.077305361	-0.286701902
ab_99547_ATTGGCTCCTGAGCCACCATCCTC	0.595420565	0.228630619	-0.215635526
ab_99547_ATTGGCTCTGAAGAGAATGCCTAA	0.709763517	0.042135507	-0.296146274
ab_99547_CAACCACAACAGCAGAAAACATCG	0.634731865	0.332179197	-0.197771899
ab_99547_CAACCACACACTTCGAAAGGTACA	0.604665716	0.137714132	-0.254448644
ab_99547_CAACCACACAGCGTTACCAGTTCA	0.546347745	0.089007487	-0.403178861
ab_99547_CAACCACACCGTGAGAACGTATCA	0.558166489	0.064516494	-0.245310699
ab_99547_CAACCACACCTCCTGAAGTCACTA	0.4288706	0.175733381	-0.324932441
ab_99547_CAACCACAGCTAACGATGAAGAGA	0.824024023	0.138091713	-0.138183119
ab_99547_CAACCACATGGTGGTAAGTACAAG	0.399231079	0.060110527	-0.294034781
ab_99547_CAACCACATTCACGCAACACGACC	0.519293966	0.091030373	-0.386270873
ab_99547_CAAGACTAAAACATCGCCGACAAC	0.62717864	0.010133364	-0.289197363
ab_99547_CAAGACTAAAGGACACATCCTGTA	0.632749639	0.099631211	-0.303985115
ab_99547_CAAGACTAAGCCATGCACGTATCA	0.459579738	0.120285167	-0.277989507
ab_99547_CAAGACTAATTGGCTCCGCTGATC	0.572233465	0.083875232	-0.292646458
ab_99547_CAAGACTAATTGGCTCGCCACATA	0.690014045	0.15243749	-0.231876465
ab_99547_CAAGACTACCAGTTCATGGCTTCA	0.503215074	0.044252175	-0.398724947
ab_99547_CAAGACTACCTCTATCAACCGAGA	0.696849351	0.231463788	-0.133255123
ab_99547_CAAGACTACCTCTATCATGCCTAA	0.663067554	0.246918701	-0.237663133
ab_99547_CAAGACTACGGATTGCCCGACAAC	0.513251642	0.250484192	-0.274814891
ab_99547_CAAGACTACTAAGGTCATCATTCC	0.384298015	0.033494452	-0.352752748
ab_99547_CAAGACTAGACAGTGCCCAGTTCA	0.630632214	0.198350522	-0.354567554
ab_99547_CAAGACTAGATAGACACATCAAGT	0.618015993	0.265085549	-0.381738368
ab_99547_CAAGACTAGATGAATCTAGGATGA	0.708681619	0.096776054	-0.375958123
ab_99547_CAAGACTAGTCTGTCAAAGACGGA	0.656528476	0.152500074	-0.27356582
ab_99547_CAAGGAGCAACCGAGAAAGGTACA	0.599206525	0.000408236	-0.30796621
ab_99547_CAAGGAGCAACGCTTAGAGTTAGC	0.421755763	0.119936695	-0.314605949
ab_99547_CAAGGAGCAACTCACCATCATTCC	0.369580634	0.053239455	-0.317643799

ab_99547_CAAGGAGCACAAGCTACAAGACTA	0.462896021	0.147519105	-0.3260724
ab_99547_CAAGGAGCACAGATTCCTCAATGA	0.557255965	0.082987675	-0.249215699
ab_99547_CAAGGAGCACCTCCAAAGATCGCA	0.438512351	0.19252038	-0.364128954
ab_99547_CAAGGAGCAGATCGCAAAGACGGA	0.679274607	0.00364005	-0.380725943
ab_99547_CAAGGAGCCAACCACAAACGTGAT	0.472868302	0.078280294	-0.132343922
ab_99547_CAAGGAGCCAATGGAATGGCTTCA	0.679163548	-0.019100455	-0.491049755
ab_99547_CAAGGAGCCACCTTACTGGCTTCA	0.518542098	0.031044675	-0.412981334
ab_99547_CAAGGAGCCACTTCGACGAACTTA	0.424157291	0.096201002	-0.263465589
ab_99547_CAAGGAGCCACTTCGATCCGTCTA	0.240334737	0.172142897	-0.153861332
ab_99547_CAAGGAGCCAGATCTGAACGTGAT	0.607959418	0.156423228	-0.181180193
ab_99547_CAAGGAGCCCTAATCCACAGATTC	0.651385422	0.015695397	-0.314634423
ab_99547_CAAGGAGCCGCTGATCAGGCTAAC	0.515551491	0.050259008	-0.310667934
ab_99547_CAAGGAGCCTGGCATAACACAGAA	0.580548034	0.074485015	-0.299680851
ab_99547_CAAGGAGCGCGAGTAAGCGAGTAA	0.294226084	0.200244797	-0.302918361
ab_99547_CAAGGAGCGTCTGTCACCAGTTCA	0.458516098	0.060313864	-0.330008233
ab_99547_CAAGGAGCTAGGATGAATGCCTAA	0.414270987	0.11440852	-0.26751515
ab_99547_CAAGGAGCTAGGATGAGTGTTCTA	0.480858625	0.17061121	-0.038975393
ab_99547_CAAGGAGCTATCAGCAAACTCACC	0.569133796	0.073574662	-0.195968336
ab_99547_CAAGGAGCTGGTGGTAACAAGCTA	0.668022404	0.134994959	-0.294962557
ab_99547_CAAGGAGCTTCACGCAAAGACGGA	0.363298409	0.095651014	-0.135453271
ab_99547_CAATGGAAAAACATCGACGTATCA	0.365958846	0.141665711	-0.31155873
ab_99547_CAATGGAAAACTCACCCAACCACA	0.588232378	0.154603889	-0.045782192
ab_99547_CAATGGAAACACGACCAGATCGCA	0.414467321	0.218013004	-0.281367066
ab_99547_CAATGGAAAGCCATGCCATACCAA	0.407785805	0.067406628	-0.336934036
ab_99547_CAATGGAAAGGCTAACAGTACAAG	0.473658606	0.126365734	-0.343636654
ab_99547_CAATGGAAATCCTGTAACACGACC	0.736997272	0.113565439	-0.34138358
ab_99547_CAATGGAAATCCTGTACAGCGTTA	0.556323215	0.147241055	0.014946597
ab_99547_CAATGGAAATTGAGGATGGTGGTA	0.494653434	0.121627343	-0.299593174
ab_99547_CAATGGAAATTGGCTCAACGTGAT	0.455023095	0.250366222	-0.278001595
ab_99547_CAATGGAACTAAGGTCATGCCTAA	0.344431193	0.169820235	-0.220857967
ab_99547_CAATGGAAGTCGTAGAACAGATTC	0.501373329	0.174484823	-0.229820997
ab_99547_CAATGGAATAGGATGACCGTGAGA	0.43122851	0.108519753	-0.183808315

ab_99547_CACCTTACAAGGACACCGCATACA	0.605221311	0.072381043	-0.25857327
ab_99547_CACCTTACAATCCGTCAGATGTAC	0.508676682	0.179907178	-0.246300665
ab_99547_CACCTTACACACAGAATTCACGCA	0.454110618	0.238035937	-0.284544332
ab_99547_CACCTTACACTATGCACGCTGATC	0.39661132	0.172353759	-0.321582819
ab_99547_CACCTTACAGTACAAGCGGATTGC	0.620261417	0.009132278	-0.315444469
ab_99547_CACCTTACCAGATCTGAGATCGCA	0.447923191	0.128788157	-0.285780201
ab_99547_CACCTTACCATACCAAAAGGTACA	0.459226986	0.180256122	-0.289506387
ab_99547_CACCTTACCCGACAACCCGACAAC	0.559808735	0.098790236	-0.379383526
ab_99547_CACCTTACGACAGTGCGATGAATC	0.406443582	0.146831122	-0.224180249
ab_99547_CACCTTACGCCACATAACAGCAGA	0.546656728	0.120509337	-0.341269589
ab_99547_CACCTTACGTCGTAGAGAGTTAGC	0.547410519	0.239206151	-0.219551594
ab_99547_CACTTCGAAACGCTTAACACAGAA	0.409388084	0.112013286	-0.306810634
ab_99547_CACTTCGAAAGAGATCCCTAATCC	0.719583108	0.091903323	-0.392929787
ab_99547_CACTTCGAAAGAGATCGCTCGGTA	0.47291043	0.092999931	-0.211959354
ab_99547_CACTTCGAAGATCGCAGAATCTGA	0.291825407	0.126707707	-0.286739561
ab_99547_CACTTCGAAGGCTAACCAATGGAA	0.641828565	0.084872543	-0.334202336
ab_99547_CACTTCGACAAGACTAGAGTTAGC	0.425665426	0.113856322	-0.332095903
ab_99547_CACTTCGACAAGGAGCGTCGTAGA	0.499062403	0.17004975	-0.242573736
ab_99547_CACTTCGACATACCAACACCTTAC	0.458470288	0.052444677	-0.181473691
ab_99547_CACTTCGACGCATACAAACGCTTA	0.349796088	0.116376775	-0.322016668
ab_99547_CACTTCGACTGGCATACACTTCGA	0.35281383	0.175797863	-0.333991426
ab_99547_CACTTCGAGAGTTAGCACAGATTC	0.563459544	0.237817417	-0.265992775
ab_99547_CACTTCGAGTCGTAGAAATCCGTC	0.546294574	0.207017499	-0.163360557
ab_99547_CACTTCGAGTCGTAGATAGGATGA	0.649571548	0.069273982	-0.339585318
ab_99547_CACTTCGATTCACGCAACACGACC	0.388216574	-0.045568367	-0.352009349
ab_99547_CACTTCGATTCACGCAACAGCAGA	0.564930416	0.104898759	-0.225980096
ab_99547_CAGATCTGACAGATTCGAACAGGC	0.411886113	0.190511093	-0.301113438
ab_99547_CAGATCTGACCACTGTGATGAATC	0.384938101	0.033165358	-0.355001067
ab_99547_CAGATCTGAGATCGCAAATCCGTC	0.369044987	0.016084502	-0.292950929
ab_99547_CAGATCTGATCATTCCAGTCACTA	0.568331147	0.076512831	-0.330244351
ab_99547_CAGATCTGCATACCAAAGAGTCAA	0.748496343	0.333273773	-0.268408045
ab_99547_CAGATCTGGATAGACAGTGTTCTA	0.267246393	0.126478104	-0.319899957

ab_99547_CAGCGTTAACAGATTCACACAGAA	0.384110539	-0.033637276	-0.386992305
ab_99547_CAGCGTTAACATTGGCAACGCTTA	0.421304272	0.099193103	-0.317071784
ab_99547_CAGCGTTAATCCTGTAAACTCACC	0.375415891	0.161088478	-0.272768568
ab_99547_CAGCGTTACACCTTACATTGGCTC	0.343420957	0.117134713	-0.312729588
ab_99547_CAGCGTTACCTCTATCCTGGCATA	0.41169138	0.162248413	-0.262885672
ab_99547_CAGCGTTACTAAGGTCAACTCACC	0.336969625	0.130344731	-0.406902972
ab_99547_CAGCGTTACTAAGGTCGACTAGTA	0.446299605	0.099153708	-0.259765988
ab_99547_CAGCGTTAGTCTGTCATGGTGGTA	0.5952463	0.115175075	-0.322413993
ab_99547_CATACCAAAATGTTGCACAAGCTA	0.730350397	0.258776478	-0.237624998
ab_99547_CATACCAAAGATCGCAGTGTTCTA	0.51807676	0.084510891	-0.329922999
ab_99547_CATACCAAATTGAGGAGTCGTAGA	0.541148737	0.243560825	-0.246575364
ab_99547_CATACCAACAAGACTAGGAGAACA	0.375142622	0.092791145	-0.308888369
ab_99547_CATACCAACATACCAACTAAGGTC	0.381702037	0.137839473	-0.216970141
ab_99547_CATACCAACATCAAGTCTGAGCCA	0.317253822	0.112684539	-0.381942396
ab_99547_CATACCAACGCATACACACCTTAC	0.343150704	0.145781554	-0.27780756
ab_99547_CATACCAACTCAATGAATGCCTAA	0.519103476	0.27743556	-0.12598695
ab_99547_CATACCAAGACTAGTAATAGCGAC	0.739721894	0.132708165	-0.322880339
ab_99547_CATACCAAGATAGACAAAGACGGA	0.422667234	0.158267922	-0.31516091
ab_99547_CATACCAATGGCTTCACTCAATGA	0.470813397	0.2191675	-0.217000687
ab_99547_CATCAAGTAACAACCAGGTGCGAA	0.196755923	0.000415458	-0.181035309
ab_99547_CATCAAGTAACCGAGAGAGCTGAA	0.523128582	0.169765032	-0.353879158
ab_99547_CATCAAGTACAGCAGAAAGAGATC	0.473220767	0.212259612	-0.333297929
ab_99547_CATCAAGTACCACTGTACTATGCA	0.419144796	0.123498535	-0.347188564
ab_99547_CATCAAGTAGATGTACGCTCGGTA	0.460325819	0.128255061	-0.123787198
ab_99547_CATCAAGTCCTCCTGAATCATTCC	0.489902089	0.146178128	-0.202563692
ab_99547_CATCAAGTGAATCTGACACTTCGA	0.663330158	0.203221738	-0.226881214
ab_99547_CATCAAGTTGGCTTCAATAGCGAC	0.296241239	0.227428109	-0.327532524
ab_99547_CCAGTTCAATCCTGTAACACAGAA	0.661244762	-0.017227428	-0.318984839
ab_99547_CCAGTTCACAACCACAACCTCCAA	0.541225824	0.244184628	-0.180218892
ab_99547_CCAGTTCACTCAATGAGATGAATC	0.593799882	0.086303949	-0.293733638
ab_99547_CCAGTTCAGAACAGGCAAGGACAC	0.38001736	0.246184826	-0.270430705
ab_99547_CCAGTTCAGCCACATAAATGTTGC	0.516755498	0.171964382	-0.191199321

ab_99547_CCAGTTCATGAAGAGACCGTGAGA	0.435570788	0.1257274	-0.373139543
ab_99547_CCATCCTCAATCCGTCGAATCTGA	0.721163057	0.134820399	-0.177644648
ab_99547_CCATCCTCAATCCGTCTGGCTTCA	0.603916451	0.166063913	-0.086080813
ab_99547_CCATCCTCACACGACCATAGCGAC	0.653381661	-0.06673651	-0.236884164
ab_99547_CCATCCTCACACGACCGACAGTGC	0.435785982	0.116001355	-0.240619365
ab_99547_CCATCCTCAGGCTAACCGAACTTA	0.596266399	0.132083717	-0.301179846
ab_99547_CCATCCTCATGCCTAACCTAATCC	0.535474398	0.082078606	-0.272350394
ab_99547_CCATCCTCCAATGGAAAGCACCTC	0.301394638	0.102385528	-0.181708233
ab_99547_CCATCCTCCATCAAGTATCCTGTA	0.328608545	0.113011963	-0.306324462
ab_99547_CCATCCTCCCAGTTCACCTAATCC	0.534071924	-0.006504587	-0.334768917
ab_99547_CCATCCTCCCGACAACGCCACATA	0.381344418	0.221387972	-0.239218153
ab_99547_CCATCCTCCGACTGGAAACGTGAT	0.458032741	0.118938375	-0.303530983
ab_99547_CCATCCTCCTCAATGACAACCACA	0.491703518	0.118392631	-0.29023509
ab_99547_CCATCCTCGCGAGTAAACACGACC	0.339100053	0.20989299	-0.123831965
ab_99547_CCATCCTCGTACGCAAACACGACC	0.475537813	0.053921141	-0.334976712
ab_99547_CCATCCTCTATCAGCAACCACTGT	0.474253447	0.098407816	-0.176419924
ab_99547_CCGAAGTAAACTCACCCCTAATCC	0.689822032	0.101696489	-0.250157934
ab_99547_CCGAAGTAACACAGAAATAGCGAC	0.443926787	0.168581751	-0.276248313
ab_99547_CCGAAGTAACATTGGCACACAGAA	0.728113564	0.151953369	-0.105422087
ab_99547_CCGAAGTAACATTGGCCGCATACA	0.388157661	0.098488351	-0.036317267
ab_99547_CCGAAGTAACCTCCAACGCATACA	0.550983774	0.160499668	-0.30332209
ab_99547_CCGAAGTAAGATCGCACGCTGATC	0.60374052	0.178632132	-0.199626934
ab_99547_CCGAAGTACAATGGAACCTAATCC	0.70847799	0.051917404	-0.337200742
ab_99547_CCGAAGTACCATCCTCCAACCACA	0.655167532	0.075149471	-0.352333454
ab_99547_CCGAAGTACGACACACGCTCGGTA	0.372011868	0.178024119	-0.292619211
ab_99547_CCGAAGTACTGAGCCAAGATCGCA	0.588601904	0.128849681	-0.275238306
ab_99547_CCGACAACACACAGAAGTCTGTCA	0.252737837	0.05680742	-0.179963208
ab_99547_CCGACAACAGAGTCAAGAATCTGA	0.635637691	0.153912111	-0.035198366
ab_99547_CCGACAACAGATGTACGTGTTCTA	0.50506517	-0.085513275	-0.258475759
ab_99547_CCGACAACAGCACCTCCTGTAGCC	0.550937245	0.076410892	-0.231256527
ab_99547_CCGACAACAGCCATGCAATCCGTC	0.353967165	0.117389141	-0.22273384
ab_99547_CCGACAACATCCTGTACTAAGGTC	0.415113178	0.025200653	-0.391253169

ab_99547_CCGACAACCAAGACTAGACTAGTA	0.41669463	0.030788909	-0.275231801
ab_99547_CCGACAACCACCTTACAGAGTCAA	0.434207566	0.186413739	-0.271595394
ab_99547_CCGACAACCACTTCGAAACAACCA	0.487888372	0.030975861	-0.314599575
ab_99547_CCGACAACCGACTGGATGGAACAA	0.250380429	0.053168437	-0.298333653
ab_99547_CCGACAACCGGATTGCACGCTCGA	0.526716709	0.053177388	-0.32143209
ab_99547_CCGACAACGAATCTGAGTCGTAGA	0.699125582	0.020095023	-0.334874285
ab_99547_CCGACAACGAGCTGAACCTCTATC	0.412845222	0.077328985	-0.382986197
ab_99547_CCGTGAGAACACAGAAAGCACCTC	0.345788269	0.19368411	-0.375943913
ab_99547_CCGTGAGAACACAGAACACTTCGA	0.414919866	0.119177517	-0.287096298
ab_99547_CCGTGAGAACTATGCATATCAGCA	0.674399169	0.104754011	-0.288975622
ab_99547_CCGTGAGAAGTACAAGACATTGGC	0.745769973	0.236651405	-0.242063945
ab_99547_CCGTGAGAAGTGGTCAGATAGACA	0.668734959	0.212919039	-0.322069895
ab_99547_CCTAATCCACAAGCTAAATCCGTC	0.696951781	0.136879189	-0.209459825
ab_99547_CCTAATCCACCTCCAAAGCACCTC	0.340516609	0.116874208	-0.365323
ab_99547_CCTAATCCACGTATCAAACGCTTA	0.558226109	0.035738195	-0.399495122
ab_99547_CCTAATCCCAATGGAAACCACTGT	0.452457271	0.082663079	-0.233944689
ab_99547_CCTAATCCCAATGGAACTGGCATA	0.371581663	0.273302793	-0.284547023
ab_99547_CCTAATCCCCTCCTGACACTTCGA	0.563044069	0.153234754	-0.28975733
ab_99547_CCTAATCCCGACTGGAAACAACCA	0.478673592	0.176053803	-0.212195601
ab_99547_CCTAATCCGAACAGGCACACAGAA	0.431776587	0.111609087	-0.355883017
ab_99547_CCTAATCCGCCAAGACCGACTGGA	0.599793942	0.071026234	-0.119382482
ab_99547_CCTAATCCGCGAGTAAGTCGTAGA	0.601147904	-0.059923984	-0.355659057
ab_99547_CCTAATCCTGGAACAAAGTCACTA	0.453556005	0.063216353	-0.236731621
ab_99547_CCTCCTGAAAGGACACCCTCTATC	0.21556322	0.151260197	-0.21958981
ab_99547_CCTCCTGAACCACTGTAGCAGGAA	0.803885773	0.061857236	-0.257761312
ab_99547_CCTCCTGAACCACTGTCGGATTGC	0.585743641	0.099670521	-0.344179495
ab_99547_CCTCCTGAATAGCGACACAGCAGA	0.343228118	0.044812215	-0.075958918
ab_99547_CCTCCTGAATGCCTAATATCAGCA	0.549653826	0.020045646	-0.284324211
ab_99547_CCTCCTGACATACCAAGCTAACGA	0.729424632	0.031832843	-0.401642329
ab_99547_CCTCCTGACCGACAACAACAACCA	0.588569269	0.175485054	-0.217529175
ab_99547_CCTCCTGACCGTGAGAATCATTCC	0.492176097	0.107187346	-0.143585996
ab_99547_CCTCCTGACCTAATCCACACAGAA	0.455267823	0.300090986	-0.158251344

ab_99547_CCTCCTGACGCATACAGTGTTCTA	0.354166439	0.102839895	-0.261650405
ab_99547_CCTCCTGACGCTGATCAGCCATGC	0.456861714	0.072383781	-0.420726529
ab_99547_CCTCCTGACGGATTGCCCGTGAGA	0.563647353	0.12734293	-0.21549672
ab_99547_CCTCCTGACTGGCATAGGAGAACA	0.579410525	0.180114655	-0.128172384
ab_99547_CCTCCTGAGTCGTAGAGCTCGGTA	0.474014402	0.068948899	-0.39524301
ab_99547_CCTCCTGAGTCGTAGATTCACGCA	0.649875009	0.087006155	-0.326154928
ab_99547_CCTCCTGATGGAACAAGCTAACGA	0.510338022	0.054406808	-0.249471476
ab_99547_CCTCTATCAATCCGTCCCAGTTCA	0.324092796	0.064579258	-0.150267939
ab_99547_CCTCTATCACACAGAAATGCCTAA	0.687401974	-0.005453586	-0.299443357
ab_99547_CCTCTATCAGAGTCAATGGAACAA	0.380167813	0.028636959	-0.442151632
ab_99547_CCTCTATCAGTACAAGAGATCGCA	0.455268973	0.005823459	-0.291054327
ab_99547_CCTCTATCCAACCACAAGATGTAC	0.457988923	0.108072082	-0.116682591
ab_99547_CCTCTATCCCATCCTCAAGGACAC	0.499135975	0.185519287	-0.271933658
ab_99547_CCTCTATCCTCAATGAACGCTCGA	0.711911575	0.139124085	-0.327368478
ab_99547_CCTCTATCCTGGCATACATACCAA	0.295989616	0.089996816	-0.200313182
ab_99547_CCTCTATCGTACGCAAAGTACAAG	0.634765667	0.093017782	-0.271699475
ab_99547_CCTCTATCGTCTGTCACTAAGGTC	0.632473471	0.187251524	-0.215885344
ab_99547_CGAACTTAACTATGCACTGTAGCC	0.481452905	0.156609717	-0.231623759
ab_99547_CGAACTTAATAGCGACGTCGTAGA	0.446100781	0.319388233	-0.27561366
ab_99547_CGAACTTACAACCACACAGCGTTA	0.553637919	0.087522459	-0.29072339
ab_99547_CGAACTTACAATGGAAAACGCTTA	0.566739033	0.118342037	-0.236972582
ab_99547_CGAACTTACTGGCATAACTATGCA	0.673246339	0.15087256	-0.338407993
ab_99547_CGAACTTAGACTAGTAACTATGCA	0.37923071	0.057295774	-0.158526973
ab_99547_CGAACTTAGAGTTAGCGTACGCAA	0.34235172	0.164105339	-0.241704888
ab_99547_CGAACTTAGGAGAACACCTCCTGA	0.582008462	0.143679706	-0.224920025
ab_99547_CGAACTTAGTCTGTCAACAGCAGA	0.653505249	0.112901623	-0.251275214
ab_99547_CGAACTTATGGTGGTATTCACGCA	0.561412357	0.159141973	-0.255867847
ab_99547_CGACACACAAGGACACGGTGCGAA	0.513665374	0.001892402	-0.208877276
ab_99547_CGACACACAGATCGCACCGAAGTA	0.570380908	0.095337524	-0.330459373
ab_99547_CGACACACAGTGGTCACTAAGGTC	0.318951403	0.108276459	-0.206260181
ab_99547_CGACACACATTGGCTCTATCAGCA	0.547620442	0.081268924	-0.296654526
ab_99547_CGACACACCAAGACTAGAGTTAGC	0.648969583	-0.075009759	-0.252410139

ab_99547_CGACACACCAAGGAGCAAGACGGA	0.779446451	0.281862883	-0.137081687
ab_99547_CGACACACCAAGGAGCAGTCACTA	0.448429974	0.134468638	-0.315563576
ab_99547_CGACACACCCGAAGTAGCCACATA	0.72639136	0.09765218	-0.315858552
ab_99547_CGACACACCGACTGGAACAAGCTA	0.409421881	0.004866173	-0.387957404
ab_99547_CGACACACGATGAATCAGATCGCA	0.449984147	0.021604154	-0.314040978
ab_99547_CGACACACGCCAAGACCGAACTTA	0.688228834	0.100588709	-0.316745513
ab_99547_CGACACACGCTAACGAATTGGCTC	0.557500112	0.155829708	-0.353479684
ab_99547_CGACACACGGTGCGAACAAGGAGC	0.367197504	0.112024527	-0.199544912
ab_99547_CGACACACGGTGCGAACTAAGGTC	0.407840901	0.106301112	-0.354916612
ab_99547_CGACACACGTACGCAAACAGCAGA	0.690254009	0.048571775	-0.34695659
ab_99547_CGACACACGTACGCAAATCCTGTA	0.406707792	0.113490043	-0.3025374
ab_99547_CGACACACGTCTGTCAACACAGAA	0.481700957	0.208541007	-0.393955833
ab_99547_CGACACACTAGGATGAAAGGTACA	0.344810303	0.099394399	-0.024772266
ab_99547_CGACTGGAAAACATCGAACGTGAT	0.328085156	0.001446652	-0.341693526
ab_99547_CGACTGGAACATTGGCACTATGCA	0.5528897	0.137188301	-0.137001123
ab_99547_CGACTGGAATAGCGACTGAAGAGA	0.53428709	-0.004754458	-0.431695845
ab_99547_CGACTGGACATCAAGTCCATCCTC	0.645655324	0.136665633	-0.034964637
ab_99547_CGACTGGACTAAGGTCGCGAGTAA	0.44725569	0.170907611	-0.234312877
ab_99547_CGACTGGAGCTCGGTACAGATCTG	0.454994937	0.209895403	-0.196122075
ab_99547_CGACTGGAGGAGAACAAAGGACAC	0.169464742	0.218458329	-0.143628021
ab_99547_CGACTGGAGTCTGTCAGGAGAACA	0.485564983	0.129581626	-0.170173949
ab_99547_CGACTGGAGTGTTCTAGCGAGTAA	0.344094721	0.116847333	-0.287852837
ab_99547_CGACTGGATAGGATGAAGATGTAC	0.473958032	-0.040358515	-0.375833261
ab_99547_CGCATACAAACGCTTAGAACAGGC	0.398413409	0.173154976	-0.247312585
ab_99547_CGCATACAAAGAGATCAACAACCA	0.526765498	0.032965782	-0.293411085
ab_99547_CGCATACAACACGACCCCGTGAGA	0.578151371	0.137430833	-0.238914318
ab_99547_CGCATACAACACGACCCTGTAGCC	0.427940724	0.139362695	-0.23427222
ab_99547_CGCATACAACATTGGCATAGCGAC	0.633872921	0.133269276	-0.393651858
ab_99547_CGCATACAACATTGGCCTGGCATA	0.6484292	0.211557358	-0.229869904
ab_99547_CGCATACAAGTGGTCAAACAACCA	0.649669457	-0.048053337	-0.410593907
ab_99547_CGCATACACAACCACAAGTACAAG	0.354228986	0.209358967	-0.250125085
ab_99547_CGCATACACAAGACTAACAAGCTA	0.623812331	0.166554021	-0.351700964

ab_99547_CGCATACACAATGGAAAACGTGAT	0.388831314	0.16244413	-0.296341059
ab_99547_CGCATACATATCAGCAACATTGGC	0.383256873	0.118949768	-0.392344481
ab_99547_CGCATACATCCGTCTAAATCCGTC	0.483962573	0.06707914	-0.29106642
ab_99547_CGCATACATTCACGCAAAACATCG	0.344347759	0.152047233	-0.256371193
ab_99547_CGCTGATCAACTCACCAAGGTACA	0.665461025	0.138605115	-0.235013554
ab_99547_CGCTGATCAATCCGTCTCTTCACA	0.448373616	0.248741409	-0.300426578
ab_99547_CGCTGATCACACAGAAACCTCCAA	0.596670219	0.26121134	-0.287808242
ab_99547_CGCTGATCAGCAGGAACTCAATGA	0.501228575	0.150977813	-0.320638743
ab_99547_CGCTGATCAGCCATGCCACTTCGA	0.65981201	0.067674315	-0.052983248
ab_99547_CGCTGATCCAGCGTTAACGCTCGA	0.567675492	0.132467458	-0.307688439
ab_99547_CGCTGATCCCTAATCCTAGGATGA	0.519013902	0.054551413	-0.279466786
ab_99547_CGCTGATCCGACACACGAGTTAGC	0.824712118	0.150251033	-0.276181423
ab_99547_CGCTGATCCGCATACACGACTGGA	0.626423631	0.045075855	-0.368549599
ab_99547_CGCTGATCCGCTGATCCACTTCGA	0.537099009	0.088224781	-0.09268101
ab_99547_CGCTGATCGCGAGTAATCTTCACA	0.3690799	0.084999231	-0.215761917
ab_99547_CGCTGATCGTCTGTCAAACAACCA	0.510749803	0.05620376	-0.400035356
ab_99547_CGCTGATCTGAAGAGATATCAGCA	0.451516895	0.156390361	-0.277290415
ab_99547_CGGATTGCAATGTTGCGCTAACGA	0.687715852	0.189259548	-0.154636617
ab_99547_CGGATTGCACATTGGCCACTTCGA	0.582942794	-0.019644567	-0.395590633
ab_99547_CGGATTGCAGAGTCAAATCCTGTA	0.436759054	0.075950193	-0.263880627
ab_99547_CGGATTGCAGTACAAGAGCCATGC	0.590873589	-0.018335954	-0.359745043
ab_99547_CGGATTGCCATACCAAAAGGACAC	0.688982772	0.094876197	-0.296642538
ab_99547_CGGATTGCCCAGTTCAGAACAGGC	0.446277279	0.03468659	-0.21685932
ab_99547_CGGATTGCCCATCCTCACACGACC	0.53284133	0.129220651	-0.165207597
ab_99547_CGGATTGCCCGAAGTAGGAGAACA	0.364381744	0.162587549	-0.365666683
ab_99547_CGGATTGCCTGAGCCAGATGAATC	0.498462083	0.2289846	-0.332673943
ab_99547_CGGATTGCGACTAGTAGACAGTGC	0.51148833	0.217997269	-0.156326186
ab_99547_CGGATTGCGCTAACGAACAGATTC	0.518714594	-0.009929349	-0.345458453
ab_99547_CGGATTGCGGAGAACAAGTACAAG	0.518338954	0.184498924	-0.270176224
ab_99547_CGGATTGCTAGGATGAAGCAGGAA	0.315227968	0.074511084	-0.319961417
ab_99547_CGGATTGCTATCAGCAAAACATCG	0.473851145	0.098335254	-0.278887163
ab_99547_CTAAGGTCAAACATCGGCTAACGA	0.498979636	0.092997401	-0.238159577

ab_99547_CTAAGGTCAACCGAGACGGATTGC	0.366911514	0.139566602	-0.336802999
ab_99547_CTAAGGTCAACGCTTACCGACAAC	0.550574126	0.145972139	-0.183643238
ab_99547_CTAAGGTCAAGGACACAACGCTTA	0.454522429	0.065445245	-0.32362092
ab_99547_CTAAGGTCACACGACCCAAGGAGC	0.695827822	0.172828061	-0.376783465
ab_99547_CTAAGGTCACCACTGTGTCTGTCA	0.459878744	0.09100847	-0.349578954
ab_99547_CTAAGGTCACCTCCAAAGTGGTCA	0.438877139	0.112723414	-0.303498811
ab_99547_CTAAGGTCAGATGTACAGTCACTA	0.475949899	0.292382801	-0.288580514
ab_99547_CTAAGGTCAGTACAAGTGGCTTCA	0.670465695	0.178335284	-0.218727491
ab_99547_CTAAGGTCATTGGCTCAAGAGATC	0.575769347	0.106959678	-0.280187188
ab_99547_CTAAGGTCCAAGGAGCAACGCTTA	0.499993951	0.059888788	-0.145289133
ab_99547_CTAAGGTCCAAGGAGCCACCTTAC	0.537404323	0.217411138	-0.267439686
ab_99547_CTAAGGTCCACCTTACTGGAACAA	0.539118065	0.090622031	-0.35040143
ab_99547_CTAAGGTCCATACCAAACTATGCA	0.503961091	0.168481656	-0.225683302
ab_99547_CTAAGGTCCCGTGAGAAGCACCTC	0.501579802	0.102763153	-0.270224438
ab_99547_CTAAGGTCCGGATTGCGTACGCAA	0.388126905	0.182973555	-0.314921784
ab_99547_CTAAGGTCCTAAGGTCAATCCGTC	0.345378733	0.049620138	-0.354707316
ab_99547_CTAAGGTCGAACAGGCAATGTTGC	0.509245106	0.204690422	-0.076192178
ab_99547_CTAAGGTCGATAGACAGTCTGTCA	0.50939735	0.225174663	-0.273461062
ab_99547_CTAAGGTCGATGAATCAGTCACTA	0.407268969	0.083423371	-0.40028902
ab_99547_CTAAGGTCGCCAAGACAACGTGAT	0.454769674	0.219371255	-0.370090438
ab_99547_CTAAGGTCGCCACATAAACGTGAT	0.603837194	0.039285413	-0.246218288
ab_99547_CTAAGGTCGGAGAACAAACCGAGA	0.548476379	0.065084141	-0.301901494
ab_99547_CTAAGGTCTAGGATGACATACCAA	0.61056058	0.122386261	-0.299031464
ab_99547_CTCAATGAAATGTTGCGTGTTCTA	0.48544318	0.028607206	-0.295738037
ab_99547_CTCAATGAACCTCCAACTGTAGCC	0.729658822	0.23930252	-0.16282037
ab_99547_CTCAATGAACCTCCAATCTTCACA	0.31959921	0.197239623	-0.27246492
ab_99547_CTCAATGAAGATCGCACCGAAGTA	0.210769197	-0.046966768	-0.388134786
ab_99547_CTCAATGAAGATGTACCTGGCATA	0.55583381	0.136225801	-0.08763247
ab_99547_CTCAATGAATTGGCTCAAGAGATC	0.381536123	0.08577668	-0.29349255
ab_99547_CTCAATGACAAGGAGCCTGTAGCC	0.325966667	0.024625902	-0.324198143
ab_99547_CTCAATGACCTAATCCAAACATCG	0.371823526	0.162368637	-0.313569867
ab_99547_CTCAATGACCTCTATCTTCACGCA	0.684203117	0.10841263	-0.252503779

ab_99547_CTCAATGACGAACTTAGAGCTGAA	0.446290885	0.106449965	-0.31983155
ab_99547_CTCAATGACTGTAGCCGCCACATA	0.363148099	0.167994855	-0.18536992
ab_99547_CTCAATGAGAGTTAGCCGGATTGC	0.540490977	0.019839631	-0.375476828
ab_99547_CTCAATGAGCTAACGAAAGACGGA	0.351660949	0.143841205	-0.339660184
ab_99547_CTGAGCCAAACTCACCAACGCTTA	0.579303354	-0.081003175	-0.384109543
ab_99547_CTGAGCCAACCTCCAACACTTCGA	0.41485064	0.198727487	-0.249750336
ab_99547_CTGAGCCAAGATCGCAACATTGGC	0.475498365	0.145039259	-0.128838725
ab_99547_CTGAGCCAATTGAGGACGACTGGA	0.500253333	0.184051315	-0.192732059
ab_99547_CTGAGCCACAATGGAAGCCACATA	0.56177683	0.136080869	-0.225273199
ab_99547_CTGAGCCACGAACTTAACGCTCGA	0.486104117	0.062143868	-0.346661512
ab_99547_CTGAGCCAGCCAAGACCTGAGCCA	0.374084926	0.043697804	-0.130115649
ab_99547_CTGAGCCAGCCACATAAGCACCTC	0.38277575	0.065367858	-0.214522144
ab_99547_CTGAGCCAGTGTTCTAATCCTGTA	0.420319001	0.081131784	-0.21029633
ab_99547_CTGGCATAACAAGCTACAACCACA	0.376601	0.099939036	-0.239674826
ab_99547_CTGGCATAACAAGCTACCTCCTGA	0.540599752	0.177773521	-0.334182517
ab_99547_CTGGCATAAGATGTACACAGATTC	0.522064637	0.137762692	-0.319295376
ab_99547_CTGGCATACCAGTTCACAGCGTTA	0.479346725	0.154641146	-0.233356914
ab_99547_CTGGCATACCTAATCCCTGGCATA	0.47633446	0.148538521	-0.417381336
ab_99547_CTGGCATAGTCTGTCAGGAGAACA	0.427687395	0.100453364	-0.164553523
ab_99547_CTGTAGCCAAGAGATCCTCAATGA	0.439681577	0.157544953	-0.178226217
ab_99547_CTGTAGCCACAGCAGATGGAACAA	0.400858963	0.155262095	-0.282614138
ab_99547_CTGTAGCCAGAGTCAAATTGGCTC	0.54827257	0.061818771	-0.278386775
ab_99547_CTGTAGCCAGAGTCAAGCCACATA	0.501415072	0.080089645	-0.30758459
ab_99547_CTGTAGCCCAAGGAGCCGACACAC	0.223224826	-0.020028485	-0.388491499
ab_99547_CTGTAGCCCAAGGAGCGTACGCAA	0.479764064	0.025004072	-0.202550944
ab_99547_CTGTAGCCCCTCTATCATTGGCTC	0.484473444	0.137310952	-0.239897874
ab_99547_CTGTAGCCCTCAATGATCCGTCTA	0.567272229	0.112798664	-0.330339146
ab_99547_CTGTAGCCTGAAGAGAGCCACATA	0.480021842	0.131676929	-0.328618658
ab_99547_CTGTAGCCTGGAACAACTGTAGCC	0.606383233	0.23739977	-0.184248086
ab_99547_GAACAGGCACAGATTCAGATCGCA	0.582806467	0.136149313	-0.313583117
ab_99547_GAACAGGCACAGATTCTGGCTTCA	0.53083199	0.086270928	-0.278462624
ab_99547_GAACAGGCACCACTGTACAGCAGA	0.514033633	0.166561617	-0.339848494

ab_99547_GAACAGGCAGATCGCACACCTTAC	0.435222633	0.185072063	-0.327678339
ab_99547_GAACAGGCATTGAGGAAACCGAGA	0.538087057	0.128131927	-0.296726085
ab_99547_GAACAGGCATTGAGGAGACAGTGC	0.430667104	0.12011387	-0.251346514
ab_99547_GAACAGGCCAAGACTAGAACAGGC	0.712482904	0.140509679	-0.109872981
ab_99547_GAACAGGCCAAGACTAGCGAGTAA	0.322164603	0.070983207	-0.256133282
ab_99547_GAACAGGCCCTAATCCGGTGCGAA	0.309739507	0.113705806	-0.286460016
ab_99547_GAACAGGCCCTCCTGAACGTATCA	0.550550435	0.242549877	-0.290314189
ab_99547_GAACAGGCGACAGTGCAGCACCTC	0.638872307	0.229293486	-0.126188152
ab_99547_GAACAGGCGCCACATAACAAGCTA	0.758345932	0.14768956	-0.367896485
ab_99547_GAACAGGCTATCAGCAAACGTGAT	0.700600978	0.019138961	-0.273336365
ab_99547_GAATCTGAAACAACCAAGCAGGAA	0.616861347	0.233458087	-0.331240803
ab_99547_GAATCTGAACTATGCAATAGCGAC	0.65905519	0.14312224	-0.358718344
ab_99547_GAATCTGAAGAGTCAAAGTACAAG	0.682333702	0.060909605	-0.235904195
ab_99547_GAATCTGAAGATCGCAACAGATTC	0.405187372	0.228773507	-0.334554955
ab_99547_GAATCTGAAGCAGGAAAAGGTACA	0.864514689	0.232446943	-0.280914828
ab_99547_GAATCTGAATCATTCCAACAACCA	0.677260685	0.141314224	-0.160413449
ab_99547_GAATCTGAATCATTCCGATAGACA	0.497857985	0.093172624	-0.403387791
ab_99547_GAATCTGACACCTTACGTACGCAA	0.285442235	0.145458242	-0.23157985
ab_99547_GAATCTGACACTTCGACAAGACTA	0.411196533	0.148111309	-0.275509182
ab_99547_GAATCTGACCAGTTCACCGAAGTA	0.445445391	0.088086964	-0.322948605
ab_99547_GAATCTGACCGAAGTAAACAACCA	0.457264528	0.25640575	-0.31329189
ab_99547_GAATCTGAGAATCTGAACGCTCGA	0.708940895	0.080110281	-0.269859225
ab_99547_GAATCTGATCTTCACAGGAGAACA	0.363499765	0.126082675	-0.190965366
ab_99547_GACAGTGCAACTCACCCTAAGGTC	0.753478045	0.160252773	-0.091635581
ab_99547_GACAGTGCAAGACGGAAGATGTAC	0.403932328	0.127699104	-0.149917559
ab_99547_GACAGTGCAAGGACACCCATCCTC	0.300351766	-0.010914719	-0.250101292
ab_99547_GACAGTGCACGCTCGACACCTTAC	0.668191079	0.101646084	-0.233034657
ab_99547_GACAGTGCAGCACCTCATTGGCTC	0.67814642	0.149867709	-0.159852157
ab_99547_GACAGTGCAGCCATGCGTGTTCTA	0.608148503	0.167483736	-0.341038884
ab_99547_GACAGTGCAGGCTAACAAGAGATC	0.552384269	-0.043474793	-0.339553367
ab_99547_GACAGTGCATGCCTAACACTTCGA	0.450353539	0.048448323	-0.343605442
ab_99547_GACAGTGCATTGAGGACGCTGATC	0.399872787	0.152805816	-0.358200716

ab_99547_GACAGTGCCCGTGAGAAGCACCTC	0.368369699	0.068263322	-0.367106407
ab_99547_GACAGTGCGCCAAGACCAATGGAA	0.463261086	0.173879522	-0.350886621
ab_99547_GACAGTGCTGGTGGTATCCGTCTA	0.296158065	0.172529541	-0.329362011
ab_99547_GACTAGTAAAGAGATCACAGCAGA	0.468917114	0.101858794	-0.156055422
ab_99547_GACTAGTAAATGTTGCAACAACCA	0.265551812	0.122743338	-0.353515574
ab_99547_GACTAGTAACAGCAGAGAGTTAGC	0.482723769	0.145565026	-0.262453379
ab_99547_GACTAGTAACATTGGCTGGCTTCA	0.603022129	0.050285233	-0.399833149
ab_99547_GACTAGTAATCATTCCCAGATCTG	0.306335935	0.183446192	-0.21567356
ab_99547_GACTAGTACACTTCGAGAGTTAGC	0.423431679	0.105843165	-0.257610973
ab_99547_GACTAGTACAGATCTGCTGAGCCA	0.554986252	0.076810091	-0.265290472
ab_99547_GACTAGTACCATCCTCAAACATCG	0.344314078	0.22808082	-0.17679006
ab_99547_GACTAGTACCGACAACAACCGAGA	0.264746936	0.155048405	-0.222628747
ab_99547_GACTAGTACGGATTGCCGCATACA	0.467298185	0.191977095	-0.216014658
ab_99547_GACTAGTACTGTAGCCCCTCTATC	0.58147838	0.070542061	-0.305072731
ab_99547_GACTAGTAGCCAAGACAGATCGCA	0.292087918	0.261176648	-0.210621295
ab_99547_GACTAGTAGCCAAGACGGAGAACA	0.351829412	0.147940173	-0.191718539
ab_99547_GACTAGTAGGTGCGAAAAGAGATC	0.645093612	0.09231538	-0.301474926
ab_99547_GACTAGTAGGTGCGAAACGTATCA	0.455696592	0.465340438	-0.142029443
ab_99547_GACTAGTATGGAACAACAACCACA	0.342013665	0.1445177	-0.23667191
ab_99547_GACTAGTATGGTGGTAAGTCACTA	0.547997064	0.278925204	-0.358494834
ab_99547_GAGCTGAAAACAACCAAGATGTAC	0.637982112	0.17437545	-0.32880489
ab_99547_GAGCTGAAACAGATTCCAAGGAGC	0.387888127	0.069367635	-0.165962162
ab_99547_GAGCTGAAACTATGCAAACGTGAT	0.447214838	0.086555897	-0.289226284
ab_99547_GAGCTGAAAGATCGCAACATTGGC	0.488327641	0.058440712	-0.403931169
ab_99547_GAGCTGAAAGCCATGCCGACTGGA	0.709653927	0.194717113	-0.323022035
ab_99547_GAGCTGAAATTGAGGACGAACTTA	0.241043247	0.139255952	-0.27572947
ab_99547_GAGCTGAACAGCGTTAAACGCTTA	0.553225425	0.152970701	-0.313652788
ab_99547_GAGCTGAACATCAAGTCCAGTTCA	0.422242495	0.090167355	-0.207352807
ab_99547_GAGCTGAACCAGTTCAGTACGCAA	0.350443671	0.094992207	-0.23959543
ab_99547_GAGCTGAACCGTGAGAGTACGCAA	0.432580863	0.064135508	-0.348969289
ab_99547_GAGCTGAACTGTAGCCAGATGTAC	0.318485869	0.070938527	-0.246904048
ab_99547_GAGCTGAAGACAGTGCACAGATTC	0.263728896	0.147258614	-0.203397006

ab_99547_GAGCTGAAGCTCGGTAGAGTTAGC	0.496399098	0.066046703	-0.285957579
ab_99547_GAGTTAGCACACGACCCCGACAAC	0.331221332	0.070067035	-0.260415444
ab_99547_GAGTTAGCACGCTCGACGAACTTA	0.382105717	0.191866632	-0.205191405
ab_99547_GAGTTAGCAGAGTCAAAACCGAGA	0.402300333	0.073013759	-0.235390713
ab_99547_GAGTTAGCCCATCCTCCTCAATGA	0.490729319	-0.017454888	-0.262517419
ab_99547_GAGTTAGCCGCTGATCGAACAGGC	0.383673587	0.215917201	-0.2392375
ab_99547_GAGTTAGCCTAAGGTCGAACAGGC	0.56021929	0.220109182	-0.321703214
ab_99547_GAGTTAGCGAATCTGAAACGTGAT	0.543479334	-0.005107999	-0.217208111
ab_99547_GAGTTAGCGACTAGTATCTTCACA	0.434828109	0.146665071	-0.272769708
ab_99547_GAGTTAGCGCTAACGACGACACAC	0.758380713	0.158949178	-0.218597403
ab_99547_GAGTTAGCGGAGAACAACCTCCAA	0.743776106	0.094063115	-0.263565242
ab_99547_GAGTTAGCGTCTGTCAGGAGAACA	0.236140928	0.024802464	-0.314485836
ab_99547_GAGTTAGCGTGTTCTACCGAAGTA	0.661538061	0.041839689	-0.361700714
ab_99547_GAGTTAGCTGAAGAGAAACCGAGA	0.602355691	0.199520324	-0.19257404
ab_99547_GAGTTAGCTGGCTTCAGCCAAGAC	0.671322468	0.157033874	-0.065926385
ab_99547_GATAGACAAACCGAGAGCGAGTAA	0.627860037	0.099994705	-0.375313414
ab_99547_GATAGACAAACGTGATAATCCGTC	0.430084362	0.158437303	-0.322788419
ab_99547_GATAGACAACAAGCTATCTTCACA	0.449874991	0.111196087	-0.108661632
ab_99547_GATAGACAACACGACCAACTCACC	0.508398752	0.065416603	-0.294304214
ab_99547_GATAGACAACCTCCAAAGCAGGAA	0.438483792	0.211234603	-0.158028632
ab_99547_GATAGACAAGAGTCAAAACGCTTA	0.816624319	0.113046681	-0.330632998
ab_99547_GATAGACAAGATCGCAGATAGACA	0.487894922	0.019670529	-0.291602647
ab_99547_GATAGACAATCCTGTAGGAGAACA	0.616770175	0.111116648	-0.426981326
ab_99547_GATAGACAATTGGCTCAAGAGATC	0.470876802	0.177412895	-0.314532173
ab_99547_GATAGACAATTGGCTCCCTCTATC	0.447810104	0.069840131	-0.380277721
ab_99547_GATAGACACAACCACACTCAATGA	0.541465973	0.046743077	-0.323896773
ab_99547_GATAGACACAAGGAGCATAGCGAC	0.22978802	-0.078129053	-0.364107294
ab_99547_GATAGACACACCTTACATTGAGGA	0.515424439	0.073889217	-0.180342203
ab_99547_GATAGACACATACCAAGCCACATA	0.445284062	0.167785643	-0.235348141
ab_99547_GATAGACACGACTGGACCGACAAC	0.372371102	0.156877246	-0.269060075
ab_99547_GATAGACAGACAGTGCCGGATTGC	0.290296599	0.15447448	-0.209781282
ab_99547_GATAGACATGGTGGTAGAATCTGA	0.467533851	0.23055949	-0.241293697

ab_99547_GATGAATCAAGGACACCAGATCTG	0.488610615	0.090125589	-0.296297052
ab_99547_GATGAATCACACGACCACGCTCGA	0.289003237	0.120556092	-0.319370889
ab_99547_GATGAATCACAGATTCCGAACTTA	0.374486185	0.03898353	-0.390393771
ab_99547_GATGAATCCATACCAATGGAACAA	0.698289388	0.16883111	-0.096250049
ab_99547_GATGAATCCCTAATCCCAGCGTTA	0.64313605	0.054710746	-0.346423822
ab_99547_GATGAATCCGAACTTATTCACGCA	0.468357212	-0.040551176	-0.285221984
ab_99547_GATGAATCCGGATTGCAGTGGTCA	0.869082669	0.033819435	-0.337284107
ab_99547_GATGAATCCTGGCATATGGAACAA	0.601642464	0.169231338	-0.292954979
ab_99547_GATGAATCGAACAGGCTATCAGCA	0.613422583	0.015239267	-0.272942933
ab_99547_GATGAATCGCTCGGTAAGATGTAC	0.474927987	0.229456588	-0.197589375
ab_99547_GATGAATCGGAGAACACGAACTTA	0.734861917	0.152798318	-0.396168206
ab_99547_GCCAAGACAAGAGATCGTCTGTCA	0.413096702	0.093283342	-0.341109735
ab_99547_GCCAAGACACACAGAAAGTCACTA	0.576980992	0.040031115	-0.292646035
ab_99547_GCCAAGACACACAGAACACTTCGA	0.550146269	0.151033774	-0.306847719
ab_99547_GCCAAGACAGTCACTAGGTGCGAA	0.590799392	0.018770621	-0.351345362
ab_99547_GCCAAGACCACTTCGAACGCTCGA	0.560199186	0.093489747	-0.256977169
ab_99547_GCCAAGACCATCAAGTCAAGGAGC	0.509696454	0.157541854	-0.266158334
ab_99547_GCCAAGACCCTCCTGAGTCTGTCA	0.526159555	0.03302164	-0.422936326
ab_99547_GCCAAGACCCTCCTGAGTGTTCTA	0.541652022	0.126047668	-0.222922903
ab_99547_GCCAAGACCTGAGCCAGACTAGTA	0.268265126	0.254119414	-0.167310249
ab_99547_GCCAAGACGACAGTGCATTGGCTC	0.70436545	0.131616922	-0.282781346
ab_99547_GCCAAGACGTACGCAACACCTTAC	0.767467021	0.053488652	-0.24849983
ab_99547_GCCAAGACTGAAGAGAAACTCACC	0.477924512	0.005001484	-0.328798657
ab_99547_GCCACATAACAGATTCCCAGTTCA	0.432859887	0.168008336	-0.280281572
ab_99547_GCCACATAACAGCAGAACCACTGT	0.35764516	0.139020673	-0.210840067
ab_99547_GCCACATAACCACTGTAGCACCTC	0.427168898	0.046278484	-0.215320353
ab_99547_GCCACATAAGTCACTATTCACGCA	0.341949479	0.120130498	-0.271002551
ab_99547_GCCACATAAGTGGTCAGCTCGGTA	0.579103425	0.11518047	-0.208099441
ab_99547_GCCACATAATCATTCCGATAGACA	0.452717652	0.163276918	-0.254899357
ab_99547_GCCACATACAACCACACTAAGGTC	0.395748402	0.006786445	-0.209966703
ab_99547_GCCACATAGAATCTGAAGATCGCA	0.588441868	0.084909677	-0.227469808
ab_99547_GCCACATAGTACGCAACCGTGAGA	0.315494091	0.137604852	-0.145072168

ab_99547_GCCACATATAGGATGACGGATTGC	0.586843258	0.147710712	-0.318869164
ab_99547_GCCACATATCTTCACAAACGCTTA	0.462649655	0.089464586	-0.348803086
ab_99547_GCGAGTAAAAGGACACGAGTTAGC	0.351246514	0.222817429	-0.271318698
ab_99547_GCGAGTAAAAGGTACAGACTAGTA	0.432279409	0.028517731	-0.326738426
ab_99547_GCGAGTAAAATGTTGCTAGGATGA	0.621547556	0.081111079	-0.275461766
ab_99547_GCGAGTAAACACAGAAGCCACATA	0.420815545	0.078574546	-0.241558304
ab_99547_GCGAGTAAACAGCAGAGATAGACA	0.59866279	0.180639451	-0.250426959
ab_99547_GCGAGTAACAACCACAAGATGTAC	0.372854745	0.091654818	-0.315652856
ab_99547_GCGAGTAACAAGACTAGTGTTCTA	0.448263219	0.031280623	-0.118060889
ab_99547_GCGAGTAACAGCGTTAGCTCGGTA	0.426938494	0.167646125	-0.423200748
ab_99547_GCGAGTAACCTAATCCATCCTGTA	0.421047908	-0.038136875	-0.324126022
ab_99547_GCGAGTAACGACTGGAGTGTTCTA	0.538613469	0.034896137	-0.132934363
ab_99547_GCGAGTAACTAAGGTCACAGATTC	0.434178265	0.113311869	-0.317925898
ab_99547_GCGAGTAAGCTCGGTAAGCAGGAA	0.4355571	0.177731204	-0.187841288
ab_99547_GCGAGTAAGTCTGTCAACGCTCGA	0.698080985	0.064634284	-0.386860951
ab_99547_GCGAGTAATAGGATGACGCTGATC	0.445210575	0.246735586	-0.221817547
ab_99547_GCTAACGAAACAACCAGGAGAACA	0.674746615	0.171030606	-0.122627467
ab_99547_GCTAACGAACAAGCTAAATCCGTC	0.604366307	0.116229625	-0.328205471
ab_99547_GCTAACGACAACCACAACCTCCAA	0.430461188	0.098272524	-0.266678635
ab_99547_GCTAACGACAGATCTGACTATGCA	0.290419475	0.166284743	-0.273851137
ab_99547_GCTAACGACGACTGGACACTTCGA	0.490024302	0.022800395	-0.359196657
ab_99547_GCTAACGACGCTGATCCTGTAGCC	0.547432593	0.16292982	-0.296920147
ab_99547_GCTAACGACTGGCATACAGATCTG	0.511712888	0.192469124	-0.315519881
ab_99547_GCTAACGAGCCACATAAGTACAAG	0.517417259	0.118330974	-0.343936698
ab_99547_GCTAACGAGCCACATACAAGACTA	0.650877626	0.143032342	-0.291704101
ab_99547_GCTAACGAGCTAACGAACCTCCAA	0.791774282	-0.104428489	-0.371904374
ab_99547_GCTAACGATCCGTCTAAACTCACC	0.517828092	0.129686804	-0.147206183
ab_99547_GCTCGGTAACAGCAGAGCTCGGTA	0.695973902	0.057135088	-0.315608122
ab_99547_GCTCGGTAACATTGGCATAGCGAC	0.355558312	0.018528346	-0.352101154
ab_99547_GCTCGGTAATAGCGACCCGACAAC	0.648896012	0.272345721	-0.166297312
ab_99547_GCTCGGTACATCAAGTACCTCCAA	0.466246995	0.192765089	-0.270865607
ab_99547_GCTCGGTACCGACAACGAACAGGC	0.72233599	0.148168257	-0.437109237

ab_99547_GCTCGGTAGAACAGGCACAAGCTA	0.532370081	0.114782305	-0.046036517
ab_99547_GCTCGGTATCTTCACACAAGACTA	0.522034593	0.054111797	-0.278591041
ab_99547_GCTCGGTATGGCTTCAGAGCTGAA	0.038911407	-0.001330697	-0.375506412
ab_99547_GCTCGGTATGGTGGTAGAATCTGA	0.744437799	0.115348142	-0.360225261
ab_99547_GGAGAACAAACAACCACGGATTGC	0.448773714	-0.018350043	-0.404149035
ab_99547_GGAGAACAAACGTGATCGACACAC	0.360514963	0.014287109	-0.368043683
ab_99547_GGAGAACAAAGGACACAATGTTGC	0.505013715	0.22308533	-0.196240982
ab_99547_GGAGAACAACACAGAACTAAGGTC	0.406455681	0.13314196	-0.418262298
ab_99547_GGAGAACAAGATGTACGGTGCGAA	0.404578709	0.135793442	-0.366456359
ab_99547_GGAGAACAATTGAGGATGGAACAA	0.455379605	0.031781204	-0.293617094
ab_99547_GGAGAACACATCAAGTAAGGTACA	0.411510634	0.032573657	-0.390151505
ab_99547_GGAGAACACCAGTTCACCAGTTCA	0.568293756	0.075775182	-0.428540231
ab_99547_GGAGAACACTCAATGAGCCACATA	0.482215477	0.023366248	-0.391180852
ab_99547_GGAGAACAGCTCGGTAAAACATCG	0.538948185	0.107649376	-0.143008449
ab_99547_GGAGAACATCTTCACAGTCGTAGA	0.42211998	0.13839515	-0.33281429
ab_99547_GGAGAACATGAAGAGAGAGCTGAA	0.340169224	0.142761383	-0.361798493
ab_99547_GGAGAACATTCACGCAGCCAAGAC	0.447236737	0.112539549	-0.307856162
ab_99547_GGTGCGAAAAGACGGACCTAATCC	0.476437803	0.122233013	-0.219102838
ab_99547_GGTGCGAAAATCCGTCAAGACGGA	0.61838478	0.086926543	-0.297190657
ab_99547_GGTGCGAAACATTGGCGCCACATA	0.64517281	0.244777782	-0.195058736
ab_99547_GGTGCGAAAGTGGTCAAATCCGTC	0.862912525	0.05700978	-0.386069269
ab_99547_GGTGCGAAATCCTGTACGACACAC	0.498188461	0.046878854	-0.328464679
ab_99547_GGTGCGAAATCCTGTAGTCGTAGA	0.570864672	0.15142731	-0.3470066
ab_99547_GGTGCGAACAAGACTACAGATCTG	0.579403683	0.1757418	-0.202936733
ab_99547_GGTGCGAACAATGGAAACGCTCGA	0.464526473	0.079817894	-0.342737917
ab_99547_GGTGCGAACAATGGAAGCTAACGA	0.821062109	0.087613438	-0.254113121
ab_99547_GGTGCGAACACTTCGAGAATCTGA	0.404889796	0.136550658	-0.223406494
ab_99547_GGTGCGAACCGTGAGAAACAACCA	0.588492866	0.00250815	-0.352397792
ab_99547_GGTGCGAAGACAGTGCCTAAGGTC	0.491069686	0.102662211	-0.229677526
ab_99547_GGTGCGAAGACTAGTACAACCACA	0.471928807	0.142073151	-0.266244436
ab_99547_GGTGCGAAGATGAATCGTGTTCTA	0.647411514	0.126288183	-0.35847437
ab_99547_GGTGCGAAGTCTGTCATGAAGAGA	0.637598897	0.185100634	-0.243151059

ab_99547_GGTGCGAATTCACGCACGAACTTA	0.314153559	0.170874403	-0.326222886
ab_99547_GTACGCAAAACGCTTACAGCGTTA	0.62484547	0.279336563	-0.215861549
ab_99547_GTACGCAAAAGGTACACATACCAA	0.357418862	0.114144267	-0.237849732
ab_99547_GTACGCAAAGCAGGAATGGTGGTA	0.52415073	0.077269914	-0.166601358
ab_99547_GTACGCAAATTGGCTCACGTATCA	0.603815528	0.080310981	-0.282925747
ab_99547_GTACGCAACAAGGAGCATAGCGAC	0.572126997	0.152637062	-0.106427726
ab_99547_GTACGCAACAATGGAAAGTCACTA	0.369849103	0.154086926	-0.368605184
ab_99547_GTACGCAACATACCAAGAGTTAGC	0.549671218	0.142963863	-0.36319941
ab_99547_GTACGCAACCAGTTCAGAGCTGAA	0.544267122	0.232602996	-0.403065868
ab_99547_GTACGCAACCGAAGTAATCATTCC	0.516669103	0.262598344	-0.301330377
ab_99547_GTACGCAACCGTGAGACAGATCTG	0.458340778	0.181169668	-0.266877653
ab_99547_GTACGCAACCTAATCCGATGAATC	0.492654458	0.229467529	-0.391968286
ab_99547_GTACGCAACCTCTATCACACAGAA	0.626982261	0.117980371	-0.185047906
ab_99547_GTACGCAAGTACGCAAAGCACCTC	0.348100365	0.155863224	-0.252520871
ab_99547_GTACGCAAGTGTTCTAAATGTTGC	0.582133578	0.09597608	-0.277629236
ab_99547_GTACGCAATGGAACAACATCAAGT	0.600158051	0.07872075	-0.376056274
ab_99547_GTCGTAGAAACGTGATCCTCTATC	0.476099887	0.220845635	-0.168233649
ab_99547_GTCGTAGAACCACTGTAACCGAGA	0.4642192	0.166811832	-0.366725581
ab_99547_GTCGTAGAAGCACCTCAATGTTGC	0.473335319	0.204104036	-0.378014476
ab_99547_GTCGTAGAATCCTGTAAATCCGTC	0.566478775	0.134828899	-0.291088674
ab_99547_GTCGTAGACACCTTACCATACCAA	0.574120577	0.139938457	-0.148358265
ab_99547_GTCGTAGACACTTCGACCAGTTCA	0.502735269	0.089383311	-0.153460342
ab_99547_GTCGTAGACACTTCGACCTCTATC	0.501431621	0.112215883	-0.32434777
ab_99547_GTCGTAGACACTTCGATGAAGAGA	0.546886705	0.172376815	-0.226563509
ab_99547_GTCGTAGACCTCCTGAACTATGCA	0.542631682	0.064296852	-0.338841179
ab_99547_GTCGTAGACGCATACAAACTCACC	0.314821794	0.190093483	-0.172608147
ab_99547_GTCGTAGACGCTGATCGTACGCAA	0.519111714	0.257957445	-0.260714434
ab_99547_GTCGTAGACTAAGGTCATAGCGAC	0.478350892	0.120107933	-0.273471269
ab_99547_GTCGTAGAGATAGACACGAACTTA	0.531701498	-0.045760929	-0.189597108
ab_99547_GTCGTAGAGCCAAGACGCTCGGTA	0.693657916	0.096158193	-0.362023499
ab_99547_GTCGTAGATGGAACAATCCGTCTA	0.381874038	0.108601925	-0.098409761
ab_99547_GTCTGTCAAAGGACACCCATCCTC	0.564317415	0.248625125	-0.198835561

ab_99547_GTCTGTCAAGGCTAACCATCAAGT	0.459773088	0.228076715	-0.240363504
ab_99547_GTCTGTCAAGTACAAGATAGCGAC	0.652784584	-0.039698343	-0.354399866
ab_99547_GTCTGTCAAGTGGTCACCGAAGTA	0.440542244	0.060683001	-0.239484997
ab_99547_GTCTGTCACAGATCTGACCTCCAA	0.530444426	0.14918214	-0.303578921
ab_99547_GTCTGTCACCATCCTCAGGCTAAC	0.522425838	0.167298916	-0.324653846
ab_99547_GTCTGTCACGCATACAACAGATTC	0.351629735	0.104920615	-0.305283681
ab_99547_GTCTGTCACTGTAGCCCCATCCTC	0.415835867	0.079332935	-0.294682293
ab_99547_GTCTGTCATTCACGCACGCATACA	0.620527702	0.091746627	-0.338731068
ab_99547_GTGTTCTAAACGCTTAGCTCGGTA	0.518569847	0.237921455	-0.289392066
ab_99547_GTGTTCTAAACGCTTAGTCGTAGA	0.495580402	0.233149929	-0.288865026
ab_99547_GTGTTCTAAAGACGGATCCGTCTA	0.505678512	0.104926104	-0.288319259
ab_99547_GTGTTCTAAAGGACACAGTGGTCA	0.47085388	0.050557595	-0.271326541
ab_99547_GTGTTCTAACAGCAGATCCGTCTA	0.472146651	0.1220637	-0.136282364
ab_99547_GTGTTCTAACATTGGCTGGCTTCA	0.686872157	0.08692606	-0.1792103
ab_99547_GTGTTCTAACCTCCAACAGCGTTA	0.461507838	0.078504195	-0.215576839
ab_99547_GTGTTCTACACCTTACCGACACAC	0.557039027	0.180066056	-0.271425107
ab_99547_GTGTTCTACGACTGGAATCATTCC	0.663681908	0.028433599	-0.323965116
ab_99547_GTGTTCTAGCGAGTAACAAGACTA	0.493290839	0.136302102	-0.211132893
ab_99547_GTGTTCTATCTTCACACCATCCTC	0.441170098	0.162267471	-0.279618646
ab_99547_GTGTTCTATGAAGAGAATTGGCTC	0.39653922	0.161163721	-0.252680613
ab_99547_GTGTTCTATGGAACAACAAGACTA	0.480047414	0.091411574	-0.219925268
ab_99547_GTGTTCTATGGAACAACCTAATCC	0.426505519	0.254356492	-0.24988419
ab_99547_TAGGATGAAACCGAGACGACACAC	0.453064787	0.078180454	-0.075848563
ab_99547_TAGGATGAAAGGTACAGCGAGTAA	0.556981327	0.195358953	-0.368485559
ab_99547_TAGGATGAAATGTTGCAGATCGCA	0.37591745	0.202904187	-0.265283292
ab_99547_TAGGATGAAGGCTAACGAACAGGC	0.318775634	0.11034833	-0.331094324
ab_99547_TAGGATGACAGATCTGAAGGACAC	0.610970808	0.01320493	-0.388744425
ab_99547_TAGGATGACCTCTATCACACAGAA	0.323214313	0.123204583	-0.254760093
ab_99547_TAGGATGAGAACAGGCCGACTGGA	0.261918268	0.129302807	-0.3333939
ab_99547_TAGGATGAGAGCTGAACTGTAGCC	0.429931206	0.128450065	-0.387715386
ab_99547_TAGGATGAGCGAGTAACAACCACA	0.64216395	0.030536553	-0.385901215
ab_99547_TAGGATGATCTTCACATTCACGCA	0.446681283	0.11711558	-0.335616718

ab_99547_TATCAGCAAACCGAGAGGAGAACA	0.74968091	0.155255735	-0.226842596
ab_99547_TATCAGCAAAGGTACACCTAATCC	0.324587606	0.013388126	-0.27282422
ab_99547_TATCAGCAAATCCGTCATAGCGAC	0.567243936	-0.00933053	-0.280288789
ab_99547_TATCAGCAACATTGGCACCACTGT	0.472517737	0.016759266	-0.232745261
ab_99547_TATCAGCAAGCACCTCCTGGCATA	0.383004483	0.09739044	-0.296817527
ab_99547_TATCAGCACACTTCGAACGCTCGA	0.374269949	0.106284048	-0.168476167
ab_99547_TATCAGCACCGACAACATTGGCTC	0.573733855	0.144763854	-0.203030677
ab_99547_TATCAGCACCGTGAGAAGTCACTA	0.437690663	0.014596741	-0.229501081
ab_99547_TATCAGCACGACACACTCCGTCTA	0.52678284	0.109758369	-0.294096108
ab_99547_TATCAGCACGACTGGAAACAACCA	0.597035291	0.108215648	-0.398181743
ab_99547_TATCAGCAGAATCTGACCTCTATC	0.461920358	-0.074506994	-0.409322259
ab_99547_TATCAGCAGCTCGGTACAGATCTG	0.482580702	0.123073205	-0.108326525
ab_99547_TATCAGCATGGCTTCACGGATTGC	0.478047771	0.091569044	-0.397597959
ab_99547_TATCAGCATTCACGCAGAATCTGA	0.589330936	0.12187609	-0.396726351
ab_99547_TCCGTCTAAGCAGGAAAGATCGCA	0.465184057	0.080036129	-0.271354878
ab_99547_TCCGTCTAAGTACAAGTAGGATGA	0.470721394	0.127246412	-0.133514057
ab_99547_TCCGTCTACACCTTACAATCCGTC	0.492907544	0.159718237	-0.28691531
ab_99547_TCCGTCTACACCTTACACACGACC	0.79168865	0.148589999	-0.371071729
ab_99547_TCCGTCTACCGTGAGAGATAGACA	0.593660591	0.056548279	-0.318879296
ab_99547_TCCGTCTACCTCCTGAGATAGACA	0.44795712	0.104892116	-0.393636171
ab_99547_TCCGTCTACGACACACACTATGCA	0.510319944	0.143607152	-0.337789481
ab_99547_TCCGTCTAGTACGCAACGCTGATC	0.371243019	0.221157011	-0.241939468
ab_99547_TCTTCACAAACAACCACACTTCGA	0.670138317	0.180910982	-0.241726148
ab_99547_TCTTCACAAATGTTGCGCCACATA	0.662498495	0.165463497	-0.249245305
ab_99547_TCTTCACAAGATGTACCGAACTTA	0.410796887	0.183805911	-0.339368216
ab_99547_TCTTCACAATTGGCTCGACTAGTA	0.54315767	0.092457607	-0.21230359
ab_99547_TCTTCACACACTTCGATTCACGCA	0.555901434	0.297215424	-0.14116664
ab_99547_TCTTCACAGAATCTGAACCTCCAA	0.621149738	0.225982926	-0.281881897
ab_99547_TCTTCACAGCGAGTAACCGAAGTA	0.450208676	-0.018813852	-0.367468804
ab_99547_TCTTCACATCCGTCTACCATCCTC	0.53710864	0.130013673	-0.0621199
ab_99547_TGAAGAGAAACAACCACAACCACA	0.495220024	0.046517936	-0.385442478
ab_99547_TGAAGAGAAGATCGCAACTATGCA	0.56460166	0.104998644	-0.371879282

ab_99547_TGAAGAGAAGTACAAGGAGTTAGC	0.630077503	0.009788889	-0.38541251
ab_99547_TGAAGAGAAGTGGTCAACGCTCGA	0.363048913	0.039985647	-0.329913945
ab_99547_TGAAGAGAATCCTGTAAACCGAGA	0.324168115	0.014062832	-0.41580879
ab_99547_TGAAGAGACACCTTACAAGGACAC	0.368334851	0.101162055	-0.173042514
ab_99547_TGAAGAGACGAACTTAATCATTCC	0.458233754	0.149604072	-0.314413328
ab_99547_TGAAGAGACGGATTGCGCCACATA	0.470030712	0.207353689	-0.269970269
ab_99547_TGAAGAGAGCTAACGAACCACTGT	0.404164542	0.055637425	-0.350862777
ab_99547_TGAAGAGAGTACGCAAAGGCTAAC	0.526754862	0.220994596	-0.281803307
ab_99547_TGAAGAGATGGCTTCACCGTGAGA	0.533898469	0.092108139	-0.358114318
ab_99547_TGGAACAAAACAACCAGAATCTGA	0.391363746	0.1629599	-0.250728845
ab_99547_TGGAACAAAACGCTTAAAGACGGA	0.474793206	0.140829844	-0.126494668
ab_99547_TGGAACAAACATTGGCGTACGCAA	0.305038842	0.066710741	-0.289701128
ab_99547_TGGAACAAACTATGCAAGCAGGAA	0.434647683	0.183252531	-0.215065103
ab_99547_TGGAACAAAGAGTCAAGAGCTGAA	0.599349163	0.253470922	-0.362583334
ab_99547_TGGAACAAAGCCATGCAAGGACAC	0.602061916	0.114218191	-0.394452323
ab_99547_TGGAACAAAGTACAAGCTGGCATA	0.343450005	0.104206635	-0.382100876
ab_99547_TGGAACAAATAGCGACGCCAAGAC	0.583649193	0.148240779	-0.233856093
ab_99547_TGGAACAACATACCAAAACTCACC	0.527677053	0.109557397	-0.34284934
ab_99547_TGGAACAACTGTAGCCAGATCGCA	0.457308856	0.089841464	-0.261272642
ab_99547_TGGAACAAGACAGTGCCAGCGTTA	0.286364049	0.114256022	-0.261939035
ab_99547_TGGAACAAGCTAACGACCATCCTC	0.674045801	0.102916953	-0.396138797
ab_99547_TGGAACAATCTTCACAATTGGCTC	0.380010655	0.057536626	-0.372003097
ab_99547_TGGAACAATGGAACAAACAGATTC	0.394334156	0.083947973	-0.14410251
ab_99547_TGGCTTCAACACAGAAACAGCAGA	0.162876776	0.100898291	-0.285125064
ab_99547_TGGCTTCAAGGCTAACAACGTGAT	0.462891622	0.118422028	-0.238351939
ab_99547_TGGCTTCAATTGAGGAAGCCATGC	0.439025758	0.073604161	-0.330769058
ab_99547_TGGCTTCAGATGAATCGAGTTAGC	0.615991275	0.261721925	-0.177282992
ab_99547_TGGCTTCAGGTGCGAACGAACTTA	0.720503327	0.118140673	-0.31294408
ab_99547_TGGTGGTAACACGACCCTAAGGTC	0.539896341	0.270491794	-0.178641638
ab_99547_TGGTGGTAAGGCTAACCCGTGAGA	0.391716544	0.127704245	-0.273597191
ab_99547_TGGTGGTAAGTGGTCAAAGACGGA	0.42679114	0.164038494	-0.347669614
ab_99547_TGGTGGTAATCATTCCAAGAGATC	0.214003122	0.194358997	-0.102779613

ab_99547_TGGTGGTACAACCACAAAGAGATC	0.658065221	0.074006367	-0.308904786
ab_99547_TGGTGGTACAGCGTTAGCGAGTAA	0.531783728	0.122521277	-0.275663537
ab_99547_TGGTGGTACCGACAACCCATCCTC	0.516110026	0.084665311	-0.314780525
ab_99547_TGGTGGTACCTCTATCAGCACCTC	0.29942124	0.064473539	-0.171964035
ab_99547_TGGTGGTAGCCACATACTGAGCCA	0.489053589	0.16191837	-0.253725453
ab_99547_TGGTGGTAGCTCGGTAAGATGTAC	0.31196183	0.141629237	-0.316394729
ab_99547_TGGTGGTAGTACGCAAGATAGACA	0.442231258	0.174873653	-0.270845434
ab_99547_TGGTGGTAGTCGTAGACAACCACA	0.594571152	0.145893313	-0.277508701
ab_99547_TGGTGGTAGTCGTAGATAGGATGA	0.576455036	0.089196098	-0.33859764
ab_99547_TGGTGGTATATCAGCACCTCTATC	0.398212757	0.042498546	-0.305003044
ab_99547_TGGTGGTATCCGTCTAACAGATTC	0.461203497	0.208172279	-0.309774294
ab_99547_TGGTGGTATCCGTCTACGGATTGC	0.494630768	0.107583383	-0.36174796
ab_99547_TGGTGGTATCTTCACAATCCTGTA	0.381596901	0.242440329	-0.237314306
ab_99547_TGGTGGTATGGCTTCAAACAACCA	0.407328201	0.099511446	-0.297373357
ab_99547_TTCACGCAAACCGAGACCATCCTC	0.742031694	0.156017293	-0.286295966
ab_99547_TTCACGCAAAGACGGAACAAGCTA	0.618852478	0.287818412	-0.354703911
ab_99547_TTCACGCAAAGAGATCCATACCAA	0.55413446	0.192903776	-0.073793069
ab_99547_TTCACGCAAAGGACACATCATTCC	0.340199189	0.165266642	-0.181370571
ab_99547_TTCACGCAACGTATCAACACAGAA	0.433954717	0.120201672	-0.228292816
ab_99547_TTCACGCAATTGGCTCTCTTCACA	0.341542386	0.165995422	-0.142345615
ab_99547_TTCACGCACCGACAACAGTGGTCA	0.348436482	0.059984345	-0.255496865
ab_99547_TTCACGCACCTCCTGACCGACAAC	0.406482062	0.12620968	-0.233321299
ab_99547_TTCACGCACCTCTATCCCAGTTCA	0.545892205	0.045380425	-0.352565148
ab_99547_TTCACGCACGCATACACAATGGAA	0.488702747	0.057171311	-0.286236475
ab_99547_TTCACGCACGCTGATCACAAGCTA	0.643491254	0.015743474	-0.280559586
ab_99547_TTCACGCACGGATTGCAAGAGATC	0.477676157	0.146547303	-0.268028503
ab_99547_TTCACGCACTGAGCCAGCGAGTAA	0.318778388	-0.002785064	-0.231625881
ab_99547_TTCACGCAGACAGTGCCAAGACTA	0.435949284	0.167677451	-0.105800155
ab_99547_TTCACGCAGCTCGGTAAGCAGGAA	0.450124574	0.129647828	-0.216620088
ab_99547_TTCACGCATTCACGCACAGATCTG	0.290063093	0.085112897	-0.229645783
ab_99547_AAACATCGAGTGGTCACAAGGAGC	0.367667715	0.242365702	-0.42820042
ab_99547_AAACATCGCCGAAGTACAGATCTG	0.730086446	0.16971506	-0.423949746

ab_99547_AAACATCGCTCAATGAACGCTCGA	0.747070424	0.235368349	-0.290549349
ab_99547_AAACATCGGAGTTAGCACCACTGT	0.459004078	0.121738436	-0.286696254
ab_99547_AAACATCGTAGGATGAGTCGTAGA	0.584093342	-0.001817217	-0.346329466
ab_99547_AACAACCAACAGATTCACGTATCA	0.600291154	0.114829663	-0.237771465
ab_99547_AACAACCACAATGGAACTAAGGTC	0.757332648	0.054726604	-0.435357801
ab_99547_AACAACCACCTAATCCAACGTGAT	0.635873123	0.242808205	-0.48753696
ab_99547_AACAACCACGCATACAGCCAAGAC	1.036511182	0.173768029	-0.334824139
ab_99547_AACAACCATTCACGCAACGTATCA	0.393212106	0.134309169	-0.357579752
ab_99547_AACGCTTAAACCGAGACGACTGGA	0.603972994	0.134811338	-0.288667749
ab_99547_AACGCTTAAATGTTGCGAGCTGAA	0.822215533	0.163130779	-0.241091515
ab_99547_AACGCTTAATCATTCCCTGGCATA	0.765229191	0.05881258	-0.185777392
ab_99547_AACGTGATGAACAGGCACAGCAGA	0.876786196	0.05669539	-0.243169422
ab_99547_AACGTGATGAGTTAGCACAGCAGA	0.422433602	0.099738777	-0.246884426
ab_99547_AACGTGATGAGTTAGCCGGATTGC	0.810480587	0.117629299	-0.268361437
ab_99547_AACGTGATGGTGCGAAAATGTTGC	0.467544349	0.10520339	-0.33189034
ab_99547_AACTCACCAAGAGATCCGCTGATC	0.676230964	0.085092604	-0.391872197
ab_99547_AAGACGGAGCTCGGTACGCATACA	0.491332783	-0.123411376	-0.555348963
ab_99547_AAGACGGATATCAGCACAATGGAA	0.356198549	0.213965638	-0.161260072
ab_99547_AAGAGATCAACTCACCATTGAGGA	0.727287642	0.105369943	-0.455245683
ab_99547_AAGAGATCACGTATCACCTCCTGA	0.447893901	0.233423853	-0.35212471
ab_99547_AAGAGATCATAGCGACGAGCTGAA	0.575558455	0.207013719	-0.448397583
ab_99547_AAGAGATCGCCACATAGAGTTAGC	0.782304366	0.11683547	-0.205000836
ab_99547_AAGAGATCGCTCGGTAGAATCTGA	0.654343021	0.300805856	-0.056675473
ab_99547_AAGGACACCATCAAGTCGGATTGC	0.562334696	0.162841038	-0.233909375
ab_99547_AAGGACACCCTCCTGAAATGTTGC	0.719986594	0.104673221	-0.357040706
ab_99547_AAGGACACCGACACACTGGTGGTA	0.69920608	0.269983614	-0.14984884
ab_99547_AAGGACACCTGGCATACCATCCTC	0.595119256	0.018241668	-0.315853969
ab_99547_AAGGTACAAAGGACACTGGAACAA	0.354548543	0.242337381	-0.16202511
ab_99547_AAGGTACACCGACAACCGGATTGC	0.606696089	0.095314009	-0.361666866
ab_99547_AAGGTACATGGAACAAGACAGTGC	0.600214181	0.058692486	-0.25125503
ab_99547_AATGTTGCCGACACACACGCTCGA	0.519877738	0.055588354	-0.403008815
ab_99547_AATGTTGCGACAGTGCCGACTGGA	0.582773465	-0.041114947	-0.504624523

ab_99547_ACAAGCTACCTCCTGACATCAAGT	0.767131583	-0.036725653	-0.374953517
ab_99547_ACAAGCTACGACACACCATCAAGT	0.449484314	0.078456266	-0.306876852
ab_99547_ACAAGCTAGGTGCGAACGACACAC	0.71904391	0.235671499	-0.381599211
ab_99547_ACACGACCAGCACCTCACCTCCAA	0.512460906	-0.040052697	-0.474644441
ab_99547_ACACGACCATTGGCTCGAGTTAGC	0.563899028	0.147762805	-0.257370346
ab_99547_ACACGACCGCTCGGTACCTAATCC	0.296648115	0.054350885	-0.387532217
ab_99547_ACAGATTCACGCTCGAATGCCTAA	0.70569021	0.161442646	-0.332136674
ab_99547_ACAGATTCCCAGTTCACGCATACA	0.437740178	0.06730634	-0.198678155
ab_99547_ACAGATTCCGCATACAAACGTGAT	0.73181755	-0.007719842	-0.354994279
ab_99547_ACAGATTCGAGTTAGCACAGCAGA	0.582797348	0.177297577	-0.211946762
ab_99547_ACAGCAGATGAAGAGAGTACGCAA	0.874623152	0.155536063	-0.289769599
ab_99547_ACATTGGCATCCTGTAATGCCTAA	0.458330224	0.076755213	-0.330038435
ab_99547_ACATTGGCCGCATACATCCGTCTA	0.640714466	0.074169112	-0.432242636
ab_99547_ACCACTGTACTATGCAGCTAACGA	0.628924707	0.211776773	-0.229942647
ab_99547_ACCACTGTAGCAGGAAATAGCGAC	0.689823552	0.167312398	-0.158842299
ab_99547_ACCACTGTCGAACTTAGCTAACGA	0.638165424	0.216727291	-0.27788797
ab_99547_ACCACTGTGAATCTGAGCCACATA	0.726643314	0.062241587	-0.421487231
ab_99547_ACCACTGTGATAGACACCGAAGTA	0.757663997	0.244004973	-0.245073558
ab_99547_ACCACTGTGATGAATCGAGCTGAA	0.473108433	0.068600357	-0.477461227
ab_99547_ACCACTGTGCCAAGACCGGATTGC	0.647517133	0.068705665	-0.484814187
ab_99547_ACGCTCGAGCCAAGACATAGCGAC	0.691180063	0.167710102	-0.479375404
ab_99547_ACGTATCAACCACTGTTTCACGCA	0.511990226	0.076680248	-0.346874294
ab_99547_ACGTATCACTGGCATACGCTGATC	0.510588775	0.035478922	-0.418735175
ab_99547_ACTATGCAAAGACGGACATCAAGT	0.662998392	-0.115201397	-0.377265069
ab_99547_ACTATGCAACCACTGTGAGTTAGC	0.584619289	0.055929094	-0.384034289
ab_99547_ACTATGCACGACTGGACAGATCTG	0.517985089	0.301055604	-0.24733119
ab_99547_ACTATGCATGGAACAAGACTAGTA	0.899270116	0.290888403	-0.195397274
ab_99547_AGAGTCAAAAGACGGAAACTCACC	0.762156621	0.105397897	-0.274246688
ab_99547_AGAGTCAAACACGACCATAGCGAC	0.661643032	0.109947497	-0.093760153
ab_99547_AGAGTCAACCGACAACAACGTGAT	0.378837241	0.066864589	-0.274925422
ab_99547_AGCACCTCAAGACGGAAGTACAAG	0.848584037	0.262228332	-0.2250604
ab_99547_AGCACCTCATCCTGTAGATAGACA	0.530428962	0.05542701	-0.377283404

ab_99547_AGCACCTCGCGAGTAACGACACAC	0.689865961	0.139702174	-0.463695994
ab_99547_AGCAGGAAACTATGCATAGGATGA	0.688418926	0.070059047	-0.336815615
ab_99547_AGCAGGAACTAAGGTCACCACTGT	0.574462661	0.050142096	-0.365994169
ab_99547_AGCAGGAAGCTCGGTAACCTCCAA	0.486740928	0.156800546	-0.343984313
ab_99547_AGCCATGCAAGAGATCAATGTTGC	0.777973477	0.088159131	-0.331097431
ab_99547_AGCCATGCCGCTGATCAGCAGGAA	0.749507757	0.139589141	-0.43526188
ab_99547_AGCCATGCGTGTTCTAAGCACCTC	0.647511764	0.127728529	-0.357023719
ab_99547_AGGCTAACAAGACGGACTCAATGA	0.404613447	0.107497236	-0.268935301
ab_99547_AGGCTAACAGCCATGCACACGACC	0.722020503	0.143291241	-0.398035796
ab_99547_AGGCTAACAGGCTAACATTGGCTC	0.531895286	0.160056256	-0.442448643
ab_99547_AGGCTAACCCTCTATCATGCCTAA	0.335626178	0.05691895	-0.211771465
ab_99547_AGGCTAACTCCGTCTACGACTGGA	0.6184998	0.149358386	-0.379520381
ab_99547_AGTACAAGCTGAGCCAATCATTCC	0.560159274	0.209595607	-0.331499304
ab_99547_AGTCACTAGCCACATATGAAGAGA	0.538233521	0.042492186	0.038180079
ab_99547_AGTGGTCACCAGTTCAAGCACCTC	0.588350385	0.122885579	-0.338082341
ab_99547_AGTGGTCAGCGAGTAATGAAGAGA	0.5793168	0.051672297	-0.36495654
ab_99547_ATAGCGACATTGAGGAGTACGCAA	0.762247938	0.104680893	-0.457056579
ab_99547_ATAGCGACTCTTCACATCCGTCTA	0.45909922	0.090485186	-0.429422689
ab_99547_ATCATTCCAATCCGTCGGTGCGAA	0.791062347	-0.030082483	-0.163993882
ab_99547_ATCATTCCATTGAGGAAAGAGATC	0.602079241	0.183849156	-0.1456064
ab_99547_ATCATTCCTATCAGCAGCGAGTAA	0.463625806	0.045900754	-0.474432916
ab_99547_ATCCTGTAACATTGGCCAAGACTA	0.631141104	0.156876314	-0.264211909
ab_99547_ATGCCTAAACCTCCAACCTCCTGA	0.458951539	0.181609868	-0.413113936
ab_99547_ATGCCTAAAGTGGTCAAGATGTAC	0.369910233	-0.003192482	-0.240899466
ab_99547_ATGCCTAAGCCAAGACCCGACAAC	0.543446716	0.078490837	-0.331952466
ab_99547_ATGCCTAAGCCACATAAACTCACC	0.677677312	0.155628371	-0.43103161
ab_99547_ATGCCTAATCCGTCTAATGCCTAA	0.381891248	0.154311837	-0.343973904
ab_99547_ATTGAGGAAAGGTACAAATGTTGC	0.734306098	0.237184789	-0.239821641
ab_99547_ATTGAGGAACTATGCAAATGTTGC	0.759523393	0.150960246	-0.232786881
ab_99547_ATTGAGGAACTATGCAACGTATCA	0.567853751	0.223985844	-0.35718657
ab_99547_ATTGAGGAAGATCGCAAGCCATGC	0.795987288	0.065595067	-0.429483864
ab_99547_ATTGAGGACCGTGAGAGTCTGTCA	0.771593859	0.110491372	-0.326774046

ab_99547_ATTGGCTCAACGCTTACGGATTGC	0.747428193	0.01065156	-0.569138427
ab_99547_ATTGGCTCCAACCACAGCGAGTAA	0.78615222	0.113730792	-0.281329269
ab_99547_ATTGGCTCCAGATCTGCCAGTTCA	0.563191678	-0.030260687	-0.389101652
ab_99547_ATTGGCTCCGAACTTAATTGGCTC	0.581735012	0.22395969	-0.295544641
ab_99547_CAACCACAGAGCTGAAAATCCGTC	0.464715865	0.125916472	-0.377069056
ab_99547_CAAGACTAGAATCTGAACCTCCAA	0.57621636	0.142564293	-0.396901551
ab_99547_CAAGGAGCAACCGAGAACAGCAGA	0.294209429	0.127493091	-0.455422119
ab_99547_CAAGGAGCACAAGCTACCGAAGTA	0.568411887	0.318600258	-0.341365925
ab_99547_CAAGGAGCGACAGTGCCGCTGATC	0.874482383	0.123380737	-0.376973037
ab_99547_CAATGGAAACACAGAAACATTGGC	0.69106855	0.465870063	-0.399789144
ab_99547_CAATGGAAAGCCATGCAGCACCTC	0.456426115	0.16993004	-0.36412657
ab_99547_CAATGGAAGCCACATAGAATCTGA	0.652127879	0.02789194	-0.472456973
ab_99547_CACCTTACACCACTGTAAGAGATC	0.746757159	0.20165388	-0.344183568
ab_99547_CACCTTACCCGAAGTACTGTAGCC	0.504043949	0.032765645	-0.32032404
ab_99547_CACTTCGAACAAGCTAACAGCAGA	0.723522119	-0.013539406	-0.407851436
ab_99547_CACTTCGACCGTGAGACAGCGTTA	0.592109404	0.233081713	-0.30468917
ab_99547_CACTTCGACTGGCATAAACTCACC	0.258579549	0.0706393	-0.445614115
ab_99547_CAGATCTGCCTCTATCCCAGTTCA	0.452639802	0.165050575	-0.420568668
ab_99547_CATACCAAACAGATTCCGACACAC	0.389381967	0.057053992	-0.383230504
ab_99547_CATACCAACCAGTTCAGAGTTAGC	0.743645441	0.102118547	-0.427884451
ab_99547_CATACCAACCGAAGTAGACTAGTA	0.526679829	0.077986066	-0.233413849
ab_99547_CATACCAACCTCCTGAAACGTGAT	0.726285157	0.101221419	-0.325411266
ab_99547_CATACCAACGGATTGCAGTGGTCA	0.724231041	0.233946458	-0.192937462
ab_99547_CATACCAAGTACGCAAGCCACATA	0.458184987	0.133946317	-0.173394353
ab_99547_CATACCAATGAAGAGAAGTACAAG	0.829221297	0.246973231	-0.436813557
ab_99547_CATCAAGTAATCCGTCGAGCTGAA	0.433133076	0.118441425	-0.394220786
ab_99547_CATCAAGTCGCTGATCTTCACGCA	0.710779297	-0.038092601	-0.317938913
ab_99547_CATCAAGTGATAGACACAAGACTA	0.509368514	0.215237421	-0.402801954
ab_99547_CATCAAGTGGAGAACACGGATTGC	0.488747427	0.060575959	-0.428104649
ab_99547_CCAGTTCAAATCCGTCGATGAATC	0.524926942	0.160702073	-0.292781456
ab_99547_CCAGTTCACAACCACAATAGCGAC	0.66357641	0.105002473	-0.297132631
ab_99547_CCAGTTCAGATAGACAGCGAGTAA	0.556486194	0.129642271	-0.391098266

ab_99547_CCAGTTCAGGAGAACATGGAACAA	0.492585836	0.053751935	-0.28605585
ab_99547_CCATCCTCAAACATCGTAGGATGA	0.694442772	0.064231649	-0.46070122
ab_99547_CCATCCTCGTACGCAAAGATGTAC	0.580108932	0.04107229	-0.383659639
ab_99547_CCATCCTCTGAAGAGAGGAGAACA	0.41003937	0.123622816	-0.364004606
ab_99547_CCGAAGTACTGAGCCAATTGGCTC	0.820430816	0.028791906	-0.471326929
ab_99547_CCGACAACATCATTCCACATTGGC	0.750111469	0.273432533	-0.449126312
ab_99547_CCGACAACCGAACTTAATGCCTAA	0.62969785	-0.001894776	-0.435758398
ab_99547_CCGTGAGAAACAACCAAGTACAAG	0.764731019	0.240809458	-0.280430083
ab_99547_CCGTGAGAGCTCGGTAAAGAGATC	0.479635762	0.143826858	-0.387618219
ab_99547_CCGTGAGAGCTCGGTAGTCGTAGA	0.620833778	0.040776503	-0.452393714
ab_99547_CCTAATCCAACGCTTAAATCCGTC	0.835961018	0.185014693	-0.15591467
ab_99547_CCTAATCCACACGACCACACGACC	0.562168212	0.210767595	-0.164950072
ab_99547_CCTAATCCATTGAGGAACAGATTC	0.577172937	0.175009491	-0.350943609
ab_99547_CCTAATCCGGAGAACAAGAGTCAA	0.66812383	0.149309701	-0.253491601
ab_99547_CCTCCTGAAACCGAGAACTATGCA	0.434196395	0.06680949	-0.45628903
ab_99547_CCTCCTGAACAAGCTAGTACGCAA	0.60042702	0.102722399	-0.354622932
ab_99547_CCTCCTGAGCCACATAGCTAACGA	0.507694955	0.017413413	-0.274393421
ab_99547_CGAACTTAACAGCAGACGCTGATC	0.379358925	0.146839755	-0.276078756
ab_99547_CGAACTTACACTTCGATGAAGAGA	0.440577629	0.125648971	-0.440795899
ab_99547_CGAACTTAGAATCTGAAGATCGCA	0.617367654	0.0397152	-0.328805372
ab_99547_CGACACACACAAGCTACAAGGAGC	0.680243805	-0.007102771	-0.269103669
ab_99547_CGACACACAGTCACTAAAACATCG	0.726250337	0.194825065	-0.392465155
ab_99547_CGACACACCCAGTTCAACGCTCGA	0.594833732	-0.008563347	-0.228586865
ab_99547_CGCATACAAACAACCAATTGGCTC	0.847723696	0.098018129	-0.29346669
ab_99547_CGCATACAAATGTTGCCGACTGGA	0.596889222	0.031510102	-0.172395956
ab_99547_CGCATACACGCTGATCAGCACCTC	0.480937165	0.06497129	-0.421607607
ab_99547_CGCATACACGCTGATCGCGAGTAA	0.499386993	0.267064446	-0.19738919
ab_99547_CGCATACACTGGCATAAATGTTGC	0.509389989	0.095090893	-0.263612585
ab_99547_CGCTGATCAAACATCGCACTTCGA	0.740008398	0.146229831	-0.443113698
ab_99547_CGCTGATCACAGATTCCTGAGCCA	0.585111159	0.126021677	-0.229699055
ab_99547_CGCTGATCCAGATCTGGTGTTCTA	0.63908016	-0.031530729	-0.413848325
ab_99547_CGCTGATCCCGACAACTATCAGCA	0.616125055	0.209379638	-0.201243998

ab_99547_CGCTGATCGAGCTGAAAACAACCA	0.467695094	0.159428125	-0.284417344
ab_99547_CGCTGATCGAGCTGAAGGAGAACA	0.53622274	0.149126908	-0.134322352
ab_99547_CGCTGATCGAGTTAGCCGGATTGC	0.656233364	0.183806279	-0.339959271
ab_99547_CGCTGATCGGTGCGAATTCACGCA	0.418845416	0.11565277	-0.292863205
ab_99547_CGGATTGCATCATTCCGTCTGTCA	0.65231916	0.131458239	-0.274715393
ab_99547_CGGATTGCGCGAGTAAAGTGGTCA	0.487321489	0.136428358	-0.348404691
ab_99547_CTAAGGTCACGTATCAGCCACATA	0.615503963	0.199938988	-0.429797711
ab_99547_CTAAGGTCAGGCTAACTGGTGGTA	0.729945156	0.124112682	-0.220145191
ab_99547_CTAAGGTCATTGGCTCATCCTGTA	0.846494223	0.287872772	-0.234607793
ab_99547_CTCAATGACAGCGTTAGGAGAACA	0.746020839	0.025807632	-0.411113752
ab_99547_CTCAATGAGTCGTAGAGGTGCGAA	0.820737962	0.148150583	-0.334051979
ab_99547_CTGAGCCAAAGGACACAAGACGGA	0.510272071	0.095085028	-0.396383948
ab_99547_CTGAGCCAAATCCGTCAAGGACAC	0.312838186	0.102921019	-0.371237077
ab_99547_CTGAGCCAATTGGCTCTAGGATGA	0.539969957	0.043174151	-0.23287734
ab_99547_CTGAGCCAGAACAGGCTAGGATGA	0.50337556	0.145567553	-0.32465382
ab_99547_CTGGCATAGATGAATCGCTAACGA	0.597479635	0.10152478	-0.370129105
ab_99547_CTGTAGCCACCTCCAACATCAAGT	0.474280772	0.250026051	-0.319762494
ab_99547_CTGTAGCCACTATGCAGTCGTAGA	0.537348024	0.028368625	-0.260815184
ab_99547_CTGTAGCCAGCACCTCACACAGAA	0.551696567	0.020528947	-0.42404367
ab_99547_CTGTAGCCAGTACAAGCCATCCTC	0.637286839	0.086860271	-0.36438707
ab_99547_CTGTAGCCCAAGACTAAACCGAGA	0.736788047	-0.00596465	-0.445850296
ab_99547_CTGTAGCCCTAAGGTCAACCGAGA	0.674976936	0.118689656	-0.467541387
ab_99547_CTGTAGCCGACTAGTAAGTACAAG	0.784446562	-0.025307401	-0.349821373
ab_99547_CTGTAGCCGTACGCAAAGATGTAC	0.497196337	0.194232631	-0.148063682
ab_99547_GAACAGGCAATGTTGCAGTCACTA	0.690298669	0.105453632	-0.453301998
ab_99547_GAATCTGACTAAGGTCTTCACGCA	0.466900674	0.079979597	-0.356529953
ab_99547_GACAGTGCAACGTGATGACAGTGC	0.649149522	0.000935404	-0.555161197
ab_99547_GACAGTGCAATGTTGCGTGTTCTA	0.697086521	0.077265782	-0.513364158
ab_99547_GACAGTGCATTGGCTCATCCTGTA	0.656212196	0.092790699	-0.431437198
ab_99547_GACAGTGCCGACTGGAAGGCTAAC	1.336473647	0.293926251	-0.128656445
ab_99547_GACTAGTAAATCCGTCACTATGCA	0.700434073	0.003552428	-0.331114153
ab_99547_GACTAGTACGAACTTAAGTGGTCA	0.69312977	0.067448671	-0.377377853

ab_99547_GACTAGTAGTCTGTCAAACGCTTA	0.544487869	-0.056881175	-0.42451377
ab_99547_GAGCTGAACAATGGAACGACACAC	0.451556048	0.067783199	-0.409999643
ab_99547_GAGCTGAAGTACGCAACACTTCGA	0.527476569	0.190892254	-0.337190929
ab_99547_GAGTTAGCAACGTGATAGAGTCAA	0.598083442	0.171305469	-0.304788435
ab_99547_GAGTTAGCAATGTTGCGACAGTGC	0.652028956	0.148815415	-0.262285307
ab_99547_GAGTTAGCCTCAATGAGAACAGGC	0.842167164	0.225491837	-0.272353215
ab_99547_GAGTTAGCGCTAACGACCGTGAGA	0.336973088	0.122662959	-0.411790029
ab_99547_GAGTTAGCGTACGCAAGTCGTAGA	0.494860525	0.148558717	-0.303527582
ab_99547_GAGTTAGCTGGAACAAGCCAAGAC	0.657856877	0.226051177	-0.385237038
ab_99547_GATAGACAAGTGGTCAACGCTCGA	0.503070095	0.04069625	-0.386318849
ab_99547_GATAGACACATACCAAGGTGCGAA	0.452545911	0.16853602	-0.346381028
ab_99547_GATAGACACCTCCTGACAAGACTA	0.583135801	0.274630044	-0.293556225
ab_99547_GATAGACACTGGCATAAGCACCTC	0.545410547	0.386949463	-0.339720386
ab_99547_GATGAATCAAGGACACATCCTGTA	0.53895665	0.152726861	-0.293940672
ab_99547_GATGAATCCAAGGAGCCAAGACTA	0.338400014	0.091178249	-0.114670116
ab_99547_GCCAAGACAATCCGTCAGGCTAAC	0.581560787	0.105379837	-0.362833533
ab_99547_GCCAAGACACTATGCAGAGCTGAA	0.791397268	0.203862721	-0.173553561
ab_99547_GCCAAGACCCTCCTGATGGCTTCA	0.711565012	0.149890067	-0.264789049
ab_99547_GCCACATAAACGCTTAGCTAACGA	0.321441912	-0.033807803	-0.336549443
ab_99547_GCCACATAACGCTCGAACAGCAGA	0.538164137	0.035594282	-0.371091918
ab_99547_GCCACATAATCCTGTACCTCTATC	0.540870301	0.050305936	-0.37070757
ab_99547_GCCACATACTAAGGTCGACAGTGC	0.809024258	0.169821602	-0.442445344
ab_99547_GCCACATAGCGAGTAAAACGCTTA	0.895917889	0.021394331	-0.410217749
ab_99547_GCGAGTAAGAGCTGAACCGAAGTA	0.560559891	0.088833257	-0.304136686
ab_99547_GCTAACGAAGCACCTCGACAGTGC	0.293593814	0.189609948	-0.21988689
ab_99547_GCTAACGAGATAGACACCTCTATC	0.452884945	0.038558032	-0.328183199
ab_99547_GCTAACGAGTCGTAGAAGATCGCA	0.544133811	0.079443789	-0.362191579
ab_99547_GGAGAACAACAGATTCACAGCAGA	0.2319375	0.013739694	-0.396952782
ab_99547_GGAGAACAACGCTCGACAATGGAA	0.440030178	-0.079525937	-0.428186449
ab_99547_GGAGAACACATCAAGTGACTAGTA	0.520064814	-0.003987771	-0.429959172
ab_99547_GGAGAACAGCGAGTAAGACAGTGC	0.812940682	-0.008595632	-0.370938859
ab_99547_GGAGAACAGGTGCGAAAGTACAAG	0.571460958	0.059429536	-0.312522612

ab_99547_GGAGAACAGTCGTAGAAGATGTAC	0.578580874	0.249107225	-0.218905137
ab_99547_GGTGCGAAACACGACCAAGAGATC	0.506908081	0.112978811	-0.399734095
ab_99547_GGTGCGAAACAGCAGAAGATGTAC	0.77415975	0.027915884	-0.466608912
ab_99547_GGTGCGAAACATTGGCGACTAGTA	0.415435217	-0.062389552	-0.380211671
ab_99547_GGTGCGAACGACTGGAACAGATTC	0.298957612	0.248956355	-0.367806375
ab_99547_GGTGCGAACGCATACATAGGATGA	0.602358115	0.049503217	-0.351069563
ab_99547_GGTGCGAACTGAGCCAATAGCGAC	0.623082819	0.092733064	-0.35363768
ab_99547_GTACGCAACATACCAAGAACAGGC	0.711738396	0.08934359	-0.418194099
ab_99547_GTACGCAACCATCCTCACACAGAA	0.321933793	-0.063059694	-0.458429965
ab_99547_GTACGCAACCGACAACACGTATCA	0.603336966	0.095562187	-0.317700615
ab_99547_GTACGCAAGGAGAACAGAGTTAGC	0.46685421	0.045890624	-0.298020757
ab_99547_GTCGTAGAGCGAGTAAGACAGTGC	0.613545403	0.132359504	-0.452601721
ab_99547_GTCGTAGAGCTAACGAAGTACAAG	0.577053577	0.06705812	-0.437115223
ab_99547_GTCTGTCAAACCGAGAGCTCGGTA	0.43236323	0.056421821	-0.379942665
ab_99547_GTCTGTCAAATCCGTCATTGGCTC	0.725294937	0.099898068	-0.294006358
ab_99547_GTCTGTCAAGCCATGCCCGACAAC	0.683104464	-0.004539024	-0.272259672
ab_99547_GTCTGTCAATCCTGTATGGTGGTA	0.562161326	0.110682314	-0.460733173
ab_99547_GTCTGTCACACCTTACCTGTAGCC	0.734513995	0.030984953	-0.308243921
ab_99547_GTCTGTCACGCTGATCATTGAGGA	0.479236161	0.034189991	-0.4266378
ab_99547_GTGTTCTAAGCCATGCGGAGAACA	0.436256939	0.048114753	-0.362277061
ab_99547_TAGGATGAAACAACCACGCATACA	0.866473284	0.191067842	-0.114491057
ab_99547_TAGGATGAATAGCGACACACAGAA	0.461988895	0.228529221	-0.287800929
ab_99547_TAGGATGAATAGCGACACGTATCA	0.767612432	0.013662064	-0.3155936
ab_99547_TAGGATGACCAGTTCAACTATGCA	0.673827902	0.060546008	-0.388098663
ab_99547_TAGGATGACCATCCTCTATCAGCA	0.331282142	0.324612863	-0.253749801
ab_99547_TATCAGCAGAACAGGCCGACACAC	0.461187901	-0.001090596	-0.372598273
ab_99547_TATCAGCATAGGATGAACTATGCA	0.690833272	0.036032424	-0.308555266
ab_99547_TCCGTCTAACAGCAGACAACCACA	0.528776051	0.091697423	-0.44498497
ab_99547_TCCGTCTACCTCTATCTATCAGCA	0.505725062	0.091769386	-0.337024928
ab_99547_TCTTCACAACACAGAACGGATTGC	0.744621813	0.115301547	-0.123485331
ab_99547_TCTTCACAACAGCAGAGACAGTGC	0.451049197	0.215609068	-0.271831619
ab_99547_TCTTCACACTGGCATAAAGGACAC	0.795882376	0.088963048	-0.310119645

ab_99547_TCTTCACAGCCACATACGCATACA	1.005043351	0.148595299	-0.331125155
ab_99547_TCTTCACATATCAGCACTGTAGCC	0.705760422	0.153224474	-0.216625631
ab_99547_TGGAACAAAACGTGATGCGAGTAA	0.685672739	0.068418924	-0.325535427
ab_99547_TGGAACAACATCAAGTCGGATTGC	0.494517379	0.0771141	-0.151008861
ab_99547_TGGCTTCAAGATGTACCCTCTATC	0.688385567	0.10565748	-0.287454458
ab_99547_TGGCTTCACCAGTTCAAAGGTACA	0.635720106	0.118710636	-0.23430719
ab_99547_TGGCTTCACTAAGGTCCCAGTTCA	0.708079627	0.119759069	-0.218317356
ab_99547_TGGCTTCACTAAGGTCGTCTGTCA	0.76303123	0.234245124	-0.111320894
ab_99547_TGGCTTCATCCGTCTACCATCCTC	0.811853458	0.149766096	-0.352403463
ab_99547_TGGTGGTAATTGGCTCCCTAATCC	0.529127885	0.030940908	-0.388514891
ab_99547_TGGTGGTAGTCGTAGACAAGGAGC	0.644708778	0.172276109	-0.353183033
ab_99547_ACACAGAACCTCCTGAGACAGTGC	1.363196599	0.198679826	-0.210753742
ab_99547_AGATGTACGAGTTAGCCAGATCTG	0.901190122	0.434286848	-0.319357752
ab_99547_AGCACCTCATTGAGGAGCTCGGTA	0.911772865	0.249028858	-0.309960893
ab_99547_TATCAGCAATAGCGACCCGAAGTA	0.501062949	0.315136295	-0.14434999
ab_99547_AAACATCGAACGCTTAAGCAGGAA	0.778107327	0.062327811	-0.23326534
ab_99547_AACAACCAGAACAGGCAGTCACTA	0.35401177	0.059135799	-0.332850101
ab_99547_AACTCACCAACGCTTACCTAATCC	0.460308129	0.033610759	-0.382208875
ab_99547_AAGACGGACAGATCTGAAGACGGA	0.461104901	0.098329743	-0.382646217
ab_99547_AAGGACACAAGGACACACAAGCTA	0.659636155	0.127669058	-0.303731039
ab_99547_AAGGTACACAACCACAGTCGTAGA	0.441203594	0.039708511	-0.260916348
ab_99547_AATGTTGCACGTATCACGCTGATC	0.612805659	-0.000502658	-0.159776955
ab_99547_ACACAGAAATTGGCTCAACCGAGA	0.484926381	0.125525079	-0.304033008
ab_99547_ACACGACCTGGAACAAGCCAAGAC	0.424335011	0.084916168	-0.25506754
ab_99547_ACAGCAGAAACAACCACCTCTATC	0.567279847	0.087419109	-0.385073987
ab_99547_ACCACTGTCCTAATCCAAGACGGA	0.629783881	0.146118171	-0.236821645
ab_99547_ACCACTGTGATGAATCAGTCACTA	0.263445978	-0.006328451	-0.352299803
ab_99547_ACGTATCACCAGTTCACTGTAGCC	0.486236997	0.157130515	-0.385337853
ab_99547_AGAGTCAATGAAGAGAGAGCTGAA	0.709375483	-0.035381304	-0.347232775
ab_99547_AGCACCTCACACAGAAACATTGGC	0.430159095	0.200317012	-0.320876421
ab_99547_AGCACCTCATCCTGTAAACAACCA	0.785565288	0.140997034	-0.368032172
ab_99547_AGGCTAACACCTCCAACTCAATGA	0.484130215	0.036586603	-0.455419743

ab_99547_AGTACAAGGATGAATCGATGAATC	0.675933433	0.195398306	-0.239932307
ab_99547_ATTGGCTCTTCACGCATGGAACAA	0.417986605	0.054764208	-0.196610526
ab_99547_CACTTCGAAAGAGATCCTGAGCCA	0.487216184	0.140997793	-0.360014873
ab_99547_CACTTCGATTCACGCAAGCCATGC	0.557596006	-0.008836189	-0.229618425
ab_99547_CAGCGTTACCGAAGTAACGTATCA	0.572563896	0.206165125	-0.42062372
ab_99547_CAGCGTTAGAGCTGAAAACAACCA	0.553660546	-0.063621631	-0.469352823
ab_99547_CCAGTTCACATCAAGTCCGACAAC	0.559546572	0.090532394	-0.43056535
ab_99547_CCAGTTCACCTAATCCGAATCTGA	0.5493263	0.06736334	-0.123463873
ab_99547_CCGAAGTAAACGTGATGCCACATA	0.369354002	-0.082424949	-0.483533549
ab_99547_CCGAAGTAAAGAGATCAGTGGTCA	0.503790006	0.189068156	-0.328488701
ab_99547_CCGAAGTACAATGGAAAAACATCG	0.477506878	0.115614961	-0.422450891
ab_99547_CCGTGAGACTAAGGTCACACGACC	0.45300036	0.194778364	-0.098900712
ab_99547_CCGTGAGATGAAGAGACACCTTAC	0.341759265	0.084254347	-0.292009072
ab_99547_CCTAATCCAACCGAGAAAACATCG	0.646869416	0.112322048	-0.329084314
ab_99547_CCTAATCCCGGATTGCAAGGTACA	0.326198197	0.009694306	-0.484592922
ab_99547_CCTCTATCCAGCGTTAGTGTTCTA	0.58288182	0.111239498	-0.3619974
ab_99547_CGAACTTAATTGAGGAAGCAGGAA	0.508792682	0.236790882	-0.344063202
ab_99547_CGAACTTATGGCTTCATCCGTCTA	0.456373393	0.145433801	-0.170419565
ab_99547_CGCATACACAGCGTTATCTTCACA	0.648617634	0.076416487	-0.415669234
ab_99547_CTAAGGTCTAGGATGACAGATCTG	0.585911017	0.013600912	-0.249512282
ab_99547_CTGAGCCATCCGTCTAGTCTGTCA	0.585260562	0.043778179	-0.221222279
ab_99547_GAACAGGCACTATGCAAGCACCTC	0.38905413	0.184743017	-0.42889676
ab_99547_GAGCTGAAATAGCGACGAACAGGC	0.30974076	0.153257964	-0.231387744
ab_99547_GCCACATAGAACAGGCGAGTTAGC	0.473656536	0.170722466	-0.322745944
ab_99547_GCCACATAGAGCTGAACATCAAGT	0.794662739	0.117176924	-0.328658998
ab_99547_GGAGAACAACCTCCAACACCTTAC	0.287520007	0.143332288	-0.289522737
ab_99547_GTCGTAGATCCGTCTAAGCCATGC	0.840795162	0.025456865	-0.297554982
ab_99547_TCTTCACACAGATCTGCACTTCGA	0.362744131	-0.034288581	-0.389219637
ab_99547_TGGAACAAGAGCTGAACCTCCTGA	0.528434489	0.157432468	-0.415309004
ab_99547_TGGTGGTACAAGGAGCAATGTTGC	0.556616897	0.149454489	-0.133414812
ab_99547_TTCACGCAATCCTGTAGATGAATC	0.710995359	-0.012695096	-0.158825797
ab_99547_ATCCTGTACCTCTATCGGAGAACA	0.954078644	0.204887228	-0.260261034

ab_99547_CATCAAGTCATCAAGTCAATGGAA	0.465980168	0.220659031	-0.098590143
ab_99547_CTGAGCCAACACAGAAAACGCTTA	0.500090848	0.134345628	-0.324761191
ab_99547_AAACATCGGAGTTAGCTCCGTCTA	0.666674694	0.101347823	-0.274079719
ab_99547_AACCGAGACCAGTTCAACCACTGT	0.764463138	0.079887623	-0.344479864
ab_99547_AAGAGATCGGAGAACACGACTGGA	0.669223634	0.216422341	-0.474047244
ab_99547_AAGGTACACGCATACAAGCAGGAA	0.474622986	0.356479253	-0.291750101
ab_99547_ACCTCCAAGTACGCAACCTAATCC	0.575825212	0.077658279	-0.315720929
ab_99547_ACTATGCAACACGACCAAGGTACA	0.71368054	0.136115926	-0.300777267
ab_99547_AGAGTCAAAGCAGGAACGCTGATC	0.831832279	0.082601289	-0.422334258
ab_99547_AGCACCTCACGCTCGAATTGGCTC	0.851630787	0.059553385	-0.349408737
ab_99547_AGCCATGCCACCTTACTCTTCACA	0.602149934	0.26035475	-0.153255732
ab_99547_AGGCTAACCGCTGATCCCGACAAC	0.996405806	0.071717499	-0.550904341
ab_99547_ATAGCGACAGCCATGCCGCATACA	0.845843297	0.208774122	-0.268979505
ab_99547_ATCCTGTACAACCACACGACACAC	0.799223489	0.250926783	-0.255390064
ab_99547_CAATGGAACTAAGGTCATAGCGAC	0.648902914	0.071406926	-0.420940067
ab_99547_CACTTCGAAGTACAAGGAGCTGAA	0.751556012	0.090784139	-0.166392913
ab_99547_CAGCGTTACGACTGGAACAGATTC	0.774498745	0.06358655	-0.388599338
ab_99547_CATCAAGTGCCAAGACCCAGTTCA	0.444025488	0.104193772	-0.364584091
ab_99547_CCAGTTCATCTTCACACCTAATCC	0.410575687	0.255816639	-0.376105436
ab_99547_CCGAAGTAAGTGGTCAAAGACGGA	0.587445977	0.381206938	-0.331776908
ab_99547_CCTCCTGAAATGTTGCATAGCGAC	0.782675299	0.177261652	-0.281912838
ab_99547_CGACACACACACGACCAATCCGTC	0.774938576	0.224379477	-0.346805109
ab_99547_CGACTGGAAAGAGATCCTCAATGA	0.800782177	0.117858275	-0.351960408
ab_99547_CGCATACACTGTAGCCGCTCGGTA	0.771654355	0.328204514	-0.243689817
ab_99547_CTGGCATAGCTAACGACGCTGATC	0.81555314	0.164312688	-0.22413456
ab_99547_GAACAGGCACGTATCATCCGTCTA	0.678824109	0.075901135	-0.101983664
ab_99547_GACAGTGCAACTCACCTGGTGGTA	0.816708143	0.303863839	-0.411171516
ab_99547_GAGCTGAAAGCACCTCACAGATTC	0.558813309	0.135731017	-0.219321319
ab_99547_GATGAATCGGAGAACAGACAGTGC	0.649371805	0.212286961	-0.32847442
ab_99547_GCCAAGACGAACAGGCAGCACCTC	0.40291676	0.115856969	-0.334276917
ab_99547_GGTGCGAAGACTAGTACCGACAAC	0.318586028	-0.011206679	-0.271647805
ab_99547_GGTGCGAATCTTCACAAGGCTAAC	0.382076843	0.155068674	-0.434447136

ab_99547_TGGAACAAACAAGCTATGGAACAA	0.474122423	0.171321006	-0.402419676
ab_99547_TGGAACAAGAATCTGAAGAGTCAA	0.865555785	0.057668698	-0.206547576
ab_99547_AAGAGATCACGTATCAACAAGCTA	0.825925154	0.387977338	-0.141662852
ab_99547_CAAGGAGCGATGAATCCTAAGGTC	0.913963022	0.30348665	-0.317283612
ab_99547_GATAGACACGGATTGCAACTCACC	0.411526976	0.102237158	-0.102450629
ab_99547_GCTAACGACGCATACACAGCGTTA	0.657358111	0.142399684	-0.205781879
ab_99547_ATCCTGTAAGTGGTCATGGAACAA	0.591655282	0.056653138	-0.264081704
A_98763_AACGCTTAAACGTGATCTGGCATA	0.611481033	0.539236013	0.729260422
A_98763_ACACAGAAATCATTCCAAACATCG	0.494089085	0.467321155	0.665160897
A_98763_ACATTGGCACACAGAACAATGGAA	0.579031887	0.207958538	0.719122177
A_98763_AGTACAAGGTCGTAGAGAATCTGA	0.613983765	0.332441256	0.606950996
A_98763_AGTGGTCACACTTCGATGGAACAA	0.467969961	0.013781926	0.408206294
A_98763_AAGACGGAACATTGGCAAGGTACA	0.462309101	1.615811403	1.673053877
A_98763_AAGACGGAAGCAGGAAAGATCGCA	1.549858282	1.214208606	1.035661696
A_98763_ACACAGAAGACAGTGCATTGGCTC	0.715868192	1.493091641	1.672393815
A_98763_ACCTCCAACTAAGGTCGCCACATA	0.579808866	1.619418481	1.474762435
A_98763_ACGCTCGACACCTTACTGGAACAA	1.112210945	1.423172539	1.298477639
A_98763_ACTATGCAAGTGGTCATATCAGCA	1.537078314	1.228515596	1.777914109
A_98763_CAAGACTAACATTGGCCCTCTATC	1.115459629	1.367459535	0.788655763
A_98763_CATACCAATCCGTCTAACAGCAGA	1.165674644	1.382352966	1.200717403
A_98763_CCTCCTGACAAGACTAAACGTGAT	1.072361301	1.526376538	1.620173327
A_98763_CGACACACGAACAGGCGGTGCGAA	1.666225138	1.187838936	1.278380615
A_98763_CTAAGGTCGAATCTGATCCGTCTA	0.340393628	0.90344243	1.356221883
A_98763_GAGCTGAACATACCAAGCCACATA	0.50489505	1.561489157	1.5891477
A_98763_GAGTTAGCTGGTGGTACCGACAAC	0.462781419	1.648570651	1.931308102
A_98763_TCCGTCTAATAGCGACCCTCCTGA	0.732344337	1.390797456	1.47432945
A_98763_TGGCTTCAAGTCACTAAGTCACTA	0.984382421	0.896041137	1.244614883
A_98763_TGGCTTCACGGATTGCATTGAGGA	0.840198942	1.615812065	1.202386941
A_98763_AACCGAGAAGAGTCAAACGTATCA	1.012914125	1.974888877	1.14608114
A_98763_AACTCACCCTGTAGCCTCCGTCTA	1.127014566	1.78579019	1.154655747
A_98763_AAGGTACAGAATCTGACACTTCGA	1.160714397	1.673426057	0.970614982
A_98763_AAGGTACATGGAACAATGAAGAGA	1.385121679	1.993382698	0.987745224

A_98763_AATCCGTCTGGAACAAATTGAGGA	0.778579864	2.078823608	0.931529187
A_98763_ACACAGAAATTGAGGACGACTGGA	1.055602639	1.987244909	1.234842546
A_98763_ACACAGAACCTCTATCGATAGACA	0.952201796	1.795826359	0.292518841
A_98763_ACATTGGCACGTATCAAAGGTACA	1.358501285	1.848607524	0.719104166
A_98763_ACTATGCAACACGACCCCTCCTGA	1.192089667	2.067942795	1.301046387
A_98763_AGCACCTCTATCAGCAACAGCAGA	1.089709245	2.228742671	1.068312472
A_98763_AGTGGTCAACCTCCAACGACTGGA	1.084653174	2.026382689	0.883982125
A_98763_ATAGCGACGCTAACGACATCAAGT	0.477279562	1.295376289	0.871271718
A_98763_ATCATTCCCCGACAACGGTGCGAA	1.117050884	2.14508507	0.883378322
A_98763_CAATGGAAAGCAGGAAAAGACGGA	1.342039652	2.23347243	0.650967005
A_98763_CATCAAGTTGAAGAGACGACTGGA	1.091154946	2.170681854	1.323224363
A_98763_CCAGTTCAAGTGGTCACGACACAC	1.297256055	2.110650821	0.858176137
A_98763_CCGAAGTAAATCCGTCAAGAGATC	1.193355822	2.047149954	1.118078801
A_98763_CCGACAACATAGCGACTCCGTCTA	1.056670498	2.457239367	1.095284908
A_98763_CCGTGAGAGAACAGGCCCTCTATC	0.990425372	2.201652078	0.950892616
A_98763_CCTAATCCGGTGCGAAACAAGCTA	1.040815041	2.199677571	1.187652826
A_98763_CCTCTATCACTATGCAGCTAACGA	0.700383912	1.70293964	0.948173732
A_98763_CCTCTATCCAGATCTGTTCACGCA	0.865495575	1.862663424	0.800172913
A_98763_CGACACACGTACGCAAATCATTCC	1.163614692	2.432264232	1.341554722
A_98763_CTAAGGTCGCTAACGAAACAACCA	0.53345806	1.941914474	0.695700595
A_98763_CTAAGGTCGCTAACGAACAAGCTA	1.152252235	2.172080687	0.609058905
A_98763_CTGTAGCCCCGTGAGAACATTGGC	1.227397194	2.039586685	0.444250403
A_98763_GACAGTGCACGTATCAGAGTTAGC	1.073260225	2.084540748	1.47804133
A_98763_GCCAAGACGAGCTGAAGTACGCAA	1.042622331	2.221273322	0.736332424
A_98763_GCTCGGTACTGTAGCCACGTATCA	0.727830175	2.268424012	0.973259064
A_98763_GTGTTCTAGTCTGTCAACACAGAA	0.883045453	2.586576386	1.165731588
A_98763_TGGCTTCAAAGACGGAGAACAGGC	1.175126745	1.685117743	0.768179161
A_98763_TGGCTTCATCTTCACAGACTAGTA	1.134685604	1.816523112	0.127224719
A_98763_ACATTGGCCGAACTTATGGCTTCA	0.650398142	1.15208971	1.028208055
A_98763_ATCATTCCAGTGGTCACTGAGCCA	0.935642658	0.944416927	1.386225313
A_98763_CATCAAGTGATAGACATGGTGGTA	0.961501325	1.192999578	1.361616933
A_98763_CCTCCTGACAATGGAACTGAGCCA	0.511798582	1.520248876	1.238395518

A_98763_CGCATACACGGATTGCATCCTGTA	0.497567134	1.831719975	1.097792984
A_98763_CTGAGCCACGCATACAGCTCGGTA	0.689867101	1.957509431	0.868529616
A_98763_CTGTAGCCGATAGACACCTCCTGA	0.525037887	1.746923226	1.048256769
A_98763_GGAGAACAACGTATCAAGCCATGC	0.725930715	1.665430286	1.430251158
A_98763_GGAGAACAGTCTGTCACATCAAGT	0.591179723	2.036094728	1.806168574
A_98763_GGTGCGAAGGTGCGAAAGCCATGC	0.195918667	1.618255426	1.540269353
A_98763_GTGTTCTAAGTGGTCAACCACTGT	1.546776796	2.064429578	0.762260335
A_98763_TGGAACAACGACACACACCTCCAA	0.788307204	2.102707543	1.009272532
A_98763_AAACATCGAGAGTCAAGCTCGGTA	0.973809473	1.040179654	0.355711437
A_98763_AAACATCGGTACGCAACAACCACA	0.530674212	1.027151556	-0.055067772
A_98763_AACCGAGACATCAAGTGAATCTGA	0.735992531	1.698183876	0.227444251
A_98763_AACCGAGAGAGCTGAAGTCTGTCA	0.812040216	0.991151101	-0.082007148
A_98763_AACCGAGAGCGAGTAAGTCTGTCA	0.914860531	0.613481553	0.791006895
A_98763_AACGCTTAAAACATCGAGGCTAAC	0.530501348	0.538510137	0.363943298
A_98763_AACGTGATCCGACAACCCTCCTGA	1.187985357	1.184742876	-0.164013713
A_98763_AACGTGATGCGAGTAAACGCTCGA	0.601449484	0.384814743	0.321333816
A_98763_AACGTGATTCCGTCTAGTGTTCTA	1.219958157	1.552405323	0.526323738
A_98763_AACTCACCAAGACGGAGGAGAACA	0.818038618	1.485367337	1.161574123
A_98763_AACTCACCCTCAATGACACTTCGA	1.084195657	1.04428811	0.291723223
A_98763_AACTCACCCTGGCATATGGTGGTA	0.499517404	1.313196369	0.389562663
A_98763_AAGAGATCACACGACCCCGACAAC	1.112901888	1.957622786	0.883748272
A_98763_AAGAGATCAGCCATGCCCGAAGTA	0.543271959	0.426282222	-0.286775021
A_98763_AAGAGATCGCCACATACAAGGAGC	1.06332495	1.123662882	0.509205039
A_98763_AAGGACACCACCTTACATAGCGAC	0.120024321	0.204976364	0.324696359
A_98763_AAGGACACCGCATACAACAGCAGA	1.026209867	1.50563879	0.581593246
A_98763_AAGGTACAAGATGTACATGCCTAA	0.68763693	1.058849384	0.222599341
A_98763_AAGGTACAGAGCTGAAGCTAACGA	1.264430022	1.426551739	0.224152369
A_98763_AATCCGTCAAGAGATCCAGATCTG	1.430371523	1.783516959	0.564651006
A_98763_AATCCGTCCCTCTATCTGAAGAGA	0.920498262	1.433706076	0.634470209
A_98763_AATGTTGCAGTCACTACGCTGATC	0.89844658	1.365830283	0.56278725
A_98763_AATGTTGCATTGAGGAACCACTGT	1.098453084	1.748954317	0.66962374
A_98763_ACAAGCTAACCACTGTCCATCCTC	0.802007553	1.199792876	0.329428417

A_98763_ACAAGCTAATGCCTAAGCTCGGTA	0.369584473	0.272031537	0.352800694
A_98763_ACAAGCTAGCGAGTAACCGAAGTA	0.803166642	1.857923555	1.08855158
A_98763_ACAAGCTATAGGATGAAGCAGGAA	0.650936824	0.623626794	0.406788418
A_98763_ACACGACCAACGTGATACCACTGT	0.560102452	0.902755973	0.594272964
A_98763_ACACGACCGCTAACGACACTTCGA	0.700199086	1.009959339	0.329285469
A_98763_ACAGATTCCAAGACTACCTCCTGA	0.827723648	1.314110223	0.452173097
A_98763_ACAGATTCGCCACATAAAACATCG	0.495320226	0.524640347	0.348360159
A_98763_ACAGCAGACCAGTTCATGAAGAGA	1.068291287	1.064690084	0.137677807
A_98763_ACAGCAGACGGATTGCACACGACC	1.167326713	1.195741585	0.082539387
A_98763_ACATTGGCAACAACCAACATTGGC	0.590244051	0.292804789	0.625839646
A_98763_ACCACTGTAACGCTTACACTTCGA	0.391722949	0.646039298	0.653306964
A_98763_ACCACTGTACACAGAACCTCCTGA	1.157320996	1.964007066	0.891199246
A_98763_ACCACTGTAGAGTCAAATTGGCTC	0.958835587	1.888425997	1.249399558
A_98763_ACCACTGTCATACCAAGCCACATA	0.913936371	1.125841577	0.140907641
A_98763_ACGCTCGAAGCACCTCAAGACGGA	0.988968444	1.923689196	0.701952638
A_98763_ACGCTCGAAGTACAAGGGTGCGAA	0.171751917	0.233267587	0.52389524
A_98763_ACGCTCGAATAGCGACGGTGCGAA	0.72595149	1.224666631	0.456194823
A_98763_ACGCTCGAGAATCTGAGAATCTGA	0.746739629	0.327888591	-0.421821352
A_98763_ACGCTCGAGTCTGTCACGACACAC	0.736742397	0.761967689	-0.157151617
A_98763_ACGTATCAACACGACCACAGCAGA	0.76205168	1.018955793	0.166702943
A_98763_ACGTATCAACACGACCTGAAGAGA	1.107435935	1.535966166	0.474286592
A_98763_ACGTATCACTGGCATAAACAACCA	0.409200606	0.394702798	0.063253162
A_98763_ACGTATCAGACTAGTATGGCTTCA	0.771247019	1.419826061	0.471154127
A_98763_ACTATGCAACACGACCACACAGAA	0.759783305	1.139234874	0.526783783
A_98763_ACTATGCAAGTACAAGGCGAGTAA	1.185035085	1.436857979	0.692022292
A_98763_ACTATGCACTGTAGCCATGCCTAA	0.888415285	0.970834968	0.112932306
A_98763_AGAGTCAACGAACTTAGCGAGTAA	1.037294739	1.293934898	0.42035744
A_98763_AGAGTCAACTGGCATAAACGTGAT	1.275027667	1.763867625	1.070776239
A_98763_AGAGTCAAGGTGCGAACGGATTGC	0.918800825	0.63203515	-0.199757228
A_98763_AGATCGCAACAAGCTACATCAAGT	0.5469732	1.245251903	0.360430358
A_98763_AGATCGCACTCAATGAACCTCCAA	0.357756569	0.311316939	0.781803514
A_98763_AGATCGCAGTGTTCTAAACCGAGA	0.970280931	1.92152866	0.497474901

A_98763_AGATGTACAACGCTTAGAACAGGC	0.79911041	0.936688269	-0.075887007
A_98763_AGATGTACAAGGTACACTGAGCCA	0.893626801	1.146997096	0.180655092
A_98763_AGATGTACATTGAGGACCATCCTC	0.717437012	0.877624079	0.368908808
A_98763_AGATGTACCCATCCTCGTACGCAA	0.463925501	0.462681269	0.623393568
A_98763_AGATGTACGTCTGTCAAGATCGCA	0.873047454	1.853340415	0.190757874
A_98763_AGCACCTCATAGCGACAGCCATGC	0.943730351	1.208855762	0.410801608
A_98763_AGCACCTCCCTCTATCATGCCTAA	0.707265331	1.307869926	1.020994542
A_98763_AGCACCTCTGGTGGTAAACAACCA	0.867036042	1.343130968	0.080303842
A_98763_AGCAGGAAGCGAGTAAAGTCACTA	0.792396667	1.07595635	0.084333792
A_98763_AGGCTAACCACCTTACGCCACATA	1.038404355	1.624147568	0.208856211
A_98763_AGGCTAACGGAGAACAGCCACATA	0.763018302	0.627958105	0.052994126
A_98763_AGTACAAGCATACCAAATTGGCTC	0.950236205	0.964216315	1.036875963
A_98763_AGTACAAGCCTAATCCAATGTTGC	0.308548416	0.186984271	0.08100253
A_98763_AGTCACTAATCCTGTAATCATTCC	0.897264631	1.525572341	0.318020466
A_98763_AGTCACTACACTTCGAAGCCATGC	1.070021421	1.723336334	0.772175667
A_98763_AGTCACTAGAATCTGATCCGTCTA	0.752316095	1.504802462	0.8624073
A_98763_AGTCACTAGATAGACAAATGTTGC	0.608035261	1.469171463	1.121361891
A_98763_AGTGGTCAAAGGACACTGGAACAA	1.158839398	1.479655243	0.501375633
A_98763_AGTGGTCAGGAGAACATGGTGGTA	0.787459448	1.052117019	0.000804439
A_98763_ATAGCGACAAGACGGAGGTGCGAA	1.174895536	1.411555128	0.913626161
A_98763_ATAGCGACAGATGTACAGCCATGC	0.808332234	0.645893762	0.422219774
A_98763_ATAGCGACAGTCACTAAACGCTTA	0.778548738	1.693519078	0.852402054
A_98763_ATCATTCCACCACTGTACGTATCA	0.752569692	0.434044416	0.401199858
A_98763_ATCATTCCAGATGTACACACGACC	1.122267167	1.34849132	0.21590095
A_98763_ATCCTGTAAGTCACTAAACAACCA	1.06033145	1.172229914	0.830296982
A_98763_ATCCTGTAAGTCACTAACCTCCAA	0.870358046	1.496590206	0.524563107
A_98763_ATCCTGTACCTCCTGAACATTGGC	0.55802107	0.660310645	0.933270725
A_98763_ATGCCTAAAGTCACTAAAGACGGA	1.168775515	1.723545783	0.869194452
A_98763_ATGCCTAAGAACAGGCACACAGAA	0.706499303	1.38644701	1.014416904
A_98763_ATGCCTAAGAGCTGAACGCATACA	0.970876175	1.173196119	0.30484533
A_98763_ATGCCTAATGAAGAGATGAAGAGA	1.04461498	2.116463298	0.78159443
A_98763_ATTGAGGAGCCAAGACGTCTGTCA	0.281387558	0.532232619	0.149011312

A_98763_ATTGGCTCCACTTCGATTCACGCA	0.687081904	0.437612876	0.560289461
A_98763_ATTGGCTCCCGTGAGATATCAGCA	1.158634174	1.572472932	0.375475199
A_98763_ATTGGCTCTGAAGAGACGAACTTA	1.240530842	1.62124917	0.095456781
A_98763_CAACCACAAGATGTACCCGAAGTA	1.25673482	1.681631488	0.492112997
A_98763_CAAGACTATTCACGCATGGCTTCA	0.930487954	1.726413929	0.321115959
A_98763_CAAGGAGCATCATTCCCAAGACTA	0.587308255	0.963091833	0.09670142
A_98763_CAAGGAGCGACAGTGCCGGATTGC	0.985652421	1.711244202	0.641484806
A_98763_CAATGGAAATCATTCCAGTGGTCA	0.639092945	1.985568249	0.54655882
A_98763_CAATGGAACAGATCTGAAGAGATC	0.784909925	0.463004064	0.703976255
A_98763_CAATGGAAGCCACATAAGTGGTCA	0.659596253	1.348523975	0.13446612
A_98763_CACCTTACCAACCACACATCAAGT	1.234876255	1.746987316	0.340884807
A_98763_CACCTTACCGACTGGAAGTGGTCA	0.677832816	0.535746827	-0.078708144
A_98763_CACTTCGACCATCCTCCCATCCTC	0.917844115	1.515011314	0.142721115
A_98763_CACTTCGAGAACAGGCCAGATCTG	0.537735049	0.481280803	0.559756033
A_98763_CACTTCGAGGAGAACAACAGATTC	0.663073536	0.336377314	0.10358821
A_98763_CACTTCGATGGAACAACCGACAAC	0.893893037	1.721833019	0.598715286
A_98763_CAGATCTGAGCAGGAAACAGCAGA	0.91646847	1.072046559	0.281626002
A_98763_CAGATCTGCAACCACAGACAGTGC	0.445643923	0.646122767	0.303580846
A_98763_CAGATCTGCGAACTTACCTAATCC	0.772160366	1.807422158	1.001975832
A_98763_CAGCGTTAACAGATTCCAGATCTG	1.132950622	1.621313367	1.191013462
A_98763_CATACCAAAGGCTAACGAGTTAGC	0.883292038	0.964160833	0.777240566
A_98763_CATACCAACGCTGATCTCTTCACA	0.964005649	1.336832491	0.935651547
A_98763_CATACCAACTGGCATAGTCGTAGA	0.967513725	1.848208382	1.136579034
A_98763_CATCAAGTCATCAAGTAACAACCA	0.650858513	1.216061955	0.559292858
A_98763_CCAGTTCAACGCTCGAAACGTGAT	0.808906162	0.975903371	0.335809298
A_98763_CCATCCTCAAGGTACACCATCCTC	0.673370802	1.160117899	0.799234462
A_98763_CCATCCTCAATGTTGCCAACCACA	0.74820487	1.44280071	0.687849704
A_98763_CCATCCTCCCGTGAGAACAGATTC	0.612011328	1.341499932	0.170976177
A_98763_CCGAAGTAATTGGCTCACAGATTC	0.888207108	1.149035327	0.30697849
A_98763_CCGAAGTACCAGTTCAACAGATTC	1.005401003	1.958946223	1.185379227
A_98763_CCGAAGTAGACAGTGCAGCCATGC	0.798398952	1.146423805	0.455864383
A_98763_CCGAAGTAGATGAATCACATTGGC	1.035980764	1.666617667	0.545630946

A_98763_CCGAAGTAGCTCGGTATTCACGCA	0.43831832	0.405041721	-0.026462849
A_98763_CCGACAACTGGCTTCAAAGAGATC	0.895005702	0.847887121	0.388802135
A_98763_CCGTGAGAGCTCGGTAGCTAACGA	0.344104426	0.744068793	-0.301124718
A_98763_CCGTGAGAGTGTTCTAACACAGAA	0.847789512	1.654779565	0.030005351
A_98763_CCTAATCCAACCGAGACTGGCATA	0.942409374	1.443751846	0.703255888
A_98763_CCTAATCCACAAGCTACTCAATGA	0.696735492	1.427592478	0.630184331
A_98763_CCTAATCCAGCCATGCATCATTCC	0.832449053	1.598368022	0.796613241
A_98763_CCTAATCCCGGATTGCCCGAAGTA	0.851280708	0.698609496	0.285010694
A_98763_CCTAATCCGCCACATACAACCACA	0.501320085	0.566966371	-0.02121384
A_98763_CCTCCTGAAATCCGTCATGCCTAA	1.134764063	0.919567401	0.60623209
A_98763_CCTCCTGAGAACAGGCGCGAGTAA	0.421571421	0.338167099	0.288216282
A_98763_CCTCTATCACCACTGTAAGAGATC	0.948792513	1.167688392	-0.080855037
A_98763_CCTCTATCCATACCAATTCACGCA	1.003530319	1.569966122	0.797811515
A_98763_CCTCTATCCCTAATCCCCGAAGTA	0.703109013	0.824610597	0.558516548
A_98763_CCTCTATCGAATCTGATATCAGCA	0.838112609	1.472610278	-0.263004891
A_98763_CGAACTTACGAACTTAGACTAGTA	0.512098177	1.11787305	0.081902049
A_98763_CGACACACACGCTCGAAGCACCTC	0.854372191	1.530997094	0.837651272
A_98763_CGACACACGCTAACGAAGTCACTA	0.570494347	0.471101559	0.069969504
A_98763_CGACACACTGGTGGTACGAACTTA	0.699074221	0.741401539	0.216933249
A_98763_CGACTGGACGACTGGACAGATCTG	0.923343571	1.432285808	0.866839505
A_98763_CGACTGGACTGGCATACGGATTGC	0.932505969	0.996934705	0.008062334
A_98763_CGACTGGAGATGAATCTGGCTTCA	1.149686	1.515767377	0.623106609
A_98763_CGCATACAACAGCAGACAACCACA	1.04752782	1.765368185	0.893954746
A_98763_CGCATACAATGCCTAACTGAGCCA	0.602819411	0.548774938	0.474273846
A_98763_CGCATACACAATGGAAACACAGAA	1.126162727	1.286326207	0.713715001
A_98763_CGCATACACGACACACAACCGAGA	0.480506127	0.283571332	0.701033583
A_98763_CGCATACACGCATACAATTGGCTC	1.04790752	1.816200418	0.076721982
A_98763_CGCATACAGGAGAACACTGGCATA	1.083193243	1.049797539	-0.297206327
A_98763_CGCTGATCAACCGAGAGTCTGTCA	0.825228335	1.775134862	0.667683805
A_98763_CGCTGATCCCGACAACACGCTCGA	1.254463049	1.268299841	0.5859464
A_98763_CGCTGATCGGTGCGAACAGATCTG	0.91923366	1.916489885	1.104228202
A_98763_CGGATTGCCGCTGATCAAGACGGA	1.100144702	1.267816358	0.519902453

A_98763_CTAAGGTCATTGGCTCGGAGAACA	0.531075843	0.387123227	0.360737876
A_98763_CTAAGGTCCATCAAGTCGCATACA	0.387689884	0.396430049	-0.104370927
A_98763_CTAAGGTCGGTGCGAATATCAGCA	0.804156408	1.275127853	0.785019278
A_98763_CTCAATGAATTGAGGACCGAAGTA	1.243586121	1.571976889	0.950565635
A_98763_CTGAGCCACTGGCATAAGCCATGC	1.33149709	1.804342601	0.46684346
A_98763_CTGGCATAAACGTGATTGAAGAGA	0.778452541	1.3602606	0.970007268
A_98763_CTGGCATAACTATGCAGAACAGGC	1.224288026	1.24486448	-0.034220269
A_98763_CTGGCATAAGCACCTCAACGTGAT	1.105994031	1.615601922	0.84009506
A_98763_CTGGCATACAGATCTGGCTAACGA	1.0097126	1.721055496	0.580230586
A_98763_CTGGCATACGACTGGAATCATTCC	0.616614157	0.361677671	0.307537463
A_98763_CTGGCATAGACAGTGCAATGTTGC	0.688597515	1.375860408	0.82628326
A_98763_CTGTAGCCACACGACCACAAGCTA	1.141529928	1.675437765	0.292130795
A_98763_CTGTAGCCCCGAAGTAAGCACCTC	0.499205287	0.078288566	-0.385879305
A_98763_CTGTAGCCCCGTGAGAAGATCGCA	1.20364495	1.593542288	0.133863503
A_98763_GAATCTGAAAGAGATCCGAACTTA	0.453450643	1.312739775	0.324810286
A_98763_GAATCTGAACACAGAAAAACATCG	0.915435232	0.590440749	0.650961896
A_98763_GAATCTGACCTAATCCGAGTTAGC	1.05437929	0.622421523	0.239823419
A_98763_GAATCTGACTGGCATAATTGGCTC	1.387885582	1.431490342	0.600592219
A_98763_GAATCTGAGCCACATAACAAGCTA	0.875942857	1.2649177	0.780069043
A_98763_GACAGTGCAAGGTACAACAGCAGA	0.526410546	1.03340258	0.27149079
A_98763_GACAGTGCCAAGGAGCAAGACGGA	0.677446252	0.531466036	-0.252734274
A_98763_GACAGTGCCAAGGAGCCGCTGATC	1.03440065	1.460440766	1.080726882
A_98763_GAGCTGAAAAGGTACAGAGTTAGC	0.668683303	1.168658006	0.210931358
A_98763_GAGCTGAAAATGTTGCGAACAGGC	0.864928908	1.396111709	0.245063983
A_98763_GAGCTGAAAGCACCTCAACAACCA	0.703567762	1.093349769	0.445042425
A_98763_GAGCTGAACATCAAGTAGCACCTC	0.633083371	1.506976165	0.836253166
A_98763_GAGCTGAACCTAATCCACACAGAA	0.7561994	0.661900777	0.085999258
A_98763_GAGTTAGCAGATGTACCTGGCATA	0.728053842	0.985751642	0.443682105
A_98763_GAGTTAGCGTGTTCTAATCATTCC	0.980580289	1.330023687	0.004176309
A_98763_GATAGACAATAGCGACAAACATCG	0.594373737	0.853149027	0.424787623
A_98763_GATAGACACGGATTGCACCTCCAA	0.539587252	0.830762013	-0.044301253
A_98763_GATGAATCACAGCAGACGACACAC	1.066917079	1.698384288	-0.016500994

A_98763_GATGAATCCGAACTTAAGTGGTCA	0.919958369	1.502472967	0.581400713
A_98763_GCCAAGACAGCAGGAAAGATGTAC	0.714742283	1.668935866	1.062531146
A_98763_GCCAAGACGTACGCAAATTGGCTC	1.099576613	2.142153251	0.906473606
A_98763_GCCACATAATGCCTAAAACGTGAT	1.38434203	1.551450219	0.624986504
A_98763_GCTAACGAAGATCGCAGTACGCAA	1.115135167	1.877901143	0.995820758
A_98763_GCTAACGAGAACAGGCCCTAATCC	1.078781918	1.414578859	0.32991061
A_98763_GCTAACGAGCTCGGTACTGGCATA	0.491770662	0.265527904	0.236404406
A_98763_GCTCGGTACAAGGAGCAAGGTACA	1.26983312	1.540062893	0.933377683
A_98763_GGAGAACAACATTGGCATTGGCTC	0.415162381	0.283432935	0.415043213
A_98763_GGTGCGAACCTAATCCATCATTCC	0.900737252	1.366271394	0.530414889
A_98763_GTACGCAACATCAAGTCTCAATGA	0.205605118	0.140624346	0.263215873
A_98763_GTACGCAATAGGATGACTCAATGA	0.828573319	0.794547378	0.598389052
A_98763_GTCGTAGAACCTCCAAATCCTGTA	0.541990093	0.17657038	-0.002199708
A_98763_GTCGTAGACGAACTTAAGTCACTA	0.855920002	1.86674416	0.951432072
A_98763_GTCGTAGACTGTAGCCATGCCTAA	0.853648484	2.137340112	0.759701414
A_98763_GTCTGTCAAGAGTCAAGCCACATA	0.698728683	0.826827325	0.447459402
A_98763_GTCTGTCAGACTAGTAAGCAGGAA	0.904452444	1.238994745	0.34911863
A_98763_GTCTGTCAGGAGAACAAAGAGATC	0.942487714	1.346185593	0.227917896
A_98763_GTCTGTCATCCGTCTAGTCGTAGA	0.286604478	1.048735174	0.0356804
A_98763_GTGTTCTAAGAGTCAACAGATCTG	1.005268379	0.914710316	0.611532699
A_98763_GTGTTCTAAGATCGCACCTCCTGA	0.326894753	0.314503358	0.229188977
A_98763_TAGGATGAAAGGACACCTAAGGTC	1.229930023	1.740316759	0.45338484
A_98763_TAGGATGAAATGTTGCCCGAAGTA	0.664730645	0.544253647	0.019759482
A_98763_TATCAGCAAACGTGATCTGAGCCA	0.686265968	1.616666006	0.319303707
A_98763_TATCAGCAAACTCACCCCATCCTC	1.143739008	1.862480371	0.31121071
A_98763_TATCAGCAGACAGTGCCCGTGAGA	0.991343492	1.11442175	0.34144555
A_98763_TATCAGCAGTGTTCTATATCAGCA	0.658099844	0.104946129	-0.465074894
A_98763_TCCGTCTAAGCAGGAAAAGAGATC	0.871765665	1.693822176	0.752226025
A_98763_TCCGTCTATCCGTCTAACATTGGC	1.526477244	1.292805559	0.314470656
A_98763_TCTTCACAATAGCGACTGGTGGTA	1.156870739	0.787212228	0.603050749
A_98763_TCTTCACACACTTCGAGAGTTAGC	0.82582994	1.318611934	0.860003088
A_98763_TCTTCACATTCACGCAACCTCCAA	0.20481848	0.238930036	0.288565021

A_98763_TGAAGAGACGGATTGCACATTGGC	1.14646279	1.54316682	0.322394136
A_98763_TGAAGAGAGAATCTGAATAGCGAC	0.731819647	1.469298745	0.442087622
A_98763_TGAAGAGAGAGCTGAACACTTCGA	1.101334893	1.129095308	0.600807975
A_98763_TGGAACAAAAACATCGTGAAGAGA	0.589370552	0.341284838	0.003655844
A_98763_TGGAACAACCAGTTCAAGCAGGAA	0.968218666	0.866248593	-0.237897268
A_98763_TGGAACAACGCATACAAACCGAGA	0.859371234	1.212396712	0.090738235
A_98763_TGGAACAACTGGCATAACCTCCAA	0.927207055	1.715721199	0.635108949
A_98763_TGGTGGTAACAAGCTACCATCCTC	0.630916111	0.518521652	0.345134148
A_98763_TGGTGGTAATCCTGTACCTCTATC	0.561044695	0.778076766	0.109241783
A_98763_TGGTGGTACCTAATCCAACCGAGA	0.342274801	0.51642858	0.53188906
A_98763_TGGTGGTATGAAGAGACATCAAGT	0.457668313	0.384034204	0.173306168
A_98763_TTCACGCAAGCAGGAAATCATTCC	1.457186942	1.662063217	0.331385218
B_98618_AACGCTTAATCATTCCATCATTCC	0.207051761	0.091499183	-0.482986614
B_98618_AACGCTTACTGTAGCCATGCCTAA	0.297716399	0.051572832	-0.38837926
B_98618_AAGACGGAAATGTTGCAGAGTCAA	0.140856224	0.22208759	-0.073665817
B_98618_AAGGTACACGCATACAGCGAGTAA	0.214623737	0.232861041	-0.203098579
B_98618_AATGTTGCAGGCTAACGCGAGTAA	0.166655738	0.030867158	-0.519067943
B_98618_ACAAGCTAACCTCCAACATCAAGT	-0.008666356	0.040654778	0.057578658
B_98618_ACACAGAAAAGAGATCCAAGACTA	0.265137261	0.071231589	-0.367235578
B_98618_ACACAGAAGAGTTAGCCCTCCTGA	0.053700659	-0.003074429	-0.296418786
B_98618_ACATTGGCGCCACATAGATGAATC	-0.003121184	0.109858032	-0.255904447
B_98618_AGAGTCAACCTCCTGAAGGCTAAC	-0.083937418	0.087424247	-0.520009771
B_98618_AGGCTAACAGTACAAGCCTCCTGA	0.059899506	0.096848089	-0.302866408
B_98618_AGTCACTAATCCTGTACAATGGAA	0.066771561	0.170968758	-0.123691787
B_98618_AGTCACTAGCCACATACGGATTGC	0.238202156	0.208023755	-0.43305582
B_98618_ATAGCGACCCGACAACGCGAGTAA	-0.019625807	0.072996799	-0.1404421
B_98618_ATAGCGACGCGAGTAAGAGTTAGC	0.187728878	0.14029482	-0.344010972
B_98618_ATGCCTAAACAAGCTAAGGCTAAC	0.08132092	0.095831787	-0.362512643
B_98618_ATTGAGGAAGATCGCAACGCTCGA	0.373164869	0.116007943	-0.210942419
B_98618_ATTGAGGAGTCTGTCAGATGAATC	0.181645459	0.226084886	-0.284005987
B_98618_ATTGGCTCAACGCTTAACAGATTC	0.097779862	0.086874308	-0.326697434
B_98618_ATTGGCTCACAGATTCGCTAACGA	0.021664645	0.205889707	-0.418161627

B_98618_CAGCGTTACGGATTGCCACCTTAC	-0.017010902	0.139492461	-0.333302405
B_98618_CCAGTTCAAACGTGATGGTGCGAA	0.21342408	0.098358707	-0.244975796
B_98618_CGAACTTAAAGGACACAACGCTTA	0.057839197	0.085992163	-0.478646863
B_98618_CGACTGGAAACGCTTACCTAATCC	0.006353501	0.02376178	-0.506403789
B_98618_CGCTGATCCGACACACCCATCCTC	0.271550109	0.168222942	-0.283016471
B_98618_GAATCTGAATTGGCTCATTGAGGA	-0.018864377	0.015974748	-0.237850659
B_98618_GACTAGTAAGAGTCAAAAACATCG	0.178443935	0.154996437	-0.155966727
B_98618_GAGCTGAAGTACGCAAAGTGGTCA	0.053318931	0.191984002	-0.228272504
B_98618_GCGAGTAAAACCGAGAGATGAATC	-0.202415545	0.182044769	-0.371750661
B_98618_GCTAACGAGATGAATCACATTGGC	0.351123592	0.077213384	-0.280373714
B_98618_GTCTGTCACAAGACTACCGTGAGA	0.470833752	0.2171069	-0.297871758
B_98618_TCCGTCTAAATGTTGCGCTCGGTA	-0.057163186	0.029835238	-0.339594486
B_98618_TGGAACAACCATCCTCAGCACCTC	-0.214491397	0.039601695	-0.113483593
B_98618_TGGCTTCAAAACATCGGTCGTAGA	0.193322509	0.271266259	-0.192698702
B_98618_TTCACGCATTCACGCAGTACGCAA	0.164796141	0.095630879	-0.318725474
B_98618_AAACATCGAAGGTACAGAATCTGA	0.458702906	0.391756453	-0.278761216
B_98618_AAACATCGGATGAATCAAGAGATC	0.358235867	0.306920649	-0.263373378
B_98618_AAACATCGTATCAGCAGCTCGGTA	0.419494917	0.493342443	-0.195250297
B_98618_AACAACCAAACGTGATAGATCGCA	0.522925476	0.276306531	-0.502267672
B_98618_AACAACCACATCAAGTGACAGTGC	0.210642269	0.288675491	-0.262307028
B_98618_AACCGAGAAACGCTTAGATAGACA	0.351245532	0.25758975	-0.33618517
B_98618_AACCGAGAAGATCGCAAATGTTGC	-0.04245493	0.183517376	-0.314098394
B_98618_AACCGAGAAGATCGCAACAGATTC	0.266253787	0.152222396	-0.560626904
B_98618_AACCGAGAAGTCACTAAGTACAAG	0.59805735	0.489712694	-0.389423299
B_98618_AACCGAGAGACAGTGCCTAAGGTC	0.182492358	0.186348669	-0.131683831
B_98618_AACCGAGAGACTAGTAAGCACCTC	0.377907016	0.301410576	-0.393974096
B_98618_AACCGAGAGCTAACGAACCTCCAA	0.31918281	0.395587416	-0.321555879
B_98618_AACCGAGAGTCGTAGACCGAAGTA	0.666640535	0.222890852	-0.340904824
B_98618_AACGCTTAATTGGCTCCATACCAA	0.097903071	0.240509218	-0.551863323
B_98618_AACGCTTACAACCACAAACAACCA	0.129989281	0.205795728	-0.506930183
B_98618_AACGCTTACTGGCATACATCAAGT	0.457556424	0.277307303	-0.259074133
B_98618_AACGCTTACTGGCATACCGACAAC	0.459726377	0.143675642	-0.504519119

B_98618_AACGCTTATATCAGCAGAGCTGAA	0.27323784	0.415669804	-0.50026235
B_98618_AACGTGATCAAGACTAACTATGCA	0.341733543	0.36959612	-0.373862828
B_98618_AACGTGATCTGAGCCAGAGTTAGC	0.380394474	0.064237211	-0.428481273
B_98618_AACGTGATGATAGACATTCACGCA	1.091765681	0.343683256	-0.419262943
B_98618_AACTCACCGCCAAGACCTGTAGCC	0.216984861	0.385967957	0.166711279
B_98618_AACTCACCGCCAAGACTCTTCACA	0.384117164	0.233682118	-0.59508668
B_98618_AACTCACCGCGAGTAACCTCCTGA	0.223090511	0.168927987	-0.560209662
B_98618_AACTCACCTCCGTCTACCTCTATC	0.400794088	0.262446709	-0.536983919
B_98618_AAGACGGAAATGTTGCATTGGCTC	0.49585417	0.219597604	-0.531770275
B_98618_AAGACGGAACCACTGTGCTAACGA	0.41133936	0.177719028	-0.613475976
B_98618_AAGACGGAATCCTGTAAAGGTACA	0.298957636	0.234448947	-0.382164168
B_98618_AAGACGGACAAGACTACGCTGATC	0.424974301	0.323439597	-0.179005281
B_98618_AAGACGGACCATCCTCGCTAACGA	0.247494303	0.1531764	-0.446051567
B_98618_AAGAGATCAAGAGATCGTGTTCTA	0.439532845	0.328871183	-0.583071649
B_98618_AAGAGATCAAGGACACCGAACTTA	0.103365751	0.288772161	-0.266892601
B_98618_AAGAGATCAATCCGTCTTCACGCA	0.273820059	0.175791441	-0.43001251
B_98618_AAGAGATCAGCCATGCTGGAACAA	0.439965528	0.322758541	0.003489636
B_98618_AAGAGATCGGAGAACACGACACAC	0.463520669	0.240354483	-0.385782086
B_98618_AAGGACACCGCATACACGACACAC	0.050484826	0.272540295	-0.069616886
B_98618_AAGGACACGCTAACGACCTCTATC	0.54709604	0.3714575	-0.386128989
B_98618_AAGGACACGTCTGTCAATCCTGTA	0.259624765	0.190656825	-0.545096352
B_98618_AAGGTACAACACGACCTTCACGCA	0.147999614	0.250442837	-0.036500834
B_98618_AAGGTACAAGTACAAGGGAGAACA	0.488633587	0.188535696	-0.391399499
B_98618_AAGGTACACCAGTTCAAGTACAAG	0.513464118	0.391059254	-0.359093497
B_98618_AAGGTACACCAGTTCAGACTAGTA	0.670535872	0.674127984	-0.139744256
B_98618_AAGGTACAGACTAGTAACACAGAA	0.448359916	0.456899684	-0.574728575
B_98618_AAGGTACAGTGTTCTACAACCACA	0.382633247	0.180230629	-0.517254898
B_98618_AATCCGTCAATCCGTCCGACTGGA	0.367687383	0.20877934	-0.157823752
B_98618_AATCCGTCAGCACCTCATCCTGTA	0.795890952	0.326198023	-0.397854413
B_98618_AATCCGTCCAAGACTATATCAGCA	0.435126028	0.436473856	-0.268155813
B_98618_AATCCGTCGGTGCGAATATCAGCA	0.536318389	0.588125362	-0.37351156
B_98618_AATCCGTCTCTTCACAGTACGCAA	0.365035163	0.260893129	-0.144132583

B_98618_AATGTTGCAACCGAGAATTGGCTC	0.46208806	0.474171428	-0.219213699
B_98618_AATGTTGCAGTACAAGAGCCATGC	0.416068084	0.343927803	-0.1365636
B_98618_AATGTTGCATTGAGGACGCATACA	0.093548328	0.187696829	-0.55572493
B_98618_AATGTTGCGCCAAGACCGACTGGA	0.540138279	0.321229743	-0.322071455
B_98618_AATGTTGCGCTAACGAAGTACAAG	0.644918064	0.566973713	-0.366800946
B_98618_AATGTTGCGTGTTCTAAAACATCG	0.446187759	0.236818893	-0.458722741
B_98618_ACAAGCTAAACAACCATGGCTTCA	0.193460844	0.15788836	-0.351852212
B_98618_ACAAGCTAACACAGAACTGAGCCA	0.357802403	0.099480649	-0.506006738
B_98618_ACAAGCTAACTATGCAACATTGGC	0.266045907	0.199054816	-0.378201588
B_98618_ACAAGCTAAGATCGCAACCTCCAA	0.621774599	0.525871186	-0.430073518
B_98618_ACAAGCTACCGAAGTACCTCCTGA	0.450431086	0.055883102	-0.557599864
B_98618_ACAAGCTACGGATTGCCAGATCTG	0.587268358	0.461763869	-0.193027429
B_98618_ACAAGCTACTAAGGTCCCTAATCC	0.11652724	0.290123215	-0.389129412
B_98618_ACAAGCTAGTGTTCTACGACTGGA	0.389158929	0.169104018	-0.52715121
B_98618_ACACAGAAAAGGTACAATTGAGGA	0.032130969	0.416463981	-0.407407254
B_98618_ACACAGAAATGCCTAAATGCCTAA	0.285865234	0.187564569	-0.117161296
B_98618_ACACAGAACTCAATGACTAAGGTC	0.156995242	0.137458275	-0.246273168
B_98618_ACACAGAACTGGCATAAGCACCTC	0.249101426	0.031086851	-0.573562027
B_98618_ACACAGAAGCCAAGACCAATGGAA	1.204370406	0.522408548	-0.605284711
B_98618_ACACAGAAGGAGAACACCAGTTCA	0.200128962	0.165212785	-0.515761334
B_98618_ACACGACCAGATCGCACTCAATGA	0.513413171	0.065212725	-0.32167045
B_98618_ACACGACCGATGAATCCGACACAC	0.180384594	0.08759193	-0.417759402
B_98618_ACACGACCTCCGTCTACCGAAGTA	0.42814265	0.120682127	-0.517601313
B_98618_ACAGATTCAAGGACACACATTGGC	0.193751171	0.298360957	-0.305063304
B_98618_ACAGATTCACATTGGCACACAGAA	0.271988492	0.245101442	-0.487490779
B_98618_ACAGATTCCCGAAGTACTGAGCCA	0.224706584	0.345183066	-0.430247156
B_98618_ACAGATTCGCTCGGTACGCATACA	0.460915375	0.292169147	-0.281989827
B_98618_ACAGCAGAACGCTCGAAATGTTGC	0.28301857	0.210335476	-0.295803294
B_98618_ACAGCAGACAGCGTTACAATGGAA	0.100498266	0.095077708	-0.458479638
B_98618_ACAGCAGACGAACTTAAGCAGGAA	0.55061343	0.345822637	-0.278317701
B_98618_ACAGCAGACTGAGCCACGCTGATC	0.475948224	0.481077238	-0.300551347
B_98618_ACATTGGCACGCTCGAAAGGTACA	0.22936552	0.122581921	-0.439824143

B_98618_ACATTGGCCCAGTTCACAATGGAA	0.253345102	0.093416504	-0.536666066
B_98618_ACCACTGTCCGACAACCCATCCTC	0.246482765	0.198506592	-0.522603091
B_98618_ACCACTGTCTGTAGCCGACTAGTA	0.041073312	0.265571554	0.274338963
B_98618_ACCACTGTGACTAGTACAATGGAA	0.107013476	0.151068288	-0.266421391
B_98618_ACCACTGTGTCTGTCAGAATCTGA	0.241097229	0.421130318	-0.237679765
B_98618_ACCTCCAAACACAGAAGTACGCAA	0.228568443	0.384438071	-0.555251867
B_98618_ACCTCCAAGCCACATACATACCAA	0.563948264	0.248805463	-0.309686136
B_98618_ACCTCCAAGCGAGTAAAGCACCTC	0.403678155	0.546761443	-0.296639821
B_98618_ACGTATCAAGTGGTCAAGTCACTA	0.286947941	0.302543365	-0.209318006
B_98618_ACGTATCACCGAAGTAACGCTCGA	0.372309093	0.42542494	-0.521736633
B_98618_ACGTATCACGCATACACACCTTAC	0.407555988	0.150481258	-0.365168585
B_98618_ACGTATCATAGGATGAATCCTGTA	0.415217633	0.205737184	-0.476664213
B_98618_ACGTATCATCTTCACACACTTCGA	0.200565015	0.290586555	-0.320313664
B_98618_ACTATGCAAACCGAGAAAGGTACA	0.863277235	0.624067839	-0.47511378
B_98618_ACTATGCAAAGGACACAGCCATGC	0.26026464	0.610190086	0.029714234
B_98618_ACTATGCAACAAGCTATCTTCACA	0.190316747	0.368767071	-0.300870298
B_98618_ACTATGCAACAGCAGAGATAGACA	0.510686734	0.268500153	-0.468956532
B_98618_ACTATGCAACATTGGCGCTAACGA	0.047603809	0.384457662	-0.429806993
B_98618_ACTATGCACAGCGTTACTAAGGTC	0.110683655	0.21202768	-0.303597952
B_98618_ACTATGCATAGGATGAGAATCTGA	0.373776797	0.383494424	-0.2524654
B_98618_AGAGTCAAAACGCTTAACGCTCGA	0.210044023	0.222102301	-0.449393276
B_98618_AGAGTCAAACACAGAAGACTAGTA	0.349558258	0.30536574	-0.378952568
B_98618_AGAGTCAAACAGATTCGAGCTGAA	0.412947819	0.381973606	0.032841393
B_98618_AGAGTCAACAAGACTAACCTCCAA	0.143197008	0.180122887	-0.299216445
B_98618_AGAGTCAACCGAAGTAAACAACCA	0.338432536	0.413093463	-0.302076065
B_98618_AGAGTCAACCGTGAGACGAACTTA	0.167935943	0.067517928	-0.53367649
B_98618_AGAGTCAACGCATACAATGCCTAA	0.328219746	0.283066236	-0.500450237
B_98618_AGAGTCAAGAACAGGCGCCACATA	0.469273841	0.355972517	-0.130137947
B_98618_AGAGTCAAGGTGCGAATCCGTCTA	0.395194785	0.348399458	-0.236958849
B_98618_AGAGTCAATGGAACAAAACCGAGA	0.149313595	0.342148468	-0.283106801
B_98618_AGATCGCAACATTGGCCAACCACA	0.249779358	0.239705229	-0.508922607
B_98618_AGATCGCACAGCGTTAGATAGACA	0.876579312	0.26356065	-0.360767334

B_98618_AGATCGCACGCATACATCTTCACA	0.164583028	0.400595655	-0.248613861
B_98618_AGATCGCAGACTAGTAGGTGCGAA	0.430228702	0.446965793	-0.227165868
B_98618_AGATGTACACAGATTCACAGCAGA	0.634192552	0.458498818	-0.195943576
B_98618_AGATGTACAGCAGGAAGATGAATC	-0.058702626	0.253481753	-0.422938072
B_98618_AGATGTACCCATCCTCCGACACAC	0.325628235	0.167388327	-0.254738945
B_98618_AGCACCTCACGCTCGAGAACAGGC	0.456396797	0.28771754	-0.369672513
B_98618_AGCACCTCCGCATACACTAAGGTC	0.270851298	0.144059127	-0.104261229
B_98618_AGCACCTCCTGTAGCCGCCAAGAC	0.6611718	0.317336746	-0.471211021
B_98618_AGCACCTCGAATCTGAACACGACC	0.422207887	0.450467287	-0.072642361
B_98618_AGCACCTCTGGCTTCATGGCTTCA	0.311070306	0.379462589	-0.164829816
B_98618_AGCAGGAAACTATGCAAACCGAGA	0.118770569	0.276395088	-0.269656127
B_98618_AGCAGGAAAGATCGCACAGATCTG	0.124916228	0.310220169	-0.464356697
B_98618_AGCAGGAACATCAAGTAGCCATGC	0.544146854	0.387300181	-0.250503169
B_98618_AGCAGGAACCTCCTGAGGAGAACA	0.130589074	0.389564516	-0.106548262
B_98618_AGCAGGAACTAAGGTCAGATGTAC	0.45593297	0.326986251	-0.19405913
B_98618_AGCAGGAAGAGTTAGCAGTGGTCA	0.212074181	0.198138384	-0.214282616
B_98618_AGCAGGAATGGAACAATGGCTTCA	0.851541411	0.410941868	-0.215009929
B_98618_AGCCATGCAATGTTGCCAGCGTTA	0.136917553	0.37583454	-0.376713214
B_98618_AGCCATGCGATAGACAAGATCGCA	0.201221405	0.299885242	-0.330027087
B_98618_AGGCTAACAATCCGTCACTATGCA	0.474506743	0.329786668	-0.301053942
B_98618_AGGCTAACATGCCTAAGTCGTAGA	0.441802866	0.16889727	-0.592634529
B_98618_AGGCTAACCGACTGGAGAACAGGC	0.305563024	0.278451531	-0.384427265
B_98618_AGGCTAACGGTGCGAAAACGTGAT	0.434353998	0.323810006	-0.337459187
B_98618_AGTACAAGAGCACCTCCGCATACA	0.42200542	0.395480197	-0.067889008
B_98618_AGTACAAGCCGACAACCCATCCTC	0.649886044	0.278328327	-0.52561542
B_98618_AGTACAAGGACAGTGCGCTCGGTA	0.507463146	0.574340953	-0.298786769
B_98618_AGTACAAGGCTCGGTACGACACAC	0.616704503	0.138921965	-0.358790751
B_98618_AGTACAAGGTACGCAACAACCACA	0.387788508	0.180961643	-0.569463406
B_98618_AGTACAAGTCCGTCTAACTATGCA	-0.024304005	0.166568998	-0.301808066
B_98618_AGTCACTAATTGGCTCTGAAGAGA	0.431345081	0.293280156	-0.565918214
B_98618_AGTCACTACACCTTACTAGGATGA	0.231638703	0.291267815	-0.238544763
B_98618_AGTCACTACTGTAGCCAGTACAAG	0.050834534	0.110237852	-0.401148467

B_98618_AGTCACTATATCAGCACGACACAC	0.453353447	0.25950221	-0.473712694
B_98618_AGTGGTCAAATCCGTCAATGTTGC	0.338413841	0.44423009	-0.168733372
B_98618_AGTGGTCAACGTATCAACGTATCA	0.57294103	0.353642353	-0.474444711
B_98618_AGTGGTCAATCATTCCAATCCGTC	0.401580997	0.26293869	-0.319927808
B_98618_AGTGGTCATATCAGCAAGTACAAG	0.289798393	0.221287463	-0.381299277
B_98618_ATAGCGACAATCCGTCAAGAGATC	0.138509241	0.066811462	-0.595839889
B_98618_ATAGCGACCCGAAGTAAACAACCA	0.03299277	0.246008847	-0.474986203
B_98618_ATAGCGACCCTCTATCTAGGATGA	0.802234211	0.538545398	-0.252521382
B_98618_ATAGCGACCTAAGGTCAAGGACAC	0.430739346	0.165362934	-0.530483419
B_98618_ATAGCGACGAACAGGCACGCTCGA	0.440032271	0.21716783	-0.577648325
B_98618_ATAGCGACGATGAATCTGGCTTCA	0.497223768	0.202067686	-0.577220175
B_98618_ATCATTCCAACCGAGAACAAGCTA	0.471123871	0.474281762	-0.186409586
B_98618_ATCATTCCAGAGTCAAGGTGCGAA	0.090956965	0.218999676	-0.452331448
B_98618_ATCATTCCCAGATCTGAAGAGATC	0.564588014	0.491496723	-0.250252914
B_98618_ATCCTGTAAACCGAGAAGATCGCA	0.486097478	0.210389144	-0.259530291
B_98618_ATCCTGTAACGCTCGAGAGTTAGC	0.087025576	0.07900518	-0.303229216
B_98618_ATCCTGTACAGATCTGAGTGGTCA	0.539006034	0.291602817	-0.424954087
B_98618_ATCCTGTACCATCCTCACATTGGC	0.64504388	0.175011196	-0.421789705
B_98618_ATCCTGTAGACTAGTAAAACATCG	0.384796993	0.238919277	-0.307165774
B_98618_ATGCCTAAAACAACCAGTCGTAGA	0.454651094	0.364465723	-0.34659136
B_98618_ATGCCTAAGAACAGGCAACGTGAT	0.319129199	0.304222252	-0.151619778
B_98618_ATGCCTAATGAAGAGAGAACAGGC	0.241853777	0.138054884	-0.225200297
B_98618_ATTGAGGAACGCTCGAGTCGTAGA	0.523453726	0.36236595	-0.425439079
B_98618_ATTGAGGAGGAGAACACAAGACTA	0.570312601	0.258651696	-0.109015672
B_98618_ATTGGCTCAAGGACACTGGCTTCA	0.761444609	0.312532976	-0.230891966
B_98618_ATTGGCTCAATGTTGCCCATCCTC	0.503115313	0.361655516	-0.151425878
B_98618_ATTGGCTCATGCCTAAACACGACC	0.231043054	0.142509523	-0.42313251
B_98618_ATTGGCTCCTGAGCCACTGTAGCC	0.260091284	0.270594069	-0.218660758
B_98618_ATTGGCTCGTCTGTCACATACCAA	0.279507672	0.186848473	-0.189868876
B_98618_ATTGGCTCTAGGATGAAAGGACAC	0.486183467	0.145928124	-0.301268344
B_98618_CAACCACAAAGACGGAAACAACCA	0.415146935	0.257404705	-0.472605177
B_98618_CAAGACTAAAACATCGGAGTTAGC	0.709100733	0.419763693	-0.274075259

B_98618_CAAGACTAAAGGACACCACTTCGA	0.260244385	0.344010974	-0.443723354
B_98618_CAAGACTAACACGACCCGACACAC	0.144092058	0.214364618	-0.403025381
B_98618_CAAGACTAAGCCATGCGACTAGTA	0.506078001	0.312994222	-0.29306151
B_98618_CAAGACTAGACTAGTAACAAGCTA	0.545500664	0.418166747	-0.232815131
B_98618_CAAGACTAGACTAGTACTGGCATA	1.208087793	0.416847009	-0.453077828
B_98618_CAAGACTATCTTCACAACAGCAGA	0.183313098	0.27975306	-0.08928784
B_98618_CAAGGAGCAATCCGTCTGGCTTCA	0.106175159	0.179391142	-0.329476085
B_98618_CAAGGAGCAGTACAAGCCGAAGTA	0.260832759	0.248801048	-0.378307374
B_98618_CAAGGAGCAGTGGTCAGACTAGTA	0.291025982	0.279064193	-0.254024674
B_98618_CAAGGAGCCTCAATGAGAGTTAGC	0.247756579	0.229203216	-0.252174189
B_98618_CAAGGAGCGTCGTAGAATCATTCC	0.239791919	0.147616981	-0.561244352
B_98618_CAATGGAAAACAACCAGCGAGTAA	0.330702004	0.175318499	-0.367646828
B_98618_CAATGGAAACATTGGCCTGTAGCC	0.55343462	0.445168457	-0.213669282
B_98618_CAATGGAACAACCACACTGTAGCC	0.004215289	0.030347063	-0.548538786
B_98618_CAATGGAACCTAATCCTTCACGCA	0.281767467	0.311626922	-0.51966903
B_98618_CAATGGAACTGAGCCAAAGGTACA	0.399547756	0.200628217	-0.235287977
B_98618_CAATGGAATCTTCACAGAGCTGAA	0.22330257	0.152968028	-0.466670464
B_98618_CACCTTACCCAGTTCAGACAGTGC	0.655571681	0.383110527	-0.265569665
B_98618_CACCTTACCTGAGCCAAACGTGAT	0.30141646	0.359382835	-0.165673019
B_98618_CACTTCGAAAACATCGCCTAATCC	0.415112568	0.406658376	-0.121415514
B_98618_CACTTCGAAACAACCAACCTCCAA	0.376225824	0.181395678	-0.426940934
B_98618_CACTTCGAAACTCACCCGAACTTA	0.44260986	0.086560389	-0.502916735
B_98618_CACTTCGAAAGGACACGAGCTGAA	-0.064251468	0.253516805	-0.344451878
B_98618_CACTTCGAACCACTGTAACCGAGA	0.708793527	0.561303086	-0.235618147
B_98618_CACTTCGACCGACAACGTCGTAGA	0.242011818	0.250152687	-0.499527746
B_98618_CACTTCGACCGTGAGAAAGGTACA	0.323216665	0.207084922	-0.533203913
B_98618_CACTTCGAGCTCGGTAAAGAGATC	0.347422634	0.084023436	-0.445703943
B_98618_CACTTCGAGGAGAACAAGTGGTCA	0.34804135	0.153643067	-0.474310918
B_98618_CAGATCTGAACGCTTAACACGACC	0.391050214	0.149771798	-0.490890252
B_98618_CAGATCTGCAGCGTTACCATCCTC	0.174985271	0.19919949	-0.281160826
B_98618_CAGATCTGGGTGCGAACTGGCATA	0.354816593	0.064402429	-0.434411553
B_98618_CAGATCTGGTCTGTCAAATGTTGC	0.416385865	0.362514849	-0.299291504

B_98618_CAGCGTTACATACCAAAGATCGCA	0.236445036	0.268104732	-0.398819245
B_98618_CAGCGTTACTGTAGCCCCTAATCC	0.188941008	0.601194216	-0.290291633
B_98618_CAGCGTTAGACTAGTACAAGGAGC	0.147227703	0.203740108	-0.497086356
B_98618_CATACCAAAAGAGATCGTCTGTCA	0.158883735	0.125143195	-0.439793926
B_98618_CATACCAAACCACTGTGACTAGTA	0.424027852	0.262166038	-0.437543938
B_98618_CATACCAAAGTACAAGGGTGCGAA	1.130109164	0.310179267	-0.562725475
B_98618_CATACCAAGACAGTGCACCTCCAA	0.307369334	0.338910805	0.119269403
B_98618_CATACCAAGCCACATAAACGCTTA	0.198228492	0.17140454	0.143001599
B_98618_CATCAAGTAAGGTACAAACGCTTA	0.399648572	0.215900485	-0.273217173
B_98618_CATCAAGTAGAGTCAAAACGTGAT	0.24599988	0.142501574	-0.281453003
B_98618_CATCAAGTATTGGCTCAAGAGATC	0.458843768	0.142078604	-0.564214653
B_98618_CATCAAGTATTGGCTCGAGCTGAA	0.21024525	0.137286166	-0.459933558
B_98618_CATCAAGTCACTTCGACACTTCGA	-0.025948626	0.065761488	-0.376648432
B_98618_CATCAAGTCTAAGGTCATGCCTAA	0.431315553	0.374992458	-0.332363844
B_98618_CCAGTTCAATAGCGACCACTTCGA	0.349693569	0.307504207	-0.3161103
B_98618_CCAGTTCACACCTTACAAACATCG	0.187741563	0.235729226	-0.233869275
B_98618_CCAGTTCACTAAGGTCTCCGTCTA	1.227809114	0.509710304	-0.524062912
B_98618_CCAGTTCAGCTAACGAACACAGAA	0.314910478	0.301673146	-0.474728523
B_98618_CCATCCTCATAGCGACCGCATACA	-0.049580229	0.058497564	-0.389117709
B_98618_CCATCCTCGAGCTGAACGACTGGA	0.372308615	0.147865978	-0.234754539
B_98618_CCGAAGTAACGCTCGAAATCCGTC	0.031153829	0.082952706	-0.378874891
B_98618_CCGAAGTAATCATTCCATGCCTAA	0.084426848	0.218988942	-0.44555937
B_98618_CCGAAGTATAGGATGACAATGGAA	0.103773844	0.090385241	-0.354653218
B_98618_CCGACAACAACTCACCGTCGTAGA	0.206437284	0.154766634	-0.444772361
B_98618_CCGACAACACACGACCACAGCAGA	0.91031341	0.214823617	-0.636814685
B_98618_CCGACAACAGCAGGAAGATGAATC	0.5028508	0.220151447	-0.485318817
B_98618_CCGACAACATAGCGACCCATCCTC	0.169774695	0.190307728	-0.358499773
B_98618_CCGACAACGCCACATACAGATCTG	0.16857296	0.207671559	-0.381992103
B_98618_CCGTGAGAAACGTGATTTCACGCA	0.520486846	0.401071267	-0.147313945
B_98618_CCGTGAGAAGCACCTCAAGGTACA	0.640222898	0.522485284	-0.286112878
B_98618_CCGTGAGAAGCCATGCGAACAGGC	0.587485403	0.595289449	-0.354133625
B_98618_CCGTGAGACAACCACAGTCGTAGA	0.25513158	0.262481076	-0.412586666

B_98618_CCGTGAGACAAGACTACGCTGATC	0.170350037	0.111078949	-0.597918109
B_98618_CCGTGAGACCGACAACCAAGACTA	0.440653366	0.272355321	-0.279537784
B_98618_CCGTGAGACGCTGATCCAAGGAGC	0.451035564	0.339878133	-0.287499703
B_98618_CCGTGAGAGATAGACAGTACGCAA	0.266652283	0.616999451	-0.280486142
B_98618_CCGTGAGAGCCACATAGATAGACA	0.15350139	0.22556812	-0.117216941
B_98618_CCGTGAGATCTTCACAAGATGTAC	0.535251131	0.373993423	-0.470298122
B_98618_CCGTGAGATGAAGAGAATCCTGTA	1.022175767	0.547071309	-0.335221852
B_98618_CCTAATCCGACTAGTACGGATTGC	0.068408683	0.189999889	-0.002621974
B_98618_CCTCCTGAAACCGAGACCAGTTCA	0.331062256	0.4058417	-0.427468359
B_98618_CCTCCTGAAATCCGTCAGTCACTA	0.442957061	0.198624954	-0.619469346
B_98618_CCTCCTGAACGCTCGATTCACGCA	0.097577467	0.283746531	-0.265561931
B_98618_CCTCCTGATTCACGCATGGTGGTA	0.116228258	0.160328926	-0.175659564
B_98618_CCTCTATCAACTCACCCGCTGATC	0.246014053	0.293329865	-0.433903942
B_98618_CCTCTATCAAGAGATCAGTGGTCA	0.13194171	0.197905162	-0.366820851
B_98618_CCTCTATCAAGAGATCTAGGATGA	0.529008167	0.29700862	-0.426928496
B_98618_CCTCTATCACGCTCGAAAGGACAC	0.100612544	0.133850484	-0.481845502
B_98618_CCTCTATCACGTATCAACAGCAGA	0.272090412	0.175629729	-0.292291482
B_98618_CCTCTATCCCTCCTGACACCTTAC	0.168832172	0.333419772	-0.255332988
B_98618_CCTCTATCCTGGCATAACACAGAA	0.067906496	0.074664384	-0.483063909
B_98618_CCTCTATCCTGTAGCCACACGACC	0.372613384	0.315173371	-0.376178072
B_98618_CCTCTATCGCCAAGACGTCGTAGA	0.713036814	0.328551501	-0.247352729
B_98618_CGAACTTAAAACATCGACAGATTC	0.380800057	0.295686932	-0.243720682
B_98618_CGAACTTAAGCAGGAAAAGGACAC	0.263002687	0.166007764	-0.46733715
B_98618_CGAACTTAATGCCTAAACCTCCAA	0.582824688	0.340281724	-0.212594298
B_98618_CGAACTTAGCTAACGAAGAGTCAA	1.435031887	0.507767163	-0.360181329
B_98618_CGACACACAAGAGATCAAGGACAC	0.206500858	0.249361444	-0.427951793
B_98618_CGACACACAAGAGATCGATAGACA	0.06495412	0.11769257	-0.522386576
B_98618_CGACACACACTATGCACACCTTAC	0.418989169	0.316602173	-0.325825331
B_98618_CGACACACCAAGGAGCGAATCTGA	0.648052012	0.37827183	-0.367300776
B_98618_CGACACACCCGTGAGAACGCTCGA	0.28739183	0.209098636	-0.127790225
B_98618_CGACACACGATGAATCGAATCTGA	0.306191421	0.371331464	-0.245960564
B_98618_CGACACACGTACGCAACTGTAGCC	0.835292928	0.25846512	-0.372473329

B_98618_CGACTGGACCGAAGTAAAGGACAC	0.264781694	0.135115664	-0.224340676
B_98618_CGACTGGACCGAAGTATGGCTTCA	0.283394776	0.281019168	-0.584365696
B_98618_CGACTGGAGCCACATAACGCTCGA	0.330919482	0.076499112	-0.43951776
B_98618_CGCATACAGAGTTAGCACGTATCA	0.271340788	0.150634424	-0.398738567
B_98618_CGCATACAGGAGAACATTCACGCA	0.468879448	0.1379009	-0.423963531
B_98618_CGCATACATCCGTCTAAAGGACAC	0.323962202	0.360580173	-0.34168245
B_98618_CGCTGATCAGTGGTCACCGTGAGA	0.551685186	0.389207567	-0.241870242
B_98618_CGCTGATCCGCTGATCTTCACGCA	0.153539871	0.309376796	-0.49377796
B_98618_CGCTGATCCGGATTGCACGTATCA	0.351618697	0.167012135	-0.347719778
B_98618_CGCTGATCCTGTAGCCCCGACAAC	0.382904777	0.141483895	-0.477392443
B_98618_CGCTGATCGAACAGGCAAGACGGA	0.497034127	0.179330661	-0.429387959
B_98618_CGCTGATCGACAGTGCCCGAAGTA	0.148746257	0.194233499	-0.412787851
B_98618_CGCTGATCGTCTGTCAATGCCTAA	0.532201227	0.226969902	-0.380089982
B_98618_CGCTGATCTATCAGCAAAGACGGA	0.262189375	0.223906981	-0.55525647
B_98618_CGGATTGCAGATGTACACAGATTC	0.269279532	0.275653399	-0.413560723
B_98618_CGGATTGCATCCTGTAAAGACGGA	0.538312586	0.209329996	-0.322296553
B_98618_CGGATTGCCTGAGCCACCGACAAC	0.288185464	0.345523169	-0.372722572
B_98618_CGGATTGCGTCTGTCAAGCAGGAA	0.245238263	0.336634985	-0.444644212
B_98618_CTAAGGTCAAGGTACAACACAGAA	0.621179829	0.242469893	-0.378732263
B_98618_CTAAGGTCAGTGGTCAAGGCTAAC	0.487453828	0.418598978	-0.341330799
B_98618_CTAAGGTCCAAGACTACAAGGAGC	0.327376151	0.420340373	-0.366546119
B_98618_CTAAGGTCCATCAAGTGATGAATC	0.395243407	0.264476516	-0.447746408
B_98618_CTAAGGTCCCTCCTGACTGGCATA	1.450989165	0.541534788	-0.173601029
B_98618_CTAAGGTCGCCAAGACAAGAGATC	0.112681636	0.122069757	-0.430810831
B_98618_CTCAATGAAACTCACCAATCCGTC	0.918435043	0.402642983	-0.391023375
B_98618_CTCAATGAAAGACGGAAGCACCTC	0.385520495	0.133951967	-0.443101621
B_98618_CTCAATGAACCACTGTAGAGTCAA	1.318296572	0.509947004	-0.651387142
B_98618_CTCAATGAACCTCCAAACACAGAA	0.240521741	0.264821928	-0.310091485
B_98618_CTGAGCCAAAACATCGGATGAATC	0.407145699	0.298711468	-0.196965503
B_98618_CTGAGCCAAACGCTTAGGAGAACA	0.145387552	0.189345774	-0.20234701
B_98618_CTGAGCCACTGGCATACAACCACA	0.618681518	0.19406191	-0.503978484
B_98618_CTGAGCCAGAGCTGAAACGTATCA	0.900305707	0.323237738	-0.376173963

B_98618_CTGGCATAAAGGACACCCTCTATC	0.821244832	0.272076407	-0.597579875
B_98618_CTGGCATAAATGTTGCACTATGCA	0.292429255	0.309932888	-0.522007509
B_98618_CTGTAGCCAAGAGATCAGTCACTA	0.424332116	0.248585701	-0.182539772
B_98618_CTGTAGCCCATACCAAACACAGAA	0.293821248	0.256477629	-0.412729649
B_98618_CTGTAGCCCATCAAGTGCGAGTAA	0.369300825	0.212367339	-0.069643405
B_98618_CTGTAGCCCTGAGCCAAAGAGATC	0.399021144	0.346130519	-0.370893781
B_98618_GAACAGGCAGATCGCAGAACAGGC	0.33895822	0.305586768	-0.540047977
B_98618_GAACAGGCAGCACCTCTGGCTTCA	0.62563952	0.519617094	-0.36488527
B_98618_GAACAGGCCAAGGAGCCCTAATCC	0.55858514	0.296259803	0.01604292
B_98618_GAACAGGCCACCTTACGAATCTGA	0.24305162	0.16832013	-0.292245361
B_98618_GAACAGGCCACTTCGACTGTAGCC	0.605918792	0.538998605	-0.445284579
B_98618_GAACAGGCGGTGCGAAAACGTGAT	0.641074928	0.184290398	-0.359482393
B_98618_GAATCTGAACATTGGCACAAGCTA	0.440962565	0.241193983	-0.363318919
B_98618_GAATCTGACACTTCGACCATCCTC	0.112669988	0.213131876	-0.412230823
B_98618_GAATCTGACCGAAGTAGGAGAACA	0.281228822	0.209875971	-0.334578158
B_98618_GAATCTGACTGAGCCAACCACTGT	0.311275942	0.442258914	-0.446800389
B_98618_GAATCTGACTGGCATAACGCTCGA	0.844340124	0.29080918	-0.406262787
B_98618_GAATCTGAGTCGTAGACCGACAAC	0.375324398	0.15083344	-0.38475283
B_98618_GACAGTGCACCACTGTCCGAAGTA	0.116487979	0.328686203	-0.408924703
B_98618_GACAGTGCCAATGGAACCTAATCC	0.233691046	0.130446123	-0.232585834
B_98618_GACAGTGCCCTCTATCAGTACAAG	0.564968566	0.425554303	-0.376494529
B_98618_GACAGTGCCTGGCATATATCAGCA	0.823811194	0.225431797	-0.423996759
B_98618_GACAGTGCGCTCGGTAGCGAGTAA	0.479244416	0.246704808	-0.303920629
B_98618_GACTAGTAAGATCGCAAGATGTAC	0.047550369	0.213458073	-0.545082727
B_98618_GACTAGTACAACCACAAGATGTAC	0.416919298	0.289130236	-0.217690208
B_98618_GAGCTGAACGACACACGGAGAACA	0.284769636	0.277693996	-0.460895712
B_98618_GAGTTAGCAGCCATGCCTGGCATA	0.260657576	0.351723026	-0.594525382
B_98618_GAGTTAGCCCGAAGTACGCTGATC	0.408088214	0.456602682	-0.373982013
B_98618_GAGTTAGCCGCATACACTGAGCCA	0.555187343	0.583055242	-0.177878904
B_98618_GAGTTAGCGATGAATCACGTATCA	0.04830344	0.104978265	-0.456456222
B_98618_GAGTTAGCTGAAGAGACCGACAAC	0.5513647	0.291875653	-0.524819844
B_98618_GAGTTAGCTGGCTTCAACAGATTC	0.14919821	0.313691058	-0.319023022

B_98618_GATAGACAAGGCTAACACGCTCGA	0.221883454	0.114297607	-0.327179557
B_98618_GATAGACACACCTTACACAGATTC	0.508569236	0.490340795	-0.185108941
B_98618_GATAGACACCTCTATCGCTCGGTA	0.565138022	0.158967013	-0.348954381
B_98618_GATAGACAGACAGTGCGTCTGTCA	0.210745583	0.244460847	-0.17133771
B_98618_GATGAATCAGGCTAACCACTTCGA	0.04069916	0.150321275	-0.454548704
B_98618_GATGAATCATTGGCTCGACAGTGC	0.160430068	0.235767338	-0.031946148
B_98618_GATGAATCCATACCAACTCAATGA	0.541234543	0.225672572	-0.352553084
B_98618_GCCAAGACAGTACAAGAGAGTCAA	0.154358649	0.252937473	-0.148489627
B_98618_GCCAAGACAGTGGTCATAGGATGA	0.34420525	0.412274039	-0.440930567
B_98618_GCCACATAAGATGTACCAACCACA	0.505470559	0.523093722	-0.224068818
B_98618_GCCACATAATCATTCCGACTAGTA	0.342447997	0.19672045	-0.508444254
B_98618_GCCACATAATCCTGTAGATGAATC	0.158449826	0.190506466	-0.243882561
B_98618_GCCACATACACTTCGATCTTCACA	0.260654693	0.082650315	-0.414261049
B_98618_GCCACATACCGTGAGACATACCAA	0.48192185	0.380941709	-0.300499391
B_98618_GCCACATAGTCGTAGAACATTGGC	0.16663815	0.171417668	-0.380264321
B_98618_GCGAGTAAAACAACCAATGCCTAA	0.248390012	0.165920698	-0.556188627
B_98618_GCGAGTAAGACTAGTACTGGCATA	0.015410002	0.280371739	-0.395098679
B_98618_GCTAACGAAAGACGGAACAAGCTA	0.865186139	0.377237246	-0.268877133
B_98618_GCTAACGAAAGAGATCCAGATCTG	0.200175628	0.156389589	-0.341789918
B_98618_GCTAACGAACGCTCGACAGCGTTA	0.405461004	0.376556191	-0.256216795
B_98618_GCTAACGAAGTACAAGAATCCGTC	0.972632589	0.390614484	-0.275737283
B_98618_GCTAACGACAATGGAAACACGACC	0.458375317	0.498606675	-0.241180084
B_98618_GCTCGGTAAAACATCGACACGACC	0.297394108	0.335683284	-0.208934434
B_98618_GCTCGGTACATCAAGTTCTTCACA	0.331847001	0.408800368	-0.4955638
B_98618_GCTCGGTAGATAGACAACAAGCTA	0.484702835	0.213824583	-0.354009511
B_98618_GCTCGGTAGTCTGTCAAGATCGCA	0.46052609	0.259528939	-0.150457741
B_98618_GCTCGGTAGTGTTCTAATGCCTAA	0.749806812	0.426235555	-0.373838727
B_98618_GCTCGGTATGGTGGTAAAGGACAC	0.546646123	0.311143684	-0.366829542
B_98618_GGAGAACAACAGATTCCCTCCTGA	0.373476528	0.151897851	-0.385281359
B_98618_GGAGAACAACCTCCAAGACAGTGC	0.194679266	0.136124712	-0.144298797
B_98618_GGAGAACAATGCCTAACTAAGGTC	0.32201952	0.27310294	-0.413872624
B_98618_GGAGAACATATCAGCACTAAGGTC	0.604449395	0.412966915	-0.226989302

B_98618_GGTGCGAAAATGTTGCCCGACAAC	0.530194324	0.417086773	-0.285413839
B_98618_GGTGCGAATTCACGCAACACGACC	0.268423031	0.080205135	-0.404294566
B_98618_GTACGCAAAATCCGTCAACGCTTA	0.36635366	0.22333257	-0.586305628
B_98618_GTACGCAAGCTCGGTAAACGCTTA	0.391686484	0.285880919	-0.103663417
B_98618_GTACGCAAGTACGCAACAAGGAGC	0.346590549	0.484571339	-0.121998
B_98618_GTACGCAATGAAGAGAAACAACCA	1.19919901	0.50736252	-0.53522867
B_98618_GTCGTAGAAAGACGGACTCAATGA	0.302490381	0.045797853	-0.490351479
B_98618_GTCGTAGAAAGACGGATCCGTCTA	0.645136011	0.505301198	-0.124499603
B_98618_GTCGTAGAATCCTGTAAACCGAGA	0.406558119	0.163435642	-0.214353992
B_98618_GTCGTAGACCTAATCCGTCTGTCA	0.364761302	0.268340719	-0.183552611
B_98618_GTCTGTCAAGATCGCAAACGCTTA	0.197502293	0.216371073	-0.081538078
B_98618_GTCTGTCAAGATCGCACCAGTTCA	0.16127441	0.069556233	-0.354225149
B_98618_GTCTGTCACGACTGGAACAGCAGA	0.615259997	0.53354975	-0.335781672
B_98618_GTCTGTCAGATAGACACCTCCTGA	0.310013748	0.314555064	-0.422453121
B_98618_GTGTTCTACAATGGAAACCTCCAA	0.678800434	0.598230118	-0.023213814
B_98618_GTGTTCTACCTAATCCTTCACGCA	0.197851441	0.241329221	-0.349350579
B_98618_GTGTTCTAGCTAACGACAACCACA	0.157603177	0.183059515	-0.543705241
B_98618_TAGGATGAAACAACCATGGAACAA	0.313413667	0.314910387	-0.326442222
B_98618_TAGGATGACCGAAGTACAACCACA	0.303655375	0.489769986	-0.155090874
B_98618_TAGGATGAGATGAATCCTGTAGCC	0.32422642	0.371743487	-0.241422671
B_98618_TATCAGCACGCTGATCATGCCTAA	0.415317408	0.323563853	-0.265062207
B_98618_TATCAGCAGAGTTAGCGTCGTAGA	0.227164225	0.265322111	-0.297982268
B_98618_TATCAGCAGCCACATAGTACGCAA	0.318198725	0.408828738	-0.240794901
B_98618_TATCAGCATCTTCACAAGCACCTC	0.580087847	0.357619681	-0.451743918
B_98618_TCCGTCTAAATCCGTCAGAGTCAA	0.051734463	0.149459708	-0.360129763
B_98618_TCCGTCTACTGGCATATGGTGGTA	0.460827335	0.604515945	-0.015469282
B_98618_TCCGTCTAGAGCTGAAAGGCTAAC	0.372577997	0.347647258	-0.439447769
B_98618_TCCGTCTAGTCGTAGAACATTGGC	0.110840865	0.210390288	-0.508540521
B_98618_TCCGTCTATCCGTCTATGGCTTCA	0.697551876	0.263029199	-0.229964577
B_98618_TCTTCACAAATGTTGCCTGAGCCA	0.551077947	0.356865913	-0.199852561
B_98618_TCTTCACAATTGGCTCCCGTGAGA	0.33250413	0.312347908	-0.102070395
B_98618_TCTTCACACACTTCGACAGATCTG	1.410974004	0.438302372	-0.43393126

B_98618_TCTTCACACCAGTTCACACCTTAC	0.537916341	0.349231521	-0.190416914
B_98618_TCTTCACACTAAGGTCAGCACCTC	0.332380479	0.137149047	-0.464194749
B_98618_TCTTCACAGGAGAACAAGCACCTC	0.498506716	0.246233495	-0.341930983
B_98618_TCTTCACATCCGTCTACCATCCTC	0.367776738	0.184213669	-0.553813582
B_98618_TGAAGAGAAAACATCGAACGTGAT	0.157748856	0.448485999	-0.150880756
B_98618_TGGAACAAAATCCGTCAACGTGAT	0.462342464	0.165083751	-0.526785588
B_98618_TGGAACAACTGAGCCAGCCAAGAC	0.318092476	-0.065306561	-0.607905236
B_98618_TGGCTTCAACGCTCGACGACTGGA	0.778088003	0.204589788	-0.512871471
B_98618_TGGCTTCAAGCACCTCGCCAAGAC	0.047776358	0.01295784	-0.417875181
B_98618_TGGCTTCACCAGTTCAATTGAGGA	0.111775582	0.208331554	0.114601817
B_98618_TGGCTTCAGAGCTGAACAAGGAGC	0.26760532	0.310226424	-0.351497275
B_98618_TGGTGGTAACATTGGCCGGATTGC	0.119087838	0.309502342	-0.130149445
B_98618_TGGTGGTAGATGAATCCCAGTTCA	0.420178365	0.3027987	-0.161186065
B_98618_TGGTGGTAGCTCGGTAAGAGTCAA	0.444842798	0.427969314	-0.291584041
B_98618_TTCACGCAAAGAGATCAGTACAAG	0.030744101	0.180208025	-0.399141907
B_98618_TTCACGCAATCATTCCAAGGACAC	0.166223451	0.184397149	-0.493819654
B_98618_TTCACGCAATCCTGTAGCGAGTAA	0.269655202	0.342066179	-0.116530779
B_98618_TTCACGCAATTGGCTCACACAGAA	0.520903914	0.325000086	-0.43265339
B_98618_TTCACGCACTCAATGACACCTTAC	0.210018625	0.272867514	-0.303757866
B_98618_TTCACGCACTGAGCCAAGTGGTCA	0.267196907	0.157433108	-0.306326747
B_98618_TTCACGCATTCACGCAATAGCGAC	0.396266453	0.393099173	-0.245056356
B_98618_AAACATCGAAGAGATCCAGATCTG	0.579344708	0.506015744	-0.221899897
B_98618_AAACATCGCAGATCTGACATTGGC	0.43211705	0.599891026	-0.526203903
B_98618_AAACATCGCCGTGAGAGAACAGGC	0.717444039	0.986495175	-0.535778132
B_98618_AAACATCGGTGTTCTAGGTGCGAA	1.008565103	0.826207033	-0.517495654
B_98618_AAACATCGTCCGTCTAATGCCTAA	0.784358687	0.844541187	-0.241030936
B_98618_AACAACCACAAGACTAATGCCTAA	0.472303741	0.501507469	-0.441708517
B_98618_AACAACCACTGGCATATGGAACAA	0.035554325	0.459192546	-0.511560771
B_98618_AACAACCAGAGCTGAAAGCACCTC	0.997742445	1.008592997	-0.068872173
B_98618_AACCGAGAAGGCTAACTGGTGGTA	0.382107846	0.476847757	-0.534490873
B_98618_AACGTGATCAATGGAACGCATACA	0.360157679	0.470834839	-0.309296409
B_98618_AACGTGATCCATCCTCGGAGAACA	0.570566397	0.639402859	-0.436227326

B_98618_AACGTGATGATGAATCGTCGTAGA	0.358752614	0.627203541	-0.14951233
B_98618_AACTCACCACAAGCTAGAGTTAGC	0.332491006	0.232955714	-0.614689938
B_98618_AACTCACCAGGCTAACATTGGCTC	0.663749069	0.693350509	-0.258436168
B_98618_AACTCACCCAACCACACCGACAAC	0.577249706	0.411857172	-0.644632353
B_98618_AAGACGGAATTGGCTCGATGAATC	0.520872258	0.765489157	-0.257812933
B_98618_AAGACGGAGTACGCAAGGAGAACA	0.545101704	0.293836335	-0.476396316
B_98618_AAGACGGAGTGTTCTACCTCCTGA	0.335483744	0.404341572	-0.533981613
B_98618_AAGACGGAGTGTTCTAGATGAATC	0.728832939	0.998771041	-0.356192391
B_98618_AAGAGATCACTATGCAATTGAGGA	0.551448788	0.779846988	-0.429820878
B_98618_AAGAGATCAGATCGCATGGTGGTA	0.460893527	0.178986868	-0.610911425
B_98618_AAGAGATCCTGTAGCCGTCTGTCA	0.64227493	0.600115849	-0.32654767
B_98618_AAGAGATCGAATCTGACACCTTAC	0.235602647	0.389556037	-0.188249235
B_98618_AAGAGATCGGTGCGAACGGATTGC	0.502594631	0.661971632	-0.339785205
B_98618_AAGGACACAGTACAAGACGCTCGA	0.639861782	1.787503748	-0.216817783
B_98618_AAGGACACTGGAACAACGGATTGC	0.727894567	0.5007132	-0.508644408
B_98618_AAGGTACAACAGATTCAGATCGCA	0.438508899	0.245080229	-0.41130526
B_98618_AAGGTACAAGGCTAACAAACATCG	0.379356197	0.404997118	-0.501911836
B_98618_AAGGTACAGCCAAGACCTCAATGA	0.596755031	0.860042058	-0.412172411
B_98618_AAGGTACATGGTGGTATATCAGCA	0.078850044	0.165028105	-0.536629272
B_98618_AATCCGTCCAAGGAGCAAGGACAC	0.53164195	0.570707371	-0.14621682
B_98618_AATCCGTCCCATCCTCAGGCTAAC	0.253447429	0.279677896	-0.581214456
B_98618_AATCCGTCGATAGACACACTTCGA	0.674632467	0.482874957	-0.435759541
B_98618_AATCCGTCGTCTGTCAATCCTGTA	0.856792094	0.685269208	-0.383459672
B_98618_AATGTTGCACTATGCATAGGATGA	0.401600702	0.471784505	-0.423386022
B_98618_AATGTTGCAGTCACTAAAGGTACA	0.598077434	0.579053063	-0.590031347
B_98618_AATGTTGCCGCATACACGACTGGA	0.474220162	0.638568158	-0.144361846
B_98618_AATGTTGCCTGAGCCAAGTACAAG	0.642499438	1.149832797	-0.409562904
B_98618_ACAAGCTACAGCGTTAAACTCACC	0.794223467	0.782164807	-0.533222688
B_98618_ACACAGAAAACCGAGAATCATTCC	0.500027086	0.490422791	-0.409634995
B_98618_ACACAGAAAACGTGATGACAGTGC	0.441409983	0.166068595	-0.543372212
B_98618_ACACAGAACCAGTTCAACGCTCGA	0.759676119	0.459684797	-0.521747715
B_98618_ACACAGAACGCATACAGGTGCGAA	0.655100296	0.751281237	-0.42521164

B_98618_ACACAGAAGAGCTGAAAGCAGGAA	0.975188555	0.383208144	-0.538983127
B_98618_ACACGACCAACGTGATAAGGACAC	0.496121212	0.676335377	-0.293562655
B_98618_ACACGACCAACTCACCACAGATTC	0.867729964	0.582296887	-0.561004937
B_98618_ACACGACCACAAGCTACAAGGAGC	0.335168886	0.522377009	-0.295741324
B_98618_ACACGACCCCTCCTGAACATTGGC	0.82045388	0.785351447	-0.260723116
B_98618_ACACGACCTAGGATGAATGCCTAA	0.545288305	0.530464807	-0.250016016
B_98618_ACAGATTCACCTCCAACCTCTATC	0.400375475	0.228289865	-0.522142277
B_98618_ACAGATTCGATAGACAAGATCGCA	0.420235063	0.532577211	-0.453583378
B_98618_ACAGATTCGCTCGGTAACATTGGC	0.625279253	0.743392351	-0.459206292
B_98618_ACAGATTCTGGCTTCATAGGATGA	0.644596245	0.65067511	-0.492595575
B_98618_ACAGCAGAAACCGAGAAACGCTTA	0.272410755	0.349555663	-0.503153648
B_98618_ACAGCAGAACGTATCACAATGGAA	0.88587301	0.774902035	-0.506559881
B_98618_ACAGCAGATGGTGGTACCTCTATC	0.416871469	0.419876423	-0.124836595
B_98618_ACATTGGCAGTGGTCAAATCCGTC	0.589820783	0.37221134	-0.415227941
B_98618_ACATTGGCATCATTCCAACAACCA	1.099275744	0.917272406	-0.544744289
B_98618_ACATTGGCCAGATCTGCCAGTTCA	0.647656413	0.45247087	-0.376565579
B_98618_ACATTGGCGCTAACGAGTCTGTCA	0.652224913	0.80380265	-0.317151455
B_98618_ACCACTGTACACGACCCCGTGAGA	0.74069737	0.912459342	-0.371040207
B_98618_ACCACTGTGAATCTGAATTGAGGA	1.136994908	0.97590058	0.102685638
B_98618_ACCTCCAAAGATCGCAGTACGCAA	0.592359971	0.707978896	-0.329994241
B_98618_ACCTCCAAATTGGCTCACACAGAA	0.635671543	0.456384837	-0.458695066
B_98618_ACGCTCGACAGATCTGTGGAACAA	0.686863276	0.558315986	-0.480421416
B_98618_ACGCTCGACGACACACCAGATCTG	0.900213834	0.48087895	-0.560784718
B_98618_ACGCTCGATATCAGCACCGTGAGA	0.515518772	0.432920921	-0.599806348
B_98618_ACGTATCAAACGTGATAGTCACTA	0.569154531	0.145644711	-0.494532741
B_98618_ACGTATCAAATCCGTCGACTAGTA	0.58642804	0.677932286	-0.241191167
B_98618_ACGTATCAAATGTTGCCAATGGAA	0.554342117	0.8647606	-0.429581197
B_98618_ACGTATCACAGCGTTAACACAGAA	0.371942099	0.729102981	-0.564430607
B_98618_ACGTATCACTGTAGCCACCTCCAA	0.693174947	0.380817722	-0.503349664
B_98618_ACGTATCAGACTAGTAATCCTGTA	0.951498184	0.954170492	-0.48033055
B_98618_ACGTATCAGCCAAGACACAAGCTA	0.378929983	0.443674807	-0.62819525
B_98618_ACGTATCATCTTCACAACAAGCTA	0.757113129	0.764453786	-0.574361865

B_98618_ACTATGCAAGATGTACGATGAATC	0.675150601	0.890110491	-0.384029041
B_98618_ACTATGCAGGAGAACAGAACAGGC	0.694132126	0.779126625	-0.362792378
B_98618_AGAGTCAAAAGGTACACGCTGATC	0.494864215	0.252958342	-0.502431655
B_98618_AGAGTCAAACCTCCAAGCTCGGTA	0.462110146	0.267158343	-0.307910405
B_98618_AGAGTCAAATAGCGACCCGTGAGA	0.493224128	0.613301827	-0.151253075
B_98618_AGAGTCAACGGATTGCATTGAGGA	0.648787501	0.710845787	-0.555692495
B_98618_AGAGTCAAGCTCGGTACCGACAAC	0.377605691	0.372630209	-0.513037572
B_98618_AGATCGCACCTCCTGAACAGATTC	0.82191941	0.701735032	-0.442987661
B_98618_AGATCGCAGATAGACAGCCAAGAC	0.352538205	0.47462446	-0.505737822
B_98618_AGATGTACAAGGACACCGCTGATC	0.424897241	0.70551233	-0.533110605
B_98618_AGCACCTCACAAGCTAGAGCTGAA	0.593964753	0.709758958	-0.477088559
B_98618_AGCACCTCCAATGGAACGAACTTA	1.125071664	0.860352611	-0.453385554
B_98618_AGCACCTCCCGAAGTAAGTACAAG	0.557474638	0.674010582	-0.465228259
B_98618_AGCAGGAAAAGAGATCATCATTCC	0.697389704	0.756904497	-0.162185763
B_98618_AGCAGGAACGAACTTAAGATCGCA	0.63486699	0.543798653	-0.564511117
B_98618_AGCAGGAATAGGATGATTCACGCA	1.108166561	1.420646527	-0.364286455
B_98618_AGCCATGCAACGTGATCATACCAA	0.851961828	0.710501952	-0.474921826
B_98618_AGCCATGCCAAGACTACGCTGATC	0.603654456	0.300871583	-0.465676323
B_98618_AGCCATGCCAGCGTTAAGAGTCAA	0.820102227	1.271278646	-0.468414053
B_98618_AGGCTAACCCGAAGTAGACTAGTA	0.713942403	0.51155922	-0.418618209
B_98618_AGGCTAACCGACTGGACTGAGCCA	0.829307637	0.568459504	-0.50721457
B_98618_AGGCTAACTATCAGCAGAGCTGAA	0.890220134	0.510830893	-0.43138239
B_98618_AGTACAAGAGAGTCAAAGTACAAG	0.589626624	0.480364805	-0.309451518
B_98618_AGTCACTAATAGCGACGTGTTCTA	0.461624535	0.668313449	-0.324533849
B_98618_AGTGGTCAATTGAGGAAAGAGATC	0.547683694	0.445711551	-0.53281366
B_98618_AGTGGTCACATACCAACTGGCATA	0.494988507	0.548570253	-0.41587086
B_98618_AGTGGTCACGACACACGATGAATC	1.169779821	0.836903521	-0.077848618
B_98618_ATAGCGACCCGTGAGATGGAACAA	0.702121508	0.524503117	-0.342318121
B_98618_ATCATTCCAGTGGTCAGAATCTGA	0.414450629	0.459389247	-0.490848943
B_98618_ATCATTCCCAAGACTAAGCAGGAA	0.735378347	0.551109771	-0.459118951
B_98618_ATCATTCCCAAGGAGCGCGAGTAA	0.632353582	0.503677822	-0.612606351
B_98618_ATCATTCCGATGAATCAGCCATGC	0.75696331	0.571393879	-0.62947273

B_98618_ATCATTCCGTCGTAGAAGATGTAC	0.428394532	0.903772821	-0.186720587
B_98618_ATCCTGTAAGGCTAACATCCTGTA	0.859771729	1.388800056	-0.412769098
B_98618_ATCCTGTAGAATCTGACATCAAGT	0.436596314	0.548751849	-0.384163439
B_98618_ATGCCTAAAACAACCACTCAATGA	0.560541596	0.603281217	-0.405223738
B_98618_ATGCCTAAACGCTCGACCATCCTC	0.805912228	0.366653363	-0.548104895
B_98618_ATGCCTAAGCTCGGTAGTGTTCTA	0.877126746	0.928710526	-0.331646458
B_98618_ATGCCTAATCCGTCTAGAATCTGA	0.730840435	0.627115037	-0.387205056
B_98618_ATTGAGGAAACTCACCAATCCGTC	0.572542967	0.567616921	-0.482278935
B_98618_ATTGAGGAAACTCACCCAAGACTA	0.848595732	0.921946882	-0.245720166
B_98618_ATTGAGGAAAGGTACAAACGCTTA	0.766388883	0.772247171	-0.466442955
B_98618_ATTGAGGAAGCAGGAACAAGGAGC	0.313860995	0.598815942	-0.37544325
B_98618_ATTGGCTCCGCATACACGCATACA	0.662967157	0.79730514	-0.414886808
B_98618_ATTGGCTCGACTAGTACAACCACA	0.820719121	0.810992653	-0.133428168
B_98618_ATTGGCTCGTCTGTCACCTCTATC	0.641931245	0.454051005	-0.444444025
B_98618_ATTGGCTCTGGTGGTAAGTCACTA	0.721240402	0.768378189	-0.475527276
B_98618_CAACCACAGTACGCAACGACACAC	0.718429124	0.905476829	-0.570206742
B_98618_CAACCACATCCGTCTACACCTTAC	0.836627015	0.744324523	-0.550739346
B_98618_CAAGACTAAAGGTACAACACGACC	0.673527762	0.538687064	-0.47449313
B_98618_CAAGACTACATCAAGTACACGACC	0.671242526	0.637288905	-0.423166178
B_98618_CAAGACTACGACTGGACTGGCATA	0.99118115	1.925530272	-0.370390573
B_98618_CAAGACTACTGTAGCCACACGACC	0.947548065	1.224367269	-0.381214988
B_98618_CAAGACTAGAACAGGCCGACACAC	0.406506889	0.847573075	0.008223655
B_98618_CAAGACTATTCACGCAAGTGGTCA	0.720478321	0.743626623	-0.381358586
B_98618_CAAGGAGCACGTATCACTGTAGCC	0.357744475	0.622847229	-0.40883728
B_98618_CAAGGAGCGAGCTGAACCATCCTC	0.645704118	0.634421612	-0.591929417
B_98618_CAAGGAGCGCCACATATATCAGCA	0.449086918	0.50902207	-0.56702827
B_98618_CAATGGAAACAAGCTACTGGCATA	0.409009884	0.445264855	-0.33812923
B_98618_CAATGGAAACAGATTCAAACATCG	0.53962929	0.675682029	-0.199511461
B_98618_CAATGGAACCATCCTCGTGTTCTA	0.686443836	0.502478247	-0.271638611
B_98618_CAATGGAACCGAAGTACTGTAGCC	0.511741417	0.808526274	-0.275492172
B_98618_CAATGGAAGGAGAACACGACACAC	0.493702801	0.350967606	-0.537642033
B_98618_CAATGGAAGTCTGTCAACATTGGC	0.421619525	0.482138528	-0.437176416

B_98618_CACTTCGAAAGGACACCGGATTGC	0.429753725	0.678860565	-0.363435007
B_98618_CACTTCGAAGATGTACCGACACAC	0.839948944	0.858929619	-0.285936819
B_98618_CACTTCGACGACACACCCATCCTC	0.538500549	0.606952685	-0.472542161
B_98618_CACTTCGAGAACAGGCGGTGCGAA	0.676297745	0.837491275	-0.468107694
B_98618_CAGATCTGAATGTTGCAATGTTGC	0.545561194	0.706768148	-0.445091339
B_98618_CAGATCTGAGATCGCACTGTAGCC	0.597517449	0.429204293	-0.511461541
B_98618_CAGATCTGAGCCATGCGACAGTGC	1.015321772	1.137705643	-0.528439421
B_98618_CAGATCTGCTGGCATAATGCCTAA	0.567575675	0.531092059	-0.48214684
B_98618_CAGCGTTAGATAGACACAAGGAGC	0.510412463	0.595364604	-0.236961618
B_98618_CATACCAAAACCGAGAGGTGCGAA	1.076137833	2.051834421	-0.242900711
B_98618_CATACCAACAAGGAGCCTGTAGCC	0.172931461	0.676808854	-0.298366146
B_98618_CATACCAACTGGCATATTCACGCA	0.452159544	0.488973697	-0.532220244
B_98618_CATCAAGTATTGGCTCGGAGAACA	0.462398382	0.654515156	-0.215148731
B_98618_CATCAAGTCAGCGTTAACAGCAGA	0.658208536	0.751576827	-0.391211917
B_98618_CATCAAGTCATCAAGTGACAGTGC	0.851967091	0.826389197	-0.322657527
B_98618_CATCAAGTCGACTGGACTGGCATA	0.947978036	0.650069142	-0.38821638
B_98618_CATCAAGTCGGATTGCCGACACAC	0.778232469	0.471760831	-0.157026597
B_98618_CATCAAGTTATCAGCAAGCACCTC	0.399144242	0.459634932	-0.470065557
B_98618_CCAGTTCACAATGGAACATACCAA	0.688348201	1.512468527	-0.509119846
B_98618_CCAGTTCACATACCAACCTCTATC	0.458828457	0.576278491	-0.083103175
B_98618_CCATCCTCAAGGTACATTCACGCA	0.377148277	0.696297868	-0.41310414
B_98618_CCGAAGTAAAGGTACAGACAGTGC	0.342847445	0.497242575	-0.31883551
B_98618_CCGAAGTAATGCCTAAGATGAATC	0.726375792	0.519009155	-0.457781536
B_98618_CCGAAGTACGACACACCAAGACTA	0.351467203	0.389111071	-0.190950226
B_98618_CCGAAGTACTGTAGCCAATCCGTC	0.40663471	0.6142189	-0.220605281
B_98618_CCGACAACGACAGTGCTGGTGGTA	0.777923028	0.687788007	-0.46878034
B_98618_CCGTGAGAAAGACGGACCGTGAGA	0.414971408	0.358790882	-0.567300316
B_98618_CCGTGAGAAAGGACACGGTGCGAA	0.466470712	0.82486079	-0.443955302
B_98618_CCGTGAGACATACCAATATCAGCA	0.811522615	0.676870143	-0.591871895
B_98618_CCGTGAGAGTCGTAGATCTTCACA	0.598362726	0.665509342	-0.266521317
B_98618_CCTAATCCATTGGCTCGCCACATA	0.502155888	0.571652935	-0.243973236
B_98618_CCTAATCCCAAGACTACGACTGGA	0.185195173	0.292360833	-0.568324062

B_98618_CCTCCTGAAAACATCGCGCTGATC	0.654333555	1.188653122	-0.322664377
B_98618_CCTCCTGAAACGTGATAGTACAAG	0.511988505	0.644738982	-0.335176053
B_98618_CCTCCTGAAGAGTCAAAAGGTACA	0.844354164	2.008115715	-0.375390388
B_98618_CCTCCTGAATCCTGTAGATGAATC	0.696509998	0.239735856	-0.547141791
B_98618_CCTCCTGACAAGACTATCTTCACA	0.265299536	0.357709729	-0.443390727
B_98618_CCTCCTGACTGGCATATGGAACAA	0.54569357	0.704502201	-0.584622326
B_98618_CCTCCTGATGGTGGTATAGGATGA	0.736292332	0.348457651	-0.552102694
B_98618_CCTCTATCCCGACAACAATGTTGC	0.343913702	0.300966258	-0.593793415
B_98618_CCTCTATCCCTCCTGAAACAACCA	0.848470162	0.729457099	-0.607616529
B_98618_CCTCTATCCTAAGGTCACACGACC	0.572224619	0.678120765	-0.570911528
B_98618_CGACACACCAATGGAAAGATGTAC	0.414983513	0.574264741	-0.271491107
B_98618_CGACACACCATACCAAACATTGGC	0.683731271	0.560054465	-0.369627497
B_98618_CGACTGGAAAGGTACAAACGTGAT	1.108810857	1.059828111	-0.50291736
B_98618_CGACTGGAAGCCATGCTGAAGAGA	0.695183451	0.834231522	-0.326354018
B_98618_CGACTGGACAACCACACACCTTAC	0.61620878	0.578034687	-0.420399917
B_98618_CGACTGGACAGATCTGCCGACAAC	0.836877178	0.85973061	-0.573750667
B_98618_CGACTGGAGACAGTGCCGACTGGA	0.568752589	0.527587746	-0.424465055
B_98618_CGACTGGAGTCTGTCACTGAGCCA	0.558014258	0.778876505	-0.361155571
B_98618_CGCATACAAACCGAGAACGTATCA	0.664655548	0.939421098	-0.557470538
B_98618_CGCATACAAGATGTACGCTAACGA	0.669604284	0.462906946	-0.102135183
B_98618_CGCATACACACTTCGAAGATGTAC	0.58185353	0.701500556	-0.235911445
B_98618_CGCATACACAGATCTGAGAGTCAA	0.682672472	0.426822498	-0.518980053
B_98618_CGCTGATCCGACTGGACGCTGATC	0.92482641	0.878791361	-0.578486597
B_98618_CGCTGATCGACAGTGCATCCTGTA	0.350604214	0.582731376	-0.377536763
B_98618_CGCTGATCGCTCGGTAAGTCACTA	0.796292089	1.822229255	-0.464285735
B_98618_CGCTGATCGTACGCAAGAACAGGC	0.921270064	0.87145871	-0.479427461
B_98618_CGGATTGCACACGACCATGCCTAA	0.530072293	0.364893526	-0.565545776
B_98618_CTAAGGTCAACAACCACAAGACTA	0.577432571	0.691734316	-0.117114426
B_98618_CTAAGGTCCCGAAGTAAACGTGAT	0.824947866	0.623171541	-0.429669816
B_98618_CTAAGGTCCGACACACCACTTCGA	0.893666289	0.667751012	-0.468384043
B_98618_CTAAGGTCGAACAGGCAAGAGATC	0.229364165	0.406625175	-0.427305815
B_98618_CTAAGGTCGTCTGTCAGAGCTGAA	0.880577823	0.682661902	-0.490302933

B_98618_CTCAATGAACAAGCTAGCTCGGTA	0.443767399	0.934100754	-0.581481246
B_98618_CTCAATGAAGCCATGCACATTGGC	0.823580823	0.702136442	-0.257198191
B_98618_CTCAATGACAACCACAAGCCATGC	0.818163725	0.746775531	-0.283377071
B_98618_CTGAGCCACACCTTACCAATGGAA	1.13997735	2.048028279	0.303290972
B_98618_CTGAGCCATGAAGAGACTCAATGA	0.526745739	0.515055499	-0.177083512
B_98618_CTGGCATAAATCCGTCCACCTTAC	0.477930146	0.497617123	-0.538812042
B_98618_CTGGCATACAATGGAAAACCGAGA	0.689017815	0.644869591	-0.312028058
B_98618_CTGGCATACAGATCTGCGCATACA	0.567266294	0.39207221	-0.570919447
B_98618_CTGGCATACTGTAGCCCACCTTAC	0.455493093	0.829852587	-0.143493785
B_98618_CTGGCATATGGAACAACACTTCGA	0.432878879	0.286246302	-0.515063697
B_98618_CTGTAGCCAGTCACTAAGATGTAC	1.157796506	0.596049945	-0.541568801
B_98618_CTGTAGCCTCCGTCTAAAGACGGA	0.82674825	0.687655879	-0.305526237
B_98618_GAACAGGCAACGCTTACCTCTATC	0.885990033	1.083950623	0.024889554
B_98618_GAACAGGCACAAGCTAGACTAGTA	0.46202761	0.498488296	-0.435896349
B_98618_GAACAGGCAGAGTCAAACAAGCTA	0.749361704	0.735487139	-0.210016406
B_98618_GAACAGGCAGTGGTCAACACGACC	0.760165832	0.75175585	-0.321051894
B_98618_GAATCTGAAACGCTTAGATGAATC	0.697265984	0.874580528	-0.489033176
B_98618_GAATCTGAACCACTGTCAAGGAGC	0.598886714	0.46070364	-0.445581132
B_98618_GAATCTGACACTTCGACATCAAGT	0.784797324	0.616431443	-0.548853159
B_98618_GAATCTGACAGATCTGAGCCATGC	0.558021217	0.533639088	-0.292115697
B_98618_GAATCTGAGAGTTAGCCAAGGAGC	0.657066948	1.051143977	-0.456861386
B_98618_GACAGTGCACAGATTCGATAGACA	0.884729635	0.789438967	-0.340262175
B_98618_GACAGTGCCCTAATCCCCTCTATC	0.924225353	1.888806882	-0.167358594
B_98618_GACTAGTAAACCGAGAACGTATCA	0.212090722	0.545553848	-0.353603931
B_98618_GACTAGTAACAGCAGAACAGATTC	0.610476649	0.503983507	-0.457699102
B_98618_GACTAGTAAGATCGCACGACTGGA	0.66270253	0.741345625	-0.102512885
B_98618_GACTAGTACCAGTTCATGGAACAA	1.111156119	1.797758837	-0.464154876
B_98618_GACTAGTACCTCTATCACATTGGC	0.56918256	0.420796994	-0.538941151
B_98618_GACTAGTATTCACGCAGAGTTAGC	0.963837978	0.679778863	-0.180615976
B_98618_GAGCTGAAACATTGGCAGTACAAG	0.597937052	0.980165496	-0.523231591
B_98618_GAGCTGAACAAGACTACGACTGGA	0.5011444	0.650213721	-0.379234263
B_98618_GAGTTAGCAACCGAGACTAAGGTC	0.494749486	0.208957424	-0.467627027

B_98618_GAGTTAGCAACGTGATCTGAGCCA	0.270859898	0.620739477	-0.498750692
B_98618_GAGTTAGCAAGGTACACTGAGCCA	0.322075349	0.402088237	-0.634994081
B_98618_GAGTTAGCCAAGGAGCGATGAATC	0.244157121	0.331134971	-0.279954359
B_98618_GAGTTAGCCGAACTTACTGAGCCA	0.755031177	0.45506167	-0.539337201
B_98618_GAGTTAGCGAGCTGAATAGGATGA	0.465733174	0.657412901	-0.251658724
B_98618_GAGTTAGCGATGAATCGAACAGGC	0.486912444	0.747840102	-0.315184277
B_98618_GATAGACAATAGCGACACACAGAA	0.723589988	1.450585174	-0.475592471
B_98618_GATAGACAATTGAGGAGCTAACGA	0.60633579	0.768347251	-0.123193568
B_98618_GATAGACACTAAGGTCACACAGAA	0.878979465	1.896206539	-0.367720779
B_98618_GATAGACAGCCAAGACGATAGACA	0.59598981	0.481655636	-0.410793689
B_98618_GATAGACAGCCACATACTCAATGA	0.943106973	0.778475926	0.151292523
B_98618_GATGAATCAATCCGTCCAGATCTG	0.567416116	0.552342764	-0.322346463
B_98618_GATGAATCGCTCGGTACAATGGAA	0.302253264	0.547502293	-0.361788503
B_98618_GCCAAGACCGACTGGATAGGATGA	0.684285318	0.770279556	-0.276676768
B_98618_GCCACATACCGTGAGATTCACGCA	0.710947535	0.558724547	-0.315709574
B_98618_GCCACATACCTCCTGACAAGACTA	0.830977558	1.30300038	-0.208475686
B_98618_GCGAGTAAACCTCCAATCCGTCTA	0.563093859	0.721671566	-0.557840224
B_98618_GCGAGTAACAAGACTAGTGTTCTA	0.814827003	0.78377978	-0.401764937
B_98618_GCGAGTAACCGACAACAGCCATGC	0.549721654	0.566769077	-0.568834097
B_98618_GCTAACGAAAACATCGCCTCTATC	0.402784554	0.3751576	-0.431378644
B_98618_GCTAACGAAACAACCAAACAACCA	0.480832926	0.511999212	-0.22744549
B_98618_GCTAACGAAAGACGGAGAGTTAGC	0.356821086	0.542113116	-0.390208525
B_98618_GCTAACGACAACCACAGAGCTGAA	1.373240472	2.02585709	-0.077901155
B_98618_GCTAACGACAGATCTGAACTCACC	0.665556006	0.920073461	-0.578276761
B_98618_GCTAACGACGACACACAAGAGATC	0.667359302	0.770350692	-0.339979717
B_98618_GCTAACGACTAAGGTCATAGCGAC	1.191709475	1.396184588	-0.330105393
B_98618_GCTAACGAGAATCTGAAGGCTAAC	0.505466684	0.615439457	-0.535180244
B_98618_GCTCGGTACTGTAGCCCATACCAA	0.69125628	0.660841331	-0.502566704
B_98618_GGAGAACAACGTATCAAGTCACTA	0.939944395	0.516189795	-0.374562537
B_98618_GGAGAACAAGGCTAACCCTCTATC	0.520844453	0.513353649	-0.165567197
B_98618_GGAGAACACGCTGATCCACCTTAC	0.647341239	0.991856705	-0.453509114
B_98618_GGAGAACACTGAGCCAGAACAGGC	0.840537282	0.789887575	-0.240285769

B_98618_GGTGCGAACCATCCTCCTAAGGTC	1.242651849	2.002360539	-0.237046117
B_98618_GGTGCGAACGACACACGCCAAGAC	0.315609028	0.155670386	-0.495881284
B_98618_GGTGCGAAGCTAACGACAATGGAA	0.58613583	0.581782464	-0.404807452
B_98618_GGTGCGAAGTCTGTCAGACAGTGC	1.282043011	2.058778127	-0.234248029
B_98618_GTACGCAACCAGTTCACAATGGAA	0.551696392	0.471847372	-0.279122814
B_98618_GTCGTAGACCTCCTGAAGTACAAG	0.500034872	0.594377789	-0.431104431
B_98618_GTCTGTCAAACGTGATATTGAGGA	0.658615469	0.477528232	-0.518985971
B_98618_GTCTGTCAGAACAGGCATTGGCTC	0.621693443	0.690419202	-0.433400645
B_98618_GTGTTCTAAAGACGGACACCTTAC	0.595337997	0.97334029	-0.339791768
B_98618_GTGTTCTAACAAGCTATCTTCACA	0.671555151	0.589239881	-0.298493654
B_98618_GTGTTCTACCTCCTGACACCTTAC	0.604488781	0.775942292	-0.563633394
B_98618_TAGGATGAAAGACGGAATCATTCC	1.299125635	0.531387853	-0.372866906
B_98618_TAGGATGAAATGTTGCCGAACTTA	0.560236877	0.673968696	-0.34428012
B_98618_TAGGATGACAAGACTAACGCTCGA	0.936504866	1.174528562	-0.229091885
B_98618_TAGGATGACACCTTACAGTACAAG	0.462079997	0.500030174	-0.552053399
B_98618_TAGGATGACCGACAACGGTGCGAA	0.542583298	0.43103684	-0.493233746
B_98618_TAGGATGACCTCCTGAAGCACCTC	0.702512051	0.28969743	-0.604543176
B_98618_TATCAGCAAAGGACACACCACTGT	0.766984364	0.901627739	-0.470430471
B_98618_TATCAGCAATTGGCTCCGCATACA	0.837191059	0.866623333	-0.272251915
B_98618_TATCAGCACACTTCGACAAGGAGC	0.817646153	0.623105127	-0.476898263
B_98618_TATCAGCACAGATCTGAGTACAAG	0.79400276	0.658328578	-0.149942065
B_98618_TATCAGCACCTAATCCACACGACC	0.635570525	0.390778352	-0.376667364
B_98618_TATCAGCACGCTGATCAAGAGATC	0.779704919	0.454839922	-0.482676885
B_98618_TCCGTCTACCGACAACCACTTCGA	0.67693373	1.457831968	-0.181142107
B_98618_TCTTCACAAAACATCGCAGATCTG	0.661406875	0.245308404	-0.563980316
B_98618_TCTTCACAAGTGGTCACTGTAGCC	0.320643996	0.370895097	-0.459526679
B_98618_TCTTCACAATCCTGTACAACCACA	0.666262828	0.752344582	-0.300454635
B_98618_TCTTCACAATCCTGTACCTCCTGA	0.42519635	0.393540541	-0.484594044
B_98618_TCTTCACAGAGTTAGCATGCCTAA	0.484321086	0.584742766	-0.585540262
B_98618_TCTTCACAGATAGACAAAGAGATC	1.148734926	1.202755572	-0.409147276
B_98618_TCTTCACAGCCACATAAGCCATGC	0.295533365	0.369341354	-0.423047318
B_98618_TGAAGAGAACAGCAGAACGCTCGA	0.434948189	0.692106647	-0.581034134

B_98618_TGAAGAGAACAGCAGACAATGGAA	0.268702236	0.582486231	-0.252578838
B_98618_TGAAGAGAACGTATCAGATGAATC	0.745979528	0.613115679	-0.164675301
B_98618_TGAAGAGACACCTTACAGTACAAG	0.640181472	0.518803226	-0.410653689
B_98618_TGAAGAGACACCTTACTGGCTTCA	0.736337702	0.708402657	-0.564074974
B_98618_TGGAACAAACCACTGTACACGACC	0.306459483	0.742303236	-0.465702802
B_98618_TGGAACAAACGCTCGATGGAACAA	0.529962047	0.529864292	-0.539884127
B_98618_TGGAACAAATCATTCCCCTAATCC	0.460504694	0.473672411	-0.534980092
B_98618_TGGAACAAGAATCTGACACTTCGA	0.374411049	0.447107818	-0.533206953
B_98618_TGGCTTCAAAGAGATCCAACCACA	0.65188207	0.507390127	-0.323252609
B_98618_TGGTGGTAACACAGAAAGGCTAAC	0.439673352	0.337527933	-0.629322362
B_98618_TGGTGGTAACACAGAAATTGAGGA	0.41942666	0.570897969	-0.295933276
B_98618_TGGTGGTACGGATTGCAACGCTTA	0.713988179	0.960955591	-0.515091349
B_98618_TTCACGCAGCTCGGTACAAGGAGC	0.503755555	0.858273611	-0.286357053
B_98618_AACCGAGAATAGCGACAATGTTGC	0.409160446	0.154045749	-0.252264469
B_98618_AATCCGTCCCTAATCCAATCCGTC	0.587281534	0.133608004	-0.342659982
B_98618_CGAACTTAGGAGAACAGAGCTGAA	0.086988135	0.314232879	-0.478491328
B_98618_CTAAGGTCACACAGAACAACCACA	0.067493814	0.160520063	-0.479906718
B_98618_TCCGTCTAACCACTGTTGGTGGTA	0.242628747	0.239190502	-0.42994806
B_98618_AAACATCGAAGGTACAATTGAGGA	0.072045016	0.336736921	-0.298847121
B_98618_AAACATCGCAAGACTATGGCTTCA	-0.054347804	0.108566778	-0.391981597
B_98618_AAACATCGCCTCCTGAATAGCGAC	0.067693711	0.108950269	-0.512895846
B_98618_AAACATCGCTCAATGAGATGAATC	-0.033410636	0.024343496	-0.448542478
B_98618_AACAACCAACGCTCGACACCTTAC	-0.086963611	-4.95E-05	-0.442522321
B_98618_AACAACCAACGTATCAACTATGCA	0.061599305	0.193513206	-0.371621314
B_98618_AACAACCAAGTGGTCAATGCCTAA	0.236681709	0.085018376	-0.424913891
B_98618_AACAACCAGAATCTGACACCTTAC	0.008557034	0.030458347	-0.429223484
B_98618_AACCGAGAAAGACGGAGATAGACA	-0.070985417	0.09552899	-0.413221162
B_98618_AACCGAGAAGATGTACGATGAATC	0.050991767	0.141281996	-0.284243057
B_98618_AACGCTTAACGTATCAGATGAATC	0.518048839	0.073136586	-0.568017453
B_98618_AACGCTTAATCATTCCGCGAGTAA	0.150267038	0.172236011	-0.251928071
B_98618_AACGCTTACCTCCTGACGCATACA	0.079201308	0.081399886	-0.201215397
B_98618_AACGCTTACGGATTGCGTCGTAGA	0.089124675	0.13983697	-0.499897283

B_98618_AACGTGATCAAGACTAAATCCGTC	-0.02672435	-0.046462928	-0.491888058
B_98618_AACTCACCACCTCCAAACAGATTC	0.083981422	0.153192954	-0.321925319
B_98618_AAGACGGAAATCCGTCAATCCGTC	0.109407102	0.304942305	-0.19712923
B_98618_AAGACGGAACTATGCAGAGCTGAA	0.774882149	0.173278755	-0.496683697
B_98618_AAGACGGATGAAGAGAAGTGGTCA	0.231518761	0.382942132	-0.23961467
B_98618_AAGAGATCCCTCTATCCCGTGAGA	-0.107465282	0.199283344	-0.579940366
B_98618_AAGAGATCGACTAGTACGCTGATC	0.550843862	0.009674923	-0.582204752
B_98618_AAGAGATCGAGTTAGCCATACCAA	-0.057370371	0.180678884	-0.367016383
B_98618_AAGGACACACACAGAAACAGATTC	0.254841642	0.116859986	-0.051820011
B_98618_AAGGACACCCTCCTGAAACGTGAT	0.119464098	0.239130534	-0.226740451
B_98618_AAGGACACCGAACTTAAGTACAAG	0.068928849	0.02213715	-0.472934974
B_98618_AAGGTACAAGATCGCACTGTAGCC	0.043696323	0.057539635	-0.496146658
B_98618_AAGGTACAAGCACCTCCGACACAC	-0.101681315	0.091183581	-0.453171849
B_98618_AAGGTACAAGGCTAACGAATCTGA	0.130436618	0.068550374	-0.214200964
B_98618_AAGGTACACATACCAACTGAGCCA	0.137115356	0.23162256	-0.281534775
B_98618_AAGGTACACCAGTTCACCGAAGTA	0.405185211	-0.032461603	-0.413224265
B_98618_AAGGTACAGACTAGTACATCAAGT	0.468212894	0.068538491	-0.299194556
B_98618_AATCCGTCTGGTGGTAAACGCTTA	0.450034192	0.018697773	-0.449690763
B_98618_AATGTTGCCCATCCTCACGCTCGA	0.046312928	0.120463115	-0.505464133
B_98618_AATGTTGCGCTCGGTAAGAGTCAA	0.177341853	0.004847244	-0.291713308
B_98618_AATGTTGCTGAAGAGAGAGCTGAA	0.535944844	0.143815894	-0.424257551
B_98618_ACAAGCTAATCATTCCAGAGTCAA	0.25925523	0.051767797	-0.484086627
B_98618_ACACAGAAAACAACCAAACGTGAT	0.288475488	0.136856293	-0.343846302
B_98618_ACACAGAAAAGAGATCACGCTCGA	0.121764706	0.20181336	-0.27476188
B_98618_ACACGACCAGATCGCAGTACGCAA	0.114340206	0.049149074	-0.424514592
B_98618_ACACGACCAGGCTAACCATACCAA	0.227451681	0.147343472	-0.404937655
B_98618_ACACGACCATCATTCCACGCTCGA	0.004173254	0.202057629	-0.323634262
B_98618_ACACGACCCTGAGCCACAACCACA	0.073942837	0.108814909	-0.232303064
B_98618_ACACGACCGAATCTGACAATGGAA	0.287066145	0.114768909	-0.332664455
B_98618_ACAGATTCACCACTGTACCACTGT	0.130465794	-0.05306751	-0.426118614
B_98618_ACAGATTCCACCTTACTAGGATGA	-0.000335987	0.075896408	-0.482493407
B_98618_ACAGCAGAATCATTCCAAACATCG	0.0521891	0.217540172	-0.416324118

B_98618_ACAGCAGAATTGAGGACATCAAGT	0.064265304	0.102045018	-0.219191749
B_98618_ACAGCAGAATTGGCTCACGTATCA	0.081780813	0.182416929	-0.467272723
B_98618_ACATTGGCAGCCATGCCAGATCTG	0.078581832	0.094118767	-0.252003777
B_98618_ACATTGGCAGTGGTCAAGCCATGC	0.166907042	0.117723629	-0.535439016
B_98618_ACCACTGTCCGACAACCAATGGAA	0.204763807	0.214971465	-0.43591767
B_98618_ACCACTGTGCTCGGTAAGGCTAAC	0.125289048	0.20077099	-0.397453741
B_98618_ACCTCCAACAATGGAAATTGGCTC	0.729694251	0.18656636	-0.603352144
B_98618_ACGCTCGACAGCGTTAAAACATCG	-0.044856244	0.259192949	-0.199751121
B_98618_ACGCTCGAGAACAGGCCATCAAGT	0.321809089	0.266913328	-0.301365191
B_98618_ACGCTCGATCCGTCTAAGCCATGC	0.084885221	0.221150212	-0.364249759
B_98618_ACGTATCAAGTCACTAAGTACAAG	0.09228465	0.075250865	-0.437192491
B_98618_ACTATGCAAAACATCGCCTCTATC	0.15325353	0.161525216	-0.45592638
B_98618_ACTATGCACATACCAAGTCGTAGA	0.780500996	0.28057555	-0.179412384
B_98618_ACTATGCACGACACACAAGACGGA	0.110133435	0.174451991	-0.15595156
B_98618_AGAGTCAAACACAGAACATACCAA	0.273385371	0.102405269	-0.455090176
B_98618_AGAGTCAAACGTATCAAGCCATGC	0.250070752	0.101655175	-0.209748724
B_98618_AGAGTCAAATCCTGTATCTTCACA	1.019776878	0.19335095	-0.296483227
B_98618_AGATCGCACTCAATGATAGGATGA	-0.060627349	0.053793615	-0.249493109
B_98618_AGATGTACCTCAATGACAGATCTG	0.370842675	0.14686844	-0.411430554
B_98618_AGATGTACGAACAGGCCATCAAGT	0.547833529	0.124807002	-0.2412169
B_98618_AGATGTACGATAGACAAAGGTACA	0.505519803	0.13003504	-0.392954964
B_98618_AGCACCTCAACTCACCAGCCATGC	0.043232784	0.171386462	0.185852727
B_98618_AGCACCTCCGGATTGCCTAAGGTC	0.035275654	0.004330645	-0.5506265
B_98618_AGCACCTCGAACAGGCGCTCGGTA	0.26728138	0.150773266	-0.366010742
B_98618_AGCCATGCACAGCAGAAACAACCA	-0.041153923	0.221476341	-0.339810408
B_98618_AGCCATGCACGCTCGAGGTGCGAA	0.068985316	0.216353226	-0.309089748
B_98618_AGCCATGCGCTCGGTAACAAGCTA	0.115653653	0.037438344	-0.18136811
B_98618_AGGCTAACAACGTGATACATTGGC	-0.110307907	-0.031240911	-0.467586036
B_98618_AGGCTAACAAGGACACAGCCATGC	-0.213986607	0.057919445	-0.329340961
B_98618_AGGCTAACAGGCTAACACACAGAA	-0.104810298	0.177874166	-0.427298234
B_98618_AGGCTAACATTGAGGACAAGACTA	0.770329459	0.226234796	-0.502551011
B_98618_AGGCTAACGAATCTGACCAGTTCA	-0.118643008	0.043920547	-0.348349237

B_98618_AGGCTAACGCCAAGACGTCGTAGA	0.074523323	0.140455144	-0.2934427
B_98618_AGGCTAACTGGAACAAAACCGAGA	0.604097377	0.171433038	-0.330536753
B_98618_AGTACAAGAGTACAAGCCGAAGTA	-0.138162955	0.039258306	-0.295124516
B_98618_AGTACAAGCAAGACTAAGCAGGAA	0.066406719	0.063512128	-0.439146543
B_98618_AGTACAAGCCGTGAGACTAAGGTC	0.087275736	0.075200865	-0.388532845
B_98618_AGTACAAGCGCATACATATCAGCA	0.06101751	0.0697337	-0.260427869
B_98618_AGTACAAGGCTAACGAAGCACCTC	0.688318605	0.193057983	-0.390549321
B_98618_AGTACAAGGGAGAACAAGATCGCA	0.667039139	0.146580262	-0.649644892
B_98618_AGTCACTAATTGGCTCCGGATTGC	0.166284633	0.074273269	-0.480493123
B_98618_AGTCACTAGTGTTCTAGACTAGTA	0.160517658	0.203923342	-0.227562423
B_98618_AGTCACTATGAAGAGAGTCGTAGA	0.039024022	0.114870937	-0.473503904
B_98618_AGTGGTCAAACGTGATCCAGTTCA	0.043091674	0.145827692	-0.368849638
B_98618_AGTGGTCACGAACTTACGACACAC	0.599149306	0.263712328	-0.215580161
B_98618_ATAGCGACACAGATTCAATCCGTC	0.672158758	0.07609542	-0.418211959
B_98618_ATAGCGACCGACACACATCCTGTA	0.118480901	0.265360425	-0.294823271
B_98618_ATAGCGACTGGTGGTAATCATTCC	-0.125516093	0.027294207	-0.269012609
B_98618_ATCATTCCAATGTTGCCACCTTAC	0.152961881	0.058344676	-0.496498865
B_98618_ATCATTCCACGCTCGACTGAGCCA	-0.038752775	0.015518373	-0.565478918
B_98618_ATCCTGTACCATCCTCTGGAACAA	-0.048776239	0.111450484	-0.391579585
B_98618_ATCCTGTAGTCGTAGATCCGTCTA	0.09571759	0.171133693	-0.425075432
B_98618_ATGCCTAACCGTGAGAGCCAAGAC	0.404022641	0.176570025	-0.520158964
B_98618_ATGCCTAACCTCCTGAAGTACAAG	0.159033878	0.177087149	-0.097813451
B_98618_ATGCCTAATAGGATGACCTCTATC	0.57031953	0.198913327	-0.441040393
B_98618_ATTGAGGAACAAGCTACCTCTATC	-0.062423232	-0.077074974	-0.52193954
B_98618_ATTGAGGAACGCTCGAGCTAACGA	0.456914816	0.13319062	-0.199413469
B_98618_ATTGAGGACGACTGGAAGTCACTA	0.472553831	0.081057749	-0.330747089
B_98618_ATTGGCTCACAGATTCCACTTCGA	-0.02890083	0.153992317	-0.333561234
B_98618_ATTGGCTCACTATGCAAACCGAGA	0.205491175	0.027489479	-0.317761635
B_98618_ATTGGCTCCCGAAGTATCCGTCTA	0.088762552	0.129172514	-0.484507596
B_98618_ATTGGCTCGCTAACGACAAGGAGC	-0.096935162	0.08711606	-0.30241712
B_98618_CAACCACAAGAGTCAAAACGTGAT	0.134581455	0.162354244	-0.404780015
B_98618_CAACCACACATACCAAGCTAACGA	0.39682046	0.362778538	-0.248203525

B_98618_CAAGACTAAAGGACACCCGTGAGA	0.135899629	0.107525441	-0.262711498
B_98618_CAAGACTAACACAGAAATGCCTAA	-0.027039311	0.135606663	-0.447803742
B_98618_CAAGACTAATCATTCCACGTATCA	0.122815284	-0.004220164	-0.383815855
B_98618_CAAGACTACTGAGCCACGAACTTA	0.101289762	0.133246269	-0.29099627
B_98618_CAAGACTAGATAGACAGATGAATC	0.16381866	0.022008583	-0.401588122
B_98618_CAAGGAGCCTCAATGAAGATCGCA	0.229946952	0.175559716	-0.403901767
B_98618_CAATGGAAAAGAGATCAAGACGGA	0.016565973	-0.050115364	-0.561825887
B_98618_CAATGGAACACTTCGAGAATCTGA	0.575831054	0.140595146	-0.239957859
B_98618_CAATGGAATGGCTTCACTGGCATA	0.717114067	0.169937311	-0.186496496
B_98618_CACCTTACACACGACCGCTCGGTA	0.27470661	0.215814386	-0.389220135
B_98618_CACCTTACGACAGTGCGAGCTGAA	0.327135286	0.16909621	-0.457358637
B_98618_CACTTCGACAGATCTGGTACGCAA	0.153846366	0.041411181	-0.469340161
B_98618_CACTTCGACCGAAGTAAGCACCTC	0.139521996	0.057115533	-0.386647449
B_98618_CAGATCTGAGATCGCACCAGTTCA	0.132688749	0.078466438	-0.401150728
B_98618_CAGATCTGATTGGCTCACCACTGT	0.273735827	0.31944742	-0.117612653
B_98618_CAGATCTGCAATGGAACTCAATGA	0.224446599	0.033047836	-0.51846127
B_98618_CAGCGTTAAAGAGATCCAAGGAGC	0.313211942	0.222996066	-0.431989321
B_98618_CAGCGTTAACATTGGCAATCCGTC	0.073276191	0.355568981	-0.00882126
B_98618_CAGCGTTAACCACTGTATGCCTAA	0.115432674	0.031027254	-0.508954537
B_98618_CAGCGTTAGAGCTGAACCGAAGTA	0.067628616	-0.022093141	-0.453696813
B_98618_CATCAAGTAAGACGGAATGCCTAA	0.378823415	0.053868288	-0.46982288
B_98618_CATCAAGTACACGACCACAAGCTA	0.318178964	0.104169373	-0.436442796
B_98618_CATCAAGTAGTGGTCAATTGGCTC	0.441573663	-0.015527779	-0.550078294
B_98618_CATCAAGTGAACAGGCACGTATCA	0.365246843	0.066360635	-0.194300672
B_98618_CCAGTTCACCTCTATCGCCACATA	0.42658531	0.311428076	-0.150267982
B_98618_CCATCCTCCAAGACTATGGCTTCA	0.006329405	0.141686354	-0.485441628
B_98618_CCATCCTCCAGATCTGATTGAGGA	0.246824073	0.138840825	-0.218569796
B_98618_CCATCCTCGCCAAGACAGAGTCAA	0.035599129	0.111015665	-0.401945387
B_98618_CCGAAGTAAAACATCGCCATCCTC	0.141663785	-0.067834227	-0.595608785
B_98618_CCGAAGTAAACAACCACTGGCATA	0.14832721	0.072952091	-0.467935915
B_98618_CCGAAGTAAACGTGATACACAGAA	-0.053255602	0.119192195	-0.490897298
B_98618_CCGAAGTAAGCCATGCGTCTGTCA	0.052639977	0.066488104	-0.390997991

B_98618_CCGAAGTACGGATTGCCCGTGAGA	0.049225248	0.208814738	-0.303934684
B_98618_CCGACAACCCTAATCCACACAGAA	0.223307454	0.124148463	-0.354961066
B_98618_CCGACAACGAGTTAGCAGAGTCAA	0.219453557	0.047267794	-0.473907204
B_98618_CCGACAACTAGGATGACAAGACTA	0.211681207	0.206038436	-0.290999927
B_98618_CCGTGAGAAGCACCTCAGAGTCAA	0.146308342	0.177337992	-0.334281365
B_98618_CCGTGAGACGAACTTAAGATGTAC	-0.017281771	0.281931871	-0.507466349
B_98618_CCTAATCCCGGATTGCATAGCGAC	0.302549277	0.148311374	-0.440107172
B_98618_CCTAATCCGAGTTAGCACAGCAGA	0.171072994	0.188801322	-0.471764421
B_98618_CCTAATCCGCTCGGTACCGAAGTA	0.065254718	-0.033233563	-0.450356945
B_98618_CCTCCTGAGGAGAACACAACCACA	0.086117105	0.062208207	-0.321611454
B_98618_CCTCCTGATGGTGGTAATAGCGAC	-0.063771097	0.209780846	-0.106985616
B_98618_CCTCTATCACAAGCTACACTTCGA	0.004344519	0.134888445	-0.482335556
B_98618_CGAACTTAAGCAGGAACCATCCTC	0.029039646	0.150300886	-0.386490067
B_98618_CGAACTTAATAGCGACTTCACGCA	-0.012429576	0.212564531	-0.333547108
B_98618_CGAACTTACTGGCATATAGGATGA	0.168399716	0.249692544	-0.319488841
B_98618_CGACACACACAAGCTACCATCCTC	-0.008313204	0.12726298	-0.26548454
B_98618_CGACTGGAGTCTGTCAATCCTGTA	0.158375654	0.155726682	-0.487201543
B_98618_CGCATACAAGATCGCACAAGACTA	0.132749556	0.070799017	-0.377489659
B_98618_CGCATACACAATGGAAGAATCTGA	0.245157772	0.259896477	-0.162754069
B_98618_CGCTGATCCTGGCATACATCAAGT	0.61677703	0.122707686	-0.220225073
B_98618_CGCTGATCGAGTTAGCCCAGTTCA	0.11308245	0.154712844	-0.423212084
B_98618_CTAAGGTCAACGCTTAACCTCCAA	0.277781413	0.079116328	-0.313457496
B_98618_CTCAATGAAAGAGATCAGATGTAC	0.187186546	0.113866551	-0.356130062
B_98618_CTGAGCCAAAGGTACAACGCTCGA	0.050989242	0.089335712	-0.272475093
B_98618_CTGAGCCAAAGGTACACAAGGAGC	0.063714335	-0.080944237	-0.413864115
B_98618_CTGAGCCAACTATGCAACACAGAA	0.264108683	0.160444315	-0.212516081
B_98618_CTGAGCCAAGCACCTCGCGAGTAA	0.114876595	0.177058237	-0.38536793
B_98618_CTGAGCCAAGGCTAACTGGTGGTA	0.019631627	0.058399813	-0.524185274
B_98618_CTGAGCCAATTGGCTCTGGAACAA	-0.063024252	0.009273174	-0.356386498
B_98618_CTGAGCCACACTTCGACATCAAGT	0.42197609	0.174223148	-0.450949739
B_98618_CTGAGCCACCATCCTCCGACTGGA	0.038955046	0.221256215	-0.080034847
B_98618_CTGAGCCACCGTGAGAAGCACCTC	0.304842969	0.171543525	-0.506325141

B_98618_CTGGCATAACAGATTCATGCCTAA	0.130631428	0.231450597	-0.412084343
B_98618_CTGGCATACCGTGAGATCTTCACA	0.032664552	0.135904634	-0.223379507
B_98618_CTGGCATACGCATACATTCACGCA	0.053682143	0.138683722	-0.218815407
B_98618_CTGTAGCCAACGTGATCCGTGAGA	0.289090938	0.186505729	-0.431005271
B_98618_CTGTAGCCACGTATCACGACACAC	0.051503033	0.167117738	-0.419915185
B_98618_CTGTAGCCAGTCACTACAAGGAGC	0.541130279	0.100353942	-0.454231842
B_98618_CTGTAGCCCAGATCTGCAGATCTG	0.171382629	0.120181558	-0.458642972
B_98618_CTGTAGCCCCTCTATCGATGAATC	0.170755094	0.047399545	-0.333017178
B_98618_CTGTAGCCGAACAGGCCTAAGGTC	-0.028399595	-0.067787139	-0.602280493
B_98618_CTGTAGCCGAGCTGAACTAAGGTC	0.386599256	0.183859526	-0.406483468
B_98618_GAACAGGCCCGACAACCGACTGGA	-0.0364203	-0.016688986	-0.446891254
B_98618_GAATCTGAAAGGTACACAGATCTG	0.236305166	0.037796597	-0.194542487
B_98618_GAATCTGACTGTAGCCGCTAACGA	0.068169967	0.17682923	-0.268717917
B_98618_GAATCTGAGCCAAGACCAAGGAGC	0.219296934	0.174840926	-0.373538778
B_98618_GACAGTGCAAACATCGGCTCGGTA	0.247054733	0.244282188	-0.453638891
B_98618_GACAGTGCAACAACCACAATGGAA	0.347289821	0.08856696	-0.479067165
B_98618_GACAGTGCGCTCGGTAGACTAGTA	0.085783588	-0.032067265	-0.478845862
B_98618_GACTAGTACAGATCTGCGGATTGC	0.67134889	0.414500058	-0.400033146
B_98618_GACTAGTAGACAGTGCAATCCGTC	0.452380743	0.005739295	-0.410487481
B_98618_GAGCTGAAAACGTGATACAAGCTA	0.228204079	0.097872018	-0.458736063
B_98618_GAGCTGAAGATAGACAAAGACGGA	0.230624802	0.179298979	-0.27443261
B_98618_GAGTTAGCCGAACTTACCGACAAC	0.561505123	0.126754908	-0.481715451
B_98618_GATGAATCAAGAGATCATCCTGTA	0.541218196	0.067634437	-0.423562719
B_98618_GATGAATCACGCTCGAAGTGGTCA	0.208013902	0.186534471	-0.464322017
B_98618_GCCAAGACAAACATCGCACTTCGA	0.850841026	0.06898714	-0.58690001
B_98618_GCCAAGACACAAGCTAGTACGCAA	0.12465463	0.060625727	-0.320297549
B_98618_GCCAAGACCCTAATCCGATGAATC	0.235536666	0.157571795	-0.253551757
B_98618_GCCACATACCTCCTGACGGATTGC	0.250933601	0.133607973	-0.347629615
B_98618_GCTAACGACCTCTATCTTCACGCA	0.259180131	0.132923768	-0.431245985
B_98618_GCTAACGACTGAGCCAGAGCTGAA	0.271941049	0.137962448	-0.436624632
B_98618_GCTAACGAGCCACATAATAGCGAC	0.436592641	0.173785454	-0.311472885
B_98618_GCTCGGTAAAGAGATCACCTCCAA	0.579437301	0.119101565	-0.296636672

B_98618_GCTCGGTAAGATCGCAAGATCGCA	0.220240961	0.260880831	-0.390680639
B_98618_GCTCGGTAAGGCTAACAGATGTAC	0.310961487	0.211564405	-0.32870144
B_98618_GCTCGGTATCTTCACACGACTGGA	0.337087102	0.025359695	-0.481742947
B_98618_GGAGAACAAACGTGATACACAGAA	-0.046612389	0.09869179	-0.516305212
B_98618_GGAGAACAGTACGCAAACAAGCTA	0.241502247	0.080944557	-0.477887993
B_98618_GGTGCGAAATAGCGACACCACTGT	0.60133097	0.105592082	-0.483938564
B_98618_GGTGCGAACGCTGATCCGCTGATC	0.351162854	0.168492586	-0.161111165
B_98618_GTACGCAAACAAGCTAAGTACAAG	0.68473708	0.196733901	-0.464866473
B_98618_GTACGCAAAGAGTCAAGAATCTGA	0.032822352	0.111055317	-0.443775719
B_98618_GTACGCAAGCTCGGTAATTGGCTC	0.113494142	0.122859136	-0.307069695
B_98618_GTACGCAATTCACGCAAACTCACC	-0.075597845	0.125003217	-0.242510588
B_98618_GTCGTAGACAACCACAAGTGGTCA	0.328466855	0.113331702	-0.403602433
B_98618_GTCTGTCAACAAGCTAAGTGGTCA	0.112176976	0.129968671	-0.371507114
B_98618_GTGTTCTAATTGGCTCAGTGGTCA	0.178266363	0.090354158	-0.468720201
B_98618_GTGTTCTACCATCCTCAACGTGAT	0.225070231	0.10291922	-0.455697355
B_98618_GTGTTCTACCGACAACAGATCGCA	0.087608131	0.094296621	-0.17932589
B_98618_GTGTTCTACGACTGGACCATCCTC	0.011367738	0.260298462	-0.275690082
B_98618_GTGTTCTATAGGATGAAGATCGCA	0.392176438	0.215121603	-0.291168766
B_98618_TAGGATGAAGAGTCAACCGTGAGA	0.046065677	0.180446399	-0.231478969
B_98618_TAGGATGAAGATCGCATGAAGAGA	0.14447517	0.127054218	-0.236474773
B_98618_TAGGATGAAGCACCTCCACCTTAC	0.02580436	0.093218582	-0.571274551
B_98618_TAGGATGAAGCCATGCAACGCTTA	0.057151289	0.129990017	-0.413248319
B_98618_TAGGATGACATACCAAAGCAGGAA	0.455184319	0.165426301	-0.344603321
B_98618_TAGGATGACCGACAACCAAGACTA	0.410209855	0.166922805	-0.452412188
B_98618_TAGGATGACTGGCATAATTGAGGA	0.153510309	0.101690299	-0.477103114
B_98618_TAGGATGATGAAGAGAAATCCGTC	0.030375387	0.137971572	-0.40768851
B_98618_TATCAGCAGATGAATCAGATGTAC	0.279543858	0.267190622	-0.291594906
B_98618_TATCAGCATGGCTTCAACGTATCA	0.196878404	0.16743884	-0.498423882
B_98618_TCCGTCTAATAGCGACTATCAGCA	0.197370967	0.080330142	-0.455714261
B_98618_TCTTCACAAGATGTACAATCCGTC	0.003623531	0.185130569	-0.150845887
B_98618_TCTTCACATGGAACAAGCGAGTAA	0.006384996	0.253353477	-0.392140469
B_98618_TGAAGAGAAAACATCGGAGTTAGC	0.345108756	0.118425857	-0.146504829

B_98618_TGAAGAGAAGTCACTAAAGACGGA	-0.136265907	0.052735362	-0.351273561
B_98618_TGGAACAAAACCGAGAAAGACGGA	0.387134834	-0.038045236	-0.360248627
B_98618_TGGAACAAACAGATTCACTATGCA	0.054302994	0.051347768	-0.403383981
B_98618_TGGAACAAAGCCATGCCACCTTAC	0.124090414	0.216670097	-0.276379668
B_98618_TGGAACAACAAGGAGCAGCCATGC	0.327300688	0.131675956	-0.269276069
B_98618_TGGAACAAGGAGAACACAACCACA	0.290207105	0.290088574	-0.230955526
B_98618_TGGCTTCAAGAGTCAATGGCTTCA	-0.172589856	0.094748277	-0.208208021
B_98618_TGGTGGTAAACAACCAACGTATCA	0.202437563	0.173822225	-0.463186787
B_98618_TGGTGGTAGTGTTCTATCTTCACA	0.193120373	0.247921135	-0.438356799
ab_99547_AACAACCAATCCTGTACAGATCTG	0.575699644	0.198269267	-0.391960979
ab_99547_AACTCACCCCATCCTCTTCACGCA	0.694892236	0.004928995	-0.538985855
ab_99547_AAGGTACAACAGCAGAAATCCGTC	0.374736785	0.071327299	-0.440349973
ab_99547_AATGTTGCCATACCAAACAGCAGA	0.849939348	0.134439239	-0.401778556
ab_99547_ACCACTGTTATCAGCAACGCTCGA	0.445028216	0.07044087	-0.364521598
ab_99547_AGATGTACCAGATCTGCTGGCATA	0.480411791	0.142396934	-0.388238189
ab_99547_ATCATTCCTCTTCACACTGAGCCA	0.417734062	0.159971576	-0.365733449
ab_99547_CACCTTACGAGCTGAAACAGCAGA	0.741534125	0.11139269	-0.511134447
ab_99547_CTGTAGCCAAGGTACATGGTGGTA	0.636072236	0.165700287	-0.220293517
ab_99547_CTGTAGCCGCCAAGACCGCATACA	0.562929189	0.029350272	-0.531285509
ab_99547_GACAGTGCAACAACCAAAGACGGA	0.833824996	0.108912762	-0.360769378
ab_99547_GATAGACACAACCACATCCGTCTA	0.637839093	0.001232748	-0.442137603
ab_99547_TCTTCACAAACAACCACATACCAA	0.523415128	0.037895175	-0.43287716
ab_99547_AAACATCGCGACACACAATGTTGC	0.978164853	0.207209403	-0.493900983
ab_99547_AAACATCGGAATCTGACATCAAGT	1.215477932	0.388167422	-0.456838185
ab_99547_AAACATCGGAGTTAGCACAGCAGA	1.054757645	0.232290866	-0.342201527
ab_99547_AACCGAGAACATTGGCCCAGTTCA	0.495983898	0.143827999	-0.450156597
ab_99547_AACCGAGAGAGTTAGCATGCCTAA	0.674981924	0.309734018	-0.525279708
ab_99547_AACGCTTACAAGACTACTAAGGTC	0.481291984	0.350526804	-0.566622885
ab_99547_AACGCTTAGTACGCAACAAGGAGC	0.734811863	0.221802856	-0.449495162
ab_99547_AACGTGATAGTACAAGCCGACAAC	0.981373234	0.200547179	-0.372717031
ab_99547_AACGTGATCGCATACACCGTGAGA	0.740796853	0.121707829	-0.553138176
ab_99547_AACGTGATCTAAGGTCCAACCACA	0.95924413	0.332399821	-0.401017611

ab_99547_AACGTGATTAGGATGAAGCCATGC	0.643903044	0.244289688	-0.306422587
ab_99547_AACTCACCAATGTTGCTATCAGCA	0.796172956	0.401770441	-0.37534452
ab_99547_AACTCACCCTAAGGTCGCTAACGA	1.232190483	0.416172959	-0.302021621
ab_99547_AAGACGGAGGTGCGAAGTGTTCTA	0.770599939	0.36837719	-0.292833107
ab_99547_AAGAGATCATCCTGTAGAGCTGAA	0.336539818	-0.028273043	-0.403895481
ab_99547_AAGAGATCCGAACTTAAACCGAGA	1.070361293	0.264251725	-0.338099838
ab_99547_AAGAGATCGCTAACGATGGCTTCA	0.833871437	0.385526747	-0.326381359
ab_99547_AAGGACACAACAACCAAACCGAGA	1.036180335	0.429946103	-0.323966297
ab_99547_AAGGACACGCCAAGACGAGCTGAA	1.291689138	0.263797969	-0.514216503
ab_99547_AAGGTACACCATCCTCCAAGACTA	0.745129834	0.185739842	-0.479610239
ab_99547_AAGGTACACTCAATGAAGTCACTA	0.776348088	0.490413096	-0.521347685
ab_99547_AAGGTACACTCAATGACACTTCGA	0.555717758	0.128224695	-0.51900923
ab_99547_AATCCGTCGTCTGTCACAAGACTA	0.808442638	0.247769342	-0.380877
ab_99547_AATGTTGCCCTAATCCCATCAAGT	0.642818827	0.271830097	-0.539566291
ab_99547_AATGTTGCCGAACTTAAACTCACC	0.576590521	0.111151033	-0.397835287
ab_99547_ACAAGCTACTGAGCCAAGCAGGAA	0.640167628	0.144544187	-0.565537224
ab_99547_ACAAGCTAGCGAGTAATAGGATGA	0.841209372	0.309753891	-0.519856729
ab_99547_ACACAGAAAAGGTACATGAAGAGA	0.697653412	0.605450599	-0.44467983
ab_99547_ACACAGAACAATGGAAACCTCCAA	0.742417814	0.546633743	-0.477963299
ab_99547_ACACAGAACCGAAGTAAGCAGGAA	0.902456242	0.226619067	-0.476486532
ab_99547_ACACAGAACCGTGAGAACGTATCA	0.919096982	0.21833482	-0.54488424
ab_99547_ACACGACCATGCCTAAAAGGTACA	1.053565218	0.438349771	-0.511646339
ab_99547_ACACGACCGAGTTAGCGAGTTAGC	0.747900145	0.253496945	-0.441066219
ab_99547_ACAGATTCGTCTGTCACAATGGAA	0.8348015	0.445898301	-0.397519969
ab_99547_ACAGATTCTGAAGAGACGGATTGC	1.14645962	0.27891637	-0.421749052
ab_99547_ACAGCAGAAGCCATGCCTGTAGCC	0.974365152	0.341776672	-0.318487684
ab_99547_ACATTGGCAACTCACCGATGAATC	0.805655482	0.402481341	-0.403001152
ab_99547_ACATTGGCCCGTGAGAAGTACAAG	0.461495119	0.31227199	-0.427007422
ab_99547_ACCACTGTAATGTTGCGCGAGTAA	0.563463999	0.330019538	-0.402301092
ab_99547_ACCACTGTATTGAGGAACACAGAA	0.595632317	0.17249311	-0.346074834
ab_99547_ACCACTGTCACTTCGACTCAATGA	0.816241633	-0.025113632	-0.492803514
ab_99547_ACCACTGTGGAGAACAAGATCGCA	0.760062866	0.143032696	-0.410009262

ab_99547_ACCTCCAACAGATCTGTGGTGGTA	0.76880094	0.245824457	-0.550177924
ab_99547_ACGCTCGAACACAGAAGAATCTGA	0.868245343	0.232538829	-0.476432983
ab_99547_ACGCTCGAACGTATCAAGCACCTC	0.909168682	0.290230965	-0.498840364
ab_99547_ACGCTCGAGTACGCAAAGTGGTCA	0.769032529	0.363934172	-0.321339099
ab_99547_ACGTATCACAACCACAAACGCTTA	0.546517635	0.217307308	-0.405263714
ab_99547_ACGTATCACTGGCATAAGGCTAAC	0.82286427	0.30873808	-0.384191699
ab_99547_ACGTATCAGGAGAACAAGAGTCAA	0.955825162	0.288645636	-0.469231734
ab_99547_ACGTATCATTCACGCAACAAGCTA	0.746193325	0.314507525	-0.509651896
ab_99547_ACTATGCACCTAATCCGTACGCAA	0.648128044	0.286811623	-0.555726395
ab_99547_ACTATGCATAGGATGAAAGAGATC	0.711412656	0.293552392	-0.367882817
ab_99547_ACTATGCATCCGTCTACGACACAC	0.277551109	0.175016206	-0.384058095
ab_99547_ACTATGCATGGAACAACAAGGAGC	0.563067707	0.094532724	-0.325251092
ab_99547_AGAGTCAAATCATTCCGTACGCAA	0.455291586	0.191516688	-0.395780474
ab_99547_AGATCGCAATTGGCTCACAAGCTA	0.634713578	0.252127887	-0.309495615
ab_99547_AGATCGCACGGATTGCCCAGTTCA	0.927850593	0.192644203	-0.552455662
ab_99547_AGATCGCATCCGTCTAAGATGTAC	1.095990859	0.314315091	-0.486930491
ab_99547_AGATGTACAGTGGTCAACGTATCA	0.912434034	0.218291314	-0.561526836
ab_99547_AGATGTACATCCTGTAACAGATTC	0.762834175	0.211132058	-0.420267577
ab_99547_AGATGTACATTGGCTCTTCACGCA	0.971651188	0.158903268	-0.547412556
ab_99547_AGATGTACCCTAATCCAACGTGAT	0.595846157	0.071393925	-0.372767124
ab_99547_AGATGTACCTGGCATAGAGTTAGC	1.052314167	0.107355817	-0.566813498
ab_99547_AGCACCTCCATCAAGTAGATCGCA	0.704604791	0.454813493	-0.351114209
ab_99547_AGCACCTCGAGTTAGCTATCAGCA	0.759509777	0.328646782	-0.415489221
ab_99547_AGCAGGAACCATCCTCTGGCTTCA	1.07380832	0.282120361	-0.538192516
ab_99547_AGCAGGAACGGATTGCGCGAGTAA	1.025830538	0.039320513	-0.531890962
ab_99547_AGCAGGAAGACTAGTAACAAGCTA	0.910153437	0.319975304	-0.520111643
ab_99547_AGCCATGCCCAGTTCAATCATTCC	0.50270951	0.199291768	-0.538526493
ab_99547_AGCCATGCCTGAGCCATCCGTCTA	0.619902336	0.151499771	-0.557569149
ab_99547_AGCCATGCCTGGCATACTAAGGTC	1.348190865	0.477563122	-0.558836604
ab_99547_AGCCATGCGAGCTGAAGAGTTAGC	0.92109667	0.207809062	-0.360092547
ab_99547_AGCCATGCGTCGTAGACGACTGGA	0.687446694	0.251781651	-0.429851527
ab_99547_AGCCATGCTCCGTCTAAGATCGCA	1.038890182	0.380874291	-0.512122481

ab_99547_AGGCTAACAGATGTACGTACGCAA	0.355838932	0.154122671	-0.462442602
ab_99547_AGGCTAACCCAGTTCACATACCAA	0.843266218	0.433399492	-0.546899126
ab_99547_AGGCTAACCTGAGCCACCGTGAGA	0.527556558	0.144992941	-0.533770147
ab_99547_AGTACAAGAGCCATGCGACAGTGC	0.958970857	0.282234011	-0.507534759
ab_99547_AGTACAAGCCTAATCCGAATCTGA	1.052249188	0.432209142	-0.450013345
ab_99547_AGTCACTACAGATCTGATTGGCTC	0.472792809	0.277285441	-0.32818335
ab_99547_AGTCACTACTAAGGTCCGACACAC	1.189334141	0.38005713	-0.570835062
ab_99547_AGTGGTCAACAGATTCAGTGGTCA	0.795846264	0.366437755	-0.327221108
ab_99547_AGTGGTCACTGAGCCAGCTCGGTA	0.885987645	0.368135003	-0.448633541
ab_99547_ATAGCGACCGAACTTACGGATTGC	0.82471112	0.046119878	-0.476557576
ab_99547_ATAGCGACTCCGTCTAAGCCATGC	0.825770014	0.257164517	-0.447462572
ab_99547_ATCATTCCAACAACCAAGCAGGAA	0.734887748	0.289245647	-0.296558021
ab_99547_ATCATTCCACCACTGTCCTCCTGA	0.782637739	0.36209966	-0.379246818
ab_99547_ATCATTCCATCCTGTATTCACGCA	0.394372238	0.167847299	-0.360367772
ab_99547_ATCATTCCCAACCACACATACCAA	0.822868951	0.379191142	-0.469948787
ab_99547_ATCATTCCCATCAAGTACAGCAGA	0.611750621	0.330113537	-0.486174851
ab_99547_ATCATTCCCCATCCTCCTGGCATA	0.69356462	0.544324309	-0.470279215
ab_99547_ATCATTCCCCGTGAGATGGAACAA	0.857319676	0.225051783	-0.43264558
ab_99547_ATCATTCCCTGGCATAACAAGCTA	0.887302538	0.470150147	-0.365015449
ab_99547_ATCATTCCGATGAATCCCGACAAC	0.725590362	0.046012258	-0.500374699
ab_99547_ATCATTCCGCCACATATGGTGGTA	0.850457263	0.440136976	-0.495035775
ab_99547_ATCATTCCTGAAGAGAACACGACC	0.773432024	0.122671262	-0.510594514
ab_99547_ATCCTGTAAAGGACACATCCTGTA	0.908216884	0.296612644	-0.466420188
ab_99547_ATCCTGTACTAAGGTCATAGCGAC	0.560567859	0.349165891	-0.479683646
ab_99547_ATCCTGTAGACAGTGCACCACTGT	0.603622005	0.237525648	-0.440248654
ab_99547_ATCCTGTAGCTAACGAAAACATCG	0.883164336	0.477978394	-0.57365821
ab_99547_ATGCCTAAGTACGCAAAACCGAGA	0.774891201	0.22997033	-0.477677552
ab_99547_ATTGAGGAAGATCGCAGACAGTGC	0.796961884	0.052742376	-0.541133289
ab_99547_ATTGAGGAATCCTGTAAGTCACTA	0.571533548	0.366251198	-0.379721362
ab_99547_ATTGAGGATGAAGAGAGATGAATC	0.866550856	0.351658893	-0.456107474
ab_99547_ATTGGCTCAACAACCAGATGAATC	1.284990866	0.380475005	-0.538364578
ab_99547_ATTGGCTCACAAGCTAAAGGTACA	0.501308312	0.057132142	-0.457783976

ab_99547_ATTGGCTCACGCTCGAACGTATCA	0.526342378	0.234968389	-0.539405211
ab_99547_ATTGGCTCCAGATCTGTCCGTCTA	1.316129746	0.368926087	-0.503673898
ab_99547_ATTGGCTCGCCAAGACAAGGACAC	0.977135957	0.134671407	-0.450591456
ab_99547_ATTGGCTCGCGAGTAAAGCCATGC	0.71545421	0.212779018	-0.385980544
ab_99547_CAACCACAGCCACATAGAGTTAGC	0.525038411	0.346347488	-0.361773724
ab_99547_CAAGACTAAATCCGTCAGTGGTCA	1.018652385	0.463595722	-0.292485476
ab_99547_CAAGACTAATCATTCCTGAAGAGA	1.052491542	0.298723992	-0.408338353
ab_99547_CAAGACTAATCCTGTAAAACATCG	0.727115165	0.267795084	-0.451068029
ab_99547_CAAGACTACTAAGGTCCCGAAGTA	0.538592706	0.205563335	-0.449827365
ab_99547_CAAGACTAGCCACATAGTACGCAA	0.582164307	0.185227244	-0.404112284
ab_99547_CAAGGAGCAGATGTACCGACACAC	0.610159966	0.045758401	-0.526369023
ab_99547_CAAGGAGCATAGCGACATAGCGAC	0.940394751	0.443577639	-0.383868865
ab_99547_CAAGGAGCCGAACTTAGTACGCAA	0.973987908	0.406468754	-0.445108979
ab_99547_CAATGGAAATAGCGACACACAGAA	0.760134826	0.148798874	-0.506611673
ab_99547_CAATGGAACAGCGTTAATCCTGTA	1.069900473	0.27234987	-0.436813894
ab_99547_CAATGGAAGACTAGTACGACACAC	0.824132546	0.211664466	-0.417259512
ab_99547_CACCTTACGTGTTCTACGACACAC	0.872692194	0.493423603	-0.561880526
ab_99547_CACTTCGAATGCCTAAATAGCGAC	0.967896859	0.161579219	-0.514733889
ab_99547_CACTTCGAGACAGTGCGAATCTGA	0.86956529	0.198840908	-0.499417796
ab_99547_CAGATCTGACCACTGTCCTCTATC	0.578982859	0.240240151	-0.539761682
ab_99547_CAGATCTGAGAGTCAAGCCACATA	0.845472573	0.061415837	-0.587472607
ab_99547_CAGCGTTAAACGTGATCCGAAGTA	1.315707316	0.587739518	-0.506277304
ab_99547_CAGCGTTAACACAGAAACACAGAA	1.011722864	0.184996757	-0.565872245
ab_99547_CAGCGTTAACACAGAACCGACAAC	1.080012715	0.58474825	-0.601350438
ab_99547_CAGCGTTAACCACTGTAGCCATGC	0.698159309	0.094428477	-0.485739642
ab_99547_CAGCGTTACCATCCTCAGTGGTCA	0.712564659	0.289104214	-0.450699895
ab_99547_CAGCGTTACCTCCTGACTGAGCCA	0.587785356	0.336594663	-0.489219429
ab_99547_CAGCGTTACTAAGGTCTTCACGCA	0.521005631	0.317159268	-0.444024081
ab_99547_CAGCGTTAGACTAGTAACCACTGT	0.923823182	0.382071611	-0.437375134
ab_99547_CATACCAAACAGATTCAGGCTAAC	0.749532884	0.402049987	-0.362281832
ab_99547_CATACCAAAGTACAAGATAGCGAC	0.949736382	0.28176315	-0.527972262
ab_99547_CATACCAAATCCTGTACTAAGGTC	0.630068085	0.246552891	-0.41774165

ab_99547_CATACCAAGGAGAACACATCAAGT	0.676627232	0.271022565	-0.394998985
ab_99547_CATCAAGTAGATCGCAAGATCGCA	1.077723362	0.213892561	-0.42297583
ab_99547_CATCAAGTATTGGCTCACGTATCA	0.533378443	0.127933773	-0.310257929
ab_99547_CATCAAGTCGGATTGCAGTGGTCA	0.704756591	0.148975705	-0.325376183
ab_99547_CATCAAGTGATAGACAAAGGTACA	0.753097503	0.298941445	-0.338619604
ab_99547_CCAGTTCAAACTCACCGAACAGGC	0.595031369	0.604769375	-0.401819631
ab_99547_CCAGTTCACGACACACCCTAATCC	0.93315302	0.273787553	-0.461801474
ab_99547_CCAGTTCAGCCACATAGACAGTGC	1.36305663	0.421209302	-0.469905583
ab_99547_CCATCCTCATCCTGTACACCTTAC	0.427990058	0.274210449	-0.457865684
ab_99547_CCGAAGTAATGCCTAAACATTGGC	0.691503327	0.201539895	-0.490440998
ab_99547_CCGAAGTAGAGCTGAACCATCCTC	0.779132478	0.447787367	-0.484727118
ab_99547_CCGAAGTATAGGATGACCGAAGTA	0.944835449	0.177599159	-0.48862586
ab_99547_CCGACAACACAAGCTAAGCAGGAA	0.939081797	0.356297787	-0.397269183
ab_99547_CCGTGAGAATCATTCCGAGCTGAA	0.641552846	0.113994522	-0.246588106
ab_99547_CCGTGAGACAATGGAAGATGAATC	0.763953122	0.155904274	-0.496693947
ab_99547_CCTAATCCATAGCGACCCGTGAGA	0.794647586	0.521802049	-0.359717674
ab_99547_CCTAATCCCAGCGTTATATCAGCA	0.63958835	0.033129419	-0.563182586
ab_99547_CCTAATCCCATACCAAAAGGACAC	0.80528027	0.281486447	-0.358706387
ab_99547_CCTAATCCCTGTAGCCCAAGACTA	0.814380895	0.149804754	-0.544806331
ab_99547_CCTAATCCGCCAAGACCTGAGCCA	1.007016428	0.23511396	-0.511963652
ab_99547_CCTCCTGAAGATGTACGCCAAGAC	0.644041621	0.108598352	-0.474241384
ab_99547_CCTCTATCACTATGCAGGAGAACA	0.839350149	0.223723259	-0.510168954
ab_99547_CGAACTTAAAACATCGACCACTGT	0.664810136	0.192819623	-0.412347113
ab_99547_CGAACTTAAAGACGGAGCTAACGA	0.982692449	0.511518906	-0.454591129
ab_99547_CGAACTTAACCACTGTCAAGGAGC	0.466946701	0.404505329	-0.328025748
ab_99547_CGAACTTAGACAGTGCCATACCAA	0.315143157	0.164970022	-0.54203366
ab_99547_CGACACACAAGGACACGTGTTCTA	0.980680298	0.430670059	-0.490859403
ab_99547_CGACACACAGAGTCAAATGCCTAA	0.693031888	0.128600412	-0.246506897
ab_99547_CGACACACGACAGTGCGCCACATA	1.002818386	0.194751913	-0.53656345
ab_99547_CGACACACGCCACATAACCACTGT	0.966272292	0.381146119	-0.385713949
ab_99547_CGACACACTCCGTCTATCTTCACA	0.705616599	0.300393389	-0.375203573
ab_99547_CGACACACTGGTGGTAACGTATCA	0.72149511	0.22121345	-0.616244321

ab_99547_CGACTGGAACAAGCTAAGTGGTCA	1.060776282	0.199324608	-0.450525902
ab_99547_CGACTGGAACGTATCAAACGTGAT	0.977146894	0.245933006	-0.482419094
ab_99547_CGACTGGACAATGGAAACGCTCGA	0.851025604	0.18816693	-0.505575643
ab_99547_CGACTGGACTGAGCCAGCCAAGAC	0.862473195	0.460734314	-0.543939312
ab_99547_CGCATACAACACGACCGCCAAGAC	0.907623928	0.398857007	-0.388584553
ab_99547_CGCATACACAATGGAACGACTGGA	0.78031322	0.302926018	-0.308769447
ab_99547_CGCATACAGAACAGGCAGTCACTA	0.671975993	0.161723188	-0.441375629
ab_99547_CGCTGATCGATAGACAAGCCATGC	0.756994312	0.575555948	-0.576445335
ab_99547_CTAAGGTCCAAGACTATCCGTCTA	0.781366787	0.201980787	-0.388678991
ab_99547_CTAAGGTCCTGAGCCAAAGGTACA	0.606468179	0.306497331	-0.504003805
ab_99547_CTCAATGAAGGCTAACCTAAGGTC	0.914548268	0.365948373	-0.532693045
ab_99547_CTCAATGACTCAATGAGAGCTGAA	0.571354127	0.192747355	-0.212258676
ab_99547_CTGAGCCAAACGCTTACATCAAGT	0.533775137	0.257586366	-0.395305226
ab_99547_CTGAGCCAATCATTCCGCGAGTAA	0.561343298	0.262269731	-0.488595557
ab_99547_CTGAGCCACTCAATGAGAGTTAGC	0.849407556	0.150537299	-0.321269189
ab_99547_CTGGCATAACGTATCAAGTCACTA	0.394529378	0.281763213	-0.484635115
ab_99547_CTGGCATACATCAAGTAGCCATGC	0.469977927	0.3766918	-0.416584879
ab_99547_CTGGCATAGATGAATCCTGGCATA	0.353257844	0.229764908	-0.161352347
ab_99547_CTGGCATAGCCAAGACCAATGGAA	0.880008263	0.363614383	-0.443497383
ab_99547_CTGTAGCCAACCGAGACCGACAAC	0.681591088	0.350726943	-0.386276487
ab_99547_GAACAGGCAAGAGATCAGATGTAC	0.674515233	0.293359289	-0.512991128
ab_99547_GAACAGGCAAGGACACCTGAGCCA	1.08018183	0.381493999	-0.426663055
ab_99547_GAATCTGAAGATGTACTGAAGAGA	0.681281947	0.53311005	-0.452103935
ab_99547_GAATCTGAATGCCTAAACAGCAGA	1.015095686	0.213802694	-0.509378754
ab_99547_GAATCTGAGTGTTCTAACTATGCA	1.109844806	0.305569978	-0.484340959
ab_99547_GACAGTGCAACCGAGAATTGGCTC	1.046434887	0.212156885	-0.57387033
ab_99547_GACTAGTAACAGCAGACAATGGAA	0.464857431	0.261769744	-0.389283011
ab_99547_GACTAGTACCAGTTCATGGAACAA	1.039836319	0.329023896	-0.461668234
ab_99547_GACTAGTACTAAGGTCCAGATCTG	0.850826385	0.41378874	-0.520229886
ab_99547_GACTAGTAGCCAAGACCCATCCTC	0.523291302	0.346439529	-0.41362198
ab_99547_GACTAGTAGTCGTAGAAGCACCTC	0.591238936	0.270717147	-0.519485706
ab_99547_GACTAGTATGGCTTCACCGACAAC	1.008654699	0.305837999	-0.433780837

ab_99547_GAGCTGAAACAGATTCTGAAGAGA	0.649238428	0.258559301	-0.465198316
ab_99547_GAGCTGAACCGTGAGACGAACTTA	0.57709249	0.271354016	-0.256833461
ab_99547_GAGCTGAACTAAGGTCCAATGGAA	0.85426975	0.309364311	-0.449058642
ab_99547_GAGTTAGCCTCAATGACCGTGAGA	0.618970729	0.206103639	-0.303770921
ab_99547_GATAGACAATGCCTAAAGCAGGAA	0.848138413	0.447404253	-0.360992029
ab_99547_GATAGACAATTGAGGACCAGTTCA	0.387048126	0.22978131	-0.445112435
ab_99547_GATAGACACCAGTTCATGGTGGTA	0.880748833	0.161033071	-0.416784245
ab_99547_GATAGACACCGACAACCCGACAAC	0.688363562	0.403811215	-0.341726035
ab_99547_GATAGACAGACAGTGCCAAGACTA	0.695180653	0.444555589	-0.198338479
ab_99547_GATGAATCATGCCTAAAAGAGATC	0.667537577	0.143830543	-0.489764176
ab_99547_GATGAATCGAGTTAGCATCATTCC	0.907827889	0.133379745	-0.485494414
ab_99547_GCCAAGACCCGACAACATTGAGGA	0.391792683	0.152149027	-0.477911794
ab_99547_GCCAAGACGTCTGTCACATACCAA	0.788754193	0.022180209	-0.539919853
ab_99547_GCCAAGACTCTTCACATGGTGGTA	0.545408758	0.454429262	-0.344701794
ab_99547_GCCACATAAACAACCAAACGTGAT	0.872907378	0.403872017	-0.475967506
ab_99547_GCCACATAAGAGTCAACGAACTTA	1.038341044	0.504541552	-0.499272455
ab_99547_GCCACATACAACCACAAATCCGTC	0.830190103	0.498849346	-0.361087733
ab_99547_GCCACATAGCTAACGAATCCTGTA	0.65060286	0.384280693	-0.549845728
ab_99547_GCCACATAGCTAACGACCAGTTCA	0.65816777	0.290145238	-0.379085765
ab_99547_GCGAGTAACCTCCTGAACAGATTC	1.065053425	0.63318867	-0.568206152
ab_99547_GCGAGTAAGTACGCAAACAAGCTA	0.643324685	0.363202409	-0.272630758
ab_99547_GCTAACGAAGCAGGAACTGTAGCC	0.77628683	0.126283873	-0.373330869
ab_99547_GCTAACGATGGTGGTAACGTATCA	1.17580724	0.556396559	-0.472672677
ab_99547_GCTCGGTACAACCACACCGAAGTA	0.816103975	0.176407388	-0.410186565
ab_99547_GCTCGGTATCCGTCTACTAAGGTC	0.83678666	0.292893637	-0.250207422
ab_99547_GGTGCGAAAAGGTACAGGTGCGAA	0.561657289	0.264706758	-0.312615676
ab_99547_GGTGCGAACAATGGAAGGTGCGAA	0.590610619	0.19845718	-0.486931427
ab_99547_GGTGCGAACACTTCGAAACGCTTA	0.997252331	0.543948427	-0.338201428
ab_99547_GGTGCGAACCATCCTCAACGCTTA	0.570783334	0.175398812	-0.519549968
ab_99547_GGTGCGAACGACTGGATGGAACAA	0.772087844	0.15803618	-0.416301175
ab_99547_GGTGCGAAGACAGTGCAAACATCG	0.534013053	0.070926177	-0.412747928
ab_99547_GGTGCGAATGAAGAGAGTCTGTCA	0.747854695	0.096891082	-0.540237442

ab_99547_GTACGCAAATCATTCCCAAGACTA	0.316910936	0.100516958	-0.466740128
ab_99547_GTACGCAATCCGTCTAACACAGAA	0.630822489	0.546100499	-0.347703141
ab_99547_GTCGTAGAAGATGTACACAAGCTA	0.761160386	0.196180303	-0.522489402
ab_99547_GTCGTAGACACTTCGAACACGACC	0.658940545	0.328044243	-0.512559512
ab_99547_GTCGTAGACTGGCATAAGCACCTC	0.741174346	0.170594889	-0.595489891
ab_99547_GTCGTAGAGAGTTAGCCGACACAC	0.659384832	0.205560283	-0.572864161
ab_99547_GTCGTAGAGCCAAGACAACAACCA	0.724848621	0.519602087	-0.521677729
ab_99547_GTCGTAGAGCGAGTAACGACACAC	0.678867833	0.143797766	-0.591315505
ab_99547_GTCTGTCAAACGTGATTATCAGCA	0.898762824	0.151312705	-0.512308945
ab_99547_GTCTGTCAATAGCGACAGTGGTCA	0.763618336	0.28434758	-0.285427893
ab_99547_GTCTGTCAATTGAGGAACGCTCGA	0.812778292	0.395980986	-0.517960419
ab_99547_GTGTTCTATCTTCACACGCATACA	1.06312522	0.186886783	-0.517369301
ab_99547_TAGGATGAAAGGTACATATCAGCA	0.665680661	0.075233287	-0.549399531
ab_99547_TAGGATGAACTATGCACAAGACTA	0.957493683	0.212749988	-0.288817238
ab_99547_TAGGATGACGCATACAGCTCGGTA	0.9937743	0.327103914	-0.454875667
ab_99547_TAGGATGACGCTGATCTAGGATGA	0.666325963	0.174473693	-0.494506697
ab_99547_TAGGATGAGAGCTGAAAGTCACTA	0.8547383	0.16269819	-0.5348145
ab_99547_TATCAGCAACAGCAGAAAACATCG	0.910759546	0.38948053	-0.268651297
ab_99547_TATCAGCACCAGTTCAACAGCAGA	0.304297974	0.426619758	-0.345918251
ab_99547_TCCGTCTAACGCTCGAACAGATTC	0.978620779	0.521568762	-0.521495037
ab_99547_TCCGTCTAAGAGTCAAAGTACAAG	0.958189671	0.133143679	-0.474995133
ab_99547_TCTTCACAACAGCAGAATTGGCTC	1.22014809	0.419437429	-0.479493257
ab_99547_TCTTCACAAGCAGGAAAGCACCTC	1.025235808	0.235784328	-0.476897321
ab_99547_TCTTCACAAGTGGTCACGGATTGC	0.417310952	0.188127634	-0.413376165
ab_99547_TCTTCACAGTCGTAGAAGCCATGC	0.837237976	0.138643712	-0.577478332
ab_99547_TGAAGAGAAACTCACCCTAAGGTC	0.469986258	0.12749167	-0.33575432
ab_99547_TGAAGAGAAAGGACACATAGCGAC	0.729208274	0.302447445	-0.484613524
ab_99547_TGAAGAGAAAGGTACAAGAGTCAA	0.804618843	0.058118839	-0.543968618
ab_99547_TGAAGAGAATCATTCCCTAAGGTC	1.027830721	0.433536637	-0.508083927
ab_99547_TGAAGAGACCAGTTCAACACAGAA	0.821383815	0.144230577	-0.480612148
ab_99547_TGAAGAGACGACACACGATGAATC	0.602970182	0.146608384	-0.475468854
ab_99547_TGGAACAAAATGTTGCGCGAGTAA	0.833745488	0.377248525	-0.532983401

ab_99547_TGGAACAACACCTTACCCGAAGTA	0.849054618	0.259201274	-0.376866246
ab_99547_TGGAACAACACCTTACCGACACAC	0.532818592	0.03809656	-0.577361796
ab_99547_TGGAACAACACCTTACGAGCTGAA	0.760111939	0.173696077	-0.466393664
ab_99547_TGGAACAATCTTCACAAACCGAGA	0.760054174	0.297993967	-0.514263494
ab_99547_TGGCTTCACGGATTGCCGAACTTA	0.853821725	0.363835458	-0.55448584
ab_99547_TGGTGGTACAAGACTAACAGATTC	1.057641956	0.07551438	-0.585408437
ab_99547_TGGTGGTACACTTCGATGGAACAA	0.967229141	0.205480581	-0.201489128
ab_99547_TTCACGCAACATTGGCGGAGAACA	0.658774239	0.157074545	-0.28637023
ab_99547_TTCACGCAAGAGTCAACTGGCATA	0.676094467	0.309611475	-0.531651428
ab_99547_TTCACGCAAGTACAAGAATGTTGC	1.092827573	0.182413653	-0.401575393
ab_99547_TTCACGCAGCTCGGTACTAAGGTC	1.067197094	0.302225032	-0.41236557
ab_99547_GACAGTGCGTGTTCTAATCCTGTA	0.677336363	0.349529067	-0.597384029
ab_99547_AAACATCGAACCGAGAGAGCTGAA	1.296955748	0.591358118	-0.59209281
ab_99547_AAACATCGTTCACGCAGAGTTAGC	0.978718704	1.255071104	-0.535325897
ab_99547_AACAACCAAGGCTAACACAAGCTA	0.671464031	0.506971517	-0.436618078
ab_99547_AACAACCATGGAACAAAACTCACC	0.945584834	0.799953579	-0.537647082
ab_99547_AACCGAGAAGAGTCAACCTCTATC	0.946956736	0.486336063	-0.569388968
ab_99547_AACCGAGAAGATCGCAAATGTTGC	0.582998216	0.56483249	-0.336299277
ab_99547_AACCGAGAATTGGCTCAACTCACC	0.593821225	0.631170002	-0.511109189
ab_99547_AACGCTTAACATTGGCATGCCTAA	0.661298908	0.60506453	-0.557641102
ab_99547_AACGCTTAACCACTGTGTGTTCTA	0.721005569	0.546193134	-0.314798235
ab_99547_AACGCTTAATTGAGGAACCACTGT	0.92176936	1.12866905	-0.394207627
ab_99547_AACGTGATAGCACCTCGAATCTGA	0.997374762	1.046977064	-0.451533452
ab_99547_AACGTGATCAGATCTGAGCCATGC	0.861725233	0.618382555	-0.541706184
ab_99547_AACGTGATGTACGCAAGCCAAGAC	0.515450893	0.569651385	-0.44714485
ab_99547_AACTCACCGCCACATAAATCCGTC	0.92023192	0.662594681	-0.408339506
ab_99547_AACTCACCGCTAACGAACACGACC	1.026526528	0.627063552	-0.232480312
ab_99547_AACTCACCGTCTGTCAACAGATTC	1.199996947	1.909028783	-0.388157303
ab_99547_AAGACGGATAGGATGAACGCTCGA	0.767641185	0.760080138	-0.279075254
ab_99547_AAGACGGATGAAGAGAAGTACAAG	0.799318392	0.68029003	-0.489515456
ab_99547_AAGAGATCGATAGACATGGAACAA	0.784609896	0.910994151	-0.329978145
ab_99547_AAGGACACATCATTCCAACTCACC	0.544586644	0.574038026	-0.582205259

ab_99547_AAGGACACGCCAAGACATTGAGGA	0.535513524	0.602169598	-0.509147994
ab_99547_AAGGTACAAGCAGGAAGACTAGTA	0.931715518	0.619694953	-0.244966479
ab_99547_AAGGTACAGTCTGTCAATCATTCC	0.761231452	0.666086271	-0.461257654
ab_99547_AATCCGTCACAAGCTAACACGACC	0.620977949	0.869019964	-0.502900662
ab_99547_AATCCGTCACCTCCAAAGCCATGC	1.079881357	0.631569844	-0.521253121
ab_99547_AATGTTGCATTGAGGAAACCGAGA	0.788591849	0.34806834	-0.543685146
ab_99547_AATGTTGCCCATCCTCACAGATTC	0.66194727	0.595325535	-0.455785789
ab_99547_AATGTTGCCGCTGATCACCACTGT	1.14777932	1.237740097	-0.477848111
ab_99547_ACAAGCTACGCTGATCGAGCTGAA	0.957614404	0.92814494	-0.441507938
ab_99547_ACAAGCTAGAACAGGCAAGGTACA	1.196119001	0.722872267	-0.600914998
ab_99547_ACACAGAACAACCACAAGAGTCAA	1.086636217	0.639203363	-0.260384103
ab_99547_ACACGACCAATGTTGCGTACGCAA	0.910553567	0.81043313	-0.474449984
ab_99547_ACACGACCATCATTCCGTCGTAGA	0.876299931	0.50620605	-0.481995289
ab_99547_ACACGACCGCGAGTAACAGATCTG	0.740535324	0.576653078	-0.570432001
ab_99547_ACAGCAGAATCATTCCCGAACTTA	1.122496003	1.234723757	-0.484998492
ab_99547_ACAGCAGACAGCGTTAAGCAGGAA	1.094632479	1.086014394	-0.297491215
ab_99547_ACATTGGCATCATTCCCCGAAGTA	1.37123025	0.689659366	-0.480320438
ab_99547_ACATTGGCCACCTTACGGTGCGAA	0.946028981	0.407006694	-0.581996976
ab_99547_ACATTGGCGCCACATAGTACGCAA	0.772779277	0.500159366	-0.439582093
ab_99547_ACATTGGCTAGGATGAACGTATCA	0.592565941	0.60353933	-0.515641963
ab_99547_ACCACTGTAGCACCTCGGTGCGAA	1.06848493	0.727684291	-0.534476881
ab_99547_ACCACTGTATCCTGTACATACCAA	0.690233825	0.455532045	-0.462948425
ab_99547_ACCACTGTCAAGGAGCCCTAATCC	0.374362408	0.28002339	-0.268764266
ab_99547_ACCACTGTCATACCAACGCTGATC	1.108332833	0.562233558	-0.584151472
ab_99547_ACCTCCAATATCAGCACCTCCTGA	0.753875862	0.326076786	-0.503428141
ab_99547_ACGTATCAAACGTGATGGTGCGAA	0.641919874	0.580208948	-0.53274439
ab_99547_ACGTATCAACACAGAATAGGATGA	0.837085498	1.06711072	-0.549926642
ab_99547_ACGTATCAAGCCATGCGAACAGGC	0.827061094	0.603057593	-0.398744929
ab_99547_ACGTATCAATAGCGACAACGCTTA	0.994730111	1.270167009	-0.534690917
ab_99547_ACTATGCAAGGCTAACGCCAAGAC	1.145772229	0.85447263	-0.516517568
ab_99547_ACTATGCACCGTGAGATTCACGCA	1.042728909	0.649501089	-0.481525716
ab_99547_ACTATGCACCTAATCCACACAGAA	0.603652337	0.607976251	-0.42927029

ab_99547_AGAGTCAAAGTGGTCACAACCACA	0.871702254	0.712453608	-0.359029252
ab_99547_AGAGTCAACGCTGATCGCCAAGAC	1.034101815	0.361747687	-0.509271889
ab_99547_AGAGTCAACGGATTGCCCGAAGTA	0.769069073	0.736380581	-0.437157128
ab_99547_AGAGTCAAGCCAAGACCCGAAGTA	0.797786471	0.803956491	-0.488961221
ab_99547_AGATGTACTGGCTTCATGGCTTCA	0.610269863	0.695429197	-0.496204495
ab_99547_AGCACCTCCCATCCTCACTATGCA	0.78109737	0.702983165	-0.519620129
ab_99547_AGCACCTCTGGTGGTACCGAAGTA	0.852262231	0.806221411	-0.549279649
ab_99547_AGGCTAACAGCAGGAACAAGACTA	0.991042965	1.386288296	-0.500832052
ab_99547_AGTACAAGCCGAAGTAACACGACC	0.700673903	0.56634699	-0.605591433
ab_99547_AGTCACTAAGTGGTCACAGATCTG	0.98676768	1.728823022	-0.448994441
ab_99547_AGTCACTACAGATCTGACAGATTC	0.843770841	0.838388091	-0.514570448
ab_99547_AGTGGTCAACACAGAAAGATCGCA	1.316508388	0.745208341	-0.490902679
ab_99547_AGTGGTCAGAATCTGAAACCGAGA	0.820952022	0.911145216	-0.197482781
ab_99547_ATAGCGACAACGTGATAGTGGTCA	0.753751444	0.459179621	-0.524723628
ab_99547_ATAGCGACAGATGTACCAGATCTG	1.128148898	1.578999725	-0.387422365
ab_99547_ATCATTCCAAGGACACCTGTAGCC	0.842135962	0.692930458	-0.49814295
ab_99547_ATCATTCCACCACTGTCCAGTTCA	0.803597998	0.733329189	-0.408932878
ab_99547_ATCATTCCGCCACATAGCCACATA	0.56481949	0.424880584	-0.544013674
ab_99547_ATCCTGTACCTCTATCATGCCTAA	1.033242622	0.629963492	-0.378825255
ab_99547_ATTGAGGACACTTCGACTAAGGTC	0.745103335	0.953853563	-0.509430101
ab_99547_ATTGAGGATGGTGGTACAATGGAA	0.772907706	0.645965187	-0.435797942
ab_99547_ATTGGCTCCGCATACAACAGCAGA	0.877646476	0.586490983	-0.522036082
ab_99547_CAACCACACACCTTACAGTCACTA	1.298072192	0.363181255	-0.471384821
ab_99547_CAACCACACAGATCTGAATCCGTC	0.523303375	0.473444745	-0.507684246
ab_99547_CAACCACACATACCAAGGAGAACA	0.799077814	1.293776877	-0.519992247
ab_99547_CAACCACATTCACGCAGCCAAGAC	0.829468264	0.507299939	-0.51686479
ab_99547_CAAGGAGCCCTCTATCGCGAGTAA	0.592051218	0.41318795	-0.412995546
ab_99547_CAAGGAGCCTAAGGTCGAATCTGA	0.897582504	0.663692408	-0.460338746
ab_99547_CACCTTACAAACATCGGGAGAACA	0.507729013	0.869534754	-0.470592203
ab_99547_CACCTTACCCATCCTCAAGACGGA	0.878533625	0.346931407	-0.5196794
ab_99547_CACCTTACGCCAAGACCGACACAC	0.656436552	0.444743454	-0.471419241
ab_99547_CACTTCGAAATGTTGCAAGAGATC	0.813426476	0.48118439	-0.508279604

ab_99547_CACTTCGAAGCCATGCGCTCGGTA	0.966593613	0.97452872	-0.352020521
ab_99547_CACTTCGATGGTGGTAGAGTTAGC	0.60119526	0.823385906	-0.435670768
ab_99547_CAGCGTTAACCTCCAAGGTGCGAA	0.631584149	0.849459644	-0.450401529
ab_99547_CAGCGTTAGCTAACGAATTGGCTC	1.032893967	0.421171986	-0.395304775
ab_99547_CATACCAACGACTGGAGCCAAGAC	0.949659812	0.760723407	-0.343069931
ab_99547_CATACCAAGGTGCGAAACGTATCA	1.076702754	1.017442303	-0.519702689
ab_99547_CCATCCTCACCTCCAACTAAGGTC	0.859483014	0.78333227	-0.374093886
ab_99547_CCATCCTCGAACAGGCCCAGTTCA	0.865046089	0.706622778	-0.505377617
ab_99547_CCGAAGTACCTCTATCGATAGACA	1.302975495	0.69901159	-0.450785935
ab_99547_CCGAAGTAGACTAGTATGGTGGTA	0.65726508	0.437714845	-0.401382982
ab_99547_CCGACAACAACTCACCTCTTCACA	0.77102422	0.615833874	-0.328712379
ab_99547_CCGACAACACACAGAAATCCTGTA	1.062955355	0.931949745	-0.471396254
ab_99547_CCGACAACACACAGAACGGATTGC	0.781645225	0.699832747	-0.477498266
ab_99547_CCTAATCCACGTATCAGCTAACGA	0.617718948	0.572331732	-0.519427703
ab_99547_CCTAATCCGATGAATCAGATGTAC	0.87509147	0.49963514	-0.550142922
ab_99547_CCTCCTGAAGCAGGAAAAGGTACA	0.792834928	0.536471182	-0.494616544
ab_99547_CCTCCTGACGACACACCATACCAA	0.857348854	0.603148582	-0.428709925
ab_99547_CCTCTATCATTGGCTCAGATCGCA	0.886523735	0.503825707	-0.411090664
ab_99547_CCTCTATCCCGACAACGCTAACGA	1.342190566	0.733891041	-0.52884742
ab_99547_CCTCTATCGATAGACACAATGGAA	0.907826138	0.853504073	-0.439974177
ab_99547_CGAACTTAAACGTGATATCATTCC	0.963390731	1.0032816	-0.560234101
ab_99547_CGAACTTAAGCCATGCCAAGGAGC	0.365782991	0.619438351	-0.413156036
ab_99547_CGAACTTACACCTTACCAAGACTA	0.734719422	0.737891042	-0.342210986
ab_99547_CGAACTTAGAGCTGAACCGACAAC	0.856099213	0.853371145	-0.58680572
ab_99547_CGACACACATCCTGTAAGAGTCAA	1.305392006	1.468372575	-0.308318654
ab_99547_CGACACACCAACCACAAACAACCA	0.827721135	0.547971841	-0.500900193
ab_99547_CGACACACCGAACTTAGCTCGGTA	1.080305369	0.291403298	-0.522397756
ab_99547_CGACTGGAATTGAGGAAAGGACAC	0.558397112	0.640384403	-0.556828593
ab_99547_CGCATACACAATGGAACCATCCTC	0.882173695	0.932702416	-0.510421244
ab_99547_CGCTGATCACACGACCCAATGGAA	0.571675947	0.87671328	-0.339092861
ab_99547_CGCTGATCCCTCTATCTGGCTTCA	0.886290263	0.662200924	-0.347681223
ab_99547_CTCAATGAACCTCCAACACTTCGA	0.812052136	0.594849948	-0.510783564

ab_99547_CTGAGCCAAAGAGATCCCGTGAGA	0.876414959	0.577311011	-0.321806149
ab_99547_CTGAGCCAAGTACAAGAACTCACC	0.929180677	0.648588197	-0.488957225
ab_99547_CTGAGCCACGAACTTATGGAACAA	0.762170331	0.992706721	-0.57065595
ab_99547_CTGGCATACGAACTTAACTATGCA	0.90579113	0.879284111	-0.509637464
ab_99547_CTGGCATAGGTGCGAAAAGAGATC	0.918407445	0.825211326	-0.560510845
ab_99547_CTGTAGCCAGATCGCACCTCCTGA	0.791021531	0.931787135	-0.367184165
ab_99547_GAATCTGACTGAGCCACGCATACA	0.514482048	0.705877897	-0.529231088
ab_99547_GAATCTGAGAATCTGAACAAGCTA	0.854540885	0.445667264	-0.489212628
ab_99547_GAATCTGATAGGATGAAGCACCTC	0.793152464	0.786243429	-0.324393945
ab_99547_GACTAGTACTAAGGTCACTATGCA	0.786612409	0.665676673	-0.433886065
ab_99547_GACTAGTACTCAATGAGAACAGGC	0.988468144	1.059090986	-0.366643606
ab_99547_GAGCTGAAGATGAATCACATTGGC	0.803251185	1.05082241	-0.556095685
ab_99547_GATAGACAAACGCTTACCTCCTGA	0.507294608	0.648000782	-0.423747682
ab_99547_GATAGACACGAACTTACACCTTAC	0.67742789	0.50765565	-0.396619749
ab_99547_GATAGACACTGGCATAAGTACAAG	0.625675421	0.710033258	-0.451391768
ab_99547_GATGAATCCTGAGCCAAAGAGATC	0.696212494	0.348104244	-0.505477526
ab_99547_GATGAATCGTCTGTCATATCAGCA	1.594787531	0.637934993	-0.505843963
ab_99547_GCCAAGACCCGACAACAAGGACAC	0.722604194	0.432759472	-0.302400083
ab_99547_GCCAAGACGAACAGGCCATACCAA	0.673559593	0.805489	-0.569161012
ab_99547_GCCAAGACTTCACGCAAACGCTTA	0.872623793	0.636884054	-0.489627819
ab_99547_GCCACATAGATGAATCACCTCCAA	0.94953785	0.788288052	-0.313127725
ab_99547_GCCACATAGTGTTCTAAATCCGTC	0.779998459	0.682748307	-0.565851861
ab_99547_GCGAGTAATGGTGGTAAACCGAGA	0.904004587	0.828201444	-0.548393314
ab_99547_GCTAACGAAAGAGATCCACCTTAC	0.681333602	0.62469949	-0.596867507
ab_99547_GCTAACGAAGATGTACAAGGTACA	0.939770055	0.641869344	-0.549562911
ab_99547_GCTCGGTAGGAGAACACTGAGCCA	0.546276745	0.91187663	-0.551210758
ab_99547_GCTCGGTATGAAGAGACACCTTAC	0.953350442	0.498122908	-0.559596791
ab_99547_GGAGAACAAGATCGCAAAGGACAC	0.700360407	0.469304713	-0.57909907
ab_99547_GGAGAACAAGATGTACCAAGGAGC	0.702754948	0.177278006	-0.573329227
ab_99547_GGAGAACAGTACGCAAACAGCAGA	0.860920065	0.668031503	-0.51050684
ab_99547_GGTGCGAAAGCAGGAACTGGCATA	0.900489052	0.664983938	-0.415585667
ab_99547_GTACGCAAAAGACGGAGTGTTCTA	1.132404629	1.192297826	-0.235277917

ab_99547_GTACGCAAACAGATTCGCCAAGAC	0.904188251	0.773816717	-0.531104645
ab_99547_GTACGCAACTGTAGCCACGCTCGA	0.88883851	0.877236411	-0.519245032
ab_99547_GTCGTAGACGACACACAATCCGTC	0.580108781	0.784100768	-0.466055861
ab_99547_GTCTGTCAAACTCACCCCTCTATC	0.772663247	0.598286215	-0.476552371
ab_99547_GTCTGTCAAGCCATGCCATCAAGT	0.994864448	0.589841633	-0.385876104
ab_99547_GTCTGTCACAACCACACAATGGAA	1.007382214	1.108039814	-0.4213213
ab_99547_GTCTGTCAGCCACATAAAGACGGA	0.948238921	0.724117517	-0.438330306
ab_99547_GTGTTCTAACACGACCCCGTGAGA	0.737457507	0.855016792	-0.429639172
ab_99547_GTGTTCTAAGTCACTAAAGGTACA	1.283724114	0.713710204	-0.481327875
ab_99547_TAGGATGAGATAGACACCGTGAGA	0.585093975	0.719416154	-0.417470988
ab_99547_TATCAGCAAGATGTACTAGGATGA	0.911527718	0.457256422	-0.523553339
ab_99547_TATCAGCAAGTGGTCAGACAGTGC	0.639313579	0.352220325	-0.477806299
ab_99547_TATCAGCACGCTGATCCACTTCGA	0.817017639	0.356541616	-0.581983682
ab_99547_TATCAGCATTCACGCACCTCCTGA	0.766364709	0.69150364	-0.585367081
ab_99547_TCCGTCTAACGTATCAATCCTGTA	0.666181945	0.561797012	-0.400414391
ab_99547_TCCGTCTAGAGCTGAAATCATTCC	0.753503952	0.699083779	-0.571563396
ab_99547_TCTTCACACTGGCATACGACACAC	0.923137499	0.886551169	-0.517716665
ab_99547_TCTTCACATATCAGCATTCACGCA	0.592223459	0.588679157	-0.577736227
ab_99547_TGAAGAGAAACGCTTAATAGCGAC	1.055758765	0.647031204	-0.481023205
ab_99547_TGGAACAACCTCTATCACATTGGC	0.740001147	0.448362182	-0.403412092
ab_99547_TGGCTTCAAACGTGATTGAAGAGA	0.88179481	0.971668623	-0.472369184
ab_99547_TGGTGGTAACCACTGTTATCAGCA	1.114224636	1.083737414	-0.574677029
ab_99547_TTCACGCACTAAGGTCACAAGCTA	0.761929289	0.710557599	-0.42630718
ab_99547_AAACATCGACACGACCACATTGGC	0.853205766	0.311674815	-0.483153825
ab_99547_AACCGAGAGTGTTCTAAGGCTAAC	0.55627581	0.2109702	-0.355658662
ab_99547_AACTCACCATGCCTAACCTCCTGA	0.584034727	0.124761945	-0.551417003
ab_99547_ACAGATTCAGTGGTCAGCCACATA	0.600734901	0.468482295	-0.563185211
ab_99547_ACAGATTCATCATTCCGAATCTGA	0.887778681	0.159915487	-0.605318997
ab_99547_AGCACCTCTCCGTCTAAGTGGTCA	0.584045108	0.52600228	-0.478053211
ab_99547_CAAGACTAGAGTTAGCATTGGCTC	0.713185638	0.19562743	-0.514733463
ab_99547_CAAGGAGCATTGGCTCGCTAACGA	0.608587137	0.219792043	-0.527328883
ab_99547_CCGTGAGAGGAGAACACGACTGGA	0.583326817	0.237567715	-0.36712188

ab_99547_GACAGTGCCACCTTACATTGAGGA	0.818116958	0.195569886	-0.45424807
ab_99547_GCGAGTAACATACCAATGAAGAGA	0.626614552	0.418248781	-0.432974182
ab_99547_GCTAACGACAACCACAAACTCACC	0.826535529	0.40289403	-0.402500395
ab_99547_GTACGCAAGACAGTGCAGTGGTCA	0.696234114	0.075390119	-0.50506492
ab_99547_AACCGAGAAACTCACCCGCATACA	0.893638115	0.099711985	-0.481228225
ab_99547_AACCGAGAAAGAGATCCTGAGCCA	0.617099099	0.16176279	-0.434883962
ab_99547_AACCGAGATGGTGGTACATACCAA	0.935400615	0.135598773	-0.483620844
ab_99547_AACGTGATAAGACGGAACGCTCGA	0.821627272	-0.023813321	-0.513195905
ab_99547_AACTCACCAGAGTCAACAATGGAA	0.655018791	0.249794593	-0.352545891
ab_99547_AATCCGTCAGCCATGCAACGTGAT	0.583979706	0.083747616	-0.560420754
ab_99547_AATGTTGCACAGATTCAAGAGATC	0.815113868	0.199089911	-0.365884925
ab_99547_AATGTTGCCGACACACCAACCACA	0.665941524	0.111303016	-0.422822376
ab_99547_AATGTTGCCTAAGGTCCAATGGAA	0.554301803	0.13353562	-0.434896956
ab_99547_AATGTTGCGCTCGGTAAACTCACC	0.66207578	0.079610671	-0.438253182
ab_99547_ACAAGCTAGCGAGTAAAACAACCA	0.636969182	0.203976001	-0.240631009
ab_99547_ACACAGAACCGTGAGACGCATACA	0.850261542	0.355143744	-0.397747241
ab_99547_ACACAGAAGTCTGTCAAATGTTGC	0.779472481	0.164899748	-0.450977227
ab_99547_ACACGACCCAGCGTTACAAGGAGC	0.832213661	0.319925199	-0.59557132
ab_99547_ACACGACCCCATCCTCCCTCCTGA	0.625447497	0.062830194	-0.631219038
ab_99547_ACACGACCTTCACGCAATGCCTAA	0.713266669	0.067564673	-0.440993487
ab_99547_ACAGCAGACCTCCTGACCTCCTGA	0.630011715	0.177145286	-0.446124506
ab_99547_ACAGCAGAGTGTTCTAAGTGGTCA	0.709540316	0.010840821	-0.556426896
ab_99547_ACGCTCGATCTTCACACCGAAGTA	0.554060444	0.231975013	-0.453649354
ab_99547_ACGTATCAACGTATCAATAGCGAC	0.820196332	0.342051825	-0.268596263
ab_99547_ACGTATCAACGTATCATCCGTCTA	0.78422912	0.090225336	-0.414438861
ab_99547_ACTATGCAGACAGTGCTGGCTTCA	0.645312186	0.375703772	-0.31716254
ab_99547_ACTATGCAGGAGAACATTCACGCA	0.447471788	0.122364397	-0.4432286
ab_99547_AGATCGCAAACTCACCCCAGTTCA	0.496769589	0.142272847	-0.524154393
ab_99547_AGATGTACCCAGTTCAAATCCGTC	0.618101306	0.116315637	-0.4298034
ab_99547_AGCACCTCCTGTAGCCACAGCAGA	0.595658776	0.306983996	-0.425543432
ab_99547_AGCACCTCGTCTGTCAAGCAGGAA	0.791224044	0.161726923	-0.354945764
ab_99547_AGGCTAACTATCAGCATCTTCACA	0.886320265	0.059286997	-0.404955506

ab_99547_AGTGGTCAAGATCGCAGAGCTGAA	0.436626107	0.057863877	-0.457230665
ab_99547_AGTGGTCACCATCCTCCATCAAGT	0.967284598	0.175620022	-0.424619433
ab_99547_ATAGCGACAGATCGCATATCAGCA	0.70157231	0.235097067	-0.445693918
ab_99547_ATTGAGGAGCCACATACCAGTTCA	0.583374061	0.161237752	-0.384161272
ab_99547_CAACCACAGCCACATACAGATCTG	0.519827215	0.167931765	-0.377713132
ab_99547_CAAGGAGCAAACATCGAGTCACTA	0.633372149	0.048353641	-0.502992079
ab_99547_CAAGGAGCATGCCTAAAACAACCA	1.036499373	0.274966653	-0.176449384
ab_99547_CAATGGAAAAGGTACACTCAATGA	0.852892957	0.163447784	-0.229378488
ab_99547_CAATGGAAAGCAGGAACCGACAAC	0.846231424	0.005949138	-0.430124644
ab_99547_CAATGGAACGAACTTAAAGGTACA	0.804078305	0.006485574	-0.383118613
ab_99547_CACTTCGAAAGACGGACATACCAA	0.708931122	0.168097296	-0.430714172
ab_99547_CACTTCGAACTATGCACCGTGAGA	0.852943363	0.234011584	-0.321530094
ab_99547_CACTTCGACGAACTTAATGCCTAA	0.61933988	0.118192212	-0.451758511
ab_99547_CAGATCTGATAGCGACAGATCGCA	0.771907934	0.070326167	-0.477497584
ab_99547_CAGCGTTAGAACAGGCAAGGTACA	0.559517544	0.114266114	-0.479044601
ab_99547_CAGCGTTATGAAGAGATGGCTTCA	0.68360582	0.135652193	-0.437091511
ab_99547_CATACCAACAAGACTACATACCAA	0.757429476	0.155025568	-0.456013459
ab_99547_CATACCAACACTTCGATGGTGGTA	0.64888837	0.122301859	-0.363117135
ab_99547_CATACCAAGCTAACGAATTGAGGA	0.686804339	0.237867991	-0.238897694
ab_99547_CATACCAATGGAACAACGCATACA	0.911831643	0.172817161	-0.415894168
ab_99547_CCAGTTCAAGGCTAACACAGCAGA	0.666304688	0.133423735	-0.436795681
ab_99547_CCATCCTCCATCAAGTATAGCGAC	0.596539841	0.079785147	-0.446001618
ab_99547_CCATCCTCTTCACGCATCTTCACA	0.651227566	0.157482613	-0.476504932
ab_99547_CCGACAACCCTAATCCATAGCGAC	0.744315242	0.133360222	-0.512402925
ab_99547_CCGACAACTAGGATGAAGTCACTA	1.101350005	0.126358372	-0.439603946
ab_99547_CCGTGAGAATCCTGTAAACCGAGA	0.540900718	0.044379152	-0.422031447
ab_99547_CCTCCTGAACAGCAGACAACCACA	0.61002684	0.132061076	-0.481227198
ab_99547_CCTCCTGAGCCAAGACCACCTTAC	0.55643985	0.150244545	-0.377688484
ab_99547_CGACACACTCTTCACACCGAAGTA	0.742020968	0.124658078	-0.492556066
ab_99547_CGACTGGACAGATCTGATAGCGAC	0.860763887	0.157203494	-0.363981734
ab_99547_CGCTGATCGAGTTAGCGTACGCAA	0.65370929	0.123802663	-0.406853009
ab_99547_CGGATTGCCCTCCTGAATTGGCTC	0.596724813	0.099157601	-0.454812167

ab_99547_CGGATTGCCGAACTTAGAATCTGA	0.64665377	-0.002154276	-0.446161789
ab_99547_CTAAGGTCACGTATCATATCAGCA	0.646158193	0.02971468	-0.515067502
ab_99547_CTAAGGTCCAAGGAGCATCCTGTA	0.88423451	0.040642244	-0.433332959
ab_99547_CTAAGGTCCCGAAGTAAGAGTCAA	0.661632855	0.179482757	-0.327987098
ab_99547_CTCAATGACTAAGGTCATTGAGGA	0.561215724	0.215969471	-0.383590312
ab_99547_CTCAATGATGGCTTCACTGGCATA	0.534203254	0.331340759	-0.373258574
ab_99547_CTGAGCCAACAGATTCGAGCTGAA	1.162739308	0.178031608	-0.486951307
ab_99547_CTGAGCCAAGTACAAGTGAAGAGA	0.869635427	0.100253687	-0.440773745
ab_99547_CTGAGCCACAATGGAACTGTAGCC	0.594748981	0.307765729	-0.356977837
ab_99547_CTGAGCCATCTTCACAAACGTGAT	0.488271048	0.176069832	-0.424742026
ab_99547_CTGGCATAAACGCTTAGGAGAACA	0.722951937	0.158057612	-0.507109656
ab_99547_CTGGCATAACGTATCAAATCCGTC	1.036260849	0.196897587	-0.563098654
ab_99547_CTGTAGCCAAGGACACACATTGGC	0.701976246	0.161818725	-0.399860874
ab_99547_CTGTAGCCACAAGCTAAACCGAGA	0.886856395	0.202894346	-0.329701478
ab_99547_CTGTAGCCTAGGATGACTGGCATA	0.633336883	0.358908661	-0.336892349
ab_99547_GACAGTGCACTATGCAGTCGTAGA	0.591924661	0.131118546	-0.510051982
ab_99547_GACAGTGCCAAGACTAGAATCTGA	0.445351504	-0.05731642	-0.465196576
ab_99547_GACAGTGCGCTCGGTAAGTGGTCA	0.874274365	0.348005904	-0.373268121
ab_99547_GACTAGTAATCATTCCGTGTTCTA	0.357242684	0.004402944	-0.454384654
ab_99547_GACTAGTATTCACGCACGCATACA	0.733296112	-0.064542032	-0.5737432
ab_99547_GAGCTGAAAGAGTCAACCGAAGTA	0.801033526	0.039278562	-0.532092029
ab_99547_GATAGACAACTATGCAAGATCGCA	0.733103334	0.11989697	-0.360193761
ab_99547_GATAGACACTGAGCCAAACGCTTA	0.65203978	0.063414688	-0.48229332
ab_99547_GATGAATCACACGACCCGACTGGA	0.863308927	0.148888629	-0.584188247
ab_99547_GCCAAGACATAGCGACACGTATCA	0.462095149	0.121664672	-0.266613926
ab_99547_GCCACATACATACCAAACACGACC	0.634245111	0.121571331	-0.457262078
ab_99547_GCCACATACCGAAGTAGCCAAGAC	0.651679616	0.237391435	-0.39263186
ab_99547_GCGAGTAAATTGGCTCCGCTGATC	0.988059026	0.192416802	-0.445606158
ab_99547_GCTAACGAATTGGCTCCGACACAC	0.463013005	0.053018658	-0.537747474
ab_99547_GCTAACGAGAGCTGAACGACTGGA	0.863906599	0.175042048	-0.38560461
ab_99547_GCTAACGATGGTGGTACGCTGATC	0.426445878	0.101633926	-0.396471616
ab_99547_GGAGAACAATCATTCCCGGATTGC	0.53406346	0.030567488	-0.603697667

ab_99547_GTACGCAAGAACAGGCGGTGCGAA	0.745001981	0.003582061	-0.430096389
ab_99547_GTACGCAAGACAGTGCAACCGAGA	0.626746338	0.201355705	-0.470775886
ab_99547_GTCGTAGAGCCAAGACCGCTGATC	0.613446034	0.057551225	-0.520549306
ab_99547_GTCTGTCAACACAGAAAATGTTGC	0.660885492	0.260599994	-0.403743195
ab_99547_GTCTGTCAGCTCGGTAAACGTGAT	0.364609891	-0.082694483	-0.451166894
ab_99547_GTGTTCTACTCAATGAGCTAACGA	0.307812606	0.212461044	-0.402886951
ab_99547_TATCAGCATATCAGCAAACCGAGA	0.657349245	0.115474235	-0.339515138
ab_99547_TCTTCACAACGTATCAACACAGAA	0.925165385	0.159065642	-0.45322826
ab_99547_TCTTCACAAGGCTAACAAGAGATC	0.626777513	0.128531678	-0.456593784
ab_99547_TGAAGAGAGATAGACAGTCGTAGA	0.625273712	0.002077862	-0.407593463
ab_99547_TGGAACAAAACTCACCACAGCAGA	0.612634063	0.193654051	-0.435210757
ab_99547_TGGAACAACTGGCATAAAGAGATC	0.705919514	0.255290346	-0.511553089
ab_99547_TGGAACAAGACAGTGCCCTCTATC	1.043783135	0.237222906	-0.321831243
ab_99547_TGGCTTCAAACGTGATGAATCTGA	0.741107011	0.144789865	-0.557339898
ab_99547_TGGTGGTAAGCCATGCAACTCACC	0.578392352	0.175432204	-0.517252699
ab_99547_TGGTGGTAATCATTCCCACCTTAC	0.922207873	0.32512888	-0.393106108
ab_99547_TTCACGCAGCTCGGTACCGTGAGA	0.621298953	-0.033950819	-0.388800711
ab_99547_AAGGACACCCATCCTCAACGCTTA	1.36098018	0.666750667	-0.431739349
ab_99547_CCGAAGTACTAAGGTCGCGAGTAA	0.34724937	0.468042087	-0.452915427
ab_99547_TGGCTTCAAAGAGATCACTATGCA	0.786588119	0.569989265	-0.196615036
A_98763_AACTCACCACAAGCTAAGATCGCA	0.264221812	-0.001783836	0.266166608
A_98763_AAGAGATCATGCCTAAACAAGCTA	-0.577832665	0.069317369	0.254639731
A_98763_ACAAGCTAACGCTCGAGACTAGTA	0.364550796	0.163573599	0.140128374
A_98763_ACAAGCTACATACCAAGATAGACA	0.123894729	0.090944222	1.210771229
A_98763_ACACGACCGCCACATACACTTCGA	0.479099436	0.431715311	0.740483054
A_98763_ACCACTGTCGACACACAAGAGATC	0.300130761	-0.012545482	-0.457815813
A_98763_AGATCGCAACAGCAGAGAACAGGC	0.416382856	0.65508801	0.941373163
A_98763_AGATCGCAGGAGAACAAGATCGCA	0.240489706	0.177942147	0.331629365
A_98763_AGCACCTCTTCACGCAACGCTCGA	0.699797415	0.617361754	1.075155157
A_98763_AGTACAAGACGTATCACCGTGAGA	0.295967	0.18554886	0.240110608
A_98763_AGTGGTCACGAACTTAGACAGTGC	0.329028056	0.303276728	0.301682526
A_98763_ATCATTCCATCCTGTAAGATCGCA	0.400720096	0.057043489	-0.144876499

A_98763_ATCATTCCTAGGATGACTCAATGA	0.327854159	0.061092842	0.425211374
A_98763_ATTGAGGAGCCACATATGAAGAGA	0.383192142	0.319059336	0.378498728
A_98763_CAACCACATGGAACAAATTGAGGA	0.606201933	1.126942484	0.009959948
A_98763_CAAGACTAGACTAGTAGACAGTGC	0.048636811	0.129041432	0.363606708
A_98763_CAAGGAGCAGATCGCACAGCGTTA	0.25866487	0.396666999	0.432373054
A_98763_CAAGGAGCAGCCATGCCCGAAGTA	0.403627796	0.13252706	0.310142338
A_98763_CAAGGAGCCGGATTGCCACTTCGA	0.378480226	-0.026558832	0.364787629
A_98763_CACCTTACAGAGTCAATTCACGCA	0.211032035	0.140603793	0.553261369
A_98763_CCGAAGTATCCGTCTACCTAATCC	0.322479204	0.037472075	-0.489841538
A_98763_CGGATTGCGGAGAACAGAATCTGA	0.192939091	0.017089243	0.611683608
A_98763_CTCAATGATGGTGGTAGCTAACGA	0.238088479	0.576209608	1.231016398
A_98763_GAACAGGCCGAACTTAATTGAGGA	0.374574015	0.307566784	0.450762469
A_98763_GAATCTGAAGATGTACAGCCATGC	0.025715544	0.210035329	0.910049573
A_98763_GACAGTGCTGGAACAAAACTCACC	0.390059094	0.146284443	0.054277699
A_98763_GAGCTGAAAGGCTAACTGGAACAA	-0.002670558	0.077932066	0.199665186
A_98763_GAGCTGAACAGATCTGCTGGCATA	0.43244946	0.453523467	0.832341621
A_98763_GAGCTGAAGCTAACGACAGATCTG	0.341687239	0.204347719	0.003763232
A_98763_GAGTTAGCAACTCACCTATCAGCA	0.573806592	0.162352802	0.172700671
A_98763_GATAGACAACAGCAGACAACCACA	0.326769418	0.273589635	0.468859918
A_98763_GATAGACACCAGTTCACCGACAAC	0.464039071	0.234805119	-0.189695187
A_98763_GATGAATCCGAACTTAGACAGTGC	0.11490261	0.132831093	-0.009759275
A_98763_GCCACATATTCACGCAATTGAGGA	0.235977745	-0.075271079	-0.29456596
A_98763_GCTAACGACACCTTACATAGCGAC	0.239311986	0.134686042	0.639640934
A_98763_GCTCGGTAAGTGGTCAAATGTTGC	0.582352057	0.140417607	-0.297547366
A_98763_GCTCGGTAAGTGGTCATTCACGCA	0.261821065	0.429457129	0.724141383
A_98763_GTACGCAAACAGATTCGTCTGTCA	0.173502739	0.409122576	1.098107077
A_98763_GTACGCAACTGGCATAACGTATCA	0.375166247	0.271147219	1.321480093
A_98763_GTCGTAGAAGATGTACCAACCACA	0.169655419	0.257269367	0.25763386
A_98763_GTCTGTCACGACTGGACGGATTGC	0.504230404	-0.004596277	0.148594723
A_98763_GTGTTCTAACTATGCACGAACTTA	0.42781345	0.070495361	-0.290352767
A_98763_TGAAGAGACTAAGGTCTCTTCACA	0.205914537	0.080021073	0.399229207
A_98763_TGGCTTCACATCAAGTTGGAACAA	0.360305722	0.153936393	0.393511613

A_98763_TGGCTTCACCGTGAGAGAGTTAGC	0.321799406	0.129315355	0.665372624
A_98763_TGGTGGTAACACAGAATGGCTTCA	0.010830895	0.002237065	-0.018436833
A_98763_AAACATCGCAGATCTGAAGAGATC	0.610512256	0.033899825	-0.08043602
A_98763_AAACATCGCTGGCATAAAGGTACA	0.269347136	0.100033826	-0.065291125
A_98763_AACAACCAAACCGAGAGGTGCGAA	0.476823436	0.081411386	-0.134383804
A_98763_AACGCTTAACACAGAATGGTGGTA	0.309021047	0.329473771	0.56665606
A_98763_AAGACGGAAGTCACTACCGAAGTA	0.345155925	0.004002637	-0.350817749
A_98763_AAGACGGACATCAAGTAGTCACTA	0.262126164	0.345926756	0.402340746
A_98763_ACAAGCTACGAACTTAAAGAGATC	0.322250479	0.220848893	0.181368278
A_98763_ACACGACCACAGCAGAACAGATTC	0.329123544	0.318445696	0.834786289
A_98763_ACACGACCTGGCTTCAGATAGACA	0.401080136	0.741970887	0.56926802
A_98763_ACAGCAGAACAAGCTACCGACAAC	0.459013065	0.614168485	-0.007272051
A_98763_ACATTGGCGCTAACGACTAAGGTC	0.496306398	0.033425606	0.496136632
A_98763_AGAGTCAAAGTCACTAAACCGAGA	0.260491719	0.347714175	0.576160018
A_98763_AGAGTCAATGGCTTCATGGTGGTA	0.310712054	0.026611886	-0.423158518
A_98763_AGATCGCAAGTGGTCACAGATCTG	0.718554084	0.299342829	-0.321299485
A_98763_AGATCGCACGAACTTAGACAGTGC	0.93244885	0.615628108	1.206908814
A_98763_AGATGTACACAAGCTAAACCGAGA	0.113863956	0.186321617	0.529368479
A_98763_AGATGTACAGCAGGAAATCATTCC	0.61292427	0.29773366	-0.148567142
A_98763_AGCCATGCAACCGAGAAGCAGGAA	0.373937689	0.070708877	0.439990692
A_98763_AGCCATGCAACGTGATGACAGTGC	0.435495449	0.16205428	-0.195109601
A_98763_AGTCACTAACGTATCAGTCTGTCA	0.639032151	0.330411925	0.145874506
A_98763_AGTGGTCAAGATGTACTGGCTTCA	0.359281611	0.198392638	0.202293648
A_98763_AGTGGTCATGAAGAGAAACGCTTA	0.132511615	0.126542908	1.047978239
A_98763_ATAGCGACTGAAGAGAACGTATCA	0.179510032	0.102223929	-0.219949339
A_98763_ATCATTCCATAGCGACAGATCGCA	0.509930055	0.173878264	-0.082271366
A_98763_ATCATTCCGAATCTGATTCACGCA	0.265294084	0.685925701	0.55655193
A_98763_ATGCCTAACAACCACACGACTGGA	0.288109336	0.063731192	-0.107533007
A_98763_ATTGAGGAGAGTTAGCCCTCTATC	0.181620394	0.134584947	0.301438635
A_98763_CAAGACTATCCGTCTAAACTCACC	0.370793807	0.605057148	1.016987773
A_98763_CAATGGAAACCTCCAAAGGCTAAC	0.345645547	0.011581518	0.099241202
A_98763_CACTTCGAGTCGTAGAATTGAGGA	0.179219825	-0.121769988	-0.165121373

A_98763_CAGCGTTAAACGCTTAATCCTGTA	0.049667307	-0.197847113	0.536066201
A_98763_CCAGTTCAAAGACGGAAGCAGGAA	0.138915459	-0.136327339	0.052991158
A_98763_CCAGTTCAACTATGCAGTACGCAA	0.447738751	0.3906664	0.965737257
A_98763_CCGAAGTACCTCTATCGAGTTAGC	0.424927711	0.291445604	0.809273821
A_98763_CCGACAACGATGAATCACCTCCAA	0.326446785	-0.064270599	-0.1577764
A_98763_CCGTGAGACCTAATCCACCTCCAA	0.047891676	0.029644702	-0.050090345
A_98763_CCGTGAGAGATAGACAACCACTGT	0.347083936	0.375711787	0.518602538
A_98763_CCGTGAGATCCGTCTACTGGCATA	0.193892508	0.094224509	0.348328293
A_98763_CCTAATCCACTATGCAAGGCTAAC	0.096815761	-0.034398813	0.057802567
A_98763_CCTCCTGATATCAGCAGCTCGGTA	0.049229053	0.077558504	0.514618975
A_98763_CGACACACTCTTCACAACCTCCAA	0.366098592	-0.098614973	-0.529264489
A_98763_CGCATACACCTCCTGACCTAATCC	0.215407079	0.056640931	0.463827741
A_98763_CGCATACAGGAGAACAAAGAGATC	0.342216307	0.09064015	0.116242999
A_98763_CTAAGGTCAACGTGATAGTGGTCA	0.161671011	0.095739115	0.295216439
A_98763_CTAAGGTCACACGACCCGGATTGC	0.535868387	0.221156978	-0.137306828
A_98763_CTCAATGACACCTTACAGTCACTA	0.808886294	1.029197707	0.359412857
A_98763_CTCAATGAGGTGCGAAGACTAGTA	0.759900716	0.612763568	0.223709029
A_98763_CTGAGCCAAGCCATGCGAATCTGA	0.389168213	0.02802046	0.15901486
A_98763_GAATCTGACTGGCATACTCAATGA	0.699928216	0.49470662	0.515795955
A_98763_GAATCTGAGAGTTAGCAACCGAGA	0.311681556	0.03759515	0.518967227
A_98763_GAGCTGAAATAGCGACACAAGCTA	0.248711432	0.115300013	0.10810824
A_98763_GCCACATAACAGATTCGATAGACA	0.477699617	0.466508435	0.477348157
A_98763_GCCACATACAACCACAGCTCGGTA	0.293255405	0.033676976	0.039063588
A_98763_GCTCGGTAAACGCTTAAGCAGGAA	0.665237472	0.2074541	0.474115643
A_98763_GCTCGGTAACGTATCAGTCTGTCA	0.575629392	0.602779888	0.427638303
A_98763_GGAGAACAACATTGGCCTGGCATA	0.08222262	0.011024849	-0.1305232
A_98763_GTACGCAAAGATGTACAGTGGTCA	0.07824676	0.01782797	-0.068368113
A_98763_GTCGTAGAAAGACGGAATAGCGAC	0.638804642	0.368338533	0.357928538
A_98763_GTCGTAGAACGCTCGACCGAAGTA	0.641436486	0.646333283	0.272237198
A_98763_GTCTGTCAATAGCGACAGATCGCA	0.797403694	1.215468167	1.286744693
A_98763_GTGTTCTAATCATTCCAGCAGGAA	0.358618825	0.407237615	0.616985425
A_98763_GTGTTCTAGCTAACGAAGCACCTC	0.59172159	0.118288069	0.004755405

A_98763_TAGGATGACTGGCATACTGAGCCA	0.096211131	-0.095063864	-0.381170401
A_98763_TCCGTCTAAGTCACTAGTACGCAA	0.780799901	0.414116703	0.605810799
A_98763_TCCGTCTACCTAATCCACACGACC	0.284411838	-0.081339011	-0.311175807
A_98763_TCCGTCTACGAACTTAAATGTTGC	0.597654504	0.432165319	0.420535579
A_98763_TGAAGAGAGCGAGTAACCTCTATC	0.508334058	0.273040259	-0.294656546
A_98763_TGGAACAACCATCCTCACCTCCAA	0.516277127	0.021207476	0.229141894
A_98763_TGGAACAAGTCTGTCAACAGCAGA	0.236081841	0.007703634	-0.165355808
A_98763_TGGCTTCAAAGGTACAACAAGCTA	0.502997587	0.134101451	0.396887193
A_98763_TGGTGGTACACTTCGAACAGATTC	0.458203137	0.137688674	0.326519733
A_98763_TTCACGCAGGAGAACAAGGCTAAC	0.411727434	0.097115182	-0.040815865
A_98763_TTCACGCAGTACGCAATGGTGGTA	0.529585768	0.304400737	0.11408563
A_98763_AAGAGATCACAGCAGACCGACAAC	0.530950323	0.528838207	0.699856095
A_98763_TCTTCACATGGCTTCAACCTCCAA	0.439680738	0.345485686	0.552843461
A_98763_TGGCTTCAGACTAGTACGCTGATC	0.695057671	0.947910237	0.813233847
A_98763_AACAACCATCTTCACATGAAGAGA	0.387658317	0.717460064	1.274904237
A_98763_AGAGTCAAGCGAGTAAATCATTCC	0.354758486	0.490829268	0.930912333
A_98763_ATGCCTAAACATTGGCATCCTGTA	0.049904146	0.274709326	1.031491116
A_98763_CTAAGGTCCCTCCTGAACAGCAGA	0.436912095	0.534223299	1.137943211
A_98763_AATCCGTCTTCACGCATCCGTCTA	0.671080601	0.6060467	-0.057951826
A_98763_GCCAAGACGCGAGTAACACTTCGA	0.604202835	0.300041457	-0.083182791
B_98618_ATCATTCCCTGAGCCAACAGATTC	0.632789383	0.169056824	-0.006432792
B_98618_CCTCTATCCGACTGGACAACCACA	0.626831697	0.147416513	-0.193809273
B_98618_TGGCTTCAGTGTTCTAACAAGCTA	0.322142363	0.045513313	-0.495293443
B_98618_AACTCACCACACGACCGCGAGTAA	0.145240396	0.211018196	-0.130733109
B_98618_ACAGCAGAACGTATCACCGTGAGA	0.129279578	0.551194864	-0.253836389
B_98618_ACCACTGTATTGGCTCCCATCCTC	0.456554488	0.552055367	-0.298328912
B_98618_ACCACTGTGCCACATAGTGTTCTA	0.242419137	0.252552414	-0.414639754
B_98618_AGAGTCAATCTTCACACATACCAA	0.070919899	0.155230087	-0.409734177
B_98618_ATGCCTAAAACGCTTAAAGGTACA	0.989000355	0.550446667	-0.381346968
B_98618_CCGAAGTAGCTAACGAGAATCTGA	0.569875238	0.346052615	-0.204554449
B_98618_GCTAACGACCATCCTCCAAGACTA	0.211902588	0.146408732	-0.251392057
B_98618_AAACATCGCCTCTATCCTGAGCCA	-0.164985135	0.038184398	-0.325052983

B_98618_AACAACCACAAGACTACATACCAA	-0.172637933	-0.039864942	-0.393582229
B_98618_AACAACCATGAAGAGAAGATGTAC	-0.051012608	-0.133529024	-0.532987772
B_98618_AACCGAGAAGTACAAGGCTAACGA	0.144900275	0.082204864	-0.024343409
B_98618_AACGCTTACCATCCTCCTAAGGTC	-0.033998747	-0.05462373	-0.542924856
B_98618_AACGCTTAGAGCTGAAAAACATCG	0.081975114	-0.068517003	-0.441583876
B_98618_AACGCTTAGCTCGGTAGCTCGGTA	-0.114226373	0.049378736	-0.445125162
B_98618_AACGTGATACACGACCCACTTCGA	0.059621438	0.058600101	-0.32276171
B_98618_AACGTGATAGATCGCAGGTGCGAA	0.186587645	0.049392051	-0.148777672
B_98618_AACGTGATCCGACAACAACGCTTA	0.162241023	0.091727511	-0.397883931
B_98618_AACTCACCAAGAGATCAGGCTAAC	0.232829875	0.032730971	-0.477369227
B_98618_AACTCACCGCTCGGTAAACTCACC	-0.154428652	-0.028456111	-0.402576631
B_98618_AAGACGGAAGCCATGCACGTATCA	0.017409411	0.015318294	-0.232612873
B_98618_AAGACGGAGATGAATCACACGACC	-0.104725555	0.053643092	-0.291507165
B_98618_AAGACGGATCCGTCTATCCGTCTA	0.029074456	-0.031398942	-0.368776749
B_98618_AAGAGATCACACGACCAGAGTCAA	-0.101616555	-0.109101651	-0.612321645
B_98618_AAGAGATCGAGCTGAAGACAGTGC	0.115464131	0.095225858	-0.504354468
B_98618_AAGAGATCTGGTGGTAGAGCTGAA	0.076860074	0.036175474	-0.494892955
B_98618_AAGGACACCAGATCTGCCTCCTGA	0.04174339	-0.079432832	-0.452976503
B_98618_AAGGTACAACGCTCGAGAATCTGA	-0.026784051	-0.101925141	-0.497804876
B_98618_AAGGTACAGATGAATCGTGTTCTA	0.155765385	-0.042189145	-0.556702726
B_98618_AAGGTACAGCTAACGACAAGACTA	-0.159980631	-0.031200524	-0.425272022
B_98618_AATCCGTCCGCATACAAGAGTCAA	0.191512164	0.075506374	-0.53565673
B_98618_AATGTTGCCATACCAACCTAATCC	0.111017913	0.373807182	-0.154194686
B_98618_ACAAGCTAACCTCCAAAAGAGATC	0.228722451	0.056859805	-0.18603821
B_98618_ACAAGCTAACCTCCAAGAATCTGA	-0.082373908	-0.024545077	-0.34347728
B_98618_ACAAGCTACGACACACATTGAGGA	0.353128217	0.130384371	-0.372454685
B_98618_ACAAGCTACTGTAGCCGAGTTAGC	0.047159169	-0.072719923	-0.393046727
B_98618_ACAAGCTAGAATCTGAAGTCACTA	0.113027314	0.141477627	-0.4663892
B_98618_ACAAGCTAGATAGACAGACTAGTA	-0.053790157	0.082605238	-0.21066898
B_98618_ACACAGAACGACTGGAAGCAGGAA	0.025720411	0.014654062	-0.425984364
B_98618_ACACGACCATCCTGTAACACAGAA	0.020516131	0.128666504	-0.099353
B_98618_ACACGACCCAAGACTAATCATTCC	0.351785561	-0.223610099	-0.585073282

B_98618_ACAGATTCCGCTGATCTGGAACAA	0.173214691	0.011665998	-0.406982559
B_98618_ACAGATTCTCCGTCTAGCTAACGA	0.348518003	0.043835635	-0.121119727
B_98618_ACAGCAGAAGCACCTCCCGTGAGA	-0.0369502	0.021736183	-0.349313123
B_98618_ACAGCAGAAGCAGGAACATCAAGT	0.267314887	0.171284531	-0.444601173
B_98618_ACAGCAGACAAGGAGCCGGATTGC	0.002655943	0.039829672	-0.375963199
B_98618_ACAGCAGAGATAGACAAAACATCG	0.521000756	0.388755435	-0.327196196
B_98618_ACCACTGTCGACACACAGAGTCAA	0.241057371	0.029864136	-0.348038417
B_98618_ACCACTGTGACAGTGCCGCATACA	0.418739512	-0.030915424	-0.173576698
B_98618_ACCTCCAAACAAGCTACATACCAA	-0.025524628	-0.171188232	-0.439961246
B_98618_ACCTCCAAACCACTGTACCTCCAA	0.525856314	0.104774444	0.101184039
B_98618_ACCTCCAAAGTCACTAGATAGACA	-0.054936589	-0.244454852	-0.531152999
B_98618_ACCTCCAACCTAATCCACATTGGC	0.121966856	0.068965734	-0.248217884
B_98618_ACGCTCGACAATGGAAATCATTCC	0.049639884	-0.034471071	-0.39621188
B_98618_ACGTATCACGACTGGAGCCACATA	-0.040827018	-0.011551598	-0.55251182
B_98618_ACGTATCAGCTCGGTACCGACAAC	0.235862407	0.123241908	-0.357971603
B_98618_ACTATGCAAGGCTAACAATGTTGC	0.021013034	0.031075585	-0.146763121
B_98618_AGAGTCAACAACCACATGGTGGTA	0.739717837	0.301586857	-0.389170835
B_98618_AGATCGCAAACCGAGAGTACGCAA	-0.13430167	-0.035709751	-0.407841159
B_98618_AGATCGCAACACAGAACCAGTTCA	0.157804089	-0.054297267	-0.418250406
B_98618_AGATCGCACAACCACACTGAGCCA	-0.157695147	0.057410469	-0.39838279
B_98618_AGATCGCACCGTGAGACCATCCTC	0.08949843	0.047332932	-0.028667815
B_98618_AGATCGCAGATAGACAAGTGGTCA	-0.070763519	-0.07622997	-0.462537374
B_98618_AGATCGCAGTACGCAAAGATGTAC	0.102174974	0.173931781	-0.434955
B_98618_AGATCGCATGGCTTCACGGATTGC	-0.065794081	-0.180928262	-0.554023295
B_98618_AGATGTACACGTATCAACGTATCA	-0.010019622	-0.122921032	-0.526709667
B_98618_AGCACCTCCATCAAGTCAGATCTG	0.074744342	0.243710493	-0.436723723
B_98618_AGCAGGAACCAGTTCATCTTCACA	0.295094162	0.243206721	-0.432322833
B_98618_AGCAGGAAGCCAAGACGACAGTGC	-0.233887654	-0.02322767	-0.574522193
B_98618_AGCAGGAAGTCGTAGAGGAGAACA	-0.11940756	0.022940122	-0.227901332
B_98618_AGCAGGAATCTTCACAAGTCACTA	-0.255964584	0.025578299	-0.435085383
B_98618_AGCCATGCCCAGTTCAACCACTGT	0.03782856	-0.072941625	-0.217037048
B_98618_AGCCATGCGAATCTGACCTCTATC	-0.085848054	-0.084317248	-0.448468228

B_98618_AGCCATGCGACAGTGCACGTATCA	0.355564781	-0.016220154	-0.456180806
B_98618_AGGCTAACAAACATCGAACGCTTA	-0.027776939	-0.040886123	-0.471137688
B_98618_AGGCTAACAACTCACCGCCACATA	-0.176766794	0.053100221	-0.22038863
B_98618_AGTACAAGACGCTCGACGCATACA	-0.070999629	0.002335791	-0.417289466
B_98618_AGTACAAGCAGATCTGTGGCTTCA	0.054922985	-0.138231943	-0.437955178
B_98618_AGTACAAGTGGCTTCAAATGTTGC	0.048735098	0.116764041	-0.465831588
B_98618_AGTCACTAAACGTGATTCCGTCTA	0.122930973	0.064182251	-0.240187874
B_98618_AGTCACTAAAGACGGACGCATACA	-0.047208235	-0.039477432	-0.535576695
B_98618_AGTCACTAATAGCGACACACGACC	0.186535364	-0.005644053	-0.233396631
B_98618_AGTGGTCACGGATTGCTCCGTCTA	0.156932915	-0.02130238	-0.452298379
B_98618_AGTGGTCAGACTAGTACGAACTTA	0.244629483	0.06191207	-0.186771095
B_98618_ATAGCGACATGCCTAAAAGGACAC	-0.01183533	-0.038092837	-0.436959235
B_98618_ATAGCGACCAATGGAAAGATGTAC	-0.094126172	-0.030087609	-0.445994835
B_98618_ATAGCGACGGAGAACAACACAGAA	0.026943011	0.013090911	-0.474026902
B_98618_ATGCCTAAATTGAGGACGACTGGA	0.291574961	-0.050364252	-0.440521177
B_98618_ATGCCTAACTGTAGCCCAAGGAGC	0.008741009	0.114086349	-0.310563292
B_98618_ATTGAGGACAAGACTACAGATCTG	-0.113112518	-0.112082483	-0.489282734
B_98618_ATTGAGGACAAGACTAGCCAAGAC	-0.573146619	-0.030704127	-0.087006859
B_98618_ATTGAGGATAGGATGAGATGAATC	0.130771216	-0.0549388	-0.379244547
B_98618_ATTGGCTCAGATGTACCGCATACA	0.259452994	0.113008502	-0.323382884
B_98618_ATTGGCTCCAACCACACACCTTAC	-0.108382377	0.026343872	-0.414838883
B_98618_ATTGGCTCCCAGTTCATGGAACAA	0.648017609	0.341003699	-0.181295732
B_98618_CAACCACACGACACACACCACTGT	0.211596578	-0.001980977	-0.356148494
B_98618_CAACCACACGGATTGCTAGGATGA	0.032925339	0.106870126	-0.378771226
B_98618_CAACCACACTGAGCCACCGACAAC	-0.124151294	0.012250785	-0.446109546
B_98618_CAAGACTACATACCAAACTATGCA	0.075068946	0.02992123	-0.375497289
B_98618_CAAGACTAGCGAGTAAAACTCACC	0.062872844	-0.092375119	-0.259912618
B_98618_CAATGGAAAAGGTACAAGCAGGAA	0.043835771	0.045937233	-0.17480227
B_98618_CAATGGAAAGTGGTCAATTGAGGA	0.038224092	0.037021178	-0.524426882
B_98618_CAATGGAACCGACAACAATGTTGC	0.149023348	0.038819289	-0.333054378
B_98618_CACCTTACAACTCACCACGCTCGA	0.208498334	0.007541474	-0.455121968
B_98618_CACCTTACAGCAGGAACCGAAGTA	0.233706296	0.089479389	-0.567309792

B_98618_CACCTTACTTCACGCACGGATTGC	-0.116103828	-0.1490836	-0.494548877
B_98618_CACTTCGACAATGGAAATTGGCTC	-0.302404569	0.017135164	-0.416829761
B_98618_CACTTCGACTAAGGTCAACTCACC	0.208508457	0.103756987	-0.457996934
B_98618_CACTTCGAGACAGTGCCTGGCATA	0.00200735	0.041643923	-0.218485966
B_98618_CAGATCTGCCAGTTCACCTCCTGA	0.101969567	0.056225567	-0.373290549
B_98618_CAGATCTGGAGTTAGCCATACCAA	0.120236242	0.019240483	-0.427816275
B_98618_CAGCGTTAATAGCGACCATACCAA	-0.105280301	-0.035820227	-0.405618378
B_98618_CATACCAACCATCCTCCTGTAGCC	0.264755192	-0.067084902	-0.519075174
B_98618_CATACCAAGTCTGTCAATCCTGTA	0.059755441	-0.01301976	-0.432821225
B_98618_CATCAAGTACCTCCAACCTCCTGA	0.567176029	0.011184682	-0.378600861
B_98618_CATCAAGTAGCAGGAAAATGTTGC	0.064915513	0.002866713	-0.252416155
B_98618_CATCAAGTCAGCGTTACTAAGGTC	-0.074706439	-0.01983213	-0.459458451
B_98618_CATCAAGTGATAGACAGACTAGTA	0.359771795	0.062716731	-0.027168548
B_98618_CATCAAGTGCCAAGACCATCAAGT	0.27859712	0.013428636	-0.431402282
B_98618_CCAGTTCACGACACACAGTACAAG	-0.023747601	0.037415546	-0.224811564
B_98618_CCAGTTCACGCTGATCCCATCCTC	0.039934577	-0.135389703	-0.474222916
B_98618_CCATCCTCAACTCACCGTCGTAGA	-0.086340138	-0.07346516	-0.534819581
B_98618_CCATCCTCCATCAAGTTGGAACAA	-0.006425685	0.038586164	-0.489202075
B_98618_CCGAAGTACCTCTATCACATTGGC	0.475575919	0.115859687	-0.56400423
B_98618_CCGAAGTAGCGAGTAACAAGACTA	0.048465717	0.036182991	-0.420762657
B_98618_CCGACAACAGTGGTCACACTTCGA	-0.069746157	0.013501522	-0.452998561
B_98618_CCGACAACGACAGTGCGTCTGTCA	0.199805841	-0.006069386	-0.294106905
B_98618_CCGTGAGACCAGTTCAGATGAATC	-0.041976822	-0.089907907	-0.32613385
B_98618_CCGTGAGAGGAGAACAAATCCGTC	0.092069225	0.019742833	-0.421801163
B_98618_CCGTGAGATGGCTTCAGTACGCAA	-0.10976671	-0.083920179	-0.497928554
B_98618_CCGTGAGATGGTGGTACCTCTATC	-0.103601856	-0.172385658	-0.492863728
B_98618_CCTAATCCACGCTCGAAGTCACTA	-0.055872001	-0.002662887	-0.445907722
B_98618_CCTAATCCAGATCGCACCAGTTCA	0.094217808	0.011221104	-0.45295747
B_98618_CCTAATCCAGATCGCACGCTGATC	0.238248324	-0.002820718	-0.440187637
B_98618_CCTAATCCCAAGACTAAAGGTACA	0.709035186	0.183079759	-0.49939411
B_98618_CCTCCTGACTAAGGTCATTGGCTC	0.417749404	0.15667558	-0.089230859
B_98618_CCTCTATCAGCACCTCCTGTAGCC	0.17640016	-0.052404517	-0.510754233

B_98618_CCTCTATCCTAAGGTCCAACCACA	0.081656857	-0.000288673	-0.338474725
B_98618_CCTCTATCTCTTCACAAATCCGTC	0.46831799	0.24458433	-0.240816273
B_98618_CGAACTTACTGTAGCCAGTACAAG	-0.069992886	0.030733707	-0.421508743
B_98618_CGACACACAACTCACCTTCACGCA	0.041524297	-0.038174363	-0.355906052
B_98618_CGACACACCGCATACAAGAGTCAA	0.557018039	0.063686792	-0.426565262
B_98618_CGACTGGAACCTCCAAAAGGACAC	0.111321835	-0.039904325	-0.396321983
B_98618_CGACTGGACTGTAGCCCCGACAAC	0.093436856	0.018675877	-0.510523484
B_98618_CGACTGGAGCCACATAACTATGCA	-0.014709953	0.132260291	-0.387881269
B_98618_CGCATACATGGTGGTACCATCCTC	-0.114936833	-0.027298482	-0.368022611
B_98618_CGCTGATCCAGATCTGGTACGCAA	0.206518959	0.075445947	-0.441932155
B_98618_CGCTGATCGATAGACAATTGAGGA	0.120654965	0.06331922	-0.302726117
B_98618_CGGATTGCACCACTGTGAATCTGA	-0.071143575	-0.113785494	-0.402577912
B_98618_CGGATTGCCAACCACACGACACAC	0.672859378	0.174745307	-0.206298828
B_98618_CTAAGGTCCATCAAGTCGAACTTA	-0.014187557	0.033975871	-0.302466812
B_98618_CTAAGGTCCGACTGGACAGCGTTA	-0.036686165	0.061441302	-0.490000671
B_98618_CTAAGGTCTATCAGCAACAAGCTA	0.130692378	0.163551984	-0.427190046
B_98618_CTGAGCCACACTTCGAGGAGAACA	-0.106699414	-0.052362285	-0.393898767
B_98618_CTGAGCCACCGTGAGAATTGGCTC	-0.157300579	-0.066412527	-0.308098332
B_98618_CTGAGCCACCTCTATCCAGATCTG	-0.004505705	0.011325537	-0.142710252
B_98618_CTGAGCCAGCTAACGAACACGACC	-0.150209072	-0.038218192	-0.380380078
B_98618_CTGAGCCAGTGTTCTAACACGACC	0.101454719	-0.01210006	-0.443467838
B_98618_CTGAGCCATCTTCACACTGGCATA	0.212356882	0.154165177	-0.503443362
B_98618_CTGGCATAAACAACCAAACAACCA	0.300181754	-0.073196027	-0.316780222
B_98618_CTGGCATAGTGTTCTATGGAACAA	0.358215378	0.085542664	-0.443699416
B_98618_CTGGCATATGGAACAAAACGCTTA	-0.006964691	-0.081341456	-0.474517987
B_98618_CTGTAGCCCAATGGAAAATGTTGC	0.147914624	-0.070617285	-0.285534487
B_98618_CTGTAGCCCACTTCGAAGTGGTCA	0.245533646	-0.011309229	-0.375337086
B_98618_CTGTAGCCCCAGTTCAACTATGCA	0.050747388	-0.054165098	-0.495152401
B_98618_GAACAGGCCCTCCTGACTGGCATA	0.283350407	0.199284989	-0.015343226
B_98618_GAACAGGCCTGTAGCCCAGATCTG	-0.063057503	-0.071801288	-0.491768684
B_98618_GAACAGGCTCTTCACAGCTCGGTA	0.244628456	0.007487179	-0.324765101
B_98618_GAACAGGCTGGAACAACAGCGTTA	0.231161334	0.038927023	-0.498070547

B_98618_GAATCTGAAAGGTACACAAGACTA	0.066937388	0.056156769	-0.627206777
B_98618_GAATCTGAAGAGTCAAATAGCGAC	-0.035412657	-0.113966541	-0.44808186
B_98618_GAATCTGACGACACACCCTCCTGA	0.148617663	0.126120679	-0.158927568
B_98618_GAATCTGACGACTGGACATACCAA	0.024696393	0.049634951	-0.27893456
B_98618_GAATCTGAGACTAGTACGCTGATC	0.172911285	0.111529554	-0.240064565
B_98618_GAATCTGAGCCACATATAGGATGA	-0.096122083	0.006338851	-0.469427279
B_98618_GACAGTGCAATGTTGCCAAGGAGC	0.050087934	0.008662226	-0.19476103
B_98618_GACAGTGCGATAGACAGCTAACGA	-0.175805066	0.031682884	-0.26771841
B_98618_GACAGTGCTGGAACAACCAGTTCA	0.324398594	0.156232753	-0.214545756
B_98618_GACTAGTAACAGATTCTAGGATGA	0.423431737	0.036077782	-0.428495869
B_98618_GAGCTGAAAACAACCAGCTCGGTA	-0.042158512	0.094407395	-0.339135527
B_98618_GAGCTGAAAGCACCTCAACGTGAT	-0.146507962	0.146137878	-0.436709078
B_98618_GAGCTGAAAGCACCTCCCTCCTGA	0.026062336	-0.023412833	-0.252224367
B_98618_GAGCTGAACCGAAGTACTCAATGA	-0.033907401	-0.098281718	-0.433275496
B_98618_GAGCTGAAGCTCGGTAAACGCTTA	0.146499031	0.036426881	-0.319675502
B_98618_GAGTTAGCATAGCGACTCTTCACA	0.022789354	-0.022595432	-0.405476483
B_98618_GAGTTAGCTTCACGCACGACACAC	-0.015270701	0.103699315	-0.269671285
B_98618_GATAGACAACAGCAGAGTACGCAA	0.034365707	0.100623469	-0.090126897
B_98618_GATAGACAGAGCTGAACCTAATCC	-0.08299115	0.093354319	-0.490705865
B_98618_GATGAATCAATCCGTCGAACAGGC	-0.018737016	0.01936917	-0.362178043
B_98618_GATGAATCACAGATTCAATCCGTC	0.354121538	0.174711736	-0.163868111
B_98618_GCCACATAACACAGAACCTAATCC	0.09801047	-0.037929842	-0.491546546
B_98618_GCCACATAACATTGGCAATCCGTC	-0.159150901	-0.129415231	-0.436206972
B_98618_GCCACATACAACCACAGTCTGTCA	0.1722134	0.479078758	-0.144510664
B_98618_GCCACATACCGTGAGACGACACAC	0.468875467	0.358833478	-0.531270394
B_98618_GCGAGTAACGCATACAGCCAAGAC	0.23019729	-0.012331599	-0.447385058
B_98618_GCGAGTAACGGATTGCCTAAGGTC	0.164647197	0.077899403	-0.38809604
B_98618_GCGAGTAAGAGTTAGCACGTATCA	0.436159139	0.112264888	-0.386452529
B_98618_GCTAACGAACCACTGTGATAGACA	0.217697498	0.123902367	-0.345020076
B_98618_GCTAACGACAACCACAAACGCTTA	0.394281734	0.15494543	-0.099958042
B_98618_GCTAACGACAACCACAACATTGGC	0.152626492	-0.117001709	-0.378098148
B_98618_GCTAACGACAAGGAGCGGAGAACA	-0.154844957	0.073740142	-0.099026834

B_98618_GCTAACGAGTCGTAGAAAGAGATC	0.029251497	0.14998299	-0.297697738
B_98618_GCTAACGATGAAGAGAAGTGGTCA	0.328196712	0.054746722	-0.382466105
B_98618_GCTCGGTAGAGCTGAAGTCGTAGA	0.360848178	-0.013076141	-0.385138937
B_98618_GCTCGGTAGCTAACGACCTCTATC	-0.008770516	-0.031680565	-0.144444137
B_98618_GCTCGGTAGCTCGGTACACTTCGA	0.170879004	0.143204419	-0.510107059
B_98618_GCTCGGTATCTTCACAACGCTCGA	0.223226341	-0.23430129	-0.598780424
B_98618_GCTCGGTATGGAACAACAAGGAGC	-0.056979621	-0.044786352	-0.473223448
B_98618_GGAGAACAACGTATCAACACAGAA	0.298538623	0.190350961	-0.605394595
B_98618_GGAGAACACTAAGGTCCAATGGAA	0.022128212	0.033530102	-0.284590024
B_98618_GGAGAACATCCGTCTACGCTGATC	-0.060794087	-0.032921599	-0.335803856
B_98618_GGAGAACATGGCTTCAAGATGTAC	0.084901805	0.125713225	-0.127925839
B_98618_GGTGCGAAAACGTGATAAGACGGA	-0.002998728	-0.048566964	-0.492504342
B_98618_GGTGCGAAACCTCCAAAGTGGTCA	-0.083910461	-0.012196533	-0.258380684
B_98618_GGTGCGAACGACTGGAACCACTGT	0.232139312	0.095309184	-0.540796912
B_98618_GGTGCGAAGCCAAGACGGAGAACA	-0.142902603	-0.027426759	-0.385794352
B_98618_GGTGCGAAGGTGCGAAAAGAGATC	0.364964809	-0.033571057	-0.435815014
B_98618_GTACGCAAACACAGAACAACCACA	-0.047478814	-0.018197078	-0.44261967
B_98618_GTACGCAACAAGACTACACTTCGA	-0.106191342	0.127154237	-0.268625964
B_98618_GTACGCAACTGGCATAAGAGTCAA	0.457237632	0.366287138	-0.606080105
B_98618_GTCGTAGAAACGTGATTGGCTTCA	-0.11548867	-0.101137808	-0.37509936
B_98618_GTCGTAGAGCGAGTAATCTTCACA	0.107287226	-0.001146063	-0.269649937
B_98618_GTCTGTCAATCATTCCGGTGCGAA	0.137201324	0.020183057	-0.426644045
B_98618_GTGTTCTAAAGGTACATGGTGGTA	-0.229245581	0.106086287	-0.359409945
B_98618_GTGTTCTACCGACAACCCTCTATC	0.574825265	0.154286432	-0.494458354
B_98618_GTGTTCTAGTACGCAACTGAGCCA	0.009874339	-0.06373484	-0.481822383
B_98618_GTGTTCTATGAAGAGACAAGGAGC	-0.11646335	0.016179011	-0.408948303
B_98618_TAGGATGAACAGATTCCTGGCATA	0.049338684	0.072669954	-0.411282479
B_98618_TAGGATGAGAATCTGAATTGAGGA	-0.453723924	0.088263797	-0.405097664
B_98618_TAGGATGATGGCTTCACATCAAGT	0.078254096	0.034493342	-0.477952259
B_98618_TATCAGCACGAACTTAACCACTGT	0.422772646	-0.117436307	-0.20513992
B_98618_TCCGTCTAAGGCTAACCTCAATGA	-0.159196781	-0.007901072	-0.49191993
B_98618_TCCGTCTAATCCTGTACGCATACA	0.11988049	-0.04315086	-0.333900832

B_98618_TCTTCACAAGATCGCAACGCTCGA	0.009833161	-0.009832055	-0.579880286
B_98618_TCTTCACAGTACGCAATGGTGGTA	0.359706946	0.311949849	-0.30258137
B_98618_TGAAGAGAAACCGAGAATCCTGTA	0.257562279	-0.030435508	-0.249530213
B_98618_TGAAGAGAACAAGCTAACAAGCTA	0.256513235	-0.077002682	-0.576736114
B_98618_TGAAGAGACGACACACGTCTGTCA	0.027530652	0.038314411	-0.361677551
B_98618_TGAAGAGACTGTAGCCAGTGGTCA	0.014380035	-0.134274877	-0.471257447
B_98618_TGGAACAACCGAAGTAACAGATTC	0.03050953	-0.113534185	-0.361144146
B_98618_TGGAACAAGCCACATACTGGCATA	0.284954233	-0.147417661	-0.518817311
B_98618_TGGAACAAGGTGCGAAAACTCACC	-0.248811566	-0.122883265	-0.569941701
B_98618_TGGCTTCACTGTAGCCCCTAATCC	0.282694353	0.379480585	-0.415701302
B_98618_TGGCTTCAGACAGTGCGTCGTAGA	-0.061208076	-0.110527833	-0.553696603
B_98618_TGGTGGTAAAGGACACCTGAGCCA	-0.064838833	-0.197119556	-0.593359926
B_98618_TGGTGGTAAGCCATGCCACCTTAC	-0.507894678	-0.108311254	-0.37054108
B_98618_TGGTGGTAAGTACAAGAAACATCG	0.122694926	-0.009537366	-0.461543961
B_98618_TTCACGCAAAGGACACACGCTCGA	0.099429196	0.068622311	-0.318773236
B_98618_TTCACGCAAGCCATGCCCAGTTCA	0.382620973	0.131416556	-0.421810789
B_98618_TTCACGCAATCATTCCAACTCACC	0.16463142	0.064515667	0.0074433
B_98618_TTCACGCACCTCCTGAATGCCTAA	0.356066483	0.325834176	-0.385479102
B_98618_TTCACGCACTGTAGCCAAGGTACA	0.173290592	0.085311785	-0.14658527
B_98618_TTCACGCAGAACAGGCACGTATCA	0.070547204	-0.026039386	-0.133877267
B_98618_TTCACGCAGTGTTCTACCATCCTC	0.092453235	-0.017010006	-0.44248627
B_98618_TTCACGCATCTTCACATTCACGCA	0.148660056	-0.008584167	-0.388117702
B_98618_AAACATCGCTGGCATACACTTCGA	0.421748037	-0.021417523	-0.57407651
B_98618_AAACATCGTCTTCACACCGTGAGA	0.179229743	-0.115901794	-0.637205242
B_98618_AACCGAGAGGTGCGAATCCGTCTA	0.275197867	0.013713691	-0.471238191
B_98618_AACCGAGATAGGATGAGGAGAACA	-0.03618291	0.048883959	-0.512034938
B_98618_AACGCTTAGAACAGGCGAGTTAGC	0.42791429	0.207258299	-0.592177694
B_98618_AACTCACCACAAGCTAAGTACAAG	-0.058446445	-0.054359152	-0.574941971
B_98618_AACTCACCGCCAAGACACAGCAGA	0.202411684	0.116955156	-0.64061611
B_98618_AAGACGGACCGAAGTAACCTCCAA	-0.087315758	-0.172479339	-0.535045543
B_98618_AAGACGGACCTAATCCCAACCACA	0.654049266	0.579284524	-0.617873001
B_98618_AAGGTACACGGATTGCGTCGTAGA	0.197901462	0.021089974	-0.53735739

B_98618_AATGTTGCCCTCTATCAAACATCG	0.133710319	-0.054619641	-0.453548367
B_98618_ACAAGCTAACATTGGCAGTGGTCA	-0.01907067	0.023535782	-0.518247265
B_98618_ACAAGCTACCGTGAGATCTTCACA	0.24418043	0.211025107	-0.402532704
B_98618_ACACGACCCACTTCGAGGTGCGAA	0.228067341	0.299635331	-0.315033947
B_98618_ACAGATTCAAGGACACGACAGTGC	0.195947801	-0.019208874	-0.429043851
B_98618_ACAGATTCACATTGGCAGCACCTC	0.24036987	0.070280988	-0.309881536
B_98618_ACAGATTCGCTCGGTACAAGGAGC	0.049234304	0.140211551	-0.491933703
B_98618_ACAGCAGAAAACATCGCAAGACTA	0.031222414	0.094411098	-0.641246918
B_98618_ACAGCAGAGTACGCAAATCCTGTA	0.313600471	0.248142078	-0.638155622
B_98618_ACATTGGCAAGAGATCGTACGCAA	0.227742143	0.04651622	-0.210248691
B_98618_ACATTGGCTAGGATGAACGCTCGA	0.478735244	0.115311805	-0.273190736
B_98618_ACCACTGTCAGATCTGCCATCCTC	0.257351111	0.316234311	-0.312258525
B_98618_ACCACTGTCGCTGATCCACCTTAC	-0.070153886	-0.054754052	-0.475185169
B_98618_ACCACTGTCTGAGCCAAAGGACAC	0.037566571	-0.060860857	-0.481868636
B_98618_ACCACTGTGGAGAACAAACAACCA	0.126822709	0.053496664	-0.613322811
B_98618_ACGTATCAAACTCACCAGAGTCAA	0.038857529	0.107437261	-0.332030286
B_98618_ACGTATCAGATGAATCCTCAATGA	0.117880593	0.206846893	-0.247789954
B_98618_ACTATGCACGGATTGCCGACTGGA	-0.047878914	0.051374905	-0.471520772
B_98618_AGAGTCAAAGTACAAGACTATGCA	0.234637861	0.082568789	-0.39244861
B_98618_AGAGTCAAGACAGTGCCCTCCTGA	-0.00763913	-0.10779231	-0.571657592
B_98618_AGAGTCAAGGAGAACACCGTGAGA	-0.059856012	-0.066526496	-0.541313767
B_98618_AGAGTCAATTCACGCACTGAGCCA	-0.278853218	0.077630367	-0.416290765
B_98618_AGATCGCAAACTCACCCTGAGCCA	0.274040066	0.239634787	-0.305632256
B_98618_AGATCGCAGAGTTAGCCCTAATCC	0.899286286	0.12908178	-0.482587748
B_98618_AGATCGCAGGAGAACAGCCACATA	0.068367803	0.107468912	-0.273062552
B_98618_AGATGTACCGCATACAGCCAAGAC	-0.073293601	0.045602309	-0.535138659
B_98618_AGATGTACTGAAGAGACTGGCATA	0.042041733	0.025045908	-0.264216674
B_98618_AGCACCTCAATCCGTCGTACGCAA	0.14875006	0.005362627	-0.492647974
B_98618_AGTACAAGACTATGCATATCAGCA	-0.007716217	0.098431565	-0.518015181
B_98618_AGTACAAGAGTACAAGAAGAGATC	0.424024698	0.279094158	-0.375190132
B_98618_AGTACAAGCTGGCATACCAGTTCA	0.429496978	0.128419419	-0.23011351
B_98618_AGTACAAGTCTTCACAACAGCAGA	0.23223809	0.149618511	-0.570684215

B_98618_AGTCACTAACAGATTCAAACATCG	-0.071759062	-0.027223905	-0.428649124
B_98618_AGTGGTCACGAACTTACCTCCTGA	0.427073967	0.29189782	-0.402078393
B_98618_ATAGCGACAAGACGGAGTGTTCTA	0.012401385	-0.149890286	-0.643472621
B_98618_ATCATTCCAGAGTCAAAACAACCA	0.137450236	0.156421662	-0.285293816
B_98618_ATCCTGTACCTCTATCAACGTGAT	0.117757833	0.032772987	-0.488023695
B_98618_ATGCCTAAGACTAGTATGGAACAA	0.069439619	0.199200505	-0.407213515
B_98618_ATTGAGGAAAGAGATCCCTCCTGA	0.284755552	0.212649596	-0.274749117
B_98618_ATTGAGGACCTCCTGAACAAGCTA	-0.021001389	0.071593183	-0.364706557
B_98618_ATTGAGGACTGGCATACAGCGTTA	0.106388697	0.13833241	-0.275770865
B_98618_ATTGAGGAGGAGAACACCTCCTGA	0.044560079	-0.054813931	-0.417286294
B_98618_ATTGGCTCACCACTGTCAACCACA	0.044873391	0.023542994	-0.557520399
B_98618_ATTGGCTCACGTATCACAATGGAA	0.169607148	0.168016066	-0.443914897
B_98618_ATTGGCTCTGAAGAGACAAGGAGC	0.019283846	0.047589998	-0.338821851
B_98618_CAACCACAACTATGCAAACAACCA	-0.0466233	0.005746409	-0.490932263
B_98618_CAACCACAATGCCTAACATCAAGT	0.210041898	0.208029865	-0.359940463
B_98618_CAACCACACCATCCTCATAGCGAC	0.126778218	0.022137096	-0.64309905
B_98618_CAAGACTAAAGGTACACGACACAC	0.159192503	0.120378429	-0.414210051
B_98618_CAATGGAACAAGGAGCGCCACATA	-0.061019027	0.038637721	-0.370150699
B_98618_CACCTTACCGCATACAATCATTCC	0.302807327	0.136096194	-0.496848787
B_98618_CACTTCGAACAGATTCAGTACAAG	-0.137299743	-0.048615792	-0.600104416
B_98618_CACTTCGACAAGACTAGATGAATC	0.363463603	0.136477731	-0.323676117
B_98618_CACTTCGATAGGATGAGACTAGTA	-0.119745572	0.032435711	-0.474030042
B_98618_CACTTCGATGGTGGTAAACTCACC	0.305264117	-0.002081566	-0.4833048
B_98618_CACTTCGATTCACGCAATCCTGTA	0.11367199	0.103845306	-0.481608405
B_98618_CAGCGTTAAACCGAGACACTTCGA	0.392158754	0.139318399	-0.459839575
B_98618_CAGCGTTACAAGGAGCACACAGAA	0.346272012	0.118134233	-0.27165287
B_98618_CAGCGTTACGCATACACCATCCTC	0.238015028	0.120599	-0.414620956
B_98618_CAGCGTTACGCTGATCACCTCCAA	0.499592321	-0.024418071	-0.395098254
B_98618_CAGCGTTACTAAGGTCGATAGACA	0.441430798	0.338004389	-0.424158294
B_98618_CAGCGTTATGGAACAAAAACATCG	0.123455161	-0.14013256	-0.64226731
B_98618_CATACCAACAAGACTAAGATGTAC	0.06198496	0.067360011	-0.121634715
B_98618_CATACCAAGCCACATAGACTAGTA	0.300143148	0.006462704	-0.564907277

B_98618_CATCAAGTTATCAGCACTGTAGCC	0.15939805	-0.001521977	-0.450184743
B_98618_CCAGTTCAGAATCTGAGAACAGGC	0.280908005	0.084923565	-0.45513113
B_98618_CCATCCTCACTATGCAGCTAACGA	0.300927854	0.115457768	-0.565508098
B_98618_CCGACAACAGTACAAGCAACCACA	-0.097359668	0.066732074	-0.508217484
B_98618_CCGACAACGATGAATCGATGAATC	0.45663137	0.154698165	-0.383892766
B_98618_CCGTGAGAACAGATTCCGGATTGC	-0.040783402	-0.010060181	-0.417731998
B_98618_CCTAATCCAGTCACTAACAAGCTA	-0.025555125	0.042373381	-0.130847892
B_98618_CCTCCTGAGCTAACGAGACTAGTA	0.185745427	0.009195741	-0.472290404
B_98618_CCTCTATCAAGAGATCACCACTGT	0.344686583	-0.056675531	-0.599656791
B_98618_CGACACACCGGATTGCCTCAATGA	-0.017970982	0.104300496	-0.182587382
B_98618_CGACACACTATCAGCATCCGTCTA	0.374526802	0.239813872	-0.647845857
B_98618_CGCATACAACAGATTCAGCCATGC	-0.116898664	-0.006400126	-0.470807732
B_98618_CGCATACAATTGGCTCGGAGAACA	0.108749192	-0.006906631	-0.241116969
B_98618_CGCATACACGCATACATAGGATGA	0.415619714	0.055960045	-0.270773514
B_98618_CGCATACATCCGTCTACAGATCTG	0.896983455	0.096377691	-0.366269924
B_98618_CGCTGATCAACGTGATGAGCTGAA	0.110319457	0.025986396	-0.428655404
B_98618_CGCTGATCCCGTGAGACTGTAGCC	0.277510882	0.136705896	-0.451073588
B_98618_CGGATTGCAACCGAGACCAGTTCA	0.428343785	0.267848222	-0.372552367
B_98618_CGGATTGCGAGTTAGCAAGAGATC	0.398058583	0.097210857	-0.508337827
B_98618_CTAAGGTCAGATCGCAACACGACC	-0.136257684	0.094489992	-0.489164731
B_98618_CTAAGGTCCCGACAACCCGAAGTA	0.338804726	0.32485658	-0.234131574
B_98618_CTCAATGAATGCCTAACAATGGAA	0.640201972	0.064298332	-0.29697249
B_98618_CTCAATGACAGCGTTAACAGCAGA	0.292503574	0.09078713	-0.315472891
B_98618_CTCAATGACTGTAGCCTATCAGCA	-0.001120279	0.367568555	-0.41384737
B_98618_CTGAGCCAACGCTCGAAAGGTACA	0.119734053	-0.005653284	-0.534968359
B_98618_CTGGCATAAGAGTCAAACCTCCAA	0.414649142	-0.004319077	-0.374355469
B_98618_CTGTAGCCAAGACGGAACGCTCGA	0.499697665	0.09717604	-0.390486671
B_98618_CTGTAGCCACGCTCGAGTGTTCTA	0.37703729	0.370630605	-0.63686805
B_98618_GAACAGGCAAGAGATCCAATGGAA	0.047855323	0.054202994	-0.488908337
B_98618_GAACAGGCCTGTAGCCACACAGAA	-0.008507426	0.196861471	-0.174463974
B_98618_GAACAGGCGACAGTGCCCATCCTC	0.100881236	-0.02411113	-0.591719015
B_98618_GACAGTGCTAGGATGATGGAACAA	0.007791724	0.040458605	-0.481098545

B_98618_GACTAGTAACAGATTCAGTCACTA	0.158391038	-0.094617761	-0.373222358
B_98618_GACTAGTAAGAGTCAAAAGACGGA	0.972811582	0.632942559	-0.44692747
B_98618_GAGCTGAAAAGACGGAAATCCGTC	0.344099329	0.272375824	-0.470069637
B_98618_GAGCTGAAGCCACATATGGCTTCA	-0.09044972	-0.073373502	-0.526605192
B_98618_GAGCTGAATGGTGGTAAGTGGTCA	0.141541512	-0.00023153	-0.575637917
B_98618_GAGTTAGCCCATCCTCACACGACC	0.347270785	0.093296694	-0.592350743
B_98618_GAGTTAGCGCCAAGACGCCACATA	-0.0244601	-0.020703632	-0.538292197
B_98618_GATAGACAGATGAATCAACGCTTA	0.559015442	0.187202132	-0.433586911
B_98618_GATGAATCACGCTCGAAACTCACC	0.348852862	0.121093513	-0.200051379
B_98618_GCCAAGACCCGAAGTATGGAACAA	0.151058019	0.102122433	-0.485869909
B_98618_GCCACATAAGCAGGAAGATGAATC	0.145641946	0.044314907	-0.508270143
B_98618_GCGAGTAACCATCCTCCGCATACA	0.230588275	0.013006825	-0.30462907
B_98618_GCTAACGAAACTCACCAGCCATGC	0.224974256	-0.08870136	-0.368645685
B_98618_GCTAACGAACGTATCACCTCTATC	0.279056262	0.126884106	-0.359361031
B_98618_GCTCGGTACATCAAGTACCTCCAA	0.012720726	-0.056935983	-0.446664942
B_98618_GCTCGGTATGGCTTCACGACTGGA	0.064745298	0.041986439	-0.387166557
B_98618_GGAGAACAAGCACCTCAAGGACAC	0.34859691	-0.018777241	-0.257720031
B_98618_GGAGAACATGGAACAATAGGATGA	-0.073770209	-0.078112129	-0.553042308
B_98618_GTACGCAAACATTGGCGTCGTAGA	0.587119409	0.38123904	-0.4250157
B_98618_GTACGCAACAAGGAGCTGGTGGTA	0.083397156	0.047895403	-0.48806833
B_98618_GTACGCAAGCTAACGAAAGACGGA	0.27139456	0.109506819	-0.433645727
B_98618_GTACGCAATGGAACAACTGTAGCC	-0.049536063	0.023803427	-0.490427702
B_98618_GTCGTAGAAACAACCAATAGCGAC	0.010261463	0.001320655	-0.493757188
B_98618_GTCGTAGAAGTGGTCATGGCTTCA	0.196108051	-0.101561907	-0.54058812
B_98618_GTCGTAGAGACTAGTACTGTAGCC	0.113926765	0.004215845	-0.063155597
B_98618_GTGTTCTAATCATTCCACAGCAGA	0.308246006	0.167800414	-0.457682892
B_98618_GTGTTCTATCTTCACAAGTCACTA	0.147706569	0.018887486	-0.339188077
B_98618_TAGGATGAAAACATCGCAGCGTTA	-0.038034006	0.014302385	-0.598544958
B_98618_TAGGATGAAAACATCGTATCAGCA	-0.288767074	0.032049702	-0.530492207
B_98618_TAGGATGACCGAAGTACATCAAGT	0.248848627	0.194379358	-0.486701617
B_98618_TAGGATGACTCAATGAATGCCTAA	0.524548263	0.458765826	-0.267129792
B_98618_TATCAGCACACTTCGACTGAGCCA	0.167741322	0.13144905	-0.377576599

B_98618_TCTTCACAAACCGAGAGTCGTAGA	0.315315828	0.02906526	-0.392242963
B_98618_TCTTCACACCATCCTCCAGCGTTA	0.324447375	0.033601745	-0.396164199
B_98618_TCTTCACAGGTGCGAAGCCACATA	0.357015847	-0.003694297	-0.617141866
B_98618_TGAAGAGAATTGAGGAGATAGACA	0.04274462	0.103708119	-0.589262418
B_98618_TGAAGAGAGTACGCAAGACAGTGC	0.034313633	0.259078698	-0.320313551
B_98618_TGAAGAGATGAAGAGACTGTAGCC	0.366550025	0.061900948	-0.480743691
B_98618_TGGAACAACGACTGGACCGAAGTA	0.052175849	0.036504494	-0.402799258
B_98618_TGGCTTCATTCACGCAAGTGGTCA	0.378520979	-0.004993018	-0.432656784
B_98618_TGGTGGTAAACAACCACGACTGGA	0.178074919	-0.01673731	-0.384978921
B_98618_TGGTGGTAATCCTGTATCCGTCTA	0.275024027	0.201639371	-0.353195059
B_98618_TGGTGGTACTCAATGATCTTCACA	-0.160065419	0.016821026	-0.566812378
B_98618_TTCACGCACGACTGGAATCCTGTA	-0.010003976	0.150359368	-0.310581739
B_98618_ACCTCCAAAGGCTAACAGTGGTCA	0.182658501	0.020880188	-0.383542982
B_98618_AGTGGTCAGCTAACGAGTGTTCTA	0.375600839	0.134531703	-0.264616764
B_98618_CAGCGTTAGGAGAACAAAACATCG	0.005766342	0.102187034	-0.333444924
B_98618_CCGAAGTATGGCTTCAAACGCTTA	0.398902191	0.212347172	-0.261937826
B_98618_CTGAGCCAGATGAATCATCATTCC	0.630403501	-0.008869646	-0.488828986
B_98618_GACAGTGCATCATTCCCGCTGATC	-0.070064388	-0.033574169	-0.511233826
B_98618_TATCAGCAGGTGCGAAAGCAGGAA	0.132675875	0.013875705	-0.451317178
ab_99547_CAAGACTAAACGTGATAGATGTAC	0.274111371	0.06698732	-0.29857837
ab_99547_CAATGGAAGATGAATCATTGAGGA	0.26769822	0.082717501	-0.440389246
ab_99547_AAGGACACCGACACACAACTCACC	0.615620882	0.061202407	-0.483836288
ab_99547_AGATGTACGAGCTGAACGCATACA	0.445492565	0.021537942	-0.393518392
ab_99547_GCCACATAGTCGTAGACGCTGATC	0.601276254	0.057038888	-0.371436204
ab_99547_GCGAGTAATGGTGGTAGCTAACGA	0.693755298	-0.060312432	-0.30972857
ab_99547_GTCTGTCATGAAGAGACAACCACA	0.612525109	0.257366677	-0.295605276
ab_99547_AAGAGATCATCATTCCGCCACATA	0.642142247	0.227249834	-0.556823746
ab_99547_AATCCGTCCAAGGAGCACGCTCGA	0.784431689	0.044652397	-0.442433025
ab_99547_ACAAGCTACCGTGAGAAGATGTAC	0.106021351	0.171126796	-0.479609511
ab_99547_ACAAGCTACGCTGATCCAATGGAA	0.480394921	0.363791515	-0.482325048
ab_99547_ACAGATTCGAATCTGACTGAGCCA	0.660742516	0.387572678	-0.430465999
ab_99547_AGATGTACCTAAGGTCATAGCGAC	0.664194932	0.642211533	-0.308736545

ab_99547_CCATCCTCGACTAGTACATACCAA	0.552449223	0.45409149	-0.346826277
ab_99547_CTCAATGATCCGTCTAGCTAACGA	0.818539631	0.400014737	-0.437696965
ab_99547_CTGGCATACCTCCTGAAATGTTGC	0.500366796	0.127932193	-0.577547741
ab_99547_GAACAGGCTGAAGAGAATAGCGAC	0.526084817	0.219455406	-0.51353901
ab_99547_GTCGTAGAGAATCTGACCGACAAC	0.557168813	0.821915996	-0.32578921
ab_99547_GTGTTCTAGAACAGGCCAAGGAGC	0.779793081	0.03857647	-0.540203379
ab_99547_AAACATCGAACAACCAGTCTGTCA	0.459192902	0.086051891	-0.382696704
ab_99547_AAACATCGAGTCACTAAGATCGCA	0.559741533	0.091633717	-0.535995447
ab_99547_AACAACCAAGCACCTCCCATCCTC	0.565979695	0.125042876	-0.195577973
ab_99547_AACAACCAAGCACCTCCTCAATGA	0.395815647	-0.00017649	-0.507783791
ab_99547_AACCGAGAAAGAGATCAGTACAAG	0.49344585	0.03696308	-0.462339872
ab_99547_AACCGAGACAAGGAGCAATGTTGC	0.393979588	0.043458328	-0.285208173
ab_99547_AACGCTTACAAGGAGCACAGATTC	0.70595093	0.109976795	-0.364836409
ab_99547_AACGTGATACTATGCAGCCACATA	0.461839013	0.066534173	-0.410662903
ab_99547_AACGTGATAGTCACTAGAGCTGAA	0.31926912	0.006820427	-0.403623214
ab_99547_AACTCACCCATCAAGTAATCCGTC	0.394441856	-0.019477383	-0.422393656
ab_99547_AACTCACCCCTCCTGACGACACAC	0.592100782	0.065645845	-0.366706889
ab_99547_AACTCACCGAATCTGAGTCTGTCA	0.597291937	0.060355476	-0.244329842
ab_99547_AACTCACCGACTAGTAACATTGGC	0.510630907	0.112872065	-0.476709004
ab_99547_AACTCACCGGAGAACAAGTCACTA	0.68551287	0.347296134	-0.452821765
ab_99547_AAGACGGATATCAGCATGAAGAGA	0.604787524	-0.058803252	-0.40523504
ab_99547_AAGGTACAAATCCGTCCCGTGAGA	0.345880568	0.111504111	-0.426853187
ab_99547_AAGGTACAAATGTTGCATAGCGAC	0.563636738	0.137434836	-0.56848691
ab_99547_AAGGTACAGATAGACACTGTAGCC	0.521666369	0.082854632	-0.445283332
ab_99547_AATCCGTCAGAGTCAAAAGGTACA	0.357829274	0.058311175	-0.241401298
ab_99547_AATGTTGCCAGCGTTAACGCTCGA	0.4767785	-0.005295517	-0.271695139
ab_99547_AATGTTGCGACTAGTACAACCACA	0.580291357	0.113200238	-0.473191447
ab_99547_ACACAGAAACAGCAGAATTGGCTC	0.554426643	0.166961987	-0.400208019
ab_99547_ACACGACCAGATGTACGATGAATC	0.316532085	0.039405356	-0.352075184
ab_99547_ACACGACCCCAGTTCACATACCAA	0.586328195	0.011600787	-0.376884445
ab_99547_ACACGACCTGGTGGTAACACGACC	0.31565653	0.034880527	-0.341935856
ab_99547_ACACGACCTGGTGGTAGTCTGTCA	0.371390519	0.183735076	-0.268101313

ab_99547_ACAGATTCCAAGACTAGTGTTCTA	0.29465751	0.077137398	-0.362608828
ab_99547_ACAGATTCCAATGGAAATCATTCC	0.536311084	0.121015081	-0.388617736
ab_99547_ACAGATTCCATCAAGTTGAAGAGA	0.399826914	-0.139586542	-0.497083385
ab_99547_ACAGATTCTGAAGAGAGCGAGTAA	0.470999462	0.097658992	-0.418603724
ab_99547_ACAGCAGAACATTGGCCATACCAA	0.770773641	0.104665107	-0.36771347
ab_99547_ACATTGGCGAATCTGAGCCACATA	-0.315285822	0.153885373	-0.354475026
ab_99547_ACATTGGCGCTAACGAGATAGACA	0.45094742	0.00285995	-0.523254476
ab_99547_ACCACTGTCAATGGAACTAAGGTC	0.333306167	-0.025809422	-0.487147992
ab_99547_ACCACTGTCTAAGGTCCGACACAC	0.388270745	0.092678342	-0.300263801
ab_99547_ACCTCCAACGCTGATCCGAACTTA	0.917496011	-0.105046627	-0.502815188
ab_99547_ACCTCCAAGAATCTGAAACAACCA	0.543495184	0.09941084	-0.21959796
ab_99547_ACCTCCAAGACAGTGCCTGGCATA	0.353001223	-0.01943841	-0.58139967
ab_99547_ACCTCCAAGATAGACAAGGCTAAC	0.595672618	0.203778706	-0.279897077
ab_99547_ACCTCCAAGTCTGTCACAATGGAA	0.537195235	0.055099269	-0.495806852
ab_99547_ACGCTCGAGAATCTGAGCTAACGA	0.416802961	-0.005135428	-0.341476997
ab_99547_ACGCTCGAGCCACATAGATAGACA	0.792368855	0.30224059	-0.406070238
ab_99547_ACGTATCACAACCACAAGTGGTCA	0.334141428	0.065653382	-0.138854613
ab_99547_ACGTATCACGACTGGACCGACAAC	0.29261043	0.016083645	-0.324391427
ab_99547_ACTATGCAGCTAACGATCCGTCTA	0.519285859	0.179581532	-0.361660652
ab_99547_AGAGTCAAAAGGACACAGAGTCAA	0.343707886	0.008193538	-0.28265911
ab_99547_AGAGTCAAACAGCAGACGGATTGC	0.651349139	0.13960162	-0.28208129
ab_99547_AGAGTCAAAGTCACTAACGTATCA	0.546811805	0.370387365	-0.583072981
ab_99547_AGAGTCAATAGGATGACACTTCGA	0.39513865	0.03310174	-0.458991944
ab_99547_AGATCGCAAGTCACTAGTACGCAA	0.215874396	-0.129065584	-0.521577861
ab_99547_AGATCGCACAGATCTGGGAGAACA	0.596739466	0.019207571	-0.489329531
ab_99547_AGATCGCATGAAGAGAAACGTGAT	0.612684703	0.031354355	-0.420671416
ab_99547_AGCACCTCAACTCACCGCCACATA	0.820281466	0.46585036	-0.62795316
ab_99547_AGCACCTCCAGATCTGCACCTTAC	0.50758312	0.013198098	-0.450786708
ab_99547_AGCACCTCCCTCCTGAAATGTTGC	0.534985322	-0.051688545	-0.420891303
ab_99547_AGCAGGAAACAGCAGAAGTGGTCA	0.323958733	0.005291149	-0.282681281
ab_99547_AGCCATGCACTATGCACCATCCTC	0.455842434	0.047416073	-0.281351213
ab_99547_AGCCATGCCAGCGTTAAGTGGTCA	0.196895594	0.07226158	-0.45021794

ab_99547_AGCCATGCCGACACACAACAACCA	0.487646786	-0.102898596	-0.477685506
ab_99547_AGGCTAACCGAACTTAAACTCACC	0.591843216	-0.12961793	-0.572285399
ab_99547_AGTACAAGCTAAGGTCACGTATCA	0.633722074	0.115542467	-0.33731028
ab_99547_AGTCACTAACACAGAAACAGATTC	0.406361825	0.096547698	-0.162135257
ab_99547_AGTCACTAACATTGGCCCTCTATC	0.341042725	-0.002907313	-0.537800867
ab_99547_AGTCACTATCTTCACACCTAATCC	0.418277372	-0.036016609	-0.312346939
ab_99547_AGTGGTCACGGATTGCCTGAGCCA	0.734257027	0.213086684	-0.312781205
ab_99547_ATAGCGACAAGAGATCCGACTGGA	0.374306314	0.058834389	-0.335230409
ab_99547_ATAGCGACAGCAGGAAGTCGTAGA	0.242156242	-0.029101088	-0.329590177
ab_99547_ATAGCGACTATCAGCAAGCACCTC	0.466182014	0.045274365	-0.429617783
ab_99547_ATCATTCCACACGACCCTGAGCCA	0.430090732	0.09039383	-0.345397278
ab_99547_ATCATTCCACGTATCAAAGACGGA	0.551202829	0.112215267	-0.06569146
ab_99547_ATCATTCCCGAACTTAATCATTCC	0.45581706	-0.024753029	-0.379792596
ab_99547_ATCATTCCTCCGTCTAATCATTCC	0.647913151	0.134180044	-0.249049574
ab_99547_ATGCCTAAAGATGTACACCTCCAA	0.509855405	0.134023452	-0.401187291
ab_99547_ATGCCTAAATCATTCCTGGTGGTA	0.394729731	0.156236021	-0.307249743
ab_99547_ATTGAGGAAGCCATGCACATTGGC	0.301020721	-0.042128404	-0.387908945
ab_99547_ATTGGCTCAAACATCGGAATCTGA	0.371224101	0.030028954	-0.444657531
ab_99547_ATTGGCTCACAGATTCGCTCGGTA	0.334061356	0.225000855	-0.323686874
ab_99547_ATTGGCTCACATTGGCCCTCCTGA	0.493395357	0.143635538	-0.538698303
ab_99547_ATTGGCTCGAACAGGCCCATCCTC	0.503786639	-0.081039507	-0.334893487
ab_99547_ATTGGCTCTAGGATGAGCGAGTAA	0.314105449	0.174675403	-0.248104994
ab_99547_ATTGGCTCTGGTGGTACTGGCATA	0.37284171	0.000495281	-0.381888454
ab_99547_CAACCACAAATCCGTCCCAGTTCA	0.463846002	0.127729179	-0.311274777
ab_99547_CAACCACAGACAGTGCGTCTGTCA	0.5560469	0.019174851	-0.557194811
ab_99547_CAAGACTAAAGACGGACAAGACTA	0.775859103	0.116292457	-0.32142469
ab_99547_CAAGACTAAATCCGTCCTCAATGA	0.257601969	0.075267141	-0.274298309
ab_99547_CAAGACTAACCACTGTTGGAACAA	0.471611783	-0.050369506	-0.297879625
ab_99547_CAAGACTAATTGAGGAACATTGGC	0.610183674	-0.02512752	-0.31414864
ab_99547_CAAGACTACATCAAGTTGGCTTCA	0.568462445	0.096021518	-0.145655698
ab_99547_CAAGACTACCTCCTGATGGCTTCA	0.732203598	-0.102273449	-0.423690824
ab_99547_CAAGGAGCAACAACCACTGGCATA	0.506250241	-0.01920565	-0.499416527

ab_99547_CAAGGAGCCATACCAAACGCTCGA	0.091472132	-0.027973248	-0.447786431
ab_99547_CAAGGAGCCCATCCTCCCGAAGTA	0.677879918	0.082037027	-0.397852804
ab_99547_CAAGGAGCGATAGACATCTTCACA	0.22367543	0.045556626	-0.302679363
ab_99547_CAATGGAAGACAGTGCAAACATCG	0.4001733	-0.02770662	-0.326907792
ab_99547_CACCTTACCGCTGATCGAGTTAGC	0.48868383	0.214431482	-0.352533564
ab_99547_CACTTCGAAACGTGATCTAAGGTC	0.75956475	0.100986676	-0.417905318
ab_99547_CACTTCGACAAGGAGCTTCACGCA	0.346861167	0.026707579	-0.310045546
ab_99547_CACTTCGAGTACGCAAAACGTGAT	0.764533524	-0.024867134	-0.582549252
ab_99547_CACTTCGATATCAGCACAGCGTTA	0.616054681	0.09987998	-0.362512255
ab_99547_CACTTCGATGAAGAGACAAGACTA	0.435624878	0.27347783	-0.340970408
ab_99547_CAGCGTTAACCACTGTGCTAACGA	0.339965293	0.019700186	-0.167206206
ab_99547_CAGCGTTAAGATCGCAGCCACATA	0.729896875	0.156718412	-0.508351379
ab_99547_CAGCGTTAAGCACCTCTCCGTCTA	0.535469324	0.087625797	-0.255660201
ab_99547_CAGCGTTAATCATTCCACACGACC	0.446632876	0.036792776	-0.386714571
ab_99547_CAGCGTTATGGAACAAGACAGTGC	0.29495223	0.156363665	-0.392303291
ab_99547_CATACCAAAGATCGCACGACACAC	0.526808876	0.041273263	-0.385586225
ab_99547_CATACCAAAGTACAAGTATCAGCA	0.233310987	0.067150501	-0.353981817
ab_99547_CATACCAACAGATCTGATAGCGAC	0.217691131	0.027178472	-0.324656352
ab_99547_CATACCAACATCAAGTACGCTCGA	0.304479782	0.117404877	-0.300280603
ab_99547_CATACCAACTGGCATACCATCCTC	0.27826525	0.120496211	-0.285638968
ab_99547_CATACCAAGAGTTAGCCAAGACTA	0.468402394	0.14961702	-0.411595635
ab_99547_CATCAAGTAAACATCGGATGAATC	0.336343762	0.09456643	-0.270149932
ab_99547_CATCAAGTCGCTGATCACCTCCAA	0.722319984	-0.070819191	-0.383113937
ab_99547_CATCAAGTCTCAATGAAAGGTACA	0.70346178	0.151334934	-0.371633542
ab_99547_CCAGTTCAAACTCACCCGACACAC	0.594193058	0.132403212	-0.250194079
ab_99547_CCAGTTCAAGAGTCAACAGATCTG	0.325193356	-0.057600246	-0.304327866
ab_99547_CCAGTTCACCAGTTCATGGCTTCA	0.435307217	0.230480666	-0.4227747
ab_99547_CCAGTTCACGAACTTAATTGGCTC	0.592995396	0.05117441	-0.418589096
ab_99547_CCAGTTCACGGATTGCGTCGTAGA	0.417140688	0.105605331	-0.411557431
ab_99547_CCAGTTCAGAGTTAGCAAGGTACA	1.028970212	0.146702512	-0.198879157
ab_99547_CCAGTTCAGGTGCGAAAAACATCG	0.259105359	-0.05103113	-0.394863029
ab_99547_CCATCCTCAACCGAGAAGATGTAC	0.622032912	0.010396424	-0.382757421

ab_99547_CCATCCTCAACGTGATCGAACTTA	0.31972009	0.102896684	-0.235754933
ab_99547_CCATCCTCGTCGTAGAATAGCGAC	0.377627166	0.017535406	-0.21441978
ab_99547_CCATCCTCGTGTTCTAGTGTTCTA	0.40432977	0.029723286	-0.482669538
ab_99547_CCGAAGTAAATCCGTCAAGGACAC	0.525253876	0.021025627	-0.437614009
ab_99547_CCGACAACAACAACCACCTAATCC	0.585514372	0.152461162	-0.377174353
ab_99547_CCGTGAGACTGTAGCCTTCACGCA	0.473738726	0.15149592	-0.371243161
ab_99547_CCGTGAGAGACTAGTACAATGGAA	0.135901272	-0.188221552	-0.424809143
ab_99547_CCGTGAGAGAGCTGAAGCTCGGTA	0.533904308	-0.058893093	-0.481388509
ab_99547_CCGTGAGAGGAGAACAATCCTGTA	0.457843765	0.084911091	-0.391256255
ab_99547_CCTAATCCACAAGCTAGGTGCGAA	0.489040291	0.155980691	-0.372096163
ab_99547_CCTAATCCAGATGTACCTAAGGTC	0.541321655	0.021907527	-0.413250017
ab_99547_CCTAATCCATTGGCTCAGAGTCAA	0.198790551	0.060649043	-0.454523868
ab_99547_CCTCCTGACGCATACAACGTATCA	0.520810641	0.022456046	-0.348587998
ab_99547_CCTCTATCCCGTGAGACTGAGCCA	0.402540809	0.124696461	-0.433391815
ab_99547_CCTCTATCGGTGCGAACCTCTATC	0.637133882	0.100478837	-0.344809461
ab_99547_CGAACTTAAACAACCATGGAACAA	0.364272079	0.116003174	-0.410393256
ab_99547_CGAACTTACGAACTTACAGCGTTA	0.50084195	-0.085278677	-0.53462302
ab_99547_CGACACACCTGTAGCCGAGTTAGC	0.394396993	-0.007373644	-0.341165437
ab_99547_CGACTGGAAATCCGTCAGCACCTC	0.321600636	0.037303789	-0.226404789
ab_99547_CGACTGGAATTGAGGACACCTTAC	0.404327892	-0.07681505	-0.438927804
ab_99547_CGACTGGACCTCTATCCGAACTTA	0.3453537	0.00443285	-0.349966425
ab_99547_CGCATACAAGTGGTCACGACACAC	0.507696059	0.066285778	-0.271283288
ab_99547_CGCATACACATACCAAACATTGGC	0.498823715	0.140403098	-0.378182316
ab_99547_CGCATACACGGATTGCCACCTTAC	0.756982253	0.051371743	-0.362750242
ab_99547_CGCTGATCAACAACCAACAGCAGA	0.461496247	0.13166259	-0.353360673
ab_99547_CGGATTGCACGCTCGAACAGCAGA	0.116363174	-0.080651506	-0.400429564
ab_99547_CGGATTGCGTCTGTCAACGTATCA	0.534013991	0.003743927	-0.51372014
ab_99547_CTAAGGTCACAGCAGAACAGATTC	0.462292104	0.022317926	-0.274621181
ab_99547_CTAAGGTCAGTACAAGGTCGTAGA	0.340607099	-0.004178625	-0.490954447
ab_99547_CTCAATGAAATCCGTCAAGAGATC	0.511032333	-0.054168649	-0.437259214
ab_99547_CTCAATGAACATTGGCTGGTGGTA	0.698258114	0.079964196	-0.364832516
ab_99547_CTCAATGAGCCACATACATCAAGT	0.919100466	0.093374816	-0.432231565

ab_99547_CTGAGCCACCTAATCCCACTTCGA	0.525747577	-0.075987357	-0.342409607
ab_99547_CTGGCATAACAAGCTAAGCACCTC	0.546982318	-0.12669542	-0.414707393
ab_99547_CTGTAGCCTGGTGGTACAAGACTA	0.348286559	0.079873509	-0.380488383
ab_99547_GAATCTGAAATGTTGCCACCTTAC	0.788439787	0.145830918	-0.404638639
ab_99547_GAATCTGACTGTAGCCTAGGATGA	0.676237395	0.046197788	-0.240133257
ab_99547_GACAGTGCACAGCAGACTCAATGA	0.353305317	0.001242484	-0.417372129
ab_99547_GACAGTGCAGAGTCAACTCAATGA	0.369215349	-0.009237186	-0.360718902
ab_99547_GACTAGTACCGACAACGACTAGTA	0.299181194	0.027754717	-0.4160343
ab_99547_GACTAGTAGCTAACGAGATAGACA	0.566323015	0.088432635	-0.36713136
ab_99547_GACTAGTAGTCGTAGACTGTAGCC	0.225846053	0.015956739	-0.359752317
ab_99547_GAGCTGAACCTAATCCCCTCCTGA	0.226449525	-0.027929291	-0.439925959
ab_99547_GAGCTGAAGAGTTAGCAGTGGTCA	0.562279072	0.042034014	-0.2476591
ab_99547_GAGTTAGCAGATCGCACACTTCGA	0.90520502	0.235512923	-0.508461749
ab_99547_GAGTTAGCAGATCGCACGAACTTA	0.943049561	0.166230451	-0.288170698
ab_99547_GAGTTAGCATCATTCCTGGTGGTA	0.703255514	0.058954592	-0.260120001
ab_99547_GAGTTAGCCGAACTTAAACCGAGA	0.509203446	0.139462373	-0.294753812
ab_99547_GAGTTAGCGAGTTAGCTATCAGCA	0.399690928	-0.052214189	-0.4959652
ab_99547_GAGTTAGCTTCACGCAAAGAGATC	0.443015496	0.070347232	-0.121945019
ab_99547_GATAGACACAAGACTAACGCTCGA	0.431340028	0.049079353	-0.503923895
ab_99547_GATAGACAGAACAGGCAAACATCG	0.327516279	-0.060769482	-0.243369864
ab_99547_GATGAATCAAGGACACGCCAAGAC	0.689156835	0.128220652	-0.191406053
ab_99547_GATGAATCAATCCGTCAAGAGATC	0.427032593	-0.011699868	-0.391083749
ab_99547_GATGAATCCCTCTATCGATAGACA	0.50780001	0.044731489	-0.387241188
ab_99547_GATGAATCCGACACACACAGCAGA	0.537942282	0.160757323	-0.451622184
ab_99547_GATGAATCGTCTGTCATGGAACAA	0.687001124	-0.040621915	-0.407942218
ab_99547_GATGAATCGTGTTCTAATCCTGTA	0.424627202	0.115926464	-0.40446568
ab_99547_GCCAAGACCGGATTGCGATGAATC	0.558614037	0.225319391	-0.474378709
ab_99547_GCCACATAAGGCTAACAAGGACAC	0.427619277	0.106224453	-0.43185524
ab_99547_GCCACATACGCATACATAGGATGA	0.484525088	0.136977729	-0.397650716
ab_99547_GCGAGTAAAAACATCGTCCGTCTA	0.554659463	0.055009124	-0.406536635
ab_99547_GCGAGTAAACGCTCGACAAGACTA	0.586325179	0.053585318	-0.373006727
ab_99547_GCGAGTAAAGGCTAACCCTAATCC	0.183559932	0.113928164	-0.368418875

ab_99547_GCTAACGAAACCGAGAAGATGTAC	0.904570785	0.230024609	-0.374938991
ab_99547_GCTAACGACGAACTTAGTCGTAGA	0.561274233	-0.058488102	-0.459040381
ab_99547_GCTCGGTACACTTCGAAATCCGTC	0.075251847	-0.057293412	-0.394540968
ab_99547_GGAGAACAACCACTGTCTCAATGA	0.592269916	0.286692199	-0.345561045
ab_99547_GGAGAACACAACCACACTGAGCCA	0.669197625	0.106412703	-0.583070558
ab_99547_GGTGCGAAAACCGAGATGGTGGTA	0.309201242	-0.047903589	-0.379670349
ab_99547_GGTGCGAACACCTTACCGACACAC	0.372976965	0.022758884	-0.353344628
ab_99547_GTACGCAACCGAAGTACAAGGAGC	0.482695878	0.136908793	-0.233982982
ab_99547_GTACGCAAGGAGAACAGACTAGTA	0.694800278	0.032104855	-0.514028818
ab_99547_GTCGTAGAAAGGTACAATAGCGAC	0.458659731	0.125804782	-0.293708922
ab_99547_GTCGTAGAAATCCGTCTTCACGCA	0.492319949	-0.054159078	-0.528506124
ab_99547_GTCGTAGAAGCACCTCGCTAACGA	0.457529286	0.092067037	-0.255789666
ab_99547_GTCGTAGAATTGAGGACGACACAC	0.386613012	-0.101386717	-0.523896389
ab_99547_GTCGTAGACCTCCTGAGTCGTAGA	0.400538906	0.069486459	-0.41444062
ab_99547_GTCTGTCAAAGGTACAGAATCTGA	0.884019777	0.17304053	-0.140584021
ab_99547_GTCTGTCACATCAAGTTAGGATGA	0.615205687	0.15228997	-0.21593332
ab_99547_GTGTTCTAACCACTGTGCTAACGA	0.521493879	0.240430439	-0.532199099
ab_99547_GTGTTCTACCATCCTCGCTAACGA	0.541324393	0.102979277	-0.545876841
ab_99547_TATCAGCAACCACTGTCCAGTTCA	0.373899587	0.067228197	-0.396063799
ab_99547_TATCAGCACAATGGAAGATAGACA	0.5365275	0.056238992	-0.195702259
ab_99547_TATCAGCACCGTGAGAGGAGAACA	0.675391493	0.126448075	-0.389503054
ab_99547_TCCGTCTAAGTGGTCAAATGTTGC	0.646962479	0.156309398	-0.395520048
ab_99547_TCCGTCTAGCCAAGACACAGATTC	0.410675701	0.045830433	-0.364267066
ab_99547_TCTTCACAATCCTGTAAGGCTAAC	0.610666179	0.165439943	-0.269696749
ab_99547_TGAAGAGAAACGCTTAAACGCTTA	0.639878323	0.183949787	-0.256335437
ab_99547_TGAAGAGAACACGACCAAGGACAC	0.352802874	-0.048635953	-0.597598644
ab_99547_TGAAGAGAAGTACAAGCCTAATCC	0.431996759	0.009445213	-0.354970362
ab_99547_TGAAGAGAGAATCTGACTGTAGCC	0.301788436	0.016312196	-0.474338067
ab_99547_TGGAACAACGACTGGATAGGATGA	0.865642253	0.20517516	-0.446964682
ab_99547_TGGTGGTAAAGAGATCGAGTTAGC	0.317246304	-0.104181692	-0.548062586
ab_99547_TGGTGGTAAGGCTAACACAAGCTA	0.522249061	0.100633863	-0.37843768
ab_99547_TGGTGGTACCGTGAGAACAAGCTA	0.413854037	0.048976517	-0.486624219

ab_99547_TTCACGCACACTTCGAGTCGTAGA	0.69006866	0.114062827	-0.340832695
ab_99547_TTCACGCACGCATACACACTTCGA	0.592541996	0.153845337	-0.507860214
ab_99547_AAACATCGCTGGCATACTGGCATA	0.450739151	0.010331785	-0.468719871
ab_99547_AACAACCAAGAGTCAAGCGAGTAA	0.811980609	0.300711218	-0.492254316
ab_99547_AACCGAGAGCCACATAATCATTCC	0.410472441	0.017677882	-0.518825471
ab_99547_AACGCTTAGTCTGTCAACCTCCAA	0.668192547	0.395622037	-0.461794492
ab_99547_AACGCTTATTCACGCACCGACAAC	-0.230151696	0.188613603	-0.261737787
ab_99547_AACGTGATACACGACCAACAACCA	0.314068511	-0.077654957	-0.427201451
ab_99547_AACGTGATACCTCCAACTGGCATA	0.502107742	0.161536365	-0.389216716
ab_99547_AACGTGATATCATTCCTGGTGGTA	0.55512092	-0.023344671	-0.374784406
ab_99547_AACGTGATCTAAGGTCGTCGTAGA	0.566499189	0.136329703	-0.416268356
ab_99547_AACGTGATCTGGCATATCTTCACA	0.572151095	-0.002943614	-0.464439198
ab_99547_AACGTGATGGTGCGAACTGGCATA	0.280060442	-0.025744541	-0.552377674
ab_99547_AACGTGATTCCGTCTAGTCGTAGA	0.492197897	0.110565633	-0.392279861
ab_99547_AACTCACCATCATTCCAGCCATGC	0.616833333	0.150879426	-0.39913898
ab_99547_AACTCACCATTGAGGACATCAAGT	0.477625714	0.11005075	-0.29004306
ab_99547_AAGACGGAAAACATCGAACAACCA	0.521542802	0.200863592	-0.529745638
ab_99547_AAGACGGAACACAGAACAATGGAA	0.628414554	-0.074344847	-0.524565254
ab_99547_AAGACGGAAGTGGTCAAAGACGGA	0.299822721	-0.032039239	-0.579437344
ab_99547_AAGACGGAGCTAACGAAAGACGGA	0.627547141	-0.006754379	-0.399995566
ab_99547_AAGAGATCAAGACGGAAAGAGATC	0.553316861	0.077419875	-0.491466036
ab_99547_AAGGTACAAATCCGTCCATCAAGT	0.171537956	0.069696671	-0.496916569
ab_99547_AAGGTACACGACACACAATGTTGC	0.703148286	0.154030487	-0.404165272
ab_99547_AATCCGTCAATCCGTCAACCGAGA	0.463941502	0.032902142	-0.373985496
ab_99547_AATCCGTCAGGCTAACACGTATCA	0.689418063	0.200025061	-0.586121174
ab_99547_AATCCGTCCGCATACAGCTCGGTA	0.526851863	0.05820383	-0.484870394
ab_99547_AATCCGTCTGAAGAGAACACAGAA	0.923806952	0.292241455	-0.306256826
ab_99547_AATGTTGCAATGTTGCAGCAGGAA	0.273944368	-0.066512245	-0.425052915
ab_99547_AATGTTGCATAGCGACAAGAGATC	0.524038148	0.135016173	-0.500129424
ab_99547_AATGTTGCCGACACACCTGTAGCC	0.489745431	0.070625898	-0.326617317
ab_99547_AATGTTGCGGAGAACAACACAGAA	0.469184405	-0.023410941	-0.43284504
ab_99547_ACAAGCTAACGCTCGAGACTAGTA	0.432310144	0.106604969	-0.439230837

ab_99547_ACAAGCTAAGATCGCAAGCAGGAA	0.579531357	0.092791752	-0.38953354
ab_99547_ACACAGAAGAATCTGAACTATGCA	0.729128777	0.178756365	-0.502972166
ab_99547_ACACGACCACCTCCAAGCCAAGAC	0.340325334	-0.030875208	-0.42298301
ab_99547_ACACGACCACTATGCAAGATCGCA	0.354358433	0.110191895	-0.372784504
ab_99547_ACACGACCAGCCATGCAGTCACTA	0.393270727	0.129723329	-0.322652618
ab_99547_ACACGACCCGACTGGAACAGATTC	0.326479577	0.027752734	-0.504069251
ab_99547_ACAGATTCATTGAGGAAAGACGGA	0.62684575	0.002021064	-0.537632911
ab_99547_ACAGATTCCAACCACACGGATTGC	0.562507015	0.211206595	-0.237441436
ab_99547_ACAGCAGAGAGTTAGCTATCAGCA	0.659802076	0.105470619	-0.281416826
ab_99547_ACAGCAGATGGAACAATCCGTCTA	0.584741225	0.074999018	-0.401229518
ab_99547_ACATTGGCAGATGTACACACGACC	0.332611751	0.137899774	-0.332699152
ab_99547_ACATTGGCCCTCTATCCAATGGAA	0.388399583	-0.039206618	-0.372599846
ab_99547_ACATTGGCCGCTGATCAAGACGGA	0.598571769	0.063635705	-0.431159806
ab_99547_ACCACTGTAGATGTACCCTCCTGA	0.982615765	0.509962432	-0.433558185
ab_99547_ACCACTGTATCCTGTAATTGGCTC	0.262086434	0.119121308	-0.483071105
ab_99547_ACCACTGTCTGAGCCATCCGTCTA	0.577495107	0.008995393	-0.324039807
ab_99547_ACCACTGTGACTAGTAGATAGACA	0.304970273	0.108296974	-0.42291376
ab_99547_ACCACTGTGTACGCAATATCAGCA	0.465674323	0.058923382	-0.284920191
ab_99547_ACCTCCAACCGACAACAACGCTTA	0.614481767	0.226523473	-0.555704198
ab_99547_ACGCTCGAAACGTGATAACGTGAT	0.364308878	0.033956729	-0.393785762
ab_99547_ACGCTCGAAAGGACACGTACGCAA	0.546941314	0.109521268	-0.353074285
ab_99547_ACGCTCGACCAGTTCAAGATGTAC	0.678959624	-0.0573895	-0.426264778
ab_99547_ACGCTCGATCCGTCTAACAGATTC	0.395383094	0.162658255	-0.527412745
ab_99547_ACGTATCAAAGGACACAGATGTAC	0.510559697	0.182533354	-0.448617842
ab_99547_ACGTATCACATACCAATCTTCACA	0.450276339	0.093812317	-0.336118711
ab_99547_ACTATGCACAATGGAACGAACTTA	0.547989127	0.1679065	-0.220504825
ab_99547_ACTATGCACGCTGATCATTGAGGA	0.478540316	0.053161812	-0.446871253
ab_99547_ACTATGCAGAGCTGAAAAGACGGA	0.397302585	0.081606538	-0.474250461
ab_99547_ACTATGCAGCCAAGACGCCACATA	0.765385561	0.222229196	-0.616315045
ab_99547_ACTATGCATGGTGGTAACATTGGC	0.767772623	0.217358488	-0.431289599
ab_99547_AGAGTCAAACTATGCACGACACAC	0.594352807	0.036128463	-0.506011555
ab_99547_AGAGTCAACAACCACACGCATACA	0.513542555	-0.087768913	-0.631436401

ab_99547_AGAGTCAAGACAGTGCGAACAGGC	0.355629399	-0.063032491	-0.513428972
ab_99547_AGATCGCAATGCCTAACACCTTAC	0.768291194	-0.07851911	-0.410560546
ab_99547_AGATCGCATTCACGCAACACGACC	0.529601226	0.033912158	-0.428105769
ab_99547_AGATGTACAAACATCGCCGAAGTA	0.585612779	0.065852244	-0.523469749
ab_99547_AGATGTACAGAGTCAAACAGCAGA	0.530876368	0.034233765	-0.423660191
ab_99547_AGATGTACCGCATACAACACAGAA	0.277598483	-0.010506526	-0.312883541
ab_99547_AGATGTACGACTAGTATATCAGCA	0.657860637	-0.03426708	-0.562092983
ab_99547_AGCACCTCAACGCTTAGATAGACA	0.518893325	-0.058899015	-0.561264029
ab_99547_AGCAGGAAAACAACCACTAAGGTC	0.498042173	0.015696633	-0.442666781
ab_99547_AGCAGGAAACAGCAGACCGACAAC	0.286281955	0.078856472	-0.538300194
ab_99547_AGCAGGAACAAGACTACACCTTAC	0.586948282	0.073155763	-0.589821725
ab_99547_AGCAGGAATGGAACAAAACAACCA	0.815992137	0.116780007	-0.360293928
ab_99547_AGCCATGCACAGATTCGGAGAACA	0.590978064	0.073464605	-0.422504986
ab_99547_AGGCTAACACTATGCAAACGCTTA	0.804167858	0.258733677	-0.399874492
ab_99547_AGGCTAACATCATTCCAATGTTGC	0.6828104	0.211354129	-0.279884993
ab_99547_AGGCTAACCCATCCTCCTAAGGTC	0.727427668	0.371471353	-0.517775107
ab_99547_AGGCTAACTCCGTCTACCGACAAC	0.608522071	0.02324478	-0.342747796
ab_99547_AGGCTAACTTCACGCAACATTGGC	0.478799566	0.295461877	-0.615118596
ab_99547_AGTACAAGAACAACCAACATTGGC	0.591485032	0.119461029	-0.477184768
ab_99547_AGTACAAGACAGCAGATGGCTTCA	0.619552575	-0.047170216	-0.55086695
ab_99547_AGTACAAGAGTCACTAAGCAGGAA	0.596092148	0.073625367	-0.363196389
ab_99547_AGTACAAGGAACAGGCCATCAAGT	0.484530406	0.022405987	-0.535914318
ab_99547_AGTACAAGGCGAGTAACGACACAC	0.424484381	-0.015269405	-0.46674448
ab_99547_AGTCACTAGAACAGGCACGCTCGA	0.57672297	-0.034195129	-0.472131901
ab_99547_AGTGGTCAACAAGCTACATACCAA	0.552507531	0.070839459	-0.469929144
ab_99547_AGTGGTCAAGCCATGCCCGAAGTA	0.523499751	0.011183947	-0.557057802
ab_99547_AGTGGTCACAACCACACAAGGAGC	0.443946892	0.201115957	-0.408643099
ab_99547_ATAGCGACAAGGTACAGTCGTAGA	0.820348759	0.042710097	-0.418876994
ab_99547_ATAGCGACACAGATTCCGAACTTA	0.248492295	0.181565825	-0.466896028
ab_99547_ATAGCGACACGTATCAAACAACCA	0.499528969	0.112772653	-0.34053924
ab_99547_ATAGCGACCCTCCTGAAACAACCA	0.544154602	0.065370734	-0.40970386
ab_99547_ATAGCGACCTGAGCCAACGTATCA	0.572288481	0.124202474	-0.494097794

ab_99547_ATCATTCCAGGCTAACACGCTCGA	0.757794273	0.155848955	-0.611182252
ab_99547_ATCATTCCCGACTGGAGATGAATC	0.456027354	0.196006322	-0.433837097
ab_99547_ATCCTGTACAAGGAGCCGCTGATC	0.483782442	0.083658866	-0.341344477
ab_99547_ATCCTGTAGATAGACAAGAGTCAA	0.705129473	0.016799327	-0.533944697
ab_99547_ATCCTGTAGCTCGGTATGGCTTCA	0.275551399	0.06567066	-0.320054313
ab_99547_ATTGAGGAAAGAGATCAAACATCG	0.660988851	0.103348668	-0.395353894
ab_99547_ATTGAGGAACGTATCACACCTTAC	0.720090233	0.179189866	-0.595365918
ab_99547_ATTGAGGAAGTCACTAATCCTGTA	0.210379908	0.038233121	-0.45918332
ab_99547_ATTGAGGAATTGGCTCCAAGACTA	-0.068847946	0.011891156	-0.442174792
ab_99547_ATTGAGGACTGGCATACAATGGAA	0.51101835	0.173906975	-0.364756171
ab_99547_ATTGGCTCAACTCACCCAGCGTTA	0.735488553	0.183325152	-0.435105228
ab_99547_ATTGGCTCACAGCAGACATACCAA	0.653473071	0.012308946	-0.582584864
ab_99547_ATTGGCTCCATCAAGTCCTCTATC	0.351108427	0.026210253	-0.577210212
ab_99547_ATTGGCTCCGACACACCCTAATCC	0.497383864	0.164271602	-0.304348571
ab_99547_ATTGGCTCGTCTGTCAGCCACATA	0.703716228	0.220165973	-0.261163277
ab_99547_CAACCACAACCTCCAAGAGTTAGC	0.596455095	0.218515651	-0.493690163
ab_99547_CAACCACACATACCAAACAAGCTA	0.60428625	0.050485646	-0.528037294
ab_99547_CAAGACTAAATCCGTCCGACACAC	0.489901354	0.005785427	-0.415556258
ab_99547_CAAGGAGCCCGACAACGTGTTCTA	0.470236638	0.359484528	-0.582198078
ab_99547_CAAGGAGCGAGCTGAACCGACAAC	0.458377132	0.095055108	-0.464796498
ab_99547_CAATGGAAAAGGTACACAACCACA	0.571265437	-0.007811013	-0.402692643
ab_99547_CAATGGAAGCCACATAAGATGTAC	0.76129551	-0.027045218	-0.332015932
ab_99547_CAATGGAAGCTAACGAGAATCTGA	0.383232924	-0.097761814	-0.484306727
ab_99547_CACCTTACATCCTGTAATCCTGTA	0.615523329	0.174837794	-0.35723929
ab_99547_CACCTTACCCGTGAGACTCAATGA	0.573172525	0.133264099	-0.51289362
ab_99547_CACCTTACTCCGTCTATGGTGGTA	0.48924381	0.031113912	-0.447076352
ab_99547_CACTTCGAAACGCTTAGTCTGTCA	0.533289236	0.058018542	-0.430715061
ab_99547_CACTTCGAACATTGGCCAAGACTA	0.392817533	0.042450217	-0.371879365
ab_99547_CACTTCGAACGTATCACCAGTTCA	0.629054609	0.120984684	-0.514599551
ab_99547_CACTTCGACACCTTACGACTAGTA	0.89862413	0.31654556	-0.547120877
ab_99547_CACTTCGACCGACAACAATCCGTC	0.497785829	0.198489546	-0.277943888
ab_99547_CACTTCGATGGAACAACCTCTATC	0.65374134	-0.099931514	-0.378803774

ab_99547_CAGATCTGAAGACGGAAACCGAGA	0.324917002	0.063211229	-0.382860554
ab_99547_CAGATCTGGTCTGTCACGGATTGC	0.287181203	0.076454842	-0.370929457
ab_99547_CATACCAAACAGATTCAGATGTAC	0.13946438	-0.051108821	-0.433885954
ab_99547_CATACCAAACCTCCAAAGCACCTC	0.468770424	0.092531831	-0.607094103
ab_99547_CATACCAAACTATGCAACAGCAGA	1.020694129	0.28201461	-0.360056628
ab_99547_CATACCAACAATGGAACCGAAGTA	0.370529233	0.115213353	-0.331622256
ab_99547_CATACCAATCTTCACAACACAGAA	0.368282332	0.168296114	-0.363099186
ab_99547_CATCAAGTAGTGGTCACCGTGAGA	0.470758783	0.040329464	-0.343585002
ab_99547_CATCAAGTCGAACTTATAGGATGA	0.645564339	0.141130833	-0.361724578
ab_99547_CATCAAGTGCTAACGAGACAGTGC	0.578924835	0.110198206	-0.297081975
ab_99547_CCAGTTCAAAACATCGCATACCAA	0.346793089	0.040517564	-0.383516319
ab_99547_CCAGTTCAAACGCTTAACTATGCA	0.748934139	0.091095774	-0.547285452
ab_99547_CCAGTTCAAAGAGATCACGTATCA	0.483884305	0.178139628	-0.28428122
ab_99547_CCAGTTCAATAGCGACAATGTTGC	0.702695031	0.045102507	-0.564490798
ab_99547_CCAGTTCAGAATCTGACGGATTGC	0.613010622	-0.005020843	-0.488739311
ab_99547_CCATCCTCAGAGTCAAACAAGCTA	0.735983937	0.046120735	-0.597563686
ab_99547_CCATCCTCCTGAGCCACAAGGAGC	0.374444019	-0.074881294	-0.515944256
ab_99547_CCGAAGTAGACAGTGCGTGTTCTA	0.468510685	-0.082276445	-0.521014526
ab_99547_CCGAAGTAGGTGCGAAGAACAGGC	0.366973006	-0.130895656	-0.466931202
ab_99547_CCGAAGTAGTGTTCTAGTCGTAGA	0.16142733	0.031486179	-0.44618801
ab_99547_CCGACAACAATCCGTCAACAACCA	0.538084437	0.05929854	-0.405170237
ab_99547_CCGACAACCCTAATCCGATGAATC	0.732058859	0.407862307	-0.533696869
ab_99547_CCGACAACCGACACACACCTCCAA	0.296080728	-0.023414318	-0.352586168
ab_99547_CCGACAACTCTTCACAAGTGGTCA	0.460111504	-0.048773495	-0.477621393
ab_99547_CCGTGAGATCCGTCTAACAAGCTA	0.659051901	0.078679646	-0.556735147
ab_99547_CCTAATCCAACCGAGAGCCAAGAC	0.639976085	0.223465999	-0.359202075
ab_99547_CCTAATCCAAGAGATCGAGCTGAA	0.294332134	-0.005337335	-0.560083524
ab_99547_CCTAATCCCAAGACTAAACAACCA	0.633984921	0.136968622	-0.429615278
ab_99547_CCTAATCCCACCTTACAAGGTACA	0.815081059	0.187072097	-0.561397037
ab_99547_CCTAATCCCCGTGAGAAAGGACAC	0.550102575	0.078141759	-0.307532887
ab_99547_CCTCCTGAATAGCGACCTGTAGCC	0.471054641	0.021427319	-0.390852093
ab_99547_CCTCCTGACCGAAGTAACCTCCAA	0.558596021	0.026704029	-0.541302531

ab_99547_CCTCCTGAGTCTGTCAAGTACAAG	0.416015927	0.058966722	-0.513957896
ab_99547_CCTCTATCAACGTGATGCTCGGTA	0.03465777	0.020322723	-0.372615893
ab_99547_CCTCTATCACCTCCAACCGACAAC	0.676490664	0.004522706	-0.557951439
ab_99547_CCTCTATCCCAGTTCAGACTAGTA	0.647347815	0.073889654	-0.360778632
ab_99547_CCTCTATCTGAAGAGAGAGTTAGC	0.360060033	-0.04084691	-0.425585488
ab_99547_CGAACTTAAACAACCAGAGTTAGC	0.450052807	0.054379373	-0.514588064
ab_99547_CGAACTTATAGGATGAATTGGCTC	0.494338355	0.115635833	-0.568964095
ab_99547_CGAACTTATATCAGCAAGATGTAC	0.357941764	-0.000614337	-0.479067833
ab_99547_CGACACACCACTTCGAGCGAGTAA	0.818206163	0.10485849	-0.486211227
ab_99547_CGACACACCCGACAACTGGTGGTA	0.516939544	-0.004870605	-0.450789173
ab_99547_CGACACACGGTGCGAACAAGACTA	0.557498004	-0.066440861	-0.544985413
ab_99547_CGACTGGACAGATCTGAGCCATGC	0.409707742	0.095374292	-0.550246501
ab_99547_CGACTGGACATCAAGTGGAGAACA	0.425144468	0.101350898	-0.569648168
ab_99547_CGCATACAAAGGTACACCTAATCC	0.565055888	-0.032807475	-0.53938279
ab_99547_CGCATACAACGCTCGACAGCGTTA	0.376339545	0.131211224	-0.220145042
ab_99547_CGCATACAATTGGCTCCAGATCTG	0.283546412	0.049212326	-0.467697959
ab_99547_CGCATACACAACCACACTCAATGA	0.613648182	0.085187676	-0.444231773
ab_99547_CGCATACACCGTGAGATGGCTTCA	0.31477406	-0.104361709	-0.508739139
ab_99547_CGCATACAGATAGACAACACGACC	0.665678585	-0.031389987	-0.556255188
ab_99547_CGCTGATCAGGCTAACGTCGTAGA	0.423152271	0.190146663	-0.449933121
ab_99547_CGCTGATCCAAGGAGCGACAGTGC	0.50056124	0.135844111	-0.147146319
ab_99547_CGCTGATCGACAGTGCCCTCTATC	0.344282744	-0.087278597	-0.395323909
ab_99547_CGGATTGCAACCGAGAAACAACCA	0.597202086	0.172544056	-0.231132856
ab_99547_CGGATTGCGACTAGTAGTGTTCTA	0.400790163	0.011210513	-0.380327533
ab_99547_CTAAGGTCGATAGACAACAAGCTA	0.646694372	0.057198572	-0.376452514
ab_99547_CTAAGGTCGCCACATATTCACGCA	0.641331319	0.136935281	-0.493879868
ab_99547_CTAAGGTCTAGGATGATATCAGCA	0.436198146	0.050553653	-0.385651201
ab_99547_CTAAGGTCTGAAGAGAAAACATCG	0.381036507	0.300694569	-0.414455592
ab_99547_CTCAATGACTGAGCCAAGCAGGAA	0.840641534	0.1069798	-0.513419223
ab_99547_CTCAATGATATCAGCAGTCTGTCA	0.349610628	0.021317232	-0.474648736
ab_99547_CTGAGCCAAGCAGGAAATCCTGTA	0.469551951	-0.028708616	-0.490312177
ab_99547_CTGAGCCAATTGGCTCACACGACC	0.579227881	0.041569446	-0.388937505

ab_99547_CTGAGCCACACCTTACGGAGAACA	0.685263256	0.067736289	-0.437737171
ab_99547_CTGAGCCAGTCGTAGAAGATCGCA	0.652904792	0.094533148	-0.273587115
ab_99547_CTGAGCCATAGGATGAAACCGAGA	0.141482578	0.155718767	-0.301347053
ab_99547_CTGAGCCATCTTCACAAAACATCG	0.562921533	0.040540713	-0.488191002
ab_99547_CTGGCATAGAGCTGAAAATGTTGC	0.505153963	0.050733699	-0.331156801
ab_99547_CTGGCATATCCGTCTAGCTAACGA	0.607185601	0.031319264	-0.408291393
ab_99547_CTGTAGCCATCATTCCACGCTCGA	0.564023665	0.236018431	-0.6106661
ab_99547_CTGTAGCCATCATTCCTGGAACAA	0.642656555	0.162603134	-0.183728116
ab_99547_CTGTAGCCATGCCTAACATCAAGT	0.460862495	0.192226294	-0.511522744
ab_99547_CTGTAGCCCAATGGAACTGTAGCC	0.323029992	-0.005447709	-0.441141254
ab_99547_GAACAGGCACACAGAAATTGAGGA	0.865259326	0.069985356	-0.541775857
ab_99547_GAACAGGCCCGACAACTGGTGGTA	0.394465136	-0.013010294	-0.267412818
ab_99547_GAATCTGAAATCCGTCAAGACGGA	0.507819044	0.305100423	-0.258291433
ab_99547_GAATCTGACTGTAGCCAAGGACAC	0.497958374	0.052306484	-0.419916929
ab_99547_GAATCTGATGGTGGTACACTTCGA	0.309698707	-0.169698849	-0.552155334
ab_99547_GACAGTGCGCCACATAGTCTGTCA	0.57197234	0.328318724	-0.375469355
ab_99547_GACAGTGCTATCAGCACTAAGGTC	0.701167575	0.02948469	-0.574178454
ab_99547_GACTAGTATGGTGGTAATGCCTAA	0.412704363	0.137469787	-0.53513286
ab_99547_GAGCTGAAAACCGAGACCGTGAGA	0.395415811	0.002451615	-0.601678413
ab_99547_GAGCTGAAAAGGACACGACAGTGC	0.444610994	0.048803957	-0.438094246
ab_99547_GAGCTGAACCGTGAGACGCTGATC	0.567549144	0.070384505	-0.494957837
ab_99547_GAGTTAGCACCACTGTCCAGTTCA	0.490537824	0.152823505	-0.475237777
ab_99547_GAGTTAGCGAGCTGAACCAGTTCA	0.541928605	0.025263232	-0.492635924
ab_99547_GAGTTAGCGATGAATCAAACATCG	0.846843527	0.227835955	-0.293863814
ab_99547_GATAGACAAGATCGCAATCCTGTA	0.358767813	-0.014316946	-0.428081453
ab_99547_GATGAATCCGACTGGAACCACTGT	0.334376198	0.023239101	-0.18372619
ab_99547_GATGAATCGACAGTGCAACGTGAT	0.65901385	0.060120128	-0.472613513
ab_99547_GCCAAGACAACGTGATATAGCGAC	0.276951975	-0.06297334	-0.422867793
ab_99547_GCCAAGACACAGATTCCCATCCTC	0.399246176	0.240120785	-0.240619145
ab_99547_GCCAAGACCAACCACAAACCGAGA	0.779040586	0.183627501	-0.57200045
ab_99547_GCCAAGACCCGACAACACACGACC	0.365118698	0.084635053	-0.296667741
ab_99547_GCCAAGACTGAAGAGAAAACATCG	0.410152125	0.044833377	-0.451271174

ab_99547_GCCACATAAAGGACACGACAGTGC	0.58425457	0.026588051	-0.404814501
ab_99547_GCCACATAATTGGCTCAACAACCA	0.508811	0.154141059	-0.28431379
ab_99547_GCCACATACGACACACACGTATCA	0.301655457	0.096127057	-0.467999868
ab_99547_GCCACATATAGGATGATATCAGCA	0.657656581	0.089880758	-0.405698858
ab_99547_GCCACATATGGCTTCACCGACAAC	0.581063821	0.049007377	-0.444716644
ab_99547_GCCACATATGGTGGTAATCCTGTA	0.471730148	0.096654928	-0.430283565
ab_99547_GCGAGTAAAGCCATGCAAGGACAC	0.797889691	0.111014037	-0.36418166
ab_99547_GCTAACGAAAGGACACACAAGCTA	0.797887741	0.146816961	-0.411846281
ab_99547_GCTAACGAAGCAGGAACTCAATGA	0.487068311	0.11630042	-0.38679981
ab_99547_GCTCGGTAACAGCAGAGTGTTCTA	0.35621278	-0.139044713	-0.53471519
ab_99547_GCTCGGTAAGATGTACCACCTTAC	0.623377933	0.197351144	-0.252379142
ab_99547_GCTCGGTAAGCAGGAACAGCGTTA	0.439777765	0.145135534	-0.435293219
ab_99547_GCTCGGTAATAGCGACGCCAAGAC	0.184986063	0.1271157	-0.314812138
ab_99547_GCTCGGTACGCATACAGAATCTGA	0.325597288	0.139219461	-0.259743251
ab_99547_GCTCGGTATATCAGCAAACTCACC	0.687220537	0.155401982	-0.348435498
ab_99547_GGAGAACAAGCCATGCTTCACGCA	0.752559554	0.009255867	-0.487139484
ab_99547_GGAGAACACAAGACTAAACGTGAT	0.551258025	0.130221383	-0.190988269
ab_99547_GGAGAACACCTCTATCCCAGTTCA	0.421771111	0.123742104	-0.522482718
ab_99547_GGAGAACACCTCTATCCGGATTGC	0.394695691	0.226243584	-0.398052463
ab_99547_GGAGAACACGCTGATCCCGAAGTA	0.44241568	-0.093636894	-0.487423764
ab_99547_GGAGAACAGATGAATCCCTCCTGA	0.47578561	0.005520786	-0.497894698
ab_99547_GGTGCGAAAAGAGATCCTAAGGTC	0.506004085	0.130718413	-0.34050687
ab_99547_GGTGCGAACATACCAATGGTGGTA	0.574265296	0.089375048	-0.342843808
ab_99547_GTACGCAAAGATGTACATGCCTAA	0.244861418	-0.094128704	-0.532681048
ab_99547_GTACGCAAGACTAGTAACCTCCAA	0.366759338	0.140537905	-0.462072292
ab_99547_GTACGCAAGCGAGTAACTGGCATA	0.073857937	-0.085988478	-0.41518449
ab_99547_GTCGTAGACGCTGATCAGTGGTCA	0.631879766	0.137761176	-0.319511452
ab_99547_GTCTGTCAGGAGAACACAAGGAGC	0.290790332	0.057420708	-0.486959678
ab_99547_GTGTTCTACGGATTGCAGATCGCA	0.641483877	0.028862316	-0.504457287
ab_99547_TAGGATGAACACGACCCAATGGAA	0.433852129	0.010954746	-0.144069598
ab_99547_TAGGATGAAGTACAAGGAATCTGA	0.213955331	0.15223038	-0.423490558
ab_99547_TATCAGCAAATGTTGCATGCCTAA	0.594623287	0.103327215	-0.383164812

ab_99547_TATCAGCAACCACTGTCCGAAGTA	0.637413868	0.224148663	-0.239086107
ab_99547_TATCAGCATCTTCACACCATCCTC	0.761425235	0.167547106	-0.529555468
ab_99547_TCCGTCTAAACCGAGATGAAGAGA	0.410265504	0.172448184	-0.186747178
ab_99547_TCCGTCTAACAAGCTACAATGGAA	0.694811198	0.198576679	-0.353507078
ab_99547_TCCGTCTAACCACTGTGATAGACA	0.504447338	0.117865874	-0.618428676
ab_99547_TCCGTCTACCGAAGTACAGCGTTA	0.345706751	-0.09080892	-0.410821902
ab_99547_TCCGTCTAGAGTTAGCAGATCGCA	0.703706331	0.137622799	-0.40651528
ab_99547_TCCGTCTATGAAGAGAACACAGAA	0.582594098	0.265676492	-0.199944539
ab_99547_TCTTCACAAACTCACCACAGCAGA	0.525032414	0.103507874	-0.311043703
ab_99547_TCTTCACAACAAGCTAAACCGAGA	0.524447625	0.135263634	-0.468420656
ab_99547_TCTTCACACAGCGTTAGAATCTGA	0.659848823	0.178844386	-0.417434419
ab_99547_TGAAGAGACAACCACACAATGGAA	0.426555277	0.083016579	-0.308093559
ab_99547_TGGAACAAAAGACGGATTCACGCA	0.855051844	0.064306087	-0.303374236
ab_99547_TGGAACAAGATAGACAAGCAGGAA	0.501553026	0.100213772	-0.517313644
ab_99547_TGGCTTCAAGTGGTCACTCAATGA	0.508755788	-0.010742381	-0.348277013
ab_99547_TGGCTTCAGCCACATACCGTGAGA	0.497321689	-0.025403097	-0.574050604
ab_99547_TGGTGGTACTAAGGTCAGAGTCAA	0.285563129	0.224223255	-0.256576955
ab_99547_AAACATCGATGCCTAAACAAGCTA	0.527470863	0.107851129	-0.438009231
ab_99547_AACCGAGAGGTGCGAAACATTGGC	0.535604392	0.016818616	-0.548235587
ab_99547_ACAAGCTATGAAGAGAACACGACC	0.573734362	0.09707818	-0.418525857
ab_99547_AGTGGTCAAAGACGGAAGAGTCAA	0.49257028	0.130832506	-0.566134991
ab_99547_ATAGCGACGAGTTAGCACCTCCAA	0.681777096	0.094692084	-0.53317273
ab_99547_CAAGGAGCAGATCGCAAAGAGATC	0.844141526	0.080827585	-0.556502464
ab_99547_CACTTCGACCGTGAGACGGATTGC	0.66621952	0.152190203	-0.424299026
ab_99547_CAGCGTTAGCTCGGTAGCTCGGTA	0.552136193	0.142038883	-0.465622727
ab_99547_CCGACAACGCTAACGATGAAGAGA	0.652700293	0.063868619	-0.252581107
ab_99547_CTAAGGTCAACCGAGATGGAACAA	0.282758042	0.109980065	-0.377941735
ab_99547_GAATCTGAAGTACAAGATAGCGAC	0.60122768	0.040537309	-0.495244874
ab_99547_GAGTTAGCACGTATCATATCAGCA	0.547678839	0.220767292	-0.448950329
ab_99547_GATGAATCAACAACCAATCCTGTA	0.634797687	0.159211665	-0.495752641
ab_99547_GCTCGGTAAAACATCGAGATCGCA	0.543115266	0.090555073	-0.473121544
ab_99547_GTCTGTCAAGTCACTAAGATCGCA	0.614868151	0.170403941	-0.438819677

ab_99547_GACTAGTAAGATGTACGAGTTAGC	0.65133567	0.114615762	-0.285303731
A_98763_ATAGCGACAAGAGATCACACGACC	0.892022212	0.547217472	1.304760585
A_98763_GAACAGGCGTACGCAACGCATACA	0.366478604	0.216494135	0.96545489
A_98763_GACTAGTAATCCTGTACCTCCTGA	0.471133976	0.191456697	0.015860378
A_98763_AAGAGATCGGAGAACAAGATGTAC	0.720123256	0.970994822	1.462442276
A_98763_AAGGACACAGAGTCAACGAACTTA	0.334470995	0.270158074	0.342315935
A_98763_AAGGACACCCGACAACCGAACTTA	0.801857378	0.713032618	1.22082116
A_98763_AAGGTACACAATGGAAAACGCTTA	0.655557161	0.420734006	0.929839611
A_98763_AAGGTACACATCAAGTCACTTCGA	0.460105361	0.431574571	0.913862345
A_98763_ACAGATTCACCTCCAAGCTAACGA	0.915509721	0.786964127	1.002008668
A_98763_ACATTGGCAAGAGATCGGAGAACA	0.67377364	0.835974925	0.870586906
A_98763_ACATTGGCAAGGACACCACTTCGA	0.807460335	0.277301871	0.533611509
A_98763_ACGCTCGACCATCCTCACACAGAA	0.647648921	0.684708511	1.160011615
A_98763_AGATCGCAAGATCGCACTGGCATA	0.714563213	0.259669981	0.962901979
A_98763_AGATCGCAAGTCACTAGACAGTGC	0.677546086	0.59444881	1.004633178
A_98763_AGATCGCACACCTTACATCCTGTA	0.782010934	0.452767226	0.935397532
A_98763_AGATGTACCAGATCTGAGTGGTCA	0.557742758	0.293654715	0.77189087
A_98763_AGGCTAACATCCTGTACCGAAGTA	0.709065593	0.71160027	0.791165476
A_98763_AGTACAAGACCACTGTACGTATCA	0.218258673	0.635097359	0.740284938
A_98763_AGTACAAGCGACACACCCTAATCC	0.309746327	0.348164589	0.727305248
A_98763_AGTGGTCACTCAATGACAGCGTTA	0.448613086	0.228388015	0.961199446
A_98763_ATAGCGACGCCACATACCTCCTGA	0.549286596	0.371351118	1.080039842
A_98763_ATTGGCTCTGGCTTCAAAGGACAC	0.289754211	0.038844215	0.383936333
A_98763_CAGCGTTAACTATGCAAACTCACC	0.905383634	0.355836232	0.461716524
A_98763_CATCAAGTAACAACCAGAACAGGC	0.482532843	0.484142381	0.682688196
A_98763_CCATCCTCGACAGTGCATTGGCTC	0.189779947	0.174078451	0.186798578
A_98763_CCGAAGTAAACCGAGAACACAGAA	0.51770675	0.326882819	0.719818618
A_98763_CCGAAGTACAAGACTAACACGACC	0.599482837	0.395964199	0.408475892
A_98763_CCGTGAGACCTCCTGACAGATCTG	0.846138355	0.810658736	1.03259762
A_98763_CGAACTTAGAACAGGCTATCAGCA	0.422794203	0.193192874	0.633497187
A_98763_CGACACACGTACGCAACCTCTATC	0.711690222	0.350621763	0.736532695
A_98763_CGCATACAAACGCTTAGTCGTAGA	0.808450472	0.892828552	0.932152025

A_98763_CGGATTGCACGTATCAGTACGCAA	0.944684943	0.824115309	0.650918909
A_98763_CTGTAGCCCATACCAAAAACATCG	0.850701889	0.556378153	0.755315142
A_98763_CTGTAGCCGCTCGGTAGTCTGTCA	0.449926491	0.118966108	0.589612664
A_98763_GAATCTGAGAATCTGAATCATTCC	0.814930932	0.406163919	0.731681295
A_98763_GAGTTAGCACCTCCAACCTAATCC	0.759164348	0.492039692	0.86528897
A_98763_GAGTTAGCCACCTTACTGAAGAGA	0.479585679	0.10802876	0.473979779
A_98763_GATAGACACGACTGGACCGAAGTA	0.665402337	0.257205237	0.897013598
A_98763_GATGAATCTGGCTTCAATCATTCC	0.618399812	0.356145863	0.863608311
A_98763_GCCACATACCATCCTCCTAAGGTC	0.534279186	0.421101089	1.607131232
A_98763_GCGAGTAAATTGAGGACAGCGTTA	0.854187977	0.411144613	0.496225994
A_98763_GCTCGGTACCTCTATCATGCCTAA	0.762729067	0.34953786	0.948666152
A_98763_GGAGAACAACAGATTCCCTAATCC	0.760280282	0.260619835	0.880070413
A_98763_AAGACGGAAACAACCAGTCTGTCA	1.383656798	0.989590649	1.401664015
A_98763_AAGAGATCCAAGGAGCCAACCACA	0.347437082	1.877300302	2.259084954
A_98763_AAGGTACAAACGCTTAGAGTTAGC	0.896546981	1.43371078	1.469764915
A_98763_AAGGTACAAATGTTGCTCCGTCTA	1.02340655	1.572505229	1.31355941
A_98763_AATCCGTCACGCTCGAGTACGCAA	0.814789642	1.176324982	0.858559736
A_98763_AATCCGTCCCTCCTGACGCATACA	1.17566635	1.477089126	1.095154374
A_98763_ACACGACCGTCGTAGAATTGAGGA	0.832072903	1.188986164	1.18512156
A_98763_AGATGTACACACGACCAACCGAGA	0.983822747	1.409111419	1.43908919
A_98763_AGGCTAACATGCCTAAATTGGCTC	1.016726829	1.60561715	1.610693318
A_98763_AGTGGTCAACACAGAACCGACAAC	0.812270076	0.768483387	0.626703626
A_98763_AGTGGTCACCTCCTGAACACAGAA	1.061137739	1.318173801	1.218860933
A_98763_AGTGGTCACGCTGATCCTCAATGA	0.862495868	1.173531972	1.131937299
A_98763_ATAGCGACAGATGTACAAACATCG	0.718172128	1.338253576	1.387036996
A_98763_ATCCTGTACCGACAACGACAGTGC	0.771797487	1.222932661	1.432659228
A_98763_ATGCCTAACTAAGGTCGCGAGTAA	1.08096266	1.213752347	1.342107074
A_98763_ATTGAGGATGGAACAACCTAATCC	1.151857629	1.446633908	1.433825601
A_98763_CAACCACAACTATGCAATGCCTAA	0.758663051	1.776676052	2.113125235
A_98763_CAACCACACCTCCTGAAACGTGAT	0.775336584	1.334470702	1.648814976
A_98763_CAACCACAGCTAACGACTGGCATA	0.504048889	1.294820089	1.639349612
A_98763_CAAGACTAACAGATTCATCATTCC	0.933426899	0.857552486	0.600900928

A_98763_CAAGACTACAGATCTGAGATGTAC	0.832070366	1.105589397	0.467893942
A_98763_CAAGGAGCCAACCACAAAGACGGA	0.478237475	1.112912753	1.97963872
A_98763_CAATGGAAAGATGTACAGATCGCA	0.805594451	0.969422684	1.415628593
A_98763_CATACCAAACTATGCAGGAGAACA	1.114925422	1.06952672	1.553265979
A_98763_CCGTGAGAGGAGAACAGCTCGGTA	1.12512376	1.122551952	1.429743526
A_98763_CCTAATCCGCCACATACGCTGATC	1.414238099	2.052776361	1.626568885
A_98763_CCTAATCCTGGTGGTAGCTCGGTA	0.909696135	1.505855482	1.142352391
A_98763_CCTCTATCGAACAGGCACACAGAA	1.173762707	1.783057722	1.027097857
A_98763_CCTCTATCTGAAGAGAGAGTTAGC	0.725505636	1.360757965	1.431155798
A_98763_CGCTGATCAGCCATGCCAACCACA	0.622983499	1.527634159	1.815803214
A_98763_CGGATTGCAGTGGTCAGCTAACGA	1.242924362	1.600195664	1.804927599
A_98763_CTAAGGTCCGAACTTACTGAGCCA	0.846256885	1.192464646	1.408071051
A_98763_CTGAGCCACATACCAATGGCTTCA	1.058380798	1.236771406	0.897352515
A_98763_CTGAGCCACTGAGCCAAGCACCTC	0.823945651	1.562778778	1.700843938
A_98763_CTGGCATAGATGAATCAGATCGCA	1.02246199	1.254800555	1.742562991
A_98763_CTGTAGCCAATGTTGCCAGATCTG	1.347715792	1.43625941	1.616418061
A_98763_CTGTAGCCAGCCATGCTGAAGAGA	0.382353457	0.780548058	0.941150727
A_98763_GAATCTGAACACAGAACCTCTATC	1.044866461	1.892902944	1.363363409
A_98763_GAATCTGAATTGAGGAACAAGCTA	0.753299442	1.122735372	2.039959488
A_98763_GAATCTGACCTAATCCACAGATTC	0.664805615	1.016144602	0.59139467
A_98763_GACTAGTAGCTAACGACGCTGATC	0.478567686	1.91116474	2.194382203
A_98763_GAGCTGAACAGATCTGAGTGGTCA	0.868348244	1.617223593	1.536031185
A_98763_GAGTTAGCCCTAATCCACACGACC	0.854371926	1.124071677	0.770407839
A_98763_GAGTTAGCGCCAAGACCTAAGGTC	0.850082752	1.123123207	0.912300967
A_98763_GATAGACAACCACTGTTGGCTTCA	1.232821376	1.207050831	1.327776074
A_98763_GATAGACACCAGTTCAAACTCACC	0.647368341	1.454097908	1.471717643
A_98763_GATGAATCACATTGGCAAGACGGA	0.474209798	1.278505098	1.218899407
A_98763_GATGAATCACATTGGCGTACGCAA	0.752601678	1.326124563	1.1805606
A_98763_GGTGCGAAAACCGAGAACACAGAA	1.164592561	1.173218877	1.76117848
A_98763_GTGTTCTAACACAGAATATCAGCA	0.73020117	1.441505294	1.450867036
A_98763_TCTTCACACCTCTATCCAGATCTG	0.776474609	1.234290502	1.565333808
A_98763_TGGAACAAACCTCCAAAGTCACTA	0.742359349	0.800268953	1.580221085

A_98763_TGGAACAAAGGCTAACAACCGAGA	0.635444253	1.381678412	1.498197545
A_98763_TGGCTTCACATACCAAAGATCGCA	1.289465897	1.823557612	1.641754102
A_98763_TGGTGGTACTCAATGACCGACAAC	0.967225893	1.352480537	1.351901537
A_98763_TTCACGCACATCAAGTCAATGGAA	0.916716103	1.318989162	0.845123223
A_98763_TTCACGCATGAAGAGACCGTGAGA	1.381728211	1.847085346	1.376265611
A_98763_ACAGATTCACAAGCTAACCTCCAA	0.581669334	0.498848819	0.50700935
A_98763_AAACATCGACACAGAATGGAACAA	1.055395491	1.715477622	1.532172113
A_98763_AACAACCAGACTAGTACGACTGGA	1.45098038	2.250760579	1.64674242
A_98763_AACCGAGAGTACGCAACCTCCTGA	1.291434666	1.879997314	1.356039385
A_98763_AGAGTCAAGAACAGGCGGTGCGAA	0.979114237	1.823347242	1.12997149
A_98763_AGAGTCAAGCCACATAGGTGCGAA	0.942550867	1.624973324	1.595164963
A_98763_AGGCTAACGACTAGTAAGATGTAC	0.904834087	1.807539214	1.811201542
A_98763_AGTCACTAACCACTGTAACAACCA	1.551547321	1.619234028	2.120521541
A_98763_CAGATCTGCGCATACATGGTGGTA	1.583221388	2.066052873	2.059645652
A_98763_CGGATTGCGGAGAACAAAACATCG	1.0532628	1.915460182	1.917655429
A_98763_GAGTTAGCACACAGAATGGTGGTA	0.805449189	1.835727099	1.821720277
A_98763_GATGAATCAGCCATGCACCTCCAA	0.657193083	1.736576265	1.888834111
A_98763_TGGAACAAAATGTTGCCAGATCTG	1.235770979	2.067868182	1.651523327
A_98763_TGGAACAAAATGTTGCCCGACAAC	1.205136569	2.009805374	1.902262421
A_98763_AAGAGATCTAGGATGAGCTCGGTA	1.158500354	0.665825654	0.7854868
A_98763_ACAGCAGAGTACGCAACTCAATGA	0.957234837	0.89053865	1.199787011
A_98763_ACCACTGTAGTCACTAAACTCACC	0.673626499	1.066704887	1.251331416
A_98763_ACGCTCGACGGATTGCTCTTCACA	0.726166485	0.74368971	1.106801119
A_98763_AGAGTCAACGACTGGACGAACTTA	0.672431566	1.053567642	1.445736935
A_98763_AGATCGCAAGTACAAGCGCATACA	0.787824568	0.792875588	1.127696888
A_98763_AGGCTAACAAGACGGAACCTCCAA	1.030289693	0.836380313	1.364022182
A_98763_AGTACAAGAACTCACCGACTAGTA	1.280561225	0.998404802	1.244173986
A_98763_CAACCACAAACAACCAACGCTCGA	0.97513049	0.572229665	1.163523734
A_98763_CACTTCGACTGGCATAAATGTTGC	0.967324617	1.277533297	1.714260274
A_98763_CTGAGCCAACACGACCAGCACCTC	0.625954954	1.005266343	1.02009556
A_98763_GACAGTGCACCACTGTGCCACATA	0.968609673	1.36019836	1.117678666
A_98763_GCTAACGACATCAAGTACATTGGC	1.027929709	0.837512261	1.347908931

A_98763_GCTCGGTACAATGGAAAGATGTAC	0.904518698	0.810729164	0.769658184
A_98763_TGAAGAGACGACTGGAAGTGGTCA	0.913568663	0.832804447	1.173265553
A_98763_AAACATCGACAGCAGACGCATACA	0.669540988	0.42448254	1.677116095
A_98763_AAACATCGGTACGCAACCTAATCC	0.608440484	0.588144845	1.097899717
A_98763_AACAACCACCAGTTCACCATCCTC	1.072459106	0.769183284	1.071154197
A_98763_AACAACCAGTCTGTCAACATTGGC	0.872811385	0.463721774	1.238366497
A_98763_AACCGAGAAGCCATGCATGCCTAA	0.381354829	0.154425736	0.872743846
A_98763_AACCGAGAAGTCACTAACATTGGC	0.340675028	0.421210564	1.476290384
A_98763_AACCGAGAGGTGCGAACAGCGTTA	1.082022738	0.744164205	1.093002758
A_98763_AACGCTTACTGTAGCCATCATTCC	0.451931269	0.412556367	0.610289423
A_98763_AACGTGATCGCTGATCACACGACC	0.539943063	0.684915903	1.578050012
A_98763_AACGTGATGACTAGTAGTACGCAA	0.306497568	0.385044364	1.328213433
A_98763_AACTCACCAGCCATGCATCCTGTA	0.409732816	0.43180899	1.936334336
A_98763_AACTCACCCGGATTGCTCCGTCTA	0.419514974	0.667782839	1.074055494
A_98763_AACTCACCGAACAGGCTTCACGCA	0.602908807	0.652187432	1.330384261
A_98763_AAGACGGAGGTGCGAAACACGACC	0.646363122	0.629240022	1.359195865
A_98763_AAGGACACACAAGCTAAACGTGAT	0.366461075	0.445208065	1.360461374
A_98763_AAGGACACGCGAGTAACCTCTATC	0.550042444	0.521370608	1.55674901
A_98763_AATCCGTCGATGAATCGCGAGTAA	0.742344882	0.218551172	0.495022693
A_98763_AATCCGTCTGAAGAGACGCATACA	0.229264756	0.395917394	1.281643875
A_98763_ACACAGAACGCTGATCTGGCTTCA	0.813459705	0.667240139	1.493529174
A_98763_ACACGACCAGAGTCAAAGCACCTC	0.439968729	0.432333442	0.929220237
A_98763_ACAGATTCCAATGGAACGCTGATC	0.325231042	0.317517508	0.742912606
A_98763_ACAGATTCCACTTCGAAATGTTGC	0.571540383	0.202245268	1.653128271
A_98763_ACATTGGCCAATGGAACAATGGAA	0.519306118	0.490468302	0.998654908
A_98763_ACATTGGCCCAGTTCAAACTCACC	0.499144079	0.181756357	0.955641007
A_98763_ACATTGGCCGCATACACGCTGATC	0.733765809	0.675987604	1.76506493
A_98763_AGATCGCACCAGTTCAACAGATTC	0.664536449	0.699960361	0.924146275
A_98763_AGATGTACCCAGTTCAATTGAGGA	0.496119919	0.420671575	0.993386367
A_98763_AGATGTACGAGCTGAAAAGACGGA	0.548536215	0.439637549	0.863393483
A_98763_AGATGTACGCCAAGACATTGAGGA	0.775865853	0.615111757	1.184007892
A_98763_AGCACCTCCTCAATGACTGAGCCA	0.637821477	0.295671932	1.121035333

A_98763_AGCCATGCCAAGGAGCAGATGTAC	0.443655815	0.53137257	1.527229483
A_98763_AGGCTAACCAACCACAAACTCACC	0.421319621	0.75987749	2.077456684
A_98763_AGTGGTCAGCTAACGAAAGAGATC	0.418877888	0.4145829	1.5439387
A_98763_AGTGGTCATGGTGGTAAATGTTGC	0.913574482	0.778662444	1.354800664
A_98763_ATAGCGACAACGCTTACATCAAGT	1.104207272	0.904630087	1.191358732
A_98763_ATAGCGACCGACTGGATGGCTTCA	0.869795519	0.548772912	1.142018133
A_98763_ATAGCGACGACTAGTACAGCGTTA	0.762775554	0.438258516	0.728422886
A_98763_ATCATTCCACAAGCTAACAGATTC	0.217709438	0.33248224	1.462164668
A_98763_ATCATTCCCAAGGAGCCCGTGAGA	0.469331301	0.382225244	0.878181977
A_98763_ATCCTGTAAAACATCGCACTTCGA	0.925814938	0.51903018	1.097658321
A_98763_ATGCCTAACCGACAACAGTCACTA	0.504954602	0.587315515	1.012928418
A_98763_ATGCCTAAGATAGACAGCTAACGA	0.594754675	0.666227229	1.915098993
A_98763_ATTGAGGATCCGTCTAAGATGTAC	0.687069571	0.922028572	1.336286036
A_98763_CAACCACAGCGAGTAAACGTATCA	0.703818839	0.584256148	0.889128693
A_98763_CAAGACTAAAGGACACAGTGGTCA	0.79559739	0.581187592	1.430293036
A_98763_CAAGACTAAGCACCTCCGCATACA	0.139467397	0.103383163	0.785820451
A_98763_CAAGACTATATCAGCAACAAGCTA	0.568286235	0.812372433	1.292078201
A_98763_CAAGGAGCACATTGGCCGGATTGC	0.849524015	0.949987977	1.387598979
A_98763_CAAGGAGCACTATGCATGGTGGTA	0.701847341	1.092366198	1.315222868
A_98763_CACCTTACAATGTTGCAACGTGAT	0.189644952	0.473014654	0.888521367
A_98763_CACCTTACACAGATTCCGGATTGC	0.168614529	0.287519046	1.207828908
A_98763_CACTTCGACCGAAGTAAGGCTAAC	0.404359466	0.081344436	0.883663362
A_98763_CACTTCGACTCAATGATTCACGCA	0.743144571	1.038114357	1.506338469
A_98763_CAGATCTGCTGGCATAGCTAACGA	0.736631467	0.673335393	1.79066813
A_98763_CAGCGTTATGAAGAGAAGTCACTA	0.805614379	0.464672729	1.202773575
A_98763_CAGCGTTATGAAGAGAGTCGTAGA	0.543872457	0.215761296	0.953456147
A_98763_CATACCAAAACGTGATATTGAGGA	0.437811709	0.210088576	0.636112462
A_98763_CATACCAACGCTGATCCAACCACA	0.806184898	0.72611309	1.409745226
A_98763_CATCAAGTAGTCACTACTGAGCCA	1.068071547	0.29582445	0.977190336
A_98763_CATCAAGTGCTAACGACAGATCTG	0.447156146	0.629489354	1.227205876
A_98763_CCATCCTCGAATCTGAACCACTGT	0.728122279	0.465066304	1.064543629
A_98763_CCATCCTCGTGTTCTAATGCCTAA	0.244592943	0.454589809	1.483321742

A_98763_CCGAAGTAAGATGTACGTACGCAA	0.264497075	0.223997421	1.218013266
A_98763_CCGAAGTACGCTGATCACACAGAA	0.796524146	0.816026938	1.183042117
A_98763_CCGACAACGAACAGGCACGCTCGA	0.502228073	0.222970994	1.276839655
A_98763_CCGTGAGAGCCACATAGACTAGTA	0.214171769	0.388314103	1.354384711
A_98763_CCGTGAGAGTACGCAACAGATCTG	0.659263738	0.518034399	1.662240924
A_98763_CCTCTATCGCTCGGTACTCAATGA	0.838935886	0.953674355	1.233468727
A_98763_CGAACTTAGACAGTGCAGCAGGAA	0.656368227	0.393689355	1.02657963
A_98763_CGACACACCCTCCTGAACTATGCA	1.142058994	0.952009018	2.028827037
A_98763_CGACACACGAACAGGCAAGAGATC	0.478408931	0.985628645	2.300422673
A_98763_CGACACACGCCACATATGAAGAGA	0.569254622	0.576680739	1.149999978
A_98763_CGACACACTGGCTTCACATCAAGT	0.330199089	0.385531636	1.303629957
A_98763_CGACTGGACGGATTGCCTGGCATA	0.149091686	0.323505206	1.586863293
A_98763_CGCATACAAAGAGATCCAACCACA	0.537763697	0.516972653	1.493135284
A_98763_CGCTGATCAATGTTGCAAGACGGA	0.803614145	0.576911126	1.537185232
A_98763_CGCTGATCCGAACTTAGAACAGGC	0.463878944	0.330163829	0.902066393
A_98763_CGGATTGCAGCCATGCCCGTGAGA	0.456570133	0.248473968	1.329217737
A_98763_CTAAGGTCAAACATCGGAACAGGC	0.524733616	0.578310268	1.205368695
A_98763_CTGGCATATGGCTTCAAACGTGAT	0.838177238	0.543422929	0.975880587
A_98763_GAATCTGAAACGCTTAGTCGTAGA	0.370234082	0.391752312	1.251652718
A_98763_GACAGTGCACCTCCAAACAAGCTA	0.321926094	0.253269521	1.203828839
A_98763_GACAGTGCTGGCTTCAAGATGTAC	0.174124528	0.488754061	1.591958461
A_98763_GACTAGTAACAAGCTATATCAGCA	1.055369378	0.502405938	1.180601607
A_98763_GACTAGTACCTCCTGATATCAGCA	0.82752538	0.742207128	1.053127635
A_98763_GAGCTGAAACAAGCTAGTCGTAGA	0.383681362	0.244516446	0.916317036
A_98763_GAGTTAGCGTCGTAGAACAAGCTA	0.733922977	0.975964341	1.080816424
A_98763_GATAGACACCGACAACACGCTCGA	0.619165369	1.001811737	1.053788971
A_98763_GATAGACACGCATACACAGATCTG	0.570047239	1.095534906	1.799469521
A_98763_GATGAATCACAGATTCAGATGTAC	0.849156077	0.64461403	1.340332757
A_98763_GATGAATCACATTGGCCGCTGATC	0.680881992	0.523962077	1.250611374
A_98763_GATGAATCCTGGCATAACAAGCTA	0.509199734	0.727254759	1.73133578
A_98763_GCCAAGACAGATCGCAAGTCACTA	0.394575136	0.30747381	1.741238123
A_98763_GCCAAGACATCCTGTAATTGGCTC	0.670638104	0.790285841	1.312688181

A_98763_GCCAAGACCATACCAAGCTCGGTA	0.405674636	0.327989498	1.013658941
A_98763_GCCAAGACGCCAAGACAGCCATGC	0.489104729	0.319391061	0.992983625
A_98763_GCCACATATGGAACAAGAACAGGC	0.654380994	0.492005472	1.035298338
A_98763_GCGAGTAACAACCACAGCTAACGA	0.895853917	0.576863284	1.425392807
A_98763_GCTCGGTACAGCGTTAGATAGACA	0.526824703	0.448603543	1.073096213
A_98763_GGTGCGAAAAGGTACAGGAGAACA	0.206034536	0.737899622	1.471156296
A_98763_GTCTGTCAACACGACCAGGCTAAC	0.510903107	0.380944521	1.053933916
A_98763_GTGTTCTAGCTCGGTAAGTGGTCA	0.411337916	0.286046711	1.323276262
A_98763_TAGGATGAAACGTGATAAGAGATC	0.405260713	0.954322062	1.787762986
A_98763_TAGGATGAGGAGAACACCGAAGTA	0.474975276	0.865054836	1.604185251
A_98763_TATCAGCAACCTCCAAGGAGAACA	0.770165807	0.602068329	1.246707826
A_98763_TCCGTCTAACAGATTCGATAGACA	0.617811426	0.243977592	1.358395071
A_98763_TCCGTCTACAACCACAACAGCAGA	0.423403815	0.477700752	2.035276314
A_98763_TCTTCACAACAGATTCTGGAACAA	1.066258785	0.815952049	1.469465269
A_98763_TCTTCACAACCTCCAACGCTGATC	0.56893898	0.270377159	0.391304047
A_98763_TGGAACAACCATCCTCGCCACATA	0.454766476	0.493140923	1.024879622
A_98763_TGGCTTCAAAGGTACAAACAACCA	0.545045362	0.852321714	1.834945222
A_98763_TGGTGGTAAACAACCACTCAATGA	0.428378902	0.875517256	1.506880373
A_98763_TGGTGGTAGCTCGGTACAACCACA	0.654012101	0.46950437	0.967276046
A_98763_TGGTGGTAGTACGCAAAAGACGGA	0.562761693	0.360802343	1.569496299
A_98763_TTCACGCACATACCAAATCCTGTA	0.515557018	0.455855561	0.974196267
A_98763_TTCACGCAGCGAGTAAAACTCACC	0.270752007	0.733790331	1.215450147
A_98763_AAACATCGAGATCGCAAAACATCG	0.783583686	2.503966968	1.708879253
A_98763_AAACATCGATGCCTAAAACAACCA	1.047482625	2.252415334	1.979870394
A_98763_AACAACCAAACGCTTAATCATTCC	1.160388182	1.363919341	1.151559131
A_98763_AACAACCAACAAGCTACCTCTATC	0.678821764	0.944901812	1.123535457
A_98763_AACAACCACCGTGAGATGGAACAA	0.892208549	2.407316001	1.859663538
A_98763_AACAACCAGCTCGGTAAACGCTTA	1.278686593	1.388569853	1.430633296
A_98763_AACCGAGAAATGTTGCAGGCTAAC	0.87360222	1.255512299	1.365172538
A_98763_AACCGAGAAGGCTAACCTAAGGTC	0.536907868	1.181854921	1.816349392
A_98763_AACCGAGAAGTCACTACGCTGATC	1.154888485	1.899327332	2.249132117
A_98763_AACGCTTAACCACTGTAACAACCA	0.645120498	1.438277311	1.620525943

A_98763_AACGCTTAAGTGGTCACCGAAGTA	1.039066474	2.414476483	1.998777437
A_98763_AACGCTTACGACACACGAATCTGA	0.662391465	2.191638201	1.718195442
A_98763_AACGCTTATTCACGCAAACGCTTA	0.887941313	1.012642614	1.248564364
A_98763_AACGTGATCGAACTTAAACAACCA	0.604295081	1.993716473	1.798035584
A_98763_AACGTGATCTGAGCCACGCTGATC	0.523616805	1.605584677	1.916534805
A_98763_AACGTGATCTGTAGCCGCCACATA	0.445756809	1.193997755	1.520815076
A_98763_AACTCACCAAACATCGAACGTGAT	0.415980701	0.965058381	1.826590479
A_98763_AACTCACCAACCGAGAGGAGAACA	1.14724608	1.105989769	2.025453103
A_98763_AACTCACCGGTGCGAAGCGAGTAA	1.166590291	2.354324785	1.336179222
A_98763_AACTCACCGTCGTAGATCCGTCTA	0.417322207	1.493606	1.375132253
A_98763_AAGACGGAATTGGCTCGTCGTAGA	0.883646794	1.6964548	1.8529552
A_98763_AAGAGATCAGAGTCAAAACTCACC	0.376720991	1.029291094	1.544604709
A_98763_AAGAGATCGCCACATAACACAGAA	0.937815576	1.259760652	1.971642287
A_98763_AAGGACACGAGCTGAACCTCCTGA	0.806171932	0.57573245	1.465192923
A_98763_AAGGACACGGAGAACACTCAATGA	0.171797719	1.084799909	1.234536222
A_98763_AAGGTACAAACAACCAAACTCACC	0.778639438	1.659128284	1.518777464
A_98763_AAGGTACAACCTCCAAGACTAGTA	0.468385923	1.357001778	1.575022313
A_98763_AAGGTACAGCCACATAAGCACCTC	0.970406332	1.107722937	1.636834873
A_98763_AATCCGTCCTGTAGCCAGTCACTA	0.866271449	2.069383248	1.837273231
A_98763_AATCCGTCGAGCTGAATGGCTTCA	0.347177366	1.385781708	1.649056072
A_98763_AATCCGTCTAGGATGAACGCTCGA	1.261745852	2.442850095	1.372230983
A_98763_ACAAGCTACAGATCTGCTGGCATA	0.771371544	1.940358355	2.198109329
A_98763_ACAAGCTACCGACAACGACAGTGC	0.811732205	2.362189323	2.206499635
A_98763_ACAAGCTACCTCTATCACGTATCA	0.93697227	1.489198394	2.309208393
A_98763_ACAAGCTAGAACAGGCAGATCGCA	0.833305742	0.956902197	1.636950131
A_98763_ACAAGCTAGAATCTGACGCATACA	0.669264579	0.994377874	1.387623299
A_98763_ACAAGCTAGGAGAACACGAACTTA	0.922037178	1.939695055	1.717878584
A_98763_ACACAGAAAATGTTGCTCCGTCTA	0.394436192	1.399995183	1.482389031
A_98763_ACACGACCAGCCATGCGAGTTAGC	0.670537417	1.560481332	1.859302309
A_98763_ACACGACCGTACGCAAGTCTGTCA	1.043198735	1.533972227	1.916274691
A_98763_ACAGCAGAAAACATCGCATCAAGT	0.634257718	1.352696834	1.280178309
A_98763_ACAGCAGAAGCACCTCAACTCACC	0.763075162	1.44214215	1.74520991

A_98763_ACAGCAGAAGCAGGAAGCGAGTAA	0.280840179	0.803091165	1.781949238
A_98763_ACAGCAGACCGACAACAGATGTAC	0.141008854	1.903123155	1.593501816
A_98763_ACATTGGCAACCGAGAAGCACCTC	1.08203281	2.088555783	1.974329275
A_98763_ACCACTGTACATTGGCGACAGTGC	0.611936216	1.060289079	1.797403838
A_98763_ACCACTGTCATCAAGTCAGCGTTA	0.886847334	1.215494142	1.472654818
A_98763_ACCTCCAAAGCAGGAAACATTGGC	0.576492392	1.679412588	1.428615253
A_98763_ACCTCCAAATTGAGGACTAAGGTC	1.04433552	1.875559663	1.726715987
A_98763_ACCTCCAATAGGATGACCTCTATC	0.198086112	1.573872208	1.840529477
A_98763_ACGCTCGAAATCCGTCGCGAGTAA	1.125986393	1.754547403	2.264664868
A_98763_ACGCTCGAAGGCTAACCGACTGGA	0.290611796	1.220663871	1.907840818
A_98763_ACGCTCGACCTCTATCGAGTTAGC	0.461134918	1.30973132	2.008709573
A_98763_ACGCTCGACGAACTTATGGAACAA	1.021835207	1.27957192	1.915912673
A_98763_ACGCTCGACGACTGGACAAGGAGC	0.674750781	0.809641071	1.334537661
A_98763_ACGCTCGACGCATACAAGGCTAAC	0.516015805	0.933286039	2.090948317
A_98763_ACGCTCGAGTGTTCTACCGAAGTA	0.702643542	1.209839772	2.017059329
A_98763_ACGTATCACGAACTTAAGCAGGAA	0.686463493	1.908020252	1.908704
A_98763_ACTATGCAACACGACCACAAGCTA	1.181370446	2.360923894	1.875763524
A_98763_ACTATGCAGGAGAACACATCAAGT	0.62801711	1.382656282	1.834347692
A_98763_ACTATGCAGTACGCAAGACTAGTA	0.495369004	1.311362916	1.830649211
A_98763_AGAGTCAACGGATTGCTGGCTTCA	1.077668201	1.865818335	1.866866002
A_98763_AGATCGCAATAGCGACCCTCTATC	0.708267605	0.893842538	1.793744049
A_98763_AGATCGCACCAGTTCAGAGTTAGC	0.548564602	0.727172538	1.578168804
A_98763_AGATCGCACCGTGAGATCCGTCTA	1.023871295	2.258976214	1.80827092
A_98763_AGATCGCAGATGAATCAATGTTGC	0.795545603	1.448120572	1.589010233
A_98763_AGATGTACAACCGAGACATCAAGT	0.986422866	1.339657957	1.599390157
A_98763_AGATGTACAAGACGGAAGATGTAC	0.557650605	1.683318235	1.669511103
A_98763_AGATGTACAATGTTGCACAGATTC	0.519192987	2.06020878	1.681848629
A_98763_AGATGTACACCTCCAAACGCTCGA	1.055763419	1.109045218	2.055903056
A_98763_AGCACCTCACGTATCACTGGCATA	0.848997572	2.094366978	1.624560354
A_98763_AGCACCTCATCATTCCAACCGAGA	0.6991058	1.799214268	1.948532719
A_98763_AGCACCTCCGACTGGACGACTGGA	0.642996673	1.853455555	1.840064085
A_98763_AGCACCTCCTCAATGAATGCCTAA	0.677636159	1.680862204	1.58957689

A_98763_AGCACCTCGACTAGTACTGGCATA	0.941843798	1.941946212	1.645176502
A_98763_AGCACCTCGCCACATACACTTCGA	0.762987296	1.06987995	1.330284744
A_98763_AGCACCTCGCTAACGACTAAGGTC	0.636218819	1.610556125	2.081706652
A_98763_AGCAGGAACCTCTATCGCTAACGA	0.322589803	1.53933785	2.181068098
A_98763_AGCAGGAACTGAGCCACAAGACTA	0.938527997	1.840137377	1.47436943
A_98763_AGCAGGAAGTCTGTCAAGGCTAAC	0.848762083	1.315216505	1.444360776
A_98763_AGCCATGCACCACTGTAGCAGGAA	0.525742819	1.491404001	2.144816022
A_98763_AGCCATGCAGCCATGCCTCAATGA	0.540569906	1.259392344	2.084499262
A_98763_AGCCATGCATAGCGACTGGTGGTA	1.016826959	1.690191471	1.414831444
A_98763_AGCCATGCCAAGGAGCGAATCTGA	0.866202941	1.27277856	1.688880954
A_98763_AGCCATGCGAATCTGATCCGTCTA	0.474952034	1.080933192	1.61918996
A_98763_AGGCTAACGAACAGGCCAGATCTG	1.33165626	1.576301007	1.838877155
A_98763_AGGCTAACGTCGTAGAACATTGGC	0.819917765	1.145293925	1.941306291
A_98763_AGTACAAGACACAGAAAAGAGATC	0.352013894	1.478896734	1.835373427
A_98763_AGTACAAGATTGGCTCACGTATCA	1.100756879	1.473439181	1.712147095
A_98763_AGTACAAGGAGTTAGCGCTAACGA	0.446252845	1.334906759	1.814361708
A_98763_AGTACAAGTCCGTCTACCGACAAC	0.502557656	1.4894743	1.708296894
A_98763_AGTCACTAACGCTCGAGTACGCAA	0.950040216	1.98297043	1.48307356
A_98763_AGTCACTAAGTCACTAAACCGAGA	1.020367341	0.952799982	1.248284446
A_98763_AGTCACTAGAACAGGCCCTAATCC	0.649508161	1.295558812	1.707651731
A_98763_AGTCACTAGCCACATACTAAGGTC	0.625108857	2.069827492	2.162566266
A_98763_AGTCACTATTCACGCACACTTCGA	0.792374964	1.398513837	1.58417588
A_98763_AGTGGTCACGAACTTACAATGGAA	0.949866058	2.174407317	1.803295547
A_98763_AGTGGTCACGACTGGACTAAGGTC	0.723588225	1.167574914	1.677498311
A_98763_AGTGGTCATCTTCACACGCATACA	0.650523161	0.918287727	1.041181659
A_98763_ATAGCGACAAACATCGACAGATTC	0.80365376	0.866478925	1.653212535
A_98763_ATAGCGACAATGTTGCGTCGTAGA	0.601890628	1.338021326	1.364581492
A_98763_ATAGCGACCAAGGAGCATCCTGTA	1.4142347	1.847791863	2.100262631
A_98763_ATAGCGACCGCTGATCCGCATACA	1.116928772	1.959707302	1.431199709
A_98763_ATAGCGACGACAGTGCGGTGCGAA	0.75023494	1.45799023	1.605105058
A_98763_ATAGCGACGGAGAACAACAAGCTA	0.805811703	1.060896014	2.282442565
A_98763_ATCATTCCAACGTGATACACAGAA	0.752348525	2.316832887	2.060737074

A_98763_ATCATTCCACAGATTCACGCTCGA	0.406785055	1.529923126	1.489352106
A_98763_ATCATTCCCAGATCTGGAACAGGC	0.430339061	0.992303806	1.551555425
A_98763_ATCATTCCCGACACACCAACCACA	1.023376678	1.407251245	1.87747568
A_98763_ATCATTCCGGTGCGAAAGCACCTC	0.575657091	2.010971522	1.677152673
A_98763_ATCCTGTAAGATGTACAACGTGAT	0.622062515	1.455463652	1.564168957
A_98763_ATCCTGTAATAGCGACATTGAGGA	0.796043302	2.135951818	2.008439939
A_98763_ATTGAGGAAAGAGATCCCGAAGTA	0.541208899	1.570317229	1.850929651
A_98763_ATTGAGGAACCTCCAAAGATGTAC	0.636037281	1.918399084	1.239882403
A_98763_ATTGAGGAAGCCATGCCGAACTTA	0.908184331	1.763542671	1.619514649
A_98763_ATTGAGGACATCAAGTACAGCAGA	0.769034035	1.035982787	1.395726257
A_98763_ATTGAGGACCATCCTCGTCGTAGA	0.424014088	1.818701562	1.95033624
A_98763_ATTGAGGACCGAAGTATGGTGGTA	0.924941994	2.46702556	2.105312313
A_98763_ATTGAGGACCTCCTGAACATTGGC	1.058431068	2.08694049	2.393224276
A_98763_ATTGAGGATAGGATGAGTACGCAA	0.743877047	1.721886072	1.684002065
A_98763_ATTGGCTCGGTGCGAATGGTGGTA	0.693135104	2.157102321	1.650382414
A_98763_CAACCACAGAGTTAGCAGTGGTCA	0.606781147	0.925866053	1.665901589
A_98763_CAAGACTACCGAAGTAGCCACATA	0.439960892	1.00170823	2.106212279
A_98763_CAAGACTACCTAATCCCCTCTATC	0.847500979	1.430539625	1.844083162
A_98763_CAAGGAGCACTATGCACGCTGATC	0.475230168	1.709200578	1.446469248
A_98763_CAAGGAGCAGCACCTCAGTCACTA	1.313716164	1.074619518	2.004729841
A_98763_CAAGGAGCCAACCACAGAACAGGC	0.768411283	1.962997293	2.007394098
A_98763_CAAGGAGCCGCATACAAGTGGTCA	0.965453138	0.973023062	1.440276179
A_98763_CAATGGAAAACCGAGAACAAGCTA	0.872571075	1.705902049	2.048752779
A_98763_CAATGGAAAAGACGGAGCTCGGTA	0.745217802	1.977067877	2.063144831
A_98763_CAATGGAAATCATTCCCCGAAGTA	0.671174926	0.850509706	1.260138404
A_98763_CAATGGAACCAGTTCAAAGGTACA	0.865249396	2.093312954	1.981810595
A_98763_CAATGGAACCAGTTCAGAATCTGA	0.936534121	2.022511923	2.173272326
A_98763_CAATGGAACTGGCATACGCTGATC	0.915426845	2.476909926	1.803080269
A_98763_CAATGGAAGCTAACGACTCAATGA	1.050427787	1.489967246	1.525223522
A_98763_CACCTTACACAGATTCCTGAGCCA	0.78253626	1.811496158	1.805603847
A_98763_CACCTTACACCACTGTGAACAGGC	0.798980123	2.048402756	1.447866228
A_98763_CACCTTACATTGAGGAGAGTTAGC	0.372973922	1.920838426	2.030657577

A_98763_CACCTTACCACCTTACTTCACGCA	0.596173239	2.847536095	1.454539239
A_98763_CACCTTACTCCGTCTAGTCGTAGA	0.821700425	0.784234419	0.931780445
A_98763_CACTTCGAAACGTGATAGTGGTCA	0.802692989	1.168144944	1.690507484
A_98763_CACTTCGAAAGGTACACAGCGTTA	0.429914021	1.729351343	1.244054844
A_98763_CACTTCGAACAAGCTACAATGGAA	0.78749735	1.99065018	1.524109427
A_98763_CACTTCGAATCCTGTAAAGAGATC	0.827598222	1.222488193	2.190129366
A_98763_CACTTCGACACCTTACAAGGTACA	0.454692752	0.859341326	1.61838575
A_98763_CACTTCGACTCAATGAAGATCGCA	0.430559092	1.309125393	1.320119081
A_98763_CACTTCGACTGTAGCCCAATGGAA	0.862050863	1.818047994	1.338506305
A_98763_CAGATCTGACAGCAGACTGAGCCA	0.371984111	2.057961357	1.644994425
A_98763_CAGATCTGCGCATACAACGCTCGA	0.638395741	0.592792987	1.469418423
A_98763_CAGATCTGCTAAGGTCGACAGTGC	0.5055834	0.831720725	1.846839609
A_98763_CAGCGTTAAACAACCATGGCTTCA	0.544813757	1.283943105	2.457123851
A_98763_CAGCGTTAAACCGAGACCATCCTC	1.094334884	1.666283111	1.468651237
A_98763_CAGCGTTAAGAGTCAACGAACTTA	1.160850574	0.934025532	1.62699075
A_98763_CAGCGTTAATTGAGGACAACCACA	0.426884374	0.632413077	1.917412265
A_98763_CAGCGTTACCTAATCCCGCTGATC	1.206642147	2.100814823	1.785690848
A_98763_CAGCGTTACGACACACACACAGAA	0.878958352	1.013821676	1.283550805
A_98763_CAGCGTTAGCCACATACAATGGAA	0.979197702	1.605332792	1.646396164
A_98763_CAGCGTTAGTCGTAGACCTCTATC	0.549333421	1.436554024	1.547670172
A_98763_CATACCAAAATCCGTCTGGCTTCA	0.706381473	1.487835953	1.17180797
A_98763_CATCAAGTAGATGTACTGGCTTCA	0.187561397	0.993681558	1.751477432
A_98763_CATCAAGTATGCCTAAACCACTGT	0.462200531	0.898836462	1.624600687
A_98763_CATCAAGTCAACCACAAAGAGATC	0.613087843	1.647879309	1.860947901
A_98763_CATCAAGTCATACCAATGGCTTCA	0.833585815	1.92650699	2.194233011
A_98763_CATCAAGTCTGAGCCAACCTCCAA	0.425029633	1.547928764	1.500566199
A_98763_CCAGTTCACACCTTACCCTCCTGA	1.029045838	1.947863594	1.555895105
A_98763_CCAGTTCACCGACAACCGAACTTA	0.501275323	1.527053226	1.867906081
A_98763_CCAGTTCACCGTGAGACTAAGGTC	0.603144265	1.587601595	1.615979188
A_98763_CCAGTTCACGCTGATCAGGCTAAC	0.339831515	1.853768675	1.348575637
A_98763_CCAGTTCATGGTGGTATAGGATGA	1.018441461	2.080002144	1.438897605
A_98763_CCATCCTCAAGAGATCGTCGTAGA	1.061788115	1.220424784	1.673126051

A_98763_CCATCCTCAGCCATGCCAACCACA	0.660128218	1.786039905	1.954960612
A_98763_CCATCCTCCAACCACAGCGAGTAA	1.07042295	2.345490952	1.214751586
A_98763_CCATCCTCCCTCTATCACACGACC	0.31627442	0.974772109	0.926932799
A_98763_CCATCCTCCTCAATGAAATGTTGC	0.754841885	1.498093119	1.873317248
A_98763_CCATCCTCGACTAGTAAAGAGATC	0.769918218	1.121582168	1.65599562
A_98763_CCGAAGTAACGCTCGAAGGCTAAC	0.695487595	1.163454141	1.975396166
A_98763_CCGAAGTACAACCACAACACGACC	0.717600101	1.981834353	2.144882825
A_98763_CCGAAGTAGCCACATAACCTCCAA	0.601249834	1.943376171	1.942665556
A_98763_CCGAAGTATGAAGAGATGGAACAA	0.735007037	1.167843324	1.755871507
A_98763_CCGAAGTATTCACGCACATACCAA	0.272268007	1.170932576	1.519805242
A_98763_CCGACAACAAGGTACAACCACTGT	0.855893416	1.527568149	1.417740668
A_98763_CCGACAACCCGTGAGAACAGATTC	1.185283407	2.045216253	1.63300899
A_98763_CCGTGAGAAAGGTACAATCATTCC	0.417741054	1.493550917	1.404644405
A_98763_CCGTGAGACCTAATCCCCGTGAGA	0.700733392	1.618672403	2.257512316
A_98763_CCGTGAGACGCTGATCCGACACAC	0.689087487	1.964304228	1.892063992
A_98763_CCTAATCCCGAACTTAGCTCGGTA	0.763483713	1.939596907	2.420291229
A_98763_CCTAATCCCGCATACATGGCTTCA	0.432789831	1.218481027	1.017325086
A_98763_CCTCCTGACCTCTATCAAACATCG	0.677794185	1.963507978	1.619839149
A_98763_CCTCTATCACCTCCAAAGCACCTC	0.920022284	1.924592832	2.015122005
A_98763_CCTCTATCATCATTCCAGTCACTA	0.667788199	2.303688985	1.710584966
A_98763_CCTCTATCTTCACGCAGCTCGGTA	1.26187906	1.423808759	2.175880883
A_98763_CGAACTTAAAGGTACAGGAGAACA	0.560177081	1.322845288	1.586510118
A_98763_CGAACTTAACCACTGTAGCAGGAA	0.63119119	1.582109085	1.829992772
A_98763_CGAACTTACCGTGAGAAGGCTAAC	0.78063259	1.828162042	1.667291865
A_98763_CGAACTTATCTTCACACCTCTATC	0.713134591	1.529673356	1.522388031
A_98763_CGACACACACCTCCAAACAGCAGA	0.542849501	1.21975753	1.775630409
A_98763_CGACACACAGTCACTAGTCTGTCA	0.813574054	1.145261051	1.921609728
A_98763_CGACACACCAGCGTTACCGTGAGA	1.129025885	1.725690033	1.595529868
A_98763_CGACTGGACTGAGCCAAACAACCA	0.422328392	1.497097979	1.265361852
A_98763_CGACTGGATCCGTCTAAGGCTAAC	0.725786905	0.925905236	1.507650669
A_98763_CGCATACAAAGGTACAATGCCTAA	0.687632698	1.576411784	2.297112717
A_98763_CGCATACAAATCCGTCACACAGAA	1.143615748	1.184447601	2.045500455

A_98763_CGCATACAAGGCTAACAAGACGGA	0.590920105	1.10062511	1.734858349
A_98763_CGCATACAGGTGCGAATATCAGCA	0.836701186	2.414476886	2.096821842
A_98763_CGCTGATCAACGTGATCAGCGTTA	1.040807327	2.271264872	1.52809123
A_98763_CGCTGATCAACGTGATCGAACTTA	1.047061477	1.643255736	1.150873381
A_98763_CGCTGATCGATGAATCACGTATCA	0.90006707	1.620074252	1.732489642
A_98763_CGCTGATCGCTAACGACTCAATGA	0.751679795	2.152561278	1.797647111
A_98763_CGGATTGCAACAACCAAACTCACC	0.379204882	2.142416008	1.868369421
A_98763_CGGATTGCTTCACGCACCGACAAC	0.882921417	1.594864926	2.433789679
A_98763_CTAAGGTCGATAGACAAGATCGCA	0.642638024	1.294066019	1.651738753
A_98763_CTAAGGTCTCTTCACACACTTCGA	0.353805596	1.626406818	1.595630375
A_98763_CTAAGGTCTGAAGAGATCTTCACA	1.280170081	2.782567784	1.243361641
A_98763_CTCAATGAACCTCCAAACAGCAGA	0.506518864	1.050728869	1.840951072
A_98763_CTCAATGAAGATCGCAGAACAGGC	1.039566523	2.042567019	1.896108042
A_98763_CTGAGCCAAAACATCGCCTCTATC	0.927076487	1.953449815	1.755110378
A_98763_CTGAGCCAAACGCTTAGAACAGGC	0.561926566	1.625284858	1.197399817
A_98763_CTGAGCCACAAGGAGCGCTAACGA	0.69829687	1.552171825	1.376856877
A_98763_CTGAGCCAGCCAAGACAGTGGTCA	0.27929456	1.243544911	1.972353053
A_98763_CTGGCATAAGTGGTCAGAACAGGC	0.658000698	1.373097719	1.758809807
A_98763_CTGGCATACGACTGGACACTTCGA	0.836223617	0.794493648	1.607251511
A_98763_CTGTAGCCAAACATCGCGCATACA	0.630036005	1.314734286	1.709478844
A_98763_CTGTAGCCAACGCTTAAAGGTACA	0.616271184	2.400973166	1.319705466
A_98763_CTGTAGCCACAGCAGAGACAGTGC	1.132477241	1.837031881	2.458635285
A_98763_CTGTAGCCCATACCAAAGCAGGAA	0.855793284	1.827472638	2.440843599
A_98763_GAACAGGCACAAGCTAACCTCCAA	0.785510772	2.901240497	1.168022311
A_98763_GAACAGGCATGCCTAAATCCTGTA	0.434721491	1.851811685	1.314492652
A_98763_GAACAGGCCACCTTACTGAAGAGA	0.862199373	1.062275217	1.321597751
A_98763_GAACAGGCCCGTGAGAGACTAGTA	1.034254701	1.357978229	2.167730481
A_98763_GAACAGGCGCTCGGTACGCATACA	0.821864684	2.406690606	1.958744416
A_98763_GAATCTGAATTGAGGAGCGAGTAA	0.882088255	2.014802805	1.715776421
A_98763_GAATCTGACCGACAACCGCTGATC	0.442483024	2.550509705	1.380255195
A_98763_GAATCTGACGCTGATCATTGAGGA	0.748852745	2.022131749	1.991763702
A_98763_GAATCTGACTCAATGAACAGATTC	0.719131912	1.603957022	2.058173973

A_98763_GAATCTGACTGTAGCCAGCCATGC	0.81880854	1.167003699	1.666440077
A_98763_GAATCTGAGCCACATACCTCCTGA	0.740044407	2.239471501	1.405914622
A_98763_GAATCTGATGAAGAGAAAGGTACA	0.81530839	1.651289512	2.340793261
A_98763_GACAGTGCAATGTTGCACACGACC	0.55120542	0.978284744	1.534097417
A_98763_GACAGTGCACAGCAGAACCACTGT	1.025585611	1.901416256	1.203198063
A_98763_GACAGTGCAGATCGCACAGATCTG	0.926841213	0.704724749	1.440244762
A_98763_GACAGTGCAGGCTAACCACTTCGA	1.075649158	1.743193411	1.968588335
A_98763_GACAGTGCCTAAGGTCGACTAGTA	0.936877073	1.754351956	1.703562858
A_98763_GACAGTGCTCTTCACACAATGGAA	0.764280402	1.901376688	2.041944834
A_98763_GACTAGTAAACCGAGAACGCTCGA	0.425074548	0.956641904	1.928470537
A_98763_GACTAGTACGACTGGATTCACGCA	0.30223053	1.371507502	1.39519232
A_98763_GACTAGTAGAATCTGAGCTCGGTA	0.78854429	1.688160918	1.849980759
A_98763_GACTAGTAGTGTTCTACAGCGTTA	1.275980699	2.021793522	2.245427919
A_98763_GACTAGTATCTTCACATGGAACAA	0.941474362	1.982176498	1.353600125
A_98763_GAGCTGAAAAACATCGGATAGACA	0.384985786	1.614777838	1.760748228
A_98763_GAGCTGAACCTCTATCAGATGTAC	0.144145792	2.675724033	1.275436681
A_98763_GAGCTGAACTCAATGACCGAAGTA	0.886179189	2.350215499	1.532233782
A_98763_GAGTTAGCAGATGTACACACGACC	0.534628461	1.16862457	1.888080738
A_98763_GAGTTAGCGATGAATCACAGCAGA	0.840192353	0.719004246	1.873797343
A_98763_GAGTTAGCGGAGAACACTGAGCCA	0.449559966	1.635583791	1.732633109
A_98763_GAGTTAGCTTCACGCAGTCGTAGA	1.018882817	1.789116792	1.765315386
A_98763_GATAGACAATAGCGACCTCAATGA	0.617549257	1.379561717	2.107008963
A_98763_GATAGACAGATGAATCAGATGTAC	0.300071801	1.493828721	1.306783434
A_98763_GATAGACAGTCGTAGACAATGGAA	0.412457835	1.390815321	1.859288129
A_98763_GATAGACATGGTGGTAAGCACCTC	0.863220946	0.920483316	2.074639685
A_98763_GATGAATCGCCAAGACCATCAAGT	0.934054973	2.087020949	1.593671896
A_98763_GATGAATCGCTAACGAAACGCTTA	0.725349292	2.315565701	1.563267866
A_98763_GATGAATCGTCTGTCAAAGGTACA	0.605124184	1.2625513	1.888337524
A_98763_GATGAATCTGGAACAACACTTCGA	0.394668972	2.002517713	1.282750472
A_98763_GCCAAGACAACGTGATACACGACC	0.894346487	1.657495292	1.503730299
A_98763_GCCAAGACAGGCTAACAACTCACC	0.68982118	1.981990766	1.728212145
A_98763_GCCAAGACCGCTGATCAGTGGTCA	0.434233132	1.66943333	1.144077917

A_98763_GCCACATAAAGACGGACTGAGCCA	0.499896564	1.328833041	1.839681477
A_98763_GCCACATACGGATTGCTCCGTCTA	0.335733648	1.486691888	1.540217217
A_98763_GCGAGTAAAACTCACCACAGATTC	1.001130643	1.745599211	1.927136392
A_98763_GCGAGTAAGCCAAGACAACTCACC	0.554519223	1.54365569	1.478416006
A_98763_GCGAGTAAGCGAGTAAGTCGTAGA	0.690263443	1.263997337	2.140314066
A_98763_GCGAGTAAGGAGAACACTCAATGA	1.209476291	2.052969929	1.533150436
A_98763_GCGAGTAATCCGTCTAGAGTTAGC	0.893459269	1.890726616	1.593523278
A_98763_GCTAACGAAACGCTTAAGATCGCA	0.903703407	1.933995385	1.625187637
A_98763_GCTAACGAGATAGACACCGTGAGA	0.89316065	2.053421308	1.963402887
A_98763_GCTAACGAGCGAGTAAGAGTTAGC	0.334578718	1.44994521	1.60158431
A_98763_GCTAACGAGGAGAACAGCTAACGA	0.715547403	1.650453361	2.137797438
A_98763_GCTCGGTAAAACATCGAACAACCA	0.494457821	1.87310738	1.940751207
A_98763_GCTCGGTAAACGCTTACGACACAC	0.8329296	1.049756034	1.64391554
A_98763_GCTCGGTACCTAATCCTCTTCACA	0.797495423	1.86079614	1.680314988
A_98763_GCTCGGTAGCTCGGTAATCATTCC	0.789849084	1.645037287	1.63678191
A_98763_GGAGAACAAGTACAAGTCCGTCTA	1.077741868	1.995664633	2.186745125
A_98763_GGAGAACACGGATTGCAGTGGTCA	0.708092637	1.34847634	1.804786167
A_98763_GGAGAACAGATGAATCAGCACCTC	0.848330757	2.038505254	2.017981627
A_98763_GGTGCGAAACACAGAACCTCTATC	0.89546728	1.538477848	2.013040397
A_98763_GGTGCGAAAGCACCTCACGCTCGA	0.807818877	1.895278646	1.92780655
A_98763_GGTGCGAAATAGCGACCAACCACA	0.92681677	1.306536842	1.776804538
A_98763_GGTGCGAACGACACACATTGGCTC	0.408776033	0.768275241	1.752197021
A_98763_GTACGCAAATAGCGACCCTCTATC	0.662990919	1.164339582	2.080342295
A_98763_GTACGCAACTGTAGCCAACGTGAT	0.804031505	2.064769665	1.886960461
A_98763_GTACGCAATGGAACAAAAACATCG	0.648648256	1.057377918	2.144731601
A_98763_GTACGCAATGGTGGTACCTCCTGA	0.285363551	0.972268852	1.914554085
A_98763_GTCGTAGAACGTATCATGGTGGTA	0.343349813	1.319535556	2.215443129
A_98763_GTCGTAGAAGCAGGAATTCACGCA	0.464450307	1.641687141	1.540331594
A_98763_GTCGTAGAATCATTCCAGCACCTC	1.176111328	2.403116112	1.681492079
A_98763_GTCGTAGACGACACACACGCTCGA	0.626167841	1.586181583	2.133031438
A_98763_GTCGTAGATGGTGGTACTCAATGA	0.971326016	1.181024059	1.76781389
A_98763_GTCTGTCAAAACATCGGACTAGTA	1.153221838	2.029454541	2.059665877

A_98763_GTCTGTCAATCCTGTAAACCGAGA	0.815190281	2.658563978	1.844639403
A_98763_GTCTGTCACCAGTTCAAACTCACC	0.628663345	2.007566173	1.880027072
A_98763_GTCTGTCACTGAGCCAAAGAGATC	0.921686334	1.67733957	1.937143179
A_98763_GTCTGTCAGGAGAACACGCTGATC	0.43022853	0.679718957	1.582078729
A_98763_GTGTTCTAAGTACAAGTGGCTTCA	0.209623028	1.47237258	1.151954562
A_98763_GTGTTCTACATACCAACATCAAGT	0.263980232	1.237663865	1.845572951
A_98763_TAGGATGAAGTGGTCACCTAATCC	1.014814474	0.824169909	1.559718063
A_98763_TATCAGCAAACAACCACACTTCGA	0.961769359	1.923740556	1.551722257
A_98763_TATCAGCAAAGGTACAAGATGTAC	0.993942351	1.572732679	1.395740396
A_98763_TATCAGCACAATGGAAGACTAGTA	0.94947653	0.797304172	1.187491415
A_98763_TATCAGCACCTCCTGAAGTCACTA	0.594473694	0.930305314	2.107453751
A_98763_TATCAGCAGCCACATAACGTATCA	0.589541124	1.153163122	1.969599235
A_98763_TCCGTCTAAACGTGATCAACCACA	0.481167574	1.468916295	1.076614046
A_98763_TCCGTCTACCTAATCCCAACCACA	0.792204273	1.649435466	2.02277145
A_98763_TCTTCACAAGTACAAGGTCTGTCA	0.702979732	0.717174327	1.720901641
A_98763_TCTTCACACAACCACAAGATCGCA	0.370632589	1.135739772	1.987728643
A_98763_TCTTCACACCTAATCCCGCATACA	0.624918234	2.096174907	2.29332064
A_98763_TCTTCACACGACTGGACCTCCTGA	0.822128853	2.241702643	1.270511832
A_98763_TCTTCACAGGAGAACAAACTCACC	0.786275765	1.415365318	1.558940245
A_98763_TCTTCACATGGAACAACACTTCGA	0.710428542	1.873251046	1.395961604
A_98763_TGAAGAGAACACGACCCGACTGGA	0.412325447	0.93123442	1.666055531
A_98763_TGAAGAGACGAACTTAGTCGTAGA	1.11481042	1.915296878	1.874151279
A_98763_TGAAGAGACTGAGCCAACATTGGC	0.662797311	1.415766805	1.843692424
A_98763_TGAAGAGACTGTAGCCCATACCAA	0.94533985	1.34379849	1.99003265
A_98763_TGAAGAGATATCAGCAAAGAGATC	0.961598928	1.471396304	2.03045517
A_98763_TGAAGAGATGGTGGTACGAACTTA	1.320862625	2.597117674	1.519480809
A_98763_TGGAACAAACAGATTCGCTAACGA	0.548581659	1.673639033	2.23438039
A_98763_TGGAACAAAGCAGGAAATGCCTAA	0.530762852	1.075402436	2.28571319
A_98763_TGGAACAAATTGAGGAAAACATCG	0.877527781	1.970503161	1.658060392
A_98763_TGGAACAAATTGAGGAAACCGAGA	0.540457211	1.552855462	1.598030459
A_98763_TGGAACAAGACAGTGCAGCCATGC	0.562253088	1.946194393	2.062089587
A_98763_TGGAACAAGAGCTGAACTAAGGTC	0.458110429	1.389504341	1.446723918

A_98763_TGGCTTCAAAGAGATCAGTGGTCA	1.108358206	2.331279053	1.502710079
A_98763_TGGCTTCACATACCAACCTCCTGA	0.70402947	2.220837632	2.170634007
A_98763_TGGCTTCACTGGCATAAAGAGATC	0.843149904	1.439768116	2.03420042
A_98763_TGGTGGTAAAGACGGAAGATGTAC	1.172080143	1.71526687	1.962151546
A_98763_TGGTGGTACCTCCTGAGACAGTGC	0.711656392	1.767746311	1.633153489
A_98763_TGGTGGTACTAAGGTCTCTTCACA	0.677258956	1.827027871	1.288266937
A_98763_TGGTGGTAGCCAAGACGGTGCGAA	0.301169533	1.332574343	2.219667323
A_98763_TTCACGCACACCTTACTGAAGAGA	1.320656363	1.043324781	2.250054729
A_98763_TTCACGCACCGTGAGAATCATTCC	0.72146776	1.393696252	1.277511257
A_98763_TTCACGCACCTCTATCTGGTGGTA	0.792092089	1.737125128	2.339746603
A_98763_TTCACGCAGAACAGGCCGCTGATC	1.149361062	2.323166741	1.341695174
A_98763_TTCACGCAGCTAACGACAGCGTTA	1.208015496	1.652166911	1.331204875
A_98763_AACCGAGAAGTGGTCAAACTCACC	1.05995182	0.869247236	0.899260086
A_98763_AAGGACACGCTAACGACAATGGAA	0.809118941	0.595148206	0.458211834
A_98763_ACATTGGCCCGAAGTATCTTCACA	0.713018971	1.021433896	0.836621597
A_98763_AGATCGCACACCTTACGAGTTAGC	0.463774751	0.308613963	0.714817589
A_98763_AGCAGGAAACCTCCAAAACTCACC	0.49551916	0.408440937	0.644480185
A_98763_AGTACAAGCTGAGCCAATCATTCC	0.696802945	1.164251466	0.736377671
A_98763_CAAGGAGCATAGCGACGACAGTGC	0.758723258	0.308382435	0.5851479
A_98763_CAATGGAAACGTATCAGGTGCGAA	0.426178197	0.989368507	1.107909908
A_98763_CAGATCTGAGATGTACTTCACGCA	1.096855938	0.551504377	0.626491994
A_98763_CCGAAGTACAATGGAACCGTGAGA	0.811511398	1.02658961	0.973959824
A_98763_CGACTGGAAACGTGATAGCCATGC	0.272054634	0.204545279	0.798130304
A_98763_GAACAGGCAAGAGATCCCTCTATC	0.817387819	1.173568651	1.313879661
A_98763_GAACAGGCCGCTGATCAACAACCA	1.00900983	1.010061692	0.99682565
A_98763_GAACAGGCCTAAGGTCAAGACGGA	0.756826063	0.982703619	0.982675576
A_98763_GATAGACAAAGGACACATTGGCTC	1.242756906	1.616538342	1.002165103
A_98763_GATAGACAGAGTTAGCGCCACATA	0.448208782	1.134840472	0.868833384
A_98763_TAGGATGAAACCGAGAACCACTGT	0.90932186	0.536911174	0.79693474
B_98618_ACGTATCAAACCGAGACTAAGGTC	-0.026003932	0.039819166	-0.074252293
B_98618_CCATCCTCCAAGGAGCGAATCTGA	0.23774316	-0.016837338	-0.438644438
B_98618_AAACATCGAGTCACTAGCTAACGA	0.130615894	0.044946576	-0.330048329

B_98618_AACCGAGAGGAGAACAGTCTGTCA	0.107846251	0.260287452	-0.144877833
B_98618_AACGCTTAACGCTCGATGGAACAA	0.319901902	0.138477096	-0.314041721
B_98618_AACGCTTAGCCAAGACATTGAGGA	0.390772924	0.05707349	-0.188165544
B_98618_AACGTGATATCATTCCGCGAGTAA	0.18447614	0.262892484	-0.158352287
B_98618_AACGTGATGCCACATACTGTAGCC	0.172185082	0.125014469	-0.01291938
B_98618_AACTCACCCCGTGAGACCATCCTC	0.312030984	0.033470939	-0.245378413
B_98618_AAGACGGATGAAGAGAACCTCCAA	0.214455506	0.145434393	-0.305070629
B_98618_AAGAGATCACAGCAGAAACTCACC	0.260277829	0.150463655	-0.309372673
B_98618_AAGAGATCTAGGATGATAGGATGA	0.156092512	0.122980593	-0.1112675
B_98618_AAGGTACACGCATACAAGCACCTC	0.112687185	0.083130466	-0.17142157
B_98618_AATCCGTCAACGCTTAGTCGTAGA	0.294558151	-0.096053734	-0.441844118
B_98618_AATCCGTCAAGGTACATGGAACAA	0.079982445	0.128680467	-0.002900633
B_98618_AATCCGTCCATCAAGTGAATCTGA	0.051445654	0.052820507	-0.137373628
B_98618_AATGTTGCCACCTTACATCATTCC	0.094667475	0.070145648	-0.247892728
B_98618_AATGTTGCCGCATACAAGCACCTC	0.29713069	0.101244683	-0.494751911
B_98618_AATGTTGCCTAAGGTCCCAGTTCA	-0.095254668	0.241122596	-0.204640066
B_98618_ACAAGCTAAAACATCGCACCTTAC	0.188260384	0.082449383	-0.21552903
B_98618_ACAAGCTAGACTAGTAAGCCATGC	0.288333718	0.184679617	-0.312422343
B_98618_ACACGACCGTCGTAGACCTCTATC	0.440917334	0.059274449	-0.076758334
B_98618_ACAGATTCACCTCCAAACCTCCAA	0.349104811	0.137573029	-0.171583053
B_98618_ACAGATTCCTGGCATACCGAAGTA	0.463198361	0.240135262	-0.224604092
B_98618_ACAGATTCGCCAAGACAGATGTAC	0.123392216	0.121307076	-0.163317248
B_98618_ACATTGGCAGCCATGCACTATGCA	0.142775466	0.042046858	0.127659485
B_98618_ACCTCCAAATTGGCTCACATTGGC	0.172749405	0.125542492	-0.109662319
B_98618_ACCTCCAATTCACGCAACTATGCA	-0.149393404	-0.008149762	-0.104788369
B_98618_ACGCTCGAACGCTCGACCGTGAGA	0.219585798	-0.045265387	-0.388591503
B_98618_ACGCTCGAAGATCGCAAGCAGGAA	-0.071620889	0.175695412	-0.201060019
B_98618_ACTATGCACCTAATCCAGCAGGAA	0.083647973	0.014244588	-0.093011902
B_98618_ACTATGCAGCTCGGTACAAGGAGC	0.045500068	0.174912276	-0.163365348
B_98618_ACTATGCATAGGATGAAATGTTGC	0.341827967	0.211596864	0.041053563
B_98618_AGAGTCAAAGCAGGAAAAGAGATC	0.227785735	0.048124366	-0.334726448
B_98618_AGAGTCAATATCAGCAGAGCTGAA	0.054040458	0.056780255	-0.328088682

B_98618_AGATCGCAGCGAGTAACAACCACA	0.029497664	0.187947724	-0.185921362
B_98618_AGATGTACGTACGCAACACTTCGA	0.043384062	0.019416403	-0.447190457
B_98618_AGCACCTCACAGATTCCGGATTGC	0.049540112	0.067674506	-0.192780141
B_98618_AGCACCTCGAGTTAGCCTGTAGCC	0.312779797	0.123209796	-0.095516242
B_98618_AGCAGGAACTAAGGTCCCTAATCC	0.322922677	0.113456853	-0.257807833
B_98618_AGCCATGCACAGATTCAGGCTAAC	0.040848862	0.195318826	-0.127448116
B_98618_AGCCATGCAGCCATGCCGCATACA	0.006113983	0.022717417	-0.194920288
B_98618_AGCCATGCGAACAGGCAAACATCG	-0.084781827	0.152244786	-0.055224311
B_98618_AGCCATGCTTCACGCAACAGATTC	0.006904843	0.103775464	-0.132149708
B_98618_AGGCTAACAGGCTAACAGCACCTC	0.244142092	0.08285152	-0.276352071
B_98618_AGTACAAGGGTGCGAAGAACAGGC	0.186684094	0.089325753	-0.209703589
B_98618_AGTCACTAAAGGACACCACTTCGA	-0.039077731	0.0482188	-0.08932413
B_98618_AGTGGTCACTGGCATAAACGCTTA	-0.089651718	0.141118301	-0.283557839
B_98618_AGTGGTCATCTTCACAGCGAGTAA	0.142426612	0.223739823	-0.138785717
B_98618_ATAGCGACCCGAAGTACCTCCTGA	0.210733444	0.033167701	-0.326861411
B_98618_ATAGCGACCCTAATCCAGATCGCA	0.019632228	0.144413552	-0.199636298
B_98618_ATAGCGACCTGAGCCACTGAGCCA	0.156532305	0.197057896	-0.15767395
B_98618_ATCATTCCAACTCACCAGATCGCA	0.071142826	0.324281812	-0.078495766
B_98618_ATCATTCCGAGCTGAACGAACTTA	0.131051323	0.129590379	-0.236290642
B_98618_ATCCTGTAATTGGCTCAGCACCTC	0.254668627	0.244527855	-0.202697006
B_98618_ATTGGCTCCCGACAACAACGCTTA	0.31178114	0.092929432	-0.118199132
B_98618_CAACCACAACCACTGTTAGGATGA	-0.108927291	0.075595577	-0.32621992
B_98618_CAACCACAAGTACAAGACACGACC	0.305828771	0.02802876	-0.157942149
B_98618_CAACCACAGCCAAGACAGTGGTCA	0.006298036	0.044205856	-0.304934736
B_98618_CAAGACTAGAATCTGACCATCCTC	0.033141014	0.100168239	-0.461525398
B_98618_CAAGGAGCACGCTCGACGCTGATC	0.517810363	0.223200755	-0.421683551
B_98618_CAATGGAACGCATACAGCGAGTAA	0.290390739	0.228793941	-0.143615644
B_98618_CACCTTACCATCAAGTAATGTTGC	0.44618994	0.109580652	-0.267378513
B_98618_CACCTTACCTGGCATAGCCACATA	0.356516073	0.139521629	-0.042515082
B_98618_CACTTCGAATCCTGTACACCTTAC	0.075158333	0.35394875	-0.202932763
B_98618_CAGATCTGAGGCTAACGAATCTGA	-0.042664896	0.073990722	-0.337311276
B_98618_CAGCGTTAAACGTGATAGAGTCAA	0.395959074	0.088385587	-0.228007219

B_98618_CAGCGTTACCGAAGTAGGAGAACA	0.232675396	0.240152007	-0.200554567
B_98618_CATACCAAAGATGTACCTGGCATA	0.028765644	0.0654373	-0.29909721
B_98618_CATACCAACAAGACTAATCCTGTA	0.1727817	0.245602761	-0.13490378
B_98618_CATACCAACGACTGGAGTCTGTCA	0.194743942	-0.03280486	-0.206437663
B_98618_CATCAAGTTGGCTTCATTCACGCA	0.14829022	0.086970267	-0.11672373
B_98618_CCAGTTCACCGACAACAGTGGTCA	-0.00351267	0.151018278	-0.274862123
B_98618_CCATCCTCAAGGTACACGAACTTA	0.267646011	-0.077590404	-0.221318175
B_98618_CCATCCTCCCTCCTGAAGATGTAC	0.227158325	-0.005050682	-0.158604288
B_98618_CCATCCTCCTGAGCCACTGTAGCC	-0.013307518	0.056271618	-0.32084062
B_98618_CCGAAGTAAATGTTGCAAGGACAC	0.145604717	0.130951481	-0.144665928
B_98618_CCGACAACATAGCGACGTACGCAA	0.597543602	0.198547152	-0.212345415
B_98618_CCGTGAGAACGCTCGAAGCAGGAA	0.100933317	0.102306333	-0.375483529
B_98618_CCGTGAGAAGGCTAACGACTAGTA	-0.036450282	0.164964323	-0.247270626
B_98618_CCTAATCCGCTAACGACCGTGAGA	0.118600195	0.019560382	-0.168082454
B_98618_CCTCCTGACAACCACACAACCACA	0.05003035	0.106945477	-0.264795959
B_98618_CCTCCTGAGTGTTCTAACATTGGC	0.108939145	0.106694321	-0.092727786
B_98618_CGAACTTAGATAGACAATCATTCC	0.206425101	-0.017828656	-0.275922246
B_98618_CGACACACCTGTAGCCGTCGTAGA	0.114125388	0.200159544	-0.250247753
B_98618_CGACACACTTCACGCAAATGTTGC	0.189116516	0.17859592	-0.227509563
B_98618_CGACTGGAAATCCGTCCACTTCGA	0.173941372	0.299934551	-0.259982018
B_98618_CGCATACACAAGGAGCGCGAGTAA	0.300966649	0.091979069	-0.034927284
B_98618_CGCTGATCAAGGACACACACAGAA	0.215977834	0.122063046	-0.169740444
B_98618_CTAAGGTCACAAGCTAACCTCCAA	0.021247045	0.02366904	-0.298518415
B_98618_CTCAATGAGCCACATAGCCAAGAC	0.004848195	0.236345878	0.068922943
B_98618_CTGAGCCAAACAACCACGACACAC	0.439229893	0.108617964	-0.122803383
B_98618_CTGAGCCAAGTCACTAGACAGTGC	0.223502642	0.05583757	-0.326930365
B_98618_CTGAGCCATCCGTCTAACCACTGT	0.234145101	0.195403823	0.050030251
B_98618_CTGAGCCATCCGTCTACAGCGTTA	-0.0362991	0.093630394	-0.096134187
B_98618_CTGGCATACGACACACGCTCGGTA	0.27909796	0.013808245	-0.279378425
B_98618_CTGTAGCCACGCTCGAGATGAATC	0.165942667	0.076794911	-0.32235479
B_98618_GACAGTGCAGAGTCAACACCTTAC	0.317194117	0.215492526	-0.164764459
B_98618_GACAGTGCAGATCGCAACCTCCAA	-0.110866679	-0.003522856	-0.131496966

B_98618_GAGTTAGCAGAGTCAAACACAGAA	0.10518747	0.128304828	-0.110646315
B_98618_GAGTTAGCAGCCATGCCCTAATCC	0.123020436	-0.007837065	-0.152724241
B_98618_GAGTTAGCCTGGCATAAGCACCTC	0.027051427	0.114470235	-0.040601141
B_98618_GATAGACAAAGAGATCTGGTGGTA	0.181259059	0.095148843	-0.049897241
B_98618_GATGAATCGTGTTCTAATAGCGAC	0.101277569	0.171371824	-0.081368918
B_98618_GCCAAGACACATTGGCGCCACATA	0.347633223	0.177662445	-0.240606567
B_98618_GCCAAGACGACAGTGCGATAGACA	-0.067561097	0.105022979	-0.125579459
B_98618_GCCAAGACTGAAGAGAAGGCTAAC	0.256230301	0.14298416	-0.236547714
B_98618_GCCACATAAAGACGGAACATTGGC	0.201695438	0.128039153	-0.071227757
B_98618_GCCACATAGAGCTGAACGAACTTA	0.2483254	0.091196193	-0.287253269
B_98618_GCGAGTAAAATCCGTCACCTCCAA	0.006811844	0.089926765	-0.123203003
B_98618_GCGAGTAAACAGATTCTGGTGGTA	0.025510171	0.075094741	-0.215525182
B_98618_GCGAGTAAGTACGCAAAATCCGTC	-0.058503983	0.103066035	-0.313615839
B_98618_GCGAGTAATATCAGCACCATCCTC	0.100382557	0.002560971	-0.249695769
B_98618_GCTCGGTACTGGCATAAGTCACTA	0.042249596	0.072036461	-0.264398299
B_98618_GGAGAACAGCTCGGTACAAGGAGC	0.166611406	0.114649793	-0.229084275
B_98618_GGAGAACATAGGATGAAGGCTAAC	0.355914998	0.009233868	-0.13467394
B_98618_GGTGCGAAAAGACGGACAGCGTTA	0.31239363	0.282760482	-0.148908557
B_98618_GGTGCGAAAATCCGTCCGCTGATC	0.177758221	0.170507948	-0.150076887
B_98618_GGTGCGAAACAGATTCAAGAGATC	0.326082313	0.213215379	-0.292652121
B_98618_GGTGCGAAACTATGCAAGTGGTCA	0.204288991	0.022468446	-0.501310317
B_98618_GGTGCGAAAGCACCTCGTCGTAGA	0.281049577	0.118287536	-0.273283219
B_98618_GTACGCAACCTCTATCACAAGCTA	0.129235026	-0.053477434	-0.196769834
B_98618_GTACGCAACGCATACACTGAGCCA	0.124155514	0.146121317	-0.165688987
B_98618_GTCGTAGAAGATCGCACCTCCTGA	0.235466627	0.155942804	-0.101376456
B_98618_GTCGTAGAGCCAAGACGTCGTAGA	0.214084394	0.224751915	-0.197236513
B_98618_GTCTGTCAAAGACGGAAACGCTTA	0.295575511	0.157831016	-0.111483738
B_98618_GTGTTCTAAATGTTGCGTCGTAGA	0.231070077	0.17270379	-0.378880836
B_98618_GTGTTCTACCTCCTGAACACAGAA	0.226629259	0.08841197	-0.337307578
B_98618_TAGGATGACAAGGAGCTCTTCACA	0.219398628	0.231428328	-0.136159248
B_98618_TATCAGCACCAGTTCACCAGTTCA	0.268001561	0.197592186	-0.086367978
B_98618_TCCGTCTAAAGACGGAAGGCTAAC	0.137497314	0.220315127	-0.075090751

B_98618_TCCGTCTAGACAGTGCGTCTGTCA	-0.133929977	0.081151474	-0.185674513
B_98618_TCTTCACACCTAATCCGGTGCGAA	0.79440326	0.183157305	-0.252080003
B_98618_TCTTCACACGAACTTAGATAGACA	0.280464261	0.059014104	-0.313746449
B_98618_TGAAGAGAGCCAAGACACGCTCGA	0.080987878	0.072247497	-0.26217774
B_98618_TGAAGAGAGCTAACGAAGCCATGC	0.057250256	0.08477441	-0.423448441
B_98618_TGGAACAAAACAACCAGATGAATC	0.277938358	0.054158695	-0.23037042
B_98618_TGGCTTCACCGACAACATTGAGGA	0.274912366	0.156367024	-0.239262841
B_98618_TGGTGGTAGAACAGGCTGGAACAA	0.196615662	0.130511002	-0.266186584
B_98618_TGGTGGTAGAGTTAGCTATCAGCA	0.227001871	0.098383895	-0.112857392
B_98618_TTCACGCAGGAGAACAATGCCTAA	-0.03047078	-0.093886618	-0.125934429
B_98618_AACAACCAGAACAGGCATTGAGGA	0.136153826	0.15437275	-0.272644841
B_98618_AACCGAGATTCACGCACCGAAGTA	0.586490784	0.50938446	-0.162661616
B_98618_AACGCTTACGCTGATCATCCTGTA	0.884728483	0.706444478	-0.010540835
B_98618_AAGGTACAACAGATTCGTACGCAA	0.173954614	0.762839517	0.229017459
B_98618_ACACAGAACGACACACGACAGTGC	0.834128372	0.42266586	-0.363911976
B_98618_ACGTATCACGGATTGCTGAAGAGA	-0.114106953	0.036848375	-0.538301044
B_98618_ACTATGCAAATCCGTCGCTAACGA	0.354287375	0.219883517	-0.280147561
B_98618_AGAGTCAAGACAGTGCCCATCCTC	0.962910885	0.324403108	-0.336707467
B_98618_AGATGTACGTACGCAAAACGCTTA	0.063325469	0.137927123	-0.374774114
B_98618_CAACCACATGGCTTCAAGTCACTA	0.252528179	0.490702261	-0.17016596
B_98618_CAATGGAAACACGACCTATCAGCA	0.688919232	0.515658768	-0.352344275
B_98618_CACTTCGAATGCCTAACGCTGATC	0.404929518	0.39177205	-0.163159102
B_98618_CCGACAACAATGTTGCCAACCACA	0.446446199	0.281215822	-0.241047926
B_98618_CCGACAACGCTCGGTACCGTGAGA	0.696006908	1.000678492	-0.04268151
B_98618_CCTAATCCGCTCGGTACAACCACA	0.570337782	0.423494755	-0.26995413
B_98618_CCTCCTGACGCATACACCTAATCC	0.771016609	0.279754208	-0.41657183
B_98618_CGCTGATCTATCAGCAACCACTGT	0.280621724	0.153551887	0.065386511
B_98618_CGGATTGCAGATGTACGATAGACA	0.245904912	0.205669105	-0.225995176
B_98618_CTGAGCCAAGAGTCAACCTCTATC	0.485044613	0.161585383	-0.238387726
B_98618_CTGGCATAACACAGAAGAACAGGC	0.753890755	0.410691319	-0.277541727
B_98618_CTGTAGCCAAACATCGGACTAGTA	0.464008764	0.281024627	-0.18923422
B_98618_GAACAGGCAGCACCTCCGAACTTA	0.279630745	0.175398939	-0.371443637

B_98618_GCGAGTAAAAGGACACGACTAGTA	0.585887193	0.214313137	-0.282050912
B_98618_GCGAGTAAGGAGAACACAAGGAGC	0.734298804	0.489976493	-0.235117146
B_98618_GCTAACGAGTCTGTCAAAGGTACA	0.386627444	0.356415233	-0.28975197
B_98618_GGAGAACACTGGCATAACATTGGC	1.163553443	0.515648123	-0.065801503
B_98618_GTGTTCTACACCTTACGCTAACGA	0.257484414	0.291721839	-0.323250649
B_98618_TCCGTCTAACATTGGCTGGCTTCA	0.187218356	0.211851271	-0.390005256
B_98618_TCTTCACACATACCAACGAACTTA	0.841633231	0.303982345	-0.344825687
B_98618_TGGTGGTACCAGTTCAAGGCTAAC	0.453788448	0.175579926	-0.247614654
B_98618_TGGTGGTACGACACACACACAGAA	0.30974518	0.441215218	-0.16678602
B_98618_TTCACGCACCTAATCCACAGCAGA	0.223292899	0.579421558	0.146220235
B_98618_AACGCTTAAACGCTTATAGGATGA	0.629285885	0.538946136	-0.180741892
B_98618_ATGCCTAAAACCGAGACAGATCTG	0.652247412	0.64487016	-0.517969251
B_98618_CAATGGAAACCTCCAAGAACAGGC	0.435757157	1.089767277	-0.084348067
B_98618_CAATGGAAAGGCTAACCATACCAA	1.174404505	0.812273379	0.223824005
B_98618_CCAGTTCAGATGAATCCAACCACA	1.011770571	0.601618123	0.122689145
B_98618_CCTCCTGAAAGGACACAGATGTAC	0.801879352	0.840607433	-0.430808531
B_98618_CGACTGGAACAGCAGAACAGCAGA	1.47397001	1.209741949	0.174292122
B_98618_GCTCGGTAAGCAGGAAAATGTTGC	0.648245691	0.64941426	-0.582016694
B_98618_TATCAGCACACTTCGAAGCCATGC	0.8292646	0.83930377	-0.31983258
B_98618_AAACATCGCAAGGAGCCGCATACA	0.363853476	0.194664657	-0.315566637
B_98618_AAACATCGGCTAACGAATCCTGTA	0.962248242	0.148603578	-0.499529243
B_98618_AACAACCATGGCTTCAGCCAAGAC	0.565352217	0.282149069	-0.291860486
B_98618_AAGGACACACGTATCAGAATCTGA	0.019192141	0.104220624	-0.27218203
B_98618_AATGTTGCAAGGTACACGGATTGC	0.506727123	0.20834943	-0.257274552
B_98618_ACAAGCTAATCATTCCAACGTGAT	0.310046612	0.037370606	-0.128241873
B_98618_ACACGACCCAGCGTTATCTTCACA	0.593510155	0.20048704	-0.163130616
B_98618_ACAGATTCAGATCGCACATACCAA	0.436090309	0.168909906	-0.22613935
B_98618_ACAGCAGACAAGGAGCAGATCGCA	0.313584875	0.350962375	-0.380262679
B_98618_ACAGCAGACTCAATGAGTGTTCTA	0.127764181	0.221762435	0.010856502
B_98618_ACATTGGCACAGATTCAACCGAGA	0.570713475	0.14543351	-0.195221155
B_98618_AGTACAAGGCCACATAAGAGTCAA	0.137514772	0.191952916	-0.075223322
B_98618_ATCATTCCATTGGCTCCATCAAGT	0.289189333	0.251753167	-0.301519879

B_98618_CAACCACAAAGACGGAGTACGCAA	0.3909354	0.13009572	-0.277035806
B_98618_CACCTTACACTATGCAAGAGTCAA	0.03255383	0.223716266	-0.2342086
B_98618_CACCTTACCGAACTTAAGAGTCAA	-0.101636323	0.05561823	-0.335212859
B_98618_CAGATCTGGCCAAGACGATGAATC	0.063693311	0.155118209	-0.090705174
B_98618_CCGAAGTAGATAGACACAAGGAGC	0.344516057	0.17606934	-0.034241586
B_98618_CCGTGAGACAATGGAAAGAGTCAA	0.408109952	0.264878271	-0.372489803
B_98618_CCGTGAGAGACAGTGCAAGGACAC	0.195108741	0.032793678	0.004908633
B_98618_CCTCCTGAACTATGCACCGAAGTA	0.104879369	0.316839528	-0.195106111
B_98618_CCTCTATCAGCCATGCGTGTTCTA	0.323638413	0.166886125	-0.389738466
B_98618_CGCATACAAAGGTACAGACTAGTA	0.417896845	0.148394705	-0.201002915
B_98618_CGCTGATCAACGTGATGTCTGTCA	0.645417059	0.328492866	-0.402799158
B_98618_CGCTGATCCCTAATCCAGATGTAC	0.246854005	0.146152054	-0.181387873
B_98618_CTCAATGACAAGACTACAGATCTG	0.101247664	0.025252831	-0.290435701
B_98618_CTCAATGAGAATCTGATCTTCACA	0.224791732	0.108375691	-0.131162117
B_98618_CTGGCATAAATGTTGCAAGAGATC	0.211175007	-0.014424902	-0.332329916
B_98618_GACTAGTACGCTGATCAACAACCA	0.345740139	0.345769361	-0.222959781
B_98618_GAGCTGAAGAACAGGCTATCAGCA	0.602624388	0.547336844	-0.213290595
B_98618_GAGTTAGCCGAACTTACAGATCTG	0.748942743	0.415991993	-0.358870294
B_98618_GCCAAGACAACGTGATACGCTCGA	0.566419259	0.189618147	-0.104533192
B_98618_GCCAAGACACACGACCACTATGCA	0.355760215	0.299814487	-0.263200277
B_98618_GCTCGGTAATTGGCTCAACGCTTA	-0.056067782	0.068015128	-0.208207885
B_98618_GGAGAACAAACCGAGACGGATTGC	0.295903731	0.130589462	-0.269734943
B_98618_GGTGCGAATAGGATGACGAACTTA	0.190439484	0.254027574	-0.232350074
B_98618_GTCGTAGACCTCCTGATGAAGAGA	0.173747143	0.019424924	-0.167253868
B_98618_GTCTGTCACACCTTACCCTCCTGA	0.015132523	0.176577341	-0.281532024
B_98618_GTCTGTCACAGATCTGGATGAATC	0.455436076	0.087064653	-0.063569599
B_98618_TGGTGGTAGGTGCGAACGACACAC	0.060682111	0.183888899	-0.261863524
B_98618_TGGTGGTATCTTCACATGGTGGTA	0.213354776	0.242107859	-0.163171242
B_98618_TTCACGCAAGCAGGAACAAGACTA	0.339600746	0.220709873	-0.119406807
B_98618_AAACATCGCCATCCTCCTGAGCCA	0.136227326	0.0959512	-0.261336176
B_98618_ACGCTCGAAACCGAGAAGCAGGAA	0.298327495	0.089997584	-0.16259017
B_98618_CCGAAGTAAAGACGGAAAGGTACA	0.571649228	0.253147841	-0.053600981

B_98618_CCTCCTGATGGCTTCAGATGAATC	1.222036408	0.296278639	-0.201594102
B_98618_AACGTGATGCTAACGAAACCGAGA	0.960183438	0.335719502	-0.128844595
B_98618_ACGTATCACCGTGAGAAGAGTCAA	0.397829913	0.862423878	0.638565938
B_98618_ACTATGCACTCAATGACGACACAC	0.589123633	0.92275114	0.423393447
B_98618_AGCACCTCCCTCCTGACTGTAGCC	0.485650712	0.042086586	-0.476678783
B_98618_AGCCATGCGATGAATCACGCTCGA	0.591314926	0.389537566	-0.128199461
B_98618_AGTACAAGCCAGTTCAGTCTGTCA	1.125490552	0.399014268	-0.023776049
B_98618_AGTCACTACCTCCTGAACATTGGC	0.498901661	0.238688563	-0.137250235
B_98618_ATCATTCCGATGAATCACACAGAA	0.62753813	0.332396721	-0.420533868
B_98618_CAAGGAGCCACCTTACGCCAAGAC	0.464606881	0.218748042	-0.43865294
B_98618_CCAGTTCACTAAGGTCAACGTGAT	0.46778258	1.090147351	0.301763676
B_98618_CGACTGGAAAGAGATCCGCATACA	0.684470365	0.351505612	-0.319372581
B_98618_CTCAATGAACGCTCGAACGTATCA	0.997043428	0.876352838	-0.141429746
B_98618_GAACAGGCAACGTGATAGCCATGC	0.281216662	0.841842491	0.568274804
B_98618_GATAGACAAGATCGCATATCAGCA	1.235549873	0.523221053	-0.222278376
B_98618_GCTAACGACACCTTACAGCAGGAA	0.748202022	0.348662271	-0.167016431
B_98618_GCTCGGTAAAGACGGATAGGATGA	0.564031693	1.118559047	-0.207450765
ab_99547_AATGTTGCACATTGGCCAGATCTG	0.640627777	0.175932835	-0.280049094
ab_99547_AAACATCGCCTAATCCCATCAAGT	0.484304816	0.164362467	-0.25738416
ab_99547_AACAACCACTAAGGTCCGCTGATC	0.85913218	0.204666895	-0.128915848
ab_99547_AACCGAGACAGATCTGACCACTGT	0.567511534	0.177251099	-0.225718515
ab_99547_AACGTGATGGAGAACAACAAGCTA	0.600681384	0.127637453	-0.198315957
ab_99547_AACTCACCGAGCTGAATCTTCACA	0.650380088	0.135071312	-0.3204867
ab_99547_AATCCGTCCAGATCTGCACTTCGA	0.861602883	-0.001636609	-0.352233973
ab_99547_ACAAGCTAAAGGTACATCCGTCTA	0.477008464	0.200173545	-0.140576383
ab_99547_ACACAGAAATCATTCCAGAGTCAA	1.050985613	0.098159783	-0.443077791
ab_99547_ACAGATTCACAGCAGACCATCCTC	0.525009108	0.21491682	-0.242149638
ab_99547_ACAGATTCCGACACACCCGAAGTA	0.696215652	0.171317708	-0.375735907
ab_99547_ACGCTCGATATCAGCAGTCGTAGA	0.711618378	0.118656868	-0.355668204
ab_99547_AGCACCTCCATACCAAGCTCGGTA	0.574820113	0.172279751	-0.024960825
ab_99547_AGGCTAACCTGGCATAACGCTCGA	0.640275531	0.125836996	-0.197894227
ab_99547_ATCCTGTAATAGCGACGTCGTAGA	0.591677193	0.203549479	-0.299966344

ab_99547_ATTGGCTCTATCAGCAAATCCGTC	0.519840713	0.065718608	-0.253627252
ab_99547_CAAGACTAAACCGAGACGAACTTA	0.648297994	0.058477906	-0.367304622
ab_99547_CCATCCTCGAGCTGAACAGCGTTA	0.743181396	0.086650586	-0.183519441
ab_99547_CCGAAGTACTCAATGAATGCCTAA	0.65206521	0.065550456	-0.423047938
ab_99547_CTCAATGAATCCTGTACCGACAAC	0.402653771	0.071969316	-0.330465269
ab_99547_CTGGCATAGCCACATACCTAATCC	0.18456677	0.23288489	-0.230045052
ab_99547_CTGGCATAGGTGCGAAAGTGGTCA	0.759980396	0.244841733	-0.257078907
ab_99547_GAACAGGCACATTGGCCAGATCTG	0.817284298	0.380177894	-0.400021556
ab_99547_GACAGTGCGTACGCAAATAGCGAC	0.646616408	0.077019052	-0.241448968
ab_99547_GAGTTAGCCATACCAAAATGTTGC	0.438729433	0.135072329	-0.159945671
ab_99547_GCGAGTAACGGATTGCAAACATCG	0.66956052	0.142720603	-0.396675309
ab_99547_GTCGTAGACCTCTATCGACAGTGC	0.823051259	0.196333218	-0.320808615
ab_99547_GTCGTAGAGACAGTGCGAGCTGAA	0.51457513	0.26681319	-0.135129887
ab_99547_GTCTGTCAGCTAACGACCGAAGTA	0.53654964	0.11779209	-0.153485239
ab_99547_GTGTTCTAAGCCATGCGAATCTGA	0.561853671	0.094417001	-0.139081277
ab_99547_GTGTTCTAATTGGCTCACCTCCAA	0.459565926	0.191306116	-0.215310375
ab_99547_TCCGTCTAAACGTGATGTCTGTCA	0.507762166	0.128103446	-0.197873645
ab_99547_TCTTCACACGACACACGTCTGTCA	0.670074051	0.131517172	-0.188219206
ab_99547_TGGAACAAATCCTGTAAACCGAGA	0.723380324	0.147087881	-0.275374322
ab_99547_TGGCTTCAGAGTTAGCCCGTGAGA	0.565347424	0.263253511	-0.148923664
ab_99547_TGGTGGTAGTGTTCTACGGATTGC	0.721631745	0.051316529	-0.43321522
ab_99547_TTCACGCATGGTGGTAAACCGAGA	0.719424302	0.14402485	-0.290475798
ab_99547_AACGCTTAGGTGCGAAAACGTGAT	1.053447188	0.378808663	-0.40064317
ab_99547_AACGTGATAGTACAAGAAGGACAC	0.984348009	0.23428845	-0.076309498
ab_99547_AACGTGATCTAAGGTCAGCACCTC	0.734308882	0.383896535	-0.2602665
ab_99547_ACATTGGCCGCTGATCGATGAATC	0.925427845	0.427703087	-0.32307767
ab_99547_AGCCATGCGCTCGGTAGTCTGTCA	0.882000596	0.286173274	-0.344216606
ab_99547_AGGCTAACTCTTCACACACCTTAC	1.015427684	0.444566799	-0.399062704
ab_99547_ATTGGCTCGTGTTCTATTCACGCA	0.9436141	0.237032075	-0.21716052
ab_99547_CAACCACAAATCCGTCCCTCTATC	0.587190044	0.293275903	-0.171624573
ab_99547_CAAGGAGCGACTAGTAAAGGTACA	0.983849988	0.116492254	-0.320399324
ab_99547_CAATGGAAGTACGCAACATACCAA	0.720995768	0.325275779	-0.247832363

ab_99547_CACCTTACCAGATCTGAACCGAGA	0.937789223	0.596618774	-0.219004971
ab_99547_GTCTGTCAAACGTGATAACCGAGA	0.693205294	0.535652957	-0.486084155
ab_99547_TGAAGAGAACATTGGCCAAGGAGC	0.950531633	0.472535517	-0.173211886
ab_99547_AAACATCGAAGAGATCACCACTGT	0.528634772	0.094931799	-0.326052367
ab_99547_AACGTGATACAGCAGACAAGACTA	0.742569642	0.978939279	-0.369718255
ab_99547_AAGGACACCGACACACAACGTGAT	0.646367164	0.582797999	-0.475973125
ab_99547_ACGCTCGACTAAGGTCAACGCTTA	0.989618619	0.667664673	-0.373365913
ab_99547_ACGTATCAGCGAGTAAGATGAATC	1.382989143	0.616593336	-0.39384652
ab_99547_ATAGCGACACTATGCAAGTACAAG	1.573918854	0.732223467	-0.24434112
ab_99547_CAAGACTAGCTAACGAATGCCTAA	0.978675093	0.937439161	-0.537390952
ab_99547_CTGGCATAGAATCTGACAATGGAA	1.122833425	0.966176017	-0.322742424
ab_99547_GGAGAACATGAAGAGACACTTCGA	1.316713854	1.040773835	-0.213945923
ab_99547_GTACGCAAGGTGCGAAGATAGACA	0.886985498	0.525478931	-0.543595327
ab_99547_GTGTTCTAGATGAATCCAATGGAA	0.860221737	0.496183562	-0.426112618
ab_99547_TAGGATGAGGAGAACAAACTCACC	0.922435812	0.967239934	-0.31211911
ab_99547_CCATCCTCAGCACCTCTGGCTTCA	0.815314508	0.460247028	-0.38760199
ab_99547_GATAGACACCGACAACGAATCTGA	0.938475861	0.436488995	-0.534070185
ab_99547_AAGAGATCACAGCAGAAACCGAGA	0.698888442	0.28930418	-0.237771759
ab_99547_ACCACTGTAGATCGCAATTGAGGA	0.717373317	0.133275901	-0.40354665
ab_99547_ACGCTCGAATTGGCTCTAGGATGA	0.632349417	0.15361671	-0.358173881
ab_99547_AGTGGTCAACACAGAAGTACGCAA	0.741745858	0.219177654	-0.523939324
ab_99547_ATTGAGGAAACGTGATCCTCTATC	0.813923191	0.187299071	-0.318593676
ab_99547_CACCTTACCGGATTGCATGCCTAA	0.733186801	0.299764671	-0.356716708
ab_99547_CAGATCTGGCGAGTAAGGAGAACA	0.734125602	0.158987435	-0.256124359
ab_99547_CCTCCTGACTAAGGTCCGCATACA	0.981294042	0.367806493	-0.141758389
ab_99547_CGCATACAACAGCAGAATAGCGAC	0.823670154	0.265622145	-0.369273653
ab_99547_CTGGCATAACAGCAGAGACTAGTA	0.987554621	0.097646313	-0.396742256
ab_99547_GCTAACGAGAACAGGCACCACTGT	0.496091184	0.136097098	-0.336873755
ab_99547_GCTCGGTAAAGGTACAGCCACATA	0.867610765	0.128915437	-0.305645741
ab_99547_GTACGCAAGCTCGGTAAAACATCG	0.900222072	0.105415236	-0.221263618
ab_99547_TCTTCACAGCCACATACAGCGTTA	0.760879252	0.161118043	-0.320494078
ab_99547_TGAAGAGATCTTCACATATCAGCA	0.703764103	0.057629945	-0.328912685

ab_99547_AGTGGTCAGCTCGGTACGAACTTA	0.930805031	0.169277975	-0.119095131
ab_99547_CGCTGATCAATGTTGCCATCAAGT	0.818497178	0.31669801	-0.091777745
ab_99547_GATAGACACATACCAAAATCCGTC	0.925344084	0.382626732	-0.09582458
ab_99547_TAGGATGATTCACGCATATCAGCA	0.730095678	0.115336553	-0.096886429
ab_99547_ATAGCGACGTCGTAGATCCGTCTA	0.767707264	0.721632396	-0.233453805
ab_99547_TCCGTCTACCATCCTCATTGGCTC	0.840190059	0.577990333	-0.067961311
A_98763_AACCGAGAAATGTTGCATCCTGTA	0.281177346	0.625748488	1.714452389
A_98763_AAGGTACAGCCACATATCTTCACA	0.299007321	0.027763746	0.643854533
A_98763_ACAAGCTACTCAATGAGCTAACGA	0.230872019	0.465537671	1.198374857
A_98763_ACATTGGCTTCACGCAACACGACC	0.321368395	0.194651861	0.288831397
A_98763_ATGCCTAAACAGATTCCATCAAGT	0.299212407	0.22561304	0.500949323
A_98763_ATTGAGGATATCAGCAAACGTGAT	0.60508843	0.581582342	1.6485445
A_98763_ATTGGCTCGAGTTAGCCTCAATGA	0.263602711	0.243375098	0.999245729
A_98763_CACTTCGAAGATCGCAGAATCTGA	0.086546874	0.132976448	0.43978584
A_98763_CTAAGGTCCATCAAGTCCGAAGTA	0.254169175	0.557779875	0.987705889
A_98763_GACTAGTAGCCAAGACCTCAATGA	0.533862898	0.103903248	0.219793039
A_98763_TCTTCACAAGGCTAACGACAGTGC	0.042615553	0.161648092	0.724704506
A_98763_AACTCACCCCTCCTGATGGCTTCA	0.463459718	0.372678562	0.629383303
A_98763_AACTCACCCGCTGATCATCATTCC	0.68852535	0.448754093	1.117826359
A_98763_AATGTTGCGCCACATACCTCTATC	0.648996601	0.439801614	1.105139435
A_98763_AGCACCTCGTCGTAGACGACTGGA	0.21857715	0.483120814	1.013514478
A_98763_AGTACAAGAACAACCAAGATGTAC	0.776341744	0.32707414	0.535512075
A_98763_CCATCCTCGACAGTGCACGCTCGA	0.554246919	0.272385645	0.695189071
A_98763_CCGTGAGACAAGACTAACCACTGT	0.251529266	0.468397583	0.828835632
A_98763_GCTAACGACTAAGGTCCAATGGAA	0.576744117	0.482699181	1.028197628
A_98763_GCTAACGAGGTGCGAACTGGCATA	0.78191695	0.619816938	0.526377212
A_98763_GCCACATAACCTCCAAAGATCGCA	0.341863395	0.321643181	0.461817709
A_98763_GAATCTGAATTGGCTCCCATCCTC	0.536897963	0.528942863	0.595855635
A_98763_AAACATCGAAGAGATCGACTAGTA	0.224459087	0.438706061	1.260132761
A_98763_AAACATCGCTGAGCCAGACAGTGC	0.223176345	0.648236233	1.135438839
A_98763_AAACATCGTCTTCACATGGCTTCA	0.241936913	0.330679301	0.585946479
A_98763_AACAACCAACATTGGCAATGTTGC	0.340017468	0.237573938	0.877895976

A_98763_AACAACCACGAACTTATGGTGGTA	0.412909082	0.694001073	1.739071702
A_98763_AACAACCAGAATCTGACGGATTGC	0.382606278	0.153268211	0.747439292
A_98763_AACCGAGAAACCGAGAACCTCCAA	0.000776127	0.238187435	1.584144406
A_98763_AACCGAGAAAGAGATCACACAGAA	0.185098285	0.103798187	0.421099541
A_98763_AACCGAGAACATTGGCTGGCTTCA	0.55206241	0.422839877	1.137759867
A_98763_AACCGAGAACTATGCACCTCCTGA	0.041401541	0.477049051	1.859972404
A_98763_AACCGAGAAGTACAAGCCTAATCC	0.109945964	0.246672864	0.547098811
A_98763_AACCGAGACACTTCGACTGAGCCA	0.187609839	0.447873388	0.973129311
A_98763_AACGTGATACATTGGCCGACTGGA	0.484917356	0.435000335	0.710414467
A_98763_AACTCACCACACAGAAAACGTGAT	0.476753249	0.506028623	1.209842782
A_98763_AAGAGATCAACAACCAGGTGCGAA	0.176481534	0.562059834	1.573677953
A_98763_AAGAGATCACATTGGCCCGTGAGA	0.473708932	0.285963519	0.643632422
A_98763_AAGAGATCCAAGACTAAACTCACC	0.540504222	0.639239227	1.621410535
A_98763_AAGAGATCCCGAAGTATCTTCACA	0.211386896	0.350614697	1.183792145
A_98763_AAGAGATCCCTCCTGAACAGCAGA	-0.040389988	0.393591497	1.297722237
A_98763_AAGGACACAATGTTGCGTCGTAGA	0.062665958	0.454882828	1.407955896
A_98763_AAGGACACACAAGCTATATCAGCA	0.48740661	0.588504363	1.780156799
A_98763_AAGGACACATCCTGTACTGGCATA	0.378705842	0.599409286	1.410598162
A_98763_AAGGACACCATACCAACCTAATCC	0.234786954	0.232606402	0.829773626
A_98763_AAGGACACGTGTTCTAAGATCGCA	0.231580233	0.410087193	1.192064341
A_98763_AAGGACACTTCACGCACGACACAC	0.158163137	0.398084728	0.936825222
A_98763_AAGGTACACAAGACTAGGAGAACA	0.35336322	0.374119449	1.115298628
A_98763_AAGGTACACAATGGAAGATAGACA	0.292383342	0.141802323	0.881957466
A_98763_AAGGTACACACTTCGATGGTGGTA	0.325327127	0.472029699	0.896528249
A_98763_AAGGTACACCGTGAGAACACAGAA	0.323758526	0.405071852	1.143109339
A_98763_AAGGTACAGCGAGTAAACAAGCTA	0.151429074	0.308531539	1.550857998
A_98763_AATCCGTCAGTGGTCAATAGCGAC	0.203146822	0.189754621	0.29939599
A_98763_AATGTTGCACAAGCTACCTAATCC	0.297248878	0.616624482	1.387658563
A_98763_AATGTTGCCGGATTGCGACAGTGC	0.102680519	0.706247687	1.923164761
A_98763_AATGTTGCGCCACATAACACAGAA	0.234398249	0.11021731	0.625351971
A_98763_ACAAGCTACTGGCATAAGCCATGC	0.246069639	0.276624232	0.777412023
A_98763_ACACAGAAAAGGTACAAAGAGATC	0.509670367	0.339503624	0.735442876

A_98763_ACACAGAAACCACTGTGCGAGTAA	0.128118656	0.432067073	1.381586728
A_98763_ACACAGAAGCCACATATGGAACAA	0.475368257	0.796027282	1.478153978
A_98763_ACACGACCAGGCTAACGAATCTGA	0.372819185	0.251277554	0.330975276
A_98763_ACACGACCCCTCTATCTCCGTCTA	-0.000116677	0.433864037	1.551028673
A_98763_ACAGATTCAACAACCAATCATTCC	0.250411222	0.360039769	2.205868052
A_98763_ACAGATTCAACCGAGAAAGGTACA	0.480194162	0.601773361	1.524292916
A_98763_ACAGATTCACACAGAAACAGATTC	0.147762808	0.376614096	1.188878729
A_98763_ACAGCAGAACAGATTCAACAACCA	0.148010209	0.179698728	1.005363734
A_98763_ACAGCAGAACATTGGCTGGTGGTA	0.174104581	0.548910464	1.344883015
A_98763_ACAGCAGAAGATCGCAGACAGTGC	0.673189521	0.312968567	0.830898118
A_98763_ACAGCAGAATTGGCTCAGCCATGC	0.678755694	0.39098057	1.117985095
A_98763_ACAGCAGACGGATTGCGAATCTGA	0.446291851	0.377306706	1.371637455
A_98763_ACATTGGCAGATGTACACCACTGT	0.387325597	0.355986884	0.598983789
A_98763_ACATTGGCTGGTGGTAGTACGCAA	0.407707773	0.496859163	1.411325972
A_98763_ACCACTGTAAGACGGAACGCTCGA	0.450881288	0.17724555	0.409596325
A_98763_ACCACTGTCAGATCTGCGACACAC	0.376682081	0.339363779	1.097595981
A_98763_ACCACTGTCCTCCTGAAAGGTACA	0.109725525	0.096547598	0.608486631
A_98763_ACCACTGTGATAGACAGGAGAACA	0.184681705	0.46688292	1.145098498
A_98763_ACCTCCAAAGAGTCAACGACTGGA	0.216757582	0.591838372	1.943032153
A_98763_ACCTCCAACCATCCTCAGCAGGAA	0.384284655	0.474965473	1.190356707
A_98763_ACGCTCGAGACTAGTAACCACTGT	0.473441089	0.722553336	1.527546353
A_98763_ACGTATCAACGCTCGAATGCCTAA	0.396523904	0.46939124	1.341845331
A_98763_ACTATGCAGAGTTAGCAAGACGGA	0.434714664	0.809378972	1.432820179
A_98763_ACTATGCAGTCGTAGAAGCAGGAA	0.54645423	0.433125963	1.190476064
A_98763_AGAGTCAAGACTAGTAGTCGTAGA	-0.094860873	0.424765701	1.819590509
A_98763_AGATCGCAAAACATCGAAGGTACA	0.110366574	0.373288706	0.987410809
A_98763_AGATCGCAGCTCGGTACCTAATCC	0.218217576	0.322277403	1.323612276
A_98763_AGATGTACAGCAGGAAAGTCACTA	0.285051862	0.162984072	0.34658489
A_98763_AGATGTACGTACGCAAACATTGGC	0.122044176	0.217199122	0.749091848
A_98763_AGATGTACGTACGCAAGACAGTGC	0.532353949	0.438627747	1.191898465
A_98763_AGCACCTCAATCCGTCCCTCCTGA	0.366941973	0.437409329	0.424465613
A_98763_AGCACCTCGCGAGTAAACCACTGT	-0.012874479	0.531024789	1.716531809

A_98763_AGCACCTCTAGGATGAAACAACCA	0.158056425	0.325701126	1.335386927
A_98763_AGCAGGAACAAGGAGCAGCCATGC	0.2931798	0.198427185	0.822280534
A_98763_AGCCATGCAATCCGTCGAATCTGA	0.350149037	0.211979858	1.019173761
A_98763_AGCCATGCCGCTGATCAACGTGAT	0.349209391	0.608813866	1.148086156
A_98763_AGCCATGCGCCAAGACGTACGCAA	0.424049147	0.224071041	0.993580036
A_98763_AGGCTAACTGAAGAGAAAGGACAC	0.13115019	0.264439385	1.139976802
A_98763_AGTACAAGTGAAGAGAGAGTTAGC	0.256998243	0.209765621	0.59194088
A_98763_AGTCACTAAAGACGGACAATGGAA	0.443290654	0.22188607	1.335598337
A_98763_AGTCACTACAAGGAGCAACTCACC	0.517224725	0.220935469	0.407202506
A_98763_AGTCACTACGACTGGACAACCACA	0.204503257	0.405070257	0.731795696
A_98763_ATAGCGACCGACTGGAGCTAACGA	0.156400948	0.302751598	0.926446619
A_98763_ATAGCGACGACAGTGCTATCAGCA	0.061862062	0.350369803	1.72844804
A_98763_ATAGCGACGGAGAACAGACTAGTA	0.248738571	-0.003327072	1.036591205
A_98763_ATAGCGACTCTTCACAAACTCACC	0.478485241	0.157308667	0.325090818
A_98763_ATCATTCCAACAACCAAAACATCG	0.287456395	0.267300605	0.857325882
A_98763_ATCATTCCACTATGCAACACAGAA	0.57696703	0.61218318	1.130906021
A_98763_ATCATTCCCACCTTACATGCCTAA	0.379405771	0.333575859	1.190850406
A_98763_ATCATTCCGCTAACGACGACTGGA	-0.042514102	0.388143591	1.296853386
A_98763_ATCCTGTAACCACTGTAACCGAGA	0.126363438	0.444435662	1.362584819
A_98763_ATTGGCTCATTGAGGAACACAGAA	0.352214048	0.495046648	1.535741094
A_98763_ATTGGCTCCCATCCTCCTAAGGTC	0.360410586	0.261658664	0.797131888
A_98763_ATTGGCTCGAACAGGCTGGCTTCA	0.169505371	0.228292818	1.210608383
A_98763_CAAGACTAAAACATCGCCTCCTGA	0.501100765	0.393596837	1.237177848
A_98763_CAAGACTAAACGTGATGATAGACA	0.360890119	0.217388364	0.562469308
A_98763_CAAGACTAAAGACGGAGACTAGTA	0.468789084	0.526281088	1.54199199
A_98763_CAAGACTACCTAATCCAAGGTACA	0.488679513	0.725451526	1.298687912
A_98763_CAAGACTACGCTGATCCAACCACA	0.318488515	0.587666619	1.248797137
A_98763_CAAGGAGCAACAACCAACATTGGC	0.315054433	0.372050279	0.566172683
A_98763_CAAGGAGCACGTATCAAAGGTACA	0.481943272	0.513994117	1.203152565
A_98763_CAAGGAGCCCTAATCCCGCTGATC	0.603596159	0.409028087	1.419381999
A_98763_CAATGGAAAAACATCGCAGCGTTA	0.479084143	0.299962634	1.024187833
A_98763_CAATGGAAAAGGACACGACAGTGC	0.215969956	0.486507573	1.631869589

A_98763_CAATGGAACTGGCATAAAACATCG	0.164621889	0.243880053	0.435953788
A_98763_CACCTTACAACGCTTACTAAGGTC	0.389568111	0.367413982	0.852499458
A_98763_CACCTTACAGATCGCAATCATTCC	0.269878078	0.287614035	0.799103958
A_98763_CACTTCGATTCACGCAGACAGTGC	0.575779217	0.316136271	1.382169198
A_98763_CAGATCTGATTGGCTCCAATGGAA	0.322857213	0.464771773	1.002800516
A_98763_CAGATCTGTGGTGGTACGGATTGC	0.358312133	0.359220204	1.318200093
A_98763_CATACCAAAACCGAGAACCTCCAA	0.480692643	0.753519986	1.491019688
A_98763_CATACCAACAAGACTAAGATGTAC	0.447036643	0.534508561	0.945118122
A_98763_CATACCAATGAAGAGAACAGCAGA	0.110045862	0.429047902	1.153747594
A_98763_CCAGTTCAATAGCGACGCCACATA	0.104788932	0.55835114	1.836596788
A_98763_CCAGTTCACATCAAGTCGAACTTA	0.325026564	0.32916022	0.895396405
A_98763_CCAGTTCACCATCCTCACACAGAA	0.180391399	0.210857037	0.64737505
A_98763_CCAGTTCAGCTCGGTAAATGTTGC	0.387925194	0.20179412	0.324441417
A_98763_CCATCCTCCGCTGATCCGCTGATC	0.448526895	0.597655459	1.391042484
A_98763_CCATCCTCCTAAGGTCACAGATTC	0.261223585	0.472379419	1.096477741
A_98763_CCATCCTCGCTAACGAGTCTGTCA	-0.019880141	0.417549978	1.265482332
A_98763_CCATCCTCTCCGTCTAACACGACC	0.299947302	0.265247071	0.602037827
A_98763_CCGAAGTAACATTGGCAGGCTAAC	0.354428503	0.684403824	1.717196966
A_98763_CCGACAACCTGTAGCCGACAGTGC	0.22934829	0.59769263	1.755982336
A_98763_CCGACAACGTACGCAAAGATGTAC	0.944816574	0.489109826	0.743973689
A_98763_CCGTGAGAAACCGAGAAAGACGGA	0.106407253	0.315508743	1.005614639
A_98763_CCGTGAGAAGCACCTCAAACATCG	0.107336116	0.254274501	0.823891844
A_98763_CCGTGAGAATCCTGTAACCACTGT	0.186750748	0.410971437	1.820122004
A_98763_CCGTGAGACCGACAACCACTTCGA	0.349115774	0.393480085	1.303502766
A_98763_CCGTGAGACGCTGATCATCATTCC	0.190054483	0.372432283	0.800310372
A_98763_CCTAATCCCAAGGAGCGATAGACA	0.183095106	0.298731642	1.401544055
A_98763_CCTAATCCGCCACATACCGAAGTA	0.220693372	0.390156314	1.255327112
A_98763_CCTCCTGAAATCCGTCCCTCCTGA	0.188136016	0.395161045	1.168631417
A_98763_CCTCCTGAAGTCACTAACGCTCGA	0.316183238	0.067361559	0.996738505
A_98763_CCTCCTGACGCATACAGAACAGGC	0.288818665	0.524132015	0.915774641
A_98763_CCTCTATCACGCTCGAATTGAGGA	0.595090905	0.71555219	1.53610911
A_98763_CGAACTTAGACTAGTAATGCCTAA	0.431208201	0.44325672	0.84113731

A_98763_CGACACACAGAGTCAACGGATTGC	0.378470009	0.559931297	1.67494465
A_98763_CGACACACAGCAGGAATCTTCACA	0.368582888	0.378046322	0.93562709
A_98763_CGCATACAACCACTGTCTCAATGA	0.209042914	0.484636621	1.137238699
A_98763_CGCATACAATTGAGGAACCTCCAA	0.228339918	0.208935551	1.1096998
A_98763_CGCTGATCCGCTGATCAATGTTGC	0.838359226	0.469380234	1.044399823
A_98763_CGCTGATCGATAGACACGGATTGC	0.566865767	0.334648105	1.243447729
A_98763_CGGATTGCAAGAGATCACACAGAA	0.272520649	0.364651637	1.074222643
A_98763_CGGATTGCCGGATTGCCAATGGAA	0.342188233	0.055539511	0.787428266
A_98763_CTAAGGTCATTGAGGACCATCCTC	0.494311821	0.373940596	1.103477777
A_98763_CTAAGGTCCAATGGAACTGGCATA	0.054746886	0.471491171	1.727780452
A_98763_CTAAGGTCCCGAAGTAGACAGTGC	0.160809817	0.346869976	0.389079399
A_98763_CTAAGGTCGCGAGTAAAACCGAGA	0.404922301	0.568952032	1.592578412
A_98763_CTCAATGAAGCACCTCCAGCGTTA	0.033470159	0.122427388	0.494577714
A_98763_CTCAATGAATGCCTAAACACGACC	0.085996611	0.162272752	1.650653898
A_98763_CTCAATGACTGTAGCCCAATGGAA	0.227392291	0.263314617	0.71903129
A_98763_CTGGCATAAAACATCGAGTCACTA	0.249218449	0.109320809	0.232988313
A_98763_CTGGCATACAACCACAGATAGACA	0.208718112	0.483564618	1.959465321
A_98763_CTGTAGCCCAACCACAAGCACCTC	0.11195752	0.166576396	0.781172707
A_98763_GAACAGGCAAGACGGAAACAACCA	0.446389295	0.469797465	1.528523847
A_98763_GAACAGGCGCCAAGACAGCACCTC	0.278769787	0.685195471	1.538644808
A_98763_GAATCTGAGACAGTGCCGACACAC	0.128604298	0.209296072	1.095780185
A_98763_GACTAGTAAAACATCGCACTTCGA	0.303320891	0.79667479	0.950048192
A_98763_GACTAGTACAACCACAACAGCAGA	0.582456987	0.868929956	1.504216795
A_98763_GACTAGTATGGCTTCAATGCCTAA	0.373297383	0.54926286	1.163194493
A_98763_GAGCTGAAAACCGAGATGGAACAA	0.22430046	0.252397264	0.72546811
A_98763_GAGCTGAAACAGATTCCATCAAGT	0.378904879	0.196258233	1.403658686
A_98763_GAGCTGAACCAGTTCACCTCTATC	0.092477328	0.40907792	0.942093512
A_98763_GAGCTGAACTGAGCCAGACTAGTA	0.39020732	0.282821686	0.320045913
A_98763_GAGTTAGCCACTTCGAAAACATCG	0.776426536	0.930952245	1.778598776
A_98763_GAGTTAGCCGACTGGACCTCCTGA	-0.019452815	0.50291655	1.619599637
A_98763_GAGTTAGCGAGCTGAACGCATACA	0.470725038	0.451953049	0.971591832
A_98763_GATAGACAAATCCGTCAACCGAGA	0.422850867	0.274339057	1.354644859

A_98763_GATAGACAACAGCAGAATAGCGAC	0.163362147	0.087675714	1.124569096
A_98763_GATAGACACAACCACAATGCCTAA	0.048992273	0.322425947	1.210835443
A_98763_GATGAATCAGCAGGAACAGATCTG	0.228832941	0.401696786	1.311529294
A_98763_GATGAATCCTGGCATAAGTCACTA	0.132342436	0.140670703	0.648059133
A_98763_GATGAATCGCCACATACCTAATCC	-0.137910446	0.101434602	1.435722142
A_98763_GATGAATCGTACGCAAAAGACGGA	0.45035498	0.296765936	0.810487957
A_98763_GATGAATCTATCAGCAACATTGGC	0.262542029	0.014866215	0.578062847
A_98763_GCCAAGACATTGAGGACGAACTTA	0.162788109	0.964046939	1.608910778
A_98763_GCCAAGACCCTCCTGAAGCCATGC	0.280993907	0.211446895	0.670023739
A_98763_GCCACATAAACTCACCAACCGAGA	0.000559201	0.366632393	1.077015492
A_98763_GCCACATACAAGGAGCATCCTGTA	0.427024937	0.619024741	1.345750653
A_98763_GCCACATAGCTAACGATGAAGAGA	0.381344484	0.509834608	1.873779159
A_98763_GCGAGTAAGTGTTCTAACACGACC	0.543438649	0.559690122	1.882586335
A_98763_GCTAACGAACCTCCAAGAATCTGA	0.434352293	0.711954606	1.302132921
A_98763_GCTCGGTAAAACATCGACCACTGT	0.083987651	0.352342173	1.283956769
A_98763_GCTCGGTAGCTAACGAGACTAGTA	0.23997916	0.411466493	0.770147513
A_98763_GGAGAACAACACAGAAGGAGAACA	0.33154823	0.069444674	0.877138452
A_98763_GGTGCGAACCGTGAGACCGTGAGA	0.542272546	0.592477055	1.921712565
A_98763_GTACGCAAAGCCATGCATAGCGAC	0.080449369	0.078604196	0.848152952
A_98763_GTACGCAAATAGCGACCGACTGGA	-0.136576215	0.200623542	1.650001635
A_98763_GTACGCAAGCTCGGTATCTTCACA	0.491145787	0.170725214	0.69439068
A_98763_GTCGTAGAATCCTGTACAGCGTTA	0.33718474	0.376220309	1.347884375
A_98763_GTCGTAGATCTTCACACCTCCTGA	0.478995494	0.394631729	1.291782233
A_98763_GTCTGTCACGAACTTACCTCTATC	0.156381118	0.239467469	0.843482618
A_98763_GTCTGTCACTGTAGCCGTCTGTCA	0.23496003	0.180913562	0.727460531
A_98763_GTCTGTCAGAACAGGCATTGAGGA	0.390324717	0.461734068	1.244817091
A_98763_GTGTTCTAGGAGAACATCTTCACA	0.233241261	0.234526647	1.013905413
A_98763_TAGGATGAAGATCGCACAACCACA	0.439054912	0.201499489	1.042179792
A_98763_TAGGATGACACTTCGACAGATCTG	0.435477239	1.030231651	1.721644006
A_98763_TAGGATGAGATGAATCAAGAGATC	0.268794086	0.181153198	0.913319802
A_98763_TATCAGCACCATCCTCAAGACGGA	0.348078006	0.262417618	1.067331134
A_98763_TATCAGCAGCCAAGACGTCTGTCA	0.5794463	0.440115746	1.600860291

A_98763_TCCGTCTAAAACATCGCGCTGATC	0.408885751	0.202714992	0.995573705
A_98763_TCCGTCTAATTGAGGAACGCTCGA	0.666023463	0.405387863	0.930306547
A_98763_TCCGTCTACAAGGAGCTATCAGCA	0.603224862	0.41494234	1.096989638
A_98763_TCCGTCTACAGATCTGACATTGGC	-0.029741501	0.137829198	1.282342875
A_98763_TCTTCACAAAGACGGAGAACAGGC	0.302217189	0.355051263	1.509904687
A_98763_TCTTCACACAAGGAGCCGACTGGA	0.213086984	0.14030772	1.086158491
A_98763_TCTTCACATTCACGCACGAACTTA	0.221992362	0.509549514	1.124264051
A_98763_TGAAGAGACCATCCTCGACTAGTA	0.557082633	0.299959309	0.810692631
A_98763_TGAAGAGACTCAATGAAAGAGATC	0.58721416	0.709956422	1.340416918
A_98763_TGGAACAAAAGGACACAACCGAGA	0.002198808	-0.005294344	0.432042231
A_98763_TGGAACAAAGCCATGCGATAGACA	0.067284219	0.334070955	1.16805418
A_98763_TGGAACAAGAGTTAGCACCTCCAA	0.243517027	0.317862365	0.64374756
A_98763_TGGCTTCAACACAGAAACGTATCA	0.49572279	0.286351959	0.773050076
A_98763_TGGCTTCACCTAATCCCGACACAC	0.462210053	-0.02883577	0.769037966
A_98763_TGGTGGTAATCATTCCTCTTCACA	0.470309249	0.384248526	1.005947283
A_98763_TGGTGGTACTGAGCCAAGCACCTC	0.307212854	0.251685634	0.70973209
A_98763_TTCACGCAACAAGCTACTAAGGTC	0.439741582	0.451344982	0.970242203
A_98763_TTCACGCACTCAATGAACCACTGT	0.437209095	0.425627955	0.789939046
A_98763_TTCACGCAGAATCTGAAAGGTACA	0.163692989	0.102355608	0.634020056
A_98763_TTCACGCAGTACGCAAAGATGTAC	0.267581275	0.164702708	0.801713624
A_98763_TTCACGCAGTGTTCTACCGTGAGA	0.21517884	0.28681211	0.966803269
A_98763_TTCACGCATGGAACAAGCCACATA	0.288231917	0.154228512	1.028106558
A_98763_AATCCGTCACACGACCACACAGAA	0.467538334	0.793778291	1.553098723
A_98763_ACACGACCGAATCTGAGAATCTGA	0.544422098	1.005288862	1.87363038
A_98763_CAGCGTTACATACCAAAGCAGGAA	0.539897941	0.695671599	1.711499931
A_98763_CCTAATCCAGTACAAGAACGTGAT	0.615993275	0.835649835	1.434639074
A_98763_CCTCCTGAATTGGCTCCGACACAC	0.334811969	0.875299941	1.171813441
A_98763_GACTAGTAATTGAGGAAACAACCA	0.42893782	0.758567601	1.608135271
A_98763_TGGCTTCACCAGTTCAGCTCGGTA	0.403043395	0.465682973	1.121544704
A_98763_ATGCCTAAAACCGAGATATCAGCA	0.594603945	0.284347658	1.118973773
B_98618_AAACATCGACGTATCAAAACATCG	0.39184879	0.3734774	-0.075847023
B_98618_GGAGAACACGGATTGCAAGGTACA	0.393602817	-0.078572271	-0.500486386

B_98618_AACGCTTAGACAGTGCCAAGGAGC	0.310792599	-0.017063663	-0.412095516
B_98618_AACTCACCACATTGGCCCGACAAC	0.29726392	0.020816123	-0.423715782
B_98618_AAGACGGAACAAGCTACGCTGATC	0.136094209	-0.04168774	-0.396233244
B_98618_AAGACGGAGCTCGGTAACGTATCA	0.189539251	-0.006955889	-0.147996161
B_98618_AAGACGGATCCGTCTAACACGACC	0.210749513	0.165540308	-0.159809714
B_98618_AAGAGATCATTGAGGACCTCTATC	0.189055341	0.01575928	-0.023669537
B_98618_AAGGACACCTAAGGTCGCTCGGTA	0.366534241	0.027019723	-0.158698157
B_98618_AATCCGTCCAAGACTAAACGCTTA	0.24279881	0.062293337	-0.109740872
B_98618_ACAAGCTAACCTCCAAAGTGGTCA	0.207827625	0.223980415	-0.042338638
B_98618_ACACAGAAACACGACCACACGACC	0.115048825	0.000369816	-0.436520991
B_98618_ACACGACCGAGCTGAAAGTACAAG	0.491578596	0.100330496	-0.117914801
B_98618_ACAGATTCTGGAACAAAGTACAAG	0.030401798	-0.069175926	-0.402012257
B_98618_ACAGCAGATATCAGCACCAGTTCA	0.067262005	-0.089098805	-0.324396611
B_98618_ACATTGGCCATCAAGTGACTAGTA	0.208009882	-0.090668992	-0.451194212
B_98618_ACATTGGCGGAGAACACAACCACA	0.208036705	0.143371344	-0.213634016
B_98618_ACGCTCGAAAGAGATCCAAGGAGC	0.058210532	-0.121429201	-0.196371043
B_98618_ACGTATCAACTATGCAACAGCAGA	0.339404721	-0.045416251	-0.14222274
B_98618_ACGTATCACTCAATGACGACTGGA	0.048096387	0.039563867	-0.347513192
B_98618_AGATCGCACTGGCATAAGTACAAG	0.272470197	0.068005087	-0.130419127
B_98618_AGCACCTCATTGAGGATCCGTCTA	0.245608457	0.335183109	0.044499555
B_98618_AGCACCTCGAATCTGACGACACAC	0.209533387	0.424582789	-0.280459889
B_98618_AGCACCTCTCCGTCTAATAGCGAC	0.266826119	0.196961113	-0.349695226
B_98618_AGCAGGAAAATGTTGCTGGAACAA	0.170453511	0.121520959	-0.154347715
B_98618_AGCAGGAACAACCACAAGTCACTA	0.058231145	-0.017850164	-0.511758001
B_98618_AGCAGGAAGCGAGTAACACTTCGA	0.272265349	0.092129267	-0.388082329
B_98618_AGCCATGCCTGAGCCACAAGACTA	0.188734275	0.00202761	-0.335490011
B_98618_AGCCATGCGCCACATAACCACTGT	0.315576225	0.271961653	0.080603981
B_98618_AGCCATGCTCCGTCTACGCTGATC	0.263030871	-0.024822476	-0.262857258
B_98618_AGGCTAACACTATGCAACACGACC	0.284091035	0.241797194	-0.23372552
B_98618_AGGCTAACCTCAATGAGTGTTCTA	0.472476852	0.047404509	-0.296845194
B_98618_AGTACAAGTGGCTTCACCTAATCC	0.298285145	0.122235041	-0.224868714
B_98618_AGTCACTACAGATCTGAAGAGATC	0.326449716	0.081340613	-0.272761164

B_98618_ATAGCGACACCACTGTGCCACATA	0.118718358	-0.026806901	-0.328990751
B_98618_ATAGCGACGCCAAGACCGCTGATC	0.132994543	0.024868716	-0.305563109
B_98618_ATAGCGACTTCACGCAGTCTGTCA	0.335878241	0.241220239	0.081712327
B_98618_ATCATTCCACAAGCTAAACAACCA	0.271954566	0.12821099	-0.241734881
B_98618_ATCATTCCAGATGTACACACGACC	0.207937665	0.013735445	-0.235485173
B_98618_ATCCTGTACGAACTTACTAAGGTC	0.202058855	0.054952514	0.008226686
B_98618_ATGCCTAAGACAGTGCAGATCGCA	0.356568336	-0.075299103	-0.192591158
B_98618_ATTGAGGAGCCACATAATTGAGGA	0.081149076	0.016187086	-0.411299168
B_98618_ATTGGCTCCTAAGGTCATTGAGGA	0.321990026	0.138815868	0.017451027
B_98618_ATTGGCTCTTCACGCAAGCCATGC	0.304952686	0.144110459	-0.092866743
B_98618_CAACCACACAGATCTGCCTCTATC	0.341573636	0.019594103	-0.2727323
B_98618_CAACCACAGCCAAGACCAAGGAGC	0.721645645	0.170008221	-0.210336064
B_98618_CAAGGAGCCTGAGCCAGCCAAGAC	0.339504369	-0.037948751	-0.392823143
B_98618_CAATGGAACAGCGTTACAAGACTA	0.27051628	-0.135378154	-0.48752067
B_98618_CAATGGAATGGTGGTACGAACTTA	0.329421626	0.084790956	0.013894781
B_98618_CACTTCGAAAGGTACATCCGTCTA	0.60690598	0.068603677	-0.116140589
B_98618_CACTTCGAAGCACCTCTCTTCACA	0.20666551	0.055949972	-0.072250125
B_98618_CACTTCGAAGGCTAACTATCAGCA	0.243902076	0.11951694	-0.314019559
B_98618_CACTTCGACTGAGCCAACACAGAA	0.276939776	0.108816933	-0.061993196
B_98618_CAGATCTGACATTGGCACAGATTC	0.292044699	0.107406101	-0.410794193
B_98618_CAGCGTTACAGATCTGAAGACGGA	0.197121655	0.109664136	-0.161716961
B_98618_CATACCAAGACTAGTAGTCGTAGA	0.04909463	0.221137431	0.01597467
B_98618_CCATCCTCACGTATCAGCGAGTAA	0.158663597	-0.024984524	-0.368205689
B_98618_CCGAAGTAAACGCTTAACACGACC	0.345970901	0.115835703	-0.43287941
B_98618_CCGACAACGAGTTAGCACATTGGC	0.190712031	-0.10041924	-0.300172172
B_98618_CCTAATCCAGCACCTCCACTTCGA	0.293508364	0.040414778	-0.158129825
B_98618_CCTCTATCCAATGGAAACCACTGT	0.125571501	0.084922842	-0.207371699
B_98618_CGAACTTAACCACTGTTGGTGGTA	0.376867268	0.160335464	-0.188367938
B_98618_CGACACACGAGCTGAAATCATTCC	0.415136568	0.082706778	-0.271829909
B_98618_CGCATACACTCAATGAGTACGCAA	0.134151579	0.219269254	-0.002315997
B_98618_CGCTGATCCTGAGCCAGTGTTCTA	0.371308484	0.169284464	0.025137808
B_98618_CGCTGATCGAGCTGAAGCGAGTAA	0.285469971	-0.202289774	-0.382102772

B_98618_CGGATTGCCGAACTTAAAGAGATC	0.239723159	-0.001750202	-0.261261196
B_98618_CGGATTGCCTGAGCCATAGGATGA	0.105390865	-0.006837288	-0.289494467
B_98618_CGGATTGCGTCGTAGACAACCACA	0.210084384	0.042373545	-0.167272265
B_98618_CTCAATGACCATCCTCGAATCTGA	0.338708635	-0.000754988	-0.262409783
B_98618_CTGAGCCAAAGGACACGCCAAGAC	0.268991723	0.204768762	-0.099263075
B_98618_CTGGCATACCTCTATCATTGAGGA	0.253205787	0.030457199	-0.388347864
B_98618_CTGGCATACTCAATGAACAAGCTA	0.366012307	0.034015963	-0.257184401
B_98618_GAACAGGCAGATCGCACCAGTTCA	0.383327539	0.118008686	-0.421938626
B_98618_GAACAGGCCCAGTTCACCGTGAGA	-0.025584622	-0.01731569	-0.109126942
B_98618_GAACAGGCGAATCTGACCTCTATC	0.349796759	0.093565022	-0.171728251
B_98618_GAACAGGCGATGAATCCTAAGGTC	0.423127368	0.093973485	-0.281510514
B_98618_GAATCTGAACCACTGTACAAGCTA	0.525692916	0.117202162	-0.211192124
B_98618_GAATCTGACCGTGAGAAGTCACTA	-0.003084343	0.033029793	-0.070159736
B_98618_GAATCTGACCTAATCCAGCACCTC	0.120157469	0.092930297	-0.132972953
B_98618_GACAGTGCCTAAGGTCAGAGTCAA	0.1438267	0.270408274	-0.11607478
B_98618_GACAGTGCTGGCTTCAAATGTTGC	0.398034621	0.088377918	0.042422954
B_98618_GACTAGTACACCTTACCAATGGAA	0.27325205	0.369788958	0.211552531
B_98618_GAGCTGAAACAGCAGAATGCCTAA	0.380919705	-0.068636149	-0.213695096
B_98618_GAGCTGAACTCAATGACAACCACA	0.363429089	0.107011263	0.129553544
B_98618_GAGCTGAATATCAGCACACCTTAC	0.240946298	-0.016043896	-0.422980544
B_98618_GAGTTAGCGATGAATCAGTCACTA	0.199860119	0.156440478	-0.144120025
B_98618_GATGAATCAACGCTTAAACCGAGA	0.401724329	0.109696224	0.004161053
B_98618_GCCAAGACGGAGAACACCAGTTCA	0.35691786	-0.003376275	-0.366018113
B_98618_GCGAGTAAAGTCACTAAACGTGAT	0.160738814	-0.003583028	0.245150808
B_98618_GCGAGTAAATAGCGACGACTAGTA	0.289207211	0.143845506	0.161507644
B_98618_GCGAGTAACCTCCTGAGCCACATA	0.338310624	0.05827421	-0.075256819
B_98618_GCTAACGAAACGCTTAAGCACCTC	0.173323172	-0.011512885	-0.372521409
B_98618_GCTAACGACGCTGATCACATTGGC	0.268320811	0.112412357	-0.128982948
B_98618_GCTCGGTAGAACAGGCACCTCCAA	0.336977089	-0.012590247	-0.317898668
B_98618_GCTCGGTAGCTAACGAAAGACGGA	0.185342457	-0.07258317	-0.240611498
B_98618_GGAGAACAAACCGAGAAACTCACC	0.244174486	0.153644505	-0.135519278
B_98618_GGAGAACATATCAGCACACCTTAC	0.287617322	0.215322401	0.139901355

B_98618_GGTGCGAACATCAAGTAGATCGCA	0.260997477	0.228378551	-0.06567863
B_98618_GGTGCGAACTCAATGAGGAGAACA	0.322834192	0.080806001	-0.350794025
B_98618_GTACGCAAAAGGACACAACGTGAT	0.412694681	0.014239707	-0.288999933
B_98618_GTACGCAAGAACAGGCGACAGTGC	0.436244978	0.093867444	-0.254699351
B_98618_GTCTGTCACGCTGATCGGAGAACA	0.316545506	0.021318426	-0.090802367
B_98618_GTCTGTCAGAATCTGACCAGTTCA	0.416867132	-0.001536253	-0.386186538
B_98618_GTGTTCTAAAGGACACAATGTTGC	0.054934069	0.039047724	-0.211292586
B_98618_TAGGATGAAATGTTGCCCAGTTCA	0.149209717	0.098648419	-0.280074074
B_98618_TATCAGCACGACACACACATTGGC	0.027217853	0.031675942	-0.309620757
B_98618_TGGAACAAGCCACATACTGTAGCC	0.107976939	0.035416424	-0.234298799
B_98618_TGGAACAAGCTAACGATAGGATGA	0.208596862	0.060281161	-0.0625146
B_98618_TGGTGGTAACGCTCGAAGTACAAG	0.30223235	-0.049328582	-0.047968203
B_98618_TGGTGGTATAGGATGACCAGTTCA	0.485237928	0.257975332	-0.278762819
B_98618_TTCACGCAATGCCTAACACTTCGA	0.370139956	0.105845522	-0.324129779
ab_99547_AACGCTTAACGCTCGAGTCGTAGA	0.483952631	0.154012117	-0.194143384
ab_99547_AATCCGTCCAACCACAAAGGTACA	0.498544865	0.027082108	-0.239187034
ab_99547_ACAAGCTACATCAAGTCGACTGGA	0.552213371	0.055633074	-0.412160583
ab_99547_ACACAGAACCAGTTCAAACGCTTA	0.503347477	0.100155726	-0.347412131
ab_99547_ACATTGGCGCCACATAAGAGTCAA	0.50544734	0.26249751	-0.263534973
ab_99547_ACCACTGTGTACGCAAACGCTCGA	0.608126507	0.121492548	-0.358683835
ab_99547_AGAGTCAAAGTGGTCACAAGACTA	0.46043668	-0.131966812	-0.369088964
ab_99547_AGATCGCAAAGACGGAACAGATTC	0.426264307	0.117065242	-0.322787371
ab_99547_ATGCCTAAAGCAGGAAACAAGCTA	0.416864764	0.085634544	-0.234363359
ab_99547_CACTTCGATTCACGCAGAGTTAGC	0.584876304	0.122656495	-0.385660187
ab_99547_CCGAAGTAGCGAGTAAACGCTCGA	0.446178384	0.091032677	-0.250132297
ab_99547_GAATCTGACCTCTATCAATGTTGC	0.487350364	0.007540588	-0.468007496
ab_99547_GGTGCGAATATCAGCAATAGCGAC	0.511298343	0.011555295	-0.296574371
ab_99547_TATCAGCAAGATGTACACCACTGT	0.605421063	0.04021514	-0.255442586
ab_99547_AAGGTACACAAGACTAAACCGAGA	0.734930449	0.103532872	-0.475533971
ab_99547_AGCAGGAACCGTGAGAATGCCTAA	0.433569241	0.094024448	-0.337833737
ab_99547_AGGCTAACGCTCGGTAGCGAGTAA	0.256644405	-0.07723445	-0.324097022
ab_99547_ATAGCGACGAATCTGAACAAGCTA	0.712685116	0.17106754	-0.432890413

ab_99547_CAGATCTGGTACGCAACAGCGTTA	0.4804072	0.02384402	-0.424353222
ab_99547_CCAGTTCAAGCACCTCACGCTCGA	0.315526403	-0.058087287	-0.309672932
ab_99547_TCTTCACACAACCACAAGTACAAG	0.439212015	-0.015670651	-0.280641992
ab_99547_CGGATTGCATAGCGACCACTTCGA	0.659477876	0.380730955	-0.389018469
ab_99547_AGATCGCAACAAGCTAAGCACCTC	0.62242723	0.126532286	-0.196216998
ab_99547_GTCTGTCAACAGCAGAATGCCTAA	1.00589963	0.213556335	-0.535081797
ab_99547_AAACATCGTGGAACAAGGAGAACA	0.683525382	0.21438131	-0.448245149
ab_99547_AAGACGGATGAAGAGAGAGTTAGC	0.622922765	0.004698031	-0.491660321
ab_99547_ACAAGCTACGGATTGCGAACAGGC	0.433261912	0.057126915	-0.161039776
ab_99547_ACATTGGCCTCAATGACAATGGAA	0.499053056	0.091837386	-0.345969303
ab_99547_AGAGTCAAGACAGTGCATTGAGGA	0.57592125	0.100821809	-0.296994888
ab_99547_AGCACCTCCTAAGGTCCGGATTGC	0.435767196	0.164769652	-0.180725633
ab_99547_CGGATTGCTGGCTTCAAGATGTAC	0.629026351	0.097765306	-0.358284616
ab_99547_GACAGTGCATGCCTAAAGTCACTA	0.811616287	0.248132857	-0.273778403
ab_99547_GGAGAACAAATCCGTCCCTCTATC	0.394454081	0.11183444	-0.293894944
ab_99547_GTCGTAGACAGATCTGCCGTGAGA	0.721337278	0.118786127	-0.549694963
ab_99547_TGGAACAATATCAGCACACTTCGA	0.615372439	-0.072072694	-0.312744432

2
