# Supplementary material for: Multiomics Reveals IL-17 Drives Epithelial Keratinization and Proliferation via EHF in Odontogenic Keratocysts
Source: Int J Mol Sci. 2026 May 4;27(9):4115. doi: 10.3390/ijms27094115 (PMC13163638; doi:10.3390/ijms27094115)
Supplement: Supplementary file 1 [file ijms-27-04115-s001.zip › ijms-4235677-supplementary/Supplementary Table S15.pdf]

Supplementary Table S15. Detailed reagents and conditions for IHC deparaffinization, rehydration, antigen retrieval, and blocking.

Procedure	Reagent / Buffer	Manufacturer (Catalog No.)	Incubation Time & Temperature
Deparaffinization	Xylene	Sinopharm Chemical Reagent Co., Ltd. (10023418)	2 × 10 min, room temperature
Rehydration	Graded ethanol (100%, 95%, 75%, 50%)	Sinopharm Chemical Reagent Co., Ltd. (10009218)	5 min each concentration, room temperature
Antigen retrieval	Citrate antigen retrieval buffer (powder, pH 6.0)	Servicebio (G1201-1L)	20 min at 95–100°C (pressure cooker or water bath)
Blocking	UltraSensitive SP IHC Kit (ready-to-use)	Maxin (KIT-97 series)	Follow manufacturer's instructions
