# Supplementary material for: Multiomics Reveals IL-17 Drives Epithelial Keratinization and Proliferation via EHF in Odontogenic Keratocysts
Source: Int J Mol Sci. 2026 May 4;27(9):4115. doi: 10.3390/ijms27094115 (PMC13163638; doi:10.3390/ijms27094115)
Supplement: Supplementary file 1 [file ijms-27-04115-s001.zip › ijms-4235677-supplementary/Supplementary Table S2.pdf]

Supplementary Table S2. All EpC GO enrichment.

ONTOLOGY	ID	Description	GeneRatio	BgRatio	pvalue	p.adjust	qvalue	geneID	Count
BP	GO:0008544	epidermis development	27/111	362/18903	1.79273422035172e-22	3.22512886241274e-19	2.75326234472964e-19	S100A7/SPRR1B/KRT6A/KRT17/SPRR3/KRT14/CSTA/KRT5/DSP/SFN/KRT6B/KRT16/KRTDAP/SOSTDC1/TGM3/AKR1C3/ANXA1/COL17A1/SPRR1A/FABP5/TP63/KRT8/SPINK5/ALDH3A2/CNFN/LAMB3/SPRR2D	27
BP	GO:0043588	skin development	25/111	302/18903	6.1679777610725e-22	5.54809599608471e-19	4.73635765968672e-19	S100A7/SPRR1B/KRT6A/KRT17/SPRR3/CSTA/KRT5/DSP/SFN/KRT6B/KRT16/CLDN4/SOSTDC1/TGM3/AKR1C3/ANXA1/SPRR1A/TP63/KRT8/ITGA2/STMN1/SPINK5/JUP/CNFN/SPRR2D	25
BP	GO:0030216	keratinocyte differentiation	19/111	170/18903	2.72921069188667e-19	1.63661667823471e-16	1.39716435068865e-16	S100A7/SPRR1B/KRT6A/KRT17/SPRR3/CSTA/KRT5/DSP/SFN/KRT6B/KRT16/TGM3/AKR1C3/ANXA1/SPRR1A/TP63/KRT8/CNFN/SPRR2D	19
BP	GO:0009913	epidermal cell differentiation	20/111	235/18903	6.78583133383339e-18	3.05192764239157e-15	2.60540208317445e-15	S100A7/SPRR1B/KRT6A/KRT17/SPRR3/CSTA/KRT5/DSP/SFN/KRT6B/KRT16/TGM3/AKR1C3/ANXA1/SPRR1A/TP63/KRT8/SPINK5/CNFN/SPRR2D	20
BP	GO:0031424	keratinization	13/111	84/18903	2.11922469185618e-15	7.62497044129853e-13	6.50936594824877e-13	SPRR1B/KRT6A/KRT17/SPRR3/KRT5/SFN/KRT6B/KRT16/TGM3/SPRR1A/KRT8/CNFN/SPRR2D	13
BP	GO:0045104	intermediate filament cytoskeleton organization	11/111	89/18903	4.29866866859805e-12	1.25247152626751e-09	1.06922322742126e-09	KRT6A/KRT17/KRT14/KRT5/DSP/KRT19/KRT6B/KRT16/PKP1/KRT8/KRT18	11

BP	GO:0045103	intermediate filament-based process	11/111	90/18903	4.8734300632977e-12	1.25247152626751e-09	1.06922322742126e-09	KRT6A/KRT17/KRT14/KRT5/DSP/KRT19/KRT6B/KRT16/PKP1/KRT8/KRT18	11
BP	GO:0045109	intermediate filament organization	10/111	69/18903	8.20929712257883e-12	1.84606569043991e-09	1.57596901340033e-09	KRT6A/KRT17/KRT14/KRT5/DSP/KRT19/KRT6B/KRT16/PKP1/KRT8	10
BP	GO:0018149	peptide cross-linking	8/111	36/18903	2.88865007808065e-11	5.77409054496343e-09	4.9292870923037e-09	SPRR1B/SPRR3/PI3/CSTA/DSP/TGM3/ANXA1/SPRR1A	8
BP	GO:0052547	regulation of peptidase activity	18/111	459/18903	1.84401993702779e-10	3.317391866713e-08	2.8320264085511e-08	S100A8/SERPINB3/S100A9/PI3/PERP/CSTA/SERPINB4/SFN/SLPI/SERPINB5/CSTB/CLDN4/SOX2/SPINT2/TP63/SPINK5/SERPINB13/CRYAB	18
BP	GO:0052548	regulation of endopeptidase activity	17/111	428/18903	5.11228365267995e-10	8.36090753742839e-08	7.13762856388521e-08	S100A8/SERPINB3/S100A9/PI3/PERP/CSTA/SERPINB4/SFN/SLPI/SERPINB5/CSTB/SOX2/SPINT2/TP63/SPINK5/SERPINB13/CRYAB	17
BP	GO:0006692	prostanoid metabolic process	7/111	50/18903	1.61293625794035e-08	2.23205563694976e-06	1.90548502051414e-06	GSTA1/GSTP1/AKR1C2/AKR1C3/CBR1/ANXA1/FABP5	7
BP	GO:0006693	prostaglandin metabolic process	7/111	50/18903	1.61293625794035e-08	2.23205563694976e-06	1.90548502051414e-06	GSTA1/GSTP1/AKR1C2/AKR1C3/CBR1/ANXA1/FABP5	7
BP	GO:0010951	negative regulation of endopeptidase activity	12/111	252/18903	2.97704252300108e-08	3.82549964205639e-06	3.26579326395382e-06	SERPINB3/PI3/CSTA/SERPINB4/SFN/SLPI/SERPINB5/CSTB/SPINT2/SPINK5/SERPINB13/CRYAB	12

BP	GO:0010466	negative regulation of peptidase activity	12/111	263/18903	4.7652559562168e-08	5.71513031015601e-06	4.8789532913125e-06	SERPINB3/PI3/CSTA/SERPINB4/SFN/SLPI/SERPINB5/CSTB/SPINT2/SPINK5/SERPINB13/CRYAB	12
BP	GO:0045861	negative regulation of proteolysis	13/111	352/18903	1.50823041805166e-07	1.69581657629684e-05	1.4477027499588e-05	SERPINB3/PI3/CSTA/SERPINB4/SFN/SLPI/SERPINB5/CSTB/IL1R2/SPINT2/SPINK5/SERPINB13/CRYAB	13
BP	GO:0051346	negative regulation of hydrolase activity	13/111	373/18903	2.92956276172513e-07	3.1001667107903e-05	2.64658332467923e-05	SERPINB3/PI3/CSTA/SERPINB4/SFN/SLPI/SERPINB5/CSTB/ANXA1/SPINT2/SPINK5/SERPINB13/CRYAB	13
BP	GO:0098754	detoxification	9/111	154/18903	3.11322464708255e-07	3.11149507783417e-05	2.65625424566868e-05	S100A9/GSTA1/GPX2/MT1X/GSTP1/NFE2L2/TXN/AKR1B10/GSTM3	9
BP	GO:0051238	sequestering of metal ion	5/111	26/18903	3.80126796085792e-07	3.46171079508398e-05	2.95523012786491e-05	S100A8/S100A7/S100A9/LCN2/ANXA1	5
BP	GO:1990748	cellular detoxification	8/111	115/18903	3.84848337419008e-07	3.46171079508398e-05	2.95523012786491e-05	S100A9/GSTA1/GPX2/GSTP1/NFE2L2/TXN/AKR1B10/GSTM3	8
BP	GO:0045216	cell-cell junction organization	10/111	208/18903	4.11093862970327e-07	3.52170409277914e-05	3.00644584498099e-05	PERP/DSP/CLDN4/PKP1/CDH1/JUP/GJB2/HOPX/CD9/CLDN7	10
BP	GO:0061436	establishment of skin barrier	5/111	27/18903	4.64342778020881e-07	3.79705753481621e-05	3.24151250302615e-05	SFN/KRT16/CLDN4/TP63/STMN1	5

BP	GO:0097237	cellular response to toxic substance	8/111	123/18903	6.44590729517397e-07	5.04182053218173e-05	4.3041550314228e-05	S100A9/GSTA1/GPX2/GSTP1/NFE2L2/TXN/AKR1B10/GSTM3	8
BP	GO:0033561	regulation of water loss via skin	5/111	31/18903	9.59187759318776e-07	7.18991157922699e-05	6.13796026686883e-05	SFN/KRT16/CLDN4/TP63/STMN1	5
BP	GO:0009636	response to toxic substance	10/111	250/18903	2.18781562867829e-06	0.000157435212639689	0.000134400968515437	S100A9/GSTA1/GPX2/MT1X/GSTP1/NFE2L2/TXN/AKR1B10/CDH1/GSTM3	10
BP	GO:0042742	defense response to bacterium	12/111	382/18903	2.54848990808479e-06	0.000176335897870944	0.000150536306716426	S100A8/S100A7/S100A9/KRT6A/PI3/SLPI/SPRR2A/S100A14/GBP6/LCN2/PLAC8/SPINK5	12
BP	GO:0033559	unsaturated fatty acid metabolic process	7/111	115/18903	5.17360336183666e-06	0.000320941808549798	0.000273985020142275	GSTA1/GSTP1/AKR1C2/AKR1C3/CBR1/ANXA1/FABP5	7
BP	GO:0042303	molting cycle	7/111	115/18903	5.17360336183666e-06	0.000320941808549798	0.000273985020142275	KRT17/KRT14/KRT16/SOSTDC1/TGM3/TP63/SPINK5	7
BP	GO:0042633	hair cycle	7/111	115/18903	5.17360336183666e-06	0.000320941808549798	0.000273985020142275	KRT17/KRT14/KRT16/SOSTDC1/TGM3/TP63/SPINK5	7
BP	GO:0019730	antimicrobial humoral response	7/111	125/18903	8.97383118590451e-06	0.000538130743448074	0.000459397182464375	S100A7/S100A9/KRT6A/PI3/SLPI/SPRR2A/SPINK5	7

BP	GO:0006690	icosanoid metabolic process	7/111	126/18903	9.45613189052802e-06	0.0005487606861623223	0.0004684718651368 55	GSTA1/GSTP1/AKR1C2/AKR1C3/CBR1/ANXA1/FABP5	7
BP	GO:0002934	desmosome organization	3/111	10/18903	2.29484271762943e-05	0.001179549156861 53	0.00100697008271318	PERP/DSP/JUP	3
BP	GO:0030638	polyketide metabolic process	3/111	10/18903	2.29484271762943e-05	0.00117954915686153	0.00100697008271318	AKR1C2/AKR1C3/AKR1B10	3
BP	GO:0030647	aminoglycoside antibiotic metabolic process	3/111	10/18903	2.29484271762943e-05	0.00117954915686153	0.00100697008271318	AKR1C2/AKR1C3/AKR1B10	3
BP	GO:0044598	doxorubicin metabolic process	3/111	10/18903	2.29484271762943e-05	0.00117954915686153	0.00100697008271318	AKR1C2/AKR1C3/AKR1B10	3
BP	GO:0098869	cellular oxidant detoxification	6/111	100/18903	2.72479423189767e-05	0.0013616402286622	0.00116241952758442	S100A9/GSTA1/GPX2/GSTP1/NFE2L2/TXN	6
BP	GO:0034113	heterotypic cell-cell adhesion	5/111	61/18903	2.9195953022666e-05	0.00141955458075071	0.00121186046828079	PERP/DSP/DSC2/JUP/IL1RN	5
BP	GO:0031069	hair follicle morphogenesis	4/111	31/18903	3.13530178001112e-05	0.00147407369876959	0.00125840292940805	KRT17/SOSTDC1/TGM3/TP63	4

BP	GO:0007043	cell-cell junction assembly	7/111	152/18903	3.19560168160167e-05	0.00147407369876959	0.00125840292940805	CLDN4/PKP1/JUP/GJB2/HOPX/CD9/CLDN7	7
BP	GO:0001516	prostaglandin biosynthetic process	4/111	32/18903	3.56703754934094e-05	0.00156514647591813	0.00133615090744247	AKR1C3/CBR1/ANXA1/FABP5	4
BP	GO:0046457	prostanoid biosynthetic process	4/111	32/18903	3.56703754934094e-05	0.00156514647591813	0.00133615090744247	AKR1C3/CBR1/ANXA1/FABP5	4
BP	GO:0050891	multicellular organismal water homeostasis	5/111	64/18903	3.68984643841863e-05	0.00158048422445598	0.00134924459991298	SFN/KRT16/CLDN4/TP63/STMN1	5
BP	GO:0098911	regulation of ventricular cardiac muscle cell action potential	3/111	12/18903	4.17129329367131e-05	0.00174515270588713	0.00148982054234184	DSP/DSC2/JUP	3
BP	GO:0098742	cell-cell adhesion via plasma-membrane adhesion molecules	9/111	282/18903	4.31331994280471e-05	0.00176355967661492	0.00150553440108901	DSG3/DSC2/CLDN4/DSC3/LGALS7B/CDH1/MPZL2/IL1RN/CLDN7	9

BP	GO:0120254	olefinic compound metabolic process	7/111	160/18903	4.43497255297218e-05	0.00177300347173266	0.00151359648065179	GSTA1/GSTP1/AKR1C2/AKR1C3/CBR1/AKR1B10/ADH7	7
BP	GO:0048730	epidermis morphogenesis	4/111	35/18903	5.12387576000926e-05	0.00200388097657753	0.00171069444710607	KRT17/SOSTDC1/TGM3/TP63	4
BP	GO:0030104	water homeostasis	5/111	69/18903	5.31360869919715e-05	0.00203386852124589	0.00173629453351145	SFN/KRT16/CLDN4/TP63/STMN1	5
BP	GO:0042246	tissue regeneration	5/111	73/18903	6.97062172508143e-05	0.00261253093404614	0.00223029322300303	ANXA1/KLF5/SOX2/HOPX/CD9	5
BP	GO:0043403	skeletal muscle tissue regeneration	4/111	39/18903	7.904825648899e-05	0.00284415626847386	0.00242802960457761	ANXA1/KLF5/HOPX/CD9	4
BP	GO:1901661	quinone metabolic process	4/111	39/18903	7.904825648899e-05	0.00284415626847386	0.00242802960457761	AKR1C2/AKR1C3/CBR1/AKR1B10	4
BP	GO:0007162	negative regulation of cell adhesion	9/111	308/18903	8.50024734035372e-05	0.00294649210694597	0.00251539275271886	TACSTD2/EPCAM/ANXA1/SPINT2/LGALS7B/CDH1/CD9/IL1RN/CLDN7	9
BP	GO:0086069	bundle of His cell to Purkinje myocyte	3/111	15/18903	8.51681987555254e-05	0.00294649210694597	0.00251539275271886	DSP/DSC2/JUP	3

		communication							
BP	GO:0050878	regulation of body fluid levels	10/111	390/18903	0.0001008187075266638	0.00342212933661173	0.00292143980697844	SFN/KRT16/CLDN4/NFE2L2/TP63/ITGA2/STMN1/HSPB1/AGR2/CD9	10
BP	GO:0017014	protein nitrosylation	3/111	17/18903	0.0001261990186487 23	0.00412785517361915	0.00352391135327249	S100A8/S100A9/TXN	3
BP	GO:0018119	peptidyl-cysteine S-nitrosylation	3/111	17/18903	0.000126199018648723	0.00412785517361915	0.00352391135327249	S100A8/S100A9/TXN	3
BP	GO:0022408	negative regulation of cell-cell adhesion	7/111	202/18903	0.000190621250096905	0.00612370765936306	0.00522775195284556	EPCAM/ANXA1/SPINT2/LGALS7B/CDH1/CD9/IL1RN	7
BP	GO:0001942	hair follicle development	5/111	91/18903	0.000198484887832959	0.00626446163528936	0.00534791230560087	KRT17/SOSTDC1/TGM3/TP63/SPINK5	5
BP	GO:0000302	response to reactive oxygen species	7/111	204/18903	0.000202494577911893	0.00628082320109475	0.00536188002129677	S100A7/GSTP1/AKR1C3/NFE2L2/ANXA1/TXN/CRYAB	7
BP	GO:0034329	cell junction assembly	10/111	430/18903	0.000222682150341585	0.00668797769957046	0.00570946400846862	CLDN4/COL17A1/PKP1/CDH1/ITGA2/JUP/GJB2/HOPX/CD9/CLDN7	10

BP	GO:0022404	molting cycle process	5/111	94/18903	0.000231036624604767	0.00668797769957046	0.00570946400846862	KRT17/SOSTDC1/TGM3/TP63/SPINK5	5
BP	GO:0022405	hair cycle process	5/111	94/18903	0.000231036624604767	0.00668797769957046	0.00570946400846862	KRT17/SOSTDC1/TGM3/TP63/SPINK5	5
BP	GO:0006979	response to oxidative stress	10/111	434/18903	0.000239807164537882	0.00668797769957046	0.00570946400846862	S100A7/GPX2/GSTP1/AKR1C3/NFE2L2/ANXA1/TXN/HSPB1/GJB2/CRYAB	10
BP	GO:0016137	glycoside metabolic process	3/111	21/18903	0.000242642177490875	0.00668797769957046	0.00570946400846862	AKR1C2/AKR1C3/AKR1B10	3
BP	GO:0034109	homotypic cell-cell adhesion	5/111	95/18903	0.000242741804113537	0.00668797769957046	0.00570946400846862	DSP/DSC2/JUP/HSPB1/CD9	5
BP	GO:0098773	skin epidermis development	5/111	95/18903	0.000242741804113537	0.00668797769957046	0.00570946400846862	KRT17/SOSTDC1/TGM3/TP63/SPINK5	5
BP	GO:0006636	unsaturated fatty acid biosynthetic process	4/111	52/18903	0.000245362161296081	0.00668797769957046	0.00570946400846862	AKR1C3/CBR1/ANXA1/FABP5	4
BP	GO:0042060	wound healing	10/111	442/18903	0.000277391651806268	0.00740117529689784	0.00631831412666332	S100A8/KRT6A/SPRR3/DSP/CLDN4/NFE2L2/ANXA1/ITGA2/HSPB1/CD9	10

BP	GO:1902644	tertiary alcohol metabolic process	3/111	22/18903	0.000279755375313537	0.00740117529689784	0.00631831412666332	AKR1C2/AKR1C3/AKR1B10	3
BP	GO:0006749	glutathione metabolic process	4/111	57/18903	0.000350029201213266	0.00912612366641543	0.00779088641602066	GSTA1/GSTP1/NFE2L2/GSTM3	4
BP	GO:0097193	intrinsic apoptotic signaling pathway	8/111	298/18903	0.000376614001123692	0.00967897982887889	0.00826285455096943	S100A8/S100A9/PERP/SFN/CD24/NFE2L2/TP63/HSPB1	8
BP	GO:0019748	secondary metabolic process	4/111	60/18903	0.000426309451466818	0.0108018408899832	0.00922143053654689	AKR1C2/AKR1C3/NFE2L2/AKR1B10	4
BP	GO:0034308	primary alcohol metabolic process	5/111	108/18903	0.000439653654715801	0.0109852350671351	0.00937799243026832	AKR1C2/AKR1C3/AKR1B10/ADH7/ALDH3A2	5
BP	GO:0046456	icosanoid biosynthetic process	4/111	61/18903	0.000454177065392724	0.0111926649402947	0.00955507337286208	AKR1C3/CBR1/ANXA1/FABP5	4
BP	GO:2000116	regulation of cysteine-type endopeptidase activity	7/111	235/18903	0.000476472429865477	0.0115834310990269	0.00988866678767753	S100A8/S100A9/PERP/SFN/SOX2/TP63/CRYAB	7

BP	GO:0007568	aging	6/111	169/18903	0.000487652737928763	0.01169716367378 46	0.009985759222990 39	KRT14/KRT16/NFE2L2/TP63/GJB2/CRYAB	6
BP	GO:0019731	antibacterial humoral response	4/111	63/18903	0.000513770177220494	0.0121614809055219	0.0103821425008961	PI3/SLPI/SPRR2A/SPINK5	4
BP	GO:0006631	fatty acid metabolic process	9/111	400/18903	0.000587420922982725	0.01372428883696	0.0117162970147887	GSTA1/GSTP1/AKR1C2/AKR1C3/CBR1/ANXA1/FABP5/ADH7/ALDH3A2	9
BP	GO:0006805	xenobiotic metabolic process	5/111	118/18903	0.000658823651628361	0.0151951762728131	0.0129719798613465	GSTA1/ALDH3A1/GSTP1/CBR1/GSTM3	5
BP	GO:0097305	response to alcohol	7/111	253/18903	0.000737627732405595	0.0166622969023166	0.0142244470205553	S100A8/AKR1C2/AKR1C3/ADH7/CDH1/JUP/CLDN7	7
BP	GO:0071466	cellular response to xenobiotic stimulus	6/111	183/18903	0.000740958172420973	0.0166622969023166	0.0142244470205553	GSTA1/ALDH3A1/GSTP1/CBR1/NFE2L2/GSTM3	6
BP	GO:0098901	regulation of cardiac muscle cell action potential	3/111	31/18903	0.000785771299169781	0.0174518835457585	0.0148985097529397	DSP/DSC2/JUP	3
BP	GO:2000379	positive regulation of reactive	4/111	71/18903	0.000808441089805083	0.0177364087873091	0.0151414062904444	GSTP1/AKR1C3/CBR1/NFE2L2	4

		oxygen species metabolic process							
BP	GO:0071621	granulocyte chemotaxis	5/111	130/18903	0.00101959092589548	0.022099326213084	0.0188659880898099	S100A8/S100A7/S100A9/S100A14/ANXA1	5
BP	GO:0086004	regulation of cardiac muscle cell contraction	3/111	34/18903	0.00103276809148967	0.0221184499594038	0.0188823138531759	DSP/DSC2/JUP	3
BP	GO:0086005	ventricular cardiac muscle cell action potential	3/111	36/18903	0.00122182307985415	0.0258595261253837	0.0220760355852286	DSP/DSC2/JUP	3
BP	GO:0002237	response to molecule of bacterial origin	8/111	360/18903	0.0012880651715022	0.0269445260875867	0.0230022899047945	S100A8/S100A7/S100A9/SLPI/CD24/S100A14/GSTP1/GJB2	8
BP	GO:0061844	antimicrobial humoral immune response mediated by antimicrobial peptide	4/111	81/18903	0.00132352550916478	0.0272264314538356	0.0232429498792581	S100A7/S100A9/KRT6A/SPINK5	4

BP	GO:0043281	regulation of cysteine-type endopeptidase activity involved in apoptotic process	6/111	205/18903	0.00133180987656339	0.0272264314538356	0.0232429498792581	S100A8/S100A9/SFN/SOX2/TP63/CRYAB	6
BP	GO:0010631	epithelial cell migration	8/111	366/18903	0.0014301577208871	0.0289084689873696	0.0246788895892877	S100A2/TACSTD2/KRT16/NFE2L2/ANXA1/ITGA2/JUP/HSPB1	8
BP	GO:2000659	regulation of interleukin-1-mediated signaling pathway	2/111	10/18903	0.00149117041976868	0.0297672609259097	0.0254120322348101	IL1R2/IL1RN	2
BP	GO:0090132	epithelium migration	8/111	369/18903	0.00150573693399543	0.0297672609259097	0.0254120322348101	S100A2/TACSTD2/KRT16/NFE2L2/ANXA1/ITGA2/JUP/HSPB1	8
BP	GO:0090130	tissue migration	8/111	374/18903	0.00163873107285061	0.0318107472866219	0.0271565374279169	S100A2/TACSTD2/KRT16/NFE2L2/ANXA1/ITGA2/JUP/HSPB1	8
BP	GO:0006882	cellular zinc ion homeostasis	3/111	40/18903	0.00166215133126318	0.0318107472866219	0.0271565374279169	S100A8/S100A9/MT1X	3

BP	GO:1903115	regulation of actin filament-based movement	3/111	40/18903	0.00166215133126318	0.0318107472866219	0.0271565374279169	DSP/DSC2/JUP	3
BP	GO:0008300	isoprenoid catabolic process	2/111	11/18903	0.00181555960457969	0.0341000583792477	0.0291109008954229	AKR1C3/AKR1B10	2
BP	GO:0042180	cellular ketone metabolic process	6/111	218/18903	0.00181968071395652	0.0341000583792477	0.0291109008954229	AKR1C2/AKR1C3/CBR1/ANXA1/FABP5/AKR1B10	6
BP	GO:2000377	regulation of reactive oxygen species metabolic process	5/111	149/18903	0.00186574240942061	0.0346027896345121	0.0295400778659215	GSTP1/AKR1C3/CBR1/NFE2L2/CRYAB	5
BP	GO:0055069	zinc ion homeostasis	3/111	42/18903	0.00191496188139425	0.0347981457033157	0.0297068515146646	S100A8/S100A9/MT1X	3
BP	GO:0086091	regulation of heart rate by cardiac conduction	3/111	42/18903	0.00191496188139425	0.0347981457033157	0.0297068515146646	DSP/DSC2/JUP	3

BP	GO:0016101	diterpenoid metabolic process	4/111	90/18903	0.00195231125491 81	0.03512207947597 66	0.02998339074658 43	AKR1C3/AKR1B10/ADH7/ALDH3A2	4
BP	GO:0043589	skin morphogenesis	2/111	12/18903	0.00217032893890 829	0.03865764119897 04	0.03300166672086 7	TP63/ITGA2	2
BP	GO:0022407	regulation of cell-cell adhesion	9/111	490/18903	0.00239951791819 935	0.04207790794985 62	0.03592151645583 23	CD24/EPCAM/ANXA1/SOX2/SPINT2/LGALS7B/CDH1/CD9/IL1RN	9
BP	GO:0097530	granulocyte migration	5/111	158/18903	0.0024091298048	0.04207790794985 62	0.03592151645583 23	S100A8/S100A7/S100A9/S100A14/ANXA1	5
BP	GO:0031392	regulation of prostaglandin biosynthetic process	2/111	13/18903	0.00255511694112 347	0.04377767025791 54	0.03737258764009 16	ANXA1/FABP5	2
BP	GO:0070486	leukocyte aggregation	2/111	13/18903	0.00255511694112 347	0.04377767025791 54	0.03737258764009 16	S100A8/S100A9	2
BP	GO:0006959	humoral immune response	7/111	320/18903	0.00282417654685 546	0.04758286239790 92	0.04062104457947 37	S100A7/S100A9/KRT6A/PI3/SLPI/SPRR2A/SPINK5	7
BP	GO:0006633	fatty acid biosynthetic process	5/111	164/18903	0.00283010910315 525	0.04758286239790 92	0.04062104457947 37	GSTP1/AKR1C3/CBR1/ANXA1/FABP5	5

BP	GO:0097306	cellular response to alcohol	4/111	100/18903	0.0028649092533514	0.0477219606183257	0.0407397914292368	AKR1C2/AKR1C3/CDH1/JUP	4
BP	GO:0006721	terpenoid metabolic process	4/111	101/18903	0.00296974150057374	0.0485687723593832	0.0414627066922209	AKR1C3/AKR1B10/ADH7/ALDH3A2	4
BP	GO:1902476	chloride transmembrane transport	4/111	101/18903	0.00296974150057374	0.0485687723593832	0.0414627066922209	FXYD3/CLCA2/CLDN4/CLCA4	4
BP	GO:0097529	myeloid leukocyte migration	6/111	242/18903	0.00306296437925437	0.0496420983628704	0.0423789950624194	S100A8/S100A7/S100A9/S100A14/ANXA1/CD9	6
CC	GO:0001533	cornified envelope	19/113	59/19869	5.21742693873829e-29	9.80876264482799e-27	8.07328168415294e-27	SPRR1B/KRT17/SPRR3/PI3/CSTA/DSP/DSG3/SPRR2A/SERPINB5/DSC2/KRT16/DSC3/ANXA1/SPRR1A/PKP1/JUP/HSPB1/CNFN/SPRR2D	19
CC	GO:0030057	desmosome	7/113	25/19869	7.04314393322404e-11	6.6205552972306e-09	5.44916925359965e-09	PERP/DSP/DSG3/DSC2/DSC3/PKP1/JUP	7
CC	GO:0045111	intermediate filament cytoskeleton	13/113	253/19869	2.10977574985952e-09	1.32212613657863e-07	1.08820012361175e-07	S100A8/KRT6A/KRT17/KRT14/KRT5/DSP/KRT19/KRT6B/KRT16/PKP1/KRT8/JUP/KRT18	13
CC	GO:0016328	lateral plasma membrane	8/113	64/19869	2.89248334708472e-09	1.34978117875545e-07	1.1109621124135e-07	TACSTD2/CLDN4/EPCAM/ANXA1/CDH1/JUP/GJB2/CLDN7	8
CC	GO:0005882	intermediate filament	12/113	215/19869	3.58984356051981e-09	1.34978117875545e-07	1.1109621124135e-07	KRT6A/KRT17/KRT14/KRT5/DSP/KRT19/KRT6B/KRT16/PKP1/KRT8/JUP/KRT18	12

CC	GO:0045095	keratin filament	7/113	101/19869	1.76683391060182e-06	5.53405127226238e-05	4.55490222297072e-05	KRT6A/KRT17/KRT14/KRT5/KRT6B/KRT8/KRT18	7
CC	GO:0034774	secretory granule lumen	11/113	322/19869	2.28368070569335e-06	5.53405127226238e-05	4.55490222297072e-05	S100A8/S100A7/SERPINB3/S100A9/SLPI/CSTB/GSTP1/LCN2/PLAC8/FABP5/JUP	11
CC	GO:0060205	cytoplasmic vesicle lumen	11/113	325/19869	2.49730265117146e-06	5.53405127226238e-05	4.55490222297072e-05	S100A8/S100A7/SERPINB3/S100A9/SLPI/CSTB/GSTP1/LCN2/PLAC8/FABP5/JUP	11
CC	GO:0031983	vesicle lumen	11/113	327/19869	2.64927986438093e-06	5.53405127226238e-05	4.55490222297072e-05	S100A8/S100A7/SERPINB3/S100A9/SLPI/CSTB/GSTP1/LCN2/PLAC8/FABP5/JUP	11
CC	GO:0045178	basal part of cell	10/113	272/19869	3.52524620685015e-06	6.62746286887829e-05	5.4548546569155e-05	DSP/TACSTD2/CLCA2/CLDN4/EPCAM/RHCG/ANXA1/ITGA2/ATP1B3/CLDN7	10
CC	GO:0016327	apicolateral plasma membrane	4/113	21/19869	5.50884554248107e-06	9.41511783624037e-05	7.74928511717432e-05	KRT19/CLDN4/JUP/CLDN7	4
CC	GO:0009925	basal plasma membrane	9/113	254/19869	1.48635724960031e-05	0.000232862635770716	0.000191661855869514	DSP/TACSTD2/CLCA2/CLDN4/EPCAM/RHCG/ANXA1/ATP1B3/CLDN7	9
CC	GO:0005912	adherens junction	7/113	179/19869	7.41000946418766e-05	0.00107160136866714	0.000882001126506548	DSC2/ANXA1/PKP1/TRIM29/CDH1/JUP/KRT18	7
CC	GO:0043296	apical junction complex	5/113	151/19869	0.00172369395406088	0.0227137548096977	0.018694971763861	CLDN4/EPCAM/CDH1/JUP/CLDN7	5

CC	GO:0035578	azurophil granule lumen	4/113	91/19869	0.00181226767098652	0.0227137548096977	0.0186949717638611	S100A7/SERPINB3/PLAC8/FABP5	4
CC	GO:0016323	basolateral plasma membrane	6/113	229/19869	0.00199041125932197	0.02338733229703 31	0.0192493720473901	DSP/EPCAM/RHCG/ANXA1/ATP1B3/CLDN7	6
CC	GO:0014704	intercalated disc	3/113	50/19869	0.00289031017387133	0.0319634301581065	0.0263080864123273	DSP/DSC2/JUP	3
CC	GO:0062023	collagen-containing extracellular matrix	8/113	433/19869	0.00333127777814791	0.0331262836586633	0.0272651942767274	S100A8/S100A7/S100A9/SLPI/CSTB/ANXA1/COL17A1/LAMB3	8
CC	GO:0005775	vacuolar lumen	5/113	176/19869	0.00334786909316278	0.0331262836586633	0.0272651942767274	S100A7/SERPINB3/PLAC8/FABP5/SERPINB13	5
CC	GO:0042599	lamellar body	2/113	17/19869	0.00412352634390574	0.0370497593611734	0.0304944827888717	KRTDAP/SPINK5	2
CC	GO:0101002	ficolin-1-rich granule	5/113	185/19869	0.0041385369499183	0.0370497593611734	0.0304944827888717	DSP/CSTB/GSTP1/PKP1/JUP	5
CC	GO:0016324	apical plasma membrane	7/113	368/19869	0.00509849053371023	0.043394308698952	0.0357164802841318	CLDN4/EPCAM/RHCG/ANXA1/ATP1B3/MAL2/CLCA4	7

CC	GO:0035580	specific granule lumen	3/113	62/19869	0.00530887819189306	0.043394308698952	0.03571648028413 18	SLPI/LCN2/JUP	3
MF	GO:0030280	structural constituent of skin epidermis	7/112	37/18432	2.24134123584938e-09	5.24473849188755e-07	3.79848356812368e-07	KRT6A/PI3/KRT5/KRT6B/SPRR1A/PKP1/KRT8	7
MF	GO:0098631	cell adhesion mediator activity	8/112	64/18432	4.81376114366123e-09	5.63210053808365e-07	4.07902917962873e-07	DSP/DSC2/EPCAM/ANXA1/TRIM29/ITGA2/JUP/KRT18	8
MF	GO:0098632	cell-cell adhesion mediator activity	7/112	54/18432	3.54177547121265e-08	2.76258486754587e-06	2.00079245917627e-06	DSP/DSC2/EPCAM/ANXA1/TRIM29/JUP/KRT18	7
MF	GO:0004867	serine-type endopeptidase inhibitor activity	8/112	98/18432	1.44271936001994e-07	6.88346271913236e-06	4.98532387665457e-06	SERPINB3/PI3/SERPINB4/SLPI/SERPINB5/SPINT2/SPINK5/SERPINB13	8
MF	GO:0004866	endopeptidase inhibitor activity	10/112	180/18432	1.47082536733597e-07	6.88346271913236e-06	4.98532387665457e-06	SERPINB3/PI3/CSTA/SERPINB4/SLPI/SERPINB5/CSTB/SPINT2/SPINK5/SERPINB13	10
MF	GO:0030414	peptidase inhibitor activity	10/112	187/18432	2.09947283095172e-07	8.18794404071171e-06	5.93008992602152e-06	SERPINB3/PI3/CSTA/SERPINB4/SLPI/SERPINB5/CSTB/SPINT2/SPINK5/SERPINB13	10
MF	GO:0008106	alcohol dehydrogena	5/112	24/18432	2.93491224163308e-07	8.63746764254534e-06	6.25565582748448e-06	ALDH3A1/AKR1C2/AKR1C3/CBR1/AKR1B10	5

		se (NADP+) activity							
MF	GO:0061135	endopeptidase regulator activity	10/112	194/18432	2.95298039061379e-07	8.63746764254534e-06	6.25565582748448e-06	SERPINB3/PI3/CSTA/SERPINB4/SLPI/SERPINB5/CSTB/SPINT2/SPINK5/SERPINB13	10
MF	GO:0033293	monocarboxylic acid binding	7/112	81/18432	6.07764788305499e-07	1.42216960463487e-05	1.03000137807563e-05	S100A8/S100A9/GSTA1/GSTP1/AKR1C2/AKR1C3/FABP5	7
MF	GO:0048306	calcium-dependent protein binding	7/112	81/18432	6.07764788305499e-07	1.42216960463487e-05	1.03000137807563e-05	S100A8/S100A7/S100A9/S100A2/S100A14/ANXA1/S100A16	7
MF	GO:0004857	enzyme inhibitor activity	13/112	395/18432	8.24157899897056e-07	1.75320862341737e-05	1.26975523333422e-05	SERPINB3/PI3/CSTA/SERPINB4/SFN/SLPI/SERPINB5/CSTB/ANXA1/SPINT2/SPINK5/SERPINB13/HSPB1	13
MF	GO:0004033	aldo-keto reductase (NADP) activity	5/112	31/18432	1.13415169949741e-06	2.21159581401994e-05	1.60174055806213e-05	ALDH3A1/AKR1C2/AKR1C3/CBR1/AKR1B10	5
MF	GO:0061134	peptidase regulator activity	10/112	232/18432	1.51633570985567e-06	2.7294042777402e-05	1.97676153268634e-05	SERPINB3/PI3/CSTA/SERPINB4/SLPI/SERPINB5/CSTB/SPINT2/SPINK5/SERPINB13	10
MF	GO:0098641	cadherin binding involved in cell-cell adhesion	4/112	18/18432	3.7015843949811e-06	6.18693391732556e-05	4.48086532024029e-05	EPCAM/ANXA1/TRIM29/KRT18	4

MF	GO:0005200	structural constituent of cytoskeleton	7/112	107/18432	4.00377954444257e-06	6.24589608933041e-05	4.52356846775617e-05	KRT6A/KRT14/KRT5/DSP/KRT19/KRT6B/KRT16	7
MF	GO:0016903	oxidoreductase activity, acting on the aldehyde or oxo group of donors	5/112	47/18432	9.47675550531222e-06	0.000138597549265191	0.000100378791865478	ALDH3A1/AKR1C3/AKR1B10/ADH7/ALDH3A2	5
MF	GO:0005504	fatty acid binding	5/112	49/18432	1.16675547970284e-05	0.000160600460147333	0.000116314323363565	S100A8/S100A9/GSTA1/GSTP1/FABP5	5
MF	GO:0016614	oxidoreductase activity, acting on CH-OH group of donors	7/112	138/18432	2.13198077228252e-05	0.000277157500396728	0.000200730353413735	ALDH3A1/AKR1C2/AKR1C3/CBR1/AKR1B10/ADH7/ALDH3A2	7
MF	GO:0050786	RAGE receptor binding	3/112	10/18432	2.5407564149806e-05	0.000312914211108137	0.000226627026488574	S100A8/S100A7/S100A9	3
MF	GO:0004032	alditol:NADP+ 1-oxidoreductase activity	3/112	12/18432	4.6168980055267e-05	0.000514454349187261	0.000372591768867067	AKR1C2/AKR1C3/AKR1B10	3

MF	GO:0086080	protein binding involved in heterotypic cell-cell adhesion	3/112	12/18432	4.6168980055267e-05	0.000514454349187261	0.000372591768867067	DSP/DSC2/JUP	3
MF	GO:0031406	carboxylic acid binding	7/112	171/18432	8.36540901936933e-05	0.000889775322969283	0.000644416675654766	S100A8/S100A9/GSTA1/GSTP1/AKR1C2/AKR1C3/FABP5	7
MF	GO:0016620	oxidoreductase activity, acting on the aldehyde or oxo group of donors, NAD or NADP as acceptor	4/112	39/18432	9.0179031298519e-05	0.000917473622776237	0.000664477072725 93	ALDH3A1/AKR1C3/AKR1B10/ALDH3A2	4
MF	GO:0016616	oxidoreductase activity, acting on the CH-OH group of donors, NAD or NADP as acceptor	6/112	126/18432	0.000119901354015878	0.00116903820165481	0.000846671841954223	ALDH3A1/AKR1C2/AKR1C3/CBR1/AKR1B10/ADH7	6

MF	GO:0016209	antioxidant activity	5/112	85/18432	0.000168720600802 15	0.001579224823508 13	0.001143748072806 16	S100A9/GSTA1/GPX2/GSTP1/TXN	5
MF	GO:0004602	glutathione peroxidase activity	3/112	22/18432	0.000309177078888	0.002782593709992	0.002015283793561 46	GSTA1/GPX2/GSTP1	3
MF	GO:0004869	cysteine-type endopeptidase inhibitor activity	4/112	56/18432	0.000371972134166 053	0.003223758496105 8	0.002334795851880 49	SERPINB3/CSTA/CSTB/SERPINB13	4
MF	GO:0004364	glutathione transferase activity	3/112	26/18432	0.000512834882926 663	0.004285834378744 26	0.003104000607187 7	GSTA1/GSTP1/GSTM3	3
MF	GO:0097110	scaffold protein binding	4/112	66/18432	0.000696691469362 511	0.005621579442442 33	0.004071409312789 99	KRT5/DSP/KRT8/KRT18	4
MF	GO:0045296	cadherin binding	8/112	333/18432	0.000973822056525 46	0.007595812040898 58	0.005501240389494 7	SFN/EPCAM/ANXA1/PKP1/TRIM29/CDH1/JUP/KRT18	8
MF	GO:0005254	chloride channel activity	4/112	78/18432	0.001304051952785 96	0.009843488933932 77	0.007129112543244 15	FXYD3/CLCA2/CLDN4/CLCA4	4
MF	GO:0002020	protease binding	5/112	139/18432	0.001596090623903 03	0.011671412687290 9	0.008452979948960 12	SERPINB3/CSTA/SERPINB4/CSTB/SERPINB13	5

MF	GO:0032052	bile acid binding	2/112	11/18432	0.00194190255548841	0.0137698544843723	0.0099727691047411	AKR1C2/AKR1C3	2
MF	GO:0005253	anion channel activity	4/112	91/18432	0.00230038524365935	0.0155178722661302	0.0112387648891002	FXYD3/CLCA2/CLDN4/CLCA4	4
MF	GO:0035325	Toll-like receptor binding	2/112	12/18432	0.00232104927057504	0.0155178722661302	0.0112387648891002	S100A8/S100A9	2
MF	GO:0019215	intermediate filament binding	2/112	13/18432	0.00273219385767815	0.0172792800728835	0.0125144583523807	KRT14/PKP1	2
MF	GO:0045295	gamma-catenin binding	2/112	13/18432	0.00273219385767815	0.0172792800728835	0.0125144583523807	DSC3/CDH1	2
MF	GO:0044594	17-beta-hydroxysteroid dehydrogenase (NAD+) activity	2/112	14/18432	0.0031749404833931	0.0190496429003586	0.0137966374581994	AKR1C2/AKR1C3	2
MF	GO:0052650	NADP-retinol dehydrogenase activity	2/112	14/18432	0.0031749404833931	0.0190496429003586	0.0137966374581994	AKR1C3/AKR1B10	2

MF	GO:0004029	aldehyde dehydrogenase (NAD+) activity	2/112	15/18432	0.00364889686857753	0.0208254113962718	0.0150827316005387	ALDH3A1/ALDH3A2	2
MF	GO:0036041	long-chain fatty acid binding	2/112	15/18432	0.00364889686857753	0.0208254113962718	0.0150827316005387	S100A8/S100A9	2
MF	GO:0015108	chloride transmembrane transporter activity	4/112	107/18432	0.00412480575805797	0.0229262332583649	0.0166042445101068	FXYD3/CLCA2/CLDN4/CLCA4	4
MF	GO:0004252	serine-type endopeptidase activity	5/112	174/18432	0.00421294029961406	0.0229262332583649	0.0166042445101068	TMPRSS11D/TMPRSS11A/KLK11/TMPRSS11E/KLK10	5
MF	GO:0004175	endopeptidase activity	8/112	426/18432	0.00447929141919751	0.0236829842573 7	0.0171523187828956	CLCA2/TMPRSS11D/CAPNS2/TMPRSS11A/KLK11/TMPRSS11E/KLK10/CLCA4	8
MF	GO:0004601	peroxidase activity	3/112	55/18432	0.00455442004949558	0.0236829842573 7	0.0171523187828956	GSTA1/GPX2/GSTP1	3
MF	GO:0004030	aldehyde dehydrogenase [NAD(P)+] activity	2/112	17/18432	0.00468888740461195	0.0238521663625912	0.0172748483327809	ALDH3A1/ALDH3A2	2

MF	GO:0016655	oxidoreductase activity, acting on NAD(P)H, quinone or similar compound as acceptor	3/112	57/18432	0.00503521667101491	0.02410729720010 94	0.01745962595239 59	AKR1C2/AKR1C3/CBR1	3
MF	GO:0016684	oxidoreductase activity, acting on peroxide as acceptor	3/112	57/18432	0.00503521667101491	0.0241072972001094	0.0174596259523959	GSTA1/GPX2/GSTP1	3
MF	GO:0004303	estradiol 17-beta-dehydrogenase activity	2/112	18/18432	0.00525415451797256	0.0241072972001094	0.0174596259523959	AKR1C2/AKR1C3	2
MF	GO:0005229	intracellular calcium activated chloride channel activity	2/112	18/18432	0.00525415451797256	0.0241072972001094	0.0174596259523959	CLCA2/CLCA4	2
MF	GO:0061778	intracellular chloride channel activity	2/112	18/18432	0.00525415451797256	0.0241072972001094	0.0174596259523959	CLCA2/CLCA4	2

MF	GO:0016765	transferase activity, transferring alkyl or aryl (other than methyl) groups	3/112	59/18432	0.00554550868637997	0.02495478908870899	0.01807341899812 09	GSTA1/GSTP1/GSTM3	3
MF	GO:0004745	NAD-retinol dehydrogenase activity	2/112	19/18432	0.00584909726094033	0.0253460881307414	0.0183568159651344	AKR1C3/ADH7	2
MF	GO:0030506	ankyrin binding	2/112	19/18432	0.00584909726094033	0.0253460881307414	0.0183568159651344	RHCG/CDH1	2
MF	GO:0008236	serine-type peptidase activity	5/112	192/18432	0.00636111470914265	0.0270636516716251	0.0196007553717123	TMPRSS11D/TMPRSS11A/KLK11/TMPRSS11E/KLK10	5
MF	GO:0017171	serine hydrolase activity	5/112	196/18432	0.00692665038836867	0.0289435034085405	0.0209622314384841	TMPRSS11D/TMPRSS11A/KLK11/TMPRSS11E/KLK10	5
MF	GO:0019955	cytokine binding	4/112	144/18432	0.0115798175063748	0.0475381981840651	0.034429374303349	SOSTDC1/IL1R2/PLP2/IL1RN	4
MF	GO:0016628	oxidoreductase activity, acting on the CH-CH group of	2/112	28/18432	0.0124782292856781	0.0494899263194692	0.0358429066011451	AKR1C2/AKR1C3	2

		donors, NAD or NADP as acceptor							
MF	GO:0043236	laminin binding	2/112	28/18432	0.01247822928567 81	0.04948992631946 92	0.03584290660114 51	LYPD3/ITGA2	2
