# Supplementary material for: Multiomics Reveals IL-17 Drives Epithelial Keratinization and Proliferation via EHF in Odontogenic Keratocysts
Source: Int J Mol Sci. 2026 May 4;27(9):4115. doi: 10.3390/ijms27094115 (PMC13163638; doi:10.3390/ijms27094115)
Supplement: Supplementary file 1 [file ijms-27-04115-s001.zip › ijms-4235677-supplementary/Supplementary Table S3.pdf]

Supplementary Table S3. EpC1 GO enrichment.

ONTOLOGY	ID	Description	GeneRatio	BgRatio	pvalue	p.adjust	qvalue	geneID	Count
BP	GO:0002181	cytoplasmic translation	62/284	161/18903	6.13070105301882e-72	2.22299220182462e-68	1.82372222903486e-68	RPS27/RPL34/RPL3/RPL31/RPL13A/RPL13/RPS3A/RPL5/RPL11/RPL15/RPS3/RPLP1/RPS6/RPL7/RPL21/RPS19/RPL23/RPL23A/RPS15/RPS18/RPL9/RPL12/RPL10/RACK1/RPL18/RPL6/RPL18A/RPL14/RPL37/PABPC1/RPS14/RPS11/RPL30/RPL35A/RPS27A/RPS4X/RPS5/RPL24/RPL4/RPL19/RPS23/RPS25/RPS13/RPS20/RPS2/RPL32/RPL10A/RPS16/RPS7/EIF4A2/RPS24/RPS9/RPL26/RPS15A/RPS28/RPL39/EIF3E/RPS21/RPL22/EIF3H/EIF4B/NCK1	62
BP	GO:0042254	ribosome biogenesis	28/284	310/18903	2.93064701090618e-14	5.31326303077291e-11	4.35895181727415e-11	RPS27/RPL5/RPL11/RPS6/RPL7/RPS19/RPL23A/RPS15/RPL10/RPL6/RPL14/RPS14/RPL35A/RPS5/RPL24/NPM1/RPS25/RPL10A/RPS16/RPS7/RPS24/RPL26/RPS28/RPS21/RSL1D1/RSL24D1/NOP53/DDX17	28
BP	GO:0042255	ribosome assembly	14/284	61/18903	2.59453179692873e-13	3.13592409855453e-10	2.57268310811249e-10	RPS27/RPL5/RPL11/RPS19/RPL23A/RPS15/RPL10/RPL6/RPS14/RPS5/RPL24/NPM1/RPS28/NOP53	14
BP	GO:0042273	ribosomal large subunit biogenesis	15/284	76/18903	3.87466822514526e-13	3.51238674609418e-10	2.88152958006855e-10	RPL5/RPL11/RPL7/RPL23A/RPL10/RPL6/RPL14/RPL35A/RPL24/NPM1/RPL10A/RPL26/RSL1D1/RSL24D1/NOP53	15
BP	GO:0022613	ribonucleoprotein complex biogenesis	33/284	489/18903	5.47645378936282e-13	3.97152428804591e-10	3.25820177026091e-10	RPS27/RPL13A/RPL5/RPL11/RPS6/RPL7/RPS19/RPL23A/RPS15/RPL10/RPL6/RPL14/RPS14/RPL35A/RPS5/RPL24/NPM1/RPS25/RPL10A/RPS16/RPS7/RPS24/RPL26/RPS28/EIF3E/RPS21/EIF3H/RSL1D1/RSL24D1/EIF4B/NOP53/SF3B1/DDX17	33
BP	GO:0006364	rRNA processing	21/284	229/18903	4.11224235123582e-11	2.48516512759685e-08	2.03880647098113e-08	RPS27/RPL5/RPL11/RPS6/RPL7/RPS19/RPS15/RPL14/RPS14/RPL35A/RPS25/RPL10A/RPS16/RPS7/RPS24/RPL26/RPS28/RPS21/RSL1D1/NOP53/DDX17	21

BP	GO:1904667	negative regulation of ubiquitin protein ligase activity	7/284	12/18903	1.19216297103645e-10	5.50000374430394e-08	4.51215217040756e-08	RPL5/RPL11/RPL23/RPS15/RPL37/RPS20/RPS7	7
BP	GO:0016072	rRNA metabolic process	22/284	268/18903	1.21345918241675e-10	5.50000374430394e-08	4.51215217040756e-08	RPS27/RPL5/RPL11/RPS6/RPL7/RPS19/RPS15/RPL14/RPS14/RPL35A/RPS25/RPL10A/RPS16/RPS7/RPS24/RPL26/RPS28/RPS21/RSL1D1/CAVIN1/NOP53/DDX17	22
BP	GO:0042274	ribosomal small subunit biogenesis	13/284	78/18903	1.39334081780175e-10	5.61361533927683e-08	4.60535807147105e-08	RPS27/RPS6/RPS19/RPS15/RPS14/RPS5/NPM1/RPS25/RPS16/RPS7/RPS24/RPS28/RPS21	13
BP	GO:0006417	regulation of translation	27/284	486/18903	5.70425095749704e-09	2.02620322664485e-06	1.66227837503943e-06	RPL13A/RPL5/RPS3/RPL10/SOX4/RACK1/PABPC1/RPS14/RPS4X/IGFBP5/NPM1/CIRBP/ZFP36L2/EIF4A2/RPS9/RPL26/EEF2/EIF3E/RPL22/DIO2/EIF3H/ITGA2/APP/EIF4B/RPS6KA3/BTG2/NCK1	27
BP	GO:1901796	regulation of signal transduction by p53 class mediator	13/284	105/18903	6.14678309241404e-09	2.02620322664485e-06	1.66227837503943e-06	RPL5/RPL11/RPL23/RPS15/SOX4/RPL37/NPM1/RPS20/RPS7/RPL26/UBB/SNAI2/NOP53	13
BP	GO:0072331	signal transduction by p53 class mediator	16/284	173/18903	8.03921462672801e-09	2.42918268637631e-06	1.99287899430994e-06	RPL5/RPL11/RPL23/RPS15/SOX4/TP63/RPL37/NPM1/RPS20/RPS7/RPL26/UBB/BOK/SNAI2/NOP53/ATRX	16

BP	GO:1901798	positive regulation of signal transduction by p53 class mediator	8/284	30/18903	1.03372977575658e-08	2.88331089761028e-06	2.36544157594178e-06	RPL11/RPL23/RPS15/RPL37/RPS20/RPS7/RPL26/UBB	8
BP	GO:0048732	gland development	25/284	441/18903	1.44555915692735e-08	3.74399821644183e-06	3.07154148682457e-06	SOSTDC1/NFIB/PTCH1/TP63/IGFBP5/DKK3/JUN/TNC/SERPINE2/SEMA3C/ITGA2/FGFR2/EDN1/ARID5B/TGFBR3/NF1/CAV1/SNAI2/PSAP/SIX1/FOXE1/ARHGAP5/TBX3/WLS/LBH	25
BP	GO:0022618	ribonucleoprotein complex assembly	18/284	234/18903	1.71249936385966e-08	4.13968179557008e-06	3.3961566331701e-06	RPS27/RPL13A/RPL5/RPL11/RPS19/RPL23A/RPS15/RPL10/RPL6/RPS14/RPS5/RPL24/RPS28/EIF3E/EIF3H/EIF4B/NOP53/SF3B1	18
BP	GO:0051444	negative regulation of ubiquitin-protein transferase activity	7/284	22/18903	2.25666545097759e-08	5.11416807827797e-06	4.19561616083071e-06	RPL5/RPL11/RPL23/RPS15/RPL37/RPS20/RPS7	7
BP	GO:0071826	ribonucleoprotein complex subunit organization	18/284	242/18903	2.88110277349883e-08	6.14522273923927e-06	5.04148386248155e-06	RPS27/RPL13A/RPL5/RPL11/RPS19/RPL23A/RPS15/RPL10/RPL6/RPS14/RPS5/RPL24/RPS28/EIF3E/EIF3H/EIF4B/NOP53/SF3B1	18
BP	GO:1904666	regulation of ubiquitin protein	7/284	24/18903	4.46326416323031e-08	8.99099769770729e-06	7.37613130133852e-06	RPL5/RPL11/RPL23/RPS15/RPL37/RPS20/RPS7	7

		ligase activity							
BP	GO:0031397	negative regulation of protein ubiquitination	11/284	84/18903	5.10120923359002e-08	9.73525509526179e-06	7.98671318234094e-06	RPL5/RPL11/RPS3/RPL23/RPS15/SOX4/RPL37/RPS20/RPS7/CAV1/HSPA1A	11
BP	GO:0000027	ribosomal large subunit assembly	7/284	26/18903	8.26720796004616e-08	1.49884480315637e-05	1.22963840500476e-05	RPL5/RPL11/RPL23A/RPL10/RPL6/RPL24/NOP53	7
BP	GO:0140694	non-membrane-bounded organelle assembly	22/284	386/18903	1.00219554520541e-07	1.73045764138801e-05	1.41965143396015e-05	RPS27/RPL5/RPL11/RPS3/RPS19/RPL23A/RPS15/RPL10/RPL6/RPS14/RPS5/RPL24/RPS23/GSN/NPM1/CIRBP/ITGB1/RPS28/EDN1/NOP53/HSPA1A/FLNA	22
BP	GO:1903321	negative regulation of protein modification by small protein conjugation or removal	11/284	96/18903	2.06523713234295e-07	3.40388629176161e-05	2.79251681148382e-05	RPL5/RPL11/RPS3/RPL23/RPS15/SOX4/RPL37/RPS20/RPS7/CAV1/HSPA1A	11
BP	GO:0000028	ribosomal small subunit assembly	6/284	19/18903	2.51139933509296e-07	3.95927564741177e-05	3.24815309884334e-05	RPS27/RPS19/RPS15/RPS14/RPS5/RPS28	6

BP	GO:2001244	positive regulation of intrinsic apoptotic signaling pathway	9/284	63/18903	3.9992635482117e-07	6.04222067742318e-05	4.95698192423082e-05	RPL11/RPS3/RACK1/RPS7/RPL26/UBB/BOK/CAV1/NCK1	9
BP	GO:2001235	positive regulation of apoptotic signaling pathway	12/284	135/18903	9.38885255309063e-07	0.000136175917430027	0.000111717462379091	RPL11/RPS3/RACK1/TP63/RPS7/RPL26/UBB/BOK/NF1/CAV1/ATF3/NCK1	12
BP	GO:0001649	osteoblast differentiation	16/284	246/18903	1.04669620815064e-06	0.000145973863490546	0.000119755606649138	PTCH1/RPS15/TP63/IGFBP5/TNC/JUNB/FHL2/VCAN/RSL1D1/FGFR2/DLX5/NF1/SNAI2/RUNX2/ID3/CAT	16
BP	GO:1903320	regulation of protein modification by small protein conjugation or removal	16/284	252/18903	1.43731656632412e-06	0.00019302629146264	0.000158356983096763	RPL5/RPL11/RPS3/RPL23/RPS15/SOX4/RPL37/NPM1/RPS20/RPS2/RPS7/UBB/CAV1/EGR1/NOP53/HSPA1A	16
BP	GO:0045727	positive regulation of translation	12/284	143/18903	1.73370672077118e-06	0.000223648662763526	0.000183479293108173	RPL5/SOX4/PABPC1/RPS4X/NPM1/CIRBP/RPS9/RPL26/EEF2/EIF3E/ITGA2/NCK1	12
BP	GO:0051438	regulation of ubiquitin-protein	8/284	56/18903	1.7886958687651e-06	0.000223648662763526	0.000183479293108173	RPL5/RPL11/RPL23/RPS15/RPL37/RPS20/RPS2/RPS7	8

		transferase activity							
BP	GO:0022612	gland morphogenesis	11/284	123/18903	2.5227210196835e-06	0.000304912880579079	0.000250147705320195	SOSTDC1/NFIB/PTCH1/TP63/IGFBP5/TNC/SEMA3C/FGFR2/CAV1/SNAI2/TBX3	11
BP	GO:0034470	ncRNA processing	21/284	439/18903	3.32698492276038e-06	0.000389149913868682	0.000319254987834324	RPS27/RPL5/RPL11/RPS6/RPL7/RPS19/RPS15/RPL14/RPS14/RPL35A/RPS25/RPL10A/RPS16/RPS7/RPS24/RPL26/RPS28/RPS21/RSL1D1/NOP53/DDX17	21
BP	GO:0031396	regulation of protein ubiquitination	14/284	210/18903	3.73911981462993e-06	0.000423689013995254	0.000347590545925795	RPL5/RPL11/RPS3/RPL23/RPS15/SOX4/RPL37/NPM1/RPS20/RPS2/RPS7/UBB/CAV1/HSPA1A	14
BP	GO:0031069	hair follicle morphogenesis	6/284	31/18903	5.85628601877982e-06	0.000643481609215019	0.000527906356908191	SOSTDC1/TP63/IGFBP5/FGFR2/FOXE1/FST	6
BP	GO:0060537	muscle tissue development	20/284	422/18903	6.56903278225939e-06	0.000700568025543331	0.000574739524540713	PTCH1/FOS/IGFBP5/ITGB1/BMP5/FHL2/SEMA3C/NR4A1/FGFR2/EDN1/TGFBR3/NF1/CAV1/EGR1/ATF3/DSG2/BTG2/SIX1/TBX3/DDX17	20
BP	GO:0045861	negative regulation of proteolysis	18/284	352/18903	6.84944054754966e-06	0.000709602040726145	0.000582150946988732	IL1R2/RPL5/RPL11/RPL23/RPS7/MAP1A/SERPINE2/NR4A1/SNCA/CAST/EIF3H/COL7A1/GPC3/APP/SERPINH1/RPS6KA3/NOP53/TIMP1	18
BP	GO:0001503	ossification	20/284	429/18903	8.37100829951981e-06	0.000839301036137111	0.000688554802485986	PTCH1/RPS15/TP63/IGFBP5/CDH11/TNC/JUNB/BMP5/FHL2/VCAN/RSL1D1/GPC3/FGFR2/DLX5/NF1/SNAI2/RUNX2/ID3/KLF10/CAT	20
BP	GO:0045879	negative regulation of smoothened	6/284	33/18903	8.58996529695365e-06	0.000839301036137111	0.000688554802485986	PTCH1/HHIP/RACK1/SERPINE2/GPC3/RUNX2	6

		signaling pathway							
BP	GO:0007369	gastrulation	13/284	196/18903	8.79576375433266e-06	0.000839301036137111	0.000688554802485986	RPS6/RACK1/ITGB1/TGIF2/LAMB3/ITGB2/ITGA2/COL7A1/GPC3/FGFR2/LAMA3/WLS/ITGB4	13
BP	GO:2000059	negative regulation of ubiquitin-dependent protein catabolic process	7/284	50/18903	9.22347730489234e-06	0.000857546889936913	0.000703523532081667	RPL5/RPL11/RPL23/RPS7/MAP1A/EIF3H/NOP53	7
BP	GO:0008544	epidermis development	18/284	362/18903	1.00382399315901e-05	0.000909966449798642	0.000746528053859832	COL17A1/SOSTDC1/SLITRK6/PTCH1/TP63/IGFBP5/KRT15/KRT14/LAMB3/TXNIP/ALDH3A2/COL7A1/FGFR2/NF1/LAMA3/FOXE1/FZD3/FST	18
BP	GO:0034250	positive regulation of cellular amide metabolic process	12/284	170/18903	1.04211687836408e-05	0.000921638000231255	0.000756103285816915	RPL5/SOX4/PABPC1/RPS4X/NPM1/CIRBP/RPS9/RPL26/EEF2/EIF3E/ITGA2/NCK1	12
BP	GO:2001242	regulation of intrinsic apoptotic signaling pathway	12/284	172/18903	1.17323369717362e-05	0.00101289175855989	0.000830967024614696	RPL11/RPS3/RACK1/RPS7/RPL26/UBB/ACKR3/BOK/CAV1/SNAI2/HSPA1A/NCK1	12

BP	GO:0048730	epidermis morphogenesis	6/284	35/18903	1.22752388840021e-05	0.00103511665566027	0.000849200124508931	SOSTDC1/TP63/IGFBP5/FGFR2/FOXE1/FST	6
BP	GO:0006413	translational initiation	10/284	123/18903	1.68569245711549e-05	0.00138916382943199	0.00113965714923645	RPL13A/RPS3A/RPS3/RPS5/NPM1/EIF4A2/EIF3E/EIF3H/EIF4B/NCK1	10
BP	GO:0048863	stem cell differentiation	14/284	248/18903	2.47472493222509e-05	0.0019940783564996	0.00163592342888143	EPCAM/TP63/ZFP36L2/RPS7/ITGB1/SEMA3C/FGFR2/EDN1/SNAI2/RUNX2/SIX1/TBX3/CHD3/LBH	14
BP	GO:0002183	cytoplasmic translational initiation	6/284	40/18903	2.72558017729296e-05	0.00210275611124772	0.0017250816530862	RPL13A/EIF4A2/EIF3E/EIF3H/EIF4B/NCK1	6
BP	GO:0140467	integrated stress response signaling	6/284	40/18903	2.72558017729296e-05	0.00210275611124772	0.0017250816530862	FOS/JUN/JUNB/AGR2/BOK/NCK1	6
BP	GO:0008589	regulation of smoothened signaling pathway	8/284	84/18903	3.81842182377454e-05	0.0028283927900 49	0.00232038726875445	PTCH1/HHIP/RACK1/SERPINE2/GPC3/FGFR2/RUNX2/RAB34	8
BP	GO:0003007	heart morphogenesis	14/284	258/18903	3.82215241898513e-05	0.00282839279004 9	0.00232038726875445	PTCH1/SOX4/JUN/BMP5/FHL2/SMARCD3/SEMA3C/FGFR2/EDN1/TGFBR3/SNAI2/SIX1/TBX3/FLNA	14
BP	GO:0007229	integrin-mediated signaling pathway	9/284	113/18903	5.24451666831489e-05	0.00376255871751719	0.00308676835013313	DST/ITGB1/ITGB2/ITGA2/LAMA3/TIMP1/FUT8/ITGB4/FLNA	9

BP	GO:0043620	regulation of DNA-templated transcription in response to stress	6/284	45/18903	5.43102020661693e-05	0.00376255871751719	0.00308676835013313	JUN/EGR1/RPS6KA3/ATF3/NCK1/CHD6	6
BP	GO:2001233	regulation of apoptotic signaling pathway	17/284	374/18903	5.49429394950722e-05	0.00376255871751719	0.00308676835013313	RPL11/RPS3/RACK1/TP63/RPS7/SLC25A6/RPL26/UBB/BMP5/ACKR3/BOK/NF1/CAV1/SNAI2/ATF3/HSPA1A/NCK1	17
BP	GO:0061564	axon development	20/284	490/18903	5.56862023095642e-05	0.00376255871751719	0.00308676835013313	SLITRK6/NFIB/PTCH1/RPL24/CDH11/ITGB1/MAP1A/JUN/TNC/FLRT3/SEMA3C/CHL1/FGFR2/EDN1/DLX5/APP/RGMA/LAMA3/FZD3/FLNA	20
BP	GO:0035107	appendage morphogenesis	10/284	142/18903	5.81419161555488e-05	0.00376255871751719	0.00308676835013313	PTCH1/SOX4/TP63/SEMA3C/GPC3/FGFR2/DLX5/RUNX2/ATRX/TBX3	10
BP	GO:0035108	limb morphogenesis	10/284	142/18903	5.81419161555488e-05	0.00376255871751719	0.00308676835013313	PTCH1/SOX4/TP63/SEMA3C/GPC3/FGFR2/DLX5/RUNX2/ATRX/TBX3	10
BP	GO:1903051	negative regulation of proteolysis involved in protein catabolic process	7/284	66/18903	5.85999501950797e-05	0.00376255871751719	0.00308676835013313	RPL5/RPL11/RPL23/RPS7/MAP1A/EIF3H/NOP53	7

BP	GO:0042303	molting cycle	9/284	115/18903	6.01843369045773e-05	0.00376255871751719	0.00308676835013313	SOSTDC1/TP63/IGFBP5/KRT14/FGFR2/NF1/FOXE1/FZD3/FST	9
BP	GO:0042633	hair cycle	9/284	115/18903	6.01843369045773e-05	0.00376255871751719	0.00308676835013313	SOSTDC1/TP63/IGFBP5/KRT14/FGFR2/NF1/FOXE1/FZD3/FST	9
BP	GO:0060443	mammary gland morphogenesis	6/284	46/18903	6.16763385240462e-05	0.00379048141505409	0.0031096758727735	SOSTDC1/PTCH1/IGFBP5/FGFR2/CAV1/TBX3	6
BP	GO:0001942	hair follicle development	8/284	91/18903	6.79964960967838e-05	0.00410925491411563	0.00337119470121949	SOSTDC1/TP63/IGFBP5/FGFR2/NF1/FOXE1/FZD3/FST	8
BP	GO:0072091	regulation of stem cell proliferation	8/284	92/18903	7.35161290061752e-05	0.00436999153731789	0.00358510061383004	NFIB/EPCAM/PTCH1/TP63/NF1/SNAI2/RUNX2/TBX3	8
BP	GO:1900101	regulation of endoplasmic reticulum unfolded protein response	5/284	30/18903	7.7399533634418e-05	0.00451450855325549	0.00370366103622958	RACK1/AGR2/BOK/PIK3R1/NCK1	5
BP	GO:0048483	autonomic nervous system development	6/284	48/18903	7.87965696803417e-05	0.00451450855325549	0.00370366103622958	SOX4/TP63/ACKR3/NF1/SIX1/FZD3	6

BP	GO:0044703	multi-organism reproductive process	12/284	209/18903	7.98623484097378e-05	0.00451450855325549	0.00370366103622958	PNOC/FOS/IGFBP5/IGFBP7/JUNB/ACSL4/SERPINE2/ITGA2/MAGED2/EDN1/TIMP1/ITGB4	12
BP	GO:0048736	appendage development	11/284	178/18903	8.21725219290851e-05	0.00451450855325549	0.00370366103622958	PTCH1/SOX4/TP63/SEMA3C/GPC3/FGFR2/DLX5/RUNX2/ATRX/TBX3/ITGB4	11
BP	GO:0060173	limb development	11/284	178/18903	8.21725219290851e-05	0.00451450855325549	0.00370366103622958	PTCH1/SOX4/TP63/SEMA3C/GPC3/FGFR2/DLX5/RUNX2/ATRX/TBX3/ITGB4	11
BP	GO:0022404	molting cycle process	8/284	94/18903	8.5674933835949e-05	0.00456848985425222	0.0037479467959813	SOSTDC1/TP63/IGFBP5/FGFR2/NF1/FOXE1/FZD3/FST	8
BP	GO:0022405	hair cycle process	8/284	94/18903	8.5674933835949e-05	0.00456848985425222	0.0037479467959813	SOSTDC1/TP63/IGFBP5/FGFR2/NF1/FOXE1/FZD3/FST	8
BP	GO:0098773	skin epidermis development	8/284	95/18903	9.23524244253385e-05	0.00485318682559822	0.00398150955646082	SOSTDC1/TP63/IGFBP5/FGFR2/NF1/FOXE1/FZD3/FST	8
BP	GO:0050821	protein stabilization	12/284	214/18903	0.000100024138425527	0.00518125037044232	0.00425064985248933	RPL5/RPL11/ATP1B3/RPL23/SOX4/NPM1/PFDN5/RPS7/NOP53/PIK3R1/HSPA1A/FLNA	12
BP	GO:0035914	skeletal muscle cell differentiation	7/284	72/18903	0.000102668618804481	0.00524332974345139	0.00430157919557396	FOS/NR4A1/EGR1/ATF3/BTG2/SIX1/DDX17	7

BP	GO:0006979	response to oxidative stress	18/284	434/18903	0.0001074128628803 04	0.005409431122277 51	0.004437847229528 33	GAS5/RPS3/RACK1/FOS/JUN/PDLIM1/TXNIP/SNCA/EDN1/APP/APEX1/SELENOP/HSPA1A/FUT8/PYCR2/CHD6/CAT/SESN3	18
BP	GO:0044706	multi-multicellular organism process	12/284	218/18903	0.0001191667807124 02	0.005919160915933 85	0.004856024834798 11	PNOC/FOS/IGFBP5/IGFBP7/JUNB/ACSL4/SERPINE2/ITGA2/MAGED2/EDN1/TIMP1/ITGB4	12
BP	GO:0031647	regulation of protein stability	15/284	325/18903	0.0001271512121212 91	0.006230409393943 25	0.005111370205615 48	RPL5/RPL11/ATP1B3/RPL23/SOX4/GSN/NPM1/PFDN5/RPS7/SNCA/NOP53/PIK3R1/HSPA1A/TBX3/FLNA	15
BP	GO:0001704	formation of primary germ layer	9/284	127/18903	0.0001297183300772 61	0.006245834049944 12	0.005124024450646 53	ITGB1/LAMB3/ITGB2/ITGA2/COL7A1/FGFR2/LAMA3/WLS/ITGB4	9
BP	GO:0050673	epithelial cell proliferation	19/284	481/18903	0.0001309110280738 43	0.006245834049944 12	0.005124024450646 53	NFIB/PTCH1/TP63/CDH13/IGFBP5/JUN/BMP5/NR4A1/SPARC/GPC3/FGFR2/DLX5/TGFBR3/NF1/CAV1/SNAI2/RUNX2/SIX1/FST	19
BP	GO:0099174	regulation of presynapse organization	5/284	34/18903	0.0001438898581268 07	0.006689033661125 68	0.005487621309937 34	SLITRK6/GPC4/SNCA/IL1RAP/APP	5
BP	GO:1905606	regulation of presynapse assembly	5/284	34/18903	0.0001438898581268 07	0.006689033661125 68	0.005487621309937 34	SLITRK6/GPC4/SNCA/IL1RAP/APP	5
BP	GO:1905898	positive regulation of response to endoplasmic	5/284	35/18903	0.0001658256280006 08	0.007611186419369 69	0.006244146898463 94	AGR2/BOK/CAV1/PIK3R1/NCK1	5

		reticulum stress							
BP	GO:0006414	translational elongation	6/284	55/18903	0.000170484981019283	0.00763183384167803	0.006261085852638	EEF1A1/RPLP1/RACK1/EEF2/DIO2/EEF1D	6
BP	GO:0043616	keratinocyte proliferation	6/284	55/18903	0.000170484981019283	0.00763183384167803	0.006261085852638	PTCH1/TP63/CDH13/FGFR2/SNAI2/FST	6
BP	GO:0051963	regulation of synapse assembly	8/284	104/18903	0.000174239220873102	0.00770477335226667	0.00632092475208454	SLITRK6/GPC4/FLRT3/SNCA/IL1RAP/APP/SIX1/CLSTN1	8
BP	GO:0097193	intrinsic apoptotic signaling pathway	14/284	298/18903	0.000177121919797762	0.00773788049622512	0.00634808554658815	RPL11/RPS3/RACK1/TP63/RPS7/RPL26/UBB/ACKR3/BOK/CAV1/SNAI2/PIK3R1/HSPA1A/NCK1	14
BP	GO:0050678	regulation of epithelial cell proliferation	17/284	414/18903	0.000187447772309108	0.00809149550467651	0.00663818802688647	NFIB/PTCH1/TP63/CDH13/JUN/BMP5/NR4A1/SPARC/GPC3/FGFR2/DLX5/TGFBR3/NF1/CAV1/SNAI2/RUNX2/SIX1	17
BP	GO:0003151	outflow tract morphogenesis	7/284	80/18903	0.000199929347944202	0.0085287507723021	0.00699690820173766	JUN/SEMA3C/FGFR2/EDN1/TGFBR3/SIX1/TBX3	7
BP	GO:0042476	odontogenesis	9/284	135/18903	0.000206147642585371	0.00869175990714599	0.00713063938734711	SOSTDC1/TP63/FGFR2/EDN1/RUNX2/ID3/FST/WLS/ZNF22	9

BP	GO:0052547	regulation of peptidase activity	18/284	459/18903	0.0002146440490608 11	0.008945969217178 16	0.007339190352641 88	RPS3/RACK1/TP63/GSN/SERPINE2/NR4A1/SNCA/CAST/F2R/COL7A1/BOK/GPC3/BEX3/APP/SERPINH1/CAV1/RPS6KA3/TIMP1	18
BP	GO:0006446	regulation of translational initiation	7/284	82/18903	0.0002332477586841 83	0.009502880595380 3	0.007796075293217 04	RPL13A/NPM1/EIF4A2/EIF3E/EIF3H/EIF4B/NCK1	7
BP	GO:1905897	regulation of response to endoplasmic reticulum stress	7/284	82/18903	0.0002332477586841 83	0.009502880595380 3	0.007796075293217 04	RACK1/AGR2/BOK/CAV1/PIK3R1/HSPA1A/NCK1	7
BP	GO:0030879	mammary gland development	9/284	138/18903	0.0002430905032017 84	0.009617838181222 65	0.007890385432732 95	SOSTDC1/PTCH1/IGFBP5/ITGA2/FGFR2/CAV1/ARHGAP5/TBX3/LBH	9
BP	GO:0060973	cell migration involved in heart development	4/284	21/18903	0.0002440267823145 29	0.009617838181222 65	0.007890385432732 95	FLRT3/SEMA3C/EDN1/SNAI2	4
BP	GO:1901522	positive regulation of transcription from RNA polymerase II promoter involved in cellular	4/284	21/18903	0.0002440267823145 29	0.009617838181222 65	0.007890385432732 95	JUN/DLX5/RUNX2/CHD6	4

		response to chemical stimulus							
BP	GO:2000058	regulation of ubiquitin-dependent protein catabolic process	10/284	170/18903	0.000257204487359384	0.0100282093673 67	0.008227050155943 62	RPL5/RPL11/RPL23/RACK1/RPS7/MAP1A/EIF3H/CAV1/NOP53/HSPA1A	10
BP	GO:0060070	canonical Wnt signaling pathway	14/284	310/18903	0.000265580153464442	0.01024461315385 18	0.008404585819602 62	SOSTDC1/SOX4/PFDN5/DKK3/GPC3/FGFR2/EDN1/AMER2/DLX5/CAV1/SNAI2/EGR1/FZD3/WLS	14
BP	GO:0042177	negative regulation of protein catabolic process	8/284	111/18903	0.000272905019079365	0.01041635367559 77	0.008545480154219 23	RPL11/MAP1A/SERPINE2/SNCA/EIF3H/NOP53/TIMP1/FLNA	8
BP	GO:0043618	regulation of transcription from RNA polymerase II promoter in response to stress	5/284	39/18903	0.000280037812887872	0.010577261557619	0.008677487491459 72	JUN/EGR1/ATF3/NCK1/CHD6	5

BP	GO:0008306	associative learning	7/284	85/18903	0.000291478090549 86	0.01058174365428 87	0.00868116456208 661	FOS/ITGB1/MAP1A/APP/NF1/SPECC1/BTG2	7
BP	GO:0007178	transmembrane receptor protein serine/threonine kinase signaling pathway	16/284	390/18903	0.00029162249573 9184	0.01058174365428 87	0.00868116456208 661	SOSTDC1/FOS/DKK3/JUN/TGIF2/BMP5/GPC3/DLX5/TGFBR3/CAV1/RGMA/RUNX2/EGR1/FST/HSPA1A/FUT8	16
BP	GO:0051348	negative regulation of transferase activity	13/284	276/18903	0.000292814038126 505	0.01058174365428 87	0.00868116456208 661	RPL5/RPL11/RPL23/RPS15/RACK1/RPL37/NPM1/RPS20/RPS7/EIF4A2/SNCA/NF1/CAV1	13
BP	GO:0048485	sympathetic nervous system development	4/284	22/18903	0.00029474796168 868	0.01058174365428 87	0.00868116456208 661	SOX4/TP63/NF1/FZD3	4
BP	GO:0071404	cellular response to low-density lipoprotein particle stimulus	4/284	22/18903	0.00029474796168 868	0.01058174365428 87	0.00868116456208 661	GAS5/CDH13/ITGB1/ITGB2	4
BP	GO:1990000	amyloid fibril formation	5/284	40/18903	0.000316149528761434	0.01123880579695 06	0.00922021226294 956	HNRNPA1/GSN/PFDN5/SNCA/APP	5

BP	GO:0002065	columnar/cuboidal epithelial cell differentiation	8/284	116/18903	0.000368187590190573	0.0129616330294273	0.01063360378005068	NFIB/SOX4/TP63/BMP5/AGR2/FGFR2/CAV1/FST	8
BP	GO:0072203	cell proliferation involved in metanephros development	3/284	10/18903	0.000372387988462585	0.0129834504438974	0.0106515025849723	PTCH1/GPC3/EGR1	3
BP	GO:0042176	regulation of protein catabolic process	15/284	361/18903	0.000392985967326687	0.01357111540501049	0.01113361748035031	EEF1A1/RPL11/RACK1/MAP1A/UBB/SERPINE2/SNCA/EIF3H/GPC3/CAV1/RGMA/NOP53/HSPA1A/TIMP1/FLNA	15
BP	GO:0060571	morphogenesis of an epithelial fold	4/284	24/18903	0.000418157680644294	0.0143041485850586	0.01173499111718874	SOSTDC1/TP63/BMP5/FGFR2	4
BP	GO:0034599	cellular response to oxidative stress	13/284	288/18903	0.000439553368263492	0.0148955188161067	0.01222014578172778	GAS5/RPS3/RACK1/FOS/JUN/SNCA/EDN1/APEX1/HSPA1A/FUT8/PYCR2/CHD6/CAT	13
BP	GO:0048762	mesenchymal cell differentiation	12/284	252/18903	0.000451347146048615	0.0151535625145581	0.01243184244379452	PDPN/RPS7/BMP5/SEMA3C/FGFR2/EDN1/TGFBR3/SNAI2/SIX1/TBX3/DDX17/FLNA	12

BP	GO:0070997	neuron death	15/284	368/18903	0.0004801904492788	0.0159655147382566	0.0130979605336642	RACK1/FOS/TP63/JUN/ITGB2/UBB/SNCA/F2R/CHL1/BOK/APP/NF1/EGR1/BTG2/SIX1	15
BP	GO:0007405	neuroblast proliferation	6/284	67/18903	0.000504996489929371	0.0159655147382566	0.0130979605336642	HHIP/ITGB1/SMARCD3/FGFR2/NF1/FZD3	6
BP	GO:0015808	L-alanine transport	3/284	11/18903	0.000506352508245864	0.0159655147382566	0.0130979605336642	SLC7A8/SLC1A4/SLC3A2	3
BP	GO:0060174	limb bud formation	3/284	11/18903	0.000506352508245864	0.0159655147382566	0.0130979605336642	SOX4/SEMA3C/FGFR2	3
BP	GO:0060742	epithelial cell differentiation involved in prostate gland development	3/284	11/18903	0.000506352508245864	0.0159655147382566	0.0130979605336642	TP63/FGFR2/PSAP	3
BP	GO:0071679	commissural neuron axon guidance	3/284	11/18903	0.000506352508245864	0.0159655147382566	0.0130979605336642	NFIB/PTCH1/FZD3	3
BP	GO:1903897	regulation of PERK-mediated unfolded	3/284	11/18903	0.000506352508245864	0.0159655147382566	0.0130979605336642	AGR2/BOK/NCK1	3

		protein response							
BP	GO:0016331	morphogenesis of embryonic epithelium	9/284	153/18903	0.0005200139399458 26	0.0162549185020997	0.0133353847031479	PTCH1/SOX4/TP63/RPS7/BMP5/FGFR2/RGMA/SIX1/FZD3	9
BP	GO:0016055	Wnt signaling pathway	17/284	456/18903	0.000569489592669894	0.0172111469677624	0.0141198656866771	SOSTDC1/SOX4/RACK1/PFDN5/DKK3/GPC4/GPC3/FGFR2/EDN1/AMER2/DLX5/APP/CAV1/SNAI2/EGR1/FZD3/WLS	17
BP	GO:0061138	morphogenesis of a branching epithelium	10/284	188/18903	0.00057085909167582	0.0172111469677624	0.0141198656866771	PTCH1/HHIP/TP63/SEMA3C/GPC3/FGFR2/EDN1/SNAI2/SIX1/TBX3	10
BP	GO:0035148	tube formation	9/284	155/18903	0.000571316015582858	0.0172111469677624	0.0141198656866771	NFIB/PTCH1/SOX4/RPS7/BMP5/FGFR2/RGMA/SIX1/FZD3	9
BP	GO:0035994	response to muscle stretch	4/284	26/18903	0.000574586345411226	0.0172111469677624	0.0141198656866771	FOS/GSN/JUN/EDN1	4
BP	GO:0072089	stem cell proliferation	8/284	124/18903	0.000575632230197245	0.0172111469677624	0.0141198656866771	NFIB/EPCAM/PTCH1/TP63/NF1/SNAI2/RUNX2/TBX3	8
BP	GO:0030111	regulation of Wnt signaling pathway	14/284	336/18903	0.000594035200439346	0.0172111469677624	0.0141198656866771	SOSTDC1/SOX4/RACK1/PFDN5/DKK3/GPC3/FGFR2/AMER2/DLX5/APP/CAV1/SNAI2/EGR1/WLS	14

BP	GO:0007565	female pregnancy	10/284	189/18903	0.000594926307863 78	0.01721114696776 24	0.01411986568667 71	PNOC/FOS/IGFBP5/IGFBP7/JUNB/ACSL4/ITGA2/MAGED2/TIMP1/ITGB4	10
BP	GO:0071478	cellular response to radiation	10/284	189/18903	0.000594926307863 78	0.01721114696776 24	0.01411986568667 71	NPM1/RPL26/TRPM1/CCND2/EEF1D/XPA/SNAI2/EGR1/PIK3R1/TIMP1	10
BP	GO:0060828	regulation of canonical Wnt signaling pathway	12/284	260/18903	0.00059581421534 79	0.01721114696776 24	0.01411986568667 71	SOSTDC1/SOX4/PFDN5/DKK3/GPC3/FGFR2/AMER2/DLX5/CAV1/SNAI2/EGR1/WLS	12
BP	GO:0198738	cell-cell signaling by wnt	17/284	458/18903	0.000598070744053 52	0.01721114696776 24	0.01411986568667 71	SOSTDC1/SOX4/RACK1/PFDN5/DKK3/GPC4/GPC3/FGFR2/EDN1/AMER2/DLX5/APP/CAV1/SNAI2/EGR1/FZD3/WLS	17
BP	GO:0035272	exocrine system development	5/284	46/18903	0.000612017335599 068	0.01747381778647 42	0.01433535839538 31	NFIB/SEMA3C/FGFR2/SNAI2/WLS	5
BP	GO:0003206	cardiac chamber morphogenesis	8/284	126/18903	0.000640020833571 868	0.01813059017602 81	0.01487416838547 78	SOX4/BMP5/FHL2/SMARCD3/SEMA3C/FGFR2/TGFBR3/TBX3	8
BP	GO:0071280	cellular response to copper ion	4/284	27/18903	0.000666602502114 68	0.01841119786057 49	0.01510437633291 27	SNCA/MT1F/APP/MT1E	4
BP	GO:2000647	negative regulation of	4/284	27/18903	0.000666602502114 68	0.01841119786057 49	0.01510437633291 27	NFIB/PTCH1/NF1/SNAI2	4

		stem cell proliferation							
BP	GO:0043589	skin morphogenesis	3/284	12/18903	0.000667651532256925	0.0184111978605749	0.0151043763329127	TP63/ITGA2/ITGB4	3
BP	GO:1903050	regulation of proteolysis involved in protein catabolic process	11/284	227/18903	0.000670236656810778	0.0184111978605749	0.0151043763329127	RPL5/RPL11/RPL23/RACK1/RPS7/MAP1A/UBB/EIF3H/CAV1/NOP53/HSPA1A	11
BP	GO:0048661	positive regulation of smooth muscle cell proliferation	7/284	98/18903	0.000692531278958687	0.0188805896052947	0.0154894609761555	CDH13/IGFBP5/JUN/ITGA2/FGFR2/EDN1/NF1	7
BP	GO:0060411	cardiac septum morphogenesis	6/284	72/18903	0.000742734063232163	0.0200981620394017	0.0164883461327109	SOX4/BMP5/SEMA3C/FGFR2/TGFBR3/TBX3	6
BP	GO:0062197	cellular response to chemical stress	14/284	345/18903	0.000768661825408441	0.0206395103177745	0.0169324632502194	GAS5/RPS3/RACK1/FOS/JUN/SNCA/EDN1/CAV1/APEX1/HSPA1A/FUT8/PYCR2/CHD6/CAT	14
BP	GO:0048608	reproductive structure development	13/284	306/18903	0.000774123939111234	0.0206395103177745	0.0169324632502194	PTCH1/TP63/TNC/UBB/BMP5/SERPINE2/BOK/FGFR2/ARID5B/PSAP/ATRX/FST/TBX3	13

BP	GO:0034620	cellular response to unfolded protein	7/284	100/18903	0.0007812329755332 74	0.02067701291447 92	0.016963230033477	RACK1/AGR2/BOK/ATF3/PIK3R1/HSPA1A/NCK1	7
BP	GO:0052548	regulation of endopeptidase activity	16/284	428/18903	0.0007990162403446 22	0.02084838241675 84	0.01710381998715 68	RPS3/RACK1/TP63/GSN/SERPINE2/NR4A1/SNCA/CAST/F2R/COL7A1/BOK/BEX3/APP/SERPINH1/RPS6KA3/TIMP1	16
BP	GO:0002067	glandular epithelial cell differentiation	6/284	73/18903	0.0007992071582816 91	0.02084838241675 84	0.01710381998715 68	NFIB/TP63/BMP5/AGR2/FGFR2/CAV1	6
BP	GO:0099054	presynapse assembly	5/284	49/18903	0.0008207775046409 97	0.02125813737020 18	0.01743997915876 28	SLITRK6/GPC4/SNCA/IL1RAP/APP	5
BP	GO:0007517	muscle organ development	14/284	348/18903	0.000835761170158 35	0.02143258841430 95	0.01758309718083 97	FOS/ITGB1/NR4A1/FGFR2/ARID5B/TGFBR3/NF1/CAV1/EGR1/ATF3/ID3/BTG2/SIX1/DDX17	14
BP	GO:0034329	cell junction assembly	16/284	430/18903	0.0008393346814208 35	0.02143258841430 95	0.01758309718083 97	COL17A1/DST/SLITRK6/CDH11/GPC4/FLRT3/SNCA/ITGA2/IL1RAP/APP/CAV1/SNAI2/LAMA3/SIX1/CLSTN1/ITGB4	16
BP	GO:0007519	skeletal muscle tissue development	9/284	164/18903	0.0008561502772310 88	0.02146419979143 22	0.01760903086207 43	FOS/NR4A1/NF1/CAV1/EGR1/ATF3/BTG2/SIX1/DDX17	9

BP	GO:1900103	positive regulation of endoplasmic reticulum unfolded protein response	3/284	13/18903	0.000858331210633666	0.02146419979143 22	0.01760903086207 43	AGR2/BOK/PIK3R1	3
BP	GO:1901029	negative regulation of mitochondrial outer membrane permeabilization involved in apoptotic signaling pathway	3/284	13/18903	0.000858331210633666	0.02146419979143 22	0.01760903086207 43	SLC25A6/BOK/HSPA1A	3
BP	GO:0061458	reproductive system development	13/284	310/18903	0.000872164877072464	0.02158722604435 48	0.01770996046138 9	PTCH1/TP63/TNC/UBB/BMP5/SERPINE2/BOK/FGFR2/ARID5B/PSAP/ATRX/FST/TBX3	13
BP	GO:0008630	intrinsic apoptotic signaling pathway in response to	7/284	102/18903	0.000878682916387839	0.02158722604435 48	0.01770996046138 9	RPS3/TP63/RPL26/ACKR3/BOK/SNAI2/PIK3R1	7

		DNA damage							
BP	GO:0060037	pharyngeal system development	4/284	29/18903	0.000881111267116524	0.0215872260443548	0.017709960461389	PTCH1/BMP5/EDN1/SIX1	4
BP	GO:2000116	regulation of cysteine-type endopeptidase activity	11/284	235/18903	0.000890764743022659	0.02167726817584	0.0177838301927378	RPS3/RACK1/TP63/GSN/NR4A1/SNCA/CAST/F2R/BOK/BEX3/RPS6KA3	11
BP	GO:0007411	axon guidance	11/284	236/18903	0.000922130452500366	0.0222909668051088	0.0182873028685336	NFIB/PTCH1/RPL24/FLRT3/SEMA3C/CHL1/EDN1/DLX5/APP/LAMA3/FZD3	11
BP	GO:0061448	connective tissue development	12/284	274/18903	0.000941927783767436	0.0226187426751041	0.0185562071587785	NFIB/TGFBI/BGN/UBB/BMP5/EDN1/ARID5B/SERPINH1/SNAI2/RUNX2/EGR1/TIMP1	12
BP	GO:0034764	positive regulation of transmembrane transport	11/284	237/18903	0.000954405012781934	0.0226187750088058	0.0185562336850481	ATP1B3/ITGB1/SNCA/F2R/GPC3/EDN1/AKAP9/GLRX/CAV1/PIK3R1/FLNA	11
BP	GO:0097485	neuron projection guidance	11/284	237/18903	0.000954405012781934	0.0226187750088058	0.0185562336850481	NFIB/PTCH1/RPL24/FLRT3/SEMA3C/CHL1/EDN1/DLX5/APP/LAMA3/FZD3	11
BP	GO:0030509	BMP signaling pathway	9/284	167/18903	0.000973418602782375	0.0229195834655123	0.0188030141590088	SOSTDC1/BMP5/GPC3/DLX5/TGFBR3/RGMA/RUNX2/EGR1/FST	9

BP	GO:0030850	prostate gland development	5/284	51/18903	0.0009867402096266698	0.02298031447533 19	0.0188528373174117	PTCH1/TP63/TNC/FGFR2/PSAP	5
BP	GO:0048332	mesoderm morphogenesis	6/284	76/18903	0.000988673209639211	0.0229803144753319	0.0188528373174117	ITGB1/ITGA2/FGFR2/TBX3/WLS/ITGB4	6
BP	GO:0035270	endocrine system development	8/284	135/18903	0.00100554913077703	0.0232237015808758	0.0190525098463016	SOX4/DKK3/BMP5/EDN1/ARID5B/NF1/SIX1/FOXE1	8
BP	GO:0007409	axonogenesis	16/284	438/18903	0.00101840540672195	0.0233717595238847	0.0191739752124999	SLITRK6/NFIB/PTCH1/RPL24/CDH11/ITGB1/MAP1A/FLRT3/SEMA3C/CHL1/FGFR2/EDN1/DLX5/APP/LAMA3/FZD3	16
BP	GO:0001763	morphogenesis of a branching structure	10/284	203/18903	0.00102991992802404	0.0234873563460073	0.0192688097755441	PTCH1/HHIP/TP63/SEMA3C/GPC3/FGFR2/EDN1/SNAI2/SIX1/TBX3	10
BP	GO:0001837	epithelial to mesenchymal transition	9/284	169/18903	0.00105859767540222	0.0238490469320135	0.0195655373849305	PDPN/BMP5/FGFR2/EDN1/TGFBR3/SNAI2/TBX3/DDX17/FLNA	9
BP	GO:0030968	endoplasmic reticulum unfolded protein response	6/284	77/18903	0.00105893451628631	0.0238490469320135	0.0195655373849305	RACK1/AGR2/BOK/ATF3/PIK3R1/NCK1	6
BP	GO:1901888	regulation of cell junction assembly	10/284	204/18903	0.00106897497220589	0.0239265632667812	0.019629131068576	SLITRK6/GPC4/FLRT3/SNCA/IL1RAP/APP/CAV1/SNAI2/SIX1/CLSTN1	10

BP	GO:0099172	presynapse organization	5/284	52/18903	0.00107848714286548	0.0239913765646027	0.0196823032982748	SLITRK6/GPC4/SNCA/IL1RAP/APP	5
BP	GO:0030198	extracellular matrix organization	13/284	318/18903	0.0010997303476652	0.0243147697599634	0.01994761208281 03	COL17A1/PDPN/TGFBI/LOXL4/ITGB1/LAMB3/COL18A1/POSTN/APP/SERPINH1/NF1/CAV1/CTSV	13
BP	GO:0043281	regulation of cysteine-type endopeptidase activity involved in apoptotic process	10/284	205/18903	0.00110923386293673	0.02437625446671 87	0.01999805356720 38	RPS3/RACK1/TP63/GSN/NR4A1/SNCA/F2R/BOK/BEX3/RPS6KA3	10
BP	GO:0043062	extracellular structure organization	13/284	319/18903	0.00113138127427826	0.02471318373815 05	0.02027446722327 44	COL17A1/PDPN/TGFBI/LOXL4/ITGB1/LAMB3/COL18A1/POSTN/APP/SERPINH1/NF1/CAV1/CTSV	13
BP	GO:0060603	mammary gland duct morphogenesis	4/284	31/18903	0.00114010757805899	0.02475467112599 93	0.02030850309230 82	SOSTDC1/PTCH1/FGFR2/TBX3	4
BP	GO:0001655	urogenital system development	14/284	360/18903	0.00115581429716796	0.02494632524720 85	0.02046573435962 82	EPCAM/PTCH1/SOX4/TP63/TNC/GPC3/FGFR2/ARID5B/NF1/EGR1/PSAP/ID3/SIX1/CAT	14
BP	GO:0045229	external encapsulatin	13/284	321/18903	0.00119696992480274	0.02554404191811 91	0.02095609558469 66	COL17A1/PDPN/TGFBI/LOXL4/ITGB1/LAMB3/COL18A1/POSTN/APP/SERPINH1/NF1/CAV1/CTSV	13

		g structure organization							
BP	GO:1905475	regulation of protein localization to membrane	9/284	172/18903	0.00119759711143967	0.02554404191811191	0.0209560955846966	RACK1/GSN/ITGB1/ITGB2/GPC4/AGR2/GPC3/ARHGEF16/PIK3R1	9
BP	GO:0072175	epithelial tube formation	8/284	139/18903	0.00121412090439326	0.0257450432709354	0.0211209952343204	PTCH1/SOX4/RPS7/BMP5/FGFR2/RGMA/SIX1/FZD3	8
BP	GO:0007435	salivary gland morphogenesis	4/284	32/18903	0.00128773424595006	0.0270976417015934	0.022230654468808	NFIB/SEMA3C/FGFR2/SNAI2	4
BP	GO:0030178	negative regulation of Wnt signaling pathway	9/284	174/18903	0.00129819223399221	0.0270976417015934	0.022230654468808	SOSTDC1/RACK1/PFDN5/DKK3/GPC3/AMER2/CAV1/SNAI2/EGR1	9
BP	GO:0006986	response to unfolded protein	8/284	141/18903	0.001330605509039	0.0270976417015934	0.022230654468808	RACK1/AGR2/BOK/SERPINH1/ATF3/PIK3R1/HSPA1A/NCK1	8
BP	GO:0090090	negative regulation of canonical Wnt signaling pathway	8/284	141/18903	0.001330605509039	0.0270976417015934	0.022230654468808	SOSTDC1/PFDN5/DKK3/GPC3/AMER2/CAV1/SNAI2/EGR1	8

BP	GO:0030099	myeloid cell differentiation	15/284	407/18903	0.00133100958425685	0.0270976417015934	0.0222306544688 08	RPS6/RPS19/FOS/RPS14/JUN/JUNB/FAM210B/GPC3/APP/TGFBR3/NF1/PIK3R1/HSPA1A/KLF10/FLNA	15
BP	GO:0031330	negative regulation of cellular catabolic process	11/284	247/18903	0.00133170041417601	0.02709764170159 34	0.02223065446880 8	RPL5/RPL11/RPL23/PABPC1/CIRBP/RPS7/MAP1A/SNCA/EIF3H/NOP53/TIMP1	11
BP	GO:0060572	morphogenesis of an epithelial bud	3/284	15/18903	0.00133547073605397	0.02709764170159 34	0.02223065446880 8	SOSTDC1/TP63/FGFR2	3
BP	GO:1901214	regulation of neuron death	13/284	325/18903	0.00133769384020552	0.02709764170159 34	0.02223065446880 8	RACK1/FOS/JUN/ITGB2/UBB/SNCA/F2R/CHL1/BOK/NF1/EGR1/BTG2/SIX1	13
BP	GO:0009612	response to mechanical stimulus	10/284	211/18903	0.00137764756572969	0.02762597949063 51	0.02266409790127 87	SLITRK6/PTCH1/FOS/GSN/JUN/TXNIP/SERPINE2/ITGA2/EDN1/BTG2	10
BP	GO:0072332	intrinsic apoptotic signaling pathway by p53 class mediator	6/284	81/18903	0.00137901331710 01	0.02762597949063 51	0.02266409790127 87	RPL11/TP63/RPS7/RPL26/UBB/BOK	6
BP	GO:0030490	maturation of SSU-rRNA	5/284	55/18903	0.00139178313065799	0.02763554874228 62	0.02267194842677 18	RPS19/RPS14/RPS16/RPS28/RPS21	5

BP	GO:0050680	negative regulation of epithelial cell proliferation	9/284	176/18903	0.00140549473855966	0.0276355487422862	0.0226719484267718	NFIB/PTCH1/SPARC/GPC3/FGFR2/TGFBR3/NF1/CAV1/SNAI2	9
BP	GO:0060538	skeletal muscle organ development	9/284	176/18903	0.00140549473855966	0.0276355487422862	0.0226719484267718	FOS/NR4A1/NF1/CAV1/EGR1/ATF3/BTG2/SIX1/DDX17	9
BP	GO:0009314	response to radiation	16/284	452/18903	0.00140997697664726	0.0276355487422862	0.0226719484267718	FOS/NPM1/CIRBP/ITGB1/RPL26/TRPM1/CCND2/EEF1D/XPA/APP/NF1/SNAI2/EGR1/PIK3R1/TIMP1/CAT	16
BP	GO:1902253	regulation of intrinsic apoptotic signaling pathway by p53 class mediator	4/284	33/18903	0.00144821561994009	0.02823241848334482	0.0231616148384306	RPL11/RPS7/RPL26/UBB	4
BP	GO:0045445	myoblast differentiation	7/284	112/18903	0.00151865901812313	0.02904284715939995	0.02382648302391	SOSTDC1/SOX4/ITGB1/SMARCD3/ID3/TBX3/DDX17	7
BP	GO:0071772	response to BMP	9/284	178/18903	0.00151982624245767	0.02904284715939995	0.02382648302391	SOSTDC1/BMP5/GPC3/DLX5/TGFBR3/RGMA/RUNX2/EGR1/FST	9

BP	GO:0071773	cellular response to BMP stimulus	9/284	178/18903	0.00151982624245767	0.02904284715939 95	0.02382648302391	SOSTDC1/BMP5/GPC3/DLX5/TGFBR3/RGMA/RUNX2/EGR1/FST	9
BP	GO:0007224	smoothened signaling pathway	8/284	144/18903	0.00152182596808767	0.0290428471593995	0.02382648302391	PTCH1/HHIP/RACK1/SERPINE2/GPC3/FGFR2/RUNX2/RAB34	8
BP	GO:0009895	negative regulation of catabolic process	13/284	332/18903	0.00161706846350662	0.0303187003413098	0.0248731811665867	RPL5/RPL11/RPL23/PABPC1/CIRBP/RPS7/MAP1A/SERPINE2/SNCA/EIF3H/NOP53/TIMP1/FLNA	13
BP	GO:0007431	salivary gland development	4/284	34/18903	0.00162212572151519	0.0303187003413098	0.0248731811665867	NFIB/SEMA3C/FGFR2/SNAI2	4
BP	GO:0048333	mesodermal cell differentiation	4/284	34/18903	0.00162212572151519	0.0303187003413098	0.0248731811665867	ITGB1/ITGA2/FGFR2/ITGB4	4
BP	GO:0055094	response to lipoprotein particle	4/284	34/18903	0.00162212572151519	0.0303187003413098	0.0248731811665867	GAS5/CDH13/ITGB1/ITGB2	4
BP	GO:0043484	regulation of RNA splicing	9/284	180/18903	0.00164151656172935	0.0305237900145161	0.0250414348364219	HNRNPA1/NPM1/RPS13/CIRBP/SON/RBM39/PIK3R1/HSPA1A/DDX17	9
BP	GO:0021675	nerve development	6/284	84/18903	0.00166387252084888	0.0307816416357043	0.0252529739200802	SLITRK6/RPL24/SERPINE2/ACKR3/EDN1/SIX1	6

BP	GO:0060562	epithelial tube morphogenesis	13/284	335/18903	0.00175078745088196	0.0322251537913603	0.02643721793316 81	SOSTDC1/PTCH1/HHIP/SOX4/RPS7/BMP5/GPC3/FGFR2/EDN1/RGMA/SIX1/FZD3/TBX3	13
BP	GO:0035137	hindlimb morphogenesis	4/284	35/18903	0.00181003541100095	0.0331474161630 78	0.0271938334475 74	PTCH1/TP63/GPC3/TBX3	4
BP	GO:1990778	protein localization to cell periphery	13/284	337/18903	0.00184492025541205	0.03361648666394 02	0.0275786545453 29	ATP1B3/PTCH1/RACK1/ITGB1/RAMP1/GPC4/AGR2/ARHGEF16/CAV1/RAB34/PIK3R1/CLSTN1/FLNA	13
BP	GO:0046916	cellular transition metal ion homeostasis	7/284	116/18903	0.0018581726363512	0.0336886698970473	0.0276378730017289	FTH1/FTL/CYBRD1/MT1F/APP/SLC39A10/MT1E	7
BP	GO:0061351	neural precursor cell proliferation	8/284	149/18903	0.00188836991451212	0.0340658174627907	0.0279472813742406	HHIP/ITGB1/SMARCD3/FGFR2/NF1/BTG2/FZD3/NAP1L1	8
BP	GO:1903078	positive regulation of protein localization to plasma membrane	5/284	59/18903	0.00190783478759314	0.0342465789099639	0.0280955763925 91	RACK1/ITGB1/AGR2/ARHGEF16/PIK3R1	5

BP	GO:0010566	regulation of ketone biosynthetic process	3/284	17/18903	0.00195200235234607	0.03469588494905 31	0.02846418290882 35	DKK3/BMP5/EGR1	3
BP	GO:0032328	alanine transport	3/284	17/18903	0.00195200235234607	0.03469588494905 31	0.02846418290882 35	SLC7A8/SLC1A4/SLC3A2	3
BP	GO:0034767	positive regulation of ion transmembrane transport	9/284	186/18903	0.00205415907939287	0.036017126468084	0.029548117223214	ATP1B3/ITGB1/SNCA/F2R/EDN1/AKAP9/GLRX/CAV1/FLNA	9
BP	GO:0002011	morphogenesis of an epithelial sheet	5/284	60/18903	0.00205613490868543	0.036017126468084	0.029548117223214	PDPN/ITGB1/BMP5/FLRT3/FLNA	5
BP	GO:0046686	response to cadmium ion	5/284	60/18903	0.00205613490868543	0.036017126468084	0.029548117223214	FOS/JUN/MT1F/MT1E/CAT	5
BP	GO:0032435	negative regulation of proteasomal ubiquitin-dependent protein catabolic process	4/284	37/18903	0.00223011672583212	0.0383676143701256	0.0314764357447601	RPL11/MAP1A/EIF3H/NOP53	4

BP	GO:0071276	cellular response to cadmium ion	4/284	37/18903	0.00223011672583212	0.03836761437012 56	0.0314764357447601	FOS/JUN/MT1F/MT1E	4
BP	GO:0071402	cellular response to lipoprotein particle stimulus	4/284	37/18903	0.00223011672583212	0.0383676143701256	0.0314764357447601	GAS5/CDH13/ITGB1/ITGB2	4
BP	GO:0048678	response to axon injury	6/284	89/18903	0.00223527870882482	0.0383676143701256	0.0314764357447601	JUN/TNC/FLRT3/RGMA/DPYSL3/FLNA	6
BP	GO:0035967	cellular response to topologically incorrect protein	7/284	120/18903	0.0022538063598557	0.0383676143701256	0.0314764357447601	RACK1/AGR2/BOK/ATF3/PIK3R1/HSPA1A/NCK1	7
BP	GO:0071774	response to fibroblast growth factor	7/284	120/18903	0.0022538063598557	0.0383676143701256	0.0314764357447601	HHIP/ZFP36L2/FLRT3/NR4A1/FGFR2/RUNX2/IER2	7
BP	GO:0036499	PERK-mediated unfolded protein response	3/284	18/18903	0.00231654748263221	0.0388879683890018	0.0319033293660751	AGR2/BOK/NCK1	3

BP	GO:0061687	detoxification of inorganic compound	3/284	18/18903	0.00231654748263221	0.03888796838900018	0.03190332936607051	MT1F/MT1E/CAT	3
BP	GO:2000811	negative regulation of anoikis	3/284	18/18903	0.00231654748263221	0.03888796838900018	0.03190332936607051	ITGB1/CAV1/SNAI2	3
BP	GO:0010586	miRNA metabolic process	6/284	90/18903	0.00236536874803092	0.0393432434878904	0.0322768328437246	GAS5/NFIB/FOS/JUN/EGR1/DDX17	6
BP	GO:2000177	regulation of neural precursor cell proliferation	6/284	90/18903	0.00236536874803092	0.0393432434878904	0.0322768328437246	ITGB1/SMARCD3/NF1/BTG2/FZD3/NAP1L1	6
BP	GO:0043516	regulation of DNA damage response, signal transduction by p53 class mediator	4/284	38/18903	0.00246340771108946	0.0406014380018654	0.0333090439786546	SOX4/NPM1/RPL26/SNAI2	4
BP	GO:1902229	regulation of intrinsic apoptotic signaling	4/284	38/18903	0.00246340771108946	0.0406014380018654	0.0333090439786546	RPS3/RPL26/ACKR3/SNAI2	4

		pathway in response to DNA damage							
BP	GO:0060021	roof of mouth development	6/284	91/18903	0.00250107572093241	0.04103574915882 76	0.03366534883236 47	ARID5B/DLX5/TGFBR3/SNAI2/FOXE1/TBX3	6
BP	GO:0001701	in utero embryonic development	14/284	393/18903	0.00260353421907927	0.0425243922449614	0.0348866178431 39	RPL13/PTCH1/ITGB1/JUNB/BMP5/HSD17B2/FGFR2/EDN1/RBBP8/CCNB1IP1/SPECC1/TBX3/FUT8/CMTM3	14
BP	GO:0001656	metanephros development	6/284	92/18903	0.00264255058745367	0.0429681095520494	0.0352506394153603	PTCH1/GPC3/NF1/EGR1/ID3/SIX1	6
BP	GO:0042471	ear morphogenesis	7/284	124/18903	0.00271149570622069	0.0435270125939164	0.0357091582983737	SLITRK6/FGFR2/EDN1/DLX5/SIX1/FZD3/TBX3	7
BP	GO:0000462	maturation of SSU-rRNA from tricistronic rRNA transcript (SSU-rRNA, 5.8S rRNA, LSU-rRNA)	4/284	39/18903	0.00271293569945535	0.0435270125939164	0.0357091582983737	RPS19/RPS14/RPS16/RPS21	4

BP	GO:0035136	forelimb morphogenesis	4/284	39/18903	0.00271293569945535	0.0435270125939164	0.0357091582983737	TP63/RUNX2/ATRX/TBX3	4
BP	GO:0010952	positive regulation of peptidase activity	9/284	194/18903	0.00272876346432274	0.0435880895226178	0.0357592652454257	RPS3/RACK1/GSN/SNCA/F2R/BOK/BEX3/APP/CAV1	9
BP	GO:0043280	positive regulation of cysteine-type endopeptidase activity involved in apoptotic process	7/284	125/18903	0.00283633052542178	0.0451076073911376	0.0370058636419296	RPS3/RACK1/GSN/SNCA/F2R/BOK/BEX3	7
BP	GO:0060485	mesenchyme development	12/284	313/18903	0.00287067904755397	0.0454545075389987	0.0372904573127443	PDPN/RPS7/BMP5/SEMA3C/FGFR2/EDN1/TGFBR3/SNAI2/SIX1/TBX3/DDX17/FLNA	12
BP	GO:1902692	regulation of neuroblast proliferation	4/284	40/18903	0.0029792452739769	0.0467651227854556	0.0383656739314592	ITGB1/SMARCD3/NF1/FZD3	4
BP	GO:1903115	regulation of actin filament-based movement	4/284	40/18903	0.0029792452739769	0.0467651227854556	0.0383656739314592	PDPN/AKAP9/CAV1/DSG2	4

BP	GO:0001838	embryonic epithelial tube formation	7/284	127/18903	0.00309917844903311	0.0484380217939399	0.0397381047956786	PTCH1/SOX4/RPS7/BMP5/RGMA/SIX1/FZD3	7
BP	GO:0003215	cardiac right ventricle morphogenesis	3/284	20/18903	0.003165381054636	0.0486342021360599	0.03989904933274046	SOX4/SMARCD3/SEMA3C	3
BP	GO:0006700	C21-steroid hormone biosynthetic process	3/284	20/18903	0.003165381054636	0.04863420213605599	0.03989904933274046	DKK3/BMP5/EGR1	3
BP	GO:0035795	negative regulation of mitochondrial membrane permeability	3/284	20/18903	0.003165381054636	0.04863420213605599	0.03989904933274046	SLC25A6/BOK/HSPA1A	3
BP	GO:0055093	response to hyperoxia	3/284	20/18903	0.003165381054636	0.04863420213605599	0.03989904933274046	PDPN/CAV1/CAT	3
BP	GO:0003002	regionalization	13/284	360/18903	0.0032617062342082	0.0492965869895302	0.0404424637362942	SOSTDC1/PTCH1/HHIP/TP63/SMARCD3/SEMA3C/GPC3/FGFR2/EDN1/BTG2/SIX1/TBX3/WLS	13
BP	GO:0010038	response to metal ion	13/284	360/18903	0.0032617062342082	0.0492965869895302	0.0404424637362942	FOS/JUN/JUNB/TXNIP/CYBRD1/SNCA/MT1F/EDN1/APP/CUTA/CAV1/MT1E/CAT	13

BP	GO:0046688	response to copper ion	4/284	41/18903	0.00326287393201524	0.0492965869895302	0.0404424637362942	SNCA/MT1F/APP/MT1E	4
BP	GO:0120178	steroid hormone biosynthetic process	4/284	41/18903	0.00326287393201524	0.0492965869895302	0.0404424637362942	DKK3/BMP5/HSD17B2/EGR1	4
BP	GO:0001667	ameboidal-type cell migration	16/284	492/18903	0.00328541318494115	0.0493452047930718	0.0404823493323717	CDH13/ITGB1/JUN/PDLIM1/SEMA3C/NR4A1/SPARC/ITGA2/GPC3/EDN1/ARID5B/NF1/SNAI2/TIMP1/FUT8/ITGB4	16
BP	GO:0035966	response to topologically incorrect protein	8/284	163/18903	0.00329330931051389	0.0493452047930718	0.0404823493323717	RACK1/AGR2/BOK/SERPINH1/ATF3/PIK3R1/HSPA1A/NCK1	8
BP	GO:0035019	somatic stem cell population maintenance	5/284	67/18903	0.00333981028307456	0.0496317708460178	0.0407174454700979	SOX4/TP63/ZFP36L2/KLF10/LBH	5
BP	GO:1904377	positive regulation of protein localization to cell periphery	5/284	67/18903	0.00333981028307456	0.0496317708460178	0.0407174454700979	RACK1/ITGB1/AGR2/ARHGEF16/PIK3R1	5
CC	GO:0022626	cytosolic ribosome	56/289	105/19869	6.57766388290196e-76	2.348226006196e-73	2.04253773205903e-73	RPS27/RPL34/RPL3/RPL31/RPL13A/RPL13/RPS3A/RPL5/RPL11/RPL15/RPS3/RPLP1/RPS6/RPL7/RPL21/RPS19/RPL23/RPL23A/RPS15/RPS18/RPL9/RPL12/RPL10/RACK1/RPL18/RPL6/RPL18A/RPL14/RPL37/RPS14/RPS11/RP	56

								L30/RPL35A/RPS27A/RPS4X/RPS5/RPL24/RPL4/RPL19/RPS23/RPS25/RPS13/RPS20/RPS2/RPL32/RPL10A/RPS16/RPS7/RPS24/RPS9/RPL26/RPS15A/RPS28/RPL39/RPS21/RPL22	
CC	GO:0005840	ribosome	62/289	232/19869	3.03135774800793e-61	5.41097358019416e-59	4.70658176664389e-59	RPS27/RPL34/RPL3/RPL31/RPL13A/RPL13/RPS3A/RPL5/RPL11/RPL15/RPS3/RPLP1/RPS6/RPL7/RPL21/RPS19/RPL23/RPL23A/RPS15/RPS18/RPL9/RPL12/RPL10/RACK1/RPL18/RPL6/BTF3/RPL18A/RPL14/RPL37/RPS14/RPS11/RPL30/RPL35A/RPS27A/RPS4X/RPS5/RPL24/RPL4/RPL19/RPS23/NPM1/RPS25/RPS13/RPS20/RPS2/RPL32/RPL10A/RPS16/RPS7/RPS24/RPS9/RPL26/RPS15A/RPS28/RPL39/RPS21/RPL22/EIF3H/RSL24D1/APEX1/NCK1	62
CC	GO:0044391	ribosomal subunit	57/289	188/19869	9.3861370884174e-60	1.11695031352167e-57	9.71547523187064e-58	RPS27/RPL34/RPL3/RPL31/RPL13A/RPL13/RPS3A/RPL5/RPL11/RPL15/RPS3/RPLP1/RPS6/RPL7/RPL21/RPS19/RPL23/RPL23A/RPS15/RPS18/RPL9/RPL12/RPL10/RACK1/RPL18/RPL6/RPL18A/RPL14/RPL37/RPS14/RPS11/RPL30/RPL35A/RPS27A/RPS4X/RPS5/RPL24/RPL4/RPL19/RPS23/NPM1/RPS25/RPS13/RPS20/RPS2/RPL32/RPL10A/RPS16/RPS7/RPS24/RPS9/RPL26/RPS15A/RPS28/RPL39/RPS21/RPL22	57
CC	GO:0022625	cytosolic large ribosomal subunit	31/289	59/19869	8.28716360767469e-42	7.39629351984966e-40	6.43345595858956e-40	RPL34/RPL3/RPL31/RPL13A/RPL13/RPL5/RPL11/RPL15/RPLP1/RPL7/RPL21/RPL23/RPL23A/RPL9/RPL12/RPL10/RPL18/RPL6/RPL18A/RPL14/RPL37/RPL30/RPL35A/RPL24/RPL4/RPL19/RPL32/RPL10A/RPL26/RPL39/RPL22	31
CC	GO:0022627	cytosolic small ribosomal subunit	25/289	45/19869	9.99207406903862e-35	7.13434088529357e-33	6.20560389550819e-33	RPS27/RPS3A/RPS3/RPS6/RPS19/RPS15/RPS18/RACK1/RPS14/RPS11/RPS27A/RPS4X/RPS5/RPS23/RPS25/RPS13/RPS20/RPS2/RPS16/RPS7/RPS24/RPS9/RPS15A/RPS28/RPS21	25
CC	GO:0015934	large ribosomal subunit	32/289	115/19869	2.73759616831371e-32	1.62886972014666e-30	1.41682608710973e-30	RPL34/RPL3/RPL31/RPL13A/RPL13/RPL5/RPL11/RPL15/RPLP1/RPL7/RPL21/RPL23/RPL23A/RPL9/RPL12/RPL10/RPL18/RPL6/RPL18A/RPL14/RPL37/RPL30/RPL35A/RPL24/RPL4/RPL19/NPM1/RPL32/RPL10A/RPL26/RPL39/RPL22	32

CC	GO:0030055	cell-substrate junction	50/289	432/19869	1.68439425432584e-30	8.59041069706179e-29	7.47212488761087e-29	COL17A1/DST/RPL3/RPL31/RPL13A/RPS3A/RPL5/RPS3/RPLP1/RPL7/RPS19/RPL23/RPS15/RPS18/RPL9/RPL12/RPL18/RPL6/PABPC1/RPS14/RPS11/CDH13/RPL30/RPS4X/RPS5/RPL4/RPL19/GSN/NPM1/RPS13/RPS2/RPL10A/RPS16/RPS7/ITGB1/RPS9/TNC/ITGB2/PDLIM1/FHL2/RPL22/FLRT3/ITGA2/PCBP2/CAV1/LAMA3/HSPA1A/ITGB4/FLNA/CAT	50
CC	GO:0005925	focal adhesion	48/289	422/19869	6.17223625986092e-29	2.59295827720243e-27	2.25541115074367e-27	DST/RPL3/RPL31/RPL13A/RPS3A/RPL5/RPS3/RPLP1/RPL7/RPS19/RPL23/RPS15/RPS18/RPL9/RPL12/RPL18/RPL6/PABPC1/RPS14/RPS11/CDH13/RPL30/RPS4X/RPS5/RPL4/RPL19/GSN/NPM1/RPS13/RPS2/RPL10A/RPS16/RPS7/ITGB1/RPS9/TNC/ITGB2/PDLIM1/FHL2/RPL22/FLRT3/ITGA2/PCBP2/CAV1/HSPA1A/ITGB4/FLNA/CAT	48
CC	GO:0015935	small ribosomal subunit	26/289	77/19869	6.53686960639268e-29	2.59295827720243e-27	2.25541115074367e-27	RPS27/RPS3A/RPS3/RPS6/RPS19/RPS15/RPS18/RACK1/RPS14/RPS11/RPS27A/RPS4X/RPS5/RPS23/NPM1/RPS25/RPS13/RPS20/RPS2/RPS16/RPS7/RPS24/RPS9/RPS15A/RPS28/RPS21	26
CC	GO:0042788	polysomal ribosome	16/289	31/19869	6.53988860578388e-22	2.33474023226485e-20	2.03080751442763e-20	RPL31/RPL11/RPL18/RPL6/BTF3/RPL18A/RPL30/RPL24/RPL19/RPS23/RPL32/RPL10A/RPS28/RPL39/RPS21/EIF3H	16
CC	GO:0005844	polysome	20/289	66/19869	2.06999040705862e-21	6.7180597756357e-20	5.84351358930423e-20	RPL31/RPL11/RPS3/RPS6/RPL18/RPL6/BTF3/RPL18A/RPL30/RPS4X/RPL24/RPL19/RPS23/RPL32/RPL10A/EEF2/RPS28/RPL39/RPS21/EIF3H	20
CC	GO:0062023	collagen-containing extracellular matrix	25/289	433/19869	5.39097999158027e-09	1.60381654749513e-07	1.39503429606682e-07	COL17A1/DST/BCAM/TGFBI/BGN/LOXL4/CDH13/IGFBP7/SDC2/LAMB3/TNC/GPC4/COL18A1/SERPINE2/POSTN/SPARC/VCAN/COL7A1/GPC3/FGFR2/SERPINH1/LAMA3/PSAP/TIMP1/ITGB4	25
CC	GO:0005604	basement membrane	11/289	99/19869	2.06763543367921e-07	5.67804499864215e-06	4.93888625858598e-06	COL17A1/DST/TGFBI/LAMB3/TNC/COL18A1/SPARC/COL7A1/LAMA3/TIMP1/ITGB4	11

CC	GO:0014069	postsynaptic density	19/289	321/19869	2.6722288211449e-07	6.8141834939195e-06	5.92712407697554e-06	RPS27/RPS3/RPL7/RPS19/PTCH1/RPS18/RPL12/RPL6/RPL14/RPS14/RPL30/RPS25/RPS13/RPL10A/FLRT3/AKAP9/PCBP2/PSD3/CLSTN1	19
CC	GO:0032279	asymmetric synapse	19/289	327/19869	3.54920258272938e-07	8.44710214689591e-06	7.34747201336959e-06	RPS27/RPS3/RPL7/RPS19/PTCH1/RPS18/RPL12/RPL6/RPL14/RPS14/RPL30/RPS25/RPS13/RPL10A/FLRT3/AKAP9/PCBP2/PSD3/CLSTN1	19
CC	GO:0099572	postsynaptic specialization	19/289	342/19869	7.00881935607859e-07	1.56384281882504e-05	1.36026428292315e-05	RPS27/RPS3/RPL7/RPS19/PTCH1/RPS18/RPL12/RPL6/RPL14/RPS14/RPL30/RPS25/RPS13/RPL10A/FLRT3/AKAP9/PCBP2/PSD3/CLSTN1	19
CC	GO:0098984	neuron to neuron synapse	19/289	351/19869	1.03468699667153e-06	2.17284269301021e-05	1.8899855357158e-05	RPS27/RPS3/RPL7/RPS19/PTCH1/RPS18/RPL12/RPL6/RPL14/RPS14/RPL30/RPS25/RPS13/RPL10A/FLRT3/AKAP9/PCBP2/PSD3/CLSTN1	19
CC	GO:0005791	rough endoplasmic reticulum	9/289	80/19869	2.417928797295e-06	4.79555878130174e-05	4.17128067369605e-05	RPL18/RPL6/TP63/RPL4/RPS23/RPS28/RPS21/EDN1/APP	9
CC	GO:0005788	endoplasmic reticulum lumen	14/289	312/19869	0.000205152201366845	0.00385470188884018	0.00335290301402876	COL17A1/IGFBP5/IGFBP7/SDC2/TNC/COL18A1/VCAN/COL7A1/GPC3/EDN1/APP/SERPINH1/TIMP1/ESD	14
CC	GO:0043202	lysosomal lumen	7/289	98/19869	0.000573356337364108	0.0102344106219493	0.00890211155381116	BGN/SDC2/GPC4/VCAN/GPC3/PSAP/CTSV	7
CC	GO:0098636	protein complex involved in cell adhesion	5/289	52/19869	0.000934656592719337	0.0158891620762287	0.0138207365840704	ITGB1/TNC/ITGB2/ITGA2/ITGB4	5

CC	GO:0008305	integrin complex	4/289	31/19869	0.00101212013177367	0.0164239494110546	0.0142859061661834	ITGB1/ITGB2/ITGA2/ITGB4	4
CC	GO:0031091	platelet alpha granule	6/289	91/19869	0.00213146660716137	0.0330840686415916	0.0287772379456569	SERPINE2/SPARC/SNCA/MAGED2/APP/TIMP1	6
MF	GO:0003735	structural constituent of ribosome	56/286	176/18432	1.5374192054031e-58	7.90233471577195e-56	6.76464450377365e-56	RPS27/RPL34/RPL3/RPL31/RPL13A/RPL13/RPS3A/RPL5/RPL11/RPL15/RPS3/RPLP1/RPS6/RPL7/RPL21/RPS19/RPL23/RPL23A/RPS15/RPS18/RPL9/RPL12/RPL10/RPL18/RPL6/RPL18A/RPL14/RPL37/RPS14/RPS11/RPL30/RPL35A/RPS27A/RPS4X/RPS5/RPL24/RPL4/RPL19/RPS23/RPS25/RPS13/RPS20/RPS2/RPL32/RPL10A/RPS16/RPS7/RPS24/RPS9/RPL26/RPS15A/RPS28/RPL39/RPS21/RPL22/RSL24D1	56
MF	GO:0019843	rRNA binding	20/286	67/18432	1.01221338936685e-20	2.60138841067281e-18	2.22686945660707e-18	RPL3/RPL5/RPL11/RPS3/RPL23/RPL23A/RPS18/RPL9/RPL12/RPL37/RPS14/RPS11/RPS4X/RPS5/NPM1/RPS13/CIRBP/RPS9/CAVIN1/NOP53	20
MF	GO:0045296	cadherin binding	25/286	333/18432	8.55815083529315e-11	1.46629650978023e-08	1.255195455843e-08	RPL34/RPL15/EPCAM/RPL23A/RACK1/RPL6/RPL14/CDH13/RPL24/CDH11/CTNNAL1/RPS2/ITGB1/EEF2/PDLIM1/EIF3E/EEF1D/CAST/RSL1D1/ARHGEF16/SLC3A2/ARHGAP18/HSPA1A/NCK1/FLNA	25
MF	GO:0055106	ubiquitin-protein transferase regulator activity	7/286	22/18432	2.81167378354842e-08	3.61300081185971e-06	3.09284116190326e-06	RPL5/RPL11/RPL23/RPS15/RPL37/RPS20/RPS7	7
MF	GO:0048027	mRNA 5'-UTR binding	7/286	26/18432	1.0282924148806e-07	1.05708460249726e-05	9.04897325094927e-06	RPS3A/RPL5/RPS14/RPS13/RPS7/RPL26/RSL1D1	7

MF	GO:0004857	enzyme inhibitor activity	20/286	395/18432	3.94570147469504e-06	0.0003380150929988875	0.000289351441477636	RPL5/RPL11/RPL23/RPS15/RACK1/RPL37/NPM1/RPS20/RPS7/TXNIP/SERPINE2/SNCA/CAST/COL7A1/GPC3/APP/SERPINH1/RPS6KA3/TIMP1/NCK1	20
MF	GO:0008097	5S rRNA binding	4/286	10/18432	1.10745837843382e-05	0.000813190866449978	0.000696116695015545	RPL3/RPL5/RPL11/NOP53	4
MF	GO:0005201	extracellular matrix structural constituent	12/286	173/18432	1.70899182034146e-05	0.000981767066976978	0.000840423170174845	COL17A1/TGFBI/BGN/IGFBP7/LAMB3/TNC/COL18A1/POSTN/SPARC/VCAN/COL7A1/LAMA3	12
MF	GO:0097371	MDM2/MDM4 family protein binding	4/286	11/18432	1.71904739353946e-05	0.000981767066976978	0.000840423170174845	RPS15/TP63/RPL37/RPS20	4
MF	GO:0015026	coreceptor activity	6/286	48/18432	9.3997734137369e-05	0.004831483534660077	0.004135900302040424	ITGB1/RAMP1/GPC4/ACKR3/TGFBR3/RGMA	6
MF	GO:0140658	ATP-dependent chromatin remodeler activity	5/286	33/18432	0.000144370877538895	0.006746057368635660	0.005774835101555820	SMARCAD1/ATRX/SMARCA1/CHD3/CHD6	5
MF	GO:0050840	extracellular matrix binding	6/286	56/18432	0.000224202196544359	0.009603327418650060	0.008220747206626510	BCAM/TGFBI/BGN/ITGB1/SPARC/ITGA2	6

MF	GO:0090079	translation regulator activity, nucleic acid binding	8/286	108/18432	0.000280676814285367	0.01046783471131166	0.00896079235988188	EEF1A1/PABPC1/EIF4A2/EEF2/EIF3E/EEF1D/EIF3H/EIF4B	8
MF	GO:0061134	peptidase regulator activity	12/286	232/18432	0.000285116120541696	0.01046783471131166	0.00896079235988188	RACK1/SERPINE2/SNCA/CAST/COL7A1/GPC3/BEX3/APP/SERPINH1/CAV1/RPS6KA3/TIMP1	12
MF	GO:0008135	translation factor activity, RNA binding	7/286	84/18432	0.000328830599985547	0.01126792855095047	0.00964569759957605	EEF1A1/EIF4A2/EEF2/EIF3E/EEF1D/EIF3H/EIF4B	7
MF	GO:0017134	fibroblast growth factor binding	4/286	23/18432	0.000398250123687824	0.0123720083734973	0.0105908242885969	RPS19/RPS2/FGFR2/TGFBR3	4
MF	GO:0015180	L-alanine transmembrane transporter activity	3/286	10/18432	0.000409190938423063	0.0123720083734973	0.0105908242885969	SLC7A8/SLC1A4/SLC3A2	3
MF	GO:0098631	cell adhesion mediator activity	6/286	64/18432	0.000466818442502453	0.0133302599692367	0.0114111174833933	BCAM/EPCAM/ITGB1/PDLIM1/ITGA2/DSG2	6

MF	GO:0019838	growth factor binding	8/286	132/18432	0.00106744972876863	0.028877324241425	0.02471988845569945	IL1R2/RPS19/IGFBP5/RPS2/IGFBP7/FGFR2/TGFBR3/ITGB4	8
MF	GO:0022858	alanine transmembrane transporter activity	3/286	15/18432	0.0014647747505184	0.0376447110883229	0.0322250445114049	SLC7A8/SLC1A4/SLC3A2	3
