# Supplementary material for: Multiomics Reveals IL-17 Drives Epithelial Keratinization and Proliferation via EHF in Odontogenic Keratocysts
Source: Int J Mol Sci. 2026 May 4;27(9):4115. doi: 10.3390/ijms27094115 (PMC13163638; doi:10.3390/ijms27094115)
Supplement: Supplementary file 1 [file ijms-27-04115-s001.zip › ijms-4235677-supplementary/Supplementary Table S4.pdf]

Supplementary Table S4. EpC2 GO enrichment.

ONTOLOGY	ID	Description	GeneRatio	BgRatio	pvalue	p.adjust	qvalue	geneID	Count
BP	GO:0030216	keratinocyte differentiation	32/418	170/18903	8.10160535588705e-21	3.54040154052264e-17	2.57631050317208e-17	S100A7/SPRR2F/SPRR2E/SPRR2D/AQP3/IL1A/SPRR3/ANXA1/EPHA2/TGM1/ALOX15B/KRT16/UGCG/SPRR1B/KRT80/WNT5A/IVL/CSTA/CDH3/CNFN/KRT4/CERS3/KRT6C/GRHL1/SCEL/ZFP36/SPRR1A/ERRFI1/PPL/MAFB/SFN/KRT5	32
BP	GO:0008544	epidermis development	44/418	362/18903	2.63252009409715e-20	5.56655137666473e-17	4.05071702009012e-17	S100A7/SPRR2F/SPRR2E/SPRR2D/AQP3/IL1A/CST6/SPRR3/ANXA1/EPHA2/PLS1/TGM1/ALOX15B/KRT16/UGCG/SPRR1B/KRT80/SPINT1/PITX2/WNT5A/FABP5/CRABP2/IVL/CSTA/CDH3/ZNF750/CNFN/SULT2B1/LAMC2/EMP1/KRT4/CERS3/KRT6C/GRHL1/SCEL/ZFP36/SLC9A3R1/SPRR1A/ERRFI1/PPL/MAFB/KLK7/SFN/KRT5	44
BP	GO:0009913	epidermal cell differentiation	36/418	235/18903	3.82143115102842e-20	5.56655137666473e-17	4.05071702009012e-17	S100A7/SPRR2F/SPRR2E/SPRR2D/AQP3/IL1A/SPRR3/ANXA1/EPHA2/PLS1/TGM1/ALOX15B/KRT16/UGCG/SPRR1B/KRT80/PITX2/WNT5A/IVL/CSTA/CDH3/CNFN/SULT2B1/KRT4/CERS3/KRT6C/GRHL1/SCEL/ZFP36/SLC9A3R1/SPRR1A/ERRFI1/PPL/MAFB/SFN/KRT5	36
BP	GO:0002237	response to molecule of bacterial origin	41/418	360/18903	5.98025505135385e-18	6.53342864360408e-15	4.75430276582631e-15	S100A7/S100A8/S100A9/CXCL1/IL36G/IL1A/CLDN1/CXCL6/TNIP3/IL36A/LYN/GJB2/SLPI/CD14/CXCL8/IL1B/SOD2/CSF3/CD24/CXCL3/CD55/CXCL2/WNT5A/ASS1/TNFAIP3/ZC3H12A/CAPN2/LY96/S100A14/NFKBIA/THBD/B2M/CEBPB/AKIRIN2/TRIB1/ADM/DUSP10/XBP1/ZFP36/GJB6/PTGS2	41
BP	GO:0032496	response to lipopolysaccharide	39/418	339/18903	2.83130063941074e-17	2.47455675884499e-14	1.80070720666523e-14	S100A7/S100A8/S100A9/CXCL1/IL36G/IL1A/CLDN1/CXCL6/TNIP3/IL36A/LYN/GJB2/SLPI/CD14/CXCL8/IL1B/SOD2/CSF3/CXCL3/CD55/CXCL2/WNT5A/ASS1/TNFAIP3/ZC3H12A/CAPN2/LY96/S100A14/NFKBIA/THBD/CEBPB/AKIRIN2/TRIB1/ADM/DUSP10/XBP1/ZFP36/GJB6/PTGS2	39

BP	GO:0043588	skin development	34/418	302/18903	6.44195608632748e-15	4.69189134954185e-12	3.41423672575357e-12	S100A7/SPRR2F/SPRR2E/SPRR2D/AQP3/IL1A/CLDN1/SPRR3/ANXA1/CLDN4/EPHA2/TGM1/ALOX15B/KRT16/UGCG/SPRR1B/KRT80/WNT5A/IVL/CSTA/CDH3/CNFN/KRT4/CERS3/KRT6C/GRHL1/SCEL/ZFP36/SPRR1A/ERRFI1/PPL/MAFB/SFN/KRT5	34
BP	GO:0031424	keratinization	19/418	84/18903	2.0819553386694e-14	1.29973497571219e-11	9.45802568138387e-12	SPRR2F/SPRR2E/SPRR2D/IL1A/SPRR3/TGM1/KRT16/SPRR1B/KRT80/IVL/CDH3/CNFN/KRT4/CERS3/KRT6C/SPRR1A/PPL/SFN/KRT5	19
BP	GO:0042060	wound healing	40/418	442/18903	4.05196781286545e-14	2.21338741777775e-11	1.61065720561402e-11	S100A8/PLAUR/SAA1/IL1A/CEACAM1/CLDN1/LRG1/SPRR3/LYN/PLAT/DUOX2/ANXA1/CLEC7A/HMOX1/PLEK/TGFA/CLDN4/VEGFA/HBEGF/SERPINB2/WNT5A/TNFAIP3/CD59/PLSCR1/CDKN1A/PLAU/HSPB1/THBD/CDH3/ANXA2/F3/MMP12/S100A10/SDC4/XBP1/ODAM/RAP2B/HIF1A/PPL/VPS4B	40
BP	GO:0022407	regulation of cell-cell adhesion	42/418	490/18903	5.80788569505618e-14	2.82005116526617e-11	2.05211961225318e-11	PLAUR/IL1A/CEACAM1/VNN1/LYN/FUT3/ANXA1/IL1B/IL1RN/CD24/NFKBIZ/LGALS3/VEGFA/CD47/CD55/MAP3K8/DLG1/WNT5A/IL4R/ASS1/CEACAM6/RUNX1/ZC3H12A/HAS2/TNFRSF21/B2M/CEBPB/FXYD5/CD46/TFRC/CITED2/AFDN/SDC4/DUSP10/HSPH1/BMP2/XBP1/HLA-E/LGALS7B/IRF1/SELENOK/PRNP	42
BP	GO:0052547	regulation of peptidase activity	40/418	459/18903	1.39772633426471e-13	6.10806408073679e-11	4.44476974296178e-11	S100A8/S100A9/SERPINB4/PRSS22/ECM1/PLAUR/CST6/LYN/SLPI/SERPINB1/CLEC7A/CLDN4/SERPINB3/CSTB/TNFSF10/VEGFA/SPINK7/SERPINB2/SPINT1/GRN/CTSD/CSTA/AKIRIN2/ANXA2/F3/CTSB/PI3/PTGS2/LGMN/IFI16/APLP2/TNFAIP8/DNAJB6/SERPINB13/CST3/PMAIP1/SFN/CRYAB/SERPINB11/PRNP	40
BP	GO:0007159	leukocyte cell-cell adhesion	37/418	414/18903	5.63159717541667e-13	2.23727996877917e-10	1.628043547075e-10	S100A8/S100A9/IL1A/CEACAM1/LRG1/VNN1/LYN/ANXA1/IL1B/CD24/CD177/NFKBIZ/LGALS3/CD47/CD55/MAP3K8/DLG1/IL4R/ASS1/RUNX1/ZC3H12A/HAS2/TNFRSF21/B2M/CEBPB/CD46/TFRC/SDC4/DUSP10/HSPH1/XBP1/HLA-E/EZR/LGALS7B/IRF1/SELENOK/PRNP	37

BP	GO:0032103	positive regulation of response to external stimulus	39/418	464/18903	8.9916534359429e-13	3.27446045958921e-10	2.38278816052487e-10	S100A7/S100A8/S100A9/S100A12/LYN/PLAT/CXCL17/CD14/CXCL8/CLEC7A/IL1B/NFKBIZ/VEGFA/OASL/CD47/GRN/WNT5A/CTSC/PLSCR1/PLAU/LY96/HSPB1/S100A14/NFKBIA/THBD/FABP4/CEBPB/AKIRIN2/VAMP8/F3/MMP12/RAC1/HLA-E/TMSB4X/PTGS2/LGMN/IFI16/RIOK3/KLK7	39
BP	GO:0097529	myeloid leukocyte migration	27/418	242/18903	5.29887171236522e-12	1.72214805707014e-09	1.25318783100298e-09	S100A7/S100A8/S100A9/CXCL1/S100A12/SAA1/IL1A/CXCL6/LYN/CXCL17/CXCL8/ANXA1/IL1B/ADGRE2/CD177/LGALS3/CXCL3/VEGFA/CCL20/CD47/CXCL2/S100A14/DUSP1/EMP2/RAC1/LGMN/SELENOK	27
BP	GO:0071216	cellular response to biotic stimulus	28/418	261/18903	5.51717913020181e-12	1.72214805707014e-09	1.25318783100298e-09	CXCL1/IL36G/IL1A/CXCL6/TNIP3/IL36A/LYN/CD14/CXCL8/CLEC7A/IL1B/CSF3/CXCL3/CD55/CDA/CXCL2/WNT5A/ASS1/TNFAIP3/ZC3H12A/CAPN2/LY96/NFKBIA/CEBPB/TRIB1/XBP1/ZFP36/HSPA5	28
BP	GO:0097530	granulocyte migration	22/418	158/18903	6.30106014025727e-12	1.83570885419495e-09	1.33582474973454e-09	S100A7/S100A8/S100A9/CXCL1/S100A12/SAA1/IL1A/CXCL6/CXCL17/CXCL8/ANXA1/IL1B/ADGRE2/CD177/LGALS3/CXCL3/CCL20/CXCL2/S100A14/EMP2/RAC1/SELENOK	22
BP	GO:0010631	epithelial cell migration	33/418	366/18903	8.40375333783809e-12	2.29527513039703e-09	1.67024597589532e-09	CEACAM1/LRG1/PRSS3/TACSTD2/ANXA1/HMOX1/CIB1/EPHA2/KRT16/VEGFA/HBEGF/GRN/WNT5A/GLUL/ZC3H12A/HAS2/HSPB1/ADIPOR1/S100P/EMP2/ANXA3/DCN/DUSP10/RAC1/S100A2/ID1/PLK2/PKN2/TMSB4X/PTGS2/LGMN/HIF1A/GADD45A	33
BP	GO:0090132	epithelium migration	33/418	369/18903	1.04848194686118e-11	2.69521535751962e-09	1.96127799471679e-09	CEACAM1/LRG1/PRSS3/TACSTD2/ANXA1/HMOX1/CIB1/EPHA2/KRT16/VEGFA/HBEGF/GRN/WNT5A/GLUL/ZC3H12A/HAS2/HSPB1/ADIPOR1/S100P/EMP2/ANXA3/DCN/DUSP10/RAC1/S100A2/ID1/PLK2/PKN2/TMSB4X/PTGS2/LGMN/HIF1A/GADD45A	33

BP	GO:0050673	epithelial cell proliferation	38/418	481/18903	1.16408261265176e-11	2.82613389849345e-09	2.05654594901812e-09	CRNN/ECM1/SDR16C5/CEACAM1/CLDN1/LRG1/TACSTD2/SERPINB1/EHF/HMOX1/TGFA/EPHA2/TGM1/VEGFA/DLG1/GRN/WNT5A/TNFAIP3/GLUL/HAS2/AREG/B2M/CEBPB/CDH3/CDKN2B/F3/MMP12/KRT4/DUSP10/BMP2/XBP1/ODAM/ID2/ID1/ZFP36/ERRFI1/HIF1A/SFN	38
BP	GO:0090130	tissue migration	33/418	374/18903	1.50801617056598e-11	3.46843719230175e-09	2.52394285389464e-09	CEACAM1/LRG1/PRSS3/TACSTD2/ANXA1/HMOX1/CIB1/EPHA2/KRT16/VEGFA/HBEGF/GRN/WNT5A/GLUL/ZC3H12A/HAS2/HSPB1/ADIPOR1/S100P/EMP2/ANXA3/DCN/DUSP10/RAC1/S100A2/ID1/PLK2/PKN2/TMSB4X/PTGS2/LGMN/HIF1A/GADD45A	33
BP	GO:0097193	intrinsic apoptotic signaling pathway	29/418	298/18903	2.55465538871929e-11	5.58192202435166e-09	4.06190206806368e-09	S100A8/S100A9/PLAUR/VNN1/SOD2/HMOX1/ERO1A/CD24/EPHA2/BCL2A1/MUC1/BCL3/IL19/CDKN1A/HSPB1/PPP1R15A/CYLD/CEBPB/SKIL/MDM2/XBP1/SHISA5/SLC9A3R1/PTGS2/HIF1A/IFI16/SELENOK/PMAIP1/SFN	29
BP	GO:0071222	cellular response to lipopolysaccharide	25/418	222/18903	2.69501747402145e-11	5.60820302927321e-09	4.08102646066106e-09	CXCL1/IL36G/IL1A/CXCL6/TNIP3/IL36A/LYN/CD14/CXCL8/IL1B/CSF3/CXCL3/CD55/CXCL2/WNT5A/ASS1/TNFAIP3/ZC3H12A/CAPN2/LY96/NFKBIA/CEBPB/TRIB1/XBP1/ZFP36	25
BP	GO:0052548	regulation of endopeptidase activity	35/418	428/18903	3.02974926138829e-11	6.01818376012129e-09	4.37936484146126e-09	S100A8/S100A9/SERPINB4/PLAUR/CST6/LYN/SLPI/SERPINB1/CLEC7A/SERPINB3/CSTB/TNFSF10/VEGFA/SPINK7/SERPINB2/SPINT1/CTSD/CSTA/AKIRIN2/ANXA2/F3/PI3/PTGS2/LGMN/IFI16/APLP2/TNFAIP8/DNAJB6/SERPINB13/CST3/PMAIP1/SFN/CRYAB/SERPINB11/PRNP	35
BP	GO:0010466	negative regulation of peptidase activity	27/418	263/18903	3.70887163467092e-11	7.04685610587474e-09	5.1279181731537e-09	SERPINB4/ECM1/PLAUR/CST6/SLPI/SERPINB1/SERPINB3/CSTB/VEGFA/SPINK7/SERPINB2/SPINT1/CSTA/ANXA2/CTSB/PI3/PTGS2/IFI16/APLP2/TNFAIP8/DNAJB6/SERPINB13/CST3/SFN/CRYAB/SERPINB11/PRNP	27
BP	GO:19	regulation of leukocyte cell-cell adhesion	32/418	377/18903	8.48603152763926e-11	1.48392886646509e-08	1.07983839710732e-08	IL1A/CEACAM1/VNN1/LYN/ANXA1/IL1B/CD24/NFKBIZ/LGALS3/CD47/CD55/MAP3K8/DLG1/IL4R/ASS1/RUNX1/ZC3H12A/HAS2/TNFRSF21/B2M/CEBPB/	32

	03037							CD46/TFRC/SDC4/DUSP10/HSPH1/XBP1/HLA-E/LGALS7B/IRF1/SELENOK/PRNP	
BP	GO:0071219	cellular response to molecule of bacterial origin	25/418	234/18903	8.48929557474305e-11	1.48392886646509e-08	1.07983839710732e-08	CXCL1/IL36G/IL1A/CXCL6/TNIP3/IL36A/LYN/CD14/CXCL8/IL1B/CSF3/CXCL3/CD55/CXCL2/WNT5A/ASS1/TNFAIP3/ZC3H12A/CAPN2/LY96/NFKBIA/CEBPB/TRIB1/XBP1/ZFP36	25
BP	GO:0001667	ameboidal-type cell migration	37/418	492/18903	9.10114540250584e-11	1.52969251572887e-08	1.11314009153725e-08	CEACAM1/LRG1/PRSS3/TACSTD2/ANXA1/HMOX1/CIB1/EPHA2/KRT16/VEGFA/HBEGF/GRN/PITX2/WNT5A/GLUL/ZC3H12A/HAS2/HSPB1/ZFAND5/ADIPOR1/S100P/SDC4/EMP2/ANXA3/DCN/DUSP10/RAC1/S100A2/ID1/PLK2/PKN2/SLC9A3R1/TMSB4X/PTGS2/LGMN/HIF1A/GADD45A	37
BP	GO:0002683	negative regulation of immune system process	35/418	449/18903	1.1354607768805e-10	1.78848769179717e-08	1.30146243933981e-08	SERPINB4/CEACAM1/LYN/ANXA1/HMOX1/LGALS3/CD47/CD55/DLG1/GRN/IL4R/TNFAIP3/RUNX1/ZC3H12A/CD59/TNFRSF21/CEBPB/CD46/TRIB1/DUSP1/MMP12/SDC4/DUSP10/ISG15/HLA-B/OAS1/HLA-E/EZR/ID2/LGALS7B/PLK2/IFI16/IRF1/MAFB/PRNP	35
BP	GO:0019221	cytokine-mediated signaling pathway	37/418	496/18903	1.14594177048789e-10	1.78848769179717e-08	1.30146243933981e-08	CXCL1/IL36G/ECM1/IL1A/CEACAM1/CXCL6/IL36A/LYN/CXCL8/DUOX2/IL1B/IL1RN/CSF3/CD24/CIB1/CXCL3/IL13RA1/OASL/CCL20/UGCG/CXCL2/WNT5A/IL4R/TNFAIP3/NFKBIA/CYLD/TXNDC17/ADIPOR1/F3/MMP12/IFI27/MX1/ISG15/OAS1/TMSB4X/HIF1A/IRF1	37
BP	GO:0001819	positive regulation of cytokine production	36/418	486/18903	2.5142342783924e-10	3.78869096433613e-08	2.75698793285787e-08	SAA1/IL1A/IL36A/CXCL17/CD14/ANXA1/CLEC7A/IL1B/HMOX1/ALOX15B/BCL3/CD55/WNT5A/IL4R/RUNX1/RSAD2/LY96/HSPB1/B2M/S100A13/CEBPB/CD46/AKIRIN2/F3/MMP12/XBP1/ISG15/OAS1/LUM/HLA-E/PTGS2/HIF1A/IFI16/RIOK3/IRF1/SELENOK	36
BP	GO:00	negative regulation of	31/418	373/18903	2.8554155789103e-10	4.15938869327934e-08	3.02674051364492e-08	SERPINB4/ECM1/PLAUR/CST6/SLPI/ANXA1/SERPINB1/SERPINB3/CSTB/LGALS3/VEGFA/SPINK7/SERPINB2/SPINT1/SORT1/PPP1R15A/CSTA/ANXA2/CT	31

	51346	hydrolase activity						SB/PI3/PTGS2/IFI16/APLP2/TNFAIP8/DNAJB6/SERPINB13/CST3/SFN/CRYAB/SERPINB11/PRNP	
BP	GO:0045861	negative regulation of proteolysis	30/418	352/18903	3.04920958046113e-10	4.29840189245649e-08	3.1278988599569e-08	SERPINB4/ECM1/PLAUR/CST6/PLAT/SLPI/SERPINB1/SERPINB3/CSTB/VEGFA/SPINK7/SERPINB2/SPINT1/PLAU/CSTA/N4BP1/ANXA2/CTSB/PI3/PTGS2/IFI16/APLP2/TNFAIP8/DNAJB6/SERPINB13/CST3/SFN/CRYAB/SERPINB11/PRNP	30
BP	GO:0050863	regulation of T cell activation	31/418	376/18903	3.48102450724076e-10	4.75377409270066e-08	3.4592681040705e-08	IL1A/CEACAM1/PRDM1/VNN1/LYN/ANXA1/IL1B/CD24/NFKBIZ/LGALS3/CD47/CD55/MAP3K8/DLG1/IL4R/RUNX1/ZC3H12A/TNFRSF21/B2M/CEBPB/CD46/TFRC/SDC4/DUSP10/HSPH1/XBP1/HLA-E/LGALS7B/IRF1/SELENOK/PRNP	31
BP	GO:0010951	negative regulation of endopeptidase activity	25/418	252/18903	4.14550451270046e-10	5.44269885472286e-08	3.9605909286084e-08	SERPINB4/PLAUR/CST6/SLPI/SERPINB1/SERPINB3/CSTB/VEGFA/SPINK7/SERPINB2/SPINT1/CSTA/ANXA2/PI3/PTGS2/IFI16/APLP2/TNFAIP8/DNAJB6/SERPINB13/CST3/SFN/CRYAB/SERPINB11/PRNP	25
BP	GO:0002697	regulation of immune effector process	31/418	379/18903	4.23459407461275e-10	5.44269885472286e-08	3.9605909286084e-08	SERPINB4/CEACAM1/CXCL6/CFH/LYN/ANXA1/CLEC7A/IL1B/ADGRE2/HMOX1/CD177/NFKBIZ/LGALS3/DNASE1L3/CD47/CD55/GRN/WNT5A/IL4R/ZC3H12A/CD59/RSAD2/B2M/CD46/TFRC/VAMP8/DUSP10/XBP1/HLA-B/HLA-E/KLK7	31
BP	GO:0002831	regulation of response to biotic stimulus	30/418	361/18903	5.59894050667313e-10	6.99067714690331e-08	5.08703737463444e-08	SERPINB4/CEACAM1/CXCL6/CFH/LYN/CD14/CLEC7A/IL1B/OASL/CD55/GRN/WNT5A/TNFAIP3/ZC3H12A/PLSCR1/LY96/AKIRIN2/TRIB1/N4BP1/MMP12/DUSP10/ISG15/HLA-B/OAS1/HLA-E/IFI16/RIOK3/IRF1/SELENOK/KLK7	30
BP	GO:0071621	granulocyte chemotaxis	18/418	130/18903	5.88114896848738e-10	7.13906138674718e-08	5.19501492216385e-08	S100A7/S100A8/S100A9/CXCL1/S100A12/SAA1/CXCL6/CXCL17/CXCL8/ANXA1/IL1B/ADGRE2/LGALS3/CXCL3/CCL20/CXCL2/S100A14/RAC1	18

BP	GO:0019058	viral life cycle	28/418	319/18903	6.21801775240569e-10	7.34398312919267e-08	5.34413417639192e-08	APOBEC3A/CLDN1/TMPRSS2/SLPI/CXCL8/CTSL/SERPINB3/EPHA2/OASL/CD55/ZC3H12A/PLSCR1/RSAD2/USP6NL/CD46/N4BP1/TFRC/VAMP8/CTSB/STOM/IFI27/MX1/ISG15/NECTIN4/OAS1/PKN2/IFI16/VPS4B	28
BP	GO:0050878	regulation of body fluid levels	31/418	390/18903	8.53342482863422e-10	9.81343855292936e-08	7.14112919869917e-08	AQP3/PLAUR/SAA1/CEACAM1/CLDN1/TMPRSS11F/LYN/PLAT/PLEK/CLDN4/KRT16/VEGFA/UGCG/SERPINB2/HAS2/CD59/PLSCR1/PLAU/HSPB1/THBD/VAMP8/ANXA2/ADM/F3/EMP2/XBP1/GRHL1/RAP2B/HIF1A/AKR1B1/SFN	31
BP	GO:0050678	regulation of epithelial cell proliferation	32/418	414/18903	9.08970942774477e-10	1.01851359485243e-07	7.41160922569958e-08	CRNN/ECM1/CEACAM1/CLDN1/LRG1/TACSTD2/HMOX1/TGFA/TGM1/VEGFA/DLG1/GRN/WNT5A/TNFAIP3/GLUL/HAS2/AREG/B2M/CDH3/CDKN2B/F3/MMP12/KRT4/DUSP10/BMP2/XBP1/ODAM/ID1/ZFP36/ERRFI1/HIF1A/SFN	32
BP	GO:0050777	negative regulation of immune response	21/418	190/18903	1.51114837042465e-09	1.65092959468893e-07	1.20136295448759e-07	SERPINB4/CEACAM1/LYN/ANXA1/HMOX1/LGALS3/CD55/GRN/IL4R/TNFAIP3/ZC3H12A/CD59/CD46/MMP12/DUSP10/ISG15/HLA-B/OAS1/HLA-E/PLK2/IFI16	21
BP	GO:0010632	regulation of epithelial cell migration	26/418	292/18903	1.90713106668783e-09	2.032722624738e-07	1.47918946147983e-07	CEACAM1/TACSTD2/ANXA1/HMOX1/CIB1/EPHA2/VEGFA/HBEGF/GRN/WNT5A/GLUL/ZC3H12A/HAS2/HSPB1/ADIPOR1/EMP2/ANXA3/DCN/DUSP10/RAC1/PLK2/TMSB4X/PTGS2/LGMN/HIF1A/GADD45A	26
BP	GO:0060326	cell chemotaxis	27/418	319/18903	2.80566396140298e-09	2.9192265503169e-07	2.1242884279194e-07	S100A7/S100A8/S100A9/CXCL1/S100A12/SAA1/CXCL6/LYN/CXCL17/CXCL8/ANXA1/IL1B/ADGRE2/EPHA2/LGALS3/CXCL3/VEGFA/HBEGF/CCL20/CXCL2/WNT5A/HSPB1/S100A14/DUSP1/RAC1/TMSB4X/LGMN	27
BP	GO:00	positive regulation of cell adhesion	34/418	484/18903	3.16646046385993e-09	3.21800749466695e-07	2.34170797094757e-07	PLAUR/SAA1/IL1A/VNN1/LYN/FUT3/ANXA1/IL1B/CD24/CIB1/NFKBIZ/VEGFA/CD47/CD55/MAP3K8/WNT5A/IL4R/CEACAM6/RUNX1/HAS2/B2M/CD46/TF	34

	45785							RC/CITED2/AFDN/S100A10/SDC4/EMP2/DUSP10/RAC1/HSPH1/XBP1/HLA-E/SELENOK	
BP	GO:0022409	positive regulation of cell-cell adhesion	27/418	322/18903	3.43620811172443e-09	3.41277942005358e-07	2.48344131710993e-07	PLAUR/IL1A/VNN1/LYN/FUT3/ANXA1/IL1B/CD24/NFKBIZ/CD47/CD55/MAP3K8/WNT5A/IL4R/CEACAM6/RUNX1/HAS2/B2M/CD46/TFRC/CITED2/AFDN/DUSP10/HSPH1/XBP1/HLA-E/SELENOK	27
BP	GO:0018149	peptide cross-linking	10/418	36/18903	3.81937935392532e-09	3.70904172814526e-07	2.69902807677389e-07	SPRR2E/SPRR3/ANXA1/TGM1/SPRR1B/IVL/CSTA/PI3/DCN/SPRR1A	10
BP	GO:0043542	endothelial cell migration	25/418	281/18903	3.99554276508469e-09	3.79576562683045e-07	2.76213608542811e-07	CEACAM1/PRSS3/ANXA1/HMOX1/CIB1/EPHA2/VEGFA/GRN/WNT5A/GLUL/ZC3H12A/HSPB1/S100P/EMP2/ANXA3/DCN/RAC1/S100A2/ID1/PLK2/TMSB4X/PTGS2/LGMN/HIF1A/GADD45A	25
BP	GO:0030595	leukocyte chemotaxis	23/418	240/18903	4.10753503059353e-09	3.81913363482845e-07	2.77914072282711e-07	S100A7/S100A8/S100A9/CXCL1/S100A12/SAA1/CXCL6/LYN/CXCL17/CXCL8/ANXA1/IL1B/ADGRE2/LGALS3/CXCL3/VEGFA/CCL20/CXCL2/WNT5A/S100A14/DUSP1/RAC1/LGMN	23
BP	GO:1990266	neutrophil migration	17/418	130/18903	4.33438968214178e-09	3.94610060644991e-07	2.87153316441893e-07	S100A8/S100A9/CXCL1/S100A12/SAA1/IL1A/CXCL6/CXCL8/IL1B/CD177/LGALS3/CXCL3/CCL20/CXCL2/EMP2/RAC1/SELENOK	17
BP	GO:0016032	viral process	31/418	421/18903	5.34884456770488e-09	4.77029607364701e-07	3.47129096434725e-07	APOBEC3A/CLDN1/TMPRSS2/SLPI/CXCL8/CTSL/ST3GAL1/SERPINB3/EPHA2/GALNT1/OASL/CD55/ZC3H12A/PLSCR1/RSAD2/USP6NL/CD46/N4BP1/TFRC/VAMP8/CTSB/STOM/IFI27/MX1/ISG15/NECTIN4/OAS1/ZFP36/PKN2/IFI16/VPS4B	31

BP	GO:0050900	leukocyte migration	30/418	398/18903	5.55970282702801e-09	4.85918027082248e-07	3.53597099798982e-07	S100A7/S100A8/S100A9/CXCL1/ECM1/S100A12/SAA1/IL1A/CXCL6/LYN/CXCL17/CXCL8/ANXA1/IL1B/ADGRE2/HMOX1/CD177/LGALS3/CXCL3/VEGFA/CCL20/CD47/CXCL2/WNT5A/S100A14/DUSP1/EMP2/RAC1/LGMN/SELENOK	30
BP	GO:0050679	positive regulation of epithelial cell proliferation	22/418	224/18903	5.70117201290665e-09	4.8851219012553e-07	3.55484843157709e-07	CRNN/ECM1/CLDN1/LRG1/HMOX1/TGFA/TGM1/VEGFA/GRN/WNT5A/TNFAIP3/GLUL/HAS2/AREG/CDH3/F3/MMP12/BMP2/XBP1/ODAM/ID1/HIF1A	22
BP	GO:0019730	antimicrobial humoral response	16/418	125/18903	1.69419420624909e-08	1.42377474640549e-06	1.03606491843695e-06	S100A7/SPRR2A/S100A9/CXCL1/S100A12/CXCL6/PRSS3/SLPI/CXCL8/PGLYRP4/BCL3/CXCL3/CXCL2/PI3/HLA-E/KLK7	16
BP	GO:0038066	p38MAPK cascade	11/418	57/18903	4.01028652889953e-08	3.24744125299855e-06	2.3631265868502e-06	IL1B/VEGFA/DLG1/ZC3H12A/CYLD/DUSP1/DUSP10/BMP2/EZR/ZFP36/GADD45A	11
BP	GO:1900744	regulation of p38MAPK cascade	10/418	45/18903	4.0128564682362e-08	3.24744125299855e-06	2.3631265868502e-06	IL1B/VEGFA/DLG1/ZC3H12A/CYLD/DUSP1/DUSP10/BMP2/EZR/GADD45A	10
BP	GO:0010594	regulation of endothelial cell migration	21/418	230/18903	4.56034292118502e-08	3.6233997391961e-06	2.63670736170334e-06	CEACAM1/ANXA1/HMOX1/CIB1/EPHA2/VEGFA/GRN/WNT5A/GLUL/ZC3H12A/HSPB1/EMP2/ANXA3/DCN/RAC1/PLK2/TMSB4X/PTGS2/LGMN/HIF1A/GADD45A	21
BP	GO:00	humoral immune response	25/418	320/18903	5.35515195442899e-08	4.17893107872405e-06	3.04096128840789e-06	S100A7/SPRR2A/S100A9/CXCL1/S100A12/CXCL6/CFH/PRSS3/SLPI/CXCL8/IL1B/PGLYRP4/BCL3/CXCL3/CD55/CXCL2/CD59/TNFRSF21/CD46/PI3/IGHG4/IGKC/HLA-E/IGHG3/KLK7	25

	06959								
BP	GO:0050867	positive regulation of cell activation	31/418	467/18903	5.87312211203051e-08	4.50272695255673e-06	3.27658391513281e-06	IL1A/VNN1/LYN/ANXA1/CLEC7A/IL1B/CD24/PLEK/CD177/NFKBIZ/CD47/CD55/MAP3K8/WNT5A/IL4R/CTSC/RUNX1/CDKN1A/B2M/CD46/AKIRIN2/TFRC/VAMP8/DUSP10/HSPH1/IGHG4/XBP1/IGKC/HLA-E/IGHG3/SELENOK	31
BP	GO:0061041	regulation of wound healing	16/418	137/18903	6.31743833446768e-08	4.75986302096962e-06	3.4636989488978e-06	PLAUR/CEACAM1/CLDN1/PLAT/DUOX2/ANXA1/CLEC7A/CLDN4/HBEGF/SERPINB2/TNFAIP3/PLAU/THBD/ANXA2/F3/XBP1	16
BP	GO:0002698	negative regulation of immune effector process	15/418	122/18903	8.20391675562578e-08	6.07646037662452e-06	4.42177208184576e-06	SERPINB4/CEACAM1/ANXA1/HMOX1/LGALS3/CD47/CD55/GRN/IL4R/ZC3H12A/CD59/CD46/DUSP10/HLA-B/HLA-E	15
BP	GO:0002696	positive regulation of leukocyte activation	30/418	450/18903	8.80005317510348e-08	6.4093720625337e-06	4.66402818280484e-06	IL1A/VNN1/LYN/ANXA1/CLEC7A/IL1B/CD24/CD177/NFKBIZ/CD47/CD55/MAP3K8/WNT5A/IL4R/CTSC/RUNX1/CDKN1A/B2M/CD46/AKIRIN2/TFRC/VAMP8/DUSP10/HSPH1/IGHG4/XBP1/IGKC/HLA-E/IGHG3/SELENOK	30
BP	GO:0090303	positive regulation of wound healing	11/418	62/18903	1.00035780897663e-07	7.16649774627521e-06	5.21498005335358e-06	CLDN1/PLAT/DUOX2/ANXA1/CLEC7A/CLDN4/HBEGF/PLAU/THBD/F3/XBP1	11
BP	GO:0051384	response to glucocorticoid	16/418	142/18903	1.04793935095312e-07	7.29351474863971e-06	5.30740890175613e-06	CLDN1/PLAT/GJB2/ANXA1/IL1RN/PAPPA/ASS1/AREG/FBXO32/FOSL1/ADM/ANXA3/ZFP36/FOSB/PTGS2/ERRFI1	16

BP	GO:1901342	regulation of vasculature development	26/418	355/18903	1.05146780129131e-07	7.29351474863971e-06	5.30740890175613e-06	ECM1/IL1A/CEACAM1/LRG1/CXCL8/IL1B/HMOX1/EPHA2/VEGFA/GRN/WNT5A/TNFAIP3/GLUL/RUNX1/ZC3H12A/HSPB1/ADM/F3/EMP2/ANXA3/DCN/XBP1/ID1/PLK2/HIF1A/GADD45A	26
BP	GO:1903036	positive regulation of response to wounding	12/418	77/18903	1.17242587399826e-07	8.00547042089437e-06	5.82549106142886e-06	CLDN1/PLAT/DUOX2/ANXA1/CLEC7A/CLDN4/HBEGF/GRN/PLAU/THBD/F3/XBP1	12
BP	GO:0045766	positive regulation of angiogenesis	18/418	187/18903	1.90442966503307e-07	1.25095823725364e-05	9.10308282486628e-06	ECM1/IL1A/LRG1/CXCL8/IL1B/HMOX1/VEGFA/GRN/WNT5A/RUNX1/ZC3H12A/HSPB1/ADM/F3/EMP2/ANXA3/XBP1/HIF1A	18
BP	GO:1904018	positive regulation of vasculature development	18/418	187/18903	1.90442966503307e-07	1.25095823725364e-05	9.10308282486628e-06	ECM1/IL1A/LRG1/CXCL8/IL1B/HMOX1/VEGFA/GRN/WNT5A/RUNX1/ZC3H12A/HSPB1/ADM/F3/EMP2/ANXA3/XBP1/HIF1A	18
BP	GO:0019079	viral genome replication	15/418	130/18903	1.91794512347812e-07	1.25095823725364e-05	9.10308282486628e-06	APOBEC3A/SLPI/CXCL8/OASL/ZC3H12A/PLSCR1/RSAD2/N4BP1/STOM/IFI27/MX1/ISG15/OAS1/PKN2/IFI16	15
BP	GO:0010595	positive regulation of endothelial cell migration	15/418	131/18903	2.12284076591848e-07	1.3642373745682e-05	9.92740240532466e-06	ANXA1/HMOX1/CIB1/VEGFA/GRN/WNT5A/ZC3H12A/HSPB1/ANXA3/RAC1/PLK2/TMSB4X/PTGS2/LGMN/HIF1A	15
BP	GO:00	acute inflammatory response	14/418	114/18903	2.25431132404496e-07	1.42773050522848e-05	1.03894347977724e-05	S100A8/SAA1/IL1A/VNN1/SAA2/IL1B/DNASE1L3/ASS1/PLSCR1/CEBPB/TFRC/F3/HLA-E/PTGS2	14

	02526								
BP	GO:0031638	zymogen activation	11/418	67/18903	2.29040226764997e-07	1.42986541566148e-05	1.04049703016099e-05	PLAUR/PRSS3/PLAT/CTSL/RUNX1/PLAU/THBD/ANXA2/S100A10/LGMN/IFI16	11
BP	GO:0045765	regulation of angiogenesis	25/418	349/18903	2.82270448167974e-07	1.73240921171698e-05	1.26065475818307e-05	ECM1/IL1A/CEACAM1/LRG1/CXCL8/IL1B/HMOX1/EPHA2/VEGFA/GRN/WNT5A/TNFAIP3/GLUL/RUNX1/ZC3H12A/HSPB1/ADM/F3/EMP2/ANXA3/DCN/XBP1/PLK2/HIF1A/GADD45A	25
BP	GO:1903034	regulation of response to wounding	17/418	172/18903	2.85431266003713e-07	1.73240921171698e-05	1.26065475818307e-05	PLAUR/CEACAM1/CLDN1/PLAT/DUOX2/ANXA1/CLEC7A/CLDN4/HBEGF/SERPINB2/GRN/TNFAIP3/PLAU/THBD/ANXA2/F3/XBP1	17
BP	GO:2001233	regulation of apoptotic signaling pathway	26/418	374/18903	2.91238131975036e-07	1.7434392283985e-05	1.26868117764468e-05	S100A8/S100A9/PLAUR/IL1A/VNN1/IL1B/SOD2/HMOX1/MUC1/TNFSF10/MAL/LGALS3/IL19/TNFAIP3/CTSC/HSPB1/CYLD/SKIL/GCLM/MDM2/XBP1/TPD52L1/SLC9A3R1/PTGS2/HIF1A/PMAIP1	26
BP	GO:0051090	regulation of DNA-binding transcription factor activity	29/418	454/18903	3.55301337059583e-07	2.09819843641943e-05	1.52683547547226e-05	S100A8/S100A9/CRNN/S100A12/ADGRF1/CLEC7A/IL1B/CSF3/HMOX1/CIB1/BHLHE40/BCL3/VEGFA/WNT5A/TNFAIP3/ZC3H12A/NFKBIA/CYLD/FOSL1/TRIB1/TFRC/ANXA3/BMP2/MTPN/ID2/ID1/PIM1/TMSB4X/PRNP	29
BP	GO:0031349	positive regulation of defense response	23/418	307/18903	3.8772734669227e-07	2.25915800672696e-05	1.64396394997522e-05	S100A8/S100A9/S100A12/LYN/CLEC7A/IL1B/NFKBIZ/CD47/GRN/WNT5A/CTSC/PLSCR1/NFKBIA/FABP4/CEBPB/AKIRIN2/VAMP8/MMP12/HLA-E/PTGS2/IFI16/RIOK3/KLK7	23

BP	GO:0010634	positive regulation of epithelial cell migration	17/418	176/18903	3.96753924527908e-07	2.28133506603547e-05	1.66010194736677e-05	ANXA1/HMOX1/CIB1/VEGFA/HBEGF/GRN/WNT5A/ZC3H12A/HAS2/HSPB1/ANXA3/RAC1/PLK2/TMSB4X/PTGS2/LGMN/HIF1A	17
BP	GO:0007162	negative regulation of cell adhesion	23/418	308/18903	4.10605538775394e-07	2.33031974603698e-05	1.69574754974773e-05	CEACAM1/TACSTD2/ANXA1/IL1RN/MUC1/LGALS3/VEGFA/DLG1/IL4R/ASS1/RUNX1/ZC3H12A/TNFRSF21/CEBPB/FXYD5/CLDN7/DUSP1/MMP12/SDC4/BMP2/LGALS7B/IRF1/PRNP	23
BP	GO:0045088	regulation of innate immune response	20/418	240/18903	4.21315536594757e-07	2.36044730117832e-05	1.71767103380939e-05	SERPINB4/CEACAM1/CFH/LYN/CLEC7A/GRN/WNT5A/TNFAIP3/PLSCR1/AKIRIN2/N4BP1/MMP12/DUSP10/ISG15/HLA-B/OAS1/HLA-E/IFI16/RIOK3/IRF1	20
BP	GO:0045071	negative regulation of viral genome replication	10/418	57/18903	4.28710759098324e-07	2.3705235373566e-05	1.72500339789336e-05	APOBEC3A/SLPI/OASL/PLSCR1/RSAD2/N4BP1/MX1/ISG15/OAS1/IFI16	10
BP	GO:0042742	defense response to bacterium	26/418	382/18903	4.37520998802144e-07	2.3705235373566e-05	1.72500339789336e-05	S100A7/S100A8/SPRR2A/S100A9/S100A12/CXCL6/LCN2/SLPI/IL1B/PGLYRP4/EPHA2/BCL3/CCL20/GRN/S100A14/CEBPB/AKIRIN2/PI3/ANXA3/IGHG4/ISG15/OAS1/IGKC/HLA-E/IGHG3/KLK7	26
BP	GO:0002695	negative regulation of leukocyte activation	18/418	198/18903	4.47811556524545e-07	2.3705235373566e-05	1.72500339789336e-05	CEACAM1/LYN/ANXA1/HMOX1/LGALS3/DLG1/GRN/IL4R/TNFAIP3/RUNX1/ZC3H12A/TNFRSF21/CEBPB/SDC4/ID2/LGALS7B/IRF1/PRNP	18
BP	GO:00	regulation of adaptive	18/418	198/18903	4.47811556524545e-07	2.3705235373566e-05	1.72500339789336e-05	CEACAM1/ANXA1/CLEC7A/IL1B/NFKBIZ/CD55/IL4R/TNFAIP3/ZC3H12A/RSAD2/B2M/CD46/AKIRIN2/TFRC/DUSP10/HLA-B/HLA-E/IRF1	18

	02819	immune response							
BP	GO:0042445	hormone metabolic process	20/418	241/18903	4.50236735928141e-07	2.3705235373566e-05	1.72500339789336e-05	SDR16C5/RDH10/DUOX2/DHRS9/CTSL/ERO1A/MME/TIPARP/ALDH1A3/DHRS3/SRD5A3/CRABP2/ADM/CTSB/BMP2/SCPEP1/RBP1/HIF1A/AKR1B1/AKR1B10	20
BP	GO:0045069	regulation of viral genome replication	12/418	87/18903	4.63654882704535e-07	2.4121093302605e-05	1.75526491309574e-05	APOBEC3A/SLPI/CXCL8/OASL/PLSCR1/RSAD2/N4BP1/MX1/ISG15/OAS1/PKN2/IFI16	12
BP	GO:0010838	positive regulation of keratinocyte proliferation	6/418	15/18903	4.76802801498013e-07	2.45132734417214e-05	1.78380342207492e-05	CRNN/LRG1/TGM1/HAS2/AREG/CDH3	6
BP	GO:0010038	response to metal ion	25/418	360/18903	5.0421224448728e-07	2.55904873829266e-05	1.8621910727164e-05	S100A8/AQP3/IL1A/CLDN1/CD14/SOD2/HMOX1/CPNE8/WNT5A/ASS1/PLSCR1/B2M/FABP4/TFRC/LTA4H/ANXA11/NDRG1/S100A16/FOSB/PTGS2/LGMN/HIF1A/HSPA5/PRNP/MT1X	25
BP	GO:0050866	negative regulation of cell activation	19/418	221/18903	5.09467368950713e-07	2.55904873829266e-05	1.8621910727164e-05	CEACAM1/LYN/ANXA1/HMOX1/LGALS3/DLG1/GRN/IL4R/TNFAIP3/RUNX1/ZC3H12A/TNFRSF21/THBD/CEBPB/SDC4/ID2/LGALS7B/IRF1/PRNP	19
BP	GO:0022408	negative regulation of cell-cell adhesion	18/418	202/18903	6.01751909973232e-07	2.98824528020798e-05	2.1745125837669e-05	CEACAM1/ANXA1/IL1RN/LGALS3/VEGFA/DLG1/IL4R/ASS1/RUNX1/ZC3H12A/TNFRSF21/CEBPB/FXYD5/SDC4/BMP2/LGALS7B/IRF1/PRNP	18

BP	GO:0030593	neutrophil chemotaxis	13/418	106/18903	6.19844323615803e-07	3.04350527438321e-05	2.21472466190815e-05	S100A8/S100A9/CXCL1/S100A12/SAA1/CXCL6/CXCL8/IL1B/LGALS3/CXCL3/CCL20/CXCL2/RAC1	13
BP	GO:0042129	regulation of T cell proliferation	17/418	182/18903	6.38634855239446e-07	3.10092701932931e-05	2.25650982184604e-05	IL1A/ANXA1/IL1B/CD24/LGALS3/CD55/DLG1/TNFRSF21/CEBPB/CD46/TFRC/SDC4/HLA-E/LGALS7B/IRF1/SELENOK/PRNP	17
BP	GO:0030856	regulation of epithelial cell differentiation	16/418	164/18903	7.61420804297373e-07	3.65649331294453e-05	2.66078918424796e-05	AQP3/IL1A/CEACAM1/IL1B/CD24/ALOX15B/VEGFA/CEBPB/SULT2B1/CDKN2B/BMP2/GRHL1/ID1/ZFP36/ERRFI1/SFN	16
BP	GO:0001666	response to hypoxia	22/418	296/18903	7.95066440602807e-07	3.77656559286333e-05	2.74816443599666e-05	AQP3/IL1A/PLAT/SOD2/HMOX1/ERO1A/CD24/VEGFA/CAPN2/PLAU/TFRC/CITED2/AGTRAP/ADM/NDRG1/MDM2/BMP2/PTGS2/HIF1A/ATP1B1/PMAIP1/CRYAB	22
BP	GO:0016485	protein processing	20/418	250/18903	8.05022321126999e-07	3.78273929389783e-05	2.7526569690149e-05	PLAUR/TMPRSS2/PRSS3/PLAT/CTSL/ERO1A/MME/KLK13/RUNX1/CAPN2/PLAU/THBD/ANXA2/F3/S100A10/HM13/LGMN/IFI16/MAFB/PRNP	20
BP	GO:0031960	response to corticosteroid	16/418	165/18903	8.26379425846618e-07	3.841785203138e-05	2.79562401509813e-05	CLDN1/PLAT/GJB2/ANXA1/IL1RN/PAPPA/ASS1/AREG/FBXO32/FOSL1/ADM/ANXA3/ZFP36/FOSB/PTGS2/ERRFI1	16
BP	GO:00	positive regulation of T cell activation	20/418	251/18903	8.57196482674812e-07	3.94310382030414e-05	2.86935243674306e-05	IL1A/VNN1/LYN/ANXA1/IL1B/CD24/NFKBIZ/CD47/CD55/MAP3K8/IL4R/RUNX1/B2M/CD46/TFRC/DUSP10/HSPH1/XBP1/HLA-E/SELENOK	20

	508 70								
BP	GO :00 512 50	negative regulation of lymphocyte activation	16/418	166/189 03	8.963009 44322418 e-07	4.07984 229242 167e-05	2.968855 48967984 e-05	CEACAM1/LYN/ANXA1/LGALS3/DLG1/IL4R/TNFAIP3/RUNX1/ZC3H12A/TN FRSF21/CEBPB/SDC4/ID2/LGALS7B/IRF1/PRNP	16
BP	GO :00 069 53	acute-phase response	9/418	48/1890 3	9.064157 27649638 e-07	4.07984 229242 167e-05	2.968855 48967984 e-05	SAA1/IL1A/SAA2/IL1B/ASS1/PLSCR1/CEBPB/TFRC/PTGS2	9
BP	GO :19 030 39	positive regulation of leukocyte cell-cell adhesion	21/418	275/189 03	9.149303 08140328 e-07	4.07984 229242 167e-05	2.968855 48967984 e-05	IL1A/VNN1/LYN/ANXA1/IL1B/CD24/NFKBIZ/CD47/CD55/MAP3K8/IL4R/RUN X1/HAS2/B2M/CD46/TFRC/DUSP10/HSPH1/XBP1/HLA-E/SELENOK	21
BP	GO :00 458 24	negative regulation of innate immune response	11/418	77/1890 3	9.767989 90388122 e-07	4.31172 887676 373e-05	3.137596 75700427 e-05	SERPINB4/CEACAM1/GRN/TNFAIP3/MMP12/DUSP10/ISG15/HLA-B/OAS1/HLA-E/IFI16	11
BP	GO :00 512 51	positive regulation of lymphocyte activation	26/418	400/189 03	1.046272 30296425 e-06	4.57220 996395 376e-05	3.327145 92342631 e-05	IL1A/VNN1/LYN/ANXA1/CLEC7A/IL1B/CD24/NFKBIZ/CD47/CD55/MAP3K8/I L4R/RUNX1/CDKN1A/B2M/CD46/AKIRIN2/TFRC/DUSP10/HSPH1/IGHG4/XB P1/IGKC/HLA-E/IGHG3/SELENOK	26
BP	GO :00 021 38	retinoic acid biosynthetic process	5/418	10/1890 3	1.187295 97695821 e-06	5.13711 229634 395e-05	3.738219 0165615e -05	RDH10/DHRS9/ALDH1A3/CRABP2/RBP1	5

BP	GO:0050891	multicellular organismal water homeostasis	10/418	64/18903	1.31012004568874e-06	5.61296529378412e-05	4.08449190714726e-05	AQP3/CLDN1/TMPRSS11F/CLDN4/KRT16/UGCG/HAS2/GRHL1/AKR1B1/SFN	10
BP	GO:2001242	regulation of intrinsic apoptotic signaling pathway	16/418	172/18903	1.44014260365459e-06	6.11011959026266e-05	4.44626551419571e-05	S100A8/S100A9/PLAUR/VNN1/SOD2/MUC1/IL19/HSPB1/CYLD/SKIL/MDM2/XBP1/SLC9A3R1/PTGS2/HIF1A/PMAIP1	16
BP	GO:0002274	myeloid leukocyte activation	19/418	237/18903	1.47021953239268e-06	6.17774938130387e-05	4.49547895481609e-05	S100A12/CXCL6/LYN/CXCL8/ANXA1/ADGRE2/HMOX1/CD177/DNASE1L3/GRN/WNT5A/IL4R/CTSC/ZC3H12A/PLSCR1/S100A13/VAMP8/ANXA3/NDRG1	19
BP	GO:0002699	positive regulation of immune effector process	20/418	261/18903	1.57691996228233e-06	6.56299070016552e-05	4.77581474291221e-05	LYN/ANXA1/CLEC7A/IL1B/ADGRE2/HMOX1/CD177/NFKBIZ/CD55/WNT5A/IL4R/RSAD2/B2M/CD46/TFRC/VAMP8/XBP1/HLA-B/HLA-E/KLK7	20
BP	GO:0036293	response to decreased oxygen levels	22/418	309/18903	1.62791271680848e-06	6.70149222055701e-05	4.87660074630922e-05	AQP3/IL1A/PLAT/SOD2/HMOX1/ERO1A/CD24/VEGFA/CAPN2/PLAU/TFRC/CITED2/AGTRAP/ADM/NDRG1/MDM2/BMP2/PTGS2/HIF1A/ATP1B1/PMAIP1/CRYAB	22
BP	GO:0032102	negative regulation of response to external stimulus	27/418	436/18903	1.64204861186179e-06	6.70149222055701e-05	4.87660074630922e-05	SERPINB4/PLAUR/SAA1/CEACAM1/LYN/PLAT/CXCL17/SERPINB2/GRN/WNT5A/TNFAIP3/PLAU/THBD/CYLD/TRIB1/ANXA2/DUSP1/MMP12/DUSP10/ISG15/HLA-B/OAS1/HLA-E/PLK2/ZFP36/IFI16/RIOK3	27

BP	GO:0050670	regulation of lymphocyte proliferation	19/418	239/18903	1.66699381393529e-06	6.70149222055701e-05	4.87660074630922e-05	IL1A/LYN/ANXA1/IL1B/CD24/LGALS3/CD55/DLG1/CDKN1A/TNFRSF21/CEBPB/CD46/TFRC/SDC4/HLA-E/LGALS7B/IRF1/SELENOK/PRNP	19
BP	GO:0010755	regulation of plasminogen activation	6/418	18/18903	1.67153924952109e-06	6.70149222055701e-05	4.87660074630922e-05	PLAUR/PLAT/RUNX1/PLAU/ANXA2/S100A10	6
BP	GO:0050729	positive regulation of inflammatory response	15/418	154/18903	1.72107271541955e-06	6.83735251489402e-05	4.97546475912196e-05	S100A8/S100A9/S100A12/IL1B/NFKBIZ/CD47/GRN/WNT5A/CTSC/NFKBIA/FABP4/CEBPB/VAMP8/HLA-E/PTGS2	15
BP	GO:0002832	negative regulation of response to biotic stimulus	13/418	116/18903	1.75524054827821e-06	6.91027134772594e-05	5.0285269761484e-05	SERPINB4/CEACAM1/GRN/TNFAIP3/TRIB1/MMP12/DUSP10/ISG15/HLA-B/OAS1/HLA-E/IFI16/RIOK3	13
BP	GO:0002703	regulation of leukocyte mediated immunity	19/418	241/18903	1.88739094007148e-06	7.3641950072432e-05	5.35884213341724e-05	SERPINB4/CEACAM1/CXCL6/LYN/CLEC7A/IL1B/ADGRE2/HMOX1/CD177/DNASE1L3/CD55/IL4R/RSAD2/B2M/CD46/TFRC/VAMP8/HLA-B/HLA-E	19
BP	GO:0050727	regulation of inflammatory response	26/418	414/18903	1.98223376609985e-06	7.66580668836845e-05	5.57832157185622e-05	S100A8/S100A9/S100A12/SAA1/LYN/CXCL17/ANXA1/IL1B/NFKBIZ/DNASE1L3/CD47/GRN/WNT5A/TNFAIP3/CTSC/NFKBIA/CYLD/FABP4/CEBPB/VAMP8/DUSP10/HLA-E/PLK2/ZFP36/TMSB4X/PTGS2	26
BP	GO:00	regulation of mononuclear	19/418	243/18903	2.13391237964439e-06	8.12122474733132e-05	5.90972418684522e-05	IL1A/LYN/ANXA1/IL1B/CD24/LGALS3/CD55/DLG1/CDKN1A/TNFRSF21/CEBPB/CD46/TFRC/SDC4/HLA-E/LGALS7B/IRF1/SELENOK/PRNP	19

	32944	cell proliferation							
BP	GO:0016102	diterpenoid biosynthetic process	5/418	11/18903	2.13716440719245e-06	8.12122474733132e-05	5.90972418684522e-05	RDH10/DHRS9/ALDH1A3/CRABP2/RBP1	5
BP	GO:0045444	fat cell differentiation	19/418	244/18903	2.26781327695723e-06	8.54340001750267e-05	6.21693639717585e-05	LRG1/STEAP4/SOD2/ERO1A/WNT5A/ZC3H12A/SORT1/FABP4/CEBPB/ADRB2/DUSP10/BMP2/XBP1/CEBPD/ID2/PIM1/ZFP36/PTGS2/MAFB	19
BP	GO:0070663	regulation of leukocyte proliferation	20/418	269/18903	2.50984683837817e-06	9.37438519975437e-05	6.8216349966176e-05	IL1A/LYN/ANXA1/IL1B/CD24/LGALS3/CD55/DLG1/TNFAIP3/CDKN1A/TNFRSF21/CEBPB/CD46/TFRC/SDC4/HLA-E/LGALS7B/IRF1/SELENOK/PRNP	20
BP	GO:0030104	water homeostasis	10/418	69/18903	2.665418275654e-06	9.87108293610848e-05	7.18307636998283e-05	AQP3/CLDN1/TMPRSS11F/CLDN4/KRT16/UGCG/HAS2/GRHL1/AKR1B1/SFN	10
BP	GO:0043616	keratinocyte proliferation	9/418	55/18903	2.99726305830248e-06	0.000110067559367915	8.00949287848898e-05	CRNN/SDR16C5/LRG1/TGM1/HAS2/AREG/CDH3/ZFP36/SFN	9
BP	GO:0034754	cellular hormone metabolic process	14/418	141/18903	3.03395208151124e-06	0.000110486421635034	8.03997301600478e-05	SDR16C5/RDH10/DHRS9/TIPARP/ALDH1A3/DHRS3/SRD5A3/CRABP2/ADM/BMP2/SCPEP1/RBP1/AKR1B1/AKR1B10	14

BP	GO:0050792	regulation of viral process	15/418	162/18903	3.24756464678949e-06	0.00011696653892188	8.51152388493311e-05	APOBEC3A/TMPRSS2/SLPI/CXCL8/OASL/PLSCR1/RSAD2/N4BP1/STOM/MX1/ISG15/OAS1/ZFP36/PKN2/IFI16	15
BP	GO:0034142	toll-like receptor 4 signaling pathway	8/418	42/18903	3.26542740239572e-06	0.00011696653892188	8.51152388493311e-05	TNIP3/LYN/CD14/TNFAIP3/LY96/S100A14/NFKBIA/OAS1	8
BP	GO:0002548	monocyte chemotaxis	10/418	71/18903	3.47982748822704e-06	0.000123632895313432	8.99662716468454e-05	S100A7/S100A12/LYN/CXCL17/ANXA1/LGALS3/CCL20/S100A14/DUSP1/LGMN	10
BP	GO:0009266	response to temperature stimulus	16/418	186/18903	4.01853329321954e-06	0.00014162089105943	0.000103055934455146	CRNN/IL1A/LYN/CD14/SLC52A3/SOD2/HMOX1/ERO1A/HSPB1/NFKBIA/ADM/ADRB2/HSPA6/PTGS2/DNAJB6/CRYAB	16
BP	GO:0033561	regulation of water loss via skin	7/418	31/18903	4.08600253961765e-06	0.000142846648785033	0.000103947904607873	CLDN1/TMPRSS11F/CLDN4/KRT16/UGCG/GRHL1/SFN	7
BP	GO:0002685	regulation of leukocyte migration	18/418	231/18903	4.14515295150062e-06	0.000143764431730617	0.000104615764966444	S100A7/ECM1/IL1A/LYN/CXCL17/CXCL8/ANXA1/HMOX1/LGALS3/VEGFA/CCL20/CD47/WNT5A/S100A14/DUSP1/RAC1/LGMN/SELENOK	18
BP	GO:00	cellular response to	22/418	328/18903	4.30816324941891e-06	0.00014824152	0.000107873693961828	AQP3/LYN/IL1B/HMOX1/CYP24A1/CDA/GLUL/ZC3H12A/CDKN1A/FOSL1/CDKN2B/UPP1/XBP1/MTPN/PIM1/PTGS2/IFI16/SH3GLB1/GADD45A/HSPA5/IRF1/PMAIP1	22

	71496	external stimulus				2834336			
BP	GO:0006979	response to oxidative stress	26/418	434/18903	4.67715624826389e-06	0.000159681037538384	0.0001161981005428060	S100A7/IL1A/VNN1/GJB2/DUOX2/ANXA1/NCOA7/SOD2/HMOX1/ERO1A/GPX3/TNFAIP3/ZC3H12A/AREG/NET1/CAPN2/HSPB1/FOSL1/GCLM/PNPLA8/PTGS2/HIF1A/SELENOK/CRYAB/SETX/PRNP	26
BP	GO:1903038	negative regulation of leukocyte cell-cell adhesion	14/418	147/18903	4.95961585959078e-06	0.000168011793072959	0.000122260297934098	CEACAM1/ANXA1/LGALS3/DLG1/IL4R/ASS1/RUNX1/ZC3H12A/TNFRSF21/CEBPB/SDC4/LGALS7B/IRF1/PRNP	14
BP	GO:0002443	leukocyte mediated immunity	27/418	463/18903	5.04841526411501e-06	0.000169704420801405	0.000123492004152967	SERPINB4/CEACAM1/CXCL6/LYN/CLEC7A/IL1B/ADGRE2/HMOX1/CD177/BCL3/DNASE1L3/CD55/IL4R/CTSC/RSAD2/B2M/S100A13/CD46/TFRC/VAMP8/EMP2/ANXA3/IGHG4/HLA-B/IGKC/HLA-E/IGHG3	27
BP	GO:0042098	T cell proliferation	17/418	212/18903	5.22190382734873e-06	0.000173621776402295	0.000126342619899153	IL1A/ANXA1/IL1B/CD24/LGALS3/CD55/DLG1/TNFRSF21/CEBPB/CD46/TFRC/SDC4/HLA-E/LGALS7B/IRF1/SELENOK/PRNP	17
BP	GO:0048525	negative regulation of viral process	11/418	91/18903	5.24441063732334e-06	0.000173621776402295	0.000126342619899153	APOBEC3A/SLPI/OASL/PLSCR1/RSAD2/N4BP1/MX1/ISG15/OAS1/ZFP36/IFI16	11
BP	GO:0050868	negative regulation of T cell activation	13/418	128/18903	5.31541738650785e-06	0.00017464942841383	0.000127090430745075	CEACAM1/ANXA1/LGALS3/DLG1/IL4R/RUNX1/ZC3H12A/TNFRSF21/CEBPB/SDC4/LGALS7B/IRF1/PRNP	13

BP	GO:1903706	regulation of hemopoiesis	25/418	413/18903	5.96085770207191e-06	0.000194395135507867	0.000141459160392453	CEACAM1/PRDM1/VNN1/LYN/ANXA1/CSF3/CIB1/NFKBIZ/IL4R/RUNX1/ZC3H12A/NFKBIA/B2M/CEBPB/CD46/TRIB1/DUSP10/XBP1/ISG15/HLA-B/ID2/ZFP36/HIF1A/IRF1/MAFB	25
BP	GO:0048732	gland development	26/418	441/18903	6.22779488759977e-06	0.000201596027102304	0.000146699168463461	CEACAM1/CLDN1/ELF3/ANXA1/SOD2/HMOX1/TGFA/EPHA2/ALDH1A3/ALOX15B/VEGFA/PITX2/WNT5A/ASS1/TNFAIP3/AREG/CEBPB/CITED2/CDKN2B/PITX1/BMP2/XBP1/ID2/SLC9A3R1/HIF1A/MAFB	26
BP	GO:0001890	placenta development	14/418	150/18903	6.27980458958433e-06	0.000201618613337 36	0.000146715604213457	PRDM1/GJB2/MME/SPINT1/CEBPB/FOSL1/CITED2/ADM/CTSB/PHLDA2/PTGS2/HIF1A/DNAJB6/KRT19	14
BP	GO:0002886	regulation of myeloid leukocyte mediated immunity	9/418	60/18903	6.32076659661747e-06	0.000201618613337 36	0.000146715604213457	CXCL6/LYN/ADGRE2/HMOX1/CD177/DNASE1L3/IL4R/VAMP8/HLA-E	9
BP	GO:0070482	response to oxygen levels	22/418	337/18903	6.64195377459098e-06	0.000209238817141 03	0.000152260741077454	AQP3/IL1A/PLAT/SOD2/HMOX1/ERO1A/CD24/VEGFA/CAPN2/PLAU/TFRC/CITED2/AGTRAP/ADM/NDRG1/MDM2/BMP2/PTGS2/HIF1A/ATP1B1/PMAIP1/CRYAB	22
BP	GO:0010837	regulation of keratinocyte proliferation	8/418	46/18903	6.6819064958506e-06	0.000209238817141 03	0.000152260741077454	CRNN/LRG1/TGM1/HAS2/AREG/CDH3/ZFP36/SFN	8

BP	GO:0006469	negative regulation of protein kinase activity	17/418	216/18903	6.70330306630302e-06	0.00020923881714103	0.000152260741077454	CEACAM1/GPRC5A/LYN/IL1B/SERPINB3/TNFAIP3/CDKN1A/HSPB1/FABP4/TRIB1/DUSP1/CDKN2B/DUSP10/BMP2/ERRFI1/GADD45A/SFN	17
BP	GO:0007249	I-kappaB kinase/NF-kappaB signaling	20/418	288/18903	7.02770940150881e-06	0.000217809149536124	0.000158497275863816	ECM1/S100A12/IL1A/TNIP3/CLEC7A/IL1B/HMOX1/TNFSF10/BCL3/WNT5A/TNFAIP3/ZC3H12A/HSPB1/NFKBIA/S100A13/TFRC/S100A4/PLK2/SHISA5/RIOK3	20
BP	GO:1903131	mononuclear cell differentiation	27/418	473/18903	7.46114703772919e-06	0.000229614172921666	0.000167087659013935	IL1A/PRDM1/VNN1/LYN/LY6D/ANXA1/IL1B/CTSL/ST3GAL1/BCL3/NFKBIZ/VEGFA/IL4R/RUNX1/ZC3H12A/RSAD2/B2M/CEBPB/CD46/DUSP10/XBP1/KLF6/HLA-B/ID2/IFI16/IRF1/MAFB	27
BP	GO:0002366	leukocyte activation involved in immune response	20/418	293/18903	9.06855005414975e-06	0.000277129816340101	0.000201664259945428	CEACAM1/LYN/ANXA1/ST3GAL1/ADGRE2/HMOX1/CD177/BCL3/NFKBIZ/LGALS3/DNASE1L3/GRN/IL4R/ZC3H12A/S100A13/CD46/TFRC/VAMP8/ANXA3/XBP1	20
BP	GO:0032623	interleukin-2 production	9/418	63/18903	9.5445155740435e-06	0.00028765195212807	0.000209321100175575	IL1A/ANXA1/CLEC7A/IL1B/TNFAIP3/RUNX1/EZR/ZFP36/PRNP	9
BP	GO:0032663	regulation of interleukin-2 production	9/418	63/18903	9.5445155740435e-06	0.00028765195212807	0.000209321100175575	IL1A/ANXA1/CLEC7A/IL1B/TNFAIP3/RUNX1/EZR/ZFP36/PRNP	9

BP	GO:0031098	stress-activated protein kinase signaling cascade	18/418	246/18903	9.91778371608367e-06	0.000296854211227984	0.0002160174809393 57	IL1A/LYN/IL1B/SERPINB3/VEGFA/DLG1/WNT5A/ZC3H12A/CYLD/TRIB1/DUSP1/DUSP10/BMP2/TPD52L1/EZR/ZFP36/GADD45A/CRYAB	18
BP	GO:0032642	regulation of chemokine production	11/418	98/18903	1.0812319560193e-05	0.000317112996496936	0.000230759571821569	CXCL6/CLEC7A/IL1B/HMOX1/EPHA2/ALOX15B/WNT5A/IL4R/OAS1/HIF1A/SELENOK	11
BP	GO:1990868	response to chemokine	11/418	98/18903	1.0812319560193e-05	0.000317112996496936	0.000230759571821569	CXCL1/CXCL6/LYN/CXCL8/CIB1/CXCL3/CCL20/CXCL2/ZC3H12A/DUSP1/HIF1A	11
BP	GO:1990869	cellular response to chemokine	11/418	98/18903	1.0812319560193e-05	0.000317112996496936	0.000230759571821569	CXCL1/CXCL6/LYN/CXCL8/CIB1/CXCL3/CCL20/CXCL2/ZC3H12A/DUSP1/HIF1A	11
BP	GO:0002263	cell activation involved in immune response	20/418	297/18903	1.10705580830173e-05	0.000322522258818572	0.000234695831359967	CEACAM1/LYN/ANXA1/ST3GAL1/ADGRE2/HMOX1/CD177/BCL3/NFKBIZ/LGALS3/DNASE1L3/GRN/IL4R/ZC3H12A/S100A13/CD46/TFRC/VAMP8/ANXA3/XBP1	20
BP	GO:0061844	antimicrobial humoral immune response mediated by	10/418	81/18903	1.16309104955224e-05	0.000336603171294257	0.000244942353481862	S100A7/S100A9/CXCL1/S100A12/CXCL6/CXCL8/PGLYRP4/CXCL3/CXCL2/KLK7	10

		antimicrobial peptide							
BP	GO:0032602	chemokine production	11/418	99/18903	1.19259687736414e-05	0.000342871602242191	0.000249503820395919	CXCL6/CLEC7A/IL1B/HMOX1/EPHA2/ALOX15B/WNT5A/IL4R/OAS1/HIF1A/SELENOK	11
BP	GO:0002822	regulation of adaptive immune response based on somatic recombination of immune receptors built from immunoglobulin superfamily domains	15/418	182/18903	1.34434854708707e-05	0.000383974062141862	0.000279413619590646	CEACAM1/ANXA1/CLEC7A/IL1B/NFKBIZ/CD55/IL4R/TNFAIP3/ZC3H12A/RSAD2/B2M/CD46/TFRC/HLA-B/HLA-E	15
BP	GO:0050873	brown fat cell differentiation	8/418	51/18903	1.48055634225805e-05	0.000420131897121279	0.000305725270674065	LRG1/ERO1A/FABP4/CEBPB/ADRB2/DUSP10/PIM1/PTGS2	8
BP	GO:0009615	response to virus	24/418	409/18903	1.53156340983728e-05	0.000426345107666238	0.000310246554320055	APOBEC3A/DUOX2/IL1B/BCL3/OASL/TNFAIP3/ZC3H12A/PLSCR1/RSAD2/HSPB1/FOSL1/VAMP8/MMP12/IFI27/MX1/ISG15/OAS1/HIF1A/IFI16/RIOK3/IRF1/SELENOK/PMAIP1/NPC2	24

BP	GO:0032640	tumor necrosis factor production	15/418	184/18903	1.53172040969335e-05	0.000426345107666238	0.000310246554320055	IL1A/CD14/CLEC7A/BCL3/CD47/WNT5A/TNFAIP3/ZC3H12A/LY96/HSPB1/OAS1/HLA-E/ZFP36/ERRFI1/SELENOK	15
BP	GO:0032680	regulation of tumor necrosis factor production	15/418	184/18903	1.53172040969335e-05	0.000426345107666238	0.000310246554320055	IL1A/CD14/CLEC7A/BCL3/CD47/WNT5A/TNFAIP3/ZC3H12A/LY96/HSPB1/OAS1/HLA-E/ZFP36/ERRFI1/SELENOK	15
BP	GO:0006720	isoprenoid metabolic process	12/418	121/18903	1.55655849053511e-05	0.000430516493901168	0.000313282025310232	SDR16C5/RDH10/DHRS9/ALDH1A3/DHRS3/SRD5A3/CRABP2/SCPEP1/RBP1/AKR1B1/AKR1B10/NPC2	12
BP	GO:0008630	intrinsic apoptotic signaling pathway in response to DNA damage	11/418	102/18903	1.58860201661245e-05	0.000432929957087119	0.000315038275408933	SOD2/HMOX1/EPHA2/BCL2A1/MUC1/BCL3/CDKN1A/SKIL/SHISA5/IFI16/SFN	11
BP	GO:0031341	regulation of cell killing	11/418	102/18903	1.58860201661245e-05	0.000432929957087119	0.000315038275408933	SERPINB4/CEACAM1/CXCL6/CFH/CLEC7A/DNASE1L3/CD55/CD59/B2M/HLA-B/HLA-E	11
BP	GO:0045576	mast cell activation	9/418	67/18903	1.59500510505781e-05	0.000432929957087119	0.000315038275408933	S100A12/LYN/ADGRE2/HMOX1/IL4R/PLSCR1/S100A13/VAMP8/NDRG1	9

BP	GO:0043122	regulation of I-kappaB kinase/NF-kappaB signaling	18/418	255/18903	1.61627339209312e-05	0.000435994736015243	0.000317268480670131	ECM1/S100A12/IL1A/TNIP3/CLEC7A/IL1B/HMOX1/TNFSF10/WNT5A/TNFAIP3/ZC3H12A/HSPB1/S100A13/TFRC/S100A4/PLK2/SHISA5/RIOK3	18
BP	GO:0046651	lymphocyte proliferation	20/418	305/18903	1.63095423230668e-05	0.000437255827925166	0.000318186163112592	IL1A/LYN/ANXA1/IL1B/CD24/LGALS3/CD55/DLG1/CDKN1A/TNFRSF21/CEBPB/CD46/TFRC/SDC4/EMP2/HLA-E/LGALS7B/IRF1/SELENOK/PRNP	20
BP	GO:1903900	regulation of viral life cycle	13/418	142/18903	1.65278912927343e-05	0.000440407835056395	0.000320479843359116	APOBEC3A/TMPRSS2/SLPI/CXCL8/OASL/PLSCR1/RSAD2/N4BP1/MX1/ISG15/OAS1/PKN2/IFI16	13
BP	GO:0045936	negative regulation of phosphate metabolic process	25/418	440/18903	1.75862862957667e-05	0.000465770127954547	0.000338935699518412	CEACAM1/GPRC5A/LYN/IL1B/CIB1/PLEK/SERPINB3/LGALS3/CDA/TNFAIP3/ZC3H12A/CDKN1A/HSPB1/PPP1R15A/FABP4/TRIB1/DUSP1/CDKN2B/PTPN13/DUSP10/BMP2/ERRFI1/GADD45A/SFN/PRNP	25
BP	GO:0010563	negative regulation of phosphorus metabolic process	25/418	441/18903	1.82688879428921e-05	0.000480933977773727	0.000349970263002392	CEACAM1/GPRC5A/LYN/IL1B/CIB1/PLEK/SERPINB3/LGALS3/CDA/TNFAIP3/ZC3H12A/CDKN1A/HSPB1/PPP1R15A/FABP4/TRIB1/DUSP1/CDKN2B/PTPN13/DUSP10/BMP2/ERRFI1/GADD45A/SFN/PRNP	25
BP	GO:0071675	regulation of mononuclear cell migration	12/418	123/18903	1.83859446688007e-05	0.0004811117234746463	0.000350103617046625	S100A7/ECM1/LYN/CXCL17/LGALS3/CCL20/CD47/WNT5A/S100A14/DUSP1/LGMN/SELENOK	12

BP	GO:2000116	regulation of cysteine-type endopeptidase activity	17/418	235/18903	2.02255168096656e-05	0.006104217013325	0.000382840139611527	S100A8/S100A9/PLAUR/CLEC7A/TNFSF10/VEGFA/CTSD/F3/PTGS2/LGMN/IFI16/TNFAIP8/DNAJB6/CST3/PMAIP1/SFN/CRYAB	17
BP	GO:0043618	regulation of transcription from RNA polymerase II promoter in response to stress	7/418	39/18903	2.05185702462178e-05	0.006007447952799833	0.000383817961076309	HMOX1/MUC1/VEGFA/CEBPB/CITED2/HIF1A/HSPA5	7
BP	GO:0045730	respiratory burst	7/418	39/18903	2.05185702462178e-05	0.00052744795279983 3	0.000383817961076309	CLEC7A/CD24/CD55/GRN/DUSP10/RAC1/SELENOK	7
BP	GO:0001906	cell killing	15/418	189/18903	2.10501129500102e-05	0.000528672376962899	0.000384708960810531	SERPINB4/S100A12/CEACAM1/CXCL6/CFH/CLEC7A/PGLYRP4/DNASE1L3/CD55/CTSC/CD59/B2M/EMP2/HLA-B/HLA-E	15
BP	GO:0007565	female pregnancy	15/418	189/18903	2.10501129500102e-05	0.000528672376962899	0.000384708960810531	PRDM1/GJB2/IL1B/PAPPA/CLDN4/VEGFA/CAPN2/THBD/FOSL1/CITED2/ADM/EMP2/CTSB/FOSB/PTGS2	15
BP	GO:0071706	tumor necrosis factor superfamily	15/418	189/18903	2.10501129500102e-05	0.000528672376962899	0.000384708960810531	IL1A/CD14/CLEC7A/BCL3/CD47/WNT5A/TNFAIP3/ZC3H12A/LY96/HSPB1/OAS1/HLA-E/ZFP36/ERRFI1/SELENOK	15

		cytokine production							
BP	GO:1903555	regulation of tumor necrosis factor superfamily cytokine production	15/418	189/18903	2.10501129500102e-05	0.000528672376962899	0.000384708960810531	IL1A/CD14/CLEC7A/BCL3/CD47/WNT5A/TNFAIP3/ZC3H12A/LY96/HSPB1/OAS1/HLA-E/ZFP36/ERRFI1/SELENOK	15
BP	GO:0001523	retinoid metabolic process	10/418	87/18903	2.20208367717096e-05	0.000549114146855946	0.000399584207551924	SDR16C5/RDH10/DHRS9/ALDH1A3/DHRS3/CRABP2/SCPEP1/RBP1/AKR1B1/AKR1B10	10
BP	GO:0031639	plasminogen activation	6/418	27/18903	2.25137429445513e-05	0.000549114146855946	0.000399584207551924	PLAUR/PLAT/RUNX1/PLAU/ANXA2/S100A10	6
BP	GO:0042730	fibrinolysis	6/418	27/18903	2.25137429445513e-05	0.000549114146855946	0.000399584207551924	PLAUR/PLAT/SERPINB2/PLAU/THBD/ANXA2	6
BP	GO:0061436	establishment of skin barrier	6/418	27/18903	2.25137429445513e-05	0.000549114146855946	0.000399584207551924	CLDN1/CLDN4/KRT16/UGCG/GRHL1/SFN	6
BP	GO:00	negative regulation of protein	21/418	338/18903	2.25761763527968e-05	0.00054911414	0.000399584207551924	CEACAM1/GPRC5A/LYN/IL1B/CIB1/SERPINB3/TNFAIP3/ZC3H12A/CDKN1A/HSPB1/FABP4/TRIB1/DUSP1/CDKN2B/PTPN13/DUSP10/BMP2/ERRFI1/GADD45A/SFN/PRNP	21

	01933	phosphorylation				6855946			
BP	GO:0032943	mononuclear cell proliferation	20/418	312/18903	2.26179740123731e-05	0.000549114146855946	0.000399584207551924	IL1A/LYN/ANXA1/IL1B/CD24/LGALS3/CD55/DLG1/CDKN1A/TNFRSF21/CEBPB/CD46/TFRC/SDC4/EMP2/HLA-E/LGALS7B/IRF1/SELENOK/PRNP	20
BP	GO:0032722	positive regulation of chemokine production	9/418	70/18903	2.28830941022383e-05	0.000552481332744648	0.000402034470967501	CLEC7A/IL1B/HMOX1/ALOX15B/WNT5A/IL4R/OAS1/HIF1A/SELENOK	9
BP	GO:0007568	aging	14/418	169/18903	2.45230535917816e-05	0.000588822770308162	0.000428479727592667	CLDN1/GJB2/SOD2/MME/KRT16/ASS1/CTSC/TFRC/ADM/CDKN2B/GCLM/GJB6/PTGS2/CRYAB	14
BP	GO:0002444	myeloid leukocyte mediated immunity	11/418	107/18903	2.50332784572583e-05	0.000596545488707174	0.000434099463178218	CXCL6/LYN/ADGRE2/HMOX1/CD177/DNASE1L3/IL4R/S100A13/VAMP8/ANXA3/HLA-E	11
BP	GO:0051403	stress-activated MAPK cascade	17/418	239/18903	2.51177047876705e-05	0.000596545488707174	0.000434099463178218	IL1A/IL1B/SERPINB3/VEGFA/DLG1/WNT5A/ZC3H12A/CYLD/TRIB1/DUSP1/DUSP10/BMP2/TPD52L1/EZR/ZFP36/GADD45A/CRYAB	17
BP	GO:0016114	terpenoid biosynthetic process	5/418	17/18903	2.56481267729905e-05	0.000604265332754827	0.000439717107130515	RDH10/DHRS9/ALDH1A3/CRABP2/RBP1	5

BP	GO:0002687	positive regulation of leukocyte migration	13/418	148/18903	2.57193024925395e-05	0.000604265332754827	0.000439717107130515	S100A7/IL1A/CXCL17/CXCL8/LGALS3/VEGFA/CCL20/CD47/WNT5A/S100A14/RAC1/LGMN/SELENOK	13
BP	GO:0033673	negative regulation of kinase activity	17/418	240/18903	2.64942626174376e-05	0.000619143998065253	0.000450544145045195	CEACAM1/GPRC5A/LYN/IL1B/SERPINB3/TNFAIP3/CDKN1A/HSPB1/FABP4/TRIB1/DUSP1/CDKN2B/DUSP10/BMP2/ERRFI1/GADD45A/SFN	17
BP	GO:0010952	positive regulation of peptidase activity	15/418	194/18903	2.86034631723404e-05	0.00066487837267621	0.000483824536638524	S100A8/S100A9/PRSS22/LYN/CLEC7A/CLDN4/SERPINB3/TNFSF10/GRN/CTSD/AKIRIN2/F3/LGMN/IFI16/PMAIP1	15
BP	GO:0042119	neutrophil activation	7/418	41/18903	2.88709253932666e-05	0.000667544677082407	0.000485764776458136	CXCL6/CXCL8/ANXA1/CD177/DNASE1L3/GRN/ANXA3	7
BP	GO:0001818	negative regulation of cytokine production	22/418	371/18903	2.95234324691492e-05	0.000679038946790433	0.000494129027641551	CEACAM1/ANXA1/SERPINB1/HMOX1/CD24/EPHA2/BCL3/SPINK7/TNFAIP3/ZC3H12A/TNFRSF21/CYLD/N4BP1/CDH3/RAC1/OAS1/EZR/LGALS7B/ZFP36/TMSB4X/ERRFI1/PRNP	22
BP	GO:0016101	diterpenoid metabolic process	10/418	90/18903	2.96885324486109e-05	0.000679261187436805	0.000494290749667972	SDR16C5/RDH10/DHRS9/ALDH1A3/DHRS3/CRABP2/SCPEP1/RBP1/AKR1B1/AKR1B10	10
BP	GO:00	positive regulation of proteolysis	22/418	372/18903	3.07505672726492e-05	0.00069989572386186	0.000509306270453253	S100A8/S100A9/PRSS22/LYN/CLEC7A/IL1B/CLDN4/SERPINB3/TNFSF10/GRN/CTSC/CTSD/ZC3H12A/AKIRIN2/TRIB1/ANXA2/F3/S100A10/MDM2/LGMN/IFI16/PMAIP1	22

	45862								
BP	GO:0032612	interleukin-1 production	12/418	130/18903	3.20657333093635e-05	0.000722117643090156	0.000525476911905422	SAA1/CEACAM1/ANXA1/SERPINB1/CLEC7A/WNT5A/TNFAIP3/ZC3H12A/HSPB1/S100A13/ERRFI1/IFI16	12
BP	GO:0032652	regulation of interleukin-1 production	12/418	130/18903	3.20657333093635e-05	0.000722117643090156	0.000525476911905422	SAA1/CEACAM1/ANXA1/SERPINB1/CLEC7A/WNT5A/TNFAIP3/ZC3H12A/HSPB1/S100A13/ERRFI1/IFI16	12
BP	GO:0071677	positive regulation of mononuclear cell migration	9/418	73/18903	3.22226408243891e-05	0.000722117643090156	0.000525476911905422	S100A7/CXCL17/LGALS3/CCL20/CD47/WNT5A/S100A14/LGMN/SELENOK	9
BP	GO:0046631	alpha-beta T cell activation	14/418	174/18903	3.39304123660002e-05	0.000756509704282759	0.000550503629203472	PRDM1/ANXA1/CTSL/BCL3/NFKBIZ/CD55/IL4R/RUNX1/ZC3H12A/RSAD2/HSPH1/HLA-E/LGALS7B/IRF1	14
BP	GO:0070661	leukocyte proliferation	21/418	348/18903	3.46600519484225e-05	0.000768093530931064	0.000558933049968143	IL1A/LYN/ANXA1/IL1B/CD24/LGALS3/CD55/DLG1/TNFAIP3/CDKN1A/TNFRSF21/CEBPB/CD46/TFRC/SDC4/EMP2/HLA-E/LGALS7B/IRF1/SELENOK/PRNP	21
BP	GO:0090025	regulation of monocyte chemotaxis	6/418	29/18903	3.48014917904693e-05	0.000768093530931064	0.000558933049968143	S100A7/LYN/CXCL17/S100A14/DUSP1/LGMN	6

BP	GO:0070302	regulation of stress-activated protein kinase signaling cascade	15/418	198/18903	3.62735610464139e-05	0.000796560109411199	0.000579647859937669	IL1A/LYN/IL1B/SERPINB3/VEGFA/DLG1/WNT5A/ZC3H12A/CYLD/DUSP1/DUSP10/BMP2/TPD52L1/EZR/GADD45A	15
BP	GO:0031348	negative regulation of defense response	18/418	272/18903	3.81361375095198e-05	0.000832898523817064	0.000606090916644912	SERPINB4/SAA1/CEACAM1/LYN/CXCL17/GRN/TNFAIP3/CYLD/MMP12/DUSP10/ISG15/HLA-B/OAS1/HLA-E/PLK2/ZFP36/IFI16/RIOK3	18
BP	GO:0032635	interleukin-6 production	14/418	176/18903	3.85001148309032e-05	0.000832898523817064	0.000606090916644912	IL1A/IL36A/CLEC7A/IL1B/CD47/WNT5A/TNFAIP3/ZC3H12A/CAPN2/CEBPB/AKIRIN2/XBP1/HLA-B/SELENOK	14
BP	GO:0032675	regulation of interleukin-6 production	14/418	176/18903	3.85001148309032e-05	0.000832898523817064	0.000606090916644912	IL1A/IL36A/CLEC7A/IL1B/CD47/WNT5A/TNFAIP3/ZC3H12A/CAPN2/CEBPB/AKIRIN2/XBP1/HLA-B/SELENOK	14
BP	GO:0007584	response to nutrient	13/418	154/18903	3.91041312779623e-05	0.000841798294013277	0.000612567179625222	AQP3/IL1A/KYNU/SOD2/HMOX1/CYP24A1/ASS1/TFRC/CDKN2B/GCLM/XBP1/PIM1/PTGS2	13
BP	GO:0071548	response to dexamethasone	7/418	43/18903	3.98364483438982e-05	0.000853359212072720	0.000620979930066648	CLDN1/PLAT/GJB2/PAPPA/ASS1/FBXO32/ERRFI1	7

BP	GO:0043536	positive regulation of blood vessel endothelial cell migration	9/418	75/18903	4.00960816691356e-05	0.000854731106800599	0.000621978242477324	ANXA1/HMOX1/CIB1/VEGFA/HSPB1/PLK2/TMSB4X/PTGS2/HIF1A	9
BP	GO:0097191	extrinsic apoptotic signaling pathway	16/418	224/18903	4.11583064380996e-05	0.000873115529779104	0.000635356380937655	IL1A/IL1B/HMOX1/CIB1/BCL2A1/TNFSF10/MAL/LGALS3/IL19/TNFAIP3/SORT1/CYLD/SKIL/GCLM/IFI27/PMAIP1	16
BP	GO:0030099	myeloid cell differentiation	23/418	407/18903	4.13763432346806e-05	0.000873500579398812	0.000635636577228426	CEACAM1/LYN/CSF3/CIB1/EPHA2/FAM20C/VEGFA/RUNX1/NFKBIA/B2M/CEBPB/TRIB1/TFRC/CITED2/ANXA2/CDKN2B/ISG15/PTBP3/ID2/ZFP36/HIF1A/IFI16/MAFB	23
BP	GO:0008299	isoprenoid biosynthetic process	6/418	30/18903	4.26957807739008e-05	0.000897021932605512	0.000652752802216368	RDH10/DHRS9/ALDH1A3/SRD5A3/CRABP2/RBP1	6
BP	GO:0060054	positive regulation of epithelial cell proliferation involved in wound healing	4/418	10/18903	4.45347941711986e-05	0.000931182059943244	0.000677610743848859	CLDN1/LRG1/MMP12/ODAM	4
BP	GO:0009408	response to heat	11/418	114/18903	4.52844321867932e-05	0.000942347469791839	0.000685735687400011	CRNN/IL1A/LYN/CD14/SLC52A3/HMOX1/HSPB1/HSPA6/PTGS2/DNAJB6/CRYAB	11

BP	GO:0051348	negative regulation of transferase activity	18/418	276/18903	4.61507658190987e-05	0.000955823917675172	0.000695542347415801	CEACAM1/GPRC5A/LYN/IL1B/SERPINB3/TNFAIP3/CDKN1A/HSPB1/FABP4/TRIB1/DUSP1/CDKN2B/DUSP10/BMP2/ZFP36/ERRFI1/GADD45A/SFN	18
BP	GO:0036003	positive regulation of transcription from RNA polymerase II promoter in response to stress	5/418	19/18903	4.64671520191064e-05	0.000956052706307941	0.000695708834338501	MUC1/VEGFA/CEBPB/HIF1A/HSPA5	5
BP	GO:2001235	positive regulation of apoptotic signaling pathway	12/418	135/18903	4.65993653188996e-05	0.000956052706307941	0.000695708834338501	S100A8/S100A9/VNN1/TNFSF10/MAL/IL19/CTSC/CYLD/SKIL/TPD52L1/SLC9A3R1/PMAIP1	12
BP	GO:0046634	regulation of alpha-beta T cell activation	11/418	115/18903	4.90969888783849e-05	0.00100258804391842	0.000729572077725533	PRDM1/ANXA1/NFKBIZ/CD55/IL4R/RUNX1/ZC3H12A/HSPH1/HLA-E/LGALS7B/IRF1	11
BP	GO:0044409	entry into host	13/418	158/18903	5.1089849003641e-05	0.00103843088439959	0.000755654510844551	CLDN1/TMPRSS2/CXCL8/CTSL/SERPINB3/EPHA2/CD55/PLSCR1/CD46/TFRC/VAMP8/CTSB/NECTIN4	13

BP	GO:0002275	myeloid cell activation involved in immune response	10/418	96/18903	5.20464910734542e-05	0.00105297762032868	0.000766240007470298	LYN/ADGRE2/HMOX1/CD177/DNASE1L3/GRN/IL4R/S100A13/VAMP8/ANXA3	10
BP	GO:0043620	regulation of DNA-templated transcription in response to stress	7/418	45/18903	5.40061674852768e-05	0.00108574969166197	0.000790087876312372	HMOX1/MUC1/VEGFA/CEBPB/CITED2/HIF1A/HSPA5	7
BP	GO:0034612	response to tumor necrosis factor	17/418	254/18903	5.41632569295903e-05	0.00108574969166197	0.000790087876312372	CLDN1/CD14/CXCL8/CIB1/CCL20/ASS1/TNFAIP3/ZC3H12A/HAS2/TNFRSF21/NFKBIA/CYLD/FABP4/TXNDC17/ZFP36/TMSB4X/PTGS2	17
BP	GO:0071674	mononuclear cell migration	15/418	206/18903	5.72131325209216e-05	0.00114165017861382	0.000830766033865437	S100A7/ECM1/S100A12/SAA1/LYN/CXCL17/ANXA1/LGALS3/CCL20/CD47/WNT5A/S100A14/DUSP1/LGMN/SELENOK	15
BP	GO:0044403	biological process involved in symbiotic interaction	19/418	307/18903	5.80757187162387e-05	0.0011535949581362	0.000839458115989268	SPRR2A/CLDN1/CXCL6/TMPRSS2/CXCL8/CTSL/SERPINB3/EPHA2/CD55/ZC3H12A/PLSCR1/CD46/TFRC/VAMP8/CTSB/STOM/IFI27/NECTIN4/VPS4B	19
BP	GO:00	movement in host	14/418	183/18903	5.90189309991682e-05	0.00116702592066228	0.00084923167682061	CLDN1/TMPRSS2/CXCL8/CTSL/SERPINB3/EPHA2/CD55/PLSCR1/CD46/TFRC/VAMP8/CTSB/NECTIN4/VPS4B	14

	440<br>00								
BP	GO<br>:00<br>432<br>99	leukocyte degranulation	9/418	79/1890<br>3	6.078942<br>23814561<br>e-05	0.00118<br>684016<br>506391	0.000863<br>65028029<br>8223	CEACAM1/LYN/ADGRE2/HMOX1/CD177/IL4R/S100A13/VAMP8/ANXA3	9
BP	GO<br>:00<br>607<br>49	mammary gland alveolus development	5/418	20/1890<br>3	6.083574<br>30147176<br>e-05	0.00118<br>684016<br>506391	0.000863<br>65028029<br>8223	TGFA/VEGFA/AREG/ID2/HIF1A	5
BP	GO<br>:00<br>613<br>77	mammary gland lobule development	5/418	20/1890<br>3	6.083574<br>30147176<br>e-05	0.00118<br>684016<br>506391	0.000863<br>65028029<br>8223	TGFA/VEGFA/AREG/ID2/HIF1A	5
BP	GO<br>:00<br>454<br>28	regulation of nitric oxide biosynthetic process	8/418	62/1890<br>3	6.359887<br>50702816<br>e-05	0.00123<br>500971<br>17058	0.000898<br>70271927<br>3326	CLEC7A/IL1B/SOD2/CD47/ASS1/ZC3H12A/RAC1/PTGS2	8
BP	GO<br>:00<br>452<br>16	cell-cell junction organization	15/418	208/189<br>03	6.387006<br>74703685<br>e-05	0.00123<br>500971<br>17058	0.000898<br>70271927<br>3326	CLDN1/GJB2/IL1B/CLDN4/EPHA2/CD177/VEGFA/DLG1/CLDN7/AFDN/GRHL1/HOPX/PKN2/GJB6/CTNND1	15
BP	GO<br>:00<br>516<br>04	protein maturation	20/418	336/189<br>03	6.417454<br>13152276<br>e-05	0.00123<br>543059<br>712575	0.000899<br>00899287<br>4113	PLAUR/TMPRSS2/PRSS3/PLAT/CTSL/ERO1A/MME/KLK13/RUNX1/CAPN2/PLAU/THBD/ANXA2/F3/S100A10/HM13/LGMN/IFI16/MAFB/PRNP	20

BP	GO:0044703	multi-organism reproductive process	15/418	209/18903	6.74460054358981e-05	0.00129271510418805	0.000940694286342789	PRDM1/GJB2/IL1B/PAPPA/CLDN4/VEGFA/CAPN2/THBD/FOSL1/CITED2/ADM/EMP2/CTSB/FOSB/PTGS2	15
BP	GO:0001889	liver development	12/418	141/18903	7.1283353705658e-05	0.00135776521985222	0.000988030526116672	CEACAM1/CLDN1/ANXA1/SOD2/HMOX1/TGFA/ASS1/TNFAIP3/CEBPB/CITED2/CDKN2B/XBP1	12
BP	GO:0031663	lipopolysaccharide-mediated signaling pathway	8/418	63/18903	7.14613273606433e-05	0.00135776521985222	0.000988030526116672	LYN/CD14/IL1B/CD55/TNFAIP3/LY96/NFKBIA/TRIB1	8
BP	GO:0036230	granulocyte activation	7/418	47/18903	7.20555742048675e-05	0.00136312926093191	0.000991933878664409	CXCL6/CXCL8/ANXA1/CD177/DNASE1L3/GRN/ANXA3	7
BP	GO:0002833	positive regulation of response to biotic stimulus	14/418	188/18903	7.89993863733256e-05	0.00148804878642859	0.00108283641666886	LYN/CD14/CLEC7A/OASL/GRN/WNT5A/PLSCR1/LY96/AKIRIN2/MMP12/HLA-E/IFI16/RIOK3/KLK7	14
BP	GO:0006721	terpenoid metabolic process	10/418	101/18903	8.03824771855857e-05	0.00150760268369532	0.00109706556845563	SDR16C5/RDH10/DHRS9/ALDH1A3/DHRS3/CRABP2/SCPEP1/RBP1/AKR1B1/AKR1B10	10
BP	GO:00	transition metal ion homeostasis	12/418	143/18903	8.16979016681063e-05	0.00152572577046848	0.00111025353548965	S100A8/S100A9/HEPHL1/STEAP4/LCN2/SOD2/HMOX1/B2M/TFRC/HIF1A/PRNP/MT1X	12

	55076								
BP	GO:0017157	regulation of exocytosis	15/418	213/18903	8.35633077055214e-05	0.00155392193477927	0.00113077156810025	CEACAM1/LYN/DOC2B/ANXA1/ADGRE2/HMOX1/CD177/IL4R/RAB27B/VAMP8/ANXA2/S100A10/SDC4/RAB10/VPS4B	15
BP	GO:0061008	hepaticobiliary system development	12/418	144/18903	8.7379111087011e-05	0.00161799455699253	0.0011773964968504	CEACAM1/CLDN1/ANXA1/SOD2/HMOX1/TGFA/ASS1/TNFAIP3/CEBPB/CITED2/CDKN2B/XBP1	12
BP	GO:0080164	regulation of nitric oxide metabolic process	8/418	65/18903	8.96180412366377e-05	0.00164778000651255	0.0011990710344874	CLEC7A/IL1B/SOD2/CD47/ASS1/ZC3H12A/RAC1/PTGS2	8
BP	GO:0042573	retinoic acid metabolic process	6/418	34/18903	8.97417944050316e-05	0.00164778000651255	0.0011990710344874	RDH10/DHRS9/ALDH1A3/CRABP2/SCPEP1/RBP1	6
BP	GO:0043300	regulation of leukocyte degranulation	7/418	49/18903	9.47491290678512e-05	0.00173244223442054	0.00126067878843417	CEACAM1/LYN/ADGRE2/HMOX1/CD177/IL4R/VAMP8	7
BP	GO:0030449	regulation of complement activation	5/418	22/18903	9.96305180920619e-05	0.00179911307463765	0.00130919441129238	CFH/IL1B/CD55/CD59/CD46	5

BP	GO:0072574	hepatocyte proliferation	5/418	22/18903	9.96305180920619e-05	0.00179911307463765	0.00130919441129238	CEACAM1/TGFA/TNFAIP3/CEBPB/XBP1	5
BP	GO:0072575	epithelial cell proliferation involved in liver morphogenesis	5/418	22/18903	9.96305180920619e-05	0.00179911307463765	0.00130919441129238	CEACAM1/TGFA/TNFAIP3/CEBPB/XBP1	5
BP	GO:0002460	adaptive immune response based on somatic recombination of immune receptors built from immunoglobulin superfamily domains	21/418	375/18903	0.000100922518183341	0.00181494405128065	0.00132071443548569	CEACAM1/ANXA1/CLEC7A/IL1B/BCL3/NFKBIZ/CD55/IL4R/TNFAIP3/CTSC/ZC3H12A/RSAD2/B2M/CD46/TFRC/EMP2/IGHG4/HLA-B/IGKC/HLA-E/IGHG3	21
BP	GO:0090050	positive regulation of cell migration involved in	6/418	35/18903	0.000106306050248087	0.00189430188837111	0.0013784622437117	ANXA1/HMOX1/CIB1/VEGFA/PLK2/PTGS2	6

		sprouting angiogenesis							
BP	GO:0045598	regulation of fat cell differentiation	12/418	147/18903	0.000106509092808098	0.00189430188837111	0.0013784622437117	SOD2/WNT5A/ZC3H12A/SORT1/CEBPB/DUSP10/BMP2/XBP1/ID2/PIM1/ZFP36/PTGS2	12
BP	GO:0006692	prostanoid metabolic process	7/418	50/18903	0.000108101673148339	0.00189430188837111	0.0013784622437117	ANXA1/IL1B/FABP5/PTGR1/PNPLA8/PTGS2/AKR1B1	7
BP	GO:0006693	prostaglandin metabolic process	7/418	50/18903	0.000108101673148339	0.00189430188837111	0.0013784622437117	ANXA1/IL1B/FABP5/PTGR1/PNPLA8/PTGS2/AKR1B1	7
BP	GO:0030195	negative regulation of blood coagulation	7/418	50/18903	0.000108101673148339	0.00189430188837111	0.0013784622437117	PLAUR/CEACAM1/PLAT/SERPINB2/PLAU/THBD/ANXA2	7
BP	GO:0002573	myeloid leukocyte differentiation	15/418	218/18903	0.000108369673247775	0.00189430188837111	0.0013784622437117	CEACAM1/LYN/CSF3/EPHA2/FAM20C/VEGFA/RUNX1/CEBPB/TRIB1/TFRC/CITED2/ANXA2/ID2/IFI16/MAFB	15
BP	GO:0044706	multi-multicellular organism process	15/418	218/18903	0.000108369673247775	0.00189430188837111	0.0013784622437117	PRDM1/GJB2/IL1B/PAPPA/CLDN4/VEGFA/CAPN2/THBD/FOSL1/CITED2/ADM/EMP2/CTSB/FOSB/PTGS2	15

BP	GO:0030217	T cell differentiation	18/418	296/18903	0.000113042282919858	0.00196810667872422	0.00143216916209223	IL1A/PRDM1/VNN1/ANXA1/IL1B/CTSL/BCL3/NFKBIZ/IL4R/RUNX1/ZC3H12A/RSAD2/B2M/CD46/DUSP10/XBP1/IRF1/MAFB	18
BP	GO:0032872	regulation of stress-activated MAPK cascade	14/418	195/18903	0.00011674349490109	0.00202448044729272	0.0014731917213709	IL1A/IL1B/SERPINB3/VEGFA/DLG1/WNT5A/ZC3H12A/CYLD/DUSP1/DUSP10/BMP2/TPD52L1/EZR/GADD45A	14
BP	GO:0045921	positive regulation of exocytosis	9/418	86/18903	0.000118563848275697	0.00204792101567112	0.00149024916014512	DOC2B/CD177/IL4R/RAB27B/VAMP8/ANXA2/S100A10/SDC4/VPS4B	9
BP	GO:1900047	negative regulation of hemostasis	7/418	51/18903	0.000122944236059255	0.00211522169912971	0.00153922311286784	PLAUR/CEACAM1/PLAT/SERPINB2/PLAU/THBD/ANXA2	7
BP	GO:0072576	liver morphogenesis	5/418	23/18903	0.00012500697052725	0.00214227631844738	0.00155891045598688	CEACAM1/TGFA/TNFAIP3/CEBPB/XBP1	5
BP	GO:0042326	negative regulation of phosphorylation	21/418	381/18903	0.000125923152419055	0.00214954756277839	0.00156420165895544	CEACAM1/GPRC5A/LYN/IL1B/CIB1/SERPINB3/TNFAIP3/ZC3H12A/CDKN1A/HSPB1/FABP4/TRIB1/DUSP1/CDKN2B/PTPN13/DUSP10/BMP2/ERRFI1/GADD45A/SFN/PRNP	21

BP	GO:0001892	embryonic placenta development	9/418	87/18903	0.0001297034265261 56	0.00220192334378349	0.00160231492751293	PRDM1/SPINT1/CEBPB/CITED2/ADM/PHLDA2/HIF1A/DNAJB6/KRT19	9
BP	GO:0046683	response to organophosphorus	11/418	128/18903	0.0001299991356284 08	0.00220192334378349	0.00160231492751293	PLAT/DUOX2/IL1B/ASS1/AREG/THBD/FOSL1/EZR/FOSB/PTGS2/HSPA5	11
BP	GO:0045109	intermediate filament organization	8/418	69/18903	0.000137475271150 15	0.00231161023403155	0.00168213284764767	KRT13/KRT16/KRT80/KRT4/KRT6C/DNAJB6/KRT5/KRT19	8
BP	GO:0046718	viral entry into host cell	12/418	151/18903	0.0001375328743359 73	0.00231161023403155	0.00168213284764767	CLDN1/TMPRSS2/CTSL/SERPINB3/EPHA2/CD55/PLSCR1/CD46/TFRC/VAMP8/CTSB/NECTIN4	12
BP	GO:1902106	negative regulation of leukocyte differentiation	10/418	108/18903	0.0001412844226598 73	0.00236556677020553	0.00172139641401684	CEACAM1/LYN/ANXA1/IL4R/RUNX1/ZC3H12A/TRIB1/ID2/IRF1/MAFB	10
BP	GO:0031392	regulation of prostaglandin biosynthetic process	4/418	13/18903	0.0001438355763924 24	0.00238996756210986	0.00173915259668406	ANXA1/IL1B/FABP5/PTGS2	4
BP	GO:00	leukocyte aggregation	4/418	13/18903	0.0001438355763924 24	0.00238996756210986	0.00173915259668406	S100A8/S100A9/IL1B/HAS2	4

	70486								
BP	GO:0007043	cell-cell junction assembly	12/418	152/18903	0.000146399344835737	0.00242335279140974	0.0017634466537032	CLDN1/GJB2/IL1B/CLDN4/EPHA2/DLG1/CLDN7/AFDN/HOPX/PKN2/GJB6/CTNND1	12
BP	GO:0030193	regulation of blood coagulation	8/418	70/18903	0.000152261358511171	0.00251087598752384	0.00182713630213405	PLAUR/CEACAM1/PLAT/SERPINB2/PLAU/THBD/ANXA2/F3	8
BP	GO:0007596	blood coagulation	15/418	225/18903	0.000153769617175629	0.00252621513931391	0.00183829843089662	PLAUR/SAA1/CEACAM1/LYN/PLAT/PLEK/SERPINB2/CD59/PLSCR1/PLAU/HSPB1/THBD/ANXA2/F3/RAP2B	15
BP	GO:0045104	intermediate filament cytoskeleton organization	9/418	89/18903	0.000154618514419747	0.00253064759555916	0.00184152387960598	KRT13/KRT16/KRT80/KRT4/KRT6C/PPL/DNAJB6/KRT5/KRT19	9
BP	GO:0031667	response to nutrient levels	24/418	474/18903	0.000155688501350351	0.00253865205560087	0.00184734863542581	AQP3/IL1A/KYNU/SOD2/HMOX1/CYP24A1/ASS1/GLUL/ZC3H12A/CDKN1A/TFRC/ADM/CDKN2B/ADRB2/GCLM/UPP1/XBP1/PIM1/ZFP36/PTGS2/IFI16/SH3GLB1/HSPA5/PMAIP1	24
BP	GO:0051607	defense response to virus	18/418	304/18903	0.000157761507317982	0.00256289140141108	0.00186498733558806	APOBEC3A/IL1B/OASL/TNFAIP3/ZC3H12A/PLSCR1/RSAD2/VAMP8/MMP12/IFI27/MX1/ISG15/OAS1/IFI16/RIOK3/IRF1/SELENOK/PMAIP1	18

BP	GO:0043534	blood vessel endothelial cell migration	13/418	177/18903	0.000162101422839966	0.00262364154744685	0.00190919453567071	ANXA1/HMOX1/CIB1/EPHA2/VEGFA/HSPB1/EMP2/ID1/PLK2/TMSB4X/PTGS2/HIF1A/GADD45A	13
BP	GO:0140546	defense response to symbiont	18/418	305/18903	0.000164319306922801	0.00264972461716842	0.00192817489304246	APOBEC3A/IL1B/OASL/TNFAIP3/ZC3H12A/PLSCR1/RSAD2/VAMP8/MMP12/IFI27/MX1/ISG15/OAS1/IFI16/RIOK3/IRF1/SELENOK/PMAIP1	18
BP	GO:0045103	intermediate filament-based process	9/418	90/18903	0.000168499623527651	0.00268738450662713	0.00195557957232821	KRT13/KRT16/KRT80/KRT4/KRT6C/PPL/DNAJB6/KRT5/KRT19	9
BP	GO:0051591	response to cAMP	9/418	90/18903	0.000168499623527651	0.00268738450662713	0.00195557957232821	PLAT/DUOX2/ASS1/AREG/THBD/FOSL1/EZR/FOSB/HSPA5	9
BP	GO:0070098	chemokine-mediated signaling pathway	9/418	90/18903	0.000168499623527651	0.00268738450662713	0.00195557957232821	CXCL1/CXCL6/LYN/CXCL8/CIB1/CXCL3/CCL20/CXCL2/HIF1A	9
BP	GO:0030098	lymphocyte differentiation	22/418	419/18903	0.00017507973773502	0.00278094677301578	0.00202366378448288	IL1A/PRDM1/VNN1/LY6D/ANXA1/IL1B/CTSL/ST3GAL1/BCL3/NFKBIZ/IL4R/RUNX1/ZC3H12A/RSAD2/B2M/CD46/DUSP10/XBP1/KLF6/ID2/IRF1/MAFB	22
BP	GO:20	regulation of extrinsic apoptotic	12/418	155/18903	0.000175993848733945	0.00278094677301578	0.00202366378448288	IL1A/IL1B/HMOX1/TNFSF10/MAL/LGALS3/IL19/TNFAIP3/CYLD/SKIL/GCLM/PMAIP1	12

	01236	signaling pathway							
BP	GO:0042149	cellular response to glucose starvation	7/418	54/18903	0.000177665671920932	0.00278094677301578	0.00202366378448288	ZC3H12A/UPP1/XBP1/IFI16/SH3GLB1/HSPA5/PMAIP1	7
BP	GO:0042572	retinol metabolic process	7/418	54/18903	0.000177665671920932	0.00278094677301578	0.00202366378448288	SDR16C5/RDH10/DHRS9/ALDH1A3/DHRS3/AKR1B1/AKR1B10	7
BP	GO:0050819	negative regulation of coagulation	7/418	54/18903	0.000177665671920932	0.00278094677301578	0.00202366378448288	PLAUR/CEACAM1/PLAT/SERPINB2/PLAU/THBD/ANXA2	7
BP	GO:0051701	biological process involved in interaction with host	14/418	203/18903	0.000178184232595977	0.00278094677301578	0.00202366378448288	CLDN1/TMPRSS2/CXCL8/CTSL/SERPINB3/EPHA2/CD55/PLSCR1/CD46/TFRC/VAMP8/CTSB/NECTIN4/VPS4B	14
BP	GO:0048568	embryonic organ development	23/418	449/18903	0.000179798277597869	0.00279615114983163	0.00203472783900791	RDH10/PRDM1/CXCL8/EPHA2/PLS1/ALDH1A3/VEGFA/DLG1/SPINT1/PITX2/WNT5A/CEBPB/CITED2/ADM/PHLDA2/ID2/SLC9A3R1/GJB6/HIF1A/DNAJB6/MAFB/RBBP6/KRT19	23
BP	GO:0002702	positive regulation of production of molecular	11/418	133/18903	0.000182694010146909	0.00283110930617728	0.00206016649740132	CLEC7A/IL1B/CD55/WNT5A/IL4R/RSAD2/B2M/TFRC/XBP1/HLA-E/KLK7	11

		mediator of immune response							
BP	GO:2000117	negative regulation of cysteine-type endopeptidase activity	9/418	91/18903	0.000183403766019913	0.00283206522087286	0.00206086210580679	PLAUR/VEGFA/PTGS2/IFI16/TNFAIP8/DNAJB6/CST3/SFN/CRYAB	9
BP	GO:0045600	positive regulation of fat cell differentiation	8/418	72/18903	0.00018577363756435	0.00284852910932004	0.00207284269282328	ZC3H12A/CEBPB/BMP2/XBP1/ID2/PIM1/ZFP36/PTGS2	8
BP	GO:1900046	regulation of hemostasis	8/418	72/18903	0.00018577363756435	0.00284852910932004	0.00207284269282328	PLAUR/CEACAM1/PLAT/SERPINB2/PLAU/THBD/ANXA2/F3	8
BP	GO:0031579	membrane raft organization	5/418	25/18903	0.00019032135226872	0.00290805702592415	0.00211616049026059	MAL/DLG1/ANXA2/S100A10/EMP2	5
BP	GO:0001701	in utero embryonic development	21/418	393/18903	0.000192881024423533	0.00293689922205867	0.00213714863298549	RDH10/PRDM1/ELF3/VEGFA/SPINT1/CAPN2/CEBPB/FOSL1/ZFAND5/CITED2/ADM/SKIL/PHLDA2/TM4SF1/BMP2/HM13/HOPX/HIF1A/DNAJB6/RBBP6/KRT19	21
BP	GO:00	coagulation	15/418	230/18903	0.000195566206850099	0.00296744556921851	0.00215937686730317	PLAUR/SAA1/CEACAM1/LYN/PLAT/PLEK/SERPINB2/CD59/PLSCR1/PLAU/HSPB1/THBD/ANXA2/F3/RAP2B	15

	50817								
BP	GO:0043281	regulation of cysteine-type endopeptidase activity involved in apoptotic process	14/418	205/18903	0.0001973227501101 36	0.00298373847052352	0.00217123302889354	S100A8/S100A9/PLAUR/CLEC7A/TNFSF10/VEGFA/CTSD/F3/PTGS2/TNFAIP8/DNAJB6/PMAIP1/SFN/CRYAB	14
BP	GO:0071634	regulation of transforming growth factor beta production	6/418	39/18903	0.000198321149987564	0.00298849457050226	0.00217469398951881	CD24/CD46/CDH3/LUM/PTGS2/HIF1A	6
BP	GO:0071385	cellular response to glucocorticoid stimulus	7/418	55/18903	0.000199753228387926	0.00299973061187367	0.00218287033083713	PLAT/GJB2/ANXA1/ASS1/FBXO32/ZFP36/ERRFI1	7
BP	GO:0043409	negative regulation of MAPK cascade	13/418	181/18903	0.000202271479935789	0.00302714509355959	0.00220281954176647	LYN/IL1B/SERPINB3/DLG1/DUSP5/PSCA/CYLD/DUSP1/DUSP10/BMP2/EZR/SLC9A3R1/ERRFI1	13
BP	GO:0007599	hemostasis	15/418	231/18903	0.000205013532002157	0.00305390256288062	0.00222229065216485	PLAUR/SAA1/CEACAM1/LYN/PLAT/PLEK/SERPINB2/CD59/PLSCR1/PLAU/HSPB1/THBD/ANXA2/F3/RAP2B	15

BP	GO:0002456	T cell mediated immunity	10/418	113/18903	0.0002054570602944486	0.00305390256288062	0.00222229065216485	CEACAM1/IL1B/CD55/CTSC/RSAD2/B2M/CD46/EMP2/HLA-B/HLA-E	10
BP	GO:0071900	regulation of protein serine/threonine kinase activity	21/418	395/18903	0.0002066672785504448	0.00306147799073037	0.00222780320606924	S100A12/LYN/IL1B/CD24/CIB1/TGFA/SERPINB3/VEGFA/WNT5A/TNFAIP3/CDKN1A/CCNL1/TRIB1/DUSP1/CDKN2B/ADRB2/DUSP10/BMP2/TPD52L1/GADD45A/SFN	21
BP	GO:1903707	negative regulation of hemopoiesis	10/418	114/18903	0.0002208582745356609	0.00326064412067775	0.00237273416561905	CEACAM1/LYN/ANXA1/IL4R/RUNX1/ZC3H12A/TRIB1/ID2/IRF1/MAFB	10
BP	GO:0001954	positive regulation of cell-matrix adhesion	7/418	56/18903	0.0002240047651958 26	0.00329596237005306	0.00239843485967248	CIB1/VEGFA/CEACAM6/S100A10/SDC4/EMP2/RAC1	7
BP	GO:2001234	negative regulation of apoptotic signaling pathway	15/418	233/18903	0.0002250989647498 22	0.00330094790589505	0.0024020627782028	PLAUR/IL1A/IL1B/SOD2/HMOX1/MUC1/LGALS3/IL19/TNFAIP3/HSPB1/GCLM/MDM2/XBP1/PTGS2/HIF1A	15
BP	GO:0006887	exocytosis	20/418	369/18903	0.0002263868749837 93	0.00330873124976313	0.00240772663026241	CEACAM1/LYN/RAB31/DOC2B/ANXA1/ADGRE2/HMOX1/PLEK/CD177/RAB11FIP1/IL4R/S100A13/RAB27B/VAMP8/ANXA2/S100A10/SDC4/ANXA3/RAB10/VPS4B	20

BP	GO:0140467	integrated stress response signaling	6/418	40/18903	0.000229012786731572	0.00333595292672323	0.00242753553935466	PPP1R15A/CEBPB/FOSL1/CEBPD/HSPA5/MAFB	6
BP	GO:0033598	mammary gland epithelial cell proliferation	5/418	26/18903	0.000231387941857 63	0.00334822948979419	0.00243646905664657	EPHA2/WNT5A/AREG/CEBPB/ID2	5
BP	GO:0051238	sequestering of metal ion	5/418	26/18903	0.00023138794185763	0.00334822948979419	0.00243646905664657	S100A7/S100A8/S100A9/LCN2/ANXA1	5
BP	GO:0071356	cellular response to tumor necrosis factor	15/418	234/18903	0.00023576430409762	0.00338769957839667	0.00246519099755181	CLDN1/CXCL8/CIB1/CCL20/ASS1/TNFAIP3/ZC3H12A/HAS2/TNFRSF21/NFKBIA/CYLD/FABP4/TXNDC17/ZFP36/TMSB4X	15
BP	GO:0120254	olefinic compound metabolic process	12/418	160/18903	0.000236675441000866	0.00338769957839667	0.00246519099755181	SDR16C5/RDH10/DHRS9/ALDH1A3/ALOX15B/DHRS3/ADM/BMP2/PNPLA8/PTGS2/AKR1B1/AKR1B10	12
BP	GO:0034101	erythrocyte homeostasis	11/418	137/18903	0.000236966193398834	0.00338769957839667	0.00246519099755181	LYN/HMOX1/VEGFA/B2M/CITED2/ISG15/PTBP3/ID2/ZFP36/HIF1A/MAFB	11
BP	GO:00	CD4-positive, alpha-beta T cell activation	10/418	115/18903	0.000237216492217 25	0.00338769957839667	0.00246519099755181	ANXA1/CTSL/BCL3/NFKBIZ/CD55/IL4R/RUNX1/ZC3H12A/RSAD2/LGALS7B	10

	35710								
BP	GO:0050818	regulation of coagulation	8/418	75/18903	0.000247212480605373	0.00351895289982241	0.00256070256783415	PLAUR/CEACAM1/PLAT/SERPINB2/PLAU/THBD/ANXA2/F3	8
BP	GO:0046916	cellular transition metal ion homeostasis	10/418	116/18903	0.000254578218848099	0.00361203511807206	0.00262843745434077	S100A8/S100A9/HEPHL1/STEAP4/LCN2/HMOX1/TFRC/HIF1A/PRNP/MT1X	10
BP	GO:0031668	cellular response to extracellular stimulus	16/418	262/18903	0.000255831579818396	0.00361807120972942	0.00263282985055825	AQP3/LYN/HMOX1/CYP24A1/GLUL/ZC3H12A/CDKN1A/FOSL1/CDKN2B/UPP1/XBP1/PIM1/IFI16/SH3GLB1/HSPA5/PMAIP1	16
BP	GO:1902105	regulation of leukocyte differentiation	18/418	317/18903	0.000263757210462312	0.00367800123374114	0.00267644025704733	CEACAM1/PRDM1/VNN1/LYN/ANXA1/NFKBIZ/IL4R/RUNX1/ZC3H12A/CEBPB/CD46/TRIB1/DUSP10/XBP1/HLA-B/ID2/IRF1/MAFB	18
BP	GO:0043405	regulation of MAP kinase activity	13/418	186/18903	0.000264242628542215	0.00367800123374114	0.00267644025704733	S100A12/LYN/IL1B/CD24/TGFA/SERPINB3/VEGFA/WNT5A/TRIB1/DUSP1/DUSP10/BMP2/TPD52L1	13
BP	GO:0035358	regulation of peroxisome proliferator activated receptor	4/418	15/18903	0.000265119082066009	0.00367800123374114	0.00267644025704733	ALOX15B/FABP5/CITED2/BMP2	4

		signaling pathway							
BP	GO:0071639	positive regulation of monocyte chemotactic protein-1 production	4/418	15/18903	0.000265119082066009	0.00367800123374114	0.00267644025704733	CLEC7A/IL1B/OAS1/SELENOK	4
BP	GO:2001028	positive regulation of endothelial cell chemotaxis	4/418	15/18903	0.000265119082066009	0.00367800123374114	0.00267644025704733	VEGFA/HSPB1/TMSB4X/LGMN	4
BP	GO:2001279	regulation of unsaturated fatty acid biosynthetic process	4/418	15/18903	0.000265119082066009	0.00367800123374114	0.00267644025704733	ANXA1/IL1B/FABP5/PTGS2	4
BP	GO:0006809	nitric oxide biosynthetic process	8/418	76/18903	0.000271048506021884	0.00374836066872036	0.00272764002895441	CLEC7A/IL1B/SOD2/CD47/ASS1/ZC3H12A/RAC1/PTGS2	8
BP	GO:0046632	alpha-beta T cell differentiation	10/418	117/18903	0.000272991516248925	0.00376332153314764	0.00273852688224473	PRDM1/ANXA1/CTSL/BCL3/NFKBIZ/IL4R/RUNX1/ZC3H12A/RSAD2/IRF1	10

BP	GO:0002221	pattern recognition receptor signaling pathway	13/418	187/18903	0.0002784121671671 64	0.00380817300801753	0.00277116479759628	TNIP3/LYN/CD14/OASL/TNFAIP3/RSAD2/LY96/S100A14/NFKBIA/CYLD/OAS1/RIOK3/IRF1	13
BP	GO:0042026	protein refolding	5/418	27/18903	0.000278859350701512	0.00380817300801753	0.00277116479759628	HSPB1/B2M/HSPA6/HSPA5/CRYAB	5
BP	GO:0071549	cellular response to dexamethasone stimulus	5/418	27/18903	0.000278859350701512	0.00380817300801753	0.00277116479759628	PLAT/GJB2/ASS1/FBXO32/ERRFI1	5
BP	GO:0071604	transforming growth factor beta production	6/418	42/18903	0.000301544619289254	0.00410514014421819	0.00298726445277205	CD24/CD46/CDH3/LUM/PTGS2/HIF1A	6
BP	GO:0051091	positive regulation of DNA-binding transcription factor activity	16/418	266/18903	0.000303114744047955	0.00411370009779368	0.00299349343500775	S100A8/S100A9/CRNN/S100A12/ADGRF1/CLEC7A/IL1B/CSF3/CIB1/VEGFA/WNT5A/FOSL1/TFRC/ANXA3/BMP2/MTPN	16
BP	GO:0045637	regulation of myeloid cell differentiation	14/418	214/18903	0.000306995822345012	0.00415347289055017	0.00302243565033317	CEACAM1/LYN/CSF3/CIB1/RUNX1/NFKBIA/B2M/CEBPB/TRIB1/ISG15/ID2/ZFP36/HIF1A/MAFB	14

BP	GO:0045604	regulation of epidermal cell differentiation	7/418	59/18903	0.000311314419576978	0.00418521689873334	0.00304553540914692	AQP3/ALOX15B/SULT2B1/GRHL1/ZFP36/ERRFI1/SFN	7
BP	GO:1903078	positive regulation of protein localization to plasma membrane	7/418	59/18903	0.000311314419576978	0.00418521689873334	0.00304553540914692	CIB1/EPHA2/PLS1/LGALS3/DLG1/EZR/PRNP	7
BP	GO:0032609	interferon-gamma production	10/418	119/18903	0.00031317298075 19	0.00418521689873334	0.00304553540914692	CD14/CLEC7A/IL1B/BCL3/CD47/WNT5A/ZC3H12A/ISG15/LGALS7B/PRNP	10
BP	GO:0032649	regulation of interferon-gamma production	10/418	119/18903	0.0003131729807519	0.00418521689873334	0.00304553540914692	CD14/CLEC7A/IL1B/BCL3/CD47/WNT5A/ZC3H12A/ISG15/LGALS7B/PRNP	10
BP	GO:0014074	response to purine-containing compound	11/418	142/18903	0.000323420423759179	0.00430898552386467	0.00313560044986033	PLAT/DUOX2/IL1B/ASS1/AREG/THBD/FOSL1/EZR/FOSB/PTGS2/HSPA5	11
BP	GO:0043433	negative regulation of DNA-binding transcription factor activity	13/418	190/18903	0.000324879717375004	0.00431527162592331	0.00314017477584351	HMOX1/BHLHE40/BCL3/TNFAIP3/ZC3H12A/NFKBIA/CYLD/TRIB1/ID2/ID1/PIM1/TMSB4X/PRNP	13

BP	GO:0031664	regulation of lipopolysaccharide-mediated signaling pathway	5/418	28/18903	0.000333367363591319	0.00441459205725474	0.0032124491400618	CD14/CD55/TNFAIP3/LY96/TRIB1	5
BP	GO:0002821	positive regulation of adaptive immune response	10/418	120/18903	0.000335045243143411	0.00442340698651573	0.00321886366524486	CLEC7A/IL1B/NFKBIZ/CD55/RSAD2/B2M/AKIRIN2/TFRC/HLA-B/HLA-E	10
BP	GO:0048872	homeostasis of number of cells	17/418	296/18903	0.000340273781803626	0.00447890489904171	0.0032592488739022	SPRR2A/CXCL6/LYN/ANXA1/HMOX1/VEGFA/TNFAIP3/B2M/CITED2/SKIL/ISG15/PTBP3/ID2/ZFP36/HIF1A/MAFB/PMAIP1	17
BP	GO:0010212	response to ionizing radiation	11/418	143/18903	0.000343563967266768	0.00450863224311043	0.00328088112885382	IL1A/ANXA1/SOD2/NET1/CDKN1A/THBD/MDM2/IFI16/GADD45A/HSPA5/CRYAB	11
BP	GO:0050921	positive regulation of chemotaxis	11/418	144/18903	0.000364752188454567	0.004772356477684	0.00347279029726204	S100A7/CXCL17/CXCL8/VEGFA/WNT5A/HSPB1/S100A14/F3/RAC1/TMSB4X/LGMN	11
BP	GO:0046394	carboxylic acid biosynthetic process	18/418	326/18903	0.000369460611543668	0.0048180807482016	0.00350606333621993	CEACAM1/RDH10/KYNU/ANXA1/DHRS9/IL1B/ALDH1A3/ALOX15B/OSBPL3/ASS1/FABP5/GLUL/CRABP2/LTA4H/XBP1/PNPLA8/RBP1/PTGS2	18

BP	GO:0072330	monocarboxylic acid biosynthetic process	14/418	218/18903	0.000370451975147766	0.0048180807482016	0.00350606333621993	CEACAM1/RDH10/ANXA1/DHRS9/IL1B/ALDH1A3/ALOX15B/OSBPL3/FABP5/CRABP2/XBP1/PNPLA8/RBP1/PTGS2	14
BP	GO:0002532	production of molecular mediator involved in inflammatory response	9/418	100/18903	0.000373941358998245	0.00484903186594164	0.00352858611755021	LYN/CLEC7A/SPINK7/GRN/IL4R/ZC3H12A/VAMP8/DUSP10/HIF1A	9
BP	GO:0071901	negative regulation of protein serine/threonine kinase activity	10/418	122/18903	0.000382625320097939	0.0049469604994911	0.00359984768612854	LYN/IL1B/SERPINB3/TNFAIP3/CDKN1A/DUSP1/CDKN2B/DUSP10/BMP2/GADD45A	10
BP	GO:1901184	regulation of ERBB signaling pathway	8/418	80/18903	0.000385941606390813	0.00497511746291401	0.00362033719269258	PLAUR/CEACAM1/GPRC5A/TGFA/HBEGF/AREG/LGMN/ERRFI1	8
BP	GO:0001837	epithelial to mesenchymal transition	12/418	169/18903	0.000391068958298661	0.00501164618112947	0.00364691873134822	LRG1/IL1B/SERPINB3/WNT5A/HAS2/ZNF750/ADIPOR1/EMP2/BMP2/S100A4/BAMBI/HIF1A	12
BP	GO:00	myeloid cell homeostasis	12/418	169/18903	0.000391068958298661	0.00501164618112947	0.00364691873134822	LYN/ANXA1/HMOX1/VEGFA/B2M/CITED2/ISG15/PTBP3/ID2/ZFP36/HIF1A/MAFB	12

	02262								
BP	GO:0010575	positive regulation of vascular endothelial growth factor production	5/418	29/18903	0.000395568586883162	0.00505448749906263	0.00367809387803642	IL1A/CXCL17/IL1B/PTGS2/HIF1A	5
BP	GO:0016053	organic acid biosynthetic process	18/418	328/18903	0.000397404709991947	0.00506314455587408	0.00368439352120814	CEACAM1/RDH10/KYNU/ANXA1/DHRS9/IL1B/ALDH1A3/ALOX15B/OSBPL3/ASS1/FABP5/GLUL/CRABP2/LTA4H/XBP1/PNPLA8/RBP1/PTGS2	18
BP	GO:1905477	positive regulation of protein localization to membrane	9/418	101/18903	0.000402659075697308	0.00511517488603848	0.00372225540906233	CIB1/EPHA2/PLS1/LGALS3/DLG1/STOM/EZR/MFF/PRNP	9
BP	GO:0002224	toll-like receptor signaling pathway	10/418	123/18903	0.000408447109621311	0.00515871060417668	0.00375393586299356	TNIP3/LYN/CD14/TNFAIP3/RSAD2/LY96/S100A14/NFKBIA/OAS1/IRF1	10
BP	GO:0022612	gland morphogenesis	10/418	123/18903	0.000408447109621311	0.00515871060417668	0.00375393586299356	CEACAM1/ELF3/TGFA/EPHA2/WNT5A/TNFAIP3/AREG/CEBPB/XBP1/SLC9A3R1	10

BP	GO:0001570	vasculogenesis	8/418	81/18903	0.000420113787039544	0.00529077017107437	0.00385003412906556	CEACAM1/EPHA2/TIPARP/VEGFA/HAS2/CITED2/ADM/EMP2	8
BP	GO:0033003	regulation of mast cell activation	6/418	45/18903	0.000442796707451059	0.00555687050241501	0.00404367235644845	LYN/ADGRE2/HMOX1/IL4R/PLSCR1/VAMP8	6
BP	GO:0019885	antigen processing and presentation of endogenous peptide antigen via MHC class I	4/418	17/18903	0.000446329873306103	0.00555687050241501	0.00404367235644845	B2M/HLA-C/HLA-B/HLA-E	4
BP	GO:0031649	heat generation	4/418	17/18903	0.000446329873306103	0.00555687050241501	0.00404367235644845	IL1A/IL1B/ADRB2/PTGS2	4
BP	GO:0090594	inflammatory response to wounding	4/418	17/18903	0.000446329873306103	0.00555687050241501	0.00404367235644845	IL1A/HMOX1/GRN/HIF1A	4
BP	GO:0046209	nitric oxide metabolic process	8/418	82/18903	0.000456703684283467	0.00566987244408736	0.00412590260233359	CLEC7A/IL1B/SOD2/CD47/ASS1/ZC3H12A/RAC1/PTGS2	8

BP	GO:0051047	positive regulation of secretion	17/418	304/18903	0.0004619223922307 76	0.00571841601713453	0.00416122721612993	S100A8/IL1A/DOC2B/IL1B/CD177/IL4R/GLUL/RAB27B/VAMP8/ANXA2/S100A10/SDC4/BMP2/XBP1/EZR/HIF1A/VPS4B	17
BP	GO:0007176	regulation of epidermal growth factor-activated receptor activity	5/418	30/18903	0.000466143148105015	0.00573815649920822	0.00417559214358859	GPRC5A/TGFA/HBEGF/AREG/ERRFI1	5
BP	GO:0070293	renal absorption	5/418	30/18903	0.000466143148105015	0.00573815649920822	0.00417559214358859	AQP3/SLC5A1/CLDN4/HAS2/SLC9A3R1	5
BP	GO:2001244	positive regulation of intrinsic apoptotic signaling pathway	7/418	63/18903	0.00046809908934432	0.00574604781020977	0.00418133456212061	S100A8/S100A9/VNN1/IL19/SKIL/SLC9A3R1/PMAIP1	7
BP	GO:2001057	reactive nitrogen species metabolic process	8/418	83/18903	0.000495837163587751	0.0060694913301918	0.0044167007848993	CLEC7A/IL1B/SOD2/CD47/ASS1/ZC3H12A/RAC1/PTGS2	8

BP	GO:0002920	regulation of humoral immune response	6/418	46/18903	0.00049984117372698	0.00610141321001928	0.00443992997891563	CFH/IL1B/CD55/CD59/CD46/KLK7	6
BP	GO:0010950	positive regulation of endopeptidase activity	12/418	174/18903	0.000508756673943542	0.00619294335691721	0.00450653544050268	S100A8/S100A9/LYN/CLEC7A/SERPINB3/TNFSF10/CTSD/AKIRIN2/F3/LGMN/IFI16/PMAIP1	12
BP	GO:0048002	antigen processing and presentation of peptide antigen	7/418	64/18903	0.000515746369495902	0.00622946501035702	0.0045331118382003	CTSL/CTSD/B2M/HLA-C/HLA-B/HLA-E/LGMN	7
BP	GO:0071384	cellular response to corticosteroid stimulus	7/418	64/18903	0.000515746369495902	0.00622946501035702	0.0045331118382003	PLAT/GJB2/ANXA1/ASS1/FBXO32/ZFP36/ERRFI1	7
BP	GO:0043535	regulation of blood vessel endothelial cell migration	11/418	150/18903	0.000516284972121754	0.00622946501035702	0.0045331118382003	ANXA1/HMOX1/CIB1/EPHA2/VEGFA/HSPB1/PLK2/TMSB4X/PTGS2/HIF1A/GADD45A	11
BP	GO:0048762	mesenchymal cell differentiation	15/418	252/18903	0.000517458992851167	0.00622946501035702	0.0045331118382003	RDH10/LRG1/IL1B/SERPINB3/PITX2/WNT5A/HAS2/CITED2/ZNF750/ADIPOR1/EMP2/BMP2/S100A4/BAMBI/HIF1A	15

BP	GO:0002688	regulation of leukocyte chemotaxis	10/418	127/18903	0.0005266879708588845	0.00632314954025591	0.00460128502014046	S100A7/LYN/CXCL17/CXCL8/VEGFA/WNT5A/S100A14/DUSP1/RAC1/LGMN	10
BP	GO:0032760	positive regulation of tumor necrosis factor production	9/418	105/18903	0.0005363062012281117	0.00642098109415582	0.00467247594494634	IL1A/CD14/CLEC7A/WNT5A/LY96/HSPB1/OAS1/HLA-E/SELENOK	9
BP	GO:1900745	positive regulation of p38MAPK cascade	5/418	31/18903	0.00054579334436557	0.00651671288217907	0.00474213889366807	IL1B/VEGFA/ZC3H12A/BMP2/GADD45A	5
BP	GO:0051960	regulation of nervous system development	22/418	456/18903	0.00055892594362134	0.00658876664170989	0.00479457160655319	LYN/IL1B/MME/TYMP/BHLHE40/VEGFA/DLG1/SPINT1/WNT5A/CTSC/CRABP2/TNFRSF21/B2M/ANXA2/SKIL/CDKN2B/S100A10/DUSP10/BMP2/ID2/ID1/HIF1A	22
BP	GO:0030218	erythrocyte differentiation	10/418	128/18903	0.00056030566875973	0.00658876664170989	0.00479457160655319	LYN/VEGFA/B2M/CITED2/ISG15/PTBP3/ID2/ZFP36/HIF1A/MAFB	10
BP	GO:0045646	regulation of erythrocyte differentiation	6/418	47/18903	0.000562443944388754	0.00658876664170989	0.00479457160655319	LYN/B2M/ISG15/ZFP36/HIF1A/MAFB	6

BP	GO:1901136	carbohydrate derivative catabolic process	12/418	176/18903	0.000563532822027221	0.00658876664170989	0.00479457160655319	APOBEC3A/NCCRP1/CTSL/PGLYRP4/TYMP/CHI3L2/FUCA1/CDA/MMP12/GM2A/UPP1/CST3	12
BP	GO:0006977	DNA damage response, signal transduction by p53 class mediator resulting in cell cycle arrest	4/418	18/18903	0.000563889868192105	0.00658876664170989	0.00479457160655319	MUC1/CDKN1A/MDM2/PLK2	4
BP	GO:0016264	gap junction assembly	4/418	18/18903	0.000563889868192105	0.00658876664170989	0.00479457160655319	GJB2/IL1B/HOPX/GJB6	4
BP	GO:0051917	regulation of fibrinolysis	4/418	18/18903	0.000563889868192105	0.00658876664170989	0.00479457160655319	PLAUR/PLAT/PLAU/THBD	4
BP	GO:0060263	regulation of respiratory burst	4/418	18/18903	0.000563889868192105	0.00658876664170989	0.00479457160655319	CLEC7A/GRN/DUSP10/RAC1	4

BP	GO:0042102	positive regulation of T cell proliferation	9/418	106/18903	0.000574853275097959	0.00669895683247489	0.0048747557728307	IL1A/ANXA1/IL1B/CD24/CD55/CD46/TFRC/HLA-E/SELENOK	9
BP	GO:0002253	activation of immune response	20/418	397/18903	0.000577493817753609	0.00671182974357253	0.00488412324589488	CEACAM1/CFH/LYN/CLEC7A/IL1B/NFKBIZ/LGALS3/CD47/CD55/ZC3H12A/CD59/PLSCR1/TNFRSF21/CD46/IGHG4/IGKC/EZR/IGHG3/IFI16/PRNP	20
BP	GO:0034605	cellular response to heat	7/418	66/18903	0.000622598856076384	0.00718550831520451	0.00522881383120145	IL1A/LYN/SLC52A3/HMOX1/HSPA6/PTGS2/DNAJB6	7
BP	GO:0045682	regulation of epidermis development	7/418	66/18903	0.000622598856076384	0.00718550831520451	0.00522881383120145	AQP3/ALOX15B/SULT2B1/GRHL1/ZFP36/ERRFI1/SFN	7
BP	GO:0001659	temperature homeostasis	12/418	178/18903	0.00062318252893879	0.00718550831520451	0.00522881383120145	IL1A/LCN2/IL1B/VEGFA/IL4R/FABP5/FABP4/CEBPB/ADIPOR1/ADRB2/ID1/PTGS2	12
BP	GO:0042542	response to hydrogen peroxide	10/418	130/18903	0.000632881158089628	0.00721041200982145	0.00524693597053369	ANXA1/SOD2/HMOX1/TNFAIP3/AREG/NET1/CAPN2/FOSL1/CRYAB/SETX	10
BP	GO:00	receptor-mediated endocytosis	15/418	257/18903	0.000634270489349077	0.00721041200982145	0.00524693597053369	CEACAM1/RAB31/CD14/CXCL8/CTSL/VEGFA/B2M/TFRC/ANXA2/ADM/ADRB2/ITSN2/RAC1/EZR/CLTB	15

	06898								
BP	GO:0001516	prostaglandin biosynthetic process	5/418	32/18903	0.000635242248004865	0.00721041200982145	0.00524693597053369	ANXA1/IL1B/FABP5/PNPLA8/PTGS2	5
BP	GO:0035456	response to interferon-beta	5/418	32/18903	0.000635242248004865	0.00721041200982145	0.00524693597053369	PLSCR1/CAPN2/OAS1/IFI16/IRF1	5
BP	GO:0043304	regulation of mast cell degranulation	5/418	32/18903	0.000635242248004865	0.00721041200982145	0.00524693597053369	LYN/ADGRE2/HMOX1/IL4R/VAMP8	5
BP	GO:0046457	prostanoid biosynthetic process	5/418	32/18903	0.000635242248004865	0.00721041200982145	0.00524693597053369	ANXA1/IL1B/FABP5/PNPLA8/PTGS2	5
BP	GO:0000302	response to reactive oxygen species	13/418	204/18903	0.000639078544335537	0.00723516383094895	0.00526494759323059	S100A7/IL1A/ANXA1/SOD2/HMOX1/TNFAIP3/AREG/NET1/CAPN2/FOSL1/HIF1A/CRYAB/SETX	13
BP	GO:0072659	protein localization to plasma membrane	16/418	285/18903	0.000645215368831827	0.00728576527595629	0.00530176969737781	VAMP5/RAB31/CIB1/EPHA2/PLS1/LGALS3/DLG1/VAMP8/AFDN/S100A10/EMP2/RAB10/EZR/SLC9A3R1/ATP1B1/PRNP	16

BP	GO:0031669	cellular response to nutrient levels	14/418	231/18903	0.000659786371805476	0.00743109908451013	0.00540752748026137	AQP3/HMOX1/CYP24A1/GLUL/ZC3H12A/CDKN1A/CDKN2B/UPP1/XBP1/PIM1/IFI16/SH3GLB1/HSPA5/PMAIP1	14
BP	GO:0051098	regulation of binding	19/418	372/18903	0.000667458203561085	0.00749818084720294	0.00545634212679756	PLAUR/SLPI/CSF3/HMOX1/BCL3/WNT5A/CDKN1A/NFKBIA/B2M/ANXA2/ADRB2/S100A10/PHLDA2/BMP2/BAMBI/ID1/HOPX/TMSB4X/IFI16	19
BP	GO:0070613	regulation of protein processing	7/418	67/18903	0.000682234189977392	0.00762497035857085	0.0055486054325527	PLAUR/PLAT/RUNX1/PLAU/ANXA2/S100A10/PRNP	7
BP	GO:1904377	positive regulation of protein localization to cell periphery	7/418	67/18903	0.000682234189977392	0.00762497035857085	0.0055486054325527	CIB1/EPHA2/PLS1/LGALS3/DLG1/EZR/PRNP	7
BP	GO:0002483	antigen processing and presentation of endogenous peptide antigen	4/418	19/18903	0.000701869259171572	0.00778469203700449	0.00566483310701928	B2M/HLA-C/HLA-B/HLA-E	4
BP	GO:0002544	chronic inflammatory response	4/418	19/18903	0.000701869259171572	0.00778469203700449	0.00566483310701928	S100A8/S100A9/VNN1/TNFAIP3	4

BP	GO:0045953	negative regulation of natural killer cell mediated cytotoxicity	4/418	19/18903	0.000701869259171572	0.00778469203700449	0.00566483310701928	SERPINB4/CEACAM1/HLA-B/HLA-E	4
BP	GO:1903557	positive regulation of tumor necrosis factor superfamily cytokine production	9/418	109/18903	0.000704322658661126	0.00779212662873195	0.00567024317605666	IL1A/CD14/CLEC7A/WNT5A/LY96/HSPB1/OAS1/HLA-E/SELENOK	9
BP	GO:0043367	CD4-positive, alpha-beta T cell differentiation	8/418	88/18903	0.000734336084363419	0.00808324606717416	0.00588208752714275	ANXA1/CTSL/BCL3/NFKBIZ/IL4R/RUNX1/ZC3H12A/RSAD2	8
BP	GO:0055072	iron ion homeostasis	8/418	88/18903	0.000734336084363419	0.00808324606717416	0.00588208752714275	HEPHL1/STEAP4/LCN2/SOD2/HMOX1/B2M/TFRC/HIF1A	8
BP	GO:0002704	negative regulation of leukocyte mediated immunity	7/418	68/18903	0.000746306065612683	0.00819436559479253	0.00596294796142797	SERPINB4/CEACAM1/HMOX1/CD55/CD46/HLA-B/HLA-E	7

BP	GO:0072593	reactive oxygen species metabolic process	14/418	235/18903	0.0007804491314133378	0.00854777620119414	0.00622012089697 88	DUOX2/CLEC7A/SOD2/CD177/GPX3/IL19/ZC3H12A/CDKN1A/RAC1/HIF1A/ARF4/GADD45A/PMAIP1/CRYAB	14
BP	GO:1902041	regulation of extrinsic apoptotic signaling pathway via death domain receptors	6/418	50/18903	0.0007872930288752 86	0.0086011763404625	0.006258979579558 52	HMOX1/MAL/LGALS3/TNFAIP3/SKIL/PMAIP1	6
BP	GO:0006022	aminoglycan metabolic process	10/418	134/18903	0.0008014133096193 43	0.0087308981757554	0.006353376704554 28	HS3ST1/IL1B/PGLYRP4/ST3GAL1/CHI3L2/FUCA1/DSE/HAS2/GALNT5/DCN	10
BP	GO:0071347	cellular response to interleukin-1	9/418	111/18903	0.000803162715481 39	0.0087308981757554	0.006353376704554 28	CXCL8/IL1B/IL1RN/CCL20/CD47/ZC3H12A/HAS2/CEBPB/HIF1A	9
BP	GO:0032743	positive regulation of interleukin-2 production	5/418	34/18903	0.0008465237399319 43	0.00915670481064998	0.006663231418276 18	IL1A/ANXA1/CLEC7A/IL1B/RUNX1	5
BP	GO:00	regulation of mast cell activation	5/418	34/18903	0.0008465237399319 43	0.00915670481064998	0.006663231418276 18	LYN/ADGRE2/HMOX1/IL4R/VAMP8	5

	33006	involved in immune response							
BP	GO:0043254	regulation of protein-containing complex assembly	20/418	410/18903	0.000860274021869412	0.00923406318377728	0.00671952423899582	CLEC7A/CSF3/PLEK/LGALS3/CDC42EP1/VEGFA/DLG1/CLDN7/TFRC/RAC1/MTPN/CLIP1/ISG15/TMSB4X/SH3GLB1/ARPC3/RIOK3/HSPA5/TMSB10/CRYAB	20
BP	GO:0002716	negative regulation of natural killer cell mediated immunity	4/418	20/18903	0.000862127638210784	0.00923406318377728	0.00671952423899582	SERPINB4/CEACAM1/HLA-B/HLA-E	4
BP	GO:0007171	activation of transmembrane receptor protein tyrosine kinase activity	4/418	20/18903	0.000862127638210784	0.00923406318377728	0.00671952423899582	TGFA/HBEGF/AREG/ADRB2	4
BP	GO:0051546	keratinocyte migration	4/418	20/18903	0.000862127638210784	0.00923406318377728	0.00671952423899582	LRG1/KRT16/HBEGF/HAS2	4
BP	GO:0009612	response to mechanical stimulus	13/418	211/18903	0.000874345831172689	0.00929020493397849	0.00676037796111102	IL1B/CAPN2/NFKBIA/FOSL1/CITED2/MTPN/SCEL/FOSB/PTGS2/PPL/GADD45A/IRF1/KRT5	13

BP	GO:0001913	T cell mediated cytotoxicity	6/418	51/18903	0.000875872867917422	0.00929020493397849	0.00676037796111102	CEACAM1/CTSC/B2M/EMP2/HLA-B/HLA-E	6
BP	GO:0032873	negative regulation of stress-activated MAPK cascade	6/418	51/18903	0.000875872867917422	0.00929020493397849	0.00676037796111102	SERPINB3/DLG1/CYLD/DUSP1/DUSP10/EZR	6
BP	GO:0070303	negative regulation of stress-activated protein kinase signaling cascade	6/418	51/18903	0.000875872867917422	0.00929020493397849	0.00676037796111102	SERPINB3/DLG1/CYLD/DUSP1/DUSP10/EZR	6
BP	GO:0002700	regulation of production of molecular mediator of immune response	12/418	187/18903	0.000961293586862973	0.0101653350578176	0.00739720033955609	CLEC7A/IL1B/HMOX1/CD55/WNT5A/IL4R/RSAD2/B2M/TFRC/XBP1/HLA-E/KLK7	12
BP	GO:0001893	maternal placenta development	5/418	35/18903	0.000969893354879253	0.0101653350578176	0.00739720033955609	PRDM1/GJB2/CITED2/CTSB/PTGS2	5

BP	GO:2000515	negative regulation of CD4-positive, alpha-beta T cell activation	5/418	35/18903	0.000969893354879253	0.0101653350578176	0.007397200339556 09	ANXA1/IL4R/RUNX1/ZC3H12A/LGALS7B	5
BP	GO:0006636	unsaturated fatty acid biosynthetic process	6/418	52/18903	0.000971934667183085	0.0101653350578176	0.00739720033955609	ANXA1/IL1B/ALOX15B/FABP5/PNPLA8/PTGS2	6
BP	GO:0043303	mast cell degranulation	6/418	52/18903	0.000971934667183085	0.0101653350578176	0.00739720033955609	LYN/ADGRE2/HMOX1/IL4R/S100A13/VAMP8	6
BP	GO:0002824	positive regulation of adaptive immune response based on somatic recombination of immune receptors built from immunoglobulin superfamily domains	9/418	114/18903	0.000972336396834731	0.0101653350578176	0.00739720033955609	CLEC7A/IL1B/NFKBIZ/CD55/RSAD2/B2M/TFRC/HLA-B/HLA-E	9

BP	GO:0030879	mammary gland development	10/418	138/18903	0.00100523253721472	0.0104841675122395	0.00762921114162963	ELF3/TGFA/EPHA2/VEGFA/WNT5A/AREG/CEBPB/XBP1/ID2/HIF1A	10
BP	GO:1990748	cellular detoxification	9/418	115/18903	0.00103475223779282	0.0107591915061063	0.00782934301817344	S100A9/DUOX2/SOD2/GPX3/TXNDC17/TXNRD1/PIM1/PTGS2/AKR1B10	9
BP	GO:0045055	regulated exocytosis	14/418	242/18903	0.00103652622976447	0.0107591915061063	0.00782934301817344	CEACAM1/LYN/RAB31/DOC2B/ADGRE2/HMOX1/PLEK/CD177/RAB11FIP1/IL4R/S100A13/VAMP8/ANXA3/RAB10	14
BP	GO:0071605	monocyte chemotactic protein-1 production	4/418	21/18903	0.00104653328178532	0.0107608245680043	0.00783053137900543	CLEC7A/IL1B/OAS1/SELENOK	4
BP	GO:0071637	regulation of monocyte chemotactic protein-1 production	4/418	21/18903	0.00104653328178532	0.0107608245680043	0.00783053137900543	CLEC7A/IL1B/OAS1/SELENOK	4
BP	GO:0090026	positive regulation of monocyte chemotaxis	4/418	21/18903	0.00104653328178532	0.0107608245680043	0.00783053137900543	S100A7/CXCL17/S100A14/LGMN	4

BP	GO:1901522	positive regulation of transcription from RNA polymerase II promoter involved in cellular response to chemical stimulus	4/418	21/18903	0.00104653328178532	0.0107608245680043	0.00783053137900543	VEGFA/BMP2/XBP1/HIF1A	4
BP	GO:1903317	regulation of protein maturation	7/418	72/18903	0.0010516598646608	0.0107881540107223	0.00785041870803133	PLAUR/PLAT/RUNX1/PLAU/ANXA2/S100A10/PRNP	7
BP	GO:0043123	positive regulation of I-kappaB kinase/NF-kappaB signaling	12/418	189/18903	0.00105417498455108	0.0107886292329935	0.00785076452194951	ECM1/S100A12/IL1A/CLEC7A/IL1B/HMOX1/TNFSF10/S100A13/TFRC/S100A4/PLK2/SHISA5	12
BP	GO:0046635	positive regulation of alpha-beta T cell activation	7/418	73/18903	0.00114149188739074	0.0116346043090763	0.00846637109905324	ANXA1/NFKBIZ/CD55/IL4R/RUNX1/HSPH1/HLA-E	7

BP	GO:0001765	membrane raft assembly	3/418	10/18903	0.00114748614581508	0.0116346043090763	0.00846637109905324	ANXA2/S100A10/EMP2	3
BP	GO:0006222	UMP biosynthetic process	3/418	10/18903	0.00114748614581508	0.0116346043090763	0.00846637109905324	CDA/CMPK1/UPP1	3
BP	GO:0060368	regulation of Fc receptor mediated stimulatory signaling pathway	3/418	10/18903	0.00114748614581508	0.0116346043090763	0.00846637109905324	LYN/CD47/PLSCR1	3
BP	GO:0071887	leukocyte apoptotic process	9/418	117/18903	0.00116932292114308	0.011828567512489	0.00860751594730322	LYN/ANXA1/CTSL/ST3GAL1/BCL3/LGALS3/WNT5A/TNFRSF21/HIF1A	9
BP	GO:0042180	cellular ketone metabolic process	13/418	218/18903	0.00117835046180205	0.0118923591641454	0.00865393641692964	FMO2/CEACAM1/RDH10/KYNU/ANXA1/DHRS9/IL1B/FABP5/ADM/BMP2/PTGS2/AKR1B1/AKR1B10	13
BP	GO:0070555	response to interleukin-1	10/418	141/18903	0.00118444843063671	0.0119061687988311	0.0086639855332455	CXCL8/ANXA1/IL1B/IL1RN/CCL20/CD47/ZC3H12A/HAS2/CEBPB/HIF1A	10

BP	GO:0043434	response to peptide hormone	20/418	421/18903	0.00118572323069572	0.0119061687988311	0.0086639855332455	CEACAM1/LYN/PLAT/RAB31/GJB2/ANXA1/IL1B/TNFSF10/ASS1/AREG/SORT1/CA2/ADIPOR1/AGTRAP/ADM/LTA4H/XBP1/RAB10/PTGS2/ERRFI1	20
BP	GO:0002279	mast cell activation involved in immune response	6/418	54/18903	0.00118817138028594	0.0119061687988311	0.0086639855332455	LYN/ADGRE2/HMOX1/IL4R/S100A13/VAMP8	6
BP	GO:0009314	response to radiation	21/418	452/18903	0.00119061687988311	0.0119061687988311	0.0086639855332455	IL1A/PRDM1/ANXA1/SOD2/MME/BHLHE40/BCL3/NET1/CDKN1A/IVL/THBD/N4BP1/MDM2/ID2/PTGS2/HIF1A/IFI16/GADD45A/SERPINB13/HSPA5/CRYAB	21
BP	GO:0007009	plasma membrane organization	11/418	166/18903	0.00119438486213512	0.0119165795605719	0.00867156132783033	S100A9/UGCG/PLSCR1/ANXA2/S100A10/EMP2/NDRG1/SLC9A3R1/SH3GLB1/MAFB/VPS4B	11
BP	GO:0031099	regeneration	12/418	192/18903	0.00120739569908661	0.0119916345568375	0.00872617800703506	CLDN1/ANXA1/HMOX1/GRN/RUNX1/CDKN1A/CEBPB/ADM/ANXA3/DUSP10/MTPN/HOPX	12
BP	GO:0031334	positive regulation of protein-containing complex assembly	12/418	192/18903	0.00120739569908661	0.0119916345568375	0.00872617800703506	CLEC7A/CSF3/PLEK/LGALS3/CDC42EP1/VEGFA/DLG1/TFRC/RAC1/CLIP1/ISG15/SH3GLB1	12

BP	GO:0030330	DNA damage response, signal transduction by p53 class mediator	7/418	74/18903	0.00123723222707288	0.0122323638740011	0.00890135403188183	MUC1/BCL3/CDKN1A/NDRG1/MDM2/PLK2/PMAIP1	7
BP	GO:0042058	regulation of epidermal growth factor receptor signaling pathway	7/418	74/18903	0.00123723222707288	0.0122323638740011	0.00890135403188183	PLAUR/CEACAM1/GPRC5A/TGFA/HBEGF/AREG/ERRFI1	7
BP	GO:0006821	chloride transport	9/418	118/18903	0.00124172064658651	0.0122490275972529	0.00891348003644494	CLCA4/SLC6A14/CLIC3/CLDN4/SLC12A8/CLCA2/CA2/FXYD3/PRNP	9
BP	GO:0070665	positive regulation of leukocyte proliferation	11/418	167/18903	0.00125385990322478	0.0122883629448908	0.00894210392786103	IL1A/LYN/ANXA1/IL1B/CD24/CD55/CDKN1A/CD46/TFRC/HLA-E/SELENOK	11
BP	GO:0009129	pyrimidine nucleoside monophosphate metabolic process	4/418	22/18903	0.0012569561181616	0.0122883629448908	0.00894210392786103	TYMP/CDA/CMPK1/UPP1	4

BP	GO:0010042	response to manganese ion	4/418	22/18903	0.00125695611816 16	0.01228836294 48908	0.00894210392786 103	SOD2/TFRC/PTGS2/HSPA5	4
BP	GO:0043371	negative regulation of CD4-positive, alpha-beta T cell differentiation	4/418	22/18903	0.00125695611816 16	0.01228836294 48908	0.00894210392786 103	ANXA1/IL4R/RUNX1/ZC3H12A	4
BP	GO:0061097	regulation of protein tyrosine kinase activity	8/418	96/18903	0.00130036677234 085	0.01265613094 68363	0.00920972457916 236	GPRC5A/CD24/TGFA/HBEGF/AREG/RAP2B/ERRFI1/PRNP	8
BP	GO:0070664	negative regulation of leukocyte proliferation	8/418	96/18903	0.00130036677234 085	0.01265613094 68363	0.00920972457916 236	LYN/DLG1/TNFAIP3/TNFRSF21/CEBPB/SDC4/LGALS7B/PRNP	8
BP	GO:0002448	mast cell mediated immunity	6/418	55/18903	0.00130920217439 591	0.01268561752 13084	0.00923118162877 823	LYN/ADGRE2/HMOX1/IL4R/S100A13/VAMP8	6
BP	GO:0070169	positive regulation of biomineral tissue development	6/418	55/18903	0.00130920217439 591	0.01268561752 13084	0.00923118162877 823	ODAPH/FAM20C/CEBPB/ADRB2/BMP2/ISG15	6

BP	GO:0071479	cellular response to ionizing radiation	7/418	75/18903	0.00133914602450336	0.0129470533784949	0.00942142557062097	NET1/CDKN1A/MDM2/IFI16/GADD45A/HSPA5/CRYAB	7
BP	GO:0002690	positive regulation of leukocyte chemotaxis	8/418	97/18903	0.00139049120515477	0.0134137893300802	0.00976106408916591	S100A7/CXCL17/CXCL8/VEGFA/WNT5A/S100A14/RAC1/LGMN	8
BP	GO:1903532	positive regulation of secretion by cell	15/418	278/18903	0.00140102648199256	0.0134856513795319	0.00981335729677607	IL1A/DOC2B/IL1B/CD177/IL4R/GLUL/RAB27B/VAMP8/ANXA2/S100A10/SDC4/BMP2/EZR/HIF1A/VPS4B	15
BP	GO:0010720	positive regulation of cell development	16/418	307/18903	0.00141060321766709	0.0135341349100097	0.00984863821826789	LYN/CLEC7A/IL1B/MME/CIB1/VEGFA/SPINT1/HAS2/CRABP2/TRIB1/SKIL/S100A10/RAC1/BMP2/ID2/HIF1A	16
BP	GO:0009636	response to toxic substance	14/418	250/18903	0.00141225755582709	0.0135341349100097	0.00984863821826789	S100A9/CLDN1/LYN/SLC6A14/DUOX2/SOD2/GPX3/ASS1/TXNDC17/TXNRD1/PIM1/PTGS2/AKR1B10/MT1X	14
BP	GO:0030851	granulocyte differentiation	5/418	38/18903	0.001420453093138	0.0135532314781944	0.00986253457680969	CEACAM1/CSF3/RUNX1/TRIB1/CITED2	5
BP	GO:00	type 2 immune response	5/418	38/18903	0.001420453093138	0.0135532314781944	0.00986253457680969	ECM1/ANXA1/BCL3/IL4R/RSAD2	5

	42092								
BP	GO:0001836	release of cytochrome c from mitochondria	6/418	56/18903	0.00143942586349991	0.013594937824692	0.00989288381751046	PLAUR/SOD2/TNFSF10/MFF/PMAIP1/SFN	6
BP	GO:0002707	negative regulation of lymphocyte mediated immunity	6/418	56/18903	0.00143942586349991	0.013594937824692	0.00989288381751046	SERPINB4/CEACAM1/CD55/CD46/HLA-B/HLA-E	6
BP	GO:0010718	positive regulation of epithelial to mesenchymal transition	6/418	56/18903	0.00143942586349991	0.013594937824692	0.00989288381751046	LRG1/IL1B/SERPINB3/EMP2/BMP2/BAMBI	6
BP	GO:0110151	positive regulation of biomineralization	6/418	56/18903	0.00143942586349991	0.013594937824692	0.00989288381751046	ODAPH/FAM20C/CEBPB/ADRB2/BMP2/ISG15	6
BP	GO:1990778	protein localization to cell periphery	17/418	337/18903	0.00144037899607149	0.013594937824692	0.00989288381751046	VAMP5/RAB31/CIB1/EPHA2/PLS1/MAL/LGALS3/DLG1/VAMP8/AFDN/S100A10/EMP2/RAB10/EZR/SLC9A3R1/ATP1B1/PRNP	17
BP	GO:01	positive regulation of	8/418	98/18903	0.0014855076416386	0.0139906646421567	0.010180849785368	LCN2/VEGFA/IL4R/FABP5/FABP4/CEBPB/ADIPOR1/ADRB2	8

	20162	cold-induced thermogenesis							
BP	GO:0016338	calcium-independent cell-cell adhesion via plasma membrane cell-adhesion molecules	4/418	23/18903	0.00149526120187409	0.0140522396821285	0.010225657251526	CLDN1/CLDN4/CLDN7/BMP2	4
BP	GO:0048545	response to steroid hormone	17/418	339/18903	0.00153420812795013	0.0143873165646825	0.0104694889418056	CLDN1/PLAT/GJB2/ANXA1/IL1RN/PAPPA/CLDN4/ASS1/AREG/FBXO32/FOSL1/ADM/ANXA3/ZFP36/FOSB/PTGS2/ERRFI1	17
BP	GO:0050671	positive regulation of lymphocyte proliferation	10/418	146/18903	0.00154055039690646	0.0143997317045572	0.0104785232998837	IL1A/ANXA1/IL1B/CD24/CD55/CDKN1A/CD46/TFRC/HLA-E/SELENOK	10
BP	GO:0001660	fever generation	3/418	11/18903	0.00155200769630353	0.0143997317045572	0.0104785232998837	IL1A/IL1B/PTGS2	3
BP	GO:0009174	pyrimidine ribonucleoside monophosphate biosynthetic process	3/418	11/18903	0.00155200769630353	0.0143997317045572	0.0104785232998837	CDA/CMPK1/UPP1	3

BP	GO:0061418	regulation of transcription from RNA polymerase II promoter in response to hypoxia	3/418	11/18903	0.00155200769630353	0.0143997317045572	0.0104785232998837	VEGFA/CITED2/HIF1A	3
BP	GO:0097278	complement-dependent cytotoxicity	3/418	11/18903	0.00155200769630353	0.0143997317045572	0.0104785232998837	CFH/CD55/CD59	3
BP	GO:0042063	gliogenesis	16/418	310/18903	0.00155854974481126	0.014429793188189	0.0105003987044487	S100A8/S100A9/GPR157/LYN/ANXA1/IL1B/MAL/SPINT1/GRN/AREG/TNFRSF21/CDKN2B/DUSP10/NDRG1/BMP2/ID2	16
BP	GO:0043297	apical junction assembly	7/418	77/18903	0.001562581024581	0.0144365308190676	0.0105053016028913	CLDN1/CLDN4/EPHA2/DLG1/CLDN7/AFDN/PKN2	7
BP	GO:0071248	cellular response to metal ion	12/418	198/18903	0.00156946520537931	0.0144695420833494	0.0105293235297599	CLDN1/HMOX1/CPNE8/WNT5A/B2M/FABP4/FOSB/PTGS2/LGMN/HSPA5/PRNP/MT1X	12
BP	GO:0010332	response to gamma radiation	6/418	57/18903	0.00157929076911595	0.0145263753005056	0.0105706804246242	IL1A/SOD2/CDKN1A/MDM2/HSPA5/CRYAB	6

BP	GO:0070371	ERK1 and ERK2 cascade	17/418	340/18903	0.00158302511913571	0.0145263753005056	0.0105706804246242	S100A7/IL1A/CEACAM1/LYN/CXCL17/IL1B/CIB1/EPHA2/CCL20/DLG1/DUSP5/PSCA/DUSP10/BMP2/EZR/SLC9A3R1/ERRFI1	17
BP	GO:0032755	positive regulation of interleukin-6 production	8/418	99/18903	0.00158560206369363	0.0145263753005056	0.0105706804246242	IL1A/IL36A/CLEC7A/IL1B/WNT5A/AKIRIN2/XBP1/SELENOK	8
BP	GO:0045616	regulation of keratinocyte differentiation	5/418	39/18903	0.00160017837889541	0.0146292458488973	0.0106455381692205	AQP3/ALOX15B/GRHL1/ZFP36/ERRFI1	5
BP	GO:0006869	lipid transport	20/418	433/18903	0.00165575500308491	0.0150807250160179	0.0109740744967819	IL1A/CEACAM1/ANXA1/IL1B/OSBPL3/UGCG/FABP5/PLSCR1/CRABP2/NFKBIA/FABP4/ANXA2/GM2A/LRP10/PNPLA8/GLTP/RBP1/PTGS2/VPS4B/NPC2	20
BP	GO:0030203	glycosaminoglycan metabolic process	9/418	123/18903	0.00165991504180884	0.0150807250160179	0.0109740744967819	HS3ST1/IL1B/PGLYRP4/ST3GAL1/FUCA1/DSE/HAS2/GALNT5/DCN	9
BP	GO:0097237	cellular response to toxic substance	9/418	123/18903	0.00165991504180884	0.0150807250160179	0.0109740744967819	S100A9/DUOX2/SOD2/GPX3/TXNDC17/TXNRD1/PIM1/PTGS2/AKR1B10	9
BP	GO:00	regulation of cell	16/418	312/18903	0.00166426039469849	0.0150888338689469	0.0109799752181353	TACSTD2/ANXA1/CIB1/CLDN4/CDC42EP1/VEGFA/DLG1/WNT5A/HAS2/ITSN2/S100A10/RAC1/RND3/BAMBI/EZR/SLC9A3R1	16

	22604	morphogenesis							
BP	GO:0043154	negative regulation of cysteine-type endopeptidase activity involved in apoptotic process	7/418	78/18903	0.00168465852731016	0.015179294359475	0.01104580230277 59	PLAUR/VEGFA/PTGS2/TNFAIP8/DNAJB6/SFN/CRYAB	7
BP	GO:0061045	negative regulation of wound healing	7/418	78/18903	0.00168465852731016	0.015179294359475	0.0110458023027759	PLAUR/CEACAM1/PLAT/SERPINB2/PLAU/THBD/ANXA2	7
BP	GO:0070373	negative regulation of ERK1 and ERK2 cascade	7/418	78/18903	0.00168465852731016	0.015179294359475	0.0110458023027759	LYN/DLG1/PSCA/DUSP10/EZR/SLC9A3R1/ERRFI1	7
BP	GO:0010001	glial cell differentiation	13/418	227/18903	0.00169423309740308	0.0152341535712993	0.0110857227360943	S100A8/S100A9/GPR157/LYN/IL1B/MAL/SPINT1/GRN/TNFRSF21/DUSP10/NDRG1/BMP2/ID2	13
BP	GO:0032147	activation of protein kinase activity	10/418	148/18903	0.00170533518062664	0.0152985130952347	0.0111325564400106	GPRC5A/TGFA/VEGFA/HBEGF/DLG1/WNT5A/AREG/ADRB2/EMP2/PRNP	10

BP	GO:0043393	regulation of protein binding	12/418	200/18903	0.00170839230903308	0.0152985130952347	0.01113255644001006	SLPI/CSF3/WNT5A/CDKN1A/B2M/ANXA2/ADRB2/PHLDA2/BMP2/BAMBI/ID1/HOPX	12
BP	GO:0060485	mesenchyme development	16/418	313/18903	0.00171933346926414	0.0153598063287419	0.0111771588387641	RDH10/LRG1/IL1B/SERPINB3/PITX2/WNT5A/HAS2/CITED2/ZNF750/ADIPOR1/EMP2/MDM2/BMP2/S100A4/BAMBI/HIF1A	16
BP	GO:0045620	negative regulation of lymphocyte differentiation	6/418	58/18903	0.00172925257614823	0.0153598063287419	0.0111771588387641	ANXA1/IL4R/RUNX1/ZC3H12A/ID2/IRF1	6
BP	GO:0048008	platelet-derived growth factor receptor signaling pathway	6/418	58/18903	0.00172925257614823	0.0153598063287419	0.0111771588387641	PLAT/TIPARP/VEGFA/ZFAND5/F3/SLC9A3R1	6
BP	GO:1901653	cellular response to peptide	18/418	373/18903	0.00172929627316728	0.0153598063287419	0.0111771588387641	CEACAM1/LYN/PLAT/RAB31/GJB2/IL1B/ASS1/CA2/ADIPOR1/AGTRAP/ADRB2/MDM2/XBP1/RAB10/ID1/LGMN/ERRFI1/PRNP	18
BP	GO:0006213	pyrimidine nucleoside metabolic process	4/418	24/18903	0.00176330267047632	0.015504291086482	0.0112822987768908	APOBEC3A/TYMP/CDA/UPP1	4

BP	GO:0032528	microvillus organization	4/418	24/18903	0.00176330267047632	0.015504291086482	0.0112822987768908	PLS1/FXYD5/EZR/SLC9A3R1	4
BP	GO:0035357	peroxisome proliferator activated receptor signaling pathway	4/418	24/18903	0.00176330267047632	0.015504291086482	0.0112822987768908	ALOX15B/FABP5/CITED2/BMP2	4
BP	GO:0035458	cellular response to interferon-beta	4/418	24/18903	0.00176330267047632	0.015504291086482	0.0112822987768908	CAPN2/OAS1/IFI16/IRF1	4
BP	GO:2001026	regulation of endothelial cell chemotaxis	4/418	24/18903	0.00176330267047632	0.015504291086482	0.0112822987768908	VEGFA/HSPB1/TMSB4X/LGMN	4
BP	GO:0032946	positive regulation of mononuclear cell proliferation	10/418	149/18903	0.00179292090329078	0.0156848682562715	0.0114137027585682	IL1A/ANXA1/IL1B/CD24/CD55/CDKN1A/CD46/TFRC/HLA-E/SELENOK	10
BP	GO:2000377	regulation of reactive oxygen species	10/418	149/18903	0.00179292090329078	0.0156848682562715	0.0114137027585682	CLEC7A/SOD2/CD177/ZC3H12A/CDKN1A/RAC1/HIF1A/ARF4/GADD45A/CRYAB	10

		metabolic process							
BP	GO:0002478	antigen processing and presentation of exogenous peptide antigen	5/418	40/18903	0.00179605917777596	0.0156848682562715	0.0114137027585682	CTSL/CTSD/B2M/HLA-E/LGMN	5
BP	GO:0015908	fatty acid transport	8/418	101/18903	0.00180178578138404	0.0156848682562715	0.0114137027585682	IL1A/ANXA1/IL1B/FABP5/CRABP2/FABP4/PNPLA8/RBP1	8
BP	GO:2001243	negative regulation of intrinsic apoptotic signaling pathway	8/418	101/18903	0.00180178578138404	0.0156848682562715	0.0114137027585682	PLAUR/SOD2/MUC1/HSPB1/MDM2/XBP1/PTGS2/HIF1A	8
BP	GO:0002720	positive regulation of cytokine production involved in immune response	7/418	79/18903	0.00181402177103886	0.0157287205147615	0.0114456135553642	CLEC7A/IL1B/CD55/WNT5A/RSAD2/B2M/HLA-E	7

BP	GO:2000514	regulation of CD4-positive, alpha-beta T cell activation	7/418	79/18903	0.00181402177103886	0.0157287205147615	0.01144561355536 42	ANXA1/NFKBIZ/CD55/IL4R/RUNX1/ZC3H12A/LGALS7B	7
BP	GO:0070372	regulation of ERK1 and ERK2 cascade	16/418	315/18903	0.00183407602651365	0.01587111333833458	0.01154923121646 22	S100A7/IL1A/CEACAM1/LYN/CXCL17/IL1B/CIB1/EPHA2/CCL20/DLG1/PSCA/DUSP10/BMP2/EZR/SLC9A3R1/ERRFI1	16
BP	GO:0001952	regulation of cell-matrix adhesion	9/418	125/18903	0.00185590784084949	0.0159966809950932	0.01164060539231 04	CIB1/VEGFA/CEACAM6/PLAU/MMP12/S100A10/SDC4/EMP2/RAC1	9
BP	GO:0003014	renal system process	9/418	125/18903	0.00185590784084949	0.0159966809950932	0.01164060539231 04	AQP3/SLC5A1/CLDN4/HAS2/ADM/EMP2/SLC9A3R1/LGMN/AKR1B1	9
BP	GO:0071709	membrane assembly	6/418	59/18903	0.00188977387689215	0.0162565193740525	0.01182968686715 95	UGCG/ANXA2/S100A10/EMP2/MAFB/VPS4B	6
BP	GO:0050920	regulation of chemotaxis	13/418	230/18903	0.00190300226967708	0.0163381530815105	0.01188909080073 31	S100A7/LYN/CXCL17/CXCL8/VEGFA/WNT5A/HSPB1/S100A14/DUSP1/F3/RAC1/TMSB4X/LGMN	13
BP	GO:00	regulation of neurogenesis	18/418	377/18903	0.00194291277686952	0.0166480957547447	0.01211463260871 58	LYN/IL1B/MME/BHLHE40/VEGFA/SPINT1/WNT5A/CRABP2/B2M/ANXA2/SKIL/CDKN2B/S100A10/DUSP10/BMP2/ID2/ID1/HIF1A	18

	50767								
BP	GO:0070227	lymphocyte apoptotic process	7/418	80/18903	0.00195096096490921	0.0166843432811219	0.01214100952722 37	LYN/ST3GAL1/BCL3/LGALS3/WNT5A/TNFRSF21/HIF1A	7
BP	GO:0006690	icosanoid metabolic process	9/418	126/18903	0.00196061161221316	0.0167015063262602	0.0121534988827248	ANXA1/IL1B/ALOX15B/FABP5/LTA4H/PTGR1/PNPLA8/PTGS2/AKR1B1	9
BP	GO:0038127	ERBB signaling pathway	9/418	126/18903	0.00196061161221316	0.0167015063262602	0.0121534988827248	PLAUR/CEACAM1/GPRC5A/FAM83A/TGFA/HBEGF/AREG/LGMN/ERRFI1	9
BP	GO:0071456	cellular response to hypoxia	10/418	151/18903	0.0019790074194481	0.0168254132742961	0.0122436645794649	AQP3/HMOX1/ERO1A/VEGFA/CITED2/NDRG1/MDM2/PTGS2/HIF1A/PMAIP1	10
BP	GO:0070232	regulation of T cell apoptotic process	5/418	41/18903	0.00200893922745264	0.0170467270368312	0.0124047121229115	ST3GAL1/BCL3/LGALS3/WNT5A/HIF1A	5
BP	GO:0032677	regulation of interleukin-8 production	8/418	103/18903	0.00204059723223722	0.0172041075528791	0.012519236159761	CD14/ANXA1/CLEC7A/IL1B/BCL3/WNT5A/F3/TMSB4X	8

BP	GO:0070167	regulation of biomineral tissue development	8/418	103/18903	0.00204059723223722	0.0172041075528791	0.012519236159761	ECM1/ODAPH/FAM20C/CEBPB/ADRB2/BMP2/ISG15/HIF1A	8
BP	GO:1903076	regulation of protein localization to plasma membrane	8/418	103/18903	0.00204059723223722	0.0172041075528791	0.012519236159761	CIB1/EPHA2/PLS1/LGALS3/DLG1/VAMP8/EZR/PRNP	8
BP	GO:0002711	positive regulation of T cell mediated immunity	6/418	60/18903	0.00206132370540952	0.0172041075528791	0.012519236159761	IL1B/CD55/RSAD2/B2M/HLA-B/HLA-E	6
BP	GO:0043407	negative regulation of MAP kinase activity	6/418	60/18903	0.00206132370540952	0.0172041075528791	0.012519236159761	LYN/IL1B/SERPINB3/DUSP1/DUSP10/BMP2	6
BP	GO:0001911	negative regulation of leukocyte mediated cytotoxicity	4/418	25/18903	0.00206291815965873	0.0172041075528791	0.012519236159761	SERPINB4/CEACAM1/HLA-B/HLA-E	4
BP	GO:0046697	decidualization	4/418	25/18903	0.00206291815965873	0.0172041075528791	0.012519236159761	GJB2/CITED2/CTSB/PTGS2	4

BP	GO:0050860	negative regulation of T cell receptor signaling pathway	4/418	25/18903	0.00206291815965873	0.0172041075528791	0.0125192361597 61	CEACAM1/LGALS3/EZR/PRNP	4
BP	GO:0098581	detection of external biotic stimulus	4/418	25/18903	0.00206291815965873	0.0172041075528791	0.0125192361597 61	CLEC7A/PGLYRP4/LY96/HLA-B	4
BP	GO:0072332	intrinsic apoptotic signaling pathway by p53 class mediator	7/418	81/18903	0.00209577083308608	0.0174116322064376	0.01267024952322	MUC1/BCL3/CDKN1A/MDM2/SHISA5/IFI16/PMAIP1	7
BP	GO:1901657	glycosyl compound metabolic process	7/418	81/18903	0.00209577083308608	0.0174116322064376	0.01267024952322	APOBEC3A/TYMP/FUCA1/CDA/UPP1/AKR1B1/AKR1B10	7
BP	GO:0032637	interleukin-8 production	8/418	104/18903	0.0021689883330914	0.0179857286823708	0.0130880130915193	CD14/ANXA1/CLEC7A/IL1B/BCL3/WNT5A/F3/TMSB4X	8
BP	GO:0045580	regulation of T cell differentiation	11/418	179/18903	0.00217781746887397	0.0179906660472198	0.0130916059565581	PRDM1/VNN1/ANXA1/NFKBIZ/IL4R/RUNX1/ZC3H12A/CD46/DUSP10/XBP1/IRF1	11

BP	GO:0048771	tissue remodeling	11/418	179/18903	0.00217781746887397	0.0179906660472198	0.01309160595655 81	IL1A/CEACAM1/ELF3/EPHA2/TNFAIP3/RUNX1/TFRC/ADRB2/MDM2/HIF1A/CST3	11
BP	GO:0031670	cellular response to nutrient	5/418	42/18903	0.0022396682285558	0.0183669059309026	0.0133653915012 06	HMOX1/CYP24A1/CDKN2B/XBP1/PIM1	5
BP	GO:0045429	positive regulation of nitric oxide biosynthetic process	5/418	42/18903	0.0022396682285558	0.0183669059309026	0.0133653915012 06	CLEC7A/IL1B/SOD2/ASS1/PTGS2	5
BP	GO:0050691	regulation of defense response to virus by host	5/418	42/18903	0.0022396682285558	0.0183669059309026	0.0133653915012 06	IL1B/TNFAIP3/ZC3H12A/MMP12/SELENOK	5
BP	GO:0030865	cortical cytoskeleton organization	6/418	61/18903	0.00224437706341007	0.0183669059309026	0.0133653915012 06	PLEK/PLS1/DLG1/RAC1/RND3/EZR	6
BP	GO:0046456	icosanoid biosynthetic process	6/418	61/18903	0.00224437706341007	0.0183669059309026	0.0133653915012 06	ANXA1/IL1B/FABP5/LTA4H/PNPLA8/PTGS2	6

BP	GO:0051092	positive regulation of NF-kappaB transcription factor activity	10/418	154/18903	0.00228706113680053	0.0186463753131328	0.0135687582370165	S100A8/S100A9/CRNN/S100A12/CLEC7A/IL1B/CIB1/WNT5A/TFRC/MTPN	10
BP	GO:0098754	detoxification	10/418	154/18903	0.00228706113680053	0.0186463753131328	0.0135687582370165	S100A9/DUOX2/SOD2/GPX3/TXNDC17/TXNRD1/PIM1/PTGS2/AKR1B10/MT1X	10
BP	GO:0110149	regulation of biomineralization	8/418	105/18903	0.00230364252953374	0.0187465881826116	0.0136416820184679	ECM1/ODAPH/FAM20C/CEBPB/ADRB2/BMP2/ISG15/HIF1A	8
BP	GO:0002726	positive regulation of T cell cytokine production	4/418	26/18903	0.00239592365380438	0.0193533943939467	0.0140832480944509	IL1B/CD55/RSAD2/B2M	4
BP	GO:0019883	antigen processing and presentation of endogenous antigen	4/418	26/18903	0.00239592365380438	0.0193533943939467	0.0140832480944509	B2M/HLA-C/HLA-B/HLA-E	4
BP	GO:0046639	negative regulation of alpha-beta T cell differentiation	4/418	26/18903	0.00239592365380438	0.0193533943939467	0.0140832480944509	ANXA1/IL4R/RUNX1/ZC3H12A	4

BP	GO:0051894	positive regulation of focal adhesion assembly	4/418	26/18903	0.00239592365380438	0.0193533943939467	0.0140832480944509	VEGFA/S100A10/SDC4/RAC1	4
BP	GO:0032233	positive regulation of actin filament bundle assembly	6/418	62/18903	0.00243941443875025	0.0196321198846014	0.0142860735087031	PLEK/CD47/S100A10/SDC4/RAC1/ID1	6
BP	GO:0070228	regulation of lymphocyte apoptotic process	6/418	62/18903	0.00243941443875025	0.0196321198846014	0.0142860735087031	LYN/ST3GAL1/BCL3/LGALS3/WNT5A/HIF1A	6
BP	GO:0016266	O-glycan processing	5/418	43/18903	0.00248910039255719	0.0199951630799171	0.0145502559711983	ST3GAL1/GALNT1/B3GNT5/GALNT5/GALNT3	5
BP	GO:0045619	regulation of lymphocyte differentiation	12/418	210/18903	0.00256327319710142	0.020553218112538	0.0149563463610688	PRDM1/VNN1/ANXA1/NFKBIZ/IL4R/RUNX1/ZC3H12A/CD46/DUSP10/XBP1/ID2/IRF1	12
BP	GO:0010717	regulation of epithelial to mesenchymal transition	8/418	107/18903	0.00259257653368057	0.0207203278027927	0.0150779502088972	LRG1/IL1B/SERPINB3/ZNF750/ADIPOR1/EMP2/BMP2/BAMBI	8

BP	GO:0045741	positive regulation of epidermal growth factor-activated receptor activity	3/418	13/18903	0.00260308008323415	0.0207203278027927	0.0150779502088972	TGFA/HBEGF/AREG	3
BP	GO:0045916	negative regulation of complement activation	3/418	13/18903	0.00260308008323415	0.0207203278027927	0.0150779502088972	CD55/CD59/CD46	3
BP	GO:0051918	negative regulation of fibrinolysis	3/418	13/18903	0.00260308008323415	0.0207203278027927	0.0150779502088972	PLAT/PLAU/THBD	3
BP	GO:0032481	positive regulation of type I interferon production	6/418	63/18903	0.00264692131763507	0.020925169414096	0.0152270111525916	CD14/MMP12/ISG15/OAS1/RIOK3/IRF1	6
BP	GO:0044091	membrane biogenesis	6/418	63/18903	0.00264692131763507	0.020925169414096	0.0152270111525916	UGCG/ANXA2/S100A10/EMP2/MAFB/VPS4B	6
BP	GO:00	intrinsic apoptotic signaling	6/418	63/18903	0.00264692131763507	0.020925169414096	0.0152270111525916	ERO1A/PPP1R15A/CEBPB/XBP1/SELENOK/PMAIP1	6

	70059	pathway in response to endoplasmic reticulum stress							
BP	GO:0010876	lipid localization	21/418	483/18903	0.00265062615305932	0.020925169414096	0.01522701115259 16	IL1A/CEACAM1/ANXA1/IL1B/OSBPL3/UGCG/FABP5/ZC3H12A/PLSCR1/CRABP2/NFKBIA/FABP4/ANXA2/GM2A/LRP10/PNPLA8/GLTP/RBP1/PTGS2/VPS4B/NPC2	21
BP	GO:1903829	positive regulation of protein localization	20/418	451/18903	0.00265275603098608	0.020925169414096	0.0152270111525916	IL1A/DOC2B/IL1B/CIB1/EPHA2/PLS1/LGALS3/DLG1/WNT5A/GLUL/ZC3H12A/TFRC/STOM/EZR/PTGS2/MFF/HIF1A/SH3GLB1/SFN/PRNP	20
BP	GO:0070304	positive regulation of stress-activated protein kinase signaling cascade	9/418	132/18903	0.00269197121162127	0.0211962417924053	0.0154242674828029	IL1A/LYN/IL1B/VEGFA/WNT5A/ZC3H12A/BMP2/TPD52L1/GADD45A	9
BP	GO:0019882	antigen processing and presentation	8/418	108/18903	0.00274728161135406	0.0215314710115567	0.0156682100267163	CTSL/CTSD/B2M/HLA-C/RAB10/HLA-B/HLA-E/LGMN	8
BP	GO:00	primary alcohol	8/418	108/18903	0.00274728161135406	0.0215314710115567	0.0156682100267163	SDR16C5/RDH10/DHRS9/ALDH1A3/DHRS3/BMP2/AKR1B1/AKR1B10	8

	34308	metabolic process							
BP	GO:0042771	intrinsic apoptotic signaling pathway in response to DNA damage by p53 class mediator	5/418	44/18903	0.00275809301098901	0.0215314710115567	0.01566821002671 63	MUC1/BCL3/CDKN1A/SHISA5/IFI16	5
BP	GO:1904407	positive regulation of nitric oxide metabolic process	5/418	44/18903	0.00275809301098901	0.0215314710115567	0.0156682100267163	CLEC7A/IL1B/SOD2/ASS1/PTGS2	5
BP	GO:0008625	extrinsic apoptotic signaling pathway via death domain receptors	7/418	85/18903	0.0027597592566037	0.0215314710115567	0.0156682100267163	HMOX1/MAL/LGALS3/TNFAIP3/SORT1/SKIL/PMAIP1	7
BP	GO:0090200	positive regulation of release of cytochrome c from mitochondria	4/418	27/18903	0.00276410874999618	0.0215314710115567	0.0156682100267163	PLAUR/TNFSF10/MFF/PMAIP1	4

BP	GO:0002064	epithelial cell development	12/418	213/18903	0.00287868740415683	0.0223840995661305	0.01628865826551 38	IL1A/CLDN1/PRDM1/IL1B/EPHA2/VEGFA/WNT5A/AFDN/EZR/HIF1A/AKR1B1/SFN	12
BP	GO:0036294	cellular response to decreased oxygen levels	10/418	159/18903	0.002885189889575	0.0223948131748539	0.016296454438452	AQP3/HMOX1/ERO1A/VEGFA/CITED2/NDRG1/MDM2/PTGS2/HIF1A/PMAIP1	10
BP	GO:0033273	response to vitamin	7/418	86/18903	0.00294848839258174	0.0228455572262096	0.01662445583051 41	AQP3/IL1A/KYNU/SOD2/CYP24A1/PIM1/PTGS2	7
BP	GO:0001909	leukocyte mediated cytotoxicity	9/418	134/18903	0.00297870916554066	0.0230388655812614	0.0167651241529545	SERPINB4/CEACAM1/CXCL6/DNASE1L3/CTSC/B2M/EMP2/HLA-B/HLA-E	9
BP	GO:0046636	negative regulation of alpha-beta T cell activation	5/418	45/18903	0.00304750504893126	0.023529323434328	0.0171220248332181	ANXA1/IL4R/RUNX1/ZC3H12A/LGALS7B	5
BP	GO:0007173	epidermal growth factor receptor signaling pathway	8/418	110/18903	0.00307825145362383	0.023724795154032	0.0172642674118585	PLAUR/CEACAM1/GPRC5A/FAM83A/TGFA/HBEGF/AREG/ERRFI1	8

BP	GO:0032613	interleukin-10 production	6/418	65/18903	0.00310130756016571	0.0238184780982849	0.01733243943994 19	CLEC7A/BCL3/CD47/TNFRSF21/CD46/ISG15	6
BP	GO:0032653	regulation of interleukin-10 production	6/418	65/18903	0.00310130756016571	0.0238184780982849	0.01733243943994 19	CLEC7A/BCL3/CD47/TNFRSF21/CD46/ISG15	6
BP	GO:0001910	regulation of leukocyte mediated cytotoxicity	7/418	87/18903	0.0031469408351907	0.023961150889126	0.0174362608300734	SERPINB4/CEACAM1/CXCL6/DNASE1L3/B2M/HLA-B/HLA-E	7
BP	GO:0050672	negative regulation of lymphocyte proliferation	7/418	87/18903	0.0031469408351907	0.023961150889126	0.0174362608300734	LYN/DLG1/TNFRSF21/CEBPB/SDC4/LGALS7B/PRNP	7
BP	GO:0030194	positive regulation of blood coagulation	4/418	28/18903	0.00316923231439699	0.023961150889126	0.0174362608300734	PLAT/PLAU/THBD/F3	4
BP	GO:0031342	negative regulation of cell killing	4/418	28/18903	0.00316923231439699	0.023961150889126	0.0174362608300734	SERPINB4/CEACAM1/HLA-B/HLA-E	4
BP	GO:00	negative regulation of	4/418	28/18903	0.00316923231439699	0.023961150889126	0.0174362608300734	TNFAIP3/EZR/ZFP36/PRNP	4

	32703	interleukin-2 production							
BP	GO:0044331	cell-cell adhesion mediated by cadherin	4/418	28/18903	0.00316923231439699	0.023961150889126	0.01743626083007 34	VEGFA/WNT5A/AFDN/CTNND1	4
BP	GO:0071624	positive regulation of granulocyte chemotaxis	4/418	28/18903	0.00316923231439699	0.023961150889126	0.0174362608300734	S100A7/CXCL8/S100A14/RAC1	4
BP	GO:0090314	positive regulation of protein targeting to membrane	4/418	28/18903	0.00316923231439699	0.023961150889126	0.0174362608300734	CIB1/STOM/MFF/PRNP	4
BP	GO:1900048	positive regulation of hemostasis	4/418	28/18903	0.00316923231439699	0.023961150889126	0.0174362608300734	PLAT/PLAU/THBD/F3	4
BP	GO:0009100	glycoprotein metabolic process	18/418	395/18903	0.00319959458005504	0.0241489262777902	0.0175729028749137	HS3ST1/NCCRP1/FUT3/PHLDA1/CTSL/ST3GAL1/GALNT1/HBEGF/DSE/SRD5A3/B3GNT5/GALNT5/MMP12/DCN/BMP2/GALNT3/HIF1A/CST3	18
BP	GO:0032611	interleukin-1 beta production	8/418	111/18903	0.00325495737503296	0.0243038240313169	0.0176856202333153	SERPINB1/CLEC7A/WNT5A/TNFAIP3/ZC3H12A/HSPB1/ERRFI1/IFI16	8

BP	GO:0032651	regulation of interleukin-1 beta production	8/418	111/18903	0.00325495737503296	0.0243038240313169	0.0176856202333153	SERPINB1/CLEC7A/WNT5A/TNFAIP3/ZC3H12A/HSPB1/ERRFI1/IFI16	8
BP	GO:1901222	regulation of NIK/NF-kappaB signaling	8/418	111/18903	0.00325495737503296	0.0243038240313169	0.0176856202333153	CD14/IL1B/BCL3/ZC3H12A/NFKBIA/CYLD/ADIPOR1/TMSB4X	8
BP	GO:0046049	UMP metabolic process	3/418	14/18903	0.00325904825683106	0.0243038240313169	0.0176856202333153	CDA/CMPK1/UPP1	3
BP	GO:0046131	pyrimidine ribonucleoside metabolic process	3/418	14/18903	0.00325904825683106	0.0243038240313169	0.0176856202333153	APOBEC3A/CDA/UPP1	3
BP	GO:0046133	pyrimidine ribonucleoside catabolic process	3/418	14/18903	0.00325904825683106	0.0243038240313169	0.0176856202333153	APOBEC3A/CDA/UPP1	3
BP	GO:1902043	positive regulation of extrinsic apoptotic signaling pathway via	3/418	14/18903	0.00325904825683106	0.0243038240313169	0.0176856202333153	MAL/SKIL/PMAIP1	3

		death domain receptors							
BP	GO:0010565	regulation of cellular ketone metabolic process	9/418	136/18903	0.003289032391240710	0.0244856414816386	0.0178179267532229	FMO2/CEACAM1/RDH10/ANXA1/IL1B/FABP5/ADM/BMP2/PTGS2	9
BP	GO:0032956	regulation of actin cytoskeleton organization	17/418	365/18903	0.00330400945011732	0.0245553083282528	0.0178686225363487	IL1A/TACSTD2/CSF3/PLEK/CDC42EP1/CD47/DLG1/S100A10/SDC4/RAC1/RND3/MTPN/ODAM/ID1/TMSB4X/ARPC3/TMSB10	17
BP	GO:0006879	cellular iron ion homeostasis	6/418	66/18903	0.00334917843057464	0.02478938428189 8	0.0180389569831 66	HEPHL1/STEAP4/LCN2/HMOX1/TFRC/HIF1A	6
BP	GO:0032945	negative regulation of mononuclear cell proliferation	7/418	88/18903	0.00335543754205233	0.024789384281898	0.01803895698316 6	LYN/DLG1/TNFRSF21/CEBPB/SDC4/LGALS7B/PRNP	7
BP	GO:0046889	positive regulation of lipid biosynthetic process	7/418	88/18903	0.00335543754205233	0.024789384281898	0.018038956983166	IL1A/RDH10/ANXA1/IL1B/CAPN2/ADM/PTGS2	7
BP	GO:20	positive regulation of signaling	5/418	46/18903	0.00335819576541959	0.024789384281898	0.018038956983166	TGFA/HBEGF/AREG/ADRB2/HIF1A	5

	002 73	receptor activity							
BP	GO :00 024 49	lymphocyte mediated immunity	17/418	367/189 03	0.003491 33473375 458	0.02568 540873 14941	0.018690 98392818 11	SERPINB4/CEACAM1/IL1B/BCL3/CD55/IL4R/CTSC/RSAD2/B2M/CD46/TFRC/ EMP2/IGHG4/HLA-B/IGKC/HLA-E/IGHG3	17
BP	GO :00 325 35	regulation of cellular component size	17/418	367/189 03	0.003491 33473375 458	0.02568 540873 14941	0.018690 98392818 11	CSF3/PLEK/SLC12A8/PLS1/CDC42EP1/VEGFA/DLG1/WNT5A/CRABP2/PPP1R 15A/RAC1/MTPN/EZR/SLC9A3R1/TMSB4X/ARPC3/TMSB10	17
BP	GO :00 027 09	regulation of T cell mediated immunity	7/418	89/1890 3	0.003574 30239339 119	0.02625 159909 09572	0.019102 99430417 48	CEACAM1/IL1B/CD55/RSAD2/B2M/HLA-B/HLA-E	7
BP	GO :00 315 71	mitotic G1 DNA damage checkpoint signaling	4/418	29/1890 3	0.003613 01851079 92	0.02640 282757 89173	0.019213 04157916 63	MUC1/CDKN1A/MDM2/PLK2	4
BP	GO :00 346 98	response to gonadotropin	4/418	29/1890 3	0.003613 01851079 92	0.02640 282757 89173	0.019213 04157916 63	PLAT/GJB2/PAPPA/GCLM	4
BP	GO :19 021 75	regulation of oxidative stress-induced intrinsic apoptotic	4/418	29/1890 3	0.003613 01851079 92	0.02640 282757 89173	0.019213 04157916 63	VNN1/SOD2/HSPB1/HIF1A	4

		signaling pathway							
BP	GO:0110053	regulation of actin filament organization	14/418	278/18903	0.00372806855953428	0.0271980961688895	0.01979174961488899	TACSTD2/CSF3/PLEK/CDC42EP1/CD47/DLG1/S100A10/SDC4/RAC1/MTPN/ID1/TMSB4X/ARPC3/TMSB10	14
BP	GO:0045926	negative regulation of growth	13/418	249/18903	0.00377428780113131	0.027489396151573	0.02000372534599 59	ALOX15B/CDA/OSGIN1/WNT5A/CDKN1A/CITED2/ADIPOR1/ADRB2/DUSP10/LGMN/HIF1A/CRYAB/MT1X	13
BP	GO:0043583	ear development	12/418	221/18903	0.00387623167898743	0.0281823526751736	0.0205079820382 27	RDH10/GJB2/PLS1/ALDH1A3/WNT5A/SDC4/LRP10/BMP2/CEBPD/SLC9A3R1/GJB6/MAFB	12
BP	GO:0048839	inner ear development	11/418	193/18903	0.00388544294231319	0.0281823526751736	0.02050798203822 7	GJB2/PLS1/ALDH1A3/WNT5A/SDC4/LRP10/BMP2/CEBPD/SLC9A3R1/GJB6/MAFB	11
BP	GO:0042446	hormone biosynthetic process	6/418	68/18903	0.00388877772611663	0.0281823526751736	0.02050798203822 7	DUOX2/SRD5A3/ADM/BMP2/HIF1A/AKR1B1	6
BP	GO:0002705	positive regulation of leukocyte mediated immunity	9/418	140/18903	0.00398571210176613	0.0287096860750533	0.0208917166404278	CLEC7A/IL1B/CD177/CD55/RSAD2/B2M/TFRC/HLA-B/HLA-E	9

BP	GO:0002679	respiratory burst involved in defense response	3/418	15/18903	0.00400753055052231	0.0287096860750533	0.0208917166404278	GRN/DUSP10/SELENOK	3
BP	GO:0006206	pyrimidine nucleobase metabolic process	3/418	15/18903	0.00400753055052231	0.0287096860750533	0.0208917166404278	TYMP/CDA/CMPK1	3
BP	GO:0009173	pyrimidine ribonucleoside monophosphate metabolic process	3/418	15/18903	0.00400753055052231	0.0287096860750533	0.0208917166404278	CDA/CMPK1/UPP1	3
BP	GO:0032352	positive regulation of hormone metabolic process	3/418	15/18903	0.00400753055052231	0.0287096860750533	0.0208917166404278	RDH10/ADM/HIF1A	3
BP	GO:0045064	T-helper 2 cell differentiation	3/418	15/18903	0.00400753055052231	0.0287096860750533	0.0208917166404278	ANXA1/BCL3/IL4R	3
BP	GO:2000345	regulation of hepatocyte proliferation	3/418	15/18903	0.00400753055052231	0.0287096860750533	0.0208917166404278	CEACAM1/TNFAIP3/XBP1	3

BP	GO:0001938	positive regulation of endothelial cell proliferation	8/418	115/18903	0.00404184558658245	0.028755620872486	0.02092514287746l2	ECM1/LRG1/HMOX1/VEGFA/WNT5A/F3/BMP2/HIF1A	8
BP	GO:0033559	unsaturated fatty acid metabolic process	8/418	115/18903	0.00404184558658245	0.028755620872486	0.0209251428774612	ANXA1/IL1B/ALOX15B/FABP5/PTGR1/PNPLA8/PTGS2/AKR1B1	8
BP	GO:0032309	icosanoid secretion	5/418	48/18903	0.00404684366969768	0.028755620872486	0.0209251428774612	IL1A/ANXA1/IL1B/PNPLA8/PTGS2	5
BP	GO:0045581	negative regulation of T cell differentiation	5/418	48/18903	0.00404684366969768	0.028755620872486	0.0209251428774612	ANXA1/IL4R/RUNX1/ZC3H12A/IRF1	5
BP	GO:0061028	establishment of endothelial barrier	5/418	48/18903	0.00404684366969768	0.028755620872486	0.0209251428774612	CLDN1/IL1B/VEGFA/AFDN/EZR	5
BP	GO:0001959	regulation of cytokine-mediated signaling pathway	10/418	167/18903	0.00409589984793104	0.02878546528l7772	0.0209468603194626	ECM1/IL1RN/CD24/WNT5A/TNFAIP3/CYLD/MMP12/ISG15/OAS1/HIF1A	10

BP	GO:0002474	antigen processing and presentation of peptide antigen via MHC class I	4/418	30/18903	0.00409715318198293	0.0287854652817772	0.02094686031946 26	B2M/HLA-C/HLA-B/HLA-E	4
BP	GO:0008209	androgen metabolic process	4/418	30/18903	0.00409715318198293	0.0287854652817772	0.02094686031946 26	DHRS9/TIPARP/SRD5A3/ADM	4
BP	GO:0019886	antigen processing and presentation of exogenous peptide antigen via MHC class II	4/418	30/18903	0.00409715318198293	0.0287854652817772	0.02094686031946 26	CTSL/CTSD/B2M/LGMN	4
BP	GO:0044819	mitotic G1/S transition checkpoint signaling	4/418	30/18903	0.00409715318198293	0.0287854652817772	0.02094686031946 26	MUC1/CDKN1A/MDM2/PLK2	4
BP	GO:0050820	positive regulation of coagulation	4/418	30/18903	0.00409715318198293	0.0287854652817772	0.02094686031946 26	PLAT/PLAU/THBD/F3	4

BP	GO:0071480	cellular response to gamma radiation	4/418	30/18903	0.00409715318198293	0.0287854652817772	0.0209468603194626	CDKN1A/MDM2/HSPA5/CRYAB	4
BP	GO:0030858	positive regulation of epithelial cell differentiation	6/418	69/18903	0.00418151417372031	0.0291438866653234	0.0212076795413566	CD24/ALOX15B/SULT2B1/CDKN2B/BMP2/SFN	6
BP	GO:0048645	animal organ formation	6/418	69/18903	0.00418151417372031	0.0291438866653234	0.0212076795413566	RDH10/WNT5A/CITED2/EMP2/BMP2/PIM1	6
BP	GO:0061180	mammary gland epithelium development	6/418	69/18903	0.00418151417372031	0.0291438866653234	0.0212076795413566	EPHA2/WNT5A/AREG/CEBPB/ID2/HIF1A	6
BP	GO:0070830	bicellular tight junction assembly	6/418	69/18903	0.00418151417372031	0.0291438866653234	0.0212076795413566	CLDN1/CLDN4/EPHA2/DLG1/CLDN7/AFDN	6
BP	GO:1902305	regulation of sodium ion transmembrane transport	6/418	69/18903	0.00418151417372031	0.0291438866653234	0.0212076795413566	DLG1/FXYD5/STOM/FXYD3/SLC9A3R1/ATP1B1	6
BP	GO:00	positive regulation of	11/418	195/18903	0.0041987671440336	0.0292175356997243	0.0212612731178772	CSF3/PLEK/CDC42EP1/CD47/DLG1/S100A10/SDC4/RAC1/CLIP1/ID1/VPS4B	11

	51495	cytoskeleton organization							
BP	GO:0098742	cell-cell adhesion via plasma-membrane adhesion molecules	14/418	282/18903	0.00422824253249 21	0.02937586624 32281	0.02137648847905 39	CEACAM1/CLDN1/IL1RN/CLDN4/CD177/CEACAM6/FXYD5/CLDN7/MPZL2/CDH3/CEACAM5/BMP2/NECTIN4/LGALS7B	14
BP	GO:0002028	regulation of sodium ion transport	7/418	92/18903	0.00429638385011 011	0.02980190067 45733	0.02168650895769 86	DLG1/FXYD5/ADRB2/STOM/FXYD3/SLC9A3R1/ATP1B1	7
BP	GO:0019884	antigen processing and presentation of exogenous antigen	5/418	49/18903	0.00442650885191 47	0.03055899475 96639	0.02223743783426 34	CTSL/CTSD/B2M/HLA-E/LGMN	5
BP	GO:0033628	regulation of cell adhesion mediated by integrin	5/418	49/18903	0.00442650885191 47	0.03055899475 96639	0.02223743783426 34	LYN/CIB1/EPHA2/MUC1/PLAU	5
BP	GO:2000404	regulation of T cell migration	5/418	49/18903	0.00442650885191 47	0.03055899475 96639	0.02223743783426 34	S100A7/ECM1/CCL20/WNT5A/SELENOK	5

BP	GO:0062197	cellular response to chemical stress	16/418	345/18903	0.00448130387542742	0.0308041551069454	0.0224158382700426	VNN1/GJB2/ANXA1/NCOA7/SOD2/HMOX1/TNFAIP3/ZC3H12A/NET1/HSPB1/PNPLA8/PTGS2/ERRFI1/HIF1A/AKR1B1/SETX	16
BP	GO:0045582	positive regulation of T cell differentiation	8/418	117/18903	0.00448655138499761	0.0308041551069454	0.0224158382700426	VNN1/ANXA1/NFKBIZ/IL4R/RUNX1/CD46/DUSP10/XBP1	8
BP	GO:0042130	negative regulation of T cell proliferation	6/418	70/18903	0.00449021665975382	0.0308041551069454	0.0224158382700426	DLG1/TNFRSF21/CEBPB/SDC4/LGALS7B/PRNP	6
BP	GO:0043627	response to estrogen	6/418	70/18903	0.00449021665975382	0.0308041551069454	0.0224158382700426	HMOX1/CD24/MME/IL4R/CITED2/KRT19	6
BP	GO:2000106	regulation of leukocyte apoptotic process	7/418	93/18903	0.0045600108297733	0.0312339299782278	0.02272858062488889	LYN/ANXA1/ST3GAL1/BCL3/LGALS3/WNT5A/HIF1A	7
BP	GO:2001056	positive regulation of cysteine-type endopeptidase activity	9/418	143/18903	0.00458009592225316	0.0313224087327798	0.02279296562248805	S100A8/S100A9/CLEC7A/TNFSF10/CTSD/F3/LGMN/IFI16/PMAIP1	9

BP	GO:0002507	tolerance induction	4/418	31/18903	0.00462328056533365	0.0314210514315833	0.0228647468083375	LYN/TNFAIP3/HLA-B/HLA-E	4
BP	GO:0010039	response to iron ion	4/418	31/18903	0.00462328056533365	0.0314210514315833	0.0228647468083375	HMOX1/B2M/TFRC/HIF1A	4
BP	GO:0035767	endothelial cell chemotaxis	4/418	31/18903	0.00462328056533365	0.0314210514315833	0.0228647468083375	VEGFA/HSPB1/TMSB4X/LGMN	4
BP	GO:0150117	positive regulation of cell-substrate junction organization	4/418	31/18903	0.00462328056533365	0.0314210514315833	0.0228647468083375	VEGFA/S100A10/SDC4/RAC1	4
BP	GO:0002286	T cell activation involved in immune response	8/418	118/18903	0.00472253907506386	0.0319961174543087	0.023283215904966	CEACAM1/ANXA1/BCL3/NFKBIZ/LGALS3/IL4R/ZC3H12A/CD46	8
BP	GO:0002718	regulation of cytokine production involved in	8/418	118/18903	0.00472253907506386	0.0319961174543087	0.023283215904966	CLEC7A/IL1B/HMOX1/CD55/WNT5A/RSAD2/B2M/HLA-E	8

		immune response							
BP	GO:0061077	chaperone-mediated protein folding	6/418	71/18903	0.00481539267621071	0.0323726166660724	0.0235571901597506	ERO1A/HSPB1/HSPH1/HSPA6/DNAJB6/HSPA5	6
BP	GO:2000379	positive regulation of reactive oxygen species metabolic process	6/418	71/18903	0.00481539267621071	0.0323726166660724	0.0235571901597506	CLEC7A/SOD2/CD177/ZC3H12A/CDKN1A/GADD45A	6
BP	GO:0042304	regulation of fatty acid biosynthetic process	5/418	50/18903	0.00483086616558574	0.0323726166660724	0.0235571901597506	CEACAM1/ANXA1/IL1B/FABP5/PTGS2	5
BP	GO:2001238	positive regulation of extrinsic apoptotic signaling pathway	5/418	50/18903	0.00483086616558574	0.0323726166660724	0.0235571901597506	TNFSF10/MAL/CYLD/SKIL/PMAIP1	5
BP	GO:1903035	negative regulation of response to wounding	7/418	94/18903	0.00483566144988278	0.0323726166660724	0.0235571901597506	PLAUR/CEACAM1/PLAT/SERPINB2/PLAU/THBD/ANXA2	7

BP	GO:0009130	pyrimidine nucleoside monophosphate biosynthetic process	3/418	16/18903	0.00485218853919392	0.0323726166660724	0.02355719015975 06	CDA/CMPK1/UPP1	3
BP	GO:0030033	microvillus assembly	3/418	16/18903	0.00485218853919392	0.0323726166660724	0.0235571901597506	FXYD5/EZR/SLC9A3R1	3
BP	GO:0030852	regulation of granulocyte differentiation	3/418	16/18903	0.00485218853919392	0.0323726166660724	0.0235571901597506	CEACAM1/RUNX1/TRIB1	3
BP	GO:0046135	pyrimidine nucleoside catabolic process	3/418	16/18903	0.00485218853919392	0.0323726166660724	0.0235571901597506	APOBEC3A/CDA/UPP1	3
BP	GO:2001212	regulation of vasculogenesis	3/418	16/18903	0.00485218853919392	0.0323726166660724	0.0235571901597506	CEACAM1/ADM/EMP2	3
BP	GO:0032388	positive regulation of intracellular transport	11/418	199/18903	0.00488569403994496	0.0325464679185358	0.0236836997668064	IL1B/CIB1/ZC3H12A/ANXA2/STOM/EZR/PTGS2/MFF/SH3GLB1/SFN/PRNP	11

BP	GO:0045089	positive regulation of innate immune response	9/418	145/18903	0.00501318777955993	0.0333449476357335	0.0242647445038061	LYN/CLEC7A/WNT5A/PLSCR1/AKIRIN2/MMP12/HLA-E/IFI16/RIOK3	9
BP	GO:1905475	regulation of protein localization to membrane	10/418	172/18903	0.00503616537945457	0.0334468734167424	0.0243389147517713	CIB1/EPHA2/PLS1/LGALS3/DLG1/VAMP8/STOM/EZR/MFF/PRNP	10
BP	GO:0034109	homotypic cell-cell adhesion	7/418	95/18903	0.00512367244209684	0.0339249220787321	0.0246867854028302	PLAUR/CEACAM1/LYN/PLEK/HSPB1/CEACAM5/RAP2B	7
BP	GO:0045638	negative regulation of myeloid cell differentiation	7/418	95/18903	0.00512367244209684	0.0339249220787321	0.0246867854028302	CEACAM1/LYN/CIB1/NFKBIA/TRIB1/ZFP36/MAFB	7
BP	GO:0071241	cellular response to inorganic substance	12/418	229/18903	0.00513484437711376	0.0339474582874238	0.0247031847492008	CLDN1/HMOX1/CPNE8/WNT5A/B2M/FABP4/FOSB/PTGS2/LGMN/HSPA5/PRNP/MT1X	12
BP	GO:0006516	glycoprotein catabolic process	4/418	32/18903	0.00519300032495204	0.0342283731825647	0.0249076033685483	NCCRP1/CTSL/MMP12/CST3	4
BP	GO:00	chaperone cofactor-dependent	4/418	32/18903	0.00519300032495204	0.0342283731825647	0.0249076033685483	ERO1A/HSPH1/HSPA6/HSPA5	4

	51085	protein refolding							
BP	GO:0002367	cytokine production involved in immune response	8/418	120/18903	0.00522297387475965	0.0343740901094875	0.025013639942373	CLEC7A/IL1B/HMOX1/CD55/WNT5A/RSAD2/B2M/HLA-E	8
BP	GO:0032692	negative regulation of interleukin-1 production	5/418	51/18903	0.00526075674141925	0.0345706871578979	0.0251567014100951	CEACAM1/SERPINB1/TNFAIP3/ZC3H12A/ERRFI1	5
BP	GO:0031214	biomineral tissue development	10/418	174/18903	0.0054565246243298	0.0357791876833433	0.0260361136917692	ECM1/ODAPH/FAM20C/CEBPB/ADRB2/BMP2/ODAM/ISG15/PTGS2/HIF1A	10
BP	GO:1902903	regulation of supramolecular fiber organization	17/418	384/18903	0.00546637551834189	0.0357791876833433	0.0260361136917692	TACSTD2/CSF3/CIB1/PLEK/CDC42EP1/CD47/DLG1/S100A10/SDC4/RAC1/MTPN/CLIP1/ID1/TMSB4X/ARPC3/TMSB10/CRYAB	17
BP	GO:0106106	cold-induced thermogenesis	9/418	147/18903	0.00547740882383447	0.0357791876833433	0.0260361136917692	LCN2/VEGFA/IL4R/FABP5/FABP4/CEBPB/ADIPOR1/ADRB2/ID1	9
BP	GO:0120161	regulation of cold-induced thermogenesis	9/418	147/18903	0.00547740882383447	0.0357791876833433	0.0260361136917692	LCN2/VEGFA/IL4R/FABP5/FABP4/CEBPB/ADIPOR1/ADRB2/ID1	9

BP	GO:0031623	receptor internalization	8/418	121/18903	0.00548790135960633	0.0357942223007159	0.02604705421425 09	CEACAM1/RAB31/CXCL8/VEGFA/TFRC/ANXA2/ADM/EZR	8
BP	GO:0042246	tissue regeneration	6/418	73/18903	0.00551719721800889	0.0358782021468733	0.0261081654066492	ANXA1/RUNX1/CDKN1A/DUSP10/MTPN/HOPX	6
BP	GO:0046637	regulation of alpha-beta T cell differentiation	6/418	73/18903	0.00551719721800889	0.0358782021468733	0.0261081654066492	PRDM1/ANXA1/NFKBIZ/IL4R/RUNX1/ZC3H12A	6
BP	GO:0045927	positive regulation of growth	13/418	261/18903	0.00558114945620805	0.0362401532297611	0.0263715531511762	S100A8/S100A9/CIB1/PLS1/VEGFA/HBEGF/CRABP2/ITSN2/MTPN/EZR/PIM1/HOPX/SFN	13
BP	GO:0001763	morphogenesis of a branching structure	11/418	203/18903	0.00565881242812368	0.0366899262773004	0.0266988479546488	RDH10/PRDM1/TACSTD2/EPHA2/VEGFA/DLG1/SPINT1/WNT5A/AREG/ADM/BMP2	11
BP	GO:0010469	regulation of signaling receptor activity	10/418	175/18903	0.00567674406158113	0.0366972951909904	0.0267042102305148	GPRC5A/TGFA/HBEGF/PSCA/AREG/PLAU/ADRB2/PHLDA2/ERRFI1/HIF1A	10
BP	GO:00	cellular response to oxygen levels	10/418	175/18903	0.00567674406158113	0.0366972951909904	0.0267042102305148	AQP3/HMOX1/ERO1A/VEGFA/CITED2/NDRG1/MDM2/PTGS2/HIF1A/PMAIP1	10

	71453								
BP	GO:0010043	response to zinc ion	5/418	52/18903	0.00571701440889119	0.0368486031959506	0.0268143153691357	S100A8/SOD2/ASS1/LTA4H/MT1X	5
BP	GO:0043392	negative regulation of DNA binding	5/418	52/18903	0.00571701440889119	0.0368486031959506	0.0268143153691357	HMOX1/NFKBIA/ID1/TMSB4X/IFI16	5
BP	GO:0030278	regulation of ossification	8/418	122/18903	0.00576295800115748	0.0369976232358087	0.0269227555812063	ECM1/FAM20C/DHRS3/WNT5A/ADRB2/BMP2/ISG15/HIF1A	8
BP	GO:0002921	negative regulation of humoral immune response	3/418	17/18903	0.0057963011104556	0.0369976232358087	0.0269227555812063	CD55/CD59/CD46	3
BP	GO:0010224	response to UV-B	3/418	17/18903	0.0057963011104556	0.0369976232358087	0.0269227555812063	MME/CDKN1A/IVL	3
BP	GO:0017014	protein nitrosylation	3/418	17/18903	0.0057963011104556	0.0369976232358087	0.0269227555812063	S100A8/S100A9/NCOA7	3

BP	GO:0018119	peptidyl-cysteine S-nitrosylation	3/418	17/18903	0.0057963011104556	0.0369976232358087	0.0269227555812063	S100A8/S100A9/NCOA7	3
BP	GO:1903729	regulation of plasma membrane organization	3/418	17/18903	0.0057963011104556	0.0369976232358087	0.0269227555812063	ANXA2/S100A10/SH3GLB1	3
BP	GO:0002828	regulation of type 2 immune response	4/418	33/18903	0.00580786488324137	0.0369976232358087	0.0269227555812063	ECM1/ANXA1/IL4R/RSAD2	4
BP	GO:0050858	negative regulation of antigen receptor-mediated signaling pathway	4/418	33/18903	0.00580786488324137	0.0369976232358087	0.0269227555812063	CEACAM1/LGALS3/EZR/PRNP	4
BP	GO:0050864	regulation of B cell activation	11/418	204/18903	0.00586639634681392	0.0373160873880303	0.0271544983738985	LYN/TNFAIP3/CDKN1A/TNFRSF21/AKIRIN2/TFRC/IGHG4/XBP1/IGKC/ID2/IGHG3	11
BP	GO:0032732	positive regulation of interleukin-1 production	6/418	74/18903	0.00589484118779736	0.0373339941893833	0.0271675289524574	SAA1/CLEC7A/WNT5A/HSPB1/S100A13/IFI16	6

BP	GO:0048662	negative regulation of smooth muscle cell proliferation	6/418	74/18903	0.00589484118779736	0.0373339941893833	0.0271675289524574	SOD2/HMOX1/TNFAIP3/CDKN1A/TRIB1/BMP2	6
BP	GO:0050688	regulation of defense response to virus	6/418	74/18903	0.00589484118779736	0.0373339941893833	0.0271675289524574	IL1B/TNFAIP3/ZC3H12A/MMP12/RIOK3/SELENOK	6
BP	GO:1902905	positive regulation of supramolecular fiber organization	10/418	176/18903	0.00590384539166473	0.037336909351049	0.0271696502829144	CSF3/PLEK/CDC42EP1/CD47/DLG1/S100A10/SDC4/RAC1/CLIP1/ID1	10
BP	GO:0045860	positive regulation of protein kinase activity	18/418	420/18903	0.00600545086268536	0.0379245957658021	0.0275973030973114	S100A12/GPRC5A/IL1B/CD24/CIB1/TGFA/VEGFA/HBEGF/DLG1/WNT5A/AREG/CDKN1A/ADRB2/SDC4/EMP2/BMP2/TPD52L1/PRNP	18
BP	GO:0051222	positive regulation of protein transport	14/418	294/18903	0.00606389267785781	0.03823839971463	0.02782565471224479	IL1A/DOC2B/IL1B/CIB1/GLUL/ZC3H12A/STOM/EZR/PTGS2/MFF/HIF1A/SH3GLB1/SFN/PRNP	14
BP	GO:1901654	response to ketone	11/418	205/18903	0.00607992932374114	0.0382842811883988	0.027859042146249	CLDN1/PLAT/GJB2/PAPPA/CLDN4/CDA/ASS1/FBXO32/FOSL1/FOSB/ERRFI1	11

BP	GO:0007015	actin filament organization	19/418	454/18903	0.00619405313099276	0.038931077762426	0.02832970876075 85	TACSTD2/CSF3/PLEK/PLS1/CDC42EP1/CD47/DLG1/S100A10/SDC4/EMP2/RAC1/RND3/MTPN/EZR/ID1/TMSB4X/TPM4/ARPC3/TMSB10	19
BP	GO:0071622	regulation of granulocyte chemotaxis	5/418	53/18903	0.00620046455895846	0.038931077762426	0.0283297087607585	S100A7/CXCL17/CXCL8/S100A14/RAC1	5
BP	GO:0016239	positive regulation of macroautophagy	6/418	75/18903	0.00629098859330118	0.0393862752904386	0.0286609508978478	HMOX1/HSPB8/ADRB2/DCN/HIF1A/SH3GLB1	6
BP	GO:0120192	tight junction assembly	6/418	75/18903	0.00629098859330118	0.0393862752904386	0.0286609508978478	CLDN1/CLDN4/EPHA2/DLG1/CLDN7/AFDN	6
BP	GO:2001237	negative regulation of extrinsic apoptotic signaling pathway	7/418	99/18903	0.00640609669843225	0.0400495601890543	0.0291436158812798	IL1A/IL1B/HMOX1/LGALS3/IL19/TNFAIP3/GCLM	7
BP	GO:0002495	antigen processing and presentation of peptide	4/418	34/18903	0.00646937703568262	0.0401579227925185	0.0292224701327709	CTSL/CTSD/B2M/LGMN	4

		antigen via MHC class II							
BP	GO:0034405	response to fluid shear stress	4/418	34/18903	0.00646937703568262	0.0401579227925185	0.02922247013277709	ASS1/HAS2/CITED2/PTGS2	4
BP	GO:0035335	peptidyl-tyrosine dephosphorylation	4/418	34/18903	0.00646937703568262	0.0401579227925185	0.02922247013277709	DUSP5/DUSP1/PTPN13/DUSP10	4
BP	GO:0090313	regulation of protein targeting to membrane	4/418	34/18903	0.00646937703568262	0.0401579227925185	0.02922247013277709	CIB1/STOM/MFF/PRNP	4
BP	GO:1902624	positive regulation of neutrophil migration	4/418	34/18903	0.00646937703568262	0.0401579227925185	0.02922247013277709	IL1A/CXCL8/RAC1/SELENOK	4
BP	GO:0002285	lymphocyte activation involved in immune response	11/418	207/18903	0.00652530323039323	0.04044762427 9175	0.02943328265624 18	CEACAM1/ANXA1/ST3GAL1/BCL3/NFKBIZ/LGALS3/IL4R/ZC3H12A/CD46/TFRC/XBP1	11
BP	GO:0002706	regulation of lymphocyte mediated immunity	10/418	179/18903	0.00662787160801295	0.0409671837723007	0.029811360273665	SERPINB4/CEACAM1/IL1B/CD55/RSAD2/B2M/CD46/TFRC/HLA-B/HLA-E	10

BP	GO:0060759	regulation of response to cytokine stimulus	10/418	179/18903	0.00662787160801295	0.0409671837723007	0.029811360273665	ECM1/IL1RN/CD24/WNT5A/TNFAIP3/CYLD/MMP12/ISG15/OAS1/HIF1A	10
BP	GO:0010811	positive regulation of cell-substrate adhesion	8/418	125/18903	0.00665134056930547	0.0409962740308391	0.0298325289286197	CIB1/VEGFA/CEACAM6/HAS2/S100A10/SDC4/EMP2/RAC1	8
BP	GO:1904375	regulation of protein localization to cell periphery	8/418	125/18903	0.00665134056930547	0.0409962740308391	0.0298325289286197	CIB1/EPHA2/PLS1/LGALS3/DLG1/VAMP8/EZR/PRNP	8
BP	GO:0001655	urogenital system development	16/418	360/18903	0.00667486477389657	0.0410833226224338	0.0298958732126635	RDH10/PRDM1/TACSTD2/ANXA1/CD24/MME/TIPARP/ALOX15B/VEGFA/DLG1/WNT5A/ASS1/HAS2/SDC4/BMP2/AKR1B1	16
BP	GO:0010822	positive regulation of mitochondrion organization	6/418	76/18903	0.00670614443754676	0.0411953703841942	0.0299774091125257	PLAUR/TNFSF10/DCN/MFF/HIF1A/PMAIP1	6
BP	GO:0045778	positive regulation of ossification	5/418	54/18903	0.00671192304657809	0.0411953703841942	0.0299774091125257	FAM20C/WNT5A/ADRB2/BMP2/ISG15	5
BP	GO:00	leukocyte homeostasis	7/418	100/18903	0.00676099916983345	0.0413595619342036	0.0300968894624182	CXCL6/LYN/ANXA1/TNFAIP3/SKIL/HIF1A/PMAIP1	7

	017<br>76								
BP	GO<br>:00<br>988<br>69	cellular<br>oxidant<br>detoxification	7/418	100/189<br>03	0.006760<br>99916983<br>345	0.04135<br>956193<br>42036	0.030096<br>88946241<br>82	S100A9/DUOX2/SOD2/GPX3/TXNDC17/TXNRD1/PTGS2	7
BP	GO<br>:00<br>323<br>10	prostaglandin<br>secretion	3/418	18/1890<br>3	0.006842<br>78335890<br>829	0.04135<br>956193<br>42036	0.030096<br>88946241<br>82	IL1A/IL1B/PTGS2	3
BP	GO<br>:00<br>323<br>54	response to<br>follicle-<br>stimulating<br>hormone	3/418	18/1890<br>3	0.006842<br>78335890<br>829	0.04135<br>956193<br>42036	0.030096<br>88946241<br>82	PLAT/PAPPA/GCLM	3
BP	GO<br>:00<br>359<br>70	peptidyl-<br>threonine<br>dephosphoryla<br>tion	3/418	18/1890<br>3	0.006842<br>78335890<br>829	0.04135<br>956193<br>42036	0.030096<br>88946241<br>82	DUSP5/DUSP1/DUSP10	3
BP	GO<br>:00<br>381<br>28	ERBB2<br>signaling<br>pathway	3/418	18/1890<br>3	0.006842<br>78335890<br>829	0.04135<br>956193<br>42036	0.030096<br>88946241<br>82	TGFA/HBEGF/AREG	3
BP	GO<br>:00<br>431<br>16	negative<br>regulation of<br>vascular<br>permeability	3/418	18/1890<br>3	0.006842<br>78335890<br>829	0.04135<br>956193<br>42036	0.030096<br>88946241<br>82	CEACAM1/VEGFA/ADM	3

BP	GO:0045623	negative regulation of T-helper cell differentiation	3/418	18/18903	0.00684278335890829	0.0413595619342036	0.0300968894624182	ANXA1/IL4R/ZC3H12A	3
BP	GO:0060602	branch elongation of an epithelium	3/418	18/18903	0.00684278335890829	0.0413595619342036	0.0300968894624182	RDH10/WNT5A/AREG	3
BP	GO:0060644	mammary gland epithelial cell differentiation	3/418	18/18903	0.00684278335890829	0.0413595619342036	0.0300968894624182	CEBPB/ID2/HIF1A	3
BP	GO:0097067	cellular response to thyroid hormone stimulus	3/418	18/18903	0.00684278335890829	0.0413595619342036	0.0300968894624182	CTSL/GCLM/CTSB	3
BP	GO:0006575	cellular modified amino acid metabolic process	10/418	180/18903	0.00688393551973515	0.0415508262724345	0.0302360703767372	VNN1/MBOAT2/DUOX2/SOD2/ASS1/PLSCR1/GCLM/SERINC2/CTSB/ETHE1	10
BP	GO:0006024	glycosaminoglycan biosynthetic process	6/418	77/18903	0.0071408117682199	0.0427996955403938	0.0311448585396916	HS3ST1/IL1B/ST3GAL1/DSE/HAS2/GALNT5	6

BP	GO:0009123	nucleoside monophosphate metabolic process	6/418	77/18903	0.00714081176821 99	0.04279969554 03938	0.03114485853969 16	TYMP/DLG1/CDA/CMPK1/UPP1/AK2	6
BP	GO:0032729	positive regulation of interferon-gamma production	6/418	77/18903	0.00714081176821 99	0.04279969554 03938	0.03114485853969 16	CD14/CLEC7A/IL1B/BCL3/WNT5A/ISG15	6
BP	GO:0110148	biomineralization	10/418	181/18903	0.007147602810075 57	0.04279969554 03938	0.03114485853969 16	ECM1/ODAPH/FAM20C/CEBPB/ADRB2/BMP2/ODAM/ISG15/PTGS2/HIF1A	10
BP	GO:0002446	neutrophil mediated immunity	4/418	35/18903	0.007178987833205 64	0.04279969554 03938	0.03114485853969 16	CXCL6/CD177/DNASE1L3/ANXA3	4
BP	GO:0035633	maintenance of blood-brain barrier	4/418	35/18903	0.007178987833205 64	0.04279969554 03938	0.03114485853969 16	CLDN1/VEGFA/GJB6/PTGS2	4
BP	GO:0060914	heart formation	4/418	35/18903	0.007178987833205 64	0.04279969554 03938	0.03114485853969 16	WNT5A/EMP2/BMP2/PIM1	4

BP	GO:1901658	glycosyl compound catabolic process	4/418	35/18903	0.00717898783320564	0.0427996955403938	0.0311448585396916	APOBEC3A/FUCA1/CDA/UPP1	4
BP	GO:2000406	positive regulation of T cell migration	4/418	35/18903	0.00717898783320564	0.0427996955403938	0.0311448585396916	S100A7/CCL20/WNT5A/SELENOK	4
BP	GO:0008360	regulation of cell shape	9/418	154/18903	0.00736834488055521	0.0438687563052129	0.0319228020710703	ANXA1/CDC42EP1/VEGFA/DLG1/RAC1/RND3/BAMBI/EZR/SLC9A3R1	9
BP	GO:0001503	ossification	18/418	429/18903	0.00741057461895373	0.0440504312617799	0.0320550048998765	ECM1/CYP24A1/EPHA2/FAM20C/DHRS3/WNT5A/RUNX1/AREG/SORT1/CEBPB/ADRB2/BMP2/ISG15/CEBPD/BAMBI/PTGS2/HIF1A/TPM4	18
BP	GO:0046890	regulation of lipid biosynthetic process	10/418	182/18903	0.00741902000198399	0.0440504312617799	0.0320550048998765	IL1A/CEACAM1/RDH10/ANXA1/IL1B/FABP5/CAPN2/ADM/BMP2/PTGS2	10
BP	GO:0042594	response to starvation	11/418	211/18903	0.00749254720908387	0.0444266367757076	0.0323287654340389	GLUL/ZC3H12A/CDKN1A/ADM/UPP1/XBP1/ZFP36/IFI16/SH3GLB1/HSPA5/PMAIP1	11
BP	GO:00	positive regulation of kinase activity	20/418	496/18903	0.00750721002927288	0.0444532626394614	0.0323481407765417	S100A12/GPRC5A/LYN/IL1B/CD24/CIB1/TGFA/EPHA2/VEGFA/HBEGF/DLG1/WNT5A/AREG/CDKN1A/ADRB2/SDC4/EMP2/BMP2/TPD52L1/PRNP	20

	33674								
BP	GO:0060249	anatomical structure homeostasis	15/418	333/18903	0.00753948850031941	0.0445839847718482	0.0324432658065165	CLDN1/VEGFA/TNFAIP3/HSPB1/B2M/CA2/CDH3/TFRC/ADRB2/OAS1/IGKC/IGHG3/GJB6/PTGS2/HIF1A	15
BP	GO:0034329	cell junction assembly	18/418	430/18903	0.00758188646640953	0.0447741133219049	0.0325816202205166	CLDN1/GJB2/ADGRF1/IL1B/CLDN4/EPHA2/VEGFA/DLG1/WNT5A/CLDN7/AFDN/S100A10/SDC4/RAC1/HOPX/PKN2/GJB6/CTNND1	18
BP	GO:0090049	regulation of cell migration involved in sprouting angiogenesis	6/418	78/18903	0.00759549120496239	0.0447939224908038	0.03259603513060 78	ANXA1/HMOX1/CIB1/VEGFA/PLK2/PTGS2	6
BP	GO:0043370	regulation of CD4-positive, alpha-beta T cell differentiation	5/418	56/18903	0.00782207447402145	0.0459899780692655	0.03346639136390 49	ANXA1/NFKBIZ/IL4R/RUNX1/ZC3H12A	5
BP	GO:0071715	icosanoid transport	5/418	56/18903	0.00782207447402145	0.0459899780692655	0.0334663913639049	IL1A/ANXA1/IL1B/PNPLA8/PTGS2	5
BP	GO:00	positive regulation of cellular	20/418	498/18903	0.00782987269646076	0.0459899780692655	0.0334663913639049	CLDN1/CLEC7A/CSF3/PLEK/EPHA2/LGALS3/CDC42EP1/VEGFA/CD47/DLG1/TFRC/S100A10/SDC4/RAC1/CLIP1/ISG15/ID1/HOPX/SH3GLB1/VPS4B	20

	44089	component biogenesis							
BP	GO:0002504	antigen processing and presentation of peptide or polysaccharide antigen via MHC class II	4/418	36/18903	0.00793809471723 57	0.04620988726 04511	0.03362641681652 97	CTSL/CTSD/B2M/LGMN	4
BP	GO:0033280	response to vitamin D	4/418	36/18903	0.00793809471723 57	0.04620988726 04511	0.03362641681652 97	AQP3/CYP24A1/PIM1/PTGS2	4
BP	GO:0048536	spleen development	4/418	36/18903	0.00793809471723 57	0.04620988726 04511	0.03362641681652 97	BCL3/PITX2/CITED2/CDKN2B	4
BP	GO:0050869	negative regulation of B cell activation	4/418	36/18903	0.00793809471723 57	0.04620988726 04511	0.03362641681652 97	LYN/TNFAIP3/TNFRSF21/ID2	4
BP	GO:0071214	cellular response to abiotic stimulus	15/418	335/18903	0.00794421482866 839	0.04620988726 04511	0.03362641681652 97	IL1B/MME/NET1/CDKN1A/N4BP1/MDM2/MTPN/PTGS2/ERRFI1/AKR1B1/IFI16/GADD45A/HSPA5/IRF1/CRYAB	15

BP	GO:0104004	cellular response to environmental stimulus	15/418	335/18903	0.00794421482866839	0.0462098872604511	0.03362641681652 97	IL1B/MME/NET1/CDKN1A/N4BP1/MDM2/MTPN/PTGS2/ERRFI1/AKR1B1/IFI16/GADD45A/HSPA5/IRF1/CRYAB	15
BP	GO:1902107	positive regulation of leukocyte differentiation	10/418	184/18903	0.00798569329169237	0.0462098872604511	0.03362641681652 97	VNN1/ANXA1/NFKBIZ/IL4R/RUNX1/CD46/TRIB1/DUSP10/XBP1/ID2	10
BP	GO:1903708	positive regulation of hemopoiesis	10/418	184/18903	0.00798569329169237	0.0462098872604511	0.03362641681652 97	VNN1/ANXA1/NFKBIZ/IL4R/RUNX1/CD46/TRIB1/DUSP10/XBP1/ID2	10
BP	GO:0002283	neutrophil activation involved in immune response	3/418	19/18903	0.00799420475260894	0.0462098872604511	0.03362641681652 97	CD177/DNASE1L3/ANXA3	3
BP	GO:0042574	retinal metabolic process	3/418	19/18903	0.00799420475260894	0.0462098872604511	0.03362641681652 97	SDR16C5/RDH10/ALDH1A3	3
BP	GO:1902176	negative regulation of oxidative stress-induced intrinsic apoptotic	3/418	19/18903	0.00799420475260894	0.0462098872604511	0.03362641681652 97	SOD2/HSPB1/HIF1A	3

		signaling pathway							
BP	GO:2001185	regulation of CD8-positive, alpha-beta T cell activation	3/418	19/18903	0.00799420475260894	0.0462098872604511	0.03362641681652 97	RUNX1/HLA-E/IRF1	3
BP	GO:0002292	T cell differentiation involved in immune response	6/418	79/18903	0.00807068047350407	0.0465288570833942	0.0338585275801358	ANXA1/BCL3/NFKBIZ/IL4R/ZC3H12A/CD46	6
BP	GO:0002437	inflammatory response to antigenic stimulus	6/418	79/18903	0.00807068047350407	0.0465288570833942	0.0338585275801358	IL36G/IL36A/LYN/IL1RN/HLA-E/PLK2	6
BP	GO:0090316	positive regulation of intracellular protein transport	9/418	157/18903	0.00831660380885538	0.0478834764752279	0.0348442689224771	IL1B/CIB1/ZC3H12A/STOM/PTGS2/MFF/SH3GLB1/SFN/PRNP	9
BP	GO:0032874	positive regulation of stress-activated MAPK cascade	8/418	130/18903	0.00835743438037786	0.0479921001869268	0.0349233131795028	IL1A/IL1B/VEGFA/WNT5A/ZC3H12A/BMP2/TPD52L1/GADD45A	8

BP	GO:0045621	positive regulation of lymphocyte differentiation	8/418	130/18903	0.00835743438037786	0.0479921001869268	0.0349233131795028	VNN1/ANXA1/NFKBIZ/IL4R/RUNX1/CD46/DUSP10/XBP1	8
BP	GO:0002823	negative regulation of adaptive immune response based on somatic recombination of immune receptors built from immunoglobulin superfamily domains	5/418	57/18903	0.00842234213795468	0.0481749151084581	0.0350563455480313	CEACAM1/CD55/IL4R/ZC3H12A/CD46	5
BP	GO:0032768	regulation of monooxygenase activity	5/418	57/18903	0.00842234213795468	0.0481749151084581	0.0350563455480313	IL1A/IL1B/CDH3/HM13/HIF1A	5
BP	GO:0070231	T cell apoptotic process	5/418	57/18903	0.00842234213795468	0.0481749151084581	0.0350563455480313	ST3GAL1/BCL3/LGALS3/WNT5A/HIF1A	5

BP	GO:0001894	tissue homeostasis	13/418	275/18903	0.00850016592616242	0.0485565033952023	0.03533402306561 63	CLDN1/VEGFA/TNFAIP3/HSPB1/B2M/CDH3/TFRC/ADRB2/OAS1/IGKC/IGHG3/GJB6/PTGS2	13
BP	GO:0003151	outflow tract morphogenesis	6/418	80/18903	0.00856687394717 39	0.0488736803513707	0.0355648291801736	VEGFA/PITX2/DHRS3/WNT5A/CITED2/HIF1A	6
BP	GO:0015698	inorganic anion transport	10/418	186/18903	0.00858513932538681	0.0489140271863629	0.0355941891195959	CLCA4/SLC6A14/CLIC3/CLDN4/SLC12A8/CLCA2/CA2/CEBPB/FXYD3/PRNP	10
BP	GO:0008207	C21-steroid hormone metabolic process	4/418	37/18903	0.00874803989314208	0.0494495337597708	0.0359838712485289	DHRS9/ADM/BMP2/AKR1B1	4
BP	GO:0031076	embryonic camera-type eye development	4/418	37/18903	0.00874803989314208	0.0494495337597708	0.0359838712485289	RDH10/ALDH1A3/WNT5A/CITED2	4
BP	GO:0044319	wound healing, spreading of cells	4/418	37/18903	0.00874803989314208	0.0494495337597708	0.0359838712485289	CEACAM1/LRG1/HBEGF/MMP12	4
BP	GO:00	'de novo' post-translational protein folding	4/418	37/18903	0.00874803989314208	0.0494495337597708	0.0359838712485289	ERO1A/HSPH1/HSPA6/HSPA5	4

	51084								
BP	GO:0090505	epiboly involved in wound healing	4/418	37/18903	0.00874803989314208	0.0494495337597708	0.0359838712485289	CEACAM1/LRG1/HBEGF/MMP12	4
BP	GO:0032479	regulation of type I interferon production	7/418	105/18903	0.00875833847369854	0.0494495337597708	0.0359838712485289	CD14/CYLD/MMP12/ISG15/OAS1/RIOK3/IRF1	7
BP	GO:0032606	type I interferon production	7/418	105/18903	0.00875833847369854	0.0494495337597708	0.0359838712485289	CD14/CYLD/MMP12/ISG15/OAS1/RIOK3/IRF1	7
BP	GO:0062012	regulation of small molecule metabolic process	15/418	339/18903	0.00880560251148834	0.0496522360970375	0.0361313754664941	FMO2/CEACAM1/RDH10/ANXA1/IL1B/PLEK/CDA/FABP5/ADIPOR1/ADM/BMP2/TMSB4X/PTGS2/HIF1A/PMAIP1	15
CC	GO:0001533	cornified envelope	21/432	59/19869	1.81623510959442e-20	7.11964162961014e-18	5.46782359309479e-18	SPRR2A/SPRR2F/SPRR2E/SPRR2D/CST6/SPRR3/ANXA1/TGM1/KRT16/SPRR1B/SERPINB2/HSPB1/IVL/CSTA/CNFN/ANXA2/PI3/SCEL/SPRR1A/PPL/KLK7	21
CC	GO:0070820	tertiary granule	22/432	164/19869	9.85614809595293e-12	1.93180502680677e-09	1.48360966075923e-09	CXCL1/CEACAM1/LRG1/PRSS3/QPCT/CSTB/CD177/LGALS3/CD47/CD55/CDA/CTSD/CD59/PLAU/B2M/VAMP8/LTA4H/STOM/RAC1/YPEL5/RAP2B/CST3	22

CC	GO:0034774	secretory granule lumen	30/432	322/19869	2.32781823838082e-11	2.68086229304051e-09	2.05887920464443e-09	S100A7/S100A8/S100A9/CXCL1/ECM1/S100A12/LRG1/LCN2/SLPI/SERPINB1/QPCT/SERPINB3/CSTB/VEGFA/FUCA1/CDA/GRN/FABP5/CTSC/CTSD/B2M/PDXK/ANXA2/PYGL/S100P/S100A11/GM2A/HSPA6/TMSB4X/NPC2	30
CC	GO:0060205	cytoplasmic vesicle lumen	30/432	325/19869	2.93469115400143e-11	2.68086229304051e-09	2.05887920464443e-09	S100A7/S100A8/S100A9/CXCL1/ECM1/S100A12/LRG1/LCN2/SLPI/SERPINB1/QPCT/SERPINB3/CSTB/VEGFA/FUCA1/CDA/GRN/FABP5/CTSC/CTSD/B2M/PDXK/ANXA2/PYGL/S100P/S100A11/GM2A/HSPA6/TMSB4X/NPC2	30
CC	GO:0031983	vesicle lumen	30/432	327/19869	3.41946721051085e-11	2.68086229304051e-09	2.05887920464443e-09	S100A7/S100A8/S100A9/CXCL1/ECM1/S100A12/LRG1/LCN2/SLPI/SERPINB1/QPCT/SERPINB3/CSTB/VEGFA/FUCA1/CDA/GRN/FABP5/CTSC/CTSD/B2M/PDXK/ANXA2/PYGL/S100P/S100A11/GM2A/HSPA6/TMSB4X/NPC2	30
CC	GO:0042581	specific granule	20/432	160/19869	3.16795677568078e-10	2.06973176011145e-08	1.5895362067451e-08	CXCL1/PLAUR/CEACAM1/LRG1/LCN2/SLPI/QPCT/CD177/CD47/CTSD/CD59/PLAU/B2M/PDXK/VAMP8/ANXA11/STOM/ANXA3/RAP2B/ATP6V1D	20
CC	GO:0030667	secretory granule membrane	26/432	313/19869	5.92045575911352e-09	3.31545522510357e-07	2.54624112346837e-07	PLAUR/CEACAM1/VNN1/RAB31/CD14/MME/CD177/LGALS3/CD47/CD55/CEACAM6/FABP5/CD59/PLAU/CYB5R1/CD46/RAB27B/VAMP8/STOM/RAC1/HLA-C/RAB10/HLA-B/RAP2B/APLP2/ATP6V1D	26
CC	GO:1904724	tertiary granule lumen	11/432	55/19869	2.28848551812017e-08	1.12135790387888e-06	8.61193234450485e-07	CXCL1/LRG1/PRSS3/QPCT/CSTB/CDA/CTSD/B2M/LTA4H/YPEL5/CST3	11
CC	GO:00	membrane raft	25/432	326/19869	5.6559987478146e-08	2.13927509183171e-06	1.64294488792661e-06	LYN/CD14/HMOX1/CD24/MME/CD177/MAL/EFHD2/CD55/DLG1/CTSD/HAS2/PLSCR1/CAPN2/ANXA2/S100A10/SDC4/EMP2/STOM/EZR/RAP2B/SLC9A3R1/PTGS2/ATP1B1/PRNP	25

	451<br>21								
CC	GO<br>:00<br>057<br>75	vacuolar<br>lumen	18/432	176/198<br>69	5.934739<br>77590538<br>e-08	2.13927<br>509183<br>171e-06	1.642944<br>88792661<br>e-06	S100A7/CTSL/CSF3/SERPINB3/FUCA1/GRN/FABP5/CTSC/CTSD/ANXA2/GM2<br>A/SDC4/CTSB/DCN/LUM/LGMN/SERPINB13/NPC2	18
CC	GO<br>:00<br>988<br>57	membrane<br>microdomain	25/432	327/198<br>69	6.003067<br>85973183<br>e-08	2.13927<br>509183<br>171e-06	1.642944<br>88792661<br>e-06	LYN/CD14/HMOX1/CD24/MME/CD177/MAL/EFHD2/CD55/DLG1/CTSD/HAS2/<br>PLSCR1/CAPN2/ANXA2/S100A10/SDC4/EMP2/STOM/EZR/RAP2B/SLC9A3R1/<br>PTGS2/ATP1B1/PRNP	25
CC	GO<br>:00<br>451<br>77	apical part of<br>cell	29/432	435/198<br>69	1.038582<br>94623409<br>e-07	3.39270<br>429103<br>135e-06	2.605567<br>74230657<br>e-06	RHCG/CLCA4/CEACAM1/CLDN1/SLC5A1/PLAT/SLC6A14/DUOX2/ANXA1/C<br>TSL/SLC52A3/CIB1/CLDN4/MUC1/MAL/DLG1/CEACAM6/CA2/RAB27B/CEA<br>CAM5/ADRB2/EMP2/CTSB/LMO7/EZR/SLC9A3R1/GJB6/LGMN/ATP1B1	29
CC	GO<br>:00<br>163<br>24	apical plasma<br>membrane	26/432	368/198<br>69	1.563038<br>24531597<br>e-07	4.71316<br>147818<br>355e-06	3.619667<br>51546857<br>e-06	RHCG/CLCA4/CEACAM1/CLDN1/SLC5A1/SLC6A14/DUOX2/ANXA1/CTSL/S<br>LC52A3/CIB1/CLDN4/MUC1/MAL/DLG1/CEACAM6/RAB27B/CEACAM5/ADR<br>B2/EMP2/CTSB/LMO7/EZR/SLC9A3R1/GJB6/ATP1B1	26
CC	GO<br>:00<br>306<br>66	endocytic<br>vesicle<br>membrane	18/432	194/198<br>69	2.609279<br>58622687<br>e-07	7.30598<br>284143<br>524e-06	5.610932<br>04256305<br>e-06	LYN/RAB31/TGFA/HBEGF/RAB11FIP1/WNT5A/AREG/B2M/TFRC/VAMP8/AD<br>RB2/ANXA3/MDM2/HLA-C/RAB10/HLA-B/HLA-E/UBC	18
CC	GO<br>:00<br>059<br>12	adherens<br>junction	17/432	179/198<br>69	4.028634<br>47565158<br>e-07	1.05281<br>647630<br>361e-05	8.085540<br>07043054<br>e-06	CEACAM1/LYN/ANXA1/ALOX15B/CDC42EP1/VEGFA/DLG1/CNN3/CDH3/AF<br>DN/ANXA2/S100A11/NDRG1/RAB10/NECTIN4/EZR/CTNND1	17

CC	GO:0030139	endocytic vesicle	24/432	343/19869	5.61607441995161e-07	1.37593823288815e-05	1.05670873954353e-05	SAA1/LYN/RAB31/CTSL/CSF3/TGFA/HBEGF/RAB11FIP1/WNT5A/AREG/B2M/TFRC/VAMP8/ADRB2/ANXA11/ANXA3/MDM2/HSPH1/HLA-C/RAB10/HLA-B/HLA-E/CLTB/UBC	24
CC	GO:0043202	lysosomal lumen	12/432	98/19869	1.43899452145881e-06	3.31815207301089e-05	2.54831227948742e-05	CTSL/CSF3/FUCA1/CTSD/GM2A/SDC4/CTSB/DCN/LUM/LGMN/SERPINB13/NPC2	12
CC	GO:1905286	serine-type peptidase complex	5/432	13/19869	5.29118056060568e-06	0.000115230154430968	8.84957684405394e-05	PLAUR/CFH/PLAU/THBD/F3	5
CC	GO:0005766	primary lysosome	14/432	155/19869	7.63407117066561e-06	0.000149627794945046	0.000114912860779493	S100A7/VNN1/SERPINB3/FUCA1/GRN/CEACAM6/FABP5/CTSC/VAMP8/ANXA2/GM2A/ANXA11/STOM/NPC2	14
CC	GO:0042582	azurophil granule	14/432	155/19869	7.63407117066561e-06	0.000149627794945046	0.000114912860779493	S100A7/VNN1/SERPINB3/FUCA1/GRN/CEACAM6/FABP5/CTSC/VAMP8/ANXA2/GM2A/ANXA11/STOM/NPC2	14
CC	GO:0016328	lateral plasma membrane	9/432	64/19869	9.54620236124988e-06	0.000178195777409998	0.000136852825830449	CEACAM1/CLDN1/GJB2/TACSTD2/ANXA1/CLDN4/DLG1/CLDN7/ATP1B1	9
CC	GO:00	collagen-containing	25/432	433/19869	1.02184062845008e-05	0.00018207342	0.000139830822840537	S100A7/S100A8/S100A9/ECM1/SLPI/ANXA1/SERPINB1/CTSL/CSTB/LGALS3/DLG1/WNT5A/CTSC/CTSD/PLSCR1/S100A6/LAMC2/ANXA2/F3/S100A10/ANXA11/CTSB/DCN/S100A4/LUM	25

	62023	extracellular matrix				1069287			
CC	GO:0005925	focal adhesion	24/432	422/19869	1.96452017226144e-05	0.000334511543704145	0.00025690199113691	S100A7/PLAUR/ANXA1/MME/EPHA2/ALOX15B/CDC42EP1/CD59/CAPN2/PLAU/HSPB1/CNN3/B2M/CD46/SDC4/ZNF185/RAC1/RND3/RAB10/LMO7/EZR/TPM4/ARPC3/HSPA5	24
CC	GO:0031225	anchored component of membrane	14/432	169/19869	2.04802985941313e-05	0.000334511543704145	0.00025690199113691	PLAUR/VNN1/CD14/LY6D/CD24/LYPD3/CD177/CD55/PSCA/CEACAM6/CD59/RAB27B/CEACAM5/PRNP	14
CC	GO:0035579	specific granule membrane	10/432	91/19869	2.84396574297939e-05	0.000432384697339053	0.000332067732113237	PLAUR/CEACAM1/CD177/CD47/CD59/PLAU/VAMP8/STOM/RAP2B/ATP6V1D	10
CC	GO:0030055	cell-substrate junction	24/432	432/19869	2.8678576864325e-05	0.000432384697339053	0.000332067732113237	S100A7/PLAUR/ANXA1/MME/EPHA2/ALOX15B/CDC42EP1/CD59/CAPN2/PLAU/HSPB1/CNN3/B2M/CD46/SDC4/ZNF185/RAC1/RND3/RAB10/LMO7/EZR/TPM4/ARPC3/HSPA5	24
CC	GO:0030670	phagocytic vesicle membrane	9/432	77/19869	4.35944526914545e-05	0.000632926868705561	0.000486082396481714	RAB31/RAB11FIP1/B2M/VAMP8/ANXA3/HLA-C/RAB10/HLA-B/HLA-E	9
CC	GO:0055038	recycling endosome membrane	10/432	97/19869	4.95737035404839e-05	0.000694031849566774	0.000533010496713473	B2M/TFRC/VAMP8/RAC1/NDRG1/HLA-C/RAB10/HLA-B/HLA-E/RAP2B	10

CC	GO:0101002	ficolin-1-rich granule	14/432	185/19869	5.57410155517735e-05	0.000739968344157546	0.0005682893298309830983	LRG1/QPCT/CSTB/LGALS3/CD55/CDA/CTSD/PYGL/LTA4H/CTSB/RAC1/YPEL5/HSPA6/CST3	14
CC	GO:0035580	specific granule lumen	8/432	62/19869	5.66302304202203e-05	0.000739968344157546	0.000568289329830983	CXCL1/LRG1/LCN2/SLPI/QPCT/CTSD/B2M/PDXK	8
CC	GO:1904090	peptidase inhibitor complex	4/432	11/19869	6.44291506150832e-05	0.000789257095034770	0.000606142666970849	PLAT/PLAU/CSTA/CTSB	4
CC	GO:1905370	serine-type endopeptidase complex	4/432	11/19869	6.44291506150832e-05	0.000789257095034770	0.000606142666970849	PLAUR/CFH/PLAU/THBD	4
CC	GO:1904813	ficolin-1-rich granule lumen	11/432	124/19869	8.44864173285806e-05	0.00100359623008496	0.000770753280892315	LRG1/QPCT/CSTB/CDA/CTSD/PYGL/LTA4H/CTSB/YPEL5/HSPA6/CST3	11
CC	GO:0030662	coated vesicle membrane	14/432	198/19869	0.000115630377348192	0.00133315023295563	0.00102384792326882	TGFA/HBEGF/WNT5A/AREG/CD59/B2M/TFRC/VAMP8/ADRB2/HLA-C/HLA-B/HLA-E/CLTB/ATP6V1D	14
CC	GO:00	intrinsic component of external side	5/432	24/19869	0.000143419241636545	0.00160629550632931	0.00123362114610683	CD14/CD24/CD59/F3/PRNP	5

	31233	of plasma membrane							
CC	GO:0009925	basal plasma membrane	16/432	254/19869	0.0001495943560755513	0.00162891632171114	0.00125099373794143	RHCG/AQP3/CEACAM1/CLDN1/TACSTD2/ANXA1/TGFA/CLDN4/DLG1/CLCA2/CLDN7/TFRC/CEACAM5/ANXA2/EZR/ATP1B1	16
CC	GO:0035578	azurophil granule lumen	9/432	91/19869	0.000162107712805308	0.00171746549782921	0.00131899874430492	S100A7/SERPINB3/FUCA1/GRN/FABP5/CTSC/ANXA2/GM2A/NPC2	9
CC	GO:0070821	tertiary granule membrane	8/432	73/19869	0.000182869032951841	0.00188643844518741	0.00144876851590655	CEACAM1/CD177/CD47/CD59/PLAU/VAMP8/STOM/RAP2B	8
CC	GO:0019898	extrinsic component of membrane	18/432	316/19869	0.000208240665388502	0.00209308566236648	0.00160747180299896	PLAUR/LYN/ANXA1/ALOX15B/MAL/ZC3H12A/S100A6/GNA15/CYLD/CDH3/ANXA2/S100A10/RAC1/EZR/ERRFI1/CTNND1/UBC/PRNP	18
CC	GO:0045178	basal part of cell	16/432	272/19869	0.000324272105452562	0.00317786663343511	0.00244057426735349	RHCG/AQP3/CEACAM1/CLDN1/TACSTD2/ANXA1/TGFA/CLDN4/DLG1/CLCA2/CLDN7/TFRC/CEACAM5/ANXA2/EZR/ATP1B1	16
CC	GO:0012507	ER to Golgi transport vesicle membrane	7/432	63/19869	0.00042385236589168	0.00405244213242777	0.00311224073543057	TGFA/AREG/CD59/B2M/HLA-C/HLA-B/HLA-E	7

CC	GO:0030135	coated vesicle	17/432	310/19869	0.000479449000268507	0.0044748573358394	0.00343665198187451	TGFA/HBEGF/WNT5A/CTSC/AREG/CD59/SORT1/B2M/RAB27B/TFRC/VAMP8/ADRB2/HLA-C/HLA-B/HLA-E/CLTB/ATP6V1D	17
CC	GO:0042470	melanosome	9/432	110/19869	0.000668982885666456	0.00596002934502842	0.00457725132298101	CTSD/RAB27B/TFRC/ANXA2/ANXA11/CTSB/STOM/RAC1/HSPA5	9
CC	GO:0048770	pigment granule	9/432	110/19869	0.000668982885666456	0.00596002934502842	0.00457725132298101	CTSD/RAB27B/TFRC/ANXA2/ANXA11/CTSB/STOM/RAC1/HSPA5	9
CC	GO:0045334	clathrin-coated endocytic vesicle	8/432	90/19869	0.000765578242199291	0.00666903713204715	0.00512176321097069	TGFA/HBEGF/WNT5A/AREG/TFRC/VAMP8/ADRB2/CLTB	8
CC	GO:0031362	anchored component of external side of plasma membrane	4/432	20/19869	0.000809907053358666	0.00690181662862167	0.00530053586408646	CD14/CD24/CD59/PRNP	4
CC	GO:0045335	phagocytic vesicle	10/432	139/19869	0.000937680223555275	0.00779806733962904	0.00598884870873766	RAB31/RAB11FIP1/B2M/VAMP8/ANXA11/ANXA3/HLA-C/RAB10/HLA-B/HLA-E	10

CC	GO:0030669	clathrin-coated endocytic vesicle membrane	7/432	72/19869	0.0009548653885260005	0.00779806733962904	0.00598884870873766	TGFA/HBEGF/WNT5A/AREG/TFRC/VAMP8/ADRB2	7
CC	GO:0016327	apicolateral plasma membrane	4/432	21/19869	0.0009834250536531740	0.00786740042922539	0.0060420959257746	CLDN4/THBD/CLDN7/KRT19	4
CC	GO:0030134	COPII-coated ER to Golgi transport vesicle	8/432	95/19869	0.00109289710547169	0.00840202592236307	0.00645268371051514	TGFA/CTSC/AREG/CD59/B2M/HLA-C/HLA-B/HLA-E	8
CC	GO:0071735	IgG immunoglobulin complex	3/432	10/19869	0.00109312071949111	0.00840202592236307	0.00645268371051514	IGHG4/IGKC/IGHG3	3
CC	GO:0019897	extrinsic component of plasma membrane	11/432	172/19869	0.00139421418306474	0.0103983430332947	0.00798583809753567	LYN/ANXA1/S100A6/GNA15/CYLD/CDH3/ANXA2/S100A10/RAC1/ERRFI1/CTNND1	11
CC	GO:0071682	endocytic vesicle lumen	4/432	23/19869	0.0014058984203179	0.0103983430332947	0.00798583809753567	SAA1/CTSL/CSF3/HSPH1	4
CC	GO:00	ruffle	11/432	178/19869	0.00183220909279177	0.0133004808217477	0.0102146549812563	ADGRE2/CIB1/PLEK/EPHA2/S100A6/S100A11/RAC1/CLIP1/EZR/SLC9A3R1/ARF4	11

	01726								
CC	GO:0042611	MHC protein complex	4/432	25/19869	0.00194073736615697	0.0138321645006097	0.0106229834779118	B2M/HLA-C/HLA-B/HLA-E	4
CC	GO:0046658	anchored component of plasma membrane	6/432	62/19869	0.00224468260780526	0.0157127782546368	0.01206727868105584	CD14/CD24/LYPD3/CD177/CD59/PRNP	6
CC	GO:0030665	clathrin-coated vesicle membrane	9/432	132/19869	0.00240967058129 41	0.0165717696117068	0.0127269766620 52	TGFA/HBEGF/WNT5A/AREG/TFRC/VAMP8/ADRB2/CLTB/ATP6V1D	9
CC	GO:0031528	microvillus membrane	4/432	28/19869	0.00298407150471	0.0201682074111434	0.0154890099881499	CEACAM1/S100P/EZR/SLC9A3R1	4
CC	GO:0071556	integral component of lumenal side of endoplasmic reticulum membrane	4/432	29/19869	0.00340289386900771	0.0222322399441837	0.0170741692374773	HLA-C/HLA-B/HLA-E/HM13	4

CC	GO:0098553	lumenal side of endoplasmic reticulum membrane	4/432	29/19869	0.00340289386900771	0.0222322399441837	0.01707416923774773	HLA-C/HLA-B/HLA-E/HM13	4
CC	GO:0055037	recycling endosome	11/432	198/19869	0.00416259886432886	0.0267498156527363	0.02054362856252 04	RAB11FIP1/B2M/TFRC/VAMP8/RAC1/NDRG1/HLA-C/RAB10/HLA-B/HLA-E/RAP2B	11
CC	GO:0016323	basolateral plasma membrane	12/432	229/19869	0.00451250740363949	0.0285306919713981	0.0219113262723412	RHCG/AQP3/CLDN1/ANXA1/TGFA/DLG1/CLDN7/TFRC/CEACAM5/ANXA2/EZR/ATP1B1	12
CC	GO:0032587	ruffle membrane	7/432	97/19869	0.00524929710343493	0.0326622930880396	0.0250843604274418	ADGRE2/CIB1/PLEK/EPHA2/RAC1/EZR/ARF4	7
CC	GO:0031968	organelle outer membrane	12/432	235/19869	0.00552655581953394	0.0338501543946454	0.0259966277037287	HMOX1/BCL2A1/ASS1/RSAD2/PPP1R15A/MAOA/IFI27/PTGS2/MFF/SH3GLB1/UBC/PMAIP1	12
CC	GO:0005741	mitochondrial outer membrane	11/432	207/19869	0.00578751181365452	0.0349031481685011	0.0268053178737683	HMOX1/BCL2A1/ASS1/RSAD2/PPP1R15A/MAOA/IFI27/MFF/SH3GLB1/UBC/PMAIP1	11

CC	GO:0019867	outer membrane	12/432	237/19869	0.00590215412369 75	0.03505521843 16579	0.02692210652914 65	HMOX1/BCL2A1/ASS1/RSAD2/PPP1R15A/MAOA/IFI27/PTGS2/MFF/SH3GLB1/UBC/PMAIP1	12
CC	GO:0030136	clathrin-coated vesicle	11/432	211/19869	0.00665348560252 324	0.03847801011 72954	0.02955078113197 23	TGFA/HBEGF/WNT5A/AREG/SORT1/RAB27B/TFRC/VAMP8/ADRB2/CLTB/ATP6V1D	11
CC	GO:0031904	endosome lumen	4/432	35/19869	0.00677291504615 66	0.03847801011 72954	0.02955078113197 23	CTSL/B2M/CTSB/LGMN	4
CC	GO:0098576	lumenal side of membrane	4/432	35/19869	0.00677291504615 66	0.03847801011 72954	0.02955078113197 23	HLA-C/HLA-B/HLA-E/HM13	4
CC	GO:0005811	lipid droplet	7/432	102/19869	0.00688441307275 22	0.03855271320 74123	0.02960815246326 51	SDR16C5/RDH10/DHRS3/RSAD2/FABP4/ANXA2/RBP1	7
CC	GO:0033116	endoplasmic reticulum-Golgi intermediate compartment membrane	6/432	80/19869	0.00792494308818 333	0.04375461536 01108	0.03360316861705 61	TGFA/GALNT1/CD55/CTSC/AREG/CD59	6

CC	GO:0005796	Golgi lumen	7/432	106/19869	0.00844309290109614	0.0459222394294883	0.03526788527613 76	HS3ST1/ERO1A/MUC1/WNT5A/SDC4/DCN/LUM	7
CC	GO:0031253	cell projection membrane	15/432	344/19869	0.00866752714820682	0.0459222394294883	0.0352678852761376	CEACAM1/SLC5A1/GPR157/ADGRE2/CIB1/PLEK/EPHA2/DLG1/DHRS3/S100P/RAC1/EZR/SLC9A3R1/CLTB/ARF4	15
CC	GO:0005793	endoplasmic reticulum-Golgi intermediate compartment	8/432	133/19869	0.00866899417801564	0.0459222394294883	0.0352678852761376	GJB2/TGFA/GALNT1/CD55/CTSC/AREG/CD59/HSPA5	8
CC	GO:1905369	endopeptidase complex	6/432	82/19869	0.00890826711307079	0.04656054277765	0.035758096762642	PLAUR/CFH/CAPN2/PLAU/HSPB1/THBD	6
MF	GO:0048306	calcium-dependent protein binding	20/429	81/18432	1.78625142667625e-15	1.09854462740589e-12	9.45773123808582e-13	S100A7/S100A8/S100A9/CRNN/S100A12/ANXA1/CD177/S100A6/S100A7A/S100A14/S100A13/ANXA2/S100P/S100A11/S100A10/ANXA11/ANXA3/S100A2/S100A4/S100A16	20
MF	GO:0050786	RAGE receptor binding	6/429	10/18432	2.97938921722619e-08	9.16162184297055e-06	7.88754092770935e-06	S100A7/S100A8/S100A9/S100A12/S100A13/S100A4	6

MF	GO:0061134	peptidase regulator activity	21/429	232/18432	1.24700899895473e-07	2.55636844785719e-05	2.2008614964008e-05	SERPINB4/PRSS22/CST6/SLPI/SERPINB1/SERPINB3/CSTB/MAL/SPINK7/SERPINB2/SPINT1/CTSC/CSTA/ANXA2/PI3/APLP2/TNFAIP8/SERPINB13/CST3/SERPINB11/PRNP	21
MF	GO:0004866	endopeptidase inhibitor activity	18/429	180/18432	2.26815162634278e-07	3.48728312550203e-05	3.00231649486953e-05	SERPINB4/CST6/SLPI/SERPINB1/SERPINB3/CSTB/SPINK7/SERPINB2/SPINT1/CSTA/ANXA2/PI3/APLP2/TNFAIP8/SERPINB13/CST3/SERPINB11/PRNP	18
MF	GO:0030414	peptidase inhibitor activity	18/429	187/18432	4.01731839555287e-07	4.17403633365354e-05	3.59356487090754e-05	SERPINB4/CST6/SLPI/SERPINB1/SERPINB3/CSTB/SPINK7/SERPINB2/SPINT1/CSTA/ANXA2/PI3/APLP2/TNFAIP8/SERPINB13/CST3/SERPINB11/PRNP	18
MF	GO:0008236	serine-type peptidase activity	18/429	192/18432	5.94071938985071e-07	4.17403633365354e-05	3.59356487090754e-05	TMPRSS11E/PRSS22/PRSS27/TMPRSS2/TMPRSS11F/PRSS3/PLAT/TMPRSS11A/KLK12/KLK13/CTSC/TMPRSS11D/PLAU/F3/MMP12/SCPEP1/KLK11/KLK7	18
MF	GO:0004857	enzyme inhibitor activity	27/429	395/18432	6.51265151477031e-07	4.17403633365354e-05	3.59356487090754e-05	SERPINB4/CST6/SLPI/ANXA1/SERPINB1/CIB1/SERPINB3/CSTB/LGALS3/SPINK7/SERPINB2/SPINT1/CDKN1A/HSPB1/CSTA/TRIB1/ANXA2/CDKN2B/PI3/ANXA3/APLP2/TNFAIP8/SERPINB13/CST3/SFN/SERPINB11/PRNP	27
MF	GO:0004252	serine-type endopeptidase activity	17/429	174/18432	6.83782075997993e-07	4.17403633365354e-05	3.59356487090754e-05	TMPRSS11E/PRSS22/PRSS27/TMPRSS2/TMPRSS11F/PRSS3/PLAT/TMPRSS11A/KLK12/KLK13/CTSC/TMPRSS11D/PLAU/F3/MMP12/KLK11/KLK7	17
MF	GO:00	endopeptidase regulator activity	18/429	194/18432	6.92078437343095e-07	4.17403633365354e-05	3.59356487090754e-05	SERPINB4/CST6/SLPI/SERPINB1/SERPINB3/CSTB/SPINK7/SERPINB2/SPINT1/CSTA/ANXA2/PI3/APLP2/TNFAIP8/SERPINB13/CST3/SERPINB11/PRNP	18

	61135								
MF	GO:0005125	cytokine activity	20/429	237/18432	7.64870132197354e-07	4.17403633365354e-05	3.59356487090754e-05	CXCL1/IL36G/IL1A/CXCL6/IL36A/CXCL8/IL1B/IL1RN/CSF3/TNFSF10/CXCL3/VEGFA/CCL20/CXCL2/GRN/IL19/WNT5A/AREG/NAMPT/BMP2	20
MF	GO:0017171	serine hydrolase activity	18/429	196/18432	8.04566812174569e-07	4.17403633365354e-05	3.59356487090754e-05	TMPRSS11E/PRSS22/PRSS27/TMPRSS2/TMPRSS11F/PRSS3/PLAT/TMPRSS11A/KLK12/KLK13/CTSC/TMPRSS11D/PLAU/F3/MMP12/SCPEP1/KLK11/KLK7	18
MF	GO:0002020	protease binding	15/429	139/18432	8.76724141314417e-07	4.17403633365354e-05	3.59356487090754e-05	SERPINB4/ECM1/SERPINB3/CSTB/CD177/FAM20C/TNFAIP3/CSTA/ANXA2/F3/RIOK3/SERPINB13/CST3/UBC/PRNP	15
MF	GO:0004175	endopeptidase activity	28/429	426/18432	8.8231662337392e-07	4.17403633365354e-05	3.59356487090754e-05	TMPRSS11E/CLCA4/PRSS22/PRSS27/TMPRSS2/TMPRSS11F/PRSS3/PLAT/TMPRSS11A/CTSL/PAPPA/ADAM28/KLK12/MME/KLK13/CTSC/CTSD/CAPN2/TMPRSS11D/PLAU/CLCA2/F3/MMP12/CTSB/HM13/LGMN/KLK11/KLK7	28
MF	GO:0070851	growth factor receptor binding	15/429	140/18432	9.61360712459141e-07	4.22312027258837e-05	3.63582284486427e-05	IL36G/ECM1/IL1A/IL36A/LYN/IL1B/IL1RN/CSF3/TGFA/VEGFA/HBEGF/AREG/PLSCR1/SLC9A3R1/ARF4	15
MF	GO:0045296	cadherin binding	24/429	333/18432	1.08378683184221e-06	4.44352601055305e-05	3.82557737836232e-05	GPRC5A/ANXA1/EPHA2/CDC42EP1/EFHD2/DLG1/CNN3/FXYD5/CD46/CDH3/AFDN/ANXA2/S100P/S100A11/NDRG1/RAB10/EZR/PKN2/SH3GLB1/PPL/CTNND1/HSPA5/BZW1/SFN	24

MF	GO:0098641	cadherin binding involved in cell-cell adhesion	6/429	18/18432	2.24765447426078e-06	8.63942188543986e-05	7.43796184574454e-05	ANXA1/CDC42EP1/CNN3/ANXA2/S100A11/RAB10	6
MF	GO:0004867	serine-type endopeptidase inhibitor activity	12/429	98/18432	2.88863514519063e-06	0.000104500624370132	8.99680172155348e-05	SERPINB4/SLPI/SERPINB1/SERPINB3/SPINK7/SERPINB2/SPINT1/ANXA2/PI3/APLP2/SERPINB13/SERPINB11	12
MF	GO:0044548	S100 protein binding	5/429	14/18432	1.12317588590826e-05	0.000383751761018656	0.000330384485737927	S100A6/ANXA2/S100A11/ANXA11/EZR	5
MF	GO:0030280	structural constituent of skin epidermis	7/429	37/18432	1.98299605966461e-05	0.000641864514049333	0.00055260222604504	SPRR2E/KRT80/PI3/KRT4/KRT6C/SPRR1A/KRT5	7
MF	GO:0005126	cytokine receptor binding	19/429	273/18432	2.33594956943022e-05	0.000718304492599791	0.000618411912328104	CXCL1/IL36G/ECM1/IL1A/CXCL6/IL36A/LRG1/CXCL8/IL1B/IL1RN/CSF3/TNFSF10/CXCL3/VEGFA/IL13RA1/CCL20/CXCL2/S100A14/BAMBI	19
MF	GO:0005149	interleukin-1 receptor binding	5/429	17/18432	3.2767404943339e-05	0.000959616859054927	0.000826165648446609	IL36G/IL1A/IL36A/IL1B/IL1RN	5

MF	GO:0045236	CXCR chemokine receptor binding	5/429	18/18432	4.4505770508369e-05	0.00124413858466577	0.00107111973998611	CXCL1/CXCL6/CXCL8/CXCL3/CXCL2	5
MF	GO:0048018	receptor ligand activity	26/429	491/18432	9.20974725643892e-05	0.00246260633161302	0.00212013861326718	CXCL1/IL36G/IL1A/CXCL6/IL36A/CXCL8/IL1B/IL1RN/CSF3/TGFA/TYMP/TNFSF10/LGALS3/CXCL3/VEGFA/HBEGF/CCL20/CXCL2/GRN/OSGIN1/IL19/WNT5A/AREG/ADM/NAMPT/BMP2	26
MF	GO:0030546	signaling receptor activator activity	26/429	498/18432	0.000115691096105176	0.00296458433769515	0.00255230795354841	CXCL1/IL36G/IL1A/CXCL6/IL36A/CXCL8/IL1B/IL1RN/CSF3/TGFA/TYMP/TNFSF10/LGALS3/CXCL3/VEGFA/HBEGF/CCL20/CXCL2/GRN/OSGIN1/IL19/WNT5A/AREG/ADM/NAMPT/BMP2	26
MF	GO:0052650	NADP-retinol dehydrogenase activity	4/429	14/18432	0.000240670040377925	0.00574192869492488	0.00494341486272523	RDH10/DHRS3/AKR1B1/AKR1B10	4
MF	GO:0098632	cell-cell adhesion mediator activity	7/429	54/18432	0.000242748204988694	0.00574192869492488	0.00494341486272523	ANXA1/CDC42EP1/CD47/CNN3/ANXA2/S100A11/RAB10	7
MF	GO:0004869	cysteine-type endopeptidase inhibitor activity	7/429	56/18432	0.000305452753222056	0.00695753493450238	0.00598997017039743	CST6/SERPINB3/CSTB/CSTA/TNFAIP8/SERPINB13/CST3	7
MF	GO:00	virus receptor activity	8/429	77/18432	0.000417146370370178	0.00916232206348785	0.00788814377053382	CLDN1/SERPINB3/EPHA2/CD55/PLSCR1/CD46/TFRC/NECTIN4	8

	01618								
MF	GO:0140272	exogenous protein binding	8/429	78/18432	0.000455538222671895	0.00966055196355915	0.00831708624333804	CLDN1/SERPINB3/EPHA2/CD55/PLSCR1/CD46/TFRC/NECTIN4	8
MF	GO:0098631	cell adhesion mediator activity	7/429	64/18432	0.000697708260751737	0.0143030193454106	0.0123139387774 78	ANXA1/CDC42EP1/CD47/CNN3/ANXA2/S100A11/RAB10	7
MF	GO:0004745	NAD-retinol dehydrogenase activity	4/429	19/18432	0.000849775350425769	0.0162534827701651	0.0139931567537 75	SDR16C5/RDH10/DHRS9/DHRS3	4
MF	GO:0005504	fatty acid binding	6/429	49/18432	0.000920476974276126	0.0162534827701651	0.0139931567537 75	S100A8/S100A9/FABP5/CRABP2/FABP4/RBP1	6
MF	GO:0008009	chemokine activity	6/429	49/18432	0.000920476974276126	0.0162534827701651	0.0139931567537 75	CXCL1/CXCL6/CXCL8/CXCL3/CCL20/CXCL2	6
MF	GO:0005154	epidermal growth factor receptor binding	5/429	33/18432	0.000924994954399637	0.0162534827701651	0.0139931567537 75	TGFA/HBEGF/AREG/PLSCR1/ARF4	5

MF	GO:0030291	protein serine/threonine kinase inhibitor activity	5/429	33/18432	0.000924994954399637	0.0162534827701651	0.0139931567537755	CIB1/CDKN1A/HSPB1/CDKN2B/SFN	5
MF	GO:0042379	chemokine receptor binding	7/429	71/18432	0.00129980679511209	0.0221081955769886	0.01903367115999919	CXCL1/CXCL6/CXCL8/CXCL3/CCL20/CXCL2/S100A14	7
MF	GO:0008330	protein tyrosine/threonine phosphatase activity	3/429	10/18432	0.00133008656316842	0.0221081955769886	0.01903367115999919	DUSP5/DUSP1/DUSP10	3
MF	GO:0005544	calcium-dependent phospholipid binding	6/429	54/18432	0.00154209801874341	0.0249576389875578	0.02148685051046091	DOC2B/ANXA1/CPNE8/ANXA2/ANXA11/ANXA3	6
MF	GO:0019887	protein kinase regulator activity	13/429	218/18432	0.00185358188156615	0.0292295604400815	0.02516468789278730	GPRC5A/CD24/CIB1/TGFA/HBEGF/AREG/CDKN1A/HSPB1/CCNL1/TRIB1/CDKN2B/BMP2/SFN	13
MF	GO:0071949	FAD binding	5/429	39/18432	0.00200177487475198	0.0307772886993117	0.02649717794737749	STEAP4/ERO1A/CYB5R1/TXNRD1/SQOR	5

MF	GO:0008106	alcohol dehydrogenase (NADP+) activity	4/429	24/18432	0.00212511690190411	0.0318767535285617	0.027443743303152	RDH10/DHRS3/AKR1B1/AKR1B10	4
MF	GO:0035325	Toll-like receptor binding	3/429	12/18432	0.00235538953013205	0.034489632405505	0.02969325648261 71	S100A8/S100A9/LY96	3
MF	GO:0140677	molecular function activator activity	6/429	59/18432	0.00244089601932166	0.0349104895786703	0.0300555862354663	EPHA2/CDKN1A/CITED2/DNAJB6/ATP1B1/PRNP	6
MF	GO:0033293	monocarboxylic acid binding	7/429	81/18432	0.00278823349408896	0.038971899974198	0.0335521877398743	S100A8/S100A9/FABP5/CRABP2/FABP4/PYGL/RBP1	7
MF	GO:0004859	phospholipase inhibitor activity	3/429	13/18432	0.00300947151547419	0.0402353256960137	0.0346399124092338	ANXA1/ANXA2/ANXA3	3
MF	GO:0017017	MAP kinase tyrosine/serine/threonine phosphatase activity	3/429	13/18432	0.00300947151547419	0.0402353256960137	0.0346399124092338	DUSP5/DUSP1/DUSP10	3

MF	GO:0038187	pattern recognition receptor activity	4/429	27/18432	0.0033221871861388	0.04347117275 47949	0.03742575934216 83	CD14/CLEC7A/PGLYRP4/LY96	4
MF	GO:0016209	antioxidant activity	7/429	85/18432	0.00365734710774027	0.0468597598179222	0.040343105157749	S100A9/DUOX2/SOD2/GPX3/TXNDC17/TXNRD1/PTGS2	7
MF	GO:0030297	transmembrane receptor protein tyrosine kinase activator activity	3/429	14/18432	0.00376459919050045	0.0472495612685261	0.0406786980198008	TGFA/HBEGF/AREG	3
MF	GO:0030145	manganese ion binding	6/429	65/18432	0.00398278771627598	0.0489882889101945	0.0421756257113014	SOD2/FAM20C/GALNT1/GLUL/PIM1/GALNT3	6
