# Supplementary material for: Multiomics Reveals IL-17 Drives Epithelial Keratinization and Proliferation via EHF in Odontogenic Keratocysts
Source: Int J Mol Sci. 2026 May 4;27(9):4115. doi: 10.3390/ijms27094115 (PMC13163638; doi:10.3390/ijms27094115)
Supplement: Supplementary file 1 [file ijms-27-04115-s001.zip › ijms-4235677-supplementary/Supplementary Table S5.pdf]

Supplementary Table S5. EpC3 GO enrichment.

ONTOLOGY	ID	Description	GeneRatio	BgRatio	pvalue	p.adjust	qvalue	geneID	Count
BP	GO:0006119	oxidative phosphorylation	48/510	147/18903	7.09066266365052e-39	2.88448157157303e-35	2.5705518119592e-35	COX6C/COX6A1/COX7B/ATP5F1E/CYCS/COX5B/NUPR1/UQCRQ/ATP5PD/COX5A/UQCR10/COX7A2/NDUFA4/NDUFB2/ATP5MF/NDUFC2/COX6B1/ATP5ME/ATP5PB/UQCR11/COX8A/NDUFS6/NDUFB9/UQCRB/NDUFB8/NDUFAB1/SDHB/ATP5PF/GHITM/UQCRC2/NDUFB3/SDHC/NDUFC1/ATP5F1B/COX4I1/NDUFA3/ATP5F1C/COX7C/CYC1/NDUFA13/NDUFA12/NDUFB1/ATP5PO/NDUFS8/ATP5MG/NDUFA1/CHCHD2/NDUFS5	48
BP	GO:0009060	aerobic respiration	53/510	194/18903	2.48842748807493e-38	5.0614615107444e-35	4.51060224680529e-35	COX6C/COX6A1/COX7B/ATP5F1E/CYCS/COX5B/NUPR1/UQCRQ/ATP5PD/COX5A/IDH1/UQCR10/COX7A2/NDUFA4/NDUFB2/ATP5MF/NDUFC2/COX6B1/ATP5ME/ATP5PB/UQCR11/COX8A/SUCLG1/NDUFS6/NDUFB9/UQCRB/NDUFB8/HIF1A/OXA1L/NDUFAB1/SDHB/ATP5PF/GHITM/UQCRC2/NDUFB3/SDHC/NDUFC1/ATP5F1B/COX4I1/NDUFA3/ATP5F1C/MDH2/COX7C/CYC1/NDUFA13/NDUFA12/NDUFB1/ATP5PO/NDUFS8/ATP5MG/NDUFA1/CHCHD2/NDUFS5	53
BP	GO:0045333	cellular respiration	53/510	240/18903	3.30340695594885e-33	4.47941983226664e-30	3.99190651097819e-30	COX6C/COX6A1/COX7B/ATP5F1E/CYCS/COX5B/NUPR1/UQCRQ/ATP5PD/COX5A/IDH1/UQCR10/COX7A2/NDUFA4/NDUFB2/ATP5MF/NDUFC2/COX6B1/ATP5ME/ATP5PB/UQCR11/COX8A/SUCLG1/NDUFS6/NDUFB9/UQCRB/NDUFB8/HIF1A/OXA1L/NDUFAB1/SDHB/ATP5PF/GHITM/UQCRC2/NDUFB3/SDHC/NDUFC1/ATP5F1B/COX4I1/NDUFA3/ATP5F1C/MDH2/COX7C/CYC1/NDUFA13/NDUFA12/NDUFB1/ATP5PO/NDUFS8/ATP5MG/NDUFA1/CHCHD2/NDUFS5	53

BP	GO:0042773	ATP synthesis coupled electron transport	36/510	101/18903	5.89694237950334e-31	4.79775231996392e-28	4.27559359052832e-28	COX6C/COX6A1/COX7B/CYCS/COX5B/UQCRQ/COX5A/UQCR10/COX7A2/NDUFA4/NDUFB2/NDUFC2/COX6B1/UQCR11/COX8A/NDUFS6/NDUFB9/UQCRB/NDUFB8/NDUFAB1/SDHB/GHITM/UQCRC2/NDUFB3/SDHC/NDUFC1/COX4I1/NDUFA3/COX7C/CYC1/NDUFA12/NDUFB1/NDUFS8/NDUFA1/CHCHD2/NDUFS5	36
BP	GO:0042775	mitochondrial ATP synthesis coupled electron transport	36/510	101/18903	5.89694237950334e-31	4.79775231996392e-28	4.27559359052832e-28	COX6C/COX6A1/COX7B/CYCS/COX5B/UQCRQ/COX5A/UQCR10/COX7A2/NDUFA4/NDUFB2/NDUFC2/COX6B1/UQCR11/COX8A/NDUFS6/NDUFB9/UQCRB/NDUFB8/NDUFAB1/SDHB/GHITM/UQCRC2/NDUFB3/SDHC/NDUFC1/COX4I1/NDUFA3/COX7C/CYC1/NDUFA12/NDUFB1/NDUFS8/NDUFA1/CHCHD2/NDUFS5	36
BP	GO:0019646	aerobic electron transport chain	33/510	93/18903	2.15518071602142e-28	1.46121252546253e-25	1.30218287473294e-25	COX6C/COX6A1/COX7B/CYCS/COX5B/UQCRQ/COX5A/UQCR10/COX7A2/NDUFA4/NDUFB2/NDUFC2/COX6B1/UQCR11/COX8A/NDUFS6/NDUFB9/UQCRB/NDUFB8/NDUFAB1/SDHB/UQCRC2/NDUFB3/SDHC/NDUFC1/COX4I1/NDUFA3/COX7C/CYC1/NDUFB1/NDUFS8/NDUFA1/NDUFS5	33
BP	GO:0015980	energy derivation by oxidation of organic compounds	55/510	333/18903	1.2389377396115e-27	6.35185554546164e-25	5.66055749587794e-25	COX6C/COX6A1/COX7B/ATP5F1E/CYCS/COX5B/NUPR1/UQCRQ/ATP5PD/COX5A/PHLDA2/IDH1/UQCR10/COX7A2/NDUFA4/NDUFB2/ATP5MF/NDUFC2/COX6B1/ATP5ME/ATP5PB/UQCR11/COX8A/SUCLG1/NDUFS6/NDUFB9/UQCRB/NDUFB8/HIF1A/OXA1L/NDUFAB1/SDHB/ATP5PF/GHITM/UQCRC2/ACADVL/NDUFB3/SDHC/NDUFC1/ATP5F1B/COX4I1/NDUFA3/ATP5F1C/MDH2/COX7C/CYC1/NDUFA13/NDUFA12/NDUFB1/ATP5PO/NDUFS8/ATP5MG/NDUFA1/CHCHD2/NDUFS5	55
BP	GO:0022904	respiratory electron transport chain	36/510	122/18903	1.2491358004841e-27	6.35185554546164e-25	5.66055749587794e-25	COX6C/COX6A1/COX7B/CYCS/COX5B/UQCRQ/COX5A/UQCR10/COX7A2/NDUFA4/NDUFB2/NDUFC2/COX6B1/UQCR11/COX8A/NDUFS6/NDUFB9/UQCRB/NDUFB8/NDUFAB1/SDHB/GHITM/UQCRC2/NDUFB3/SDHC/NDUFC1/COX4I1/NDUFA3/COX7C/CYC1/NDUFA12/NDUFB1/NDUFS8/NDUFA1/CHCHD2/NDUFS5	36

BP	GO:0015986	proton motive force-driven ATP synthesis	29/510	76/18903	3.66371062651092e-26	1.65599720318294e-23	1.47576835060861e-23	ATP5F1E/ATP5PD/NDUFB2/ATP5MF/NDUFC2/ATP5ME/ATP5PB/ATP5MC1/NDUFS6/NDUFB9/ATP5MC3/NDUFB8/NDUFAB1/SDHB/ATP5PF/NDUFB3/SDHC/NDUFC1/ATP5F1B/NDUFA3/ATP5F1C/NDUFA13/NDUFA12/NDUFB1/ATP5PO/NDUFS8/ATP5MG/NDUFA1/NDUFS5	29
BP	GO:0042776	proton motive force-driven mitochondrial ATP synthesis	27/510	67/18903	3.31952099209222e-25	1.35038113958312e-22	1.20341371544901e-22	ATP5F1E/ATP5PD/NDUFB2/ATP5MF/NDUFC2/ATP5ME/ATP5PB/NDUFS6/NDUFB9/NDUFB8/NDUFAB1/SDHB/ATP5PF/NDUFB3/SDHC/NDUFC1/ATP5F1B/NDUFA3/ATP5F1C/NDUFA13/NDUFA12/NDUFB1/ATP5PO/NDUFS8/ATP5MG/NDUFA1/NDUFS5	27
BP	GO:0022900	electron transport chain	39/510	176/18903	9.92974509359978e-25	3.67220027643308e-22	3.27253991410121e-22	GPX2/COX6C/ME1/COX6A1/COX7B/CYCS/COX5B/CYB5A/UQCRQ/COX5A/UQCR10/COX7A2/NDUFA4/NDUFB2/NDUFC2/COX6B1/UQCR11/COX8A/NDUFS6/NDUFB9/UQCRB/NDUFB8/NDUFAB1/SDHB/GHITM/UQCRC2/NDUFB3/SDHC/NDUFC1/COX4I1/NDUFA3/COX7C/CYC1/NDUFA12/NDUFB1/NDUFS8/NDUFA1/CHCHD2/NDUFS5	39
BP	GO:0046034	ATP metabolic process	42/510	217/18903	4.33937035888424e-24	1.47104655166176e-21	1.31094662421029e-21	TSPO/EIF6/ATP5F1E/NUPR1/ATP5PD/HK1/ATP1B1/NDUFB2/ATP5MF/NDUFC2/TPI1/ATP5ME/ATP5PB/ATP5MC1/NDUFS6/NDUFB9/ENO1/ATP5MC3/NDUFB8/LDHA/HIF1A/GPI/NDUFAB1/PGAM1/SDHB/ATP5PF/NDUFB3/SDHC/NDUFC1/ATP5F1B/NDUFA3/ATP5F1C/TMSB4X/NDUFA13/NDUFA12/NDUFB1/PGK1/ATP5PO/NDUFS8/ATP5MG/NDUFA1/NDUFS5	42
BP	GO:0006754	ATP biosynthetic process	31/510	106/18903	8.06807254052785e-24	2.52468608422056e-21	2.24991431818445e-21	ATP5F1E/ATP5PD/NDUFB2/ATP5MF/NDUFC2/ATP5ME/ATP5PB/ATP5MC1/NDUFS6/NDUFB9/ENO1/ATP5MC3/NDUFB8/NDUFAB1/SDHB/ATP5PF/NDUFB3/SDHC/NDUFC1/ATP5F1B/NDUFA3/ATP5F1C/TMSB4X/NDUFA13/NDUFA12/NDUFB1/ATP5PO/NDUFS8/ATP5MG/NDUFA1/NDUFS5	31

BP	GO:0009206	purine ribonucleoside triphosphate biosynthetic process	31/510	117/18903	2.17469449525953e-22	6.31904086193983e-20	5.63131416667204e-20	ATP5F1E/ATP5PD/NDUFB2/ATP5MF/NDUFC2/ATP5ME/ATP5PB/ATP5MC1/NDUFS6/NDUFB9/ENO1/ATP5MC3/NDUFB8/NDUFAB1/SDHB/ATP5PF/NDUFB3/SDHC/NDUFC1/ATP5F1B/NDUFA3/ATP5F1C/TMSB4X/NDUFA13/NDUFA12/NDUFB1/ATP5PO/NDUFS8/ATP5MG/NDUFA1/NDUFS5	31
BP	GO:0009145	purine nucleoside triphosphate biosynthetic process	31/510	118/18903	2.87690434974698e-22	7.80216459651382e-20	6.95302356528324e-20	ATP5F1E/ATP5PD/NDUFB2/ATP5MF/NDUFC2/ATP5ME/ATP5PB/ATP5MC1/NDUFS6/NDUFB9/ENO1/ATP5MC3/NDUFB8/NDUFAB1/SDHB/ATP5PF/NDUFB3/SDHC/NDUFC1/ATP5F1B/NDUFA3/ATP5F1C/TMSB4X/NDUFA13/NDUFA12/NDUFB1/ATP5PO/NDUFS8/ATP5MG/NDUFA1/NDUFS5	31
BP	GO:0009205	purine ribonucleoside triphosphate metabolic process	42/510	242/18903	3.63990954328547e-22	9.25447001380332e-20	8.24726872833893e-20	TSPO/EIF6/ATP5F1E/NUPR1/ATP5PD/HK1/ATP1B1/NDUFB2/ATP5MF/NDUFC2/TPI1/ATP5ME/ATP5PB/ATP5MC1/NDUFS6/NDUFB9/ENO1/ATP5MC3/NDUFB8/LDHA/HIF1A/GPI/NDUFAB1/PGAM1/SDHB/ATP5PF/NDUFB3/SDHC/NDUFC1/ATP5F1B/NDUFA3/ATP5F1C/TMSB4X/NDUFA13/NDUFA12/NDUFB1/PGK1/ATP5PO/NDUFS8/ATP5MG/NDUFA1/NDUFS5	42
BP	GO:0009144	purine nucleoside triphosphate metabolic process	42/510	247/18903	8.23721616230387e-22	1.9711173734266e-19	1.75659272216561e-19	TSPO/EIF6/ATP5F1E/NUPR1/ATP5PD/HK1/ATP1B1/NDUFB2/ATP5MF/NDUFC2/TPI1/ATP5ME/ATP5PB/ATP5MC1/NDUFS6/NDUFB9/ENO1/ATP5MC3/NDUFB8/LDHA/HIF1A/GPI/NDUFAB1/PGAM1/SDHB/ATP5PF/NDUFB3/SDHC/NDUFC1/ATP5F1B/NDUFA3/ATP5F1C/TMSB4X/NDUFA13/NDUFA12/NDUFB1/PGK1/ATP5PO/NDUFS8/ATP5MG/NDUFA1/NDUFS5	42
BP	GO:0009201	ribonucleoside triphosphate biosynthetic process	31/510	123/18903	1.11577805643162e-21	2.43079246254265e-19	2.16623951793119e-19	ATP5F1E/ATP5PD/NDUFB2/ATP5MF/NDUFC2/ATP5ME/ATP5PB/ATP5MC1/NDUFS6/NDUFB9/ENO1/ATP5MC3/NDUFB8/NDUFAB1/SDHB/ATP5PF/NDUFB3/SDHC/NDUFC1/ATP5F1B/NDUFA3/ATP5F1C/TMSB4X/NDUFA13/NDUFA12/NDUFB1/ATP5PO/NDUFS8/ATP5MG/NDUFA1/NDUFS5	31

BP	GO:0009199	ribonucleoside triphosphate metabolic process	42/510	249/18903	1.13532587975198e-21	2.43079246254265e-19	2.16623951793119e-19	TSPO/EIF6/ATP5F1E/NUPR1/ATP5PD/HK1/ATP1B1/NDUFB2/ATP5MF/NDUFC2/TPI1/ATP5ME/ATP5PB/ATP5MC1/NDUFS6/NDUFB9/ENO1/ATP5MC3/NDUFB8/LDHA/HIF1A/GPI/NDUFAB1/PGAM1/SDHB/ATP5PF/NDUFB3/SDHC/NDUFC1/ATP5F1B/NDUFA3/ATP5F1C/TMSB4X/NDUFA13/NDUFA12/NDUFB1/PGK1/ATP5PO/NDUFS8/ATP5MG/NDUFA1/NDUFS5	42
BP	GO:0009142	nucleoside triphosphate biosynthetic process	32/510	134/18903	1.46738392248977e-21	2.98465889834419e-19	2.65982643634461e-19	ATP5F1E/ATP5PD/NDUFB2/ATP5MF/NDUFC2/ATP5ME/ATP5PB/ATP5MC1/NDUFS6/NDUFB9/ENO1/ATP5MC3/NDUFB8/NDUFAB1/SDHB/ATP5PF/NDUFB3/SDHC/NDUFC1/ATP5F1B/NDUFA3/ATP5F1C/TMSB4X/NDUFA13/NDUFA12/NDUFB1/ATP5PO/CMPK1/NDUFS8/ATP5MG/NDUFA1/NDUFS5	32
BP	GO:0009141	nucleoside triphosphate metabolic process	43/510	267/18903	2.37789695207166e-21	4.60632609572738e-19	4.10500105410265e-19	TSPO/EIF6/ATP5F1E/NUPR1/ATP5PD/HK1/ATP1B1/NDUFB2/ATP5MF/NDUFC2/TPI1/ATP5ME/ATP5PB/ATP5MC1/NDUFS6/NDUFB9/ENO1/ATP5MC3/NDUFB8/LDHA/HIF1A/GPI/NDUFAB1/PGAM1/SDHB/ATP5PF/NDUFB3/SDHC/NDUFC1/ATP5F1B/NDUFA3/ATP5F1C/TMSB4X/NDUFA13/NDUFA12/NDUFB1/PGK1/ATP5PO/CMPK1/NDUFS8/ATP5MG/NDUFA1/NDUFS5	43
BP	GO:0009150	purine ribonucleotide metabolic process	54/510	447/18903	1.62612679552321e-20	3.00685627463109e-18	2.67960798267078e-18	SULT2B1/TSPO/ELOVL6/EIF6/ACSL1/ATP5F1E/APRT/NUPR1/ATP5PD/HK1/ATP1B1/NDUFB2/ATP5MF/NDUFC2/TPI1/ATP5ME/ATP5PB/HMGCS1/ATP5MC1/SUCLG1/NDUFS6/NDUFB9/ENO1/ATP5MC3/GUCY1A1/HMGCR/NDUFB8/PANK3/LDHA/HIF1A/GPI/NDUFAB1/PGAM1/SDHB/ATP5PF/NDUFB3/SDHC/NDUFC1/ELOVL1/ATP5F1B/TECR/NDUFA3/ATP5F1C/GUK1/TMSB4X/NDUFA13/NDUFA12/NDUFB1/PGK1/ATP5PO/NDUFS8/ATP5MG/NDUFA1/NDUFS5	54
BP	GO:0009259	ribonucleotide metabolic process	55/510	466/18903	2.106352086449e-20	3.7254957772498e-18	3.32003505067751e-18	SULT2B1/TSPO/ELOVL6/EIF6/ACSL1/ATP5F1E/APRT/NUPR1/ATP5PD/HK1/ATP1B1/NDUFB2/ATP5MF/NDUFC2/TPI1/ATP5ME/ATP5PB/HMGCS1/ATP5MC1/SUCLG1/NDUFS6/NDUFB9/ENO1/ATP5MC3/GUCY1A1/HMGCR/NDUFB8/PANK3/LDHA/HIF1A/GPI/NDUFAB1/PGAM1/SDHB/ATP5PF/NDUFB3/SDHC/NDUFC1/ELOVL1/ATP5F1B/TECR/NDUFA3/ATP5F1C/GUK1/TMSB4	55

								X/NDUFA13/NDUFA12/NDUFB1/PGK1/ATP5PO/CMPK1/NDUFS8/ATP5MG/NDUFA1/NDUFS5	
BP	GO:0009152	purine ribonucleotide biosynthetic process	38/510	220/18903	4.40922840202378e-20	7.44112766451193e-18	6.63127973828574e-18	ELOVL6/ACSL1/ATP5F1E/APRT/ATP5PD/NDUFB2/ATP5MF/NDUFC2/ATP5ME/ATP5PB/ATP5MC1/NDUFS6/NDUFB9/ENO1/ATP5MC3/GUCY1A1/NDUFB8/PANK3/NDUFAB1/SDHB/ATP5PF/NDUFB3/SDHC/NDUFC1/ELOVL1/ATP5F1B/TECR/NDUFA3/ATP5F1C/TMSB4X/NDUFA13/NDUFA12/NDUFB1/ATP5PO/NDUFS8/ATP5MG/NDUFA1/NDUFS5	38
BP	GO:1902600	proton transmembrane transport	33/510	160/18903	4.57296439559484e-20	7.44112766451193e-18	6.63127973828574e-18	COX6A1/COX7B/ATP5F1E/COX5B/CYB5A/SLC9A9/ATP5PD/COX5A/SLC25A5/SLC9A3R1/UQCR10/ATP1B1/NDUFA4/ATP5MF/COX6B1/ATP5ME/ATP5PB/COX8A/ATP5MC1/ATP5MC3/CLCN3/ATP5PF/ATP6V0D1/ATP5F1B/COX4I1/ATP5F1C/ATP6V0E1/TMSB4X/CYC1/ATP6V0B/ATP5PO/ATP5MG/COX17	33
BP	GO:0019693	ribose phosphate metabolic process	55/510	475/18903	5.16895769374258e-20	7.87148895252161e-18	7.0148030721121e-18	SULT2B1/TSPO/ELOVL6/EIF6/ACSL1/ATP5F1E/APRT/NUPR1/ATP5PD/HK1/ATP1B1/NDUFB2/ATP5MF/NDUFC2/TPI1/ATP5ME/ATP5PB/HMGCS1/ATP5MC1/SUCLG1/NDUFS6/NDUFB9/ENO1/ATP5MC3/GUCY1A1/HMGCR/NDUFB8/PANK3/LDHA/HIF1A/GPI/NDUFAB1/PGAM1/SDHB/ATP5PF/NDUFB3/SDHC/NDUFC1/ELOVL1/ATP5F1B/TECR/NDUFA3/ATP5F1C/GUK1/TMSB4X/NDUFA13/NDUFA12/NDUFB1/PGK1/ATP5PO/CMPK1/NDUFS8/ATP5MG/NDUFA1/NDUFS5	55
BP	GO:0009260	ribonucleotide biosynthetic process	39/510	234/18903	5.22443957025771e-20	7.87148895252161e-18	7.0148030721121e-18	ELOVL6/ACSL1/ATP5F1E/APRT/ATP5PD/NDUFB2/ATP5MF/NDUFC2/ATP5ME/ATP5PB/ATP5MC1/NDUFS6/NDUFB9/ENO1/ATP5MC3/GUCY1A1/NDUFB8/PANK3/NDUFAB1/SDHB/ATP5PF/NDUFB3/SDHC/NDUFC1/ELOVL1/ATP5F1B/TECR/NDUFA3/ATP5F1C/TMSB4X/NDUFA13/NDUFA12/NDUFB1/ATP5PO/CMPK1/NDUFS8/ATP5MG/NDUFA1/NDUFS5	39
BP	GO:004	ribose phosphate	39/510	241/18903	1.53715825021488e-19	2.23327134352647e-17	1.99021541869926e-17	ELOVL6/ACSL1/ATP5F1E/APRT/ATP5PD/NDUFB2/ATP5MF/NDUFC2/ATP5ME/ATP5PB/ATP5MC1/NDUFS6/NDUFB9/ENO1/ATP5MC3/GUCY1A1/NDUFB8/PANK3/NDUFAB1/SDHB/ATP5PF/NDUFB3/SDHC/NDUFC1/ELOVL1/A	39

	6390	biosynthetic process						TP5F1B/TECR/NDUFA3/ATP5F1C/TMSB4X/NDUFA13/NDUFA12/NDUFB1/ATP5PO/CMPK1/NDUFS8/ATP5MG/NDUFA1/NDUFS5	
BP	GO:0006164	purine nucleotide biosynthetic process	39/510	242/18903	1.7875581564072e-19	2.5075126138843e-17	2.23460990586802e-17	ELOVL6/ACSL1/ATP5F1E/APRT/ATP5PD/NDUFB2/ATP5MF/NDUFC2/ATP5ME/ATP5PB/ATP5MC1/NDUFS6/NDUFB9/ENO1/ATP5MC3/GUCY1A1/NDUFB8/PANK3/NDUFAB1/SDHB/ATP5PF/NDUFB3/SDHC/NDUFC1/ELOVL1/ATP5F1B/TECR/NDUFA3/ATP5F1C/GUK1/TMSB4X/NDUFA13/NDUFA12/NDUFB1/ATP5PO/NDUFS8/ATP5MG/NDUFA1/NDUFS5	39
BP	GO:0006163	purine nucleotide metabolic process	54/510	476/18903	2.94512888320561e-19	3.99359476562681e-17	3.55895574517899e-17	SULT2B1/TSPO/ELOVL6/EIF6/ACSL1/ATP5F1E/APRT/NUPR1/ATP5PD/HK1/ATP1B1/NDUFB2/ATP5MF/NDUFC2/TPI1/ATP5ME/ATP5PB/HMGCS1/ATP5MC1/SUCLG1/NDUFS6/NDUFB9/ENO1/ATP5MC3/GUCY1A1/HMGCR/NDUFB8/PANK3/LDHA/HIF1A/GPI/NDUFAB1/PGAM1/SDHB/ATP5PF/NDUFB3/SDHC/NDUFC1/ELOVL1/ATP5F1B/TECR/NDUFA3/ATP5F1C/GUK1/TMSB4X/NDUFA13/NDUFA12/NDUFB1/PGK1/ATP5PO/NDUFS8/ATP5MG/NDUFA1/NDUFS5	54
BP	GO:0072522	purine-containing compound biosynthetic process	39/510	251/18903	6.71483656954931e-19	8.8115984402989e-17	7.85259665382949e-17	ELOVL6/ACSL1/ATP5F1E/APRT/ATP5PD/NDUFB2/ATP5MF/NDUFC2/ATP5ME/ATP5PB/ATP5MC1/NDUFS6/NDUFB9/ENO1/ATP5MC3/GUCY1A1/NDUFB8/PANK3/NDUFAB1/SDHB/ATP5PF/NDUFB3/SDHC/NDUFC1/ELOVL1/ATP5F1B/TECR/NDUFA3/ATP5F1C/GUK1/TMSB4X/NDUFA13/NDUFA12/NDUFB1/ATP5PO/NDUFS8/ATP5MG/NDUFA1/NDUFS5	39
BP	GO:0009165	nucleotide biosynthetic process	41/510	304/18903	1.58488227940047e-17	2.01478159768785e-15	1.79550479284711e-15	ME1/ELOVL6/ACSL1/ATP5F1E/APRT/ATP5PD/NDUFB2/ATP5MF/NDUFC2/ATP5ME/ATP5PB/ATP5MC1/NDUFS6/NDUFB9/ENO1/ATP5MC3/GUCY1A1/NDUFB8/PANK3/NDUFAB1/SDHB/ATP5PF/NDUFB3/SDHC/NDUFC1/ELOVL1/ATP5F1B/TECR/NDUFA3/ATP5F1C/GUK1/TMSB4X/NDUFA13/NDUFA12/NDUFB1/ATP5PO/CMPK1/NDUFS8/ATP5MG/NDUFA1/NDUFS5	41
BP	GO:190	nucleoside phosphate	41/510	306/18903	2.01198366412716e-17	2.48022713505129e-15	2.21029401571098e-15	ME1/ELOVL6/ACSL1/ATP5F1E/APRT/ATP5PD/NDUFB2/ATP5MF/NDUFC2/ATP5ME/ATP5PB/ATP5MC1/NDUFS6/NDUFB9/ENO1/ATP5MC3/GUCY1A1/NDUFB8/PANK3/NDUFAB1/SDHB/ATP5PF/NDUFB3/SDHC/NDUFC1/ELOV	41

	1293	biosynthetic process						L1/ATP5F1B/TECR/NDUFA3/ATP5F1C/GUK1/TMSB4X/NDUFA13/NDUFA12/NDUFB1/ATP5PO/CMPK1/NDUFS8/ATP5MG/NDUFA1/NDUFS5	
BP	GO:0006123	mitochondrial electron transport, cytochrome c to oxygen	12/510	24/18903	2.636806561397e-13	3.15486149757735e-11	2.81150520044931e-11	COX6C/COX6A1/COX7B/CYCS/COX5B/COX5A/COX7A2/NDUFA4/COX6B1/COX8A/COX4I1/COX7C	12
BP	GO:0008544	epidermis development	37/510	362/18903	3.82282926929013e-12	4.44321984784922e-10	3.95964631682262e-10	KRTDAP/KRT6B/CSTA/KRT6A/SPINK5/TGM3/AKR1C3/SFN/SULT2B1/SCEL/KRT17/CALML5/KRT16/KRT6C/DSP/TMEM79/SPRR1B/ZNF750/GRHL1/SPRR1A/SLC9A3R1/GRHL3/SOX21/PPL/ANXA1/GJB5/ASAH1/TRIM16/CERS3/EMP1/KLK7/HDAC1/IVL/KRT10/KLF4/HES1/SPRR3	37
BP	GO:0009913	epidermal cell differentiation	29/510	235/18903	8.9701414463947e-12	1.0136259834426e-09	9.03308980742203e-10	KRT6B/CSTA/KRT6A/SPINK5/TGM3/AKR1C3/SFN/SULT2B1/SCEL/KRT17/KRT16/KRT6C/DSP/TMEM79/SPRR1B/GRHL1/SPRR1A/SLC9A3R1/PPL/ANXA1/ASAH1/TRIM16/CERS3/HDAC1/IVL/KRT10/KLF4/HES1/SPRR3	29
BP	GO:0010257	NADH dehydrogenase complex assembly	15/510	59/18903	3.19044073273103e-11	3.41545076335522e-09	3.04373348574119e-09	NDUFB2/NDUFC2/NDUFB9/NDUFB8/OXA1L/NDUFAB1/NDUFB3/NDUFC1/NDUFA3/NDUFA13/NDUFA12/NDUFB1/NDUFS8/NDUFA1/NDUFS5	15
BP	GO:0032981	mitochondrial respiratory chain complex I assembly	15/510	59/18903	3.19044073273103e-11	3.41545076335522e-09	3.04373348574119e-09	NDUFB2/NDUFC2/NDUFB9/NDUFB8/OXA1L/NDUFAB1/NDUFB3/NDUFC1/NDUFA3/NDUFA13/NDUFA12/NDUFB1/NDUFS8/NDUFA1/NDUFS5	15

BP	GO:0043588	skin development	32/510	302/18903	4.44632657195047e-11	4.63786063966526e-09	4.13310356647704e-09	KRT6B/CSTA/KRT6A/SPINK5/TGM3/AKR1C3/SFN/SCEL/KRT17/CLDN4/KRT16/JUP/KRT6C/DSP/TMEM79/SPRR1B/GRHL1/SPRR1A/GRHL3/SOX21/PPL/ANXA1/ASAH1/DHCR24/TRIM16/CERS3/ELOVL1/HDAC1/IVL/KRT10/KLF4/SPRR3	32
BP	GO:0006120	mitochondrial electron transport, NADH to ubiquinone	14/510	51/18903	4.69589780283243e-11	4.77572806548058e-09	4.25596632446181e-09	NDUFA4/NDUFB2/NDUFC2/NDUFS6/NDUFB9/NDUFB8/NDUFAB1/NDUFB3/NDUFC1/NDUFA3/NDUFB1/NDUFS8/NDUFA1/NDUFS5	14
BP	GO:0033108	mitochondrial respiratory chain complex assembly	18/510	99/18903	1.42113028409089e-10	1.41003853553213e-08	1.25657835645931e-08	COA3/NDUFB2/NDUFC2/NDUFB9/STMP1/NDUFB8/OXA1L/NDUFAB1/NDUFB3/NDUFC1/NDUFA3/NDUFA13/NDUFA12/NDUFB1/NDUFS8/NDUFA1/COX17/NDUFS5	18
BP	GO:0030216	keratinocyte differentiation	23/510	170/18903	2.05958471979768e-10	1.99485491431832e-08	1.77774681077273e-08	KRT6B/CSTA/KRT6A/TGM3/AKR1C3/SFN/SCEL/KRT17/KRT16/KRT6C/DSP/TMEM79/SPRR1B/GRHL1/SPRR1A/PPL/ANXA1/ASAH1/TRIM16/CERS3/IVL/KRT10/SPRR3	23
BP	GO:0052548	regulation of endopeptidase activity	36/510	428/18903	1.77241756638649e-09	1.66555157990181e-07	1.48428288598609e-07	CSTA/SPINK5/CSTB/PERP/SFN/SERPINB13/SERPINB11/SERPINB5/WFDC5/SERPINB3/ANXA2/FETUB/CARD18/LGMN/PI3/CYCS/SLPI/FAM162A/PDCD5/PYCARD/CASP1/A2ML1/ANXA8L1/DHCR24/CD44/EPHA4/SERPINB2/GPI/F3/SERPINB6/PSMA3/SOX2/NDUFA13/SPINT2/HDAC1/KLF4	36
BP	GO:1990748	cellular detoxification	18/510	115/18903	1.80148155151622e-09	1.66555157990181e-07	1.48428288598609e-07	GSTA1/GSTM3/TXN/GPX2/GSTP1/AKR1B10/RDH12/NQO1/PRDX6/ALDH1A1/MGST2/NFE2L2/RDH11/TXNL1/GSTO1/SELENOW/PRDX1/GPX3	18

BP	GO:0052547	regulation of peptidase activity	37/510	459/18903	3.3458423263445e-09	3.02464146301543e-07	2.69545753729367e-07	CSTA/SPINK5/CSTB/PERP/SFN/SERPINB13/SERPINB11/SERPINB5/CLDN4/WFDC5/SERPINB3/ANXA2/FETUB/CARD18/LGMN/PI3/CYCS/SLPI/FAM162A/PDCD5/PYCARD/CASP1/A2ML1/ANXA8L1/DHCR24/CD44/EPHA4/SERPINB2/GPI/F3/SERPINB6/PSMA3/SOX2/NDUFA13/SPINT2/HDAC1/KLF4	37
BP	GO:0097237	cellular response to toxic substance	18/510	123/18903	5.45164573078254e-09	4.82115105061378e-07	4.29644574297828e-07	GSTA1/GSTM3/TXN/GPX2/GSTP1/AKR1B10/RDH12/NQO1/PRDX6/ALDH1A1/MGST2/NFE2L2/RDH11/TXNL1/GSTO1/SELENOW/PRDX1/GPX3	18
BP	GO:0006805	xenobiotic metabolic process	17/510	118/18903	1.84498926480437e-08	1.5968970913243e-06	1.42310034221417e-06	GSTA1/GSTM3/GSTP1/ALDH3A1/NQO1/CYP2C18/GSTM4/UGT1A7/ACSL1/EPHX1/AADAC/CES2/GSTA4/GSTO1/AKR1C1/GUK1/CBR1	17
BP	GO:0018149	peptide cross-linking	10/510	36/18903	2.53280920361965e-08	2.14655580006765e-06	1.91293747747063e-06	CSTA/TGM3/DSP/SPRR1B/PI3/SPRR1A/ANXA1/IVL/KRT10/SPRR3	10
BP	GO:0006122	mitochondrial electron transport, ubiquinol to cytochrome c	7/510	14/18903	2.9091945925472e-08	2.41522522499633e-06	2.15236652561387e-06	CYCS/UQCRQ/UQCR10/UQCR11/UQCRB/UQCRC2/CYC1	7
BP	GO:0006631	fatty acid metabolic process	32/510	400/18903	4.72389752118768e-08	3.84336302323829e-06	3.42507432904639e-06	GSTA1/DBI/AKR1C2/AKR1C3/ADH7/GSTP1/MGLL/CYP2C18/ELOVL6/DECR1/EIF6/GSTM4/ACSL1/EPHX1/LIPH/SCD/MSMO1/APPL2/ANXA1/CES2/ASAH1/HPGD/PCCB/NDUFAB1/AKR1C1/ACADVL/PTGR1/ELOVL1/TECR/INSIG1/ECH1/CBR1	32

BP	GO:0031424	keratinization	14/510	84/18903	5.09975175566692e-08	4.06780198863785e-06	3.62508669690751e-06	KRT6B/KRT6A/TGM3/SFN/KRT17/KRT16/KRT6C/TMEM79/SPRR1B/SPRR1A/PPL/CERS3/IVL/SPRR3	14
BP	GO:0033559	unsaturated fatty acid metabolic process	16/510	115/18903	7.98843914288597e-08	6.24941739101157e-06	5.56926809880552e-06	GSTA1/AKR1C2/AKR1C3/GSTP1/MGLL/CYP2C18/ELOVL6/EPHX1/SCD/ANXA1/CES2/HPGD/AKR1C1/PTGR1/ELOVL1/CBR1	16
BP	GO:0009636	response to toxic substance	24/510	250/18903	8.28325992273517e-08	6.35779271050692e-06	5.66584849531279e-06	GSTA1/GSTM3/TXN/GPX2/GSTP1/AKR1B10/RDH12/NQO1/PRDX6/CDH1/ALDH1A1/MGST2/NFE2L2/EPHX1/NUPR1/RDH11/SDC1/SCN9A/TXNL1/GSTO1/SELENOW/PRDX1/PON2/GPX3	24
BP	GO:0098754	detoxification	18/510	154/18903	1.89444887389435e-07	1.42715148500041e-05	1.27182883463784e-05	GSTA1/GSTM3/TXN/GPX2/GSTP1/AKR1B10/RDH12/NQO1/PRDX6/ALDH1A1/MGST2/NFE2L2/RDH11/TXNL1/GSTO1/SELENOW/PRDX1/GPX3	18
BP	GO:0045216	cell-cell junction organization	21/510	208/18903	2.30051033853465e-07	1.70154110130163e-05	1.51635552266284e-05	PERP/DSG1/POF1B/CLDN7/CD9/CLDN4/JUP/DSP/CDH1/PKP1/GJB6/GRHL1/HOPX/GJA1/GJB2/TJP1/F2RL1/EPHA4/CTNND1/CDC42/ACTB	21
BP	GO:0006066	alcohol metabolic process	29/510	367/18903	2.63379896728409e-07	1.91326682123423e-05	1.70503827882076e-05	AKR1C2/AKR1C3/ADH7/SULT2B1/DEGS2/AKR1B10/RDH12/SPTSSB/CYP2C18/ALDH1A1/TM7SF2/FDFT1/RDH11/ALDH3B2/MSMO1/IDH1/SPTLC2/SQLE/TPI1/DHCR7/PTS/HMGCS1/ASAH1/DHCR24/HMGCR/AKR1C1/ACADVL/SC5D/INSIG1	29
BP	GO:000	icosanoid metabolic process	16/510	126/18903	2.89820254838789e-07	2.06840139769156e-05	1.84328893382232e-05	GSTA1/AKR1C2/AKR1C3/GSTP1/MGLL/CYP2C18/MGST2/EPHX1/PYCARD/CASP1/ANXA1/CES2/HPGD/AKR1C1/PTGR1/CBR1	16

	GO:0006690								
BP	GO:0006749	glutathione metabolic process	11/510	57/18903	2.97438818457982e-07	2.0861743335984e-05	1.85912756945425e-05	GSTA1/GSTM3/GSTP1/MGST2/NFE2L2/GSTM4/IDH1/GSTA4/GSTO1/ETHE1/GLO1	11
BP	GO:0061436	establishment of skin barrier	8/510	27/18903	3.7551656969463e-07	2.58915492460636e-05	2.30736675473382e-05	SFN/CLDN4/KRT16/TMEM79/GRHL1/GRHL3/ELOVL1/KLF4	8
BP	GO:0010951	negative regulation of endopeptidase activity	23/510	252/18903	3.82116387533116e-07	2.59074910747453e-05	2.30878743625272e-05	CSTA/SPINK5/CSTB/SFN/SERPINB13/SERPINB11/SERPINB5/WFDC5/SERPINB3/ANXA2/FETUB/CARD18/PI3/SLPI/A2ML1/ANXA8L1/DHCR24/CD44/SERPINB2/GPI/SERPINB6/SPINT2/KLF4	23
BP	GO:0006692	prostanoid metabolic process	10/510	50/18903	7.29243498248963e-07	4.78477830786578e-05	4.26403159247781e-05	GSTA1/AKR1C2/AKR1C3/GSTP1/ANXA1/CES2/HPGD/AKR1C1/PTGR1/CBR1	10
BP	GO:0006693	prostaglandin metabolic process	10/510	50/18903	7.29243498248963e-07	4.78477830786578e-05	4.26403159247781e-05	GSTA1/AKR1C2/AKR1C3/GSTP1/ANXA1/CES2/HPGD/AKR1C1/PTGR1/CBR1	10
BP	GO:0010466	negative regulation of peptidase activity	23/510	263/18903	8.10872002118566e-07	5.23591635653703e-05	4.6660704683314e-05	CSTA/SPINK5/CSTB/SFN/SERPINB13/SERPINB11/SERPINB5/WFDC5/SERPINB3/ANXA2/FETUB/CARD18/PI3/SLPI/A2ML1/ANXA8L1/DHCR24/CD44/SERPINB2/GPI/SERPINB6/SPINT2/KLF4	23

BP	GO:0016126	sterol biosynthetic process	11/510	65/18903	1.18762162819946e-06	7.5488199742428e-05	6.72725145973508e-05	TM7SF2/FDFT1/MSMO1/SQLE/DHCR7/HMGCS1/ERG28/DHCR24/HMGCR/SC5D/INSIG1	11
BP	GO:0033561	regulation of water loss via skin	8/510	31/18903	1.21333654933153e-06	7.593620127201e-05	6.7671758314134e-05	SFN/CLDN4/KRT16/TMEM79/GRHL1/GRHL3/ELOVL1/KLF4	8
BP	GO:0032970	regulation of actin filament-based process	29/510	406/18903	2.0740578748268e-06	0.000127837385375688	0.000113924327287137	DSC2/TACSTD2/JUP/DSTN/DSP/SRI/S100A10/KANK1/ARPC2/GRHL3/SCIN/PFN1/PYCARD/TMSB10/TJP1/PAK1/F2RL1/ARPC3/DBNL/CAPG/FRMD6/SDC4/CDC42/CAPZA1/ABRACL/CAPZB/BRK1/TMSB4X/ARF1	29
BP	GO:0021762	substantia nigra development	9/510	44/18903	2.15840981209616e-06	0.000131050912173242	0.000116788113006429	DYNLL1/COX6B1/ATP5PB/NDRG2/CALM1/ATP5PF/CDC42/ACTB/YWHAQ	9
BP	GO:0071466	cellular response to xenobiotic stimulus	18/510	183/18903	2.43634423633149e-06	0.00014575071107936	0.000129888073528261	GSTA1/GSTM3/GSTP1/ALDH3A1/NQO1/CYP2C18/NFE2L2/GSTM4/UGT1A7/ACSL1/EPHX1/AADAC/CES2/GSTA4/GSTO1/AKR1C1/GUK1/CBR1	18
BP	GO:0098869	cellular oxidant detoxification	13/510	100/18903	2.88009973059062e-06	0.000169800662377429	0.000151320571657576	GSTA1/TXN/GPX2/GSTP1/NQO1/PRDX6/MGST2/NFE2L2/TXNL1/GSTO1/SELENOW/PRDX1/GPX3	13

BP	GO:0006790	sulfur compound metabolic process	25/510	338/18903	5.72765233855621e-06	0.0003328584244749 52	0.0002966321000599 64	GSTA1/GSTM3/SULT2B1/GSTP1/B4GALT4/TST/MGST2/ELOVL6/NFE2L2/GSTM4/ACSL1/GLRX3/IDH1/SQOR/TSTD1/HMGCS1/SUCLG1/UGDH/GSTA4/NDUFAB1/GSTO1/ELOVL1/TECR/ETHE1/GLO1	25
BP	GO:0110053	regulation of actin filament organization	22/510	278/18903	7.17762470850238e-06	0.000411247567805 46	0.000366489836858 15	TACSTD2/DSTN/S100A10/KANK1/ARPC2/SCIN/PFN1/PYCARD/TMSB10/TJP1/PAK1/F2RL1/ARPC3/DBNL/CAPG/SDC4/CDC42/CAPZA1/CAPZB/BRK1/TMSB4X/ARF1	22
BP	GO:0051346	negative regulation of hydrolase activity	26/510	373/18903	1.07767653058953e-05	0.0006088872397830 87	0.0005426195864547 31	CSTA/SPINK5/CSTB/SFN/SERPINB13/FKBP1A/SERPINB11/SERPINB5/WFDC5/SERPINB3/ANXA2/FETUB/CARD18/PI3/SLPI/A2ML1/ANXA1/ANXA8L1/DHCR24/CD44/SERPINB2/GPI/SERPINB6/LGALS3/SPINT2/KLF4	26
BP	GO:0045861	negative regulation of proteolysis	25/510	352/18903	1.15544870749559e-05	0.000642002720334 72	0.0005721309757368 87	CSTA/SPINK5/CSTB/SFN/SERPINB13/SERPINB11/SERPINB5/WFDC5/SERPINB3/ANXA2/FETUB/CARD18/PI3/SLPI/A2ML1/ANXA8L1/DHCR24/CD44/EPHA4/SERPINB2/GIPC1/GPI/SERPINB6/SPINT2/KLF4	25
BP	GO:0031638	zymogen activation	10/510	67/18903	1.16785155616444e-05	0.000642002720334 72	0.0005721309757368 87	PERP/ANXA2/LGMN/CYCS/S100A10/PYCARD/DHCR24/ENO1/PGK1/PRSS3	10
BP	GO:0006739	NADP metabolic process	8/510	43/18903	1.6780168928764e-05	0.0009101563626961 58	0.0008111003760093 07	NQO1/TALDO1/ME1/PGD/IDH1/PGAM1/DERA/DCXR	8
BP	GO:000	cellular aldehyde	10/510	70/18903	1.73787658018378e-05	0.0009302213063404 75	0.0008289815709353 09	AKR1C3/ALDH3A1/TALDO1/ALDH1A1/RDH11/ALDH3B2/IDH1/TPI1/AKR1C1/GLO1	10

	6081	metabolic process							
BP	GO:0007015	actin filament organization	29/510	454/18903	1.77926551355616e-05	0.000940006767421 62	0.0008377020408321 85	POF1B/TACSTD2/DSTN/EMP2/S100A10/KANK1/ARPC2/SCIN/PFN1/PYCARD/TMSB10/NEBL/PLS3/TJP1/PAK1/F2RL1/ARPC3/DBNL/CAPG/ACTR3/SDC4/CDC42/CAPZA1/CAPZB/BRK1/TMSB4X/ABI1/CFL1/ARF1	29
BP	GO:2000379	positive regulation of reactive oxygen species metabolic process	10/510	71/18903	1.97463744033978e-05	0.001029849372731 06	0.0009177667131619 73	AKR1C3/GSTP1/TSPO/NFE2L2/F2RL1/CLCN3/ROMO1/AKR1C1/DCXR/CBR1	10
BP	GO:0006695	cholesterol biosynthetic process	9/510	58/18903	2.31915042694307e-05	0.001179287992100 55	0.001050941325051 57	TM7SF2/FDFT1/MSMO1/DHCR7/HMGCS1/DHCR24/HMGCR/SC5D/INSIG1	9
BP	GO:1902653	secondary alcohol biosynthetic process	9/510	58/18903	2.31915042694307e-05	0.001179287992100 55	0.001050941325051 57	TM7SF2/FDFT1/MSMO1/DHCR7/HMGCS1/DHCR24/HMGCR/SC5D/INSIG1	9
BP	GO:0008202	steroid metabolic process	23/510	323/18903	2.47143120397449e-05	0.001241207671329 41	0.001106122035930 88	AKR1C2/AKR1C3/SULT2B1/TSPO/TM7SF2/LGMN/FDFT1/UGT1A7/MSMO1/SQLE/DHCR7/HMGCS1/ERG28/ASAH1/DHCR24/BDH1/HMGCR/AKR1C1/ACADVL/SC5D/ATP8B1/INSIG1/CBR1	23

BP	GO:0008064	regulation of actin polymerization or depolymerization	15/510	159/18903	2.74018169840928e-05	0.001359397457210085	0.001211448750870568	DSTN/KANK1/ARPC2/SCIN/PFN1/PYCARD/TMSB10/F2RL1/ARPC3/DBNL/CAPG/CAPZA1/CAPZB/BRK1/TMSB4X	15
BP	GO:0120254	olefinic compound metabolic process	15/510	160/18903	2.95014239507192e-05	0.001445925212420802	0.001288559341610417	GSTA1/AKR1C2/AKR1C3/ADH7/GSTP1/MGLL/AKR1B10/RDH12/CYP2C18/ALDH1A1/EPHX1/RDH11/AKR1C1/ELOVL1/CBR1	15
BP	GO:0008154	actin polymerization or depolymerization	17/510	200/18903	3.16385140020705e-05	0.001532208035240313	0.001365451656930146	DSTN/KANK1/ARPC2/SCIN/PFN1/PYCARD/TMSB10/F2RL1/ARPC3/DBNL/CAPG/CAPZA1/CAPZB/BRK1/TMSB4X/ABI1/CFL1	17
BP	GO:0030901	midbrain development	11/510	91/18903	3.34108571294306e-05	0.001599004315320381	0.001424978228520952	DYNLL1/UQCRQ/COX6B1/ATP5PB/NDRG2/CALM1/ATP5PF/CDC42/ACTB/YWHAQ/HES1	11
BP	GO:0030832	regulation of actin filament length	15/510	162/18903	3.41303163443396e-05	0.001614443335910597	0.001438736958260078	DSTN/KANK1/ARPC2/SCIN/PFN1/PYCARD/TMSB10/F2RL1/ARPC3/DBNL/CAPG/CAPZA1/CAPZB/BRK1/TMSB4X	15
BP	GO:0006090	pyruvate metabolic process	12/510	108/18903	3.47862108670968e-05	0.001626555239160494	0.001449530674240418	ME1/EIF6/NUPR1/HK1/TPI1/ENO1/VDAC1/LDHA/HIF1A/GPI/PGAM1/PGK1	12

BP	GO:0006633	fatty acid biosynthetic process	15/510	164/18903	3.9387184175753e-05	0.00182076210485185	0.00162260122369968	AKR1C3/GSTP1/MGLL/ELOVL6/EIF6/GSTM4/LIPH/SCD/ANXA1/NDUFAB1/ACADVL/ELOVL1/TECR/INSIG1/CBR1	15
BP	GO:0006734	NADH metabolic process	7/510	37/18903	5.09276144433284e-05	0.00232779253433101	0.00207444948720074	NQO1/ME1/HK1/TPI1/ENO1/MDH2/PGK1	7
BP	GO:2000377	regulation of reactive oxygen species metabolic process	14/510	149/18903	5.27306685418135e-05	0.00238342621808997	0.00212402833284217	AKR1C3/GSTP1/TSPO/EIF6/NFE2L2/NDUFC2/F2RL1/VDAC1/CLCN3/HIF1A/ROMO1/AKR1C1/DCXR/CBR1	14
BP	GO:0048857	neural nucleus development	9/510	65/18903	5.89066029468959e-05	0.00263273294449966	0.00234620200301631	DYNLL1/COX6B1/ATP5PB/NDRG2/CALM1/ATP5PF/CDC42/ACTB/YWHAQ	9
BP	GO:0009185	ribonucleoside diphosphate metabolic process	12/510	114/18903	5.9540666394781e-05	0.00263273294449966	0.00234620200301631	EIF6/NUPR1/HK1/TPI1/ENO1/LDHA/HIF1A/GPI/PGAM1/GUK1/PGK1/CMPK1	12
BP	GO:0050878	regulation of body fluid levels	25/510	390/18903	6.32890681846185e-05	0.00276838633736589	0.00246709169018479	SFN/HSPB1/CD9/CLDN4/KRT16/ANXA2/EMP2/TMEM79/NPR3/GRHL1/NFE2L2/APRT/GRHL3/F2RL1/SERPINB2/SOCS2/HIF1A/GPI/F3/ACTB/ELOVL1/CLIC1/VAMP8/CCND1/KLF4	25

BP	GO:0007043	cell-cell junction assembly	14/510	152/18903	6.56003344735584e-05	0.00283877121585798	0.00252981629855997	DSG1/POF1B/CLDN7/CD9/CLDN4/JUP/PKP1/GJB6/HOPX/GJA1/GJB2/TJP1/CTNND1/ACTB	14
BP	GO:0110096	cellular response to aldehyde	5/510	17/18903	6.62938214126125e-05	0.00283877121585798	0.00252981629855997	AKR1B10/RDH12/ALDH1A1/RDH11/SGK1	5
BP	GO:0072593	reactive oxygen species metabolic process	18/510	235/18903	7.36446459153438e-05	0.00310038911122938	0.0027629612635393	AKR1C3/GSTP1/NQO1/TSPO/EIF6/NFE2L2/NDUFC2/F2RL1/VDAC1/CLCN3/HIF1A/ROMO1/AKR1C1/PRDX1/DCXR/NDUFA13/GPX3/CBR1	18
BP	GO:0042060	wound healing	27/510	442/18903	7.39276656315758e-05	0.00310038911122938	0.0027629612635393	KRT6A/HSPB1/CD9/CLDN4/ANXA2/DSP/NFE2L2/S100A10/KANK1/SDC1/ARL8B/GRHL3/PPL/ANXA1/PAK1/CD44/F2RL1/SERPINB2/HIF1A/HBEGF/CHMP4B/F3/SDC4/ACTB/CLIC1/CHMP2A/SPRR3	27
BP	GO:0001667	ameboidal-type cell migration	29/510	492/18903	7.6005870159967e-05	0.0031550191817423	0.00281164572321081	HSPB1/TACSTD2/KRT16/JUP/RAB25/EMP2/LGMN/RAB11A/NFE2L2/GJA1/ANXA3/KANK1/SLC9A3R1/APPL2/PFN1/ANXA1/HIF1A/GIPC1/GPI/HBEGF/FGFBP1/SDC4/CDC42/C1QBP/ATP5F1B/TMSB4X/PRSS3/KLF4/S100A2	29
BP	GO:0006096	glycolytic process	10/510	83/18903	7.79418159489054e-05	0.00320270007353684	0.00285413731130282	EIF6/NUPR1/HK1/TPI1/ENO1/LDHA/HIF1A/GPI/PGAM1/PGK1	10

BP	GO:0006165	nucleoside diphosphate phosphorylation	11/510	100/18903	8.04251407481744e-05	0.00327169472563574	0.00291562299722856	EIF6/NUPR1/HK1/TPI1/ENO1/LDHA/HIF1A/GPI/PGAM1/PGK1/CMPK1	11
BP	GO:0051156	glucose 6-phosphate metabolic process	6/510	28/18903	8.50974358543082e-05	0.00342748880252798	0.00305446137657361	TALDO1/PGD/HK1/GPI/PGAM1/DERA	6
BP	GO:0006757	ATP generation from ADP	10/510	84/18903	8.63749487511885e-05	0.00344483619137093	0.00306992077914441	EIF6/NUPR1/HK1/TPI1/ENO1/LDHA/HIF1A/GPI/PGAM1/PGK1	10
BP	GO:0006721	terpenoid metabolic process	11/510	101/18903	8.80996981019364e-05	0.0034795104065891	0.00310082125971455	AKR1C3/ADH7/AKR1B10/RDH12/CYP2C18/ALDH1A1/FDFT1/UGT1A7/RDH11/HMGCS1/AKR1C1	11
BP	GO:0046939	nucleotide phosphorylation	11/510	102/18903	9.63915502358783e-05	0.00363961974179467	0.00324350525041164	EIF6/NUPR1/HK1/TPI1/ENO1/LDHA/HIF1A/GPI/PGAM1/PGK1/CMPK1	11
BP	GO:0002934	desmosome organization	4/510	10/18903	9.66270727910089e-05	0.00363961974179467	0.00324350525041164	PERP/JUP/DSP/GRHL1	4
BP	GO:003	polyketide metabolic process	4/510	10/18903	9.66270727910089e-05	0.00363961974179467	0.00324350525041164	AKR1C2/AKR1C3/AKR1B10/AKR1C1	4

	0638								
BP	GO:0030647	aminoglycoside antibiotic metabolic process	4/510	10/18903	9.66270727910089e-05	0.00363961974179467	0.00324350525041164	AKR1C2/AKR1C3/AKR1B10/AKR1C1	4
BP	GO:0044598	doxorubicin metabolic process	4/510	10/18903	9.66270727910089e-05	0.00363961974179467	0.00324350525041164	AKR1C2/AKR1C3/AKR1B10/AKR1C1	4
BP	GO:0007006	mitochondrial membrane organization	12/510	120/18903	9.82363886912517e-05	0.0036662901761102	0.00326727303382589	TIMM8B/HEBP2/TMEM14A/SLC25A5/PDCD5/OXA1L/BLOC1S2/GHITM/ROMO1/VDAC2/NDUFA13/TIMM13	12
BP	GO:0006720	isoprenoid metabolic process	12/510	121/18903	0.000106434916992979	0.00393615674843127	0.00350776893898393	AKR1C3/ADH7/AKR1B10/RDH12/CYP2C18/ALDH1A1/FDFT1/UGT1A7/RDH11/HMGCS1/HMGCR/AKR1C1	12
BP	GO:0009135	purine nucleoside diphosphate metabolic process	11/510	104/18903	0.000114989037393592	0.00417656610818869	0.00372201357879259	EIF6/NUPR1/HK1/TPI1/ENO1/LDHA/HIF1A/GPI/PGAM1/GUK1/PGK1	11
BP	GO:000	purine ribonucleoside	11/510	104/18903	0.000114989037393592	0.00417656610818869	0.00372201357879259	EIF6/NUPR1/HK1/TPI1/ENO1/LDHA/HIF1A/GPI/PGAM1/GUK1/PGK1	11

	9179	diphosphate metabolic process							
BP	GO:0046165	alcohol biosynthetic process	13/510	143/18903	0.000135471806010353	0.00487698501637272	0.00434620307312209	SPTSSB/TM7SF2/FDFT1/MSMO1/SPTLC2/DHCR7/PTS/HMGCS1/ASAH1/DHCR24/HMGCR/SC5D/INSIG1	13
BP	GO:0045104	intermediate filament cytoskeleton organization	10/510	89/18903	0.00014108931895774	0.00503466096070251	0.0044867185086838	KRT6B/KRT6A/KRT17/KRT16/KRT6C/DSP/PKP1/PPL/KRT18/KRT19	10
BP	GO:0045103	intermediate filament-based process	10/510	90/18903	0.000154963972408166	0.00548168208483843	0.00488508852149862	KRT6B/KRT6A/KRT17/KRT16/KRT6C/DSP/PKP1/PPL/KRT18/KRT19	10
BP	GO:0032956	regulation of actin cytoskeleton organization	23/510	365/18903	0.000158645710872507	0.00556354096404619	0.00495803836882862	TACSTD2/DSTN/S100A10/KANK1/ARPC2/GRHL3/SCIN/PFN1/PYCARD/TMSB10/TJP1/PAK1/F2RL1/ARPC3/DBNL/CAPG/SDC4/CDC42/CAPZA1/CAPZB/BRK1/TMSB4X/ARF1	23
BP	GO:0008637	apoptotic mitochondrial changes	11/510	108/18903	0.000161482313497948	0.00561461582315943	0.00500355454509162	SFN/TMEM14A/SLC25A5/FAM162A/PDCD5/PYCARD/GGCT/BLOC1S2/GHITM/BIK/VDAC2	11
BP	GO:0010631	epithelial cell migration	23/510	366/18903	0.000165121316476885	0.00569248741888108	0.00507295106107396	HSPB1/TACSTD2/KRT16/JUP/RAB25/EMP2/LGMN/RAB11A/NFE2L2/ANXA3/KANK1/PFN1/ANXA1/HIF1A/GIPC1/GPI/HBEGF/FGFBP1/ATP5F1B/TMSB4X/PRSS3/KLF4/S100A2	23

BP	GO:0031333	negative regulation of protein-containing complex assembly	13/510	146/18903	0.000166909291165488	0.00570577307950594	0.00508479078968547	CLDN7/CTNNBIP1/KANK1/SCIN/PFN1/TMSB10/LMO4/CAPG/CDC42/CAPZA1/CAPZB/VDAC2/TMSB4X	13
BP	GO:0070585	protein localization to mitochondrion	12/510	128/18903	0.000182059278471599	0.00607907852956455	0.00541746790245312	TSPO/TIMM8B/UBL5/HK1/PDCD5/RALA/MTCH2/OXA1L/ROMO1/TIMM17A/NDUFA13/TIMM13	12
BP	GO:0006887	exocytosis	23/510	369/18903	0.000185975789917508	0.00607907852956455	0.00541746790245312	RAB25/ANXA2/TMEM79/SYTL5/RAB11A/S100A10/VSNL1/ANXA3/SDC1/ARL8B/SCIN/ANXA1/RALA/PAK1/RAB10/F2RL1/GIPC1/SYNGR2/SDC4/RALB/VAMP8/TMEM167A/CHMP2A	23
BP	GO:0090132	epithelium migration	23/510	369/18903	0.000185975789917508	0.00607907852956455	0.00541746790245312	HSPB1/TACSTD2/KRT16/JUP/RAB25/EMP2/LGMN/RAB11A/NFE2L2/ANXA3/KANK1/PFN1/ANXA1/HIF1A/GIPC1/GPI/HBEGF/FGFBP1/ATP5F1B/TMSB4X/PRSS3/KLF4/S100A2	23
BP	GO:0046031	ADP metabolic process	10/510	92/18903	0.000186181515617106	0.00607907852956455	0.00541746790245312	EIF6/NUPR1/HK1/TPI1/ENO1/LDHA/HIF1A/GPI/PGAM1/PGK1	10
BP	GO:0097581	lamellipodium organization	10/510	92/18903	0.000186181515617106	0.00607907852956455	0.00541746790245312	ABLIM1/KANK1/ARPC2/CD44/ACTR3/CDC42/SNX1/CAPZB/BRK1/ABI1	10

BP	GO:0010644	cell communication by electrical coupling	6/510	32/18903	0.0001867956775308 67	0.006079078529564 55	0.005417467902453 12	GJB6/SRI/GJA1/ATP1B1/GJB2/CALM1	6
BP	GO:0010822	positive regulation of mitochondrion organization	9/510	76/18903	0.0002020362282114 24	0.006522883939397 42	0.005812972180118 18	SLC25A5/FAM162A/PDCD5/PYCARD/RALA/VDAC1/HIF1A/BIK/SSBP1	9
BP	GO:1902652	secondary alcohol metabolic process	13/510	149/18903	0.0002044221645731 71	0.006547947759713 86	0.005835308203812 7	SULT2B1/TM7SF2/FDFT1/MSMO1/IDH1/SQLE/DHCR7/HMGCS1/DHCR24/HMGCR/ACADVL/SC5D/INSIG1	13
BP	GO:0001676	long-chain fatty acid metabolic process	11/510	111/18903	0.0002060502358463 49	0.006548534057991 79	0.005835830692885 09	GSTA1/AKR1C3/GSTP1/MGLL/CYP2C18/ELOVL6/GSTM4/ACSL1/EPHX1/ELOVL1/CBR1	11
BP	GO:0010821	regulation of mitochondrion organization	13/510	150/18903	0.0002184347903408 24	0.006888315714003 65	0.006138632541279 45	TSPO/TMEM14A/SLC25A5/FAM162A/PDCD5/PYCARD/RALA/MTCH2/VDAC1/HIF1A/GHITM/BIK/SSBP1	13
BP	GO:0090130	tissue migration	23/510	374/18903	0.0002259441742403 1	0.007070314621612 16	0.006300823773956 49	HSPB1/TACSTD2/KRT16/JUP/RAB25/EMP2/LGMN/RAB11A/NFE2L2/ANXA3/KANK1/PFN1/ANXA1/HIF1A/GIPC1/GPI/HBEGF/FGFBP1/ATP5F1B/TMSB4X/PRSS3/KLF4/S100A2	23

BP	GO:0009132	nucleoside diphosphate metabolic process	12/510	132/18903	0.000243038934622666	0.00754719378660308	0.00672580225665296	EIF6/NUPR1/HK1/TPI1/ENO1/LDHA/HIF1A/GPI/PGAM1/GUK1/PGK1/CMPK1	12
BP	GO:0016125	sterol metabolic process	13/510	154/18903	0.000283023503042327	0.00872226977557716	0.00777298998786104	SULT2B1/TM7SF2/FDFT1/MSMO1/SQLE/DHCR7/HMGCS1/ERG28/DHCR24/HMGCR/ACADVL/SC5D/INSIG1	13
BP	GO:0045039	protein insertion into mitochondrial inner membrane	4/510	13/18903	0.000308426547448928	0.00939928014251917	0.00837631858687472	TIMM8B/ROMO1/NDUFA13/TIMM13	4
BP	GO:0010634	positive regulation of epithelial cell migration	14/510	176/18903	0.000309612472737849	0.00939928014251917	0.00837631858687472	HSPB1/RAB25/LGMN/RAB11A/NFE2L2/ANXA3/PFN1/ANXA1/HIF1A/GPI/HBEGF/FGFBP1/ATP5F1B/TMSB4X	14
BP	GO:0050891	multicellular organismal water homeostasis	8/510	64/18903	0.00031209594054155	0.00940449100831871	0.00838096233313917	SFN/CLDN4/KRT16/TMEM79/GRHL1/GRHL3/ELOVL1/KLF4	8
BP	GO:0032272	negative regulation of protein	9/510	81/18903	0.000328533549511408	0.00982701823097358	0.0087575042145301	KANK1/SCIN/PFN1/TMSB10/CAPG/CAPZA1/CAPZB/VDAC2/TMSB4X	9

		polymerization							
BP	GO:0006979	response to oxidative stress	25/510	434/18903	0.0003315837100510 95	0.00984585790137 12	0.00877429348763 712	TXN/GPX2/AKR1C3/GSTP1/HSPB1/NQO1/PRDX6/NFE2L2/MAPK13/IDH1/SDC1/GJB2/ANXA1/DHCR24/ATOX1/HMOX2/HIF1A/ROMO1/PRDX1/PON2/NDUFA12/NDUFS8/CHCHD2/GPX3/PSMB5	25
BP	GO:0043254	regulation of protein-containing complex assembly	24/510	410/18903	0.000344029191467 24	0.0101413822528169	0.00903765473236594	CLDN7/CTNNBIP1/KANK1/ARPC2/SCIN/PFN1/PYCARD/TMSB10/PAK1/LMO4/STMP1/ARPC3/DBNL/CAPG/CDC42/LGALS3/CAPZA1/BIK/RALB/CAPZB/BRK1/VDAC2/TMSB4X/HES1	24
BP	GO:0006694	steroid biosynthetic process	14/510	178/18903	0.0003475106193410 39	0.0101703107876212	0.00906343485808813	AKR1C3/TSPO/TM7SF2/FDFT1/MSMO1/SQLE/DHCR7/HMGCS1/ERG28/ASAH1/DHCR24/HMGCR/SC5D/INSIG1	14
BP	GO:1901617	organic hydroxy compound biosynthetic process	17/510	245/18903	0.0003744189206082 37	0.0108795440645308	0.00969547941785541	AKR1C3/SPTSSB/TM7SF2/FDFT1/MSMO1/SPTLC2/SQLE/DHCR7/PTS/HMGCS1/ERG28/ASAH1/DHCR24/HMGCR/GIPC1/SC5D/INSIG1	17
BP	GO:0030837	negative regulation of actin filament polymerization	8/510	66/18903	0.000386394137423 73	0.0111478819222676	0.00993461298460118	KANK1/SCIN/PFN1/TMSB10/CAPG/CAPZA1/CAPZB/TMSB4X	8

BP	GO:0008203	cholesterol metabolic process	12/510	139/18903	0.0003916763464983 56	0.0112206998419388 8	0.0099995058364739 7	SULT2B1/TM7SF2/FDFT1/MSMO1/SQLE/DHCR7/HMGCS1/DHCR24/HMGCR/ACADVL/SC5D/INSIG1	12
BP	GO:0030833	regulation of actin filament polymerization	12/510	140/18903	0.0004181515469443 92	0.0118953880627258	0.0106007650178615	KANK1/ARPC2/SCIN/PFN1/PYCARD/TMSB10/ARPC3/DBNL/CAPG/CAPZA1/CAPZB/TMSB4X	12
BP	GO:0090151	establishment of protein localization to mitochondrial membrane	6/510	37/18903	0.0004275465664853 43	0.0120781905032109	0.0107636723316924	TIMM8B/PDCD5/OXA1L/ROMO1/NDUFA13/TIMM13	6
BP	GO:0010939	regulation of necrotic cell death	7/510	52/18903	0.000466951030701 14	0.0131003916751189	0.0116746232285643	TSPO/NUPR1/HEBP2/NDUFC2/ASAH1/PELI1/YBX3	7
BP	GO:0072655	establishment of protein localization to mitochondrion	11/510	123/18903	0.0005025571227030 73	0.0140027559942199	0.0124787796005002	TSPO/TIMM8B/UBL5/HK1/PDCD5/RALA/OXA1L/ROMO1/TIMM17A/NDUFA13/TIMM13	11

BP	GO:0030104	water homeostasis	8/510	69/18903	0.000524250166583278	0.01440979511932 96	0.01284151901644 96	SFN/CLDN4/KRT16/TMEM79/GRHL1/GRHL3/ELOVL1/KLF4	8
BP	GO:0045109	intermediate filament organization	8/510	69/18903	0.000524250166583278	0.01440979511932 96	0.01284151901644 96	KRT6B/KRT6A/KRT17/KRT16/KRT6C/DSP/PKP1/KRT19	8
BP	GO:0001523	retinoid metabolic process	9/510	87/18903	0.000560224732578651	0.01529526316865 74	0.01363061800777 73	AKR1C3/ADH7/AKR1B10/RDH12/CYP2C18/ALDH1A1/UGT1A7/RDH11/AKR1C1	9
BP	GO:1901661	quinone metabolic process	6/510	39/18903	0.000573304867911082	0.01554802801774 85	0.01385587343919 83	AKR1C2/AKR1C3/AKR1B10/NQO1/AKR1C1/CBR1	6
BP	GO:0046394	carboxylic acid biosynthetic process	20/510	326/18903	0.000586400551422639	0.01569109944727 04	0.01398337382818 38	AKR1C3/GSTP1/MGLL/ALDH1A1/MGST2/ELOVL6/EIF6/GSTM4/LIPH/SCD/PYCARD/CASP1/ANXA1/UGDH/NDUFAB1/ACADVL/ELOVL1/TECR/INSIG1/CBR1	20
BP	GO:0042572	retinol metabolic process	7/510	54/18903	0.000590151970361939	0.01569109944727 04	0.01398337382818 38	AKR1C3/ADH7/AKR1B10/RDH12/CYP2C18/ALDH1A1/RDH11	7
BP	GO:190	regulation of lamellipodiu	7/510	54/18903	0.000590151970361939	0.01569109944727 04	0.01398337382818 38	KANK1/ARPC2/CD44/ACTR3/CDC42/CAPZB/BRK1	7

	274<br>3	m<br>organization							
BP	GO:<br>001<br>605<br>3	organic acid biosynthetic process	20/510	328/189<br>03	0.000633<br>04337513<br>7158	0.016658<br>59250653<br>43	0.014845<br>57071689<br>29	AKR1C3/GSTP1/MGLL/ALDH1A1/MGST2/ELOVL6/EIF6/GSTM4/LIPH/SCD/<br>PYCARD/CASP1/ANXA1/UGDH/NDUFAB1/ACADVL/ELOVL1/TECR/INSIG<br>1/CBR1	20
BP	GO:<br>000<br>683<br>9	mitochondri<br>al transport	14/510	189/189<br>03	0.000634<br>73004879<br>8628	0.016658<br>59250653<br>43	0.014845<br>57071689<br>29	TSPO/TIMM8B/HEBP2/TMEM14A/SLC25A5/UBL5/PDCD5/OXA1L/BLOC1S<br>2/ROMO1/TIMM17A/VDAC2/NDUFA13/TIMM13	14
BP	GO:<br>200<br>011<br>6	regulation of cysteine-<br>type<br>endopeptida<br>se activity	16/510	235/189<br>03	0.000676<br>96577875<br>7999	0.017555<br>65534680<br>75	0.015645<br>00259131<br>74	PERP/SFN/CARD18/LGMN/CYCS/FAM162A/PDCD5/PYCARD/CASP1/DHCR<br>24/CD44/GPI/F3/SOX2/NDUFA13/KLF4	16
BP	GO:<br>003<br>432<br>9	cell junction assembly	24/510	430/189<br>03	0.000677<br>65145563<br>8774	0.017555<br>65534680<br>75	0.015645<br>00259131<br>74	DSG1/POF1B/CLDN7/CD9/CLDN4/JUP/CDH1/PKP1/DUSP22/LRRC4/GJB6/H<br>OPX/S100A10/GJA1/GJB2/TJP1/NTRK2/PDLIM5/DBNL/CTNND1/SDC4/CDC<br>42/ACTB/CAPZA1	24
BP	GO:<br>003<br>430<br>8	primary<br>alcohol<br>metabolic<br>process	10/510	108/189<br>03	0.000681<br>85682025<br>457	0.017555<br>65534680<br>75	0.015645<br>00259131<br>74	AKR1C2/AKR1C3/ADH7/AKR1B10/RDH12/CYP2C18/ALDH1A1/RDH11/AL<br>DH3B2/AKR1C1	10
BP	GO:<br>003<br>163<br>9	plasminogen activation	5/510	27/1890<br>3	0.000692<br>12181565<br>666	0.017707<br>87135906<br>47	0.015780<br>65232122<br>83	ANXA2/S100A10/DHCR24/ENO1/PGK1	5

BP	GO:0016101	diterpenoid metabolic process	9/510	90/18903	0.000718473683292289	0.01819156309245 43	0.01621170193303 64	AKR1C3/ADH7/AKR1B10/RDH12/CYP2C18/ALDH1A1/UGT1A7/RDH11/AKR1C1	9
BP	GO:0006735	NADH regeneration	4/510	16/18903	0.000736140335963637	0.0181915630924543	0.0162117019330364	HK1/TPI1/ENO1/PGK1	4
BP	GO:0051014	actin filament severing	4/510	16/18903	0.000736140335963637	0.0181915630924543	0.0162117019330364	DSTN/SCIN/CAPG/CFL1	4
BP	GO:0061621	canonical glycolysis	4/510	16/18903	0.000736140335963637	0.0181915630924543	0.0162117019330364	HK1/TPI1/ENO1/PGK1	4
BP	GO:0061718	glucose catabolic process to pyruvate	4/510	16/18903	0.000736140335963637	0.0181915630924543	0.0162117019330364	HK1/TPI1/ENO1/PGK1	4
BP	GO:0001836	release of cytochrome c from mitochondria	7/510	56/18903	0.000737858385018426	0.0181915630924543	0.0162117019330364	SFN/FAM162A/PDCD5/PYCARD/GGCT/GHITM/BIK	7

BP	GO:0045454	cell redox homeostasis	6/510	41/18903	0.000754955663682606	0.0184705605111125	0.0164603349377094	TXN/NQO1/PRDX6/NFE2L2/GLRX3/PRDX1	6
BP	GO:0019362	pyridine nucleotide metabolic process	8/510	73/18903	0.000767336461744843	0.0184705605111125	0.0164603349377094	NQO1/TALDO1/ME1/PGD/IDH1/PGAM1/DERA/DCXR	8
BP	GO:0046496	nicotinamide nucleotide metabolic process	8/510	73/18903	0.000767336461744843	0.0184705605111125	0.0164603349377094	NQO1/TALDO1/ME1/PGD/IDH1/PGAM1/DERA/DCXR	8
BP	GO:1903578	regulation of ATP metabolic process	8/510	73/18903	0.000767336461744843	0.0184705605111125	0.0164603349377094	TSPO/EIF6/NUPR1/NDUFC2/ENO1/HIF1A/PGAM1/TMSB4X	8
BP	GO:1902903	regulation of supramolecular fiber organization	22/510	384/18903	0.000796609303228994	0.0190623920325621	0.0169877550484251	TACSTD2/DSTN/S100A10/KANK1/ARPC2/SCIN/PFN1/PYCARD/TMSB10/TJP1/PAK1/F2RL1/ARPC3/DBNL/CAPG/SDC4/CDC42/CAPZA1/CAPZB/BRK1/TMSB4X/ARF1	22
BP	GO:2001242	regulation of intrinsic apoptotic signaling pathway	13/510	172/18903	0.000813251090911706	0.0193468154258995	0.0172412234970755	HSPB1/NFE2L2/NUPR1/SLC9A3R1/EIF5A/PYCARD/CD44/ENO1/HIF1A/VDAC2/NDUFA13/HDAC1/YBX3	13

BP	GO:0072330	monocarboxylic acid biosynthetic process	15/510	218/18903	0.000885339096607939	0.02093929909884 36	0.0186603907510266	AKR1C3/GSTP1/MGLL/ELOVL6/EIF6/GSTM4/LIPH/SCD/ANXA1/NDUFAB1/ACADVL/ELOVL1/TECR/INSIG1/CBR1	15
BP	GO:0045185	maintenance of protein location	9/510	93/18903	0.000911412505823871	0.0214313645878122	0.0190989027688312	TXN/TSPO/SRI/HK1/SCIN/TMSB10/YWHAB/INSIG1/TMSB4X	9
BP	GO:0030042	actin filament depolymerization	7/510	59/18903	0.00101265275590189	0.0236751230517752	0.0210984639523661	DSTN/SCIN/F2RL1/CAPG/CAPZA1/CAPZB/CFL1	7
BP	GO:0010632	regulation of epithelial cell migration	18/510	292/18903	0.00102678459673621	0.0238683413687024	0.0212706535408091	HSPB1/TACSTD2/JUP/RAB25/EMP2/LGMN/RAB11A/NFE2L2/ANXA3/PFN1/ANXA1/HIF1A/GPI/HBEGF/FGFBP1/ATP5F1B/TMSB4X/KLF4	18
BP	GO:0016052	carbohydrate catabolic process	12/510	155/18903	0.00103574200294815	0.0239397640226879	0.0213343029793865	PGD/EIF6/NUPR1/HK1/TPI1/ENO1/LDHA/HIF1A/GPI/PGAM1/DERA/PGK1	12
BP	GO:0090150	establishment of protein localization to membrane	17/510	270/18903	0.0011143656633717	0.0256014385690416	0.0228151307850177	TIMM8B/RAB11A/CD24/ATP1B1/PDCD5/EMC2/ZDHHC3/RAB10/OXA1L/NSG1/CHMP4B/ROMO1/SEC61B/NDUFA13/TIMM13/SEC61G/KRT18	17

BP	GO:0019748	secondary metabolic process	7/510	60/18903	0.00112022027170339	0.02560143856904 16	0.0228151307850177	AKR1C2/AKR1C3/AKR1B10/NFE2L2/UGT1A7/GIPC1/AKR1C1	7
BP	GO:0001778	plasma membrane repair	5/510	30/18903	0.00114307969719705	0.0259779229508245	0.0231506408535526	ANXA2/S100A10/ARL8B/CHMP4B/CHMP2A	5
BP	GO:0032271	regulation of protein polymerization	14/510	201/18903	0.00115617913928479	0.0261296485478362	0.0232858535420866	KANK1/ARPC2/SCIN/PFN1/PYCARD/TMSB10/PAK1/ARPC3/DBNL/CAPG/CAPZA1/CAPZB/VDAC2/TMSB4X	14
BP	GO:0016264	gap junction assembly	4/510	18/18903	0.00118581390050876	0.0263600598211456	0.0234911882275075	GJB6/HOPX/GJA1/GJB2	4
BP	GO:0061620	glycolytic process through glucose-6-phosphate	4/510	18/18903	0.00118581390050876	0.0263600598211456	0.0234911882275075	HK1/TPI1/ENO1/PGK1	4
BP	GO:0072673	lamellipodium morphogenesis	4/510	18/18903	0.00118581390050876	0.0263600598211456	0.0234911882275075	KANK1/CD44/SNX1/ABI1	4

BP	GO:0034113	heterotypic cell-cell adhesion	7/510	61/18903	0.00123654924973824	0.0271907153942441	0.0242314402053968	DSC2/PERP/JUP/DSP/CD200/CD44/KLF4	7
BP	GO:0086065	cell communication involved in cardiac conduction	7/510	61/18903	0.00123654924973824	0.0271907153942441	0.0242314402053968	DSC2/JUP/DSP/SRI/GJA1/ATP1B1/CALM1	7
BP	GO:0072524	pyridine-containing compound metabolic process	8/510	79/18903	0.00129411414788611	0.0283035287827995	0.0252231416260315	NQO1/TALDO1/ME1/PGD/IDH1/PGAM1/DERA/DCXR	8
BP	GO:0006099	tricarboxylic acid cycle	5/510	31/18903	0.00133304123561714	0.0289989932967409	0.0258429159328198	IDH1/SUCLG1/SDHB/SDHC/MDH2	5
BP	GO:0006140	regulation of nucleotide metabolic process	9/510	99/18903	0.00142340937864273	0.0308001561293544	0.0274480509521028	TSPO/ME1/EIF6/NUPR1/NDUFC2/ENO1/HIF1A/PGAM1/TMSB4X	9
BP	GO:0006098	pentose-phosphate shunt	4/510	19/18903	0.0014702629474561	0.0309897910375721	0.0276170471286546	TALDO1/PGD/PGAM1/DERA	4

BP	GO:0035338	long-chain fatty-acyl-CoA biosynthetic process	4/510	19/18903	0.0014702629474561	0.03098979103757 21	0.02761704712865 46	ELOVL6/ACSL1/ELOVL1/TECR	4
BP	GO:0061615	glycolytic process through fructose-6-phosphate	4/510	19/18903	0.00147026294745 61	0.03098979103757 21	0.02761704712865 46	HK1/TPI1/ENO1/PGK1	4
BP	GO:0097202	activation of cysteine-type endopeptidase activity	4/510	19/18903	0.00147026294745 61	0.03098979103757 21	0.02761704712865 46	PERP/LGMN/CYCS/PYCARD	4
BP	GO:0099563	modification of synaptic structure	4/510	19/18903	0.00147026294745 61	0.03098979103757 21	0.02761704712865 46	CTTNBP2/PFN1/EPHA4/CDC42	4
BP	GO:0032731	positive regulation of interleukin-1 beta production	7/510	63/18903	0.00149744234485 211	0.03139997659205 35	0.02798259053538 07	HSPB1/HK1/PYCARD/CASP1/F2RL1/STMP1/TRIM16	7
BP	GO:003	diol metabolic process	5/510	32/18903	0.00154530893155 905	0.03223752171067 81	0.02872898224177 8	DEGS2/SPTSSB/SPTLC2/PTS/ASAH1	5

	4311								
BP	GO:0034754	cellular hormone metabolic process	11/510	141/18903	0.00155648755149124	0.0323050579564 61	0.028789168245627 4	AKR1C2/AKR1C3/ADH7/AKR1B10/RDH12/TSPO/CYP2C18/ALDH1A1/UGT1A7/RDH11/AKR1C1	11
BP	GO:2001243	negative regulation of intrinsic apoptotic signaling pathway	9/510	101/18903	0.00163801467929399	0.03382458738765 46	0.030143321162108	HSPB1/NFE2L2/CD44/ENO1/HIF1A/VDAC2/NDUFA13/HDAC1/YBX3	9
BP	GO:2001234	negative regulation of apoptotic signaling pathway	15/510	233/18903	0.00172044291164289	0.0353472816392085	0.0315002944587885	GSTP1/HSPB1/NFE2L2/TMEM14A/SLC25A5/TMBIM1/CD44/ENO1/HIF1A/GHITM/LGALS3/VDAC2/NDUFA13/HDAC1/YBX3	15
BP	GO:0045684	positive regulation of epidermis development	5/510	33/18903	0.00178140867666569	0.0362338524833802	0.0322903762233508	SFN/SULT2B1/TMEM79/TRIM16/KRT10	5
BP	GO:0051204	protein insertion into mitochondrial membrane	5/510	33/18903	0.00178140867666569	0.0362338524833802	0.0322903762233508	TIMM8B/PDCD5/ROMO1/NDUFA13/TIMM13	5

BP	GO:1990182	exosomal secretion	4/510	20/18903	0.00179899523110617	0.0362364180096839	0.0322926625330827	RAB11A/SDC1/SDC4/CHMP2A	4
BP	GO:0032507	maintenance of protein location in cell	7/510	65/18903	0.00179935015682305	0.0362364180096839	0.0322926625330827	TXN/TSPO/HK1/SCIN/TMSB10/INSIG1/TMSB4X	7
BP	GO:0045682	regulation of epidermis development	7/510	66/18903	0.00196698900658228	0.0394172969397868	0.0351273535839740	SFN/SULT2B1/TMEM79/GRHL1/TRIM16/KRT10/HES1	7
BP	GO:0001765	membrane raft assembly	3/510	10/18903	0.00203363636322191	0.0405531015960133	0.0361395440399187	ANXA2/EMP2/S100A10	3
BP	GO:0051604	protein maturation	19/510	336/18903	0.00205099588287553	0.0406997622026227	0.0362702429813778	PERP/FKBP1A/ANXA2/CARD18/LGMN/TMPRSS4/CYCS/S100A10/GLRX3/PYCARD/CASP1/DHCR24/ENO1/NAA20/NDUFAB1/F3/SEC11C/PGK1/PRSS3	19
BP	GO:0051205	protein insertion into membrane	7/510	67/18903	0.00214646767827282	0.0423417619941257	0.0377335373150569	TIMM8B/PDCD5/EMC2/OXA1L/ROMO1/NDUFA13/TIMM13	7
BP	GO:001	glycoside metabolic process	4/510	21/18903	0.00217537567767264	0.0423417619941257	0.0377335373150569	AKR1C2/AKR1C3/AKR1B10/AKR1C1	4

	6137								
BP	GO:0043651	linoleic acid metabolic process	4/510	21/18903	0.00217537567767264	0.0423417619941257	0.0377335373150569	GSTA1/GSTP1/CYP2C18/ELOVL1	4
BP	GO:0097734	extracellular exosome biogenesis	4/510	21/18903	0.00217537567767264	0.0423417619941257	0.0377335373150569	RAB11A/SDC1/SDC4/CHMP2A	4
BP	GO:0045921	positive regulation of exocytosis	8/510	86/18903	0.00223508204865781	0.0432967322568577	0.0385845743136716	ANXA2/S100A10/VSNL1/SDC1/F2RL1/SDC4/VAMP8/CHMP2A	8
BP	GO:0030041	actin filament polymerization	12/510	170/18903	0.00227417049081345	0.0438451448181475	0.039073300924727	KANK1/ARPC2/SCIN/PFN1/PYCARD/TMSB10/ARPC3/DBNL/CAPG/CAPZA1/CAPZB/TMSB4X	12
BP	GO:0046513	ceramide biosynthetic process	7/510	68/18903	0.00233833573238516	0.0448695743365228	0.0399862376481355	DEGS2/B4GALT4/SPTSSB/SPTLC2/ASAH1/CERS3/ELOVL1	7
BP	GO:0034332	adherens junction organization	6/510	51/18903	0.00241032405042702	0.0460337945405499	0.0410237510732427	DSP/CDH1/TJP1/EPHA4/CDC42/ACTB	6

BP	GO:0032535	regulation of cellular component size	20/510	367/18903	0.00243586318527373	0.0463041655966987	0.04126469655721995	DSTN/CDH1/RAB11A/KANK1/SLC9A3R1/ARPC2/SCIN/PFN1/PYCARD/TMSB10/F2RL1/ARPC3/CLCN3/DBNL/BLOC1S2/CAPG/CAPZA1/CAPZB/BRK1/TMSB4X	20
BP	GO:0032984	protein-containing complex disassembly	15/510	242/18903	0.00248237303393532	0.0469688069862739	0.04185700234454 47	DSTN/SCIN/EIF5A/OGFOD1/CALM1/F2RL1/PYM1/HMGA1/CHMP4B/CAPG/CAPZA1/CAPZB/VAMP8/CFL1/CHMP2A	15
BP	GO:1903034	regulation of response to wounding	12/510	172/18903	0.0025057555504 25	0.0471917295330041	0.0420556633316944	CD9/CLDN4/ANXA2/NFE2L2/KANK1/ANXA1/F2RL1/EPHA4/SERPINB2/HBEGF/F3/KLF4	12
BP	GO:0006740	NADPH regeneration	4/510	22/18903	0.00260271891111134	0.0485681675706465	0.0432822980679259	TALDO1/PGD/PGAM1/DERA	4
BP	GO:1902644	tertiary alcohol metabolic process	4/510	22/18903	0.00260271891111134	0.0485681675706465	0.0432822980679259	AKR1C2/AKR1C3/AKR1B10/AKR1C1	4
BP	GO:0006636	unsaturated fatty acid biosynthetic process	6/510	52/18903	0.00266380748778257	0.0494811363483995	0.0440959047725218	AKR1C3/ELOVL6/SCD/ANXA1/ELOVL1/CBR1	6
CC	GO:009	inner mitochondrial membrane	49/526	158/19869	8.57320660683215e-39	4.38090857609123e-36	3.30294065063218e-36	COX6C/COX6A1/COX7B/ATP5F1E/COX5B/UQCRQ/ATP5PD/COX5A/UQCR10/COX7A2/NDUFA4/NDUFB2/ATP5MF/NDUFC2/COX6B1/ATP5ME/ATP5PB/UQCR11/COX8A/ATP5MC1/NDUFS6/NDUFB9/ATP5MC3/UQCRB/NDUFB	49

	8800	protein complex						8/NDUFAB1/SDHB/ATP5PF/ROMO1/UQCRC2/TIMM17A/NDUFB3/SDHC/NDUFC1/SMDT1/ATP5F1B/COX4I1/NDUFA3/ATP5F1C/COX7C/CYC1/NDUFA13/NDUFA12/NDUFB1/ATP5PO/NDUFS8/ATP5MG/NDUFA1/NDUFS5	
CC	GO:0098798	mitochondrial protein-containing complex	59/526	295/19869	9.37960986664462e-35	2.3964903209277e-32	1.80680905852207e-32	COX6C/TIMM8B/COX6A1/COX7B/ATP5F1E/COX5B/UQCRQ/ATP5PD/COX5A/SLC25A5/UQCR10/COX7A2/NDUFA4/NDUFB2/ATP5MF/NDUFC2/COX6B1/ATP5ME/ATP5PB/UQCR11/COX8A/ATP5MC1/SUCLG1/NDUFS6/NDUFB9/ATP5MC3/UQCRB/VDAC1/NDUFB8/NDUFAB1/MRPL14/SDHB/ATP5PF/ROMO1/UQCRC2/TIMM17A/MRPL33/NDUFB3/SDHC/NDUFC1/SMDT1/ATP5F1B/MRPL36/COX4I1/NDUFA3/ATP5F1C/COX7C/CYC1/MRPL41/NDUFA13/NDUFA12/NDUFB1/TIMM13/ATP5PO/MRPL52/NDUFS8/ATP5MG/NDUFA1/NDUFS5	59
CC	GO:0070469	respirasome	37/526	101/19869	1.41743922967463e-32	2.41437148787913e-30	1.82029037916111e-30	COX6C/HIGD1A/COX6A1/COX7B/CYCS/COX5B/UQCRQ/COX5A/UQCR10/COX7A2/NDUFA4/NDUFB2/NDUFC2/COX6B1/UQCR11/COX8A/NDUFS6/NDUFB9/UQCRB/STMP1/NDUFB8/NDUFAB1/SDHB/UQCRC2/NDUFB3/SDHC/NDUFC1/COX4I1/NDUFA3/COX7C/CYC1/NDUFA13/NDUFA12/NDUFB1/NDUFS8/NDUFA1/NDUFS5	37
CC	GO:0005746	mitochondrial respirasome	35/526	94/19869	3.66707301116676e-31	4.68468577176553e-29	3.53197032128167e-29	COX6C/COX6A1/COX7B/COX5B/UQCRQ/COX5A/UQCR10/COX7A2/NDUFA4/NDUFB2/NDUFC2/COX6B1/UQCR11/COX8A/NDUFS6/NDUFB9/UQCRB/STMP1/NDUFB8/NDUFAB1/SDHB/UQCRC2/NDUFB3/SDHC/NDUFC1/COX4I1/NDUFA3/COX7C/CYC1/NDUFA13/NDUFA12/NDUFB1/NDUFS8/NDUFA1/NDUFS5	35
CC	GO:0098803	respiratory chain complex	34/526	91/19869	2.30802159646691e-30	2.35879807158918e-28	1.77839137748819e-28	COX6C/COX6A1/COX7B/COX5B/UQCRQ/COX5A/UQCR10/COX7A2/NDUFA4/NDUFB2/NDUFC2/COX6B1/UQCR11/COX8A/NDUFS6/NDUFB9/UQCRB/NDUFB8/NDUFAB1/SDHB/UQCRC2/NDUFB3/SDHC/NDUFC1/COX4I1/NDUFA3/COX7C/CYC1/NDUFA13/NDUFA12/NDUFB1/NDUFS8/NDUFA1/NDUFS5	34

CC	GO:0005743	mitochondrial inner membrane	67/526	498/19869	1.71633535948934e-28	1.46174561449842e-26	1.1020679676721e-26	COX6C/TIMM8B/COA3/HIGD1A/COX6A1/COX7B/ATP5F1E/CYCS/COX5B/UQCRQ/ATP5PD/COX5A/SLC25A5/SQOR/UQCR10/COX7A2/NDUFA4/NDUFB2/ATP5MF/NDUFC2/COX6B1/ATP5ME/ATP5PB/UQCR11/COX8A/ATP5MC1/NDUFS6/NDUFB9/ATP5MC3/UQCRB/BDH1/STMP1/NDUFB8/OXA1L/NDUFAB1/MRPL14/SDHB/ATP5PF/GHITM/ROMO1/UQCRC2/TIMM17A/MRPL33/ACADVL/NDUFB3/SDHC/NDUFC1/LGALS3/SMDT1/ATP5F1B/MRPL36/COX4I1/NDUFA3/ATP5F1C/COX7C/CYC1/MRPL41/NDUFA13/NDUFA12/NDUFB1/TIMM13/ATP5PO/MRPL52/NDUFS8/ATP5MG/NDUFA1/NDUFS5	67
CC	GO:0001533	cornified envelope	23/526	59/19869	1.96222867955734e-21	1.43242693607686e-19	1.07996345371126e-19	CSTA/DSC2/SCEL/KRT17/DSG1/SERPINB5/HSPB1/KRT16/JUP/ANXA2/DSP/DSG3/SPRR1B/PKP1/PI3/SPRR1A/PPL/ANXA1/SERPINB2/KLK7/IVL/KRT10/SPRR3	23
CC	GO:0070069	cytochrome complex	17/526	42/19869	1.64880916880305e-16	1.05317685657295e-14	7.94031783923572e-15	COX6C/COX6A1/COX7B/COX5B/UQCRQ/COX5A/UQCR10/COX7A2/NDUFA4/COX6B1/UQCR11/COX8A/UQCRB/UQCRC2/COX4I1/COX7C/CYC1	17
CC	GO:1990204	oxidoreductase complex	25/526	125/19869	2.35237144145202e-15	1.33562422953554e-13	1.00698005563911e-13	UQCRQ/UQCR10/NDUFA4/NDUFB2/NDUFC2/UQCR11/NDUFS6/NDUFB9/UQCRB/NDUFB8/LDHA/NDUFAB1/SDHB/UQCRC2/NDUFB3/SDHC/NDUFC1/NDUFA3/CYC1/NDUFA13/NDUFA12/NDUFB1/NDUFS8/NDUFA1/NDUFS5	25
CC	GO:0005753	mitochondrial proton-transporting ATP synthase complex	12/526	21/19869	2.47753828120329e-14	1.26602206169488e-12	9.54504222021478e-13	ATP5F1E/ATP5PD/ATP5MF/ATP5ME/ATP5PB/ATP5MC1/ATP5MC3/ATP5PF/ATP5F1B/ATP5F1C/ATP5PO/ATP5MG	12

CC	GO:0045259	proton-transporting ATP synthase complex	12/526	22/19869	5.32046670829686e-14	2.47159862539972e-12	1.86343618682933e-12	ATP5F1E/ATP5PD/ATP5MF/ATP5ME/ATP5PB/ATP5MC1/ATP5MC3/ATP5PF/ATP5F1B/ATP5F1C/ATP5PO/ATP5MG	12
CC	GO:0005747	mitochondrial respiratory chain complex I	16/526	49/19869	6.94671664114661e-14	2.53555157401851e-12	1.91165285012005e-12	NDUFA4/NDUFB2/NDUFC2/NDUFS6/NDUFB9/NDUFB8/NDUFAB1/NDUFB3/NDUFC1/NDUFA3/NDUFA13/NDUFA12/NDUFB1/NDUFS8/NDUFA1/NDUFS5	16
CC	GO:0030964	NADH dehydrogenase complex	16/526	49/19869	6.94671664114661e-14	2.53555157401851e-12	1.91165285012005e-12	NDUFA4/NDUFB2/NDUFC2/NDUFS6/NDUFB9/NDUFB8/NDUFAB1/NDUFB3/NDUFC1/NDUFA3/NDUFA13/NDUFA12/NDUFB1/NDUFS8/NDUFA1/NDUFS5	16
CC	GO:0045271	respiratory chain complex I	16/526	49/19869	6.94671664114661e-14	2.53555157401851e-12	1.91165285012005e-12	NDUFA4/NDUFB2/NDUFC2/NDUFS6/NDUFB9/NDUFB8/NDUFAB1/NDUFB3/NDUFC1/NDUFA3/NDUFA13/NDUFA12/NDUFB1/NDUFS8/NDUFA1/NDUFS5	16
CC	GO:0005751	mitochondrial respiratory chain complex IV	12/526	25/19869	3.97958853561827e-13	1.35571316113396e-11	1.02212589756932e-11	COX6C/COX6A1/COX7B/COX5B/COX5A/COX7A2/NDUFA4/COX6B1/COX8A/UQCRC2/COX4I1/COX7C	12
CC	GO:0033177	proton-transporting two-sector ATPase	12/526	26/19869	7.21430640813495e-13	2.3040691090981e-11	1.73712904301144e-11	ATP5PD/ATP5MF/ATP5ME/ATP5PB/ATP5MC1/ATP5MC3/ATP5PF/ATP6V0D1/ATP6V0E1/ATP6V0B/ATP5PO/ATP5MG	12

		complex, proton-transporting domain							
CC	GO:0016469	proton-transporting two-sector ATPase complex	15/526	50/19869	1.73120957815592e-12	5.20381232022163e-11	3.92336040622333e-11	ATP5F1E/ATP5PD/ATP5MF/ATP5ME/ATP5PB/ATP5MC1/ATP5MC3/ATP5PF/ATP6V0D1/ATP5F1B/ATP5F1C/ATP6V0E1/ATP6V0B/ATP5PO/ATP5MG	15
CC	GO:0045277	respiratory chain complex IV	12/526	28/19869	2.16536294992756e-12	6.14722481896103e-11	4.63463648931865e-11	COX6C/COX6A1/COX7B/COX5B/COX5A/COX7A2/NDUFA4/COX6B1/COX8A/UQCRC2/COX4I1/COX7C	12
CC	GO:0045263	proton-transporting ATP synthase complex, coupling factor F(o)	9/526	14/19869	1.06216843462462e-11	2.85667405312202e-10	2.1537598175768e-10	ATP5PD/ATP5MF/ATP5ME/ATP5PB/ATP5MC1/ATP5MC3/ATP5PF/ATP5PO/ATP5MG	9
CC	GO:0000276	mitochondrial proton-transporting ATP synthase complex,	8/526	11/19869	3.52202041964532e-11	8.99876217219378e-10	6.7845235452115e-10	ATP5PD/ATP5ME/ATP5PB/ATP5MC1/ATP5MC3/ATP5PF/ATP5PO/ATP5MG	8

		coupling factor F(o)							
CC	GO:1990351	transporter complex	35/526	405/19869	9.22242847491322e-10	2.24412426222888e-08	1.69193424652543e-08	FKBP1A/CLDN4/TIMM8B/UQCRQ/UQCR10/ATP1B1/NDUFA4/NDUFB2/NDUFC2/UQCR11/SCN9A/NDUFS6/DLG3/NDUFB9/UQCRB/CALM1/KCNK1/NDUFB8/NDUFAB1/UQCRC2/NDUFB3/NDUFC1/SMDT1/ATP8B1/NDUFA3/CLIC1/CYC1/NDUFA13/NDUFA12/NDUFB1/TIMM13/NDUFS8/NDUFA1/CLIC3/NDUFS5	35
CC	GO:0000502	proteasome complex	13/526	59/19869	3.60007713467948e-09	8.36199734464188e-08	6.30444129805115e-08	HSPB1/PSMA7/PSMB6/TXNL1/PSMD8/PSMD11/PSMD1/PSMD7/PSMA3/PSMA1/PSMB3/PSMB1/PSMB5	13
CC	GO:0030057	desmosome	9/526	25/19869	8.35535966030026e-09	1.84020555772648e-07	1.38740392239755e-07	DSC2/PERP/DSG1/POF1B/JUP/DSP/DSG3/PKP1/PPL	9
CC	GO:1902495	transmembrane transporter complex	32/526	379/19869	8.64284410673886e-09	1.84020555772648e-07	1.38740392239755e-07	FKBP1A/CLDN4/UQCRQ/UQCR10/ATP1B1/NDUFA4/NDUFB2/NDUFC2/UQCR11/SCN9A/NDUFS6/DLG3/NDUFB9/UQCRB/CALM1/KCNK1/NDUFB8/NDUFAB1/UQCRC2/NDUFB3/NDUFC1/SMDT1/NDUFA3/CLIC1/CYC1/NDUFA13/NDUFA12/NDUFB1/NDUFS8/NDUFA1/CLIC3/NDUFS5	32
CC	GO:0005912	adherens junction	21/526	179/19869	1.24039743667237e-08	2.53537236055832e-07	1.91151773398773e-07	DSC2/S100A11/POF1B/JUP/ANXA2/CDH1/PKP1/NECTIN4/TRIM29/FRMD4A/ANXA1/TJP1/DLG3/RAB10/EPHA4/PDLIM5/CTNND1/PDZD11/ACTB/PDLIM4/KRT18	21
CC	GO:0101002	ficolin-1-rich granule	21/526	185/19869	2.229524072294e-08	4.38187231131628e-07	3.3036672488243e-07	CSTB/GSTP1/DSG1/CALML5/DYNLL1/JUP/DSP/PKP1/DYNLT1/IDH1/ASAH1/DBNL/GPI/OSTF1/PGAM1/SERPINB6/PSMD11/PSMD7/DERA/LGALS3/PSMB1	21

CC	GO:1905369	endopeptidase complex	14/526	82/19869	2.95090366914538e-08	5.58485842567886e-07	4.21064617117821e-07	HSPB1/CAPNS2/PSMA7/PSMB6/TXNL1/PSMD8/PSMD11/PSMD1/PSMD7/PSMA3/PSMA1/PSMB3/PSMB1/PSMB5	14
CC	GO:0034774	secretory granule lumen	28/526	322/19869	3.99540877418288e-08	7.29162101288376e-07	5.49744214793584e-07	PLAC8/CSTB/S100A11/GSTP1/PRDX6/JUP/SERPINB3/ANXA2/DYNLT1/CREG1/APRT/HEBP2/IDH1/SLPI/PYCARD/DBNL/GPI/OSTF1/PGAM1/TOLLIP/PSMD11/CTSC/PSMD1/PSMD7/DERA/TRAPPC1/TMSB4X/PSMB1	28
CC	GO:0060205	cytoplasmic vesicle lumen	28/526	325/19869	4.86706788333775e-08	8.57610927029513e-07	6.46586876697501e-07	PLAC8/CSTB/S100A11/GSTP1/PRDX6/JUP/SERPINB3/ANXA2/DYNLT1/CREG1/APRT/HEBP2/IDH1/SLPI/PYCARD/DBNL/GPI/OSTF1/PGAM1/TOLLIP/PSMD11/CTSC/PSMD1/PSMD7/DERA/TRAPPC1/TMSB4X/PSMB1	28
CC	GO:0031983	vesicle lumen	28/526	327/19869	5.54339434680117e-08	9.442248370718e-07	7.11888537168151e-07	PLAC8/CSTB/S100A11/GSTP1/PRDX6/JUP/SERPINB3/ANXA2/DYNLT1/CREG1/APRT/HEBP2/IDH1/SLPI/PYCARD/DBNL/GPI/OSTF1/PGAM1/TOLLIP/PSMD11/CTSC/PSMD1/PSMD7/DERA/TRAPPC1/TMSB4X/PSMB1	28
CC	GO:1905368	peptidase complex	16/526	118/19869	8.97525195337586e-08	1.47946895102421e-06	1.11543029369629e-06	HSPB1/CAPNS2/PSMA7/PSMB6/TXNL1/F3/PSMD8/PSMD11/PSMD1/PSMD7/PSMA3/SEC11C/PSMA1/PSMB3/PSMB1/PSMB5	16
CC	GO:0005839	proteasome core complex	7/526	20/19869	5.03279355606712e-07	8.03674220984468e-06	6.05921855763344e-06	PSMA7/PSMB6/PSMA3/PSMA1/PSMB3/PSMB1/PSMB5	7
CC	GO:000	mitochondrial respiratory	6/526	14/19869	8.3865874503893e-07	1.26045476092616e-05	9.50306813263927e-06	UQCRQ/UQCR10/UQCR11/UQCRB/UQCRC2/CYC1	6

	5750	chain complex III							
CC	GO:0045275	respiratory chain complex III	6/526	14/19869	8.3865874503893e-07	1.26045476092616e-05	9.50306813263927e-06	UQCRQ/UQCR10/UQCR11/UQCRB/UQCRC2/CYC1	6
CC	GO:1904813	ficolin-1-rich granule lumen	15/526	124/19869	1.01472843954566e-06	1.48150352173666e-05	1.11696423721417e-05	CSTB/GSTP1/CALML5/JUP/DYNLT1/IDH1/ASAH1/DBNL/GPI/OSTF1/PGAM1/PSMD11/PSMD7/DERA/PSMB1	15
CC	GO:0030027	lamellipodium	19/526	203/19869	2.06871669225587e-06	2.93642841595208e-05	2.2138897934668e-05	CDH1/ABLIM1/DUSP22/ARPC2/PAK1/CD44/ARPC3/DBNL/CAPG/ACTR3/CTNND1/ACTB/SNX1/PDLIM4/CAPZB/BRK1/CTTNBP2NL/ABI1/CFL1	19
CC	GO:0005775	vacuolar lumen	17/526	176/19869	4.63587807629975e-06	6.40252350537615e-05	4.82711628997357e-05	PLAC8/SERPINB13/PRDX6/SERPINB3/ANXA2/LGMN/CREG1/HEBP2/SDC1/PYCARD/ASAH1/NSG1/TOLLIP/CTSC/SDC4/PSMD1/TRAPPC1	17
CC	GO:0016328	lateral plasma membrane	10/526	64/19869	6.51579324858092e-06	8.7620272369075e-05	6.60603969246708e-05	DSG1/CLDN7/CLDN4/TACSTD2/JUP/CDH1/ATP1B1/GJB2/ANXA1/NSG1	10
CC	GO:0031252	cell leading edge	28/526	421/19869	8.51744711547152e-06	0.000111600396820665	8.41399634078968e-05	S100A11/CDH1/ABLIM1/DUSP22/KANK1/SLC9A3R1/ARPC2/APPL2/ATP2B1/PAK1/LMO4/CD44/ARPC3/CLCN3/DBNL/CAPG/ACTR3/CTNND1/CDC42/ACTB/SNX1/PDLIM4/CAPZB/BRK1/CTTNBP2NL/ABI1/CFL1/ARF1	28

CC	GO:0035578	azurophil granule lumen	11/526	91/19869	2.81992929258195e-05	0.000360245967127345	0.000271603716074999	PLAC8/PRDX6/SERPINB3/ANXA2/CREG1/HEBP2/PYCARD/TOLLIP/CTSC/PSMD1/TRAPPC1	11
CC	GO:0005759	mitochondrial matrix	28/526	483/19869	9.80842653633708e-05	0.00122246486830933	0.000921664727162868	TST/DECR1/ATP5F1E/SLC25A5/ATP5PB/SUCLG1/BDH1/VDAC1/NDUFB8/PCCB/OXA1L/NDUFAB1/MRPL14/MRPL33/ACADVL/SMDT1/C1QBP/ATP5F1B/MRPL36/VDAC2/ATP5F1C/MDH2/ETHE1/MRPL41/REXO2/MRPL52/NDUFS8/SSBP1	28
CC	GO:0019774	proteasome core complex, beta-subunit complex	4/526	11/19869	0.000138196920580879	0.00168139586706736	0.00126767100081709	PSMB6/PSMB3/PSMB1/PSMB5	4
CC	GO:0140534	endoplasmic reticulum protein-containing complex	12/526	127/19869	0.000142290860013081	0.00169094487131824	0.00127487037367901	FKBP1A/SPTSSB/ELOVL6/SPTLC2/EMC2/OST4/SEC61B/SEC11C/INSIG1/SSR4/SEC61G/RPN2	12
CC	GO:0043296	apical junction complex	13/526	151/19869	0.000194802718955394	0.0022623679405956	0.00170568887889172	POF1B/CLDN7/CLDN4/JUP/CDH1/FRMD4A/TJP1/DLG3/CTNND1/FRMD6/ACTB/CCND1/YBX3	13
CC	GO:0005766	primary lysosome	13/526	155/19869	0.000252238173413164	0.0028020371003071	0.00211256685284252	PLAC8/PRDX6/SERPINB3/ANXA2/CREG1/HEBP2/NDUFC2/PYCARD/TOLLIP/CTSC/PSMD1/TRAPPC1/VAMP8	13

CC	GO:0042582	azurophil granule	13/526	155/19869	0.000252238173413164	0.0028020371003071	0.00211256685284252	PLAC8/PRDX6/SERPINB3/ANXA2/CREG1/HEBP2/NDUFC2/PYCARD/TOLLIP/CTSC/PSMD1/TRAPPC1/VAMP8	13
CC	GO:0005925	focal adhesion	24/526	422/19869	0.000400553888844318	0.00435495823828609	0.0032833756621953	HSPB1/YWHAZ/CD9/JUP/GJA1/ARPC2/PFN1/ANXA1/RALA/PAK1/CD44/RAB10/ARPC3/HMGA1/ALCAM/ACTR3/YWHAB/SDC4/CDC42/ACTB/REXO2/CFL1/YWHAQ/ARF1	24
CC	GO:0070820	tertiary granule	13/526	164/19869	0.00043631559109913	0.00464494306357615	0.00350200671803249	CSTB/DSG1/DYNLL1/DSP/PKP1/IDH1/ASAH1/DBNL/SERPINB6/PLD1/LGALS3/VAMP8/PRSS3	13
CC	GO:0030016	myofibril	16/526	233/19869	0.000505821818986831	0.0052749989694341	0.00397703084316177	FKBP1A/HSPB1/JUP/SRI/GLRX3/NEBL/PAK1/ENO1/CALM1/PDLIM5/SDC4/PDLIM4/CAPZB/MYL12B/ARF1/KRT19	16
CC	GO:0005665	RNA polymerase II, core complex	4/526	15/19869	0.000525393439032114	0.00536952094690821	0.00404829470917376	POLR2J3/POLR2I/POLR2L/POLR2E	4
CC	GO:0030055	cell-substrate junction	24/526	432/19869	0.000559098463578432	0.00560194735075645	0.00422353018926122	HSPB1/YWHAZ/CD9/JUP/GJA1/ARPC2/PFN1/ANXA1/RALA/PAK1/CD44/RAB10/ARPC3/HMGA1/ALCAM/ACTR3/YWHAB/SDC4/CDC42/ACTB/REXO2/CFL1/YWHAQ/ARF1	24
CC	GO:003	sarcomere	15/526	214/19869	0.000606264936376373	0.00595771889400628	0.00449176045979256	FKBP1A/HSPB1/JUP/SRI/GLRX3/NEBL/PAK1/ENO1/CALM1/PDLIM5/PDLIM4/CAPZB/MYL12B/ARF1/KRT19	15

	0017								
CC	GO:0030018	Z disc	11/526	130/19869	0.000687632714566566	0.006629817304594 63	0.004998482095955 57	FKBP1A/HSPB1/JUP/SRI/GLRX3/NEBL/PAK1/PDLIM5/PDLIM4/MYL12B/KRT19	11
CC	GO:0043292	contractile fiber	16/526	242/19869	0.000761934418661045	0.007210157183996 19	0.005436023337815 64	FKBP1A/HSPB1/JUP/SRI/GLRX3/NEBL/PAK1/ENO1/CALM1/PDLIM5/SDC4/PDLIM4/CAPZB/MYL12B/ARF1/KRT19	16
CC	GO:0005884	actin filament	10/526	114/19869	0.000901962339880 35	0.008380050103251 98	0.006318051988444 18	POF1B/DUSP22/GJB6/ANXA1/PLS3/PAK1/PDLIM5/DBNL/ACTB/PDLIM4	10
CC	GO:0098978	glutamatergic synapse	19/526	324/19869	0.001093745625516 82	0.009980428832840 95	0.007524640957502 91	MAL2/YWHAZ/CDH1/CTTNBP2/LRRC4/RAB11A/PLEKHA5/ARPC2/PFN1/ATP2B1/DLG3/GUCY1A1/EPHA4/CLCN3/GIPC1/NSG1/CTNND1/ACTB/C1QBP	19
CC	GO:0009295	nucleoid	6/526	45/19869	0.001134137359899 27	0.009992141222560 84	0.007533471392434 38	SLC25A5/VDAC1/ACADVL/ATP5F1B/VDAC2/SSBP1	6
CC	GO:0042645	mitochondrial nucleoid	6/526	45/19869	0.001134137359899 27	0.009992141222560 84	0.007533471392434 38	SLC25A5/VDAC1/ACADVL/ATP5F1B/VDAC2/SSBP1	6

CC	GO:0005838	proteasome regulatory particle	4/526	19/19869	0.00137163021497678	0.0116817173308856	0.00880730980142986	PSMD8/PSMD11/PSMD1/PSMD7	4
CC	GO:0033178	proton-transporting two-sector ATPase complex, catalytic domain	4/526	19/19869	0.00137163021497678	0.0116817173308856	0.00880730980142986	ATP5F1E/ATP5F1B/ATP5F1C/ATP5PO	4
CC	GO:0005921	gap junction	5/526	32/19869	0.00142224146506495	0.0119141866991507	0.00898257767409444	GJB6/GJA1/GJB2/GJB5/TJP1	5
CC	GO:0031674	I band	11/526	143/19869	0.0015042276993761	0.0123977476513095	0.00934715344603826	FKBP1A/HSPB1/JUP/SRI/GLRX3/NEBL/PAK1/PDLIM5/PDLIM4/MYL12B/KRT19	11
CC	GO:0005758	mitochondrial intermembrane space	8/526	84/19869	0.0017116284632464	0.0138832086463319	0.0104671013792511	TIMM8B/CYCS/STMP1/REXO2/TIMM13/CHCHD2/COX17/NDUFS5	8
CC	GO:0030667	secretory granule membrane	18/526	313/19869	0.00181503123819741	0.0144918900424825	0.0109260104141489	DSG1/CD9/DYNLL1/DSP/PKP1/SRI/NDUFC2/TMBIM1/ANXA7/CD44/RAB10/HMOX2/SERPINB6/PLD1/RAB18/LGALS3/RAB6A/VAMP8	18

CC	GO:0014704	intercalated disc	6/526	50/19869	0.00197975931208738	0.01549048904208 29	0.01167889378803 66	DSC2/JUP/DSP/GJA1/ATP1B1/PAK1	6
CC	GO:0005922	connexin complex	4/526	21/19869	0.00203104259455882	0.01549048904208 29	0.01167889378803 66	GJB6/GJA1/GJB2/GJB5	4
CC	GO:0016327	apicolateral plasma membrane	4/526	21/19869	0.00203104259455882	0.01549048904208 29	0.01167889378803 66	CLDN7/CLDN4/JUP/KRT19	4
CC	GO:1904090	peptidase inhibitor complex	3/526	11/19869	0.00259736649322731	0.01951844526528 17	0.01471572966751 08	CSTA/CASP1/SERPINB6	3
CC	GO:0070160	tight junction	10/526	132/19869	0.00271636005660088	0.02011681143366 74	0.01516686164326 35	POF1B/CLDN7/CLDN4/GJA1/FRMD4A/TJP1/DLG3/ACTB/CCND1/YBX3	10
CC	GO:0022624	proteasome accessory complex	4/526	23/19869	0.00288190365352716	0.02103789667074 82	0.01586130431866 07	PSMD8/PSMD11/PSMD1/PSMD7	4
CC	GO:004	apical part of cell	22/526	435/19869	0.00302516057380502	0.02177263455231 5	0.01641525233525 04	MAL2/DSG1/CLDN4/EMP2/LGMN/GJB6/GJA1/SLC9A3R1/ATP1B1/ATP2B1/ANXA1/TJP1/UPK1B/CD44/KCNK1/CLCN3/ATP6V0D1/PLD1/RAB18/CDC42/ATP8B1/MYL12B	22

	5177								
CC	GO:0098562	cytoplasmic side of membrane	13/526	204/19869	0.00317257419769594	0.02251646409753 64	0.0169760549174958	TGM3/DSG1/FKBP1A/JUP/CDH1/SAMD12/ATP2B1/MIEN1/BLOC1S2/CHMP4B/AP2S1/GNG5/RPS26	13
CC	GO:0030867	rough endoplasmic reticulum membrane	4/526	24/19869	0.00338681691456473	0.0234473084766 76	0.0176778553969789	SEC61B/RPS26/SSR4/SEC61G	4
CC	GO:0033179	proton-transporting V-type ATPase, V0 domain	3/526	12/19869	0.00339550064045797	0.0234473084766 76	0.0176778553969789	ATP6V0D1/ATP6V0E1/ATP6V0B	3
CC	GO:0031970	organelle envelope lumen	8/526	94/19869	0.00347681616888699	0.02368870749735	0.0178598556885984	TIMM8B/CYCS/STMP1/REXO2/TIMM13/CHCHD2/COX17/NDUFS5	8
CC	GO:0045335	phagocytic vesicle	10/526	139/19869	0.00393975900650643	0.0264896954253261	0.0199716315288276	RAB11A/ANXA3/APPL2/RAB10/CLCN3/ATP6V0D1/CDC42/ATP6V0E1/ATP6V0B/VAMP8	10
CC	GO:0005938	cell cortex	17/526	312/19869	0.00415754697064847	0.0275909935324853	0.0208019438312691	SPINK5/DSTN/CDH1/CTTNBP2/GLRX3/CLTB/SCIN/PFN1/UTRN/ENO1/DBNL/GIPC1/CTNND1/ACTB/CAPZB/MYL12B/KRT19	17

CC	GO:0030670	phagocytic vesicle membrane	7/526	77/19869	0.00426193146711802	0.0279211151243245	0.0210508355865748	ANXA3/APPL2/RAB10/ATP6V0D1/ATP6V0E1/ATP6V0B/VAMP8	7
CC	GO:0046930	pore complex	4/526	26/19869	0.00457036999158478	0.0295627729835421	0.0222885465279151	SLC25A5/VDAC1/PDZD11/VDAC2	4
CC	GO:0005882	intermediate filament	13/526	215/19869	0.00493983040572022	0.031530301153299	0.0237719440150529	KRT6B/KRT6A/KRT17/KRT16/JUP/KRT6C/DSP/PKP1/EIF6/PPL/KRT10/KRT18/KRT19	13
CC	GO:0031305	integral component of mitochondrial inner membrane	6/526	60/19869	0.00499795380316482	0.031530301153299	0.0237719440150529	COA3/STMP1/OXA1L/GHITM/TIMM17A/SMDT1	6
CC	GO:0031304	intrinsic component of mitochondrial inner membrane	6/526	61/19869	0.00542212473728668	0.0333819968765481	0.0251680108287498	COA3/STMP1/OXA1L/GHITM/TIMM17A/SMDT1	6
CC	GO:010	ficolin-1-rich granule membrane	6/526	61/19869	0.00542212473728668	0.0333819968765481	0.0251680108287498	DSG1/DYNLL1/DSP/PKP1/SERPINB6/LGALS3	6

	1003								
CC	GO:0005903	brush border	8/526	102/19869	0.00570765015052058	0.03472153841566 68	0.02617794429938 01	SLC9A3R1/SCIN/KCNK1/ACTR3/DCXR/CAPZB/CLIC1/MYL12B	8
CC	GO:0009925	basal plasma membrane	14/526	254/19869	0.00802299435541106	0.04823235430135 35	0.03636428401338 01	CLCA2/CLDN7/CLDN4/TACSTD2/ANXA2/DSP/ATP1B1/ATP2B1/ANXA1/TJP1/CD44/DLG3/HPGD/PDZD11	14
MF	GO:0009055	electron transfer activity	30/520	122/18432	3.84326918997627e-20	1.9907364009106e-17	1.67516412558052e-17	GPX2/ME1/COX6A1/COX7B/CYCS/COX5B/CYB5A/COX5A/UQCR10/NDUFA4/NDUFB2/NDUFC2/COX6B1/UQCR11/COX8A/NDUFS6/NDUFB9/NDUFB8/SDHB/NDUFB3/SDHC/NDUFC1/COX4I1/NDUFA3/CYC1/NDUFA12/NDUFB1/NDUFS8/NDUFA1/NDUFS5	30
MF	GO:0015453	oxidoreduction-driven active transmembrane transporter activity	24/520	71/18432	5.91600713494978e-20	1.9907364009106e-17	1.67516412558052e-17	COX6A1/COX7B/COX5B/CYB5A/COX5A/UQCR10/NDUFA4/NDUFB2/NDUFC2/COX6B1/COX8A/NDUFS6/NDUFB9/NDUFB8/NDUFB3/NDUFC1/COX4I1/NDUFA3/CYC1/NDUFA12/NDUFB1/NDUFS8/NDUFA1/NDUFS5	24
MF	GO:0016655	oxidoreductase activity, acting on NAD(P)H, quinone or similar	20/520	57/18432	3.26807291512983e-17	7.33137690627459e-15	6.16920430996438e-15	AKR1C2/AKR1C3/NQO1/NDUFA4/NDUFB2/NDUFC2/NDUFS6/NDUFB9/NDUFB8/AKR1C1/NDUFB3/NDUFC1/DCXR/NDUFA3/NDUFA12/NDUFB1/NDUFS8/NDUFA1/NDUFS5/CBR1	20

		compound as acceptor							
MF	GO:0015399	primary active transmembrane transporter activity	30/520	169/18432	6.28094471981182e-16	1.05676894910834e-13	8.892495419102e-14	COX6A1/COX7B/COX5B/CYB5A/COX5A/UQCR10/ATP1B1/NDUFA4/NDUFB2/NDUFC2/COX6B1/COX8A/ATP2B1/NDUFS6/NDUFB9/NDUFB8/ATP6V0D1/NDUFB3/NDUFC1/ATP5F1B/COX4I1/NDUFA3/ATP6V0E1/CYC1/NDUFA12/NDUFB1/ATP6V0B/NDUFS8/NDUFA1/NDUFS5	30
MF	GO:0015078	proton transmembrane transporter activity	27/520	136/18432	9.96836844816037e-16	1.34174239312239e-13	1.1290488894969e-13	COX6A1/COX7B/ATP5F1E/COX5B/CYB5A/SLC9A9/ATP5PD/COX5A/UQCR10/ATP5MF/COX6B1/ATP5ME/ATP5PB/COX8A/ATP5MC1/ATP5MC3/CLCN3/ATP5PF/ATP6V0D1/ATP5F1B/COX4I1/ATP5F1C/ATP6V0E1/CYC1/ATP6V0B/ATP5PO/ATP5MG	27
MF	GO:0050136	NADH dehydrogenase (quinone) activity	15/520	42/18432	2.28801386565604e-13	2.56638888597752e-11	2.15956396442623e-11	NQO1/NDUFA4/NDUFB2/NDUFC2/NDUFS6/NDUFB9/NDUFB8/NDUFB3/NDUFC1/NDUFA3/NDUFA12/NDUFB1/NDUFS8/NDUFA1/NDUFS5	15
MF	GO:0016651	oxidoreductase activity, acting on NAD(P)H	20/520	87/18432	2.92402312723611e-13	2.81123937804271e-11	2.3656006653429e-11	AKR1C2/AKR1C3/NQO1/NDUFA4/NDUFB2/NDUFC2/NDUFS6/NDUFB9/NDUFB8/AKR1C1/NDUFB3/NDUFC1/DCXR/NDUFA3/NDUFA12/NDUFB1/NDUFS8/NDUFA1/NDUFS5/CBR1	20
MF	GO:0003954	NADH dehydrogenase activity	15/520	44/18432	5.06103144556424e-13	4.25759270358091e-11	3.58267752330732e-11	NQO1/NDUFA4/NDUFB2/NDUFC2/NDUFS6/NDUFB9/NDUFB8/NDUFB3/NDUFC1/NDUFA3/NDUFA12/NDUFB1/NDUFS8/NDUFA1/NDUFS5	15

MF	GO:0003955	NAD(P)H dehydrogenase (quinone) activity	15/520	45/18432	7.39671679528954e-13	5.53110044803318e-11	4.65430834604184e-11	NQO1/NDUFA4/NDUFB2/NDUFC2/NDUFS6/NDUFB9/NDUFB8/NDUFB3/NDUFC1/NDUFA3/NDUFA12/NDUFB1/NDUFS8/NDUFA1/NDUFS5	15
MF	GO:0045296	cadherin binding	37/520	333/18432	1.13820625007581e-12	7.66012806301019e-11	6.44584171095563e-11	SFN/S100A11/YWHAZ/PRDX6/JUP/ANXA2/LAD1/CDH1/PKP1/IDH1/TRIM29/PFN1/PPL/ANXA1/TAGLN2/TJP1/RAB10/ENO1/PDLIM5/LDHA/PSMB6/DBNL/GIPC1/CLINT1/CHMP4B/CAPG/YWHAB/CTNND1/SNX1/PRDX1/CAPZA1/CAPZB/CLIC1/ABI1/RPS26/SERBP1/KRT18	37
MF	GO:0008137	NADH dehydrogenase (ubiquinone) activity	14/520	41/18432	2.98011426304868e-12	1.82328809002888e-10	1.53425978327291e-10	NDUFA4/NDUFB2/NDUFC2/NDUFS6/NDUFB9/NDUFB8/NDUFB3/NDUFC1/NDUFA3/NDUFA12/NDUFB1/NDUFS8/NDUFA1/NDUFS5	14
MF	GO:0046933	proton-transporting ATP synthase activity, rotational mechanism	10/520	17/18432	4.7750646706305e-12	2.67801543611194e-10	2.25349543228001e-10	ATP5F1E/ATP5PD/ATP5MF/ATP5ME/ATP5PB/ATP5PF/ATP5F1B/ATP5F1C/ATP5PO/ATP5MG	10
MF	GO:0016616	oxidoreductase activity, acting on the CH-OH group of donors,	20/520	126/18432	3.77349582170975e-10	1.93717332976885e-08	1.63009189241517e-08	AKR1C2/AKR1C3/ADH7/AKR1B10/RDH12/ALDH3A1/ME1/PGD/RDH11/IDH1/UGDH/BDH1/HPGD/HMGCR/LDHA/AKR1C1/PTGR1/DCXR/MDH2/CBR1	20

		NAD or NADP as acceptor							
MF	GO:0015252	proton channel activity	10/520	24/18432	4.02978107232747e-10	1.93717332976885e-08	1.63009189241517e-08	ATP5F1E/ATP5PD/ATP5MF/ATP5ME/ATP5PB/ATP5PF/ATP5F1B/ATP5F1C/ATP5PO/ATP5MG	10
MF	GO:0016614	oxidoreductase activity, acting on CH-OH group of donors	20/520	138/18432	1.96744382201016e-09	8.82726461475227e-08	7.42796334204539e-08	AKR1C2/AKR1C3/ADH7/AKR1B10/RDH12/ALDH3A1/ME1/PGD/RDH11/IDH1/UGDH/BDH1/HPGD/HMGCR/LDHA/AKR1C1/PTGR1/DCXR/MDH2/CBR1	20
MF	GO:0004129	cytochrome-c oxidase activity	8/520	19/18432	2.18625974073911e-08	9.19595503448386e-07	7.73820881919499e-07	COX6A1/COX7B/COX5B/CYB5A/COX5A/COX6B1/COX8A/COX4I1	8
MF	GO:0016675	oxidoreductase activity, acting on a heme group of donors	8/520	20/18432	3.55402966284285e-08	1.40697762534896e-06	1.18394300842691e-06	COX6A1/COX7B/COX5B/CYB5A/COX5A/COX6B1/COX8A/COX4I1	8
MF	GO:0016628	oxidoreductase activity, acting on the CH-CH group of	9/520	28/18432	4.52465136606477e-08	1.69171687186755e-06	1.42354528359231e-06	AKR1C2/AKR1C3/TM7SF2/DECR1/DHCR7/DHCR24/AKR1C1/PTGR1/TECR	9

		donors, NAD or NADP as acceptor							
MF	GO:0016627	oxidoreductase activity, acting on the CH-CH group of donors	12/520	60/18432	9.14156636912066e-08	3.23803903495695e-06	2.72474388176561e-06	AKR1C2/AKR1C3/TM7SF2/DECR1/DHCR7/DHCR24/SDHB/AKR1C1/ACADVL/SDHC/PTGR1/TECR	12
MF	GO:0022804	active transmembrane transporter activity	33/520	417/18432	1.06277267910726e-07	3.57623006519592e-06	3.00932474399844e-06	COX6A1/COX7B/COX5B/CYB5A/SLC9A9/COX5A/SLC25A5/UQCR10/ATP1B1/NDUFA4/NDUFB2/NDUFC2/COX6B1/COX8A/ATP2B1/NDUFS6/NDUFB9/NDUFB8/CLCN3/ATP6V0D1/NDUFB3/NDUFC1/ATP5F1B/COX4I1/NDUFA3/ATP6V0E1/CYC1/NDUFA12/NDUFB1/ATP6V0B/NDUFS8/NDUFA1/NDUFS5	33
MF	GO:0008106	alcohol dehydrogenase (NADP+) activity	8/520	24/18432	1.87816149513706e-07	6.01906041060592e-06	5.06491671370295e-06	AKR1C2/AKR1C3/AKR1B10/RDH12/ALDH3A1/RDH11/AKR1C1/CBR1	8
MF	GO:0098632	cell-cell adhesion mediator activity	11/520	54/18432	2.58905094818537e-07	7.92014221876705e-06	6.66463832595085e-06	DSC2/S100A11/JUP/ANXA2/DSP/CD200/TRIM29/ANXA1/RAB10/PDLIM5/KRT18	11
MF	GO:009	cadherin binding involved in	7/520	18/18432	3.31924124962901e-07	9.71238852608835e-06	8.17277708146639e-06	S100A11/ANXA2/TRIM29/ANXA1/RAB10/PDLIM5/KRT18	7

	8641	cell-cell adhesion							
MF	GO:0098631	cell adhesion mediator activity	11/520	64/18432	1.55896756338522e-06	4.3716048756594e-05	3.67861644342654e-05	DSC2/S100A11/JUP/ANXA2/DSP/CD200/TRIM29/ANXA1/RAB10/PDLIM5/KRT18	11
MF	GO:0004033	aldo-keto reductase (NADP) activity	8/520	31/18432	1.69256627732301e-06	4.55638841855355e-05	3.83410803031487e-05	AKR1C2/AKR1C3/AKR1B10/RDH12/ALDH3A1/RDH11/AKR1C1/CBR1	8
MF	GO:0004867	serine-type endopeptidase inhibitor activity	13/520	98/18432	3.71703937455078e-06	9.62141345797184e-05	8.09622341501345e-05	SPINK5/SERPINB13/SERPINB11/SERPINB5/WFDC5/SERPINB3/ANXA2/PI3/SLPI/A2ML1/SERPINB2/SERPINB6/SPINT2	13
MF	GO:0016209	antioxidant activity	12/520	85/18432	4.53259873857013e-06	0.000112979220409544	9.50697123333618e-05	GSTA1/TXN/GPX2/GSTP1/NQO1/PRDX6/MGST2/TXNL1/GSTO1/SELENOW/PRDX1/GPX3	12
MF	GO:0004364	glutathione transferase activity	7/520	26/18432	5.63820176651491e-06	0.000135518206745162	0.000114035810164851	GSTA1/GSTM3/GSTP1/MGST2/GSTM4/GSTA4/GSTO1	7
MF	GO:0043022	ribosome binding	11/520	73/18432	5.93428363852693e-06	0.000137716306507884	0.000115885466334936	EIF6/EIF5A/PYM1/TMEM147/OXA1L/EIF3K/SEC61B/C1QBP/SEC61G/SERBP1/RPN2	11

MF	GO:0004857	enzyme inhibitor activity	28/520	395/18432	8.26610790192628e-06	0.000185436353933213	0.000156040914078468	CSTA/SPINK5/CSTB/SFN/SERPINB13/SERPINB11/SERPINB5/HSPB1/WFDC5/SERPINB3/ANXA2/FETUB/CARD18/ENSA/UGT1A7/PI3/ANXA3/SLPI/CDKN2B/A2ML1/ANXA1/RNH1/SERPINB2/PPP1R14B/SERPINB6/YWHAB/LGALS3/SPINT2	28
MF	GO:0005200	structural constituent of cytoskeleton	13/520	107/18432	9.94701779488563e-06	0.000211644802705177	0.000178094789794925	KRT6B/KRT6A/KRT16/TUBA4A/DSP/ARPC2/PPL/ARPC3/TUBA1A/ACTR3/ACTB/TUBA1C/KRT19	13
MF	GO:0061134	peptidase regulator activity	20/520	232/18432	1.00633487170367e-05	0.000211644802705177	0.000178094789794925	CSTA/SPINK5/CSTB/SERPINB13/SERPINB11/SERPINB5/WFDC5/SERPINB3/ANXA2/FETUB/CARD18/PI3/SLPI/PYCARD/CASP1/A2ML1/SERPINB2/SERPINB6/CTSC/SPINT2	20
MF	GO:0004866	endopeptidase inhibitor activity	17/520	180/18432	1.41988355821644e-05	0.00028957019232717	0.000243667417646074	CSTA/SPINK5/CSTB/SERPINB13/SERPINB11/SERPINB5/WFDC5/SERPINB3/ANXA2/FETUB/CARD18/PI3/SLPI/A2ML1/SERPINB2/SERPINB6/SPINT2	17
MF	GO:0030414	peptidase inhibitor activity	17/520	187/18432	2.33484584938025e-05	0.000462162134303796	0.000388900020732685	CSTA/SPINK5/CSTB/SERPINB13/SERPINB11/SERPINB5/WFDC5/SERPINB3/ANXA2/FETUB/CARD18/PI3/SLPI/A2ML1/SERPINB2/SERPINB6/SPINT2	17
MF	GO:0004602	glutathione peroxidase activity	6/520	22/18432	2.48669482697023e-05	0.000478155891014561	0.000402358441175934	GSTA1/GPX2/GSTP1/PRDX6/MGST2/GPX3	6
MF	GO:006	endopeptidase regulator activity	17/520	194/18432	3.74097964030657e-05	0.000699355360535089	0.000588493288445887	CSTA/SPINK5/CSTB/SERPINB13/SERPINB11/SERPINB5/WFDC5/SERPINB3/ANXA2/FETUB/CARD18/PI3/SLPI/A2ML1/SERPINB2/SERPINB6/SPINT2	17

	1135								
MF	GO:0030280	structural constituent of skin epidermis	7/520	37/18432	6.74414007482048e-05	0.00122670439739302	0.0010322467596738	KRT6B/KRT6A/KRT6C/PKP1/PI3/SPRR1A/KRT10	7
MF	GO:0016874	ligase activity	15/520	165/18432	6.97867586139794e-05	0.00123596022492653	0.00104003534998119	ACSL1/ATP5F1E/ATP5PD/ATP5MF/ATP5ME/ATP5PB/RTCB/SUCLG1/FARSB/PCCB/ATP5PF/ATP5F1B/ATP5F1C/ATP5PO/ATP5MG	15
MF	GO:0048306	calcium-dependent protein binding	10/520	81/18432	9.13167713238761e-05	0.0015757996692556	0.00132600331908894	S100A16/S100A11/ANXA2/S100A10/ANXA3/S100A14/ANXA1/ANXA7/CALM1/S100A2	10
MF	GO:0003925	G protein activity	7/520	41/18432	0.000133592894527006	0.00224770045041687	0.0018913941383034	RAB11A/ARL8B/RALA/RIT1/RAB10/CDC42/RALB	7
MF	GO:0002020	protease binding	13/520	139/18432	0.000157712311032187	0.00258878988596736	0.00217841394955883	CSTA/CSTB/SERPINB13/SERPINB3/ANXA2/CARD18/SRI/PYCARD/A2ML1/F2RL1/NTRK2/F3/SERPINB6	13
MF	GO:0022829	wide pore channel activity	6/520	31/18432	0.000197784349139654	0.00316925873740445	0.00266686666258481	GJB6/GJA1/GJB2/GJB5/VDAC1/VDAC2	6

MF	GO:0017022	myosin binding	9/520	74/18432	0.0002289285185987 18	0.00358299751202 18	0.00301501941263 428	RAB25/RAB11A/SLC9A3R1/PYCARD/RALA/RAB10/GIPC1/RAB6A/MYL12B	9
MF	GO:0016765	transferase activity, transferring alkyl or aryl (other than methyl) groups	8/520	59/18432	0.000237986919560502	0.00364011811055041	0.00306308523262082	GSTA1/GSTM3/GSTP1/MGST2/FDFT1/GSTM4/GSTA4/GSTO1	8
MF	GO:0004032	alditol:NADP+ 1-oxidoreductase activity	4/520	12/18432	0.00025881594174213	0.00378658975635768	0.0031863381386102	AKR1C2/AKR1C3/AKR1B10/AKR1C1	4
MF	GO:0086080	protein binding involved in heterotypic cell-cell adhesion	4/520	12/18432	0.00025881594174213	0.00378658975635768	0.0031863381386102	DSC2/JUP/DSP/CD200	4
MF	GO:0016903	oxidoreductase activity, acting on the aldehyde or oxo group of donors	7/520	47/18432	0.000322983118758861	0.00462484338137688	0.00389171148694887	AKR1C3/ADH7/AKR1B10/ALDH3A1/ALDH1A1/RDH11/ALDH3B2	7

MF	GO:0022853	active ion transmembrane transporter activity	19/520	278/18432	0.0003656600273 40841	0.0051268583000 0804	0.0043141468138 0203	COX6A1/COX7B/COX5B/CYB5A/SLC9A9/COX5A/SLC25A5/UQCR10/ATP1B1/COX6B1/COX8A/ATP2B1/CLCN3/ATP6V0D1/ATP5F1B/COX4I1/ATP6V0E1/CYC1/ATP6V0B	19
MF	GO:0052650	NADP-retinol dehydrogenase activity	4/520	14/18432	0.000500378555290979	0.006826597185132 23	0.005744442458123 31	AKR1C3/AKR1B10/RDH12/RDH11	4
MF	GO:0051015	actin filament binding	16/520	219/18432	0.00050717661107966	0.00682659718513223	0.00574444245812331	POF1B/DSTN/ABLIM1/GJB6/ARPC2/SCIN/UTRN/NEBL/PLS3/ARPC3/DBNL/CAPG/ACTR3/CAPZA1/CAPZB/CFL1	16
MF	GO:0050661	NADP binding	7/520	53/18432	0.000684409282700279	0.00903151857367231	0.00759983888736327	ME1/TM7SF2/DECR1/PGD/IDH1/DHCR7/HMGCR	7
MF	GO:0016620	oxidoreductase activity, acting on the aldehyde or oxo group of donors, NAD or NADP as acceptor	6/520	39/18432	0.00072417055905245	0.00937243819696729	0.00788671580506515	AKR1C3/AKR1B10/ALDH3A1/ALDH1A1/RDH11/ALDH3B2	6

MF	GO:0004601	peroxidase activity	7/520	55/18432	0.000858431390767483	0.01064400655652 18	0.00895671467491782	GSTA1/GPX2/GSTP1/PRDX6/MGST2/PRDX1/GPX3	7
MF	GO:0005080	protein kinase C binding	7/520	55/18432	0.000858431390767483	0.0106440065565218	0.00895671467491782	HSPB1/DSP/UGT1A7/GLRX3/PDLIM5/SDC4/C1QBP	7
MF	GO:0031489	myosin V binding	4/520	16/18432	0.000869866806253636	0.0106440065565218	0.00895671467491782	RAB25/RAB11A/RAB10/RAB6A	4
MF	GO:0016684	oxidoreductase activity, acting on peroxide as acceptor	7/520	57/18432	0.00106559921303544	0.0128062191138009	0.0107761724927268	GSTA1/GPX2/GSTP1/PRDX6/MGST2/PRDX1/GPX3	7
MF	GO:0004030	aldehyde dehydrogenase [NAD(P)+] activity	4/520	17/18432	0.00111232339294062	0.0131332218148953	0.0110513386039 16	ALDH3A1/ALDH1A1/RDH11/ALDH3B2	4
MF	GO:0004745	NAD-retinol dehydrogenase activity	4/520	19/18432	0.00173225707682 31	0.0201001553914129	0.0169138712764216	AKR1C3/ADH7/RDH12/RDH11	4

MF	GO:0043021	ribonucleoprotein complex binding	12/520	159/18432	0.00187524458143956	0.0213905017509971	0.01799967145074091	EIF6/EIF5A/PYM1/TMEM147/OXA1L/EIF3K/SEC61B/C1QBP/SEC61G/SERBP1/RPN2/YBX3	12
MF	GO:0005543	phospholipid binding	25/520	474/18432	0.00215457918993215	0.0241671965804056	0.0203362035821666	GLTP/SDCBP2/PLEKHN1/ANXA2/SYTL5/PLEKHA5/ANXA3/PHLDA2/APPL2/SCIN/PFN1/ANXA1/ANXA8L1/ANXA7/SNX31/VDAC1/CLINT1/GSDMC/F3/CAPG/PLD1/SNX1/ATP8B1/VDAC2/CHMP2A	25
MF	GO:0016717	oxidoreductase activity, acting on paired donors, with oxidation of a pair of donors resulting in the reduction of molecular oxygen to two molecules of water	3/520	10/18432	0.00231027507025236	0.0250776632625 78	0.0211023427469 57	DEGS2/SCD/SC5D	3
MF	GO:004	glutathione binding	3/520	10/18432	0.00231027507025236	0.02507766326257 8	0.02110234274695 7	GSTM3/MGST2/GSTM4	3

	3295								
MF	GO:0005243	gap junction channel activity	4/520	21/18432	0.00255803310532174	0.02732629015684 97	0.02299451646889 05	GJB6/GJA1/GJB2/GJB5	4
MF	GO:0032052	bile acid binding	3/520	11/18432	0.00311045142289569	0.0317171789031637	0.0266893598966 17	AKR1C2/AKR1C3/AKR1C1	3
MF	GO:0047134	protein-disulfide reductase (NAD(P)) activity	3/520	11/18432	0.00311045142289569	0.0317171789031637	0.02668935989661 7	TXN/TXNL1/PGK1	3
MF	GO:1900750	oligopeptide binding	3/520	11/18432	0.00311045142289569	0.0317171789031637	0.02668935989661 7	GSTM3/MGST2/GSTM4	3
MF	GO:0008013	beta-catenin binding	8/520	87/18432	0.00315849735332446	0.0317263987878 71	0.0266971182417684	CTNNBIP1/CDH1/GJA1/KANK1/SLC9A3R1/CTNND1/TCF4/KLF4	8
MF	GO:0019829	ATPase-coupled cation transmembr	6/520	53/18432	0.0036565550426312	0.0360590562599317	0.0303429612385129	ATP1B1/ATP2B1/ATP6V0D1/ATP5F1B/ATP6V0E1/ATP6V0B	6

		ane transporter activity							
MF	GO:0015267	channel activity	25/520	494/18432	0.00369699090926491	0.0360590562599317	0.03034296123851 29	KCNK7/CLCA2/CLDN4/ANXA2/GJB6/ATP5F1E/ATP5PD/GJA1/ATP5MF/ATP5ME/ATP5PB/GJB2/SCN9A/GJB5/KCNK1/VDAC1/CLCN3/ATP5PF/ATP5F1B/VDAC2/ATP5F1C/CLIC1/ATP5PO/ATP5MG/CLIC3	25
MF	GO:0022803	passive transmembrane transporter activity	25/520	495/18432	0.00379381692580398	0.0364748398723725	0.0306928346779329	KCNK7/CLCA2/CLDN4/ANXA2/GJB6/ATP5F1E/ATP5PD/GJA1/ATP5MF/ATP5ME/ATP5PB/GJB2/SCN9A/GJB5/KCNK1/VDAC1/CLCN3/ATP5PF/ATP5F1B/VDAC2/ATP5F1C/CLIC1/ATP5PO/ATP5MG/CLIC3	25
MF	GO:0042625	ATPase-coupled ion transmembrane transporter activity	4/520	24/18432	0.00424820932012792	0.0391649982526862	0.0329565481503795	ATP6V0D1/ATP5F1B/ATP6V0E1/ATP6V0B	4
MF	GO:0044769	ATPase activity, coupled to transmembrane movement of ions, rotational mechanism	4/520	24/18432	0.00424820932012792	0.0391649982526862	0.0329565481503795	ATP6V0D1/ATP5F1B/ATP6V0E1/ATP6V0B	4

MF	GO:0046961	proton-transporting ATPase activity, rotational mechanism	4/520	24/18432	0.00424820932012792	0.03916499825268062	0.03295654815037950	ATP6V0D1/ATP5F1B/ATP6V0E1/ATP6V0B	4
MF	GO:0019003	GDP binding	7/520	73/18432	0.00446698717449829	0.04062543741131156	0.03418547794993001	DIRAS3/ARL8B/RALA/RIT1/RAB10/RAB18/RALB	7
MF	GO:0004869	cysteine-type endopeptidase inhibitor activity	6/520	56/18432	0.00481816012292202	0.04323495683635360	0.03638133538430950	CSTA/CSTB/SERPINB13/SERPINB3/FETUB/CARD18	6
MF	GO:0016705	oxidoreductase activity, acting on paired donors, with incorporation or reduction of molecular oxygen	12/520	179/18432	0.00493455282492857	0.04369676383127530	0.03676993656248710	AKR1C2/AKR1C3/DEGS2/CYP2C18/SCD/MSMO1/SQLE/OGFOD1/BBOX1/HMOX2/AKR1C1/SC5D	12

MF	GO:0072341	modified amino acid binding	8/520	94/18432	0.00507476708277562	0.04404006084829 79	0.03705881400857 79	GSTM3/PLEKHN1/ANXA2/MGST2/GSTM4/APPL2/SCIN/GSDMC	8
MF	GO:0004859	phospholipase inhibitor activity	3/520	13/18432	0.00516963567164 27	0.04404006084829 79	0.03705881400857 79	ANXA2/ANXA3/ANXA1	3
MF	GO:0016668	oxidoreductase activity, acting on a sulfur group of donors, NAD(P) as acceptor	3/520	13/18432	0.00516963567164 27	0.04404006084829 79	0.03705881400857 79	TXN/TXNL1/PGK1	3
