# Supplementary material for: Multiomics Reveals IL-17 Drives Epithelial Keratinization and Proliferation via EHF in Odontogenic Keratocysts
Source: Int J Mol Sci. 2026 May 4;27(9):4115. doi: 10.3390/ijms27094115 (PMC13163638; doi:10.3390/ijms27094115)
Supplement: Supplementary file 1 [file ijms-27-04115-s001.zip › ijms-4235677-supplementary/Supplementary Table S7.pdf]

Supplementary Table S7. EpC5 GO enrichment.

ONTOLOGY	ID	Description	GeneRatio	BgRatio	pvalue	p.adjust	qvalue	geneID	Count
BP	GO:0001819	positive regulation of cytokine production	43/432	486/18903	2.99797563790683e-14	1.38656373253191e-10	9.48307030727372e-11	SAA1/MMP12/B2M/CLEC7A/IFI16/LUM/CYP1B1/OAS2/RSAD2/LRRK2/RAB7B/CD74/IL20RB/RUNX1/STAT1/WNT5A/AFAP1L2/IL1A/IL1B/HLA-A/CLU/IFNGR1/IL6ST/APP/HIF1A/XBP1/CD58/EGR1/CD46/ISG15/SERPINE1/STAT3/SULF2/CXCL17/S100A13/HLA-E/PIK3R1/HILPDA/TMED10/SULF1/HSP90AA1/MDK/HSPA1A	43
BP	GO:0042060	wound healing	39/432	442/18903	5.51058788188794e-13	1.27432344768659e-09	8.7154297816175e-10	ODAM/SAA1/CLDN1/MMP12/CLEC7A/SLC7A11/TGFB2/S100A8/TGFBR2/PLAUR/PLEK/TGFB3/VAV3/WNT5A/FBLN1/CAV1/IL1A/SERPINE2/PLSCR1/CDH3/IL6ST/B4GALT1/HIF1A/CD59/XBP1/SERPING1/PLAU/LMAN1/MYL9/TNFAIP3/SERPINE1/ANXA5/HBEGF/TIMP1/PPP3CA/DST/ELK3/PROS1/COL1A1	39
BP	GO:0050792	regulation of viral process	22/432	162/18903	1.99161613302557e-11	2.39498992791419e-08	1.63799595638854e-08	IFI16/BST2/CXCL8/OAS2/IFIT1/RSAD2/IFITM3/CD74/APOBEC3A/STAT1/IFITM2/PLSCR1/N4BP1/SLPI/STOM/HLA-DRB1/SRPK2/MX1/ISG15/LY6E/ZFP36/LGALS1	22
BP	GO:0048525	negative regulation of viral process	17/432	91/18903	2.25779893569076e-11	2.39498992791419e-08	1.63799595638854e-08	IFI16/BST2/OAS2/IFIT1/RSAD2/IFITM3/APOBEC3A/STAT1/IFITM2/PLSCR1/N4BP1/SLPI/SRPK2/MX1/ISG15/LY6E/ZFP36	17
BP	GO:0045071	negative regulation of viral genome replication	14/432	57/18903	2.70284927304777e-11	2.39498992791419e-08	1.63799595638854e-08	IFI16/BST2/OAS2/IFIT1/RSAD2/IFITM3/APOBEC3A/IFITM2/PLSCR1/N4BP1/SLPI/SRPK2/MX1/ISG15	14
BP	GO:0050673	epithelial cell proliferation	38/432	481/18903	3.10701396053733e-11	2.39498992791419e-08	1.63799595638854e-08	ODAM/CLDN1/MMP12/B2M/TGFB2/HAS2/BMP2/EHF/DAB2/SDR16C5/HTRA1/ID2/STAT1/AREG/LAMB1/WNT5A/GRN/CAV1/IGFBP3/CDH3/MCC/RUNX2/IGFBP5/B4GALT1/HIF1A/XBP1/ID1/TACSTD2/TNFAIP3/STAT3/ERRFI1/KLF9/ZFP36/JUN/SULF1/ZFP36L1/SPARC/MDK	38

BP	GO:0034341	response to interferon-gamma	20/432	140/18903	6.49885356831984e-11	4.29388539335418e-08	2.93669999590994e-08	CLDN1/KYNU/ASS1/BST2/IFITM3/RAB7B/CD74/CD47/STAT1/WNT5A/GBP3/IFITM2/IFNGR1/CXCL16/CCL20/GBP1/SP100/CD58/JAK1/VIM	20
BP	GO:0019221	cytokine-mediated signaling pathway	38/432	496/18903	7.6447896457961e-11	4.41964401397587e-08	3.0227095902128e-08	MMP12/CSF3/BIRC3/CXCL1/CXCL8/CXCL6/CXCL2/OAS2/CXCL3/IFITM3/CD74/IL20RB/STAT1/WNT5A/CAV1/IL1A/IFITM2/IL1B/IL1RN/IL13RA1/NFKBIA/TXNDC17/IFNGR1/IL6ST/CCL20/BIRC2/HIF1A/MX1/SP100/WNK1/JAK1/EGR1/ISG15/TNFAIP3/STAT3/PTPN1/IFI27/HSPA1A	38
BP	GO:0045639	positive regulation of myeloid cell differentiation	17/432	103/18903	1.7563694039278e-10	9.02578721462899e-08	6.17297082900942e-08	CSF3/INHBA/RAB7B/CD74/RUNX1/ID2/STAT1/NEDD9/HLA-DRB1/HIF1A/TRIB1/ISG15/STAT3/PPP3CA/FOS/ZFP36L1/HSPA1A	17
BP	GO:0022407	regulation of cell-cell adhesion	37/432	490/18903	2.07147478263192e-10	9.58057086967261e-08	6.55240181243043e-08	TNFRSF21/B2M/ASS1/HAS2/TGFBR2/BMP2/PLAUR/CD74/VNN1/IL20RB/CD47/RUNX1/WNT5A/CAV1/IL1A/PRNP/SERPINE2/IL1B/IL1RN/HLA-A/IL6ST/HLA-DRB1/NFKBIZ/XBP1/LGALS3/WNK1/JAK1/CITED2/CD46/HLA-DRA/TFRC/HSPH1/HLA-E/PPP3CA/RDX/MDK/LGALS1	37
BP	GO:0030198	extracellular matrix organization	29/432	318/18903	2.67070247923032e-10	1.10874722242585e-07	7.58301087542461e-08	MMP13/MMP7/ANGPTL7/MMP10/MMP12/COL6A6/MMP28/TGFB2/LUM/LAMB3/HAS2/BMP2/MMP1/CYP1B1/RUNX1/LAMB1/FBLN1/CAV1/COL17A1/APP/COL12A1/B4GALT1/COL14A1/CTSV/SULF2/CST3/POSTN/SULF1/COL1A1	29
BP	GO:0043062	extracellular structure organization	29/432	319/18903	2.87674955007789e-10	1.10874722242585e-07	7.58301087542461e-08	MMP13/MMP7/ANGPTL7/MMP10/MMP12/COL6A6/MMP28/TGFB2/LUM/LAMB3/HAS2/BMP2/MMP1/CYP1B1/RUNX1/LAMB1/FBLN1/CAV1/COL17A1/APP/COL12A1/B4GALT1/COL14A1/CTSV/SULF2/CST3/POSTN/SULF1/COL1A1	29
BP	GO:0045229	external encapsulating structure organization	29/432	321/18903	3.33463678170086e-10	1.1863611627205e-07	8.11383281701302e-08	MMP13/MMP7/ANGPTL7/MMP10/MMP12/COL6A6/MMP28/TGFB2/LUM/LAMB3/HAS2/BMP2/MMP1/CYP1B1/RUNX1/LAMB1/FBLN1/CAV1/COL17A1/APP/COL12A1/B4GALT1/COL14A1/CTSV/SULF2/CST3/POSTN/SULF1/COL1A1	29

BP	GO:0050678	regulation of epithelial cell proliferation	33/432	414/18903	5.19222112692 3e-10	1.71528733657 278e-07	1.17312966063 185e-07	ODAM/CLDN1/MMP12/B2M/TGFB2/HAS2/BMP2/DAB2/HTRA1/STAT1/AREG/LAMB1/WNT5A/GRN/CAV1/CDH3/MCC/RUNX2/B4GALT1/HIF1A/XBP1/ID1/TACSTD2/TNFAIP3/STAT3/ERRFI1/KLF9/ZFP36/JUN/SULF1/ZFP36L1/SPARC/MDK	33
BP	GO:1903900	regulation of viral life cycle	19/432	142/18903	6.16120497051 45e-10	1.89970486590 864e-07	1.29925760957 165e-07	IFI16/BST2/CXCL8/OAS2/IFIT1/RSAD2/IFITM3/CD74/APOBEC3A/IFITM2/PLSCR1/N4BP1/SLPI/HLA-DRB1/SRPK2/MX1/ISG15/LY6E/LGALS1	19
BP	GO:2001233	regulation of apoptotic signaling pathway	31/432	374/18903	6.84189587166 057e-10	1.97773552540 188e-07	1.35262480883 816e-07	TNFSF10/S100A9/S100A8/GCLC/SOD2/LRRK2/PLAUR/INHBA/CD74/VNN1/ITGA6/SRPX/ITGAV/CAV1/IL1A/IL1B/IFI6/NR4A2/CLU/G0S2/HIF1A/SP100/XBP1/LGALS3/SKIL/TNFAIP3/TPD52L1/SERPINE1/MCL1/PTPN1/HSPA1A	31
BP	GO:0002683	negative regulation of immune system process	34/432	449/18903	1.07663658556 508e-09	2.86427843735 375e-07	1.95895458418 162e-07	MMP12/TNFRSF21/MMP28/SAMSN1/TGFB2/IFI16/TMEM176A/TMEM176B/BST2/HLA-B/INHBA/TGFB3/CD74/IL20RB/CD47/RUNX1/ID2/DUSP1/GRN/PRNP/TSC22D3/HLA-A/HLA-DRB1/CD59/GBP1/SERPING1/LGALS3/TRIB1/CD46/ISG15/TNFAIP3/HLA-E/PIK3R1/MDK	34
BP	GO:0045069	regulation of viral genome replication	15/432	87/18903	1.11474620264 578e-09	2.86427843735 375e-07	1.95895458418 162e-07	IFI16/BST2/CXCL8/OAS2/IFIT1/RSAD2/IFITM3/APOBEC3A/IFITM2/PLSCR1/N4BP1/SLPI/SRPK2/MX1/ISG15	15
BP	GO:0048545	response to steroid hormone	29/432	339/18903	1.19409710757 273e-09	2.87225480929 634e-07	1.96440983258 845e-07	CLDN1/TGFB2/PAPPA/ASS1/TGFBR2/CYP1B1/DAB2/FIBIN/TGFB3/PMEPA1/AREG/CAV1/FOSB/GJB2/IL1RN/ATP1A1/FBXO32/NR3C1/ERRFI1/KLF9/SGK1/ZFP36/FOS/TXNIP/IGFBP7/ZFP36L1/MDK/HSPA1A/COL1A1	29
BP	GO:0031960	response to corticosteroid	20/432	165/18903	1.28796070733 637e-09	2.87225480929 634e-07	1.96440983258 845e-07	CLDN1/PAPPA/ASS1/CYP1B1/FIBIN/AREG/FOSB/GJB2/IL1RN/FBXO32/NR3C1/ERRFI1/KLF9/SGK1/ZFP36/FOS/IGFBP7/ZFP36L1/MDK/COL1A1	20
BP	GO:0019058	viral life cycle	28/432	319/18903	1.30415894043 726e-09	2.87225480929 634e-07	1.96440983258 845e-07	CLDN1/IFI16/BST2/CXCL8/OAS2/IFIT1/RSAD2/IFITM3/CD74/APOBEC3A/ITGAV/CAV1/IFITM2/PLSCR1/N4BP1/SLPI/CTSB/STOM/HLA-DRB1/SRPK2/MX1/CD46/ISG15/TFRC/LY6E/IFI27/HSPA1A/LGALS1	28

BP	GO:0097529	myeloid leukocyte migration	24/432	242/18903	1.81007079124381e-09	3.64965822593147e-07	2.49609626604246e-07	SAA1/MMP28/TGFB2/CXCL1/S100A9/S100A8/CXCL8/CXCL6/CXCL2/CXCL3/PDGFD/VAV3/CD74/CD47/DUSP1/IL1A/IL1B/CCL20/B4GALT1/LGALS3/SERPINE1/CXCL17/PIK3R1/MDK	24
BP	GO:0052547	regulation of peptidase activity	34/432	459/18903	1.89996953634058e-09	3.64965822593147e-07	2.49609626604246e-07	TNFSF10/CLEC7A/BIRC3/IFI16/S100A9/S100A8/BST2/PLAUR/PSMB9/CRYAB/FBLN1/GRN/CAV1/PRNP/SERPINE2/TNFAIP8/IFI6/APLP2/SLPI/CTSB/APP/BIRC2/SERPING1/ARL6IP5/PSMB8/CTSD/ASPH/SERPINE1/STAT3/TIMP1/CST3/TMED10/PROS1/UACA	34
BP	GO:1903706	regulation of hemopoiesis	32/432	413/18903	1.93324738094978e-09	3.64965822593147e-07	2.49609626604246e-07	CSF3/B2M/TMEM176A/TGFBR2/TMEM176B/HLA-B/INHBA/RAB7B/CD74/VNN1/RUNX1/ID2/STAT1/PRDM1/NFKBIA/NEDD9/HLA-DRB1/NFKBIZ/HIF1A/XBP1/TRIB1/CD46/ISG15/HLA-DRA/STAT3/PPP3CA/PIK3R1/ZFP36/FOS/ZFP36L1/MDK/HSPA1A	32
BP	GO:0045785	positive regulation of cell adhesion	35/432	484/18903	2.04606485994968e-09	3.64965822593147e-07	2.49609626604246e-07	SAA1/B2M/TGFB2/HAS2/TGFBR2/DAB2/PLAUR/VAV3/CD74/VNN1/CD47/RUNX1/RIN2/ITGAV/LAMB1/WNT5A/CAV1/IL1A/IL1B/HLA-A/IL6ST/NEDD9/HLA-DRB1/NFKBIZ/XBP1/JAK1/CITED2/CD46/HLA-DRA/TFRC/HSPH1/HLA-E/PPP3CA/MDK/LGALS1	35
BP	GO:0007159	leukocyte cell-cell adhesion	32/432	414/18903	2.05169975944255e-09	3.64965822593147e-07	2.49609626604246e-07	TNFRSF21/B2M/ASS1/S100A9/S100A8/HAS2/TGFBR2/CD74/VNN1/IL20RB/CD47/RUNX1/CAV1/IL1A/PRNP/IL1B/HLA-A/IL6ST/HLA-DRB1/NFKBIZ/XBP1/LGALS3/WNK1/CD46/MSN/HLA-DRA/TFRC/HSPH1/HLA-E/PPP3CA/MDK/LGALS1	32
BP	GO:0032963	collagen metabolic process	16/432	107/18903	2.71760166851592e-09	4.65515100625412e-07	3.18377895278375e-07	MMP13/MMP7/MMP10/MMP12/MMP28/MMP1/INHBA/TGFB3/RUNX1/CTSB/HIF1A/VIM/ERRFI1/CST3/NPPC/COL1A1	16
BP	GO:0050900	leukocyte migration	31/432	398/18903	3.07751028350476e-09	4.93375463351782e-07	3.37432322588246e-07	SAA1/MMP28/TGFB2/CXCL1/S100A9/S100A8/CXCL8/CXCL6/CXCL2/CXCL3/PDGFD/VAV3/CD74/ITGA6/CD47/WNT5A/DUSP1/IL1A/IL1B/CXCL16/APP/NEDD9/CCL20/B4GALT1/LGALS3/WNK1/MSN/SERPINE1/CXCL17/PIK3R1/MDK	31
BP	GO:0016032	viral process	32/432	421/18903	3.0935974999355e-09	4.93375463351782e-07	3.37432322588246e-07	CLDN1/IFI16/BST2/CXCL8/OAS2/IFIT1/RSAD2/IFITM3/CD74/APOBEC3A/STAT1/ITGAV/GALNT1/CAV1/IFITM2/PLSCR1/N4BP1/SLPI/CTSB/STOM/HLA-	32

								DRB1/SRPK2/MX1/CD46/ISG15/TFRC/LY6E/IFI27/ZFP36/JUN/HSPA1A/LGALS1	
BP	GO:0019883	antigen processing and presentation of endogenous antigen	9/432	26/18903	3.46479721762895e-09	5.34156237717796e-07	3.65323355753508e-07	B2M/HLA-C/HLA-B/CD74/TAPBP/HLA-A/HLA-DRB1/HLA-DRA/HLA-E	9
BP	GO:0051384	response to glucocorticoid	18/432	142/18903	4.22609566578133e-09	6.11201122055703e-07	4.18016357730273e-07	CLDN1/PAPPA/ASS1/CYP1B1/FIBIN/AREG/FOSB/GJB2/IL1RN/FBXO32/NR3C1/ERRFI1/KLF9/ZFP36/FOS/IGFBP7/ZFP36L1/MDK	18
BP	GO:0002483	antigen processing and presentation of endogenous peptide antigen	8/432	19/18903	4.22885100665567e-09	6.11201122055703e-07	4.18016357730273e-07	B2M/HLA-C/HLA-B/TAPBP/HLA-A/HLA-DRB1/HLA-DRA/HLA-E	8
BP	GO:0002237	response to molecule of bacterial origin	29/432	360/18903	4.72715142652401e-09	6.62517434777986e-07	4.53112919831089e-07	CLDN1/CSF3/B2M/CXCL1/ASS1/S100A9/S100A8/CXCL8/CXCL6/CXCL2/CXCL3/SOD2/TNIP3/WNT5A/IL1A/IL1B/GJB2/ABCA1/NFKBIA/SLPI/XBP1/TRIB1/VIM/TNFAIP3/CAPN2/SERPINE1/ZFP36/FOS/DIO2	29
BP	GO:0032496	response to lipopolysaccharide	28/432	339/18903	5.06414338164003e-09	6.88872445296622e-07	4.71137797579824e-07	CLDN1/CSF3/CXCL1/ASS1/S100A9/S100A8/CXCL8/CXCL6/CXCL2/CXCL3/SOD2/TNIP3/WNT5A/IL1A/IL1B/GJB2/ABCA1/NFKBIA/SLPI/XBP1/TRIB1/VIM/TNFAIP3/CAPN2/SERPINE1/ZFP36/FOS/DIO2	28
BP	GO:0030099	myeloid cell differentiation	31/432	407/18903	5.23637076917322e-09	6.91948994497889e-07	4.73241929665128e-07	CSF3/B2M/IFI16/EPAS1/TGFBR2/INHBA/RAB7B/CD74/RUNX1/ID2/STAT1/NFKBIA/APP/NEDD9/HLA-DRB1/HIF1A/TRIB1/CITED2/ISG15/MAF/TFRC/STAT3/PTBP3/PPP3CA/PIK3R1/ZFP36/KMT2E/FOS/JUN/ZFP36L1/HSPA1A	31
BP	GO:0009615	response to virus	31/432	409/18903	5.87959700279232e-09	7.55364892719847e-07	5.16613713257045e-07	MMP12/BIRC3/IFI16/BST2/OAS2/IFIT1/RSAD2/IFITM3/IFI44/HTRA1/APOBEC3A/IFIT3/STAT1/GBP3/IFITM2/PLSCR1/IL1B/IFI6/CLU/IFNGR1/BIRC2/HIF1A/GBP1/MX1/JAK1/NPC2/ISG15/TNFAIP3/DNAJC3/IFI27/HSP90AA1	31
BP	GO:0022409	positive regulation of cell-cell adhesion	27/432	322/18903	6.94083772139665e-09	8.61594518817465e-07	5.89266919839882e-07	B2M/HAS2/TGFBR2/PLAUR/CD74/VNN1/CD47/RUNX1/WNT5A/CAV1/IL1A/IL1B/HLA-A/IL6ST/HLA-DRB1/NFKBIZ/XBP1/JAK1/CITED2/CD46/HLA-DRA/TFRC/HSPH1/HLA-E/PPP3CA/MDK/LGALS1	27

BP	GO:0019079	viral genome replication	17/432	130/18903	7.07904685731106e-09	8.61594518817465e-07	5.89266919839882e-07	IFI16/BST2/CXCL8/OAS2/IFIT1/RSAD2/IFITM3/APOBEC3A/IFITM2/PLSCR1/N4BP1/SLPI/STOM/SRPK2/MX1/ISG15/IFI27	17
BP	GO:0030595	leukocyte chemotaxis	23/432	240/18903	7.64540203514079e-09	8.96871067297156e-07	6.13393469639364e-07	SAA1/MMP28/TGFB2/CXCL1/S100A9/S100A8/CXCL8/CXCL6/CXCL2/CXCL3/VAV3/CD74/WNT5A/DUSP1/IL1B/CXCL16/NEDD9/CCL20/LGALS3/WNK1/SERPINE1/CXCL17/MDK	23
BP	GO:0070997	neuron death	29/432	368/18903	7.75672274419162e-09	8.96871067297156e-07	6.13393469639364e-07	CSF3/TNFRSF21/SLC7A11/TGFB2/GCLC/NCOA7/SOD2/LRRK2/TGFB3/WNT5A/GRN/SLC23A2/PRNP/NR4A2/CLU/IL6ST/APP/HIF1A/SRPK2/ARL6IP5/EGR1/CAPN2/NR3C1/HSPA5/MCL1/CHL1/FOS/JUN/MDK	29
BP	GO:1903037	regulation of leukocyte cell-cell adhesion	29/432	377/18903	1.33028510652314e-08	1.50062649211451e-06	1.02631752120719e-06	TNFRSF21/B2M/ASS1/HAS2/TGFBR2/CD74/VNN1/IL20RB/CD47/RUNX1/CAV1/IL1A/PRNP/IL1B/HLA-A/IL6ST/HLA-DRB1/NFKBIZ/XBP1/LGALS3/WNK1/CD46/HLA-DRA/TFRC/HSPH1/HLA-E/PPP3CA/MDK/LGALS1	29
BP	GO:0071560	cellular response to transforming growth factor beta stimulus	24/432	271/18903	1.69542581351686e-08	1.8483663413945e-06	1.26414585624819e-06	CLDN1/TGFB2/TGFBR2/BMP2/DAB2/PDGFD/TGFB3/HTRA1/PMEPA1/WNT5A/CAV1/ID1/SKIL/CITED2/NR3C1/STAT3/HSPA5/NREP/FOS/JUN/ZFP36L1/SINHCAF/HSPA1A/COL1A1	24
BP	GO:2001236	regulation of extrinsic apoptotic signaling pathway	18/432	155/18903	1.71848113902624e-08	1.8483663413945e-06	1.26414585624819e-06	TNFSF10/GCLC/INHBA/ITGA6/SRPX/ITGAV/CAV1/IL1A/IL1B/IFI6/G0S2/SP100/LGALS3/SKIL/TNFAIP3/SERPINE1/MCL1/HSPA1A	18
BP	GO:0030593	neutrophil chemotaxis	15/432	106/18903	1.84788755227448e-08	1.92975387332635e-06	1.31980890795919e-06	SAA1/TGFB2/CXCL1/S100A9/S100A8/CXCL8/CXCL6/CXCL2/CXCL3/VAV3/CD74/IL1B/CCL20/LGALS3/MDK	15
BP	GO:0010038	response to metal ion	28/432	360/18903	1.87759836323645e-08	1.92975387332635e-06	1.31980890795919e-06	CLDN1/B2M/ASS1/S100A8/GCLC/FIBIN/SOD2/LRRK2/CPNE8/DLG2/WNT5A/CAV1/IL1A/PRNP/PLSCR1/FOSB/APP/HIF1A/FABP4/TFRC/HSPA5/KCNMA1/PPP3CA/MT2A/FOS/JUN/TXNIP/MT1E	28
BP	GO:0008544	epidermis development	28/432	362/18903	2.11532759593798e-08	2.12682394156808e-06	1.45459025761868e-06	TGFB2/LAMC2/PITX2/LAMA3/LAMB3/INHBA/ZBED2/WNT5A/IL1A/FABP5/PLS1/KRT15/CDH3/COL17A1/HLA-	28

								DRB1/IGFBP5/FOSL2/KRT14/MYO6/PTHLH/ERRFI1/PPP3CA/PALLD/ZFP36/CRABP2/TXNIP/ZFP36L1/SOSTDC1	
BP	GO:0034612	response to tumor necrosis factor	23/432	254/18903	2.23299952730106e-08	2.1727098229075e-06	1.48597280633056e-06	CLDN1/TNFRSF21/BIRC3/ASS1/HAS2/CYP1B1/CXCL8/STAT1/GBP3/NFKBIA/TXNDC17/CXCL16/CCL20/BIRC2/GBP1/CD58/TNFAIP3/FABP4/ZFP36/FOS/ZFP36L1/HSPA1A/COL1A1	23
BP	GO:0045637	regulation of myeloid cell differentiation	21/432	214/18903	2.28119882956308e-08	2.1727098229075e-06	1.48597280633056e-06	CSF3/B2M/INHBA/RAB7B/CD74/RUNX1/ID2/STAT1/NFKBIA/NEDD9/HLA-DRB1/HIF1A/TRIB1/ISG15/STAT3/PPP3CA/PIK3R1/ZFP36/FOS/ZFP36L1/HSPA1A	21
BP	GO:0071356	cellular response to tumor necrosis factor	22/432	234/18903	2.30189797453984e-08	2.1727098229075e-06	1.48597280633056e-06	CLDN1/TNFRSF21/BIRC3/ASS1/HAS2/CYP1B1/CXCL8/STAT1/GBP3/NFKBIA/TXNDC17/CCL20/BIRC2/GBP1/CD58/TNFAIP3/FABP4/ZFP36/FOS/ZFP36L1/HSPA1A/COL1A1	22
BP	GO:0071559	response to transforming growth factor beta	24/432	277/18903	2.58792775766552e-08	2.39383317584061e-06	1.63720482353366e-06	CLDN1/TGFB2/TGFBR2/BMP2/DAB2/PDGFD/TGFB3/HTRA1/PMEPA1/WNT5A/CAV1/ID1/SKIL/CITED2/NR3C1/STAT3/HSPA5/NREP/FOS/JUN/ZFP36L1/SINHCAF/HSPA1A/COL1A1	24
BP	GO:0044706	multi-multicellular organism process	21/432	218/18903	3.15470375459574e-08	2.86088330686379e-06	1.9566325660599e-06	PAPPA/TGFBR2/TGFB3/FBLN1/PRDM1/SERPINE2/IL1B/FOSB/GJB2/CTSB/IGFBP5/B4GALT1/GJA1/CITED2/PTHLH/CAPN2/VMP1/TIMP1/SLC38A2/FOS/IGFBP7	21
BP	GO:0071216	cellular response to biotic stimulus	23/432	261/18903	3.70611869382746e-08	3.24652269493055e-06	2.22038138225122e-06	CSF3/CLEC7A/CXCL1/ASS1/CXCL8/CXCL6/CXCL2/CXCL3/TNIP3/WNT5A/IL1A/IL1B/ABCA1/NFKBIA/XBP1/TRIB1/VIM/TNFAIP3/CAPN2/SERPINE1/HSPA5/ZFP36/TXNIP	23
BP	GO:0051607	defense response to virus	25/432	304/18903	3.72033952067717e-08	3.24652269493055e-06	2.22038138225122e-06	MMP12/BIRC3/IFI16/BST2/OAS2/IFIT1/RSAD2/IFITM3/HTRA1/APOBEC3A/IFIT3/STAT1/GBP3/IFITM2/PLSCR1/IL1B/IFI6/BIRC2/GBP1/MX1/ISG15/TNFAIP3/DNAJC3/IFI27/HSP90AA1	25
BP	GO:0140546	defense response to symbiont	25/432	305/18903	3.96824906373108e-08	3.37158307511997e-06	2.30591343174634e-06	MMP12/BIRC3/IFI16/BST2/OAS2/IFIT1/RSAD2/IFITM3/HTRA1/APOBEC3A/IFIT3/STAT1/GBP3/IFITM2/PLSCR1/IL1B/IFI6/BIRC2/GBP1/MX1/ISG15/TNFAIP3/DNAJC3/IFI27/HSP90AA1	25

BP	GO:1901653	cellular response to peptide	28/432	373/18903	4.0094501433859e-08	3.37158307511997e-06	2.30591343174634e-06	ASS1/GCLC/CYP1B1/INHBA/RAB31/SLC39A14/STAT1/CAV1/AGTRAP/PRNP/ADRB2/IL1B/GJB2/NR4A2/APP/XBP1/GJA1/ID1/JAK1/VIM/STAT3/ERRFI1/PKM/PTPN1/PIK3R1/CA2/FOS/ZFP36L1	28
BP	GO:0071621	granulocyte chemotaxis	16/432	130/18903	4.71203828922752e-08	3.82336440134689e-06	2.61489844120567e-06	SAA1/TGFB2/CXCL1/S100A9/S100A8/CXCL8/CXCL6/CXCL2/CXCL3/VAV3/CD74/IL1B/CCL20/LGALS3/CXCL17/MDK	16
BP	GO:1990266	neutrophil migration	16/432	130/18903	4.71203828922752e-08	3.82336440134689e-06	2.61489844120567e-06	SAA1/TGFB2/CXCL1/S100A9/S100A8/CXCL8/CXCL6/CXCL2/CXCL3/VAV3/CD74/IL1A/IL1B/CCL20/LGALS3/MDK	16
BP	GO:0034340	response to type I interferon	13/432	83/18903	4.79922662120105e-08	3.82696950397498e-06	2.61736406473851e-06	MMP12/OAS2/IFIT1/IFITM3/STAT1/WNT5A/IFITM2/MX1/SP100/JAK1/ISG15/PTPN1/IFI27	13
BP	GO:1903131	mononuclear cell differentiation	32/432	473/18903	4.94333931679083e-08	3.87507531189112e-06	2.65026487902 88e-06	B2M/IFI16/TMEM176A/TGFBR2/TMEM176B/RSAD2/HLA-B/INHBA/CD74/VNN1/RUNX1/ID2/IL1A/PRDM1/IL1B/HLA-DRB1/RUNX2/DNAJB9/NFKBIZ/XBP1/EGR1/CD46/HLA-DRA/STAT3/PBX1/PIK3R1/FOS/JUN/KLF6/ZFP36L1/MDK/LGALS1	32
BP	GO:0097191	extrinsic apoptotic signaling pathway	21/432	224/18903	5.05654920206433e-08	3.89775667659126e-06	2.66577725477251e-06	TNFSF10/TGFB2/GCLC/INHBA/ITGA6/SRPX/ITGAV/CAV1/IL1A/IL1B/IFI6/G0S2/SP100/LGALS3/SKIL/TNFAIP3/SERPINE1/MCL1/PIK3R1/IFI27/HSPA1A	21
BP	GO:1901654	response to ketone	20/432	205/18903	5.44568576319426e-08	4.12890109094647e-06	2.82386293673835e-06	CLDN1/TGFB2/PAPPA/ASS1/CYP1B1/FIBIN/TGFB3/CAV1/FOSB/GJB2/FBXO32/MSN/NR3C1/ERRFI1/KLF9/SGK1/FOS/POSTN/TXNIP/IGFBP7	20
BP	GO:0052548	regulation of endopeptidase activity	30/432	428/18903	6.03597370726754e-08	4.50264167679232e-06	3.07947385234957e-06	TNFSF10/CLEC7A/BIRC3/IFI16/S100A9/S100A8/BST2/PLAUR/PSMB9/CRYAB/PRNP/SERPINE2/TNFAIP8/IFI6/APLP2/SLPI/APP/BIRC2/SERPING1/ARL6IP5/PSMB8/CTSD/ASPH/SERPINE1/STAT3/TIMP1/CST3/TMED10/PROS1/UACA	30
BP	GO:0007565	female pregnancy	19/432	189/18903	7.27838038939552e-08	5.34325544459592e-06	3.65439149041496e-06	PAPPA/TGFBR2/TGFB3/FBLN1/PRDM1/IL1B/FOSB/GJB2/CTSB/IGFBP5/GJA1/CITED2/PTHLH/CAPN2/VMP1/TIMP1/SLC38A2/FOS/IGFBP7	19

BP	GO:0044703	multi-organism reproductive process	20/432	209/18903	7.51108726402498e-08	5.42793415564306e-06	3.71230546519656e-06	PAPPA/TGFBR2/TGFB3/FBLN1/PRDM1/SERPINE2/IL1B/FOSB/GJB2/CTSB/IGFBP5/GJA1/CITED2/PTHLH/CAPN2/VMP1/TIMP1/SLC38A2/FOS/IGFBP7	20
BP	GO:0042476	odontogenesis	16/432	135/18903	8.0587886978597e-08	5.6599443672626e-06	3.8709832884493e-06	ODAM/TGFB2/FAM20A/PITX2/BMP2/INHBA/TGFB3/HTRA1/ODAPH/LAMB1/ANKH/AMTN/RUNX2/SERPINE1/SOSTDC1/COL1A1	16
BP	GO:0050870	positive regulation of T cell activation	22/432	251/18903	8.09168697585836e-08	5.6599443672626e-06	3.8709832884493e-06	B2M/TGFBR2/CD74/VNN1/CD47/RUNX1/CAV1/IL1A/IL1B/HLA-A/IL6ST/HLA-DRB1/NFKBIZ/XBP1/CD46/HLA-DRA/TFRC/HSPH1/HLA-E/PPP3CA/MDK/LGALS1	22
BP	GO:0006979	response to oxidative stress	30/432	434/18903	8.1992707590615e-08	5.6599443672626e-06	3.8709832884493e-06	ANGPTL7/SLC7A11/EPAS1/GCLC/NCOA7/CYP1B1/SOD2/LRRK2/PDGFD/VNN1/CRYAB/STAT1/AREG/IL1A/SLC23A2/PRNP/GJB2/NR4A2/APP/HIF1A/PNPLA8/ARL6IP5/TNFAIP3/CAPN2/MCL1/FOS/JUN/TXNIP/HSPA1A/COL1A1	30
BP	GO:0061448	connective tissue development	23/432	274/18903	9.05834046769452e-08	6.11995196106288e-06	4.18559445644243e-06	MMP13/TGFBR2/BMP2/PDGFD/OGN/SELENOM/SLC39A14/RUNX1/WNT5A/RUNX2/HIF1A/XBP1/EGR1/PTHLH/MAF/SULF2/ARRDC3/TIMP1/SULF1/WNT2B/NPPC/MDK/COL1A1	23
BP	GO:0001666	response to hypoxia	24/432	296/18903	9.13030670947759e-08	6.11995196106288e-06	4.18559445644243e-06	TGFB2/EPAS1/TGFBR2/BMP2/SOD2/TGFB3/CRYAB/CAV1/IL1A/AGTRAP/NR4A2/BIRC2/HIF1A/CITED2/EGR1/PLAU/CAPN2/TFRC/KCNMA1/HILPDA/ATP1B1/FOS/ZFP36L1/NPPC	24
BP	GO:0060326	cell chemotaxis	25/432	319/18903	9.5039782746361e-08	6.27941421717028e-06	4.29465484440323e-06	SAA1/MMP28/TGFB2/CXCL1/S100A9/S100A8/CXCL8/CXCL6/CXCL2/CXCL3/PDGFD/VAV3/CD74/WNT5A/DUSP1/IL1B/CXCL16/NEDD9/CCL20/LGALS3/WNK1/SERPINE1/CXCL17/HBEGF/MDK	25
BP	GO:1903039	positive regulation of leukocyte cell-cell adhesion	23/432	275/18903	9.67966572288208e-08	6.30541605187741e-06	4.31243817602085e-06	B2M/HAS2/TGFBR2/CD74/VNN1/CD47/RUNX1/CAV1/IL1A/IL1B/HLA-A/IL6ST/HLA-DRB1/NFKBIZ/XBP1/CD46/HLA-DRA/TFRC/HSPH1/HLA-E/PPP3CA/MDK/LGALS1	23
BP	GO:0097530	granulocyte migration	17/432	158/18903	1.32816704113648e-07	8.53162856285589e-06	5.83500286347242e-06	SAA1/TGFB2/CXCL1/S100A9/S100A8/CXCL8/CXCL6/CXCL2/CXCL3/VAV3/CD74/IL1A/IL1B/CCL20/LGALS3/CXCL17/MDK	17

BP	GO:1901214	regulation of neuron death	25/432	325/18903	1.35961727462783e-07	8.61401355500511e-06	5.89134810419126e-06	CSF3/SLC7A11/TGFB2/GCLC/NCOA7/SOD2/LRRK2/TGFB3/WNT5A/GRN/SLC23A2/PRNP/NR4A2/CLU/IL6ST/HIF1A/SRPK2/EGR1/CAPN2/NR3C1/MCL1/CHL1/FOS/JUN/MDK	25
BP	GO:0071357	cellular response to type I interferon	12/432	77/18903	1.67289952500272e-07	1.0455622031267e-05	7.1508720805593e-06	MMP12/OAS2/IFIT1/IFITM3/STAT1/WNT5A/IFITM2/SP100/JAK1/ISG15/PTPN1/IFI27	12
BP	GO:0050863	regulation of T cell activation	27/432	376/18903	1.73069137745214e-07	1.06725968276215e-05	7.29926679192094e-06	TNFRSF21/B2M/TGFBR2/CD74/VNN1/IL20RB/CD47/RUNX1/CAV1/IL1A/PRNP/PRDM1/IL1B/HLA-A/IL6ST/HLA-DRB1/NFKBIZ/XBP1/LGALS3/CD46/HLA-DRA/TFRC/HSPH1/HLA-E/PPP3CA/MDK/LGALS1	27
BP	GO:0048002	antigen processing and presentation of peptide antigen	11/432	64/18903	1.95696649834623e-07	1.17458476909414e-05	8.03329099545466e-06	B2M/HLA-C/HLA-B/CD74/TAPBP/HLA-A/HLA-DRB1/CTSV/CTSD/HLA-DRA/HLA-E	11
BP	GO:0010575	positive regulation of vascular endothelial growth factor production	8/432	29/18903	1.9645767113695e-07	1.17458476909414e-05	8.03329099545466e-06	CYP1B1/IL1A/IL1B/IL6ST/HIF1A/SULF2/CXCL17/SULF1	8
BP	GO:0071222	cellular response to lipopolysaccharide	20/432	222/18903	2.02349462659785e-07	1.17458476909414e-05	8.03329099545466e-06	CSF3/CXCL1/ASS1/CXCL8/CXCL6/CXCL2/CXCL3/TNIP3/WNT5A/IL1A/IL1B/ABCA1/NFKBIA/XBP1/TRIB1/VIM/TNFAIP3/CAPN2/SERPINE1/ZFP36	20
BP	GO:0002697	regulation of immune effector process	27/432	379/18903	2.0295138304771e-07	1.17458476909414e-05	8.03329099545466e-06	CFH/B2M/CLEC7A/TGFB2/BST2/CXCL6/RSAD2/HLA-B/TGFB3/CD74/IL20RB/CD47/WNT5A/GRN/IL1B/HLA-A/HLA-DRB1/DNAJB9/NFKBIZ/CD59/XBP1/SERPING1/LGALS3/CD46/HLA-DRA/TFRC/HLA-E	27
BP	GO:0036293	response to decreased oxygen levels	24/432	309/18903	2.03171419518986e-07	1.17458476909414e-05	8.03329099545466e-06	TGFB2/EPAS1/TGFBR2/BMP2/SOD2/TGFB3/CRYAB/CAV1/IL1A/AGTRAP/NR4A2/BIRC2/HIF1A/CITED2/EGR1/PLAU/CAPN2/TFRC/KCNMA1/HILPDA/ATP1B1/FOS/ZFP36L1/NPPC	24

BP	GO:0050679	positive regulation of epithelial cell proliferation	20/432	224/18903	2.34058133716908e-07	1.33644304745766e-05	9.14028189498778e-06	ODAM/CLDN1/MMP12/HAS2/BMP2/HTRA1/AREG/LAMB1/WNT5A/GRN/CDH3/RUNX2/B4GALT1/HIF1A/XBP1/ID1/TNFAIP3/STAT3/JUN/MDK	20
BP	GO:0009410	response to xenobiotic stimulus	29/432	432/18903	2.51007189970217e-07	1.41574177269787e-05	9.68262651939029e-06	GAD2/SLC1A3/TGFB2/ASS1/NNMT/TGFBR2/GCLC/CYP1B1/SOD2/FMO2/INHBA/VAV3/APOBEC3A/STAT1/PRNP/IL1B/FOSB/CDH3/ATP1A1/TFRC/HSPA5/PARP4/FOS/JUN/TXNIP/HSP90AA1/NPPC/MDK/COL1A1	29
BP	GO:0070482	response to oxygen levels	25/432	337/18903	2.70770002972422e-07	1.50880875150295e-05	1.03191358139775e-05	TGFB2/EPAS1/TGFBR2/BMP2/SOD2/TGFB3/CRYAB/CAV1/IL1A/AGTRAP/NR4A2/BIRC2/HIF1A/CITED2/EGR1/PLAU/CAPN2/TFRC/KCNMA1/HILPDA/ATP1B1/FOS/ZFP36L1/NPPC/COL1A1	25
BP	GO:0002831	regulation of response to biotic stimulus	26/432	361/18903	2.77257747563504e-07	1.52656795533477e-05	1.0440595631934e-05	CFH/MMP12/CLEC7A/BIRC3/IFI16/CXCL6/IFIT1/HLA-B/HTRA1/STAT1/WNT5A/GRN/PLSCR1/OPTN/IL1B/HLA-A/N4BP1/HLA-DRB1/BIRC2/SERPING1/TRIB1/ISG15/TNFAIP3/HLA-E/PTPN1/HSP90AA1	26
BP	GO:0048708	astrocyte differentiation	12/432	82/18903	3.40209299770386e-07	1.85113883698593e-05	1.26604203815481e-05	S100A9/S100A8/BMP2/ID2/GRN/SERPINE2/IL1B/IFNGR1/IL6ST/APP/VIM/STAT3	12
BP	GO:0007568	aging	17/432	169/18903	3.51952950470659e-07	1.89277022782186e-05	1.2945148300665e-05	CLDN1/ASS1/TGFBR2/GCLC/SOD2/INHBA/TGFB3/CRYAB/MME/GJB2/IGFBP5/SERPING1/KRT14/TFRC/TIMP1/PPP3CA/FOS	17
BP	GO:0050878	regulation of body fluid levels	27/432	390/18903	3.58400996789086e-07	1.90529265534428e-05	1.30307924422408e-05	SAA1/CLDN1/SLC7A11/HAS2/OAS2/PLAUR/PLEK/VAV3/FBLN1/CAV1/SERPINE2/PLSCR1/IL6ST/HIF1A/CD59/XBP1/SERPING1/WNK1/PLAU/LMAN1/MYL9/SERPINE1/ANXA5/PPP3CA/TMPRSS11F/AKR1B1/PROS1	27
BP	GO:0048872	homeostasis of number of cells	23/432	296/18903	3.62526858456113e-07	1.90532581859037e-05	1.30310192543136e-05	B2M/SLC7A11/EPAS1/CXCL6/SPRR2A/INHBA/CD74/IL20RB/ID2/STAT1/CDH2/TSC22D3/HIF1A/SKIL/CITED2/ISG15/TNFAIP3/STAT3/PTBP3/ZFP36/KMT2E/ZFP36L1/HSPA1A	23
BP	GO:0050867	positive regulation of cell activation	30/432	467/18903	3.97739234856901e-07	2.0669033272058e-05	1.41360898964516e-05	B2M/CLEC7A/TGFBR2/LRRK2/PLEK/VAV3/CD74/VNN1/CD47/RUNX1/WNT5A/CAV1/IL1A/IL1B/HLA-A/IL6ST/HLA-DRB1/NFKBIZ/XBP1/CD46/HLA-DRA/IGHG3/IGKC/TFRC/IGHG4/HSPH1/HLA-E/PPP3CA/MDK/LGALS1	30

BP	GO:0009612	response to mechanical stimulus	19/432	211/18903	4.11378061872054e-07	2.11402615128694e-05	1.44583751570237e-05	SLC1A3/TGFBR2/GCLC/STAT1/CDH2/SERPINE2/IL1B/FOSB/NFKBIA/CITED2/CAPN2/GADD45A/MTPN/SLC38A2/FOS/JUN/TXNIP/MDK/COL1A1	19
BP	GO:2001234	negative regulation of apoptotic signaling pathway	20/432	233/18903	4.41229541669373e-07	2.22736570331075e-05	1.5233533857067e-05	GCLC/SOD2/LRRK2/PLAUR/CD74/ITGA6/ITGAV/IL1A/IL1B/IFI6/NR4A2/CLU/HIF1A/XBP1/LGALS3/TNFAIP3/SERPINE1/MCL1/PTPN1/HSPA1A	20
BP	GO:0071548	response to dexamethasone	9/432	43/18903	4.43065177739652e-07	2.22736570331075e-05	1.5233533857067e-05	CLDN1/PAPPA/ASS1/CYP1B1/FIBIN/GJB2/FBXO32/NR3C1/ERRFI1	9
BP	GO:0071219	cellular response to molecule of bacterial origin	20/432	234/18903	4.72461551986466e-07	2.34960718057785e-05	1.60695751411356e-05	CSF3/CXCL1/ASS1/CXCL8/CXCL6/CXCL2/CXCL3/TNIP3/WNT5A/IL1A/IL1B/ABCA1/NFKBIA/XBP1/TRIB1/VIM/TNFAIP3/CAPN2/SERPINE1/ZFP36	20
BP	GO:0043434	response to peptide hormone	28/432	421/18903	4.89397256653467e-07	2.40793862981094e-05	1.6468519106872e-05	TNFSF10/ASS1/GCLC/CYP1B1/INHBA/RAB31/SLC39A14/STAT1/AREG/CAV1/AGTRAP/IL1B/GJB2/NR4A2/IGFBP5/XBP1/JAK1/EGR1/STAT3/ERRFI1/TIMP1/PKM/PTPN1/PIK3R1/CA2/FOS/ZFP36L1/COL1A1	28
BP	GO:0050729	positive regulation of inflammatory response	16/432	154/18903	5.01834328295895e-07	2.44314080880896e-05	1.67092759726223e-05	S100A9/S100A8/LRRK2/CD47/WNT5A/GRN/IL1B/NFKBIA/IL6ST/APP/NFKBIZ/FABP4/SERPINE1/HLA-E/MDK/LGALS1	16
BP	GO:0071346	cellular response to interferon-gamma	14/432	118/18903	5.15730816194343e-07	2.47823071915452e-05	1.69492650038335e-05	CLDN1/ASS1/RAB7B/CD47/STAT1/WNT5A/GBP3/IFNGR1/CCL20/GBP1/SP100/CD58/JAK1/VIM	14
BP	GO:0051346	negative regulation of hydrolase activity	26/432	373/18903	5.19758658936191e-07	2.47823071915452e-05	1.69492650038335e-05	TGFB2/BIRC3/IFI16/BST2/LRRK2/PLAUR/CRYAB/SLC39A14/PRNP/SERPINE2/TNFAIP8/IFI6/APLP2/SLPI/CTSB/APP/BIRC2/SERPING1/LGALS3/WNK1/SERPINE1/TIMP1/CST3/RDX/TMED10/PROS1	26
BP	GO:0002696	positive regulation of leukocyte activation	29/432	450/18903	5.8422641577376e-07	2.75217777280648e-05	1.88228602157234e-05	B2M/CLEC7A/TGFBR2/LRRK2/VAV3/CD74/VNN1/CD47/RUNX1/WNT5A/CAV1/IL1A/IL1B/HLA-A/IL6ST/HLA-DRB1/NFKBIZ/XBP1/CD46/HLA-DRA/IGHG3/IGKC/TFRC/IGHG4/HSPH1/HLA-E/PPP3CA/MDK/LGALS1	29

BP	GO:0051251	positive regulation of lymphocyte activation	27/432	400/18903	5.89114809746685e-07	2.75217777280648e-05	1.88228602157234e-05	B2M/CLEC7A/TGFBR2/VAV3/CD74/VNN1/CD47/RUNX1/CAV1/IL1A/IL1B/HLA-A/IL6ST/HLA-DRB1/NFKBIZ/XBP1/CD46/HLA-DRA/IGHG3/IGKC/TFRC/IGHG4/HSPH1/HLA-E/PPP3CA/MDK/LGALS1	27
BP	GO:1901342	regulation of vasculature development	25/432	355/18903	7.14334030431855e-07	3.30379489074733e-05	2.25955132783971e-05	ANGPTL7/DCN/TGFB2/TGFBR2/CYP1B1/CXCL8/RUNX1/STAT1/WNT5A/GRN/IL1A/IL1B/HIF1A/SP100/XBP1/ID1/JAK1/TNFAIP3/GADD45A/SERPINE1/STAT3/PKM/SULF1/SPARC/MDK	25
BP	GO:0007162	negative regulation of cell adhesion	23/432	308/18903	7.27958034787179e-07	3.33347119890169e-05	2.27984772749919e-05	MMP12/TNFRSF21/ASS1/BMP2/CYP1B1/CD74/IL20RB/RUNX1/DUSP1/FBLN1/PRNP/SERPINE2/IL1RN/HLA-DRB1/GBP1/LGALS3/WNK1/TACSTD2/SERPINE1/RDX/PIK3R1/MDK/COL1A1	23
BP	GO:0043903	regulation of biological process involved in symbiotic interaction	10/432	59/18903	8.09080578804673e-07	3.66862517350158e-05	2.50906825521986e-05	CXCL8/CXCL6/IFITM3/CD74/ITGAV/CAV1/IFITM2/HLA-DRB1/LY6E/LGALS1	10
BP	GO:0006986	response to unfolded protein	15/432	141/18903	8.41786538475796e-07	3.77986673830151e-05	2.58514925714846e-05	OPTN/DNAJB9/HSPA6/XBP1/ATF6/HSPA5/HSPH1/DNAJC3/PTPN1/PIK3R1/TM7SF3/DNAJB4/HSP90AA1/HSPA1A/DNAJB1	15
BP	GO:0022612	gland morphogenesis	14/432	123/18903	8.59879872015021e-07	3.82398500775911e-05	2.61532289008617e-05	TGFB2/TGFBR2/TGFB3/AREG/WNT5A/CAV1/IGFBP5/XBP1/MSN/TNFAIP3/NR3C1/SULF1/MDK/SOSTDC1	14
BP	GO:0051216	cartilage development	18/432	201/18903	8.99474907910467e-07	3.96197280865325e-05	2.70969633911875e-05	MMP13/TGFBR2/BMP2/OGN/SLC39A14/RUNX1/WNT5A/RUNX2/HIF1A/PTHLH/MAF/SULF2/TIMP1/SULF1/WNT2B/NPPC/MDK/COL1A1	18
BP	GO:0051591	response to cAMP	12/432	90/18903	9.53857447825353e-07	4.16187801527571e-05	2.84641671371915e-05	ASS1/CYP1B1/STAT1/AREG/FOSB/APP/BIRC2/IGFBP5/HSPA5/FOS/ZFP36L1/COL1A1	12

BP	GO:0045646	regulation of erythrocyte differentiation	9/432	47/18903	9.87393899938947e-07	4.2679409226333e-05	2.91895589701578e-05	B2M/INHBA/STAT1/HIF1A/ISG15/STAT3/ZFP36/ZFP36L1/HSPA1A	9
BP	GO:0060337	type I interferon signaling pathway	11/432	75/18903	1.02878492590142e-06	4.4056761873093e-05	3.01315662995493e-05	MMP12/OAS2/IFITM3/STAT1/WNT5A/IFITM2/SP100/JAK1/ISG15/PTPN1/IFI27	11
BP	GO:0035966	response to topologically incorrect protein	16/432	163/18903	1.08091899075987e-06	4.57818383292159e-05	3.13113909938649e-05	OPTN/CLU/DNAJB9/HSPA6/XBP1/ATF6/HSPA5/HSPH1/DNAJC3/PTPN1/PIK3R1/TM7SF3/DNAJB4/HSP90AA1/HSPA1A/DNAJB1	16
BP	GO:0032103	positive regulation of response to external stimulus	29/432	464/18903	1.08886534404622e-06	4.57818383292159e-05	3.13113909938649e-05	MMP12/CLEC7A/IFI16/S100A9/S100A8/CXCL8/LRRK2/PDGFD/CD74/CD47/WNT5A/GRN/PLSCR1/OPTN/IL1B/NFKBIA/IL6ST/APP/NEDD9/NFKBIZ/WNK1/PLAU/FABP4/SERPINE1/CXCL17/HLA-E/HSP90AA1/MDK/LGALS1	29
BP	GO:0033002	muscle cell proliferation	20/432	247/18903	1.11003346713989e-06	4.62513944641623e-05	3.16325326577087e-05	TGFB2/TGFBR2/BMP2/SOD2/PDGFD/OGN/TGFB3/RUNX1/ID2/STAT1/IGFBP3/IGFBP5/GJA1/TRIB1/CITED2/TNFAIP3/HBEGF/FOS/JUN/NPPC	20
BP	GO:0019885	antigen processing and presentation of endogenous peptide antigen via MHC class I	6/432	17/18903	1.37549677185792e-06	5.68006479450257e-05	3.88474417239948e-05	B2M/HLA-C/HLA-B/TAPBP/HLA-A/HLA-E	6
BP	GO:0030218	erythrocyte differentiation	14/432	128/18903	1.39707384388812e-06	5.71151885446787e-05	3.90625642279965e-05	B2M/EPAS1/INHBA/ID2/STAT1/HIF1A/CITED2/ISG15/STAT3/PTBP3/ZFP36/KMT2E/ZFP36L1/HSPA1A	14
BP	GO:0006959	humoral immune response	23/432	320/18903	1.40781221493911e-06	5.71151885446787e-05	3.90625642279965e-05	CFH/TNFRSF21/CXCL1/S100A9/C1S/CXCL8/CXCL6/CXCL2/CXCL3/SPRR2A/C1R/IL1B/HLA-A/SLPI/CLU/HLA-DRB1/CD59/SERPING1/CD46/IGHG3/IGKC/IGHG4/HLA-E	23
BP	GO:0002687	positive regulation of leukocyte migration	15/432	148/18903	1.56335983875596e-06	6.28742543847505e-05	4.30013392719602e-05	CXCL8/PDGFD/CD74/CD47/WNT5A/IL1A/APP/NEDD9/CCL20/LGALS3/WNK1/SERPINE1/CXCL17/PIK3R1/MDK	15

BP	GO:0071384	cellular response to corticosteroid stimulus	10/432	64/18903	1.76174389935918e-06	7.02419442632433e-05	4.80402941703661e-05	ASS1/CYP1B1/GJB2/FBXO32/NR3C1/ERRFI1/KLF9/SGK1/ZFP36/ZFP36L1	10
BP	GO:0043122	regulation of I-kappaB kinase/NF-kappaB signaling	20/432	255/18903	1.82175626334096e-06	7.20138693842046e-05	4.92521598860961e-05	TNFSF10/CLEC7A/BIRC3/BST2/TNIP3/CD74/STAT1/WNT5A/IL1A/NDFIP2/OPTN/IL1B/SHISA5/HLA-DRB1/BIRC2/GJA1/TNFAIP3/TFRC/S100A13/LGALS1	20
BP	GO:0002062	chondrocyte differentiation	13/432	114/18903	2.07075108516003e-06	8.04808720072702e-05	5.50429633870491e-05	TGFBR2/BMP2/SLC39A14/RUNX1/RUNX2/HIF1A/PTHLH/MAF/SULF2/SULF1/WNT2B/NPPC/MDK	13
BP	GO:0002526	acute inflammatory response	13/432	114/18903	2.07075108516003e-06	8.04808720072702e-05	5.50429633870491e-05	SAA1/SAA2/ASS1/S100A8/VNN1/IL20RB/IL1A/PLSCR1/IL1B/IL6ST/B4GALT1/TFRC/HLA-E	13
BP	GO:0032570	response to progesterone	8/432	39/18903	2.30476721677971e-06	8.88295698133845e-05	6.07528551440615e-05	TGFB2/CYP1B1/TGFB3/CAV1/FOSB/GJB2/FOS/TXNIP	8
BP	GO:0071375	cellular response to peptide hormone stimulus	22/432	306/18903	2.37506657140203e-06	9.07825032457388e-05	6.20885171558339e-05	ASS1/GCLC/CYP1B1/INHBA/RAB31/SLC39A14/STAT1/CAV1/AGTRAP/IL1B/GJB2/NR4A2/XBP1/JAK1/STAT3/ERRFI1/PKM/PTPN1/PIK3R1/CA2/FOS/ZFP36L1	22
BP	GO:0031349	positive regulation of defense response	22/432	307/18903	2.50530491118074e-06	9.4203538326918e-05	6.44282521018239e-05	MMP12/CLEC7A/IFI16/S100A9/S100A8/LRRK2/CD47/WNT5A/GRN/PLSCR1/OPTN/IL1B/NFKBIA/IL6ST/APP/NFKBIZ/FABP4/SERPINE1/HLA-E/HSP90AA1/MDK/LGALS1	22
BP	GO:0044403	biological process involved in symbiotic interaction	22/432	307/18903	2.50530491118074e-06	9.4203538326918e-05	6.44282521018239e-05	CLDN1/APOL1/CXCL8/CXCL6/SPRR2A/IFIT1/IFITM3/CD74/ITGAV/CAV1/IFITM2/PLSCR1/CTSB/STOM/HLA-DRB1/CD46/TFRC/LY6E/IFI27/JUN/HSPA1A/LGALS1	22
BP	GO:0007179	transforming growth factor beta	18/432	216/18903	2.54003431099794e-06	9.47391829706892e-05	6.47945934172225e-05	TGFB2/TGFBR2/BMP2/DAB2/TGFB3/HTRA1/PMEPA1/CAV1/ID1/SKIL/CITED2/STAT3/HSPA5/NREP/FOS/JUN/SINHCAF/HSPA1A	18

		receptor signaling pathway							
BP	GO:0007584	response to nutrient	15/432	154/18903	2.58012747577 44e-06	9.54647166036 528e-05	6.52908047553 858e-05	CYP24A1/KYNU/ASS1/TGFBR2/GCLC/CYP1B1/SOD2/STAT1/IL1A/SLC16A1/RUNX2/XBP1/TFRC/POSTN/COL1A1	15
BP	GO:0048660	regulation of smooth muscle cell proliferation	16/432	175/18903	2.76850176753 07e-06	0.00010162159265737 7	6.95016525599 812e-05	TGFBR2/BMP2/SOD2/PDGFD/OGN/TGFB3/ID2/STAT1/IGFBP3/IGFBP5/GJA1/TRIB1/TNFAIP3/HBEGF/JUN/NPPC	16
BP	GO:0002478	antigen processing and presentation of exogenous peptide antigen	8/432	40/18903	2.82388706671 43e-06	0.00010277959689347 8	7.02936418013 997e-05	B2M/CD74/HLA-A/HLA-DRB1/CTSV/CTSD/HLA-DRA/HLA-E	8
BP	GO:0052372	modulation by symbiont of entry into host	9/432	53/18903	2.84449478970 057e-06	0.00010277959689347 8	7.02936418013 997e-05	CXCL8/IFITM3/CD74/ITGAV/CAV1/IFITM2/HLA-DRB1/LY6E/LGALS1	9
BP	GO:0002573	myeloid leukocyte differentiation	18/432	218/18903	2.89554297635 851e-06	0.00010381307182680 7	7.10004622109 941e-05	CSF3/IFI16/TGFBR2/INHBA/CD74/RUNX1/ID2/APP/NEDD9/HLA-DRB1/TRIB1/CITED2/TFRC/PPP3CA/PIK3R1/FOS/JUN/ZFP36L1	18
BP	GO:0010466	negative regulation of peptidase activity	20/432	263/18903	2.92696550176 147e-06	0.00010413242650497 5	7.12188771886 09e-05	BIRC3/IFI16/BST2/PLAUR/CRYAB/PRNP/SERPINE2/TNFAIP8/IFI6/APLP2/SLPI/CTSB/APP/BIRC2/SERPING1/SERPINE1/TIMP1/CST3/TMED10/PROS1	20
BP	GO:0042445	hormone metabolic process	19/432	241/18903	3.05459817117 025e-06	0.00010784363772261 4	7.37570711479 84e-05	CPE/BMP2/CYP1B1/DAB2/SDR16C5/TIPARP/SELENOM/MME/RDH10/DHRS3/SCPEP1/CTSB/ATP1A1/HIF1A/EGR1/NR3C1/AKR1B1/CRABP2/DIO2	19

BP	GO:0034101	erythrocyte homeostasis	14/432	137/18903	3.15615672280162e-06	0.000110585036689072	7.56319852633084e-05	B2M/EPAS1/INHBA/ID2/STAT1/HIF1A/CITED2/ISG15/STAT3/PTBP3/ZFP36/KMT2E/ZFP36L1/HSPA1A	14
BP	GO:0007249	I-kappaB kinase/NF-kappaB signaling	21/432	288/18903	3.22656293265779e-06	0.000112201906492799	7.67378046112913e-05	TNFSF10/CLEC7A/BIRC3/BST2/TNIP3/CD74/STAT1/WNT5A/IL1A/NDFIP2/OPTN/IL1B/NFKBIA/SHISA5/HLA-DRB1/BIRC2/GJA1/TNFAIP3/TFRC/S100A13/LGALS1	21
BP	GO:0044409	entry into host	15/432	158/18903	3.55307463614444e-06	0.000122634105911702	8.38726573575337e-05	CLDN1/CXCL8/IFITM3/CD74/ITGAV/CAV1/IFITM2/PLSCR1/CTSB/HLA-DRB1/CD46/TFRC/LY6E/HSPA1A/LGALS1	15
BP	GO:0048659	smooth muscle cell proliferation	16/432	179/18903	3.71704908960942e-06	0.000127343348440323	8.70934309105365e-05	TGFBR2/BMP2/SOD2/PDGFD/OGN/TGFB3/ID2/STAT1/IGFBP3/IGFBP5/GJA1/TRIB1/TNFAIP3/HBEGF/JUN/NPPC	16
BP	GO:0048732	gland development	27/432	441/18903	3.77738002306518e-06	0.000128458695637327	8.78562458924989e-05	CLDN1/TGFB2/PITX2/ASS1/TGFBR2/BMP2/CYP1B1/OAS2/SOD2/TGFB3/ID2/AREG/WNT5A/CAV1/SERPINE2/IGFBP5/HIF1A/XBP1/CITED2/MSN/TNFAIP3/NR3C1/PBX1/JUN/SULF1/MDK/SOSTDC1	27
BP	GO:0007178	transmembrane receptor protein serine/threonine kinase signaling pathway	25/432	390/18903	3.89011567842504e-06	0.000131326897903035	8.98178840850346e-05	TGFB2/TGFBR2/BMP2/DAB2/INHBA/TGFB3/HTRA1/PMEPA1/WNT5A/CAV1/RUNX2/ID1/SKIL/CITED2/EGR1/VIM/STAT3/HSPA5/NREP/FOS/JUN/SULF1/SINHCAF/HSPA1A/SOSTDC1	25
BP	GO:0071385	cellular response to glucocorticoid stimulus	9/432	55/18903	3.91967871051234e-06	0.000131366043739997	8.98446569419495e-05	ASS1/CYP1B1/GJB2/FBXO32/NR3C1/ERRFI1/KLF9/ZFP36/ZFP36L1	9

BP	GO:0006457	protein folding	18/432	223/18903	3.98904960999549e-06	0.000132729168677907	9.07769335708932e-05	B2M/CD74/CRYAB/PPIC/GRN/CLU/HSPA6/LMAN1/ATF6/HSPA5/HSPH1/DNAJC3/FKBP5/DNAJB4/HSP90AA1/NPPC/HSPA1A/DNAJB1	18
BP	GO:1902105	regulation of leukocyte differentiation	22/432	317/18903	4.21552231469019e-06	0.000139262790753158	9.52454477867971e-05	TMEM176A/TGFBR2/TMEM176B/HLA-B/INHBA/CD74/VNN1/RUNX1/ID2/PRDM1/NEDD9/HLA-DRB1/NFKBIZ/XBP1/TRIB1/CD46/HLA-DRA/PPP3CA/PIK3R1/FOS/ZFP36L1/MDK	22
BP	GO:0002698	negative regulation of immune effector process	13/432	122/18903	4.44930073617845e-06	0.000144915604963559	9.91115545753613e-05	TGFB2/BST2/HLA-B/TGFB3/IL20RB/CD47/GRN/HLA-A/CD59/SERPING1/LGALS3/CD46/HLA-E	13
BP	GO:0030278	regulation of ossification	13/432	122/18903	4.44930073617845e-06	0.000144915604963559	9.91115545753613e-05	TGFB2/BMP2/TGFB3/OMD/WNT5A/ADRB2/DHRS3/ANKH/RUNX2/HIF1A/ISG15/PBX1/MDK	13
BP	GO:0030098	lymphocyte differentiation	26/432	419/18903	4.4934704607043e-06	0.000145236049221372	9.93307147448589e-05	B2M/TGFBR2/RSAD2/INHBA/CD74/VNN1/RUNX1/ID2/IL1A/PRDM1/IL1B/HLA-DRB1/RUNX2/DNAJB9/NFKBIZ/XBP1/EGR1/CD46/HLA-DRA/STAT3/PBX1/PIK3R1/KLF6/ZFP36L1/MDK/LGALS1	26
BP	GO:0007596	blood coagulation	18/432	225/18903	4.52194401900056e-06	0.000145236049221372	9.93307147448589e-05	SAA1/SLC7A11/PLAUR/PLEK/VAV3/FBLN1/CAV1/SERPINE2/PLSCR1/IL6ST/CD59/SERPING1/PLAU/LMAN1/MYL9/SERPINE1/ANXA5/PROS1	18
BP	GO:0071675	regulation of mononuclear cell migration	13/432	123/18903	4.87350502012504e-06	0.000155448004952264	0.000106314937099643	PDGFD/CD47/WNT5A/DUSP1/APP/NEDD9/CCL20/LGALS3/WNK1/MSN/SERPINE1/CXCL17/MDK	13

BP	GO:0045926	negative regulation of growth	19/432	249/18903	4.92262708343078e-06	0.000155939385348407	0.000106651004943832	TGFB2/TGFBR2/BST2/DAB2/INHBA/CRYAB/RGS4/WNT5A/OSGIN1/SERPINE2/ADRB2/IGFBP5/HIF1A/GJA1/CITED2/SEMA6D/MT2A/MT1E/HSPA1A	19
BP	GO:0062197	cellular response to chemical stress	23/432	345/18903	4.99057003448166e-06	0.000157016234078079	0.000107387489821822	SLC7A11/EPAS1/NCOA7/CYP1B1/SOD2/LRRK2/PDGFD/VNN1/CAV1/GJB2/NR4A2/LRRC8D/HIF1A/PNPLA8/ARL6IP5/TNFAIP3/ERRFI1/MCL1/AKR1B1/FOS/JUN/ZFP36L1/HSPA1A	23
BP	GO:0055076	transition metal ion homeostasis	14/432	143/18903	5.23311398067785e-06	0.00016275805975351	0.000111314473868403	B2M/S100A9/S100A8/EPAS1/SOD2/SLC39A14/HEPHL1/PRNP/APP/HIF1A/TFRC/SLC39A6/MT2A/MT1E	14
BP	GO:1902107	positive regulation of leukocyte differentiation	16/432	184/18903	5.30693812762308e-06	0.00016275805975351	0.000111314473868403	TGFBR2/CD74/VNN1/RUNX1/ID2/NEDD9/HLA-DRB1/NFKBIZ/XBP1/TRIB1/CD46/HLA-DRA/PPP3CA/FOS/ZFP36L1/MDK	16
BP	GO:1903708	positive regulation of hemopoiesis	16/432	184/18903	5.30693812762308e-06	0.00016275805975351	0.000111314473868403	TGFBR2/CD74/VNN1/RUNX1/ID2/NEDD9/HLA-DRB1/NFKBIZ/XBP1/TRIB1/CD46/HLA-DRA/PPP3CA/FOS/ZFP36L1/MDK	16
BP	GO:0002253	activation of immune response	25/432	397/18903	5.3138307076281e-06	0.00016275805975351	0.000111314473868403	CFH/TNFRSF21/CLEC7A/IFI16/C1S/C1R/VAV3/CD47/PRNP/PLSCR1/IL1B/HLA-A/CLU/HLA-DRB1/NFKBIZ/CD59/GBP1/SERPING1/LGALS3/WNK1/CD46/IGHG3/IGKC/IGHG4/HSP90AA1	25
BP	GO:0097193	intrinsic apoptotic signaling pathway	21/432	298/18903	5.49786293918145e-06	0.000167286947984962	0.000114411898422717	IFI16/S100A9/S100A8/CYP1B1/SOD2/LRRK2/PLAUR/CD74/VNN1/CAV1/SHISA5/IFI6/CLU/HIF1A/XBP1/ARL6IP5/SKIL/MCL1/PTPN1/PIK3R1/HSPA1A	21

BP	GO:0010951	negative regulation of endopeptidase activity	19/432	252/18903	5.85486962224433e-06	0.000176985437927326	0.000121044948158543	BIRC3/IFI16/BST2/PLAUR/CRYAB/PRNP/SERPINE2/TNFAIP8/IFI6/APLP2/SLPI/APP/BIRC2/SERPING1/SERPINE1/TIMP1/CST3/TMED10/PROS1	19
BP	GO:0071677	positive regulation of mononuclear cell migration	10/432	73/18903	6.0173044910121e-06	0.000180071429543076	0.000123155538156915	PDGFD/CD47/WNT5A/APP/NEDD9/CCL20/LGALS3/WNK1/SERPINE1/CXCL17	10
BP	GO:0045765	regulation of angiogenesis	23/432	349/18903	6.03482628738958e-06	0.000180071429543076	0.000123155538156915	DCN/TGFB2/TGFBR2/CYP1B1/CXCL8/RUNX1/STAT1/WNT5A/GRN/IL1A/IL1B/HIF1A/SP100/XBP1/JAK1/TNFAIP3/GADD45A/SERPINE1/STAT3/PKM/SULF1/SPARC/MDK	23
BP	GO:0050817	coagulation	18/432	230/18903	6.14574328661884e-06	0.000182205530132129	0.000124615105103169	SAA1/SLC7A11/PLAUR/PLEK/VAV3/FBLN1/CAV1/SERPINE2/PLSCR1/IL6ST/CD59/SERPING1/PLAU/LMAN1/MYL9/SERPINE1/ANXA5/PROS1	18
BP	GO:0035456	response to interferon-beta	7/432	32/18903	6.36738613912254e-06	0.000186387094262289	0.000127474985663313	IFI16/BST2/IFITM3/STAT1/IFITM2/PLSCR1/CAPN2	7
BP	GO:0051085	chaperone cofactor-dependent protein refolding	7/432	32/18903	6.36738613912254e-06	0.000186387094262289	0.000127474985663313	CD74/HSPA6/HSPA5/HSPH1/DNAJB4/HSPA1A/DNAJB1	7
BP	GO:0002685	regulation of leukocyte migration	18/432	231/18903	6.52740893488353e-06	0.000188682914523977	0.000129045156903454	MMP28/CXCL8/PDGFD/CD74/CD47/WNT5A/DUSP1/IL1A/APP/NEDD9/CCL20/LGALS3/WNK1/MSN/SERPINE1/CXCL17/PIK3R1/MDK	18

BP	GO:0007599	hemostasis	18/432	231/18903	6.52740893488353e-06	0.000188682914523977	0.000129045156903454	SAA1/SLC7A11/PLAUR/PLEK/VAV3/FBLN1/CAV1/SERPINE2/PLSCR1/IL6ST/CD59/SERPING1/PLAU/LMAN1/MYL9/SERPINE1/ANXA5/PROS1	18
BP	GO:0019882	antigen processing and presentation	12/432	108/18903	6.70643351056739e-06	0.000192378897006887	0.000131572935534724	B2M/HLA-C/HLA-B/CD74/TAPBP/HLA-A/HLA-DRB1/PSMB8/CTSV/CTSD/HLA-DRA/HLA-E	12
BP	GO:0070486	leukocyte aggregation	5/432	13/18903	6.73846082488987e-06	0.000192378897006887	0.000131572935534724	S100A9/S100A8/HAS2/IL1B/MSN	5
BP	GO:0045861	negative regulation of proteolysis	23/432	352/18903	6.94434861507481e-06	0.000197040566532031	0.000134761172672262	BIRC3/IFI16/BST2/LRRK2/PLAUR/CRYAB/PRNP/SERPINE2/TNFAIP8/N4BP1/IFI6/APLP2/SLPI/CTSB/APP/BIRC2/SERPING1/PLAU/SERPINE1/TIMP1/CST3/TMED10/PROS1	23
BP	GO:0002763	positive regulation of myeloid leukocyte differentiation	9/432	59/18903	7.14541354490564e-06	0.000199592849679682	0.000136506745556175	CD74/RUNX1/ID2/NEDD9/HLA-DRB1/TRIB1/PPP3CA/FOS/ZFP36L1	9
BP	GO:0030574	collagen catabolic process	8/432	45/18903	7.16195612542932e-06	0.000199592849679682	0.000136506745556175	MMP13/MMP7/MMP10/MMP12/MMP28/MMP1/CTSB/CST3	8
BP	GO:1901216	positive regulation of neuron death	11/432	91/18903	7.16376498309777e-06	0.000199592849679682	0.000136506745556175	TGFB2/WNT5A/GRN/PRNP/CLU/SRPK2/EGR1/CAPN2/NR3C1/MCL1/FOS	11

BP	GO:0010042	response to manganese ion	6/432	22/18903	7.52544640193364e-06	0.000208414309035587	0.000142539971243685	FIBIN/SOD2/LRRK2/APP/TFRC/HSPA5	6
BP	GO:0046683	response to organophosphorus	13/432	128/18903	7.57765113313099e-06	0.000208610931492445	0.000142674446460267	ASS1/CYP1B1/STAT1/AREG/IL1B/FOSB/APP/BIRC2/IGFBP5/HSPA5/FOS/ZFP36L1/COL1A1	13
BP	GO:0002262	myeloid cell homeostasis	15/432	169/18903	8.12574012825195e-06	0.000222376024219913	0.000152088751699764	B2M/SLC7A11/EPAS1/INHBA/ID2/STAT1/HIF1A/CITED2/ISG15/STAT3/PTBP3/ZFP36/KMT2E/ZFP36L1/HSPA1A	15
BP	GO:0032102	negative regulation of response to external stimulus	26/432	436/18903	9.12517709256754e-06	0.000248258494430146	0.000169790446830746	SAA1/MMP12/MMP28/IFI16/HLA-B/PLAUR/IL20RB/HTRA1/WNT5A/DUSP1/GRN/SERPINE2/HLA-A/HLA-DRB1/SERPING1/TRIB1/PLAU/ISG15/TNFAIP3/SERPINE1/CXCL17/HLA-E/SEMA6D/ZFP36/PROS1/MDK	26
BP	GO:0001822	kidney development	21/432	309/18903	9.5967860581009e-06	0.000259562196015887	0.000177521342595218	TGFB2/ASS1/HAS2/BMP2/LRRK2/PDGFD/BASP1/TIPARP/STAT1/WNT5A/MME/RDH10/PRDM1/TACSTD2/EGR1/SULF2/PBX1/PPP3CA/AKR1B1/SULF1/WNT2B	21
BP	GO:0046718	viral entry into host cell	14/432	151/18903	9.85001660048761e-06	0.000264862364984042	0.000181146266122798	CLDN1/IFITM3/CD74/ITGAV/CAV1/IFITM2/PLSCR1/CTSB/HLA-DRB1/CD46/TFRC/LY6E/HSPA1A/LGALS1	14
BP	GO:1902895	positive regulation of miRNA transcription	8/432	47/18903	1.0038921277459e-05	0.000267308242743599	0.000182819065591924	TGFB2/BMP2/HIF1A/EGR1/NR3C1/STAT3/FOS/JUN	8

BP	GO:2001242	regulation of intrinsic apoptotic signaling pathway	15/432	172/18903	1.00565695648403e-05	0.000267308242743599	0.000182819065591924	S100A9/S100A8/SOD2/LRRK2/PLAUR/CD74/VNN1/CAV1/CLU/HIF1A/XBP1/SKIL/MCL1/PTPN1/HSPA1A	15
BP	GO:0016049	cell growth	28/432	493/18903	1.02700453067442e-05	0.000271422625963953	0.000185632999379045	TGFB2/S100A9/NRCAM/S100A8/TGFBR2/BST2/DAB2/INHBA/CRYAB/RGS4/WNT5A/SLC23A2/OSGIN1/IGFBP3/SERPINE2/SORBS2/CXCL16/APP/IGFBP5/GJA1/HBEGF/MTPN/SGK1/SEMA6D/CRABP2/IGFBP7/HSP90AA1/HSPA1A	28
BP	GO:0045936	negative regulation of phosphate metabolic process	26/432	440/18903	1.07135232168339e-05	0.000280225820390478	0.000191653733205892	SAMSN1/TGFB2/BMP2/LRRK2/PLEK/INHBA/PMEPA1/DUSP1/FBLN1/CAV1/PRNP/IGFBP3/IL1B/ANKLE2/LGALS3/TRIB1/PTPN13/TNFAIP3/GADD45A/FABP4/STAT3/ERRFI1/DNAJC3/PTPN1/GADD45B/JUN	26
BP	GO:0007229	integrin-mediated signaling pathway	12/432	113/18903	1.07243178830518e-05	0.000280225820390478	0.000191653733205892	LAMA3/PLEK/VAV3/ITGA6/CD47/ITGAV/LAMB1/NEDD9/ISG15/CD63/TIMP1/DST	12
BP	GO:0045088	regulation of innate immune response	18/432	240/18903	1.10485636004485e-05	0.000287076441865587	0.000196339051563263	CFH/MMP12/CLEC7A/BIRC3/IFI16/HLA-B/WNT5A/GRN/PLSCR1/HLA-A/N4BP1/BIRC2/SERPING1/ISG15/TNFAIP3/HLA-E/PTPN1/HSP90AA1	18
BP	GO:0010563	negative regulation of phosphorus metabolic process	26/432	441/18903	1.1148076766323e-05	0.000288043882928737	0.000197000709690095	SAMSN1/TGFB2/BMP2/LRRK2/PLEK/INHBA/PMEPA1/DUSP1/FBLN1/CAV1/PRNP/IGFBP3/IL1B/ANKLE2/LGALS3/TRIB1/PTPN13/TNFAIP3/GADD45A/FABP4/STAT3/ERRFI1/DNAJC3/PTPN1/GADD45B/JUN	26
BP	GO:0090287	regulation of cellular response to growth factor stimulus	22/432	338/18903	1.16704599725946e-05	0.000299865985406946	0.000205086153319572	DCN/BMP2/DAB2/TGFB3/HTRA1/PMEPA1/WNT5A/CAV1/IL1B/RUNX2/HIF1A/SKIL/CITED2/HSPA5/SULF2/CD63/PTPN1/NREP/SULF1/SINHCAF/HSPA1A/SOSTDC1	22

BP	GO:0032623	interleukin-2 production	9/432	63/18903	1.24155161343 23e-05	0.000313780120881113	0.000214602392773313	CLEC7A/IL20RB/RUNX1/IL1A/PRNP/IL1B/GBP1/TNFAIP3/ZFP36	9
BP	GO:0032663	regulation of interleukin-2 production	9/432	63/18903	1.24155161343 23e-05	0.000313780120881113	0.000214602392773313	CLEC7A/IL20RB/RUNX1/IL1A/PRNP/IL1B/GBP1/TNFAIP3/ZFP36	9
BP	GO:0032757	positive regulation of interleukin-8 production	9/432	63/18903	1.24155161343 23e-05	0.000313780120881113	0.000214602392773313	CLEC7A/CD74/WNT5A/AFAP1L2/IL1B/CD58/SERPINE1/STAT3/HSPA1A	9
BP	GO:0060571	morphogenesis of an epithelial fold	6/432	24/18903	1.30589572806 637e-05	0.000328248246864508	0.000224497520757404	WNT5A/RDH10/HIF1A/SULF1/WNT2B/SOSTDC1	6
BP	GO:0040013	negative regulation of locomotion	25/432	419/18903	1.34373669419 056e-05	0.000334767076567623	0.000228955918084 94	DCN/MMP28/BST2/CYP1B1/CD74/WNT5A/DUSP1/FBLN1/IGFBP3/MCC/NEDD9/IGFBP5/SP100/GJA1/TRIB1/TACSTD2/CITED2/GADD45A/SERPINE1/STAT3/ARRDC3/TIMP1/SEMA6D/SULF1/SINHCAF	25
BP	GO:0002819	regulation of adaptive immune response	16/432	198/18903	1.34630651333 141e-05	0.000334767076567623	0.000228955918084 94	B2M/CLEC7A/SAMSN1/RSAD2/HLA-B/IL20RB/IL1B/HLA-A/IL6ST/HLA-DRB1/NFKBIZ/CD46/TNFAIP3/HLA-DRA/TFRC/HLA-E	16
BP	GO:0019884	antigen processing and presentation of exogenous antigen	8/432	49/18903	1.38336572696 342e-05	0.000342142592898708	0.000234000225698007	B2M/CD74/HLA-A/HLA-DRB1/CTSV/CTSD/HLA-DRA/HLA-E	8

BP	GO:0072001	renal system development	21/432	318/18903	1.48202247964593e-05	0.00036459329618949	0.00024935484609944	TGFB2/ASS1/HAS2/BMP2/LRRK2/PDGFD/BASP1/TIPARP/STAT1/WNT5A/MME/RDH10/PRDM1/TACSTD2/EGR1/SULF2/PBX1/PPP3CA/AKR1B1/SULF1/WNT2B	21
BP	GO:0060572	morphogenesis of an epithelial bud	5/432	15/18903	1.51390714333765e-05	0.000370466695128921	0.000253371816526295	WNT5A/RDH10/SULF1/WNT2B/SOSTDC1	5
BP	GO:2001237	negative regulation of extrinsic apoptotic signaling pathway	11/432	99/18903	1.62028102447371e-05	0.000392940118804069	0.000268741975990038	GCLC/ITGA6/ITGAV/IL1A/IL1B/IFI6/LGALS3/TNFAIP3/SERPINE1/MCL1/HSPA1A	11
BP	GO:0048771	tissue remodeling	15/432	179/18903	1.6227364906287e-05	0.000392940118804069	0.000268741975990038	EPAS1/TGFB3/RUNX1/CAV1/IL1A/ADRB2/IGFBP5/HIF1A/GJA1/TNFAIP3/TFRC/TIMP1/PPP3CA/CST3/MDK	15
BP	GO:0046885	regulation of hormone biosynthetic process	6/432	25/18903	1.68530778260127e-05	0.000405966067423484	0.000277650761333316	BMP2/DAB2/ATP1A1/HIF1A/EGR1/NR3C1	6
BP	GO:0030217	T cell differentiation	20/432	296/18903	1.69799798812688e-05	0.000406903662957867	0.000278292007325949	B2M/TGFBR2/RSAD2/CD74/VNN1/RUNX1/IL1A/PRDM1/IL1B/HLA-DRB1/RUNX2/NFKBIZ/XBP1/EGR1/CD46/HLA-DRA/STAT3/PIK3R1/ZFP36L1/MDK	20
BP	GO:1905897	regulation of response to endoplasmic reticulum stress	10/432	82/18903	1.72561908589664e-05	0.000411391148055257	0.00028136111519910	LRRK2/CAV1/CLU/DNAJB9/XBP1/ATF6/HSPA5/PTPN1/PIK3R1/HSPA1A	10

BP	GO:0051084	'de novo' post-translational protein folding	7/432	37/18903	1.76477518662078e-05	0.0004185684737498	0.000286269875076677	CD74/HSPA6/HSPA5/HSPH1/DNAJB4/HSPA1A/DNAJB1	7
BP	GO:0051701	biological process involved in interaction with host	16/432	203/18903	1.83715501204974e-05	0.000433345413180924	0.000296376208616484	CLDN1/CXCL8/IFIT1/IFITM3/CD74/ITGAV/CAV1/IFITM2/PLSCR1/CTSB/HLA-DRB1/CD46/TFRC/LY6E/HSPA1A/LGALS1	16
BP	GO:0051090	regulation of DNA-binding transcription factor activity	26/432	454/18903	1.84581721938685e-05	0.000433345413180924	0.000296376208616484	CSF3/CLEC7A/S100A9/S100A8/BMP2/CYP1B1/RAB7B/BHLHE40/ID2/WNT5A/CAV1/PRNP/IL1B/NFKBIA/CLU/APP/SP100/ID1/TRIB1/TNFAIP3/TFRC/STAT3/PPP3CA/MTPN/SGK1/HSPA1A	26
BP	GO:0002460	adaptive immune response based on somatic recombination of immune receptors built from immunoglobulin superfamily domains	23/432	375/18903	1.92460149515162e-05	0.000449559692680617	0.000307465576445008	B2M/CLEC7A/C1S/C1R/RSAD2/HLA-B/CD74/IL20RB/IL1B/HLA-A/CLU/HLA-DRB1/NFKBIZ/SERPING1/CD46/TNFAIP3/HLA-DRA/IGHG3/IGKC/TFRC/STAT3/IGHG4/HLA-E	23
BP	GO:0002821	positive regulation of adaptive immune response	12/432	120/18903	1.98175165388939e-05	0.000460582984886352	0.000315004692935076	B2M/CLEC7A/RSAD2/HLA-B/IL1B/HLA-A/IL6ST/HLA-DRB1/NFKBIZ/HLA-DRA/TFRC/HLA-E	12
BP	GO:0044000	movement in host	15/432	183/18903	2.10907358732318e-05	0.000487723267068486	0.000333566638416114	CLDN1/CXCL8/IFITM3/CD74/ITGAV/CAV1/IFITM2/PLSCR1/CTSB/HLA-DRB1/CD46/TFRC/LY6E/HSPA1A/LGALS1	15

BP	GO:0032640	tumor necrosis factor production	15/432	184/18903	2.24916646672206e-05	0.00051497004497968	0.000352201419098478	CLEC7A/OAS2/LRRK2/CD47/WNT5A/IL1A/CLU/IFNGR1/APP/TNFAIP3/STAT3/ERRFI1/HLA-E/PIK3R1/ZFP36	15
BP	GO:0032680	regulation of tumor necrosis factor production	15/432	184/18903	2.24916646672206e-05	0.00051497004497968	0.000352201419098478	CLEC7A/OAS2/LRRK2/CD47/WNT5A/IL1A/CLU/IFNGR1/APP/TNFAIP3/STAT3/ERRFI1/HLA-E/PIK3R1/ZFP36	15
BP	GO:0014074	response to purine-containing compound	13/432	142/18903	2.33378606391239e-05	0.000531712342147527	0.000363651911955236	ASS1/CYP1B1/STAT1/AREG/IL1B/FOSB/APP/BIRC2/IGFBP5/HSPA5/FOS/ZFP36L1/COL1A1	13
BP	GO:0042326	negative regulation of phosphorylation	23/432	381/18903	2.47210055262514e-05	0.000560463973328005	0.000383315900961742	SAMSN1/BMP2/LRRK2/INHBA/PMEPA1/DUSP1/FBLN1/CAV1/PRNP/IGFBP3/IL1B/ANKLE2/TRIB1/PTPN13/TNFAIP3/GADD45A/FABP4/STAT3/ERRFI1/DNAJC3/PTPN1/GADD45B/JUN	23
BP	GO:0051402	neuron apoptotic process	18/432	255/18903	2.50291010994615e-05	0.000563590933120773	0.000385454510162828	TNFRSF21/TGFB2/GCLC/SOD2/TGFB3/GRN/PRNP/NR4A2/IL6ST/APP/HIF1A/SRPK2/NR3C1/HSPA5/MCL1/CHL1/JUN/MDK	18
BP	GO:0032350	regulation of hormone metabolic process	7/432	39/18903	2.53463598030532e-05	0.000563590933120773	0.000385454510162828	BMP2/DAB2/RDH10/ATP1A1/HIF1A/EGR1/NR3C1	7
BP	GO:0043618	regulation of transcription from RNA polymerase II promoter in response to stress	7/432	39/18903	2.53463598030532e-05	0.000563590933120773	0.000385454510162828	EPAS1/HIF1A/CITED2/EGR1/ATF6/HSPA5/JUN	7

BP	GO:0071634	regulation of transforming growth factor beta production	7/432	39/18903	2.53463598030532e-05	0.000563590933120773	0.000385454510162828	TGFB2/LUM/ITGAV/CDH3/HIF1A/LAPTM4B/CD46	7
BP	GO:0009266	response to temperature stimulus	15/432	186/18903	2.55423511177052e-05	0.000565231454159744	0.000386576505206266	GCLC/SOD2/CRYAB/IL1A/ADRB2/NFKBIA/HSPA6/DNAJC3/DNAJB4/FOS/IGFBP7/HSP90AA1/DIO2/HSPA1A/DNAJB1	15
BP	GO:2000146	negative regulation of cell motility	23/432	383/18903	2.68362015868054e-05	0.000590618506966064	0.000403939371478355	DCN/MMP28/BST2/CYP1B1/CD74/DUSP1/FBLN1/IGFBP3/MCC/NEDD9/IGFBP5/SP100/GJA1/TRIB1/TACSTD2/CITED2/GADD45A/SERPINE1/STAT3/TIMP1/SEMA6D/SULF1/SINHCAF	23
BP	GO:0042026	protein refolding	6/432	27/18903	2.70985346419458e-05	0.000590618506966064	0.000403939371478355	B2M/CRYAB/HSPA6/HSPA5/HSP90AA1/HSPA1A	6
BP	GO:0045766	positive regulation of angiogenesis	15/432	187/18903	2.72003766451398e-05	0.000590618506966064	0.000403939371478355	TGFBR2/CYP1B1/CXCL8/RUNX1/WNT5A/GRN/IL1A/IL1B/HIF1A/XBP1/JAK1/SERPINE1/STAT3/PKM/MDK	15
BP	GO:1904018	positive regulation of vasculature development	15/432	187/18903	2.72003766451398e-05	0.000590618506966064	0.000403939371478355	TGFBR2/CYP1B1/CXCL8/RUNX1/WNT5A/GRN/IL1A/IL1B/HIF1A/XBP1/JAK1/SERPINE1/STAT3/PKM/MDK	15
BP	GO:0032760	positive regulation of tumor necrosis factor production	11/432	105/18903	2.83335608561341e-05	0.000609501018416839	0.000416853612595755	CLEC7A/OAS2/LRRK2/WNT5A/IL1A/CLU/IFNGR1/APP/STAT3/HLA-E/PIK3R1	11

BP	GO:1901655	cellular response to ketone	11/432	105/18903	2.83335608561341e-05	0.000609501018416839	0.000416853612595755	ASS1/CYP1B1/GJB2/FBXO32/MSN/NR3C1/ERRFI1/KLF9/SGK1/FOS/POSTN	11
BP	GO:0014910	regulation of smooth muscle cell migration	10/432	87/18903	2.91579440899296e-05	0.000624330978777429	0.000426996208529428	HAS2/CYP1B1/PDGFD/IGFBP3/IGFBP5/TRIB1/PLAU/SERPINE1/SEMA6D/MDK	10
BP	GO:0090594	inflammatory response to wounding	5/432	17/18903	3.00381652652609e-05	0.000640214351851759	0.000437859260839724	GRN/IL1A/HIF1A/TIMP1/MDK	5
BP	GO:2000116	regulation of cysteine-type endopeptidase activity	17/432	235/18903	3.06923566074309e-05	0.000644647969546244	0.000440891527393790	TNFSF10/CLEC7A/BIRC3/IFI16/S100A9/S100A8/PLAUR/PSMB9/CRYAB/TNFAIP8/IFI6/BIRC2/ARL6IP5/CTSD/ASPH/CST3/UACA	17
BP	GO:0043123	positive regulation of I-kappaB kinase/NF-kappaB signaling	15/432	189/18903	3.0803719193453e-05	0.000644647969546244	0.000440891527393790	TNFSF10/CLEC7A/BIRC3/BST2/CD74/IL1A/NDFIP2/IL1B/SHISA5/HLA-DRB1/BIRC2/GJA1/TFRC/S100A13/LGALS1	15
BP	GO:0071706	tumor necrosis factor superfamily cytokine production	15/432	189/18903	3.0803719193453e-05	0.000644647969546244	0.000440891527393790	CLEC7A/OAS2/LRRK2/CD47/WNT5A/IL1A/CLU/IFNGR1/APP/TNFAIP3/STAT3/ERRFI1/HLA-E/PIK3R1/ZFP36	15
BP	GO:1903555	regulation of tumor necrosis factor superfamily cytokine production	15/432	189/18903	3.0803719193453e-05	0.000644647969546244	0.000440891527393790	CLEC7A/OAS2/LRRK2/CD47/WNT5A/IL1A/CLU/IFNGR1/APP/TNFAIP3/STAT3/ERRFI1/HLA-E/PIK3R1/ZFP36	15

BP	GO:0050727	regulation of inflammatory response	24/432	414/18903	3.18047248236862e-05	0.000662598433826796	0.000453168317189081	SAA1/BIRC3/S100A9/S100A8/LRRK2/IL20RB/CD47/WNT5A/GRN/IL1B/NFKBIA/IL6ST/APP/HLA-DRB1/NFKBIZ/BIRC2/TNFAIP3/FABP4/SERPINE1/CXCL17/HLA-E/ZFP36/MDK/LGALS1	24
BP	GO:0050777	negative regulation of immune response	15/432	190/18903	3.27583306862891e-05	0.000679404840466759	0.000464662656182671	MMP12/SAMSN1/IFI16/HLA-B/IL20RB/GRN/HLA-A/HLA-DRB1/CD59/SERPING1/LGALS3/CD46/ISG15/TNFAIP3/HLA-E	15
BP	GO:0043616	keratinocyte proliferation	8/432	55/18903	3.31356853602354e-05	0.000680513382874779	0.00046542081719231	HAS2/SDR16C5/AREG/CDH3/KLF9/ZFP36/ZFP36L1/MDK	8
BP	GO:0002064	epithelial cell development	16/432	213/18903	3.31709016703811e-05	0.000680513382874779	0.00046542081719231	CLDN1/EPAS1/WNT5A/CDH2/IL1A/PRDM1/IL1B/IL6ST/FOSL2/B4GALT1/HIF1A/MSN/VIM/RDX/PALLD/AKR1B1	16
BP	GO:0061077	chaperone-mediated protein folding	9/432	71/18903	3.32531944929081e-05	0.000680513382874779	0.00046542081719231	CD74/CLU/HSPA6/HSPA5/HSPH1/FKBP5/DNAJB4/HSPA1A/DNAJB1	9
BP	GO:0006458	'de novo' protein folding	7/432	41/18903	3.56177988024702e-05	0.000725693037274999	0.000496320358921507	CD74/HSPA6/HSPA5/HSPH1/DNAJB4/HSPA1A/DNAJB1	7
BP	GO:0010718	positive regulation of epithelial to mesenchymal transition	8/432	56/18903	3.78954804447226e-05	0.000765356319025512	0.000523447109797249	TGFB2/TGFBR2/BMP2/DAB2/TGFB3/IL1B/MDK/COL1A1	8

BP	GO:2000630	positive regulation of miRNA metabolic process	8/432	56/18903	3.78954804447226e-05	0.000765356319025512	0.000523447109797249	TGFB2/BMP2/HIF1A/EGR1/NR3C1/STAT3/FOS/JUN	8
BP	GO:0031667	response to nutrient levels	26/432	474/18903	3.83870080978861e-05	0.000771912662837927	0.000527931164000676	CYP24A1/IFI16/KYNU/ASS1/TGFBR2/GCLC/CYP1B1/SOD2/LRRK2/DAPL1/STAT1/IL1A/ADRB2/SLC16A1/RUNX2/XBP1/TFRC/HSPA5/LAMP2/MIOS/ZFP36/SLC38A2/FOS/POSTN/WNT2B/COL1A1	26
BP	GO:1903557	positive regulation of tumor necrosis factor superfamily cytokine production	11/432	109/18903	4.02169458002253e-05	0.000805209412666849	0.000550703677966175	CLEC7A/OAS2/LRRK2/WNT5A/IL1A/CLU/IFNGR1/APP/STAT3/HLA-E/PIK3R1	11
BP	GO:0010755	regulation of plasminogen activation	5/432	18/18903	4.0813020830224e-05	0.000813621643705974	0.000556457021755096	PLAUR/RUNX1/SERPINE2/PLAU/SERPINE1	5
BP	GO:0014002	astrocyte development	7/432	42/18903	4.19085515756077e-05	0.000828320730928144	0.000566510110142605	S100A9/S100A8/GRN/IL1B/IFNGR1/APP/VIM	7
BP	GO:0071604	transforming growth factor beta production	7/432	42/18903	4.19085515756077e-05	0.000828320730928144	0.000566510110142605	TGFB2/LUM/ITGAV/CDH3/HIF1A/LAPTM4B/CD46	7
BP	GO:1903034	regulation of response to wounding	14/432	172/18903	4.27092554246085e-05	0.000839539824099022	0.000574183140009687	CLDN1/CLEC7A/PLAUR/GRN/CAV1/SERPINE2/XBP1/SERPING1/PLAU/TNFAIP3/SERPINE1/HBEGF/PROS1/MDK	14

BP	GO:0030336	negative regulation of cell migration	22/432	368/18903	4.28392212945663e-05	0.000839539824099022	0.000574183140009687	DCN/MMP28/BST2/CYP1B1/CD74/DUSP1/IGFBP3/MCC/NEDD9/IGFBP5/SP100/GJA1/TRIB1/TACSTD2/CITED2/GADD45A/SERPINE1/STAT3/TIMP1/SEMA6D/SULF1/SINHCAF	22
BP	GO:0001558	regulation of cell growth	24/432	422/18903	4.31330503987078e-05	0.000841731468751154	0.000575682062838627	TGFB2/S100A9/NRCAM/S100A8/BST2/DAB2/INHBA/CRYAB/RGS4/WNT5A/SLC23A2/OSGIN1/IGFBP3/SERPINE2/CXCL16/IGFBP5/GJA1/HBEGF/MTPN/SGK1/SEMA6D/CRABP2/IGFBP7/HSPA1A	24
BP	GO:0032732	positive regulation of interleukin-1 production	9/432	74/18903	4.64594898025936e-05	0.000902836724105024	0.000617473537624033	SAA1/CLEC7A/IFI16/WNT5A/APP/EGR1/STAT3/S100A13/TMED10	9
BP	GO:0034446	substrate adhesion-dependent cell spreading	11/432	111/18903	4.76187334364527e-05	0.000921492226542234	0.000630232521367718	LAMB3/HAS2/DAB2/ITGAV/LAMB1/FBLN1/NEDD9/GBP1/TACSTD2/PIK3R1/MDK	11
BP	GO:0031214	biomineral tissue development	14/432	174/18903	4.84916522699344e-05	0.000934474548951861	0.00063911146961032	ODAM/MMP13/FAM20A/BMP2/TGFB3/OMD/ODAPH/ADRB2/ANKH/AMTN/HIF1A/ISG15/PTHLH/COL1A1	14
BP	GO:0071364	cellular response to epidermal growth factor stimulus	7/432	43/18903	4.90823867105327e-05	0.000941933769859809	0.000644213024962441	DAB2/ID1/ERRFI1/ZFP36/FOS/ZFP36L1/COL1A1	7
BP	GO:0070663	regulation of leukocyte proliferation	18/432	269/18903	5.05516352789261e-05	0.000963987891699103	0.000659296412985674	TNFRSF21/TGFBR2/BST2/VAV3/CD74/IL20RB/IL1A/PRNP/IL1B/HLA-A/IL6ST/HLA-DRB1/LGALS3/CD46/TNFAIP3/TFRC/HLA-E/PPP3CA	18

BP	GO:0060054	positive regulation of epithelial cell proliferation involved in wound healing	4/432	10/18903	5.06484449044069e-05	0.000963987891699103	0.000659296412985674	ODAM/CLDN1/MMP12/B4GALT1	4
BP	GO:0002474	antigen processing and presentation of peptide antigen via MHC class I	6/432	30/18903	5.1292633696798e-05	0.000964343214828011	0.000659539427723055	B2M/HLA-C/HLA-B/TAPBP/HLA-A/HLA-E	6
BP	GO:0019886	antigen processing and presentation of exogenous peptide antigen via MHC class II	6/432	30/18903	5.1292633696798e-05	0.000964343214828011	0.000659539427723055	B2M/CD74/HLA-DRB1/CTSV/CTSD/HLA-DRA	6
BP	GO:1900101	regulation of endoplasmic reticulum unfolded protein response	6/432	30/18903	5.1292633696798e-05	0.000964343214828011	0.000659539427723055	DNAJB9/XBP1/ATF6/HSPA5/PTPN1/PIK3R1	6
BP	GO:0071248	cellular response to metal ion	15/432	198/18903	5.27540627647824e-05	0.000987803806830441	0.000675584737303095	CLDN1/B2M/LRRK2/CPNE8/DLG2/WNT5A/PRNP/FOSB/APP/FABP4/HSPA5/MT2A/FOS/JUN/MT1E	15
BP	GO:0051092	positive regulation of NF-kappaB transcription factor activity	13/432	154/18903	5.47671987770061e-05	0.00101652653543195	0.000695228959083471	CLEC7A/S100A9/S100A8/RAB7B/WNT5A/CAV1/IL1B/CLU/APP/TFRC/STAT3/MTPN/HSPA1A	13

BP	GO:0032635	interleukin-6 production	14/432	176/18903	5.49473802936187e-05	0.00101652653543195	0.000695228959083471	CLEC7A/HLA-B/RAB7B/CD74/CD47/WNT5A/AFAP1L2/IL1A/IL1B/APP/XBP1/TNFAIP3/CAPN2/STAT3	14
BP	GO:0032675	regulation of interleukin-6 production	14/432	176/18903	5.49473802936187e-05	0.00101652653543195	0.000695228959083471	CLEC7A/HLA-B/RAB7B/CD74/CD47/WNT5A/AFAP1L2/IL1A/IL1B/APP/XBP1/TNFAIP3/CAPN2/STAT3	14
BP	GO:0010574	regulation of vascular endothelial growth factor production	8/432	59/18903	5.5725431400849e-05	0.00102578059785103	0.000701558053267107	CYP1B1/IL1A/IL1B/IL6ST/HIF1A/SULF2/CXCL17/SULF1	8
BP	GO:0001503	ossification	24/432	429/18903	5.58911806829103e-05	0.00102578059785103	0.000701558053267107	MMP13/CYP24A1/TGFB2/BMP2/TGFB3/OMD/RUNX1/AREG/WNT5A/IGFBP3/ADRB2/DHRS3/ANKH/IL6ST/RUNX2/IGFBP5/HIF1A/ISG15/PTHLH/PBX1/PPP3CA/NPPC/MDK/COL1A1	24
BP	GO:1903573	negative regulation of response to endoplasmic reticulum stress	7/432	44/18903	5.72316730316914e-05	0.00104623117696274	0.000715544736676649	LRRK2/CLU/DNAJB9/XBP1/HSPA5/PTPN1/HSPA1A	7
BP	GO:0006809	nitric oxide biosynthetic process	9/432	76/18903	5.75261236795716e-05	0.00104747370873236	0.000716394536498602	CLEC7A/ASS1/CYP1B1/SOD2/CD47/CAV1/IL1B/CLU/HSP90AA1	9
BP	GO:0002824	positive regulation of adaptive immune response	11/432	114/18903	6.0906391315813e-05	0.00110467474445347	0.00075551581	B2M/CLEC7A/RSAD2/HLA-B/IL1B/HLA-A/HLA-DRB1/NFKBIZ/HLA-DRA/TFRC/HLA-E	11

		based on somatic recombination of immune receptors built from immunoglobulin superfamily domains					3845276		
BP	GO:0001659	temperature homeostasis	14/432	178/18903	6.21416050340404e-05	0.00112259296173529	0.0007677705490787	EPAS1/CAV1/IL1A/FABP5/ADRB2/IL1B/G0S2/GJA1/ID1/EGR1/FABP4/STAT3/ARRDC3/DIO2	14
BP	GO:0001916	positive regulation of T cell mediated cytotoxicity	6/432	31/18903	6.23851809857959e-05	0.00112259296173529	0.0007677705490787	B2M/HLA-B/HLA-A/HLA-DRB1/HLA-DRA/HLA-E	6
BP	GO:0014909	smooth muscle cell migration	10/432	95/18903	6.26224830546387e-05	0.00112259296173529	0.0007677705490787	HAS2/CYP1B1/PDGFD/IGFBP3/IGFBP5/TRIB1/PLAU/SERPINE1/SEMA6D/MDK	10
BP	GO:0002711	positive regulation of T cell mediated immunity	8/432	60/18903	6.30311147353237e-05	0.00112555562027364	0.000769796788374915	B2M/RSAD2/HLA-B/IL1B/HLA-A/HLA-DRB1/HLA-DRA/HLA-E	8
BP	GO:0045824	negative regulation of innate immune response	9/432	77/18903	6.38435485981192e-05	0.00113567850871654	0.000776720095292907	MMP12/IFI16/HLA-B/GRN/HLA-A/SERPING1/ISG15/TNFAIP3/HLA-E	9
BP	GO:0032970	regulation of actin filament-based process	23/432	406/18903	6.58860529798485e-05	0.00116752105376168	0.000798498040751945	ODAM/CSF3/BST2/IQGAP2/PLEK/TGFB3/CD47/CGNL1/RGS4/CAV1/IL1A/RND3/CDC42EP3/NEDD9/ID1/TACSTD2/SVIL/CCDC88A/MTPN/RDX/PIK3R1/DSG2/MDK	23

BP	GO:0010712	regulation of collagen metabolic process	7/432	45/18903	6.64550419354764e-05	0.00116864855114669	0.000799269165563765	INHBA/TGFB3/RUNX1/VIM/ERRFI1/CST3/NPPC	7
BP	GO:0043620	regulation of DNA-templated transcription in response to stress	7/432	45/18903	6.64550419354764e-05	0.00116864855114669	0.000799269165563765	EPAS1/HIF1A/CITED2/EGR1/ATF6/HSPA5/JUN	7
BP	GO:0034113	heterotypic cell-cell adhesion	8/432	61/18903	7.11155972169433e-05	0.00122816656914912	0.000839975087406684	NRCAM/CD47/ITGAV/IL1B/IL1RN/WNK1/CD58/DSG2	8
BP	GO:1902893	regulation of miRNA transcription	8/432	61/18903	7.11155972169433e-05	0.00122816656914912	0.000839975087406684	TGFB2/BMP2/HIF1A/EGR1/NR3C1/STAT3/FOS/JUN	8
BP	GO:0097709	connective tissue replacement	5/432	20/18903	7.11162241018348e-05	0.00122816656914912	0.000839975087406684	RUNX1/IL1A/HIF1A/TIMP1/PPP3CA	5
BP	GO:0010001	glial cell differentiation	16/432	227/18903	7.12435150839524e-05	0.00122816656914912	0.000839975087406684	TNFRSF21/S100A9/S100A8/BMP2/ID2/CDH2/GRN/SERPINE2/IL1B/CLU/IFNGR1/IL6ST/APP/VIM/STAT3/MDK	16
BP	GO:0002832	negative regulation of response to biotic stimulus	11/432	116/18903	7.14328231569977e-05	0.00122816656914912	0.000839975087406684	MMP12/IFI16/HLA-B/HTRA1/GRN/HLA-A/SERPING1/TRIB1/ISG15/TNFAIP3/HLA-E	11

BP	GO:0046916	cellular transition metal ion homeostasis	11/432	116/18903	7.14328231569977e-05	0.00122816656914912	0.000839975087406684	S100A9/S100A8/SLC39A14/HEPHL1/PRNP/APP/HIF1A/TFRC/SLC39A6/MT2A/MT1E	11
BP	GO:0043588	skin development	19/432	302/18903	7.22228459943174e-05	0.00123715060268044	0.000846119501804771	CLDN1/TGFB2/INHBA/ZBED2/ITGA6/WNT5A/IL1A/CDH3/IGFBP5/FOSL2/ERRFI1/ARRDC3/PPP3CA/PALLD/ZFP36/TXNIP/ZFP36L1/SOSTDC1/COL1A1	19
BP	GO:0048762	mesenchymal cell differentiation	17/432	252/18903	7.36024893847342e-05	0.00125396072948101	0.000857616385113045	TGFB2/PITX2/HAS2/TGFBR2/BMP2/DAB2/TGFB3/STAT1/WNT5A/CDH2/RDH10/IL1B/HIF1A/CITED2/SEMA6D/MDK/COL1A1	17
BP	GO:0061041	regulation of wound healing	12/432	137/18903	7.37464472256941e-05	0.00125396072948101	0.000857616385113045	CLDN1/CLEC7A/PLAUR/CAV1/SERPINE2/XBP1/SERPING1/PLAU/TNFAIP3/SERPINE1/HBEGF/PROS1	12
BP	GO:0000302	response to reactive oxygen species	15/432	204/18903	7.41171418428443e-05	0.00125564754953537	0.000858770045258327	CYP1B1/SOD2/LRRK2/PDGFD/CRYAB/STAT1/AREG/IL1A/HIF1A/TNFAIP3/CAPN2/FOS/JUN/TXNIP/COL1A1	15
BP	GO:0110148	biomineralization	14/432	181/18903	7.44723643475294e-05	0.00125706089455228	0.000859736668706592	ODAM/MMP13/FAM20A/BMP2/TGFB3/OMD/ODAPH/ADRB2/ANKH/AMTN/HIF1A/ISG15/PTHLH/COL1A1	14
BP	GO:0010837	regulation of keratinocyte proliferation	7/432	46/18903	7.68575456068506e-05	0.0012832712939772	0.000877662643163922	HAS2/AREG/CDH3/KLF9/ZFP36/ZFP36L1/MDK	7

BP	GO:0060443	mammary gland morphogenesis	7/432	46/18903	7.68575456068506e-05	0.0012832712939772	0.000877662643163922	TGFBR2/AREG/WNT5A/CAV1/IGFBP5/NR3C1/SOSTDC1	7
BP	GO:2001239	regulation of extrinsic apoptotic signaling pathway in absence of ligand	7/432	46/18903	7.68575456068506e-05	0.0012832712939772	0.000877662643163922	INHBA/SRPX/IL1A/IL1B/IFI6/MCL1/HSPA1A	7
BP	GO:0002822	regulation of adaptive immune response based on somatic recombination of immune receptors built from immunoglobulin superfamily domains	14/432	182/18903	7.90311025290078e-05	0.00130711034198932	0.000893966788660692	B2M/CLEC7A/RSAD2/HLA-B/IL20RB/IL1B/HLA-A/HLA-DRB1/NFKBIZ/CD46/TNFAIP3/HLA-DRA/TFRC/HLA-E	14
BP	GO:0042129	regulation of T cell proliferation	14/432	182/18903	7.90311025290078e-05	0.00130711034198932	0.000893966788660692	TNFRSF21/TGFBR2/IL20RB/IL1A/PRNP/IL1B/HLA-A/IL6ST/HLA-DRB1/LGALS3/CD46/TFRC/HLA-E/PPP3CA	14
BP	GO:0030879	mammary gland development	12/432	138/18903	7.91331666501644e-05	0.00130711034198932	0.000893966788660692	TGFBR2/OAS2/TGFB3/ID2/AREG/WNT5A/CAV1/IGFBP5/HIF1A/XBP1/NR3C1/SOSTDC1	12

BP	GO:0045428	regulation of nitric oxide biosynthetic process	8/432	62/18903	8.00425609142143e-05	0.00131275476676681	0.0008978271576977	CLEC7A/ASS1/SOD2/CD47/CAV1/IL1B/CLU/HSP90AA1	8
BP	GO:0061614	miRNA transcription	8/432	62/18903	8.00425609142143e-05	0.00131275476676681	0.0008978271576977	TGFB2/BMP2/HIF1A/EGR1/NR3C1/STAT3/FOS/JUN	8
BP	GO:0071674	mononuclear cell migration	15/432	206/18903	8.275270573097e-05	0.00135240729330649	0.000924946552804779	SAA1/PDGFD/CD47/WNT5A/DUSP1/CXCL16/APP/NEDD9/CCL20/LGALS3/WNK1/MSN/SERPINE1/CXCL17/MDK	15
BP	GO:0002718	regulation of cytokine production involved in immune response	11/432	118/18903	8.34801578187955e-05	0.00135949200673215	0.000929791972740847	B2M/CLEC7A/TGFB2/BST2/RSAD2/TGFB3/CD74/WNT5A/IL1B/HLA-A/HLA-E	11
BP	GO:0070849	response to epidermal growth factor	7/432	47/18903	8.85508048644941e-05	0.00143700867543258	0.000982807640324303	DAB2/ID1/ERRFI1/ZFP36/FOS/ZFP36L1/COL1A1	7
BP	GO:0010573	vascular endothelial growth factor production	8/432	63/18903	8.98792219731073e-05	0.00145346643925042	0.000994063533416221	CYP1B1/IL1A/IL1B/IL6ST/HIF1A/SULF2/CXCL17/SULF1	8
BP	GO:0001655	urogenital system development	21/432	360/18903	9.02557625840146e-05	0.00145447004164135	0.000994749923216446	TGFB2/ASS1/HAS2/BMP2/LRRK2/PDGFD/BASP1/TIPARP/STAT1/WNT5A/MME/RDH10/PRDM1/TACSTD2/EGR1/SULF2/PBX1/PPP3CA/AKR1B1/SULF1/WNT2B	21

BP	GO:1901522	positive regulation of transcription from RNA polymerase II promoter involved in cellular response to chemical stimulus	5/432	21/18903	9.15962095105835e-05	0.00147094607286961	0.00100601830986587	BMP2/RUNX2/HIF1A/XBP1/JUN	5
BP	GO:0034620	cellular response to unfolded protein	10/432	100/18903	9.69486016760913e-05	0.0015476389728374	0.0010584705805693	OPTN/DNAJB9/HSPA6/XBP1/ATF6/HSPA5/PTPN1/PIK3R1/TM7SF3/HSPA1A	10
BP	GO:0002367	cytokine production involved in immune response	11/432	120/18903	9.72241034222856e-05	0.0015476389728374	0.0010584705805693	B2M/CLEC7A/TGFB2/BST2/RSAD2/TGFB3/CD74/WNT5A/IL1B/HLA-A/HLA-E	11
BP	GO:0070555	response to interleukin-1	12/432	141/18903	9.73757710477151e-05	0.0015476389728374	0.0010584705805693	HAS2/GCLC/CXCL8/CD47/GBP3/IL1B/IL1RN/APP/CCL20/HIF1A/GBP1/EGR1	12
BP	GO:0006953	acute-phase response	7/432	48/18903	0.000101653146209875	0.00160996761808812	0.00110109876355159	SAA1/SAA2/ASS1/IL1A/PLSCR1/IL1B/TFRC	7
BP	GO:0042063	gliogenesis	19/432	310/18903	0.000101993624237799	0.00160996761808812	0.00110109876355159	TNFRSF21/TGFB2/S100A9/S100A8/BMP2/ID2/AREG/LAMB1/CDH2/GRN/SERPINE2/IL1B/CLU/IFNGR1/IL6ST/APP/VIM/STAT3/MDK	19
BP	GO:0051100	negative regulation of binding	13/432	164/18903	0.00010437583	0.00164196678705099	0.00112298382818509	B2M/IFI16/DAB2/IFIT1/LRRK2/CAV1/ADRB2/NFKBIA/SLPI/SP100/ID1/PPP3CA/JUN	13

					4679566				
BP	GO:0046209	nitric oxide metabolic process	9/432	82/18903	0.000104892802260461	0.00164450579815129	0.00112472032397033	CLEC7A/ASS1/CYP1B1/SOD2/CD47/CAV1/IL1B/CLU/HSP90AA1	9
BP	GO:2001243	negative regulation of intrinsic apoptotic signaling pathway	10/432	101/18903	0.00010544703266312	0.00164535507922465	0.00112530116940428	SOD2/LRRK2/PLAUR/CD74/CLU/HIF1A/XBP1/MCL1/PTPN1/HSPA1A	10
BP	GO:0002700	regulation of production of molecular mediator of immune response	14/432	187/18903	0.00010565847751994	0.00164535507922465	0.00112530116940428	B2M/CLEC7A/TGFB2/BST2/RSAD2/TGFB3/CD74/WNT5A/IL1B/HLA-A/DNAJB9/XBP1/TFRC/HLA-E	14
BP	GO:0002495	antigen processing and presentation of peptide antigen via MHC class II	6/432	34/18903	0.000107538556168786	0.00166343084374794	0.00113766365529731	B2M/CD74/HLA-DRB1/CTSV/CTSD/HLA-DRA	6
BP	GO:0045648	positive regulation of erythrocyte differentiation	6/432	34/18903	0.000107538556168786	0.00166343084374794	0.00113766365529731	INHBA/STAT1/HIF1A/ISG15/STAT3/HSPA1A	6
BP	GO:0001933	negative regulation of protein phosphorylation	20/432	338/18903	0.000108997197679636	0.00168037346422772	0.00114925115448178	SAMSN1/BMP2/LRRK2/PMEPA1/DUSP1/FBLN1/CAV1/PRNP/IGFBP3/IL1B/TRIB1/PTPN13/TNFAIP3/GADD45A/FABP4/ERRFI1/DNAJC3/PTPN1/GADD45B/JUN	20

BP	GO:0032956	regulation of actin cytoskeleton organization	21/432	365/18903	0.000109517902231392	0.00168279168711026	0.00115090504006061	ODAM/CSF3/BST2/IQGAP2/PLEK/TGFB3/CD47/CGNL1/RGS4/IL1A/RND3/CDC42EP3/NEDD9/ID1/TACSTD2/SVIL/CCDC88A/MTPN/RDX/PIK3R1/MDK	21
BP	GO:0032613	interleukin-10 production	8/432	65/18903	0.000112568682836138	0.00171132962674694	0.00117042287985765	TNFRSF21/CLEC7A/IL20RB/CD47/HLA-DRB1/CD46/ISG15/STAT3	8
BP	GO:0032653	regulation of interleukin-10 production	8/432	65/18903	0.000112568682836138	0.00171132962674694	0.00117042287985765	TNFRSF21/CLEC7A/IL20RB/CD47/HLA-DRB1/CD46/ISG15/STAT3	8
BP	GO:0080164	regulation of nitric oxide metabolic process	8/432	65/18903	0.000112568682836138	0.00171132962674694	0.00117042287985765	CLEC7A/ASS1/SOD2/CD47/CAV1/IL1B/CLU/HSP90AA1	8
BP	GO:0002761	regulation of myeloid leukocyte differentiation	11/432	122/18903	0.000112855251061149	0.00171132962674694	0.00117042287985765	INHBA/CD74/RUNX1/ID2/NEDD9/HLA-DRB1/TRIB1/PPP3CA/PIK3R1/FOS/ZFP36L1	11
BP	GO:0042098	T cell proliferation	15/432	212/18903	0.000114153630900592	0.00172536125135699	0.00118001947318981	TNFRSF21/TGFBR2/IL20RB/IL1A/PRNP/IL1B/HLA-A/IL6ST/HLA-DRB1/LGALS3/CD46/MSN/TFRC/HLA-E/PPP3CA	15
BP	GO:0031341	regulation of cell killing	10/432	102/18903	0.000114567429958795	0.00172597512560074	0.00118043931776506	CFH/B2M/CLEC7A/CXCL6/HLA-B/HLA-A/HLA-DRB1/CD59/HLA-DRA/HLA-E	10

BP	GO:2001057	reactive nitrogen species metabolic process	9/432	83/18903	0.000115311760230844	0.00173150824350429	0.00118422356113352	CLEC7A/ASS1/CYP1B1/SOD2/CD47/CAV1/IL1B/CLU/HSP90AA1	9
BP	GO:0060485	mesenchyme development	19/432	313/18903	0.000115683469674125	0.00173150824350429	0.00118422356113352	TGFB2/PITX2/HAS2/TGFBR2/BMP2/DAB2/TGFB3/BASP1/STAT1/WNT5A/CDH2/RDH10/IL1B/HIF1A/CITED2/SEMA6D/ZFP36L1/MDK/COL1A1	19
BP	GO:0030449	regulation of complement activation	5/432	22/18903	0.00011632290454254	0.00173546268873951	0.00118692810916921	CFH/IL1B/CD59/SERPING1/CD46	5
BP	GO:0034599	cellular response to oxidative stress	18/432	288/18903	0.000120905368996481	0.00179803000517274	0.00122971952558614	SLC7A11/EPAS1/NCOA7/CYP1B1/SOD2/LRRK2/PDGFD/VNN1/GJB2/NR4A2/HIF1A/PNPLA8/ARL6IP5/TNFAIP3/MCL1/FOS/JUN/HSPA1A	18
BP	GO:0031589	cell-substrate adhesion	21/432	369/18903	0.000127465743621977	0.00188951623157578	0.00129228933732807	MMP12/LAMB3/HAS2/DAB2/ITGA6/RIN2/ITGAV/LAMB1/FBLN1/COL17A1/NEDD9/GBP1/ID1/TACSTD2/PLAU/SERPINE1/FAT2/CD63/PIK3R1/MDK/COL1A1	21
BP	GO:0050670	regulation of lymphocyte proliferation	16/432	239/18903	0.000130228147768082	0.00191816937397255	0.00131188596729161	TNFRSF21/TGFBR2/VAV3/CD74/IL20RB/IL1A/PRNP/IL1B/HLA-A/IL6ST/HLA-DRB1/LGALS3/CD46/TFRC/HLA-E/PPP3CA	16
BP	GO:0051403	stress-activated MAPK cascade	16/432	239/18903	0.000130228147768082	0.00191816937397255	0.00131188596729161	TGFB2/BMP2/LRRK2/CRYAB/WNT5A/DUSP1/IL1A/IL1B/APP/ARL6IP5/TRIB1/TPD52L1/GADD45A/GADD45B/ZFP36/ZFP36L1	16

BP	GO:0090092	regulation of transmembrane receptor protein serine/threonine kinase signaling pathway	18/432	290/18903	0.000131864342327252	0.00193610343893188	0.00132415154116422	TGFB2/TGFBR2/BMP2/DAB2/INHBA/TGFB3/HTRA1/PMEPA1/WNT5A/CAV1/SKIL/CITED2/HSPA5/NREP/SULF1/SINHCAF/HSPA1A/SOSTDC1	18
BP	GO:0032964	collagen biosynthetic process	7/432	50/18903	0.000132592653306618	0.00194063614412377	0.00132725157623714	INHBA/TGFB3/RUNX1/VIM/ERRFI1/NPPC/COL1A1	7
BP	GO:0031334	positive regulation of protein-containing complex assembly	14/432	192/18903	0.000139752348861966	0.00203319026707558	0.00139055175022751	CSF3/CLEC7A/MMP1/PLEK/CDC42EP3/ABCA1/MPP7/CLU/LGALS3/MSN/ISG15/TFRC/HSP90AA1/HSPA1A	14
BP	GO:0031638	zymogen activation	8/432	67/18903	0.000139795568633521	0.00203319026707558	0.00139055175022751	IFI16/C1R/PLAUR/RUNX1/SERPINE2/PLAU/ASPH/SERPINE1	8
BP	GO:0045862	positive regulation of proteolysis	21/432	372/18903	0.000142588198058262	0.00206730537937135	0.00141388396358712	TNFSF10/CLEC7A/IFI16/S100A9/S100A8/GCLC/DAB2/LRRK2/FBLN1/GRN/CAV1/IL1B/CLU/APP/ARL6IP5/TRIB1/CTSD/ASPH/STAT3/UACA/HSPA1A	21
BP	GO:0017015	regulation of transforming growth factor beta receptor signaling pathway	12/432	147/18903	0.000144909639156143	0.00209439712842864	0.00143241271600069	BMP2/DAB2/TGFB3/HTRA1/PMEPA1/CAV1/SKIL/CITED2/HSPA5/NREP/SINHCAF/HSPA1A	12

BP	GO:0002504	antigen processing and presentation of peptide or polysaccharide antigen via MHC class II	6/432	36/18903	0.000149849866390066	0.00215905181325251	0.00147663173799688	B2M/CD74/HLA-DRB1/CTSV/CTSD/HLA-DRA	6
BP	GO:0002088	lens development in camera-type eye	9/432	86/18903	0.000151901110990204	0.0021818094358065	0.00149219626847193	SLC7A11/TGFBR2/CRYAB/WNT5A/SKIL/CITED2/VIM/MAF/WNT2B	9
BP	GO:0038034	signal transduction in absence of ligand	8/432	68/18903	0.000155318470724721	0.00219752107649405	0.00150294186853249	INHBA/SRPX/ITGAV/IL1A/IL1B/IFI6/MCL1/HSPA1A	8
BP	GO:0042446	hormone biosynthetic process	8/432	68/18903	0.000155318470724721	0.00219752107649405	0.00150294186853249	BMP2/DAB2/ATP1A1/HIF1A/EGR1/NR3C1/AKR1B1/DIO2	8
BP	GO:0097192	extrinsic apoptotic signaling pathway in absence of ligand	8/432	68/18903	0.000155318470724721	0.00219752107649405	0.00150294186853249	INHBA/SRPX/ITGAV/IL1A/IL1B/IFI6/MCL1/HSPA1A	8
BP	GO:0043523	regulation of neuron apoptotic process	15/432	218/18903	0.000155466128918997	0.00219752107649405	0.00150294186853249	TGFB2/GCLC/SOD2/TGFB3/GRN/PRNP/NR4A2/IL6ST/HIF1A/SRPK2/NR3C1/MCL1/CHL1/JUN/MDK	15
BP	GO:1901215	negative regulation of neuron death	15/432	218/18903	0.00015546612	0.00219752107649405	0.00150294186853249	CSF3/SLC7A11/GCLC/NCOA7/SOD2/LRRK2/TGFB3/GRN/SLC23A2/NR4A2/IL6ST/HIF1A/CHL1/JUN/MDK	15

					8918997				
BP	GO: 0010952	positive regulation of peptidase activity	14/432	194/18903	0.000155845819046497	0.00219752107649405	0.00150294186853249	TNFSF10/CLEC7A/IFI16/S100A9/S100A8/FBLN1/GRN/CAV1/APP/ARL6IP5/CTSD/ASPH/STAT3/UACA	14
BP	GO: 0032944	regulation of mononuclear cell proliferation	16/432	243/18903	0.000157685889910159	0.00221670893870665	0.00151606494698457	TNFRSF21/TGFBR2/VAV3/CD74/IL20RB/IL1A/PRNP/IL1B/HLA-A/IL6ST/HLA-DRB1/LGALS3/CD46/TFRC/HLA-E/PPP3CA	16
BP	GO: 0070661	leukocyte proliferation	20/432	348/18903	0.000161179560017272	0.00225895595478753	0.0015449587810268	TNFRSF21/TGFBR2/BST2/VAV3/CD74/IL20RB/IL1A/PRNP/IL1B/HLA-A/CLU/IL6ST/HLA-DRB1/LGALS3/CD46/MSN/TNFAIP3/TFRC/HLA-E/PPP3CA	20
BP	GO: 0070424	regulation of nucleotide-binding oligomerization domain containing signaling pathway	4/432	13/18903	0.000163288363443921	0.00227472494255462	0.00155574360224788	BIRC3/BIRC2/TNFAIP3/HSPA1A	4
BP	GO: 1900103	positive regulation of endoplasmic reticulum unfolded protein response	4/432	13/18903	0.000163288363443921	0.00227472494255462	0.00155574360224788	XBP1/ATF6/PTPN1/PIK3R1	4
BP	GO: 1903844	regulation of cellular response to transforming	12/432	150/18903	0.000175333334540707	0.00242933487267846	0.00166148535815619	BMP2/DAB2/TGFB3/HTRA1/PMEPA1/CAV1/SKIL/CITED2/HSPA5/NREP/SINHCAF/HSPA1A	12

		growth factor beta stimulus							
BP	GO:0060416	response to growth hormone	6/432	37/18903	0.000175437372426942	0.00242933487267846	0.00166148535815619	ASS1/IGFBP5/JAK1/STAT3/PTPN1/PIK3R1	6
BP	GO:0002443	leukocyte mediated immunity	24/432	463/18903	0.000179500098715671	0.0024745636736341	0.00169241851249399	B2M/CLEC7A/C1S/BST2/CXCL6/C1R/RSAD2/HLA-B/CD74/IL20RB/IL1B/HLA-A/CLU/HLA-DRB1/SERPING1/CD46/HLA-DRA/IGHG3/IGKC/TFRC/IGHG4/S100A13/HLA-E/KMT2E	24
BP	GO:0001667	ameboidal-type cell migration	25/432	492/18903	0.000179773706884553	0.0024745636736341	0.00169241851249399	DCN/TGFB2/PITX2/HAS2/TGFBR2/CYP1B1/RIN2/WNT5A/CDH2/GRN/MCC/HIF1A/SP100/GJA1/ID1/TACSTD2/ZFAND5/GADD45A/FAT2/HBEGF/TIMP1/SEMA6D/JUN/S100A2/SPARC	25
BP	GO:0050866	negative regulation of cell activation	15/432	221/18903	0.000180602033379881	0.00247858873703843	0.00169517135813382	TNFRSF21/SAMSN1/GCLC/INHBA/CD74/IL20RB/RUNX1/ID2/GRN/PRNP/SERPINE2/HLA-DRB1/LGALS3/TNFAIP3/MDK	15
BP	GO:0031098	stress-activated protein kinase signaling cascade	16/432	246/18903	0.000181466069750945	0.0024830786171542	0.00169824210402239	TGFB2/BMP2/LRRK2/CRYAB/WNT5A/DUSP1/IL1A/IL1B/APP/ARL6IP5/TRIB1/TPD52L1/GADD45A/GADD45B/ZFP36/ZFP36L1	16
BP	GO:0014812	muscle cell migration	10/432	108/18903	0.000184481320481188	0.00250948855066322	0.00171630454503396	HAS2/CYP1B1/PDGFD/IGFBP3/IGFBP5/TRIB1/PLAU/SERPINE1/SEMA6D/MDK	10
BP	GO:0044106	cellular amine metabolic process	10/432	108/18903	0.00018448132	0.00250948855066322	0.00171630454503396	SLC7A11/TGFB2/KYNU/NNMT/TDO2/EPAS1/SAT1/NR4A2/MAOA/MTPN	10

					0481188				
BP	GO:0009409	response to cold	7/432	53/18903	0.000192926792265514	0.00261667570154839	0.00178961262774462	SOD2/ADRB2/NFKBIA/DNAJC3/FOS/HSP90AA1/DIO2	7
BP	GO:0002709	regulation of T cell mediated immunity	9/432	89/18903	0.00019770383770361	0.00266272408418136	0.00182110631532631	B2M/RSAD2/HLA-B/IL20RB/IL1B/HLA-A/HLA-DRB1/HLA-DRA/HLA-E	9
BP	GO:0032612	interleukin-1 production	11/432	130/18903	0.000198624823576772	0.00266272408418136	0.00182110631532631	SAA1/CLEC7A/IFI16/WNT5A/APP/EGR1/TNFAIP3/STAT3/ERRFI1/S100A13/TMED10	11
BP	GO:0032652	regulation of interleukin-1 production	11/432	130/18903	0.000198624823576772	0.00266272408418136	0.00182110631532631	SAA1/CLEC7A/IFI16/WNT5A/APP/EGR1/TNFAIP3/STAT3/ERRFI1/S100A13/TMED10	11
BP	GO:0042542	response to hydrogen peroxide	11/432	130/18903	0.000198624823576772	0.00266272408418136	0.00182110631532631	CYP1B1/SOD2/LRRK2/PDGFD/CRYAB/STAT1/AREG/TNFAIP3/CAPN2/TXNIP/COL1A1	11
BP	GO:0050819	negative regulation of coagulation	7/432	54/18903	0.000217358387055037	0.00289706783899004	0.00198138010951126	PLAUR/SERPINE2/SERPING1/PLAU/SERPINE1/ANXA5/PROS1	7
BP	GO:0061900	glial cell activation	7/432	54/18903	0.000217358387055037	0.00289706783899004	0.00198138010951126	LRRK2/GRN/IL1B/CLU/IFNGR1/APP/JUN	7

BP	GO:0070423	nucleotide-binding oligomerization domain containing signaling pathway	5/432	25/18903	0.000221797785972334	0.00294774356356909	0.00201603855670558	BIRC3/NFKBIA/BIRC2/TNFAIP3/HSPA1A	5
BP	GO:0044849	estrous cycle	4/432	14/18903	0.000224489513304219	0.0029664685686629	0.00202884507512535	HAS2/CYP1B1/EGR1/MDK	4
BP	GO:0048711	positive regulation of astrocyte differentiation	4/432	14/18903	0.000224489513304219	0.0029664685686629	0.00202884507512535	BMP2/ID2/SERPINE2/IL6ST	4
BP	GO:0006956	complement activation	11/432	132/18903	0.000227121836106962	0.00299270225639516	0.00204678697706229	CFH/C1S/C1R/IL1B/CLU/CD59/SERPING1/CD46/IGHG3/IGKC/IGHG4	11
BP	GO:0018958	phenol-containing compound metabolic process	10/432	111/18903	0.000231111347934236	0.00302801695239615	0.00207093961694462	SLC7A11/TGFB2/EPAS1/WNT5A/CDH3/CTSB/NR4A2/MAOA/MTPN/DIO2	10
BP	GO:0071347	cellular response to interleukin-1	10/432	111/18903	0.000231111347934236	0.00302801695239615	0.00207093961694462	HAS2/CXCL8/CD47/GBP3/IL1B/IL1RN/CCL20/HIF1A/GBP1/EGR1	10
BP	GO:0042698	ovulation cycle	8/432	72/18903	0.000232290106075377	0.0030348636740074	0.00207562226808358	TGFB2/HAS2/CYP1B1/INHBA/TGFB3/EGR1/HSPA5/MDK	8

BP	GO:0042475	odontogenesis of dentin-containing tooth	9/432	91/18903	0.000234203096470654	0.00305123752444162	0.00208682077062806	ODAM/FAM20A/BMP2/HTRA1/ODAPH/AMTN/RUNX2/SERPINE1/SOSTDC1	9
BP	GO:0022408	negative regulation of cell-cell adhesion	14/432	202/18903	0.000237297239071217	0.00307422893754728	0.00210254519654727	TNFRSF21/ASS1/BMP2/CD74/IL20RB/RUNX1/PRNP/SERPINE2/IL1RN/HLA-DRB1/LGALS3/WNK1/RDX/MDK	14
BP	GO:0098739	import across plasma membrane	14/432	202/18903	0.000237297239071217	0.00307422893754728	0.00210254519654727	SLC1A3/SLC7A11/SLC39A14/RGS4/PRNP/KCNJ15/SLC5A1/ATP1A1/WNK1/ARL6IP5/PPP3CA/SLC39A6/ATP1B1/SLC38A2	14
BP	GO:1903320	regulation of protein modification by small protein conjugation or removal	16/432	252/18903	0.000238531342285 24	0.00308158507840568	0.0021075762527702	BIRC3/EPAS1/GCLC/LRRK2/CAV1/NDFIP2/N4BP1/BIRC2/HIF1A/EGR1/ISG15/TNFAIP3/HSPA5/ARRDC3/HSP90AA1/HSPA1A	16
BP	GO:0002702	positive regulation of production of molecular mediator of immune response	11/432	133/18903	0.000242619812726307	0.00312567307481663	0.00213772918118327	B2M/CLEC7A/RSAD2/CD74/WNT5A/IL1B/HLA-A/DNAJB9/XBP1/TFRC/HLA-E	11
BP	GO:0097305	response to alcohol	16/432	253/18903	0.000249417432288503	0.00320432117870646	0.00219151866674547	CLDN1/CSF3/S100A8/TGFBR2/CYP1B1/SOD2/INHBA/RGS4/SLC23A2/FOSB/BIRC2/KLF9/SGK1/FOS/IGFBP7/NPPC	16

BP	GO:0001654	eye development	21/432	388/18903	0.000253209827417494	0.00324403172245404	0.0022186777413313	ANGPTL7/SLC7A11/TGFB2/PITX2/TGFBR2/CYP1B1/INHBA/CRYAB/WNT5A/RDH10/PRDM1/HIF1A/SKIL/CITED2/VIM/MAF/STAT3/ATF6/PBX1/JUN/WNT2B	21
BP	GO:0046635	positive regulation of alpha-beta T cell activation	8/432	73/18903	0.000255754960195277	0.00326758754393137	0.00223478818082817	TGFBR2/RUNX1/HLA-A/HLA-DRB1/NFKBIZ/HLA-DRA/HSPH1/HLA-E	8
BP	GO:0071241	cellular response to inorganic substance	15/432	229/18903	0.000265553667921725	0.00338343171938836	0.00231401702799705	CLDN1/B2M/LRRK2/CPNE8/DLG2/WNT5A/PRNP/FOSB/APP/FABP4/HSPA5/MT2A/FOS/JUN/MT1E	15
BP	GO:0002456	T cell mediated immunity	10/432	113/18903	0.000267389496744362	0.00339746269901834	0.00232361318021055	B2M/RSAD2/HLA-B/IL20RB/IL1B/HLA-A/HLA-DRB1/CD46/HLA-DRA/HLA-E	10
BP	GO:0035872	nucleotide-binding domain, leucine rich repeat containing receptor signaling pathway	5/432	26/18903	0.000269490424039418	0.00341477592104742	0.00233545414344182	BIRC3/NFKBIA/BIRC2/TNFAIP3/HSPA1A	5
BP	GO:0006882	cellular zinc ion homeostasis	6/432	40/18903	0.000273392117950653	0.00344533663629911	0.00235635541213743	S100A9/S100A8/SLC39A14/SLC39A6/MT2A/MT1E	6
BP	GO:0032965	regulation of collagen	6/432	40/18903	0.00027339211	0.00344533663629911	0.00235635541213743	INHBA/TGFB3/RUNX1/VIM/ERRFI1/NPPC	6

		biosynthetic process			7950653				
BP	GO:0071214	cellular response to abiotic stimulus	19/432	335/18903	0.000276007487880184	0.00345385077779222	0.00236217845513869	IFI16/GCLC/MMP1/CRYAB/MME/IL1B/N4BP1/LRRC8D/EGR1/GADD45A/ERRFI1/HSPA5/TIMP1/MTPN/PIK3R1/AKR1B1/SLC38A2/ZFP36L1/COL1A1	19
BP	GO:0104004	cellular response to environmental stimulus	19/432	335/18903	0.000276007487880184	0.00345385077779222	0.00236217845513869	IFI16/GCLC/MMP1/CRYAB/MME/IL1B/N4BP1/LRRC8D/EGR1/GADD45A/ERRFI1/HSPA5/TIMP1/MTPN/PIK3R1/AKR1B1/SLC38A2/ZFP36L1/COL1A1	19
BP	GO:2001235	positive regulation of apoptotic signaling pathway	11/432	135/18903	0.000276308062223377	0.00345385077779222	0.00236217845513869	TNFSF10/S100A9/S100A8/INHBA/VNN1/SRPX/CAV1/G0S2/SKIL/TPD52L1/MCL1	11
BP	GO:0048662	negative regulation of smooth muscle cell proliferation	8/432	74/18903	0.000281124108121663	0.00349515860231907	0.00239042995162874	BMP2/SOD2/OGN/TGFB3/IGFBP3/IGFBP5/TRIB1/TNFAIP3	8
BP	GO:2000628	regulation of miRNA metabolic process	8/432	74/18903	0.000281124108121663	0.00349515860231907	0.00239042995162874	TGFB2/BMP2/HIF1A/EGR1/NR3C1/STAT3/FOS/JUN	8
BP	GO:0150063	visual system development	21/432	392/18903	0.000290559250192826	0.00360277890654643	0.00246403427918567	ANGPTL7/SLC7A11/TGFB2/PITX2/TGFBR2/CYP1B1/INHBA/CRYAB/WNT5A/RDH10/PRDM1/HIF1A/SKIL/CITED2/VIM/MAF/STAT3/ATF6/PBX1/JUN/WNT2B	21
BP	GO:0010838	positive regulation of keratinocyte proliferation	4/432	15/18903	0.00030061694	0.00370760900944551	0.00253573031542162	HAS2/AREG/CDH3/MDK	4

					6711798				
BP	GO:0032352	positive regulation of hormone metabolic process	4/432	15/18903	0.000300616946711798	0.00370760900944551	0.00253573031542162	DAB2/RDH10/HIF1A/EGR1	4
BP	GO:0060537	muscle tissue development	22/432	422/18903	0.000305917853219278	0.00375358484819202	0.00256717438835096	TGFB2/TGFBR2/BMP2/TIPARP/RUNX1/ID2/RGS4/WNT5A/CAV1/SORBS2/IGFBP5/GJA1/CITED2/ZFAND5/EGR1/SVIL/PPP3CA/MTPN/DSG2/FOXN2/FOS/CSRP2	22
BP	GO:0038066	p38MAPK cascade	7/432	57/18903	0.00030596788924722	0.00375358484819202	0.00256717438835096	BMP2/DUSP1/IL1B/GADD45A/GADD45B/ZFP36/ZFP36L1	7
BP	GO:0050818	regulation of coagulation	8/432	75/18903	0.000308512804663358	0.00377479291420115	0.00258167913676801	PLAUR/CAV1/SERPINE2/SERPING1/PLAU/SERPINE1/ANXA5/PROS1	8
BP	GO:0001914	regulation of T cell mediated cytotoxicity	6/432	41/18903	0.000314153429804627	0.00382357792854316	0.00261504447801359	B2M/HLA-B/HLA-A/HLA-DRB1/HLA-DRA/HLA-E	6
BP	GO:0046688	response to copper ion	6/432	41/18903	0.000314153429804627	0.00382357792854316	0.00261504447801359	IL1A/PRNP/APP/TFRC/MT2A/MT1E	6
BP	GO:0051017	actin filament bundle assembly	12/432	160/18903	0.000319213956444541	0.00387008330938071	0.00264685071856365	PLEK/TGFB3/CD47/CGNL1/PLS1/MYO1B/NEDD9/ID1/TACSTD2/CCDC88A/RDX/PIK3R1	12

BP	GO:0060348	bone development	15/432	233/18903	0.000319647961985607	0.00387008330938071	0.00264685071856365	MMP13/PITX2/HAS2/TGFBR2/BMP2/OGN/TGFB3/DHRS3/RUNX2/GJA1/CITED2/SULF2/SULF1/NPPC/COL1A1	15
BP	GO:0034109	homotypic cell-cell adhesion	9/432	95/18903	0.000324077722551466	0.00387901845059049	0.00265296169423031	SLC7A11/PLAUR/PLEK/SERPINE2/IL6ST/JAK1/MYL9/RDX/DSG2	9
BP	GO:0030325	adrenal gland development	5/432	27/18903	0.000324579489811572	0.00387901845059049	0.00265296169423031	CYP1B1/CITED2/NR3C1/PBX1/MDK	5
BP	GO:0031639	plasminogen activation	5/432	27/18903	0.000324579489811572	0.00387901845059049	0.00265296169423031	PLAUR/RUNX1/SERPINE2/PLAU/SERPINE1	5
BP	GO:0042730	fibrinolysis	5/432	27/18903	0.000324579489811572	0.00387901845059049	0.00265296169423031	PLAUR/SERPING1/PLAU/SERPINE1/PROS1	5
BP	GO:0071549	cellular response to dexamethasone stimulus	5/432	27/18903	0.000324579489811572	0.00387901845059049	0.00265296169423031	ASS1/GJB2/FBXO32/NR3C1/ERRFI1	5
BP	GO:0009743	response to carbohydrate	15/432	234/18903	0.000334566111657506	0.00397790113294721	0.00272059013473829	CLEC7A/TGFBR2/GCLC/SOD2/SLC39A14/IL1A/IL1B/HLA-DRB1/RUNX2/HIF1A/XBP1/EGR1/PPP3CA/TXNIP/ZFP36L1	15

BP	GO:0030100	regulation of endocytosis	14/432	209/18903	0.000336293911996186	0.00397790113294721	0.00272059013473829	B2M/DAB2/LRRK2/RAB31/ITGAV/WNT5A/CAV1/CLU/LGALS3/SERPINE1/CD63/PPP3CA/PTPN1/STON2	14
BP	GO:0045732	positive regulation of protein catabolic process	14/432	209/18903	0.000336293911996186	0.00397790113294721	0.00272059013473829	GCLC/DAB2/LRRK2/TIPARP/WNT5A/CAV1/IL1B/CLU/TRIB1/MSN/TNFAIP3/RDX/HSP90AA1/HSPA1A	14
BP	GO:0071383	cellular response to steroid hormone stimulus	14/432	209/18903	0.000336293911996186	0.00397790113294721	0.00272059013473829	ASS1/CYP1B1/DAB2/PMEPA1/GJB2/ATP1A1/FBXO32/NR3C1/ERRFI1/KLF9/SGK1/ZFP36/ZFP36L1/HSPA1A	14
BP	GO:0001706	endoderm formation	7/432	58/18903	0.000341219007921359	0.00402586201948032	0.00275339183352225	LAMA3/LAMB3/INHBA/ITGAV/LAMB1/DUSP1/COL12A1	7
BP	GO:0045807	positive regulation of endocytosis	9/432	96/18903	0.000350516515534731	0.00411456569631506	0.00281405858718105	B2M/DAB2/LRRK2/RAB31/WNT5A/CLU/SERPINE1/CD63/PPP3CA	9
BP	GO:0061097	regulation of protein tyrosine kinase activity	9/432	96/18903	0.000350516515534731	0.00411456569631506	0.00281405858718105	AREG/AFAP1L2/CAV1/PRNP/APP/NEDD9/ERRFI1/HBEGF/PTPN1	9
BP	GO:0045619	regulation of lymphocyte differentiation	14/432	210/18903	0.000352979455082452	0.00411771125830034	0.00281620991890584	TGFBR2/INHBA/CD74/VNN1/RUNX1/ID2/PRDM1/HLA-DRB1/NFKBIZ/XBP1/CD46/HLA-DRA/ZFP36L1/MDK	14

BP	GO:0002699	positive regulation of immune effector process	16/432	261/18903	0.000353124055612025	0.00411771125830034	0.00281620991890584	B2M/CLEC7A/RSAD2/HLA-B/CD74/WNT5A/IL1B/HLA-A/HLA-DRB1/DNAJB9/NFKBIZ/XBP1/CD46/HLA-DRA/TFRC/HLA-E	16
BP	GO:0009308	amine metabolic process	10/432	117/18903	0.000354345747200765	0.00411771125830034	0.00281620991890584	SLC7A11/TGFB2/KYNU/NNMT/TDO2/EPAS1/SAT1/NR4A2/MAOA/MTPN	10
BP	GO:0045582	positive regulation of T cell differentiation	10/432	117/18903	0.000354345747200765	0.00411771125830034	0.00281620991890584	TGFBR2/CD74/VNN1/RUNX1/HLA-DRB1/NFKBIZ/XBP1/CD46/HLA-DRA/MDK	10
BP	GO:0048880	sensory system development	21/432	398/18903	0.000355638284323584	0.00412237359648265	0.00281939861335542	ANGPTL7/SLC7A11/TGFB2/PITX2/TGFBR2/CYP1B1/INHBA/CRYAB/WNT5A/RDH10/PRDM1/HIF1A/SKIL/CITED2/VIM/MAF/STAT3/ATF6/PBX1/JUN/WNT2B	21
BP	GO:0045429	positive regulation of nitric oxide biosynthetic process	6/432	42/18903	0.000359527576176131	0.00414667092223093	0.00283601618692551	CLEC7A/ASS1/SOD2/IL1B/CLU/HSP90AA1	6
BP	GO:0055069	zinc ion homeostasis	6/432	42/18903	0.000359527576176131	0.00414667092223093	0.00283601618692551	S100A9/S100A8/SLC39A14/SLC39A6/MT2A/MT1E	6
BP	GO:1903036	positive regulation of response to wounding	8/432	77/18903	0.000369831617703747	0.00425490356189012	0.00291003930662414	CLDN1/CLEC7A/GRN/XBP1/PLAU/SERPINE1/HBEGF/MDK	8

BP	GO:0006576	cellular biogenic amine metabolic process	9/432	97/18903	0.000378710124502631	0.00434623902189743	0.00297250600530339	TGFB2/KYNU/NNMT/TDO2/EPAS1/SAT1/NR4A2/MAOA/MTPN	9
BP	GO:0007566	embryo implantation	7/432	59/18903	0.0003796508355298889	0.0043462502829845	0.00297251370705398	TGFBR2/FBLN1/IL1B/GJA1/VMP1/TIMP1/IGFBP7	7
BP	GO:0032703	negative regulation of interleukin-2 production	5/432	28/18903	0.0003877863471727066	0.00442842433499695	0.00302871468032224	IL20RB/PRNP/GBP1/TNFAIP3/ZFP36	5
BP	GO:0090030	regulation of steroid hormone biosynthetic process	4/432	16/18903	0.0003936199393992133	0.00448397098453537	0.00306670447989275	BMP2/DAB2/ATP1A1/NR3C1	4
BP	GO:0061572	actin filament bundle organization	12/432	164/18903	0.0003998649918721755	0.00454392036218381	0.00310770541982642	PLEK/TGFB3/CD47/CGNL1/PLS1/MYO1B/NEDD9/ID1/TACSTD2/CCDC88A/RDX/PIK3R1	12
BP	GO:0034754	cellular hormone metabolic process	11/432	141/18903	0.0004019471450844755	0.00455638614219534	0.0031162310912767	BMP2/CYP1B1/DAB2/SDR16C5/TIPARP/RDH10/DHRS3/SCPEP1/EGR1/AKR1B1/CRABP2	11
BP	GO:0032609	interferon-gamma production	10/432	119/18903	0.0004059669397548555	0.00457737072034147	0.00313058299052657	CLEC7A/INHBA/IL20RB/CD47/WNT5A/PRNP/IL1B/HLA-A/HLA-DRB1/ISG15	10

BP	GO:0032649	regulation of interferon-gamma production	10/432	119/18903	0.000405966939754855	0.00457737072034147	0.00313058299052657	CLEC7A/INHBA/IL20RB/CD47/WNT5A/PRNP/IL1B/HLA-A/HLA-DRB1/ISG15	10
BP	GO:0060349	bone morphogenesis	9/432	98/18903	0.000408746834054276	0.00457737072034147	0.00313058299052657	MMP13/HAS2/TGFBR2/TGFB3/DHRS3/RUNX2/CITED2/NPPC/COL1A1	9
BP	GO:1990868	response to chemokine	9/432	98/18903	0.000408746834054276	0.00457737072034147	0.00313058299052657	CXCL1/CXCL8/CXCL6/CXCL2/CXCL3/DUSP1/CCL20/HIF1A/WNK1	9
BP	GO:1990869	cellular response to chemokine	9/432	98/18903	0.000408746834054276	0.00457737072034147	0.00313058299052657	CXCL1/CXCL8/CXCL6/CXCL2/CXCL3/DUSP1/CCL20/HIF1A/WNK1	9
BP	GO:0046686	response to cadmium ion	7/432	60/18903	0.000421468534502757	0.00470843471515761	0.00322022106834677	GCLC/SOD2/PRNP/MT2A/FOS/JUN/MT1E	7
BP	GO:0035967	cellular response to topologically incorrect protein	10/432	120/18903	0.000434034470330542	0.00483713114525002	0.00330823990702164	OPTN/DNAJB9/HSPA6/XBP1/ATF6/HSPA5/PTPN1/PIK3R1/TM7SF3/HSPA1A	10
BP	GO:0051091	positive regulation of DNA-binding transcription factor activity	16/432	266/18903	0.00043529234348603	0.00483948819380502	0.00330985195388543	CSF3/CLEC7A/S100A9/S100A8/BMP2/RAB7B/WNT5A/CAV1/IL1B/CLU/APP/TFRC/STAT3/PPP3CA/MTPN/HSPA1A	16

BP	GO:0030512	negative regulation of transforming growth factor beta receptor signaling pathway	9/432	99/18903	0.000440717769625715	0.00486475878562656	0.00332713516945839	BMP2/TGFB3/HTRA1/PMEPA1/CAV1/SKIL/HSPA5/SINHCAF/HSPA1A	9
BP	GO:0032755	positive regulation of interleukin-6 production	9/432	99/18903	0.000440717769625715	0.00486475878562656	0.00332713516945839	CLEC7A/RAB7B/CD74/WNT5A/IL1A/IL1B/APP/XBP1/STAT3	9
BP	GO:0002720	positive regulation of cytokine production involved in immune response	8/432	79/18903	0.000440720849984331	0.00486475878562656	0.00332713516945839	B2M/CLEC7A/RSAD2/CD74/WNT5A/IL1B/HLA-A/HLA-E	8
BP	GO:0051250	negative regulation of lymphocyte activation	12/432	166/18903	0.000446251557060543	0.0049140796462024	0.00336086698989206	TNFRSF21/SAMSN1/INHBA/CD74/IL20RB/RUNX1/ID2/PRNP/HLA-DRB1/LGALS3/TNFAIP3/MDK	12
BP	GO:0016064	immunoglobulin mediated immune response	14/432	215/18903	0.000447445482633046	0.00491552341372408	0.00336185442008327	B2M/C1S/C1R/CD74/CLU/HLA-DRB1/SERPING1/CD46/HLA-DRA/IGHG3/IGKC/TFRC/IGHG4/HLA-E	14
BP	GO:0003156	regulation of animal organ formation	5/432	29/18903	0.000459859466220719	0.00500435301475489	0.00342260729657774	BMP2/WNT5A/CITED2/SULF1/WNT2B	5

BP	GO:0010955	negative regulation of protein processing	5/432	29/18903	0.000459859466220719	0.00500435301475489	0.00342260729657774	LRRK2/PRNP/SERPINE2/PLAU/SERPINE1	5
BP	GO:0034698	response to gonadotropin	5/432	29/18903	0.000459859466220719	0.00500435301475489	0.00342260729657774	PAPPA/GCLC/CYP1B1/INHBA/GJB2	5
BP	GO:1903318	negative regulation of protein maturation	5/432	29/18903	0.000459859466220719	0.00500435301475489	0.00342260729657774	LRRK2/PRNP/SERPINE2/PLAU/SERPINE1	5
BP	GO:0014911	positive regulation of smooth muscle cell migration	6/432	44/18903	0.000465549781380244	0.00504254739785393	0.0034487294294284	HAS2/CYP1B1/PDGFD/IGFBP5/SEMA6D/MDK	6
BP	GO:1904407	positive regulation of nitric oxide metabolic process	6/432	44/18903	0.000465549781380244	0.00504254739785393	0.0034487294294284	CLEC7A/ASS1/SOD2/IL1B/CLU/HSP90AA1	6
BP	GO:1900024	regulation of substrate adhesion-dependent cell spreading	7/432	61/18903	0.000466884656002769	0.00504519050002993	0.00345053711580994	HAS2/DAB2/FBLN1/NEDD9/GBP1/TACSTD2/MDK	7
BP	GO:0070665	positive regulation of leukocyte proliferation	12/432	167/18903	0.000471096353222419	0.00507883597588272	0.00347354813258096	TGFBR2/BST2/VAV3/CD74/IL1A/IL1B/HLA-A/IL6ST/CD46/TFRC/HLA-E/PPP3CA	12

BP	GO:0036473	cell death in response to oxidative stress	9/432	100/18903	0.000474716918104249	0.00510596685170267	0.00349210364480604	SLC7A11/NCOA7/CYP1B1/SOD2/LRRK2/VNN1/HIF1A/ARL6IP5/MCL1	9
BP	GO:0003151	outflow tract morphogenesis	8/432	80/18903	0.000480089145332471	0.00514823515855332	0.00352101204015994	TGFB2/PITX2/TGFBR2/WNT5A/DHRS3/HIF1A/CITED2/JUN	8
BP	GO:0030177	positive regulation of Wnt signaling pathway	11/432	144/18903	0.000480872992107034	0.00514823515855332	0.00352101204015994	BMP2/DAB2/LRRK2/WNT5A/CAV1/CDH3/WNK1/TNFAIP3/SULF2/SULF1/COL1A1	11
BP	GO:0033674	positive regulation of kinase activity	24/432	496/18903	0.000489062518154867	0.00521178374761812	0.00356447457447338	TGFB2/FAM20A/TGFBR2/BMP2/LRRK2/PDGFD/VAV3/CD74/AREG/WNT5A/AFAP1L2/PRNP/ADRB2/IL1B/CLU/NEDD9/HLA-DRB1/WNK1/EGR1/TPD52L1/CCDC88A/HBEGF/PTPN1/HSP90AA1	24
BP	GO:0043410	positive regulation of MAPK cascade	24/432	496/18903	0.000489062518154867	0.00521178374761812	0.00356447457447338	TGFB2/BMP2/LRRK2/PDGFD/INHBA/TGFB3/CD74/WNT5A/CDH2/IL1A/IGFBP3/ADRB2/IL1B/APP/HLA-DRB1/CCL20/AVPI1/ARL6IP5/TPD52L1/GADD45A/CXCL17/PTPN1/GADD45B/JUN	24
BP	GO:0031649	heat generation	4/432	17/18903	0.000505491254517351	0.00537447598193735	0.00367574402861377	IL1A/ADRB2/IL1B/ARRDC3	4
BP	GO:0019724	B cell mediated immunity	14/432	218/18903	0.000513872540808374	0.0054374734810187	0.00371882965814195	B2M/C1S/C1R/CD74/CLU/HLA-DRB1/SERPING1/CD46/HLA-DRA/IGHG3/IGKC/TFRC/IGHG4/HLA-E	14

BP	GO:0042180	cellular ketone metabolic process	14/432	218/18903	0.000513872540808374	0.0054374734810187	0.00371882965814195	SLC7A11/KYNU/TDO2/BMP2/DAB2/FMO2/CD74/RDH10/CAV1/FABP5/IL1B/GPD2/EGR1/AKR1B1	14
BP	GO:0022617	extracellular matrix disassembly	7/432	62/18903	0.000516119104468586	0.0054374734810187	0.00371882965814195	MMP13/MMP7/MMP10/MMP12/MMP1/CTSV/CST3	7
BP	GO:0060393	regulation of pathway-restricted SMAD protein phosphorylation	7/432	62/18903	0.000516119104468586	0.0054374734810187	0.00371882965814195	TGFB2/TGFBR2/BMP2/DAB2/INHBA/TGFB3/PMEPA1	7
BP	GO:0001570	vasculogenesis	8/432	81/18903	0.000522260651693337	0.00548967162291292	0.00375452932616861	HAS2/TGFBR2/TIPARP/RIN2/ITGAV/CAV1/CITED2/ZFP36L1	8
BP	GO:0043254	regulation of protein-containing complex assembly	21/432	410/18903	0.000524953467540472	0.00550546437046414	0.00376533039732455	CSF3/CLEC7A/MMP1/PLEK/CRYAB/PMEPA1/CDC42EP3/ABCA1/MPP7/CLU/BIRC2/LGALS3/MSN/ISG15/TFRC/HSPA5/SVIL/MTPN/RDX/HSP90AA1/HSPA1A	21
BP	GO:0008631	intrinsic apoptotic signaling pathway in response to oxidative stress	6/432	45/18903	0.000526948972064053	0.00551388913076074	0.00377109231019881	CYP1B1/SOD2/VNN1/HIF1A/ARL6IP5/MCL1	6
BP	GO:0050671	positive regulation of lymphocyte proliferation	11/432	146/18903	0.000540382498351705	0.00564169086879601	0.00385849924568581	TGFBR2/VAV3/CD74/IL1A/IL1B/HLA-A/IL6ST/CD46/TFRC/HLA-E/PPP3CA	11

BP	GO:0031348	negative regulation of defense response	16/432	272/18903	0.000555143254204672	0.00578274223129866	0.00395496794425092	SAA1/MMP12/IFI16/HLA-B/IL20RB/HTRA1/GRN/HLA-A/HLA-DRB1/SERPING1/ISG15/TNFAIP3/CXCL17/HLA-E/ZFP36/MDK	16
BP	GO:0060395	SMAD protein signal transduction	8/432	82/18903	0.000567381500280675	0.0058969425590969	0.0040330725212145	TGFB2/BMP2/DAB2/INHBA/TGFB3/VIM/FOS/JUN	8
BP	GO:0071496	cellular response to external stimulus	18/432	328/18903	0.000585181514117947	0.0060683060600796	0.00415027248035032	CYP24A1/IFI16/GCLC/LRRK2/DAPL1/IL1B/NR4A2/XBP1/GADD45A/HSPA5/MTPN/LAMP2/MIOS/SLC38A2/FOS/POSTN/WNT2B/COL1A1	18
BP	GO:0032677	regulation of interleukin-8 production	9/432	103/18903	0.000589866796553043	0.00608958467423622	0.0041648254784819	CLEC7A/CD74/WNT5A/AFAP1L2/IL1B/CD58/SERPINE1/STAT3/HSPA1A	9
BP	GO:0070167	regulation of biomineral tissue development	9/432	103/18903	0.000589866796553043	0.00608958467423622	0.0041648254784819	BMP2/TGFB3/OMD/ODAPH/ADRB2/ANKH/AMTN/HIF1A/ISG15	9
BP	GO:0046596	regulation of viral entry into host cell	6/432	46/18903	0.000594462686032634	0.00612336285724039	0.00418792725712828	IFITM3/CD74/IFITM2/HLA-DRB1/LY6E/LGALS1	6
BP	GO:0007369	gastrulation	13/432	196/18903	0.000596162022912577	0.00612722079104593	0.0041905657984849	LAMA3/LAMB3/TGFBR2/ETS2/INHBA/ITGAV/LAMB1/WNT5A/DUSP1/IL1RN/COL12A1/GJA1/WNK1	13

BP	GO:0019730	antimicrobial humoral response	10/432	125/18903	0.000599702193837426	0.00614993934921972	0.00420610361181343	CXCL1/S100A9/CXCL8/CXCL6/CXCL2/CXCL3/SPRR2A/HLA-A/SLPI/HLA-E	10
BP	GO:1905475	regulation of protein localization to membrane	12/432	172/18903	0.000613517490304828	0.00627769555898192	0.00429347940932931	SLC7A11/GPC6/DAB2/CDH2/PLS1/PRNP/STOM/GBP1/LGALS3/TMEM59/MFF/PIK3R1	12
BP	GO:0007492	endoderm development	8/432	83/18903	0.000615602272552655	0.00628512253985879	0.00429855891488493	LAMA3/LAMB3/INHBA/ITGAV/LAMB1/DUSP1/COL12A1/ZFP36L1	8
BP	GO:0032637	interleukin-8 production	9/432	104/18903	0.000632976479600501	0.0064312334349845	0.00439848795951714	CLEC7A/CD74/WNT5A/AFAP1L2/IL1B/CD58/SERPINE1/STAT3/HSPA1A	9
BP	GO:0034505	tooth mineralization	5/432	31/18903	0.00063372430421391	0.0064312334349845	0.00439848795951714	FAM20A/ODAPH/ANKH/AMTN/COL1A1	5
BP	GO:0016264	gap junction assembly	4/432	18/18903	0.000638256464142245	0.0064312334349845	0.00439848795951714	CAV1/IL1B/GJB2/GJA1	4
BP	GO:0032354	response to follicle-stimulating hormone	4/432	18/18903	0.000638256464142245	0.0064312334349845	0.00439848795951714	PAPPA/GCLC/CYP1B1/INHBA	4

BP	GO:0051900	regulation of mitochondrial depolarization	4/432	18/18903	0.000638256464142245	0.0064312334349845	0.00439848795951714	DCN/GCLC/LRRK2/IFI6	4
BP	GO:0097067	cellular response to thyroid hormone stimulus	4/432	18/18903	0.000638256464142245	0.0064312334349845	0.00439848795951714	GCLC/CTSB/KLF9/RDX	4
BP	GO:0032946	positive regulation of mononuclear cell proliferation	11/432	149/18903	0.0006410951875595	0.00644579400535367	0.00440844631262311	TGFBR2/VAV3/CD74/IL1A/IL1B/HLA-A/IL6ST/CD46/TFRC/HLA-E/PPP3CA	11
BP	GO:0002695	negative regulation of leukocyte activation	13/432	198/18903	0.000655743190758483	0.00657876845392188	0.00449939099949593	TNFRSF21/SAMSN1/INHBA/CD74/IL20RB/RUNX1/ID2/GRN/PRNP/HLA-DRB1/LGALS3/TNFAIP3/MDK	13
BP	GO:0071695	anatomical structure maturation	15/432	250/18903	0.000668456021826492	0.00667776650378519	0.00456709834284483	CLEC7A/TGFB2/NRCAM/EPAS1/BMP2/LRRK2/WNT5A/CDH3/NR4A2/APP/RUNX2/HIF1A/PTBP3/AKR1B1/NPPC	15
BP	GO:0001774	microglial cell activation	6/432	47/18903	0.000668498571081631	0.00667776650378519	0.00456709834284483	LRRK2/GRN/CLU/IFNGR1/APP/JUN	6
BP	GO:0001890	placenta development	11/432	150/18903	0.000677954039237246	0.00672626637300514	0.00460026866591873	EPAS1/HTRA1/MME/PRDM1/GJB2/CTSB/BIRC2/HIF1A/CITED2/KRT19/ZFP36L1	11

BP	GO:0110149	regulation of biomineralization	9/432	105/18903	0.000678627781814454	0.00672626637300514	0.00460026866591873	BMP2/TGFB3/OMD/ODAPH/ADRB2/ANKH/AMTN/HIF1A/ISG15	9
BP	GO:0001704	formation of primary germ layer	10/432	127/18903	0.000679171112690464	0.00672626637300514	0.00460026866591873	LAMA3/LAMB3/ETS2/INHBA/ITGAV/LAMB1/WNT5A/DUSP1/COL12A1/GJA1	10
BP	GO:0002688	regulation of leukocyte chemotaxis	10/432	127/18903	0.000679171112690464	0.00672626637300514	0.00460026866591873	MMP28/CXCL8/CD74/WNT5A/DUSP1/NEDD9/WNK1/SERPINE1/CXCL17/MDK	10
BP	GO:0042176	regulation of protein catabolic process	19/432	361/18903	0.000690782743744445	0.00682664570473944	0.00466892070389577	GCLC/DAB2/LRRK2/TIPARP/WNT5A/CAV1/SERPINE2/IL1B/N4BP1/CLU/TRIB1/LAPTM4B/MSN/TNFAIP3/TIMP1/CST3/RDX/HSP90AA1/HSPA1A	19
BP	GO:0046651	lymphocyte proliferation	17/432	305/18903	0.000693483294585244	0.00683872118860715	0.00467717944165337	TNFRSF21/TGFBR2/VAV3/CD74/IL20RB/IL1A/PRNP/IL1B/HLA-A/IL6ST/HLA-DRB1/LGALS3/CD46/MSN/TFRC/HLA-E/PPP3CA	17
BP	GO:0045860	positive regulation of protein kinase activity	21/432	420/18903	0.000715813757313732	0.00704391197356598	0.00481751476086846	TGFB2/FAM20A/TGFBR2/BMP2/LRRK2/PDGFD/AREG/WNT5A/AFAP1L2/PRNP/ADRB2/IL1B/CLU/NEDD9/WNK1/EGR1/TPD52L1/CCDC88A/HBEGF/PTPN1/HSP90AA1	21
BP	GO:0042102	positive regulation of T cell proliferation	9/432	106/18903	0.000726932153528183	0.0071381342039657	0.00488195579696545	TGFBR2/IL1A/IL1B/HLA-A/IL6ST/CD46/TFRC/HLA-E/PPP3CA	9

BP	GO:0010644	cell communication by electrical coupling	5/432	32/18903	0.000737133922695753	0.00722297540777089	0.00493998090477417	CAV1/GJB2/ATP1A1/GJA1/ATP1B1	5
BP	GO:1903829	positive regulation of protein localization	22/432	451/18903	0.000747043439747134	0.00730460023008562	0.004995806245555	TGFB2/TGFB3/MYH10/WNT5A/IL1A/PLS1/PRNP/IL1B/NEDD9/STOM/HLA-DRB1/HIF1A/LGALS3/MSN/TFRC/CCDC88A/RDX/MFF/PIK3R1/TM7SF3/TMED10/HSP90AA1	22
BP	GO:0010812	negative regulation of cell-substrate adhesion	7/432	66/18903	0.000755892602080062	0.00733963160033627	0.00501976511158134	MMP12/FBLN1/GBP1/TACSTD2/SERPINE1/PIK3R1/COL1A1	7
BP	GO:0031343	positive regulation of cell killing	7/432	66/18903	0.000755892602080062	0.00733963160033627	0.00501976511158134	B2M/CLEC7A/HLA-B/HLA-A/HLA-DRB1/HLA-DRA/HLA-E	7
BP	GO:0060389	pathway-restricted SMAD protein phosphorylation	7/432	66/18903	0.000755892602080062	0.00733963160033627	0.00501976511158134	TGFB2/TGFBR2/BMP2/DAB2/INHBA/TGFB3/PMEPA1	7
BP	GO:0072006	nephron development	11/432	152/18903	0.00075697389694279	0.00733963160033627	0.00501976511158134	BMP2/PDGFD/BASP1/STAT1/TACSTD2/EGR1/SULF2/PBX1/PPP3CA/SULF1/WNT2B	11
BP	GO:0030111	regulation of Wnt signaling pathway	18/432	336/18903	0.000773701314940189	0.00748612673974555	0.00511995695088145	BMP2/DAB2/LRRK2/WNT5A/CDH2/CAV1/CDH3/MCC/APP/WNK1/EGR1/TNFAIP3/TAX1BP3/SULF2/SULF1/MDK/SOSTDC1/COL1A1	18

BP	GO:0006970	response to osmotic stress	8/432	86/18903	0.000780436535036593	0.0075355302182552	0.0051537452758707	CLDN1/TSC22D3/LRRC8D/ERRFI1/KCNMA1/AKR1B1/ZFP36L1/HSP90AA1	8
BP	GO:0015833	peptide transport	15/432	254/18903	0.000786478118389488	0.00757804436989871	0.00518282181087809	SLC7A11/S100A8/CPE/CD74/TAPBP/IL1B/IL1RN/ABCA1/SLC16A1/HLA-DRB1/HIF1A/GJA1/PPP3CA/TM7SF3/CA2	15
BP	GO:0002544	chronic inflammatory response	4/432	19/18903	0.000793964008164273	0.00763426930927185	0.00522127551052334	S100A9/S100A8/VNN1/TNFAIP3	4
BP	GO:0010631	epithelial cell migration	19/432	366/18903	0.00081399626047935	0.00778326363485518	0.00532317660830494	DCN/TGFB2/HAS2/TGFBR2/CYP1B1/RIN2/WNT5A/GRN/MCC/HIF1A/SP100/ID1/TACSTD2/GADD45A/FAT2/HBEGF/JUN/S100A2/SPARC	19
BP	GO:0032874	positive regulation of stress-activated MAPK cascade	10/432	130/18903	0.000814508021463764	0.00778326363485518	0.00532317660830494	TGFB2/BMP2/WNT5A/IL1A/IL1B/APP/ARL6IP5/TPD52L1/GADD45A/GADD45B	10
BP	GO:0045621	positive regulation of lymphocyte differentiation	10/432	130/18903	0.000814508021463764	0.00778326363485518	0.00532317660830494	TGFBR2/CD74/VNN1/RUNX1/HLA-DRB1/NFKBIZ/XBP1/CD46/HLA-DRA/MDK	10
BP	GO:0070613	regulation of protein processing	7/432	67/18903	0.000827770589315491	0.00789368860945185	0.00539869912293663	LRRK2/PLAUR/RUNX1/PRNP/SERPINE2/PLAU/SERPINE1	7

BP	GO:0032231	regulation of actin filament bundle assembly	9/432	108/18903	0.000831960586457422	0.0079010630644057	0.00540374270464617	PLEK/TGFB3/CD47/CGNL1/ID1/TACSTD2/CCDC88A/RDX/PIK3R1	9
BP	GO:1902106	negative regulation of leukocyte differentiation	9/432	108/18903	0.000831960586457422	0.0079010630644057	0.00540374270464617	TMEM176A/TMEM176B/INHBA/CD74/RUNX1/ID2/TRIB1/PIK3R1/MDK	9
BP	GO:0010862	positive regulation of pathway-restricted SMAD protein phosphorylation	6/432	49/18903	0.000837828130020993	0.00791386903512798	0.00541250104137913	TGFB2/TGFBR2/BMP2/DAB2/INHBA/TGFB3	6
BP	GO:0035987	endodermal cell differentiation	6/432	49/18903	0.000837828130020993	0.00791386903512798	0.00541250104137913	LAMA3/LAMB3/INHBA/ITGAV/LAMB1/COL12A1	6
BP	GO:0003012	muscle system process	22/432	455/18903	0.000838442341018964	0.00791386903512798	0.00541250104137913	CRYAB/RGS4/CAV1/ADRB2/IL1B/ATP1A1/SORBS2/IL6ST/IGFBP5/GJA1/FBXO32/MYL9/ASPH/ERRFI1/SULF2/KCNMA1/PPP3CA/MTPN/DSG2/ATP1B1/SULF1/HSP90AA1	22
BP	GO:0002449	lymphocyte mediated immunity	19/432	367/18903	0.000840784263984664	0.00791981104058874	0.00541656493359185	B2M/C1S/C1R/RSAD2/HLA-B/CD74/IL20RB/IL1B/HLA-A/CLU/HLA-DRB1/SERPING1/CD46/HLA-DRA/IGHG3/IGKC/TFRC/IGHG4/HLA-E	19
BP	GO:0090257	regulation of muscle system process	15/432	256/18903	0.000851833018691093	0.00796660635183916	0.00544856946509853	RGS4/CAV1/ADRB2/ATP1A1/IL6ST/IGFBP5/FBXO32/MYL9/ERRFI1/KCNMA1/PPP3CA/MTPN/DSG2/ATP1B1/HSP90AA1	15

BP	GO:0002063	chondrocyte development	5/432	33/18903	0.000852642193331975	0.00796660635183916	0.00544856946509853	TGFBR2/RUNX2/PTHLH/SULF2/SULF1	5
BP	GO:0036475	neuron death in response to oxidative stress	5/432	33/18903	0.000852642193331975	0.00796660635183916	0.00544856946509853	SLC7A11/NCOA7/HIF1A/ARL6IP5/MCL1	5
BP	GO:0090183	regulation of kidney development	5/432	33/18903	0.000852642193331975	0.00796660635183916	0.00544856946509853	BASP1/STAT1/TACSTD2/PPP3CA/WNT2B	5
BP	GO:0016055	Wnt signaling pathway	22/432	456/18903	0.000862749302085448	0.0080447893591637	0.00550204085901267	CPE/BMP2/GPC6/DAB2/LRRK2/WNT5A/CDH2/CAV1/CDH3/MCC/NR4A2/APP/WNK1/EGR1/TNFAIP3/TAX1BP3/SULF2/SULF1/WNT2B/MDK/SOSTDC1/COL1A1	22
BP	GO:0050864	regulation of B cell activation	13/432	204/18903	0.000865448191182309	0.00805371807689774	0.00550814744149706	TNFRSF21/SAMSN1/INHBA/VAV3/CD74/ID2/XBP1/TNFAIP3/IGHG3/IGKC/TFRC/IGHG4/ZFP36L1	13
BP	GO:0043010	camera-type eye development	18/432	340/18903	0.000886065126816047	0.00821252747800444	0.00561676132492822	ANGPTL7/SLC7A11/TGFB2/PITX2/TGFBR2/CYP1B1/INHBA/CRYAB/WNT5A/RDH10/HIF1A/SKIL/CITED2/VIM/MAF/STAT3/JUN/WNT2B	18
BP	GO:0070371	ERK1 and ERK2 cascade	18/432	340/18903	0.000886065126816047	0.00821252747800444	0.00561676132492822	BMP2/DAB2/PDGFD/INHBA/CD74/ITGAV/FBLN1/IL1A/IL1B/APP/HLA-DRB1/CCL20/GBP1/ERRFI1/CXCL17/PTPN1/JUN/ZFP36L1	18

BP	GO:0022604	regulation of cell morphogenesis	17/432	312/18903	0.000891547530169881	0.00823035394617904	0.00562895331055886	HAS2/DAB2/MYH10/WNT5A/PALMD/FBLN1/SLC23A2/RND3/CDC42EP3/NEDD9/GBP1/TACSTD2/MSN/RDX/CUX1/SPARC/MDK	17
BP	GO:0032943	mononuclear cell proliferation	17/432	312/18903	0.000891547530169881	0.00823035394617904	0.00562895331055886	TNFRSF21/TGFBR2/VAV3/CD74/IL20RB/IL1A/PRNP/IL1B/HLA-A/IL6ST/HLA-DRB1/LGALS3/CD46/MSN/TFRC/HLA-E/PPP3CA	17
BP	GO:0090132	epithelium migration	19/432	369/18903	0.000896639398717415	0.00826087095431882	0.00564982468682289	DCN/TGFB2/HAS2/TGFBR2/CYP1B1/RIN2/WNT5A/GRN/MCC/HIF1A/SP100/ID1/TACSTD2/GADD45A/FAT2/HBEGF/JUN/S100A2/SPARC	19
BP	GO:0043281	regulation of cysteine-type endopeptidase activity involved in apoptotic process	13/432	205/18903	0.000905350192025732	0.00832454202409346	0.00569337098888212	TNFSF10/CLEC7A/BIRC3/S100A9/S100A8/PLAUR/CRYAB/TNFAIP8/IFI6/BIRC2/ARL6IP5/CTSD/UACA	13
BP	GO:0198738	cell-cell signaling by wnt	22/432	458/18903	0.000913197008317397	0.00837699007436647	0.00572924157575448	CPE/BMP2/GPC6/DAB2/LRRK2/WNT5A/CDH2/CAV1/CDH3/MCC/NR4A2/APP/WNK1/EGR1/TNFAIP3/TAX1BP3/SULF2/SULF1/WNT2B/MDK/SOSTDC1/COL1A1	22
BP	GO:0048565	digestive tract development	10/432	132/18903	0.000916487995163121	0.00837699007436647	0.00572924157575448	TGFB2/ASS1/TGFBR2/CXCL8/TGFB3/ID2/WNT5A/PRDM1/IL6ST/HIF1A	10
BP	GO:0070304	positive regulation of stress-activated	10/432	132/18903	0.00091648799	0.00837699007436647	0.00572924157575448	TGFB2/BMP2/WNT5A/IL1A/IL1B/APP/ARL6IP5/TPD52L1/GADD45A/GADD45B	10

		protein kinase signaling cascade			5163121				
BP	GO:0002269	leukocyte activation involved in inflammatory response	6/432	50/18903	0.00093399660411841	0.00848670784685196	0.00580428041645295	LRRK2/GRN/CLU/IFNGR1/APP/JUN	6
BP	GO:0030195	negative regulation of blood coagulation	6/432	50/18903	0.00093399660411841	0.00848670784685196	0.00580428041645295	PLAUR/SERPINE2/SERPING1/PLAU/SERPINE1/PROS1	6
BP	GO:2001238	positive regulation of extrinsic apoptotic signaling pathway	6/432	50/18903	0.00093399660411841	0.00848670784685196	0.00580428041645295	TNFSF10/INHBA/SRPX/CAV1/G0S2/SKIL	6
BP	GO:0050890	cognition	17/432	314/18903	0.000956307638384521	0.0086723977010361	0.00593127854147675	B2M/SLC7A11/TMPRSS11E/MME/PRNP/UBA6/APP/NEDD9/HIF1A/TUSC3/ARL6IP5/GM2A/HLA-DRA/CHL1/SGK1/FOS/MDK	17
BP	GO:0002396	MHC protein complex assembly	4/432	20/18903	0.000974675954179882	0.00870246387660609	0.00595184158161054	B2M/TAPBP/HLA-DRB1/HLA-DRA	4
BP	GO:0002501	peptide antigen assembly with MHC protein complex	4/432	20/18903	0.000974675954179882	0.00870246387660609	0.00595184158161054	B2M/TAPBP/HLA-DRB1/HLA-DRA	4
BP	GO:0006700	C21-steroid hormone	4/432	20/18903	0.00097467595	0.00870246387660609	0.00595184158161054	BMP2/DAB2/EGR1/AKR1B1	4

		biosynthetic process			0.000974675954179882				
BP	GO:0035455	response to interferon-alpha	4/432	20/18903	0.000974675954179882	0.00870246387660609	0.00595184158161054	BST2/IFITM3/IFIT3/IFITM2	4
BP	GO:0036498	IRE1-mediated unfolded protein response	4/432	20/18903	0.000974675954179882	0.00870246387660609	0.00595184158161054	DNAJB9/XBP1/HSPA5/PTPN1	4
BP	GO:0051882	mitochondrial depolarization	4/432	20/18903	0.000974675954179882	0.00870246387660609	0.00595184158161054	DCN/GCLC/LRRK2/IFI6	4
BP	GO:0098543	detection of other organism	4/432	20/18903	0.000974675954179882	0.00870246387660609	0.00595184158161054	CLEC7A/HLA-B/HLA-A/HLA-DRB1	4
BP	GO:1902236	negative regulation of endoplasmic reticulum stress-induced intrinsic apoptotic signaling pathway	4/432	20/18903	0.000974675954179882	0.00870246387660609	0.00595184158161054	LRRK2/XBP1/PTPN1/HSPA1A	4
BP	GO:0034405	response to fluid shear stress	5/432	34/18903	0.000981108337670456	0.00874301745997275	0.00597957723293727	ASS1/HAS2/TGFB3/ABCA1/CITED2	5

BP	GO:0048645	animal organ formation	7/432	69/18903	0.000987661879682112	0.00878449267986494	0.0060079432154752	TGFBR2/BMP2/WNT5A/RDH10/CITED2/SULF1/WNT2B	7
BP	GO:0046890	regulation of lipid biosynthetic process	12/432	182/18903	0.00100811686385073	0.00894921400251364	0.00612060041594389	BMP2/DAB2/CD74/RDH10/IL1A/FABP5/IL1B/ATP1A1/EGR1/CAPN2/NR3C1/IGFBP7	12
BP	GO:0001913	T cell mediated cytotoxicity	6/432	51/18903	0.0010384360079382	0.00914812673659842	0.00625664201274043	B2M/HLA-B/HLA-A/HLA-DRB1/HLA-DRA/HLA-E	6
BP	GO:0060324	face development	6/432	51/18903	0.0010384360079382	0.00914812673659842	0.00625664201274043	TGFB3/TIPARP/WNT5A/ZFAND5/ASPH/COL1A1	6
BP	GO:0060688	regulation of morphogenesis of a branching structure	6/432	51/18903	0.0010384360079382	0.00914812673659842	0.00625664201274043	LRRK2/WNT5A/TACSTD2/SULF1/WNT2B/MDK	6
BP	GO:1900047	negative regulation of hemostasis	6/432	51/18903	0.0010384360079382	0.00914812673659842	0.00625664201274043	PLAUR/SERPINE2/SERPING1/PLAU/SERPINE1/PROS1	6
BP	GO:0090101	negative regulation of transmembrane receptor protein serine/threonine kinase signaling pathway	11/432	158/18903	0.00104121629403984	0.00915518129265066	0.00626146680726378	BMP2/TGFB3/HTRA1/PMEPA1/WNT5A/CAV1/SKIL/HSPA5/SINHCAF/HSPA1A/SOSTDC1	11

BP	GO:0090130	tissue migration	19/432	374/18903	0.00105039563473975	0.00920768040953343	0.00629737231991988	DCN/TGFB2/HAS2/TGFBR2/CYP1B1/RIN2/WNT5A/GRN/MCC/HIF1A/SP100/ID1/TACSTD2/GADD45A/FAT2/HBEGF/JUN/S100A2/SPARC	19
BP	GO:0010586	miRNA metabolic process	8/432	90/18903	0.00105352366767444	0.00920768040953343	0.00629737231991988	TGFB2/BMP2/HIF1A/EGR1/NR3C1/STAT3/FOS/JUN	8
BP	GO:0070098	chemokine-mediated signaling pathway	8/432	90/18903	0.00105352366767444	0.00920768040953343	0.00629737231991988	CXCL1/CXCL8/CXCL6/CXCL2/CXCL3/CCL20/HIF1A/WNK1	8
BP	GO:0007219	Notch signaling pathway	12/432	183/18903	0.00105714125350535	0.00920768040953343	0.00629737231991988	TGFB2/TGFBR2/BMP2/GALNT11/NFKBIA/SORBS2/IL6ST/APP/CD46/STAT3/KRT19/POSTN	12
BP	GO:0008361	regulation of cell size	12/432	183/18903	0.00105714125350535	0.00920768040953343	0.00629737231991988	NRCAM/PLEK/VAV3/TMEM123/WNT5A/MSN/KCNMA1/MTPN/RDX/SEMA6D/CRABP2/HSP90AA1	12
BP	GO:0009913	epidermal cell differentiation	14/432	235/18903	0.00107032958209337	0.00930377586057381	0.00636309450037537	PITX2/ZBED2/WNT5A/IL1A/PLS1/CDH3/FOSL2/MYO6/ERRFI1/PPP3CA/PALLD/ZFP36/TXNIP/ZFP36L1	14
BP	GO:0030193	regulation of blood coagulation	7/432	70/18903	0.00107622055900692	0.00930377586057381	0.00636309450037537	PLAUR/CAV1/SERPINE2/SERPING1/PLAU/SERPINE1/PROS1	7
BP	GO:0032722	positive regulation of chemokine production	7/432	70/18903	0.00107622055900692	0.00930377586057381	0.00636309450037537	CLEC7A/CD74/WNT5A/IL1B/APP/HIF1A/EGR1	7
BP	GO:0043627	response to estrogen	7/432	70/18903	0.00107622055900692	0.00930377586057381	0.00636309450037537	TGFBR2/TGFB3/MME/CAV1/CITED2/KRT19/HSP90AA1	7

BP	GO:1901099	negative regulation of signal transduction in absence of ligand	5/432	35/18903	0.0011234085294352	0.00965755473724499	0.00660505308345291	IL1A/IL1B/IFI6/MCL1/HSPA1A	5
BP	GO:1905898	positive regulation of response to endoplasmic reticulum stress	5/432	35/18903	0.0011234085294352	0.00965755473724499	0.00660505308345291	CAV1/XBP1/ATF6/PTPN1/PIK3R1	5
BP	GO:2001240	negative regulation of extrinsic apoptotic signaling pathway in absence of ligand	5/432	35/18903	0.0011234085294352	0.00965755473724499	0.00660505308345291	IL1A/IL1B/IFI6/MCL1/HSPA1A	5
BP	GO:0031396	regulation of protein ubiquitination	13/432	210/18903	0.00112875623784196	0.00968167514560421	0.00662154965861523	BIRC3/GCLC/LRRK2/CAV1/NDFIP2/N4BP1/BIRC2/ISG15/TNFAIP3/HSPA5/ARRDC3/HSP90AA1/HSPA1A	13
BP	GO:0060021	roof of mouth development	8/432	91/18903	0.00113249432513987	0.00968167514560421	0.00662154965861523	TGFB2/TGFBR2/INHBA/TGFB3/TIPARP/WNT5A/DHRS3/ASPH	8
BP	GO:2000117	negative regulation of cysteine-type endopeptidase activity	8/432	91/18903	0.00113249432513987	0.00968167514560421	0.00662154965861523	BIRC3/IFI16/PLAUR/CRYAB/TNFAIP8/IFI6/BIRC2/CST3	8
BP	GO:0010565	regulation of cellular ketone metabolic process	10/432	136/18903	0.00115194984867357	0.00982983035076619	0.00672287686009728	SLC7A11/BMP2/DAB2/FMO2/CD74/RDH10/CAV1/FABP5/IL1B/EGR1	10

BP	GO:0002274	myeloid leukocyte activation	14/432	237/18903	0.0011606742007292	0.00988603716090706	0.00676131816068864	TGFBR2/CXCL8/CXCL6/LRRK2/CD74/WNT5A/GRN/PLSCR1/CLU/IFNGR1/APP/S100A13/KMT2E/JUN	14
BP	GO:0060541	respiratory system development	13/432	211/18903	0.00117854938248745	0.0100198362022141	0.00685282680800075	MMP12/SLC7A11/ASS1/EPAS1/TGFBR2/TGFB3/BASP1/WNT5A/MME/RDH10/IGFBP5/ERRFI1/WNT2B	13
BP	GO:0009408	response to heat	9/432	114/18903	0.00122321938709653	0.0103425770846827	0.007073557698884	GCLC/CRYAB/IL1A/HSPA6/DNAJB4/IGFBP7/HSP90AA1/HSPA1A/DNAJB1	9
BP	GO:0044344	cellular response to fibroblast growth factor stimulus	9/432	114/18903	0.00122321938709653	0.0103425770846827	0.007073557698884	GCLC/CXCL8/WNT5A/RUNX2/SULF2/ZFP36/SULF1/ZFP36L1/COL1A1	9
BP	GO:1903707	negative regulation of hemopoiesis	9/432	114/18903	0.00122321938709653	0.0103425770846827	0.007073557698884	TMEM176A/TMEM176B/INHBA/CD74/RUNX1/ID2/TRIB1/PIK3R1/MDK	9
BP	GO:0042113	B cell activation	18/432	350/18903	0.00122975986216122	0.0103789039461599	0.00709840258508349	TNFRSF21/SAMSN1/BST2/INHBA/VAV3/CD74/ID2/DNAJB9/XBP1/TNFAIP3/IGHG3/IGKC/TFRC/IGHG4/PIK3R1/KLF6/ZFP36L1/LGALS1	18
BP	GO:0030522	intracellular receptor signaling pathway	15/432	266/18903	0.00125191025317284	0.0105357956122277	0.00720570487960037	CYP24A1/BIRC3/BMP2/DAB2/PMEPA1/FABP5/DHRS3/NFKBIA/NR4A2/BIRC2/CITED2/TNFAIP3/NR3C1/STAT3/HSPA1A	15
BP	GO:0033483	gas homeostasis	3/432	10/18903	0.00126201746360522	0.0105357956122277	0.00720570487960037	SOD2/CAV1/HIF1A	3
BP	GO:0045657	positive regulation of monocyte differentiation	3/432	10/18903	0.00126201746360522	0.0105357956122277	0.00720570487960037	CD74/HLA-DRB1/ZFP36L1	3

BP	GO:0046886	positive regulation of hormone biosynthetic process	3/432	10/18903	0.00126201746360522	0.0105357956122277	0.00720570487960037	DAB2/HIF1A/EGR1	3
BP	GO:0060397	growth hormone receptor signaling pathway via JAK-STAT	3/432	10/18903	0.00126201746360522	0.0105357956122277	0.00720570487960037	JAK1/STAT3/PTPN1	3
BP	GO:2000347	positive regulation of hepatocyte proliferation	3/432	10/18903	0.00126201746360522	0.0105357956122277	0.00720570487960037	XBP1/TNFAIP3/MDK	3
BP	GO:1900046	regulation of hemostasis	7/432	72/18903	0.00127197638356204	0.0105807388021123	0.00723644269709189	PLAUR/CAV1/SERPINE2/SERPING1/PLAU/SERPINE1/PROS1	7
BP	GO:1903317	regulation of protein maturation	7/432	72/18903	0.00127197638356204	0.0105807388021123	0.00723644269709189	LRRK2/PLAUR/RUNX1/PRNP/SERPINE2/PLAU/SERPINE1	7
BP	GO:0030224	monocyte differentiation	5/432	36/18903	0.00128043412024893	0.0106129172153249	0.00725845035153374	IFI16/CD74/HLA-DRB1/JUN/ZFP36L1	5
BP	GO:0050869	negative regulation of B cell activation	5/432	36/18903	0.00128043412024893	0.0106129172153249	0.00725845035153374	TNFRSF21/SAMSN1/INHBA/ID2/TNFAIP3	5
BP	GO:0008202	steroid metabolic process	17/432	323/18903	0.00129965397743298	0.0107440394532683	0.00734812826334478	CYP24A1/CYP27A1/APOL1/BMP2/CYP1B1/DAB2/TIPARP/IL1A/ABCA1/ATP1A1/APP/NPC2/EGR1/NR3C1/PBX1/AKR1B1/IGFBP7	17
BP	GO:0046634	regulation of alpha-beta T cell activation	9/432	115/18903	0.00130089991217951	0.0107440394532683	0.00734812826334478	TGFBR2/RUNX1/PRDM1/HLA-A/HLA-DRB1/NFKBIZ/HLA-DRA/HSPH1/HLA-E	9

BP	GO:0010721	negative regulation of cell development	12/432	188/18903	0.00133296164300953	0.0109696576493222	0.00750243442075976	B2M/WNT5A/FBLN1/IL1A/IL1B/GBP1/ID1/TACSTD2/PTHLH/PPP3CA/SEMA6D/NPPC	12
BP	GO:0030308	negative regulation of cell growth	12/432	188/18903	0.00133296164300953	0.0109696576493222	0.00750243442075976	TGFB2/BST2/DAB2/INHBA/CRYAB/RGS4/WNT5A/OSGIN1/SERPINE2/GJA1/SEMA6D/HSPA1A	12
BP	GO:0002703	regulation of leukocyte mediated immunity	14/432	241/18903	0.00136063818583077	0.0111775339422154	0.00764460642875845	B2M/CLEC7A/BST2/CXCL6/RSAD2/HLA-B/IL20RB/IL1B/HLA-A/HLA-DRB1/CD46/HLA-DRA/TFRC/HLA-E	14
BP	GO:0150076	neuroinflammatory response	7/432	73/18903	0.00137975744361609	0.0113145003133412	0.00773828129538328	LRRK2/GRN/IL1B/CLU/IFNGR1/APP/JUN	7
BP	GO:0032868	response to insulin	15/432	269/18903	0.00139903169705095	0.0114522506174525	0.00783249231418371	TNFSF10/GCLC/RAB31/SLC39A14/STAT1/IL1B/XBP1/EGR1/ERRFI1/PKM/PTPN1/PIK3R1/FOS/ZFP36L1/COL1A1	15
BP	GO:0045778	positive regulation of ossification	6/432	54/18903	0.00140607591100443	0.0114875149544286	0.007856610512218	TGFB2/BMP2/TGFB3/WNT5A/ADRB2/ISG15	6
BP	GO:0030856	regulation of epithelial cell differentiation	11/432	164/18903	0.00140830723873751	0.0114875149544286	0.007856610512218	BMP2/ZBED2/STAT1/CAV1/IL1A/IL1B/ID1/SERPINE1/ERRFI1/ZFP36/ZFP36L1	11
BP	GO:0006883	cellular sodium ion homeostasis	4/432	22/18903	0.0014193786499519	0.0115371287452154	0.0078905426752483	SLC1A3/IL1A/ATP1A1/ATP1B1	4
BP	GO:0072111	cell proliferation involved in kidney development	4/432	22/18903	0.0014193786499519	0.0115371287452154	0.0078905426752483	BMP2/PDGFD/STAT1/EGR1	4

BP	GO:0002705	positive regulation of leukocyte mediated immunity	10/432	140/18903	0.00143464485748557	0.011640758712054	0.00796141790719141	B2M/CLEC7A/RSAD2/HLA-B/IL1B/HLA-A/HLA-DRB1/HLA-DRA/TFRC/HLA-E	10
BP	GO:0034110	regulation of homotypic cell-cell adhesion	5/432	37/18903	0.00145308986209612	0.0117697734013915	0.0080496544116487	PLAUR/SERPINE2/IL6ST/JAK1/RDX	5
BP	GO:0021782	glial cell development	9/432	117/18903	0.00146818887874127	0.0118712824548573	0.00811907909572604	S100A9/S100A8/GRN/IL1B/CLU/IFNGR1/APP/VIM/MDK	9
BP	GO:0042058	regulation of epidermal growth factor receptor signaling pathway	7/432	74/18903	0.00149454003090655	0.0120399823591411	0.00823445735174257	PLAUR/AREG/AFAP1L2/IFI6/ERRFI1/CCDC88A/HBEGF	7
BP	GO:0050688	regulation of defense response to virus	7/432	74/18903	0.00149454003090655	0.0120399823591411	0.00823445735174257	MMP12/IFIT1/HTRA1/STAT1/IL1B/TNFAIP3/HSP90AA1	7
BP	GO:1900407	regulation of cellular response to oxidative stress	8/432	95/18903	0.001496862671677	0.0120399823591411	0.00823445735174257	SLC7A11/NCOA7/SOD2/LRRK2/VNN1/HIF1A/PNPLA8/MCL1	8
BP	GO:0043525	positive regulation of neuron apoptotic process	6/432	55/18903	0.00154834346773327	0.0123680285634998	0.00845881669037084	TGFB2/GRN/PRNP/SRPK2/NR3C1/MCL1	6
BP	GO:0070169	positive regulation of biomineral tissue development	6/432	55/18903	0.00154834346773327	0.0123680285634998	0.00845881669037084	BMP2/TGFB3/ODAPH/ADRB2/AMTN/ISG15	6

BP	GO:0071320	cellular response to cAMP	6/432	55/18903	0.00154834346773327	0.0123680285634998	0.00845881669037084	ASS1/CYP1B1/APP/IGFBP5/HSPA5/ZFP36L1	6
BP	GO:1904645	response to amyloid-beta	6/432	55/18903	0.00154834346773327	0.0123680285634998	0.00845881669037084	MMP13/MMP12/PRNP/ADRB2/APP/GJA1	6
BP	GO:0032091	negative regulation of protein binding	8/432	96/18903	0.00160107595859903	0.0127672005319319	0.00873182079054461	B2M/DAB2/IFIT1/LRRK2/CAV1/ADRB2/SLPI/ID1	8
BP	GO:0051051	negative regulation of transport	22/432	479/18903	0.0016165915072616	0.0128249034196785	0.00877128529755536	RSAD2/LRRK2/INHBA/CD74/CRYAB/CD47/RGS4/ITGAV/CAV1/FABP5/SERPINE2/NDFIP2/PLSCR1/IL1B/GEM/SP100/GJA1/LGALS3/WNK1/ARL6IP5/LMAN1/PPP3CA	22
BP	GO:0050810	regulation of steroid biosynthetic process	7/432	75/18903	0.00161663106890218	0.0128249034196785	0.00877128529755536	BMP2/DAB2/IL1A/ATP1A1/EGR1/NR3C1/IGFBP7	7
BP	GO:2000242	negative regulation of reproductive process	7/432	75/18903	0.00161663106890218	0.0128249034196785	0.00877128529755536	WNT5A/DUSP1/IL1A/GJA1/TIMP1/SULF1/NPPC	7
BP	GO:0045742	positive regulation of epidermal growth factor receptor signaling pathway	5/432	38/18903	0.0016422921327916	0.0129839335284806	0.00888005012872469	PLAUR/AREG/AFAP1L2/CCDC88A/HBEGF	5
BP	GO:0048009	insulin-like growth factor receptor signaling pathway	5/432	38/18903	0.0016422921327916	0.0129839335284806	0.00888005012872469	BMP2/IGFBP3/CDH3/IGFBP5/PIK3R1	5

BP	GO:0002708	positive regulation of lymphocyte mediated immunity	9/432	119/18903	0.00165235108711591	0.0130411668565035	0.0089191934916172	B2M/RSAD2/HLA-B/IL1B/HLA-A/HLA-DRB1/HLA-DRA/TFRC/HLA-E	9
BP	GO:0055123	digestive system development	10/432	143/18903	0.00168169556405705	0.0132501567014716	0.00906212708686708	TGFB2/ASS1/TGFBR2/CXCL8/TGFB3/ID2/WNT5A/PRDM1/IL6ST/HIF1A	10
BP	GO:0048143	astrocyte activation	4/432	23/18903	0.00168748763916176	0.0132731808352434	0.00907787389130162	GRN/IL1B/IFNGR1/APP	4
BP	GO:0001836	release of cytochrome c from mitochondria	6/432	56/18903	0.0017013007164103	0.013277098773092	0.00908055347098524	TNFSF10/SOD2/PLAUR/IFI6/CLU/MFF	6
BP	GO:0110151	positive regulation of biomineralization	6/432	56/18903	0.0017013007164103	0.013277098773092	0.00908055347098524	BMP2/TGFB3/ODAPH/ADRB2/AMTN/ISG15	6
BP	GO:0031943	regulation of glucocorticoid metabolic process	3/432	11/18903	0.00170596596757411	0.013277098773092	0.00908055347098524	BMP2/ATP1A1/NR3C1	3
BP	GO:0044848	biological phase	3/432	11/18903	0.00170596596757411	0.013277098773092	0.00908055347098524	TGFB2/WNT5A/CDH3	3
BP	GO:0061418	regulation of transcription from RNA polymerase II promoter in response to hypoxia	3/432	11/18903	0.00170596596757411	0.013277098773092	0.00908055347098524	HIF1A/CITED2/EGR1	3

BP	GO:0071492	cellular response to UV-A	3/432	11/18903	0.00170596596757411	0.013277098773092	0.00908055347098524	MMP1/MME/TIMP1	3
BP	GO:0002690	positive regulation of leukocyte chemotaxis	8/432	97/18903	0.00171095153919196	0.013277098773092	0.00908055347098524	CXCL8/CD74/WNT5A/NEDD9/WNK1/SERPINE1/CXCL17/MDK	8
BP	GO:0060993	kidney morphogenesis	8/432	97/18903	0.00171095153919196	0.013277098773092	0.00908055347098524	BMP2/LRRK2/BASP1/STAT1/TACSTD2/PBX1/PPP3CA/WNT2B	8
BP	GO:0071774	response to fibroblast growth factor	9/432	120/18903	0.0017511335155039	0.0135661516067095	0.00927824422831567	GCLC/CXCL8/WNT5A/RUNX2/SULF2/ZFP36/SULF1/ZFP36L1/COL1A1	9
BP	GO:0021700	developmental maturation	16/432	304/18903	0.00178413282265136	0.0137986861283654	0.00943728064085077	CLEC7A/TGFB2/NRCAM/EPAS1/BMP2/LRRK2/WNT5A/CDH3/NR4A2/APP/RUNX2/HIF1A/PTBP3/CD63/AKR1B1/NPPC	16
BP	GO:0001837	epithelial to mesenchymal transition	11/432	169/18903	0.00178983168977037	0.0138196520286944	0.00945161976585529	TGFB2/HAS2/TGFBR2/BMP2/DAB2/TGFB3/WNT5A/IL1B/HIF1A/MDK/COL1A1	11
BP	GO:0032642	regulation of chemokine production	8/432	98/18903	0.00182670306853753	0.0140340559667543	0.0095982562003065	CLEC7A/CXCL6/CD74/WNT5A/IL1B/APP/HIF1A/EGR1	8
BP	GO:0048661	positive regulation of smooth muscle cell proliferation	8/432	98/18903	0.00182670306853753	0.0140340559667543	0.0095982562003065	TGFBR2/PDGFD/ID2/STAT1/IGFBP5/GJA1/HBEGF/JUN	8
BP	GO:0120162	positive regulation of cold-induced thermogenesis	8/432	98/18903	0.00182670306853753	0.0140340559667543	0.0095982562003065	EPAS1/CAV1/FABP5/ADRB2/G0S2/GJA1/FABP4/DIO2	8

BP	GO:0009595	detection of biotic stimulus	5/432	39/18903	0.00184896717020786	0.0141815475326888	0.00969912952164547	CLEC7A/HLA-B/SRPX/HLA-A/HLA-DRB1	5
BP	GO:0010810	regulation of cell-substrate adhesion	13/432	222/18903	0.00185805281263404	0.0142276395007159	0.00973065301841284	MMP12/HAS2/DAB2/RIN2/FBLN1/NEDD9/GBP1/TACSTD2/PLAU/SERPINE1/PIK3R1/MDK/COL1A1	13
BP	GO:1903053	regulation of extracellular matrix organization	6/432	57/18903	0.00186545751960596	0.0142607289721943	0.00975328376931866	ANGPTL7/HAS2/BMP2/RUNX1/LAMB1/CST3	6
BP	GO:0030216	keratinocyte differentiation	11/432	170/18903	0.00187541699320902	0.0143132072501514	0.009789175029691	ZBED2/WNT5A/IL1A/CDH3/FOSL2/ERRFI1/PPP3CA/PALLD/ZFP36/TXNIP/ZFP36L1	11
BP	GO:0007015	actin filament organization	21/432	454/18903	0.00187912056302071	0.0143178461350425	0.0097923476838242	CSF3/IQGAP2/PLEK/TGFB3/CD47/CGNL1/RGS4/PLS1/RND3/CDC42EP3/MYO1B/SORBS2/NEDD9/ID1/TACSTD2/MYO6/SVIL/CCDC88A/MTPN/RDX/PIK3R1	21
BP	GO:0016485	protein processing	14/432	250/18903	0.00191755075780946	0.0145866319981394	0.00997617733244016	IFI16/CPE/C1R/LRRK2/PLAUR/RUNX1/MME/PRNP/SERPINE2/CPD/PLAU/CAPN2/ASPH/SERPINE1	14
BP	GO:0032602	chemokine production	8/432	99/18903	0.00194854792998033	0.014798085675138	0.0101207960065524	CLEC7A/CXCL6/CD74/WNT5A/IL1B/APP/HIF1A/EGR1	8
BP	GO:0010720	positive regulation of cell development	16/432	307/18903	0.00197042407970251	0.0149396907682363	0.0102176434158862	CLEC7A/HAS2/BMP2/DAB2/ID2/MME/SERPINE2/IL1B/IL6ST/NEDD9/HIF1A/TRIB1/SKIL/CUX1/CRABP2/MDK	16
BP	GO:0035458	cellular response to interferon-beta	4/432	24/18903	0.0019888233981255	0.0150053967639974	0.0102625814567994	IFI16/STAT1/IFITM2/CAPN2	4

BP	GO:0036120	cellular response to platelet-derived growth factor stimulus	4/432	24/18903	0.0019888233981255	0.0150053967639974	0.0102625814567994	HAS2/PDGFD/ERRFI1/RDX	4
BP	GO:1902074	response to salt	4/432	24/18903	0.0019888233981255	0.0150053967639974	0.0102625814567994	DAB2/ANKH/RUNX2/HSPA5	4
BP	GO:0051592	response to calcium ion	10/432	147/18903	0.00206356857491071	0.0153922605150161	0.0105271676466853	CPNE8/WNT5A/CAV1/FOSB/HSPA5/KCNMA1/PPP3CA/FOS/JUN/TXNIP	10
BP	GO:0106106	cold-induced thermogenesis	10/432	147/18903	0.00206356857491071	0.0153922605150161	0.0105271676466853	EPAS1/CAV1/FABP5/ADRB2/G0S2/GJA1/ID1/FABP4/ARRDC3/DIO2	10
BP	GO:0120161	regulation of cold-induced thermogenesis	10/432	147/18903	0.00206356857491071	0.0153922605150161	0.0105271676466853	EPAS1/CAV1/FABP5/ADRB2/G0S2/GJA1/ID1/FABP4/ARRDC3/DIO2	10
BP	GO:1903038	negative regulation of leukocyte cell-cell adhesion	10/432	147/18903	0.00206356857491071	0.0153922605150161	0.0105271676466853	TNFRSF21/ASS1/CD74/IL20RB/RUNX1/PRNP/HLA-DRB1/LGALS3/WNK1/MDK	10
BP	GO:0098657	import into cell	14/432	252/18903	0.00206405133952325	0.0153922605150161	0.0105271676466853	SLC1A3/SLC7A11/SLC39A14/RGS4/PRNP/KCNJ15/SLC5A1/ATP1A1/WNK1/ARL6IP5/PPP3CA/SLC39A6/ATP1B1/SLC38A2	14
BP	GO:0048246	macrophage chemotaxis	5/432	40/18903	0.00207404932053032	0.0153922605150161	0.0105271676466853	SAA1/MMP28/LGALS3/CXCL17/MDK	5
BP	GO:0140467	integrated stress response signaling	5/432	40/18903	0.00207404932053032	0.0153922605150161	0.0105271676466853	MAF/HSPA5/PTPN1/FOS/JUN	5

BP	GO:1901186	positive regulation of ERBB signaling pathway	5/432	40/18903	0.00207404932053032	0.0153922605150161	0.0105271676466853	PLAUR/AREG/AFAP1L2/CCDC88A/HBEGF	5
BP	GO:0030282	bone mineralization	9/432	123/18903	0.00207614542475076	0.0153922605150161	0.0105271676466853	MMP13/BMP2/TGFB3/OMD/ADRB2/ANKH/HIF1A/ISG15/PTHLH	9
BP	GO:0001776	leukocyte homeostasis	8/432	100/18903	0.00207670714840433	0.0153922605150161	0.0105271676466853	SLC7A11/CXCL6/CD74/IL20RB/TSC22D3/HIF1A/SKIL/TNFAIP3	8
BP	GO:0010769	regulation of cell morphogenesis involved in differentiation	8/432	100/18903	0.00207670714840433	0.0153922605150161	0.0105271676466853	HAS2/DAB2/FBLN1/NEDD9/GBP1/TACSTD2/CUX1/MDK	8
BP	GO:0062012	regulation of small molecule metabolic process	17/432	339/18903	0.00216849561493299	0.0160468675505041	0.010974870438524	SLC7A11/NNMT/BMP2/DAB2/FMO2/PLEK/CD74/RDH10/CAV1/FABP5/IGFBP3/IL1B/APP/HIF1A/EGR1/NR3C1/STAT3	17
BP	GO:0072089	stem cell proliferation	9/432	124/18903	0.00219456943828	0.0162138716486342	0.011089088888568	TGFBR2/RUNX1/WNT5A/FBLN1/RUNX2/GJA1/PBX1/WNT2B/ZFP36L1	9
BP	GO:0001912	positive regulation of leukocyte mediated cytotoxicity	6/432	59/18903	0.00222944595159728	0.0163649495110338	0.0111924149714154	B2M/HLA-B/HLA-A/HLA-DRB1/HLA-DRA/HLA-E	6
BP	GO:0061098	positive regulation of protein tyrosine kinase activity	6/432	59/18903	0.00222944595159728	0.0163649495110338	0.0111924149714154	AREG/AFAP1L2/PRNP/NEDD9/HBEGF/PTPN1	6
BP	GO:0031953	negative regulation of protein	3/432	12/18903	0.00223624823588613	0.0163649495110338	0.0111924149714154	CAV1/ERRFI1/JUN	3

		autophosphorylation							
BP	GO:0033629	negative regulation of cell adhesion mediated by integrin	3/432	12/18903	0.00223624823588613	0.0163649495110338	0.0111924149714154	CYP1B1/WNK1/SERPINE1	3
BP	GO:0043380	regulation of memory T cell differentiation	3/432	12/18903	0.00223624823588613	0.0163649495110338	0.0111924149714154	HLA-DRB1/CD46/HLA-DRA	3
BP	GO:0043589	skin morphogenesis	3/432	12/18903	0.00223624823588613	0.0163649495110338	0.0111924149714154	ITGA6/ERRFI1/COL1A1	3
BP	GO:0043393	regulation of protein binding	12/432	200/18903	0.0022439659248742	0.0163954856280303	0.011213299416724	CSF3/B2M/BMP2/DAB2/IFIT1/LRRK2/WNT5A/CAV1/ADRB2/SLPI/APP/ID1	12
BP	GO:0046631	alpha-beta T cell activation	11/432	174/18903	0.00225175818661921	0.0164264694213152	0.0112344900394998	TGFBR2/RSAD2/RUNX1/PRDM1/HLA-A/HLA-DRB1/NFKBIZ/HLA-DRA/STAT3/HSPH1/HLA-E	11
BP	GO:0014912	negative regulation of smooth muscle cell migration	5/432	41/18903	0.00231847930535333	0.0167784300102349	0.0114752050482517	IGFBP3/IGFBP5/TRIB1/SERPINE1/SEMA6D	5
BP	GO:0032733	positive regulation of interleukin-10 production	5/432	41/18903	0.00231847930535333	0.0167784300102349	0.0114752050482517	CLEC7A/IL20RB/CD46/ISG15/STAT3	5
BP	GO:0043567	regulation of insulin-like growth factor	4/432	25/18903	0.00232539970520229	0.0167784300102349	0.0114752050482517	BMP2/IGFBP3/CDH3/IGFBP5	4

		receptor signaling pathway							
BP	GO:0060396	growth hormone receptor signaling pathway	4/432	25/18903	0.00232539970520229	0.0167784300102349	0.0114752050482517	JAK1/STAT3/PTPN1/PIK3R1	4
BP	GO:0071378	cellular response to growth hormone stimulus	4/432	25/18903	0.00232539970520229	0.0167784300102349	0.0114752050482517	JAK1/STAT3/PTPN1/PIK3R1	4
BP	GO:0086064	cell communication by electrical coupling involved in cardiac conduction	4/432	25/18903	0.00232539970520229	0.0167784300102349	0.0114752050482517	CAV1/ATP1A1/GJA1/ATP1B1	4
BP	GO:0098581	detection of external biotic stimulus	4/432	25/18903	0.00232539970520229	0.0167784300102349	0.0114752050482517	CLEC7A/HLA-B/HLA-A/HLA-DRB1	4
BP	GO:1901184	regulation of ERBB signaling pathway	7/432	80/18903	0.00234784035528679	0.0168876541884936	0.0115499063070096	PLAUR/AREG/AFAP1L2/IFI6/ERRFI1/CCDC88A/HBEGF	7
BP	GO:1903201	regulation of oxidative stress-induced cell death	7/432	80/18903	0.00234784035528679	0.0168876541884936	0.0115499063070096	SLC7A11/NCOA7/SOD2/LRRK2/VNN1/HIF1A/MCL1	7
BP	GO:0008630	intrinsic apoptotic signaling pathway in response to DNA damage	8/432	102/18903	0.00235287041266313	0.0168975553704456	0.0115566779830871	IFI16/SOD2/CD74/SHISA5/CLU/SKIL/MCL1/PIK3R1	8

BP	GO:0010821	regulation of mitochondrion organization	10/432	150/18903	0.00239350025358165	0.0171626956167676	0.0117380142994906	DCN/TNFSF10/GCLC/LRRK2/PLAUR/CLU/HIF1A/TFRC/MFF/HSPA1A	10
BP	GO:0051098	regulation of binding	18/432	372/18903	0.00239974271174813	0.0171808204981968	0.0117504103777141	CSF3/B2M/IFI16/BMP2/DAB2/IFIT1/LRRK2/PLAUR/WNT5A/CAV1/ADRB2/NFKBIA/SLPI/APP/SP100/ID1/PPP3CA/JUN	18
BP	GO:0006869	lipid transport	20/432	433/18903	0.0024273956398958	0.0173193945025337	0.0118451847465408	APOL1/DAB2/INHBA/SLC27A6/SELENOM/ITGAV/CAV1/IL1A/FABP5/PLSCR1/IL1B/ABCA1/NFKBIA/CLU/PNPLA8/NPC2/GM2A/FABP4/VMP1/CRABP2	20
BP	GO:0019748	secondary metabolic process	6/432	60/18903	0.00243033233127445	0.0173193945025337	0.0118451847465408	SLC7A11/CYP1B1/FMO2/WNT5A/CDH3/AKR1B1	6
BP	GO:1905517	macrophage migration	6/432	60/18903	0.00243033233127445	0.0173193945025337	0.0118451847465408	SAA1/MMP28/B4GALT1/LGALS3/CXCL17/MDK	6
BP	GO:0098659	inorganic cation import across plasma membrane	9/432	126/18903	0.0024475316755585	0.0173883778793519	0.0118923642736734	SLC39A14/PRNP/KCNJ15/SLC5A1/ATP1A1/WNK1/PPP3CA/SLC39A6/ATP1B1	9
BP	GO:0099587	inorganic ion import across plasma membrane	9/432	126/18903	0.0024475316755585	0.0173883778793519	0.0118923642736734	SLC39A14/PRNP/KCNJ15/SLC5A1/ATP1A1/WNK1/PPP3CA/SLC39A6/ATP1B1	9
BP	GO:0050680	negative regulation of epithelial cell proliferation	11/432	176/18903	0.0024616779361117	0.0174620559118353	0.0119427545980233	B2M/TGFB2/DAB2/STAT1/WNT5A/CAV1/MCC/B4GALT1/KLF9/SULF1/SPARC	11
BP	GO:0001763	morphogenesis of a branching structure	12/432	203/18903	0.00253744261980125	0.0179719327972141	0.0122914726726892	TGFBR2/BMP2/LRRK2/AREG/WNT5A/RDH10/PRDM1/TACSTD2/PBX1/SULF1/WNT2B/MDK	12

BP	GO:0070372	regulation of ERK1 and ERK2 cascade	16/432	315/18903	0.00254828363326036	0.0180211189660996	0.0123251123739697	BMP2/DAB2/PDGFD/INHBA/CD74/FBLN1/IL1A/IL1B/APP/HLA-DRB1/CCL20/GBP1/ERRFI1/CXCL17/PTPN1/JUN	16
BP	GO:0050691	regulation of defense response to virus by host	5/432	42/18903	0.00258320251199889	0.0182123652713337	0.0124559107005081	MMP12/STAT1/IL1B/TNFAIP3/HSP90AA1	5
BP	GO:2000403	positive regulation of lymphocyte migration	5/432	42/18903	0.00258320251199889	0.0182123652713337	0.0124559107005081	WNT5A/APP/NEDD9/CCL20/WNK1	5
BP	GO:0031669	cellular response to nutrient levels	13/432	231/18903	0.00263001983953709	0.0185142188095267	0.0126623561929167	CYP24A1/IFI16/LRRK2/DAPL1/XBP1/HSPA5/LAMP2/MIOS/SLC38A2/FOS/POSTN/WNT2B/COL1A1	13
BP	GO:1902882	regulation of response to oxidative stress	8/432	104/18903	0.00265703032469505	0.0186759350330009	0.0127729581278334	SLC7A11/NCOA7/SOD2/LRRK2/VNN1/HIF1A/PNPLA8/MCL1	8
BP	GO:0048736	appendage development	11/432	178/18903	0.00268715956153331	0.0187444541158781	0.0128198201122535	SLC7A11/TGFB2/PITX2/ITGA6/WNT5A/RDH10/RUNX2/ASPH/PBX1/MBNL1/CRABP2	11
BP	GO:0060173	limb development	11/432	178/18903	0.00268715956153331	0.0187444541158781	0.0128198201122535	SLC7A11/TGFB2/PITX2/ITGA6/WNT5A/RDH10/RUNX2/ASPH/PBX1/MBNL1/CRABP2	11
BP	GO:0002726	positive regulation of T cell cytokine production	4/432	26/18903	0.00269920139268645	0.0187444541158781	0.0128198201122535	B2M/RSAD2/IL1B/HLA-A	4
BP	GO:0035994	response to muscle stretch	4/432	26/18903	0.00269920139268645	0.0187444541158781	0.0128198201122535	CDH2/NFKBIA/FOS/JUN	4

BP	GO:0036119	response to platelet-derived growth factor	4/432	26/18903	0.00269920139268645	0.0187444541158781	0.0128198201122535	HAS2/PDGFD/ERRFI1/RDX	4
BP	GO:0060333	interferon-gamma-mediated signaling pathway	4/432	26/18903	0.00269920139268645	0.0187444541158781	0.0128198201122535	STAT1/IFNGR1/SP100/JAK1	4
BP	GO:0062009	secondary palate development	4/432	26/18903	0.00269920139268645	0.0187444541158781	0.0128198201122535	TGFB2/TGFBR2/TGFB3/WNT5A	4
BP	GO:1903901	negative regulation of viral life cycle	4/432	26/18903	0.00269920139268645	0.0187444541158781	0.0128198201122535	BST2/IFITM3/IFITM2/LY6E	4
BP	GO:0045471	response to ethanol	9/432	128/18903	0.00272310597711257	0.0188538400361462	0.0128946319905819	CLDN1/CSF3/S100A8/SOD2/RGS4/SLC23A2/BIRC2/FOS/NPPC	9
BP	GO:0048706	embryonic skeletal system development	9/432	128/18903	0.00272310597711257	0.0188538400361462	0.0128946319905819	TGFBR2/TGFB3/WNT5A/RDH10/RUNX2/SULF2/PBX1/SULF1/COL1A1	9
BP	GO:0019216	regulation of lipid metabolic process	17/432	347/18903	0.00275944655975651	0.0190768913884512	0.0130471826167387	BMP2/DAB2/FMO2/VAV3/CD74/ID2/RDH10/CAV1/IL1A/FABP5/IL1B/ATP1A1/NPC2/EGR1/CAPN2/NR3C1/IGFBP7	17
BP	GO:0050767	regulation of neurogenesis	18/432	377/18903	0.00276743238141859	0.0191035444239716	0.0130654113215442	BHLHE41/B2M/BMP2/BHLHE40/ID2/WNT5A/MME/SERPINE2/IL1B/IL6ST/HIF1A/ID1/SKIL/PPP3CA/SEMA6D/CUX1/CRABP2/MDK	18
BP	GO:0002706	regulation of lymphocyte mediated immunity	11/432	179/18903	0.00280599301662125	0.0192695042477944	0.0131789155652056	B2M/RSAD2/HLA-B/IL20RB/IL1B/HLA-A/HLA-DRB1/CD46/HLA-DRA/TFRC/HLA-E	11

BP	GO:0045580	regulation of T cell differentiation	11/432	179/18903	0.00280599301662125	0.0192695042477944	0.0131789155652056	TGFBR2/CD74/VNN1/RUNX1/PRDM1/HLA-DRB1/NFKBIZ/XBP1/CD46/HLA-DRA/MDK	11
BP	GO:0034976	response to endoplasmic reticulum stress	14/432	261/18903	0.00284286175942519	0.0192695042477944	0.0131789155652056	CXCL8/LRRK2/SRPX/CAV1/CLU/DNAJB9/XBP1/ATF6/HSPA5/DNAJC3/PTPN1/PIK3R1/JUN/HSPA1A	14
BP	GO:0010713	negative regulation of collagen metabolic process	3/432	13/18903	0.0028581361976188	0.0192695042477944	0.0131789155652056	ERRFI1/CST3/NPPC	3
BP	GO:0010745	negative regulation of macrophage derived foam cell differentiation	3/432	13/18903	0.0028581361976188	0.0192695042477944	0.0131789155652056	ITGAV/ABCA1/NFKBIA	3
BP	GO:0010771	negative regulation of cell morphogenesis involved in differentiation	3/432	13/18903	0.0028581361976188	0.0192695042477944	0.0131789155652056	FBLN1/GBP1/TACSTD2	3
BP	GO:0021681	cerebellar granular layer development	3/432	13/18903	0.0028581361976188	0.0192695042477944	0.0131789155652056	SERPINE2/MTPN/MDK	3
BP	GO:0031392	regulation of prostaglandin biosynthetic process	3/432	13/18903	0.0028581361976188	0.0192695042477944	0.0131789155652056	CD74/FABP5/IL1B	3

BP	GO:0034616	response to laminar fluid shear stress	3/432	13/18903	0.0028581361976188	0.0192695042477944	0.0131789155652056	ASS1/TGFB3/ABCA1	3
BP	GO:0043379	memory T cell differentiation	3/432	13/18903	0.0028581361976188	0.0192695042477944	0.0131789155652056	HLA-DRB1/CD46/HLA-DRA	3
BP	GO:0043568	positive regulation of insulin-like growth factor receptor signaling pathway	3/432	13/18903	0.0028581361976188	0.0192695042477944	0.0131789155652056	IGFBP3/CDH3/IGFBP5	3
BP	GO:0045916	negative regulation of complement activation	3/432	13/18903	0.0028581361976188	0.0192695042477944	0.0131789155652056	CD59/SERPING1/CD46	3
BP	GO:0071287	cellular response to manganese ion	3/432	13/18903	0.0028581361976188	0.0192695042477944	0.0131789155652056	LRRK2/APP/HSPA5	3
BP	GO:1900025	negative regulation of substrate adhesion-dependent cell spreading	3/432	13/18903	0.0028581361976188	0.0192695042477944	0.0131789155652056	FBLN1/GBP1/TACSTD2	3
BP	GO:1903651	positive regulation of cytoplasmic transport	3/432	13/18903	0.0028581361976188	0.0192695042477944	0.0131789155652056	DAB2/MSN/RDX	3

BP	GO:2001198	regulation of dendritic cell differentiation	3/432	13/18903	0.0028581361976188	0.0192695042477944	0.0131789155652056	TMEM176A/TMEM176B/HLA-B	3
BP	GO:0045687	positive regulation of glial cell differentiation	5/432	43/18903	0.00286916731103392	0.0192824928475873	0.01318779880 6714	BMP2/ID2/SERPINE2/IL6ST/MDK	5
BP	GO:2000027	regulation of animal organ morphogenesis	9/432	129/18903	0.00286980399608173	0.0192824928475873	0.013187798806714	BMP2/GPC6/DAB2/WNT5A/RUNX2/TACSTD2/CITED2/SULF1/WNT2B	9
BP	GO:0090303	positive regulation of wound healing	6/432	62/18903	0.00287257028583517	0.0192824928475873	0.013187798806714	CLDN1/CLEC7A/XBP1/PLAU/SERPINE1/HBEGF	6
BP	GO:0030500	regulation of bone mineralization	7/432	83/18903	0.00289506834429806	0.0193962626602303	0.0132656089431561	BMP2/TGFB3/OMD/ADRB2/ANKH/HIF1A/ISG15	7
BP	GO:0043524	negative regulation of neuron apoptotic process	10/432	154/18903	0.00289790648610143	0.0193962626602303	0.0132656089431561	GCLC/SOD2/TGFB3/GRN/NR4A2/IL6ST/HIF1A/CHL1/JUN/MDK	10
BP	GO:0031668	cellular response to extracellular stimulus	14/432	262/18903	0.0029425939637244	0.0196669032980135	0.0134507071204622	CYP24A1/IFI16/LRRK2/DAPL1/NR4A2/XBP1/HSPA5/LAMP2/MIOS/SLC38A2/FOS/POSTN/WNT2B/COL1A1	14
BP	GO:0034284	response to monosaccharide	12/432	207/18903	0.00297662985846611	0.0198656754623459	0.0135866525779458	TGFBR2/GCLC/SOD2/SLC39A14/IL1A/HLA-DRB1/RUNX2/HIF1A/XBP1/EGR1/PPP3CA/TXNIP	12
BP	GO:0006958	complement activation, classical pathway	8/432	106/18903	0.0029910763521712	0.019933325833994	0.0136329204281427	C1S/C1R/CLU/SERPING1/CD46/IGHG3/IGKC/IGHG4	8

BP	GO:0010632	regulation of epithelial cell migration	15/432	292/18903	0.0030835137824104	0.0205197859620836	0.0140340157760595	DCN/TGFB2/HAS2/TGFBR2/RIN2/WNT5A/GRN/MCC/HIF1A/SP100/TACSTD2/GADD45A/HBEGF/JUN/SPARC	15
BP	GO:0032967	positive regulation of collagen biosynthetic process	4/432	27/18903	0.00311217911492556	0.0206107557737573	0.0140962323983251	INHBA/TGFB3/RUNX1/VIM	4
BP	GO:0071280	cellular response to copper ion	4/432	27/18903	0.00311217911492556	0.0206107557737573	0.0140962323983251	PRNP/APP/MT2A/MT1E	4
BP	GO:0032731	positive regulation of interleukin-1 beta production	6/432	63/18903	0.00311500935910408	0.0206107557737573	0.0140962323983251	CLEC7A/IFI16/WNT5A/APP/EGR1/STAT3	6
BP	GO:2001244	positive regulation of intrinsic apoptotic signaling pathway	6/432	63/18903	0.00311500935910408	0.0206107557737573	0.0140962323983251	S100A9/S100A8/VNN1/CAV1/SKIL/MCL1	6
BP	GO:0010717	regulation of epithelial to mesenchymal transition	8/432	107/18903	0.00316991048317821	0.0209440514067132	0.0143241819578203	TGFB2/TGFBR2/BMP2/DAB2/TGFB3/IL1B/MDK/COL1A1	8
BP	GO:0030900	forebrain development	18/432	383/18903	0.00326964588454147	0.0215722000228306	0.0147537891479047	B2M/SLC7A11/PITX2/BMP2/LRRK2/INHBA/ID2/LAMB1/WNT5A/CDH2/NR4A2/UBA6/APP/HIF1A/SLC38A2/WNT2B/MDK/DNAJB1	18
BP	GO:0008637	apoptotic mitochondrial changes	8/432	108/18903	0.00335694647491589	0.0219990432415687	0.0150457183364811	TNFSF10/GCLC/SOD2/PLAUR/IFI6/CLU/MFF/HSPA1A	8

BP	GO:0034308	primary alcohol metabolic process	8/432	108/18903	0.00335694647491589	0.0219990432415687	0.0150457183364811	BMP2/CYP1B1/DAB2/SDR16C5/RDH10/DHRS3/GPD2/AKR1B1	8
BP	GO:0015849	organic acid transport	16/432	324/18903	0.00336012942366926	0.0219990432415687	0.0150457183364811	SLC1A3/SLC7A11/SLC27A6/RGS4/IL1A/SLC23A2/FABP5/IL1B/LRRC8D/SLC16A1/GJA1/PNPLA8/ARL6IP5/FABP4/SLC38A2/CRABP2	16
BP	GO:1902903	regulation of supramolecular fiber organization	18/432	384/18903	0.00336029373256683	0.0219990432415687	0.0150457183364811	CSF3/PLEK/TGFB3/CRYAB/CD47/CGNL1/RGS4/CDC42EP3/CLU/APP/ID1/TACSTD2/SVIL/CCDC88A/MTPN/RDX/PIK3R1/HSPA1A	18
BP	GO:0050730	regulation of peptidyl-tyrosine phosphorylation	14/432	266/18903	0.00337066998125635	0.0219990432415687	0.0150457183364811	CSF3/SAMSN1/PDGFD/CD74/AREG/AFAP1L2/CAV1/PRNP/IL6ST/APP/NEDD9/ERRFI1/HBEGF/PTPN1	14
BP	GO:0098656	anion transmembrane transport	14/432	266/18903	0.00337066998125635	0.0219990432415687	0.0150457183364811	SLC1A3/SLC7A11/APOL1/CLCA4/RGS4/FXYD3/SLC23A2/PRNP/ANKH/LRRC8D/SLC16A1/GJA1/ARL6IP5/SLC38A2	14
BP	GO:0030199	collagen fibril organization	6/432	64/18903	0.00337239387205885	0.0219990432415687	0.0150457183364811	TGFB2/LUM/CYP1B1/COL12A1/COL14A1/COL1A1	6
BP	GO:0048524	positive regulation of viral process	6/432	64/18903	0.00337239387205885	0.0219990432415687	0.0150457183364811	IFIT1/CD74/STOM/HLA-DRB1/SRPK2/LGALS1	6
BP	GO:0042886	amide transport	15/432	296/18903	0.00350150679290361	0.0228091111509566	0.0155997448668278	SLC7A11/S100A8/CPE/CD74/TAPBP/IL1B/IL1RN/ABCA1/SLC16A1/HLA-DRB1/HIF1A/GJA1/PPP3CA/TM7SF3/CA2	15
BP	GO:1900744	regulation of p38MAPK cascade	5/432	45/18903	0.00350862020864367	0.02281498768636	0.015603763982364	BMP2/DUSP1/IL1B/GADD45A/GADD45B	5

BP	GO:0032092	positive regulation of protein binding	7/432	86/18903	0.00353502228145154	0.02281498768636	0.015603763982364	CSF3/B2M/BMP2/LRRK2/WNT5A/CAV1/APP	7
BP	GO:0033273	response to vitamin	7/432	86/18903	0.00353502228145154	0.02281498768636	0.015603763982364	CYP24A1/KYNU/SOD2/IL1A/RUNX2/POSTN/COL1A1	7
BP	GO:0042116	macrophage activation	8/432	109/18903	0.00355243354413061	0.02281498768636	0.015603763982364	LRRK2/CD74/WNT5A/GRN/CLU/IFNGR1/APP/JUN	8
BP	GO:0010714	positive regulation of collagen metabolic process	4/432	28/18903	0.0035662445762177	0.02281498768636	0.015603763982364	INHBA/TGFB3/RUNX1/VIM	4
BP	GO:1903649	regulation of cytoplasmic transport	4/432	28/18903	0.0035662445762177	0.02281498768636	0.015603763982364	DAB2/LRRK2/MSN/RDX	4
BP	GO:1904385	cellular response to angiotensin	4/432	28/18903	0.0035662445762177	0.02281498768636	0.015603763982364	INHBA/CAV1/AGTRAP/CA2	4
BP	GO:0030007	cellular potassium ion homeostasis	3/432	14/18903	0.00357640347515914	0.02281498768636	0.015603763982364	ATP1A1/KCNMA1/ATP1B1	3
BP	GO:0046598	positive regulation of viral entry into host cell	3/432	14/18903	0.00357640347515914	0.02281498768636	0.015603763982364	CD74/HLA-DRB1/LGALS1	3
BP	GO:0061043	regulation of vascular wound healing	3/432	14/18903	0.00357640347515914	0.02281498768636	0.015603763982364	XBP1/TNFAIP3/SERPINE1	3

BP	GO: 0070 141	response to UV-A	3/432	14/18903	0.00357 640347 515914	0.02281 498768 636	0.01560 376398 2364	MMP1/MME/TIMP1	3
BP	GO: 0075 294	positive regulation by symbiont of entry into host	3/432	14/18903	0.00357 640347 515914	0.02281 498768 636	0.01560 376398 2364	CD74/HLA-DRB1/LGALS1	3
BP	GO: 0090 715	immunological memory formation process	3/432	14/18903	0.00357 640347 515914	0.02281 498768 636	0.01560 376398 2364	HLA-DRB1/CD46/HLA-DRA	3
BP	GO: 1902 947	regulation of tau-protein kinase activity	3/432	14/18903	0.00357 640347 515914	0.02281 498768 636	0.01560 376398 2364	CLU/EGR1/HSP90AA1	3
BP	GO: 1903 894	regulation of IRE1-mediated unfolded protein response	3/432	14/18903	0.00357 640347 515914	0.02281 498768 636	0.01560 376398 2364	DNAJB9/HSPA5/PTPN1	3
BP	GO: 0015 800	acidic amino acid transport	6/432	65/18903	0.00364 527724 901517	0.02315 852647 89769	0.01583 871910 53941	SLC1A3/SLC7A11/LRRC8D/GJA1/ARL6IP5/SLC38A2	6
BP	GO: 0048 247	lymphocyte chemotaxis	6/432	65/18903	0.00364 527724 901517	0.02315 852647 89769	0.01583 871910 53941	SAA1/WNT5A/CXCL16/NEDD9/CCL20/WNK1	6
BP	GO: 1905 330	regulation of morphogenesis of an epithelium	6/432	65/18903	0.00364 527724 901517	0.02315 852647 89769	0.01583 871910 53941	WNT5A/GJA1/TACSTD2/SULF1/WNT2B/MDK	6
BP	GO: 0001 523	retinoid metabolic process	7/432	87/18903	0.00377 060472 507729	0.02382 383449 9293	0.01629 374057 93173	CYP1B1/SDR16C5/RDH10/DHRS3/SCPEP1/AKR1B1/CRABP2	7

BP	GO:0001910	regulation of leukocyte mediated cytotoxicity	7/432	87/18903	0.00377060472507729	0.023823834499293	0.0162937405793173	B2M/CXCL6/HLA-B/HLA-A/HLA-DRB1/HLA-DRA/HLA-E	7
BP	GO:0019915	lipid storage	7/432	87/18903	0.00377060472507729	0.023823834499293	0.0162937405793173	ITGAV/CAV1/IL1B/ABCA1/NFKBIA/GM2A/HILPDA	7
BP	GO:0048145	regulation of fibroblast proliferation	7/432	87/18903	0.00377060472507729	0.023823834499293	0.0162937405793173	SOD2/PDGFD/CD74/WNT5A/CAV1/FOSL2/JUN	7
BP	GO:1990845	adaptive thermogenesis	10/432	160/18903	0.00381007983097935	0.0240404082104768	0.01644186097809	EPAS1/CAV1/FABP5/ADRB2/G0S2/GJA1/ID1/FABP4/ARRDC3/DIO2	10
BP	GO:0002920	regulation of humoral immune response	5/432	46/18903	0.00386400527237672	0.0243474446658615	0.0166518512024839	CFH/IL1B/CD59/SERPING1/CD46	5
BP	GO:0010876	lipid localization	21/432	483/18903	0.00388385971325055	0.0244195153262845	0.0167011422032398	APOL1/DAB2/INHBA/SLC27A6/SELENOM/ITGAV/CAV1/IL1A/FABP5/PLSCR1/IL1B/ABCA1/NFKBIA/CLU/PNPLA8/NPC2/GM2A/FABP4/VMP1/HILPDA/CRABP2	21
BP	GO:0035270	endocrine system development	9/432	135/18903	0.00388600287138279	0.0244195153262845	0.0167011422032398	PITX2/BMP2/CYP1B1/WNT5A/CDH2/CITED2/NR3C1/PBX1/MDK	9
BP	GO:0030324	lung development	11/432	187/18903	0.00391703055857551	0.0245477863596365	0.0167888701020103	MMP12/SLC7A11/EPAS1/TGFBR2/TGFB3/WNT5A/MME/RDH10/IGFBP5/ERRFI1/WNT2B	11
BP	GO:0048469	cell maturation	11/432	187/18903	0.00391703055857551	0.0245477863596365	0.0167888701020103	CLEC7A/NRCAM/EPAS1/LRRK2/NR4A2/APP/RUNX2/HIF1A/PTBP3/AKR1B1/NPPC	11

BP	GO:0009314	response to radiation	20/432	452/18903	0.00393731687852397	0.0246415298554443	0.0168529837190578	SLC1A3/SLC7A11/IFI16/MMP1/SOD2/BHLHE40/CRYAB/ID2/MME/IL1A/PRDM1/N4BP1/APP/HIF1A/EGR1/GADD45A/HSPA5/TIMP1/PIK3R1/FOS	20
BP	GO:0032611	interleukin-1 beta production	8/432	111/18903	0.00396977042916824	0.024777581963432	0.016946033297323 1	CLEC7A/IFI16/WNT5A/APP/EGR1/TNFAIP3/STAT3/ERRFI1	8
BP	GO:0032651	regulation of interleukin-1 beta production	8/432	111/18903	0.00396977042916824	0.024777581963432	0.0169460332973231	CLEC7A/IFI16/WNT5A/APP/EGR1/TNFAIP3/STAT3/ERRFI1	8
BP	GO:0033627	cell adhesion mediated by integrin	7/432	88/18903	0.00401792242337367	0.0250106207376894	0.0171054145813386	TGFB2/CYP1B1/ITGA6/ITGAV/WNK1/PLAU/SERPINE1	7
BP	GO:0055072	iron ion homeostasis	7/432	88/18903	0.00401792242337367	0.0250106207376894	0.0171054145813386	B2M/EPAS1/SOD2/SLC39A14/HEPHL1/HIF1A/TFRC	7
BP	GO:0097066	response to thyroid hormone	4/432	29/18903	0.00406326619381721	0.0252249746931606	0.0172520168313963	GCLC/CTSB/KLF9/RDX	4
BP	GO:1902175	regulation of oxidative stress-induced intrinsic apoptotic signaling pathway	4/432	29/18903	0.00406326619381721	0.0252249746931606	0.0172520168313963	SOD2/VNN1/HIF1A/MCL1	4
BP	GO:0045444	fat cell differentiation	13/432	244/18903	0.00419113129742659	0.0259838904163512	0.01777105905 0045	LAMB3/BMP2/SOD2/ID2/WNT5A/ADRB2/NR4A2/XBP1/PSMB8/FABP4/ZFP36/ZFP36L1/DIO2	13
BP	GO:0046677	response to antibiotic	5/432	47/18903	0.00424442273681661	0.0262088853908903	0.0179249389700427	SLC1A3/GJB2/JAK1/HSPA5/HSP90AA1	5

BP	GO:0048286	lung alveolus development	5/432	47/18903	0.00424442273681661	0.0262088853908903	0.0179249389700427	MMP12/SLC7A11/TGFB3/IGFBP5/ERRFI1	5
BP	GO:0140353	lipid export from cell	5/432	47/18903	0.00424442273681661	0.0262088853908903	0.0179249389700427	DAB2/INHBA/SELENOM/IL1A/IL1B	5
BP	GO:0001701	in utero embryonic development	18/432	393/18903	0.00427407443563501	0.0263567923530825	0.0180260963916957	EPAS1/TGFBR2/BMP2/TGFB3/LPAR6/RDH10/MED21/PRDM1/RBBP8/HIF1A/SKIL/SMIM14/CITED2/ZFAND5/CAPN2/KRT19/MBNL1/ZFP36L1	18
BP	GO:0051960	regulation of nervous system development	20/432	456/18903	0.0043387600106455	0.0265385042377653	0.0181503738798259	BHLHE41/TNFRSF21/B2M/BMP2/TYMP/BHLHE40/ID2/WNT5A/MME/SERPINE2/IL1B/IL6ST/HIF1A/ID1/SKIL/PPP3CA/SEMA6D/CUX1/CRABP2/MDK	20
BP	GO:0002440	production of molecular mediator of immune response	16/432	333/18903	0.00437443042011525	0.0265385042377653	0.0181503738798259	B2M/CLEC7A/TGFB2/BST2/RSAD2/TGFB3/CD74/WNT5A/IL1B/HLA-A/HLA-DRB1/DNAJB9/XBP1/HLA-DRA/TFRC/HLA-E	16
BP	GO:0060249	anatomical structure homeostasis	16/432	333/18903	0.00437443042011525	0.0265385042377653	0.0181503738798259	CLDN1/B2M/EPAS1/IL20RB/CAV1/ADRB2/CDH3/APP/HIF1A/GJA1/TNFAIP3/IGHG3/IGKC/TFRC/LAMP2/CA2	16
BP	GO:0001771	immunological synapse formation	3/432	15/18903	0.00439535010727097	0.0265385042377653	0.0181503738798259	NEDD9/LGALS3/MSN	3
BP	GO:0002468	dendritic cell antigen processing and presentation	3/432	15/18903	0.00439535010727097	0.0265385042377653	0.0181503738798259	CD74/HLA-DRB1/HLA-DRA	3
BP	GO:0035358	regulation of peroxisome proliferator	3/432	15/18903	0.00439535010727097	0.0265385042377653	0.0181503738798259	BMP2/FABP5/CITED2	3

		activated receptor signaling pathway							
BP	GO: 0035672	oligopeptide transmembrane transport	3/432	15/18903	0.00439535010727097	0.0265385042377653	0.0181503738798259	SLC7A11/GJA1/CA2	3
BP	GO: 0038166	angiotensin-activated signaling pathway	3/432	15/18903	0.00439535010727097	0.0265385042377653	0.0181503738798259	CAV1/AGTRAP/CA2	3
BP	GO: 0043455	regulation of secondary metabolic process	3/432	15/18903	0.00439535010727097	0.0265385042377653	0.0181503738798259	SLC7A11/WNT5A/CDH3	3
BP	GO: 0048021	regulation of melanin biosynthetic process	3/432	15/18903	0.00439535010727097	0.0265385042377653	0.0181503738798259	SLC7A11/WNT5A/CDH3	3
BP	GO: 0048820	hair follicle maturation	3/432	15/18903	0.00439535010727097	0.0265385042377653	0.0181503738798259	TGFB2/WNT5A/CDH3	3
BP	GO: 0060576	intestinal epithelial cell development	3/432	15/18903	0.00439535010727097	0.0265385042377653	0.0181503738798259	PRDM1/IL6ST/HIF1A	3
BP	GO: 1900102	negative regulation of endoplasmic reticulum unfolded protein response	3/432	15/18903	0.00439535010727097	0.0265385042377653	0.0181503738798259	DNAJB9/HSPA5/PTPN1	3

BP	GO:1900376	regulation of secondary metabolite biosynthetic process	3/432	15/18903	0.00439535010727097	0.0265385042377653	0.0181503738798259	SLC7A11/WNT5A/CDH3	3
BP	GO:2000345	regulation of hepatocyte proliferation	3/432	15/18903	0.00439535010727097	0.0265385042377653	0.0181503738798259	XBP1/TNFAIP3/MDK	3
BP	GO:2001279	regulation of unsaturated fatty acid biosynthetic process	3/432	15/18903	0.00439535010727097	0.0265385042377653	0.0181503738798259	CD74/FABP5/IL1B	3
BP	GO:0051047	positive regulation of secretion	15/432	304/18903	0.00447728500673129	0.0269979702166	0.0184646146232451	TGFB2/S100A8/BMP2/DAB2/INHBA/TGFB3/MYH10/IL1A/IL1B/HLA-DRB1/HIF1A/XBP1/PPP3CA/TM7SF3/TMED10	15
BP	GO:0016101	diterpenoid metabolic process	7/432	90/18903	0.00454922950037337	0.0273960760927433	0.0187368895951508	CYP1B1/SDR16C5/RDH10/DHRS3/SCPEP1/AKR1B1/CRABP2	7
BP	GO:0002704	negative regulation of leukocyte mediated immunity	6/432	68/18903	0.00456249322224901	0.0274402225655418	0.0187670825170875	BST2/HLA-B/IL20RB/HLA-A/CD46/HLA-E	6
BP	GO:0030323	respiratory tube development	11/432	191/18903	0.00459110895088314	0.0275173467991809	0.0188198297881169	MMP12/SLC7A11/EPAS1/TGFBR2/TGFB3/WNT5A/MME/RDH10/IGFBP5/ERRFI1/WNT2B	11
BP	GO:0007176	regulation of epidermal growth	4/432	30/18903	0.00460506517244671	0.0275173467991809	0.0188198297881169	AREG/APP/ERRFI1/HBEGF	4

		factor-activated receptor activity							
BP	GO:0009651	response to salt stress	4/432	30/18903	0.00460506517244671	0.0275173467991809	0.0188198297881169	CLDN1/AKR1B1/ZFP36L1/HSP90AA1	4
BP	GO:0060325	face morphogenesis	4/432	30/18903	0.00460506517244671	0.0275173467991809	0.0188198297881169	TGFB3/TIPARP/ASPH/COL1A1	4
BP	GO:1903203	regulation of oxidative stress-induced neuron death	4/432	30/18903	0.00460506517244671	0.0275173467991809	0.0188198297881169	SLC7A11/NCOA7/HIF1A/MCL1	4
BP	GO:0046189	phenol-containing compound biosynthetic process	5/432	48/18903	0.00465081183540918	0.0277548448242161	0.0189822608698195	SLC7A11/TGFB2/WNT5A/CDH3/NR4A2	5
BP	GO:0030004	cellular monovalent inorganic cation homeostasis	8/432	114/18903	0.00466552925922876	0.0278067948762023	0.0190177908627	SLC1A3/LRRK2/GRN/IL1A/ATP1A1/KCNMA1/ATP1B1/CA2	8
BP	GO:0032386	regulation of intracellular transport	16/432	336/18903	0.00476357060025743	0.0283545869062942	0.0193924400918155	DAB2/LRRK2/CRYAB/PRNP/IL1B/PTPN14/STOM/SP100/MSN/LMAN1/PTPN1/RDX/MFF/PIK3R1/IFI27/HSP90AA1	16
BP	GO:0042593	glucose homeostasis	13/432	248/18903	0.00479917772539565	0.0285298161696078	0.0195122839464402	GCLC/SLC39A14/FABP5/LRRC8D/SLC16A1/HLA-DRB1/IGFBP5/HIF1A/XBP1/PYGL/STAT3/PPP3CA/PIK3R1	13
BP	GO:1990778	protein localization to cell periphery	16/432	337/18903	0.00489939397263353	0.028923672505447	0.0197816525471108	GPC6/DAB2/RAB31/CDH2/CAV1/PLS1/PRNP/OPTN/GBP1/LGALS3/ARL6IP5/TMEM59/CCDC88A/RDX/PIK3R1/ATP1B1	16

BP	GO:0002260	lymphocyte homeostasis	6/432	69/18903	0.00490295335011253	0.028923672505447	0.0197816525471108	CD74/IL20RB/TSC22D3/HIF1A/SKIL/TNFAIP3	6
BP	GO:0014015	positive regulation of gliogenesis	6/432	69/18903	0.00490295335011253	0.028923672505447	0.0197816525471108	BMP2/ID2/SERPINE2/IL1B/IL6ST/MDK	6
BP	GO:0032835	glomerulus development	6/432	69/18903	0.00490295335011253	0.028923672505447	0.0197816525471108	PDGFD/BASP1/EGR1/SULF2/PPP3CA/SULF1	6
BP	GO:0061180	mammary gland epithelium development	6/432	69/18903	0.00490295335011253	0.028923672505447	0.0197816525471108	ID2/AREG/WNT5A/HIF1A/NR3C1/SOSTDC1	6
BP	GO:2000401	regulation of lymphocyte migration	6/432	69/18903	0.00490295335011253	0.028923672505447	0.0197816525471108	WNT5A/APP/NEDD9/CCL20/WNK1/MSN	6
BP	GO:0033500	carbohydrate homeostasis	13/432	249/18903	0.00496176455398216	0.0292333261938439	0.0199934327652918	GCLC/SLC39A14/FABP5/LRRC8D/SLC16A1/HLA-DRB1/IGFBP5/HIF1A/XBP1/PYGL/STAT3/PPP3CA/PIK3R1	13
BP	GO:0110053	regulation of actin filament organization	14/432	278/18903	0.00496927154305553	0.0292403064715417	0.019998206758915	CSF3/PLEK/TGFB3/CD47/CGNL1/RGS4/CDC42EP3/ID1/TACSTD2/SVIL/CCDC88A/MTPN/RDX/PIK3R1	14
BP	GO:0033628	regulation of cell adhesion mediated by integrin	5/432	49/18903	0.00508410543829421	0.0298780020992512	0.0204343433987482	TGFB2/CYP1B1/WNK1/PLAU/SERPINE1	5
BP	GO:0002028	regulation of sodium ion transport	7/432	92/18903	0.00513189717909745	0.0300619904265327	0.0205601778052304	FXYD3/SERPINE2/ADRB2/ATP1A1/STOM/WNK1/ATP1B1	7

BP	GO:0072091	regulation of stem cell proliferation	7/432	92/18903	0.00513189717909745	0.0300619904265327	0.0205601778052304	TGFBR2/WNT5A/FBLN1/RUNX2/GJA1/PBX1/ZFP36L1	7
BP	GO:0001959	regulation of cytokine-mediated signaling pathway	10/432	167/18903	0.00514675654136561	0.0300619904265327	0.0205601778052304	MMP12/CD74/WNT5A/CAV1/IL1RN/HIF1A/ISG15/TNFAIP3/PTPN1/HSPA1A	10
BP	GO:0030509	BMP signaling pathway	10/432	167/18903	0.00514675654136561	0.0300619904265327	0.0205601778052304	TGFB2/BMP2/TGFB3/HTRA1/WNT5A/RUNX2/SKIL/EGR1/SULF1/SOSTDC1	10
BP	GO:0031345	negative regulation of cell projection organization	11/432	194/18903	0.00515466879417604	0.0300619904265327	0.0205601778052304	B2M/DAB2/LRRK2/WNT5A/PRNP/ID1/TACSTD2/VIM/PPP3CA/SEMA6D/MAP4	11
BP	GO:0090090	negative regulation of canonical Wnt signaling pathway	9/432	141/18903	0.00516442825241713	0.0300619904265327	0.0205601778052304	BMP2/DAB2/WNT5A/CDH2/CAV1/MCC/EGR1/MDK/SOSTDC1	9
BP	GO:0002065	columnar/cuboidal epithelial cell differentiation	8/432	116/18903	0.0051789111981314	0.0300619904265327	0.0205601778052304	BMP2/WNT5A/CDH2/CAV1/PRDM1/IL6ST/HIF1A/SERPINE1	8
BP	GO:0098586	cellular response to virus	8/432	116/18903	0.0051789111981314	0.0300619904265327	0.0205601778052304	MMP12/BIRC3/IFI6/IFNGR1/BIRC2/HIF1A/JAK1/HSP90AA1	8
BP	GO:0002507	tolerance induction	4/432	31/18903	0.00519341196774047	0.0300619904265327	0.0205601778052304	TGFBR2/HLA-B/TNFAIP3/HLA-E	4

BP	GO: 0060055	angiogenesis involved in wound healing	4/432	31/18903	0.00519341196774047	0.0300619904265327	0.0205601778052304	B4GALT1/XBP1/TNFAIP3/SERPINE1	4
BP	GO: 0060603	mammary gland duct morphogenesis	4/432	31/18903	0.00519341196774047	0.0300619904265327	0.0205601778052304	AREG/WNT5A/NR3C1/SOSTDC1	4
BP	GO: 1900745	positive regulation of p38MAPK cascade	4/432	31/18903	0.00519341196774047	0.0300619904265327	0.0205601778052304	BMP2/IL1B/GADD45A/GADD45B	4
BP	GO: 0051881	regulation of mitochondrial membrane potential	6/432	70/18903	0.00526170954927867	0.0304192583317673	0.0208045226257663	DCN/GCLC/SOD2/LRRK2/IFI6/ARL6IP5	6
BP	GO: 0002399	MHC class II protein complex assembly	3/432	16/18903	0.00531882629985676	0.0304450144020266	0.0208221378726805	B2M/HLA-DRB1/HLA-DRA	3
BP	GO: 0002503	peptide antigen assembly with MHC class II protein complex	3/432	16/18903	0.00531882629985676	0.0304450144020266	0.0208221378726805	B2M/HLA-DRB1/HLA-DRA	3
BP	GO: 0006704	glucocorticoid biosynthetic process	3/432	16/18903	0.00531882629985676	0.0304450144020266	0.0208221378726805	BMP2/ATP1A1/NR3C1	3
BP	GO: 0006878	cellular copper ion homeostasis	3/432	16/18903	0.00531882629985676	0.0304450144020266	0.0208221378726805	PRNP/APP/MT2A	3
BP	GO: 0034112	positive regulation of homotypic cell-cell adhesion	3/432	16/18903	0.00531882629985676	0.0304450144020266	0.0208221378726805	PLAUR/IL6ST/JAK1	3

BP	GO:0061684	chaperone-mediated autophagy	3/432	16/18903	0.00531882629985676	0.0304450144020266	0.0208221378726805	CLU/LAMP2/HSP90AA1	3
BP	GO:0070431	nucleotide-binding oligomerization domain containing 2 signaling pathway	3/432	16/18903	0.00531882629985676	0.0304450144020266	0.0208221378726805	NFKBIA/TNFAIP3/HSPA1A	3
BP	GO:1905906	regulation of amyloid fibril formation	3/432	16/18903	0.00531882629985676	0.0304450144020266	0.0208221378726805	CRYAB/CLU/APP	3
BP	GO:0060070	canonical Wnt signaling pathway	15/432	310/18903	0.00534582485636087	0.0305617304829036	0.020901963038663	BMP2/DAB2/LRRK2/WNT5A/CDH2/CAV1/CDH3/MCC/NR4A2/WNK1/EGR1/WNT2B/MDK/SOSTDC1/COL1A1	15
BP	GO:0032872	regulation of stress-activated MAPK cascade	11/432	195/18903	0.00535421646903009	0.0305719150237829	0.0209089285112871	TGFB2/BMP2/WNT5A/DUSP1/IL1A/IL1B/APP/ARL6IP5/TPD52L1/GADD45A/GADD45B	11
BP	GO:0006835	dicarboxylic acid transport	7/432	93/18903	0.00544342238645168	0.0309665787667146	0.0211788493186862	SLC1A3/SLC7A11/LRRC8D/SLC16A1/GJA1/ARL6IP5/SLC38A2	7
BP	GO:0044070	regulation of anion transport	7/432	93/18903	0.00544342238645168	0.0309665787667146	0.0211788493186862	RGS4/IL1A/PRNP/IL1B/ARL6IP5/CA2/SLC38A2	7
BP	GO:0051781	positive regulation of cell division	7/432	93/18903	0.00544342238645168	0.0309665787667146	0.0211788493186862	TGFB2/PDGFD/TGFB3/IL1A/IL1B/SVIL/MDK	7

BP	GO:0046632	alpha-beta T cell differentiation	8/432	117/18903	0.00545125779038022	0.0309730556271603	0.0211832790121461	TGFBR2/RSAD2/RUNX1/PRDM1/HLA-DRB1/NFKBIZ/HLA-DRA/STAT3	8
BP	GO:0044089	positive regulation of cellular component biogenesis	21/432	498/18903	0.00548044187756468	0.0311006670966094	0.0212705558179994	CLDN1/CSF3/CLEC7A/MMP1/PLEK/TGFB3/CD47/CAV1/CDC42EP3/ABCA1/MPP7/CLU/ID1/LGALS3/MSN/ISG15/TFRC/CCDC88A/PIK3R1/HSP90AA1/HSPA1A	21
BP	GO:0002066	columnar/cuboidal epithelial cell development	5/432	50/18903	0.00554522864209152	0.0312764420361869	0.0213907728748203	WNT5A/CDH2/PRDM1/IL6ST/HIF1A	5
BP	GO:0006692	prostanoid metabolic process	5/432	50/18903	0.00554522864209152	0.0312764420361869	0.0213907728748203	CD74/FABP5/IL1B/PNPLA8/AKR1B1	5
BP	GO:0006693	prostaglandin metabolic process	5/432	50/18903	0.00554522864209152	0.0312764420361869	0.0213907728748203	CD74/FABP5/IL1B/PNPLA8/AKR1B1	5
BP	GO:0089718	amino acid import across plasma membrane	5/432	50/18903	0.00554522864209152	0.0312764420361869	0.0213907728748203	SLC1A3/SLC7A11/RGS4/ARL6IP5/SLC38A2	5
BP	GO:1902041	regulation of extrinsic apoptotic signaling pathway via death domain receptors	5/432	50/18903	0.00554522864209152	0.0312764420361869	0.0213907728748203	SP100/LGALS3/SKIL/TNFAIP3/SERPINE1	5
BP	GO:0030003	cellular cation homeostasis	21/432	499/18903	0.00560374675471959	0.031568000902044	0.0215901775728346	SLC1A3/S100A9/S100A8/LRRK2/SLC39A14/HEPHL1/WNT5A/GRN/CAV1/IL1A/PRNP/ATP1A1/APP/HIF1A/TFRC/KCNMA1/SLC39A6/ATP1B1/CA2/MT2A/MT1E	21

BP	GO:2001056	positive regulation of cysteine-type endopeptidase activity	9/432	143/18903	0.00565604923008547	0.031790862765758	0.0217425986028115	TNFSF10/CLEC7A/IFI16/S100A9/S100A8/ARL6IP5/CTSD/ASPH/UACA	9
BP	GO:0002429	immune response-activating cell surface receptor signaling pathway	15/432	312/18903	0.00566392884734803	0.031790862765758	0.0217425986028115	TNFRSF21/CLEC7A/VAV3/CD47/PRNP/PLSCR1/HLA-A/HLA-DRB1/NFKBIZ/GBP1/LGALS3/WNK1/IGHG3/IGKC/IGHG4	15
BP	GO:0002757	immune response-activating signal transduction	15/432	312/18903	0.00566392884734803	0.031790862765758	0.0217425986028115	TNFRSF21/CLEC7A/VAV3/CD47/PRNP/PLSCR1/HLA-A/HLA-DRB1/NFKBIZ/GBP1/LGALS3/WNK1/IGHG3/IGKC/IGHG4	15
BP	GO:0002286	T cell activation involved in immune response	8/432	118/18903	0.00573439750314704	0.0321473799418849	0.0219864299801682	CD74/HLA-DRB1/NFKBIZ/LGALS3/CD46/HLA-DRA/STAT3/MDK	8
BP	GO:0032436	positive regulation of proteasomal ubiquitin-dependent protein catabolic process	7/432	94/18903	0.00576890794815477	0.0322626351393178	0.0220652560099346	GCLC/DAB2/LRRK2/CAV1/CLU/TRIB1/HSPA1A	7
BP	GO:1903035	negative regulation of response to wounding	7/432	94/18903	0.00576890794815477	0.0322626351393178	0.0220652560099346	PLAUR/SERPINE2/SERPING1/PLAU/SERPINE1/PROS1/MDK	7
BP	GO:0033157	regulation of intracellular protein transport	12/432	225/18903	0.00577672609378748	0.0322673408016511	0.0220684743348988	LRRK2/PRNP/IL1B/PTPN14/STOM/SP100/LMAN1/PTPN1/MFF/PIK3R1/IFI27/HSP90AA1	12
BP	GO:0001516	prostaglandin biosynthetic process	4/432	32/18903	0.00583002311703257	0.0322920442111085	0.0220853696396884	CD74/FABP5/IL1B/PNPLA8	4

BP	GO:0046457	prostanoid biosynthetic process	4/432	32/18903	0.00583002311703257	0.0322920442111085	0.0220853696396884	CD74/FABP5/IL1B/PNPLA8	4
BP	GO:0046825	regulation of protein export from nucleus	4/432	32/18903	0.00583002311703257	0.0322920442111085	0.0220853696396884	IL1B/PTPN14/SP100/IFI27	4
BP	GO:0048710	regulation of astrocyte differentiation	4/432	32/18903	0.00583002311703257	0.0322920442111085	0.0220853696396884	BMP2/ID2/SERPINE2/IL6ST	4
BP	GO:0061036	positive regulation of cartilage development	4/432	32/18903	0.00583002311703257	0.0322920442111085	0.0220853696396884	BMP2/WNT5A/RUNX2/MDK	4
BP	GO:1902235	regulation of endoplasmic reticulum stress-induced intrinsic apoptotic signaling pathway	4/432	32/18903	0.00583002311703257	0.0322920442111085	0.0220853696396884	LRRK2/XBP1/PTPN1/HSPA1A	4
BP	GO:1990776	response to angiotensin	4/432	32/18903	0.00583002311703257	0.0322920442111085	0.0220853696396884	INHBA/CAV1/AGTRAP/CA2	4
BP	GO:0050921	positive regulation of chemotaxis	9/432	144/18903	0.00591503189950253	0.0327237111665062	0.0223805979073346	CXCL8/PDGFD/CD74/WNT5A/NEDD9/WNK1/SERPINE1/CXCL17/MDK	9
BP	GO:0070302	regulation of stress-activated protein kinase signaling cascade	11/432	198/18903	0.00598967853556949	0.0330970886822089	0.0226359605098237	TGFB2/BMP2/WNT5A/DUSP1/IL1A/IL1B/APP/ARL6IP5/TPD52L1/GADD45A/GADD45B	11

BP	GO:0002455	humoral immune response mediated by circulating immunoglobulin	8/432	119/18903	0.00602859904664476	0.0332358812622906	0.0227308843682921	C1S/C1R/CLU/SERPING1/CD46/IGHG3/IGKC/IGHG4	8
BP	GO:0030225	macrophage differentiation	5/432	51/18903	0.0060350974066734	0.0332358812622906	0.0227308843682921	INHBA/ID2/APP/HLA-DRB1/TRIB1	5
BP	GO:0014743	regulation of muscle hypertrophy	6/432	72/18903	0.00603635465088089	0.0332358812622906	0.0227308843682921	RGS4/IL6ST/IGFBP5/ERRFI1/PPP3CA/MTPN	6
BP	GO:0072659	protein localization to plasma membrane	14/432	285/18903	0.00615396823994072	0.0338431665989606	0.0231462226184641	DAB2/RAB31/CDH2/PLS1/PRNP/OPTN/GBP1/LGALS3/ARL6IP5/TMEM59/CCDC88A/RDX/PIK3R1/ATP1B1	14
BP	GO:0048705	skeletal system morphogenesis	12/432	227/18903	0.00618632139247094	0.0339806846082875	0.0232402747648146	MMP13/HAS2/TGFBR2/TGFB3/TIPARP/RDH10/DHRS3/RUNX2/CITED2/ZFAND5/NPPC/COL1A1	12
BP	GO:0090288	negative regulation of cellular response to growth factor stimulus	8/432	120/18903	0.00633413229886911	0.0344717493854636	0.0235761267489771	DCN/HTRA1/WNT5A/SKIL/SULF2/PTPN1/SULF1/SOSTDC1	8
BP	GO:0002921	negative regulation of humoral immune response	3/432	17/18903	0.00635025523814378	0.0344717493854636	0.0235761267489771	CD59/SERPING1/CD46	3
BP	GO:0006857	oligopeptide transport	3/432	17/18903	0.00635025523814378	0.0344717493854636	0.0235761267489771	SLC7A11/GJA1/CA2	3

BP	GO:0010566	regulation of ketone biosynthetic process	3/432	17/18903	0.00635025523814378	0.0344717493854636	0.0235761267489771	BMP2/DAB2/EGR1	3
BP	GO:0016045	detection of bacterium	3/432	17/18903	0.00635025523814378	0.0344717493854636	0.0235761267489771	HLA-B/HLA-A/HLA-DRB1	3
BP	GO:0017014	protein nitrosylation	3/432	17/18903	0.00635025523814378	0.0344717493854636	0.0235761267489771	S100A9/S100A8/NCOA7	3
BP	GO:0018119	peptidyl-cysteine S-nitrosylation	3/432	17/18903	0.00635025523814378	0.0344717493854636	0.0235761267489771	S100A9/S100A8/NCOA7	3
BP	GO:0090083	regulation of inclusion body assembly	3/432	17/18903	0.00635025523814378	0.0344717493854636	0.0235761267489771	CLU/HSPA1A/DNAJB1	3
BP	GO:0090713	immunological memory process	3/432	17/18903	0.00635025523814378	0.0344717493854636	0.0235761267489771	HLA-DRB1/CD46/HLA-DRA	3
BP	GO:0098712	L-glutamate import across plasma membrane	3/432	17/18903	0.00635025523814378	0.0344717493854636	0.0235761267489771	SLC1A3/SLC7A11/ARL6IP5	3
BP	GO:0006898	receptor-mediated endocytosis	13/432	257/18903	0.00642782402298101	0.0347864866218438	0.0237913837379552	B2M/TGFBR2/CXCL8/DAB2/RAB31/ITGAV/CAV1/ADRB2/CLU/CXCL16/TFRC/SERPINE1/CD63	13
BP	GO:0050708	regulation of protein secretion	13/432	257/18903	0.00642782402298101	0.0347864866218438	0.0237913837379552	SAA1/TGFB2/RSAD2/TGFB3/MYH10/IL1A/IL1B/SLC16A1/HLA-DRB1/HIF1A/PPP3CA/TM7SF3/TMED10	13

BP	GO:0046637	regulation of alpha-beta T cell differentiation	6/432	73/18903	0.00645336335600908	0.0347864866218438	0.0237913837379552	TGFBR2/RUNX1/PRDM1/HLA-DRB1/NFKBIZ/HLA-DRA	6
BP	GO:0061035	regulation of cartilage development	6/432	73/18903	0.00645336335600908	0.0347864866218438	0.0237913837379552	BMP2/WNT5A/RUNX2/PTHLH/MAF/MDK	6
BP	GO:0071260	cellular response to mechanical stimulus	6/432	73/18903	0.00645336335600908	0.0347864866218438	0.0237913837379552	GCLC/IL1B/GADD45A/MTPN/SLC38A2/COL1A1	6
BP	GO:0072678	T cell migration	6/432	73/18903	0.00645336335600908	0.0347864866218438	0.0237913837379552	WNT5A/CXCL16/APP/CCL20/WNK1/MSN	6
BP	GO:0046330	positive regulation of JNK cascade	7/432	96/18903	0.00646326429791978	0.0347992984608603	0.0238001460881673	WNT5A/IL1A/IL1B/APP/TPD52L1/GADD45A/GADD45B	7
BP	GO:0045577	regulation of B cell differentiation	4/432	33/18903	0.00651655841685333	0.0350047417862331	0.0239406541263455	INHBA/ID2/XBP1/ZFP36L1	4
BP	GO:0060351	cartilage development involved in endochondral bone morphogenesis	4/432	33/18903	0.00651655841685333	0.0350047417862331	0.0239406541263455	MMP13/TGFBR2/NPPC/COL1A1	4
BP	GO:0010043	response to zinc ion	5/432	52/18903	0.00655461724017881	0.0351275837031599	0.0240246689110658	ASS1/S100A8/SOD2/MT2A/MT1E	5

BP	GO:0043392	negative regulation of DNA binding	5/432	52/18903	0.00655461724017881	0.0351275837031599	0.0240246689110658	IFI16/NFKBIA/SP100/ID1/JUN	5
BP	GO:0007517	muscle organ development	16/432	348/18903	0.00661268290426449	0.0353977528150732	0.024209444599555	TGFB2/ASS1/BMP2/BASP1/CRYAB/WNT5A/CAV1/XBP1/CITED2/EGR1/SVIL/HBEGF/PPP3CA/MTPN/FOXN2/FOS	16
BP	GO:0006720	isoprenoid metabolic process	8/432	121/18903	0.00665126815618991	0.035481101755915	0.0242664491098776	CYP1B1/SDR16C5/RDH10/DHRS3/SCPEP1/NPC2/AKR1B1/CRABP2	8
BP	GO:0032355	response to estradiol	8/432	121/18903	0.00665126815618991	0.035481101755915	0.0242664491098776	ASS1/CYP1B1/CRYAB/AREG/GJB2/STAT3/TXNIP/COL1A1	8
BP	GO:0090100	positive regulation of transmembrane receptor protein serine/threonine kinase signaling pathway	8/432	121/18903	0.00665126815618991	0.035481101755915	0.0242664491098776	TGFB2/TGFBR2/BMP2/DAB2/INHBA/TGFB3/CITED2/SULF1	8
BP	GO:0010950	positive regulation of endopeptidase activity	10/432	174/18903	0.00682595387071124	0.0362554894678533	0.0247960730243867	TNFSF10/CLEC7A/IFI16/S100A9/S100A8/ARL6IP5/CTSD/ASPH/STAT3/UACA	10
BP	GO:0030178	negative regulation of Wnt signaling pathway	10/432	174/18903	0.00682595387071124	0.0362554894678533	0.0247960730243867	BMP2/DAB2/WNT5A/CDH2/CAV1/MCC/EGR1/TAX1BP3/MDK/SOSTDC1	10
BP	GO:0008593	regulation of Notch signaling pathway	7/432	97/18903	0.00683288862359862	0.0362554894678533	0.0247960730243867	TGFB2/GALNT11/NFKBIA/IL6ST/CD46/STAT3/POSTN	7

BP	GO:0043502	regulation of muscle adaptation	7/432	97/18903	0.00683288862359862	0.0362554894678533	0.0247960730243867	RGS4/IL6ST/IGFBP5/FBXO32/ERRFI1/PPP3CA/MTPN	7
BP	GO:0008037	cell recognition	12/432	230/18903	0.00684346860658074	0.0362554894678533	0.0247960730243867	TNFRSF21/CLEC7A/NRCAM/APP/NEDD9/B4GALT1/LGALS3/MSN/IGHG3/IGKC/IGHG4/PALLD	12
BP	GO:0050920	regulation of chemotaxis	12/432	230/18903	0.00684346860658074	0.0362554894678533	0.0247960730243867	MMP28/CXCL8/PDGFD/CD74/WNT5A/DUSP1/NEDD9/WNK1/SERPINE1/CXCL17/SEMA6D/MDK	12
BP	GO:0043500	muscle adaptation	8/432	122/18903	0.00698027838262553	0.0369379719904383	0.0252628405874861	RGS4/IL1B/IL6ST/IGFBP5/FBXO32/ERRFI1/PPP3CA/MTPN	8
BP	GO:0032147	activation of protein kinase activity	9/432	148/18903	0.00704351139936847	0.0372299888252333	0.0254625585023786	TGFB2/TGFBR2/AREG/WNT5A/PRNP/ADRB2/WNK1/CCDC88A/HBEGF	9
BP	GO:0060828	regulation of canonical Wnt signaling pathway	13/432	260/18903	0.00705909931309179	0.0372697880400109	0.0254897782213901	BMP2/DAB2/LRRK2/WNT5A/CDH2/CAV1/CDH3/MCC/WNK1/EGR1/MDK/SOSTDC1/COL1A1	13
BP	GO:0009267	cellular response to starvation	10/432	175/18903	0.00709693146788651	0.0373399476608613	0.0255377622124354	IFI16/LRRK2/DAPL1/XBP1/HSPA5/LAMP2/MIOS/SLC38A2/FOS/WNT2B	10
BP	GO:0046638	positive regulation of alpha-beta T cell differentiation	5/432	53/18903	0.00710468193330982	0.0373399476608613	0.0255377622124354	TGFBR2/RUNX1/HLA-DRB1/NFKBIZ/HLA-DRA	5
BP	GO:0048146	positive regulation of fibroblast proliferation	5/432	53/18903	0.00710468193330982	0.0373399476608613	0.0255377622124354	PDGFD/CD74/WNT5A/FOSL2/JUN	5

BP	GO:0048260	positive regulation of receptor-mediated endocytosis	5/432	53/18903	0.00710468193330982	0.0373399476608613	0.0255377622124354	B2M/DAB2/CLU/SERPINE1/CD63	5
BP	GO:0042742	defense response to bacterium	17/432	382/18903	0.00713538636852844	0.0374587536372804	0.0256190167123818	S100A9/S100A8/CXCL6/OAS2/SPRR2A/GRN/OPTN/IL1B/HLA-A/SLPI/CCL20/ISG15/IGHG3/IGKC/SERPINE1/IGHG4/HLA-E	17
BP	GO:1903828	negative regulation of regulation of protein localization	11/432	203/18903	0.00717911361374044	0.0376455787568589	0.0257467912749612	DAB2/RSAD2/LRRK2/NDFIP2/IL1B/GBP1/SP100/LMAN1/TMEM59/TAX1BP3/PPP3CA	11
BP	GO:0010975	regulation of neuron projection development	19/432	446/18903	0.00723758706206423	0.0378697632356389	0.0259001168758111	B2M/S100A9/NRCAM/DAB2/LRRK2/ITGA6/WNT5A/CDH2/GRN/ID1/SKIL/VIM/HSPA5/CCDC88A/PPP3CA/SEMA6D/CUX1/CRABP2/MDK	19
BP	GO:0032743	positive regulation of interleukin-2 production	4/432	34/18903	0.00725461842741105	0.0378697632356389	0.0259001168758111	CLEC7A/RUNX1/IL1A/IL1B	4
BP	GO:0060317	cardiac epithelial to mesenchymal transition	4/432	34/18903	0.00725461842741105	0.0378697632356389	0.0259001168758111	TGFB2/HAS2/TGFBR2/BMP2	4
BP	GO:1902624	positive regulation of neutrophil migration	4/432	34/18903	0.00725461842741105	0.0378697632356389	0.0259001168758111	CXCL8/CD74/IL1A/MDK	4
BP	GO:0002224	toll-like receptor signaling pathway	8/432	123/18903	0.00732143545795595	0.0381754667339868	0.026109195456189	BIRC3/RSAD2/RAB7B/TNIP3/CAV1/NFKBIA/BIRC2/TNFAIP3	8
BP	GO:2000377	regulation of reactive oxygen	9/432	149/18903	0.00734979302823053	0.038280172022034	0.0261808061282987	CLEC7A/TGFBR2/CYP1B1/SOD2/CRYAB/STK17A/BIRC2/HIF1A/GADD45A	9

		species metabolic process							
BP	GO: 0010634	positive regulation of epithelial cell migration	10/432	176/18903	0.00737616360835487	0.0383311872906082	0.02621569679847	TGFB2/HAS2/TGFBR2/RIN2/WNT5A/GRN/HIF1A/HBEGF/JUN/SPARC	10
BP	GO: 1902905	positive regulation of supramolecular fiber organization	10/432	176/18903	0.00737616360835487	0.0383311872906082	0.02621569679847	CSF3/PLEK/TGFB3/CD47/CDC42EP3/CLU/APP/ID1/CCDC88A/HSPA1A	10
BP	GO: 0031032	actomyosin structure organization	11/432	204/18903	0.00743770887158321	0.0386076358373427	0.0264047671558953	TGFB3/CD47/MYH10/CGNL1/TACSTD2/MYL9/CNN3/CCDC88A/KRT19/PIK3R1/CSRP2	11
BP	GO: 0002693	positive regulation of cellular extravasation	3/432	18/18903	0.00749265499294408	0.0386758139981768	0.0264513959748555	PDGFD/CD47/MDK	3
BP	GO: 0051917	regulation of fibrinolysis	3/432	18/18903	0.00749265499294408	0.0386758139981768	0.0264513959748555	PLAUR/PLAU/SERPINE1	3
BP	GO: 0060391	positive regulation of SMAD protein signal transduction	3/432	18/18903	0.00749265499294408	0.0386758139981768	0.0264513959748555	BMP2/DAB2/TGFB3	3
BP	GO: 0060602	branch elongation of an epithelium	3/432	18/18903	0.00749265499294408	0.0386758139981768	0.0264513959748555	AREG/WNT5A/RDH10	3
BP	GO: 2000641	regulation of early endosome to late endosome transport	3/432	18/18903	0.00749265499294408	0.0386758139981768	0.0264513959748555	DAB2/MSN/RDX	3

BP	GO:0008406	gonad development	12/432	233/18903	0.0075544708750673	0.0389514245230616	0.0266398931873229	TNFSF10/TGFB2/CYP1B1/INHBA/BASP1/TIPARP/WNT5A/RDH10/IL1A/CITED2/HSPA5/WNT2B	12
BP	GO:0072676	lymphocyte migration	8/432	124/18903	0.00767501242483016	0.0394983856553776	0.0270139741438201	SAA1/WNT5A/CXCL16/APP/NEDD9/CCL20/WNK1/MSN	8
BP	GO:0042572	retinol metabolic process	5/432	54/18903	0.00768617234374916	0.0394983856553776	0.0270139741438201	CYP1B1/SDR16C5/RDH10/DHRS3/AKR1B1	5
BP	GO:0045744	negative regulation of G protein-coupled receptor signaling pathway	5/432	54/18903	0.00768617234374916	0.0394983856553776	0.0270139741438201	CXCL8/PLEK/RGS4/ADRB2/ARRDC3	5
BP	GO:0045685	regulation of glial cell differentiation	6/432	76/18903	0.00782981319642405	0.0401918823900791	0.0274882746133002	TNFRSF21/BMP2/ID2/SERPINE2/IL6ST/MDK	6
BP	GO:0006694	steroid biosynthetic process	10/432	178/18903	0.00796004213622921	0.0407247730974116	0.0278527324398798	CYP27A1/BMP2/DAB2/IL1A/ATP1A1/EGR1/NR3C1/PBX1/AKR1B1/IGFBP7	10
BP	GO:0071772	response to BMP	10/432	178/18903	0.00796004213622921	0.0407247730974116	0.0278527324398798	TGFB2/BMP2/TGFB3/HTRA1/WNT5A/RUNX2/SKIL/EGR1/SULF1/SOSTDC1	10
BP	GO:0071773	cellular response to BMP stimulus	10/432	178/18903	0.00796004213622921	0.0407247730974116	0.0278527324398798	TGFB2/BMP2/TGFB3/HTRA1/WNT5A/RUNX2/SKIL/EGR1/SULF1/SOSTDC1	10
BP	GO:0051222	positive regulation of protein transport	14/432	294/18903	0.00799878868344362	0.0408021470056102	0.0279056504698398	TGFB2/TGFB3/MYH10/IL1A/PRNP/IL1B/STOM/HLA-DRB1/HIF1A/MFF/PIK3R1/TM7SF3/TMED10/HSP90AA1	14

BP	GO:0022411	cellular component disassembly	20/432	483/18903	0.00802869270771296	0.0408021470056102	0.0279056504698398	MMP13/MMP7/MMP10/MMP12/MMP1/PLEK/TGFB3/ADRB2/OPTN/NEDD9/HIF1A/CTSV/ASPH/SVIL/VMP1/MTPN/CST3/RDX/PIK3R1/LAMP2	20
BP	GO:0097306	cellular response to alcohol	7/432	100/18903	0.00803607027405855	0.0408021470056102	0.0279056504698398	CYP1B1/SOD2/INHBA/SLC23A2/KLF9/SGK1/FOS	7
BP	GO:0003014	renal system process	8/432	125/18903	0.00804128273502163	0.0408021470056102	0.0279056504698398	HAS2/SLC5A1/SULF2/KCNMA1/PPP3CA/SGK1/AKR1B1/SULF1	8
BP	GO:1904375	regulation of protein localization to cell periphery	8/432	125/18903	0.00804128273502163	0.0408021470056102	0.0279056504698398	GPC6/DAB2/PLS1/PRNP/GBP1/LGALS3/TMEM59/PIK3R1	8
BP	GO:0001893	maternal placenta development	4/432	35/18903	0.00804574228521438	0.0408021470056102	0.0279056504698398	PRDM1/GJB2/CTSB/CITED2	4
BP	GO:0055075	potassium ion homeostasis	4/432	35/18903	0.00804574228521438	0.0408021470056102	0.0279056504698398	ATP1A1/WNK1/KCNMA1/ATP1B1	4
BP	GO:2000406	positive regulation of T cell migration	4/432	35/18903	0.00804574228521438	0.0408021470056102	0.0279056504698398	WNT5A/APP/CCL20/WNK1	4
BP	GO:0002285	lymphocyte activation involved in immune response	11/432	207/18903	0.00825726695223112	0.0418224250418345	0.0286034451779716	CD74/HLA-DRB1/NFKBIZ/XBP1/LGALS3/CD46/HLA-DRA/TFRC/STAT3/MDK/LGALS1	11
BP	GO:0060759	regulation of response to cytokine stimulus	10/432	179/18903	0.00826501545691605	0.0418224250418345	0.0286034451779716	MMP12/CD74/WNT5A/CAV1/IL1RN/HIF1A/ISG15/TNFAIP3/PTPN1/HSPA1A	10

BP	GO:0006584	catecholamine metabolic process	5/432	55/18903	0.00829995523135073	0.0418618243674996	0.0286303914024094	TGFB2/EPAS1/NR4A2/MAOA/MTPN	5
BP	GO:0009712	catechol-containing compound metabolic process	5/432	55/18903	0.00829995523135073	0.0418618243674996	0.0286303914024094	TGFB2/EPAS1/NR4A2/MAOA/MTPN	5
BP	GO:1903202	negative regulation of oxidative stress-induced cell death	5/432	55/18903	0.00829995523135073	0.0418618243674996	0.0286303914024094	SLC7A11/NCOA7/SOD2/LRRK2/HIF1A	5
BP	GO:0006875	cellular metal ion homeostasis	18/432	420/18903	0.00831203567256669	0.0418770860409814	0.0286408292581847	SLC1A3/S100A9/S100A8/SLC39A14/HEPHL1/WNT5A/CAV1/IL1A/PRNP/ATP1A1/APP/HIF1A/TFRC/KCNMA1/SLC39A6/ATP1B1/MT2A/MT1E	18
BP	GO:0030968	endoplasmic reticulum unfolded protein response	6/432	77/18903	0.00833227676575194	0.0418878043930464	0.0286481598181746	DNAJB9/XBP1/ATF6/HSPA5/PTPN1/PIK3R1	6
BP	GO:0034121	regulation of toll-like receptor signaling pathway	6/432	77/18903	0.00833227676575194	0.0418878043930464	0.0286481598181746	BIRC3/RSAD2/RAB7B/CAV1/BIRC2/TNFAIP3	6
BP	GO:0003206	cardiac chamber morphogenesis	8/432	126/18903	0.00842052009498522	0.0422854565030474	0.0289201244476034	TGFB2/CPE/TGFBR2/BMP2/WNT5A/DHRS3/HIF1A/CITED2	8
BP	GO:0006721	terpenoid metabolic process	7/432	101/18903	0.00846982192112646	0.0424408736567821	0.0290264182847523	CYP1B1/SDR16C5/RDH10/DHRS3/SCPEP1/AKR1B1/CRABP2	7

BP	GO:0015908	fatty acid transport	7/432	101/18903	0.00846982192112646	0.0424408736567821	0.0290264182847523	SLC27A6/IL1A/FABP5/IL1B/PNPLA8/FABP4/CRABP2	7
BP	GO:0032869	cellular response to insulin stimulus	11/432	208/18903	0.0085454851958724	0.042727425979362	0.0292223988774925	GCLC/RAB31/SLC39A14/STAT1/IL1B/XBP1/ERRFI1/PKM/PTPN1/PIK3R1/ZFP36L1	11
BP	GO:0045216	cell-cell junction organization	11/432	208/18903	0.0085454851958724	0.042727425979362	0.0292223988774925	CLDN1/TGFB2/TGFB3/CDH2/CAV1/IL1B/GJB2/MPP7/GJA1/RDX/DSG2	11
BP	GO:0006575	cellular modified amino acid metabolic process	10/432	180/18903	0.00857889707064325	0.0428481630148218	0.0293049740789848	SLC7A11/ASS1/GCLC/SOD2/VNN1/PLSCR1/CTSB/ARL6IP5/SERINC2/DIO2	10
BP	GO:0002263	cell activation involved in immune response	14/432	297/18903	0.00870285337413665	0.0432292205456518	0.0295655892437403	CD74/GRN/APP/HLA-DRB1/NFKBIZ/XBP1/LGALS3/CD46/HLA-DRA/TFRC/STAT3/S100A13/MDK/LGALS1	14
BP	GO:0002523	leukocyte migration involved in inflammatory response	3/432	19/18903	0.0087486595525903	0.0432292205456518	0.0295655892437403	S100A9/S100A8/MDK	3
BP	GO:0003417	growth plate cartilage development	3/432	19/18903	0.0087486595525903	0.0432292205456518	0.0295655892437403	MMP13/TGFBR2/NPPC	3
BP	GO:0036003	positive regulation of transcription from RNA polymerase II promoter in response to stress	3/432	19/18903	0.0087486595525903	0.0432292205456518	0.0295655892437403	HIF1A/ATF6/HSPA5	3

BP	GO:0042574	retinal metabolic process	3/432	19/18903	0.0087486595525903	0.0432292205456518	0.0295655892437403	CYP1B1/SDR16C5/RDH10	3
BP	GO:0045953	negative regulation of natural killer cell mediated cytotoxicity	3/432	19/18903	0.0087486595525903	0.0432292205456518	0.0295655892437403	HLA-B/HLA-A/HLA-E	3
BP	GO:0051797	regulation of hair follicle development	3/432	19/18903	0.0087486595525903	0.0432292205456518	0.0295655892437403	TGFB2/WNT5A/CDH3	3
BP	GO:0055070	copper ion homeostasis	3/432	19/18903	0.0087486595525903	0.0432292205456518	0.0295655892437403	PRNP/APP/MT2A	3
BP	GO:0097501	stress response to metal ion	3/432	19/18903	0.0087486595525903	0.0432292205456518	0.0295655892437403	HSPA5/MT2A/MT1E	3
BP	GO:2001185	regulation of CD8-positive, alpha-beta T cell activation	3/432	19/18903	0.0087486595525903	0.0432292205456518	0.0295655892437403	RUNX1/HLA-A/HLA-E	3
BP	GO:0009306	protein secretion	16/432	359/18903	0.0087798622330517	0.0433371001364612	0.0296393709041402	SAA1/TGFB2/RSAD2/PLEK/TGFB3/MYH10/IL1A/IL1B/IL1RN/ABCA1/SLC16A1/HLA-DRB1/HIF1A/PPP3CA/TM7SF3/TMED10	16
BP	GO:0043154	negative regulation of cysteine-type endopeptidase activity involved	6/432	78/18903	0.00885747417670827	0.0436084325047584	0.0298249421739514	BIRC3/PLAUR/CRYAB/TNFAIP8/IFI6/BIRC2	6

		in apoptotic process							
BP	GO:0061045	negative regulation of wound healing	6/432	78/18903	0.00885747417670827	0.0436084325047584	0.0298249421739514	PLAUR/SERPINE2/SERPING1/PLAU/SERPINE1/PROS1	6
BP	GO:0045137	development of primary sexual characteristics	12/432	238/18903	0.00886708965427641	0.0436084325047584	0.0298249421739514	TNFSF10/TGFB2/CYP1B1/INHBA/BASP1/TIPARP/WNT5A/RDH10/IL1A/CITED2/HSPA5/WNT2B	12
BP	GO:0045589	regulation of regulatory T cell differentiation	4/432	36/18903	0.00889140580583506	0.0436084325047584	0.0298249421739514	HLA-DRB1/CD46/HLA-DRA/MDK	4
BP	GO:0060323	head morphogenesis	4/432	36/18903	0.00889140580583506	0.0436084325047584	0.0298249421739514	TGFB3/TIPARP/ASPH/COL1A1	4
BP	GO:0070306	lens fiber cell differentiation	4/432	36/18903	0.00889140580583506	0.0436084325047584	0.0298249421739514	SLC7A11/SKIL/VIM/MAF	4
BP	GO:0014897	striated muscle hypertrophy	7/432	102/18903	0.00892054466385533	0.04361259944000961	0.02982779204094996	RGS4/SORBS2/IL6ST/IGFBP5/ERRFI1/PPP3CA/MTPN	7
BP	GO:0019218	regulation of steroid metabolic process	7/432	102/18903	0.00892054466385533	0.04361259944000961	0.02982779204094996	BMP2/DAB2/IL1A/ATP1A1/EGR1/NR3C1/IGFBP7	7
BP	GO:0060419	heart growth	7/432	102/18903	0.00892054466385533	0.04361259944000961	0.02982779204094996	TGFB2/TGFBR2/BASP1/RUNX1/RGS4/SORBS2/CITED2	7
BP	GO:0002707	negative regulation of lymphocyte	5/432	56/18903	0.0089468821445802	0.043649082192704	0.0298527435537014	HLA-B/IL20RB/HLA-A/CD46/HLA-E	5

		mediated immunity							
BP	GO:0060986	endocrine hormone secretion	5/432	56/18903	0.0089468821445802	0.043649082192704	0.0298527435537014	DAB2/INHBA/SELENOM/IL1B/GJA1	5
BP	GO:0035592	establishment of protein localization to extracellular region	16/432	360/18903	0.00900206618589356	0.0438720296203980	0.0300052231031115	SAA1/TGFB2/RSAD2/PLEK/TGFB3/MYH10/IL1A/IL1B/IL1RN/ABCA1/SLC16A1/HLA-DRB1/HIF1A/PPP3CA/TM7SF3/TMED10	16
BP	GO:0050868	negative regulation of T cell activation	8/432	128/18903	0.00921899113311733	0.0448819305164923	0.0306959206149779	TNFRSF21/CD74/IL20RB/RUNX1/PRNP/HLA-DRB1/LGALS3/MDK	8
BP	GO:0048144	fibroblast proliferation	7/432	103/18903	0.0093886121172041	0.0456116922710808	0.0311950236755842	SOD2/PDGFD/CD74/WNT5A/CAV1/FOSL2/JUN	7
BP	GO:1903076	regulation of protein localization to plasma membrane	7/432	103/18903	0.0093886121172041	0.0456116922710808	0.0311950236755842	DAB2/PLS1/PRNP/GBP1/LGALS3/TMEM59/PIK3R1	7
BP	GO:0042594	response to starvation	11/432	211/18903	0.00945710524877361	0.0458962348117292	0.0313896297261443	IFI16/LRRK2/DAPL1/XBP1/HSPA5/LAMP2/MIOS/ZFP36/SLC38A2/FOS/WNT2B	11
BP	GO:0003018	vascular process in circulatory system	13/432	270/18903	0.00952525450180293	0.0461785137010886	0.0315826876066621	SLC1A3/GCLC/SOD2/CAV1/FABP5/ADRB2/SLC5A1/SCPEP1/SLC16A1/TFRC/KCNMA1/SLC38A2/NPPC	13
BP	GO:0001936	regulation of endothelial cell proliferation	10/432	183/18903	0.00957564206724998	0.04637418278642	0.0317165107876398	BMP2/STAT1/WNT5A/CAV1/HIF1A/STAT3/JUN/SULF1/SPARC/MDK	10

BP	GO:0010332	response to gamma radiation	5/432	57/18903	0.00962778835852001	0.0465292802070586	0.0318225859510011	SOD2/CRYAB/IL1A/EGR1/HSPA5	5
BP	GO:0032768	regulation of monooxygenase activity	5/432	57/18903	0.00962778835852001	0.0465292802070586	0.0318225859510011	CAV1/IL1A/IL1B/CDH3/HIF1A	5
BP	GO:0050714	positive regulation of protein secretion	8/432	129/18903	0.00963877210085523	0.0465337379608094	0.0318256347248324	TGFB2/TGFB3/MYH10/IL1A/HLA-DRB1/HIF1A/TM7SF3/TMED10	8
BP	GO:0048638	regulation of developmental growth	15/432	332/18903	0.0097629606173329	0.0469841461107364	0.0321336805832746	NRCAM/TGFBR2/BASP1/RUNX1/RGS4/WNT5A/SLC23A2/PLS1/ADRB2/APP/CITED2/STAT3/SEMA6D/CRABP2/NPPC	15
BP	GO:0009100	glycoprotein metabolic process	17/432	395/18903	0.00976774287695842	0.0469841461107364	0.0321336805832746	DCN/MMP12/BMP2/PHLDA1/DSE/GALNT5/GALNT1/GALNT11/B4GALT1/HIF1A/HS6ST2/TUSC3/TMEM59/SULF2/HBEGF/CST3/SULF1	17
BP	GO:0008207	C21-steroid hormone metabolic process	4/432	37/18903	0.00979301985962159	0.0469841461107364	0.0321336805832746	BMP2/DAB2/EGR1/AKR1B1	4
BP	GO:0010934	macrophage cytokine production	4/432	37/18903	0.00979301985962159	0.0469841461107364	0.0321336805832746	TGFB2/TGFB3/CD74/WNT5A	4
BP	GO:0010935	regulation of macrophage cytokine production	4/432	37/18903	0.00979301985962159	0.0469841461107364	0.0321336805832746	TGFB2/TGFB3/CD74/WNT5A	4
BP	GO:0071276	cellular response to cadmium ion	4/432	37/18903	0.00979301985962159	0.0469841461107364	0.0321336805832746	MT2A/FOS/JUN/MT1E	4

BP	GO:0014896	muscle hypertrophy	7/432	104/18903	0.00987439637027178	0.0473254748316134	0.0323671241807109	RGS4/SORBS2/IL6ST/IGFBP5/ERRFI1/PPP3CA/MTPN	7
BP	GO:0070227	lymphocyte apoptotic process	6/432	80/18903	0.00997822378775658	0.0477038495820693	0.0326259045221322	TNFRSF21/CD74/WNT5A/TSC22D3/HIF1A/LGALS3	6
BP	GO:0071230	cellular response to amino acid stimulus	6/432	80/18903	0.00997822378775658	0.0477038495820693	0.0326259045221322	ASS1/GCLC/PDGFD/XBP1/CAPN2/COL1A1	6
BP	GO:0048511	rhythmic process	14/432	302/18903	0.00998428678820391	0.0477038495820693	0.0326259045221322	BHLHE41/TGFB2/ASS1/HAS2/CYP1B1/INHBA/TGFB3/BHLHE40/ID2/ID1/EGR1/HSPA5/KLF9/MDK	14
BP	GO:0002716	negative regulation of natural killer cell mediated immunity	3/432	20/18903	0.0101205390111333	0.0479094093413425	0.0327664921924857	HLA-B/HLA-A/HLA-E	3
BP	GO:0007171	activation of transmembrane receptor protein tyrosine kinase activity	3/432	20/18903	0.0101205390111333	0.0479094093413425	0.0327664921924857	AREG/ADRB2/HBEGF	3
BP	GO:0010288	response to lead ion	3/432	20/18903	0.0101205390111333	0.0479094093413425	0.0327664921924857	CLDN1/PLSCR1/APP	3
BP	GO:0032495	response to muramyl dipeptide	3/432	20/18903	0.0101205390111333	0.0479094093413425	0.0327664921924857	NFKBIA/VIM/TNFAIP3	3

BP	GO:0045655	regulation of monocyte differentiation	3/432	20/18903	0.0101205390111333	0.0479094093413425	0.0327664921924857	CD74/HLA-DRB1/ZFP36L1	3
BP	GO:0055093	response to hyperoxia	3/432	20/18903	0.0101205390111333	0.0479094093413425	0.0327664921924857	SOD2/CAV1/COL1A1	3
BP	GO:0060749	mammary gland alveolus development	3/432	20/18903	0.0101205390111333	0.0479094093413425	0.0327664921924857	ID2/AREG/HIF1A	3
BP	GO:0061377	mammary gland lobule development	3/432	20/18903	0.0101205390111333	0.0479094093413425	0.0327664921924857	ID2/AREG/HIF1A	3
BP	GO:0071371	cellular response to gonadotropin stimulus	3/432	20/18903	0.0101205390111333	0.0479094093413425	0.0327664921924857	GCLC/CYP1B1/INHBA	3
BP	GO:0048754	branching morphogenesis of an epithelial tube	9/432	157/18903	0.0101803339363066	0.0481431947396911	0.0329263841121529	TGFBR2/BMP2/AREG/WNT5A/RDH10/TACSTD2/PBX1/WNT2B/MDK	9
BP	GO:0031331	positive regulation of cellular catabolic process	18/432	429/18903	0.0101960734553184	0.0481683756188433	0.032943605970896	DCN/GCLC/DAB2/LRRK2/CAV1/ADRB2/OPTN/IL1B/CLU/APP/HIF1A/TRIB1/ATF6/TMEM59/PTPN1/ZFP36/ZFP36L1/HSPA1A	18
BP	GO:0001961	positive regulation of cytokine-mediated signaling pathway	5/432	58/18903	0.0103434918646039	0.0486659713873784	0.0332839246700591	MMP12/CD74/WNT5A/HIF1A/HSPA1A	5
BP	GO:0045620	negative regulation of lymphocyte differentiation	5/432	58/18903	0.0103434918646039	0.0486659713873784	0.0332839246700591	INHBA/CD74/RUNX1/ID2/MDK	5

BP	GO:0048008	platelet-derived growth factor receptor signaling pathway	5/432	58/18903	0.0103434918646039	0.0486659713873784	0.0332839246700591	PDGFD/TIPARP/ZFAND5/PTPN1/TXNIP	5
BP	GO:0060350	endochondral bone morphogenesis	5/432	58/18903	0.0103434918646039	0.0486659713873784	0.0332839246700591	MMP13/TGFBR2/RUNX2/NPPC/COL1A1	5
BP	GO:0007611	learning or memory	13/432	273/18903	0.0103833143493658	0.048803687871765	0.0333781125586694	B2M/SLC7A11/MME/PRNP/UBA6/APP/NEDD9/HIF1A/ARL6IP5/GM2A/SGK1/FOS/MDK	13
BP	GO:0061844	antimicrobial humoral immune response mediated by antimicrobial peptide	6/432	81/18903	0.0105748392645972	0.04965343309519	0.0339592754369379	CXCL1/S100A9/CXCL8/CXCL6/CXCL2/CXCL3	6
BP	GO:0043405	regulation of MAP kinase activity	10/432	186/18903	0.0106585184068794	0.0499955858334862	0.0341932826013374	BMP2/LRRK2/PDGFD/WNT5A/DUSP1/CAV1/IL1B/TRIB1/TPD52L1/PTPN1	10
CC	GO:0062023	collagen-containing extracellular matrix	50/441	433/19869	7.48174806937517e-22	3.01514447195819e-19	2.15001812940992e-19	ANGPTL7/DCN/COL6A6/MMP28/TGFB2/LAMC2/LAMA3/LUM/S100A9/LAMB3/S100A8/GPC6/SPARCL1/OGN/TGFB3/OMD/HTRA1/SRPX/LAMB1/WNT5A/CDH2/FBLN1/SERPINE2/PLSCR1/COL17A1/SLPI/CTSB/CLU/AMTN/COL12A1/SERPING1/COL14A1/LGALS3/LMAN1/CTSD/SERPINE1/LGALS3BP/ANXA5/TIMP1/PKM/DST/POSTN/IGFBP7/SULF1/WNT2B/HSP90AA1/SPARC/MDK/LGALS1/COL1A1	50
CC	GO:0005788	endoplasmic reticulum lumen	29/441	312/19869	8.75372223014474e-11	1.76387502937417e-08	1.25777166780501e-08	B2M/APOL1/SPARCL1/PLAUR/PDGFD/SELENOM/LAMB1/WNT5A/CDH2/P4HA2/IGFBP3/COL17A1/SHISA5/APLP2/CLU/AMTN/APP/DNAJB9/IGFBP5/COL12A1/SERPING1/COL14A1/HSPA5/DNAJC3/TIMP1/CST3/IGFBP7/LGALS1/COL1A1	29

CC	GO:0030055	cell-substrate junction	33/441	432/19869	7.56014005060729e-10	8.28056726235838e-08	5.90464898164774e-08	B2M/LAMA3/DAB2/PLAUR/ITGA6/ITGAV/CDH2/MME/TNS4/CAV1/RND3/PPFIBP1/COL17A1/SORBS2/NEDD9/CD59/GJA1/JAK1/CD46/MSN/PLAU/VIM/CNN3/CAPN2/HSPA5/SVIL/ANXA5/MPZL1/DST/RDX/PALLD/CSRP2/HSPA1A	33
CC	GO:0030139	endocytic vesicle	29/441	343/19869	8.21892532244008e-10	8.28056726235838e-08	5.90464898164774e-08	SAA1/CSF3/B2M/HLA-C/HLA-B/RAB7B/CD74/RAB31/RIN2/AREG/ITGAV/WNT5A/TAPBP/CAV1/ADRB2/ABCA1/HLA-A/HLA-DRB1/MYO6/VIM/HLA-DRA/TFRC/HSPH1/HBEGF/HLA-E/LAMP2/PLD1/HSP90AA1/SPARC	29
CC	GO:0043202	lysosomal lumen	15/441	98/19869	4.18914442118042e-09	3.37645040347142e-07	2.40765563575212e-07	DCN/CSF3/LUM/GPC6/OGN/CD74/OMD/FUCA1/CTSB/NPC2/GM2A/CTSV/CTSD/LAMP2/HSP90AA1	15
CC	GO:0005925	focal adhesion	31/441	422/19869	6.35878015356381e-09	4.27098066981036e-07	3.0455210209174e-07	B2M/DAB2/PLAUR/ITGA6/ITGAV/CDH2/MME/TNS4/CAV1/RND3/PPFIBP1/SORBS2/NEDD9/CD59/GJA1/JAK1/CD46/MSN/PLAU/VIM/CNN3/CAPN2/HSPA5/SVIL/ANXA5/MPZL1/DST/RDX/PALLD/CSRP2/HSPA1A	31
CC	GO:0030667	secretory granule membrane	26/441	313/19869	9.05217084469272e-09	5.21146407201595e-07	3.71615434676859e-07	CPE/HLA-C/BST2/IQGAP2/HLA-B/PLAUR/RAB31/VNN1/CD47/ITGAV/MME/CAV1/FABP5/APLP2/STOM/B4GALT1/CD59/LGALS3/CD58/CD46/PLAU/CD63/LAMP2/TMED10/PLD1/SPARC	26
CC	GO:0012507	ER to Golgi transport vesicle membrane	12/441	63/19869	1.18034462070953e-08	5.94598602682428e-07	4.23992212439083e-07	B2M/HLA-C/HLA-B/CD74/AREG/HLA-A/HLA-DRB1/CD59/LMAN1/HLA-DRA/HLA-E/TMED10	12
CC	GO:0034774	secretory granule lumen	26/441	322/19869	1.6231454947428e-08	7.26808482645943e-07	5.18267508847702e-07	B2M/TGFB2/CXCL1/S100A9/S100A8/TGFB3/GRN/FABP5/SLPI/FUCA1/CLU/APP/HSPA6/SERPING1/NPC2/PYGL/GM2A/CTSD/SERPINE1/LGALS3BP/DNAJC3/TIMP1/PKM/PROS1/HSP90AA1/SPARC	26
CC	GO:0060205	cytoplasmic vesicle lumen	26/441	325/19869	1.96217667366476e-08	7.90757199486898e-07	5.63867612537346e-07	B2M/TGFB2/CXCL1/S100A9/S100A8/TGFB3/GRN/FABP5/SLPI/FUCA1/CLU/APP/HSPA6/SERPING1/NPC2/PYGL/GM2A/CTSD/SERPINE1/LGALS3BP/DNAJC3/TIMP1/PKM/PROS1/HSP90AA1/SPARC	26

CC	GO:0031983	vesicle lumen	26/441	327/19869	2.22370949152047e-08	8.14686295529771e-07	5.80930804961806e-07	B2M/TGFB2/CXCL1/S100A9/S100A8/TGFB3/GRN/FABP5/SLPI/FUCA1/CLU/APP/HSPA6/SERPING1/NPC2/PYGL/GM2A/CTSD/SERPINE1/LGALS3BP/DNAJC3/TIMP1/PKM/PROS1/HSP90AA1/SPARC	26
CC	GO:0072562	blood microparticle	17/441	145/19869	2.46088448654404e-08	8.26447040064375e-07	5.89317074409231e-07	CFH/APOL1/C1S/C1R/CLU/STOM/HSPA6/SERPING1/MSN/IGHG3/IGKC/TFRC/IGHG4/LGALS3BP/ANXA5/PROS1/HSPA1A	17
CC	GO:0042611	MHC protein complex	8/441	25/19869	4.29335895709376e-08	1.33094127669906e-06	9.4905829577862e-07	B2M/HLA-C/HLA-B/CD74/HLA-A/HLA-DRB1/HLA-DRA/HLA-E	8
CC	GO:0005775	vacuolar lumen	18/441	176/19869	8.08998166164332e-08	2.32875900688733e-06	1.66057518317942e-06	DCN/CSF3/LUM/GPC6/OGN/CD74/OMD/GRN/FABP5/FUCA1/CTSB/NPC2/GM2A/CTSV/CTSD/DNAJC3/LAMP2/HSP90AA1	18
CC	GO:0030662	coated vesicle membrane	19/441	198/19869	9.80646121114544e-08	2.63466924539441e-06	1.87871151624049e-06	GAD2/B2M/HLA-C/DAB2/HLA-B/CD74/AREG/WNT5A/ADRB2/HLA-A/HLA-DRB1/CD59/MYO6/LMAN1/HLA-DRA/TFRC/HBEGF/HLA-E/TMED10	19
CC	GO:0071556	integral component of lumenal side of endoplasmic reticulum membrane	8/441	29/19869	1.57598610692743e-07	3.73601412406915e-06	2.66405081852129e-06	HLA-C/HLA-B/CD74/TAPBP/HLA-A/HLA-DRB1/HLA-DRA/HLA-E	8
CC	GO:0098553	lumenal side of endoplasmic reticulum membrane	8/441	29/19869	1.57598610692743e-07	3.73601412406915e-06	2.66405081852129e-06	HLA-C/HLA-B/CD74/TAPBP/HLA-A/HLA-DRB1/HLA-DRA/HLA-E	8
CC	GO:0030134	COPII-coated ER to Golgi transport vesicle	13/441	95/19869	1.77935958265667e-07	3.98378839894799e-06	2.84073196529398e-06	B2M/HLA-C/HLA-B/CD74/AREG/HLA-A/APP/HLA-DRB1/CD59/LMAN1/HLA-DRA/HLA-E/TMED10	13

CC	GO:0030666	endocytic vesicle membrane	18/441	194/19869	3.52938049559952e-07	7.48600178803478e-06	5.33806579112836e-06	B2M/HLA-C/HLA-B/RAB7B/CD74/RAB31/AREG/WNT5A/TAPBP/CAV1/ADRB2/HLA-A/HLA-DRB1/HLA-DRA/TFRC/HBEGF/HLA-E/LAMP2	18
CC	GO:0098576	lumenal side of membrane	8/441	35/19869	7.68900325522688e-07	1.54933415592822e-05	1.10478836246155e-05	HLA-C/HLA-B/CD74/TAPBP/HLA-A/HLA-DRB1/HLA-DRA/HLA-E	8
CC	GO:0098857	membrane microdomain	23/441	327/19869	1.25862059376585e-06	2.41535285375066e-05	1.72232291778485e-05	BIRC3/HAS2/TGFBR2/BST2/LRRK2/CDH2/MME/CAV1/PRNP/PLSCR1/ABCA1/ATP1A1/IL6ST/APP/STOM/BIRC2/GJA1/CTSD/CAPN2/KCNMA1/LAMP2/ATP1B1/SULF1	23
CC	GO:0045121	membrane raft	22/441	326/19869	4.19873147139624e-06	7.69131264987584e-05	5.48446742435968e-05	BIRC3/HAS2/TGFBR2/BST2/LRRK2/CDH2/MME/CAV1/PRNP/PLSCR1/ABCA1/ATP1A1/IL6ST/APP/STOM/BIRC2/GJA1/CTSD/CAPN2/KCNMA1/ATP1B1/SULF1	22
CC	GO:0031091	platelet alpha granule	11/441	91/19869	5.45725377844423e-06	9.56205770744792e-05	6.81844522432619e-05	TGFB2/TGFB3/SERPINE2/APLP2/CLU/APP/SERPING1/SERPINE1/TIMP1/PROS1/SPARC	11
CC	GO:0030135	coated vesicle	21/441	310/19869	6.5390942180896e-06	0.000109802290412088	7.82970491902834e-05	GAD2/B2M/HLA-C/DAB2/HLA-B/CD74/AREG/WNT5A/ADRB2/HLA-A/APP/HLA-DRB1/CD59/MYO6/LMAN1/HLA-DRA/TFRC/HBEGF/HLA-E/STON2/TMED10	21
CC	GO:0031093	platelet alpha granule lumen	9/441	67/19869	1.64770920966511e-05	0.000265610724598016	0.000189399837574137	TGFB2/TGFB3/CLU/APP/SERPING1/SERPINE1/TIMP1/PROS1/SPARC	9
CC	GO:0030658	transport vesicle membrane	16/441	222/19869	3.88867576139435e-05	0.000602744743016125	0.000429801005206744	GAD2/B2M/CPE/HLA-C/LRRK2/HLA-B/CD74/AREG/HLA-A/HLA-DRB1/CD59/LMAN1/HLA-DRA/HLA-E/TMED10/SYNGR2	16

CC	GO:0031902	late endosome membrane	13/441	155/19869	4.36961154939545e-05	0.000639887611932788	0.000456286582362939	IFITM3/RAB7B/SLC39A14/IFITM2/NDFIP2/HLA-DRB1/LAPTM4B/HLA-DRA/TMEM59/CD63/LAPTM4A/LAMP2/PLD1	13
CC	GO:0045177	apical part of cell	24/441	435/19869	4.44586926404915e-05	0.000639887611932788	0.000456286582362939	CLDN1/SLC7A11/BST2/CLCA4/SLC39A14/CDH2/SLC23A2/ADRB2/SLC5A1/MYO1B/CTSB/ATP1A1/SORBS2/APP/SLC16A1/GJA1/MSN/KCNMA1/CHL1/RDX/ATP1B1/CA2/PLD1/HSP90AA1	24
CC	GO:0030670	phagocytic vesicle membrane	9/441	77/19869	5.1127801980247e-05	0.000710500144759985	0.000506638473343283	B2M/HLA-C/HLA-B/RAB7B/RAB31/TAPBP/HLA-A/HLA-E/LAMP2	9
CC	GO:0045335	phagocytic vesicle	12/441	139/19869	6.45655934191103e-05	0.000867331138263382	0.000618470421172531	B2M/HLA-C/HLA-B/RAB7B/RAB31/ITGAV/TAPBP/ABCA1/HLA-A/VIM/HLA-E/LAMP2	12
CC	GO:0005604	basement membrane	10/441	99/19869	7.01445230583998e-05	0.000911878799759197	0.000650236156025234	LAMC2/LAMA3/LAMB3/LAMB1/FBLN1/COL17A1/AMTN/TIMP1/DST/SPARC	10
CC	GO:0070820	tertiary granule	13/441	164/19869	7.82844040719907e-05	0.000985894213781633	0.000703014549725443	B2M/CXCL1/CD47/YPEL5/STOM/CD59/LGALS3/CD58/PLAU/CTSD/CST3/LAMP2/PLD1	13
CC	GO:0043256	laminin complex	4/441	12/19869	0.000102879329336399	0.00125637484007784	0.000895886982738019	LAMC2/LAMA3/LAMB3/LAMB1	4

CC	GO:0009897	external side of plasma membrane	24/441	462/19869	0.000113094543238807	0.00134050296838939	0.000955876480006019	CLEC2B/B2M/NRCAM/TGFBR2/CD74/ITGA6/TMEM123/ITGAV/PRNP/SERPINE2/IL13RA1/ABCA1/CTSB/IL6ST/HLA-DRB1/B4GALT1/CD59/IGHG3/CAPN2/IGKC/TFRC/IGHG4/ANXA5/HLA-E	24
CC	GO:0071682	endocytic vesicle lumen	5/441	23/19869	0.000127330180378257	0.00146611607692679	0.0010454477967899	SAA1/CSF3/HSPH1/HSP90AA1/SPARC	5
CC	GO:0042470	melanosome	10/441	110/19869	0.000170112902875404	0.00171005786925914	0.00121939610371619	CTSB/ATP1A1/STOM/CTSD/TFRC/HSPA5/CD63/RAB2A/TMED10/HSP90AA1	10
CC	GO:0048770	pigment granule	10/441	110/19869	0.000170112902875404	0.00171005786925914	0.00121939610371619	CTSB/ATP1A1/STOM/CTSD/TFRC/HSPA5/CD63/RAB2A/TMED10/HSP90AA1	10
CC	GO:0045334	clathrin-coated endocytic vesicle	9/441	90/19869	0.000173705220643028	0.00171005786925914	0.00121939610371619	CD74/AREG/WNT5A/ADRB2/HLA-DRB1/MYO6/HLA-DRA/TFRC/HBEGF	9
CC	GO:0030665	clathrin-coated vesicle membrane	11/441	132/19869	0.000177153145688048	0.00171005786925914	0.00121939610371619	GAD2/DAB2/CD74/AREG/WNT5A/ADRB2/HLA-DRB1/MYO6/HLA-DRA/TFRC/HBEGF	11
CC	GO:0005765	lysosomal membrane	22/441	418/19869	0.000180169311550122	0.00171005786925914	0.00121939610371619	BST2/DAB2/IFITM3/CD74/VNN1/SLC39A14/GRN/IFITM2/STOM/HLA-DRB1/B4GALT1/LAPTM4B/MYO6/CTSD/HLA-DRA/TMEM59/CD63/LAPTM4A/RAB2A/LAMP2/MIOS/PLD1	22

CC	GO:0098852	lytic vacuole membrane	22/441	418/19869	0.000180169311550122	0.00171005786925914	0.00121939610371619	BST2/DAB2/IFITM3/CD74/VNN1/SLC39A14/GRN/IFITM2/STOM/HLA-DRB1/B4GALT1/LAPTM4B/MYO6/CTSD/HLA-DRA/TMEM59/CD63/LAPTM4A/RAB2A/LAMP2/MIOS/PLD1	22
CC	GO:0005766	primary lysosome	12/441	155/19869	0.000182894031162582	0.00171005786925914	0.00121939610371619	BST2/VNN1/GRN/FABP5/FUCA1/STOM/B4GALT1/NPC2/GM2A/DNAJC3/CD63/LAMP2	12
CC	GO:0042582	azurophil granule	12/441	155/19869	0.000182894031162582	0.00171005786925914	0.00121939610371619	BST2/VNN1/GRN/FABP5/FUCA1/STOM/B4GALT1/NPC2/GM2A/DNAJC3/CD63/LAMP2	12
CC	GO:0005793	endoplasmic reticulum-Golgi intermediate compartment	11/441	133/19869	0.000189349682629772	0.00171005786925914	0.00121939610371619	AREG/TAPBP/GALNT1/GJB2/CD59/LMAN1/HSPA5/VMP1/RAB2A/GOLGB1/TMED10	11
CC	GO:0030669	clathrin-coated endocytic vesicle membrane	8/441	72/19869	0.000190949389867646	0.00171005786925914	0.00121939610371619	CD74/AREG/WNT5A/ADRB2/HLA-DRB1/HLA-DRA/TFRC/HBEGF	8
CC	GO:0005770	late endosome	17/441	288/19869	0.000260088085369099	0.0022785977913858	0.00162480657450261	BST2/LRRK2/IFITM3/RAB7B/CD74/SLC39A14/GRN/IFITM2/NDFIP2/HLA-DRB1/LAPTM4B/HLA-DRA/TMEM59/CD63/LAPTM4A/LAMP2/PLD1	17
CC	GO:0005774	vacuolar membrane	23/441	461/19869	0.000279788138080564	0.00239903446056314	0.00171068671211633	BST2/DAB2/IFITM3/CD74/VNN1/SLC39A14/GRN/IFITM2/STOM/HLA-DRB1/B4GALT1/LAPTM4B/MYO6/CTSD/HLA-DRA/TMEM59/VMP1/CD63/LAPTM4A/RAB2A/LAMP2/MIOS/PLD1	23

CC	GO:0030176	integral component of endoplasmic reticulum membrane	12/441	164/19869	0.000308656264010341	0.00259142654992016	0.00184787631743033	B2M/HLA-C/HLA-B/CD74/TAPBP/HLA-A/HLA-DRB1/ANKLE2/HLA-DRA/ATF6/HSPA5/HLA-E	12
CC	GO:0033116	endoplasmic reticulum-Golgi intermediate compartment membrane	8/441	80/19869	0.000396419154610135	0.00326034529199764	0.00232486421500681	AREG/TAPBP/GALNT1/CD59/LMAN1/VMP1/RAB2A/TMED10	8
CC	GO:0098791	Golgi apparatus subcompartment	20/441	387/19869	0.000442338851513842	0.00351014882123489	0.00250299236828294	LRRK2/RAB7B/CD74/RAB31/MME/GRN/GALNT1/OPTN/HLA-A/MYO1B/APP/HLA-DRB1/B4GALT1/HLA-DRA/TMEM59/SULF2/ARL5B/GOLGB1/POSTN/SULF1	20
CC	GO:0005811	lipid droplet	9/441	102/19869	0.000446074751410902	0.00351014882123489	0.00250299236828294	RSAD2/SDR16C5/RDH10/CAV1/DHRS3/G0S2/FABP4/HILPDA/SYNGR2	9
CC	GO:0042613	MHC class II protein complex	4/441	17/19869	0.000452922428546437	0.00351014882123489	0.00250299236828294	B2M/CD74/HLA-DRB1/HLA-DRA	4
CC	GO:0031227	intrinsic component of endoplasmic reticulum membrane	12/441	172/19869	0.000475744979051498	0.00361745710486328	0.00257951100856125	B2M/HLA-C/HLA-B/CD74/TAPBP/HLA-A/HLA-DRB1/ANKLE2/HLA-DRA/ATF6/HSPA5/HLA-E	12

CC	GO:0016328	lateral plasma membrane	7/441	64/19869	0.000528129244351724	0.00394140899025453	0.00281051235298286	CLDN1/GJB2/MPP7/ATP1A1/SLC16A1/TACSTD2/ATP1B1	7
CC	GO:0030133	transport vesicle	21/441	423/19869	0.000543200037637468	0.00398017482123454	0.00283815522057471	GAD2/B2M/CPE/HLA-C/SPRR2A/LRRK2/HLA-B/CD74/AREG/MME/HLA-A/APP/HLA-DRB1/CD59/LMAN1/HLA-DRA/HLA-E/MFF/STON2/TMED10/SYNGR2	21
CC	GO:0009925	basal plasma membrane	15/441	254/19869	0.000586675291731518	0.00422196683156789	0.00301057057599068	SLC1A3/CLDN1/DLG2/SLC39A14/CDH2/SLC23A2/ATP1A1/SLC16A1/B4GALT1/TACSTD2/MSN/TFRC/DST/ATP1B1/HSP90AA1	15
CC	GO:0016324	apical plasma membrane	19/441	368/19869	0.000617188908596218	0.00436363386253116	0.00311158951148232	CLDN1/BST2/CLCA4/SLC39A14/CDH2/SLC23A2/ADRB2/SLC5A1/CTSB/ATP1A1/SORBS2/SLC16A1/GJA1/MSN/KCNMA1/RDX/ATP1B1/PLD1/HSP90AA1	19
CC	GO:0030027	lamellipodium	13/441	203/19869	0.00063436358731965	0.0044077332015486	0.00314303555967812	IQGAP2/MYH10/ITGAV/CDH2/MCC/SORBS2/APP/NEDD9/PTPN13/CCDC88A/SLC39A6/RDX/PALLD	13
CC	GO:0005938	cell cortex	17/441	312/19869	0.000649510426892232	0.00443648647521305	0.0031635387429363	LAMC2/MYH10/RAI14/CDH2/CAV1/AGTRAP/PLS1/RND3/MPP7/NEDD9/MYO6/MYL9/CAPN2/ASPH/KRT19/DST/RDX	17
CC	GO:0101002	ficolin-1-rich granule	12/441	185/19869	0.000907084135275589	0.00592837106874992	0.00422736137330215	YPEL5/CTSB/HSPA6/LGALS3/CD58/PYGL/CTSD/PKM/CST3/LAMP2/HSP90AA1/HSPA1A	12

CC	GO: 0030136	clathrin-coated vesicle	13/441	211/19869	0.000908125886701815	0.00592837106874992	0.00422736137330215	GAD2/DAB2/CD74/AREG/WNT5A/ADRB2/HLA-DRB1/MYO6/HLA-DRA/TFRC/HBEGF/STON2/TMED10	13
CC	GO: 0042581	specific granule	11/441	160/19869	0.000914215391892607	0.00592837106874992	0.00422736137330215	B2M/CXCL1/PLAUR/CD47/ITGAV/SLPI/STOM/CD59/PLAU/CTSD/PLD1	11
CC	GO: 0042383	sarcolemma	10/441	136/19869	0.000926767685685471	0.00592837106874992	0.00422736137330215	TGFB3/SLC27A6/CDH2/CAV1/ATP1A1/KRT19/PPP3CA/RDX/ATP1B1/SLC38A2	10
CC	GO: 0098636	protein complex involved in cell adhesion	6/441	52/19869	0.000991949453460883	0.0062461817147615	0.00445398356570429	PLAUR/ITGA6/ITGAV/LAMB1/PLAU/LGALS1	6
CC	GO: 0005769	early endosome	20/441	414/19869	0.00102031976552717	0.00632598254626845	0.00451088738443591	B2M/HLA-C/HLA-B/IFITM3/RAB31/SLC39A14/PMEPA1/MME/CAV1/ADRB2/HLA-A/SLC5A1/MYO1B/APP/LAPTM4B/HLA-DRA/TFRC/ARRDC3/HLA-E/PTPN1	20
CC	GO: 0071735	IgG immunoglobulin complex	3/441	10/19869	0.00116024941051344	0.007073943127622	0.00504423788387307	IGHG3/IGKC/IGHG4	3
CC	GO: 0045178	basal part of cell	15/441	272/19869	0.0011760649864781	0.007073943127622	0.00504423788387307	SLC1A3/CLDN1/DLG2/SLC39A14/CDH2/SLC23A2/ATP1A1/SLC16A1/B4GALT1/TACSTD2/MSN/TFRC/DST/ATP1B1/HSP90AA1	15
CC	GO: 1904090	peptidase inhibitor complex	3/441	11/19869	0.00156916470345218	0.00916483152885838	0.00653519395945759	CTSB/PLAU/SERPINE1	3

CC	GO:1905370	serine-type endopeptidase complex	3/441	11/19869	0.00156916470345218	0.00916483152885838	0.00653519395945759	CFH/PLAUR/PLAU	3
CC	GO:0035577	azurophil granule membrane	6/441	58/19869	0.00176401924043749	0.0101557107699473	0.0072417631975855	BST2/VNN1/STOM/B4GALT1/CD63/LAMP2	6
CC	GO:1904813	ficolin-1-rich granule lumen	9/441	124/19869	0.0018045667999067	0.0102428228220056	0.00730388045032659	YPEL5/CTSB/HSPA6/PYGL/CTSD/PKM/CST3/HSP90AA1/HSPA1A	9
CC	GO:0005903	brush border	8/441	102/19869	0.00196665425470978	0.0110078008978894	0.00784936566572762	SLC7A11/MME/PLS1/SLC5A1/MYO1B/B4GALT1/SLC38A2/HSP90AA1	8
CC	GO:0030018	Z disc	9/441	130/19869	0.00249378542024176	0.0136453665313135	0.00973014251808436	CRYAB/SORBS2/FBXO32/MYL9/KRT19/PPP3CA/DST/PALLD/CSRP2	9
CC	GO:0005796	Golgi lumen	8/441	106/19869	0.00250560080227594	0.0136453665313135	0.00973014251808436	DCN/LUM/GPC6/OGN/OMD/WNT5A/APP/PROS1	8
CC	GO:1905286	serine-type peptidase complex	3/441	13/19869	0.00263150348389505	0.0141263102289336	0.010073090485827	CFH/PLAUR/PLAU	3
CC	GO:0031901	early endosome membrane	11/441	183/19869	0.00266892583506235	0.0141263102289336	0.010073090485827	B2M/HLA-C/HLA-B/IFITM3/RAB31/SLC39A14/PMEPA1/CAV1/HLA-A/HLA-DRA/HLA-E	11
CC	GO:0005771	multivesicular body	6/441	63/19869	0.00269907168145878	0.0141263102289336	0.010073090485827	BST2/LRRK2/CD74/NDFIP2/LAPTM4B/CD63	6

CC	GO:0034364	high-density lipoprotein particle	4/441	27/19869	0.00280289814044241	0.0143609400713813	0.0102403986926658	SAA1/SAA2/APOL1/CLU	4
CC	GO:0030175	filopodium	8/441	108/19869	0.00281517187503504	0.0143609400713813	0.0102403986926658	IQGAP2/ITGAV/MYO1B/APP/B4GALT1/MSN/MYO6/RDX	8
CC	GO:0001772	immunological synapse	5/441	45/19869	0.00309888807105234	0.0156106486579262	0.0111315321499643	VAV3/NEDD9/HLA-DRB1/LGALS3/HLA-DRA	5
CC	GO:0098637	protein complex involved in cell-matrix adhesion	3/441	14/19869	0.00329441432219964	0.0163907280474871	0.0116877857044899	PLAUR/LAMB1/PLAU	3
CC	GO:0035579	specific granule membrane	7/441	91/19869	0.00413239557607587	0.0203092124043729	0.0144819511202659	PLAUR/CD47/ITGAV/STOM/CD59/PLAU/PLD1	7
CC	GO:0005902	microvillus	7/441	93/19869	0.00465842445325247	0.0225483375599451	0.0160786108237299	IQGAP2/LRRK2/ITGAV/MYO1B/MSN/MYO6/RDX	7
CC	GO:0031674	I band	9/441	143/19869	0.00469990162539798	0.0225483375599451	0.0160786108237299	CRYAB/SORBS2/FBXO32/MYL9/KRT19/PPP3CA/DST/PALLD/CSRP2	9
CC	GO:0016323	basolateral plasma membrane	12/441	229/19869	0.00529991932891414	0.0251278528182635	0.0179179935206633	CLDN1/DLG2/SLC39A14/CDH2/SLC23A2/ATP1A1/SLC16A1/B4GALT1/MSN/TFRC/ATP1B1/HSP90AA1	12
CC	GO:0055038	recycling endosome membrane	7/441	97/19869	0.00586009476276736	0.0274606766208749	0.01958146719 99448	B2M/HLA-C/HLA-B/OPTN/HLA-A/TFRC/HLA-E	7

CC	GO:0005802	trans-Golgi network	13/441	262/19869	0.00595292772103014	0.0275750559951166	0.019663028044056	LRRK2/RAB7B/CD74/RAB31/MME/GRN/OPTN/MYO1B/APP/HLA-DRB1/HLA-DRA/ARL5B/POSTN	13
CC	GO:0031252	cell leading edge	18/441	421/19869	0.00641159022937342	0.0293621688913351	0.0209373700074036	SAMSN1/IQGAP2/PLEK/MYH10/ITGAV/CDH2/MCC/SORBS2/APP/NEDD9/MYO6/PTPN13/VIM/CCDC88A/SLC39A6/DST/RDX/PALLD	18
CC	GO:0031904	endosome lumen	4/441	35/19869	0.00727558446855631	0.0329445004587437	0.0234918339434166	B2M/CTSB/APP/CD63	4
CC	GO:1904724	tertiary granule lumen	5/441	55/19869	0.0073689602177466	0.0329965663083542	0.0235289606952611	B2M/CXCL1/YPEL5/CTSD/CST3	5
CC	GO:0016459	myosin complex	5/441	56/19869	0.00794742702381417	0.0346334847139459	0.024696203021829	MYH10/CGNL1/MYO1B/MYO6/MYL9	5
CC	GO:0034358	plasma lipoprotein particle	4/441	36/19869	0.00804433832067979	0.0346334847139459	0.024696203021829	SAA1/SAA2/APOL1/CLU	4
CC	GO:1990777	lipoprotein particle	4/441	36/19869	0.00804433832067979	0.0346334847139459	0.024696203021829	SAA1/SAA2/APOL1/CLU	4
CC	GO:0097449	astrocyte projection	3/441	19/19869	0.00807828179431988	0.0346334847139459	0.024696203021829	SLC7A11/GJB2/APP	3
CC	GO:1905369	endopeptidase complex	6/441	82/19869	0.00979942134053825	0.0415701768445991	0.0296425709248415	CFH/PLAUR/PSMB9/PSMB8/PLAU/CAPN2	6

CC	GO:0090575	RNA polymerase II transcription regulator complex	12/441	249/19869	0.0100222576932591	0.0420726026081604	0.0300008371739005	RUNX1/STAT1/MED21/FOSL2/HIF1A/XBP1/MAF/STAT3/ATF6/PBX1/FOS/JUN	12
CC	GO:0032994	protein-lipid complex	4/441	39/19869	0.0106641582890224	0.0436819922333997	0.031148449470336	SAA1/SAA2/APOL1/CLU	4
CC	GO:0042827	platelet dense granule	3/441	21/19869	0.0107308119878575	0.0436819922333997	0.031148449470336	LGALS3BP/CD63/LAMP2	3
CC	GO:0097038	perinuclear endoplasmic reticulum	3/441	21/19869	0.0107308119878575	0.0436819922333997	0.031148449470336	CLU/CAPN2/PIK3R1	3
CC	GO:0005581	collagen trimer	6/441	86/19869	0.0122272189487478	0.0492756923634536	0.0351371660316647	COL6A6/LUM/COL17A1/COL12A1/COL14A1/COL1A1	6
MF	GO:0005125	cytokine activity	21/438	237/18432	2.52461167864846e-07	0.000127424133638825	0.000107104338565234	CSF3/TNFSF10/TGFB2/CXCL1/BMP2/CXCL8/CXCL6/CXCL2/CXCL3/INHBA/TGFB3/AREG/WNT5A/GRN/IL1A/IL1B/IL1RN/CXCL16/CCL20/TIMP1/WNT2B	21
MF	GO:0030546	signaling receptor activator activity	32/438	498/18432	3.80370548175597e-07	0.000127424133638825	0.000107104338565234	CSF3/TNFSF10/TGFB2/CXCL1/BMP2/CXCL8/CXCL6/CXCL2/CXCL3/TYMP/PDGFD/OGN/INHBA/TGFB3/AREG/WNT5A/GRN/IL1A/OSGIN1/IL1B/IL1RN/CXCL16/APP/CCL20/LGALS3/PTHLH/HBEGF/TIMP1/SEMA6D/WNT2B/NPPC/MDK	32
MF	GO:0005201	extracellular matrix structural constituent	17/438	173/18432	8.39489833149934e-07	0.000141691963817086	0.000119096937379640	DCN/COL6A6/LAMC2/LAMA3/LUM/LAMB3/OGN/SRPX/LAMB1/FBLN1/COL17A1/COL12A1/COL14A1/POSTN/IGFBP7/SPARC/COL1A1	17

MF	GO:0048018	receptor ligand activity	31/438	491/18432	8.45922172042305e-07	0.000141691963817086	0.00011909693737964	CSF3/TNFSF10/TGFB2/CXCL1/BMP2/CXCL8/CXCL6/CXCL2/CXCL3/TYMP/PDGFD/OGN/INHBA/TGFB3/AREG/WNT5A/GRN/IL1A/OSGIN1/IL1B/IL1RN/CXCL16/CCL20/LGALS3/PTHLH/HBEGF/TIMP1/SEMA6D/WNT2B/NPPC/MDK	31
MF	GO:0023026	MHC class II protein complex binding	7/438	27/18432	2.39983701811914e-06	0.000283331957473025	0.000238150192062951	B2M/CD74/HLA-DRB1/HLA-DRA/PKM/ATP1B1/HSP90AA1	7
MF	GO:0005126	cytokine receptor binding	21/438	273/18432	2.53730111169873e-06	0.000283331957473025	0.000238150192062951	CSF3/TNFSF10/TGFB2/CXCL1/TGFBR2/CXCL8/CXCL6/CXCL2/CXCL3/TGFB3/STAT1/IL1A/IL1B/IL1RN/IL13RA1/IL6ST/CXCL16/CCL20/JAK1/CCDC88A/PIK3R1	21
MF	GO:0051087	chaperone binding	12/438	105/18432	7.39450746715099e-06	0.000645363438819592	0.000542450023202642	HLA-B/GRN/PRNP/ATP1A1/CLU/DNAJB9/BIRC2/TFRC/HSPA5/DNAJC3/DNAJB4/DNAJB1	12
MF	GO:0008083	growth factor activity	15/438	162/18432	7.70583210530856e-06	0.000645363438819592	0.000542450023202642	CSF3/TGFB2/CXCL1/BMP2/TYMP/PDGFD/OGN/INHBA/TGFB3/AREG/GRN/OSGIN1/HBEGF/TIMP1/MDK	15
MF	GO:0023023	MHC protein complex binding	7/438	36/18432	1.87530837493693e-05	0.00139606290134194	0.00117343857379094	B2M/CD74/HLA-DRB1/HLA-DRA/PKM/ATP1B1/HSP90AA1	7
MF	GO:0042605	peptide antigen binding	7/438	37/18432	2.26595457282108e-05	0.00151818956379013	0.00127609020679924	HLA-C/HLA-B/TAPBP/HLA-A/HLA-DRB1/HLA-DRA/HLA-E	7
MF	GO:0042379	chemokine receptor binding	9/438	71/18432	4.49722836036982e-05	0.00262042540692691	0.00220255709773118	CXCL1/CXCL8/CXCL6/CXCL2/CXCL3/STAT1/CXCL16/CCL20/JAK1	9

MF	GO:0045236	CXCR chemokine receptor binding	5/438	18/18432	4.91340372771132e-05	0.00262042540692691	0.00220255709773118	CXCL1/CXCL8/CXCL6/CXCL2/CXCL3	5
MF	GO:0051787	misfolded protein binding	6/438	29/18432	5.19572517900859e-05	0.00262042540692691	0.00220255709773118	CLU/DNAJB9/HSPA6/HSPA5/DNAJC3/HSPA1A	6
MF	GO:0008236	serine-type peptidase activity	15/438	192/18432	5.74341808216973e-05	0.00262042540692691	0.00220255709773118	MMP13/MMP7/MMP10/MMP12/C1S/MMP1/TMPRSS11A/C1R/HTRA1/TMPRSS11E/SCPEP1/CPD/PLAU/CTSV/TMPRSS11F	15
MF	GO:0042608	T cell receptor binding	4/438	10/18432	5.89567843906677e-05	0.00262042540692691	0.00220255709773118	HLA-A/HLA-DRB1/HLA-DRA/HLA-E	4
MF	GO:0005520	insulin-like growth factor binding	5/438	19/18432	6.53853059001521e-05	0.00262042540692691	0.00220255709773118	ITGA6/ITGAV/IGFBP3/IGFBP5/IGFBP7	5
MF	GO:0017171	serine hydrolase activity	15/438	196/18432	7.26411988270063e-05	0.00262042540692691	0.00220255709773118	MMP13/MMP7/MMP10/MMP12/C1S/MMP1/TMPRSS11A/C1R/HTRA1/TMPRSS11E/SCPEP1/CPD/PLAU/CTSV/TMPRSS11F	15
MF	GO:0140677	molecular function activator activity	8/438	59/18432	7.31477023493873e-05	0.00262042540692691	0.00220255709773118	HTRA1/PRNP/CITED2/NR3C1/PPP3CA/DNAJB4/ATP1B1/DNAJB1	8
MF	GO:0019838	growth factor binding	12/438	132/18432	7.4310571241211e-05	0.00262042540692691	0.00220255709773118	TGFBR2/TGFB3/ITGA6/HTRA1/ITGAV/IGFBP3/IL1RN/IL6ST/IGFBP5/S100A13/IGFBP7/COL1A1	12
MF	GO:0004175	endopeptidase activity	24/438	426/18432	9.12977716598314e-05	0.00305847535060435	0.00257075304410578	MMP13/MMP7/MMP10/MMP12/ADAM28/MMP28/PAPPA/C1S/MMP1/TMPRSS11A/C1R/CLCA4/PSMB9/HTRA1/TMPRSS11E/MME/CTSB/PSMB8/PLAU/CTSV/CTSD/CAPN2/TMEM59/TMPRSS11F	24

MF	GO:0004866	endopeptidase inhibitor activity	14/438	180/18432	0.000105715897463264	0.00337284053811367	0.00283498772645846	BIRC3/BST2/PRNP/SERPINE2/TNFAIP8/APLP2/SLPI/APP/BIRC2/SERPING1/SERPINE1/TIMP1/CST3/PROS1	14
MF	GO:0070851	growth factor receptor binding	12/438	140/18432	0.000130881373442135	0.003864549313791	0.00324828575471828	CSF3/PDGFD/VAV3/AREG/IL1A/PLSCR1/IL1B/IL1RN/IL6ST/APP/CCDC88A/HBEGF	12
MF	GO:0042277	peptide binding	20/438	330/18432	0.000132663633159992	0.003864549313791	0.00324828575471828	TGFB2/HLA-C/HLA-B/INHBA/CD74/CRYAB/PPIC/MME/TAPBP/PRNP/ADRB2/NFKBIA/HLA-A/CLU/HLA-DRB1/HLA-DRA/HLA-E/PPP3CA/CST3/PIK3R1	20
MF	GO:0061134	peptidase regulator activity	16/438	232/18432	0.000143894658923248	0.00396409596365114	0.00333195811555909	BIRC3/BST2/FBLN1/CAV1/PRNP/SERPINE2/TNFAIP8/APLP2/SLPI/APP/BIRC2/SERPING1/SERPINE1/TIMP1/CST3/PROS1	16
MF	GO:0008009	chemokine activity	7/438	49/18432	0.000147914028494445	0.00396409596365114	0.00333195811555909	CXCL1/CXCL8/CXCL6/CXCL2/CXCL3/CXCL16/CCL20	7
MF	GO:0030414	peptidase inhibitor activity	14/438	187/18432	0.000158325947673226	0.00407993788234853	0.00342932720668729	BIRC3/BST2/PRNP/SERPINE2/TNFAIP8/APLP2/SLPI/APP/BIRC2/SERPING1/SERPINE1/TIMP1/CST3/PROS1	14
MF	GO:0019955	cytokine binding	12/438	144/18432	0.000170902390743319	0.00423571261625097	0.00356026119354952	TGFBR2/TGFB3/CD74/IL20RB/ITGAV/IL1RN/IL13RA1/IFNGR1/IL6ST/GBP1/ZFP36/SOSTDC1	12

MF	GO:0031072	heat shock protein binding	11/438	124/18432	0.000183069047300323	0.00423571261625097	0.00356026119354952	DNAJB9/HSPA6/HIF1A/GBP1/NR3C1/TFRC/HSPA5/FKBP5/ZFP36/HSPA1A/DNAJB1	11
MF	GO:0005539	glycosaminoglycan binding	16/438	237/18432	0.000183803576411318	0.00423571261625097	0.00356026119354952	CFH/SAA1/DCN/LAMC2/TGFBR2/CXCL8/CXCL6/PRNP/SERPINE2/APLP2/APP/SULF2/HBEGF/POSTN/SULF1/MDK	16
MF	GO:0031994	insulin-like growth factor I binding	4/438	13/18432	0.000189658773861984	0.00423571261625097	0.00356026119354952	ITGA6/ITGAV/IGFBP3/IGFBP5	4
MF	GO:0004857	enzyme inhibitor activity	22/438	395/18432	0.000208934658122771	0.00451568454652442	0.0037955871679349	BIRC3/BST2/IQGAP2/LRRK2/PRNP/SERPINE2/TNFAIP8/APLP2/SLPI/APP/BIRC2/SERPING1/LGALS3/WNK1/TRIB1/SERPINE1/DNAJC3/ANXA5/TIMP1/CST3/PROS1/TXNIP	22
MF	GO:0005518	collagen binding	8/438	69/18432	0.000224297846411907	0.00469623615924931	0.00394734696810429	MMP13/DCN/MMP12/LUM/SPARCL1/CTSB/COL14A1/SPARC	8
MF	GO:0061135	endopeptidase regulator activity	14/438	194/18432	0.000232192931934892	0.00471422013322357	0.00396246311276451	BIRC3/BST2/PRNP/SERPINE2/TNFAIP8/APLP2/SLPI/APP/BIRC2/SERPING1/SERPINE1/TIMP1/CST3/PROS1	14
MF	GO:0004252	serine-type endopeptidase activity	13/438	174/18432	0.000274968334989322	0.00541849366008369	0.00455442907799651	MMP13/MMP7/MMP10/MMP12/C1S/MMP1/TMPRSS11A/C1R/HTRA1/TMPRSS11E/PLAU/CTSV/TMPRSS11F	13

MF	GO:0033218	amide binding	22/438	408/18432	0.000327308146921269	0.00626561309820715	0.00526646191286854	TGFB2/HLA-C/HLA-B/INHBA/CD74/CRYAB/PPIC/MME/TAPBP/PRNP/ADRB2/NFKBIA/HLA-A/CLU/HLA-DRB1/LAPTM4B/HLA-DRA/FKBP5/HLA-E/PPP3CA/CST3/PIK3R1	22
MF	GO:0004222	metalloendopeptidase activity	10/438	112/18432	0.000338510413368822	0.00630005491547529	0.00529541143720233	MMP13/MMP7/MMP10/MMP12/ADAM28/MMP28/PAPPA/MMP1/CLCA4/MME	10
MF	GO:0046332	SMAD binding	8/438	78/18432	0.000522431511501067	0.00946024628934364	0.00795166027462505	TGFBR2/BMP2/DAB2/PMEPA1/SKIL/CITED2/FOS/JUN	8
MF	GO:0061629	RNA polymerase II-specific DNA-binding transcription factor binding	19/438	350/18432	0.000757318468795192	0.0130383677264526	0.0109591936113938	BHLHE41/PITX2/EPAS1/NCOA7/ETS2/SDR16C5/BHLHE40/ID2/STAT1/NFKBIA/NR4A2/RBBP8/HIF1A/TRIB1/CITED2/STAT3/IFI27/FOS/JUN	19
MF	GO:0051082	unfolded protein binding	10/438	124/18432	0.000758949763181567	0.0130383677264526	0.0109591936113938	CRYAB/TAPBP/CLU/HSPA6/LMAN1/HSPA5/DNAJB4/HSP90AA1/HSPA1A/DNAJB1	10
MF	GO:0005154	epidermal growth factor receptor binding	5/438	33/18432	0.00101499822404501	0.0170012202527539	0.0142901065753705	VAV3/AREG/PLSCR1/CCDC88A/HBEGF	5
MF	GO:0044183	protein folding chaperone	7/438	68/18432	0.00113418296234889	0.0185342093847257	0.0155786363249462	CD74/HSPA6/HSPA5/HSPH1/HSP90AA1/HSPA1A/DNAJB1	7

MF	GO:0016595	glutamate binding	3/438	10/18432	0.00141210772245085	0.0220026086986528	0.0184939444678386	GAD2/SLC1A3/GCLC	3
MF	GO:0050786	RAGE receptor binding	3/438	10/18432	0.00141210772245085	0.0220026086986528	0.0184939444678386	S100A9/S100A8/S100A13	3
MF	GO:0008237	metallopeptidase activity	12/438	184/18432	0.00153932199325996	0.0234396758064585	0.0197018484783273	MMP13/MMP7/MMP10/MMP12/ADAM28/MMP28/PAPPA/CPE/MMP1/CLCA4/MME/CPD	12
MF	GO:0043177	organic acid binding	10/438	137/18432	0.00162516051372099	0.0241968343154014	0.0203382660781457	GAD2/SLC1A3/ASS1/S100A9/S100A8/TDO2/GCLC/P4HA2/FABP5/CRABP2	10
MF	GO:0044389	ubiquitin-like protein ligase binding	17/438	318/18432	0.00166210410572566	0.0242089076268738	0.0203484141089984	UBE2L6/STAT1/NFKBIA/CLU/HSPA6/HIF1A/TRIB1/JAK1/LAPTM4B/ISG15/ATF6/HSPA5/JUN/TXNIP/HSP90AA1/DIO2/HSPA1A	17
MF	GO:0140416	transcription regulator inhibitor activity	4/438	23/18432	0.00194592300335651	0.0277397534521034	0.0233162106785158	ID2/ID1/HSPA1A/DNAJB1	4
MF	GO:0050840	extracellular matrix binding	6/438	56/18432	0.00206979792852765	0.0288909294190317	0.0242838134158397	DCN/SPARCL1/ITGAV/LGALS3/SPARC/LGALS1	6
MF	GO:0031625	ubiquitin protein ligase binding	16/438	299/18432	0.00222783187681628	0.0304621909687124	0.025604512440316	UBE2L6/NFKBIA/CLU/HSPA6/HIF1A/TRIB1/JAK1/LAPTM4B/ISG15/ATF6/HSPA5/JUN/TXNIP/HSP90AA1/DIO2/HSPA1A	16
MF	GO:0086080	protein binding involved in heterotypic cell-cell adhesion	3/438	12/18432	0.00249880713661314	0.0334840156306161	0.0281444593281691	NRCAM/CD47/DSG2	3

MF	GO:0008201	heparin binding	11/438	171/18432	0.00265230051393628	0.0346843356359068	0.0291533693090497	CFH/SAA1/LAMC2/CXCL8/CXCL6/SERPINE2/APLP2/APP/HBEGF/POSTN/MDK	11
MF	GO:0030020	extracellular matrix structural constituent conferring tensile strength	5/438	41/18432	0.00274368625179561	0.0346843356359068	0.0291533693090497	COL6A6/COL17A1/COL12A1/COL14A1/COL1A1	5
MF	GO:0140662	ATP-dependent protein folding chaperone	5/438	41/18432	0.00274368625179561	0.0346843356359068	0.0291533693090497	HSPA6/HSPA5/HSPH1/HSP90AA1/HSPA1A	5
MF	GO:0008307	structural constituent of muscle	5/438	42/18432	0.00305471527336618	0.037901096910284	0.0318571670809143	SORBS2/MYL9/ASPH/KRT19/CSRP2	5
MF	GO:0052650	NADP-retinol dehydrogenase activity	3/438	14/18432	0.00399091457703232	0.0465138503746444	0.0390964806762525	RDH10/DHRS3/AKR1B1	3
MF	GO:0005200	structural constituent of cytoskeleton	8/438	107/18432	0.00400781536693759	0.0465138503746444	0.0390964806762525	PLS1/KRT15/SORBS2/HLA-DRB1/KRT14/MSN/VIM/KRT19	8
MF	GO:0001664	G protein-coupled receptor binding	15/438	289/18432	0.00401258438107648	0.0465138503746444	0.0390964806762525	SAA1/CXCL1/CXCL8/CXCL6/CXCL2/CXCL3/STAT1/WNT5A/PRNP/CXCL16/CCL20/JAK1/ARRDC3/WNT2B/HSPA1A	15
MF	GO:0001540	amyloid-beta binding	7/438	85/18432	0.00409599577925973	0.0465138503746444	0.0390964806762525	TGFB2/CD74/CRYAB/PRNP/ADRB2/CLU/CST3	7
MF	GO:0051117	ATPase binding	7/438	85/18432	0.00409599577925973	0.0465138503746444	0.0390964806762525	FXYD3/CAV1/ABCA1/PPP3CA/RDX/ATP1B1/DNAJB1	7

2
