# Supplementary material for: Multiomics Reveals IL-17 Drives Epithelial Keratinization and Proliferation via EHF in Odontogenic Keratocysts
Source: Int J Mol Sci. 2026 May 4;27(9):4115. doi: 10.3390/ijms27094115 (PMC13163638; doi:10.3390/ijms27094115)
Supplement: Supplementary file 1 [file ijms-27-04115-s001.zip › ijms-4235677-supplementary/Supplementary Table S8.pdf]

**Supplementary Table S8. EpC1 KEGG enrichment.**

| ID | Description | GeneRatio | BgRatio | pvalue | p.adjust | qvalue | geneID | Count |
|---|---|---|---|---|---|---|---|---|
| hsa03010 | Ribosome | 56/187 | 167/8577 | 2.40294186338981e-53 | 5.98332523984062e-51 | 5.8429428467689e-51 | RPS27/RPL3/RPL30/RPL34/RPL23A/RPL13A/RPL31/RPL5/RPL11/RPS3A/RPL13/RPS3/RSL24D1/RPL23/RPL15/RPL12/RPS6/RPL9/RPL21/RPL10/RPL7/RPS15/RPL37/RPL18A/RPLP1/RPS19/RPS11/RPL4/RPS18/RPS5/RPL35A/RPS25/RPS23/RPL6/RPL24/RPS4X/RPL18/RPL14/RPL26/RPS20/RPS27A/RPL10A/RPS15A/RPS9/RPS24/RPL19/RPS14/RPS13/RPL39/RPL22/RPL32/RPS7/RPS2/RPS21/RPS16/RPS28 | 56 |
| hsa05171 | Coronavirus disease - COVID-19 | 59/187 | 232/8577 | 2.34698283071863e-48 | 2.92199362424469e-46 | 2.85343702050528e-46 | RPS27/RPL3/RPL30/RPL34/FOS/RPL23A/RPL13A/RPL31/RPL5/RPL11/RPS3A/RPL13/RPS3/JUN/RSL24D1/RPL23/RPL15/RPL12/RPS6/RPL9/RPL21/RPL10/RPL7/RPS15/RPL37/RPL18A/RPLP1/RPS19/RPS11/RPL4/RPS18/RPS5/RPL35A/RPS25/RPS23/RPL6/RPL24/RPS4X/RPL18/RPL14/RPL26/RPS20/RPS27A/RPL10A/RPS15A/RPS9/RPS24/RPL19/PIK3R1/RPS14/RPS13/RPL39/RPL22/RPL32/RPS7/RPS2/RPS21/RPS16/RPS28 | 59 |
| hsa04510 | Focal adhesion | 12/187 | 203/8577 | 0.0015837404750933 | 0.0930085971647996 | 0.0908264043334124 | ITGB1/TNC/CCND2/JUN/LAMB3/ARHGAP5/ITGA2/CAV1/PIK3R1/ITGB4/LAMA3/FLNA | 12 |
| hsa04216 | Ferroptosis | 5/187 | 41/8577 | 0.00185096796163721 | 0.0930085971647996 | 0.0908264043334124 | FTH1/SLC3A2/ACSL4/PCBP2/FTL | 5 |
| hsa04978 | Mineral absorption | 6/187 | 60/8577 | 0.00186764251334939 | 0.0930085971647996 | 0.0908264043334124 | FTH1/ATP1B3/MT1F/CYBRD1/MT1E/FTL | 6 |
| hsa05205 | Proteoglycans in cancer | 11/187 | 205/8577 | 0.00515981883112393 | 0.214132481491643 | 0.209108447366601 | PTCH1/ITGB1/SDC2/EIF4B/RPS6/GPC3/ITGA2/CAV1/PIK3R1/FZD3/FLNA | 11 |

| | | | | | | | | |
|---|---|---|---|---|---|---|---|---|
| hsa 043 50 | TGF-beta signaling pathway | 7/187 | 108/ 8577 | 0.00917 406879 86346 | 0.31336 3055991 895 | 0.30601 084732 2459 | BMP5/EMP3/TGIF2/TGIF1/ID3/FST/RGMA | 7 |
| hsa 049 15 | Estrogen signaling pathway | 8/187 | 137/ 8577 | 0.01006 788934 91372 | 0.31336 3055991 895 | 0.30601 084732 2459 | KRT15/FOS/JUN/KRT14/PIK3R1/PLCB4/CREB5/HSPA1A | 8 |
| hsa 045 12 | ECM-receptor interaction | 6/187 | 89/8 577 | 0.01292 093696 65305 | 0.35747 9256074 01 | 0.34909 198120 0999 | ITGB1/TNC/LAMB3/ITGA2/ITGB4/LAMA3 | 6 |
| hsa 003 30 | Arginine and proline metabolism | 4/187 | 50/8 577 | 0.02310 745383 15097 | 0.46404 4598971 868 | 0.45315 705923 6955 | ALDH3A2/CKMT1B/PYCR2/OAT | 4 |
| hsa 051 46 | Amoebiasis | 6/187 | 102/ 8577 | 0.02379 156353 0459 | 0.46404 4598971 868 | 0.45315 705923 6955 | IL1R2/LAMB3/ITGB2/PIK3R1/PLCB4/LAMA3 | 6 |
| hsa 049 74 | Protein digestion and absorption | 6/187 | 103/ 8577 | 0.02482 181209 14503 | 0.46404 4598971 868 | 0.45315 705923 6955 | COL17A1/ATP1B3/SLC7A8/SLC3A2/COL18A1/COL7A1 | 6 |
| hsa 041 51 | PI3K-Akt signaling pathway | 14/18 7 | 359/ 8577 | 0.02505 988368 58174 | 0.46404 4598971 868 | 0.45315 705923 6955 | NR4A1/ITGB1/F2R/TNC/CCND2/EIF4B/LAMB3/RPS6/ITGA2/FGFR2/PIK3R1/CREB5/ITGB4/ LAMA3 | 14 |
| hsa 051 40 | Leishmaniasis | 5/187 | 77/8 577 | 0.02609 086098 63701 | 0.46404 4598971 868 | 0.45315 705923 6955 | ITGB1/FOS/EEF1A1/JUN/ITGB2 | 5 |
| hsa 051 62 | Measles | 7/187 | 138/ 8577 | 0.03109 118814 89806 | 0.50049 2599505 01 | 0.48874 990693 5774 | FOS/CCND2/JUN/RACK1/EIF3H/PIK3R1/HSPA1A | 7 |

| | | | | | | | | |
|---|---|---|---|---|---|---|---|---|
| hsa 05418 | Fluid shear stress and atherosclerosis | 7/187 | 139/8577 | 0.0321601670364665 | 0.5004925995050 1 | 0.488749906935774 | IL1R2/FOS/EDN1/SDC2/JUN/CAV1/PIK3R1 | 7 |
| hsa 04550 | Signaling pathways regulating pluripotency of stem cells | 7/187 | 143/8577 | 0.0366819082950939 | 0.527863288688918 | 0.515478417616318 | SMARCAD1/DLX5/TBX3/ID3/FGFR2/PIK3R1/FZD3 | 7 |
| hsa 04668 | TNF signaling pathway | 6/187 | 114/8577 | 0.0381587919534157 | 0.527863288688918 | 0.515478417616318 | FOS/EDN1/JUNB/JUN/PIK3R1/CREB5 | 6 |
| hsa 05222 | Small cell lung cancer | 5/187 | 92/8577 | 0.0502217768474658 | 0.658169601843105 | 0.642727448851225 | ITGB1/LAMB3/ITGA2/PIK3R1/LAMA3 | 5 |
| hsa 05166 | Human T-cell leukemia virus 1 infection | 9/187 | 222/8577 | 0.0536996801501821 | 0.668561017869767 | 0.652875058668003 | IL1R2/FOS/CCND2/JUN/EGR1/ITGB2/SLC25A6/PIK3R1/CREB5 | 9 |
| hsa 04024 | cAMP signaling pathway | 9/187 | 225/8577 | 0.0575098510435647 | 0.681902519516553 | 0.665903538399171 | HHIP/PTCH1/ATP1B3/FOS/EDN1/F2R/JUN/PIK3R1/CREB5 | 9 |
| hsa 04926 | Relaxin signaling pathway | 6/187 | 129/8577 | 0.0626338172009139 | 0.708900931046707 | 0.692268505904838 | FOS/EDN1/JUN/PIK3R1/PLCB4/CREB5 | 6 |
| hsa 04933 | AGE-RAGE signaling pathway in | 5/187 | 100/8577 | 0.0671062445638996 | 0.726498038974391 | 0.709452745732 76 | EDN1/JUN/EGR1/PIK3R1/PLCB4 | 5 |

| | | | | | | | | |
|---|---|---|---|---|---|---|---|---|
| | diabetic complications | | | | | | | |
| hsa04928 | Parathyroid hormone synthesis, secretion and action | 5/187 | 106/8577 | 0.0816052484555912 | 0.795314004441208 | 0.776654132428319 | FOS/EGR1/RUNX2/PLCB4/CREB5 | 5 |
| hsa04973 | Carbohydrate digestion and absorption | 3/187 | 47/8577 | 0.0823468662993591 | 0.795314004441208 | 0.776654132428319 | ATP1B3/PIK3R1/PLCB4 | 3 |
| hsa05133 | Pertussis | 4/187 | 76/8577 | 0.0838380784094352 | 0.795314004441208 | 0.776654132428319 | ITGB1/FOS/JUN/ITGB2 | 4 |
| hsa05412 | Arrhythmogenic right ventricular cardiomyopathy | 4/187 | 77/8577 | 0.0870023095011101 | 0.795314004441208 | 0.776654132428319 | ITGB1/DSG2/ITGA2/ITGB4 | 4 |
| hsa04066 | HIF-1 signaling pathway | 5/187 | 109/8577 | 0.0894329000978065 | 0.795314004441208 | 0.776654132428319 | LDHB/EDN1/RPS6/TIMP1/PIK3R1 | 5 |
| hsa01521 | EGFR tyrosine kinase inhibitor resistance | 4/187 | 79/8577 | 0.0935022817559021 | 0.802829936455849 | 0.783993723615 73 | NF1/RPS6/FGFR2/PIK3R1 | 4 |
| hsa04360 | Axon guidance | 7/187 | 182/8577 | 0.102817987323661 | 0.853389294786388 | 0.833366844623359 | PTCH1/ITGB1/SEMA3C/PIK3R1/FZD3/RGMA/NCK1 | 7 |

| | | | | | | | | |
|---|---|---|---|---|---|---|---|---|
| hsa 030 82 | ATP-dependent chromatin remodeling | 5/187 | 117/ 8577 | 0.11212 428381 9611 | 0.85575 7121386 436 | 0.83567 911663 6088 | CHD3/MORF4L1/SMARCD3/BCL7A/SMARCA1 | 5 |
| hsa 049 35 | Growth hormone synthesis, secretion and action | 5/187 | 120/ 8577 | 0.12128 844046 1517 | 0.85575 7121386 436 | 0.83567 911663 6088 | FOS/JUNB/PIK3R1/PLCB4/CREB5 | 5 |
| hsa 043 40 | Hedgehog signaling pathway | 3/187 | 56/8 577 | 0.12259 801552 5678 | 0.85575 7121386 436 | 0.83567 911663 6088 | HHIP/PTCH1/CCND2 | 3 |
| hsa 051 34 | Legionellosis | 3/187 | 56/8 577 | 0.12259 801552 5678 | 0.85575 7121386 436 | 0.83567 911663 6088 | EEF1A1/ITGB2/HSPA1A | 3 |
| hsa 041 50 | mTOR signaling pathway | 6/187 | 156/ 8577 | 0.12548 407539 6548 | 0.85575 7121386 436 | 0.83567 911663 6088 | SLC3A2/EIF4B/RPS6/RPS6KA3/PIK3R1/FZD3 | 6 |
| hsa 048 10 | Regulation of actin cytoskeleton | 8/187 | 229/ 8577 | 0.12743 796651 5883 | 0.85575 7121386 436 | 0.83567 911663 6088 | GSN/ITGB1/F2R/ITGA2/ITGB2/FGFR2/PIK3R1/ITGB4 | 8 |
| hsa 042 11 | Longevity regulating pathway | 4/187 | 89/8 577 | 0.12924 006208 7758 | 0.85575 7121386 436 | 0.83567 911663 6088 | PIK3R1/SESN3/CAT/CREB5 | 4 |
| hsa 054 10 | Hypertrophic cardiomyopathy | 4/187 | 90/8 577 | 0.13309 118037 9773 | 0.85575 7121386 436 | 0.83567 911663 6088 | ITGB1/EDN1/ITGA2/ITGB4 | 4 |

| | | | | | | | | |
|---|---|---|---|---|---|---|---|---|
| hsa04611 | Platelet activation | 5/187 | 124/8577 | 0.134034247927996 | 0.855757121386436 | 0.835679116636088 | ITGB1/F2R/ITGA2/PIK3R1/PLCB4 | 5 |
| hsa04380 | Osteoclast differentiation | 5/187 | 128/8577 | 0.147354755904912 | 0.895850697499462 | 0.874832006435747 | FOS/JUNB/JUN/FHL2/PIK3R1 | 5 |
| hsa04213 | Longevity regulating pathway - multiple species | 3/187 | 61/8577 | 0.147509552600313 | 0.895850697499462 | 0.874832006435747 | PIK3R1/CAT/HSPA1A | 3 |
| hsa05217 | Basal cell carcinoma | 3/187 | 63/8577 | 0.157902936712365 | 0.932450173704656 | 0.910572775843482 | HHIP/PTCH1/FZD3 | 3 |
| hsa05215 | Prostate cancer | 4/187 | 97/8577 | 0.161308917097491 | 0.932450173704656 | 0.910572775843482 | IL1R2/FGFR2/PIK3R1/CREB5 | 4 |
| hsa04925 | Aldosterone synthesis and secretion | 4/187 | 98/8577 | 0.165508392212612 | 0.932450173704656 | 0.910572775843482 | NR4A1/ATP1B3/PLCB4/CREB5 | 4 |
| hsa04927 | Cortisol synthesis and secretion | 3/187 | 65/8577 | 0.168515091633372 | 0.932450173704656 | 0.910572775843482 | NR4A1/PLCB4/CREB5 | 3 |
| hsa05135 | Yersinia infection | 5/187 | 137/8577 | 0.179255059927712 | 0.933076864943605 | 0.911184763483293 | ITGB1/FOS/JUN/RPS6KA3/PIK3R1 | 5 |

| | | | | | | | | |
|---|---|---|---|---|---|---|---|---|
| hsa05142 | Chagas disease | 4/187 | 102/8577 | 0.182687321717949 | 0.933076864943605 | 0.911184763483293 | FOS/JUN/PIK3R1/PLCB4 | 4 |
| hsa05165 | Human papillomavirus infection | 10/187 | 331/8577 | 0.186139743807647 | 0.933076864943605 | 0.911184763483293 | ITGB1/TNC/CCND2/LAMB3/ITGA2/PIK3R1/CREB5/FZD3/ITGB4/LAMA3 | 10 |
| hsa05031 | Amphetamine addiction | 3/187 | 69/8577 | 0.190326544169063 | 0.933076864943605 | 0.911184763483293 | FOS/JUN/CREB5 | 3 |
| hsa04960 | Aldosterone-regulated sodium reabsorption | 2/187 | 37/8577 | 0.192656286790132 | 0.933076864943605 | 0.911184763483293 | ATP1B3/PIK3R1 | 2 |
| hsa04917 | Prolactin signaling pathway | 3/187 | 70/8577 | 0.195889290233624 | 0.933076864943605 | 0.911184763483293 | FOS/CCND2/PIK3R1 | 3 |
| hsa05230 | Central carbon metabolism in cancer | 3/187 | 70/8577 | 0.195889290233624 | 0.933076864943605 | 0.911184763483293 | LDHB/FGFR2/PIK3R1 | 3 |
| hsa04137 | Mitophagy - animal | 3/187 | 72/8577 | 0.207131975564620 | 0.933076864943605 | 0.911184763483293 | JUN/UBB/RPS27A | 3 |
| hsa04010 | MAPK signaling pathway | 9/187 | 301/8577 | 0.210796442867814 | 0.933076864943605 | 0.911184763483293 | NR4A1/FOS/JUN/NF1/IL1RAP/RPS6KA3/FGFR2/HSPA1A/FLNA | 9 |
| hsa01524 | Platinum drug resistance | 3/187 | 73/8577 | 0.212807801600132 | 0.933076864943605 | 0.911184763483293 | XPA/REV3L/PIK3R1 | 3 |

| | | | | | | | | |
|---|---|---|---|---|---|---|---|---|
| hsa05145 | Toxoplasmosis | 4/187 | 111/8577 | 0.223288968315047 | 0.933076864943605 | 0.911184763483293 | ITGB1/LAMB3/LAMA3/HSPA1A | 4 |
| hsa04918 | Thyroid hormone synthesis | 3/187 | 75/8577 | 0.224258300181025 | 0.933076864943605 | 0.911184763483293 | ATP1B3/PLCB4/CREB5 | 3 |
| hsa04725 | Cholinergic synapse | 4/187 | 113/8577 | 0.232621223377253 | 0.933076864943605 | 0.911184763483293 | FOS/PIK3R1/PLCB4/CREB5 | 4 |
| hsa00380 | Tryptophan metabolism | 2/187 | 42/8577 | 0.232813022029969 | 0.933076864943605 | 0.911184763483293 | ALDH3A2/CAT | 2 |
| hsa05100 | Bacterial invasion of epithelial cells | 3/187 | 77/8577 | 0.235827373336919 | 0.933076864943605 | 0.911184763483293 | ITGB1/CAV1/PIK3R1 | 3 |
| hsa00071 | Fatty acid degradation | 2/187 | 43/8577 | 0.240917146568267 | 0.933076864943605 | 0.911184763483293 | ACSL4/ALDH3A2 | 2 |
| hsa04670 | Leukocyte transendothelial migration | 4/187 | 115/8577 | 0.242048213438277 | 0.933076864943605 | 0.911184763483293 | ITGB1/ARHGAP5/ITGB2/PIK3R1 | 4 |
| hsa04390 | Hippo signaling pathway | 5/187 | 157/8577 | 0.25784693640457 | 0.933076864943605 | 0.911184763483293 | CCND2/BMP5/ITGB2/SNAI2/FZD3 | 5 |
| hsa04722 | Neurotrophin signaling pathway | 4/187 | 119/8577 | 0.261153812017725 | 0.933076864943605 | 0.911184763483293 | JUN/BEX3/RPS6KA3/PIK3R1 | 4 |

| | | | | | | | | |
|---|---|---|---|---|---|---|---|---|
| hsa04514 | Cell adhesion molecules | 5/187 | 158/8577 | 0.262990407169877 | 0.933076864943605 | 0.911184763483293 | SLITRK6/ITGB1/VCAN/SDC2/ITGB2 | 5 |
| hsa05130 | Pathogenic Escherichia coli infection | 6/187 | 198/8577 | 0.263481140224 17 | 0.933076864943605 | 0.911184763483293 | ITGB1/FOS/F2R/RPS3/JUN/NCK1 | 6 |
| hsa00533 | Glycosaminoglycan biosynthesis - keratan sulfate | 1/187 | 14/8577 | 0.265707822677028 | 0.933076864943605 | 0.911184763483293 | FUT8 | 1 |
| hsa04152 | AMPK signaling pathway | 4/187 | 121/8577 | 0.270816325862718 | 0.933076864943605 | 0.911184763483293 | EEF2/PIK3R1/CREB5/SCD5 | 4 |
| hsa04660 | T cell receptor signaling pathway | 4/187 | 121/8577 | 0.270816325862718 | 0.933076864943605 | 0.911184763483293 | FOS/JUN/PIK3R1/NCK1 | 4 |
| hsa04919 | Thyroid hormone signaling pathway | 4/187 | 121/8577 | 0.270816325862718 | 0.933076864943605 | 0.911184763483293 | ATP1B3/DIO2/PIK3R1/PLCB4 | 4 |
| hsa00620 | Pyruvate metabolism | 2/187 | 47/8577 | 0.273421498513524 | 0.933076864943605 | 0.911184763483293 | LDHB/ALDH3A2 | 2 |
| hsa04662 | B cell receptor signaling pathway | 3/187 | 84/8577 | 0.277032795488756 | 0.933076864943605 | 0.911184763483293 | FOS/JUN/PIK3R1 | 3 |

| | | | | | | | | |
|---|---|---|---|---|---|---|---|---|
| hsa 040 12 | ErbB signaling pathway | 3/187 | 85/8 577 | 0.28298 560378 3399 | 0.93307 6864943 605 | 0.91118 476348 3293 | JUN/PIK3R1/NCK1 | 3 |
| hsa 046 10 | Complement and coagulation cascades | 3/187 | 86/8 577 | 0.28894 976305 0961 | 0.93307 6864943 605 | 0.91118 476348 3293 | F2R/SERPINE2/ITGB2 | 3 |
| hsa 049 11 | Insulin secretion | 3/187 | 86/8 577 | 0.28894 976305 0961 | 0.93307 6864943 605 | 0.91118 476348 3293 | ATP1B3/PLCB4/CREB5 | 3 |
| hsa 052 10 | Colorectal cancer | 3/187 | 86/8 577 | 0.28894 976305 0961 | 0.93307 6864943 605 | 0.91118 476348 3293 | FOS/JUN/PIK3R1 | 3 |
| hsa 050 30 | Cocaine addiction | 2/187 | 49/8 577 | 0.28966 921467 9139 | 0.93307 6864943 605 | 0.91118 476348 3293 | JUN/CREB5 | 2 |
| hsa 051 31 | Shigellosis | 7/187 | 247/ 8577 | 0.29348 700479 8033 | 0.93307 6864943 605 | 0.91118 476348 3293 | ITGB1/JUN/UBB/RPS27A/CAST/PIK3R1/PLCB4 | 7 |
| hsa 051 44 | Malaria | 2/187 | 50/8 577 | 0.29777 886187 8415 | 0.93307 6864943 605 | 0.91118 476348 3293 | SDC2/ITGB2 | 2 |
| hsa 052 35 | PD-L1 expression and PD-1 checkpoint pathway in cancer | 3/187 | 89/8 577 | 0.30689 382329 7685 | 0.93307 6864943 605 | 0.91118 476348 3293 | FOS/JUN/PIK3R1 | 3 |

| | | | | | | | | |
|---|---|---|---|---|---|---|---|---|
| hsa04015 | Rap1 signaling pathway | 6/187 | 210/8577 | 0.308756659966998 | 0.933076864943605 | 0.911184763483293 | ITGB1/F2R/ITGB2/FGFR2/PIK3R1/PLCB4 | 6 |
| hsa04310 | Wnt signaling pathway | 5/187 | 171/8577 | 0.317057694115384 | 0.933076864943605 | 0.911184763483293 | CCND2/JUN/GPC4/PLCB4/FZD3 | 5 |
| hsa04961 | Endocrine and other factor-regulated calcium reabsorption | 2/187 | 53/8577 | 0.322010834467629 | 0.933076864943605 | 0.911184763483293 | ATP1B3/PLCB4 | 2 |
| hsa00061 | Fatty acid biosynthesis | 1/187 | 18/8577 | 0.327789944966513 | 0.933076864943605 | 0.911184763483293 | ACSL4 | 1 |
| hsa04912 | GnRH signaling pathway | 3/187 | 93/8577 | 0.330880518562366 | 0.933076864943605 | 0.911184763483293 | JUN/EGR1/PLCB4 | 3 |
| hsa05323 | Rheumatoid arthritis | 3/187 | 93/8577 | 0.330880518562366 | 0.933076864943605 | 0.911184763483293 | FOS/JUN/ITGB2 | 3 |
| hsa04210 | Apoptosis | 4/187 | 136/8577 | 0.344755056276487 | 0.933076864943605 | 0.911184763483293 | FOS/JUN/CTSV/PIK3R1 | 4 |
| hsa05150 | Staphylococcus aureus infection | 3/187 | 96/8577 | 0.348864139182047 | 0.933076864943605 | 0.911184763483293 | KRT15/ITGB2/KRT14 | 3 |

| | | | | | | | | |
|---|---|---|---|---|---|---|---|---|
| hsa05414 | Dilated cardiomyopathy | 3/187 | 96/8577 | 0.348864139182047 | 0.933076864943605 | 0.911184763483293 | ITGB1/ITGA2/ITGB4 | 3 |
| hsa01212 | Fatty acid metabolism | 2/187 | 57/8577 | 0.353982580624788 | 0.933076864943605 | 0.911184763483293 | ACSL4/SCD5 | 2 |
| hsa04070 | Phosphatidylinositol signaling system | 3/187 | 97/8577 | 0.354849944142192 | 0.933076864943605 | 0.911184763483293 | IP6K2/PIK3R1/PLCB4 | 3 |
| hsa01522 | Endocrine resistance | 3/187 | 98/8577 | 0.360829151045231 | 0.933076864943605 | 0.911184763483293 | FOS/JUN/PIK3R1 | 3 |
| hsa04750 | Inflammatory mediator regulation of TRP channels | 3/187 | 98/8577 | 0.360829151045231 | 0.933076864943605 | 0.911184763483293 | IL1RAP/PIK3R1/PLCB4 | 3 |
| hsa05231 | Choline metabolism in cancer | 3/187 | 98/8577 | 0.360829151045231 | 0.933076864943605 | 0.911184763483293 | FOS/JUN/PIK3R1 | 3 |
| hsa00770 | Pantothenate and CoA biosynthesis | 1/187 | 21/8577 | 0.370899278150282 | 0.933076864943605 | 0.911184763483293 | ALDH3A2 | 1 |
| hsa05416 | Viral myocarditis | 2/187 | 60/8577 | 0.377616706413129 | 0.933076864943605 | 0.911184763483293 | ITGB2/CAV1 | 2 |

| | | | | | | | | |
|---|---|---|---|---|---|---|---|---|
| hsa04916 | Melanogenesis | 3/187 | 101/8577 | 0.378714428349072 | 0.933076864943605 | 0.911184763483293 | EDN1/PLCB4/FZD3 | 3 |
| hsa00340 | Histidine metabolism | 1/187 | 22/8577 | 0.384648908232785 | 0.933076864943605 | 0.911184763483293 | ALDH3A2 | 1 |
| hsa04620 | Toll-like receptor signaling pathway | 3/187 | 104/8577 | 0.396495710328613 | 0.933076864943605 | 0.911184763483293 | FOS/JUN/PIK3R1 | 3 |
| hsa04964 | Proximal tubule bicarbonate reclamation | 1/187 | 23/8577 | 0.398099598374277 | 0.933076864943605 | 0.911184763483293 | ATP1B3 | 1 |
| hsa05224 | Breast cancer | 4/187 | 147/8577 | 0.399435704721638 | 0.933076864943605 | 0.911184763483293 | FOS/JUN/PIK3R1/FZD3 | 4 |
| hsa04929 | GnRH secretion | 2/187 | 64/8577 | 0.408556691279741 | 0.933076864943605 | 0.911184763483293 | PIK3R1/PLCB4 | 2 |
| hsa05202 | Transcriptional misregulation in cancer | 5/187 | 193/8577 | 0.412556131217441 | 0.933076864943605 | 0.911184763483293 | IL1R2/CCND2/RUNX2/FUT8/SIX1 | 5 |
| hsa04922 | Glucagon signaling pathway | 3/187 | 107/8577 | 0.414142307523414 | 0.933076864943605 | 0.911184763483293 | LDHB/PLCB4/CREB5 | 3 |

| | | | | | | | | |
|---|---|---|---|---|---|---|---|---|
| hsa05167 | Kaposi sarcoma-associated herpesvirus infection | 5/187 | 194/8577 | 0.416888456234982 | 0.933076864943605 | 0.911184763483293 | FOS/JUN/UBB/RPS27A/PIK3R1 | 5 |
| hsa04659 | Th17 cell differentiation | 3/187 | 108/8577 | 0.419989624572261 | 0.933076864943605 | 0.911184763483293 | FOS/JUN/IL1RAP | 3 |
| hsa04931 | Insulin resistance | 3/187 | 108/8577 | 0.419989624572261 | 0.933076864943605 | 0.911184763483293 | RPS6KA3/PIK3R1/CREB5 | 3 |
| hsa00010 | Glycolysis / Gluconeogenesis | 2/187 | 67/8577 | 0.431268890315419 | 0.933076864943605 | 0.911184763483293 | LDHB/ALDH3A2 | 2 |
| hsa04720 | Long-term potentiation | 2/187 | 67/8577 | 0.431268890315419 | 0.933076864943605 | 0.911184763483293 | RPS6KA3/PLCB4 | 2 |
| hsa04921 | Oxytocin signaling pathway | 4/187 | 154/8577 | 0.433883639083521 | 0.933076864943605 | 0.911184763483293 | FOS/JUN/EEF2/PLCB4 | 4 |
| hsa04934 | Cushing syndrome | 4/187 | 155/8577 | 0.438765067846944 | 0.933076864943605 | 0.911184763483293 | NR4A1/PLCB4/CREB5/FZD3 | 4 |
| hsa04218 | Cellular senescence | 4/187 | 156/8577 | 0.443634679543116 | 0.933076864943605 | 0.911184763483293 | ZFP36L2/CCND2/SLC25A6/PIK3R1 | 4 |

| | | | | | | | | |
|---|---|---|---|---|---|---|---|---|
| hsa04924 | Renin secretion | 2/187 | 69/8577 | 0.446152725343577 | 0.933076864943605 | 0.911184763483293 | EDN1/PLCB4 | 2 |
| hsa05211 | Renal cell carcinoma | 2/187 | 69/8577 | 0.446152725343577 | 0.933076864943605 | 0.911184763483293 | JUN/PIK3R1 | 2 |
| hsa01040 | Biosynthesis of unsaturated fatty acids | 1/187 | 27/8577 | 0.449040196351 65 | 0.933076864943605 | 0.911184763483293 | SCD5 | 1 |
| hsa05203 | Viral carcinogenesis | 5/187 | 204/8577 | 0.459870208194666 | 0.933076864943605 | 0.911184763483293 | GSN/CCND2/JUN/PIK3R1/CREB5 | 5 |
| hsa05161 | Hepatitis B | 4/187 | 162/8577 | 0.472571543678176 | 0.933076864943605 | 0.911184763483293 | FOS/JUN/PIK3R1/CREB5 | 4 |
| hsa04115 | p53 signaling pathway | 2/187 | 74/8577 | 0.482388619208496 | 0.933076864943605 | 0.911184763483293 | CCND2/SESN3 | 2 |
| hsa00053 | Ascorbate and aldarate metabolism | 1/187 | 30/8577 | 0.484410038199936 | 0.933076864943605 | 0.911184763483293 | ALDH3A2 | 1 |
| hsa00630 | Glyoxylate and dicarboxylate metabolism | 1/187 | 30/8577 | 0.484410038199936 | 0.933076864943605 | 0.911184763483293 | CAT | 1 |
| hsa03320 | PPAR signaling pathway | 2/187 | 75/8577 | 0.489460682743495 | 0.933076864943605 | 0.911184763483293 | ACSL4/SCD5 | 2 |

| | | | | | | | | |
|---|---|---|---|---|---|---|---|---|
| hsa05207 | Chemical carcinogenesis - receptor activation | 5/187 | 212/8577 | 0.493612119677287 | 0.933076864943605 | 0.911184763483293 | FOS/JUN/RPS6KA3/PIK3R1/CREB5 | 5 |
| hsa00410 | beta-Alanine metabolism | 1/187 | 31/8577 | 0.495690642254763 | 0.933076864943605 | 0.911184763483293 | ALDH3A2 | 1 |
| hsa04022 | cGMP-PKG signaling pathway | 4/187 | 167/8577 | 0.496258222006293 | 0.933076864943605 | 0.911184763483293 | ATP1B3/SLC25A6/PLCB4/CREB5 | 4 |
| hsa04971 | Gastric acid secretion | 2/187 | 76/8577 | 0.496472350000713 | 0.933076864943605 | 0.911184763483293 | ATP1B3/PLCB4 | 2 |
| hsa05417 | Lipid and atherosclerosis | 5/187 | 215/8577 | 0.506072467612741 | 0.933076864943605 | 0.911184763483293 | FOS/JUN/PIK3R1/PLCB4/HSPA1A | 5 |
| hsa00052 | Galactose metabolism | 1/187 | 32/8577 | 0.506725728833088 | 0.933076864943605 | 0.911184763483293 | UGP2 | 1 |
| hsa00640 | Propanoate metabolism | 1/187 | 32/8577 | 0.506725728833088 | 0.933076864943605 | 0.911184763483293 | LDHB | 1 |
| hsa04215 | Apoptosis - multiple species | 1/187 | 32/8577 | 0.506725728833088 | 0.933076864943605 | 0.911184763483293 | BOK | 1 |

| | | | | | | | | |
|---|---|---|---|---|---|---|---|---|
| hsa03018 | RNA degradation | 2/187 | 79/8577 | 0.517137891236817 | 0.933076864943605 | 0.911184763483293 | BTG2/PABPC1 | 2 |
| hsa03020 | RNA polymerase | 1/187 | 34/8577 | 0.52808049698805 | 0.933076864943605 | 0.911184763483293 | POLR1D | 1 |
| hsa05208 | Chemical carcinogenesis - reactive oxygen species | 5/187 | 223/8577 | 0.538690185680277 | 0.933076864943605 | 0.911184763483293 | FOS/JUN/SLC25A6/PIK3R1/CAT | 5 |
| hsa04146 | Peroxisome | 2/187 | 83/8577 | 0.543808243970437 | 0.933076864943605 | 0.911184763483293 | ACSL4/CAT | 2 |
| hsa05020 | Prion disease | 6/187 | 272/8577 | 0.54713363567396 | 0.933076864943605 | 0.911184763483293 | EGR1/SLC25A6/CAV1/PIK3R1/CREB5/HSPA1A | 6 |
| hsa04068 | FoxO signaling pathway | 3/187 | 131/8577 | 0.5475546331094 | 0.933076864943605 | 0.911184763483293 | CCND2/PIK3R1/CAT | 3 |
| hsa00040 | Pentose and glucuronate interconversions | 1/187 | 36/8577 | 0.548515507462484 | 0.933076864943605 | 0.911184763483293 | UGP2 | 1 |
| hsa00500 | Starch and sucrose metabolism | 1/187 | 36/8577 | 0.548515507462484 | 0.933076864943605 | 0.911184763483293 | UGP2 | 1 |

| | | | | | | | | |
|---|---|---|---|---|---|---|---|---|
| hsa04728 | Dopaminergic synapse | 3/187 | 132/8577 | 0.55273327458313 | 0.933076864943605 | 0.911184763483293 | FOS/PLCB4/CREB5 | 3 |
| hsa00760 | Nicotinate and nicotinamide metabolism | 1/187 | 37/8577 | 0.558400485814494 | 0.933076864943605 | 0.911184763483293 | NT5E | 1 |
| hsa01250 | Biosynthesis of nucleotide sugars | 1/187 | 37/8577 | 0.558400485814494 | 0.933076864943605 | 0.911184763483293 | UGP2 | 1 |
| hsa05143 | African trypanosomiasis | 1/187 | 37/8577 | 0.558400485814494 | 0.933076864943605 | 0.911184763483293 | PLCB4 | 1 |
| hsa04270 | Vascular smooth muscle contraction | 3/187 | 134/8577 | 0.562985497517809 | 0.933076864943605 | 0.911184763483293 | EDN1/RAMP1/PLCB4 | 3 |
| hsa04714 | Thermogenesis | 5/187 | 232/8577 | 0.574174374868474 | 0.933076864943605 | 0.911184763483293 | ACSL4/RPS6/RPS6KA3/CREB5/SMARCD3 | 5 |
| hsa04621 | NOD-like receptor signaling pathway | 4/187 | 186/8577 | 0.581702383863149 | 0.933076864943605 | 0.911184763483293 | TXNIP/JUN/PSTPIP1/PLCB4 | 4 |
| hsa04371 | Apelin signaling pathway | 3/187 | 139/8577 | 0.587987328433579 | 0.933076864943605 | 0.911184763483293 | RPS6/EGR1/PLCB4 | 3 |

| | | | | | | | | |
|---|---|---|---|---|---|---|---|---|
| hsa03440 | Homologous recombination | 1/187 | 41/8577 | 0.595833608347085 | 0.933076864943605 | 0.911184763483293 | RBBP8 | 1 |
| hsa04658 | Th1 and Th2 cell differentiation | 2/187 | 92/8577 | 0.600009450426245 | 0.933076864943605 | 0.911184763483293 | FOS/JUN | 2 |
| hsa04613 | Neutrophil extracellular trap formation | 4/187 | 191/8577 | 0.602784862288864 | 0.933076864943605 | 0.911184763483293 | ITGB2/SLC25A6/PIK3R1/PLCB4 | 4 |
| hsa00513 | Various types of N-glycan biosynthesis | 1/187 | 42/8577 | 0.604687768988966 | 0.933076864943605 | 0.911184763483293 | FUT8 | 1 |
| hsa04970 | Salivary secretion | 2/187 | 93/8577 | 0.605924372005671 | 0.933076864943605 | 0.911184763483293 | ATP1B3/PLCB4 | 2 |
| hsa05017 | Spinocerebellar ataxia | 3/187 | 143/8577 | 0.607323326440372 | 0.933076864943605 | 0.911184763483293 | SLC25A6/PIK3R1/PLCB4 | 3 |
| hsa04657 | IL-17 signaling pathway | 2/187 | 94/8577 | 0.611773204948822 | 0.933076864943605 | 0.911184763483293 | FOS/JUN | 2 |
| hsa03410 | Base excision repair | 1/187 | 44/8577 | 0.621821407119096 | 0.933076864943605 | 0.911184763483293 | APEX1 | 1 |
| hsa04962 | Vasopressin-regulated water reabsorption | 1/187 | 44/8577 | 0.621821407119096 | 0.933076864943605 | 0.911184763483293 | CREB5 | 1 |

| | | | | | | | | |
|---|---|---|---|---|---|---|---|---|
| hsa04666 | Fc gamma R-mediated phagocytosis | 2/187 | 97/8577 | 0.628923860562592 | 0.933076864943605 | 0.911184763483293 | GSN/PIK3R1 | 2 |
| hsa04713 | Circadian entrainment | 2/187 | 97/8577 | 0.628923860562592 | 0.933076864943605 | 0.911184763483293 | FOS/PLCB4 | 2 |
| hsa04072 | Phospholipase D signaling pathway | 3/187 | 148/8577 | 0.630638868438427 | 0.933076864943605 | 0.911184763483293 | F2R/PIK3R1/PLCB4 | 3 |
| hsa05226 | Gastric cancer | 3/187 | 149/8577 | 0.635186494254207 | 0.933076864943605 | 0.911184763483293 | FGFR2/PIK3R1/FZD3 | 3 |
| hsa04930 | Type II diabetes mellitus | 1/187 | 46/8577 | 0.63821623789289 | 0.933076864943605 | 0.911184763483293 | PIK3R1 | 1 |
| hsa04640 | Hematopoietic cell lineage | 2/187 | 99/8577 | 0.640028933984994 | 0.933076864943605 | 0.911184763483293 | IL1R2/ITGA2 | 2 |
| hsa04061 | Viral protein interaction with cytokine and cytokine receptor | 2/187 | 100/8577 | 0.645483348200085 | 0.933076864943605 | 0.911184763483293 | CXCL14/ACKR3 | 2 |
| hsa04145 | Phagosome | 3/187 | 152/8577 | 0.648596465136223 | 0.933076864943605 | 0.911184763483293 | ITGB1/ITGA2/ITGB2 | 3 |

| | | | | | | | | |
|---|---|---|---|---|---|---|---|---|
| hsa00280 | Valine, leucine and isoleucine degradation | 1/187 | 48/8577 | 0.653903954984198 | 0.933076864943605 | 0.911184763483293 | ALDH3A2 | 1 |
| hsa04914 | Progesterone-mediated oocyte maturation | 2/187 | 102/8577 | 0.656196978820 32 | 0.933076864943605 | 0.911184763483293 | RPS6KA3/PIK3R1 | 2 |
| hsa04972 | Pancreatic secretion | 2/187 | 102/8577 | 0.65619697882032 | 0.933076864943605 | 0.911184763483293 | ATP1B3/PLCB4 | 2 |
| hsa04261 | Adrenergic signaling in cardiomyocytes | 3/187 | 154/8577 | 0.657341652530852 | 0.933076864943605 | 0.911184763483293 | ATP1B3/PLCB4/CREB5 | 3 |
| hsa00520 | Amino sugar and nucleotide sugar metabolism | 1/187 | 49/8577 | 0.661492178740553 | 0.933076864943605 | 0.911184763483293 | UGP2 | 1 |
| hsa04932 | Non-alcoholic fatty liver disease | 3/187 | 155/8577 | 0.661655664070665 | 0.933076864943605 | 0.911184763483293 | FOS/JUN/PIK3R1 | 3 |
| hsa04625 | C-type lectin receptor signaling pathway | 2/187 | 104/8577 | 0.666651992176679 | 0.933076864943605 | 0.911184763483293 | JUN/PIK3R1 | 2 |
| hsa04110 | Cell cycle | 3/187 | 157/8577 | 0.670166404359816 | 0.933076864943605 | 0.911184763483293 | TFDP2/CCND2/ATRX | 3 |

| | | | | | | | | |
|---|---|---|---|---|---|---|---|---|
| hsa 052 06 | MicroRNAs in cancer | 6/187 | 310/ 8577 | 0.67448 343266 3678 | 0.93307 6864943 605 | 0.91118 476348 3293 | SOX4/TP63/TNC/CCND2/PIK3R1/FZD3 | 6 |
| hsa 049 13 | Ovarian steroidogenesis | 1/187 | 51/8 577 | 0.67617 570795 9458 | 0.93307 6864943 605 | 0.91118 476348 3293 | HSD17B2 | 1 |
| hsa 042 17 | Necroptosis | 3/187 | 159/ 8577 | 0.67852 074422 3088 | 0.93307 6864943 605 | 0.91118 476348 3293 | FTH1/SLC25A6/FTL | 3 |
| hsa 002 70 | Cysteine and methionine metabolism | 1/187 | 52/8 577 | 0.68327 811736 7337 | 0.93307 6864943 605 | 0.91118 476348 3293 | LDHB | 1 |
| hsa 005 10 | N-Glycan biosynthesis | 1/187 | 53/8 577 | 0.69022 556511 5409 | 0.93307 6864943 605 | 0.91118 476348 3293 | FUT8 | 1 |
| hsa 006 00 | Sphingolipid metabolism | 1/187 | 53/8 577 | 0.69022 556511 5409 | 0.93307 6864943 605 | 0.91118 476348 3293 | PSAP | 1 |
| hsa 050 12 | Parkinson disease | 5/187 | 266/ 8577 | 0.69407 214796 6408 | 0.93307 6864943 605 | 0.91118 476348 3293 | UBB/SNCA/SLC25A6/RPS27A/SLC39A10 | 5 |
| hsa 034 60 | Fanconi anemia pathway | 1/187 | 55/8 577 | 0.70366 895582 066 | 0.93307 6864943 605 | 0.91118 476348 3293 | REV3L | 1 |
| hsa 052 25 | Hepatocellular carcinoma | 3/187 | 168/ 8577 | 0.71418 698235 9983 | 0.93307 6864943 605 | 0.91118 476348 3293 | PIK3R1/SMARCD3/FZD3 | 3 |

| | | | | | | | | |
|---|---|---|---|---|---|---|---|---|
| hsa01200 | Carbon metabolism | 2/187 | 115/8577 | 0.719645778308289 | 0.933076864943605 | 0.911184763483293 | ESD/CAT | 2 |
| hsa04726 | Serotonergic synapse | 2/187 | 115/8577 | 0.719645778308289 | 0.933076864943605 | 0.911184763483293 | APP/PLCB4 | 2 |
| hsa00240 | Pyrimidine metabolism | 1/187 | 58/8577 | 0.722753582186954 | 0.933076864943605 | 0.911184763483293 | NT5E | 1 |
| hsa04923 | Regulation of lipolysis in adipocytes | 1/187 | 58/8577 | 0.722753582186954 | 0.933076864943605 | 0.911184763483293 | PIK3R1 | 1 |
| hsa05213 | Endometrial cancer | 1/187 | 58/8577 | 0.722753582186954 | 0.933076864943605 | 0.911184763483293 | PIK3R1 | 1 |
| hsa04370 | VEGF signaling pathway | 1/187 | 59/8577 | 0.72883939978656 | 0.933076864943605 | 0.911184763483293 | PIK3R1 | 1 |
| hsa04730 | Long-term depression | 1/187 | 60/8577 | 0.734792326792889 | 0.933076864943605 | 0.911184763483293 | PLCB4 | 1 |
| hsa05010 | Alzheimer disease | 7/187 | 384/8577 | 0.738827049132183 | 0.933076864943605 | 0.911184763483293 | APP/SNCA/SLC25A6/SLC39A10/PIK3R1/PLCB4/FZD3 | 7 |
| hsa04071 | Sphingolipid signaling pathway | 2/187 | 121/8577 | 0.745462606243488 | 0.933076864943605 | 0.911184763483293 | PIK3R1/PLCB4 | 2 |

| | | | | | | | | |
|---|---|---|---|---|---|---|---|---|
| hsa00140 | Steroid hormone biosynthesis | 1/187 | 62/8577 | 0.746310992864062 | 0.933076864943605 | 0.911184763483293 | HSD17B2 | 1 |
| hsa00310 | Lysine degradation | 1/187 | 63/8577 | 0.751882319268574 | 0.933076864943605 | 0.911184763483293 | ALDH3A2 | 1 |
| hsa00561 | Glycerolipid metabolism | 1/187 | 63/8577 | 0.751882319268574 | 0.933076864943605 | 0.911184763483293 | ALDH3A2 | 1 |
| hsa03250 | Viral life cycle - HIV-1 | 1/187 | 63/8577 | 0.751882319268574 | 0.933076864943605 | 0.911184763483293 | MAP1A | 1 |
| hsa03420 | Nucleotide excision repair | 1/187 | 63/8577 | 0.751882319268574 | 0.933076864943605 | 0.911184763483293 | XPA | 1 |
| hsa05321 | Inflammatory bowel disease | 1/187 | 65/8577 | 0.762662477300141 | 0.933076864943605 | 0.911184763483293 | JUN | 1 |
| hsa05221 | Acute myeloid leukemia | 1/187 | 67/8577 | 0.772976657698439 | 0.933076864943605 | 0.911184763483293 | PIK3R1 | 1 |
| hsa04664 | Fc epsilon RI signaling pathway | 1/187 | 68/8577 | 0.7779653022355 | 0.933076864943605 | 0.911184763483293 | PIK3R1 | 1 |
| hsa04060 | Cytokine-cytokine | 5/187 | 297/8577 | 0.781526209544517 | 0.933076864943605 | 0.911184763483293 | CXCL14/IL1R2/BMP5/IL1RAP/ACKR3 | 5 |

| | | | | | | | | |
|---|---|---|---|---|---|---|---|---|
| | receptor interaction | | | | | | | |
| hsa04920 | Adipocytokine signaling pathway | 1/187 | 69/8577 | 0.782844898954499 | 0.933076864943605 | 0.911184763483293 | ACSL4 | 1 |
| hsa04142 | Lysosome | 2/187 | 132/8577 | 0.787538262205215 | 0.933076864943605 | 0.911184763483293 | PSAP/CTSV | 2 |
| hsa04650 | Natural killer cell mediated cytotoxicity | 2/187 | 132/8577 | 0.787538262205215 | 0.933076864943605 | 0.911184763483293 | ITGB2/PIK3R1 | 2 |
| hsa05120 | Epithelial cell signaling in Helicobacter pylori infection | 1/187 | 70/8577 | 0.787617819017441 | 0.933076864943605 | 0.911184763483293 | JUN | 1 |
| hsa04062 | Chemokine signaling pathway | 3/187 | 192/8577 | 0.794384778024741 | 0.933076864943605 | 0.911184763483293 | CXCL14/PIK3R1/PLCB4 | 3 |
| hsa05218 | Melanoma | 1/187 | 72/8577 | 0.796852858494325 | 0.933076864943605 | 0.911184763483293 | PIK3R1 | 1 |
| hsa05223 | Non-small cell lung cancer | 1/187 | 72/8577 | 0.796852858494325 | 0.933076864943605 | 0.911184763483293 | PIK3R1 | 1 |
| hsa05132 | Salmonella infection | 4/187 | 249/8577 | 0.797382522902909 | 0.933076864943605 | 0.911184763483293 | FOS/RPS3/JUN/FLNA | 4 |

| | | | | | | | | |
|---|---|---|---|---|---|---|---|---|
| hsa04144 | Endocytosis | 4/187 | 250/8577 | 0.799931209048561 | 0.933076864943605 | 0.911184763483293 | PSD3/FGFR2/CAV1/HSPA1A | 4 |
| hsa00562 | Inositol phosphate metabolism | 1/187 | 73/8577 | 0.801319468189982 | 0.933076864943605 | 0.911184763483293 | PLCB4 | 1 |
| hsa04910 | Insulin signaling pathway | 2/187 | 137/8577 | 0.8045666302104 11 | 0.933076864943605 | 0.911184763483293 | RPS6/PIK3R1 | 2 |
| hsa04020 | Calcium signaling pathway | 4/187 | 253/8577 | 0.807423380973534 | 0.933076864943605 | 0.911184763483293 | F2R/SLC25A6/FGFR2/PLCB4 | 4 |
| hsa01230 | Biosynthesis of amino acids | 1/187 | 75/8577 | 0.809961731242778 | 0.933076864943605 | 0.911184763483293 | PYCR2 | 1 |
| hsa04623 | Cytosolic DNA-sensing pathway | 1/187 | 75/8577 | 0.809961731242778 | 0.933076864943605 | 0.911184763483293 | POLR1D | 1 |
| hsa05214 | Glioma | 1/187 | 75/8577 | 0.809961731242778 | 0.933076864943605 | 0.911184763483293 | PIK3R1 | 1 |
| hsa05212 | Pancreatic cancer | 1/187 | 76/8577 | 0.814141589659339 | 0.933076864943605 | 0.911184763483293 | PIK3R1 | 1 |
| hsa05220 | Chronic myeloid leukemia | 1/187 | 76/8577 | 0.814141589659339 | 0.933076864943605 | 0.911184763483293 | PIK3R1 | 1 |

| | | | | | | | | |
|---|---|---|---|---|---|---|---|---|
| hsa04120 | Ubiquitin mediated proteolysis | 2/187 | 142/8577 | 0.820377709645816 | 0.933076864943605 | 0.911184763483293 | UBB/RPS27A | 2 |
| hsa04936 | Alcoholic liver disease | 2/187 | 142/8577 | 0.820377709645816 | 0.933076864943605 | 0.911184763483293 | ALDH3A2/SCD5 | 2 |
| hsa05169 | Epstein-Barr virus infection | 3/187 | 202/8577 | 0.821838256410081 | 0.933076864943605 | 0.911184763483293 | CCND2/JUN/PIK3R1 | 3 |
| hsa04612 | Antigen processing and presentation | 1/187 | 78/8577 | 0.822228933836764 | 0.933076864943605 | 0.911184763483293 | HSPA1A | 1 |
| hsa05022 | Pathways of neurodegeneration - multiple diseases | 8/187 | 476/8577 | 0.822538509141549 | 0.933076864943605 | 0.911184763483293 | APP/UBB/SNCA/SLC25A6/RPS27A/PLCB4/CAT/FZD3 | 8 |
| hsa05415 | Diabetic cardiomyopathy | 3/187 | 203/8577 | 0.824405262199169 | 0.933076864943605 | 0.911184763483293 | SLC25A6/PIK3R1/PLCB4 | 3 |
| hsa04723 | Retrograde endocannabinoid signaling | 2/187 | 148/8577 | 0.837838533309848 | 0.934906994174403 | 0.912971953727699 | FAAH/PLCB4 | 2 |
| hsa05170 | Human immunodeficiency virus 1 infection | 3/187 | 212/8577 | 0.846125196138735 | 0.934906994174403 | 0.912971953727699 | FOS/JUN/PIK3R1 | 3 |

| | | | | | | | | |
|---|---|---|---|---|---|---|---|---|
| hsa01232 | Nucleotide metabolism | 1/187 | 85/8577 | 0.847874905911158 | 0.934906994174403 | 0.912971953727699 | NT5E | 1 |
| hsa01240 | Biosynthesis of cofactors | 2/187 | 153/8577 | 0.851206577710833 | 0.934906994174403 | 0.912971953727699 | UGP2/ALDH3A2 | 2 |
| hsa04742 | Taste transduction | 1/187 | 86/8577 | 0.851224810833216 | 0.934906994174403 | 0.912971953727699 | PLCB4 | 1 |
| hsa04260 | Cardiac muscle contraction | 1/187 | 87/8577 | 0.854501334254979 | 0.934906994174403 | 0.912971953727699 | ATP1B3 | 1 |
| hsa03040 | Spliceosome | 3/187 | 216/8577 | 0.855010941452253 | 0.934906994174403 | 0.912971953727699 | HNRNPA1/SF3B1/HSPA1A | 3 |
| hsa04540 | Gap junction | 1/187 | 88/8577 | 0.857706075184816 | 0.934906994174403 | 0.912971953727699 | PLCB4 | 1 |
| hsa04976 | Bile secretion | 1/187 | 89/8577 | 0.860840597972005 | 0.934906994174403 | 0.912971953727699 | ATP1B3 | 1 |
| hsa05160 | Hepatitis C | 2/187 | 158/8577 | 0.863568709478364 | 0.934906994174403 | 0.912971953727699 | EIF3E/PIK3R1 | 2 |
| hsa04520 | Adherens junction | 1/187 | 93/8577 | 0.872706623025539 | 0.937387965720222 | 0.915394716048917 | SNAI2 | 1 |

| | | | | | | | | |
|---|---|---|---|---|---|---|---|---|
| hsa05163 | Human cytomegalovirus infection | 3/187 | 225/8577 | 0.873389590550569 | 0.937387965720222 | 0.915394716048917 | PIK3R1/PLCB4/CREB5 | 3 |
| hsa04630 | JAK-STAT signaling pathway | 2/187 | 166/8577 | 0.881412354183929 | 0.938264853145665 | 0.916251029704708 | CCND2/PIK3R1 | 2 |
| hsa03015 | mRNA surveillance pathway | 1/187 | 97/8577 | 0.883565788957218 | 0.938264853145665 | 0.916251029704708 | PABPC1 | 1 |
| hsa04530 | Tight junction | 2/187 | 170/8577 | 0.889506376037688 | 0.938264853145665 | 0.916251029704708 | ITGB1/JUN | 2 |
| hsa05164 | Influenza A | 2/187 | 171/8577 | 0.891448693597968 | 0.938264853145665 | 0.916251029704708 | SLC25A6/PIK3R1 | 2 |
| hsa04014 | Ras signaling pathway | 3/187 | 236/8577 | 0.893047269861536 | 0.938264853145665 | 0.916251029704708 | NF1/FGFR2/PIK3R1 | 3 |
| hsa03013 | Nucleocytoplasmic transport | 1/187 | 108/8577 | 0.908905746683394 | 0.950913995479685 | 0.928603394444335 | EEF1A1 | 1 |
| hsa04724 | Glutamatergic synapse | 1/187 | 115/8577 | 0.922090878791155 | 0.960672087108777 | 0.938132539091 64 | PLCB4 | 1 |
| hsa00230 | Purine metabolism | 1/187 | 128/8577 | 0.941745093495461 | 0.976915425585942 | 0.953994771973 59 | NT5E | 1 |

| | | | | | | | | |
|---|---|---|---|---|---|---|---|---|
| hsa04114 | Oocyte meiosis | 1/187 | 131/8577 | 0.945528584603262 | 0.976915425585942 | 0.95399477197359 | RPS6KA3 | 1 |
| hsa04140 | Autophagy - animal | 1/187 | 141/8577 | 0.956460832645809 | 0.984127055077713 | 0.961037200266124 | PIK3R1 | 1 |
| hsa04080 | Neuroactive ligand-receptor interaction | 4/187 | 367/8577 | 0.962200801614076 | 0.98560738187667 | 0.962482795237839 | PNOC/EDN1/F2R/LYPD6B | 4 |
| hsa05016 | Huntington disease | 3/187 | 306/8577 | 0.965816068987 58 | 0.98560738187667 | 0.962482795237839 | SLC25A6/PLCB4/CREB5 | 3 |
| hsa04141 | Protein processing in endoplasmic reticulum | 1/187 | 170/8577 | 0.977297041248802 | 0.990454127667572 | 0.967215825369728 | HSPA1A | 1 |
| hsa05152 | Tuberculosis | 1/187 | 180/8577 | 0.981872528256793 | 0.990454127667572 | 0.967215825369728 | ITGB2 | 1 |
| hsa05034 | Alcoholism | 1/187 | 188/8577 | 0.984862426550 56 | 0.990454127667572 | 0.967215825369728 | CREB5 | 1 |
| hsa04814 | Motor proteins | 1/187 | 193/8577 | 0.986476400247 22 | 0.990454127667572 | 0.967215825369728 | KIF21A | 1 |

| | | | | | | | | |
|---|---|---|---|---|---|---|---|---|
| hsa05014 | Amyotrophic lateral sclerosis | 2/187 | 364/8577 | 0.997398006343331 | 0.997398006343331 | 0.973996784888224 | HNRNPA1/CAT | 2 |

2
