# Supplementary material for: Multiomics Reveals IL-17 Drives Epithelial Keratinization and Proliferation via EHF in Odontogenic Keratocysts
Source: Int J Mol Sci. 2026 May 4;27(9):4115. doi: 10.3390/ijms27094115 (PMC13163638; doi:10.3390/ijms27094115)
Supplement: Supplementary file 1 [file ijms-27-04115-s001.zip › ijms-4235677-supplementary/Supplementary Table S9.pdf]

Supplementary Table S9. EpC2 KEGG enrichment.

ID	Description	GeneRatio	BgRatio	pvalue	p.adjust	qvalue	geneID	Count
hsa04657	IL-17 signaling pathway	19/243	94/8577	1.05514058667268e-11	2.81722536641606e-09	2.3102025476623e-09	S100A7/S100A8/S100A9/LCN2/CXCL1/CXCL8/IL1B/CCL20/CXCL3/CXCL6/CSF3/NFKBIA/TNFAIP3/CXCL2/S100A7A/FOSL1/FOSB/CEBPB/PTGS2	19
hsa04064	NF-kappa B signaling pathway	15/243	104/8577	1.99202824951901e-07	2.65935771310788e-05	2.18074671526292e-05	CXCL1/CXCL8/IL1B/CD14/CXCL3/NFKBIA/TNFAIP3/CXCL2/LYN/BCL2A1/PLAU/GADD45A/LY96/PTGS2/CYLD	15
hsa04668	TNF signaling pathway	14/243	114/8577	3.70632812542175e-06	0.000329863203162536	0.000270496929855342	CXCL1/IL1B/CCL20/CXCL3/CXCL6/NFKBIA/TNFAIP3/CXCL2/MAP3K8/BCL3/CEBPB/PTGS2/CYLD/IRF1	14
hsa05167	Kaposi sarcoma-associated herpesvirus infection	18/243	194/8577	9.05360395549124e-06	0.00060432806402904	0.00049556569019531	CXCL1/CXCL8/CLEC2B/CXCL3/NFKBIA/CXCL2/CDKN1A/CALML3/RAC1/VEGFA/ZFP36/HIF1A/LYN/HLA-E/HLA-C/HLA-B/UBC/PTGS2	18
hsa05146	Amoebiasis	12/243	102/8577	2.87548251954627e-05	0.00153550766543771	0.00125915866119079	SERPINB4/CXCL1/CXCL8/SERPINB3/IL1B/CD14/CXCL3/CXCL2/HSPB1/LAMC2/SERPINB13/GNA15	12
hsa05323	Rheumatoid arthritis	11/243	93/8577	5.90339113501786e-05	0.00262700905508295	0.00215421992295389	CXCL1/CXCL8/IL1B/IL1A/CCL20/CXCL3/CXCL6/CXCL2/CTSL/VEGFA/ATP6V1D	11
hsa05417	Lipid and atherosclerosis	17/243	215/8577	0.000123229768369852	0.00432833015328874	0.00354935017498151	CXCL1/CXCL8/IL1B/TNFSF10/SOD2/CD14/CXCL3/NFKBIA/CXCL2/ERO1A/CALML3/RAC1/HSPA5/HSPA6/LYN/XBP1/LY96	17
hsa05120	Epithelial cell signaling in Helicobacter pylori infection	9/243	70/8577	0.000146023784343208	0.00432833015328874	0.00354935017498151	CXCL1/CXCL8/CXCL3/HBEGF/NFKBIA/CXCL2/RAC1/LYN/ATP6V1D	9
hsa04610	Complement and coagulation cascades	10/243	86/8577	0.000148000212235962	0.00432833015328874	0.00354935017498151	PLAT/PLAUR/SERPINB2/CFH/CD55/CD59/CD46/F3/THBD/PLAU	10
hsa05134	Legionellosis	8/243	56/8577	0.000162109743568867	0.00432833015328874	0.00354935017498151	CXCL1/CXCL8/IL1B/CD14/CXCL3/NFKBIA/CXCL2/HSPA6	8

hsa04612	Antigen processing and presentation	9/243	78/8577	0.000336367735919445	0.00816456231731744	0.00669516641830092	B2M/CTSL/CTSB/HSPA5/HSPA6/HLA-E/HLA-C/HLA-B/LGMN	9
hsa04640	Hematopoietic cell lineage	10/243	99/8577	0.000472099545614972	0.0105042148899331	0.00861374609543107	IL1B/CD24/IL1A/CD14/CSF3/TFRC/MME/CD55/CD59/IL4R	10
hsa05418	Fluid shear stress and atherosclerosis	12/243	139/8577	0.000562500657979029	0.0115528981292616	0.00947369529227838	PLAT/IL1B/IL1A/ASS1/CTSL/DUSP1/CALML3/RAC1/SDC4/VEGFA/THBD/HMOX1	12
hsa04060	Cytokine-cytokine receptor interaction	19/243	297/8577	0.000755101435224201	0.0144008630860616	0.0118091051523785	CXCL1/IL1RN/CXCL8/IL36G/IL1B/TNFSF10/IL1A/CCL20/CXCL3/IL19/CXCL6/CXCL17/CSF3/CXCL2/IL36A/IL13RA1/TNFRSF21/IL4R/BMP2	19
hsa05219	Bladder cancer	6/243	41/8577	0.000951365973091946	0.0169343143210366	0.013886605080921	CXCL8/HBEGF/TYMP/CDKN1A/VEGFA/MDM2	6
hsa05202	Transcriptional misregulation in cancer	14/243	193/8577	0.00114527527750297	0.0191117811933309	0.0156721880079354	CXCL8/PLAT/CD14/NFKBIZ/CDKN1A/SPINT1/ID2/TMPRSS2/MDM2/BCL2A1/PLAU/GADD45A/RUNX1/CEBPB	14
hsa04142	Lysosome	11/243	132/8577	0.00127127748196974	0.0196259126254856	0.0160937899708299	NCOA7/CTSC/CTSD/CTSL/CTSB/GM2A/FUCA1/SORT1/CLTB/NPC2/LGMN	11
hsa05133	Pertussis	8/243	76/8577	0.00132309523317881	0.0196259126254856	0.0160937899708299	CXCL8/IL1B/IL1A/CD14/CXCL6/CALML3/LY96/IRF1	8
hsa04210	Apoptosis	11/243	136/8577	0.00161975787575741	0.0227618606751173	0.0186653539145453	TNFSF10/CTSC/NFKBIA/CTSD/CTSL/CAPN2/CTSB/PTPN13/BCL2A1/PMAIP1/GADD45A	11
hsa05205	Proteoglycans in cancer	14/243	205/8577	0.00203097937249886	0.0271135746228598	0.0222338794463033	PLAUR/DCN/HBEGF/CTSL/CDKN1A/RAC1/SDC4/VEGFA/EZR/MDM2/WNT5A/HIF1A/LUM/PLAU	14
hsa04625	C-type lectin receptor signaling pathway	9/243	104/8577	0.00268161394792518	0.0340948059093345	0.0279586817628 29	IL1B/NFKBIA/CALML3/MDM2/BCL3/CLEC7A/PTGS2/CYLD/IRF1	9
hsa04350	TGF-beta signaling pathway	9/243	108/8577	0.0034629254273757	0.0420273222322415	0.0344635640619209	DCN/TFRC/PITX2/ID2/ID1/SKIL/CDKN2B/BMP2/BAMBI	9

hsa04218	Cellular senescence	11/243	156/8577	0.00472002171058104	0.0515791506912531	0.0422963270009093	CXCL8/IL1A/CDKN1A/CALML3/CAPN2/MDM2/HLA-E/HLA-C/GADD45A/CDKN2B/HLA-B	11
hsa04115	p53 signaling pathway	7/243	74/8577	0.00481298031305512	0.0515791506912531	0.0422963270009093	CDKN1A/MDM2/CD82/SFN/PMAIP1/GADD45A/SHISA5	7
hsa05169	Epstein-Barr virus infection	13/243	202/8577	0.00482950849169037	0.0515791506912531	0.0422963270009093	NFKBIA/TNFAIP3/B2M/CDKN1A/RAC1/ISG15/MDM2/LYN/HLA-E/HLA-C/GADD45A/HLA-B/OAS1	13
hsa04216	Ferroptosis	5/243	41/8577	0.00570957673562883	0.0586329610928037	0.0480806461947691	SAT1/TFRC/GCLM/HMOX1/PRNP	5
hsa05215	Prostate cancer	8/243	97/8577	0.00610623693936038	0.0602347715108451	0.0493941749428574	PLAT/NFKBIA/CDKN1A/SPINT1/TMPRSS2/MDM2/TGFA/PLAU	8
hsa05332	Graft-versus-host disease	5/243	42/8577	0.00633465008012102	0.0602347715108451	0.0493941749428574	IL1B/IL1A/HLA-E/HLA-C/HLA-B	5
hsa04621	NOD-like receptor signaling pathway	12/243	186/8577	0.00654235345997943	0.0602347715108451	0.0493941749428574	CXCL1/CXCL8/IL1B/CXCL3/NFKBIA/TNFAIP3/CXCL2/CTSB/NAMPT/IFI16/PKN2/OAS1	12
hsa04940	Type I diabetes mellitus	5/243	43/8577	0.00700625557035802	0.0610363843755768	0.0500515196141138	IL1B/IL1A/HLA-E/HLA-C/HLA-B	5
hsa04061	Viral protein interaction with cytokine and cytokine receptor	8/243	100/8577	0.0073152220974474	0.0610363843755768	0.0500515196141138	CXCL1/CXCL8/TNFSF10/CCL20/CXCL3/IL19/CXCL6/CXCL2	8
hsa04933	AGE-RAGE signaling pathway in diabetic complications	8/243	100/8577	0.0073152220974474	0.0610363843755768	0.0500515196141138	CXCL8/IL1B/IL1A/RAC1/VEGFA/F3/THBD/PIM1	8
hsa05164	Influenza A	11/243	171/8577	0.00925129042736902	0.0748513498214402	0.0613801725324643	CXCL8/IL1B/TNFSF10/IL1A/PRSS3/NFKBIA/TMPRSS11D/RSAD2/TMPRSS2/MX1/OAS1	11
hsa05130	Pathogenic Escherichia coli infection	12/243	198/8577	0.0105314187633534	0.0827026120533932	0.0678184242345981	CXCL8/IL1B/TNFSF10/CLDN1/CLDN4/NFKBIA/CLDN7/RAC1/EZR/ARPC3/SLC9A3R1/TUBB2A	12
hsa04145	Phagosome	10/243	152/8577	0.0110546711363564	0.0833836406913607	0.0683768865121349	CD14/TFRC/CTSL/RAC1/CLEC7A/HLA-E/HLA-C/HLA-B/TUBB2A/ATP6V1D	10

hsa05163	Human cytomegalovirus infection	13/243	225/8577	0.0115483047038741	0.0833836406913607	0.0683768865121349	CXCL8/IL1B/NFKBIA/B2M/CDKN1A/CALML3/RAC1/VEGFA/MDM2/HLA-E/HLA-C/HLA-B/PTGS2	13
hsa04144	Endocytosis	14/243	250/8577	0.0115550363504882	0.0833836406913607	0.0683768865121349	TFRC/RAB31/RAB10/MDM2/HSPA6/ARPC3/ARF4/HLA-E/HLA-C/RAB11FIP1/SH3GLB1/CLTB/VPS4B/HLA-B	14
hsa05211	Renal cell carcinoma	6/243	69/8577	0.0131605499675224	0.0924701800349602	0.0758280995358633	CDKN1A/RAC1/VEGFA/TGFA/HIF1A/ELOC	6
hsa00512	Mucin type O-glycan biosynthesis	4/243	36/8577	0.0181868144150938	0.124509729457181	0.102101414260176	GALNT1/ST3GAL1/GALNT5/GALNT3	4
hsa04936	Alcoholic liver disease	9/243	142/8577	0.0193957310622578	0.129466504840571	0.106166106867096	CXCL1/CXCL8/IL1B/CD14/CXCL3/NFKBIA/CXCL2/ADIPOR1/LY96	9
hsa04530	Tight junction	10/243	170/8577	0.0225726665281125	0.146997608853806	0.120542095965273	CLDN1/CLDN4/DLG1/CLDN7/RAC1/EZR/ARPC3/AFDN/SLC9A3R1/RUNX1	10
hsa04010	MAPK signaling pathway	15/243	301/8577	0.0239641539140653	0.152343549882272	0.124925915140992	IL1B/IL1A/CD14/HSPB1/DUSP1/RAC1/VEGFA/EPHA2/MAP3K8/HSPA6/TGFA/AREG/GADD45A/DUSP10/DUSP5	15
hsa04972	Pancreatic secretion	7/243	102/8577	0.0254718348023201	0.156617356017129	0.128430554116155	CLCA4/PRSS3/CLCA2/CA2/RAC1/ATP1B1/RAB27B	7
hsa05165	Human papillomavirus infection	16/243	331/8577	0.0258096017406504	0.156617356017129	0.128430554116155	DLG1/CDKN1A/ISG15/LAMC2/VEGFA/MDM2/WNT5A/MX1/SLC9A3R1/HLA-E/HLA-C/HLA-B/ATP6V1D/PTGS2/OASL/IRF1	16
hsa04978	Mineral absorption	5/243	60/8577	0.0270303461893981	0.156893531142811	0.128657025340842	HEPHL1/SLC5A1/HMOX1/ATP1B1/MT1X	5
hsa05416	Viral myocarditis	5/243	60/8577	0.0270303461893981	0.156893531142811	0.128657025340842	CD55/RAC1/HLA-E/HLA-C/HLA-B	5
hsa04620	Toll-like receptor signaling pathway	7/243	104/8577	0.0279577045176349	0.158823555451245	0.130239698536799	CXCL8/IL1B/CD14/NFKBIA/RAC1/MAP3K8/LY96	7
hsa05203	Viral carcinogenesis	11/243	204/8577	0.0303331236820921	0.168714495798021	0.138350542582252	DLG1/NFKBIA/CDKN1A/RAC1/MDM2/LYN/HLA-E/PMAIP1/HLA-C/CDKN2B/HLA-B	11
hsa00920	Sulfur metabolism	2/243	10/8577	0.0309625853711723	0.168714495798021	0.138350542582252	ETHE1/SQOR	2

hsa04068	FoxO signaling pathway	8/243	131/8577	0.0324276174073817	0.173163476955418	0.141998829910219	TNFSF10/SOD2/CDKN1A/MDM2/PLK2/GADD45A/CDKN2B/FBXO32	8
hsa05210	Colorectal cancer	6/243	86/8577	0.034840155381453	0.179096214068291	0.146863838069011	CDKN1A/RAC1/TGFA/AREG/PMAIP1/GADD45A	6
hsa04066	HIF-1 signaling pathway	7/243	109/8577	0.0348801615413901	0.179096214068291	0.146863838069011	TFRC/CDKN1A/PFKFB3/VEGFA/HIF1A/HMOX1/ELOC	7
hsa04217	Necroptosis	9/243	159/8577	0.0366917386505388	0.184843287164035	0.151576596609972	GLUL/IL1B/TNFSF10/IL1A/TNFAIP3/CAPN2/VPS4B/PYGL/CYLD	9
hsa04915	Estrogen signaling pathway	8/243	137/8577	0.0406434175317989	0.200959120018339	0.164792024300471	KRT13/KRT16/HBEGF/CTSD/CALML3/HSPA6/TGFA/KRT19	8
hsa05162	Measles	8/243	138/8577	0.042135914006151	0.204550709811679	0.16773722704841	IL1B/IL1A/NFKBIA/TNFAIP3/CD46/HSPA6/MX1/OAS1	8
hsa00830	Retinol metabolism	5/243	68/8577	0.0431073725081374	0.205529793922727	0.168540103039334	SDR16C5/RDH10/DHRS9/DHRS3/ALDH1A3	5
hsa04670	Leukocyte transendothelial migration	7/243	115/8577	0.0445823405848057	0.208372320584843	0.170871053347713	CLDN1/CLDN4/CLDN7/RAC1/EZR/AFDN/CTNND1	7
hsa04924	Renin secretion	5/243	69/8577	0.045446968019123	0.208372320584843	0.170871053347713	CLCA4/CLCA2/CALML3/CTSB/ADRB2	5
hsa05222	Small cell lung cancer	6/243	92/8577	0.0460448199045158	0.208372320584843	0.170871053347713	NFKBIA/CDKN1A/LAMC2/GADD45A/CDKN2B/PTGS2	6
hsa05166	Human T-cell leukemia virus 1 infection	11/243	222/8577	0.0507841281875963	0.225989370434803	0.18531752040386	DLG1/NFKBIA/B2M/CDKN1A/ETS2/ZFP36/FOSL1/HLA-E/HLA-C/CDKN2B/HLA-B	11
hsa05214	Glioma	5/243	75/8577	0.0610530240545681	0.267232088894585	0.219137687719589	CDKN1A/CALML3/MDM2/TGFA/GADD45A	5
hsa05212	Pancreatic cancer	5/243	76/8577	0.0639156423614606	0.270880579531905	0.222129550729888	CDKN1A/RAC1/VEGFA/TGFA/GADD45A	5
hsa05220	Chronic myeloid leukemia	5/243	76/8577	0.0639156423614606	0.270880579531905	0.222129550729888	NFKBIA/CDKN1A/MDM2/GADD45A/RUNX1	5

hsa00051	Fructose and mannose metabolism	3/243	34/8577	0.0706356361366432	0.292126007459221	0.239551387942117	PFKFB3/AKR1B1/AKR1B10	3
hsa04380	Osteoclast differentiation	7/243	128/8577	0.0711168182953159	0.292126007459221	0.239551387942117	IL1B/IL1A/NFKBIA/RAC1/FOSL1/FOSB/CYLD	7
hsa04514	Cell adhesion molecules	8/243	158/8577	0.0798031284818302	0.318021422457443	0.260786342878565	CLDN1/CLDN4/CLDN7/SDC4/HLA-E/HLA-C/CDH3/HLA-B	8
hsa05160	Hepatitis C	8/243	158/8577	0.0798031284818302	0.318021422457443	0.260786342878565	CLDN1/CLDN4/NFKBIA/CLDN7/CDKN1A/RSAD2/MX1/OAS1	8
hsa00240	Pyrimidine metabolism	4/243	58/8577	0.0814119792789765	0.318291613510818	0.261007906998818	TYMP/CMPK1/CDA/UPP1	4
hsa00910	Nitrogen metabolism	2/243	17/8577	0.0822551360758294	0.318291613510818	0.261007906998818	GLUL/CA2	2
hsa04370	VEGF signaling pathway	4/243	59/8577	0.0854897430510487	0.326082305637571	0.26739648954313	HSPB1/RAC1/VEGFA/PTGS2	4
hsa05330	Allograft rejection	3/243	38/8577	0.0918480372507478	0.34249288129196	0.280853614463741	HLA-E/HLA-C/HLA-B	3
hsa01232	Nucleotide metabolism	5/243	85/8577	0.0929876992453153	0.34249288129196	0.280853614463741	TYMP/CMPK1/CDA/AK2/UPP1	5
hsa00590	Arachidonic acid metabolism	4/243	61/8577	0.0939375147887576	0.34249288129196	0.280853614463741	LTA4H/PTGR1/ALOX15B/PTGS2	4
hsa04062	Chemokine signaling pathway	9/243	192/8577	0.0951522299967187	0.34249288129196	0.280853614463741	CXCL1/CXCL8/CCL20/CXCL3/CXCL6/NFKBIA/CXCL2/RAC1/LYN	9
hsa05132	Salmonella infection	11/243	249/8577	0.0962058655314496	0.34249288129196	0.280853614463741	CXCL8/IL1B/TNFSF10/CD14/NFKBIA/ANXA2/RAC1/S100A10/ARPC3/LY96/TUBB2A	11
hsa03250	Viral life cycle - HIV-1	4/243	63/8577	0.102764707110923	0.356339958423592	0.292208599850609	APOBEC3A/MX1/VPS4B/ELL2	4
hsa05217	Basal cell carcinoma	4/243	63/8577	0.102764707110923	0.356339958423592	0.292208599850609	CDKN1A/WNT5A/GADD45A/BMP2	4

hsa04141	Protein processing in endoplasmic reticulum	8/243	170/8577	0.109736190489343	0.375635421290445	0.308031411899911	PPP1R15A/CRYAB/ERO1A/CAPN2/HSPH1/HSPA5/HSPA6/XBP1	8
hsa05221	Acute myeloid leukemia	4/243	67/8577	0.121503112416485	0.407440198393113	0.33411220684316	CD14/BCL2A1/RUNX1/PIM1	4
hsa05171	Coronavirus disease - COVID-19	10/243	232/8577	0.122904560022354	0.407440198393113	0.33411220684316	CXCL8/IL1B/CSF3/HBEGF/NFKBIA/ISG15/TMPRSS2/MX1/RPS4Y1/OAS1	10
hsa04520	Adherens junction	5/243	93/8577	0.123605453445102	0.407440198393113	0.33411220684316	RAC1/LMO7/AFDN/NECTIN4/CTNND1	5
hsa00220	Arginine biosynthesis	2/243	22/8577	0.127596515407988	0.415466702608935	0.340694161808234	GLUL/ASS1	2
hsa04964	Proximal tubule bicarbonate reclamation	2/243	23/8577	0.137255306948813	0.441532131991964	0.362068533232125	CA2/ATP1B1	2
hsa04622	RIG-I-like receptor signaling pathway	4/243	71/8577	0.141586904190813	0.450044088320798	0.36904857232693	CXCL8/NFKBIA/ISG15/CYLD	4
hsa01240	Biosynthesis of cofactors	7/243	153/8577	0.143617607412799	0.451128249167261	0.369937614140707	SDR16C5/KYNU/CMPK1/DHRS3/GCLM/AK2/PDXK	7
hsa00514	Other types of O-glycan biosynthesis	3/243	47/8577	0.147485110566429	0.453589400194961	0.371955825904009	GALNT1/GALNT5/GALNT3	3
hsa05170	Human immunodeficiency virus 1 infection	9/243	212/8577	0.147798793321953	0.453589400194961	0.371955825904009	APOBEC3A/NFKBIA/B2M/CALML3/RAC1/HLA-E/HLA-C/ELOC/HLA-B	9
hsa04918	Thyroid hormone synthesis	4/243	75/8577	0.162885579892228	0.49420965717301	0.405265557626596	GPX3/HSPA5/ATP1B1/DUOX2	4
hsa05144	Malaria	3/243	50/8577	0.168007036675589	0.49953893227331	0.409635710281287	CXCL8/IL1B/CSF3	3
hsa04971	Gastric acid secretion	4/243	76/8577	0.168383909755048	0.49953893227331	0.409635710281287	CALML3/CA2/EZR/ATP1B1	4
hsa05140	Leishmaniasis	4/243	77/8577	0.173947263785635	0.508952963833288	0.417355475960276	IL1B/IL1A/NFKBIA/PTGS2	4

hsa00790	Folate biosynthesis	2/243	27/8577	0.17727575144755	0.508952963833288	0.417355475960276	AKR1B1/AKR1B10	2
hsa04966	Collecting duct acid secretion	2/243	27/8577	0.17727575144755	0.508952963833288	0.417355475960276	CA2/ATP6V1D	2
hsa04928	Parathyroid hormone synthesis, secretion and action	5/243	106/8577	0.18174504641982	0.516233270149914	0.423325528055123	HBEGF/CDKN1A/CYP24A1/SLC9A3R1/MAFB	5
hsa00601	Glycosphingolipid biosynthesis - lacto and neolacto series	2/243	28/8577	0.187550987914882	0.519837771651807	0.426281318760402	B3GNT5/FUT3	2
hsa05320	Autoimmune thyroid disease	3/243	53/8577	0.189299616247058	0.519837771651807	0.426281318760402	HLA-E/HLA-C/HLA-B	3
hsa00983	Drug metabolism - other enzymes	4/243	80/8577	0.191004875519082	0.519837771651807	0.426281318760402	TYMP/CMPK1/CDA/UPP1	4
hsa04659	Th17 cell differentiation	5/243	108/8577	0.191479554603664	0.519837771651807	0.426281318760402	IL1B/NFKBIA/HIF1A/RUNX1/IL4R	5
hsa05135	Yersinia infection	6/243	137/8577	0.192748836679884	0.519837771651807	0.426281318760402	CXCL8/IL1B/NFKBIA/RAC1/ARPC3/PKN2	6
hsa04140	Autophagy - animal	6/243	141/8577	0.210413250430872	0.561803378650427	0.460694274627593	CTSD/VAMP8/CTSL/CTSB/HIF1A/SH3GLB1	6
hsa04012	ErbB signaling pathway	4/243	85/8577	0.220522422756839	0.582965216594813	0.478047565746978	HBEGF/CDKN1A/TGFA/AREG	4
hsa04923	Regulation of lipolysis in adipocytes	3/243	58/8577	0.226155602505156	0.591995547734085	0.485452686491976	FABP4/ADRB2/PTGS2	3
hsa00052	Galactose metabolism	2/243	32/8577	0.229329675947158	0.594475956096031	0.487486689800806	AKR1B1/AKR1B10	2
hsa04130	SNARE interactions in vesicular transport	2/243	33/8577	0.239884941203539	0.615858454820624	0.505020928849556	VAMP5/VAMP8	2

hsa04213	Longevity regulating pathway - multiple species	3/243	61/8577	0.248892309541936	0.627416357034012	0.514498727628916	SOD2/CRYAB/HSPA6	3
hsa05152	Tuberculosis	7/243	180/8577	0.249086643616499	0.627416357034012	0.514498727628916	IL1B/IL1A/CD14/CTSD/CALML3/CLEC7A/CEBPB	7
hsa00561	Glycerolipid metabolism	3/243	63/8577	0.264238547889156	0.655286658386831	0.537353143877236	MBOAT2/AKR1B1/AKR1B10	3
hsa05131	Shigellosis	9/243	247/8577	0.266730463925224	0.655286658386831	0.537353143877236	CXCL8/IL1B/CD14/NFKBIA/RAC1/CAPN2/MDM2/ARPC3/UBC	9
hsa04970	Salivary secretion	4/243	93/8577	0.269946565091996	0.655286658386831	0.537353143877236	CALML3/ATP1B1/ADRB2/CST3	4
hsa00040	Pentose and glucuronate interconversions	2/243	36/8577	0.271649278073833	0.655286658386831	0.537353143877236	AKR1B1/AKR1B10	2
hsa04932	Non-alcoholic fatty liver disease	6/243	155/8577	0.276141114228508	0.655286658386831	0.537353143877236	CXCL8/IL1B/IL1A/RAC1/ADIPOR1/XBP1	6
hsa05321	Inflammatory bowel disease	3/243	65/8577	0.279697866160915	0.655286658386831	0.537353143877236	IL1B/IL1A/IL4R	3
hsa00250	Alanine, aspartate and glutamate metabolism	2/243	37/8577	0.282239571964366	0.655286658386831	0.537353143877236	GLUL/ASS1	2
hsa04960	Aldosterone-regulated sodium reabsorption	2/243	37/8577	0.282239571964366	0.655286658386831	0.537353143877236	SFN/ATP1B1	2
hsa05216	Thyroid cancer	2/243	37/8577	0.282239571964366	0.655286658386831	0.537353143877236	CDKN1A/GADD45A	2
hsa04390	Hippo signaling pathway	6/243	157/8577	0.285923976542477	0.658118118421047	0.539675019245329	DLG1/ID2/ID1/WNT5A/AREG/BMP2	6
hsa05150	Staphylococcus aureus infection	4/243	96/8577	0.288980181160287	0.659467592904246	0.540781625563111	KRT13/KRT16/CFH/KRT19	4
hsa05031	Amphetamine addiction	3/243	69/8577	0.310834112276267	0.703328033709858	0.576748397443921	CALML3/FOSB/MAOA	3

hsa04650	Natural killer cell mediated cytotoxicity	5/243	132/8577	0.31928214226723	0.709559017741582	0.581857976306915	TNFSF10/RAC1/HLA-E/HLA-C/HLA-B	5
hsa05142	Chagas disease	4/243	102/8577	0.327536933828404	0.709559017741582	0.581857976306915	CXCL8/IL1B/NFKBIA/GNA15	4
hsa04630	JAK-STAT signaling pathway	6/243	166/8577	0.330762171815802	0.709559017741582	0.581857976306915	IL19/CSF3/CDKN1A/IL13RA1/IL4R/PIM1	6
hsa00533	Glycosaminoglycan biosynthesis - keratan sulfate	1/243	14/8577	0.331473372824578	0.709559017741582	0.581857976306915	ST3GAL1	1
hsa04137	Mitophagy - animal	3/243	72/8577	0.334264679856212	0.709559017741582	0.581857976306915	HIF1A/CITED2/UBC	3
hsa05218	Melanoma	3/243	72/8577	0.334264679856212	0.709559017741582	0.581857976306915	CDKN1A/MDM2/GADD45A	3
hsa05223	Non-small cell lung cancer	3/243	72/8577	0.334264679856212	0.709559017741582	0.581857976306915	CDKN1A/TGFA/GADD45A	3
hsa00380	Tryptophan metabolism	2/243	42/8577	0.334848075788162	0.709559017741582	0.581857976306915	KYNU/MAOA	2
hsa05225	Hepatocellular carcinoma	6/243	168/8577	0.340861612099453	0.713540898285628	0.585123228241319	CDKN1A/TGFA/WNT5A/TXNRD1/HMOX1/GADD45A	6
hsa01524	Platinum drug resistance	3/243	73/8577	0.342072041125694	0.713540898285628	0.585123228241319	CDKN1A/MDM2/PMAIP1	3
hsa00860	Porphyrin metabolism	2/243	43/8577	0.345253025939798	0.714265269918986	0.585717232971216	HEPHL1/HMOX1	2
hsa00604	Glycosphingolipid biosynthesis - ganglio series	1/243	15/8577	0.350444757900326	0.714265269918986	0.585717232971216	ST3GAL1	1
hsa00730	Thiamine metabolism	1/243	15/8577	0.350444757900326	0.714265269918986	0.585717232971216	AK2	1

hsa03320	PPAR signaling pathway	3/243	75/8577	0.357663915282641	0.723456555912614	0.59325434113867	FABP5/FABP4/UBC	3
hsa00360	Phenylalanine metabolism	1/243	16/8577	0.368879927700633	0.729562523674585	0.598261403210383	MAOA	1
hsa00430	Taurine and hypotaurine metabolism	1/243	16/8577	0.368879927700633	0.729562523674585	0.598261403210383	FMO2	1
hsa00603	Glycosphingolipid biosynthesis - globo and isoglobo series	1/243	16/8577	0.368879927700633	0.729562523674585	0.598261403210383	ST3GAL1	1
hsa05100	Bacterial invasion of epithelial cells	3/243	77/8577	0.373205867884403	0.732690931802467	0.600826784210184	RAC1/ARPC3/CLTB	3
hsa04015	Rap1 signaling pathway	7/243	210/8577	0.385311611396935	0.737671370759342	0.604910881600406	CALML3/RAC1/ID1/VEGFA/EPHA2/AFDN/CTNND1	7
hsa05145	Toxoplasmosis	4/243	111/8577	0.385796665892695	0.737671370759342	0.604910881600406	NFKBIA/LAMC2/HSPA6/LY96	4
hsa04973	Carbohydrate digestion and absorption	2/243	47/8577	0.386290233437988	0.737671370759342	0.604910881600406	SLC5A1/ATP1B1	2
hsa00450	Selenocompound metabolism	1/243	17/8577	0.386793977177183	0.737671370759342	0.604910881600406	TXNRD1	1
hsa00511	Other glycan degradation	1/243	18/8577	0.404201578058718	0.758789184971134	0.62222807204414	FUCA1	1
hsa00520	Amino sugar and nucleotide sugar metabolism	2/243	49/8577	0.406392709553829	0.758789184971134	0.62222807204414	UAP1/CYB5R1	2
hsa05030	Cocaine addiction	2/243	49/8577	0.406392709553829	0.758789184971134	0.62222807204414	FOSB/MAOA	2
hsa04726	Serotonergic synapse	4/243	115/8577	0.411557127420362	0.761362288093443	0.624338087614572	DUSP1/ALOX15B/PTGS2/MAOA	4

hsa00330	Arginine and proline metabolism	2/243	50/8577	0.416325445923755	0.761362288093443	0.624338087614572	SAT1/MAOA	2
hsa05110	Vibrio cholerae infection	2/243	50/8577	0.416325445923755	0.761362288093443	0.624338087614572	ERO1A/ATP6V1D	2
hsa04979	Cholesterol metabolism	2/243	51/8577	0.426174431446478	0.77015719474898	0.631550153785878	SORT1/NPC2	2
hsa04662	B cell receptor signaling pathway	3/243	84/8577	0.426903613568723	0.77015719474898	0.631550153785878	NFKBIA/RAC1/LYN	3
hsa04722	Neurotrophin signaling pathway	4/243	119/8577	0.437090726601047	0.776227712552073	0.636528145912995	NFKBIA/CALML3/RAC1/SORT1	4
hsa00100	Steroid biosynthesis	1/243	20/8577	0.437554075416342	0.776227712552073	0.636528145912995	CYP24A1	1
hsa04151	PI3K-Akt signaling pathway	11/243	359/8577	0.439022339722049	0.776227712552073	0.636528145912995	CSF3/CDKN1A/RAC1/LAMC2/VEGFA/EPHA2/MDM2/TGFA/AREG/PKN2/IL4R	11
hsa00600	Sphingolipid metabolism	2/243	53/8577	0.445608464628705	0.776227712552073	0.636528145912995	UGCG/CERS3	2
hsa04961	Endocrine and other factor-regulated calcium reabsorption	2/243	53/8577	0.445608464628705	0.776227712552073	0.636528145912995	ATP1B1/CLTB	2
hsa04071	Sphingolipid signaling pathway	4/243	121/8577	0.449743850549323	0.776227712552073	0.636528145912995	CTSD/RAC1/CERS3/SPTSSA	4
hsa00532	Glycosaminoglycan biosynthesis - chondroitin sulfate / dermatan sulfate	1/243	21/8577	0.453526303963009	0.776227712552073	0.636528145912995	DSE	1
hsa00770	Pantothenate and CoA biosynthesis	1/243	21/8577	0.453526303963009	0.776227712552073	0.636528145912995	VNN1	1
hsa04110	Cell cycle	5/243	157/8577	0.45998499699099	0.779625397843964	0.639314341618547	CDKN1A/MDM2/SFN/GADD45A/CDKN2B	5

hsa04512	ECM-receptor interaction	3/243	89/8577	0.464271304334046	0.779625397843964	0.639314341618547	CD47/LAMC2/SDC4	3
hsa04976	Bile secretion	3/243	89/8577	0.464271304334046	0.779625397843964	0.639314341618547	CA2/SLC5A1/ATP1B1	3
hsa00340	Histidine metabolism	1/243	22/8577	0.469046770084677	0.782721797578804	0.641853474852716	MAOA	1
hsa00480	Glutathione metabolism	2/243	57/8577	0.483345284152647	0.793019812642127	0.650298131399812	GPX3/GCLM	2
hsa03060	Protein export	1/243	23/8577	0.484128200227216	0.793019812642127	0.650298131399812	HSPA5	1
hsa04614	Renin-angiotensin system	1/243	23/8577	0.484128200227216	0.793019812642127	0.650298131399812	MME	1
hsa05213	Endometrial cancer	2/243	58/8577	0.492530629925771	0.801863891403542	0.657550520054945	CDKN1A/GADD45A	2
hsa00534	Glycosaminoglycan biosynthesis - heparan sulfate / heparin	1/243	24/8577	0.498782963769978	0.802259345340868	0.657874803196927	HS3ST1	1
hsa04977	Vitamin digestion and absorption	1/243	24/8577	0.498782963769978	0.802259345340868	0.657874803196927	SLC52A3	1
hsa04014	Ras signaling pathway	7/243	236/8577	0.505546259988787	0.808268571359319	0.662802534369164	CALML3/RAC1/VEGFA/ETS2/EPHA2/TGFA/AFDN	7
hsa04510	Focal adhesion	6/243	203/8577	0.51686255734606	0.815895523992305	0.669056846009854	EMP1/EMP2/RAC1/CAPN2/LAMC2/VEGFA	6
hsa05206	MicroRNAs in cancer	9/243	310/8577	0.518819869860085	0.815895523992305	0.669056846009854	CDKN1A/VEGFA/EZR/MDM2/CYP24A1/HMOX1/PLAU/PTGS2/PIM1	9
hsa04666	Fc gamma R-mediated phagocytosis	3/243	97/8577	0.521706170858957	0.815895523992305	0.669056846009854	RAC1/LYN/ARPC3	3
hsa00140	Steroid hormone biosynthesis	2/243	62/8577	0.528224827003778	0.815895523992305	0.669056846009854	SULT2B1/SRD5A3	2

hsa01522	Endocrine resistance	3/243	98/8577	0.528651406931344	0.815895523992305	0.669056846009854	HBEGF/CDKN1A/MDM2	3
hsa05231	Choline metabolism in cancer	3/243	98/8577	0.528651406931344	0.815895523992305	0.669056846009854	GPCPD1/RAC1/HIF1A	3
hsa04974	Protein digestion and absorption	3/243	103/8577	0.562518458060026	0.863174875299006	0.707827218853512	PRSS3/MME/ATP1B1	3
hsa04744	Phototransduction	1/243	29/8577	0.566065950243193	0.863654906942472	0.708220858048627	CALML3	1
hsa00630	Glyoxylate and dicarboxylate metabolism	1/243	30/8577	0.578401698265058	0.866795792957936	0.710796471260598	GLUL	1
hsa01523	Antifolate resistance	1/243	30/8577	0.578401698265058	0.866795792957936	0.710796471260598	IL1B	1
hsa04664	Fc epsilon RI signaling pathway	2/243	68/8577	0.578502767777723	0.866795792957936	0.710796471260598	RAC1/LYN	2
hsa04550	Signaling pathways regulating pluripotency of stem cells	4/243	143/8577	0.581110288162811	0.866795792957936	0.710796471260598	ID2/ID1/WNT5A/SKIL	4
hsa04920	Adipocytokine signaling pathway	2/243	69/8577	0.58649287689717	0.869964434064135	0.713394844412932	NFKBIA/ADIPOR1	2
hsa05022	Pathways of neurodegeneration - multiple diseases	13/243	476/8577	0.595886545822711	0.879014959860021	0.720816525333666	IL1B/IL1A/CALML3/RAC1/CAPN2/GPX3/HSPA5/WNT5A/PRNP/XBP1/TUBB2A/UBC/PTGS2	13
hsa04215	Apoptosis - multiple species	1/243	32/8577	0.602035219813996	0.883205514782072	0.724252896016838	PMAIP1	1
hsa05208	Chemical carcinogenesis - reactive oxygen species	6/243	223/8577	0.609428537029897	0.884858347986033	0.72560826485746	SOD2/NFKBIA/RAC1/VEGFA/HIF1A/HMOX1	6
hsa00982	Drug metabolism - cytochrome P450	2/243	72/8577	0.609790022582135	0.884858347986033	0.72560826485746	FMO2/MAOA	2

hsa05226	Gastric cancer	4/243	149/8577	0.613733385537291	0.884938192405106	0.725673739484573	CDKN1A/WNT5A/GADD45A/CDKN2B	4
hsa04024	cAMP signaling pathway	6/243	225/8577	0.61814436591285	0.884938192405106	0.725673739484573	NFKBIA/CALML3/RAC1/ATP1B1/AFDN/ADRB2	6
hsa04710	Circadian rhythm	1/243	34/8577	0.624349064300887	0.884938192405106	0.725673739484573	BHLHE40	1
hsa01230	Biosynthesis of amino acids	2/243	75/8577	0.632078501821991	0.884938192405106	0.725673739484573	GLUL/ASS1	2
hsa04623	Cytosolic DNA-sensing pathway	2/243	75/8577	0.632078501821991	0.884938192405106	0.725673739484573	IL1B/NFKBIA	2
hsa05012	Parkinson disease	7/243	266/8577	0.633051308852195	0.884938192405106	0.725673739484573	DUSP1/CALML3/HSPA5/XBP1/TUBB2A/UBC/MAOA	7
hsa04261	Adrenergic signaling in cardiomyocytes	4/243	154/8577	0.639694862338446	0.884938192405106	0.725673739484573	CALML3/TPM4/ATP1B1/ADRB2	4
hsa00350	Tyrosine metabolism	1/243	36/8577	0.645416633937627	0.884938192405106	0.725673739484573	MAOA	1
hsa00500	Starch and sucrose metabolism	1/243	36/8577	0.645416633937627	0.884938192405106	0.725673739484573	PYGL	1
hsa04721	Synaptic vesicle cycle	2/243	78/8577	0.653365297004231	0.884938192405106	0.725673739484573	CLTB/ATP6V1D	2
hsa00760	Nicotinate and nicotinamide metabolism	1/243	37/8577	0.655504885659107	0.884938192405106	0.725673739484573	NAMPT	1
hsa01250	Biosynthesis of nucleotide sugars	1/243	37/8577	0.655504885659107	0.884938192405106	0.725673739484573	UAP1	1
hsa05143	African trypanosomiasis	1/243	37/8577	0.655504885659107	0.884938192405106	0.725673739484573	IL1B	1
hsa05020	Prion disease	7/243	272/8577	0.656246299985809	0.884938192405106	0.725673739484573	IL1B/IL1A/RAC1/HSPA5/HSPA6/PRNP/TUBB2A	7

hsa01521	EGFR tyrosine kinase inhibitor resistance	2/243	79/8577	0.660240348416764	0.885850115714955	0.726421541765073	VEGFA/TGFA	2
hsa04152	AMPK signaling pathway	3/243	121/8577	0.671484421329763	0.887556141064588	0.727820529633094	RAB10/PFKFB3/ADIPOR1	3
hsa04660	T cell receptor signaling pathway	3/243	121/8577	0.671484421329763	0.887556141064588	0.727820529633094	DLG1/NFKBIA/MAP3K8	3
hsa04919	Thyroid hormone signaling pathway	3/243	121/8577	0.671484421329763	0.887556141064588	0.727820529633094	MDM2/HIF1A/ATP1B1	3
hsa00260	Glycine, serine and threonine metabolism	1/243	40/8577	0.68408644827821	0.899759023104837	0.737827229670042	MAOA	1
hsa04260	Cardiac muscle contraction	2/243	87/8577	0.711365332445695	0.923768907883338	0.757515997791186	TPM4/ATP1B1	2
hsa04926	Relaxin signaling pathway	3/243	129/8577	0.713087710634086	0.923768907883338	0.757515997791186	NFKBIA/VEGFA/GNA15	3
hsa04962	Vasopressin-regulated water reabsorption	1/243	44/8577	0.718554499868244	0.923768907883338	0.757515997791186	AQP3	1
hsa04310	Wnt signaling pathway	4/243	171/8577	0.719199931354133	0.923768907883338	0.757515997791186	RAC1/WNT5A/FOSL1/BAMBI	4
hsa04211	Longevity regulating pathway	2/243	89/8577	0.723100006545384	0.923768907883338	0.757515997791186	SOD2/ADIPOR1	2
hsa05235	PD-L1 expression and PD-1 checkpoint pathway in cancer	2/243	89/8577	0.723100006545384	0.923768907883338	0.757515997791186	NFKBIA/HIF1A	2
hsa04658	Th1 and Th2 cell differentiation	2/243	92/8577	0.739947691565786	0.940790636419356	0.771474284940769	NFKBIA/IL4R	2
hsa04912	GnRH signaling pathway	2/243	93/8577	0.745366591107625	0.943189003391344	0.773441012473864	HBEGF/CALML3	2
hsa04910	Insulin signaling pathway	3/243	137/8577	0.750528773091925	0.945241426488414	0.775124055626218	CALML3/PYGL/TRIP10	3

hsa05010	Alzheimer disease	9/243	384/8577	0.766947533802859	0.946881658313927	0.776469090988752	IL1B/IL1A/MME/CALML3/CAPN2/WNT5A/XBP1/TUBB2A/PTGS2	9
hsa04913	Ovarian steroidogenesis	1/243	51/8577	0.770103447797196	0.946881658313927	0.776469090988752	PTGS2	1
hsa04750	Inflammatory mediator regulation of TRP channels	2/243	98/8577	0.771032821179231	0.946881658313927	0.776469090988752	IL1B/CALML3	2
hsa04925	Aldosterone synthesis and secretion	2/243	98/8577	0.771032821179231	0.946881658313927	0.776469090988752	CALML3/ATP1B1	2
hsa04120	Ubiquitin mediated proteolysis	3/243	142/8577	0.771884625777908	0.946881658313927	0.776469090988752	MDM2/ELOC/UBC	3
hsa00564	Glycerophospholipid metabolism	2/243	99/8577	0.775888329793173	0.946881658313927	0.776469090988752	GPCPD1/MBOAT2	2
hsa00270	Cysteine and methionine metabolism	1/243	52/8577	0.776655742212547	0.946881658313927	0.776469090988752	GCLM	1
hsa00510	N-Glycan biosynthesis	1/243	53/8577	0.783022036012236	0.948793385746472	0.778036760241538	SRD5A3	1
hsa04916	Melanogenesis	2/243	101/8577	0.785330854868803	0.948793385746472	0.778036760241538	CALML3/WNT5A	2
hsa05224	Breast cancer	3/243	147/8577	0.791729604945238	0.952215335677381	0.780842853620719	CDKN1A/WNT5A/GADD45A	3
hsa04922	Glucagon signaling pathway	2/243	107/8577	0.811595389495318	0.964759766797962	0.791129633329297	CALML3/PYGL	2
hsa04931	Insulin resistance	2/243	108/8577	0.815684410315368	0.964759766797962	0.791129633329297	NFKBIA/PYGL	2
hsa04921	Oxytocin signaling pathway	3/243	154/8577	0.817090596140298	0.964759766797962	0.791129633329297	CDKN1A/CALML3/PTGS2	3
hsa04934	Cushing syndrome	3/243	155/8577	0.820492065698198	0.964759766797962	0.791129633329297	CDKN1A/WNT5A/CDKN2B	3

hsa04730	Long-term depression	1/243	60/8577	0.822801671050459	0.964759766797962	0.791129633329297	LYN	1
hsa04150	mTOR signaling pathway	3/243	156/8577	0.823839800861181	0.964759766797962	0.791129633329297	WNT5A/CLIP1/ATP6V1D	3
hsa05161	Hepatitis B	3/243	162/8577	0.842829463402181	0.982687627634857	0.805830973972207	CXCL8/NFKBIA/CDKN1A	3
hsa04020	Calcium signaling pathway	5/243	253/8577	0.849589464896132	0.985466388720517	0.808109634748147	CALML3/VEGFA/TGFA/ADRB2/GNA15	5
hsa04720	Long-term potentiation	1/243	67/8577	0.855312756798371	0.985466388720517	0.808109634748147	CALML3	1
hsa04022	cGMP-PKG signaling pathway	3/243	167/8577	0.857281112524316	0.985466388720517	0.808109634748147	CALML3/ATP1B1/ADRB2	3
hsa05204	Chemical carcinogenesis - DNA adducts	1/243	69/8577	0.863458239940559	0.985466388720517	0.808109634748147	PTGS2	1
hsa04917	Prolactin signaling pathway	1/243	70/8577	0.867358057488096	0.985466388720517	0.808109634748147	IRF1	1
hsa05230	Central carbon metabolism in cancer	1/243	70/8577	0.867358057488096	0.985466388720517	0.808109634748147	HIF1A	1
hsa04611	Platelet activation	2/243	124/8577	0.871126723032152	0.985554385803325	0.808181794784513	VAMP8/LYN	2
hsa04728	Dopaminergic synapse	2/243	132/8577	0.892701289340489	0.992676037290001	0.814021745540391	CALML3/MAOA	2
hsa04360	Axon guidance	3/243	182/8577	0.893898462029501	0.992676037290001	0.814021745540391	RAC1/EPHA2/WNT5A	3
hsa04810	Regulation of actin cytoskeleton	4/243	229/8577	0.893960745896005	0.992676037290001	0.814021745540391	RAC1/EZR/TMSB4X/ARPC3	4
hsa05014	Amyotrophic lateral sclerosis	7/243	364/8577	0.896853366563525	0.992676037290001	0.814021745540391	RAC1/GPX3/ANXA11/HSPA5/XBP1/TUBB2A/SETX	7

hsa04270	Vascular smooth muscle contraction	2/243	134/8577	0.897546556362612	0.992676037290001	0.814021745540391	CALML3/ADM	2
hsa05034	Alcoholism	3/243	188/8577	0.906026041435627	0.992676037290001	0.814021745540391	CALML3/FOSB/MAOA	3
hsa04371	Apelin signaling pathway	2/243	139/8577	0.908785189197928	0.992676037290001	0.814021745540391	PLAT/CALML3	2
hsa04146	Peroxisome	1/243	83/8577	0.909020394060278	0.992676037290001	0.814021745540391	SOD2	1
hsa04814	Motor proteins	3/243	193/8577	0.915162951520552	0.992676037290001	0.814021745540391	TPM4/KIF13B/TUBB2A	3
hsa04911	Insulin secretion	1/243	86/8577	0.916608358154289	0.992676037290001	0.814021745540391	ATP1B1	1
hsa04540	Gap junction	1/243	88/8577	0.921313425352786	0.992676037290001	0.814021745540391	TUBB2A	1
hsa04727	GABAergic synapse	1/243	89/8577	0.923565850566506	0.992676037290001	0.814021745540391	GLUL	1
hsa05410	Hypertrophic cardiomyopathy	1/243	90/8577	0.925754057247979	0.992676037290001	0.814021745540391	TPM4	1
hsa05414	Dilated cardiomyopathy	1/243	96/8577	0.937633298296654	0.995338118415587	0.816204725529044	TPM4	1
hsa04070	Phosphatidylinositol signaling system	1/243	97/8577	0.939420246594486	0.995338118415587	0.816204725529044	CALML3	1
hsa04713	Circadian entrainment	1/243	97/8577	0.939420246594486	0.995338118415587	0.816204725529044	CALML3	1
hsa04724	Glutamatergic synapse	1/243	115/8577	0.964123186526401	0.999997637399266	0.820025659684792	GLUL	1
hsa04613	Neutrophil extracellular trap formation	2/243	191/8577	0.97401225240582	0.999997637399266	0.820025659684792	RAC1/CLEC7A	2

hsa00230	Purine metabolism	1/243	128/8577	0.975442007615454	0.999997637399266	0.820025659684792	AK2	1
hsa05016	Huntington disease	4/243	306/8577	0.976524698053509	0.999997637399266	0.820025659684792	SOD2/GPX3/CLTB/TUBB2A	4
hsa04114	Oocyte meiosis	1/243	131/8577	0.977500809100766	0.999997637399266	0.820025659684792	CALML3	1
hsa00190	Oxidative phosphorylation	1/243	134/8577	0.979387663045649	0.999997637399266	0.820025659684792	ATP6V1D	1
hsa05415	Diabetic cardiomyopathy	2/243	203/8577	0.980743180448291	0.999997637399266	0.820025659684792	CTSD/RAC1	2
hsa05017	Spinocerebellar ataxia	1/243	143/8577	0.984153772454052	0.999997637399266	0.820025659684792	XBP1	1
hsa05207	Chemical carcinogenesis - receptor activation	2/243	212/8577	0.984651502949306	0.999997637399266	0.820025659684792	VEGFA/ADRB2	2
hsa04072	Phospholipase D signaling pathway	1/243	148/8577	0.986309249614321	0.999997637399266	0.820025659684792	CXCL8	1
hsa04723	Retrograde endocannabinoid signaling	1/243	148/8577	0.986309249614321	0.999997637399266	0.820025659684792	PTGS2	1
hsa03010	Ribosome	1/243	167/8577	0.99215155957882	0.999997637399266	0.820025659684792	RPS4Y1	1
hsa03040	Spliceosome	1/243	216/8577	0.998142063818039	0.999997637399266	0.820025659684792	HSPA6	1
hsa04080	Neuroactive ligand-receptor interaction	3/243	367/8577	0.998456367886745	0.999997637399266	0.820025659684792	PRSS3/ADM/ADRB2	3
hsa04740	Olfactory transduction	1/243	439/8577	0.999997637399266	0.999997637399266	0.820025659684792	CALML3	1
